# Past, present, and future of global health financing: a review of development assistance, government, out-of-pocket, and other private spending on health for 195 countries, 1995–2050

**DOI:** 10.1016/S0140-6736(19)30841-4

**Published:** 2019-06-01

**Authors:** Global Burden of Disease Health Financing Collaborator Network, Angela Y. Chang, Krycia Cowling, Angela E. Micah, Abigail Chapin, Catherine S. Chen, Gloria Ikilezi, Nafis Sadat, Golsum Tsakalos, Junjie Wu, Theodore Younker, Yingxi Zhao, Bianca S. Zlavog, Cristiana Abbafati, Anwar E Ahmed, Khurshid Alam, Vahid Alipour, Syed Mohamed Aljunid, Mohammed J. Almalki, Nelson Alvis-Guzman, Walid Ammar, Catalina Liliana Andrei, Mina Anjomshoa, Carl Abelardo T. Antonio, Jalal Arabloo, Olatunde Aremu, Marcel Ausloos, Leticia Avila-Burgos, Ashish Awasthi, Martin Amogre Ayanore, Samad Azari, Natasha Azzopardi-Muscat, Mojtaba Bagherzadeh, Till Winfried Bärnighausen, Bernhard T Baune, Mohsen Bayati, Yared Belete Belay, Yihalem Abebe Belay, Habte Belete, Dessalegn Ajema Berbada, Adam E. Berman, Mircea Beuran, Ali Bijani, Reinhard Busse, Lucero Cahuana-Hurtado, Luis Alberto Cámera, Ferrán Catalá-López, Bal Govind Chauhan, Maria-Magdalena Constantin, Christopher Stephen Crowe, Alexandra Cucu, Koustuv Dalal, Jan-Walter De Neve, Selina Deiparine, Feleke Mekonnen Demeke, Huyen Phuc Do, Manisha Dubey, Maha El Tantawi, Sharareh Eskandarieh, Reza Esmaeili, Mahdi Fakhar, Ali Akbar Fazaeli, Florian Fischer, Nataliya A. Foigt, Takeshi Fukumoto, Nancy Fullman, Adriana Galan, Amiran Gamkrelidze, Kebede Embaye Gezae, Alireza Ghajar, Ahmad Ghashghaee, Ketevan Goginashvili, Annie Haakenstad, Hassan Haghparast Bidgoli, Samer Hamidi, Hilda L. Harb, Edris Hasanpoor, Hamid Yimam Hassen, Simon I. Hay, Delia Hendrie, Andualem Henok, Ileana Heredia-Pi, Claudiu Herteliu, Chi Linh Hoang, Michael K. Hole, Enayatollah Homaie Rad, Naznin Hossain, Mehdi Hosseinzadeh, Sorin Hostiuc, Olayinka Stephen Ilesanmi, Seyed Sina Naghibi Irvani, Mihajlo Jakovljevic, Amir Jalali, Spencer L. James, Jost B. Jonas, Mikk Jürisson, Rajendra Kadel, Behzad Karami Matin, Amir Kasaeian, Habtamu Kebebe Kasaye, Mesfin Wudu Kassaw, Ali Kazemi Karyani, Roghayeh Khabiri, Junaid Khan, Md Nuruzzaman Khan, Young-Ho Khang, Adnan Kisa, Katarzyna Kissimova-Skarbek, Stefan Kohler, Ai Koyanagi, Kristopher J. Krohn, Ricky Leung, Lee-Ling Lim, Stefan Lorkowski, Azeem Majeed, Reza Malekzadeh, Morteza Mansourian, Lorenzo Giovanni Mantovani, Benjamin Ballard Massenburg, Martin McKee, Varshil Mehta, Atte Meretoja, Tuomo J Meretoja, Neda Milevska Kostova, Ted R Miller, Erkin M Mirrakhimov, Bahram Mohajer, Aso Mohammad Darwesh, Shafiu Mohammed, Farnam Mohebi, Ali H Mokdad, Shane Douglas Morrison, Seyyed Meysam Mousavi, Saravanan Muthupandian, Ahamarshan Jayaraman Nagarajan, Vinay Nangia, Ionut Negoi, Cuong Tat Nguyen, Huong Lan Thi Nguyen, Son Hoang Nguyen, Shirin Nosratnejad, Olanrewaju Oladimeji, Stefano Olgiati, Jacob Olusegun Olusanya, Obinna E Onwujekwe, Stanislav S Otstavnov, Adrian Pana, David M. Pereira, Bakhtiar Piroozi, Sergio I Prada, Mostafa Qorbani, Mohammad Rabiee, Navid Rabiee, Alireza Rafiei, Fakher Rahim, Vafa Rahimi-Movaghar, Usha Ram, Chhabi Lal Ranabhat, Anna Ranta, David Laith Rawaf, Salman Rawaf, Satar Rezaei, Elias Merdassa Roro, Ali Rostami, Salvatore Rubino, Mohamadreza Salahshoor, Abdallah M. Samy, Juan Sanabria, João Vasco Santos, Milena M Santric Milicevic, Bruno Piassi Sao Jose, Miloje Savic, Falk Schwendicke, Sadaf G. Sepanlou, Masood Sepehrimanesh, Aziz Sheikh, Mark G Shrime, Solomon Sisay, Shahin Soltani, Moslem Soofi, Moslem Soofi, Vinay Srinivasan, Rafael Tabarés-Seisdedos, Anna Torre, Marcos Roberto Tovani-Palone, Bach Xuan Tran, Khanh Bao Tran, Eduardo A. Undurraga, Pascual R Valdez, Job F M van Boven, Veronica Vargas, Yousef Veisani, Francesco S Violante, Sergey Konstantinovitch Vladimirov, Vasily Vlassov, Sebastian Vollmer, Giang Thu Vu, Charles D A Wolfe, Naohiro Yonemoto, Mustafa Z. Younis, Mahmoud Yousefifard, Sojib Bin Zaman, Alireza Zangeneh, Elias Asfaw Zegeye, Arash Ziapour, Adrienne Chew, Christopher J L Murray, Joseph L Dieleman

## Abstract

**Background:**

Comprehensive and comparable estimates of health spending in each country are a key input for health policy and planning, and are necessary to support the achievement of national and international health goals. Previous studies have tracked past and projected future health spending until 2040 and shown that, with economic development, countries tend to spend more on health per capita, with a decreasing share of spending from development assistance and out-of-pocket sources. We aimed to characterise the past, present, and predicted future of global health spending, with an emphasis on equity in spending across countries.

**Methods:**

We estimated domestic health spending for 195 countries and territories from 1995 to 2016, split into three categories—government, out-of-pocket, and prepaid private health spending—and estimated development assistance for health (DAH) from 1990 to 2018. We estimated future scenarios of health spending using an ensemble of linear mixed-effects models with time series specifications to project domestic health spending from 2017 through 2050 and DAH from 2019 through 2050. Data were extracted from a broad set of sources tracking health spending and revenue, and were standardised and converted to inflation-adjusted 2018 US dollars. Incomplete or low-quality data were modelled and uncertainty was estimated, leading to a complete data series of total, government, prepaid private, and out-of-pocket health spending, and DAH. Estimates are reported in 2018 US dollars, 2018 purchasing-power parity-adjusted dollars, and as a percentage of gross domestic product. We used demographic decomposition methods to assess a set of factors associated with changes in government health spending between 1995 and 2016 and to examine evidence to support the theory of the health financing transition. We projected two alternative future scenarios based on higher government health spending to assess the potential ability of governments to generate more resources for health.

**Findings:**

Between 1995 and 2016, health spending grew at a rate of 4·00% (95% uncertainty interval 3·89–4·12) annually, although it grew slower in per capita terms (2·72% [2·61–2·84]) and increased by less than $1 per capita over this period in 22 of 195 countries. The highest annual growth rates in per capita health spending were observed in upper-middle-income countries (5·55% [5·18–5·95]), mainly due to growth in government health spending, and in lower-middle-income countries (3·71% [3·10–4·34]), mainly from DAH. Health spending globally reached $8·0 trillion (7·8–8·1) in 2016 (comprising 8·6% [8·4–8·7] of the global economy and $10·3 trillion [10·1–10·6] in purchasing-power parity-adjusted dollars), with a per capita spending of US$5252 (5184–5319) in high-income countries, $491 (461–524) in upper-middle-income countries, $81 (74–89) in lower-middle-income countries, and $40 (38–43) in low-income countries. In 2016, 0·4% (0·3–0·4) of health spending globally was in low-income countries, despite these countries comprising 10·0% of the global population. In 2018, the largest proportion of DAH targeted HIV/AIDS ($9·5 billion, 24·3% of total DAH), although spending on other infectious diseases (excluding tuberculosis and malaria) grew fastest from 2010 to 2018 (6·27% per year). The leading sources of DAH were the USA and private philanthropy (excluding corporate donations and the Bill & Melinda Gates Foundation). For the first time, we included estimates of China's contribution to DAH ($644·7 million in 2018). Globally, health spending is projected to increase to $15·0 trillion (14·0–16·0) by 2050 (reaching 9·4% [7·6–11·3] of the global economy and $21·3 trillion [19·8–23·1] in purchasing-power parity-adjusted dollars), but at a lower growth rate of 1·84% (1·68–2·02) annually, and with continuing disparities in spending between countries. In 2050, we estimate that 0·6% (0·6–0·7) of health spending will occur in currently low-income countries, despite these countries comprising an estimated 15·7% of the global population by 2050. The ratio between per capita health spending in high-income and low-income countries was 130·2 (122·9–136·9) in 2016 and is projected to remain at similar levels in 2050 (125·9 [113·7–138·1]). The decomposition analysis identified governments’ increased prioritisation of the health sector and economic development as the strongest factors associated with increases in government health spending globally. Future government health spending scenarios suggest that, with greater prioritisation of the health sector and increased government spending, health spending per capita could more than double, with greater impacts in countries that currently have the lowest levels of government health spending.

**Interpretation:**

Financing for global health has increased steadily over the past two decades and is projected to continue increasing in the future, although at a slower pace of growth and with persistent disparities in per-capita health spending between countries. Out-of-pocket spending is projected to remain substantial outside of high-income countries. Many low-income countries are expected to remain dependent on development assistance, although with greater government spending, larger investments in health are feasible. In the absence of sustained new investments in health, increasing efficiency in health spending is essential to meet global health targets.

**Funding:**

Bill & Melinda Gates Foundation.

## Introduction

Financial resources are an essential input to health systems—at a minimum, these are necessary to purchase medicines and supplies, build health facilities, and pay health workers. However, limited financial resources are a universal constraint faced by all health systems. WHO has identified health financing as one of the six key building blocks of health systems and adequate financing is essential to the other five blocks.^1^ Health financing systems are tasked not only with raising sufficient financial resources to fund the health system, but doing so in a way that promotes equity.^2^ Health systems funded according to one's ability to pay, such as those based on income taxes, promote both financial equity and better health.^3^ Over-reliance on out-of-pocket spending diminishes access to care for those who are uninsured or underinsured, and risks exacerbating the burden of ill health and increasing poverty due to the high cost of care.^4^ The recognised importance of financial protection has led to its inclusion as one of two pillars of universal health coverage, alongside coverage of core health services, as outlined in Sustainable Development Goal 3.

**Research in context**

**Evidence before this study**

Understanding past trends and anticipating future trends in health financing is important for planning and allocating resources required to achieve universal health coverage and other health goals. Previous studies, including work by the Global Burden of Disease Health Financing Collaborator Network, have tracked past and projected future health spending and spending disaggregated by funding source (ie, government, prepaid private, out-of-pocket, and development assistance for health) up to 2040. A 2018 report from WHO documents the global pattern of declining external financing and increasing domestic public funding, supporting key findings from other existing studies. Research focusing on the global health financing transition by this team and others has shown that with economic development, countries tend to spend more money on health per capita and that a declining share of this spending tends to come from development assistance and out-of-pocket sources.

**Added value of this study**

This study is, to our knowledge, the first analysis of global health financing to generate past trends, characterise present patterns, and predict future scenarios for 195 countries over a period spanning 56 years, with an emphasis on equity across countries over time, providing a holistic assessment of the state of global health financing. This analysis provides new estimates of total, government, prepaid private, and out-of-pocket health spending and development assistance for health for 195 countries spanning from 1995 to 2050. The relationship between economic development and the distribution of these sources of financing provides further support for the theory of the global health financing transition. The decomposition analysis shows, for the first time, key factors that have been associated with increases in government health spending across countries, showing that increased prioritisation of the health sector and economic development are associated with the largest increases in government health spending globally. These time trends in health spending also reveal persistent disparities across income groups, with per capita health spending in high-income countries 130·2 times (95% uncertainty interval 122·9–136·9) that in low-income countries in 2016, and projected to remain stable at 125·9 times (113·7–138·1) greater in 2050. Within low-income and middle-income country groups, the gaps between countries with the highest and lowest government health spending per capita are projected to widen between now and the future. Furthermore, consistently high rates of out-of-pocket spending in low-income and middle-income countries suggest ongoing within-country inequities. Although these trends also provide evidence of the global health financing transition, many countries’ trends run counter to global norms.

**Implications of all the available evidence**

Development assistance for health has plateaued; moreover, projected future spending suggests that low levels of domestic health spending and high out-of-pocket spending will persist in many low-income countries. Increasing prioritisation of health and economic development should be supported as key mechanisms to increase government health spending and address persistent global inequities in health spending. Given the limited financial resources for health in all countries and persistently low levels of health financing in some, it is important to identify and implement policies to generate additional resources and improve the efficiency of health spending to maximise health outcomes in the future.

Empirical studies have shown that reducing government health spending per capita can lead to increased child, adult, and maternal mortality.^5^, ^6^, ^7^, ^8^ Other research has found that countries with lower levels of health spending coming from pooled financing mechanisms, such as insurance-based or tax-based financing, have lower performance on universal health coverage.^9^ These benefits and the established risks of high out-of-pocket spending have led to a focus on the composition of sources of health financing across countries. The health financing transition is a theory developed to characterise the gradual shift in the level and source of health financing observed in countries over time. Generally, countries start this transition with a low initial level of health spending per capita that is largely out of pocket or from donors, and progressively transition to higher per capita spending relying more on government financing.

Tracking financial resources for health is a prerequisite for assessing the performance of health financing systems and financial protection, characterising progress along the health financing transition, evaluating health-system efficiency and productivity, or advocating for health-system policy change. Moreover, developing future health financing scenarios enables policy makers and donors to predict the amount of services that can be provided and identify gaps where expected funding is insufficient. Established frameworks and examples from a range of countries underscore the important role of timely, comprehensive health financing estimates in decision making and analysis.^10^, ^11^ As countries work towards global commitments to universal health coverage and the other health-related targets enshrined in the UN Sustainable Development Goals, the expected resources available for health can be used to assess expected progress. In the absence of comprehensive and comparable health financing estimates, policy makers and planners cannot clearly measure how much has been spent on health, where funding has come from, or what are reasonable expectations for future spending.

This study incorporates several important methodological advancements and novel analyses. The health financing estimation methods are continuously improving and forecasting is particularly enhanced by advances in the underlying approach to project gross domestic product (GDP). The time horizon for spending forecasts is 10 years longer than previously available and alternative future scenarios are based, for the first time, on a new understanding of factors associated with increased government spending, as identified from the decomposition analysis, also new to this study. Additionally, these estimates include seven additional countries or territories not previously included. There are also several advances specific to the development assistance for health (DAH) estimates, including the addition of China as a donor, inclusion of the Coalition for Epidemic Preparedness Innovations and the European Economic Area as channels of disbursements, and spending disaggregated by new programme areas, such as antimicrobial resistance.

The objective of this analysis is to provide comprehensive and comparable national health spending estimates, by four major sources of funding, from 1995 to 2016 and into 2050, emphasising equity in spending across countries over time. We also characterise health spending patterns associated with economic development to assess support for the theory of the health financing transition, analyse factors associated with increases in government health spending, and report expected future spending under two alternative government spending scenarios.

## Methods

### Overview

The methods presented here summarise the various components of the estimation process; the appendix provides further details about data sources, methods, and additional results presented in alternative units. We defined health spending as money spent on services, supplies, and basic infrastructure to deliver health care, using the same definition used by the System of Health Accounts 2011 and the WHO Global Health Expenditure Database (GHED).^12^, ^13^

We estimated health spending from four main funding sources—government, out-of-pocket, prepaid private, and DAH—for 195 countries and territories. “Countries and territories” are referred to only as “countries”, which are categorised into four World Bank income groups and seven Global Burden of Disease (GBD) super-regions. Data tracking government, out-of-pocket, and prepaid private health spending, which together comprise total domestic health spending, were available from 1995 through 2016. Government health spending includes social health insurance and mandated private health insurance, as well as government public health programmes. Out-of-pocket health spending includes health-care spending by the patient or their household, excluding insurance premiums paid in advance of care. Prepaid private health spending includes voluntary private insurance and non-governmental agency spending on health.

DAH was defined as the financial and in-kind contributions from major development agencies to low-income and middle-income countries for maintaining or improving population health. The total amount of DAH, by source, was estimated through 2018, but was not allocated by recipient country for 2018. The sum of domestic health spending and DAH, net of administrative costs needed to run development agencies, form the envelope of total health spending for each country and year.

Domestic health spending from each of the three sources was projected for each country from 2017 to 2050, and DAH was projected from 2018 to 2050, by modelling rates of change across time. These models incorporate country-specific time trends that attenuate across time and converge to the global average, consider a broad set of covariates and time-series modelling techniques, and propagate four types of uncertainty: model, data, parameter, and fundamental uncertainty.

### Estimating domestic health spending for 1995–2016

We extracted data on GDP per capita from five leading sources of these estimates.^14^, ^15^, ^16^, ^17^, ^18^ Building from methods described by James and colleagues,^19^ we generated a single series of GDP per capita using Gaussian processes, incorporating data from all five GDP series from 1970 to 2017.^19^

We extracted data from the WHO's GHED on government domestic revenue transfers allocated for health, compulsory prepayment, voluntary prepayment, social insurance contributions, and other domestic revenue from households, corporations, and non-profit institutions serving households.^12^ Data from GHED exclude spending on major investments (eg, hospital construction, health worker education and training, and research and development). Health spending estimates were extracted in current national currency units, deflated to 2018 national currency units, and exchanged to 2018 US dollars. Deflator series and exchanges rates were taken from the IMF World Economic Outlook.^16^ To generate domestic health spending estimates in purchasing-power parity-adjusted dollars, we divided health spending in 2018 US dollars by GDP in 2018 US dollars, and then multiplied health spending fractions by GDP per capita measured in 2018 purchasing-power parity-adjusted dollars.

The extracted data were assessed for quality using point-specific metadata provided in the GHED, and weighted according to estimation methods and whether they were tied to an underlying data source. We then used a spatiotemporal Gaussian process regression model to estimate health spending across time, country, and spending category.^20^ We based weights on metadata completeness, documented source information, and documented methods for estimation.

### Estimating development assistance for health for 1990–2018

Although most of the methods used for tracking DAH have been described previously, we incorporated several major improvements.^21^, ^22^, ^23^, ^24^, ^25^ These include the addition of China as a source of funding; the inclusion of the Coalition for Epidemic Preparedness Innovations as a channel; and the addition of antimicrobial resistance as a programme area. The estimate we generated for antimicrobial resistance is restricted to funds that were disbursed through development agencies. These improvements expand the scope of our DAH resource tracking to capture some of the emerging areas of importance in the current global health financing landscape. For all DAH tracking, we include funds that were transferred through major development agencies, as well as private foundations and non-governmental agencies for whom we have data. DAH excludes spending on basic bench science. Detailed descriptions of the methodology used for tracking DAH and these improvements, including data sources and keywords used to isolate relevant projects, are included in the appendix.

### Factors associated with changes in government health spending for 1995–2016

We completed a decomposition analysis to understand the relationship between changes in per capita government health spending between 1995 and 2016 and the underlying contributing factors. A standard demographic decomposition technique popularised by Das Gupta was applied; this approach yields estimates of how changes in each of a set of prespecified factors are associated with changes in the outcome (government health spending per capita).^26^ The three factors examined were economic development, measured as GDP per person (GDP/Pop); increased total government spending, measured as the proportion of GDP that is government spending (Gov/GDP); and greater government prioritisation of the health sector, measured as the proportion of total government spending spent on the health sector (Gov Health/Gov). The product of these three factors is government health spending per capita (Gov Health/Pop):

$$\frac{Go\mathrm{v H}ealth}{Pop}=\frac{GDP}{Pop}\times \frac{Gov}{GDP}\times \frac{Go\mathrm{v H}ealth}{Gov}$$

These three factors form a comprehensive set, as all other factors that influence government health spending must operate through one or more of those factors. For example, if demand for health services increases or a population ages and requires additional health services from the government, this must lead to an increase in total government spending or a reprioritisation of existing government spending towards health. This decomposition approach measures the relative contribution of each factor to changes in per capita government health spending during the time period examined.

### Estimating health spending in the future, for 2017–50

Future health spending scenarios were estimated with an ensemble modelling framework and key covariates. A process diagram in the appendix displays the flow of input data and models for each step of the forecasting process. Ensemble modelling estimates a set of future scenarios using a large number of distinct sub-models and then takes the average across all sub-models that pass a predetermined inclusion criterion.^27^ Each sub-model has a distinct specification or set of covariates; primary covariates considered were GDP per capita, total government spending, total fertility rate, and fraction of the population older than 65 years, as well as country-specific time trends. Total fertility rates and age-specific population data were extracted from the UN World Population Prospects, while we generated our own estimates of GDP per capita and fraction of GDP from government spending.^28^

To project expected GDP per capita for each of the 195 countries from 2018 through 2050, we estimated the GDP per working-age adult growth rate (ages 20–64 years). Using out-of-sample validation, we showed that GDP per capita could be more accurately estimated (smaller root-mean-squared error) by estimating GDP per working-age adult growth rates, rather than GDP per capita growth rates.

After estimating GDP per capita, we used the same method to estimate future scenarios of total government spending as a fraction of GDP, government health spending as a fraction of total government spending, prepaid private health spending as a fraction of GDP, and out-of-pocket health spending as a fraction of GDP. We called these our reference future scenarios. Additionally, we estimated future scenarios of the share of health spending that was provided as DAH from each major donor country, which allowed us to estimate total DAH expected to be disbursed between 2019 and 2050. Next, we estimated the fraction of the total amount of DAH that we expected each low-income and middle-income country to receive. Finally, if a country was projected to reach high-income status before 2050, it was deemed ineligible to receive DAH from that year onward and the DAH it was otherwise expected to receive was reallocated to all other countries eligible to receive DAH. To estimate total health spending for each country and year, we added DAH received by countries to estimates of government, prepaid private, and out-of-pocket health spending.

### Alternative future government health spending scenarios

To assess the potential for governments to generate more resources for health, we estimated two alternative future scenarios associated with higher government health spending: one reflects increased prioritisation of the health sector, and the other reflects both increased overall government spending and increased government prioritisation of health. To generate the two scenarios, we assessed the observed 2016 fraction of government spending that was allocated to the health sector (Gov Health/Gov) and the fraction of GDP that is based on government spending (Gov/GDP) across the 195 countries. We then set the target levels of the two fractions as the 90th percentile of the observed fractions’ distributions. Building on the existing GDP per capita projections, scenario 1 adjusts all countries so that the fraction of government spending on health is at least the 90th percentile. Scenario 2 adjusts all countries so that both the fraction of government spending on health and the fraction of GDP that is based on government spending is at least the 90th percentile.

### Reporting and uncertainty analysis

All inflation-adjusted health spending estimates are reported with 2018 prices. We report health spending per capita in US dollars and purchasing-power parity-adjusted dollars and as a fraction of GDP. When not otherwise indicated, estimates are reported in 2018 US dollars. We report country spending estimates using 2017 GBD super-regions and 2018 World Bank income groups, regardless of whether a country changed, or is projected to change, income groups during the study period.^29^, ^30^ Rates were calculated to reflect each group, rather than the average of countries within the group, such that spending per capita estimates for an income group or region more heavily reflect rates in more populous countries. The uncertainty interval around each estimate was computed with the 2·5th and 97·5th percentiles of the 1000 draws. All analyses were done with R (version 3.5.2) and Stata (version 13).

### Role of the funding source

The funder of this study had no role in study design, data collection, data analysis, data interpretation, or writing of the manuscript. All authors had full access to all the data in the study, and JLD and CJLM had final responsibility for the decision to submit for publication.

## Results

### Overview

This analysis focuses on the past, present, and future of global health financing. First, we present levels of health spending and trends in health spending for the historical period from 1995 to 2016, and the analysis of factors contributing to increases in government health spending. Second, we highlight the role that DAH has played in providing resources for health, especially to low-income countries from 1990 to 2018. Third, we focus on health spending in 2016, and assess variations in the composition of financing sources across countries. Fourth, we present future scenarios of health spending, assessing levels and growth rates of health spending from 2017 to 2050, with an additional emphasis on 2030, given its significance as the target year for achieving the Sustainable Development Goals. Finally, we highlight observed and expected trends during the entire study period. All estimates made in this Article are available to view in an associated visualisation, available on Viz Hub.

### Past and present

**Figure 1:**
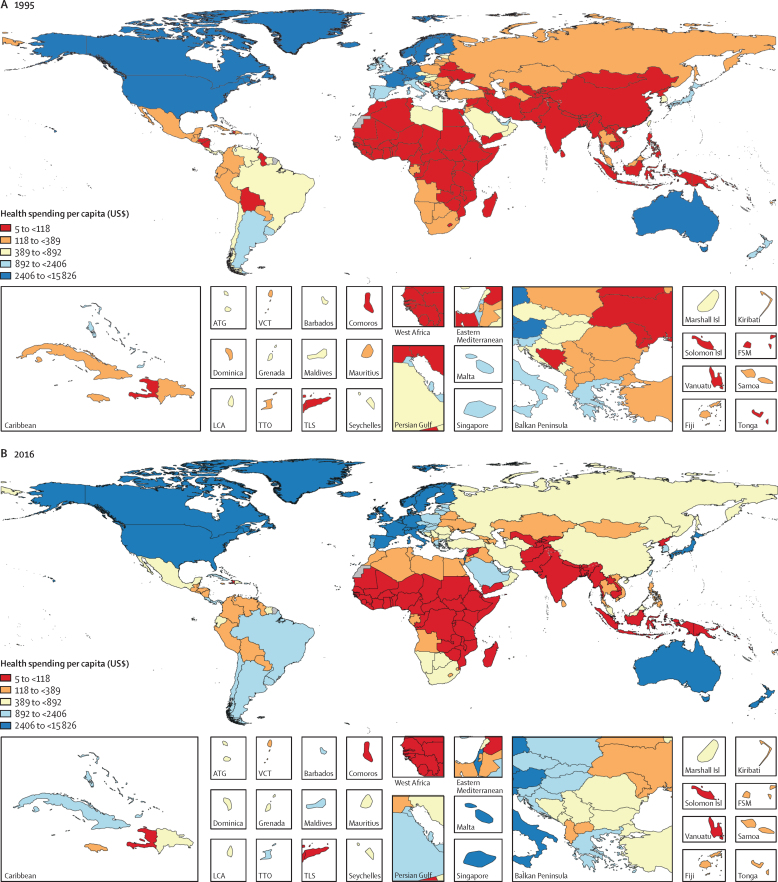

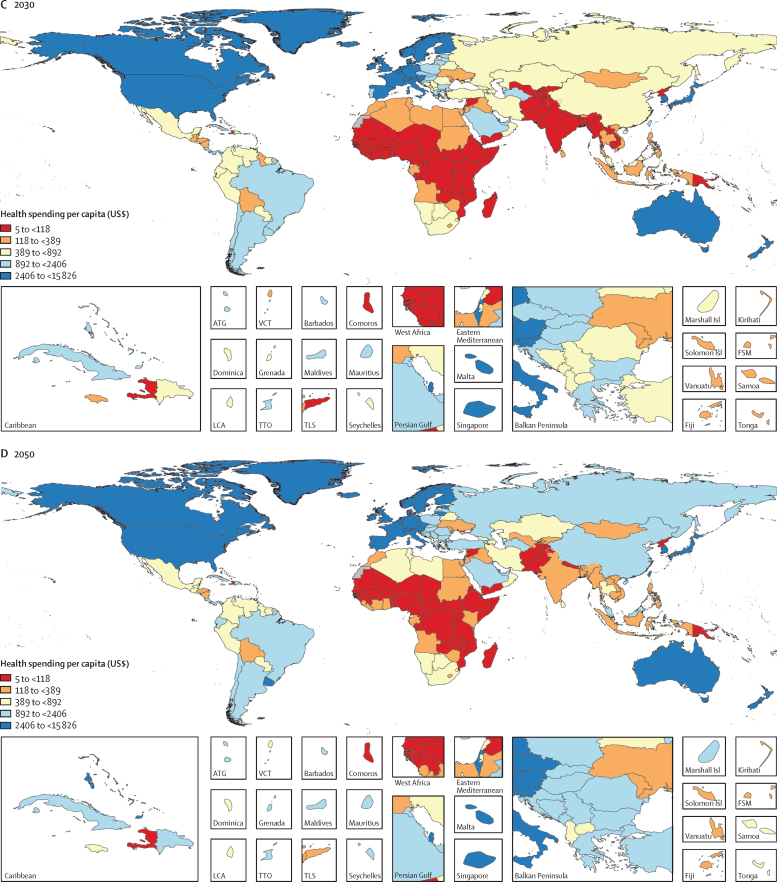
Health spending per capita in 1995 (A), 2016 (B), 2030 (C), and 2050 (D) Reported in inflation-adjusted 2018 US dollars. 2030 and 2050 values are reference scenarios. This figure was remade but with health spending measured as a percentage of gross domestic product, and is included in the appendix. ATG=Antigua and Barbuda. VCT=Saint Vincent and the Grenadines. LCA=Saint Lucia. TTO=Trinidad and Tobago. Isl=Islands. FSM=Federated States of Micronesia. TLS=Timor-Leste.

In 1995, health spending globally was $3·5 trillion (95% uncertainty interval [UI] 3·4–3·5), $4·3 trillion (4·2–4·4) in purchasing-power parity-adjusted dollars, and comprised 6·9% (6·8–7·0) of global GDP. That year, 87·6% (87·1–88·1) was spent in countries that are currently high-income, 9·8% (9·4–10·3) in upper-middle-income countries, 2·2% (2·1–2·4) in lower-middle-income countries, and only 0·3% (0·3–0·4) in low-income countries. Health spending per capita globally was $612 (603–622), ranging from $5 (4–7) in Myanmar to $7318 (5490–10 192) in Bermuda (figure 1A). In 1995, countries currently classified as high income spent $2871 (2823–2921) per capita on health, whereas those classified as upper-middle income spent $158 (150–166) per capita, those classified as lower-middle income spent $38 (35–41) per capita, and those classified as low income spent $30 (28–31) per capita. Health spending per capita was the lowest in South Asia, at $26 (21–31) per capita, and in sub-Saharan Africa, at $58 (54–62) per capita, and highest in GBD high-income countries, at $3206 (3151–3264) per capita.

**Figure 2:**
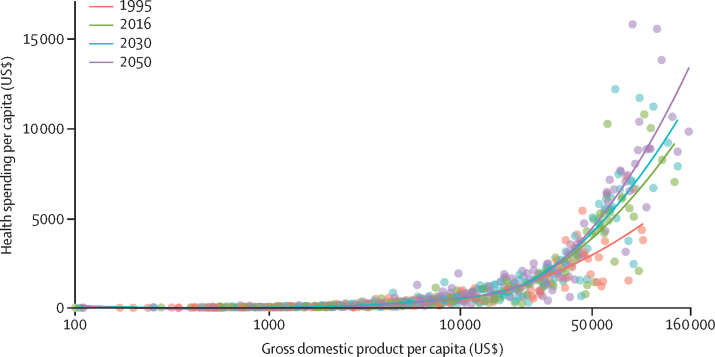
Health spending per capita by gross domestic product per capita, for 1995, 2016, 2030, and 2050 Health spending per capita and gross domestic product per capita are reported in inflation-adjusted 2018 US dollars. The lines are the trend lines reflecting model fit for each year. 2030 and 2050 values are reference scenarios. Each dot represents a country-year estimate, with the colours representing different years (1995, 2016, 2030, and 2050). The x-axis is presented in natural logarithmic scale. This figure was remade but with health spending measured as a percentage of gross domestic product, and is included in the appendix.

**Figure 3:**
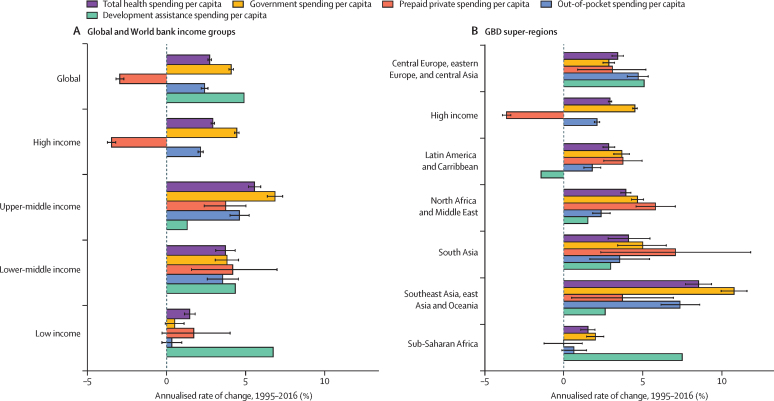
Annualised rate of change in health spending per capita by source, by World Bank income group (A) and GBD super-region (B), 1995–2016 Error bars represent 95% uncertainty intervals. This figure was remade but with health spending measured as a percentage of gross domestic product, and is included in the appendix. GBD=Global Burden of Disease.

Between 1995 and 2016, there was substantive growth in health spending in many countries, with a global growth rate of 4·00% (95% UI 3·89–4·12) annually, although this rate was lower for health spending per capita (2·72% [2·61–2·84]; figure 1B, figure 2, table 1). Countries with the largest absolute increases in annual per capita health spending during this period were the USA ($4843 [4580–5125] increase), Norway ($3913 [3501–4327] increase), and Bermuda ($3485 [535–5916] increase), while spending increased by less than $1 per capita in 22 countries. The most populous of these 22 countries are Venezuela, Yemen, and Angola. Figure 3 shows that the highest annual growth rates in per capita health spending were observed in upper-middle-income (5·55% [5·18–5·95]) and lower-middle-income countries (3·71% [3·10–4·34]). In upper-middle-income countries, the largest source of this increase was increased government health spending (6·85% [6·37–7·34]) and in lower-middle-income countries the fastest growth was in DAH (4·34%). These groups of countries also saw rapid annual growth in out-of-pocket spending: 3·54% (2·57–4·54) in lower-middle-income countries and 4·60% (4·01–5·22) in upper-middle-income countries. Although DAH per capita increased rapidly, at 6·74% annually in low-income countries, overall growth in health spending per capita remained low at 1·46% (1·13–1·80) per year in these countries. Geographically, southeast Asia, east Asia, and Oceania had the highest growth in health spending per capita (8·52% [7·69 to 9·33]) annually between 1995 and 2016, driven mainly by large growth in government health spending (10·76% [9·94 to 11·57]) and out-of-pocket spending (7·34% [6·15 to 8·59]), whereas sub-Saharan Africa had the lowest growth in health spending per capita (1·54% [1·08 to 1·97]), with only modest increases in government health spending (2·00% [1·45 to 2·53]) and out-of-pocket spending (0·65% [–0·12 to 1·44]). The negative growth (–3·49% [–3·75 to −3·22]) in prepaid private spending per capita in high-income countries (figure 3) is attributable to the enactment in 2014 of the insurance mandate in the US Affordable Care Act, which reclassified a large proportion of health spending that was originally prepaid private spending as government health spending because this spending became compulsory.^13^

**Table 1:** Health spending by source, 2016

	**Health spending per capita, 2016 (US$)**	**Health spending per capita, 2016 ($PPP)**	**Health spending per GDP, 2016**	**Government health spending per total health spending, 2016**	**Out-of-pocket spending per total health spending, 2016**	**Development assistance for health per total health spending, 2016**	**Annualised rate of change in health spending, 1995–2016 (US$)**	**Annualised rate of change in health spending per capita, 1995–2016 (US$)**	**Annualised rate of change in health spending per GDP, 1995–2016 (US$)**
**Global**									
Total	1077 (1058 to 1096)	1400 (1368 to 1432)	8·6% (8·4 to 8·7)	74·0% (72·5 to 75·5)	18·6% (18·0 to 19·4)	0·2% (0·2 to 0·2)	4·00% (3·89 to 4·12)	2·72% (2·61 to 2·84)	1·02% (0·92 to 1·12)
**World Bank income group**									
High income	5252 (5184 to 5319)	5621 (5548 to 5693)	10·8% (10·6 to 10·9)	79·6% (78·2 to 81·1)	13·8% (13·5 to 14·2)	0·0% (0·0 to 0·0)	3·61% (3·51 to 3·71)	2·92% (2·81 to 3·02)	1·52% (1·42 to 1·62)
Upper-middle income	491 (461 to 524)	1009 (948 to 1072)	5·0% (4·7 to 5·3)	53·9% (49·9 to 58·6)	35·9% (32·0 to 40·0)	0·2% (0·1 to 0·2)	6·37% (5·95 to 6·79)	5·55% (5·18 to 5·95)	1·17% (0·81 to 1·55)
Lower-middle income	81 (74 to 89)	274 (247 to 303)	3·2% (2·9 to 3·5)	32·1% (28·4 to 36·1)	56·1% (47·3 to 65·4)	3·2% (2·9 to 3·6)	5·40% (4·76 to 6·08)	3·71% (3·10 to 4·34)	0·00% (−0·63 to 0·60)
Low income	40 (38 to 43)	125 (119 to 132)	5·1% (4·9 to 5·4)	26·3% (23·3 to 29·5)	42·4% (38·3 to 47·0)	25·4% (23·9 to 26·8)	4·25% (3·88 to 4·62)	1·46% (1·13 to 1·80)	0·39% (0·05 to 0·70)
**GBD super-region**									
Central Europe, eastern Europe, and central Asia	530 (505 to 555)	1265 (1200 to 1330)	4·3% (4·1 to 4·5)	62·6% (59·4 to 65·9)	33·5% (31·3 to 35·8)	0·3% (0·2 to 0·3)	3·44% (3·10 to 3·81)	3·41% (3·06 to 3·77)	0·06% (−0·26 to 0·37)
High income	5874 (5798 to 5950)	6107 (6028 to 6185)	11·2% (11·1 to 11·4)	79·9% (78·5 to 81·5)	13·5% (13·2 to 13·9)	0·0% (0·0 to 0·0)	3·57% (3·47 to 3·68)	2·93% (2·82 to 3·03)	1·59% (1·49 to 1·69)
Latin America and Caribbean	693 (658 to 728)	1270 (1209 to 1333)	6·4% (6·1 to 6·7)	42·7% (40·3 to 44·9)	39·5% (36·0 to 43·2)	0·3% (0·3 to 0·3)	4·21% (3·83 to 4·62)	2·84% (2·48 to 3·22)	1·56% (1·20 to 1·93)
North Africa and Middle East	336 (320 to 352)	1000 (949 to 1053)	3·7% (3·5 to 3·9)	61·4% (56·9 to 65·9)	29·3% (27·5 to 31·3)	0·5% (0·4 to 0·5)	6·01% (5·66 to 6·42)	3·92% (3·60 to 4·25)	1·87% (1·58 to 2·19)
South Asia	59 (49 to 71)	219 (182 to 265)	3·0% (2·5 to 3·5)	25·0% (18·7 to 32·2)	65·2% (46·7 to 88·1)	1·9% (1·6 to 2·3)	5·76% (4·42 to 7·15)	4·09% (2·81 to 5·44)	−0·73% (−1·96 to 0·59)
Southeast Asia, east Asia, and Oceania	350 (319 to 385)	703 (643 to 769)	4·7% (4·3 to 5·1)	57·5% (50·8 to 65·5)	35·9% (30·0 to 42·8)	0·2% (0·2 to 0·2)	9·35% (8·56 to 10·14)	8·52% (7·69 to 9·33)	1·72% (0·98 to 2·45)
Sub-Saharan Africa	80 (75 to 86)	199 (186 to 214)	4·1% (3·9 to 4·3)	36·8% (34·0 to 39·8)	31·5% (27·3 to 36·3)	14·0% (13·1 to 14·9)	4·31% (3·88 to 4·76)	1·54% (1·08 to 1·97)	−0·17% (−0·58 to 0·21)
**Country**									
Afghanistan	56 (43 to 71)	200 (156 to 256)	7·4% (5·8 to 9·5)	5·7% (3·9 to 7·9)	84·3% (80·0 to 88·0)	9·7% (7·5 to 12·3)	7·06% (5·15 to 9·12)	3·41% (1·57 to 5·40)	1·07% (−0·73 to 3·01)
Albania	330 (292 to 371)	867 (768 to 976)	6·0% (5·3 to 6·7)	42·3% (36·5 to 48·1)	57·2% (51·3 to 63·0)	0·5% (0·4 to 0·6)	4·31% (3·28 to 5·26)	4·74% (3·70 to 5·68)	−0·04% (−1·04 to 0·86)
Algeria	304 (267 to 341)	1055 (926 to 1184)	4·7% (4·1 to 5·2)	69·4% (63·4 to 74·8)	29·2% (23·8 to 34·8)	0·0% (0·0 to 0·0)	6·71% (5·72 to 7·69)	5·12% (4·14 to 6·09)	3·18% (2·22 to 4·13)
American Samoa	692 (604 to 791)	692 (604 to 791)	6·4% (5·6 to 7·4)	90·1% (86·5 to 93·0)	8·3% (5·8 to 11·7)	0·0% (0·0 to 0·0)	−0·20% (−1·55 to 1·01)	−2·20% (−3·52 to −1·01)	−1·65% (−2·97 to −0·45)
Andorra	4234 (4107 to 4357)	7865 (7629 to 8093)	8·2% (7·9 to 8·4)	48·9% (47·5 to 50·3)	41·9% (40·5 to 43·2)	0·0% (0·0 to 0·0)	2·50% (2·27 to 2·73)	1·13% (0·91 to 1·36)	−0·07% (−0·29 to 0·15)
Angola	121 (100 to 143)	201 (167 to 237)	2·4% (2·0 to 2·8)	48·3% (39·7 to 56·7)	31·7% (24·3 to 40·2)	3·6% (3·0 to 4·3)	3·24% (1·98 to 4·43)	−0·12% (−1·34 to 1·04)	−3·81% (−4·98 to −2·69)
Antigua and Barbuda	760 (712 to 811)	1233 (1156 to 1316)	4·8% (4·5 to 5·2)	64·4% (61·3 to 67·7)	29·1% (26·2 to 32·2)	0·0% (0·0 to 0·0)	3·98% (3·44 to 4·57)	2·48% (1·94 to 3·06)	1·28% (0·75 to 1·86)
Argentina	1071 (1008 to 1135)	1616 (1520 to 1713)	7·9% (7·5 to 8·4)	76·1% (73·5 to 78·9)	14·8% (12·7 to 16·9)	0·7% (0·6 to 0·7)	1·83% (1·39 to 2·25)	0·68% (0·24 to 1·09)	−0·69% (−1·13 to −0·29)
Armenia	365 (323 to 411)	933 (827 to 1051)	7·8% (6·9 to 8·8)	15·8% (12·5 to 19·7)	81·1% (77·0 to 84·5)	1·9% (1·7 to 2·2)	10·73% (9·69 to 11·80)	11·04% (10·01 to 12·12)	4·25% (3·28 to 5·27)
Australia	5563 (5476 to 5650)	5083 (5004 to 5162)	7·1% (7·0 to 7·2)	68·3% (67·4 to 69·3)	18·9% (18·1 to 19·6)	0·0% (0·0 to 0·0)	4·72% (4·56 to 4·89)	3·28% (3·12 to 3·44)	1·47% (1·31 to 1·63)
Austria	5287 (5199 to 5379)	5252 (5166 to 5344)	9·2% (9·0 to 9·3)	72·6% (71·7 to 73·4)	18·9% (18·3 to 19·6)	0·0% (0·0 to 0·0)	2·20% (2·05 to 2·35)	1·76% (1·61 to 1·91)	0·43% (0·28 to 0·58)
Azerbaijan	297 (261 to 335)	1192 (1048 to 1347)	3·6% (3·2 to 4·1)	20·6% (16·5 to 25·2)	78·3% (73·6 to 82·5)	0·3% (0·3 to 0·4)	10·29% (9·06 to 11·44)	9·00% (7·79 to 10·14)	1·27% (0·14 to 2·33)
Bahrain	1169 (1109 to 1233)	2365 (2243 to 2494)	4·3% (4·0 to 4·5)	62·7% (59·9 to 65·4)	27·1% (24·8 to 29·6)	0·0% (0·0 to 0·0)	5·39% (4·91 to 5·85)	1·00% (0·55 to 1·44)	0·88% (0·42 to 1·32)
Bangladesh	37 (29 to 48)	100 (78 to 128)	3·1% (2·4 to 3·9)	19·2% (13·7 to 26·0)	71·4% (62·8 to 78·6)	6·7% (5·1 to 8·4)	5·42% (3·78 to 7·11)	3·81% (2·19 to 5·47)	−0·41% (−1·96 to 1·19)
Barbados	1188 (1124 to 1257)	1244 (1177 to 1316)	6·3% (6·0 to 6·7)	46·9% (44·1 to 49·6)	45·8% (43·0 to 48·2)	0·0% (0·0 to 0·0)	2·21% (1·73 to 2·65)	1·86% (1·38 to 2·30)	0·81% (0·33 to 1·24)
Belarus	354 (318 to 396)	1170 (1051 to 1308)	5·0% (4·5 to 5·5)	61·1% (55·1 to 66·6)	35·9% (30·5 to 41·9)	0·3% (0·3 to 0·3)	5·60% (4·67 to 6·57)	5·93% (4·99 to 6·89)	0·44% (−0·45 to 1·35)
Belgium	5014 (4894 to 5135)	5048 (4927 to 5169)	9·2% (8·9 to 9·4)	79·1% (78·1 to 80·0)	15·1% (14·4 to 16·0)	0·0% (0·0 to 0·0)	3·18% (2·96 to 3·39)	2·61% (2·40 to 2·82)	1·36% (1·15 to 1·57)
Belize	283 (249 to 317)	511 (449 to 573)	5·6% (4·9 to 6·3)	66·3% (60·1 to 72·0)	23·4% (18·4 to 29·0)	3·5% (3·1 to 3·9)	6·29% (5·27 to 7·28)	3·37% (2·38 to 4·33)	2·16% (1·18 to 3·11)
Benin	32 (27 to 38)	83 (70 to 98)	3·1% (2·6 to 3·6)	22·3% (16·7 to 28·1)	44·3% (35·3 to 53·4)	27·5% (23·0 to 32·3)	3·76% (2·46 to 5·04)	0·56% (−0·70 to 1·81)	−0·83% (−2·07 to 0·40)
Bermuda	10 802 (9469 to 12 352)	6982 (6120 to 7983)	11·5% (10·1 to 13·2)	29·1% (25·1 to 33·0)	10·2% (7·7 to 13·4)	0·0% (0·0 to 0·0)	3·05% (1·35 to 4·55)	1·93% (0·25 to 3·41)	0·87% (−0·80 to 2·33)
Bhutan	84 (69 to 100)	258 (213 to 306)	2·5% (2·1 to 3·0)	72·7% (65·4 to 78·9)	20·0% (14·5 to 27·3)	6·1% (5·1 to 7·3)	4·75% (3·41 to 6·13)	2·62% (1·31 to 3·97)	−2·38% (−3·63 to −1·10)
Bolivia	214 (185 to 246)	486 (420 to 558)	6·7% (5·8 to 7·7)	66·7% (59·7 to 73·1)	28·1% (21·7 to 35·3)	1·8% (1·6 to 2·1)	6·83% (5·69 to 7·94)	4·94% (3·83 to 6·04)	2·52% (1·43 to 3·59)
Bosnia and Herzegovina	517 (473 to 569)	1251 (1144 to 1376)	8·0% (7·3 to 8·7)	68·5% (64·1 to 72·6)	27·6% (23·6 to 32·0)	2·0% (1·8 to 2·2)	8·31% (7·40 to 9·21)	8·48% (7·57 to 9·39)	0·42% (−0·43 to 1·25)
Botswana	427 (380 to 478)	1000 (890 to 1119)	4·4% (3·9 to 4·9)	54·5% (48·7 to 60·2)	5·3% (3·8 to 7·2)	8·4% (7·5 to 9·4)	3·73% (2·97 to 4·55)	1·82% (1·07 to 2·63)	−0·99% (−1·71 to −0·20)
Brazil	1114 (1040 to 1195)	1864 (1739 to 2000)	8·0% (7·5 to 8·6)	33·3% (30·1 to 36·2)	43·9% (40·5 to 47·5)	0·1% (0·1 to 0·1)	4·58% (4·03 to 5·21)	3·35% (2·80 to 3·97)	2·21% (1·67 to 2·82)
Brunei	770 (693 to 849)	1914 (1725 to 2111)	1·7% (1·5 to 1·8)	90·5% (87·0 to 93·1)	5·3% (4·3 to 6·8)	0·0% (0·0 to 0·0)	−0·36% (−0·96 to 0·24)	−2·11% (−2·70 to −1·52)	−1·20% (−1·80 to −0·61)
Bulgaria	681 (630 to 733)	1786 (1653 to 1922)	6·8% (6·3 to 7·4)	50·9% (46·9 to 54·6)	47·4% (43·8 to 51·6)	0·1% (0·1 to 0·2)	5·65% (4·94 to 6·31)	6·38% (5·66 to 7·04)	2·91% (2·21 to 3·55)
Burkina Faso	37 (32 to 44)	103 (88 to 121)	4·4% (3·8 to 5·2)	35·9% (28·2 to 43·5)	35·4% (27·8 to 44·3)	22·3% (18·9 to 25·9)	6·61% (5·43 to 7·90)	3·51% (2·37 to 4·77)	0·55% (−0·56 to 1·77)
Burundi	28 (25 to 31)	61 (55 to 69)	10·3% (9·3 to 11·6)	26·3% (21·1 to 32·1)	24·9% (19·2 to 31·9)	47·2% (42·0 to 52·2)	3·97% (2·89 to 5·03)	0·90% (−0·16 to 1·92)	1·51% (0·45 to 2·54)
Cambodia	76 (62 to 93)	225 (186 to 277)	5·9% (4·8 to 7·2)	23·4% (17·6 to 30·1)	63·2% (55·5 to 70·4)	12·8% (10·3 to 15·4)	5·09% (3·89 to 6·38)	2·91% (1·74 to 4·18)	−2·56% (−3·67 to −1·36)
Cameroon	58 (46 to 74)	148 (118 to 187)	3·2% (2·6 to 4·1)	15·0% (10·6 to 20·2)	73·3% (66·2 to 79·7)	9·2% (7·1 to 11·4)	4·31% (2·71 to 6·08)	1·56% (−0·01 to 3·27)	−0·08% (−1·61 to 1·61)
Canada	4875 (4773 to 4991)	5217 (5108 to 5341)	8·0% (7·9 to 8·2)	73·5% (72·6 to 74·4)	14·6% (13·9 to 15·3)	0·0% (0·0 to 0·0)	3·51% (3·31 to 3·72)	2·44% (2·25 to 2·66)	1·03% (0·84 to 1·24)
Cape Verde	157 (134 to 182)	330 (282 to 383)	3·7% (3·2 to 4·3)	64·8% (57·8 to 71·4)	27·4% (21·0 to 34·5)	5·4% (4·6 to 6·2)	4·98% (3·80 to 6·16)	3·24% (2·08 to 4·40)	−0·77% (−1·89 to 0·34)
Central African Republic	22 (19 to 25)	37 (33 to 43)	5·6% (4·9 to 6·4)	13·5% (10·1 to 17·3)	36·3% (28·6 to 44·5)	49·2% (42·9 to 55·6)	1·48% (0·37 to 2·65)	−0·55% (−1·64 to 0·60)	1·29% (0·17 to 2·45)
Chad	36 (29 to 44)	99 (81 to 120)	3·1% (2·5 to 3·8)	21·9% (16·0 to 28·6)	58·0% (48·9 to 66·8)	14·8% (12·1 to 18·0)	3·83% (2·39 to 5·36)	0·18% (−1·20 to 1·67)	−2·73% (−4·07 to −1·29)
Chile	1244 (1193 to 1294)	2199 (2109 to 2288)	6·8% (6·6 to 7·1)	58·5% (56·3 to 60·7)	34·7% (32·6 to 36·7)	0·0% (0·0 to 0·0)	5·78% (5·32 to 6·22)	4·55% (4·10 to 4·99)	1·57% (1·13 to 2·00)
China	436 (391 to 487)	808 (723 to 902)	5·0% (4·5 to 5·6)	58·8% (53·3 to 64·2)	35·3% (30·3 to 40·1)	0·0% (0·0 to 0·0)	10·84% (9·66 to 12·04)	10·25% (9·08 to 11·44)	1·53% (0·46 to 2·63)
Colombia	358 (315 to 399)	853 (751 to 950)	3·9% (3·4 to 4·3)	65·1% (59·1 to 71·3)	20·6% (16·3 to 25·5)	0·1% (0·1 to 0·1)	2·06% (1·19 to 2·90)	0·81% (−0·05 to 1·64)	−1·24% (−2·08 to −0·43)
Comoros	80 (66 to 96)	157 (130 to 189)	6·3% (5·2 to 7·6)	12·8% (9·4 to 16·9)	68·4% (61·8 to 74·2)	17·7% (14·6 to 21·2)	0·85% (−0·34 to 2·07)	−1·58% (−2·74 to −0·39)	−1·56% (−2·72 to −0·37)
Congo (Brazzaville)	79 (65 to 94)	235 (194 to 281)	2·0% (1·7 to 2·4)	46·9% (37·7 to 56·4)	44·6% (35·4 to 54·2)	4·4% (3·6 to 5·2)	5·76% (4·49 to 7·15)	3·07% (1·83 to 4·43)	2·22% (1·00 to 3·57)
Costa Rica	948 (891 to 1002)	1416 (1331 to 1498)	8·1% (7·6 to 8·5)	72·7% (69·6 to 75·7)	22·1% (19·4 to 25·0)	2·5% (2·3 to 2·6)	5·72% (5·18 to 6·25)	4·11% (3·57 to 4·62)	1·53% (1·01 to 2·03)
Côte d'Ivoire	77 (63 to 92)	178 (147 to 214)	4·1% (3·4 to 5·0)	23·6% (17·9 to 29·8)	43·3% (34·3 to 52·4)	14·5% (11·9 to 17·4)	2·18% (0·87 to 3·41)	−0·19% (−1·47 to 1·02)	−0·97% (−2·23 to 0·23)
Croatia	939 (885 to 1005)	1707 (1609 to 1828)	5·5% (5·2 to 5·9)	77·7% (74·3 to 80·5)	15·2% (12·8 to 17·4)	1·0% (0·9 to 1·1)	2·34% (1·81 to 2·86)	2·78% (2·25 to 3·30)	0·25% (−0·26 to 0·76)
Cuba	1128 (1047 to 1228)	2470 (2292 to 2689)	15·0% (13·9 to 16·3)	83·3% (77·8 to 87·4)	9·3% (fv7·4 to 11·4)	0·1% (0·1 to 0·1)	8·39% (7·56 to 9·18)	8·14% (7·31 to 8·93)	4·05% (3·26 to 4·81)
Cyprus	1226 (1161 to 1293)	1712 (1622 to 1805)	3·9% (3·7 to 4·1)	42·8% (40·3 to 45·3)	45·3% (42·4 to 48·1)	0·0% (0·0 to 0·0)	3·62% (3·16 to 4·03)	2·22% (1·77 to 2·63)	1·46% (1·01 to 1·86)
Czech Republic	1515 (1457 to 1578)	2511 (2414 to 2615)	5·7% (5·5 to 6·0)	82·0% (80·3 to 83·9)	14·8% (13·4 to 16·5)	0·0% (0·0 to 0·0)	3·38% (2·99 to 3·76)	3·22% (2·83 to 3·60)	0·84% (0·47 to 1·22)
Democratic Republic of the Congo	19 (17 to 23)	30 (26 to 36)	4·0% (3·4 to 4·7)	14·8% (11·1 to 19·2)	41·2% (32·6 to 50·1)	36·0% (30·3 to 41·5)	5·25% (3·66 to 6·87)	1·99% (0·45 to 3·56)	1·92% (0·38 to 3·49)
Denmark	6195 (6033 to 6363)	5240 (5103 to 5382)	8·6% (8·4 to 8·8)	84·1% (83·4 to 84·9)	13·7% (13·1 to 14·3)	0·0% (0·0 to 0·0)	2·89% (2·65 to 3·12)	2·45% (2·21 to 2·68)	1·42% (1·19 to 1·65)
Djibouti	66 (57 to 77)	124 (107 to 144)	3·6% (3·1 to 4·2)	52·7% (45·1 to 60·2)	23·5% (16·9 to 30·9)	22·9% (19·6 to 26·5)	1·46% (0·26 to 2·60)	−0·17% (−1·35 to 0·94)	−1·28% (−2·45 to −0·18)
Dominica	438 (397 to 479)	638 (580 to 698)	5·5% (5·0 to 6·0)	66·4% (62·0 to 70·7)	31·4% (27·2 to 35·8)	0·8% (0·7 to 0·9)	1·45% (0·76 to 2·09)	1·13% (0·44 to 1·77)	−0·60% (−1·28 to 0·03)
Dominican Republic	420 (377 to 467)	995 (894 to 1107)	5·1% (4·6 to 5·7)	45·3% (40·3 to 50·7)	44·1% (38·4 to 49·9)	1·5% (1·3 to 1·6)	6·45% (5·56 to 7·35)	4·98% (4·10 to 5·86)	1·05% (0·20 to 1·90)
Ecuador	536 (489 to 586)	1015 (925 to 1110)	8·7% (8·0 to 9·6)	51·1% (46·4 to 55·8)	41·4% (36·8 to 46·4)	0·2% (0·2 to 0·2)	6·50% (5·71 to 7·34)	4·69% (3·92 to 5·52)	3·21% (2·44 to 4·02)
Egypt	125 (103 to 150)	577 (477 to 695)	3·7% (3·1 to 4·5)	31·5% (24·5 to 38·8)	60·2% (51·8 to 68·5)	0·5% (0·4 to 0·6)	3·45% (2·15 to 4·75)	1·54% (0·26 to 2·82)	−0·86% (−2·11 to 0·39)
El Salvador	313 (279 to 349)	656 (585 to 732)	7·2% (6·4 to 8·0)	64·4% (58·5 to 69·7)	27·6% (22·7 to 32·9)	1·9% (1·7 to 2·1)	2·31% (1·46 to 3·09)	1·82% (0·98 to 2·61)	0·29% (−0·54 to 1·06)
Equatorial Guinea	310 (275 to 351)	797 (708 to 903)	1·6% (1·4 to 1·8)	21·5% (17·4 to 25·8)	71·6% (66·4 to 76·1)	2·8% (2·4 to 3·1)	9·04% (7·92 to 10·17)	5·74% (4·65 to 6·84)	−6·21% (−7·17 to −5·24)
Eritrea	30 (24 to 37)	46 (37 to 57)	4·4% (3·5 to 5·4)	20·3% (14·9 to 26·9)	63·0% (53·8 to 70·9)	14·8% (11·8 to 18·1)	0·24% (−1·19 to 1·71)	−2·20% (−3·60 to −0·77)	−2·07% (−3·47 to −0·63)
Estonia	1392 (1338 to 1451)	2051 (1972 to 2137)	6·2% (5·9 to 6·4)	75·5% (73·6 to 77·3)	22·7% (21·0 to 24·5)	0·0% (0·0 to 0·0)	3·80% (3·37 to 4·24)	4·28% (3·85 to 4·72)	−0·14% (−0·55 to 0·28)
eSwatini	329 (297 to 365)	876 (792 to 972)	6·6% (5·9 to 7·3)	60·3% (55·8 to 64·8)	9·8% (7·0 to 13·2)	22·5% (20·3 to 24·9)	6·39% (5·29 to 7·50)	4·63% (3·54 to 5·71)	2·87% (1·80 to 3·94)
Ethiopia	31 (26 to 37)	83 (70 to 99)	5·4% (4·6 to 6·5)	22·6% (17·1 to 28·9)	34·2% (25·5 to 43·5)	26·3% (21·7 to 30·7)	8·94% (7·61 to 10·35)	5·83% (4·53 to 7·19)	0·55% (−0·68 to 1·85)
Federated States of Micronesia	130 (109 to 154)	144 (121 to 171)	3·9% (3·3 to 4·7)	84·1% (79·9 to 87·5)	7·7% (5·2 to 11·1)	8·1% (6·8 to 9·6)	1·56% (0·33 to 2·77)	1·79% (0·56 to 3·00)	1·54% (0·31 to 2·75)
Fiji	200 (173 to 234)	350 (303 to 408)	3·6% (3·1 to 4·2)	61·8% (53·9 to 68·8)	20·3% (15·0 to 27·0)	4·5% (3·9 to 5·2)	3·13% (2·05 to 4·22)	2·48% (1·41 to 3·57)	1·06% (0·01 to 2·14)
Finland	4656 (4550 to 4764)	4235 (4139 to 4333)	8·4% (8·2 to 8·6)	77·4% (76·4 to 78·3)	20·2% (19·4 to 21·1)	0·0% (0·0 to 0·0)	3·37% (3·16 to 3·60)	3·00% (2·79 to 3·23)	1·24% (1·03 to 1·47)
France	4945 (4826 to 5063)	5148 (5023 to 5270)	9·8% (9·5 to 10·0)	80·6% (79·2 to 81·9)	9·6% (9·0 to 10·2)	0·0% (0·0 to 0·0)	2·45% (2·23 to 2·65)	1·88% (1·67 to 2·09)	0·87% (0·65 to 1·07)
Gabon	281 (245 to 321)	649 (566 to 742)	2·2% (1·9 to 2·5)	62·1% (55·4 to 68·3)	24·4% (19·2 to 29·9)	1·1% (1·0 to 1·3)	1·57% (0·72 to 2·42)	−0·82% (−1·65 to 0·00)	−0·01% (−0·84 to 0·83)
Georgia	319 (282 to 360)	851 (751 to 959)	6·1% (5·4 to 6·9)	34·0% (28·4 to 39·6)	59·2% (53·1 to 65·0)	1·3% (1·2 to 1·5)	8·15% (6·87 to 9·40)	9·29% (8·00 to 10·55)	2·21% (1·00 to 3·39)
Germany	5263 (5095 to 5435)	5619 (5440 to 5803)	9·6% (9·3 to 9·9)	84·6% (83·5 to 85·7)	12·4% (11·8 to 13·1)	0·0% (0·0 to 0·0)	1·26% (1·01 to 1·52)	1·20% (0·95 to 1·46)	−0·12% (−0·37 to 0·13)
Ghana	75 (63 to 88)	210 (176 to 247)	3·6% (3·0 to 4·2)	39·9% (32·0 to 47·5)	39·4% (31·3 to 48·2)	13·7% (11·5 to 16·2)	6·39% (5·05 to 7·71)	3·75% (2·44 to 5·03)	0·57% (−0·70 to 1·81)
Greece	1693 (1601 to 1790)	2392 (2263 to 2529)	6·4% (6·0 to 6·7)	59·7% (56·8 to 62·7)	35·6% (32·6 to 38·3)	0·0% (0·0 to 0·0)	1·17% (0·76 to 1·57)	1·06% (0·65 to 1·46)	0·47% (0·06 to 0·86)
Greenland	4457 (4203 to 4731)	3516 (3316 to 3732)	8·1% (7·6 to 8·6)	100·0% (100·0 to 100·0)	0·0% (0·0 to 0·0)	0·0% (0·0 to 0·0)	2·51% (1·30 to 3·60)	2·52% (1·32 to 3·61)	−0·04% (−1·22 to 1·02)
Grenada	486 (438 to 536)	723 (652 to 797)	5·0% (4·5 to 5·5)	40·9% (36·2 to 45·7)	58·6% (53·9 to 63·3)	0·5% (0·4 to 0·5)	0·82% (0·13 to 1·51)	0·56% (−0·12 to 1·25)	−2·31% (−2·98 to −1·64)
Guam	1990 (1548 to 2480)	1990 (1548 to 2480)	5·5% (4·3 to 6·9)	87·4% (81·7 to 91·6)	8·8% (5·8 to 12·8)	0·0% (0·0 to 0·0)	2·88% (1·09 to 4·53)	2·01% (0·23 to 3·65)	0·97% (−0·79 to 2·59)
Guatemala	262 (227 to 301)	479 (415 to 550)	6·8% (5·9 to 7·8)	36·6% (30·7 to 42·9)	54·8% (47·5 to 61·1)	1·2% (1·1 to 1·4)	5·07% (4·03 to 6·06)	2·77% (1·75 to 3·74)	1·46% (0·46 to 2·42)
Guinea	44 (37 to 53)	119 (99 to 143)	6·0% (5·0 to 7·2)	11·1% (8·0 to 15·1)	53·4% (44·3 to 62·2)	25·7% (21·0 to 30·4)	6·11% (4·78 to 7·58)	3·49% (2·19 to 4·92)	1·82% (0·54 to 3·23)
Guinea-Bissau	49 (43 to 57)	110 (95 to 128)	6·1% (5·3 to 7·1)	34·4% (28·0 to 41·5)	33·9% (26·3 to 41·9)	31·7% (27·2 to 36·4)	1·71% (0·76 to 2·71)	−0·66% (−1·59 to 0·31)	−0·70% (−1·62 to 0·28)
Guyana	208 (180 to 239)	377 (327 to 434)	4·5% (3·9 to 5·2)	56·6% (49·8 to 63·7)	38·5% (31·4 to 45·4)	4·8% (4·2 to 5·5)	3·12% (2·06 to 4·21)	3·02% (1·96 to 4·11)	0·11% (−0·92 to 1·17)
Haiti	47 (42 to 54)	113 (100 to 130)	5·4% (4·7 to 6·1)	13·1% (9·8 to 16·7)	35·6% (28·1 to 43·7)	47·1% (40·9 to 52·9)	0·55% (−0·34 to 1·54)	−1·13% (−2·01 to −0·16)	−1·08% (−1·96 to −0·11)
Honduras	193 (165 to 222)	401 (343 to 462)	7·2% (6·1 to 8·3)	43·1% (36·2 to 50·6)	47·3% (39·3 to 54·7)	3·2% (2·7 to 3·7)	5·18% (4·04 to 6·28)	3·15% (2·03 to 4·23)	1·54% (0·44 to 2·60)
Hungary	1029 (976 to 1081)	2133 (2024 to 2242)	5·8% (5·5 to 6·1)	66·1% (63·5 to 68·6)	29·3% (27·0 to 31·7)	0·0% (0·0 to 0·0)	2·34% (1·91 to 2·77)	2·54% (2·10 to 2·97)	0·04% (−0·39 to 0·46)
Iceland	6307 (6123 to 6494)	4347 (4220 to 4476)	10·6% (10·3 to 10·9)	81·4% (80·4 to 82·3)	17·0% (16·1 to 18·0)	0·0% (0·0 to 0·0)	3·52% (3·26 to 3·79)	2·47% (2·22 to 2·74)	0·06% (−0·18 to 0·33)
India	65 (52 to 80)	247 (199 to 305)	3·0% (2·4 to 3·6)	25·4% (18·5 to 33·4)	64·2% (54·2 to 72·6)	0·9% (0·7 to 1·0)	6·07% (4·48 to 7·77)	4·46% (2·90 to 6·14)	−0·84% (−2·32 to 0·75)
Indonesia	116 (96 to 141)	388 (321 to 470)	2·3% (1·9 to 2·8)	40·3% (31·6 to 49·4)	40·1% (31·0 to 49·5)	0·7% (0·6 to 0·8)	5·94% (4·38 to 7·43)	4·59% (3·05 to 6·06)	1·70% (0·20 to 3·13)
Iran	420 (375 to 471)	1707 (1524 to 1915)	4·8% (4·3 to 5·4)	50·5% (44·5 to 56·1)	37·6% (31·9 to 43·4)	0·0% (0·0 to 0·0)	7·80% (6·92 to 8·76)	6·31% (5·44 to 7·25)	4·27% (3·41 to 5·19)
Iraq	157 (133 to 187)	505 (427 to 601)	2·0% (1·7 to 2·4)	26·2% (20·3 to 32·5)	73·5% (67·2 to 79·4)	0·3% (0·2 to 0·3)	10·14% (8·68 to 11·74)	6·70% (5·28 to 8·25)	1·56% (0·20 to 3·03)
Ireland	5097 (4901 to 5288)	5194 (4995 to 5389)	6·2% (5·9 to 6·4)	71·9% (70·4 to 73·4)	13·2% (12·3 to 14·2)	0·0% (0·0 to 0·0)	6·60% (6·12 to 7·05)	5·33% (4·86 to 5·78)	0·92% (0·47 to 1·35)
Israel	2757 (2684 to 2827)	2597 (2528 to 2663)	6·7% (6·5 to 6·9)	63·6% (62·2 to 65·1)	23·2% (21·9 to 24·4)	0·0% (0·0 to 0·0)	3·75% (3·52 to 3·97)	1·65% (1·43 to 1·87)	0·03% (−0·20 to 0·24)
Italy	3059 (2976 to 3141)	3462 (3368 to 3555)	7·4% (7·2 to 7·6)	74·4% (73·3 to 75·6)	23·1% (22·0 to 24·2)	0·0% (0·0 to 0·0)	1·70% (1·48 to 1·89)	1·40% (1·19 to 1·60)	1·15% (0·93 to 1·34)
Jamaica	314 (273 to 357)	569 (496 to 647)	5·4% (4·7 to 6·1)	60·0% (53·1 to 66·5)	21·0% (16·5 to 26·4)	1·7% (1·5 to 2·0)	1·76% (0·86 to 2·61)	1·16% (0·26 to 2·00)	1·33% (0·43 to 2·17)
Japan	4175 (4065 to 4278)	4667 (4543 to 4782)	7·2% (7·0 to 7·4)	83·7% (82·7 to 84·6)	13·3% (12·6 to 14·1)	0·0% (0·0 to 0·0)	3·94% (3·67 to 4·20)	3·89% (3·61 to 4·15)	3·07% (2·80 to 3·33)
Jordan	224 (198 to 253)	509 (450 to 574)	5·1% (4·6 to 5·8)	65·7% (59·8 to 70·7)	26·2% (21·5 to 31·4)	2·1% (1·8 to 2·3)	1·86% (1·06 to 2·67)	−0·93% (−1·70 to −0·14)	−2·26% (−3·03 to −1·48)
Kazakhstan	295 (260 to 335)	868 (763 to 983)	2·1% (1·8 to 2·3)	61·3% (54·6 to 68·1)	32·6% (26·3 to 39·0)	0·8% (0·7 to 0·9)	2·95% (2·02 to 3·84)	2·43% (1·50 to 3·31)	−2·53% (−3·41 to −1·69)
Kenya	82 (70 to 96)	168 (143 to 196)	6·3% (5·4 to 7·4)	33·9% (26·3 to 41·6)	27·1% (20·1 to 35·0)	23·9% (20·3 to 27·8)	4·09% (2·87 to 5·39)	1·51% (0·32 to 2·78)	0·19% (−0·99 to 1·44)
Kiribati	198 (176 to 224)	233 (207 to 263)	9·1% (8·1 to 10·3)	64·6% (59·1 to 69·6)	13·6% (9·8 to 18·1)	17·8% (15·7 to 20·0)	2·45% (1·55 to 3·44)	0·78% (−0·11 to 1·75)	0·55% (−0·33 to 1·52)
Kuwait	1279 (1140 to 1433)	2959 (2637 to 3314)	2·7% (2·4 to 3·1)	83·2% (80·3 to 85·8)	15·2% (12·8 to 17·9)	0·0% (0·0 to 0·0)	3·56% (2·79 to 4·33)	−0·58% (−1·32 to 0·16)	−0·02% (−0·76 to 0·73)
Kyrgyzstan	79 (65 to 96)	262 (217 to 318)	5·5% (4·6 to 6·7)	40·0% (31·7 to 48·8)	52·4% (43·3 to 61·3)	7·6% (6·2 to 9·1)	4·48% (3·17 to 5·80)	3·14% (1·85 to 4·45)	−0·09% (−1·34 to 1·18)
Laos	52 (43 to 62)	157 (130 to 189)	2·4% (2·0 to 2·9)	33·4% (24·8 to 41·7)	48·9% (39·0 to 58·6)	14·3% (11·8 to 17·3)	4·38% (3·03 to 5·82)	2·34% (1·02 to 3·75)	−2·81% (−4·07 to −1·47)
Latvia	995 (943 to 1045)	1635 (1549 to 1717)	5·4% (5·1 to 5·6)	55·1% (52·7 to 57·7)	43·9% (41·4 to 46·3)	0·0% (0·0 to 0·0)	4·29% (3·76 to 4·82)	5·41% (4·88 to 5·95)	0·25% (−0·26 to 0·76)
Lebanon	486 (437 to 540)	852 (766 to 946)	5·3% (4·8 to 5·9)	51·4% (46·4 to 56·4)	32·5% (28·3 to 37·1)	0·5% (0·5 to 0·6)	1·90% (1·25 to 2·57)	−1·22% (−1·85 to −0·57)	−2·11% (−2·74 to −1·46)
Lesotho	122 (107 to 139)	323 (282 to 367)	7·0% (6·1 to 7·9)	55·8% (49·8 to 61·7)	15·5% (11·2 to 20·5)	27·3% (23·9 to 31·0)	6·96% (5·82 to 8·17)	5·86% (4·73 to 7·06)	2·81% (1·71 to 3·97)
Liberia	81 (71 to 94)	179 (157 to 208)	14·7% (12·9 to 17·1)	9·6% (7·0 to 12·6)	42·3% (34·1 to 50·9)	42·2% (36·2 to 48·0)	14·61% (12·99 to 16·34)	10·42% (8·85 to 12·08)	4·22% (2·75 to 5·80)
Libya	257 (222 to 294)	467 (404 to 535)	4·6% (4·0 to 5·3)	65·8% (58·5 to 72·1)	29·2% (23·0 to 35·7)	0·3% (0·2 to 0·3)	−1·18% (−2·01 to −0·35)	−2·27% (−3·10 to −1·45)	1·83% (0·96 to 2·68)
Lithuania	1121 (1069 to 1176)	2044 (1949 to 2144)	5·7% (5·4 to 6·0)	66·1% (63·8 to 68·4)	32·5% (30·3 to 34·8)	0·0% (0·0 to 0·0)	5·50% (4·90 to 6·10)	6·63% (6·03 to 7·24)	1·22% (0·65 to 1·80)
Luxembourg	7027 (6713 to 7360)	6677 (6379 to 6994)	5·2% (5·0 to 5·4)	82·4% (80·9 to 83·9)	11·3% (10·1 to 12·6)	0·0% (0·0 to 0·0)	4·75% (4·30 to 5·20)	3·00% (2·55 to 3·44)	1·15% (0·71 to 1·58)
Macedonia	364 (326 to 404)	949 (849 to 1053)	5·6% (5·0 to 6·2)	63·5% (57·8 to 68·7)	34·5% (29·3 to 40·2)	0·3% (0·3 to 0·3)	1·14% (0·36 to 1·92)	0·87% (0·10 to 1·65)	−1·68% (−2·43 to −0·93)
Madagascar	23 (20 to 27)	81 (68 to 94)	4·1% (3·5 to 4·8)	46·6% (38·4 to 55·5)	27·1% (19·3 to 35·2)	19·1% (16·2 to 22·3)	3·45% (2·28 to 4·58)	0·45% (−0·68 to 1·55)	0·52% (−0·61 to 1·62)
Malawi	39 (36 to 42)	141 (130 to 153)	6·6% (6·1 to 7·2)	23·4% (18·5 to 28·2)	9·8% (6·9 to 13·2)	61·0% (56·1 to 66·0)	8·37% (7·56 to 9·14)	5·36% (4·57 to 6·10)	3·88% (3·10 to 4·61)
Malaysia	407 (366 to 455)	1151 (1032 to 1284)	3·0% (2·7 to 3·3)	52·2% (46·6 to 57·8)	36·2% (30·7 to 41·8)	0·0% (0·0 to 0·0)	6·96% (6·06 to 7·83)	4·96% (4·08 to 5·82)	2·20% (1·34 to 3·03)
Maldives	974 (903 to 1047)	1539 (1426 to 1653)	10·0% (9·3 to 10·8)	70·5% (67·1 to 73·8)	20·1% (17·6 to 22·9)	0·2% (0·2 to 0·2)	6·60% (5·91 to 7·23)	4·44% (3·77 to 5·06)	0·91% (0·26 to 1·51)
Mali	33 (28 to 38)	84 (73 to 97)	3·1% (2·7 to 3·6)	24·7% (19·0 to 30·6)	37·1% (29·6 to 46·1)	36·8% (31·8 to 42·3)	5·45% (4·28 to 6·61)	2·34% (1·20 to 3·46)	−0·64% (−1·75 to 0·44)
Malta	2799 (2725 to 2879)	4037 (3932 to 4154)	8·7% (8·5 to 9·0)	62·3% (60·9 to 63·7)	35·5% (34·2 to 36·8)	0·0% (0·0 to 0·0)	5·73% (5·42 to 6·05)	5·12% (4·81 to 5·44)	2·26% (1·96 to 2·57)
Marshall Islands	529 (480 to 586)	518 (470 to 574)	13·6% (12·3 to 15·0)	79·3% (75·2 to 83·1)	14·3% (11·2 to 17·9)	2·5% (2·3 to 2·8)	2·29% (1·58 to 2·99)	0·29% (−0·42 to 0·97)	−0·06% (−0·76 to 0·62)
Mauritania	56 (46 to 67)	191 (159 to 229)	3·2% (2·7 to 3·8)	36·9% (28·9 to 45·7)	50·4% (41·0 to 59·8)	8·4% (6·9 to 9·9)	2·92% (1·68 to 4·27)	0·10% (−1·11 to 1·41)	−1·19% (−2·38 to 0·10)
Mauritius	557 (510 to 610)	1237 (1132 to 1354)	4·6% (4·2 to 5·0)	44·1% (39·6 to 48·6)	49·3% (44·7 to 53·9)	0·2% (0·1 to 0·2)	8·11% (7·22 to 8·98)	7·48% (6·61 to 8·35)	3·60% (2·76 to 4·44)
Mexico	505 (458 to 554)	1101 (1000 to 1209)	4·2% (3·8 to 4·6)	52·5% (47·8 to 57·2)	40·0% (35·3 to 44·4)	0·1% (0·1 to 0·1)	4·10% (3·34 to 4·82)	2·64% (1·89 to 3·35)	1·25% (0·51 to 1·94)
Moldova	204 (177 to 235)	498 (432 to 574)	8·1% (7·0 to 9·3)	50·2% (42·5 to 57·5)	45·4% (38·1 to 53·0)	3·2% (2·8 to 3·7)	3·19% (2·11 to 4·31)	3·50% (2·42 to 4·63)	0·33% (−0·72 to 1·42)
Mongolia	150 (129 to 175)	506 (436 to 590)	2·8% (2·4 to 3·2)	52·2% (44·3 to 59·8)	35·5% (28·1 to 43·2)	9·1% (7·7 to 10·5)	6·11% (4·95 to 7·34)	4·71% (3·56 to 5·93)	−0·01% (−1·11 to 1·14)
Montenegro	603 (554 to 656)	1325 (1218 to 1442)	6·8% (6·2 to 7·4)	74·4% (70·3 to 78·0)	24·6% (21·0 to 28·7)	0·6% (0·6 to 0·7)	0·40% (−0·16 to 0·99)	0·35% (−0·21 to 0·94)	−3·12% (−3·66 to −2·55)
Morocco	185 (159 to 216)	500 (431 to 584)	4·8% (4·1 to 5·6)	43·7% (36·2 to 51·0)	48·6% (41·0 to 56·1)	3·7% (3·1 to 4·3)	7·89% (6·59 to 9·17)	6·81% (5·53 to 8·09)	3·55% (2·31 to 4·79)
Mozambique	32 (31 to 35)	92 (87 to 98)	4·6% (4·4 to 4·9)	19·5% (15·5 to 24·2)	5·5% (4·0 to 7·6)	73·3% (68·7 to 77·2)	8·52% (7·86 to 9·11)	5·40% (4·76 to 5·97)	−0·03% (−0·64 to 0·51)
Myanmar	59 (48 to 75)	302 (243 to 383)	3·3% (2·7 to 4·2)	19·6% (14·0 to 26·2)	71·0% (63·2 to 78·1)	9·4% (7·3 to 11·5)	13·54% (11·61 to 15·67)	12·46% (10·55 to 14·58)	3·79% (2·02 to 5·74)
Namibia	512 (462 to 568)	1119 (1009 to 1242)	7·1% (6·4 to 7·8)	58·7% (53·4 to 63·6)	8·0% (5·9 to 10·5)	6·7% (6·0 to 7·4)	3·89% (3·13 to 4·63)	1·89% (1·14 to 2·61)	−0·49% (−1·22 to 0·22)
Nepal	48 (38 to 60)	153 (120 to 193)	5·4% (4·3 to 6·9)	18·5% (13·4 to 24·6)	60·1% (50·1 to 69·1)	8·2% (6·4 to 10·2)	6·14% (4·44 to 7·80)	4·42% (2·76 to 6·06)	1·79% (0·17 to 3·38)
Netherlands	5329 (5132 to 5527)	5603 (5396 to 5812)	8·6% (8·3 to 9·0)	80·7% (78·8 to 82·5)	11·7% (10·7 to 12·8)	0·0% (0·0 to 0·0)	3·11% (2·77 to 3·43)	2·60% (2·25 to 2·91)	1·11% (0·76 to 1·41)
New Zealand	4276 (4168 to 4376)	4002 (3901 to 4096)	9·2% (8·9 to 9·4)	78·7% (77·6 to 79·7)	13·5% (12·7 to 14·4)	0·0% (0·0 to 0·0)	3·88% (3·66 to 4·11)	2·81% (2·59 to 3·04)	1·10% (0·88 to 1·32)
Nicaragua	184 (159 to 212)	502 (434 to 578)	8·0% (7·0 to 9·3)	56·2% (49·1 to 63·3)	32·7% (25·8 to 40·2)	9·0% (7·8 to 10·3)	4·76% (3·71 to 5·86)	3·27% (2·24 to 4·35)	0·57% (−0·43 to 1·63)
Niger	27 (22 to 33)	67 (55 to 82)	5·4% (4·4 to 6·5)	24·9% (18·5 to 31·6)	54·7% (46·1 to 63·5)	15·0% (12·2 to 18·1)	4·57% (3·12 to 6·01)	0·75% (−0·64 to 2·14)	−0·32% (−1·70 to 1·06)
Nigeria	71 (57 to 89)	199 (158 to 248)	2·4% (1·9 to 3·0)	14·5% (10·6 to 19·2)	75·2% (69·0 to 80·8)	8·6% (6·8 to 10·7)	6·75% (4·88 to 8·51)	4·01% (2·19 to 5·73)	0·81% (−0·95 to 2·47)
North Korea	66 (54 to 80)	44 (35 to 53)	5·8% (4·7 to 7·1)	61·9% (51·7 to 72·2)	36·8% (26·6 to 47·2)	0·3% (0·3 to 0·4)	0·92% (−0·45 to 2·37)	0·26% (−1·10 to 1·70)	0·31% (−1·06 to 1·75)
Northern Mariana Islands	261 (208 to 326)	261 (208 to 326)	1·2% (1·0 to 1·5)	84·2% (77·6 to 88·8)	14·6% (10·1 to 21·1)	0·0% (0·0 to 0·0)	−1·02% (−2·62 to 0·61)	−4·24% (−5·79 to −2·66)	−3·67% (−5·23 to −2·09)
Norway	8269 (7946 to 8608)	7708 (7407 to 8024)	7·1% (6·8 to 7·4)	85·2% (84·3 to 86·1)	14·5% (13·6 to 15·3)	0·0% (0·0 to 0·0)	4·03% (3·67 to 4·40)	3·10% (2·75 to 3·47)	1·89% (1·54 to 2·26)
Oman	764 (704 to 833)	1861 (1716 to 2029)	3·4% (3·1 to 3·7)	89·1% (86·6 to 91·2)	5·9% (4·5 to 7·4)	0·0% (0·0 to 0·0)	4·54% (3·86 to 5·24)	0·96% (0·31 to 1·64)	0·69% (0·03 to 1·37)
Pakistan	41 (33 to 51)	142 (115 to 177)	2·7% (2·2 to 3·3)	26·2% (19·7 to 34·4)	62·7% (53·1 to 71·0)	8·3% (6·6 to 10·2)	3·42% (1·96 to 4·98)	1·25% (−0·18 to 2·77)	−0·57% (−1·98 to 0·92)
Palestine	320 (277 to 373)	113 (98 to 131)	10·6% (9·1 to 12·3)	38·7% (32·5 to 45·0)	39·1% (32·7 to 45·7)	1·8% (1·6 to 2·1)	5·93% (4·69 to 7·18)	2·44% (1·24 to 3·64)	1·05% (−0·13 to 2·24)
Panama	1078 (1014 to 1142)	1872 (1759 to 1982)	8·1% (7·6 to 8·6)	64·6% (61·3 to 67·9)	28·6% (25·7 to 31·8)	0·1% (0·1 to 0·1)	6·11% (5·60 to 6·64)	4·23% (3·74 to 4·76)	0·11% (−0·37 to 0·62)
Papua New Guinea	59 (49 to 71)	73 (61 to 88)	1·8% (1·5 to 2·2)	72·8% (67·5 to 78·1)	7·4% (5·0 to 10·3)	18·4% (15·1 to 21·8)	4·15% (2·69 to 5·62)	1·70% (0·28 to 3·14)	0·85% (−0·56 to 2·27)
Paraguay	343 (302 to 392)	804 (706 to 916)	6·5% (5·7 to 7·4)	52·1% (45·7 to 58·0)	37·0% (31·2 to 43·3)	0·6% (0·5 to 0·6)	5·58% (4·61 to 6·49)	3·91% (2·95 to 4·81)	2·29% (1·35 to 3·18)
Peru	337 (299 to 378)	683 (605 to 765)	4·5% (4·0 to 5·1)	62·7% (56·4 to 68·9)	29·1% (23·5 to 34·8)	0·3% (0·2 to 0·3)	5·08% (4·16 to 5·99)	3·59% (2·68 to 4·49)	0·40% (−0·48 to 1·27)
Philippines	124 (101 to 151)	361 (294 to 441)	3·7% (3·0 to 4·5)	30·9% (23·7 to 39·0)	54·4% (44·9 to 63·0)	1·0% (0·8 to 1·2)	6·24% (4·85 to 7·62)	4·28% (2·93 to 5·64)	1·36% (0·05 to 2·69)
Poland	908 (863 to 956)	1857 (1765 to 1955)	5·1% (4·9 to 5·4)	69·9% (67·2 to 72·6)	23·2% (20·9 to 25·5)	0·0% (0·0 to 0·0)	4·98% (4·47 to 5·51)	4·92% (4·42 to 5·45)	0·85% (0·36 to 1·36)
Portugal	1954 (1882 to 2029)	2649 (2552 to 2751)	7·4% (7·1 to 7·7)	66·2% (64·4 to 67·8)	27·8% (26·3 to 29·4)	0·0% (0·0 to 0·0)	2·73% (2·41 to 3·04)	2·53% (2·21 to 2·84)	1·47% (1·16 to 1·77)
Puerto Rico	1364 (1210 to 1561)	1671 (1483 to 1913)	4·5% (3·9 to 5·1)	64·9% (56·7 to 72·3)	26·5% (19·5 to 34·1)	0·0% (0·0 to 0·0)	1·47% (0·31 to 2·66)	1·46% (0·30 to 2·65)	0·24% (−0·90 to 1·42)
Qatar	2064 (1900 to 2219)	4145 (3815 to 4456)	2·4% (2·2 to 2·5)	82·8% (80·6 to 84·9)	7·8% (6·4 to 9·3)	0·0% (0·0 to 0·0)	9·14% (8·51 to 9·77)	1·61% (1·02 to 2·20)	−0·47% (−1·04 to 0·11)
Romania	537 (490 to 587)	1181 (1077 to 1291)	4·3% (4·0 to 4·8)	78·2% (74·0 to 81·8)	20·8% (17·1 to 24·9)	0·1% (0·1 to 0·1)	4·56% (3·83 to 5·32)	5·41% (4·67 to 6·17)	1·88% (1·17 to 2·62)
Russia	574 (527 to 621)	1470 (1350 to 1592)	3·5% (3·2 to 3·8)	58·1% (53·9 to 62·6)	39·2% (34·7 to 43·4)	0·0% (0·0 to 0·0)	2·52% (1·87 to 3·22)	2·54% (1·89 to 3·24)	−0·62% (−1·25 to 0·06)
Rwanda	44 (39 to 50)	121 (107 to 138)	5·0% (4·4 to 5·7)	37·0% (30·6 to 44·2)	8·1% (5·9 to 11·1)	43·6% (37·9 to 49·0)	7·70% (6·47 to 8·86)	4·46% (3·26 to 5·59)	−0·21% (−1·36 to 0·87)
Saint Lucia	511 (464 to 559)	800 (726 to 875)	5·5% (5·0 to 6·0)	39·1% (34·8 to 43·4)	47·9% (43·4 to 52·6)	7·1% (6·4 to 7·8)	1·61% (0·97 to 2·23)	0·58% (−0·05 to 1·20)	−0·28% (−0·91 to 0·33)
Saint Vincent and the Grenadines	277 (245 to 310)	453 (400 to 507)	3·7% (3·3 to 4·2)	68·3% (62·9 to 73·2)	18·7% (14·3 to 23·8)	10·4% (9·3 to 11·7)	1·52% (0·72 to 2·37)	1·49% (0·68 to 2·33)	−0·72% (−1·51 to 0·10)
Samoa	232 (205 to 262)	320 (283 to 363)	4·9% (4·3 to 5·6)	76·7% (72·4 to 80·6)	12·2% (8·9 to 16·4)	10·1% (8·9 to 11·3)	3·30% (2·38 to 4·26)	2·61% (1·70 to 3·56)	0·42% (−0·48 to 1·35)
São Tomé and Príncipe	102 (90 to 114)	173 (154 to 195)	6·4% (5·7 to 7·2)	42·9% (37·1 to 48·8)	18·2% (13·7 to 23·7)	37·2% (32·9 to 41·8)	1·99% (1·06 to 2·89)	−0·18% (−1·09 to 0·70)	−1·78% (−2·68 to −0·93)
Saudi Arabia	1257 (1185 to 1336)	3200 (3018 to 3402)	4·5% (4·3 to 4·8)	69·5% (66·9 to 71·9)	14·2% (12·4 to 16·2)	0·0% (0·0 to 0·0)	6·77% (6·20 to 7·38)	4·30% (3·74 to 4·89)	3·65% (3·10 to 4·24)
Senegal	69 (57 to 83)	172 (143 to 207)	5·1% (4·3 to 6·2)	30·0% (22·9 to 37·9)	48·7% (39·4 to 58·3)	13·4% (11·1 to 16·0)	4·51% (3·24 to 5·93)	1·62% (0·39 to 3·00)	0·13% (−1·09 to 1·49)
Serbia	462 (420 to 504)	1121 (1018 to 1223)	6·1% (5·5 to 6·6)	58·0% (53·0 to 62·7)	40·0% (35·2 to 45·0)	0·5% (0·5 to 0·6)	4·99% (4·14 to 5·78)	5·58% (4·72 to 6·38)	1·95% (1·12 to 2·72)
Seychelles	534 (494 to 573)	1002 (926 to 1075)	3·5% (3·2 to 3·8)	97·8% (97·1 to 98·4)	2·1% (1·5 to 2·8)	0·1% (0·1 to 0·1)	0·65% (0·10 to 1·19)	−0·48% (−1·01 to 0·07)	−3·01% (−3·53 to −2·48)
Sierra Leone	82 (71 to 96)	257 (223 to 300)	14·9% (12·9 to 17·4)	9·8% (7·3 to 12·8)	46·4% (38·4 to 54·6)	39·0% (33·3 to 44·9)	5·74% (4·31 to 7·19)	2·97% (1·57 to 4·37)	2·33% (0·94 to 3·73)
Singapore	2580 (2486 to 2673)	4240 (4087 to 4393)	3·9% (3·8 to 4·1)	54·1% (52·4 to 55·9)	31·2% (29·8 to 32·7)	0·0% (0·0 to 0·0)	5·37% (5·00 to 5·73)	4·05% (3·67 to 4·40)	1·12% (0·76 to 1·46)
Slovakia	1325 (1275 to 1379)	2334 (2246 to 2428)	5·7% (5·5 to 6·0)	79·5% (77·5 to 81·4)	17·9% (16·0 to 19·8)	0·0% (0·0 to 0·0)	4·78% (4·39 to 5·18)	4·67% (4·29 to 5·07)	0·79% (0·42 to 1·18)
Slovenia	2090 (2027 to 2156)	2857 (2770 to 2947)	7·2% (7·0 to 7·4)	72·0% (70·2 to 73·6)	12·2% (11·1 to 13·4)	0·0% (0·0 to 0·0)	3·49% (3·18 to 3·78)	3·30% (3·00 to 3·60)	0·95% (0·65 to 1·24)
Solomon Islands	109 (96 to 124)	114 (99 to 129)	5·5% (4·8 to 6·3)	64·7% (59·7 to 69·2)	4·7% (3·2 to 6·6)	30·5% (26·8 to 34·8)	3·36% (2·19 to 4·43)	0·89% (−0·25 to 1·94)	1·09% (−0·05 to 2·14)
Somalia	15 (13 to 17)	30 (27 to 34)	15·6% (14·0 to 17·5)	20·0% (15·6 to 25·0)	28·7% (21·2 to 36·5)	49·8% (44·2 to 55·3)	3·63% (2·43 to 4·85)	1·00% (−0·16 to 2·19)	1·41% (0·25 to 2·61)
South Africa	512 (460 to 564)	1162 (1046 to 1282)	5·6% (5·1 to 6·2)	53·6% (48·5 to 58·8)	7·8% (5·7 to 10·0)	2·3% (2·1 to 2·6)	3·15% (2·33 to 3·90)	2·00% (1·18 to 2·74)	0·53% (−0·27 to 1·26)
South Korea	2150 (2088 to 2217)	2833 (2751 to 2922)	7·1% (6·9 to 7·3)	59·1% (57·6 to 60·4)	33·4% (32·0 to 34·8)	0·0% (0·0 to 0·0)	7·67% (7·28 to 8·09)	7·06% (6·67 to 7·48)	3·30% (2·92 to 3·70)
South Sudan	52 (44 to 62)	248 (208 to 293)	2·8% (2·4 to 3·3)	43·8% (35·2 to 52·3)	36·0% (26·9 to 46·2)	15·6% (13·1 to 18·5)	5·26% (3·85 to 6·66)	1·05% (−0·30 to 2·39)	0·53% (−0·81 to 1·86)
Spain	2687 (2608 to 2766)	3419 (3318 to 3519)	7·2% (7·0 to 7·4)	71·2% (69·8 to 72·5)	23·9% (22·7 to 25·1)	0·0% (0·0 to 0·0)	3·39% (3·12 to 3·64)	2·62% (2·35 to 2·88)	1·23% (0·96 to 1·48)
Sri Lanka	159 (134 to 188)	505 (427 to 596)	3·5% (3·0 to 4·2)	43·6% (35·7 to 51·3)	48·9% (41·0 to 57·3)	1·4% (1·2 to 1·7)	3·54% (2·31 to 4·75)	2·93% (1·70 to 4·13)	−1·61% (−2·79 to −0·46)
Sudan	113 (93 to 136)	265 (220 to 320)	5·1% (4·2 to 6·1)	23·2% (17·7 to 29·7)	69·2% (61·9 to 75·7)	3·9% (3·2 to 4·6)	5·25% (3·96 to 6·71)	2·70% (1·44 to 4·12)	0·04% (−1·19 to 1·42)
Suriname	417 (372 to 466)	939 (837 to 1047)	4·8% (4·3 to 5·4)	61·1% (55·1 to 66·6)	22·4% (18·1 to 27·2)	0·6% (0·6 to 0·7)	0·96% (0·20 to 1·71)	0·02% (−0·74 to 0·76)	−1·83% (−2·57 to −1·11)
Sweden	6095 (5899 to 6299)	5757 (5572 to 5950)	8·6% (8·3 to 8·8)	83·5% (82·5 to 84·3)	15·3% (14·5 to 16·1)	0·0% (0·0 to 0·0)	4·44% (4·08 to 4·79)	3·87% (3·50 to 4·21)	1·88% (1·52 to 2·21)
Switzerland	10 036 (9841 to 10 235)	7601 (7454 to 7752)	9·9% (9·7 to 10·1)	62·9% (62·0 to 63·7)	29·5% (28·7 to 30·2)	0·0% (0·0 to 0·0)	2·84% (2·68 to 3·01)	1·98% (1·82 to 2·16)	0·95% (0·78 to 1·12)
Syria	44 (36 to 53)	773 (631 to 934)	2·4% (2·0 to 2·9)	44·7% (35·1 to 53·8)	50·0% (40·6 to 59·9)	1·5% (1·3 to 1·9)	−2·10% (−3·30 to −0·81)	−3·18% (−4·37 to −1·91)	−1·90% (−3·11 to −0·62)
Taiwan (province of China)	1632 (1538 to 1726)	3118 (2938 to 3297)	6·4% (6·0 to 6·7)	59·6% (56·7 to 62·4)	36·9% (34·3 to 39·2)	0·0% (0·0 to 0·0)	5·31% (4·93 to 5·70)	4·76% (4·38 to 5·15)	1·14% (0·77 to 1·51)
Tajikistan	53 (43 to 66)	210 (169 to 261)	4·5% (3·6 to 5·6)	27·8% (20·7 to 35·2)	63·1% (54·9 to 71·3)	8·8% (7·0 to 10·8)	9·76% (8·29 to 11·41)	7·65% (6·20 to 9·26)	3·66% (2·27 to 5·21)
Tanzania	41 (36 to 46)	129 (116 to 147)	4·0% (3·6 to 4·6)	34·3% (27·9 to 40·8)	22·8% (16·8 to 30·0)	41·6% (36·5 to 46·3)	5·73% (4·45 to 6·98)	2·81% (1·56 to 4·01)	−0·41% (−1·61 to 0·76)
Thailand	231 (200 to 265)	654 (566 to 751)	3·2% (2·8 to 3·7)	77·3% (70·6 to 82·8)	12·3% (8·9 to 16·6)	0·3% (0·2 to 0·3)	3·80% (2·73 to 4·81)	3·22% (2·15 to 4·22)	0·70% (−0·34 to 1·68)
The Bahamas	1938 (1865 to 2020)	1976 (1901 to 2059)	6·6% (6·4 to 6·9)	49·9% (47·9 to 51·8)	27·7% (26·0 to 29·6)	0·0% (0·0 to 0·0)	2·64% (2·34 to 2·93)	0·95% (0·66 to 1·24)	0·99% (0·70 to 1·27)
The Gambia	29 (26 to 31)	104 (95 to 114)	4·8% (4·4 to 5·3)	16·2% (12·7 to 20·1)	18·2% (13·6 to 24·0)	56·9% (51·5 to 61·9)	5·24% (4·12 to 6·33)	2·11% (1·02 to 3·17)	1·75% (0·67 to 2·80)
Timor-Leste	85 (73 to 101)	209 (178 to 245)	2·0% (1·7 to 2·3)	65·2% (59·1 to 70·9)	10·6% (7·3 to 14·6)	22·9% (19·3 to 26·6)	6·79% (5·55 to 8·05)	5·07% (3·85 to 6·30)	1·21% (0·03 to 2·40)
Togo	41 (34 to 50)	108 (89 to 131)	5·6% (4·6 to 6·8)	21·9% (16·8 to 28·3)	54·8% (45·4 to 63·0)	14·6% (11·9 to 17·5)	5·06% (3·44 to 6·63)	2·31% (0·73 to 3·84)	2·05% (0·48 to 3·57)
Tonga	219 (196 to 245)	322 (287 to 360)	4·4% (3·9 to 4·9)	56·2% (50·9 to 61·2)	10·2% (7·2 to 13·4)	29·1% (25·9 to 32·6)	3·48% (2·56 to 4·40)	3·05% (2·13 to 3·96)	2·03% (1·12 to 2·93)
Trinidad and Tobago	1048 (983 to 1111)	2148 (2014 to 2278)	5·1% (4·8 to 5·4)	52·1% (49·4 to 55·0)	40·7% (38·0 to 43·3)	0·0% (0·0 to 0·0)	5·79% (5·13 to 6·42)	5·47% (4·81 to 6·09)	1·33% (0·69 to 1·93)
Tunisia	242 (211 to 275)	847 (738 to 963)	4·8% (4·2 to 5·5)	56·8% (49·5 to 63·7)	39·1% (32·1 to 46·0)	0·7% (0·6 to 0·8)	5·28% (4·22 to 6·29)	4·27% (3·22 to 5·27)	1·53% (0·51 to 2·50)
Turkey	445 (405 to 490)	1107 (1009 to 1220)	2·9% (2·6 to 3·2)	77·9% (73·4 to 82·1)	16·8% (13·2 to 20·8)	0·1% (0·1 to 0·1)	6·67% (5·84 to 7·46)	5·17% (4·35 to 5·94)	1·87% (1·08 to 2·63)
Turkmenistan	511 (462 to 565)	1382 (1249 to 1528)	5·8% (5·2 to 6·4)	21·2% (17·5 to 25·0)	73·6% (69·2 to 77·7)	0·4% (0·3 to 0·4)	7·02% (6·05 to 7·94)	5·63% (4·67 to 6·54)	−1·00% (−1·90 to −0·15)
Uganda	44 (38 to 50)	153 (134 to 177)	6·0% (5·2 to 6·9)	16·0% (12·2 to 20·2)	38·2% (30·1 to 46·3)	43·0% (37·1 to 49·0)	5·66% (4·44 to 6·85)	2·29% (1·11 to 3·43)	−0·68% (−1·83 to 0·43)
Ukraine	171 (146 to 197)	567 (485 to 654)	4·7% (4·0 to 5·4)	43·3% (36·3 to 50·6)	52·3% (44·8 to 59·5)	1·3% (1·1 to 1·5)	1·28% (0·20 to 2·31)	1·81% (0·73 to 2·85)	0·10% (−0·96 to 1·12)
United Arab Emirates	1440 (1346 to 1538)	2586 (2417 to 2762)	2·8% (2·6 to 3·0)	72·1% (68·6 to 75·3)	18·1% (15·5 to 20·9)	0·0% (0·0 to 0·0)	6·43% (5·97 to 6·91)	−0·29% (−0·72 to 0·16)	1·68% (1·24 to 2·14)
UK	4113 (4010 to 4216)	4364 (4254 to 4473)	8·3% (8·0 to 8·5)	80·0% (78·7 to 81·2)	15·3% (14·3 to 16·5)	0·0% (0·0 to 0·0)	4·97% (4·68 to 5·24)	4·37% (4·08 to 4·64)	2·82% (2·54 to 3·09)
Uruguay	1520 (1457 to 1586)	2049 (1965 to 2138)	8·6% (8·2 to 8·9)	71·2% (68·8 to 73·4)	17·2% (15·5 to 18·8)	0·0% (0·0 to 0·0)	2·44% (2·07 to 2·85)	2·10% (1·73 to 2·51)	−0·45% (−0·82 to −0·06)
USA	10 271 (10 054 to 10 498)	10 271 (10 054 to 10 498)	17·1% (16·8 to 17·5)	81·8% (81·2 to 82·5)	11·1% (10·6 to 11·5)	0·0% (0·0 to 0·0)	4·03% (3·84 to 4·23)	3·08% (2·89 to 3·28)	1·61% (1·42 to 1·80)
Uzbekistan	76 (63 to 93)	423 (348 to 513)	3·2% (2·6 to 3·9)	47·3% (37·6 to 56·7)	48·4% (39·1 to 58·4)	3·9% (3·2 to 4·7)	5·71% (4·39 to 7·13)	4·26% (2·96 to 5·66)	−0·53% (−1·78 to 0·80)
Vanuatu	96 (83 to 112)	84 (73 to 98)	2·7% (2·3 to 3·1)	60·3% (53·7 to 66·3)	10·4% (7·3 to 14·4)	25·0% (21·4 to 28·8)	3·06% (1·80 to 4·24)	0·80% (−0·44 to 1·94)	0·41% (−0·82 to 1·55)
Venezuela	384 (345 to 427)	636 (572 to 708)	4·1% (3·7 to 4·6)	33·2% (28·4 to 38·3)	33·8% (29·0 to 38·9)	0·0% (0·0 to 0·0)	−0·28% (−0·98 to 0·42)	−1·96% (−2·64 to −1·27)	−1·14% (−1·84 to −0·45)
Vietnam	119 (98 to 140)	347 (287 to 409)	5·5% (4·6 to 6·5)	49·6% (41·1 to 58·4)	46·7% (38·0 to 55·5)	2·7% (2·3 to 3·3)	7·97% (6·62 to 9·29)	6·71% (5·37 to 8·01)	1·27% (0·00 to 2·50)
Virgin Islands	2196 (1799 to 2665)	1180 (967 to 1432)	6·3% (5·1 to 7·6)	63·4% (53·6 to 72·5)	26·1% (18·6 to 34·9)	0·0% (0·0 to 0·0)	4·27% (2·82 to 5·71)	4·31% (2·86 to 5·75)	2·92% (1·49 to 4·34)
Yemen	59 (47 to 73)	126 (100 to 157)	9·2% (7·3 to 11·5)	14·1% (10·2 to 18·9)	79·8% (74·0 to 84·7)	5·2% (4·1 to 6·4)	1·96% (0·62 to 3·47)	−1·08% (−2·38 to 0·38)	2·42% (1·08 to 3·93)
Zambia	64 (57 to 72)	187 (167 to 209)	3·2% (2·9 to 3·6)	38·1% (31·6 to 45·0)	12·3% (8·7 to 16·4)	44·0% (39·2 to 49·1)	3·85% (2·76 to 4·82)	0·98% (−0·07 to 1·93)	−1·81% (−2·84 to −0·89)
Zimbabwe	106 (91 to 124)	198 (171 to 231)	9·7% (8·3 to 11·3)	45·0% (37·8 to 52·8)	26·5% (19·9 to 33·5)	18·9% (16·1 to 21·8)	1·89% (0·60 to 3·12)	0·36% (−0·92 to 1·56)	0·95% (−0·34 to 2·16)

Estimates in parentheses are 95% uncertainty intervals. PPP=2018 purchasing-power parity-adjusted dollars. GDP=Gross domestic product. GBD=Global Burden of Disease.

**Figure 4:**
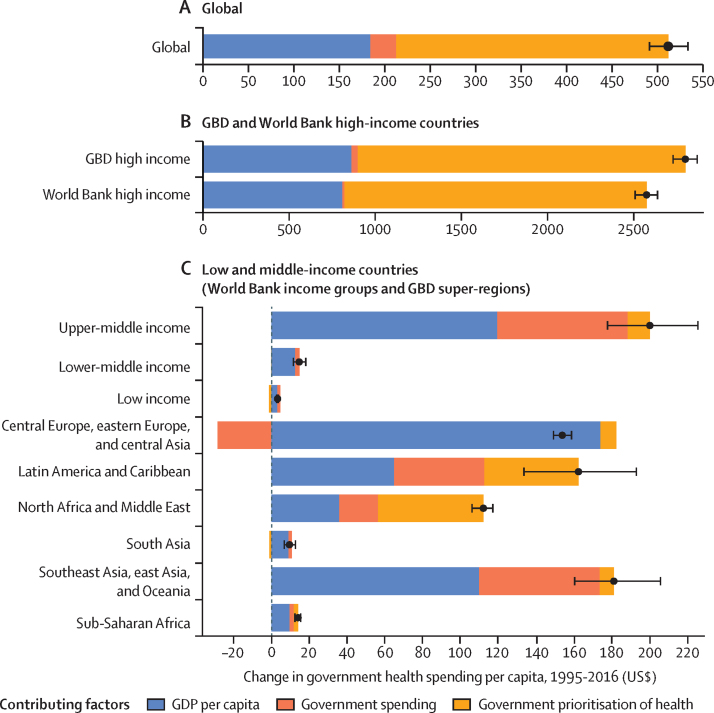
Factors of change in government health spending per capita, 1995–2016 Change in government health spending per capita by global (A), high-income (B), and low-income and middle-income countries (C), reported in inflation-adjusted 2018 US dollars. Error bars represent uncertainty intervals. Black dots represent the estimated change in government spending per capita. GBD=Global Burden of Diseases, Injuries, and Risk Factors. GDP=gross domestic product.

Governments play an important role in the changing landscape of health financing and are globally the largest source of funds for health. Figure 4 highlights the amount of change in government health spending per capita between 1995 and 2016 that is associated with each of three key factors. Globally, the primary factor driving increases in government health spending was greater prioritisation of the health sector, which was associated with an increase of $299 (95% UI 287–311) in annual government spending on health per capita between 1995 and 2016. The other key factor driving growth in government health spending per capita globally was economic development, associated with a $185 (165–207) increase per capita. Across regions and income groups, government prioritisation of health was the leading factor of change in high-income countries and in North Africa and the Middle East, whereas economic development was the key factor in upper-middle-income, lower-middle-income, and low-income countries; in central Europe, eastern Europe, and central Asia; in south Asia; in southeast Asia, east Asia, and Oceania; and in sub-Saharan Africa. Increases in total government spending also led to substantial increases in government health spending in upper-middle-income countries, particularly in southeast Asia, east Asia, and Oceania and in Latin America and the Caribbean. The smallest increase in government health spending per capita was in low-income countries, especially in south Asia and sub-Saharan Africa; in these regions, economic development was the leading factor contributing to this growth.

Globally, health spending reached $8·0 trillion (95% UI 7·8–8·1) in 2016, $10·3 trillion (10·1–10·6) in purchasing-power parity-adjusted dollars, and comprised 8·6% (8·4–8·7) of global GDP in 2016. 81·0% (80·0–81·9) was spent in high-income countries, 15·7% (14·9–16·6) in upper-middle-income countries, 3·0% (2·7–3·3) in lower-middle-income countries, and 0·4% (0·3–0·4) in low-income countries, despite low-income countries comprising 10·0% of the global population. 41·7% (40·9–42·5) of total health spending worldwide was in the USA alone, while the countries of sub-Saharan Africa collectively comprised 1·0% (0·9–1·0) of total health spending. Health spending per capita increased to $1077 (1058–1096), despite significant variation across regions and income groups (figure 1B, table 1). Per capita health spending in high-income countries was $5252 (5184–5319), ranging from $261 (208–326) in the Northern Mariana Islands to $10 802 (9469–12 352) in Bermuda; and $40 (38–43) in low-income countries, ranging from $15 (13–17) in Somalia to $106 (91–124) in Zimbabwe. Disparities persist across geographical regions, with per capita spending ranging from $37 (29–48) in Bangladesh to $84 (69–100) in Bhutan in south Asia, where health spending is the lowest of all regions (table 1).

**Figure 5:**
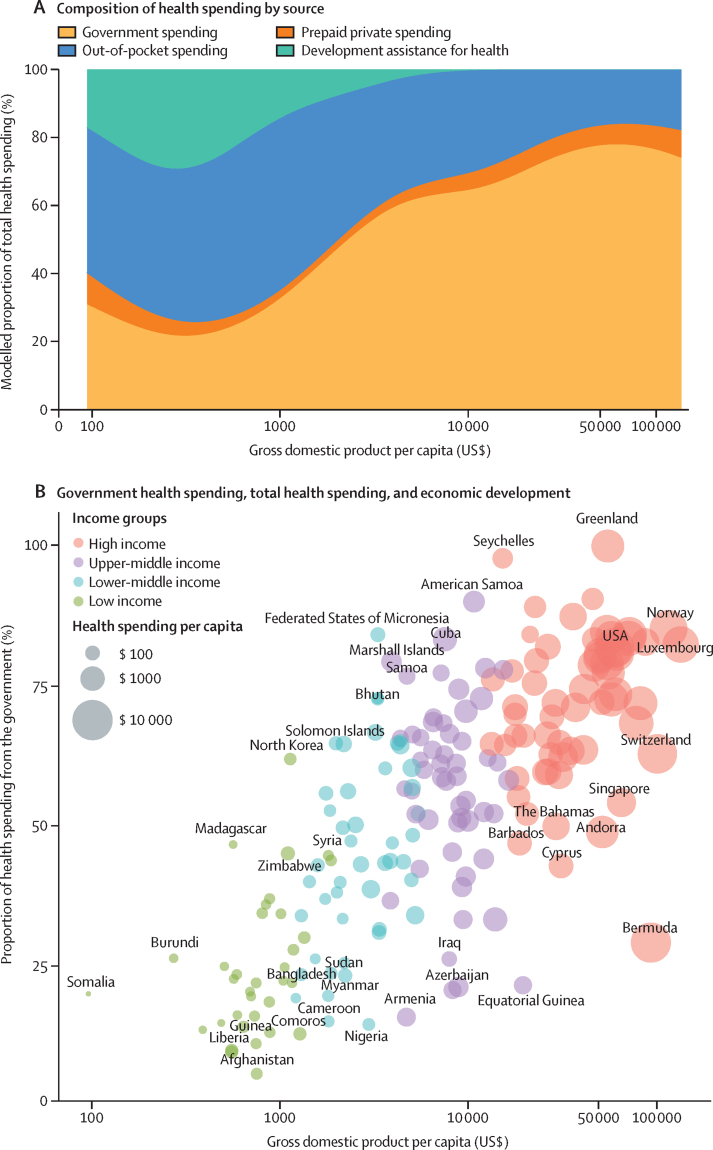
Economic development and the composition of health spending by source and proportion of health spending from the government in 2016 Composition by source (A) and proportion of health spending from the government (B). Each dot represents a country colour-coded by World Bank income group. Gross domestic product per capita reported in inflation-adjusted 2018 US dollars. The x-axes are presented in natural logarithmic scale.

Figure 2 and figure 5A collectively highlight the hypotheses made in the health financing transition.^31^ Figure 2 shows that the exponential relationship between GDP and health spending has persisted from 1995 to 2016. Figure 5A explores how the sources of health spending tend to evolve with economic development (similar figures showing this relationship in past and future years are provided in the appendix). Countries at a lower income level tend to have a higher proportion of out-of-pocket spending and DAH to finance the health sector; as countries get wealthier, less of their health spending is financed by DAH. As the proportion of health spending that is DAH subsides, countries tend to fill the gap by further increasing out-of-pocket and government health spending, with an increasing proportion from government health spending as economic development increases. This trend is seen by comparing the proportion of total spending from out-of-pocket spending in low-income and lower-middle-income countries: in 2016, lower-middle-income countries had the highest share of spending from out-of-pocket spending (56·1% [95% UI 47·3–65·4]), even higher than that of low-income countries (42·4% [38·3–47·0]), because low-income countries also had a large share of spending from DAH (25·4% [23·9–26·8]; table 1). Despite this global pattern, figure 5B and table 1 highlight the wide variation in the proportion of health spending that came from the government: 79·6% (78·2–81·1) of all spending in high-income countries in 2016 came from government health spending, as did 53·9% (49·9–58·6) in upper-middle-income countries, 32·1% (28·4–36·1) in lower-middle-income countries, and 26·3% (23·3–29·5) in low-income countries. Wide variation exists even for countries at similar levels of GDP per capita. In 2016, among low-income countries the proportion of health spending from the government ranged from 5·7% (3·9–7·9) in Afghanistan to 61·9% (51·7–72·2) in North Korea; among lower-middle-income countries it ranged from 14·5% (10·6–19·2) in Nigeria to 84·1% (79·9–87·5) in the Federated States of Micronesia; among upper-middle-income countries it ranged from 15·8% (12·5–19·7) in Armenia to 90·1% (86·5–93·0) in American Samoa; and among high-income countries it ranged from 29·1% (25·1–33·0) in Bermuda to 100·0% (100·0–100·0) in Greenland (table 1).

### Development assistance for health

**Figure 6:**
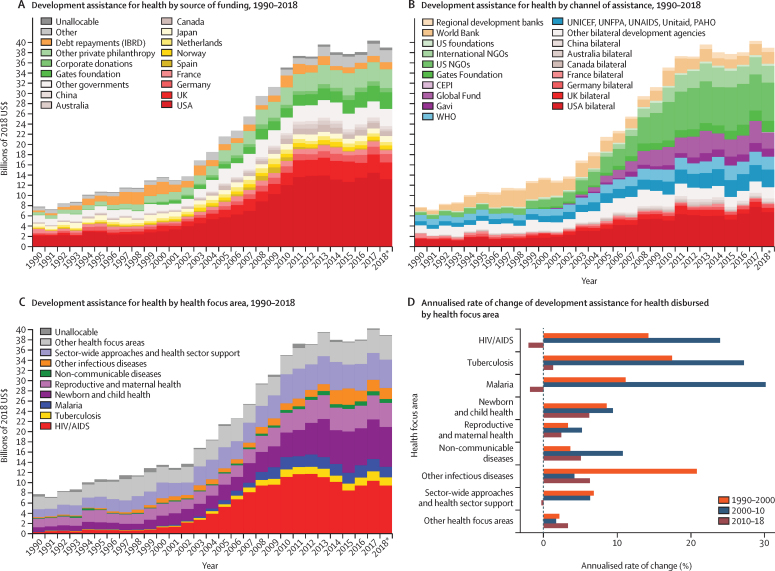
Changes in development assistance for health disbursements, 1990–2018 Development assistance for health by source of funding (A), channel of assistance (B), health focus area (C), and annualised rate of change by health focus area (D). Reported in billions of inflation-adjusted 2018 US dollars. World Bank includes the International Development Association and the International Bank for Reconstruction and Development (IBRD); and regional development banks include the Inter-American Development Bank, the African Development Bank, and the Asian Development Bank. CEPI=Coalition for Epidemic Preparedness Innovations. Gates Foundation=Bill & Melinda Gates Foundation. Gavi=Gavi, the Vaccine Alliance. NGOs=non-governmental organisations. PAHO=Pan American Health Organization. *Data for 2018 are preliminary estimates based on budget data and estimation.

Although government health spending did not grow substantially in countries that are currently classified as low-income, DAH had the fastest growth in health spending per capita in these countries (figure 3). Figure 6 (A–C) shows that in 1990, total DAH disbursed to low-income and middle-income countries was $7·7 billion. Between 1990 and 2000, DAH increased at 5·69% annually, whereas between 2000 and 2010 it increased at 10·03% annually. More recently, DAH disbursement has levelled, with annual growth from 2010 through 2018 estimated to be 1·33%.

In 2018, total DAH reached $38·9 billion, with the USA as the largest single source of contributions in terms of volume, providing $13·2 billion (33·8% of total DAH); the UK as the second largest single contributing source, providing $3·3 billion (8·4%); and the Bill & Melinda Gates Foundation as the third largest single contributing source, providing $3·2 billion (8·3%; figure 6A). Despite having a lower income per capita than all other national contributors, China provided $644·7 million of DAH in 2018. Figure 6B shows the annual total DAH by disbursing agency. The largest multilateral and public–private partnerships that disbursed DAH in 2018 included the Global Fund ($3·2 billion; 8·2% of the total disbursed), WHO ($2·6 billion, 6·6%), and UNICEF ($1·9 billion, 4·9%). The Coalition for Epidemic Preparedness Innovation disbursed $71·0 million.

Figure 6C highlights the annual total DAH targeted to different health focus areas over time. Although all health focus areas tracked in this study have more DAH targeting them now than in 1990, this growth has been especially acute for funding allocated to HIV/AIDS, malaria, and tuberculosis, all of which increased at more than 20% per year between 2000 and 2010 (figure 6D). More recently, DAH targeting newborn and child health and infectious diseases other than HIV/AIDS, malaria, and tuberculosis grew most quickly, growing at 6·19% and 6·27% annually between 2010 and 2018. During this same period, DAH for HIV/AIDS reduced, with an annualised decline of 2·05% per year between 2010 and 2018, or a reduction of $1·7 billion since the 2012 peak. Still, in 2018, HIV/AIDS received more DAH than any other health focus area ($9·5 billion [24·3% of the total]). Newborn and child health received the second most DAH ($7·8 billion [20·1%]), sector-wide approaches and health sector support received the third most DAH ($5·6 billion [14·3%]), and reproductive and maternal health received the fourth most DAH in 2018 ($4·7 billion [12·1%]). In 2018, we estimated that $48·3 million of DAH targeted antimicrobial resistance.

### Future

Sustained growth in health spending is expected to continue, with global spending projected to reach $10·6 trillion (95% UI 10·2–10·9) in 2030 and $15·0 trillion (14·0–16·0) in 2050 (table 2, figure 1C, 1D). In purchasing-power parity-adjusted dollars, these values are $14·3 trillion (13·7–15·0) in 2030 and $21·3 trillion (19·8–23·1) in 2050. These values are projected to comprise 8·9% (8·4–9·4) of global GDP in 2030 and 9·4% (7·6–11·3) of global GDP in 2050. Despite this growth, health spending is expected to remain skewed, with 69·4% (67·2–71·5) of this spending in countries that are currently considered high-income, 25·1% (23·1–27·1) in upper-middle-income countries, 4·9% (4·4–5·5) in lower-middle-income countries, and only 0·6% (0·6–0·7) in low-income countries, despite low-income countries comprising an estimated 15·7% of the global population by 2050. In per-capita terms, projected total health spending globally is $1264 (1219–1309) per capita in 2030 and $1667 (1567–1767) per capita in 2050 (table 2). Per capita spending in 2030 is projected to be $6313 (6135–6499) for high-income groups, $772 (707–847) for upper-middle-income groups, $121 (108–137) for lower-middle-income groups, and $48 (44–51) for low-income groups. In 2050, this spending is projected to increase to $8286 (7851–8725) for high-income groups, $1435 (1264–1632) for upper-middle-income groups, $200 (176–225) for lower-middle-income groups, and $66 (60–73) for low-income groups (table 2). The fastest growth in per capita health spending is predicted among lower-middle-income countries, with 2·64% (2·28–3·02) annual growth per capita projected between 2017 and 2050, and upper-middle-income countries, with 3·20% (2·84–3·58) annual growth per capita projected between 2017 and 2050 (table 2). Health spending per capita in 2050 is expected to remain the lowest in sub-Saharan Africa ($111 [102–121]) and South Asia ($180 [146–220]).

**Table 2:** Health spending by source and alternative future scenarios of government health spending, 2050

	**Health spending per capita, 2050 estimates (US$)**	**Health spending per capita, 2050 estimates ($PPP)**	**Health spending per GDP, 2050 estimates**	**Government health spending per total health spending, 2050 estimates**	**Out-of-pocket spending per total health spending, 2050 estimates**	**Development assistance for health per total health spending, 2050 estimates**	**Annualised rate of change in health spending, 2017–50 (US$)**	**Annualised rate of change in health spending per capita, 2017–50 (US$)**	**Annualised rate of change in health spending per GDP 2017–50 (US$)**	**Government health spending per capita ($US)**	**Difference between government health spending per capita reference scenario and better scenario 1, 2050 (US$)**	**Difference between government health spending per capita reference scenario and better scenario 2, 2050 (US$)**
**Global**												
Total	1667 (1567 to 1767)	2373 (2222 to 2537)	9·4% (7·6 to 11·3)	72·9% (68·4 to 77·5)	19·0% (17·4 to 20·8)	0·3% (0·2 to 0·5)	1·84% (1·68 to 2·02)	1·28% (1·12 to 1·44)	0·25% (−0·05 to 0·57)	1216 (1071 to 1373)	229 (212 to 267)	617 (605 to 660)
**World Bank income group**												
High income	8286 (7851 to 8725)	8812 (8363 to 9266)	13·1% (10·2 to 16·3)	79·8% (74·7 to 85·3)	12·7% (11·9 to 13·9)	0·0% (0·0 to 0·0)	1·38% (1·22 to 1·53)	1·32% (1·17 to 1·48)	0·54% (0·38 to 0·69)	6605 (5966 to 7270)	238 (211 to 301)	1528 (1474 to 1676)
Upper-middle income	1435 (1264 to 1632)	2858 (2530 to 3233)	6·6% (4·7 to 9·0)	62·4% (53·3 to 71·9)	29·6% (24·0 to 36·5)	0·1% (0·1 to 0·2)	3·25% (2·89 to 3·64)	3·20% (2·84 to 3·58)	0·79% (0·44 to 1·16)	894 (733 to 1081)	410 (367 to 500)	844 (811 to 910)
Lower-middle income	200 (176 to 225)	675 (594 to 768)	3·7% (2·7 to 4·8)	36·4% (31·6 to 41·4)	51·2% (41·9 to 62·5)	2·7% (1·8 to 4·6)	3·34% (2·97 to 3·73)	2·64% (2·28 to 3·02)	0·38% (0·03 to 0·74)	73 (54 to 95)	172 (168 to 180)	354 (349 to 384)
Low income	66 (60 to 73)	207 (189 to 227)	5·2% (4·3 to 6·1)	31·6% (26·4 to 37·1)	39·2% (34·2 to 45·0)	21·4% (14·7 to 35·1)	3·45% (3·21 to 3·72)	1·41% (1·18 to 1·64)	−0·02% (−0·27 to 0·21)	21 (15 to 27)	35 (34 to 37)	79 (77 to 85)
**GBD super-region**												
Central Europe, eastern Europe, and central Asia	972 (888 to 1063)	2343 (2135 to 2578)	5·6% (4·2 to 7·3)	60·1% (55·3 to 65·5)	35·8% (32·3 to 39·7)	0·3% (0·2 to 0·6)	1·44% (1·25 to 1·63)	1·76% (1·57 to 1·95)	0·73% (0·59 to 0·87)	583 (495 to 680)	506 (487 to 544)	791 (771 to 863)
High income	9224 (8738 to 9722)	9547 (9052 to 10 062)	13·7% (10·6 to 17·0)	80·0% (74·8 to 85·7)	12·6% (11·7 to 13·8)	0·0% (0·0 to 0·0)	1·38% (1·22 to 1·54)	1·31% (1·15 to 1·47)	0·52% (0·37 to 0·68)	7373 (6671 to 8094)	175 (149 to 235)	1558 (1492 to 1719)
Latin America and Caribbean	953 (889 to 1019)	1784 (1668 to 1906)	7·3% (6·3 to 8·3)	48·4% (45·0 to 52·6)	33·3% (29·8 to 37·0)	0·3% (0·2 to 0·5)	1·48% (1·29 to 1·68)	0·98% (0·79 to 1·15)	0·39% (0·20 to 0·58)	462 (386 to 554)	385 (372 to 410)	604 (592 to 651)
North Africa and Middle East	473 (438 to 513)	1415 (1312 to 1536)	4·3% (3·8 to 4·9)	60·7% (53·1 to 69·9)	30·1% (27·6 to 33·1)	0·8% (0·6 to 1·4)	2·17% (2·00 to 2·40)	1·11% (0·97 to 1·28)	0·53% (0·39 to 0·66)	287 (232 to 366)	296 (283 to 320)	567 (557 to 594)
South Asia	180 (146 to 220)	670 (542 to 823)	3·5% (2·4 to 4·8)	32·6% (24·3 to 42·2)	56·9% (38·5 to 81·7)	1·2% (0·8 to 2·1)	3·61% (2·82 to 4·40)	3·27% (2·51 to 4·07)	0·37% (−0·37 to 1·16)	58 (42 to 78)	213 (209 to 227)	357 (351 to 388)
Southeast Asia, east Asia, and Oceania	1397 (1195 to 1621)	2758 (2381 to 3185)	6·6% (4·5 to 9·5)	64·9% (53·7 to 76·5)	29·1% (22·3 to 37·8)	0·1% (0·1 to 0·2)	4·08% (3·59 to 4·59)	4·10% (3·60 to 4·60)	0·92% (0·44 to 1·43)	905 (737 to 1097)	305 (251 to 407)	799 (762 to 873)
Sub-Saharan Africa	111 (102 to 121)	283 (260 to 307)	4·4% (3·5 to 5·5)	39·0% (35·5 to 43·0)	32·5% (27·5 to 38·0)	13·4% (9·2 to 21·9)	3·07% (2·82 to 3·32)	0·97% (0·73 to 1·19)	0·15% (−0·10 to 0·39)	43 (34 to 55)	61 (59 to 65)	156 (153 to 169)

Estimates in parentheses are 95% uncertainty intervals. 2050 scenario 1 reflects the increase in government health spending if all countries met the target proportion of government spending on health. 2050 scenario 2 reflects the increase in government health spending if all countries met the target proportion of government spending on health and target proportion of GDP that is based on government spending. PPP=2018 purchasing-power parity-adjusted dollars. GDP=gross domestic product. GBD=Global Burden of Disease.

The two regions with the lowest projected growth rate in total health spending between 2017 and 2050 are the GBD high-income region, with a growth rate of 1·38% (95% UI 1·22–1·54), and central Europe, eastern Europe, and central Asia, with a growth rate of 1·44% (1·25–1·63; table 2). Despite this similarity, the growth rates in health spending per capita are actually quite distinct (1·31% [1·15–1·47] for the GBD high-income region and 1·76% [1·57–1·95] for central Europe, eastern Europe, and central Asia), because of differences in population growth. Population projections have a large impact on health spending per capita growth rates (table 2); unlike central Europe, eastern Europe, and central Asia, where population growth is lower than zero, meaning the population growth is well below replacement, population growth is expected to remain high in North Africa and the Middle East, and especially in sub-Saharan Africa. In this region, annualised health spending growth between 2017 and 2050 is expected to be 3·07% (2·82–3·32), although health spending per capita growth is expected to be 0·97% (0·73–1·19; table 2).

**Figure 7:**
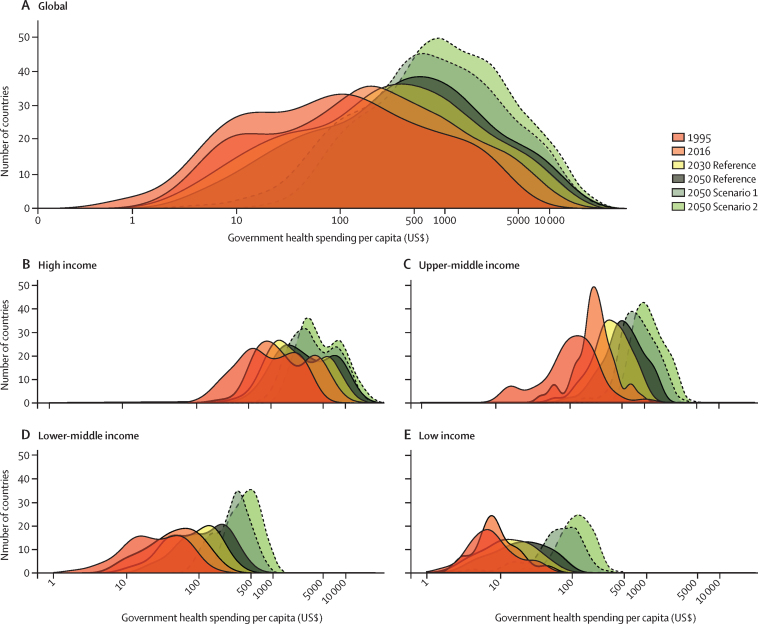
Distribution of government health spending per capita, globally and by income group, for 1995, 2016, 2030, 2050, and two future scenarios Reported in inflation-adjusted 2018 US dollars. 2050 scenario 1 reflects the increase in government health spending if all countries met the target proportion of government spending on health. 2050 scenario 2 reflects the increase in government health spending if all countries met the target proportion of government spending on health and target proportion of gross domestic product that is based on government spending. The x-axes are presented in a natural logarithmic scale. This figure was remade with health spending measured as a percentage of gross domestic product, and is included in the appendix.

Our future scenarios of government health spending (figure 7) estimate the potential additional funding governments might be able to mobilise if the health sector is further prioritised or if governments increase spending overall, or if both are achieved. In scenario 1, in 2050, increased prioritisation of health by governments could lead to an additional $229 (95% UI 212–267) in health spending per capita, compared to the reference scenario. In scenario 2, in 2050, increased prioritisation of health and increased total government spending could lead to an additional $617 (605–660) per person. In both scenarios, the potential increase in government health spending per capita is more than double the projection in the reference scenario in some countries. Furthermore, these potential gains are proportionally greater in low-income and lower-middle-income countries and south Asia and sub-Saharan Africa, relative to the low levels of government health spending in the reference scenario (table 2).

### Past to the present to the future

Examining the full set of results spanning 1995 to 2050, we observe three persistent trends. The first trend is an ongoing increase in health spending over time, as shown by the upward push in the curves in figure 2. Countries at the same level of income as other countries in the past tend to spend more on health than those other countries did, especially countries with higher levels of economic development. The second trend, seen across most regions and income groups, is of positive, albeit slowing, growth rates in health spending, as well as declining population growth rates. Because population growth was generally dropping at the same rate as health spending, or at a faster rate, health spending per capita growth appears to be flattening or increasing. Sub-Saharan Africa stands out in particular, as population growth is noticeably higher than elsewhere in the early 2000s, but is decreasing over time, leading to a slow increase in health spending per capita growth rate. The third trend is increasing disparities in total and government health spending, even among countries in the same income group. As shown in figure 2, despite the fact that the majority of countries are moving upwards over time to higher total health spending per capita, the gap between the smallest and the largest health spenders per capita has grown from $7313 (95% UI 6453–10 185) per capita in 1995, to $10 787 (9456–12 335) per capita in 2016, to a projected value of $15 806 (14 654–16 913) in 2050. Between income groups, in 1995, per capita health spending in high-income countries was 96·4 times (91·3–101·6) greater than the spending in low-income countries; this ratio increased to 130·2 (122·9–136·9) in 2016 and is projected to stay at similar levels in the future, at 133·0 (123·7–142·4) in 2030 and 125·9 (113·7–138·1) in 2050. Figure 7 shows the changes in the distribution of government health spending per capita by income group over time. Although there is clear overall shifting of distributions towards the upper end during the study period, accompanying this trend are the countries that are left behind from this positive shift and the large discrepancy in values between high-income and low-income countries, which are shown on different scales. Especially in low-income and middle-income groups, the gap between countries with the highest and lowest government health spending per capita is projected to widen between now and the future.

## Discussion

### Overview

Globally, health spending has risen steadily since 1995, reaching $8·0 trillion (95% UI 7·8–8·1) in 2016 and projected to further increase to a total of $15·0 trillion (14·0–16·0) by 2050, but at a slower rate of growth in the majority of countries. Health spending currently constitutes 8·6% (8·4–8·7) of the global economy, with the largest proportions of this spending financed by governments and spent in high-income countries. Sub-Saharan Africa and low-income countries currently have the lowest levels of spending, with 1·0% (0·9–1·0) of the global total in sub-Saharan Africa and 0·4% (0·3–0·4) of the global total in low-income countries. The composition of health spending by financing source has changed and will continue to evolve in the future. In 2016, increased proportions of global health spending came from government (74·0% [72·5–75·5]) and DAH (0·2% [0·2–0·2]), and decreased proportions from out-of-pocket spending (18·6% [18·0–19·4]). However, DAH has plateaued since 2010, leading to a renewed emphasis on domestic resource mobilisation in recent years. By 2050, we project a problematic shift in this trend, with government health spending declining to 72·9% (68·4–77·5), and slight increases in out-of-pocket spending (19·0% [17·4 to 20·8]).

Sustaining growth in government health spending is important because this spending can provide funding for essential health services.^32^ Furthermore, increased government health spending can indirectly affect health outcomes by increasing household financial resources for other health determinants, such as food and education, as a result of reduced spending on health care.^33^ Given that government spending is a source of pooled spending, it could also help spread the risk of financial burden caused by health care across the population. This pooling is particularly important given the finding that out-of-pocket spending is projected to increase in many low-income and middle-income countries. Financial protection is a core tenet of universal health coverage and these projections suggest that many countries are not on track to adequately cover their populations.

Our future government health spending scenarios suggest that, with greater prioritisation of the health sector or increased total government spending, a drastic increase in government health spending per capita could be achieved, especially in countries currently with low levels of government health spending. The two scenarios assessed how much fiscal space there is and opportunities for expansion, although without considering other demands (eg, debt) on government spending. This is consistent with findings from recent work by WHO, which concluded that low-income countries have been lagging in the growth of government health spending.^34^ The low ratio of tax revenue to GDP in many low-income countries exemplifies this challenge.^35^ Furthermore, work by the Organisation for Economic Co-operation and Development (OECD) points to the difficulty of sustaining current patterns of health financing from public sources in the future.

Patterns of past and projected health spending are useful for characterising countries’ progress along the health financing transition.^31^ This can be described as a rise in per capita health spending with a declining proportion from out-of-pocket and donor assistance. This is exemplified by the proportion of health spending that was out of pocket in 2016, which peaked among lower-middle-income countries (56·1% [47·3–65·4]). The term “missing middle” has been used to characterise the problematic situation for countries at a middle level of income—as they begin to receive less DAH but do not yet fill the gap in financing with government spending, and instead rely more on additional out-of-pocket spending.^36^ In figure 5A, which shows this relationship cross-sectionally in 2016, the “missing middle” phenomenon appears to peak for lower-middle-income countries. Key strategies to help prevent countries from falling into this circumstance include sustaining DAH as countries reach middle-income status or development of robust domestic health financing systems early in a country's economic development.

These results have important implications for policy, both at national and international levels. For countries and regions projected to have the slowest increases in government and prepaid private spending, domestic health financing reforms that increase levels of prepaid resources should be a priority as these populations risk falling further behind in the global push toward universal health coverage and in reducing child and adult mortality. Likewise, donors should consider these financing trajectories when making allocation decisions, possibly prioritising countries expected to have the slowest growth in domestic pooled spending. The projected persistence of severe global disparities in health spending requires the global community to consider and develop domestic and international policies that address the causes and effects of these inequities. High-income countries spent 130·2 times (95% UI 122·9–136·9) more on health per capita than low-income countries in 2016, and this trend is expected to continue into the future. The strong relationship between GDP and health spending suggests that supporting economic development in the poorest countries is an important approach for improving equity in health financing across countries. There are many examples of countries that have substantially increased health spending as their economies have grown. Still, there are other important cases where countries have increased health spending much faster than their economic growth. These countries, such as China, South Korea, and Cuba, highlight what is possible with political will and investments in health.

Although the beginning of the 21st century coincided with a period of substantial increase in resources dedicated towards global health goals, growth in overall DAH has plateaued more recently. For some health focus areas, such as HIV/AIDS and health-systems strengthening, which have the potential to promote sustainable health systems in recipient countries, funding has reduced. Also of note is the relatively small share of DAH currently targeted at non-communicable diseases, despite these diseases accounting for the majority of the global disease burden.^37^ Even so, contributions from emerging donors such as China have the potential to provide new financing streams. Increasingly, China has become an important stakeholder in global health, including contributing substantially to the Ebola containment efforts in 2014 and to the establishment of the Africa Center for Diseases and Control thereafter.^38^, ^39^ Globally, other innovative financing mechanisms for pooling additional resources to leverage development assistance efforts have been established. For example, the Global Financing Facility was established in 2015 as a catalyst to align financing from international partners, the private sector, and country governments around country-owned investment cases related to reproductive, maternal, and child health.

As health spending growth rates decline or sources of funding plateau, it is especially important to understand the factors that improve the efficiency of health spending. It is important to note that increases in health spending do not necessarily translate into improvements in access to care, quality of care, or health outcomes. Additional research is needed to identify policies, such as strengthening supply chains, and attributes of health systems and governments, such as reduced corruption, that lead to more efficient spending and improvements in intermediate outputs and outcomes of health systems. Understanding and implementing effective political and policy changes that support more efficient use of financial resources for health will help countries to better utilise limited resources to work toward universal health coverage and improved population health. Furthermore, whether increasing health spending should be viewed positively or negatively (and therefore promoted or curbed) should be determined according to the broader context. While additional health spending in countries with very low health spending is essential to meeting important global health goals, some high-income countries are concerned about the continuous growth in health spending and are searching for policies to curb these trends.

## Limitations

This study has some limitations. First, although we used estimation methods that account for challenges related to the reliability and completeness of publicly available historical global health spending data, we acknowledge that the input data had some weaknesses. For certain countries the extracted data were not tied to an underlying data source or they did not seem to have credible year-over-year trends. In these cases, we modelled domestic spending ourselves rather than relying on observed data. Additionally, we used the definition of spending used by the System of Health Accounts and the WHO GHED, which excludes investment spending, informal payments, and all spending that falls outside of the health system, including cross-sectoral investments. Population estimates used to compute per capita values are subject to similar data limitations, and this is especially true for countries with civil unrest and large migration patterns. Second, uncertainty intervals provided throughout this Article reflect uncertainty in both the retrospective and prospective data. The widening of uncertainty intervals as we push further into the future reflects the challenges in using trends and relationships from a short time span in the past to project into the future as well as incorporating unexpected future events and changes. Third, the out-of-sample predictive validity of our models was tested on the past 10 years of observed data. This process determined the models picked for projecting growth rates. Therefore, our future scenarios are dependent on any observed shocks in the recent past, which would be difficult to predict out of sample. Similarly, projections are based on past trends and relationships, and our models cannot anticipate events, such as natural disasters or other unexpected events, that have never occurred. Fourth, our projections of available DAH rely primarily on growth in GDP, but we acknowledge that other political and commercial factors also drive the allocation of DAH from donors to recipient countries. Fifth, we were not able to measure health spending inequities within countries (eg, those across subnational regions, income levels, ethnic groups, and so on). Although some countries are projected to have large gains in health spending during the study period, the benefits are not likely to be distributed equally across subgroups. Country-specific contexts and determinants of health spending, such as domestic policies and political movements, are not discussed here but are important when designing country-specific policies. Finally, our prediction models do not capture the dynamic nature of health spending, in that health spending leads to better health, which can also lead to economic growth.

The data going into our modelling were all prepared in US dollars. US dollars were seen to be more stable across countries and observed years than purchasing-power parity-adjusted dollars, and more comparable to existing studies. Each currency has strengths, but neither US dollars nor purchasing-power parity-adjusted dollars are a perfect measure. US dollars value spending most accurately for tradable goods, but purchasing-power parity-adjusted estimates provide a better reflection of domestic spending on non-tradeable goods and are better for cross-country comparisons. Although neither of the currencies is measured perfectly in the data, having a more stable input to our models allowed us to produce more reliable estimates.

## Conclusions

Health spending per capita, which has increased steadily since 1995, is projected to continue increasing well into the future, but at a slower rate of growth, and large existing disparities in per capita spending by country are projected to persist in the coming decades. Increasing prioritisation of health and total government spending are key factors to facilitate the health financing transition in all countries, whereby additional domestic resources are mobilised for health to gradually replace high out-of-pocket payments. Sustained increases in the quantity, equity, and efficiency of health financing are essential to achieving universal health coverage and improving health outcomes globally.
