
JOURNAL INFORMATION
==============================
NLM Title Abbreviation: Lancet
Journal ID: Lancet
NLM Journal ID: 2985213R

Lancet (London, England)

ISSN: 0140-6736
EISSN: 1474-547X

ARTICLE INFORMATION
==============================
PMCID: PMC8444082
PMID: 34509230
DOI: 10.1016/S0140-6736(21)01806-7
Article ID: S0140-6736(21)01806-7
Article version: 1
Subjects: Department of Error

Department of Error

Print publication date: 2021 Sep 11
Volume: 398
Issue: 10304
First page: 956
Last page: 956
Copyright: © 2021 The Author(s). Published by Elsevier Ltd.
Copyright year: 2021
Copyright holder: Elsevier Ltd
License: This is an open access article under the CC BY license (http://creativecommons.org/licenses/by/4.0/).
License URL: https://creativecommons.org/licenses/by/4.0/
Erratum for: Past, present, and future of global health financing: a review of development assistance, government, out-of-pocket, and other private spending on health for 195 countries, 1995–2050 393 10187 1 6 2019 2233 2260 Lancet (London, England) 10.1016/S0140-6736(19)30841-4 PMC6548764 31030984

==============================
Global Burden of Disease Health Financing Collaborator Network. Past, present, and future of global health financing: a review of development assistance, government, out-of-pocket, and other private spending on health for 195 countries, 1995–2050. Lancet 2019; 393: 2233–60—In this Article, the Global Burden of Disease Health Financing Collaborator Network has been updated to include Maciej Banach, Sarah Friedman, and Raghavendra Guru Srinivasan. The affiliations of Maciej Banach, Sarah Friedman, and Raghavendra Guru Srinivasan have also been added, and the affiliations of Martin Amogre Ayanore and Masood Sepehrimanesh have been updated. These corrections have been made to the online version as of Sept 9, 2021.

