# Supplementary material for: Past, present, and future of global health financing: a review of development assistance, government, out-of-pocket, and other private spending on health for 195 countries, 1995–2050
Source: Lancet. 2019 Jun 1;393(10187):2233–60. doi: 10.1016/S0140-6736(19)30841-4 (PMC6548764; doi:10.1016/S0140-6736(19)30841-4)
Supplement: Supplementary appendix [file mmc1.pdf]

THE LANCET

Supplementary appendix

This appendix formed part of the original submission and has been peer reviewed. We post it as supplied by the authors.

Supplement to: Global Burden of Disease Health Financing Collaborator Network. Past, present, and future of global health financing: a review of development assistance, government, out-of-pocket, and other private spending on health for 195 countries, 1995–2050. *Lancet* 2019; published online April 25. http://dx.doi.org/10.1016/S0140-6736(19)30841-4.

SUPPLEMENTARY METHODS ANNEX

Past, present, and future of global health financing: a review of development assistance, government, out-of-pocket, and other private spending on health for 195 countries, 1995-2050

Version: April 22, 2019

Global Burden of Disease Health Financing Collaborator Network

Table of Contents

SECTION 1. METHODS OVERVIEW

Overview

Estimating domestic health spending: 1995-2016

Estimating development assistance for health: 1990-2018

Factors associated with changes in government health spending: 1995-2016

Estimating health spending in the future: 2017-2050

Alternative future government health spending scenarios

Reporting and uncertainty analysis

Role of the funding source

SECTION 2. TRACKING TOTAL HEALTH SPENDING AND ITS COMPONENTS

Overview

Statistical model to fill missingness in health expenditure variables

SECTION 3. TRACKING DEVELOPMENT ASSISTANCE FOR HEALTH FROM TRADITIONAL DONORS

Overview

Tracking development assistance for health from bilateral aid agencies and the European Commission

Tracking development assistance for health from the development banks

Tracking contributions from GFATM and GAVI

Tracking expenditure by United Nations Agencies active in the health domain

Tracking development assistance for health from private foundations

Tracking non-governmental organizations

Calculating the technical assistance and program support component of development assistance for health from loan-and grant-making channels of assistance

References

SECTION 4: TRACKING DEVELOPMENT ASSISTANCE FOR HEALTH FROM CHINA

Overview

SECTION 5. FORECASTING HEALTH SPENDING FROM 2017 through 2050

Data

Covariates

Ensemble Modeling
Package and Architecture
Inclusion and Exclusion Criteria
Creating the Forecasts
Ad-hoc Draws Correlation
Future Health Scenarios
Tables and Figures
Additional Tables Figures

Abbreviations

ADB	Asian Development Bank and
AfDB	African Development Bank
BMGF	Bill & Melinda Gates Foundation
CRS	Creditor Reporting System
DAC	Development Assistance Committee
DAH	Development assistance for health
EC	European Commission
GDP	Gross domestic product
GFATM	Global Fund for AIDS, Tuberculosis and Malaria
GGHE	General government health expenditure
GHED	Global Health Expenditure Database
GHES	Government health spending (Aggregate of "Social insurance contributions", "Transfers from government domestic revenue (allocated to health purposes)", and "Compulsory prepayment (Other, and unspecified, than FS.3)" as defined in GHED)
HSS	Health System Strengthening
IBRD	International Bank for Reconstruction and Development
IDA	International Development Association
IDB	Inter-American Development Bank
LCU	Local currency units
NASA	National AIDS Spending Assessments
NGO	Non-governmental organizations
NHA	National Health Accounts
NPISH	Non-profit institutions serving households
ODA	Official development assistance
OECD	Organisation for Economic Co-operation and Development
OOP	Out-of-pocket (defined in GHED: “Other revenues from households n.e.c")
PAHO	Pan American Regional Office for WHO
PI	Private insurance
PPP	Pre-paid private (Aggregate of "Voluntary prepayment", "Other revenues from corporations n.e.c." and "Other revenues from NPISH n.e.c." as defined in GHED)
SHA 2011	System of Health Accounts 2011

SWAps	Sector Wide Approaches
TB	Tuberculosis
THE	Total health expenditure (defined in GHED: “Current health expenditure by revenues of health care financing schemes”)
UNICEF	United Nations Children’s Fund
UNAIDS	Joint United Nations Programme on HIV/AIDS
UNFPA	United Nations Population Fund
VolAg	Report of Voluntary Agencies
WHO	World Health Organization

SECTION 1. METHODS OVERVIEW

Overview

The methods presented here summarize the various components of the estimation process; interested readers are encouraged to consult the appendix for additional details on data sources, methods, and for additional results presented in alternative units. "Health spending" is defined as money spent on services, supplies, and basic infrastructure to deliver health care, using the same definition employed by the System of Health Accounts 2011 and the WHO Global Health Expenditure Database (GHED).[1,2] Health "financing" and "funding" are used interchangeably to refer to the source, as opposed to the utilization, of financial resources. "Economic development" refers to GDP per capita.

We estimated health spending from four main sources – government, out-of-pocket, prepaid private, and DAH – for 195 countries and territories. For brevity, "countries and territories" are referred to only as "countries", all of which are categorized into four World Bank income groups (high-, upper-middle-, lower-middle-, and low-income) and seven Global Burden of Disease (GBD) super-regions (Central Europe, Eastern Europe, and Central Asia; GBD high income; Latin America and Caribbean; North Arica and Middle East; South Asia; Southeast Asia, East Asia, and Oceania; and sub-Saharan Africa). Data tracking government, out-of-pocket, and prepaid private health spending, which together comprise total domestic health spending, were available from 1995 through 2016. Government health spending includes social health insurance and mandated private health insurance, as well as government public health programmes. Out-of-pocket health spending includes health care spending paid by the patient or his or her household, excluding insurance premiums paid in advance of care. Prepaid private health spending includes voluntary private insurance and non-governmental agency spending on health.

Estimates of DAH, defined as the financial and in-kind contributions from major development agencies to low- and middle-income countries for the purpose of maintaining or improving population health, were generated from 1990 through 2018, although transfers to recipient countries can only be estimated until 2017. In other words, the total amount of DAH, by source, is estimated through 2018, but is not allocated by recipient country for 2018. The sum of domestic health spending and DAH, net of the administrative costs needed to run the development agencies, form the envelope of total health spending for each country and year.

Domestic health spending from each of the three sources was projected for each country from 2017 to 2050, and DAH was projected from 2018 to 2050, by modelling rates of change across time. These models incorporate country-specific time trends that attenuate across time and converge to the global average, consider a broad set of covariates and time-series modelling techniques, and propagate four types of uncertainty - model, data, parameter, and fundamental uncertainty.

Estimating domestic health spending: 1995–2016

We extracted data on gross domestic product (GDP) per capita from five leading sources of these estimates (World Bank, International Monetary Fund [IMF], United Nations, Penn World Tables, and Maddison) and built from methods described by James and colleagues to generate a single series of GDP per capita using Gaussian processes.[3-7] This method incorporated data from all five GDP series and also propagated uncertainty through the estimates.[8] The resulting series spans 195 countries from 1970 to 2017, and larger uncertainty intervals highlight countries where the input data from the five sources was discordant or estimates were missing.

We extracted data from the WHO's Global Health Expenditure Database (GHED) on government domestic revenue transfers allocated for health, compulsory prepayment, voluntary prepayment, social insurance

contributions, and other domestic revenue from households, corporations, and non-profit institutions serving households.[9] Data from GHED excludes spending on major investments such as hospital construction, health worker education and training, and research and development. Countries report these data and the data source to WHO; when possible, these are based on National Health Accounts, but alternative sources are used in many cases. Health spending estimates were extracted in current national currency units, deflated 2018 national currency units, and exchanged to 2018 US dollars. Deflator series and exchanges rates used were based on those reported in the IMF World Economic Outlook.[5]

We estimated domestic government spending on health by aggregating transfers from government domestic revenue for health purposes, compulsory prepayment, and social insurance contributions. Then, to estimate domestic prepaid private health spending, we aggregated voluntary prepayment, other domestic revenues from non-profit institutions serving the household, and other domestic revenues from corporations. All payments by households other than social insurance contributions were designated as out-of-pocket spending. To generate domestic health spending estimates in purchasing-power parity-adjusted (PPP) dollars, we multiplied the health spending fractions by GDP per capita measured in 2018 PPP dollars.

The extracted data were evaluated for quality. Some of the extracted data points were not tied to an underlying data source and were estimated using a diverse set or poorly defined methods. Furthermore, for a given country, some data varied substantially across time. To overcome these concerns, we used a spatiotemporal Gaussian process regression model to estimate health spending across time, country, and spending category.[10] To prevent data with insufficiently described estimation methods or without data source identification from substantially influencing our model estimation, each data point was assessed and assigned a weight between one and five using the point-specific metadata provided in the GHED. We based weights on metadata completeness, documented source information, and documented methods for estimation. The distribution of data points assigned to each of the five data quality scores, by country income group, is presented in the appendix. Our guidelines for assessing the metadata, a detailed description of how the weights were created, and additional information about the spatiotemporal Gaussian process regression model can also be found in the appendix.

Estimating development assistance for health: 1990–2018

To track DAH, we relied on revenue data in financial statements, annual reports, budget documents, and project disbursement records reported by international development agencies such as the Organisation for Economic Co-operation and Development (OECD) Creditor Reporting System, the World Bank, the Global Fund, and major philanthropic entities like the Bill & Melinda Gates Foundation, Susan Thompson Buffet Foundation, and Rotary International.[11-20] We used budget and commitment data to generate estimates for recent years for which disbursement data were not yet available; for most channels data extends to 2017 or 2018 so that at most one year of estimation was needed.

Our DAH estimates tracked disbursements from the originating donor (called the source); to the development agency responsible for disbursing the funds to the recipient country (called the channel); to the recipient country. We used revenue and disbursement data to count only once the transfers between development agencies to limit double counting. In addition to reporting the source, channel, and recipient of DAH, spending was disaggregated into nine major health focus areas (as well as more detailed programme areas) by searching project titles and descriptions for keywords, as well as using other indicators in the data. Health focus areas included HIV/AIDS; malaria; tuberculosis; reproductive and maternal health; newborn and child health; non-communicable diseases; other infectious diseases; sector-wide approaches and health system strengthening; and other. The "Other" category captured projects such as general support for a conference on the Millennium Development Goals that were not allocated to any of the eight specific

health focus areas; remaining funds for which no project descriptions were available were classified as "unallocable." The list of keywords used to isolate relevant health projects for each of the health focus areas are included in the supplemental appendix.

While the majority of the methods used for tracking DAH have been described previously, we incorporated several major improvements.[21-25] These include (i) the addition of China as a source of funding; (ii) the inclusion of the Coalition for Epidemic Preparedness Innovations as a channel; and (iii) the addition of antimicrobial resistance as a programme area.[26] The estimate we generated for antimicrobial resistance is restricted to funds that were disbursed through development agencies. These improvements expand the scope of our DAH resource tracking to capture some of the emerging areas of importance in the current global health financing landscape. For all DAH tracking, we include on funds that were transferred through major development agencies included in our research, as well as private foundation and non-governmental agencies for whom we have data. This definition of DAH excludes spending on basic bench science which may have down-stream effects on the development of new drugs. Detailed descriptions of the methodology used for these improvements, including data sources and keywords used to isolate relevant projects, are included in the appendix.

Factors associated with changes in government health spending: 1995–2016

We completed a decomposition analysis to understand the relationship between changes in per capita government health spending between 1995 and 2016 and underlying factors that may have contributed to these changes.

A standard demographic decomposition technique popularised by Das Gupta was applied; this approach yields estimates of how changes in each of a set of pre-specified factors are associated with changes in the outcome (government health spending per capita).[27] The three factors examined for their role in government health spending were: (i) economic development, (ii) increased total government spending, and (iii) greater government prioritisation of the health sector. More specifically, we measured these three factors as (i) GDP per person ($\frac{GDP}{Total\ pop}$), (ii) the proportion of GDP that is government spending ($\frac{Gov}{GDP}$), and (iii) the proportion of total government spending spent on the health sector ($\frac{Gov\ Health}{Gov}$). The product of these three factors is government health spending per capita ($\frac{Gov\ Health}{Total\ pop}$).

$$\frac{Gov\ Health}{Total\ pop} \equiv \frac{GDP}{Total\ pop} \times \frac{Gov}{GDP} \times \frac{Gov\ Health}{Gov}$$

The three factors form a comprehensive set of factors, such that all other factors that influence government health spending must operate on or through one or more of the factors examined. For example, if the people of a country demand more health services or a population ages and requires more health services from the government, it must lead to an increase in total government spending or a reprioritization of the existing government spending towards health. This decomposition approach measures the relative contribution of each factor to changes in per capita government health spending during the time period examined. A detailed explanation of the decomposition analysis can be found in the supplementary appendix.

Estimating health spending in the future: 2017–2050

Future health spending scenarios were estimated using an ensemble modelling framework and key covariates. A process diagram in the supplementary appendix displays the flow of input data and models for each step of the forecasting process. Rather than using a single model, ensemble modelling estimates a large set of future scenarios using a large number of distinct sub-models, and then takes the average across

all sub-models that pass pre-determined inclusion criterion.[28] Each sub-model has a distinct specification or set of covariates. The primary covariates considered were GDP per capita, total government spending, total fertility rate, and fraction of the population older than 65 years, as well as country-specific time trends. Total fertility rates and age-specific population were extracted from the United Nations World Population Prospects, while we generated our own estimates of GDP per capita and fraction of GDP from government spending.[29] These covariates were considered because estimates of these variables already exist through 2050 or because the covariate is believed to be a fundamental driver of health spending.

In order to project expected GDP per capita for each of the 195 countries from 2018 through 2050, we estimated the GDP per working-aged adult growth rate. Working-aged adult was set to be ages 20 through 64 years. Using out-of-sample validation, we showed that GDP per capita could be more accurately estimated (smaller root-mean-squared error) by estimating GDP per working-aged adult growth rates, rather than GDP per capita growth rates. Sub-models considered in the GDP per working-aged adult growth rate ensemble model included time components such as autoregressive and moving average terms, and a convergence term, which is the one-year lag of the non-differenced dependent variable, and a random country intercept. Inclusion of the convergence term allows for countries with more GDP per working-aged adult to have slower growth rates. We also accounted for the possibility that more recent time trends are better predictors of the future by including sub-models that placed more weight on more recent data. The country-specific random intercepts estimate the country-specific time trends, conditional on all other factors included in the model. We attenuated country-specific time trends toward zero in order to progressively transition countries toward the global growth rate because the country-specific growth rates observed in the past are generated using a relatively short time span. We excluded sub-models with non-significant independent variables (p-value greater than 0.10), an estimated coefficient on the convergence term greater than zero, and predictions that fell outside the bounds of the historical growth rate of the predicted variable.

For each country, we selected the 10% of sub-models that had the lowest root-mean-squared-error in out-of-sample tests and generated 1,000 draws. The draws were simulated to capture multiple types of uncertainty. First, we propagated parameter uncertainty by randomly sampling from each sub-model's joint precision matrix. Second, we built our estimates from the best fit sub-models rather than relying on a single model. Finally, we propagated the empirical variation around the linear fit of the sub-model using a first-order random walk where the variance of the random walk was determined by the estimated residuals from the retrospective data.

After estimating GDP per capita, we used the same method to estimate future scenarios of (i) total government spending as a fraction of GDP, (ii) government health spending as a fraction of total government spending, (iii) prepaid private health spending as a fraction of GDP, and (iv) out-of-pocket health spending as a fraction of GDP. We called these our reference future scenarios. In addition, we estimated future scenarios of the share of health spending that was provided as DAH from each major donor country, which allowed us to estimate total DAH expected to be disbursed between 2019 and 2050. Next, we estimated the fraction of the total amount of DAH that we expected each low- and middle-income country to receive. Finally, if a country was projected to reach high-income status before 2050, then it was deemed ineligible to receive DAH from that year onward and DAH it was otherwise expected to receive was reallocated to all other countries eligible to receive DAH. The World Bank sets the threshold for high-income countries at GDP per capita of $12,056, measured using the Atlas method. We used linear regression to convert this estimate to GDP per capita in 2018 US dollars based on market exchange rates, and found the threshold at which a country would be considered high-income to be GDP per capita of $17,599. To estimate total health spending for each country and year, we added DAH received by countries to estimates of government, prepaid private, and out-of-pocket health spending.

Alternative future government health spending scenarios

To assess the potential for governments to generate more resources for health, we estimated two alternative future scenarios associated with higher government health spending: the first alternative scenario reflects increased prioritisation of the health sector, and the second reflects both increased overall government spending and increased government prioritisation of health. To generate the two scenarios, we assessed the observed 2016 fraction of government spending that was allocated to the health sector ($\frac{Gov\ Health}{Gov}$) and the fraction of GDP that is based on government spending ($\frac{Gov}{GDP}$) across the 195 countries. We then set the target levels of the two fractions as the 90th percentile of the observed fractions' distributions, which we believe is an aspirational yet attainable target for many countries. Building on the existing GDP per capita projections, scenario 1 adjusts all countries so that the fraction of government spending on health is at least the target fraction (ie the 90th percentile), and scenario 2 adjusts all countries so that both the fraction of government spending on health and the fraction of GDP that is based on government spending is at least the target levels (again, the 90th percentiles). These scenarios measure how much a country's government would spend if it dedicated more resources to health and if it raised and spent more government resources, in addition to dedicating more of those resources to health.

Reporting and uncertainty analysis

All inflation-adjusted health spending estimates are reported using 2018 prices. Future values are not discounted. We report health spending per capita in US dollars and purchasing-power parity adjusted dollars and as a fraction of GDP. When not otherwise indicated, estimates are reported in 2018 US dollars. We report country spending estimates using 2017 Global Burden of Disease super-regions and 2018 World Bank income groups, regardless of whether a country changed, or is projected to change, income groups during the study period.[30,31] Rates were calculated to reflect each group, rather than the average of countries within the group, such that spending per capita estimates for an income group or region more heavily reflect rates in more populous countries. The uncertainty interval around each estimate was computed using the 2.5th and 97.5th percentiles of the 1,000 draws. All analyses were done using R (version 3.5.2) and Stata (version 13).

Role of the funding source

The funder of this study had no role in study design, data collection, data analysis, data interpretation, or writing of the manuscript. All authors had full access to all the data in the study, and JLD and CJLM had final responsibility for the decision to submit for publication.

References

1WHO | Global Health Expenditure Database. WHO. http://www.who.int/health-accounts/ghed/en/ (accessed Dec 23, 2017).

2OECD, Eurostat and World Health Organization. A System of Health Accounts 2011: Revised edition. Paris: OECD Publishing, 2017 https://read.oecd-ilibrary.org/social-issues-migration-health/a-system-of-health-accounts-2011_9789264270985-en (accessed March 7, 2019).

3The Database | Penn World Table version 9.0. Groningen Growth and Development Centre: Faculty of Economics and Business. https://www.rug.nl/ggdc/productivity/pwt/ (accessed Dec 21, 2018).

4DataBank | The World Bank. The World Bank. http://databank.worldbank.org/data/home.aspx (accessed Dec 21, 2018).

5International Monetary Fund. World Economic Outlook, October 2018: Challenges to Steady Growth. International Monetary Fund, 2018 https://www.imf.org/en/Publications/WEO/Issues/2018/09/24/world-economic-outlook-october-2018 (accessed Dec 21, 2018).

6Maddison Project Database 2018. Groningen Growth and Development Centre: Faculty of Economics and Business. https://www.rug.nl/ggdc/historicaldevelopment/maddison/releases/maddison-project-database-2018 (accessed Dec 21, 2018).

7National Accounts Main Aggregates Database. United Nations Statistics Division - National Health Accounts. https://unstats.un.org/unsd/snaama/Introduction.asp (accessed Dec 21, 2018).

8James SL, Gubbins P, Murray CJ, Gakidou E. Developing a comprehensive time series of GDP per capita for 210 countries from 1950 to 2015. *Population Health Metrics* 2012; **10**: 12.

9Global Health Expenditure Database. World Health Organization. http://apps.who.int/nha/database (accessed Dec 21, 2018).

10GBD 2015 Risk Factors Collaborators. Global, regional, and national comparative risk assessment of 79 behavioural, environmental and occupational, and metabolic risks or clusters of risks, 1990–2015: a systematic analysis for the Global Burden of Disease Study 2015. *The Lancet* 2016; **388**: 1659–724.

11Find a Grant. https%3a%2f%2fwww.theglobalfund.org%2fen%2fportfolio%2ffind%2f (accessed Dec 22, 2018).

12OECD Statistics. https://stats.oecd.org/ (accessed Dec 22, 2018).

13Malaria Operational Plans (MOPs) | PMI. https://www.pmi.gov/resource-library/mops/ (accessed Dec 22, 2018).

14Key figures: donor contributions & pledges. https://www.gavi.org/investing/funding/donor-contributions-pledges/ (accessed Dec 22, 2018).

15Trustees reports and financial statements - International Finance Facility for Immunisation. https://www.iffim.org/finance/trustees-reports-and-financial-statements/ (accessed Dec 22, 2018).

16Financial reports. https://www.gavi.org/investing/funding/financial-reports/ (accessed Dec 22, 2018).

17Disbursements and commitments. https://www.gavi.org/results/disbursements/ (accessed Dec 22, 2018).

18PCB Archive. http://www.unaids.org/en/aboutunaids/unaidsprogrammecoordinatingboard/pcbmeetingarchive (accessed Dec 22, 2018).

19Executive Board. /executive-board (accessed Dec 22, 2018).

20GB | Governing Body Documentation. http://apps.who.int/gb/ (accessed Dec 22, 2018).

21Ravishankar N, Gubbins P, Cooley RJ, *et al.* Financing of global health: tracking development assistance for health from 1990 to 2007. *The Lancet* 2009; **373**: 2113–24.

22Leach-Kemon K, Chou DP, Schneider MT, *et al.* The Global Financial Crisis Has Led To A Slowdown In Growth Of Funding To Improve Health In Many Developing Countries. *Health Affairs* 2012; **31**: 228–35.

23Dieleman JL, Graves C, Johnson E, *et al.* Sources and Focus of Health Development Assistance, 1990–2014. *JAMA* 2015; **313**: 2359–68.

24Dieleman JL, Graves CM, Templin T, *et al.* Global health development assistance remained steady in 2013 but did not align with recipients' disease burden. *Health Aff (Millwood)* 2014; **33**: 878–86.

25Dieleman JL, Templin T, Sadat N, *et al.* National spending on health by source for 184 countries between 2013 and 2040. *The Lancet* 2016; **387**: 2521–35.

26Micah AE, Zhao Y, Chen CS, *et al.* Tracking development assistance for health from China, 2000–2018. *BMJ Global Health* under review.

27Das Gupta P. Standardization and Decompostion of Rates: A User's Manual. U.S. Bureau of the Census, 1993 https://www.census.gov/content/dam/Census/library/publications/1993/demo/p23-186.pdf (accessed Dec 21, 2018).

28Foreman KJ, Lozano R, Lopez AD, Murray CJ. Modeling causes of death: an integrated approach using CODEm. *Population Health Metrics* 2012; **10**: 1.

29World Population Prospects: The 2017 Revision | Multimedia Library - United Nations Department of Economic and Social Affairs. United Nations Department of Economic and Social Affairs. https://www.un.org/development/desa/publications/world-population-prospects-the-2017-revision.html (accessed Dec 21, 2018).

30World Bank Country and Lending Groups – World Bank Data Help Desk. The World Bank. https://datahelpdesk.worldbank.org/knowledgebase/articles/906519-world-bank-country-and-lending-groups (accessed Dec 21, 2018).

31Abate KH, Abay SM, Abbafati C, *et al.* Global, regional, and national age-sex-specific mortality and life expectancy, 1950–2017: a systematic analysis for the Global Burden of Disease Study 2017. *The Lancet* 2018; **392**: 1684–735.

SECTION 2. TRACKING TOTAL HEALTH SPENDING AND ITS COMPONENTS

List of Tables and Figures

eTable 1. Rules for assigning weight values to metadata

eTable 2. Countries and income groups, assigned weights

eFigure 1. Comparison of WHO GHED extracted and IHME ST-GPR THE in GDPpc space, 2000-2016

eFigure 2. Comparison of WHO GHED extracted and IHME ST-GPR THE in per GDP space, 2000-2016

eFigure 3. Health spending per GDP in 1995, 2016, 2030, and 2050

eFigure 4. Health spending per GDP by gross domestic product per capita, 1995, 2016, 2030, and 2050

eFigure 5. Annualised rate of change in health spending per GDP by source, by income group [A] and Global Burden of Disease super-region [B], 1995–2016

eFigure 6. Distribution of government health spending per GDP, global and by income group, 1995, 2016, 2030, 2050, and two future scenarios

eFigure 7. Flow of input data and models for each step of the forecasting process

eFigure 8. Economic development and the composition of health spending by source and proportion of health spending from the government, 1995, 2016, 2030, 2050, composition by source

Overview of GHED data cleaning process

We used Global Health Expenditure Database (GHED) data from the World Health Organization (WHO) to generate our estimates.[1] From the GHED, we extracted "Current health expenditure by revenues of health care financing schemes" for total health expenditure (THE), "Other revenues from households n.e.c" for out-of-pocket (OOP), "Gross Domestic Product" for GDP. We summed "Social insurance contributions", "Transfers from government domestic revenue (allocated to health purposes)", and "Compulsory prepayment (Other, and unspecified, than FS.3)" for government health spending (GHE). We summed "Voluntary prepayment", "Other revenues from corporations n.e.c." and "Other revenues from NPISH n.e.c." for pre-paid private (PPP).

To ensure we used the best possible data from the GHED, we evaluated the metadata also provided by GHED to establish the reliability of the data. To do so, we downloaded the metadata from the GHED website for each data point for the five indicators. We used the metadata to decide how each given data point should be weighted, from 0 to 5, with 0 meaning drop, and 1 through 5 meaning keep and treated these weights as inverse variance weights.

To assign the weights, we established guidelines for the metadata that informed how the underlying data points should be weighted. We gave priority to factors such as complete, documented source information and penalized factors such as having been derived or estimated. eTable 1 below describes the guidelines we created; any metadata that did not meet any of the disqualifying factors were given a value of 5 to reflect highest reliability.

We used the four primary metadata variables from the GHED database: data type, method of estimation, comments, and sources. We applied the guidelines to each unique set of metadata across these four variables. For a subset of data points, the metadata indicated that the data point was the sum of other data points. In these cases, if the indicator was a sub-indicator of GHES, PPP, or OOP, we assigned the data

point a value of 2 to reflect that even though we could not determine if the sub-components existed, as they are not reported in GHED, we did not feel that being a sum warranted dropping the data. We assigned the summed GHES, PPP, and OOP indicators the lowest value of its sub-indicators. If the summed data point was THE, however, we assigned the data point the lowest value of its sub-components, the summed GHES, PPP, and OOP indicators.

After designating each of the 3,005 unique sets of metadata a value weight, we applied these weights to the underlying data points. In total, we had 16,716 data points, as multiple data points shared the same unique set of metadata. Once the weights were applied to the data, we reassigned all high-income country data points that were a 0 based on the metadata to 3. We made this change to reflect that high-income countries have higher quality data and thus should not be dropped, but should also not be given the highest weight value. The high-income classification comes from the World Bank.[2] For all countries across all years of data their relative weighting scores can be seen in eTable 2.

Each component of health expenditure was aggregated for all component-year-country combinations by taking component values (downloaded in current national unit); dividing by GHED reported GDP for the corresponding year-country (also in current national unit) to convert each component into its fraction of GDP; and finally multiplying those fractions by IHME GDP series measured in USD 2018. (Note: though we harvested and cleaned metadata for THE and GDP, we do not model THE (we model GHE, PPP, OOP and then aggregate) or use the WHO's GDP data since we have our own methods with multiple sources for determination of our value).

Each component of health expenditure was then converted into three spending metrics for the time period between 1995-2016, measured in purchasing power parity (PPP): health spending per capita ($), annualized rate of change in health spending per capita 1995-2016 (%), and annualized rate of change in health spending 1995-2016 (%). These metrics are shown below in Table 1. Then, each component of health expenditure was aggregated and converted into six spending metrics for the time period between 2017-2050, measured in PPP: health spending per capita ($), annualized rate of change in health spending per capita 2017-2050 (%), annualized rate of change in health spending 2017-2050 (%), Government health spending per capita ($), difference between GHEpc (government health expenditure per capita) reference scenario and better scenario 1, 2050 ($), and difference between GHEpc reference scenario and better scenario 2, 2050 ($). Table 2 contains these values.

Currency exchange and deflation

To convert a metric (for example, DAH) from 2018 USD to 2018 PPP, the following steps were taken. First, we used the US deflator series to convert the series (DAH as example) from 2018 USD to nominal USD series for all country-years. Next, we converted the nominal USD series to nominal LCU (local currency unit) series by multiplying with country-year specific USD to LCU exchange rates. After which, we used country-year specific deflator series (based to year 2018) to convert from nominal LCU to 2018 LCU series. Finally, we converted from 2018 LCU to 2018 PPP (purchasing power parity) series using the country specific 2018 LCU to PPP conversion series. The source of country-year specific USD to LCU exchange rates is the OECD exchange rate database.[4]

eTable 1. Rules for assigning weight values to metadata

Data type	Methods of estimation	Sources and/or Comments	Weight
Blank			0
Estimated			0
	Derived by applying the sum of the components		Lowest weight of the components
	Interpolated but with additional information		2
	Method description is unclear or provides very little information		0
	Time trend interpolation		1
	Uses data from other countries		0
		Abstract that's not from something documented	1 or method (whichever is bigger)
		Adjusted	0 or method (whichever is bigger)
		Adjusted using something	2 or method (whichever is bigger)
		Any suggestion that the WHO is unclear or unsure about some aspect of the data point's metadata	0 or method (whichever is bigger)
		Approximation	0 or method (whichever is bigger)
		Assumption	0 or method (whichever is bigger)
		Both blank	0 or method (whichever is bigger)
		Both with no intelligible information	0 or method (whichever is bigger)
		Budget address	1 or method (whichever is smaller)
		Calculation was used to generate the estimate	1 or method (whichever is bigger)
		Consultation/contact (without an additional documented source)	1 or method (whichever is smaller)
		Consultations with additional source, but no specifics and just consult is documented	1 or method (whichever is bigger)
		Currency conversion	1 or method (whichever is bigger)
		Data delivered/provided/reported by (a non-documented source)	1 or method (whichever is smaller)
		Data provided but not clear by whom, with an additional source if additional source is not documented	1 or method (whichever is smaller)
		Derived	0 or method (whichever is bigger)
		Estimated based on	1 or method (whichever is bigger)

Data type	Methods of estimation	Sources and/or Comments	Weight
		Estimation	0 or method (whichever is bigger)
		Excludes (if it excludes what we do want)	0 (supersedes method)
		Extrapolated	0 or method (whichever is bigger)
		Forecasted	0 or method (whichever is bigger)
		Government department, no explicit documented source	1 or method (whichever is bigger)
		Government ministry, but no explicit documented source	1 or method (whichever is bigger)
		Includes (if it includes what we don't want)	0 (supersedes method)
		Inferred	0 or method (whichever is bigger)
		Interpolation	0 or method (whichever is bigger)
		Missing (if missing something that should be included)	0 (supersedes method)
		Modified	0 or method (whichever is bigger)
		Modified from something/modified using something	2 or method (whichever is bigger)
		Needs assessment	1 or method (whichever is smaller)
		Needs discussion	1 or method (whichever is smaller)
		Needs validation	1 or method (whichever is smaller)
		Needs verification	1 or method (whichever is smaller)
		Only provides hint of a source	1 or method (whichever is smaller)
		Projected	0 or method (whichever is bigger)
		Provides only a vague term that does not provide adequate information to infer or determine what the source is	0 or method (whichever is bigger)
		Reply	1 or method (whichever is smaller)
		Response	1 or method (whichever is smaller)
		Speech	1 or method (whichever is smaller)
		Sum of	2 (except for THE, which is lowest weight of components)
		Total of	3 (except for THE, which is lowest weight of components)
		Underestimated	0 (supersedes method)
		Unpublished	1 or method (whichever is smaller)
		Validated figures, but without specifics	2 or method (whichever is bigger)
		Weights	0 or method (whichever is bigger)

eTable 2. Countries and income groups, assigned weights

Country	Weight	Count
**Afghanistan**	0	36
**Albania**	0	50
**Algeria**	0	51
**Andorra**	0	0
**Angola**	0	51
**Antigua and Barbuda**	0	21
**Argentina**	0	48
**Armenia**	0	51
**Australia**	0	0
**Austria**	0	0
**Azerbaijan**	0	51
**Bahamas**	0	0
**Bahrain**	0	3
**Bangladesh**	0	35
**Barbados**	0	12
**Belarus**	0	17
**Belgium**	0	0
**Belize**	0	51
**Benin**	0	51
**Bhutan**	0	51
**Bolivia Plurinational States of**	0	51
**Bosnia and Herzegovina**	0	34
**Botswana**	0	51
**Brazil**	0	51
**Brunei Darussalam**	0	0
**Bulgaria**	0	51
**Burkina Faso**	0	51
**Burundi**	0	51
**Cabo Verde Republic of**	0	48
**Cambodia**	0	51
**Cameroon**	0	51
**Canada**	0	0
**Central African Republic**	0	51
**Chad**	0	50
**Chile**	0	27
**China**	0	51
**Colombia**	0	51
**Comoros**	0	51
**Congo**	0	34
**Cook Islands**	0	51
**Costa Rica**	0	51
**Cote d'Ivoire**	0	51
**Croatia**	0	27
**Cuba**	0	51
**Cyprus**	0	0
**Czech Republic**	0	18
**Democratic Republic of the Congo**	0	51
**Denmark**	0	0
**Djibouti**	0	46
**Dominica**	0	51
**Dominican Republic**	0	51

**Ecuador**	0	51
**Egypt**	0	34
**El Salvador**	0	45
**Equatorial Guinea**	0	27
**Eritrea**	0	34
**Estonia**	0	18
**Ethiopia**	0	50
**Fiji**	0	35
**Finland**	0	0
**France**	0	0
**Gabon**	0	45
**Gambia**	0	34
**Georgia**	0	51
**Germany**	0	0
**Ghana**	0	51
**Greece**	0	0
**Grenada**	0	51
**Guatemala**	0	51
**Guinea**	0	51
**Guinea-Bissau**	0	51
**Guyana**	0	51
**Haiti**	0	51
**Honduras**	0	51
**Hungary**	0	27
**Iceland**	0	0
**India**	0	51
**Indonesia**	0	51
**Iran**	0	50
**Iraq**	0	50
**Ireland**	0	0
**Israel**	0	0
**Italy**	0	0
**Jamaica**	0	51
**Japan**	0	0
**Jordan**	0	50
**Kazakhstan**	0	17
**Kenya**	0	49
**Kiribati**	0	51
**Kuwait**	0	0
**Kyrgyzstan**	0	51
**Lao People's Democratic Republic**	0	51
**Latvia**	0	33
**Lebanon**	0	50
**Lesotho**	0	17
**Liberia**	0	50
**Libya**	0	51
**Lithuania**	0	36
**Luxembourg**	0	0
**Madagascar**	0	51
**Malawi**	0	51
**Malaysia**	0	51
**Maldives**	0	51
**Mali**	0	47

**Malta**	0	1
**Marshall Islands**	0	51
**Mauritania**	0	51
**Mauritius**	0	50
**Mexico**	0	40
**Micronesia (Federated States of)**	0	51
**Monaco**	0	0
**Mongolia**	0	51
**Montenegro**	0	34
**Morocco**	0	50
**Mozambique**	0	51
**Myanmar**	0	51
**Namibia**	0	49
**Nauru**	0	48
**Nepal**	0	35
**Netherlands**	0	0
**New Zealand**	0	0
**Nicaragua**	0	51
**Niger**	0	102
**Nigeria**	0	51
**Niue**	0	51
**Norway**	0	0
**Oman**	0	21
**Pakistan**	0	35
**Palau**	0	48
**Panama**	0	51
**Papua New Guinea**	0	51
**Paraguay**	0	51
**Peru**	0	51
**Philippines**	0	51
**Poland**	0	27
**Portugal**	0	0
**Qatar**	0	0
**Republic of Korea**	0	3
**Republic of Moldova**	0	51
**Romania**	0	51
**Russian Federation**	0	28
**Rwanda**	0	51
**Saint Kitts and Nevis**	0	33
**Saint Lucia**	0	51
**Saint Vincent and the Grenadines**	0	51
**Samoa**	0	51
**San Marino**	0	0
**Sao Tome and Principe**	0	34
**Saudi Arabia**	0	12
**Senegal**	0	51
**Serbia**	0	49
**Seychelles**	0	41
**Sierra Leone**	0	37
**Singapore**	0	0
**Slovakia**	0	21
**Slovenia**	0	0
**Solomon Islands**	0	51

**Somalia**	0	17
**South Africa**	0	35
**Spain**	0	0
**Sri Lanka**	0	51
**Sudan**	0	48
**Suriname**	0	51
**Swaziland**	0	51
**Sweden**	0	0
**Switzerland**	0	0
**Syria**	0	51
**Tajikistan**	0	51
**Thailand**	0	37
**The former Yugoslav Republic of Macedonia**	0	34
**Timor-Leste**	0	51
**Togo**	0	51
**Tonga**	0	51
**Trinidad and Tobago**	0	18
**Tunisia**	0	50
**Turkey**	0	51
**Turkmenistan**	0	51
**Tuvalu**	0	51
**Uganda**	0	40
**Ukraine**	0	17
**United Arab Emirates**	0	0
**United Kingdom**	0	0
**United Republic of Tanzania**	0	34
**United States of America**	0	0
**Uruguay**	0	36
**Uzbekistan**	0	51
**Vanuatu**	0	51
**Venezuela (Bolivarian Republic of)**	0	48
**Viet Nam**	0	38
**Yemen**	0	51
**Zambia**	0	37
**Zimbabwe**	0	51
**Afghanistan**	1	14
**Albania**	1	0
**Algeria**	1	0
**Andorra**	1	0
**Angola**	1	0
**Antigua and Barbuda**	1	0
**Argentina**	1	0
**Armenia**	1	0
**Australia**	1	0
**Austria**	1	0
**Azerbaijan**	1	0
**Bahamas**	1	0
**Bahrain**	1	0
**Bangladesh**	1	0
**Barbados**	1	0
**Belarus**	1	0
**Belgium**	1	0
**Belize**	1	0

**Benin**	1	0
**Bhutan**	1	0
**Bolivia Plurinational States of**	1	0
**Bosnia and Herzegovina**	1	0
**Botswana**	1	0
**Brazil**	1	0
**Brunei Darussalam**	1	0
**Bulgaria**	1	0
**Burkina Faso**	1	0
**Burundi**	1	0
**Cabo Verde Republic of**	1	0
**Cambodia**	1	0
**Cameroon**	1	0
**Canada**	1	0
**Central African Republic**	1	0
**Chad**	1	0
**Chile**	1	9
**China**	1	0
**Colombia**	1	0
**Comoros**	1	0
**Congo**	1	13
**Cook Islands**	1	0
**Costa Rica**	1	0
**Cote d'Ivoire**	1	0
**Croatia**	1	0
**Cuba**	1	0
**Cyprus**	1	0
**Czech Republic**	1	0
**Democratic Republic of the Congo**	1	0
**Denmark**	1	0
**Djibouti**	1	0
**Dominica**	1	0
**Dominican Republic**	1	0
**Ecuador**	1	0
**Egypt**	1	16
**El Salvador**	1	6
**Equatorial Guinea**	1	0
**Eritrea**	1	0
**Estonia**	1	0
**Ethiopia**	1	0
**Fiji**	1	1
**Finland**	1	0
**France**	1	0
**Gabon**	1	0
**Gambia**	1	17
**Georgia**	1	0
**Germany**	1	0
**Ghana**	1	0
**Greece**	1	0
**Grenada**	1	0
**Guatemala**	1	0
**Guinea**	1	0
**Guinea-Bissau**	1	0

**Guyana**	1	0
**Haiti**	1	0
**Honduras**	1	0
**Hungary**	1	0
**Iceland**	1	0
**India**	1	0
**Indonesia**	1	0
**Iran**	1	0
**Iraq**	1	0
**Ireland**	1	0
**Israel**	1	0
**Italy**	1	0
**Jamaica**	1	0
**Japan**	1	0
**Jordan**	1	0
**Kazakhstan**	1	0
**Kenya**	1	0
**Kiribati**	1	0
**Kuwait**	1	0
**Kyrgyzstan**	1	0
**Lao People's Democratic Republic**	1	0
**Latvia**	1	0
**Lebanon**	1	0
**Lesotho**	1	0
**Liberia**	1	0
**Libya**	1	0
**Lithuania**	1	0
**Luxembourg**	1	0
**Madagascar**	1	0
**Malawi**	1	0
**Malaysia**	1	0
**Maldives**	1	0
**Mali**	1	0
**Malta**	1	0
**Marshall Islands**	1	0
**Mauritania**	1	0
**Mauritius**	1	0
**Mexico**	1	0
**Micronesia (Federated States of)**	1	0
**Monaco**	1	0
**Mongolia**	1	0
**Montenegro**	1	0
**Morocco**	1	0
**Mozambique**	1	0
**Myanmar**	1	0
**Namibia**	1	0
**Nauru**	1	0
**Nepal**	1	16
**Netherlands**	1	0
**New Zealand**	1	0
**Nicaragua**	1	0
**Niger**	1	0
**Nigeria**	1	0

**Niue**	1	0
**Norway**	1	0
**Oman**	1	0
**Pakistan**	1	0
**Palau**	1	0
**Panama**	1	0
**Papua New Guinea**	1	0
**Paraguay**	1	0
**Peru**	1	0
**Philippines**	1	0
**Poland**	1	0
**Portugal**	1	0
**Qatar**	1	0
**Republic of Korea**	1	0
**Republic of Moldova**	1	0
**Romania**	1	0
**Russian Federation**	1	0
**Rwanda**	1	0
**Saint Kitts and Nevis**	1	0
**Saint Lucia**	1	0
**Saint Vincent and the Grenadines**	1	0
**Samoa**	1	0
**San Marino**	1	0
**Sao Tome and Principe**	1	0
**Saudi Arabia**	1	0
**Senegal**	1	0
**Serbia**	1	0
**Seychelles**	1	0
**Sierra Leone**	1	14
**Singapore**	1	0
**Slovakia**	1	0
**Slovenia**	1	0
**Solomon Islands**	1	0
**Somalia**	1	0
**South Africa**	1	0
**Spain**	1	0
**Sri Lanka**	1	0
**Sudan**	1	0
**Suriname**	1	0
**Swaziland**	1	0
**Sweden**	1	0
**Switzerland**	1	0
**Syria**	1	0
**Tajikistan**	1	0
**Thailand**	1	13
**The former Yugoslav Republic of Macedonia**	1	0
**Timor-Leste**	1	0
**Togo**	1	0
**Tonga**	1	0
**Trinidad and Tobago**	1	0
**Tunisia**	1	0
**Turkey**	1	0
**Turkmenistan**	1	0

**Tuvalu**	1	0
**Uganda**	1	11
**Ukraine**	1	0
**United Arab Emirates**	1	0
**United Kingdom**	1	0
**United Republic of Tanzania**	1	17
**United States of America**	1	0
**Uruguay**	1	0
**Uzbekistan**	1	0
**Vanuatu**	1	0
**Venezuela (Bolivarian Republic of)**	1	0
**Viet Nam**	1	0
**Yemen**	1	0
**Zambia**	1	14
**Zimbabwe**	1	0
**Afghanistan**	2	1
**Albania**	2	1
**Algeria**	2	0
**Andorra**	2	0
**Angola**	2	0
**Antigua and Barbuda**	2	0
**Argentina**	2	0
**Armenia**	2	0
**Australia**	2	0
**Austria**	2	0
**Azerbaijan**	2	0
**Bahamas**	2	0
**Bahrain**	2	0
**Bangladesh**	2	0
**Barbados**	2	0
**Belarus**	2	0
**Belgium**	2	0
**Belize**	2	0
**Benin**	2	0
**Bhutan**	2	0
**Bolivia Plurinational States of**	2	0
**Bosnia and Herzegovina**	2	0
**Botswana**	2	0
**Brazil**	2	0
**Brunei Darussalam**	2	0
**Bulgaria**	2	0
**Burkina Faso**	2	0
**Burundi**	2	0
**Cabo Verde Republic of**	2	0
**Cambodia**	2	0
**Cameroon**	2	0
**Canada**	2	0
**Central African Republic**	2	0
**Chad**	2	0
**Chile**	2	0
**China**	2	0
**Colombia**	2	0
**Comoros**	2	0

**Congo**	2	0
**Cook Islands**	2	0
**Costa Rica**	2	0
**Cote d'Ivoire**	2	0
**Croatia**	2	0
**Cuba**	2	0
**Cyprus**	2	0
**Czech Republic**	2	0
**Democratic Republic of the Congo**	2	0
**Denmark**	2	0
**Djibouti**	2	1
**Dominica**	2	0
**Dominican Republic**	2	0
**Ecuador**	2	0
**Egypt**	2	1
**El Salvador**	2	0
**Equatorial Guinea**	2	0
**Eritrea**	2	0
**Estonia**	2	0
**Ethiopia**	2	0
**Fiji**	2	0
**Finland**	2	0
**France**	2	0
**Gabon**	2	0
**Gambia**	2	0
**Georgia**	2	0
**Germany**	2	0
**Ghana**	2	0
**Greece**	2	0
**Grenada**	2	0
**Guatemala**	2	0
**Guinea**	2	0
**Guinea-Bissau**	2	0
**Guyana**	2	0
**Haiti**	2	0
**Honduras**	2	0
**Hungary**	2	0
**Iceland**	2	0
**India**	2	0
**Indonesia**	2	0
**Iran**	2	1
**Iraq**	2	1
**Ireland**	2	0
**Israel**	2	0
**Italy**	2	0
**Jamaica**	2	0
**Japan**	2	0
**Jordan**	2	1
**Kazakhstan**	2	0
**Kenya**	2	0
**Kiribati**	2	0
**Kuwait**	2	0
**Kyrgyzstan**	2	0

**Lao People's Democratic Republic**	2	0
**Latvia**	2	0
**Lebanon**	2	1
**Lesotho**	2	0
**Liberia**	2	0
**Libya**	2	0
**Lithuania**	2	0
**Luxembourg**	2	0
**Madagascar**	2	0
**Malawi**	2	0
**Malaysia**	2	0
**Maldives**	2	0
**Mali**	2	0
**Malta**	2	0
**Marshall Islands**	2	0
**Mauritania**	2	0
**Mauritius**	2	0
**Mexico**	2	0
**Micronesia (Federated States of)**	2	0
**Monaco**	2	0
**Mongolia**	2	0
**Montenegro**	2	0
**Morocco**	2	1
**Mozambique**	2	0
**Myanmar**	2	0
**Namibia**	2	0
**Nauru**	2	0
**Nepal**	2	0
**Netherlands**	2	0
**New Zealand**	2	0
**Nicaragua**	2	0
**Niger**	2	0
**Nigeria**	2	0
**Niue**	2	0
**Norway**	2	0
**Oman**	2	0
**Pakistan**	2	16
**Palau**	2	0
**Panama**	2	0
**Papua New Guinea**	2	0
**Paraguay**	2	0
**Peru**	2	0
**Philippines**	2	0
**Poland**	2	0
**Portugal**	2	0
**Qatar**	2	0
**Republic of Korea**	2	0
**Republic of Moldova**	2	0
**Romania**	2	0
**Russian Federation**	2	0
**Rwanda**	2	0
**Saint Kitts and Nevis**	2	0
**Saint Lucia**	2	0

**Saint Vincent and the Grenadines**	2	0
**Samoa**	2	0
**San Marino**	2	0
**Sao Tome and Principe**	2	0
**Saudi Arabia**	2	0
**Senegal**	2	0
**Serbia**	2	1
**Seychelles**	2	0
**Sierra Leone**	2	0
**Singapore**	2	0
**Slovakia**	2	0
**Slovenia**	2	0
**Solomon Islands**	2	0
**Somalia**	2	0
**South Africa**	2	0
**Spain**	2	0
**Sri Lanka**	2	0
**Sudan**	2	1
**Suriname**	2	0
**Swaziland**	2	0
**Sweden**	2	0
**Switzerland**	2	0
**Syria**	2	0
**Tajikistan**	2	0
**Thailand**	2	0
**The former Yugoslav Republic of Macedonia**	2	0
**Timor-Leste**	2	0
**Togo**	2	0
**Tonga**	2	0
**Trinidad and Tobago**	2	0
**Tunisia**	2	1
**Turkey**	2	0
**Turkmenistan**	2	0
**Tuvalu**	2	0
**Uganda**	2	0
**Ukraine**	2	0
**United Arab Emirates**	2	0
**United Kingdom**	2	0
**United Republic of Tanzania**	2	0
**United States of America**	2	0
**Uruguay**	2	0
**Uzbekistan**	2	0
**Vanuatu**	2	0
**Venezuela (Bolivarian Republic of)**	2	0
**Viet Nam**	2	0
**Yemen**	2	0
**Zambia**	2	0
**Zimbabwe**	2	0
**Afghanistan**	3	0
**Albania**	3	0
**Algeria**	3	0
**Andorra**	3	34
**Angola**	3	0

**Antigua and Barbuda**	3	30
**Argentina**	3	3
**Armenia**	3	0
**Australia**	3	51
**Austria**	3	51
**Azerbaijan**	3	0
**Bahamas**	3	51
**Bahrain**	3	48
**Bangladesh**	3	0
**Barbados**	3	39
**Belarus**	3	0
**Belgium**	3	51
**Belize**	3	0
**Benin**	3	0
**Bhutan**	3	0
**Bolivia Plurinational States of**	3	0
**Bosnia and Herzegovina**	3	0
**Botswana**	3	0
**Brazil**	3	0
**Brunei Darussalam**	3	51
**Bulgaria**	3	0
**Burkina Faso**	3	0
**Burundi**	3	0
**Cabo Verde Republic of**	3	0
**Cambodia**	3	0
**Cameroon**	3	0
**Canada**	3	51
**Central African Republic**	3	0
**Chad**	3	0
**Chile**	3	15
**China**	3	0
**Colombia**	3	0
**Comoros**	3	0
**Congo**	3	0
**Cook Islands**	3	0
**Costa Rica**	3	0
**Cote d'Ivoire**	3	0
**Croatia**	3	24
**Cuba**	3	0
**Cyprus**	3	51
**Czech Republic**	3	33
**Democratic Republic of the Congo**	3	0
**Denmark**	3	17
**Djibouti**	3	0
**Dominica**	3	0
**Dominican Republic**	3	0
**Ecuador**	3	0
**Egypt**	3	0
**El Salvador**	3	0
**Equatorial Guinea**	3	24
**Eritrea**	3	0
**Estonia**	3	33
**Ethiopia**	3	0

**Fiji**	3	0
**Finland**	3	51
**France**	3	51
**Gabon**	3	0
**Gambia**	3	0
**Georgia**	3	0
**Germany**	3	51
**Ghana**	3	0
**Greece**	3	51
**Grenada**	3	0
**Guatemala**	3	0
**Guinea**	3	0
**Guinea-Bissau**	3	0
**Guyana**	3	0
**Haiti**	3	0
**Honduras**	3	0
**Hungary**	3	24
**Iceland**	3	51
**India**	3	0
**Indonesia**	3	0
**Iran**	3	0
**Iraq**	3	0
**Ireland**	3	51
**Israel**	3	51
**Italy**	3	34
**Jamaica**	3	0
**Japan**	3	51
**Jordan**	3	0
**Kazakhstan**	3	0
**Kenya**	3	0
**Kiribati**	3	0
**Kuwait**	3	51
**Kyrgyzstan**	3	0
**Lao People's Democratic Republic**	3	0
**Latvia**	3	18
**Lebanon**	3	0
**Lesotho**	3	0
**Liberia**	3	0
**Libya**	3	0
**Lithuania**	3	15
**Luxembourg**	3	51
**Madagascar**	3	0
**Malawi**	3	0
**Malaysia**	3	0
**Maldives**	3	0
**Mali**	3	0
**Malta**	3	16
**Marshall Islands**	3	0
**Mauritania**	3	0
**Mauritius**	3	0
**Mexico**	3	0
**Micronesia (Federated States of)**	3	0
**Monaco**	3	34

**Mongolia**	3	0
**Montenegro**	3	0
**Morocco**	3	0
**Mozambique**	3	0
**Myanmar**	3	0
**Namibia**	3	0
**Nauru**	3	3
**Nepal**	3	0
**Netherlands**	3	51
**New Zealand**	3	51
**Nicaragua**	3	0
**Niger**	3	0
**Nigeria**	3	0
**Niue**	3	0
**Norway**	3	51
**Oman**	3	30
**Pakistan**	3	0
**Palau**	3	3
**Panama**	3	0
**Papua New Guinea**	3	0
**Paraguay**	3	0
**Peru**	3	0
**Philippines**	3	0
**Poland**	3	24
**Portugal**	3	51
**Qatar**	3	51
**Republic of Korea**	3	48
**Republic of Moldova**	3	0
**Romania**	3	0
**Russian Federation**	3	6
**Rwanda**	3	0
**Saint Kitts and Nevis**	3	18
**Saint Lucia**	3	0
**Saint Vincent and the Grenadines**	3	0
**Samoa**	3	0
**San Marino**	3	34
**Sao Tome and Principe**	3	0
**Saudi Arabia**	3	39
**Senegal**	3	0
**Serbia**	3	0
**Seychelles**	3	9
**Sierra Leone**	3	0
**Singapore**	3	51
**Slovakia**	3	30
**Slovenia**	3	51
**Solomon Islands**	3	0
**Somalia**	3	0
**South Africa**	3	0
**Spain**	3	51
**Sri Lanka**	3	0
**Sudan**	3	0
**Suriname**	3	0
**Swaziland**	3	0

**Sweden**	3	51
**Switzerland**	3	51
**Syria**	3	0
**Tajikistan**	3	0
**Thailand**	3	0
**The former Yugoslav Republic of Macedonia**	3	0
**Timor-Leste**	3	0
**Togo**	3	0
**Tonga**	3	0
**Trinidad and Tobago**	3	33
**Tunisia**	3	0
**Turkey**	3	0
**Turkmenistan**	3	0
**Tuvalu**	3	0
**Uganda**	3	0
**Ukraine**	3	0
**United Arab Emirates**	3	51
**United Kingdom**	3	34
**United Republic of Tanzania**	3	0
**United States of America**	3	51
**Uruguay**	3	15
**Uzbekistan**	3	0
**Vanuatu**	3	0
**Venezuela (Bolivarian Republic of)**	3	3
**Viet Nam**	3	0
**Yemen**	3	0
**Zambia**	3	0
**Zimbabwe**	3	0
**Afghanistan**	4	0
**Albania**	4	0
**Algeria**	4	0
**Andorra**	4	0
**Angola**	4	0
**Antigua and Barbuda**	4	0
**Argentina**	4	0
**Armenia**	4	0
**Australia**	4	0
**Austria**	4	0
**Azerbaijan**	4	0
**Bahamas**	4	0
**Bahrain**	4	0
**Bangladesh**	4	0
**Barbados**	4	0
**Belarus**	4	0
**Belgium**	4	0
**Belize**	4	0
**Benin**	4	0
**Bhutan**	4	0
**Bolivia Plurinational States of**	4	0
**Bosnia and Herzegovina**	4	0
**Botswana**	4	0
**Brazil**	4	0
**Brunei Darussalam**	4	0

**Bulgaria**	4	0
**Burkina Faso**	4	0
**Burundi**	4	0
**Cabo Verde Republic of**	4	0
**Cambodia**	4	0
**Cameroon**	4	0
**Canada**	4	0
**Central African Republic**	4	0
**Chad**	4	0
**Chile**	4	0
**China**	4	0
**Colombia**	4	0
**Comoros**	4	0
**Congo**	4	0
**Cook Islands**	4	0
**Costa Rica**	4	0
**Cote d'Ivoire**	4	0
**Croatia**	4	0
**Cuba**	4	0
**Cyprus**	4	0
**Czech Republic**	4	0
**Democratic Republic of the Congo**	4	0
**Denmark**	4	0
**Djibouti**	4	1
**Dominica**	4	0
**Dominican Republic**	4	0
**Ecuador**	4	0
**Egypt**	4	0
**El Salvador**	4	0
**Equatorial Guinea**	4	0
**Eritrea**	4	0
**Estonia**	4	0
**Ethiopia**	4	0
**Fiji**	4	0
**Finland**	4	0
**France**	4	0
**Gabon**	4	0
**Gambia**	4	0
**Georgia**	4	0
**Germany**	4	0
**Ghana**	4	0
**Greece**	4	0
**Grenada**	4	0
**Guatemala**	4	0
**Guinea**	4	0
**Guinea-Bissau**	4	0
**Guyana**	4	0
**Haiti**	4	0
**Honduras**	4	0
**Hungary**	4	0
**Iceland**	4	0
**India**	4	0
**Indonesia**	4	0

**Iran**	4	0
**Iraq**	4	0
**Ireland**	4	0
**Israel**	4	0
**Italy**	4	0
**Jamaica**	4	0
**Japan**	4	0
**Jordan**	4	0
**Kazakhstan**	4	0
**Kenya**	4	0
**Kiribati**	4	0
**Kuwait**	4	0
**Kyrgyzstan**	4	0
**Lao People's Democratic Republic**	4	0
**Latvia**	4	0
**Lebanon**	4	0
**Lesotho**	4	0
**Liberia**	4	0
**Libya**	4	0
**Lithuania**	4	0
**Luxembourg**	4	0
**Madagascar**	4	0
**Malawi**	4	0
**Malaysia**	4	0
**Maldives**	4	0
**Mali**	4	0
**Malta**	4	0
**Marshall Islands**	4	0
**Mauritania**	4	0
**Mauritius**	4	0
**Mexico**	4	0
**Micronesia (Federated States of)**	4	0
**Monaco**	4	0
**Mongolia**	4	0
**Montenegro**	4	0
**Morocco**	4	0
**Mozambique**	4	0
**Myanmar**	4	0
**Namibia**	4	0
**Nauru**	4	0
**Nepal**	4	0
**Netherlands**	4	0
**New Zealand**	4	0
**Nicaragua**	4	0
**Niger**	4	0
**Nigeria**	4	0
**Niue**	4	0
**Norway**	4	0
**Oman**	4	0
**Pakistan**	4	0
**Palau**	4	0
**Panama**	4	0
**Papua New Guinea**	4	0

**Paraguay**	4	0
**Peru**	4	0
**Philippines**	4	0
**Poland**	4	0
**Portugal**	4	0
**Qatar**	4	0
**Republic of Korea**	4	0
**Republic of Moldova**	4	0
**Romania**	4	0
**Russian Federation**	4	0
**Rwanda**	4	0
**Saint Kitts and Nevis**	4	0
**Saint Lucia**	4	0
**Saint Vincent and the Grenadines**	4	0
**Samoa**	4	0
**San Marino**	4	0
**Sao Tome and Principe**	4	0
**Saudi Arabia**	4	0
**Senegal**	4	0
**Serbia**	4	0
**Seychelles**	4	0
**Sierra Leone**	4	0
**Singapore**	4	0
**Slovakia**	4	0
**Slovenia**	4	0
**Solomon Islands**	4	0
**Somalia**	4	0
**South Africa**	4	0
**Spain**	4	0
**Sri Lanka**	4	0
**Sudan**	4	0
**Suriname**	4	0
**Swaziland**	4	0
**Sweden**	4	0
**Switzerland**	4	0
**Syria**	4	0
**Tajikistan**	4	0
**Thailand**	4	0
**The former Yugoslav Republic of Macedonia**	4	0
**Timor-Leste**	4	0
**Togo**	4	0
**Tonga**	4	0
**Trinidad and Tobago**	4	0
**Tunisia**	4	0
**Turkey**	4	0
**Turkmenistan**	4	0
**Tuvalu**	4	0
**Uganda**	4	0
**Ukraine**	4	0
**United Arab Emirates**	4	0
**United Kingdom**	4	0
**United Republic of Tanzania**	4	0
**United States of America**	4	0

**Uruguay**	4	0
**Uzbekistan**	4	0
**Vanuatu**	4	0
**Venezuela (Bolivarian Republic of)**	4	0
**Viet Nam**	4	0
**Yemen**	4	0
**Zambia**	4	0
**Zimbabwe**	4	0
**Afghanistan**	5	0
**Albania**	5	0
**Algeria**	5	0
**Andorra**	5	0
**Angola**	5	0
**Antigua and Barbuda**	5	0
**Argentina**	5	0
**Armenia**	5	0
**Australia**	5	0
**Austria**	5	0
**Azerbaijan**	5	0
**Bahamas**	5	0
**Bahrain**	5	0
**Bangladesh**	5	16
**Barbados**	5	0
**Belarus**	5	0
**Belgium**	5	0
**Belize**	5	0
**Benin**	5	0
**Bhutan**	5	0
**Bolivia Plurinational States of**	5	0
**Bosnia and Herzegovina**	5	0
**Botswana**	5	0
**Brazil**	5	0
**Brunei Darussalam**	5	0
**Bulgaria**	5	0
**Burkina Faso**	5	0
**Burundi**	5	0
**Cabo Verde Republic of**	5	3
**Cambodia**	5	0
**Cameroon**	5	0
**Canada**	5	0
**Central African Republic**	5	0
**Chad**	5	1
**Chile**	5	0
**China**	5	0
**Colombia**	5	0
**Comoros**	5	0
**Congo**	5	4
**Cook Islands**	5	0
**Costa Rica**	5	0
**Cote d'Ivoire**	5	0
**Croatia**	5	0
**Cuba**	5	0
**Cyprus**	5	0

**Czech Republic**	5	0
**Democratic Republic of the Congo**	5	0
**Denmark**	5	0
**Djibouti**	5	3
**Dominica**	5	0
**Dominican Republic**	5	0
**Ecuador**	5	0
**Egypt**	5	0
**El Salvador**	5	0
**Equatorial Guinea**	5	0
**Eritrea**	5	17
**Estonia**	5	0
**Ethiopia**	5	1
**Fiji**	5	15
**Finland**	5	0
**France**	5	0
**Gabon**	5	6
**Gambia**	5	0
**Georgia**	5	0
**Germany**	5	0
**Ghana**	5	0
**Greece**	5	0
**Grenada**	5	0
**Guatemala**	5	0
**Guinea**	5	0
**Guinea-Bissau**	5	0
**Guyana**	5	0
**Haiti**	5	0
**Honduras**	5	0
**Hungary**	5	0
**Iceland**	5	0
**India**	5	0
**Indonesia**	5	0
**Iran**	5	0
**Iraq**	5	0
**Ireland**	5	0
**Israel**	5	0
**Italy**	5	0
**Jamaica**	5	0
**Japan**	5	0
**Jordan**	5	0
**Kazakhstan**	5	0
**Kenya**	5	2
**Kiribati**	5	0
**Kuwait**	5	0
**Kyrgyzstan**	5	0
**Lao People's Democratic Republic**	5	0
**Latvia**	5	0
**Lebanon**	5	0
**Lesotho**	5	0
**Liberia**	5	1
**Libya**	5	0
**Lithuania**	5	0

**Luxembourg**	5	0
**Madagascar**	5	0
**Malawi**	5	0
**Malaysia**	5	0
**Maldives**	5	0
**Mali**	5	4
**Malta**	5	0
**Marshall Islands**	5	0
**Mauritania**	5	0
**Mauritius**	5	1
**Mexico**	5	11
**Micronesia (Federated States of)**	5	0
**Monaco**	5	0
**Mongolia**	5	0
**Montenegro**	5	0
**Morocco**	5	0
**Mozambique**	5	0
**Myanmar**	5	0
**Namibia**	5	2
**Nauru**	5	0
**Nepal**	5	0
**Netherlands**	5	0
**New Zealand**	5	0
**Nicaragua**	5	0
**Niger**	5	0
**Nigeria**	5	0
**Niue**	5	0
**Norway**	5	0
**Oman**	5	0
**Pakistan**	5	0
**Palau**	5	0
**Panama**	5	0
**Papua New Guinea**	5	0
**Paraguay**	5	0
**Peru**	5	0
**Philippines**	5	0
**Poland**	5	0
**Portugal**	5	0
**Qatar**	5	0
**Republic of Korea**	5	0
**Republic of Moldova**	5	0
**Romania**	5	0
**Russian Federation**	5	0
**Rwanda**	5	0
**Saint Kitts and Nevis**	5	0
**Saint Lucia**	5	0
**Saint Vincent and the Grenadines**	5	0
**Samoa**	5	0
**San Marino**	5	0
**Sao Tome and Principe**	5	17
**Saudi Arabia**	5	0
**Senegal**	5	0
**Serbia**	5	1

**Seychelles**	5	1
**Sierra Leone**	5	0
**Singapore**	5	0
**Slovakia**	5	0
**Slovenia**	5	0
**Solomon Islands**	5	0
**Somalia**	5	0
**South Africa**	5	16
**Spain**	5	0
**Sri Lanka**	5	0
**Sudan**	5	2
**Suriname**	5	0
**Swaziland**	5	0
**Sweden**	5	0
**Switzerland**	5	0
**Syria**	5	0
**Tajikistan**	5	0
**Thailand**	5	1
**The former Yugoslav Republic of Macedonia**	5	0
**Timor-Leste**	5	0
**Togo**	5	0
**Tonga**	5	0
**Trinidad and Tobago**	5	0
**Tunisia**	5	0
**Turkey**	5	0
**Turkmenistan**	5	0
**Tuvalu**	5	0
**Uganda**	5	0
**Ukraine**	5	0
**United Arab Emirates**	5	0
**United Kingdom**	5	0
**United Republic of Tanzania**	5	0
**United States of America**	5	0
**Uruguay**	5	0
**Uzbekistan**	5	0
**Vanuatu**	5	0
**Venezuela (Bolivarian Republic of)**	5	0
**Viet Nam**	5	13
**Yemen**	5	0
**Zambia**	5	0
**Zimbabwe**	5	0
**GBD-High income**	0	357
**GBD-High income**	1	9
**GBD-High income**	2	0
**GBD-High income**	3	4207
**GBD-High income**	4	0
**GBD-High income**	5	0
**High income**	0	1769
**High income**	1	9
**High income**	2	0
**High income**	3	4207
**High income**	4	0
**High income**	5	1

**Low income**	0	4647
**Low income**	1	89
**Low income**	2	0
**Low income**	3	0
**Low income**	4	0
**Low income**	5	40
**Lower-middle income**	0	6616
**Lower-middle income**	1	49
**Lower-middle income**	2	21
**Lower-middle income**	3	0
**Lower-middle income**	4	1
**Lower-middle income**	5	62
**Upper-middle income**	0	7955
**Upper-middle income**	1	14
**Upper-middle income**	2	6
**Upper-middle income**	3	98
**Upper-middle income**	4	0
**Upper-middle income**	5	53

Statistical model to fill missingness in health expenditure variables

After the cleaning up of the data previously described above, we used Spatiotemporal Gaussian process regression (ST-GPR) in order to predict and fill out the missingness that existed in the resulting health expenditure dataset. ST-GPR is a stochastic modeling technique designed to detect signals amidst noisy data. It also serves as a powerful tool for interpolating non-linear trends. Unlike classical linear models that assume that the trend underlying data follows a definitive functional form, GPR assumes that the specific trend of interest follows a gaussian process, where each point can be estimated with a mean and covariance function.

The first step to implementing ST-GPR is to identify relevant covariates that would be helpful in predicting each health expenditure variable of interest. Using the following set of covariates, we estimated the first stage of the process (space-time) in order to predict and fill up the dependent variables. The covariates used are:

a) All-sector government expenditure per capita, logged
b) Healthcare Access and Quality Index, logged
c) Proportion of total population above the age of 65, logit transformed.

where the dependent variables were logs of GHE per capita, PPP per capita and OOP per capita.

Given the weight of data, we were able to adjust the weight of each data point that contributed to the likelihood function of the Gaussian process, by inflating the pointwise variance for data points with lower weights. For missing data points, the resulting uncertainty was determined by region specific estimates. The final resulting dataset was a complete set of GHE, PPP and OOP per capita estimates for 195 countries from 1995 through 2016, where the uncertainty around each point was constructed by simulating from a normal distribution. Goodness-of-fit was determined using out-of-sample validation and choosing the models with the best performing set of hyperparameters. Overall, ST-GPR is a stochastic modeling technique that performs well at detecting signals among datasets that are noisy. It deviates from classic linear models that assume trend of its underlying data must follow a definitive functional form. Instead, GPR assumes the trend must follow a Gaussian Process form, having a covariance function and a pointwise mean. For the distribution of the Gaussian process, we used a Matérn covariance matrix. GPRs were run repeatedly to ensure that every country-year estimate per metric had 1,000 draws.

This ST-GPR process follows the same form of that found in the Global Burden of Disease Study 2017 appendix.[3] This analysis was conducted in the following programs: Stata (version 13.1) and R (version 3.5.1).

eFigure 1 below shows the comparison of WHO GHED extracted data and IHME ST-GPR THE in GDP per capita space from 2000-2016. eFigure 2 shows the comparison of WHO GHED extracted data and IHME ST-GPR THE in per GDP space from 2000-2016.

eFigure 1. Comparison of WHO GHED extracted and IHME ST-GPR THE *in GDPpc* space, 2000-2016

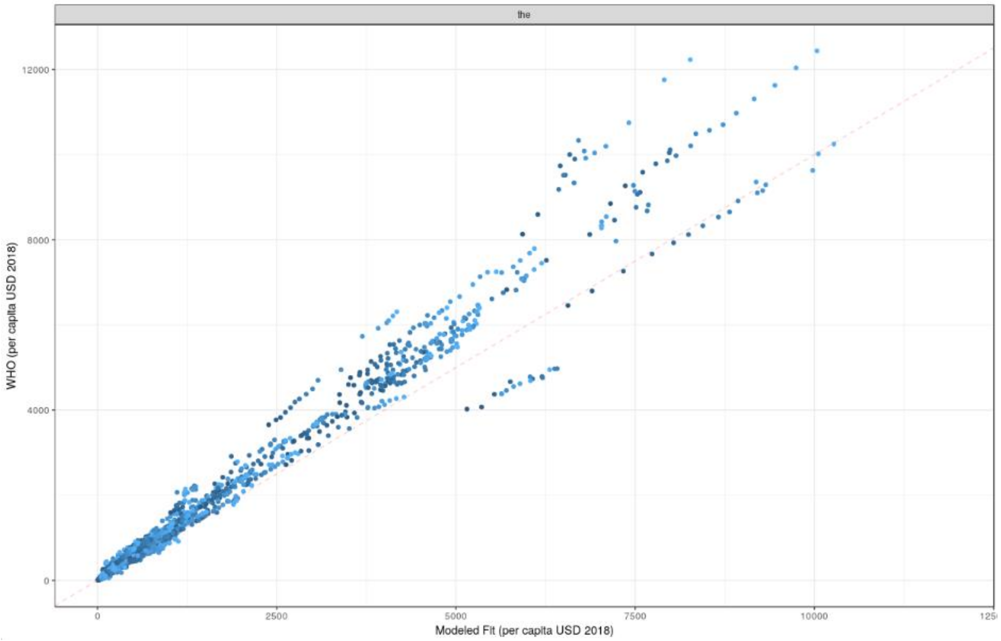

eFigure 2. Comparison of WHO GHED extracted and IHME ST-GPR THE *in per GDP* space, 2000-2016

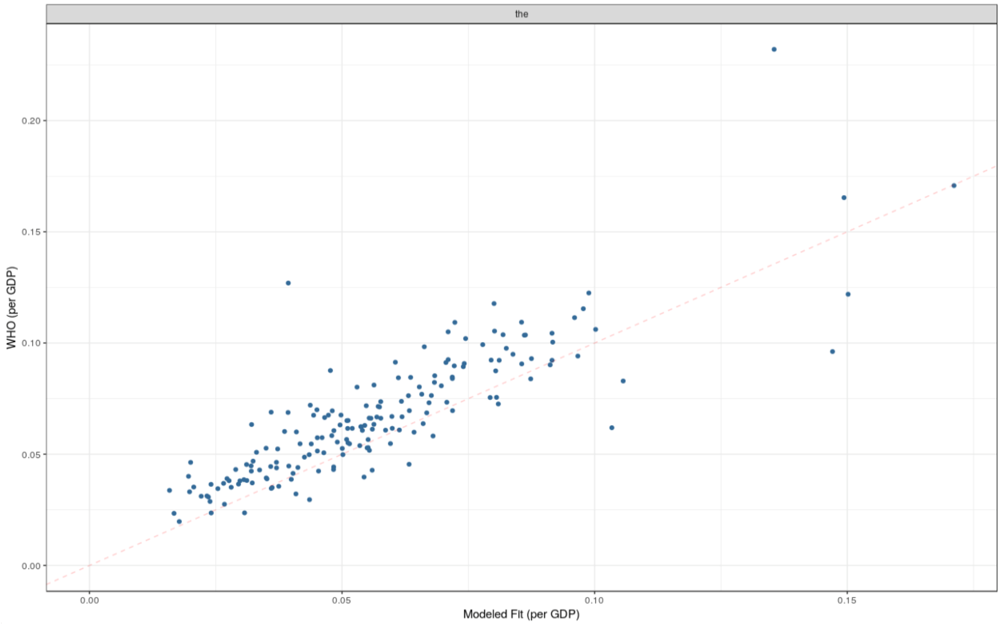

To make results in terms of percent of GDP evident we have added new versions of figures 1, 2, 3, and 8, remade using percent of GDP rather than US dollar below. They have been added as eFigures 3, 4, 5, and 6, respectively.

eFigure 3. Health spending per GDP in 1995, 2016, 2030, and 2050

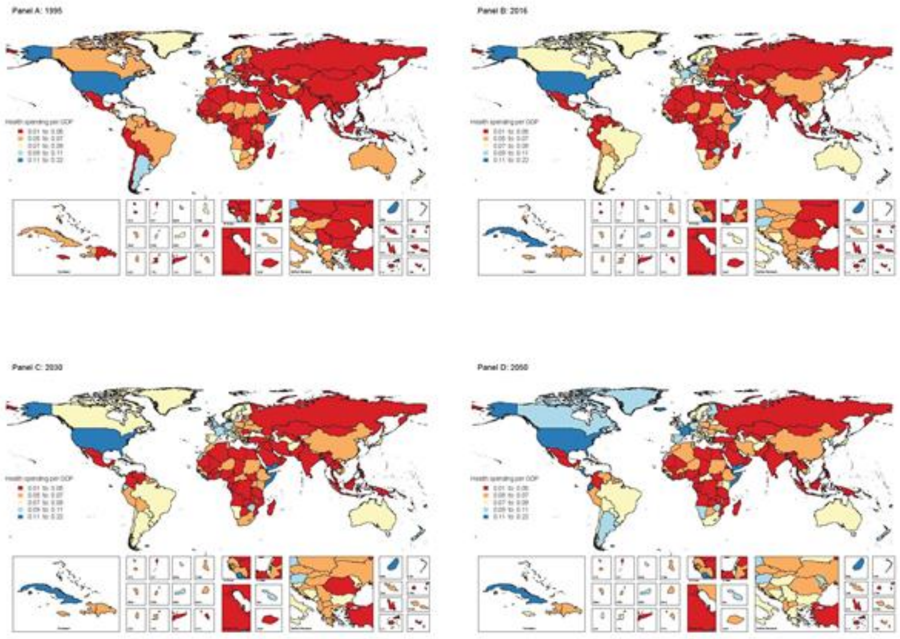

eFigure 4. Health spending per GDP by gross domestic product per capita, 1995, 2016, 2030, and 2050

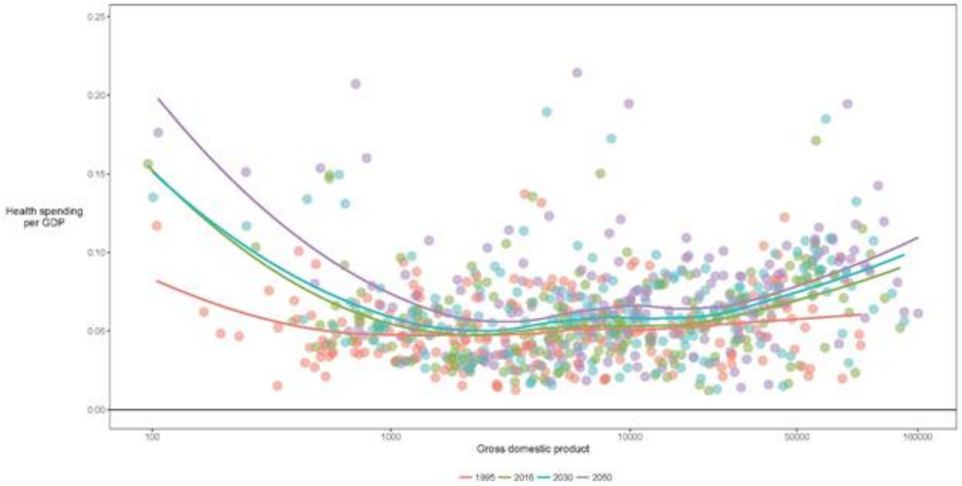

eFigure 5. Annualised rate of change in health spending per GDP by source, by income group [A] and Global Burden of Disease super-region [B], 1995–2016

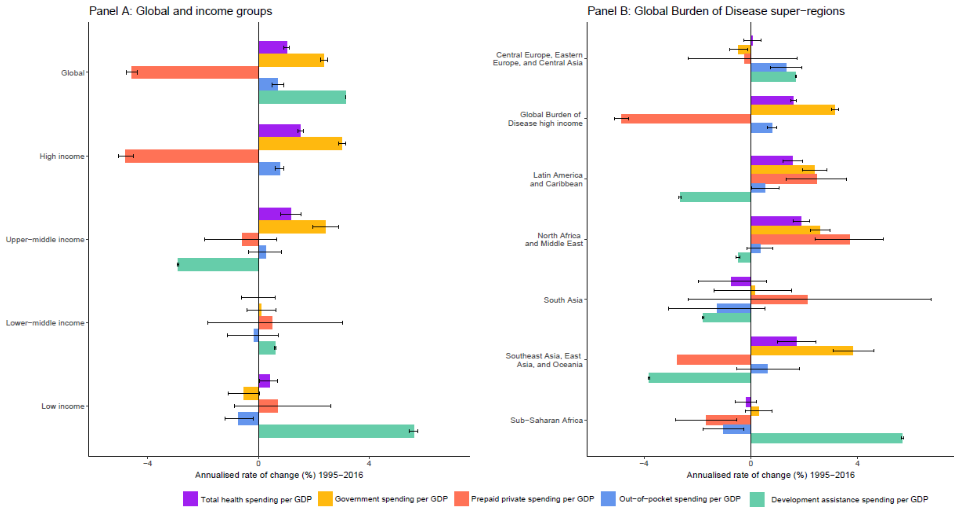

eFigure 6. Distribution of government health spending per GDP, global and by income group, 1995, 2016, 2030, 2050, and two future scenarios

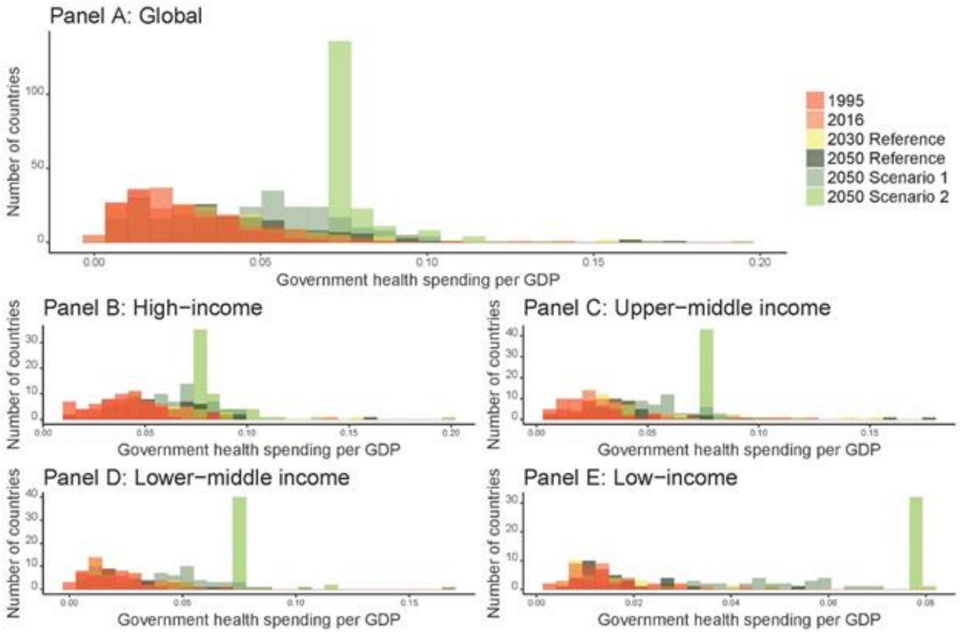

eFigure 7 is a process diagram that displays the flow of input data and models for each step of the forecasting process. eFigure 8 explores how the sources of health spending tend to evolve with economic development showing this relationship in past and future years.

eFigure 7. Flow of input data and models for each step of the forecasting process

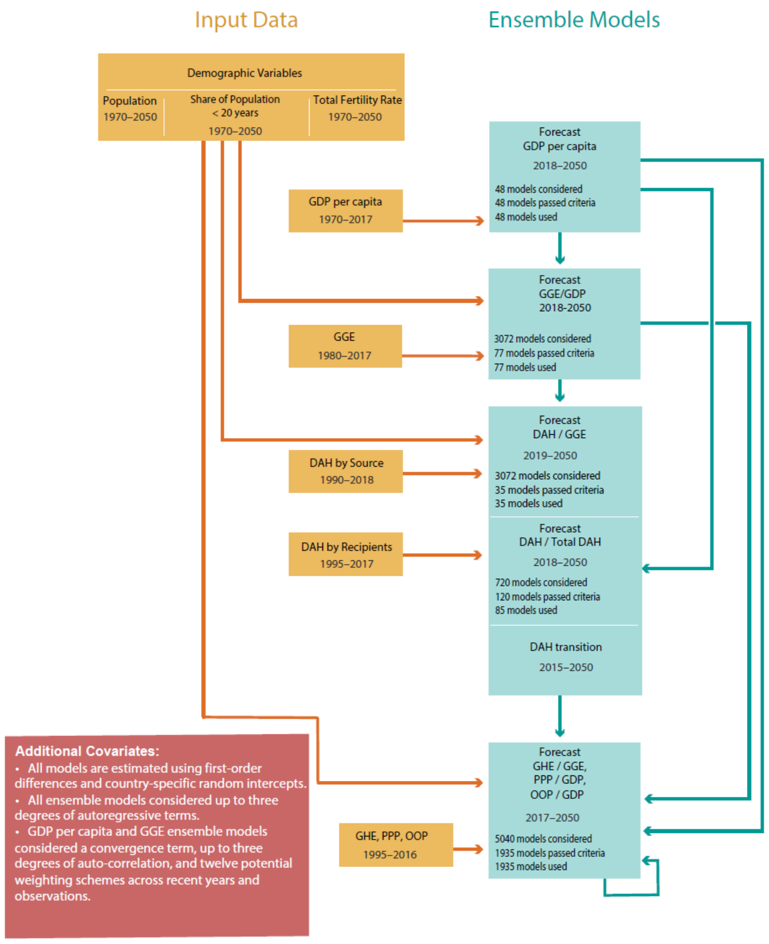

eFigure 8. Economic development and the composition of health spending by source and proportion of health spending from the government, 1995, 2016, 2030, 2050, composition by source

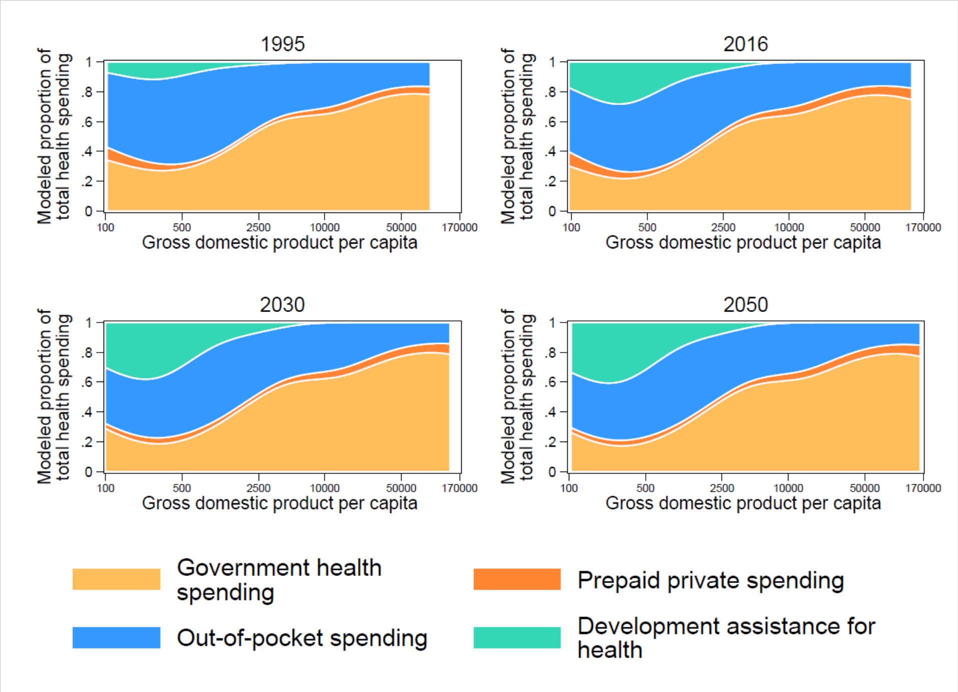

References

1Global Health Expenditure Database. World Health Organ. http://apps.who.int/nha/database (accessed Dec 21, 2018).

2World Bank Country and Lending Groups – World Bank Data Help Desk. World Bank. https://datahelpdesk.worldbank.org/knowledgebase/articles/906519-world-bank-country-and-lending-groups (accessed Dec 21, 2018).

3Stanaway JD, Afshin A, Gakidou E, *et al.* Global, regional, and national comparative risk assessment of 84 behavioural, environmental and occupational, and metabolic risks or clusters of risks for 195 countries and territories, 1990–2017: a systematic analysis for the Global Burden of Disease Study 2017. *The Lancet* 2018; **392**: 1923–94.

4 OECD. OECD Statistics [Internet]. [cited 2019 Dec 19].

Available from https://stats/oecd/org/index.aspx?queryid=169

SECTION 3. TRACKING DEVELOPMENT ASSISTANCE FOR HEALTH FROM TRADITIONAL DONORS

List of Figures

eFigure 1 Comparing CRS commitments, CRS disbursements, and DAC commitments

eFigure 2 CRS disbursement to commitment ratio and cutoff points by donor agency

eFigure 3 One- to six-year disbursement schedules for bilateral channels

eFigure 4 DAH as a percentage of corresponding budget data by bilateral agency

eFigure 5 Malaria DAH to program areas as assigned by keyword search and PMI reports

eFigure 6 World Bank's annual health sector commitments and disbursements

eFigure 7 Disbursements by the African Development Bank

eFigure 8 Disbursements by Asian Development Bank

eFigure 9 Disbursements by Inter-American Development Bank

eFigure 10 Contributions received by the Global Fund to Fight AIDS, Tuberculosis and Malaria

eFigure 11 The Global Fund to Fight AIDS, Tuberculosis and Malaria's commitments and disbursements

eFigure 12 Gavi's income and disbursements

eFigure 13 Total revenue received by non-governmental organizations

eFigure 14 Expenditure by non-governmental organizations

eFigure 15 In-kind contributions by loan- and grant-making DAH channels of assistance

List of Tables

eTable 1. Definitions of health spending sources
eTable 2. Summary of primary data sources and databases

eTable 3. Data sources received via personal correspondence

eTable 4. Additional data sources, databases and model choices used for preliminary estimates of DAH

eTable 5. Terms for keyword searches

eTable 6. Additional health focus area categorizations

eTable 7 World Bank's health sector and theme codes

eTable 8 Summary of data sources for the regional development banks

eTable 9 Summary of US non-governmental organizations in the study

eTable 10 Summary of data sources for calculating in-kind contributions

eTable 11. Rules for assigning level values to metadata

eTable 12. Supplemental table for data classification

eTable 13. Supplemental table: Gross Domestic Product

eTable 14. Supplemental table: Total expenditure on health

eTable 15. Supplemental table: General government expenditure on health

eTable 16. Supplemental table: Out of pocket expenditures

eTable 17. Supplemental table: Pre-paid private

eTable 18. Count of data sources, by financing sources and spending functions

eTable 19. Count of data sources, by GBD super region

Box 1. EXAMPLE - Australia's primary and additional data sources

Box 2. EXAMPLE. Post-keyword search weighting

Box 3. EXAMPLE. Australia's CRS disbursement to commitment ratio and cutoff year

Box 4. EXAMPLE. Australia's one- to six-year disbursement schedules

Box 5. EXAMPLE. Australia's DAH as a percentage of corresponding budget data

Overview

Development assistance for health (DAH) estimates were obtained from the Institute for Health Metrics and Evaluation's development assistance for health database. We summarize the original methodology as well as updates for this year's estimates below. A more detailed description of the original methodology used to obtain the estimates in the database can be found in Dieleman et al.[1] All known, systematically reported, available data on health-related disbursements and expenditures were extracted, as well as income and revenue from existing project databases, annual reports, and audited financial statements. The channels included and the corresponding data sources are summarized in eTable 2. Data sources obtained via personal correspondence are summarized in eTable 3.

DAH for bilateral agencies included all health-related disbursements from bilateral donor agencies, excluding funds that they transferred to any of the other channels we tracked in order to avoid double-counting. This information was extracted from the Creditor Reporting System (CRS) and Development Assistance Committee (DAC) databases of the Development Assistance Committee of the Organisation for Economic Co-operation and Development (OECD-DAC). In some cases, donor agencies did not report disbursement data to the CRS. A method for predicting disbursements from commitment data was implemented to address this challenge. For detailed description of this method see Tracking Development Assistance for Health from Bilateral Aid Agencies and the European Commission section below as well as in Dieleman et al. [1]

For other grant- and loan-making institutions, annual disbursements on health grants and loans were similarly included, excluding transfers to any other channels and ignoring any repayments on outstanding debts. For a more detailed description of this process see Dieleman et al.[1] The annual disbursements for grant- and loan-making institutions only reflect the financial transfers made by these agencies. Therefore, in-kind transfers from these institutions in the form of staff time for providing technical assistance and the costs of managing programs were estimated separately.[1]

Estimates of DAH for the United Nations (UN) agencies included annual expenditures on health both from their core budgets and from voluntary contributions. Calculating DAH for the United Nations Children's Fund (UNICEF) involved estimating the fraction of its total expenditure spent on health prior to 2001.[1]

Non-governmental organizations' (NGOs) DAH estimates utilized data from US government sources, Guidestar Research Fundamental Plus dataset and a survey of health expenditure for a sample of NGOs to estimate DAH from US-based and internationally based NGOs receiving support from the US government. We were unable to include other NGOs due to the lack of audited and comparable data.

The database also included an analysis of the composition of health funding by recipient country, health focus area and program area. In this round of updates to the databases we have made several important improvements. These improvements include the inclusion of China as a bilateral source of funding, the inclusion of European Economic Area, and Coalition for Epidemic Preparedness Innovations as disbursing agencies of DAH funding, the addition of new program areas – antimicrobial resistance/drug resistance under the HIV/AIDS, tuberculosis, malaria, and other infectious diseases health focus areas, the disaggregation of human resources for health within the Sector wide approach and Health system strengthening (SWAp/HSS) health focus area, and modifications to our health focus area key word search terms. All methodological updates made are detailed in their relevant channel sub-section below. The improvements to our health focus area keyword search terms are detailed in eTable 5 and in the section below titled Disaggregation by health focus area, respectively.

For many channels, reporting-time lags prevent primary disbursement data for the most recent year(s). For those years, the values of DAH were predicted, using channel-specific time trends. The methods employed to obtain these predictions are summarized in eTable 4. In general, these methods depend on data availability. The estimates are based on channel-specific budget, commitment, and appropriations data, and in many cases assume the most recent disbursement patterns persist. Due to the lack of more detailed disaggregated data, estimates for the most recent year are not provided for recipient countries.

We predicted ebola funding in 2018 for bilateral sources and the European Commission by assuming that 2018 ebola funding was equal to 2017 ebola funding.

In addition, for SWAp/HSS funding, we allocated SWAP/HSS projects with multiple health focus areas identified by a proportional allocation based on the relative proportions of the project going to the various health focus areas.

For countries that only began existing in certain years, we backcasted DAH in years before their existence as follows. For countries that split off from parent countries, we calculated a three-year average ratio of child country DAH received to parent country DAH received. In years before the child country split off, DAH received by the parent country would have included DAH received in the region that would split off to become the child country. Therefore we reallocated funding from the parent country, in all years before the child country split off, adding this proportion of the parent country's DAH to the child country's DAH and subtracting out this value from the parent country's DAH. By this method, total annual DAH between the parent and child country do not change, but the allocation of funding between the parent country and child country change. For any country that ceased to exist (such as former Yugoslavia and former USSR) and that had observed DAH received in certain years, we split the funding equally among its new constituent countries.

Currency exchange and deflation

All results are presented in real 2018 US dollars. All disbursement sequences were converted into real 2018 US dollars by taking disbursements in nominal US dollars in the year of disbursement and adjusting these sequences into real 2018 US dollars using US gross domestic product (GDP) deflators. Analyses were conducted in Stata (version 13.1).

eTable 2. Summary of primary data sources and databases

Channel	Source
**Bilateral agencies**	OECD-DAC and CRS databases[2]
**European Commission**	OECD-DAC and CRS databases[3]
**Joint United Nations Programme on HIV/AIDS (UNAIDS)**	Financial reports and audited financial statements[4]

**United Nations Children's Fund (UNICEF)**	Financial reports and audited financial statements[5–7]
**United Nations Population Fund (UNFPA)**	Financial reports and audited financial statements[8]
**UNITAID**	Project level data from IATI database shared through correspondence, financial reports and audited financial statements[9]
**Pan American Health Organization (PAHO)**	Financial reports and audited financial statements[10]
**World Health Organization (WHO)**	Financial reports and audited financial statements[11]
**World Bank**	Online project database and correspondence[12,13]
**African Development Bank (AfDB)**	Correspondence and compendium of statistics[14,15]
**Inter-American Development Bank (IDB)**	Online project database and correspondence[16,17]
**Gavi, the Vaccine Alliance**	Online project database, cash received database, International Finance Facility for Immunisation (IFFIm) annual reports, Advance Market Commitment for Pneumococcal Vaccines (AMC) annual reports, and annual reports[18–21]
**The Global Fund to Fight AIDS, Tuberculosis and Malaria (GFATM)**	Online grant database, contributions report and annual reports[22–24]
**Coalition for Epidemic Preparedness and Innovation (CEPI)**	Project level data through correspondence
**NGOs registered in the US**	United States Agency for International Development (USAID) Report of Voluntary Agencies (VolAg), tax filings, annual reports, financial statements, RED BOOK Expanded Database, and WHO's Model List of Essential Medicines, Guidestar Research Fundamentals Plus dataset[25–29]
**Bill & Melinda Gates Foundation (BMGF)**	Online grant database, IRS 990 tax forms, and correspondence[30,31]
**Other private US foundations**	Foundation Center's grants database[32]
**Wellcome Trust**	Project level data through correspondence
**European Economic Area**	Online project database[33] and correspondence[34]
**China**	Financial reports and yearbooks

eTable 3. Data sources received via personal correspondence

Channel	Data received
**World Bank**	Health project-level disbursement data, 1990 – August 2018[13]
**BMGF**	Health disbursement data, 2017[31]
**CEPI**	Health disbursement data, 2018
**IDB**	Health project-level loan disbursement data, 2018[17]
**African Development Bank**	Health project-level disbursement data, 2002 – December 2018[35]
**Asian Development Bank**	Health project-level disbursement data, 1990-2018
**UNITAID**	Health project-level disbursement data, 2007- 2018[36]
**UAE bilateral**	UAE Foreign Assistance in Health 1990 - 2008[37]
**Wellcome Trust**	Health project-level disbursement data, 1990-2018
**European Economic Area**	Health project-level disbursement data, 2007-2018

eTable 4. Additional data sources, databases and model choices used for preliminary estimates of DAH

Channel	Data source	Variables used	Years of budget data used for modeling*	Years underlying DAH data not available; thus modeled*	Model used
**National agencies**					
**Australia**	Australia's International Development Assistance (2008-2018); Australia's Overseas Aid Program (1998-2008)[38,39]	Health official development assistance (ODA): International development assistance budget	1998-2018	2018	Weighted average of actual DAH/budgeted DAH
**Austria**	Austria Federal Ministry of Finance budget[40]	General ODA: Federal ODA budget	2007-2018	2018	Weighted average of DAH/budgeted ODA
**Belgium**	Project Budget General – general expenses[41]	General ODA: Foreign affairs, foreign trade development and cooperation	2000-2018	2018	Weighted average of DAH/budgeted ODA
**Canada**	Canadian International Development Agency – Report on Plans and Priorities[42]	General ODA: Financial summary – planned spending	1996-2018	2018	Weighted average of DAH/budgeted ODA
**Denmark**	Danish Ministry of Foreign Affairs Budget[43]	General ODA: Budgeted expenditures on overseas development assistance	2000-2018	2018	Weighted average of DAH/budgeted ODA
**European Commission**	General budget[44]	Data not used as they were inconsistent with disbursements	–	2018	Based on weighted average of trends in member countries
**Finland**	Document Assembly in budget years 1998-2018[45]	General ODA: Ministry of Foreign Affairs' administrative appropriations,	2002-2018	2018	Weighted average of DAH/budgeted ODA

Channel	Data source	Variables used	Years of budget data used for modeling*	Years underlying DAH data not available; thus modeled*	Model used
		international development			
**France**	Budget and Financial documents[46,47]	General ODA: aggregated project data; Total ODA	2009-2018	2018	Weighted average of DAH/budgeted ODA
**Germany**	Plan of the Federal Budget[48]	General ODA: Development expenditure	2001-2018	2018	Weighted average of DAH/budgeted ODA
**Greece**	Ministry of Finance Budget (2013-2018); OECD Data (1996-2017)[2,49,50]	General ODA; ODA commitments	1996-2018	2018	Weighted average of DAH/budgeted ODA
**Ireland**	Department of Finance – budget 2000-2004; Estimates for Public Services and Summary Public Capital Programme, 2005-2018 [51]	General ODA: Summary of adjustments to gross current estimates – international co-operation	2002-2018	2018	Weighted average of DAH/budgeted ODA
**Italy**	The Italian Agency for Development Cooperation[52]	General ODA: Net development corporation	2007-2018	2018	Weighted average of DAH/budgeted ODA
**Japan**	Highlights of the Budget for FY1999-2018[53]	General ODA: Major budget expenditures	2003-2018	2018	Weighted average of DAH/budgeted ODA
**Korea, South**	ODA Korea comprehensive implementation plan[54]	General ODA: Plan for international development cooperation	2008-2018	2018	Weighted average of DAH/budgeted ODA
**Luxembourg**	State Budget[55]	General ODA: Ministry of Foreign Affairs – budgeted international development cooperation and humanitarian aid	2001-2018	2018	Weighted average of DAH/budgeted ODA
**Netherlands**	Netherlands International	General ODA: Total annual	2001-2018	2018	Weighted average of

Channel	Data source	Variables used	Years of budget data used for modeling*	Years underlying DAH data not available; thus modeled*	Model used
	Cooperation Budget (2001-2016)	official development assistance expenditure			DAH/budgeted ODA
**New Zealand**	Vote Foreign Affairs and Trade (1998-2001); VOTE Official Development Assistance (2002-2018)[56]	General ODA: Total annual official development assistance expenditure	1998-2018	2018	Weighted average of DAH/budgeted ODA
**Norway**	Norwegian Ministry of Finance National Budget (2014-2018); Correspondence (2000-2013)[57,58]	General ODA: ODA budget	2000-2018	2018	Weighted average of DAH/budgeted ODA
**Portugal**	Ministry of Finance and Public Administration State Budget 2003-2018[59]	General ODA: Integrated service expenditure – external cooperation budget	2003-2018	2018	Weighted average of DAH/budgeted ODA
**Spain**	Annual Plans of Spanish International Cooperation[60]	General ODA: Spanish total development cooperation	2003-2018	2018	Weighted average of DAH/budgeted ODA
**Sweden**	Correspondence (2000-2010); Ministry of Foreign Affairs Budget (2010-2018)[61]	General ODA: Ministry for Foreign Affairs budgets for expenditure – international development cooperation	2000-2018	2018	Weighted average of DAH/budgeted ODA
**Switzerland**	Foreign Affairs (2000-2006); Budget – Further Explanations and Statistics (2007-2018)	General ODA: Direction of development and cooperation (2000-2006); foreign affairs – international cooperation, development aid (in the	2000-2018	2018	Weighted average of DAH/budgeted ODA

Channel	Data source	Variables used	Years of budget data used for modeling*	Years underlying DAH data not available; thus modeled*	Model used
		South and East) (2007-2016)			
**United Kingdom**	IATA (Department for International Development (DFID)) [62,63]	General ODA: assistance for international development; Sum (revised) - aggregated project data	1998-2018	2018	Weighted average of DAH/budgeted ODA
**United States**	Foreign Assistance Dashboard (2006-2018); Budget of the US Government (2005-2018)[64,65]	Global health ODA: Planned foreign assistance for health; Department of Health and Human Services global health budget	2005-2018	2018	Weighted average of actual DAH/budgeted DAH
**UN agencies**					
**WHO**	Programme budget[66]	DAH budget: Programme budget	2002-2017	2018	Weighted average of DAH/budget
**UNAIDS**	Unified Budget and Workplan, bienniums 2002-2017 and 2018-2019[67,68]	DAH budget: Unified Budget and Workplan	2002-2017	2018	Weighted average of DAH/Core Budget
**UNICEF**	Financial report and audited financial statements; correspondence[7,69,70]	Total expenditure; Total health expenditure	1990-2017	2016-2018	Weighted average of DAH/budget
**UNFPA**	Audited Financial report and contributions report [71,72]	Total health expenditure	2002-2017	2016-2018	Weighted average of DAH/budget
**PAHO**	Proposed program budget[10]	Total regular budget, estimated voluntary contributions	2000-2017	2018	Weighted average of DAH/budget

Channel	Data source	Variables used	Years of budget data used for modeling*	Years underlying DAH data not available; thus modeled*	Model used
**NGOs**	VolAg (1990-2014), GuideStar (2015), sample of top NGOs [25,26]	Revenue breakdowns for: US public, non-US public, private, in-kind, BMGF; total overseas expenditures	1990-2015	2016-2018	Regression on DAH, US GDP, and USAID and private voluntary organization (PVO) revenue

* Years of budget data used for modeling versus years underlying DAH data unavailable thus modeled: The data used to estimate DAH by channel vary across channels. eTable 2 reports our primary data used for each channel. Due to reporting lags there are some years we need to estimate disbursement using additional data sources. These additional data sources, the years in which the primary data is modeled, the years the additional data is available, and the methods for this estimating these modeled years are reported in eTable 4. Years of budget data used for modeling are the years of additional data available to us. We rely on historic trends to inform our estimates so we rely on many years of additional data despite only modeling a few years of primary data. Years underlying DAH data unavailable thus modeled are the years the primary data is incomplete and thus estimated using additional data. See example below for more details for Australia

Box 1. EXAMPLE - Australia's primary and additional data sources
**Project-level data for health-related projects funded by Australia's bilateral aid agencies are available from the OECD's CRS database through 2017. This is the primary data source used to estimate DAH channeled by Australian aid agencies, as described in eTable 2. 2018 is incomplete because of lags in reporting. To estimate DAH disbursed for 2018, additional data are available from Australia's International Development Assistance budget (2008-2018) and Australia's Overseas Aid Program budget (1998-2008), as described in eTable 4. These sources provide health-specific official development assistance (ODA) budgeted by Australia, 1998-2018. We convert countries' budgeted ODA, as given in nominal local currency units, to nominal US dollars using the OECD's currency exchange rate series based on USD monthly averages. To estimate DAH disbursed in 2018, we calculated the ratio of disbursed DAH (from the CRS database) relative to budgeted DAH (from the International Development Assistance and Overseas Aid Program budgets) for 1998-2017. We combine the most recent three ratios into a single estimate by taking a weighted average, weighting substantially higher the most recent year. We multiply this ratio – the estimated disbursed DAH to budgeted DAH – by the 2018 budgeted DAH to estimate disbursed DAH in those years. These methods are described more fully in Dieleman et al.[1]**

DISAGGREGATING BY HEALTH FOCUS AREA

We improved our analysis of the disaggregation of health funding by health focus areas by augmenting our keyword search terms. In particular, we added new keywords to the antimicrobial resistance/drug resistance program area of HIV/AIDS, tuberculosis, malaria, and other infectious diseases health focus areas and human resources for health program areas as well as updated the keywords in all other health focus areas and program areas. Similar to our previous work, the analysis of health focus areas included assessments of development assistance for HIV/AIDS, tuberculosis (TB), malaria, reproductive and maternal health, newborn and child health, other infectious diseases, non-communicable diseases, and SWAps and health system strengthening, using keyword searches within descriptive fields. These were chosen as the areas of focus because of their relevance to current policy debates about global health financing and data availability.

In effect, DAH was disaggregated into eight health focus areas: HIV/AIDS; tuberculosis; malaria; reproductive ,maternal, newborn and child health; non-communicable diseases; SWAps/health sector support; other infectious diseases; and other. For most data sources, project-level data were available only through 2017. Methods to estimate health focus area allocations for 2018 are described in more detail below. Keyword searches were performed for a subset of global health channels that provide project-level data with project titles or descriptions. These sources include the bilateral development assistance agencies from 24 DAC member countries, one DAC participant country, GFATM, the World Bank, Asian Development Bank, African Development Bank, Inter-American Development Bank, Bill & Melinda Gates Foundations, Non-government Organizations, US foundations, Wellcome Trust and the European Economic Area. The keywords used are outlined in eTable 5 below. Descriptive fields were adjusted so that they were all in capitalized letters, special characters were modified, and search terms with multiple words were put between quotation marks. All keywords were translated into nine major languages (English, Spanish, French, Portuguese, Italian, Dutch, German, Norwegian, and Swedish) used in the OECD CRS, checked for double meanings across all languages, and adjusted accordingly.

Total DAH was split across the health focus areas using weighted averages based on the number of keywords present in each project's descriptive variables. If, for example, three keywords suggested the project focused on HIV/AIDS and two keywords related to tuberculosis were also tagged, three-fifths of the project's total DAH was allocated to HIV/AIDS and two-fifths was allocated to tuberculosis. To account for the sensitivity of this method, several checks were implemented after the keyword searches to ensure the project was accurately categorized. First, projects that were tagged as child and newborn vaccines and other infectious diseases were categorized as child and newborn vaccines only. Second, projects that were tagged as one of the three major infectious diseases (HIV/AIDS,

tuberculosis, or malaria) and other infectious diseases were categorized under only HIV/AIDS, tuberculosis, or malaria.

**Box 2. EXAMPLE. Post-keyword search weighting**
**A project in the CRS database had a value of $1,000 of DAH. A keyword search conducted on this project's title and description tagged five keywords: 3 keywords related to HIV/AIDs and 2 keywords related to tuberculosis. Therefore, $600, or 3/5 of total DAH, was allocated to HIV/AIDS, while $400, or 2/5 of total DAH, was allocated to tuberculosis.**

In addition to keyword searches, funds were allocated to health focus areas based on characteristics of the channel or additional channel variables. For the bilateral agencies and the EC, purpose codes from the CRS were used to supplement keyword searches. For the World Bank-IDA and -IBRD, health focus areas were also determined by the project sector codes and theme codes, which included percentages of health funds that targeted each theme. All funds from Gavi were allocated to child and newborn vaccines, health system strengthening and non-communicable diseases and all funds from UNICEF to maternal, newborn, and child health, unspecified. Funds from GFATM were distributed to malaria, HIV/AIDS, TB, and health sector support based on disease components. Within each disease component, keyword searches on programmatic budget data and project descriptions were conducted to distribute among program areas. Funds from UNAIDS were allocated to HIV/AIDS, and specific program areas were determined by budget information. UNFPA, PAHO, and WHO funds were allocated to specific health focus areas based on project expenditure data from their annual reports and annual financial reports. For all channels, projects listed as HIV/TB were distributed evenly among the two health focus categories. See eTable 6 below for more details on these categorizations.

eTable 5. Terms for keyword searches

**Health focus area level**	**Program area**	**Keywords**
**HIV/AIDS**	HIV envelope/other	" HUMANIMMUNODEFVIRUS " " SIDA " " OVC " " H I V " " HIV " " AIDS " " HUMAN IMMUNODEFICIENCY " " REVERSE TRANSCRIPTASE INHIBITOR " " ACQUIRED IMMUNE DEFICIENCY SYNDROME " " ACQUIRED IMMUNNODEFICIENCY " "RETROVIRAL " " VCT " " MALE CIRCUMCISION" " ART " " ARV " " CD4 COUNT " " HAART " " PMTCT " " MOTHER TO CHILD TRANSMISSION" " MOTHER TO CHILD AIDS TRANSMISSION" " PARENT TO CHILD TRANSMISSION" " PRESIDENT S EMERGENCY PLAN FOR AIDS RELIEF " " PEPFAR " " THREE DISEASES FUND " " 3 DISEASES FUND " " EMTCT " " 90 90 90 " " MOTHERTOCHILD"
	Care and Support	" CAREANDSUPPORT " " CARE ACTIVIT" " PAIN RELIEF " " SYMPTOM RELIEF " " PSYCHO SOCIAL SUPPORT " " CHRONICALLY ILL " " CLINICAL MONITORING " " CARE AND SUPPORT " " PSYCHOLOGICAL SERVICE" " PSYCHOLOGICAL SUPPORT " " PSYCHOSOCIAL SUPPORT " " PSYCHOSOCIAL SERVICE" " MATERIAL SUPPORT " " HOME BASED CARE " " PALLIATIVE CARE "
	Counseling and Testing	" COUNSELING " " TESTING " " VCT " " COUNSELLING " " COUNSELINGANDTESTING " " DIAGNOS"
	Orphans and Vulnerable Children	" VULNERABLECHILD" " OVC " " ORPHAN" " VULNERABLE CHILD" " INFECTED CHILD"

Health focus area level	Program area	Keywords
	Prevention of mother-to-child transmission (PMTCT)	" MOTHERTOCHILD" " MOTHER TO CHILD" " PARENT TO CHILD" " PMTCT " " EMTCT " " OPTION B "
	Prevention	" CONDOM" " PREVENT" " HIV EDUCATION " " AIDS EDUCATION " " REDUCING THE TRANSMISSION OF HIV " " REDUCE THE TRANSMISSION OF HIV " " MALE CIRCUMCISION" " SAFE BLOOD SUPPL" " SAFE INJECTION" " ABSTINENCE " " BLOOD SAFETY " " MICROBICIDE" " HARM REDUCTION " " PREP " " PEP " " PROPHYLAXIS " " ABCD " " BE FAITHFUL " " EARLY DETECTION "
	Treatment	"RETROVIRAL " " TREAT" " ART " " ARV " " CD4 COUNT " " HAART " " VIRAL LOAD " " VIRAL BURDEN " " VIRAL TITER " " DRUG REGIMEN" " FIRST LINE " " REVERSE TRANSCRIPTASE INHIBITOR "
	Antimicrobial resistance	" ANTIMICROBIAL RESISTAN" " ANTI MICROBIAL RESISTAN" " ANTIBIOTIC RESISTAN" " AMR " "DRUG RESISTAN" " MDR " " XDR " " RESISTANCE TESTING " " DRUG SUSCEPTIBILITY TESTING " " DST " " SECOND LINE "
**Tuberculosis**	Tuberculosis envelope/other	" TUBERCULOSIS " " TB " " TBC " " TUBERCULAR" " DOTS " " DIRECTLY OBSERVED TREATMENT " " RIFAMPICIN " " ISONIAZID " " THREE DISEASES FUND " " 3 DISEASES FUND " " RIFAMPIN "
	Treatment	" TREAT" " DOTS " " FIRST LINE " " DRUGS " " RIFAMPICIN " " RIFAMPIN " " ISONIAZID " " INH " " PYRAZINAMIDE " " PZA " " ETHAMBUTOL " " EMB " " STREPTOMYCIN " " SM " " STM " " PATIENT KIT " " INJECTABLE AGENT" " FLUOROQUINOLONES " " REGIMEN" " CASE MANAGEMENT " " ANTIMICROBIAL THERAPY " " DRUG SUSCEPTIBLE " " DRUG SENSITIVE "
	Diagnosis	" MICROSCOPY " " SPUTUM " " SMEAR " " CULTURE " " BACTERIOLOGICALLY CONFIRMED " " LABORATORY CONFIRMED " " GENEXPERT " " XPERT MTB RIF " " CHEST X RAY" " DIAGNOS" " LINE PROBE ASSAY "
	Antimicrobial resistance	" ANTIMICROBIAL RESISTAN" " ANTI MICROBIAL RESISTAN" " ANTIBIOTIC RESISTAN" " AMR " "DRUG RESISTAN" " MDR " " XDR " " RESISTANCE TESTING " " DRUG SUSCEPTIBILITY TESTING " " DST " " SECOND LINE "
**Malaria**	Malaria envelope/other	" MALARIA " " FALCIPARUM " " ANOPHELES " " ARTEMISININ " " PRIMAQUINE " " INDOOR RESIDUAL SPRAY" " INDOORRESIDUALSPRAY" " IRS " "VIVAX " " BEDNET" " BED NET" " SMITN " " ITN " " LLIN " " INSECTICIDAL NET" " INSECTICIDE TREATED NET" " THREE DISEASES FUND " " 3 DISEASES FUND " " CHLOROQUINE " " ANTI MALARIAL " " ANTIMALARIAL "
	Diagnosis	" DIAGNOS" " CASE DETECTION " " MICROSCOPY " " BLOOD SURVEY" " BIOLOGICAL TESTING " " EDT " " LAMP " " RDT "
	Community outreach	" COMMUNITYOUTREACH " " OUTREACH " " COMMUNITY MOBILIZATION" " AWARE" " COMMUNICATION STRATEGY " " SOCIAL COMMUNICATION " " PARTNERSHIP" " ACTIVITIES NEAR COMMUNITIES " " BCC " " BEHAVIORAL CHANGE COMMUNICATION " " BEHAVIOURAL CHANGE COMMUNICATION " " BEHAVIOR CHANGE

Health focus area level	Program area	Keywords
		COMMUNICATION " " BEHAVIOUR CHANGE COMMUNICATION " " SOCIAL MOBILIZATION "
	Vector control: bednets	" BEDNET" " BED NET" " SMITN " " ITN " " LLIN " " INSECTICIDAL NET" " INSECTICIDE TREAT"
	Vector control: irs	" INDOORRESIDUALSPRAY" " IRS " " REDUCE THE PARASITE RESERVOIR " " FOGGING " " COILS " " LARVICID" " LARVACID" " VECTOR CONTROL" "RESIDUAL SPRAY" " RESIDUALSSPRAY " "INDOOR SPRAY" " INDOORSPRAY "
	Vector control: other than bednets and irs	" PREVENT" " IPT " " SMC " " SEASONAL MALARIA CHEMOPREVENTION "
	Treatment	" ARTEMISININ " " PRIMAQUINE " " ACT " " DRUG" " TREAT" " CASE MANAGEMENT " " COMBINATION THERAPY " " ANTI MALARIAL " " ANTIMALARIAL " " CHLOROQUINE "
	Antimicrobial resistance	" ANTIMICROBIAL RESISTAN" " ANTI MICROBIAL RESISTAN" " ANTIBIOTIC RESISTAN" " AMR " "DRUG RESISTAN" " MDR " " XDR " " RESISTANCE TESTING " " DRUG SUSCEPTIBILITY TESTING " " DST " " SECOND LINE "
**Reproductive and maternal health**	envelope/other	" FERTILITY " " FAMILY PLANNING " " FP " " BIRTH" " WOMEN HEALTH " " WOMEN S HEALTH " " WOMENS HEALTH " " CONTRACEP" " IPPF " " PLANNED PARENTHOOD " " ABORTION" " UNFPA " " POSTPARTUM " " POST PARTUM " " MATERNAL " " MATERNITY " " MOTHER" " SBA " " ANTENATAL " " PRENATAL " " PERINATAL " " POSTNATAL " " FETUS" " FETAL" " IPTP " " REPRODUCTIVE HEALTH " " OBSTETRIC" " PREGNANC" " RH " " REPROD " " RHCS " " SEXUAL HEALTH " " SYPHILIS " " FISTULA " " ANEMI" " ANAEMI" " FOETUS" " FOETAL " " FGM " " FEMALE GENITAL MUTILATION " " FEMALE GENITAL CUTTING " " FEMALE CIRCUMCISION " " SBAS " " OBSTRUCTED LABOR " " OBSTRUCTED LABOUR " " MNCH" " RNCH " " RCH " " RNH " " MNH " " MCH " " EMAS " " MCNH " " PMNCH " "ECLAMPSIA " " PRETERM " " ANC " " WCAH " " TBA " " TBAS " " BIRTH ATTENDANT" " CESAREAN" " CAESAREAN" " C SECTION" " STI " " STD " " SEXUALLY TRANSMITTED "
	Family planning	" FERTILITY " " FAMILY PLANNING " " FP " " BIRTH SPACING " " CONTRACEPT" " FAMILY SIZE" " IPPF " " PLANNED PARENTHOOD " " ABORTION" " BIRTH CONTROL " " CONDOM" " IUD " " VASECTOMY " " TUBULAR LIGATION"
	Maternal health	" POSTPARTUM " " POST PARTUM " " MATERNAL HEALTH " " MATERNAL MORTALITY " " MATERNAL DEATH" " SAFE MOTHERHOOD " " BIRTH ATTENDANT" " SBA " " ANTENATAL " " PRENATAL " " PERINATAL " " POSTNATAL " " FETUS" " FETAL" " IPTP " " MATERNITY " " OBSTETRIC" " PREGNANC" " FISTULA " " SEPSIS " " SEPTICEMIA " " ANEMI" " ANAEMI" " FOETUS" " FOETAL " " SBAS " " OBSTRUCTED LABOR " " OBSTRUCTURED LABOUR " " DELIVERY ROOM" " CHILD DELIVERY " " MIDWIV" " MIDWIFE" "ECLAMPSIA " " PRETERM " " ANC " " TBA " " TBAS " " CESAREAN" " CAESAREAN" " C SECTION" " MNH " " MCH " " MNCH " " MCNH "

Health focus area level	Program area	Keywords
**Newborn and child health**	envelope/other	" NEONATAL " " PERINATAL " " POSTNATAL " " MALNUTRITION " " VITAMIN A " " BREAST FE" " BREASTFE" " MICRONUTRIENT" " FORTIFICATION " " STUNT" " WASTING " " BABY FRIENDLY HOSPITAL INITIATIVE " " BREASTMILK " " BREAST MILK " " IODINE " " IODIZED " " IODIZATION " " VAD " " LACTAT" " FOLIC ACID " " FOLAT" " VACCIN" " IMMUNIZ" " POLIO " " DIPHTHERIA " " TETANUS " " PERTUSSIS " " DTP " " HIB " " ROTAVIRUS " " MEASLES " " IMMUNIS" " HEPB " " INJECTION SAFETY " " RUBELLA " " MENINGITIS " " PENTA " " PENTAVALENT " " PNEUMONIA " " PNEUMOCOCC" " HAEMOPHILUS INFLUENZAE " " TETRA " " GAVI " " CHILDHEALTH " " CHILD HEALTH " " CHILDREN " " INFANT" " NEWBORN" " CHILD MORTALITY " " UNDER FIVE MORTALITY " " CHILD SURVIVAL " " CHILDHOOD ILLNESS" " LRI " " RESPIRATORY INFECTION" " DIARRHEA" " DIARRHOEA" " ORAL REHYDRATION " " ORT " " ORS " " UNICEF " " MNCH" " RNCH " " RCH " " RNH " " MNH " " MCH " " EMAS " " MCNH " " POLIOVIRUS "
	Child/newborn nutrition	" NUTRITION" " MALNUTRITION " " BIRTH WEIGHT " " BIRTHWEIGHT " " VITAMIN A " " BREAST FE" " BREASTFE" " FEEDING " " MICRONUTRIENT" " ZINC " " FORTIFICATION " " STUNT" " WASTING " " UNDERWEIGHT " " BABY FRIENDLY HOSPITAL INITIATIVE " " BREASTMILK " " BREAST MILK " " IODINE " " IODIZED " " IODIZATION " " VAD " " LACTAT" " FOLIC ACID " " FOLAT" " IRON " " DEWORMING "
	Child/newborn vaccines	" POLIO " " VACCIN" " IMMUNIZ" " DIPHTHERIA " " TETANUS " " PERTUSSIS " " DTP " " HIB " " ROTAVIRUS " " MEASLES " " IMMUNIS" " HEPB " " INJECTION SAFETY " " RUBELLA " " MENINGITIS " " PENTA " " PENTAVALENT " " PNEUMONIA " " PNEUMOCOCC" " HAEMOPHILUS INFLUENZAE " " TETRA " " GAVI " " POLIOVIRUS " " DPT " " PCV "
**Non-communicable diseases**	envelope/other	" TOBACCO" " SMOK" "CIGAR" " FCTC " " TFI " " SCHIZOPHRENIA " " MENTAL HEALTH " " NEUROTIC " " NEUROSIS " " NEUROSES " " NEUROLOGICAL" " PSYCHOLOG" " PSYCHIATR" " EMOTIONAL DISORDER" " OBSESSIVE COMPULSIVE " " OCD " " PTSD " " POST TRAUMATIC " " POSTTRAUMATIC " " ALCOHOL DEPENDEN" " ALCOHOL ABUSE " " ADDICTION " " BEHAVIORAL DISORDER" " DRUG ABUSE " " SUBSTANCE ABUSE " " OPIOID " " COCAINE " " AMPHETAMIN" " DEPRESSIVE DISORDER" " DEPRESSION " " DYSTHYMIA " " BIPOLAR " " ANXIETY " " EATING DISORDER " " PHOBIA " " DRUG DEPENDEN" " ATTENTION DEFICIT HYPERACTIVITY DISORDER " " ADHD " " PANIC DISORDER" " SELF HARM " " STRESS DISORDER" " SUBSTANCE USE DISORDER" " DRUG USE DISORDER" " MENTAL ILLNESS" " MENTAL DISORDER" " PSYCHOSOCIAL " " PSYCHO SOCIAL " " HEROIN " " OXYCODONE " " ATTENTION DEFICIT DISORDER " " SUICIDE PREVENTION" " HEADACHE " " ANOREXIA " " BULIMIA " " HYPERKINETIC DISORDER" " PERSONALITY DISORDER" " FETAL ALCOHOL SYNDROME" " PSYCHOSIS " " DOWN SYNDROME " " DOWN S SYNDROME " " DOWNS SYNDROME " " AUTISM " " ASPERGER " " DEVELOPMENTAL

Health focus area level	Program area	Keywords
		DISORDER" " CONDUCT DISORDER" " INTELLECTUAL DISABILIT" " MENTAL DISAB" " MENTAL RETARDATION " " ALZHEIMER" " DEMENTIA" " EPILEPSY " " MIGRAINE " " PARKINSON" " MENTALLY DISAB" " NERVOUS SYSTEM" " SYNAPSE" " NON COMMUNICABLE " " NONCOMMUNICABLE " " CANCER" " CHEMOTHERAPY " " RADIATION " " NEOPLAS" " TUMOR " " LEUKEMIA " " LYMPHOMA " " MYELOMA " " HPV " " HUMAN PAPILLOMA VIRUS " " HEP C " " HEPATITIS C " " DIABET" " INSULIN " " ENDOCRINE " " RHEUMAT" " ISCHAEMIC " " ISCHEMIC " " CIRCULATORY " " CIRRHOSIS " " DIGESTIVE DISEASE" " OTHER DIGESTIVE " " PEPTIC " " APPENDICITIS " " GASTRITIS " " GENITOURINARY " " UROGENITAL " " MUSCULOSKELETAL " " GOUT " " BACK PAIN " " MACULAR " " HEARING " " AUDIOLOG" " PERIODONTAL " " CARIES " " CONGENITAL " " OBESITY " " OVERWEIGHT " " GLAUCOMA " " HYPERTENSI" " HERNIA " "ARTHRITIS " " CLEFT LIP" " CLEFT PALATE" " PHENYLKETONURIA " " SICKLE CELL" " DREPANOCYTOSIS " " HEMOPHILIA " " HAEMOPHILIA " " THALASSEMIA " " GENETIC DISORDER" " HEART DISEASE" " CHRONIC RESPIRATORY " " COPD " " STROKE " " CATARACT" " CHRONIC OBSTRUCTIVE PULMONARY DISEASE" " ASTHMA " " SKIN DISEASE" " DERMATITIS " " PSORIASIS " " SCABIES " " PHYSICAL DISAB" " DENTAL " " ORAL HEALTH " " CVD " " IHD " " CKD " " KIDNEY DISEASE" " MSK " " EYE " " CEREBROVASCULAR " " VASCULAR " " BLOOD PRESSURE " " ACUTE GLOMERULONEPHRITIS " " ALOPECIA AREATA " " ANEURYSM " " ANGINA " " ARTERY " " ATHEROSCLEROSIS " " ATRIAL FIBRILLATION" " ATRIAL FLUTTER " " BENIGN PROSTATIC HYPERPLASIA " "BLASTOMA" " BLIND " " PREVENTABLE BLINDNESS " " AVOIDABLE BLINDNESS " " BLOOD DISORDER" " BRONCHITI" " CARCINOMA " " CARDIAC " " CARDIO" " CELLULITIS " " CEREBRAL " " CORONARY " " DEAF" " DECUBITUS ULCER " "DIALYSIS" " DUODENITIS " " ECZEMA " " EKZEMA " " EDENTULISM " " ENDOCARDITIS " " FIBROSIS " " G6PD DEFICIENCY " " GALL BLADDER " " BILE DUCT " " GLYCEMI" " GLYCAEMI" " HEMOGLOBINOPATH" " HEMOLYTIC ANEMIA " " HODGKIN" " INSOMNIA " " INTERSTITIAL LUNG DISEASE" " INTESTINAL OBSTRUCTION" " LEUKAEMIA " " MELANOMA" " MULTIPLE SCLEROSIS " " MYOCARD" " NCD " " NECK PAIN " " NEPHRITIS " " NEPHROSIS " " NEURAL TUBE DEFECT" " NEURODEGENERATIVE " " INFLAMMATORY BOWEL" " ONCOLOG" " OPTICAL " " OSTEOMYELITIS " " OTITIS MEDIA " " PANCREATITIS " " PARALYTIC ILEUS " " PERITONEAL " " PNEUMOCONIOSIS " " PROSTATE " " PRURITUS " " SARCOIDOSIS " " PYELONEPHRITIS " " REFRACTIVE ERROR" " RENAL " " RETINA " " SARCOMA " " SUBCUTANEOUS DISEASE" " URINARY DISEASE" " URINARY TRACT INFECTION" " UROLITHIASIS " " URTICARIA " " VENTRICULAR " " VISION LOSS " " ACCOMODATION DISORDER" " SENSE ORGAN " " GUILLAIN BARRE SYNDROME" " IMPETIGO " " LOSE WEIGHT " " BIRTH

Health focus area level	Program area	Keywords
		DEFECT" "PAPILLOMAVIRUS" " GENE DEFECT" " PHYSICALLY DISAB" " TUMOUR" " BRAIN INJUR" " MAMMOGRA" " ANTITUMOR " " ANTITUMOUR " " BARIATRIC" " FATTY LIVER" " IMMUNOTHERAPY " " CHROMOSOMAL ABERRATION" " PERIODONTITIS " " OSTEOPOROSIS " "MALIGNANC" " NEURON"
	Mental health	" SCHIZOPHRENIA " " MENTAL HEALTH " " NEUROTIC " " NEUROSIS " " NEUROSES " " NEUROLOGICAL" " PSYCHOLOG" " PSYCHIATR" " EMOTIONAL DISORDER" " OBSESSIVE COMPULSIVE " " OCD " " PTSD " " POST TRAUMATIC " " POSTTRAUMATIC " " ALCOHOL DEPENDEN" " ALCOHOL ABUSE " " ADDICTION " " BEHAVIORAL DISORDER" " DRUG ABUSE " " SUBSTANCE ABUSE " " OPIOID " " COCAINE " " AMPHETAMIN" " DEPRESSIVE DISORDER" " DEPRESSION " " DYSTHYMIA " " BIPOLAR " " ANXIETY " " EATING DISORDER " " PHOBIA " " DRUG DEPENDEN" " ATTENTION DEFICIT HYPERACTIVITY DISORDER " " ADHD " " PANIC DISORDER" " SELF HARM " " STRESS DISORDER" " SUBSTANCE USE DISORDER" " DRUG USE DISORDER" " MENTAL ILLNESS" " MENTAL DISORDER" " PSYCHOSOCIAL " " PSYCHO SOCIAL " " HEROIN " " OXYCODONE " " ATTENTION DEFICIT DISORDER " " SUICIDE PREVENTION" " HEADACHE " " ANOREXIA " " BULIMIA " " HYPERKINETIC DISORDER" " PERSONALITY DISORDER" " FETAL ALCOHOL SYNDROME" " PSYCHOSIS " " DOWN SYNDROME " " DOWN S SYNDROME " " DOWNS SYNDROME " " AUTISM " " ASPERGER " " DEVELOPMENTAL DISORDER" " CONDUCT DISORDER" " INTELLECTUAL DISABILIT" " MENTAL DISAB" " MENTAL RETARDATION " " ALZHEIMER" " DEMENTIA" " EPILEPSY " " MIGRAINE " " PARKINSON" " MENTALLY DISAB" " NERVOUS SYSTEM" " SYNAPSE"
	Tobacco	" TOBACCO" " SMOK" "CIGAR" " FCTC " " TFI "
**SWAps/ Health sector support**	envelope/other	" SWAP" " TRAINING " " CAPACIT" " DATA SYSTEM" " SECTOR WIDE APPROACH" " HEALTH SYSTEM" " SECTOR PROGRAM" " BUDGET SUPPORT" " SECTOR SUPPORT " " HSS " " TRACKING PROGRESS " " SKILLED WORKER" " HEALTH WORKER" " SKILLED STAFF " " HEALTH PROFESSIONAL" " FACILITIES " " ESSENTIAL MEDICINES " " POLICY DEVELOPMENT" " MEDICAL EQUIPMENT" " SURGICAL EQUIPMENT" " HOSPITAL EQUIPMENT" " HOSPITAL EQMT " " INSTITUTIONAL STRENGTHENING " " HSPSP " " M&E " " M & E " " MONITORING " " SURVEILLANCE " " GOVERNANCE " " HUMAN RESOURCE" " HUMAN CAPITAL " " SCALING UP " " REALLOCATE RESOURCES " " STRATEGIES AND PROGRAM" " HIV STRATEG" " PROGRAM IN COUNTRY ACTIVITIES " " STRATEGIC INFORMATION " " PROCUREMENT " "

Health focus area level	Program area	Keywords
		EVIDENCE BASED " " CASE REPORTING " " MEDICAL WORKER" " HEALTH CARE PERSONNEL " " OPERATIONAL RESEARCH " " SUPPORTIVE ENVIRONMENT " " INFORMATION SYSTEM" " WORKFORCE " " INFRASTRUCTUR" " MEDICAL EDUCATION " " CASE NOTIFICATION " " CASE FINDING " " LABORATORY STRENGTHENING " " LABORATORY QUALITY " " LABORATORY NETWORK" " CONTROL SERVICES " " INFECTION CONTROL " " CONTROL PROGRAM" " SCALE UP" " STOP TB STRATEGY " " HEALTH EDUCATION " " CONTINUING EDUCATION " " SUPPLY " " HEALTH MANAGEMENT" " HEALTH POLICY " " MANAGEMENT AND COORDINATION " " ADMINISTRATIVE MANAGEMENT " " MANAGEMENT AND ADMINISTRATION " " COLD CHAIN" " HEALTH PROMOTION " " TECHNICAL ASSISTANCE " " DSS " " DISTRIBUTION SYSTEMS " " SERVICE DELIVERY " " BUILDINGS " " HEALTH FACILIT" " CONSTRUCT" " MEDICAL SCHOOL" "CENTERS OF EXCELLENCE" "NURSE" "DOCTOR" "PHYSICIAN" "MEDICAL LABORATORY SCIENTIST" "SURGEON" "SPECIALIST" "PHARMACIST" "HEALTH LABOR" "LABOR MARKET" "PERSONNEL" "MEDICAL PRACTIONER" "DENTAL PRACTIONER" "TASK SHIFTING"
	Human resources	" INFRASTRUCTUR" " MEDICAL EQUIPMENT" " SURGICAL EQUIPMENT" " HOSPITAL EQUIPMENT" " HOSPITAL EQMT " " BUILDINGS " " HEALTH FACILIT" " CONSTRUCT" " MEDICAL SCHOOL" "CENTERS OF EXCELLENCE" " TRAINING " " CAPACIT" " SKILLED WORKER" " HEALTH WORKER" " SKILLED STAFF " " HEALTH PROFESSIONAL" " HUMAN RESOURCE" " HUMAN CAPITAL " " MEDICAL WORKER" " WORKFORCE " " MEDICAL EDUCATION " " HEALTH EDUCATION " " CONTINUING EDUCATION " " HEALTH MANAGEMENT" " MANAGEMENT AND COORDINATION " " ADMINISTRATIVE MANAGEMENT " " MANAGEMENT AND ADMINISTRATION " "NURSE" "DOCTOR" "PHYSICIAN" "MIDWIFE" "MIDWIVES" "MEDICAL LABORATORY SCIENTIST" "SURGEON" "SPECIALIST" "PHARMACIST" "HEALTH LABOR" "LABOR MARKET" "PERSONNEL" "MEDICAL PRACTIONER" "DENTAL PRACTIONER" "TASK SHIFTING" " TECHNICAL ASSISTANCE "
	Pandemic preparedness	" PANDEMIC PREPAREDNESS " " PANDEMIC RESPONSE" " PANDEMIC ALERT" " EPIDEMIC ALERT" " EPIDEMIC RESPONSE" " EPIDEMIC PREPAREDNESS " " OUTBREAK RESPONSE" " OUTBREAK ALERT" " OUTBREAK PREPAREDNESS " " PANDEMIC INFLUENZA " " EPIDEMIOLOGICAL INVESTIGATION" " CONTACT MANAGEMENT " " PREPAREDNESS AND RESPONSE PLAN" " PREPAREDNESS & RESPONSE PLAN" " BIOSAFETY MEASURE" "EARLY WARNING " " HEALTH SECURITY PREPAREDNESS " " HEALTH SECURITY RISK ASSESSMENT " " RAPID RESPONSE STRATEG"

Health focus area level	Program area	Keywords
**Other infectious diseases**	envelope/other	" INFECTIOUS " " COMMUNICABLE " " TRICHURIASIS " " YELLOW FEVER " " WHIPWORM " " TRACHOMA " " SCHISTOSOMIASIS " " BILHARZIA " " SNAIL FEVER " " KAYAYAMA FEVER " " RABIES " " ONCHOCERCIASIS " " RIVER BLINDNESS " " ROBLES DISEASE" " LYMPHATIC FILARIASIS " " ELEPHANTIASIS " " LEISHMANIASIS " " LEISHMANIOSIS " " HOOKWORM " " FOOD BORNE " " FOODBORNE " " ECHINOCOCCOSIS " " HYDATID DISEASE" " HYDATIDOSIS " " DENGUE " " CYSTICERCOSIS " " CHAGAS " " TRYPANOSOMIASIS " " SLEEPING SICKNESS " " ASCARIASIS " " TROPICAL DISEASE" " CHOLERA " " DYSENTERY " " PARASITE DISEASE" " FAO " " NEGLECTED TROPICAL DISEASE" "TYPHOID" " LEPROSY " " BURULI ULCER " " EBOLA" " EBOV " " EVD " " ZIKA " " ZIKV " " GUINEA WORM " " DRACUNCULIASIS " " FILARIASIS " " HEPATITIS E" " ENCEPHALITIS " " VARICELLA" " INFLUENZA" " FLU " " NTD " " HEPATITIS A " " HEPATITIS D " " HEP A " " HEP D " " ROUNDWORM" " RINGWORM" " TAPEWORM" " FLATWORM" " CHIKUNGUNYA " " LASSA " " MERS " " NIPAH " " MARBURG " " SARS "
	Antimicrobial resistance	" ANTIMICROBIAL RESISTAN" " ANTI MICROBIAL RESISTAN" " ANTIBIOTIC RESISTAN" " AMR " "DRUG RESISTAN" " MDR " " XDR " " RESISTANCE TESTING " " DRUG SUSCEPTIBILITY TESTING " " DST " " SECOND LINE "

eTable 6. Additional health focus area categorizations

Channel	Allocation criteria	Health focus area
**Bilaterals and the EC**	CRS purpose code 13030, family planning	Family planning
	CRS purpose code 13020, reproductive health care	Maternal health, non-family planning
	CRS purpose code 12240, basic nutrition	Child and newborn nutrition
	CRS purpose code 12250, infectious disease control and the keywords “child” or “vaccine” present in descriptive variables	Child and newborn vaccines
	CRS purpose code 13040, STD control including HIV/AIDS	HIV/AIDS
	CRS purpose code 12262, malaria control	Malaria, unspecified
	CRS purpose code 12250, infectious disease control and no other keywords present in the descriptive variables	Other infectious diseases
	CRS purpose code 12263, tuberculosis control	Tuberculosis
	CRS purpose code 12230, basic health infrastructure	SWAPs/health system strengthening - other
	CRS purpose code 12281, health personnel development	SWAPs/health system strengthening - Human resources
	CRS purpose code 12281, medical education/training	SWAPs/health system strengthening - Human resources
**World Bank IDA and IBRD**	Theme code population and reproductive health	Reproductive and maternal health, other

Channel	Allocation criteria	Health focus area
	Theme code tuberculosis	Tuberculosis, other
	Theme code child health	Newborn and child health, other
	Theme code HIV/AIDS	HIV/AIDS, other
	Theme code malaria	Malaria, other
	Theme code injuries and non-communicable diseases	Non-communicable diseases, other
	Theme code nutrition and food security	Newborn and child health, nutrition
	Theme code other communicable diseases	Other infectious diseases, other
	Theme code health system performance	SWAPs/health systems strengthening, other
	Theme code social analysis and monitoring	SWAPs/health systems strengthening, other
**UNFPA**	Family planning, population and development strategies, population and development, population dynamics	Family planning
	Reproductive health, maternal and newborn health, young people's SRH and sexuality education, HIV and STI prevention services, sexual and reproductive health, sexuality education	Maternal health
	Gender equality and women's empowerment, gender equality and reproductive rights, program coordination and assistance, adolescents and youth, civil society and rights for all, ending harmful practices, marginalized girls, protection rights, other	Family planning and Maternal health, unspecified, according to proportions between the two.
	HIV and STI prevention services, HIV and AIDS	HIV prevention
**UNICEF**	All DAH	HIV prevention
		Maternal, newborn, and child health (vaccines, maternal health, and health system strengthening)
**UNAIDS**	The keyword search was run on budget information for years 2008-2017<br>Program components in budget documents from 1998 to 2007	All program areas under HIV/AIDS and TB
**UNITAID**	Assigned in database received through correspondence	HIV/AIDS (treatment, counseling and testing, prevention) , TB (treatment and diagnosis), Malaria (diagnosis and treatment), HSS
**GAVI**	Vaccine DAH for HPV vaccine<br>All other vaccine DAH<br>HSS DAH	NCD other<br>Child and newborn vaccines<br>Maternal and child health HSS
**GLOBAL FUND**	Disease components for Malaria, HIV/AIDS, TB, TB/HIV, and Other (health systems strengthening)<br>Keyword search on program service delivery areas	All program areas under Malaria, TB, HIV and Swap/HSS
**WHO**	Reproductive, maternal, newborn, child, and adolescent health (divided by 2); Research in human reproduction	Maternal health, unspecified
	Nutrition	Child and newborn nutrition
	Vaccine-preventable diseases	Child and newborn vaccines

Channel	Allocation criteria	Health focus area
	Reproductive, maternal, newborn, child and adolescent health (divided by 2)	Child and newborn health, unspecified
	Aging and health; gender, equity and human rights mainstreaming	Maternal, newborn, and child health, unspecified
	HIV/AIDS	HIV/AIDS
	Malaria	Malaria
	Tuberculosis	Tuberculosis
	Mental health and substance abuse	Non-communicable diseases, mental health
	Disabilities and rehabilitation; Non-communicable diseases; Violence and injuries	Non-communicable diseases, unspecified
	Neglected tropical diseases; Tropical disease research; Infectious hazard management; Outbreak and crisis response (50%); Alert and response capacities (50%)	Other infectious diseases
	Health system information and evidence; Integrated people-centered health services; National health policies, strategies and plans; Access to medicines and health technologies and strengthening regulatory capacity; health emergency information and risk assessment (50%)	SWAps/health system strengthening
	Country health emergency preparedness and the International Health Regulations; health emergency information and risk assessment(50%); Emergency operations; Emergency core services; Outbreak and crisis response(50%); Epidemic- and pandemic-prone diseases; Alert and response capacities(50%)	SWAps/health system strengthening, pandemic preparedness
	Social determinants for health; Health and the environment; Food safety; Antimicrobial resistance	Other
**PAHO**	HIV/AIDS and STls; HIV/AIDS, TB and malaria (33%)	HIV/AIDS, unspecified
	Tuberculosis; HIV/AIDS, TB and malaria (33%)	Tuberculosis, unspecified
	HIV/AIDS, TB and malaria (33%); Malaria and other Vector-Borne Diseases (50%);	Malaria, unspecified
	Communciable diseases; Malaria and other Vector-Borne Diseases (50%); Neglected Tropical and zoonotic diseases	Other infectious diseases
	Nutrition; Food Safety	Child and newborn nutrition
	Vaccíne-Preventable Diseases	Child and newborn vaccines
	Women, maternal, newborn, child, and adolescent and adult health	Maternal and child health, unspecified
	Mental Health and psychoactive substance use disorders	Non-communicable diseases, mental health
	Non Communicable Diseases and Risk Factors; Chronic noncommunicable diseases	Non-communicable diseases, unspecified
	Health systems leadership and governance; Human resources for health; Social protection and financing; Health systems information and evidence; Health services; People-centered integrated health services; Access to medical	Swap/health system strengthening

Channel	Allocation criteria	Health focus area
	products and strengthening regulatory capacity; Health governance and financing, national health policies, strategies and plans	
	Violence and Injuries; Disabilities and Rehabilitation; Antimicrobial resistance; Aging and health; Gender, equity, human rights and ethnicity; Social determinants of health; Health and the environment; Strategic communications; Management and administration; Flexible and learning organization	Other

Disaggregating preliminary estimates by health focus area

Estimates by health focus area for years in which descriptive data were not available (usually 2018) were obtained by modeling channel-specific DAH per health focus area as a function of time. Out-of-sample validation was used to test the predictive accuracy of a large suite of models, estimating the models using 1990-2010 data and predicting 2011 and 2012. The potential models included fractional multinomial logit regression, OLS regression, autoregressive integrated moving average (ARIMA) models, epanechnikov kernel-weighted local polynomial smoothing, and multivariable fractional polynomial models. For each model, time was modeled linearly, with splines, and by including lag-dependent variables. Other methodologies considered included modeling health-focus-area-specific DAH as a dollar amount and as a fraction of the channel-specific total DAH. Lastly, models that involved transforming the dependent variable in natural log and logit transformed space were considered. In order to accommodate zero values in the logit transformation, the transformation described in Smithson and Verkuilen were applied.[73] Over 40 models and specifications were evaluated in total.

Each of the potential model and specification described above were estimated using data from 1990 through 2010, and then the estimated model was used to predict DAH by health focus area for 2011 and 2012. Since we have DAH estimates for 2011 and 2012, we compared the modeled estimates and the observed estimates and calculated average percent deviation and average total absolute deviation for each model and specification across all the channels and health focus areas. A variant of the Epanechnikov kernel-weighted local polynomial smoothing had the smallest average percent deviations and average total absolute error. In this model and specification, health focus area-specific DAH fractions were independently estimated at the channel level after they were logit transformed. Time was the only independent variable included in the model. The health focus area-specific DAH estimates were adjusted so the sum of the channel's health focus area disbursements totaled channel-specific DAH envelope. Our preferred model, the Epanechnikov kernel-weighted local polynomial smoothing, minimized both the average percent deviation and the total absolute error out of sample, predicting two years ahead. See Dieleman et al. for a table that demonstrates the performance of four models, each with their optimal specification (as determined by the out-of-sample average percent deviation and total absolute error).[1]

Tracking development assistance for health from bilateral aid agencies and the European Commission

OECD-DAC maintains two databases on aid flows: 1) the DAC annual aggregates database, which provides summaries of the total volume of flows from different donor countries and institutions, and 2) the CRS, which contains project- or activity-level data.[3] This year, we used the DAC databases to track health ODA from 24 OECD-DAC members (Austria, Australia, Belgium, Canada, Denmark, Finland, France, Germany, Greece, Ireland, Italy, Japan, Luxembourg, the Netherlands, New Zealand, Norway, Portugal, South Korea, Spain, Sweden, Switzerland, the United Kingdom, the United States, and the EC), and one DAC Participant country, United Arab Emirates, for the years 1990 to 2018. Observed data for the DAC members was available from 1990 to 2017, and observed data for the United Arab Emirates was available from 2009 to 2017. United Arab Emirates bilateral health ODA from 1990 to 2008 was obtained through personal correspondence.

These two DAC databases track the following types of resource flows:

Official development assistance (ODA), defined as “flows of official financing administered with the promotion of the economic development and welfare of developing countries as the main objective”69 is tracked from its 30 members (Austria, Australia, Belgium, Canada, Czech Republic, Denmark, Finland, France, Germany, Greece, Hungary, Iceland, Ireland, Italy, Japan, Luxembourg, the Netherlands, New Zealand, Norway, Poland, Portugal, Slovakia, Slovenia, South Korea, Spain, Sweden, Switzerland, the United Kingdom, the United States, and the EC). The CRS also now includes some private ODA, such as that funded by BMGF and the Global Fund to Fight AIDS, Tuberculosis and Malaria (GFATM), as well as assistance from a number of non-DAC countries such as the United Arab Emirates and Kuwait.

ODA includes:

• Bilateral ODA, which is given directly by DAC members as aid to recipient governments, core contributions to NGOs and public-private partnerships, and earmarked funding to international organizations.

• Multilateral ODA, which includes core contributions to multilateral agencies such as WHO, UNFPA, GFATM, Gavi, UNAIDS, UNICEF, PAHO, the World Bank, and other regional development banks. Only regular budgetary contributions to these institutions can be reported to the OECD-DAC; hence, extrabudgetary funds, including earmarked contributions that donors can report as bilateral ODA, are not included as multilateral ODA. Only 70% of core contributions to WHO can be counted as multilateral ODA.

a. Official development finance (ODF), which includes grants and loans made by multilateral agencies.

b. Other official flows (OOF), which refers to transactions that “do not meet the conditions for eligibility as Official Development Assistance or Official Aid, either because they are not primarily aimed at development, or because they have a Grant Element of less than 25 percent.”

The DAC aggregate tables include all multilateral development banks, GFATM, operational activities of UN agencies and funds, and a few other multilateral agencies. The project-level data in the CRS cover a smaller subset of multilateral institutions, including UNAIDS, UNFPA, UNICEF, public-private partnerships including Gavi and GFATM, some development banks, and BMGF, but do not reflect the core-funded operational activities of WHO prior to 2009, disbursements by Gavi prior to 2007 and BMGF prior to 2009, or all loans from the World Bank.

This research utilized the CRS as the principal source for tracking bilateral DAH. This is because the DAC aggregate tables do not report detailed project-level information about the recipient country and health focus area. The OECD sector codes for general health (121), basic health (122), and population programs (130) were used to identify health flows in the CRS. Only ODA related flows are used in our analysis, including OECD flow codes corresponding to ODA grants (11), ODA grant-like (12), ODA loans (13), and equity investment (19). In addition, we included select projects with OECD sector code for emergency aid (720) in order to capture additional funding for ebola and zika outbreaks. Projects with purpose code emergency food aid (72040) were excluded, and the remaining project descriptive variables were parsed for the keywords “EBOLA” and “ZIKA” to identify emergency aid projects targeting these diseases. Based on our definition of DAH, we also excluded any remaining projects containing any of the following keywords: " WATER " " SANITATION " " FOOD " " EARTHQUAKE " " HURRICANE " " TERREMOTO " " HURACAN " " FAMILY CARE STRUCTURES ".

To avoid double-counting, all identifiable earmarked commitments and disbursements between one disbursing agency to another were tagged and removed before calculating final DAH estimates. Income statements, expenditure statements, and keyword searches in the descriptive project fields were used to identify potential sources of double-counting.

In multilateral agencies, public-private partnerships, development banks, and foundations, when expenditure by recipient agency information was available, double counting was tagged and dropped in the disbursing agency from whom the resources was transferred. In the absence of detailed expenditure data in the disbursing agency that transferred resources ,the income statement of the receiving disbursing agency that reflected

the disbursement was used to flag the disbursement in the initial disbursing agency. Preliminary estimates were generated based on the annual disbursed envelope exclusive of resources flagged as transfers to other agencies.

Channel codes in the CRS data were used to track DAH to international and donor-country-based non-government organizations. The names of NGOs that were captured in IHME's NGO data (as detailed in the section titled "Tracking non-governmental organizations") were searched for in the CRS descriptive variables and tagged as double-counting. Research funds for HIV/AIDS channeled by the US government through the National Institutes for Health (NIH) were also removed from the total since they do not meet the definition of DAH as contributions from institutions whose primary purpose is development assistance. Official development finance (ODF) from the CRS was not counted because these expenditures were included elsewhere, either in the analysis of multilateral institutions relevant to the study or in the assessment of health spending by BMGF, the data for which were obtained via correspondence and from their annual reports, audited financial statements, and project databases. To avoid double-counting, only health assistance flows from multilateral institutions to low- and middle-income countries were counted, and not transfers to multilateral institutions. Also, for regional projects the disbursements are split amongst all countries in the specified OECD region. For example, a project allocated to recipient "North of Sahara, regional" would have its disbursements split equally between all the countries in the corresponding OECD region: Algeria, Egypt, Libya, Morocco, and Tunisia.

Allocation of funding to health focus areas was assigned as described in the section "DISAGGREGATING BY HEALTH FOCUS AREA", based on a keyword search of five descriptive variables in the CRS: project title, short description, long description, channel name, and channel reported name. Additional adjustments were made based on CRS purpose codes, as detailed in eTable 6, in order to ensure that the specified purpose corresponded to the highest-weighted health focus area.

Estimating disbursements for the 24 bilateral channels and the EC

Both the DAC tables and the CRS rely on information reported by DAC members and other institutions to the OECD-DAC. Hence, the quality of the data varies considerably over time and across donors. Three variables were used to estimate yearly donor disbursements: CRS commitments, CRS disbursements, and DAC commitments. There were two main challenges in using the data from the CRS for this research:

1. underreporting of aid activity to the CRS compared to what is reported to the DAC, and

2. underreporting of disbursement data to the CRS compared to commitment data reported to the CRS.

These issues are highlighted in eFigure 1. Methods developed to account for both these challenges are discussed below. Details on how we estimated the cost of providing technical assistance and program support for these institutions are highlighted below in the section titled calculating the technical assistance and program support component of development assistance for health from loan-and grant-making channels of assistance.

To address these two challenges, we determined a cutoff point for each channel. We defined this channel-specific cutoff year as when the ratio of total CRS disbursements to commitments was greater than 50% and did not drop subsequently below 30%. eFigure 2 below shows each donor's CRS disbursement to commitment ratio in green, and the estimated cutoff year is marked with a vertical red line. For years after the cutoff year, DAH is measured using the unadjusted disbursement data. For the time prior to the cutoff year, it was determined that the disbursement data are not of high enough quality, and adjusted commitments were used instead.

Two adjustments were made to commitments to estimate disbursements before each donor-specific cutoff point:

I. The first adjustment addressed underreporting of aid activity to the CRS (relative to the DAC). To address this challenge, all CRS commitments for the health sector were adjusted upward using the DAC commitment to CRS commitment coverage ratio. The coverage ratio of the CRS was well below 10% before 1996 but has improved steadily over time.

II. The second adjustment addressed underreporting of disbursements data to the CRS (relative to commitments reported to the CRS). To address this challenge, we pooled completed projects in the CRS that have disbursement data for each channel and computed yearly project disbursement rates (the fraction of total commitments disbursed for each year of a project) and overall project disbursement rates (the fraction of total commitments disbursed over the life of each project) by project length. Yearly disbursement schedules were calculated for projects with lengths of one, two, three, four, five, and six years. When an observed project length was more than six years, all expenditure after the sixth year was aggregated and assumed to be expended in the sixth year. This does not happen often. Yearly disbursement rates were the median of these shares, averaged across projects for every donor in each project year. The sum of these averages equals one, so that all the disbursements were expended over the lifetime of a project. We also adjusted donor disbursement schedules by the ratio of disbursements to commitments in the CRS in order to reflect the fact that not all commitments are typically disbursed. The product of these donor-specific yearly disbursement rates, the donor-specific overall disbursement rates, and the donor-specific disbursement to commitment ratios produced the donor-specific disbursement schedules. The donor-specific disbursement schedules were applied to project-level DAC-adjusted commitments reported in the CRS. eFigure 3 shows the yearly disbursement rates and overall disbursement rates for projects with one- to six-year lifespans for each of the 24 member countries and the EC.

Lastly, to address the challenge of underreporting of aid activity to the CRS compared to the DAC for all years, the difference between each donor's aggregate DAC health commitments and CRS health disbursements was added to each donor's yearly DAH. Since only aggregate commitments are reported to the DAC, several adjustments were made, based on more detailed CRS data:

I. First, each donor's yearly median project length was calculated by applying the donor-specific disbursement schedules described above to CRS projects that had disbursement in order to get adjusted DAC commitments.
II. Commitments for projects that have not opened yet were then subtracted, based on the open date reporting in the CRS. This ensured that future disbursements were not captured.
III. The donor-specific ratio of disbursements to commitments was applied to adjusted DAC commitments in order to account for the fact that not all commitments are usually disbursed.
IV. Lastly, these DAC-adjusted commitments were compared to CRS disbursements, inclusive of transfers that were later dropped as double-counting.

In addition to tracking disbursements from the EC, gross disbursements from the DAC were used to compile data on the sources of funding for the EC.

eFigure 1 Comparing CRS commitments, CRS disbursements, and DAC commitments

This figure compares commitments and disbursements from the Creditor Reporting System (CRS) and Development Assistance Committee (DAC) databases of the Development Assistance Committee of the Organisation for Economic Co-operation and Development (OECD-DAC) from 1990 to 2016. CRS disbursements are usually underreported when compared to both CRS and DAC commitments data, especially in earlier years. Because of this gap between CRS and DAC, CRS disbursements data were adjusted to fit DAC commitments data.

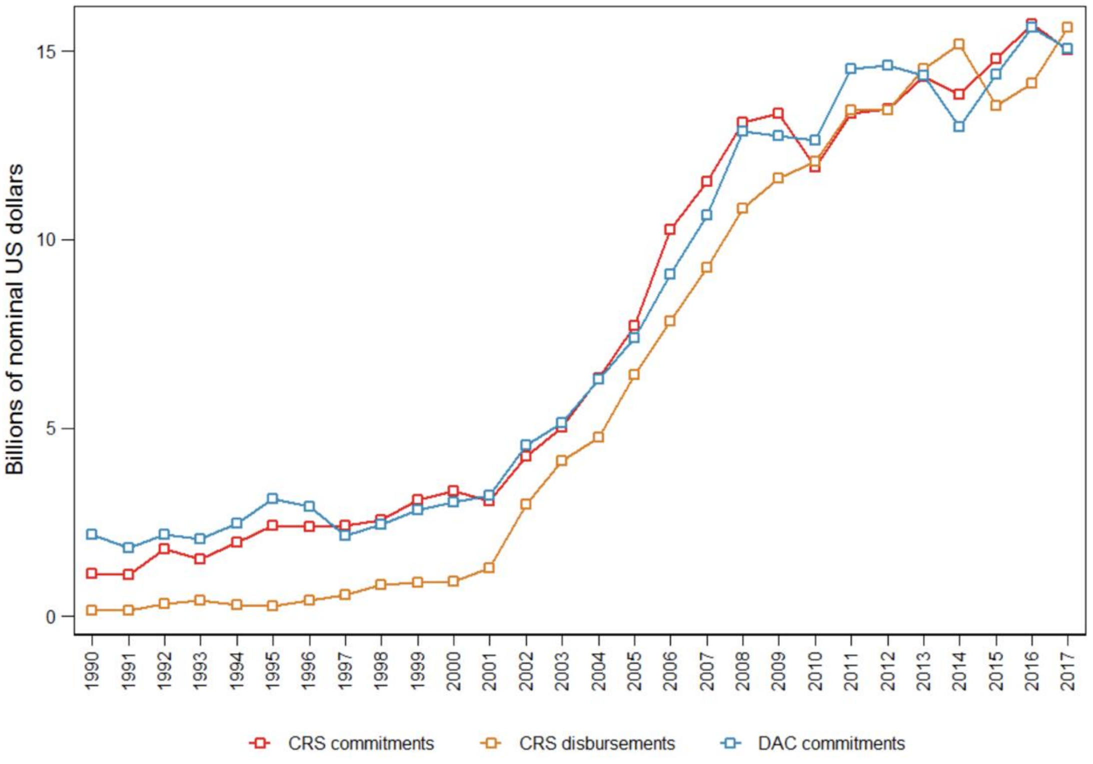

Source: OECD-DAC and OECD Creditor Reporting System

eFigure 2 CRS disbursement to commitment ratio and cutoff points by donor agency

This figure shows the channel-specific cutoff year. Before this year, we adjust CRS commitments using disbursement schedules. After this cutoff we rely on CRS-reported disbursements. The total CRS disbursements to commitments ratio is in green, and the cutoff year is marked with a vertical red line. The cutoff year is determined to be when the ratio goes above 50% and does not fall back below 30%. The vertical axis represents the CRS disbursement to commitment ratio as a percentage. ARE = United Arab Emirates, AUS = Australia, AUT = Austria, BEL = Belgium, CAN = Canada, CHE = Switzerland, DEU = Germany, DNK = Denmark, EC = European Commission, ESP = Spain, FIN = Finland, FRA = France, GBR = Great Britain, GRC = Greece, IRL = Ireland, ITA = Italy, JPN = Japan, KOR = South Korea, LUX = Luxembourg, NLD = the Netherlands, NOR = Norway, NZL = New Zealand, PRT = Portugal, SWE = Sweden, USA = United States of America

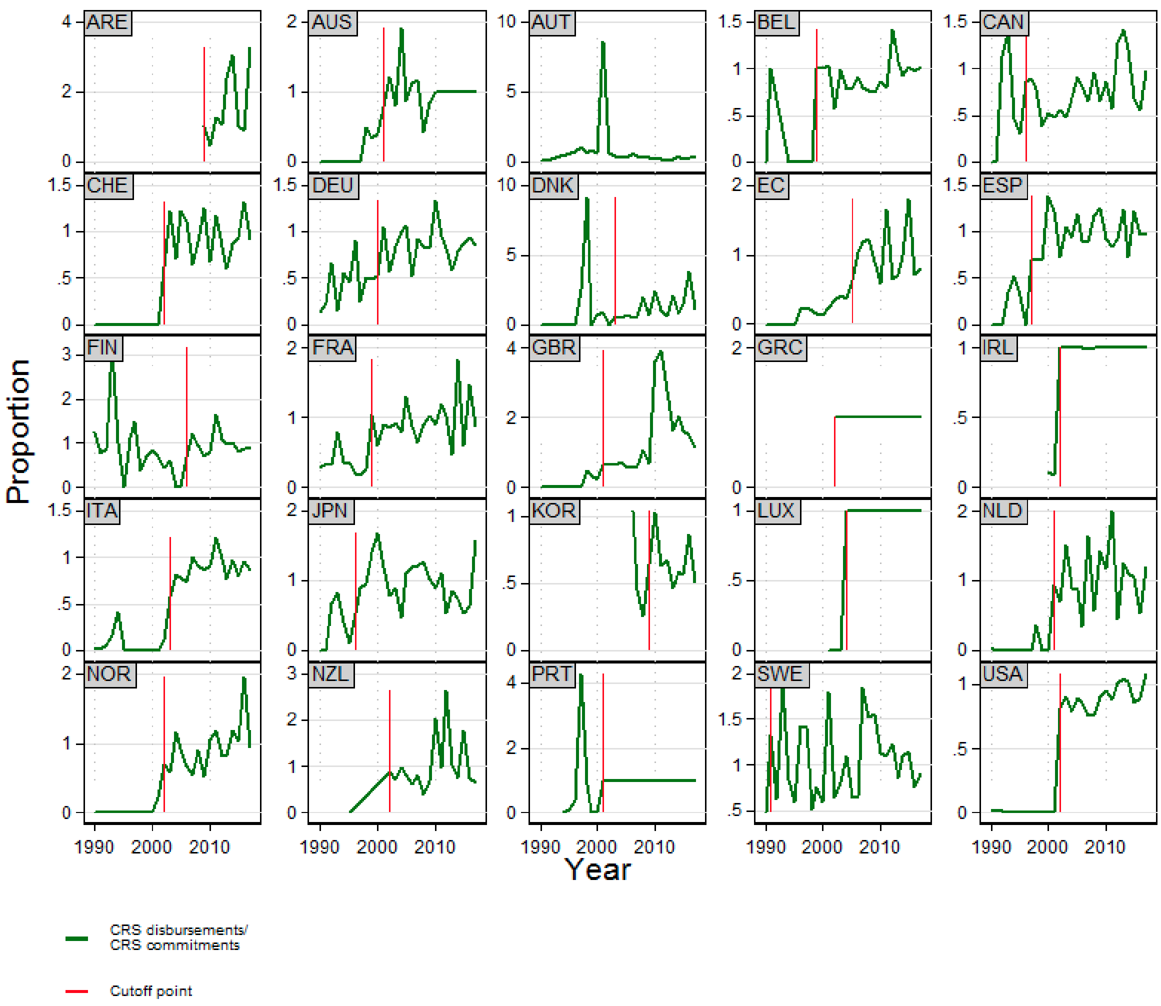

Source: OECD Creditor Reporting System

**Box 3. EXAMPLE. Australia's CRS disbursement to commitment ratio and cutoff year**
**The green line shows the ratio of Australia's disbursements to commitments, as reported in the CRS. Prior to 2001, the ratio was always below 50%. In 2001, the ratio rose above 50%; it did not fall below 30% in subsequent years, thereby defining 2001 as the cutoff year. Thus, for Australia, before 2001 DAH is based on adjusted CRS commitment data. These data are adjusted using disbursements schedules (eFigure 3) and data from the DAC. After 2001, Australia's DAH is based on the disbursements reported in the CRS.**

eFigure 3 One- to six-year disbursement schedules for bilateral channels

This figure shows the estimated disbursement schedules for bilateral channels. Before the channel-specific cutoff year, we rely on commitment data to inform our estimates of DAH. Commitment data are adjusted to reflect disbursements over time using schedules estimated from projects in the CRS that have both commitment and disbursement data. The vertical axis represents the percentage of the commitment disbursed. ARE = United Arab Emirates, AUS = Australia, AUT = Austria, BEL = Belgium, CAN = Canada, CHE = Switzerland, DEU = Germany, DNK = Denmark, EC = European Commission, ESP = Spain, FIN = Finland, FRA = France, GBR = Great Britain, GRC = Greece, IRL = Ireland, ITA = Italy, JPN = Japan, KOR = South Korea, LUX = Luxembourg,

NLD = the Netherlands, NOR = Norway, NZL = New Zealand, PRT = Portugal, SWE = Sweden, USA = United States of America

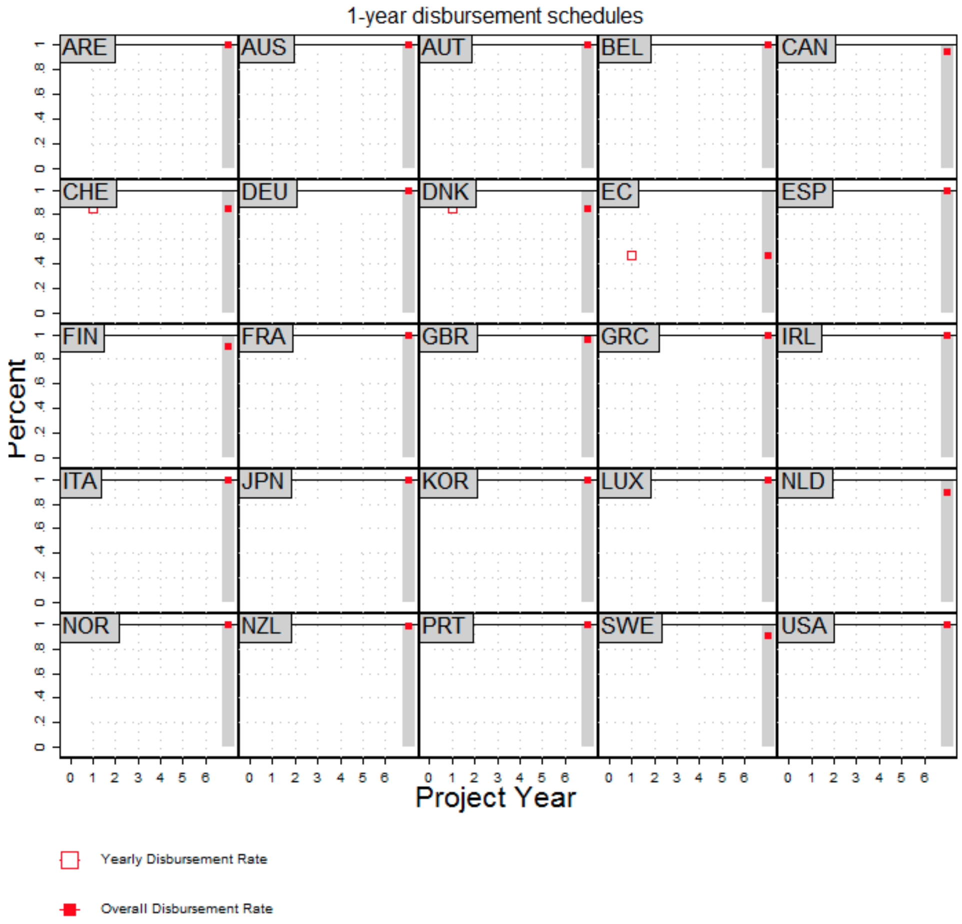

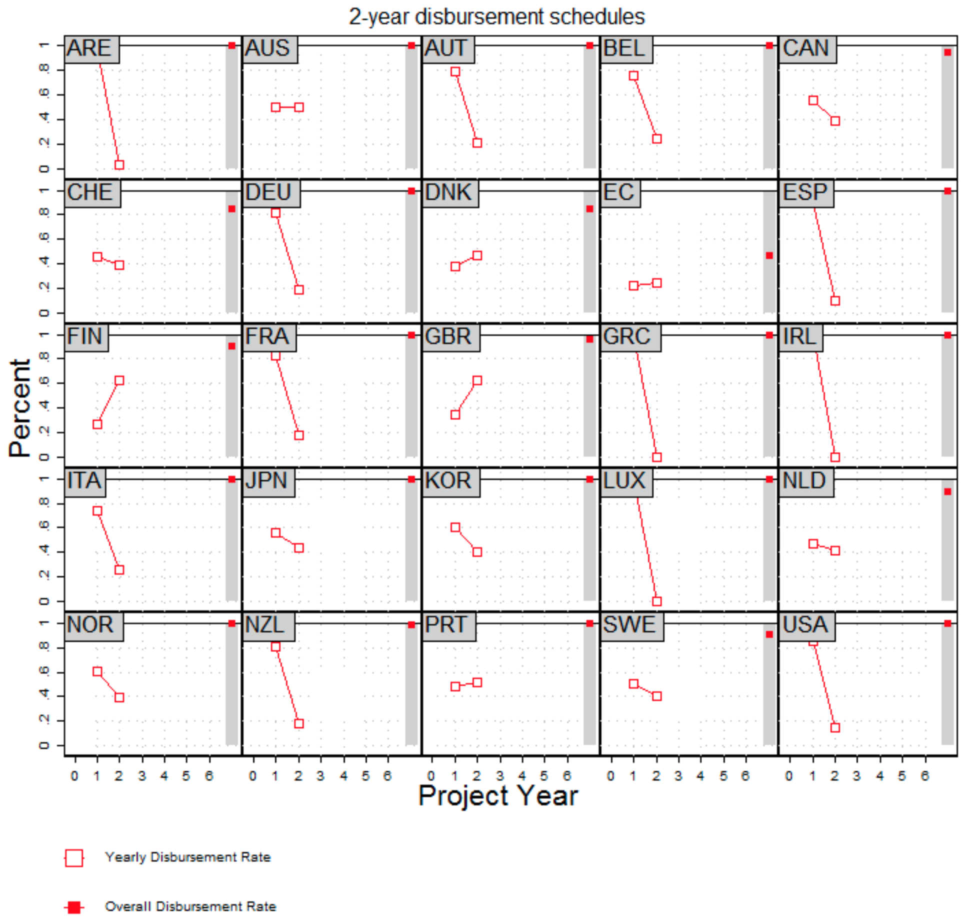
2-year disbursement schedules
ARE
AUS
AUT
BEL
CAN
CHE
DEU
DNK
EC
ESP
FIN
FRA
GBR
GRC
IRL
ITA
JPN
KOR
LUX
NLD
NOR
NZL
PRT
SWE
USA
Percent
Project Year
Yearly Disbursement Rate
Overall Disbursement Rate

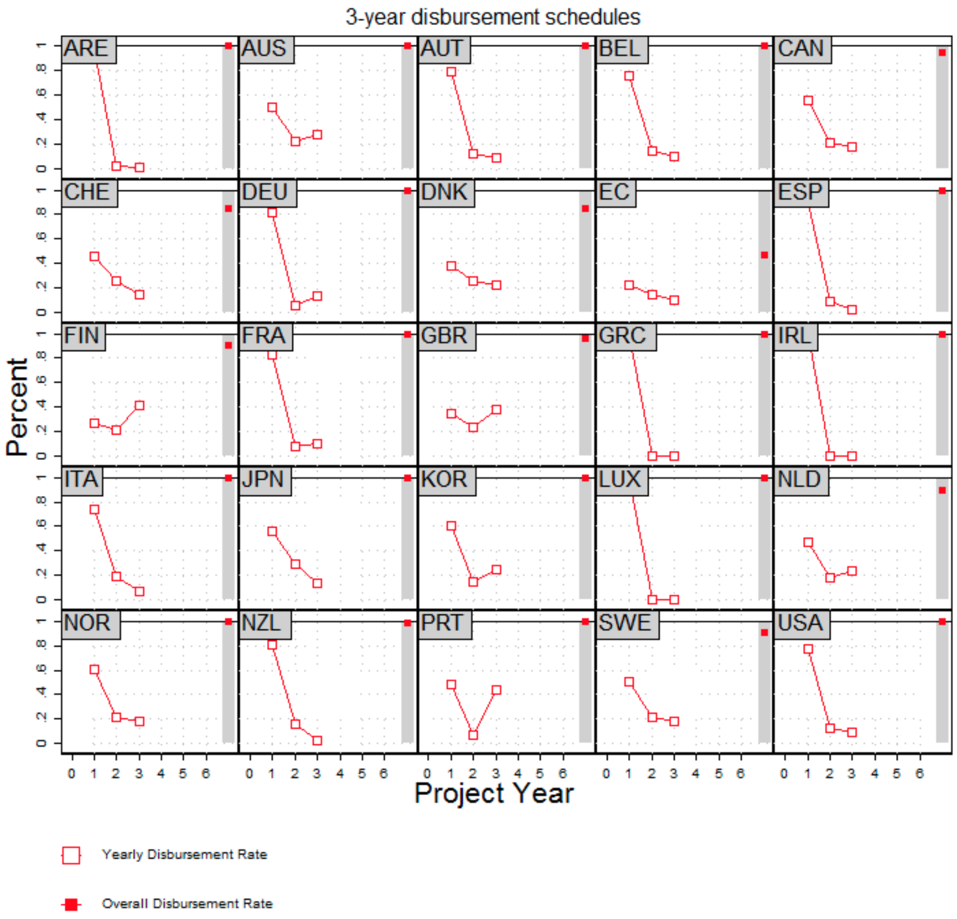
3-year disbursement schedules
ARE
AUS
AUT
BEL
CAN
CHE
DEU
DNK
EC
ESP
FIN
FRA
GBR
GRC
IRL
ITA
JPN
KOR
LUX
NLD
NOR
NZL
PRT
SWE
USA
Percent
Project Year
Yearly Disbursement Rate
Overall Disbursement Rate

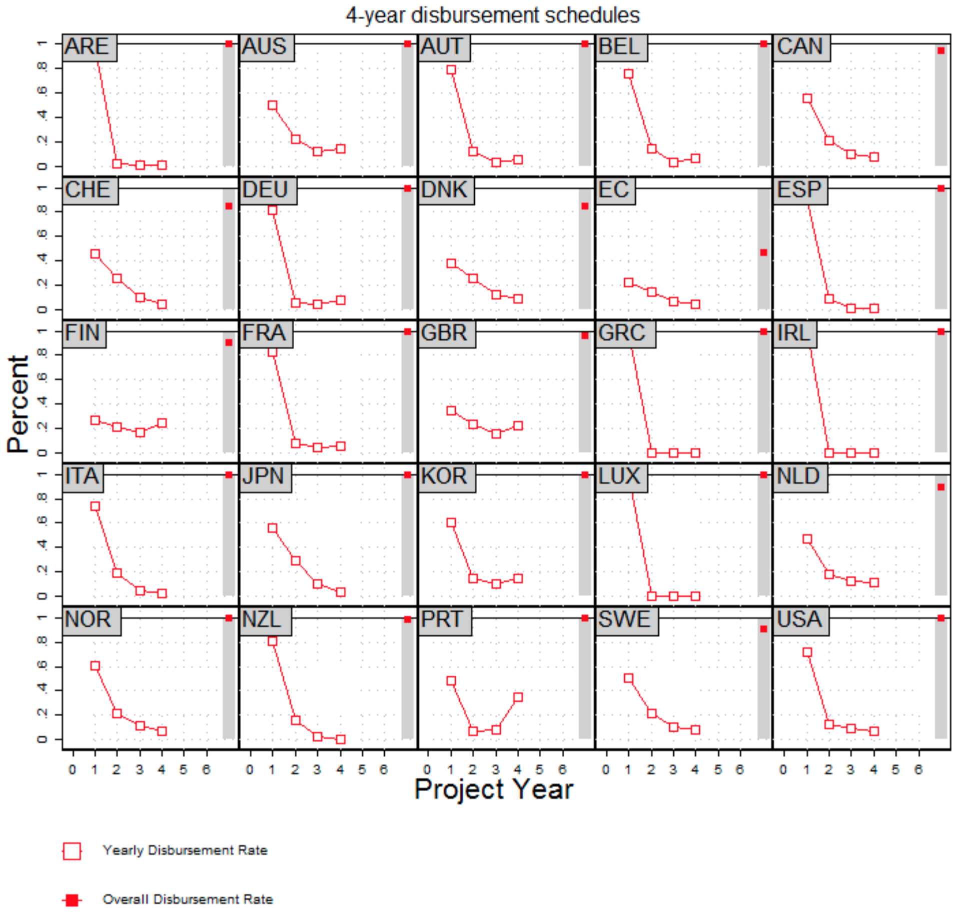
4-year disbursement schedules
ARE
AUS
AUT
BEL
CAN
CHE
DEU
DNK
EC
ESP
FIN
FRA
GBR
GRC
IRL
ITA
JPN
KOR
LUX
NLD
NOR
NZL
PRT
SWE
USA
Percent
Project Year
Yearly Disbursement Rate
Overall Disbursement Rate

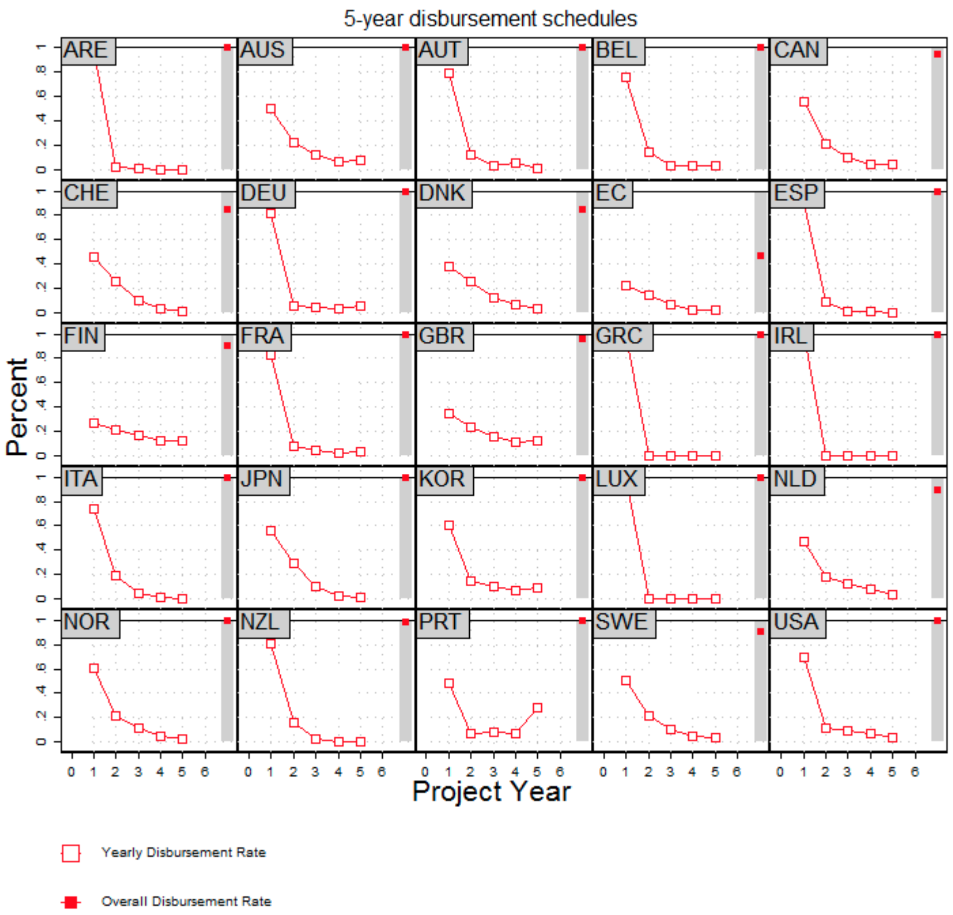
5-year disbursement schedules
ARE
AUS
AUT
BEL
CAN
CHE
DEU
DNK
EC
ESP
FIN
FRA
GBR
GRC
IRL
ITA
JPN
KOR
LUX
NLD
NOR
NZL
PRT
SWE
USA
Percent
Project Year
Yearly Disbursement Rate
Overall Disbursement Rate

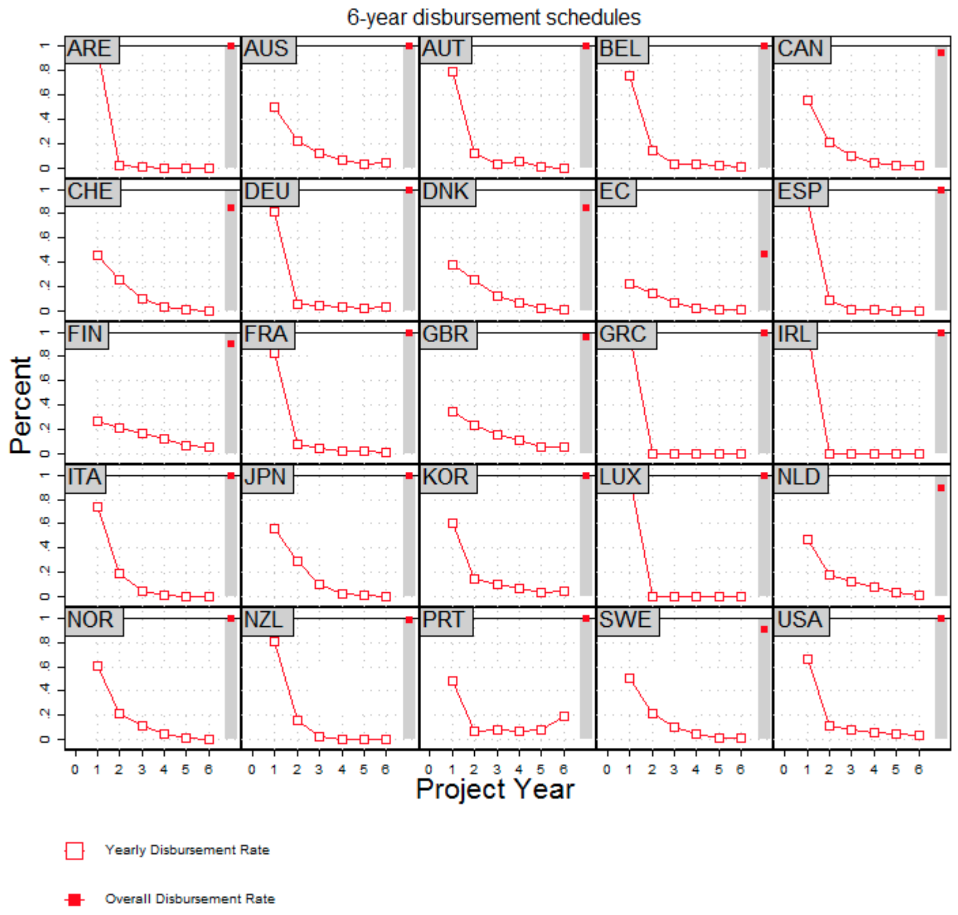
6-year disbursement schedules
ARE
AUS
AUT
BEL
CAN
CHE
DEU
DNK
EC
ESP
FIN
FRA
GBR
GRC
IRL
ITA
JPN
KOR
LUX
NLD
NOR
NZL
PRT
SWE
USA
Percent
Project Year
Yearly Disbursement Rate
Overall Disbursement Rate

Box 4. EXAMPLE. Australia's one- to six-year disbursement schedules
**To estimate disbursements using commitment data, we rely on disbursement schedules derived from CRS data that include both commitments and disbursements. Disbursement schedules are specific for each channel and the length of a project. These schedules also take into consideration the average amount of commitments for each channel that lead to disbursements. Across all Australian projects in the CRS with complete disbursements data, Australia disbursed 100% of the funds that it committed, as shown by the solid red dot on the right-hand side of the Australia panel (upper left corner of the first panel of eFigure 3). In projects with a length of one year, Australia disbursed 100% of the funds that it committed in that year. For two-year projects, Australia disbursed 50% of total disbursements in year one and 50% of total disbursements in year two. In projects with lengths of three years, Australia disbursed about 50% of total disbursements in year one and 23% and 27% of total disbursements in years two and three, respectively. This is estimated for projects ranging from one to six years. The disbursement schedules were applied to commitment data from the CRS to estimate disbursements for years prior to the cutoff year, which is 2001 for Australia.**

To predict DAH for the recent years not reported in the CRS, budget data were extracted from a variety of sources. These data are listed in eTable 4. Global health budgetary data were utilized whenever possible, but these detailed data were available as a complete time series only for Australia and the United States. For all other bilateral channels, general ODA budgets were used. In order to predict DAH for 2018 for 24 bilateral agencies, the budget ratio for each donor was calculated by dividing DAH estimates by the corresponding budget data (ODA or global health). Budget ratios were projected using a weighted average of the previous three years (placing one-half weight on the one-year lagged ratio, one-third weight on the two-year lagged ratio, and one-sixth weight on the three-year lagged ratio), and this ratio was multiplied by the observed budgeted DAH for those same years. eFigure 4 plots the budget ratio for each bilateral channel. Budget data for the EC were inconsistent and did not match the disbursement series. Instead, DAH for 2018 was estimated based on trends in DAH for EC member countries. A weighted average was applied to the percent change in DAH from 2016-2018 for all EC member countries. The weighting was based on each country's total national contributions to the EC. These data were collected from the EC's financial statements.[74] The weighted average was then applied to the EC's 2017 DAH to forecast 2018.

eFigure 4 DAH as a percentage of corresponding budget data by bilateral agency

This figure shows the trend of the ratio of DAH measured as a share of budget data. Green dots indicate that a donor provided global-health-specific budget data, so in these cases the denominator is all global-health-specific budgeted data. The numerator is estimated DAH. Red dots indicate that a donor did not have global-health-specific budget data, so overall ODA budget data were used in calculating the DAH to budget ratios. The vertical axis represents estimated DAH as a fraction of corresponding budget data. ARE = United Arab Emirates, AUS = Australia, AUT = Austria, BEL = Belgium, CAN = Canada, CHE = Switzerland, DEU = Germany, DNK = Denmark, ESP = Spain, FIN = Finland, FRA = France, GBR = Great Britain, GRC = Greece, IRL = Ireland, ITA = Italy, JPN = Japan, KOR = South Korea, LUX = Luxembourg, NLD = the Netherlands, NOR = Norway, NZL = New Zealand, PRT = Portugal, SWE = Sweden, USA = United States of America

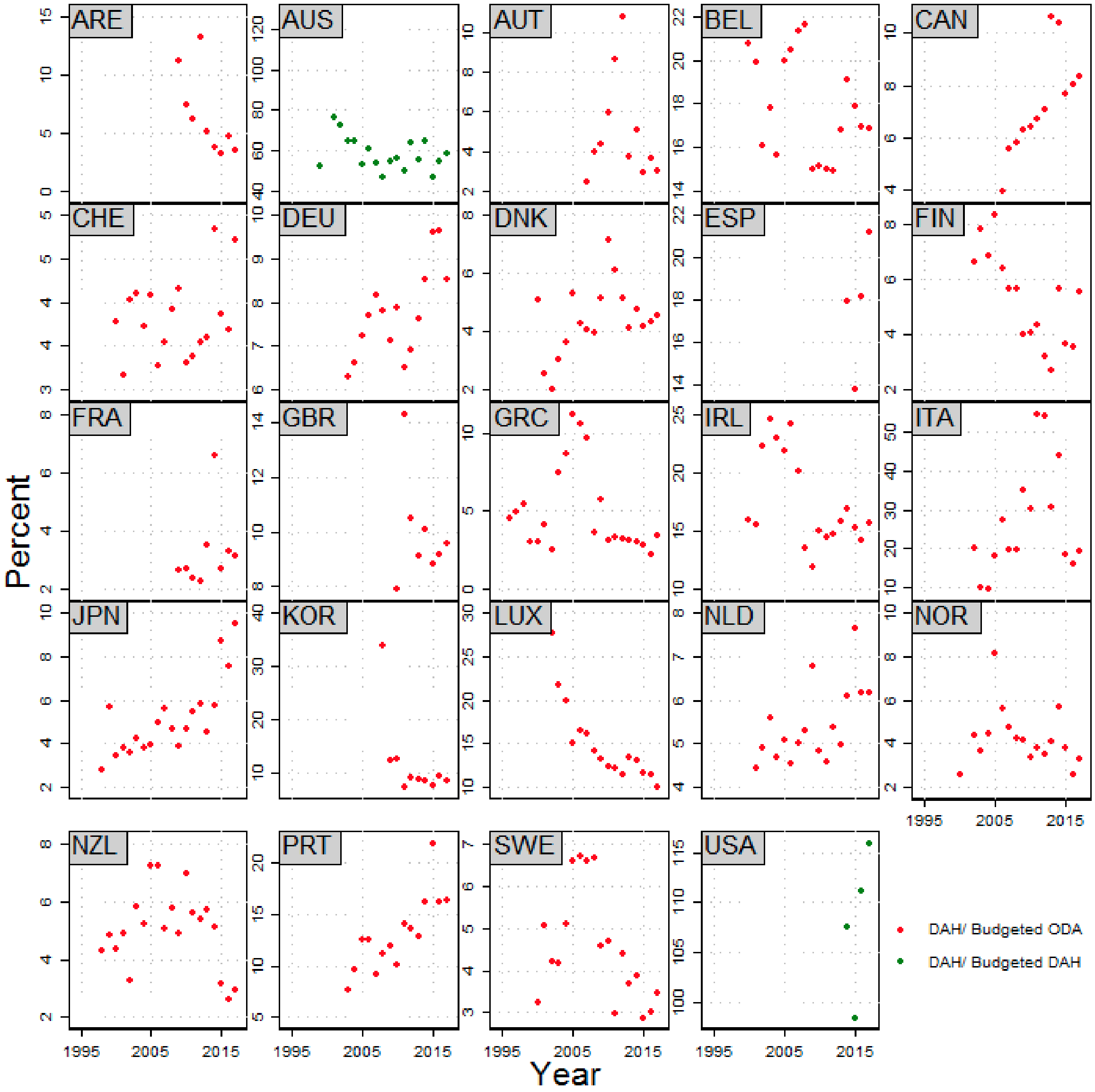

Source: IHME DAH Database (2018) and corresponding bilateral ODA/DAH budget documents outlined in eTable 2 and 4.

**Box 5. EXAMPLE. Australia's DAH as a percentage of corresponding budget data**
Australia provided global-health-specific budget data for 1998-2018 through its International Development Assistance and Overseas Aid Program budgets. For 1998-2017, health ODA and observed DAH were used to create DAH to budget ratios. These budget ratios were then applied to 2018 health ODA budget data to project DAH in 2018, using a weighted average: $(Total\ DAH_t) = \left(\frac{1}{2}\right)(Budget\ ratio_{t-1})(Budgeted\ GHE_t) + \left(\frac{1}{3}\right)(Budget\ ratio_{t-2})(Budgeted\ GHE_t) + \left(\frac{1}{6}\right)(Budget\ ratio_{t-3})(Budgeted\ GHE_t)$ where t = year to be modeled.

To supplement our estimates of development assistance for health to HIV/AIDS and malaria program areas for the United States, we used additional available data from the President's Emergency Plan for AIDS Relief (PEPFAR)

and the President's Malaria Initiative (PMI). We downloaded data on all planned funding by PEPFAR by recipient country, year, and program area from 2004 to 2017.[75] All PEPFAR projects were assigned to our eight HIV/AIDS program areas using PEPFAR budget codes, splitting out overhead costs equally to all other program areas. We then created country-year specific HIV/AIDS program area fractions out of total annual HIV/AIDS DAH, which we applied to all United States HIV/AIDS projects in the CRS from 2004 to 2017 by country-year. To inform malaria funding by program areas, we downloaded the most recently available malaria funding tables from malaria operational plans for all countries and years.[76] We used a keyword search to assign each line item in these tables to our eight malaria program areas, and then created fractions for the malaria program areas out of the total annual malaria DAH specific to each country-year. These fractions were applied to all United States malaria projects in the CRS from 2006 to 2017 by country-year.

eFigure 5 Malaria DAH to program areas as assigned by keyword search and PMI reports

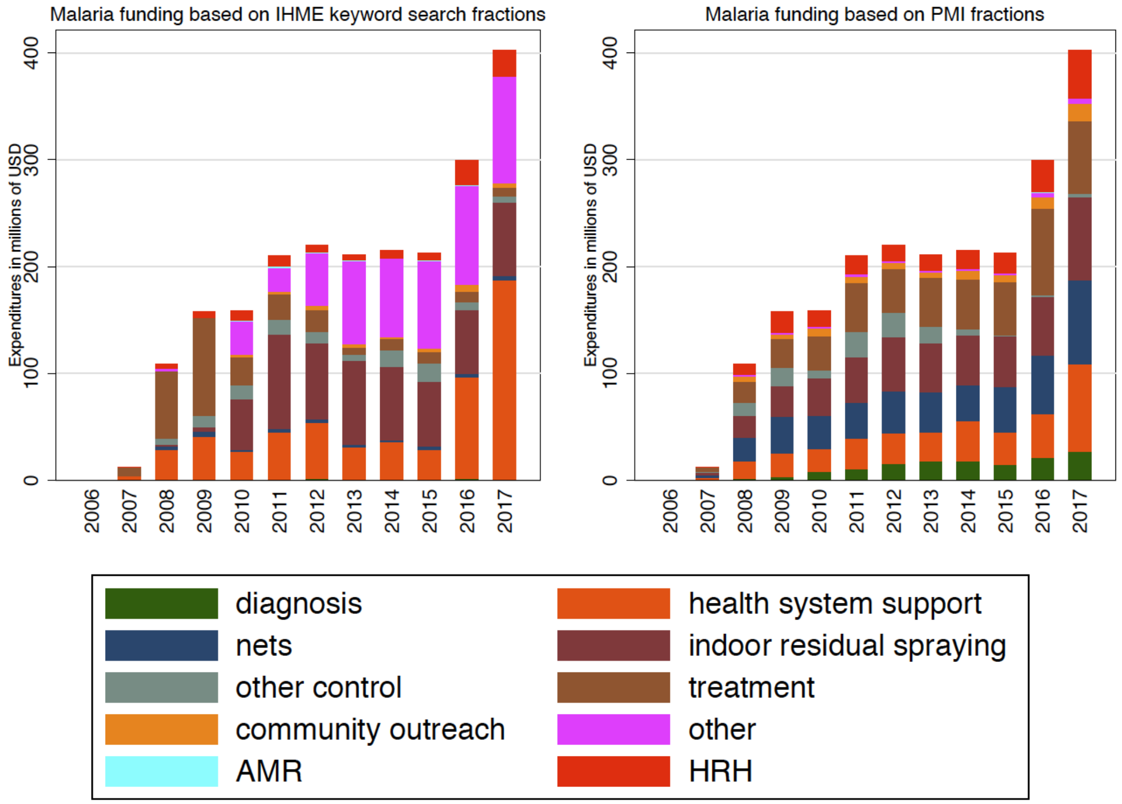

Source: IHME DAH Database (2018) and PMI malaria operational plans

This figure outlines the assignment of funding to malaria program areas for United States projects from the OECD CRS from 2006 onwards. The figure on the left shows how malaria funding is broken out based on keyword search. The figure on the right shows the breakdown of funding to malaria program areas based on PMI malaria funding tables. Using the data from PMI reduces the amount of funding allocated to the "other" program area. As such, in as often as more disaggregated information on project allocation is available, IHME uses such project information available in project budget documents or other project documents to disaggregate into program areas.

Tracking development assistance for health from the development banks

The World Bank project-level health disbursement data for 1990 through August 2018 were obtained through correspondence with Kathleen E. Krackenberger , Operations Analyst.[77] The World Bank recently underwent a recoding process for their disbursements. This recoding affected health disbursements, however the recoding was not completed for projects with disbursements prior to 2001. To create a comparable dataset adjustments had to be made. Regression analysis to predict health disbursements were explored, however, in the end the average percent change between project-level health disbursements before and after recoding was used to adjust health disbursements prior to 2001. It was observed that on average, between 2001 and 2005 (inclusive) the recoding process decreased health disbursements by 0.22%. This number was used to adjust all project-level health disbursements prior to 2001.[78] Health disbursements included all health projects as well as other sector projects with a health sector code. In addition, data were collected from the World Bank online loans database in order to fill in descriptive information for loans from the two arms of the World Bank: the International Development Association (IDA) and the International Bank for Reconstruction and Development (IBRD).[79] Along with keyword searches, health theme codes were used to allocate disbursements by health focus area. The online database contains up to five sector codes and five theme codes that can be assigned to each project. Sector codes represent economic, political, and social subdivisions, while theme codes represent the goals or objectives of World Bank activities. The codes are summarized in eTable 7. Emergency recovery loans were excluded since they do not fit the definition of DAH.

eTable 7 World Bank's health sector and theme codes

**Health sector codes**<br>**Sector codes represent economic, political, or social subdivisions within society. World Bank projects are classified by up to five sectors.**	**Health theme codes**<br>**Theme codes represent the goals or objectives of World Bank activities.**
**Historic (prior to 2001):**<br>(1) **Basic health**<br>(2) **Other population health and nutrition**<br>(3) **Targeted health**<br>(4) **Primary health, including reproductive health, child health, and health promotion**<br><br>**Current (as of 2001):**<br>**(1) Health**<br>**(2) Compulsory health finance**<br>**(3) Public administration – health**<br>**(4) Noncompulsory health finance**	Current:<br>(1) HIV/AIDS<br>(2) Malaria<br>(3) Tuberculosis<br>(4) Other communicable diseases<br>(5) Population and reproductive health<br>(6) Child health<br>(7) Nutrition and food security<br>(8) Injuries and non-communicable diseases<br>(9) Health system performance<br>(10) Social analysis and monitoring

Data on yearly government contributions were obtained from the DAC statistics in order to disaggregate IDA flows by source. Because China does not report to DAC, we generated contribution estimates using replenishment data. We split the 3-year replenishment amount over the three years of the 16th-19th replenishment, to obtain China's contributions to the World Bank from 2008-2017. We validated our use of replenishment data by extracting China's and other countries' contributions to the World Bank International Development Association (IDA) 16th, 17th and 18th replenishment and comparing the number with the contribution we extracted from the OECD Creditor's Reporting System (CRS)[80]. The trend of contribution from major donors was similar, we therefore used China's replenishment contribution as a proxy of the contribution to World Bank IDA. We further disaggregated the DAH contribution to World Bank IDA from China by multiplying China's contributing proportion over World Bank IDA's total health envelope.

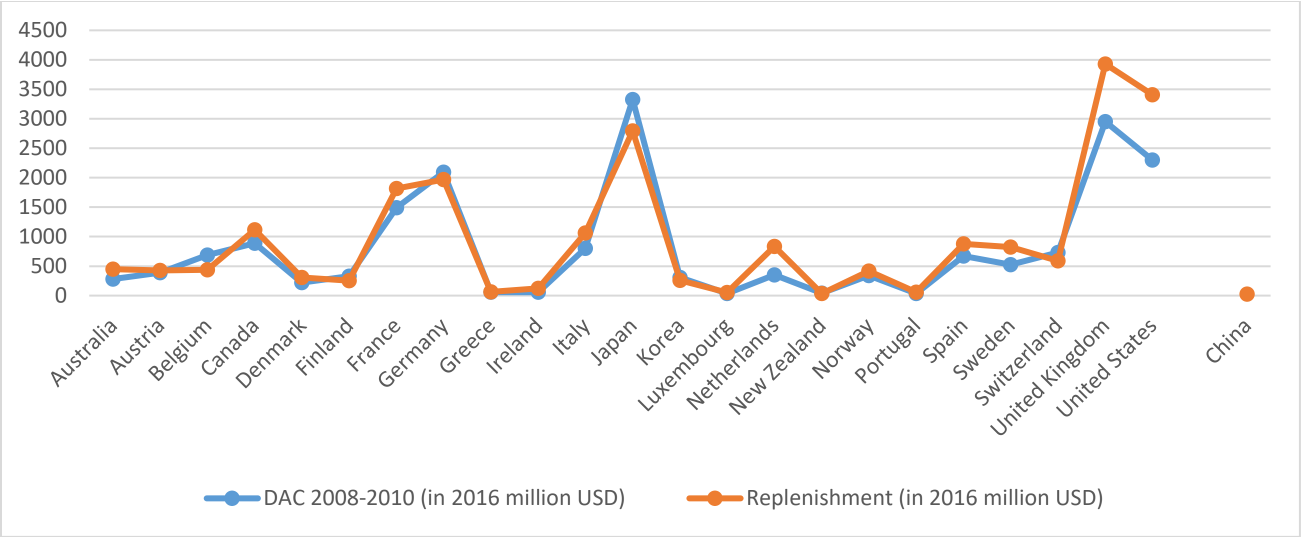

Panel a. Comparison of World Bank International Development Association 16th Replenishment

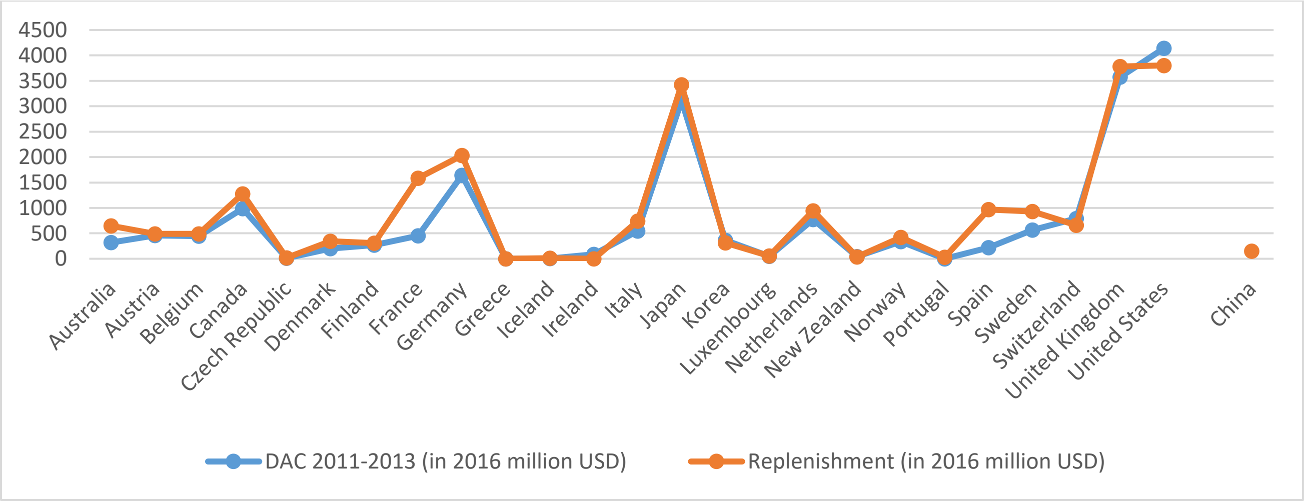

Panel b. Comparison of World Bank International Development Association 17th Replenishment

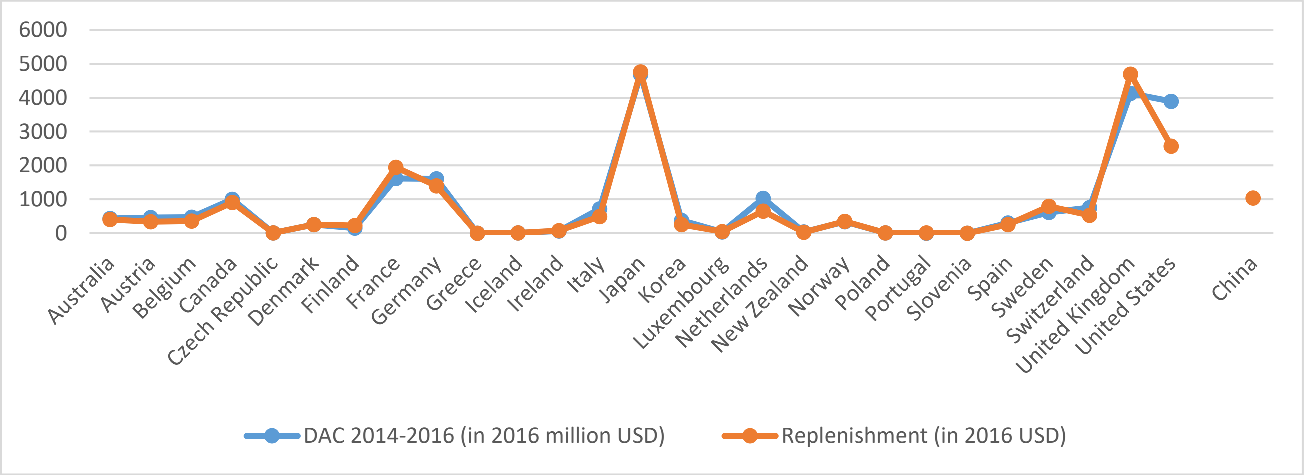

Panel c. Comparison of World Bank International Development Association 18th Replenishment

eFigure 6 Comparison of World Bank IDA Replenishment and DAC

The three figures show the comparison of country commitment to World Bank IDA replenishment rounds, and yearly government contributions reported to DAC statistics. The orange line shows the country commitment to each IDA replenishment round. The blue line shows the sum of yearly reported contribution during the three-year replenishment round. Panel a is contributions and commitments from replenishment round 16; panel b is contributions and commitments from replenishment round 17; panel c is contributions and commitments from replenishment round 18.

Details on how we estimated the cost of providing technical assistance and program support for these institutions are highlighted below in the section titled "Calculating the technical assistance and program support component of development assistance for health from loan-and grant-making channels of assistance." The data received from the World Bank captured disbursements for Jan 1st to Aug 15th of 2018, so the 2018 total estimate for IDA and IBRD were rescaled to 12 months.

Regional development banks

The Asian Development Bank (ADB), and Inter-American Development Bank (IDB) all maintain their own loan databases, which were used to estimate disbursements.[15,16,81] To estimate health disbursements from the African Development Bank (AfDB), data was received via correspondence with Ms. Josselyne Ahogny (Manager, Loan accounting division)[35] To estimate health disbursement from the Asian Development Bank (ADB), data were received via correspondence with Dr. Rouselle F. Lavado. eTable 8 provides a summary of the data sources used across the regional banks. Furthermore, eFigure 6 displays the disbursements for AfDB from 1990 to 2018 and eFigures 7, and 8 display commitments and disbursements from 1990 to 2018 for AsDB and IDB.

For AfDB, we received project level disbursement data from 2001 through December 2018. For pre-2001 estimates, data from the Compendium of Statistics were used for estimates pre-2001. Donor information for projects funded by the African Development Fund was based on subscription information from the 9th to 14th replenishment reports[82]. The sole donor to projects implemented by the Nigeria Trust Fund was Nigeria, and all income for African Development Bank projects was attributable to other sources.

For ADB, we received project-level disbursement data from 1990 through 2018, including recipient, project title and description and annual disbursement. Keyword search was performed to identify health focus area of the projects. For income source of ADB, data for country donations were extracted from the replenishment reports of Asian Development Fund, and we manually divide the three or four years' replenishment into annual contributions[83]. We extracted the total revenue of Ordinary Capital Resources and Asian Development Fund from the Financial reports of ADB to generate the total envelope of income sources, and subtracted the country contribution to provide the estimate for resources coming from the bank itself. Using the fraction for the Bank's own resources and country contribution, we provided the estimate of income source for each project. For 2018, where we do not yet have the total envelope, the 2017 fraction for income source was used as a preliminary estimate for 2018.

The IDB's project database also provided commitments and disbursements for all projects. The same methods were used for estimating annual disbursements from the IDB as were used for the ADB. Through correspondence, 2018 health loan disbursements were obtained. These numbers were used in the 2018 estimates. The dataset used to estimate disbursements for IDB was updated in September 2018. However, since these channels have so few new projects each year, it was assumed that smoothing disbursements over time for reported projects captured the majority of total disbursements for 2018. The contribution quotas by donor for the Fund for Special Operations from 1990-2016 were extracted from Annual Financial reports for the IDB. The ratio of each donor's contribution to total contribution was applied to total disbursements to assign income sources. In 2017, the Fund for Special Operations was transferred to the Ordinary Capital. Donor assignments in 2017 onwards are unallocable.

eTable 8 Summary of data sources for the regional development banks

This figure indicates the data available and used to estimate DAH. (X) indicates that project-level data are present in the dataset. (-) indicates that project-level data are not present in the dataset.

Institution	Data source	Commitments	Cumulative disbursements	Yearly disbursement	Notes
**African Development Bank (AfDB)**	Compendium of Statistics	X		(Aggregate - not at the project level)	The Compendium of Statistics was not available for 1990-1993, 1995, and 1998-1999; we estimated yearly disbursements using the average of neighboring disbursements
	Correspondence			X	Annual loan disbursements from 2001 through December 2018 were provided.
**Asian Development Bank**	Correspondence	X	X	X	Annual grant loan disbursements from 1990 through 2018 were provided.
**InterAmerican Development Bank**	Online projects database		X		As yearly disbursement amounts are not provided in the online database, we estimated yearly disbursements by allocating cumulative disbursements over each year of the project.
	Correspondence			X	Loan disbursements from January modelled December 2018 were provided.

eFigure 7 Disbursements by the African Development Bank

The orange line with triangles shows estimated disbursements based on the Compendium of Statistics from 1990 through 2001 and actual disbursements received from 2001 onwards.

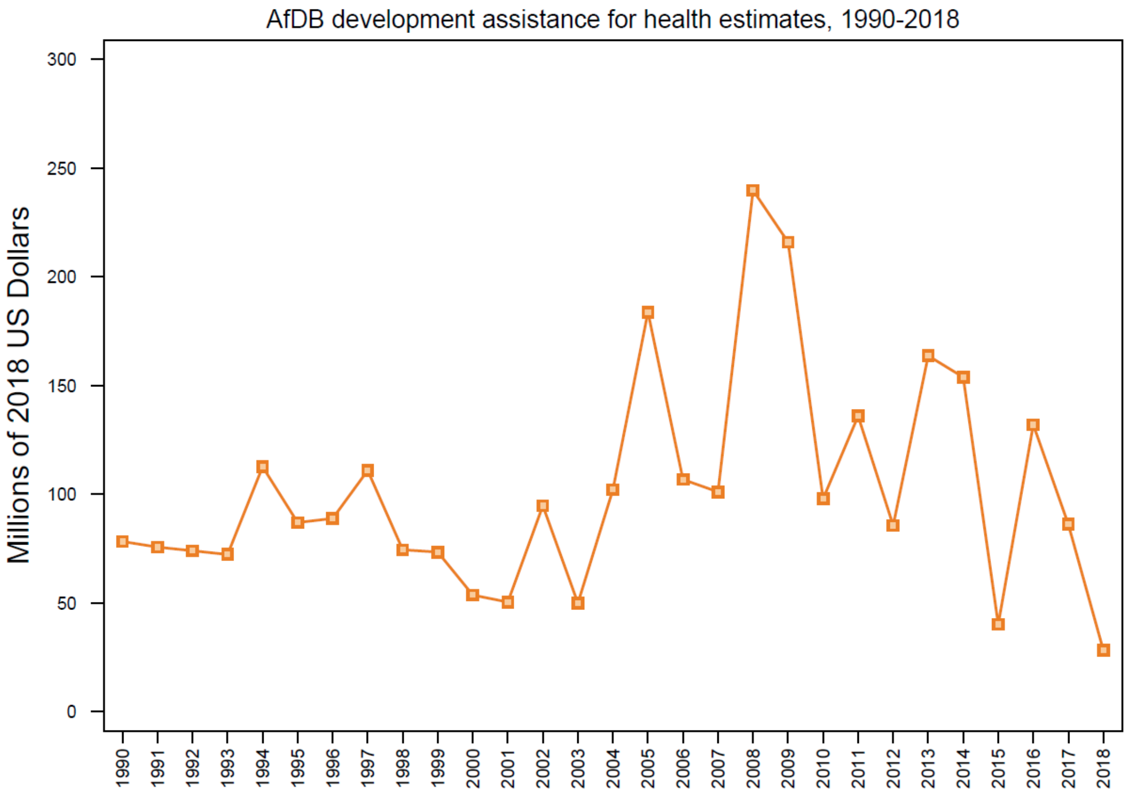

Source: IHME DAH Database (2018) and African Development Bank Compendium of Statistics.

eFigure 8 Disbursements by Asian Development Bank

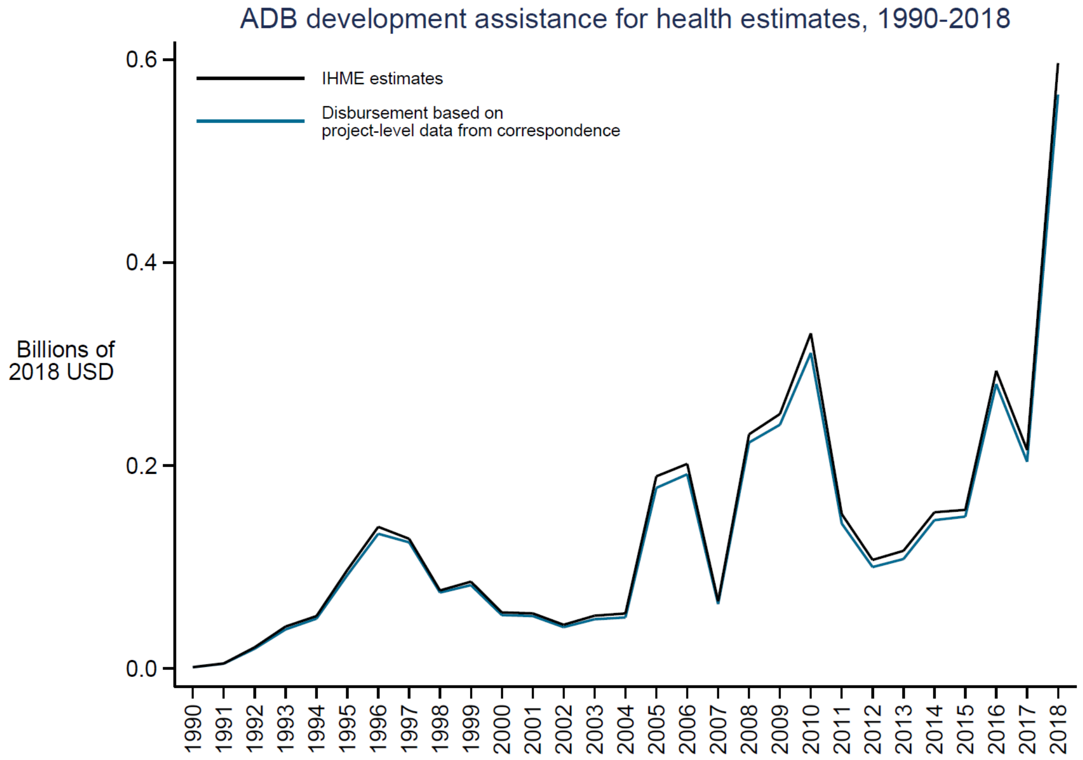

Source: IHME DAH Database (2018)

eFigure 9 Commitments and disbursements by Inter-American Development Bank

The dashed green line shows commitments from the Inter-American Development Bank's (IDB) online projects database. The orange line shows smoothed disbursements from the online projects database, and from correspondence for 2017.

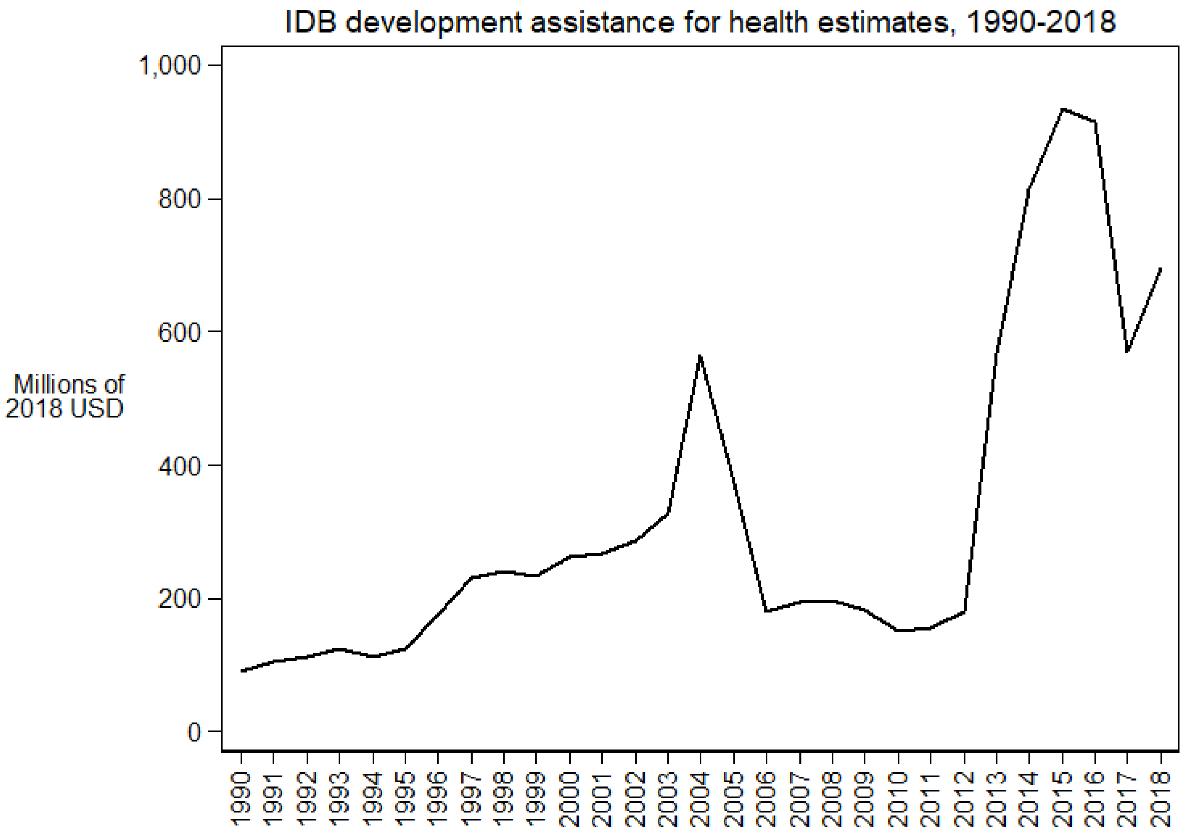

Source: IHME DAH Database (2017) and correspondence

Tracking contributions from GFATM and GAVI

The Global Fund to Fight AIDS, Tuberculosis and Malaria

The grants database made available online by the Global Fund to Fight AIDS, Tuberculosis and Malaria (GFATM) provides grant-level commitments and annual disbursements from its inception in 2002 to the present year.[22] In addition, sources of funding were compiled from the GFATM contributions dataset and annual reports, all downloaded from the GFATM website.[23,24] Regional grants were split evenly between all countries identified in the regional grant documents found on the GFATM website. eFigure 10 shows GFATM's annual contributions received from public and private sources. eFigure 10 shows GFATM's annual commitments and disbursements from its project database from 2002 through 2018.

eFigure 10 Contributions received by the Global Fund to Fight AIDS, Tuberculosis and Malaria

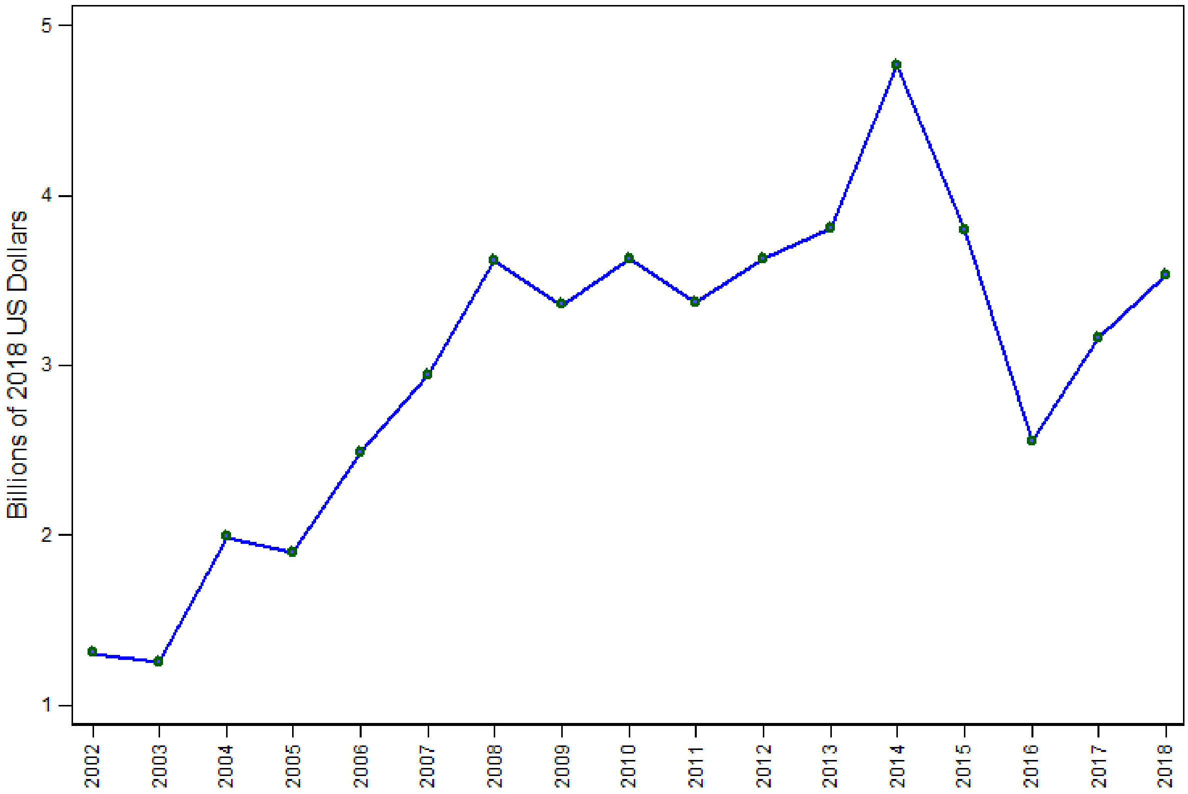

Source: GFATM pledges and contributions 2018

eFigure 11 The Global Fund to Fight AIDS, Tuberculosis and Malaria's commitments and disbursements

The dashed green line shows commitments from the Global Fund to Fight AIDS, Tuberculosis and Malaria's (GFATM) online grants database. The orange line shows disbursements from the online grants database.

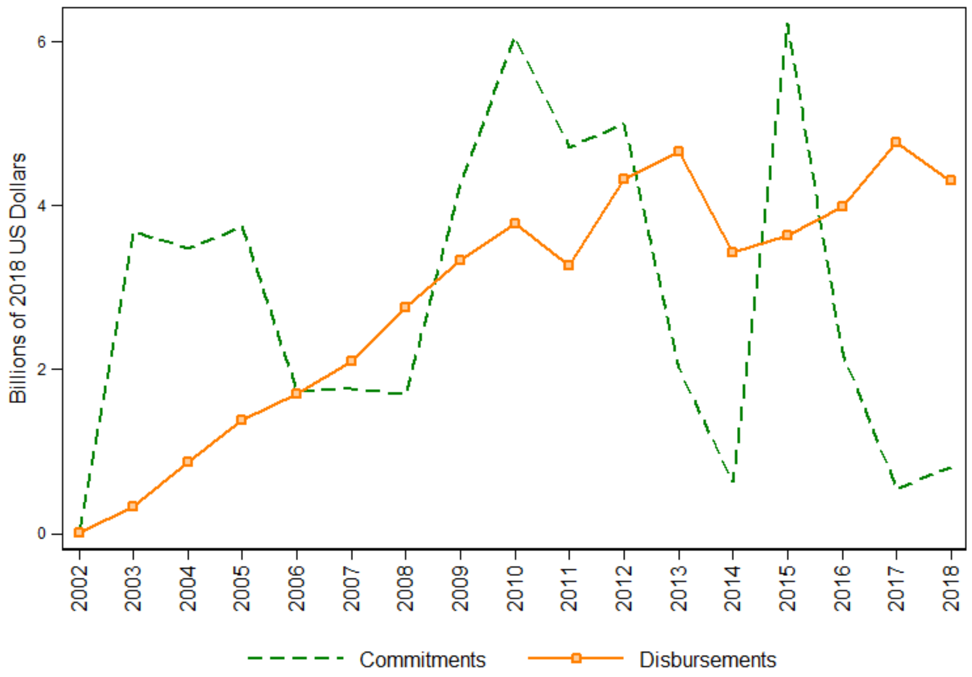

Source: IHME DAH Database (2018)

Gavi, the Vaccine Alliance

Gavi provided publicly available project-level data on commitments, disbursements, and investment cases from 2000 through the present.[18,21] Gavi's annual DAH was defined as the sum of (1) project-level disbursements by year paid; (2) investment cases (one-time investments in disease prevention and control); and (3) administrative and work plan costs. Data from Gavi's online databases include expenditure for (1) and (2), but not (3). However, project level data from the CRS for 2007-2012 did include administrative and work plan costs, so disbursements data from the online database were adjusted to match the CRS in those years. The average fraction of administrative and work plan costs was added to total disbursements in 2000-2006 and 2013-2015, the years in which the CRS did not include these data. Contributions data from Gavi's website as well as annual reports from the International Finance Facility for Immunisation (IFFIm) and Advance Market Commitment for Pneumococcal Vaccines were used to determine Gavi's annual income.[19,20,84]

All of the data sources used for Gavi estimates were complete through 2017. Donor contributions received and outstanding pledges data were available on Gavi's website. The unadjusted total pledges were used as total disbursements for 2018. Gavi disbursements were assigned to health focus areas including child and newborn vaccines, HSS, and non-communicable disease as documented in eTable 6 above. Of note, we reclassified all GAVI health system strengthening projects as maternal, newborn, child health specific health system strengthening disbursements

eFigure 12 Gavi's income and disbursements

The dashed green line shows commitments from Gavi's online database. The dashed orange line shows the disbursements from Gavi's online database, which are the sum of project-level disbursements and investment cases.

These data are adjusted using Gavi expenditure data reported to the Creditor Reporting System (CRS) to add administrative and work plan costs to the total. Adjusted disbursements are shown by the solid orange line.

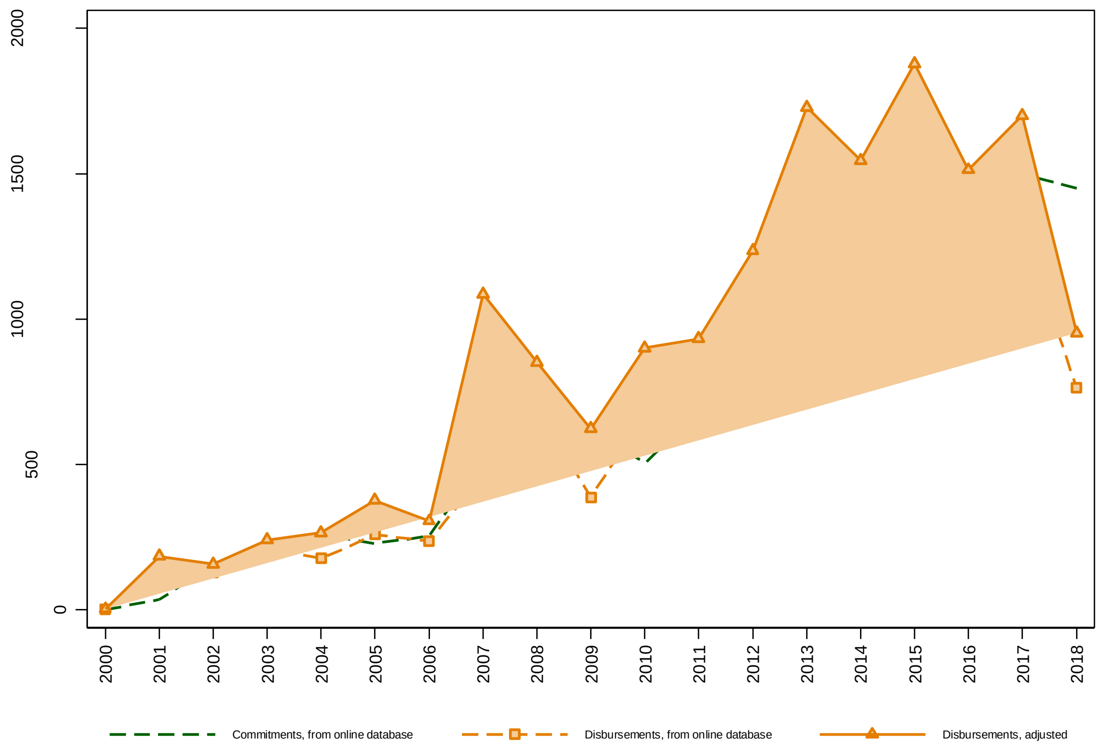

Source: IHME DAH Database (2018)

Coalition for Epidemic Preparedness and Innovation

CEPI provided a complete dataset of all disbursed funding by disease and all income received from donors in 2018, the first year of CEPI disbursements. Funding data was provided per target vaccine and all were classified under the other infectious diseases health focus area. Since CEPI donor data spans from 2017-2018, the proportions by donor across these two years was applied to total disbursements in 2018.

Tracking expenditure by United Nations Agencies active in the health domain

Data on income and expenditures were collected for six UN agencies: WHO, UNICEF, UNFPA, UNAIDS, UNITAID and PAHO. The data sources and calculations for each are described in detail below. Similar to the bilateral channels, we extracted budget data for the UN agencies to predict DAH for years for which we did not have health expenditure data. Model choices and budget measures for UN agencies are presented in eTable 4.

World Health Organization

Data on WHO's budgetary and extrabudgetary income and expenditure were compiled from annual reports and audited financial statements released by WHO.[85] Income data were extracted from WHO's assessed and voluntary

contributions, while expenditure data were extracted from both budgetary and extrabudgetary spending reports. For financial statements representing activities over a two-year period, both income and expenditure data were divided by two, in order to approximate yearly amounts, and dollars were deflated using the US GDP deflator specific to the reporting year. Expenditures from trust funds, regional offices tracked separately, and associated entities not part of WHO's program of activities, such as UNAIDS and GFATM trust funds were excluded. Expenditures from supply services funds were also excluded, as these expenditures pertain to services provided by WHO but paid for by recipient countries. Additionally, WHO projects tracked as paid Ebola expenditure were extracted from the UNOCHA Financial Tracking System database and included as WHO health expenditure.

Disbursement data were not available for WHO in 2018. Much like the bilateral agencies, the ratio of DAH to the total program budget was estimated for 1990-2017 and then predicted for 2018 using the single-year average of previous year. The predicted ratio was then multiplied by the observed program budget for 2018 to get the estimates of DAH.

United Nations Population Fund

Data on income and expenditure were extracted for UNFPA from its audited financial statements.[72] As the 1990-2005 statements represent activities over a two-year period, income and expenditure data were divided by two in order to approximate yearly amounts.

Income and expenditures associated with procurement and cost-sharing activities were excluded from estimates of health assistance because UNFPA uses cost-sharing accounts when a donor contributes to UNFPA for a project to be conducted in the donor's own country. Since this money can be considered domestic spending that goes through UNFPA before being returned to the country in the form of a UNFPA program, it is not included in calculations of total DAH. UNFPA's additional expenditures for these projects come from trust funds or regular resources and are therefore, captured in our estimates. To estimate disbursements by health focus areas, UNFPA's total health expenditure was multiplied by the proportion of funding reported for each program area from annual reports from 1997 through 2013 and from the UNFPA transparency portal for 2014 through 2017. Maternal and child health spending classified as "other" was split equally between the maternal and child health program areas. Additionally, UNFPA projects tracked as paid Ebola expenditure were extracted from the UNOCHA Financial Tracking System database and added to UNFPA health expenditure.

The disbursement data for UNFPA were available through 2017. For year 2018, much like the bilateral agencies, the ratio of DAH and income was estimated for 1990-2017 and then predicted for 2018 using the three-year weighted average of previous years. The predicted ratio was multiplied by observed income to estimate DAH for 2018.

United Nations Children's Fund

Data on income and expenditure for UNICEF were extracted from its audited financial statements.[69] As these statements represent activities over a two-year period from 1990-2011, income and expenditure data were divided by two in order to approximate yearly amounts. The audited financial statements from 2012 onwards are produced on an annual basis.

Since UNICEF's activities are not limited to the health sector, the fraction of UNICEF's expenditure that was for health was estimated using either financial data from correspondence (2001-2013 observed data used to estimate 1990 through 2000 expenditure) or a combination of annual reports and annual results reports from 2014 through 2017. The annual results reports provide the proportion of funding for each program area, the average of 2014 through 2017 proportions was used to estimate the spending proportion for the years 1990 through 2013. In the annual results report, HIV/AIDS funding was reported separately from health funding so the percentages spent on each health program were proportioned based on total spending for Health. Furthermore, UNICEF projects tracked as paid Ebola expenditure were extracted from the UNOCHA Financial Tracking System and added to estimates for UNICEF's health expenditure.

The product of observed program budget and the weighted average of the DAH to budget ratio (placing one-half weight on the one-year lagged ratio, one-third weight on the two-year lagged ratio, and one-sixth weight on the three-year lagged ratio) was used to predict DAH in 2018.

Joint United Nations Programme on HIV/AIDS

UNAIDS income and expenditure data for both its core and noncore budgets were extracted from its audited financial statements.[67] As financial data are provided on a biennial basis in all years except for 2012 and 2013, the values were divided by two to obtain yearly amounts for all biennium data. Dollars were deflated using the US GDP deflator specific to the reporting year.

For UNAIDS, budget measures were available only for a subset of reported total disbursements. UNAIDS reported total expenditure, which combined Unified Budget and Workplan (UBW) and non-UWB components, but only UBW budget data were available.[68] To predict DAH for UNAIDS in 2018, disbursements in those years were calculated by multiplying the observed UBW budget by the three-year weighted average of the ratio of DAH to the UWB budget (placing one-half weight on the one-year lagged ratio, one-third weight on the two-year lagged ratio, and one-sixth weight on the three-year lagged ratio). UNAIDS disbursements were assigned to HIV/AIDS and TB program areas as documented in eTable 6 above.

UNITAID

Data on project level disbursement was obtain through correspondence with UNITAID. Income data was extracted from the annual financial statements downloaded from UNITAID's website. The project level data provided covered project disbursements from 2007 through 2017 and project budget commitments was provided for 2018.

Pan American Health Organization

The Pan American Regional Office for WHO, or PAHO, reports its income and expenditure in its biennial financial report.[10,86] The funds transferred through the "Rotating Fund" were excluded because developing countries fund this procurement of health commodities which are then used within that funding country , and it therefore does not fit the definition of DAH.

As the financial data are provided on a biennial basis (with the exception of 2010 through 2016, where single-year financial reports were available), the quantities were divided by two to obtain yearly amounts. Dollars were deflated using the US GDP deflator specific to the reporting year.

Correspondence with PAHO revealed that data from the financial statements include both Program and nonProgram funds. The latter include funds that countries provide PAHO, so that PAHO can reinvest these funds into the countries' national health systems. These funds should not be included as development assistance for health, and PAHO provided corrected disbursement numbers for 2008 to 2013. The corresponding disbursement numbers for 2014 and 2015 were identified in the PAHO End-of-Biennium Assessment 2014-2015. These funds were provided as biennial disbursements, so they were divided by two to obtain yearly disbursements. The ratio of Program disbursements numbers provided by PAHO and the sum of Program and non-Program funds collected from financial statements was taken for the years 2008 to 2015. The average ratio was calculated, and this ratio was multiplied through disbursement numbers collected from financial statements from earlier years. In this way, Program and non-Program funds collected from audited statements from earlier years were adjusted to estimate DAH.

For PAHO, disbursement data were not available for 2018. PAHO provided budget information along with disbursements for 2008 to 2017. The average ratio between spending and budget was calculated over the years 2008 to 2017, and this ratio was used to estimate 2018 disbursements.

Tracking development assistance for health from private foundations

Previous studies on foundations outside the US have documented the severe paucity of reliable time series data and lack of comparability across countries.[87] Hence, this research focused efforts on tracking only US foundations.

US Foundations

The Foundation Center maintains a database of all grants of $10,000 or more awarded by over 1,000 US foundations. The Foundation Center has coded each grant by sector and international focus and therefore is able to identify global health grants. IHME purchased a customized dataset with cross-border health grants and health grants to US-based international programs from 1992 to 2015 from the Foundation Center.[32] Grants from BMGF, which were tracked separately, were excluded. Additionally, grants to channels that this research already tracks were excluded.

The Foundation Center adopted a new classification methodology as of FGH 2016. The Foundation Center was able to provide historic data based on the new classification system from 2002 to 2012. In order to obtain the series from 1990 to 2001, we multiplied a weighted fraction calculated based on both old and new classification data values from 2002 through 2004 by the old data series (1992 to 2001) we had previously obtained.

$$(Weighted\ fraction) = \left(\frac{1}{2}\right)\left(DAH_{new\ classification}\right)/\left(DAH_{old\ classification}\right)2002 + \left(\frac{1}{3}\right)\left(DAH_{new\ classification}\right)/\left(DAH_{old\ classification}\right)2003 + \left(\frac{1}{6}\right)\left(DAH_{new\ classification}\right)/\left(DAH_{old\ classification}\right)2004$$

$$(DAH\ Estimate_t) = (Weighted\ fraction)(DAH\ Observed_t)$$

where DAH Observed is the old data values for the series 1990 through 2001

To estimate total health grants in 1990-1991 and 2016-2018, natural log of US foundation DAH was regressed on the lagged natural log of US GDP per capita and year using ordinary least squares estimation. The missing years of data were predicted based on estimated regression coefficients from the equation. Exponents of the predicted values were used as final estimates

$$(ln\ Foundation_t) = \alpha + 1.\beta_1(\ln US\ GDP\ per\ capita_t) + \beta_2(year_t) + \varepsilon$$

Details on how we estimated the cost of providing technical assistance and program support for these US foundations are highlighted below in the section titled calculating the technical assistance and program support component of development assistance for health from loan-and grant-making channels of assistance.

Bill & Melinda Gates Foundation

BMGF has been the single largest grant-making institution in the health domain since 2000; hence, additional research was undertaken to accurately capture its annual disbursements. BMGF’s IRS 990PF filings for years 1999-2008, which report all global health grants disbursed per year, were downloaded from the BMGF website. Additionally, disbursement data for years 2009-2017 were collected from the BMGF online grants database, the OECD CRS and personal correspondence. The OECD CRS data was used to identify NGOs that are double-counted from other data sources.

An ordinary least squares linear regression model was used to predict the disbursement for BMGF for 2018. Since there is a strong correlation between market trends and BMGF annual disbursements, market data including lagged US GDP, lagged yearly average of Berkshire stock returns, lagged yearly average of the Russell Index, and lagged total assets of the BMGF Trust were utilized to predict the total disbursement for year 2018.

$$
\begin{aligned}
(BMGF\ total\ disbursement_t) \\
&= \alpha + \beta_1(US\ GDP\ per\ capita_{t-2}) + \beta_2(Berkshire\ stock\ returns_{t-2}) \\
&+ \beta_3(Russell\ Index_{t-2}) + \beta_4(BMGF\ total\ asset_{t-2}) + \varepsilon
\end{aligned}
$$

BMGF's predicted DAH was adjusted to account for in-kind DAH and double-counting. The difference between

BMGF's final DAH and DAH without in-kind added and double-counting removed from 2003-2017 was regressed using ordinary least squares on DAH without in-kind added and double-counting removed and year. The predicted difference was then subtracted from the predicted DAH from the previous regression for 2018.

The Wellcome Trust

We obtained a complete database of all grants awarded by the trust since 1990 through correspondence. While the primary focus of Wellcome Trust grants is healthcare, the Trust provided a variable that distinguishes grant work intended for low- and lower-middle-income countries from research primarily focused in high-income recipient countries. Wellcome Trust DAH is defined as this subset of low- and lower-middle-income focused grants. The annual disbursement amount was obtained by redistributing the lump sum value of the grant over the stipulated duration of the grant by calculating the number of days per year in which the grant was active and multiplying by the average amount disbursed in each day the grant was active. All data were provided in nominal GBP and were converted to real 2018 USD.

European Economic Area

DAH estimates for the European Economic Area were based on two downloadable project datasets available online. IHME DAH data for 2014 to 2018 was obtained from projects in the 2009-2014 grant period dataset with programme area "public health initiatives", and 2007-2012 data was obtained from projects in the 2004-2009 grant period dataset with sector "health and childcare". Childcare projects not related to health were identified and excluded based on manual screening and keyword search for the following terms: "NURSERY" "ORPHANAGE" "RECREATION" "FOSTER" " CARE HOME " " CHILDREN S HOME " " CHILD DEVELOPMENT CENTRE " " YOUTH CENTER" " YOUTH CAMPS " " RESIDENTIAL CENTRE" " RESIDENTIAL CARE FACILITIES " "CHILDCARE" " INFORMAL EDUCATION FOR CHILDREN " " PREVENT INSTITUTIONALISATION " " SPORTS FIELD" " SPORT FIELD" " SPORTS FACILIT" " SPORTS ACTIVIT" " SPORTS INFRASTRUCTURE" " REINTEGRATION OF JUVENILE OFFENDERS "

We determined that no health projects to low- and middle-income countries were implemented by the EEA prior to the 2004-2009 grant period, based on the report on the Financial Instrument 1999-2003 and the Financial Mechanism 1994-1998.[88,89] Project summary variables were obtained through correspondence, and were used together with project titles to assign DAH to health focus areas using a keyword search. To assign annual funding for projects that were implemented across multiple years, project grants were split based on the average daily spending across the duration of the project and the proportion of the year that the project was in implementation. The relative contributions of donors to the EEA grants (Norway, Iceland, and Liechtenstein) were obtained as constant ratios based on the 2016-2017 Annual Report.[90]

Tracking non-governmental organizations

Currently, there are no centralized, easily accessible databases for tracking program expenses of the thousands of NGOs based in high-income countries that are active in providing development assistance and humanitarian relief worldwide. This study relied on CRS data and the only comprehensive data source identified for a large subset of these NGOs, namely the United States Agency for International Development's Report of Voluntary Agencies (USAID's VolAg report).[27] The report, which includes both US-based and international NGOs that received funding from the US government, provides data from 1990 to 2014 on domestic and overseas expenditures for these NGOs as well as their revenue from US and other public sources, private contributions, and in-kind. Total revenue and expenditure data obtained from the NGOs' IRS tax forms, accessed through the GuideStar online database, were also used in tracking NGOs incorporated in the US.[26] The Guidestar Research Fundamentals Plus dataset was used to obtain information for US-based NGOs in fiscal year 2015, including domestic, overseas, and total expenses, and revenues from all public and private sources.

First, in order to track disbursements from OECD donor countries to NGOs, we utilized channel codes present in the CRS database. The code 21000 identified international NGOs and the code 22000 identified donor-country-based NGOs. In order to remove double-counting, we conducted a keyword search on channels where the donor country was the United States to exclude NGOs present in the USAID VolAg report. Allocation of funding to health focus areas for NGOs tracked through the CRS was assigned as described in the section "DISAGGREGATING BY HEALTH FOCUS AREA", based on a keyword search of five descriptive variables in the CRS: project title, short description, long description, channel name, and channel reported name. For NGOs tracked in the USAID VolAg report, allocation of funding to health focus areas was assigned as described in the section "DISAGGREGATING BY HEALTH FOCUS AREA", based on a keyword search of the NGO's description given in the VolAg report.

In order to use the USAID VolAg data, several challenges were overcome. We outline these challenges here and discuss below the methods employed to estimate a consistent series of DAH channeled through NGOs despite these challenges. First, with the exception of BMGF, it was impossible to track the amount of funding from US foundations routed through US NGOs, which may have led to double-counting in estimates of total health assistance. The second challenge relates to the incompleteness of the universe of NGOs captured through the USAID report. The report provides data on NGOs that received funding from the US government. While this covers many of the largest NGOs, it is not a comprehensive list. A related problem is that the VolAg report only includes NGOs that received funds in a given year. While many of the largest NGOs are consistently funded by the US government and are therefore in the report every year, not all NGOs are reported across all years. Third, health sector-specific expenditure is not reported in the VolAg or systematically reported in IRS tax forms. The VolAg does report overseas expenditure but does not disaggregate this expenditure by sector. Fourth, complete data are lacking in several time periods. The 2016 VolAg provided data through 2014. For NGOs incorporated in the US, IRS tax forms were obtained. Furthermore, prior to 1998 the VolAg report did not include international NGOs. Attempts were made to compile other data on the health expenditures of the top international NGOs, in terms of overseas expenditure, by searching other websites for financial documents and contacting these organizations directly. Getting reliable time series data before 2000 proved to be extremely difficult for even this small sample of international NGOs.

Estimates of the share of overseas expenditure spent on health-related projects drew upon a sample of NGOs for which such data were available. Collecting financial data on health expenditures for each NGO would have been prohibitively time-consuming. Therefore, a sample of NGOs was drawn from the list for each year; the sample included the top 30 NGOs in terms of overseas expenditure and 20 randomly selected US-based NGOs from the remaining pool, with the probability of being selected set proportional to overseas expenditure. Next, health expenditure data were collected for each NGO in this sample by seeking out annual reports, audited financial statements, 990 tax forms, and data from NGO websites. Health expenditure was carefully reviewed to ensure that expenditures on food aid, food security, disaster relief, and water and sanitation projects were not included. eTable 9 summarizes the number of NGOs included each year in the USAID report, the number of NGOs in the sample by

year, and the number of NGOs for which health expenditure data were successfully compiled.

eTable 9 Summary of US non-governmental organizations in the study

Year	Number of US NGOs in VolAG report	Number of international NGOs in VolAG report	Number of US NGOs in IHME sample	Number of US NGOs from sample for which data on health expenditure were found
**1990**	267	-	16	9
**1991**	334	-	19	14
**1992**	385	-	18	15
**1993**	411	-	17	12
**1994**	424	-	17	10
**1995**	416	-	16	12
**1996**	423	-	21	14
**1997**	425	-	23	18
**1998**	435	42	24	22
**1999**	438	-	33	28
**2000**	433	50	34	28
**2001**	442	51	33	26
**2002**	486	58	33	27
**2003**	507	54	42	32
**2004**	508	55	47	33
**2005**	494	59	45	36
**2006**	536	67	50	38
**2007**	556	68	50	40
**2008**	565	78	58	48
**2009**	580	90	57	45
**2010**	579	94	69	57
**2011**	595	112	73	63
**2012**	579	94	69	60
**2013**	519	113	69	52
**2014**	485	106	73	54
**2015**	-	-	67	58

A random effects regression model was fit to predict health expenditure as a fraction of total expenditure using the data for the sampled NGOs. A random effects model was chosen because the sample included observations for several NGOs for multiple years. A random effects model allows for the effect of each type of NGO to be captured distinctly. This model was used to predict the fraction of expenditure spent on health for the remaining NGOs. To ensure that the predicted health fractions were bounded between zero and one, the regression utilized the logit-transformed health fraction as the dependent variable. Since several NGOs in the sample were observed for multiple years, the regression included a random effect that varied by NGO. Five of the nine variables used to predict the health fraction were drawn from the VolAg reports and the Guidestar dataset. They were (1) fraction of revenue from in-kind donations, (2) fraction of revenue from the US government, (3) fraction of revenue from private financial contributions, (4) overseas expenditure as a fraction of total expenditure, and (5) calendar year. The remaining four variables used to predict the health fraction were binary indicators that were constructed based on

keyword searches on the NGO name and NGO description found in the VolAg. For both the NGO name and description, a keyword search was conducted to indicate whether the name or description was sufficiently health-related. Another keyword search was conducted independently on the NGO names and descriptions for keywords that indicated if the NGOs might focus on something other than health. These four indicators proved excellent predictors of health fractions.

$$\begin{aligned} logit(\,NGO - specific\ DAH_{it}) &= \alpha + \ \beta_1(Inkind\ contributions\ fraction_{it}) \\ &+ \ \beta_2(US\ government\ contributions\ fraction\ _{it}) \\ &+ \beta_3(Private\ financial\ contributions\ fractions_{it}) \\ &+ \beta_4(Overseas\ expenditure\ as\ a\ fraction\ of\ total\ expenditure_{it}) \\ &+ \beta_5(Health - related\ name_{it}) + \beta_6(Non - health - related\ name_{it}) \\ &+ \beta_7(Health - related\ description_{it}) + \beta_8(Non - health - related\ desciption_{it}) + U_i + \ \varepsilon \end{aligned}$$

Overseas health expenditure was calculated for individual NGOs in each year by multiplying the estimated health fraction and total overseas expenditure. For the NGOs that were sampled, the observed health fraction acquired through data collection was used. For the unsampled NGOs, the fitted fraction from the previously described random effects regression was used. Total overseas expenditure, reported in the VolAg, was not available for 2015-2018. For US-based NGOs, the 2016 NGO overseas fraction was calculated by regressing the logit transformed observed overseas fraction on a linear time trend using ordinary least squares, for each NGO independently. For these cases, the overseas health fraction was calculated as the product of estimated overseas fraction, estimated health fraction, and total expenditure found in the IRS 990 forms.

$$logit(Observed\ overseas\ health\ expenditure_i) = \alpha + \ \beta_i(year_t) + U_i + \ \varepsilon$$

At this point three reasons remained why the overseas health expenditure for some NGOs remained unknown. First, if an observation was non-US-based, then IRS tax forms were not available and total overseas expenditure could not be calculated. Second, for 2017 to 2018, no data were available. Finally, if an NGO was reported in the VolAg in multiple years but not for an intermittent year, no NGO-specific data were available for the gap year. This would be the case if an NGO received support from the US government one year and then again in a nonconsecutive year. For all three of these scenarios, a panel-based hierarchical linear regression model was used to fill in the overseas health expenditure gaps. Total overseas health expenditure (measured at the NGO-year level) was regressed on US GDP per capita and US bilateral DAH disbursed. Because the US government funds many of these NGOs, US bilateral DAH was an excellent predictor of NGO DAH. A flexible model was employed to allow both the GDP and US government DAH coefficients to vary randomly across NGOs, such that each NGO employed a unique (but not independent) relationship between overseas health expenditure, GDP, and US government DAH. A random intercept was also included to capture the significant unobserved heterogeneity present in our set of NGOs. Once fit, this model was used to predict overseas health expenditure for all remaining gaps.

$$(\,NGO\ DAH_{it}) = \alpha + \ \beta_{1i}(US\ GDP\ per\ capita_t) + \ \beta_{2i}(US\ bilateral\ DAH\ per\ capita_t) + U_i + \ \varepsilon$$

Expenditures financed from each revenue source were then calculated by multiplying overseas health expenditure by NGO-specific revenue fractions. Expenditures from in-kind sources were deflated by a constant fraction. This was determined by comparing the federal upper limit and average wholesale price valuations of drugs on the WHO's Model List of Essential Medicines from the RED BOOK Expanded Database.[28,29] eFigure 13 and eFigure 14 show the income and estimated overseas health expenditure, respectively, of the NGOs in the universe of US- and non-US-based NGOs that were tracked in this study from 1990 to 2015 in constant 2018 US dollars.

eFigure 13 Total revenue received by non-governmental organizations

The orange line shows total revenue for all sources, both public and private, received by NGOs. The green line shows estimates of private financial contributions to NGOs, while the blue line shows private in-kind donations to NGOs.

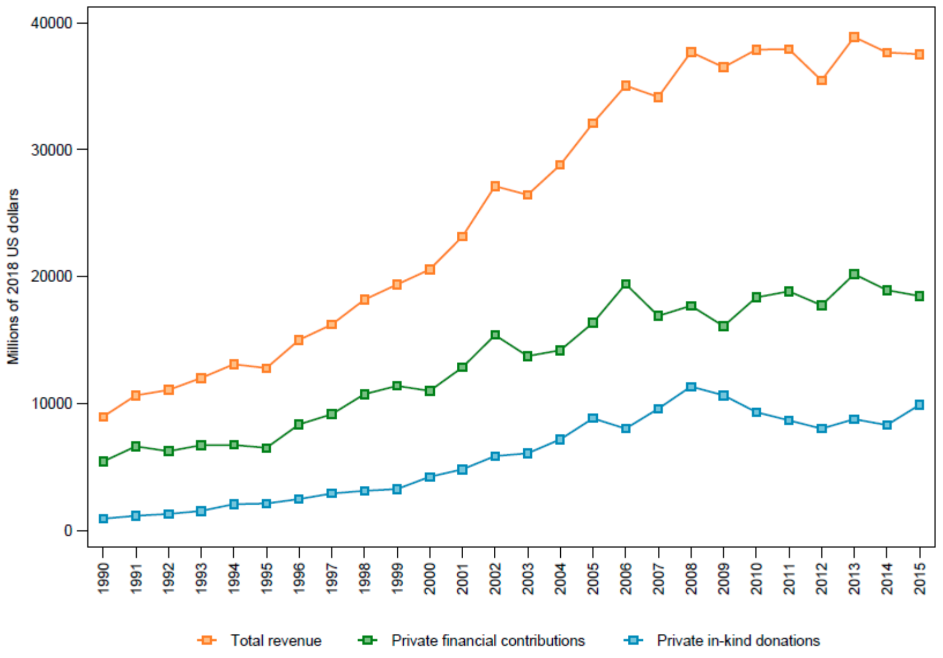

Source: IHME DAH Database (2018)

eFigure 14 Expenditure by non-governmental organizations

The orange line illustrates total overseas expenditure by NGOs, regardless of sector. The green line shows overseas expenditure by NGOs to health-specific recipients, or DAH.

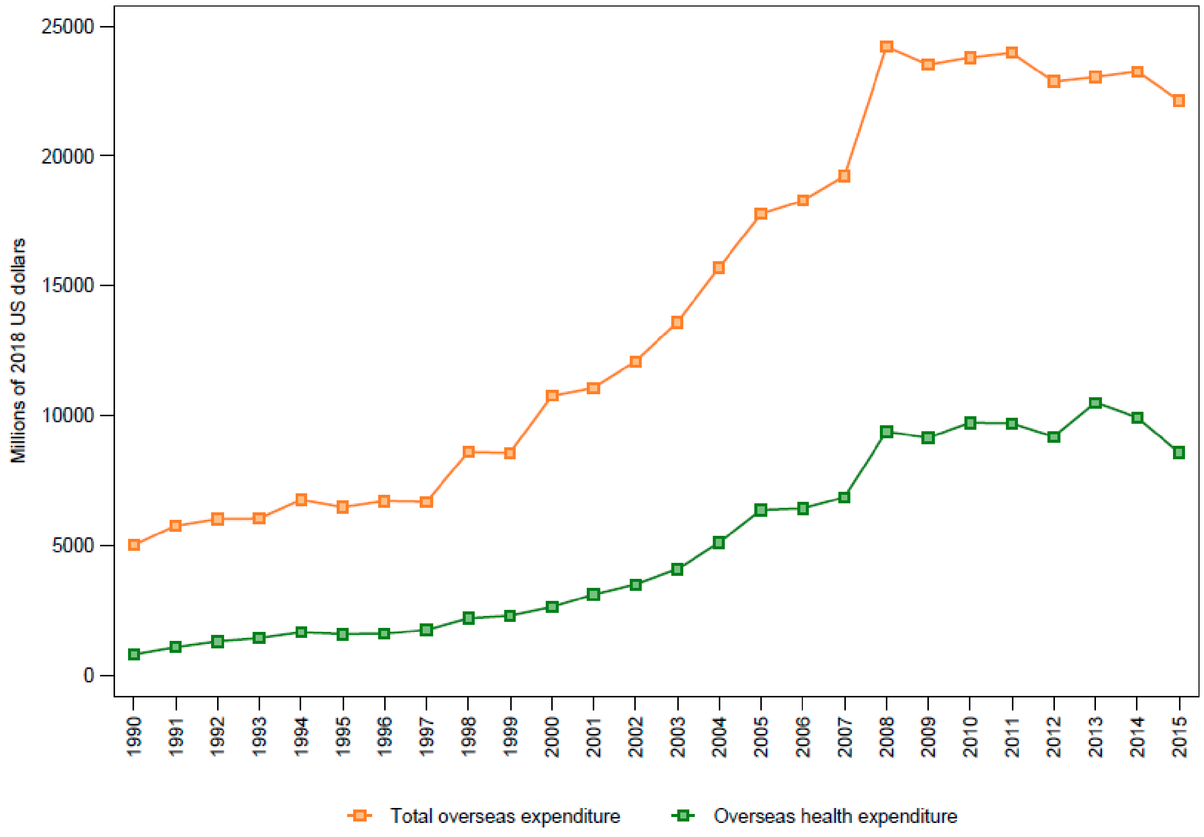

Source: IHME DAH Database (2018)

Calculating the technical assistance and program support component of development assistance for health from loan-and grant-making channels of assistance

The following methods were used to estimate the costs incurred by loan- and grant-making institutions for administering and supporting health sector loans and grants, which includes costs related to staffing and program management.

Data on the total administrative costs were compiled for institutions in our universe for which these data were readily available: IDA, IBRD, ADB, AfDB, IDB, BMGF, the Wellcome Trust, US Foundations, CEPI, GFATM, Gavi, UNICEF, UNFPA, WHO, UNITAID, PAHO, UNAIDS, USAID, and the UK Department for International Development (DFID), Japan International Cooperation Agency (JICA), Norwegian Aid Agency (NORAD) and the Swedish International Development Agency (SIDA). The sources of data for the institutions in this sample are summarized in eTable 10. The ratio of total administrative costs to total grants and loans was calculated for each source by year. It was assumed that the percentage of operating and administrative costs devoted to health would be equal to the percentage of grants and loans that were for health. In other words, if 20% of a foundation's grants were for health, the model assumed that 20% of administrative costs of the foundation were spent on facilitating these health grants. Given this assumption, the ratios of the observed administrative costs to grants/loans were used to estimate the in-kind contribution made by each of these organizations toward maintaining their health grants and loans. For the institutions not in this sample, the ratio from the institution most similar to it was used to arrive at an estimate of in-kind contributions. For example, for US foundations we selected the top 10 and bottom 10 US foundations based on total disbursements across the entire time period. For each foundation, we

calculated the ratio of the "cash basis" column of total operating and administrative expenses to grants paid. We then used the average of these top 10 and bottom 10 foundations ratio as the inkind ratio across time. Total in-kind contributions from all grant- and loan-making global health institutions are shown in eFigure 14.

eTable 10 Summary of data sources for calculating in-kind contributions

Organization	Source	Notes
**BMGF**	990 tax returns (1999-2006)<br>BMGF Trust financial statements (2007-2017)	Used "cash basis" column to calculate ratio of total operating and administrative expenses to grants paid.<br>Used "grants expenditure" statement to calculate ratio of administrative expenditure to grants/program expenditure.
**US Foundations**	990 tax returns	Calculated the average across time of the ratio of the "cash basis" column of total operating and administrative expenses to grants paid.
**Wellcome Trust**	Annual report	Calculated ratio of support costs to grants awarded by the trust
**CEPI**	Board of directors report, 2016-2017	Calculated ratio of operating expenses to total expenditure.
**GFATM**	Annual report financial statements	Calculated ratio of operating expenses to grants disbursed.
**Gavi**	Annual report financial statements	Calculated ratio of management, general, and fundraising expenses to program expenses.
**UNICEF**	Annual report financial statements	Calculated ratio of programme support to total expenditure (1990-2011)<br>Calculated ratio of institutional budget to total programme and emergency budgets (2012-2017)
**UNFPA**	Annual financial review	Calculated ratio of institutional budget to total expenses
**WHO**	Financial report and annual financial statements	Calculated ratio of general operating expenses to total expenses
**UNITAID**	Financial statement	Calculated ratio of operating expenses to program expenses
**USAID**	US government budget database	Used outlays spreadsheet to calculate ratio of total outlays for USAID operating account to sum of outlays for bilateral accounts.
**DFID**	Annual report expense summary	Calculated ratio of DFID's administration expenses to DFID's bilateral program expenses from 2002 onward.
**JICA**	Statement of income in annual report	Calculated ratio of general administrative expenses to operating expenses less depreciation

**SIDA and other Swedish agencies disbursing aid**	Swedish OpenAid website[91]	Calculated ratio of administrative costs to the sum of all program expenses
**NORAD and other Norwegian agencies disbursing aid**	Norwegian Aid statistics website[92]	Calculated ratio of general administrative expenses to operating expenses less depreciation
**IDA**	World Bank audited financial statements	Calculated ratio of management fee charged by IBRD to development credit disbursements.
**IBRD**	World Bank audited financial statements	Calculated ratio of administrative expenses to loan disbursements.
**AfDB**	Income statements in annual reports	Calculated ratio of administrative expenses to the sum of loan and grant disbursements
**ADB**	Annual report	Calculated ratio of administrative expenses to the sum of loan and grant disbursements
**IDB**	Annual report and financial statement	Calculated the ratio of administrative expenses to total loan disbursements
**EEA**	Administrative budget data[93]	Calculated ratio of donor states' management budget and donor programme partners' participation to Net allocation to beneficiary countries
**PAHO**	Audited financial statements	Calculated ratio of general operating expenses and contractual services to total expenses.
**UNAIDS**	Audited financial statements	Calculated ratio of general operating expenses to total expenses.

eFigure 15 In-kind contributions by loan- and grant-making DAH channels of assistance

This figure illustrates the proportions of financial and in-kind DAH disbursed by loan- and grant-making institutions. The proportion of in-kind DAH varies, based on the channel. The overall proportion of in-kind DAH received across all channels has grown over time.

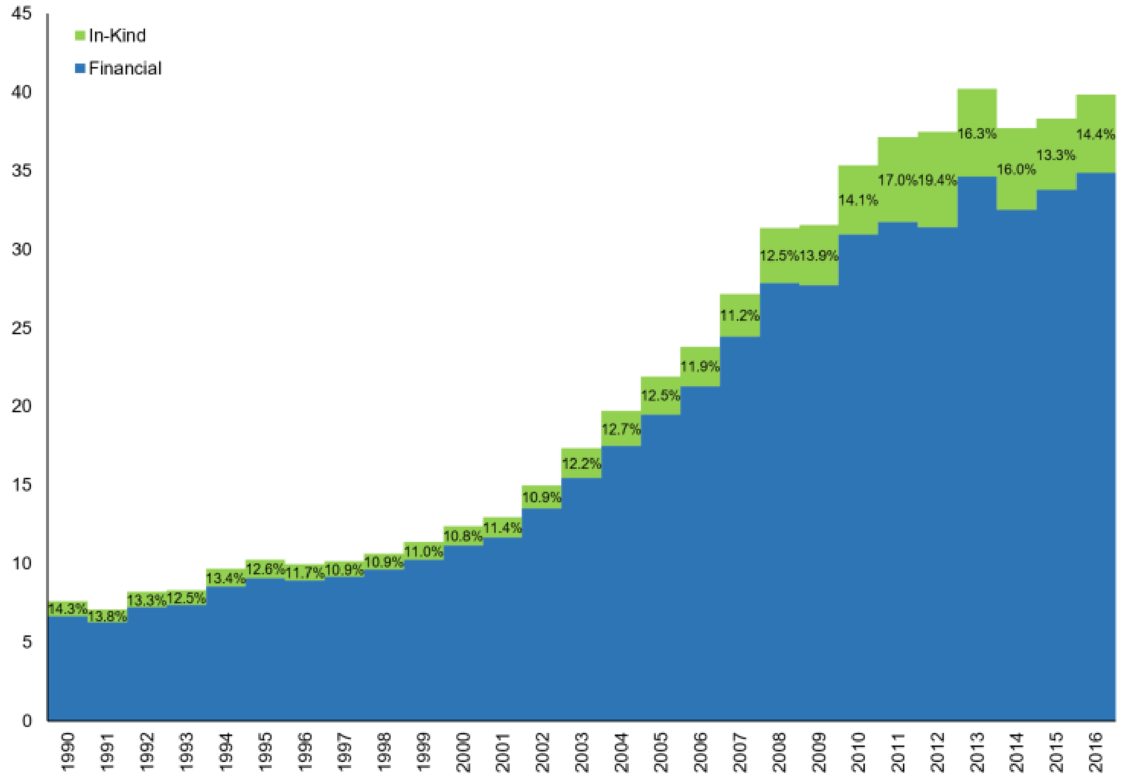

Source: IHME DAH Database (2018)

REFERENCES

1 Dieleman JL, Schneider MT, Haakenstad A, *et al*. Development assistance for health: past trends, associations, and the future of international financial flows for health. *The Lancet* 2016; **387**: 2536–44.

2 OECD. International Development Statistics (IDS) online databases. https://www.oecd.org/development/stats/idsonline.htm (accessed Jan 7, 2017).

3 European Commission. Annual reports - international cooperation and development. Int. Coop. Dev. /europeaid/annual-reports_en (accessed Jan 26, 2017).

4 UNAIDS. PCB Archive. http://www.unaids.org/en/aboutunaids/unaidsprogrammecoordinatingboard/pcbmeetingarchive (accessed Jan 26, 2017).

5 UNICEF. UNICEF integrated budget, 2014-2017. 2013. https://www.unicef.org/about/execboard/files/2013-ABL4-UNICEF_integrated_budget-11Jul2013.pdf.

6 UNICEF. United Nations Children's Fund financial report and audited financial statements. New York: United Nations.

7 UNICEF. Annual Report 2015. UNICEF. https://www.unicef.org/publications/index_92018.html (accessed Jan 30, 2017).

8 United Nations Population Fund. Annual Report 2015. http://www.unfpa.org/annual-report (accessed Jan 30, 2017).

9 UNITAID. Audited Financial Statements (2007-2016). Unitaid. https://unitaid.eu/publications/ (accessed Dec 22, 2017).

10 Pan American Health Organization, World Health Organization. Executive Committee Session. 2016; published online Dec 20. http://www.paho.org/hq/index.php?option=com_content&view=category&id=1258&layout=blog&Itemid=1160&lang=en (accessed Jan 30, 2017).

11 World Health Organization. Financial report and audited financial statement, 2014. World Health Organization, 2015 http://apps.who.int/gb/ebwha/pdf_files/WHA68/A68_38-en.pdf.

12 The World Bank. Projects & Operations. http://projects.worldbank.org/ (accessed Jan 30, 2017).

13 Paris M. Project database 1990-2016 obtained through personal correspondence. 2016; published online Sept 29.

14 African Development Bank. Compendium of statistics on bank group operations. Tunis, Tunisia: Statistics Department, African Development Bank.

15 African Development Bank. Online project database. https://www.afdb.org/en/projects-and-operations/project-portfolio/ (accessed Jan 30, 2017).

16 Inter-American Development Bank. Projects database. http://www.iadb.org/en/projects/projects,1229.html (accessed Jan 30, 2017).

17 Neret M. health disbursement data. 2016; published online Dec 16.

18 Gavi, the Vaccine Alliance. Disbursements and commitments. http://www.gavi.org/results/disbursements/ (accessed Feb 27, 2017).

19 Gavi, the Vaccine Alliance. Pneumococcal AMC. http://www.gavi.org/funding/pneumococcal-amc/ (accessed Jan 30, 2017).

20 Gavi, the Vaccine Alliance. Financial reports. http://www.gavi.org/funding/financial-reports/ (accessed Feb 27, 2017).

21 Gavi, the Vaccine Alliance. Cash Received database. http://www.gavi.org/funding/donor-contributions-pledges/cash-receipts/ (accessed Feb 27, 2017).

22 The Global Fund to fight AIDS, Tuberculosis and Malaria. Grants in detail and Disbursements. http://www.theglobalfund.org/en/portfolio/ (accessed Jan 30, 2017).

23 The Global Fund to fight AIDS, Tuberculosis and Malaria. GFATM annual reports. 2017; published online Jan 30. http://www.theglobalfund.org/en/archive/annualreports/ (accessed Jan 30, 2017).

24 The Global Fund to fight AIDS, Tuberculosis and Malaria. GFATM pledges & contributions report. http://www.theglobalfund.org/en/ (accessed Jan 30, 2017).

25 GuideStar USA. Income tax filings. http://www2.guidestar.org/Home.aspx (accessed Jan 30, 2017).

26 United States Agency for International Development. VolAg report: report of voluntary agencies engaged in overseas relief and development. https://www.usaid.gov/pvo/volag-report (accessed Jan 30, 2017).

27 Thomson Reuters. Red book expanded database. New York: Thomson Reuters, 2009.

28 World Health Organization. WHO | Essential medicines. WHO. http://www.who.int/topics/essential_medicines/en/ (accessed Feb 15, 2017).

29 GuideStar. Data Sets for Research. https://learn.guidestar.org/products/data-sets-for-research (accessed Dec 21, 2018).

30 Bill & Melinda Gates Foundation. Audited Financial statements. Seattle, WA: Bill & Melinda Gates Foundation http://www.gatesfoundation.org/Who-We-Are/General-Information/Financials (accessed Jan 30, 2017).

31 Chan S. Foundation Awards and Payments. 2016; published online Aug 16.

32 Foundation Center. Grants database. Found. Cent. http://foundationcenter.org (accessed Jan 30, 2017).

33 EEA Grants-Norway Grants, Financial Mechanism Office. EEA Grants project portal. https://eeagrants.org/project-portal (accessed Dec 21, 2018).

34 Stimpson A. EEA project data obtained through personal correspondence. Brussels, Belgium. EEA and Norway Grants. 2018; published online Sept 20.

35 Ahogny J. AfDB development support for health projects enquiry. 2017; published online Nov 16.

36 Rouzinova R. Incorporating UNITAID into Financing Global Health Landscape. 2017; published online Aug 21.

37 Al Anood Al Abdool., United Arab Emirates Ministry of Foreign Affairs and International Cooperation. UAE Foreign Assistance in Health 1990-2008 through personal correspondence. 2018; published online Jan 24.

38 Australian Government. Department of Foreign Affairs and Trade. Aid budget and statistical information. Dep. Foreign Aff. Trade. http://dfat.gov.au/aid/aid-budgets-statistics/Pages/default.aspx (accessed Feb 27, 2017).

39 Department of Foreign Affairs and Trade. Annual reports. Dep. Foreign Aff. Trade. http://dfat.gov.au/about-us/publications/corporate/annual-reports/pages/annual-reports.aspx (accessed Feb 15, 2017).

40 Austria Federal Ministry of Finance. Federal budget. https://www.bmf.gv.at/budget/das-budget/das-budget.html (accessed Jan 30, 2017).

41 Belgium House of Representatives. Project budgets. http://www.lachambre.be/kvvcr/showpage.cfm?section=/pri/budget&language=fr&rightmenu=right_pri&story=2017-budget.xml (accessed Feb 13, 2018).

42 Government of Canada. Planning and performance. GAC. http://international.gc.ca/gac-amc/publications/plans/index.aspx?lang=eng#rpp (accessed Jan 30, 2017).

43 Danish Ministry of Foreign Affairs. Foreign affairs budget. http://www.oes-cs.dk/bevillingslove/ (accessed Jan 30, 2017).

44 European Commission. General Budget - budget on-line. http://eur-lex.europa.eu/budget/www/index-en.htm (accessed Jan 30, 2017).

45 Ministry of Finance Finland. State budget bills [in Finnish]. http://budjetti.vm.fi/indox/index.jsp (accessed Jan 31, 2017).

46 Ministry of Foreign Affairs and International Development. Politique francaise en faveur du developpement. Ministry of Foreign Affairs and International Development, 2017 https://www.performance-publique.budget.gouv.fr/sites/performance_publique/files/farandole/ressources/2017/pap/pdf/DPT/DPT2017_politique_developpement.pdf (accessed Feb 12, 2018).

47 Legifrance. Republique Francaise. Budget and financial documents. https://www.legifrance.gouv.fr/initRechTexte.do (accessed Feb 15, 2018).

48 German Federal Ministry of Economic Cooperation and Development. Plan of the federal budget. Fed. Minist. Econ. Coop. Dev. http://www.bmz.de/en/ministry/InDetail/budget/index.html (accessed Feb 13, 2018).

49 Greece Standing Committee on Economic Affairs. The state budget and budgets for certain special funds and services, 2013 and 2014 [in Greek]. 2014. http://www.hellenicparliament.gr/UserFiles/7b24652e-78eb-4807-9d68-e9a5d4576eff/Proyp2014-prak.pdf.

50 Greece Standing Committee on Economic Affairs. Ratification of the State Budget for the financial year 2017. http://www.hellenicparliament.gr/Nomothetiko-Ergo/Anazitisi-Nomothetikou-Ergou?law_id=76e87dd3-8cae-433f-963f-a6c50141de97 (accessed Feb 13, 2018).

51 Department of Finance, Government of Ireland. The Budget. http://www.budget.gov.ie/Budgets/2017/2017.aspx (accessed Feb 15, 2018).

52 Ministry of Foreign Affairs and International Cooperation. Stato di previsione del ministero degli affari esteri e della cooperazione internazionale. 2017. http://www.rgs.mef.gov.it/_Documenti/VERSIONE-I/Attivit--i/Bilancio_di_previsione/Bilancio_finanziario/2017/Allegato-t-17-19/2017-DLB-04-AT-060-Esteri.pdf (accessed Feb 15, 2018).

53 Ministry of Finance Japan. Budget. http://www.mof.go.jp/english/budget/budget/index.html (accessed Jan 31, 2017).

54 Korea Official Development Assistance. Comprehensive Implementation plan for international development cooperation by year. http://www.odakorea.go.kr/hz.blltn2.YearPlanSlPL.do?brd_seq=3&blltn_div=oda (accessed Feb 15, 2018).

55 Ministry of Finance Luxembourg. State Budget[in French]. http://www.igf.etat.lu/.

56 New Zealand Treasury. Vote budget data - budgets of the New Zealand Government. 2017; published online Jan 31. http://www.treasury.govt.nz/budget (accessed Jan 31, 2017).

57 Ministry of Foreign Affairs Norway. Email correspondences. April 18, 2011, February 13, 2012, and August 14, 2013. .

58 Norwegian Ministry of Finance. National Budget. https://www.statsbudsjettet.no/english/ (accessed Feb 15, 2018).

59 Ministry of Finance Portugal. State budget report. http://www.dgo.pt/Paginas/default.aspx (accessed Jan 31, 2017).

60 Ministry of Finance and Public Function Spain. Annual plan of cooperation (PACI). http://www.aecid.es/EN/cultura/Paginas/Publicaciones/Coop_Espanola/PACI/PACI.aspx (accessed Feb 15, 2018).

61 Ministry of Foreign Affairs Sweden. International aid budget. http://www.regeringen.se/ (accessed Jan 31, 2017).

62 UK - Department for International Development. IATI Dashboard. http://dashboard.iatistandard.org/publisher/dfid.html (accessed Feb 22, 2017).

63 Her Majesty’s Treasury United Kingdom. Budget. https://www.gov.uk/government/publications/spring-budget-2017-documents (accessed Feb 15, 2018).

64 Executive Office of the President of the United States. Budget of the United States Government. https://www.gpo.gov/fdsys/browse/collection.action?collectionCode=BUDGET&browsePath=Fiscal+

Year+2014&isCollapsed=true&leafLevelBrowse=false&isDocumentResults=true&ycord=0 (accessed Jan 31, 2017).

65 US Foreign Assistance Dashboard. Foreign assistance by category, health-planned stage. https://www.foreignassistance.gov/explore (accessed Feb 15, 2018).

66 World Health Organization. Proposed programme budget. http://www.who.int/about/finances-accountability/budget/en/ (accessed Jan 31, 2017).

67 Joint United Nations Programme on HIV/AIDS. Financial report and audited financial statement. http://www.unaids.org/en/aboutunaids/unaidsprogrammecoordinatingboard/pcbmeetingarchive/ (accessed Jan 31, 2017).

68 Joint United Nations Programme on HIV/AIDS. Unified budget and workplan. http://www.unaids.org/en/aboutunaids/unaidsprogrammecoordinatingboard/pcbmeetingarchive/ (accessed Jan 31, 2017).

69 United Nations Children's Fund. Financial Report and Audited Financial Statement - Executive Board documents. https://www.unicef.org/about/execboard/index_25993.html (accessed Jan 31, 2017).

70 Sabbah L. Health expenditure data - UNICEF. 2015; published online Sept 29.

71 UNFPA. Report on contributions by member states and others to UNFPA and revenue projections. https://executiveboard.unfpa.org/ (accessed Jan 31, 2017).

72 UNFPA. Statistical and Financial review - Audited Financial report. https://executiveboard.unfpa.org/ (accessed Jan 31, 2017).

73 Smithson M, Verkuilen J. A better lemon squeezer? Maximum-likelihood regression with beta-distributed dependent variables. *Psychol Methods* 2006; **11**: 54–71.

74 Office of the European Union. EU budget 2014 Financial report. Luxembourg: Office of the European Union, 2015 http://ec.europa.eu/budget/financialreport/2014/lib/financial_report_2014_en.pdf (accessed April 17, 2017).

75 President's Emergency Plan for AIDS Relief/ US Government. PEPFAR Dashboards- Country and regional program results, FY 2016. 2017. https://data.pepfar.net/global (accessed Feb 14, 2018).

76 President's Malaria Initiative/US Government. Malaria Operational Plans (MOPs). 2017. https://www.pmi.gov/resource-library/mops (accessed Feb 14, 2018).

77 Krackenberger K. IHME-WB FGH 2018 data request. 2018; published online Sept 10.

78 Projects & Operations - Sectors. http://projects.worldbank.org/sector (accessed Jan 26, 2018).

79 Miyuki Parris. Project database 1990-2016 obtained through personal correspondence. Washington D.C.: The World Bank, 2016.

80 World Bank Group - International Development Association. IDA18 Replenishment. Int. Dev. Assoc. - World Bank. 2016; published online Feb 16. http://ida.worldbank.org/financing/replenishments/ida18-replenishment (accessed Dec 21, 2018).

81 Asian Development Bank. Online project database. https://www.adb.org/projects (accessed Jan 30, 2017).

82 African Development Bank Group. Resource Mobilization and Partnerships. https://frmb.afdb.org/?page=adf&subpage=adf-ar (accessed Dec 21, 2018).

83 Asian Development Bank. Asian Development Fund Replenishments. Asian Dev. Bank. 2014; published online Aug 5. https://www.adb.org/site/adf/replenishments (accessed Dec 21, 2018).

84 Gavi, the Vaccine Alliance. Annual financial reports. http://www.gavialliance.org/funding/financial-reports/ (accessed Jan 30, 2017).

85 World Health Organization. WHO Programmatic and Financial Report (including audited financial statements). Geneva, Switzerland: World Health Organization, 2016 http://apps.who.int/gb/ebwha/pdf_files/WHA69/A69_45-en.pdf?ua=1 (accessed April 17, 2017).

86 Pan American Health Organization. Financial Report of the Director and Report of the External Auditor. 1 January 2015 - 31 December 2015. Washington D.C.: Pan American Health Organization, 2016 http://iris.paho.org/xmlui/handle/123456789/33710.

87 Schlutter A, Volker T, Walkenhorst P. Foundations in Europe: International Reference Book on Society, Management and Law. Gutersloh, Germany; Washington, DC: Bertelsmann Stiftung; Brookings Institution Press, 2001.

88 European Economic Area. EEA grants report. Financial Instrument 1999-2003. Final report. http://eeagrants.org/content/download/10037/139187/version/3/file/141219+FI99-03+final+report.pdf.

89 European Economic Area. EEA report - Financial Mechanism 1994-1998. Final report. http://eeagrants.org/content/download/5915/65422/version/1/file/Financial+Mechanism+1994-1998+Final+Report.pdf.

90 European Economic Area. EEA Annual Report - 2016-2017. https://eeagrants.org/content/download/13045/175890/version/1/file/Annual+Report+2016-2017.pdf.

91 Swedish International Development Agency. Sweden’s aid to the world for all sectors in. Openaid.se. https://openaid.se/aid/ (accessed Dec 21, 2018).

92 NORAD. Norwegian Aid Statistics. NoradDev. https://norad.no/en/front/toolspublications/norwegian-aid-statistics/ (accessed Dec 21, 2018).

93 European Economic Area. Administrative budget - EEA Grants. https://eeagrants.org/Who-we-are/How-we-work/Administrative-budget (accessed Dec 21, 2018).

SECTION 4: Tracking development assistance for health from China

1. Overview

The estimates of DAH contributions from China is based on data from diverse sources including government reports and international development agencies databases. We generate estimates for bilateral and multilateral contributions from China separately and aggregate to obtain the total DAH contributions from China. We use project descriptions and reports where available to determine health focus areas of the contributions. For years with limited data, we use various methods inclusive of interpolation and modeling to generate estimates. Research explaining these methods completely is currently under review.

1 Data

1.1 Summary of Data Sources

We used data from seven sources for the analyses:

- World Health Organization's (WHO) Global Health Expenditure Database
- Institute for Health Metrics and Evaluation's Development Assistance for Health Database (IHME)
- International Monetary Fund's (IMF) World Economic Outlook (WEO) database
- Penn World Tables 9.0 (PWT)
- World Bank (WB) World Development Indicators database (WDI)
- Angus Maddison Project
- United Nations (UN) World Population Prospects (WPP)

Specifically, we collected health expenditure information on all available sources that is comparable across countries and complete for most countries from WHO and IHME, and demographic data from the WPP, while the underlying data for producing gross domestic product (GDP) and general government expenditure (GGE) were extracted from the IMF, WB, and PWT. Table 1 presents the definitions for the various health expenditure sources.

1.2 Institute for Health Metrics and Evaluation's Development Assistance for Health Database

Development assistance for health estimates were obtained from the Institute for Health Metrics and Evaluation's Development Assistance for Health Database. To generate these estimates, IHME collected audited budgets, annual reports, and project records from the primary development agencies providing assistance for the health sector. These records are augmented by information acquired via correspondence, and are standardized and compiled to provide a comprehensive perspective on international financial flows for health. These estimates are tracked backward to the source of the funds and forward to the country recipient, and are available from 1990 through 2018 and 2016, respectively.

1.3 World Health Organization's Global Health Expenditure Database

We used Global Health Expenditure Database (GHED) data from the WHO to generate our estimates, which spans for 185 countries between 2000 through 2016. From the GHED, we pulled the following variables:

(i) Compulsory prepayment (Other, and unspecified, than FS.3)

(ii) Other revenues from NPISH n.e.c.

(iii) Other revenues from corporations n.e.c.

(iv) Other revenues from households n.e.c.

(v) Social insurance contributions

(vi) Transfers from government domestic revenue (allocated to health purposes)

(vii) Gross Domestic Product

(viii) Voluntary prepayment

To ensure we were using the best possible data, we downloaded the metadata for each data point for all of the indicators from the GHED website. We used the metadata to decide how each given data point should be weighted, from 1 to 5, being applied as inverse variance weights. We established guidelines for the metadata that informed how the underlying data points should be weighted, giving priority to factors such as complete, documented source information and penalizing factors such as having been derived or estimated. We adjusted these data by converting them from current local currency to 2018 US$. Details of the weighting guidelines and classification are explained in the supplementary appendix our retrospective analysis paper.

Once we have an incomplete set of data points for the health expenditure variables we are interested in forecasting, we used Spatiotemporal Gaussian process regression (ST-GPR) to model the full time-series for each variable across a total of 195 countries. ST-GPR is a stochastic modeling technique that is designed to detect signals amidst noisy data. Unlike classical linear models that assume that the trend underlying data follows a definitive functional form, GPR assumes that the specific trend of interest follows a Gaussian Process, existing with some pointwise mean and covariance function. [1]. The covariates that were used in order to determine the initial fit of our health expenditure variables are: lag-distributed income, all-sector government expenditure per capita, healthcare access and

quality index [2], and proportion of total population over the age of 64; using a Matérn covariance function for the distribution of the Gaussian process. Once we determined an initial prediction of our dependent variables, using the variability of data across regions, Gaussian process regressions (GPR) were run in order to estimate 1,000 draws of each country-year estimate per metric.

1.4 United Nations World Population Prospects 2017

The United Nations (UN) World Population Prospects (WPP) provides population estimates and forecasts by age, sex, country, and year from 1950 until 2100. Using a cohort-component approach, WPP utilizes life tables to generate forecasts of age-specific mortality rates. Their modeling strategy involves a hierarchical Bayesian model (with an AR(1) process) of female life expectancy (with male life expectancy being highly correlated with female's) that prioritizes country data if available, but otherwise draws on regional data [3, 4]. A separate step models the male-female difference in life expectancy. From their final data source, we generate multiple indicators of demographic context, such as the total fertility rate (TFR), the proportion of the population under the age of 20, the proportion aged 65 years and over, and the total population for each country and year.

For a certain set of countries where either age-sex specific population or the TFR data were missing, we used a combination of a secondary data source (the US Census Bureau [5], which spanned from 2000 through 2050) and the global burden of disease (GBD) region specific rate of change to fill in and extrapolate, therefore giving us complete country-year time series for TFR and age-sex specific population spanning from 1950 through 2099 (2099 being the final year of TFR data available for other countries with existing data points).

1.5 WB, IMF, PWT, and Maddison

The WDI Database provides data on a wide range of development related variables, including data on GDP and GDP per capita. Data series in this database begin in 1960. The IMF's WEO Database provides data on various macroeconomic indicators. Macroeconomic series data are available from 1980 to present. The PWT is a database that provides real national accounts data for 167 countries and territories. The data series starts in 1950. The Maddison Project database provides historical GDP, GDP per capita, and population data dating as far back as Roman times. We utilized GDP per capita as a primary covariate to produce forecasts. GDP per capita from 1950 through 2017 was constructed using the method described in *James et al* [6] . The method utilized extracted data from a number of sources (IMF, WB, PWT and Maddison), and used multiple random effects models to

estimate a mean GDP per capita series to be used in our analysis. Similarly, we used the same methodology to produce a mean general government expenditure (GGE) per GDP series, from 1980 through 2017.

In order to forecast GDP per capita, we first convert GDP per capita to GDP per working population in the following manner:

$$\text{GDP}_{\text{working population}} = \text{GDP}_{\text{total population}} \times \frac{\text{Working population (20-64 years old)}}{\text{Total Population}}$$

We forecast GDP per working population from 2018-2050 and convert back to GDP per capita for further analysis. The out-of-sample root-mean-squared-error (RMSE) based on using GDP per working population was smaller than forecasting with GDP per capita.

2 Covariates

2.1 List of Forecasted Variables

The following are the list of variables which are forecasted in the manuscript:

- GDP: Gross Domestic Product (national income of a country)
- GGE: General Government Expenditure (all sector government expenditure in a country)
- DAH_d: Development Assistance for Health donated
- DAH_r: Development Assistance for Health received
- GHE: Government Health Expenditure
- OOP: Out-of-pocket Private Expenditure
- PPP: Prepaid Private Expenditure
- THE: Total Health Expenditure

2.2 Covariates Used for Forecasting

The following covariates are used as predictors, or independent variables, in our models:

- Total population of a country (1950 - 2050)
- Proportion of total population below the age of 15 (1950 - 2050)
- Proportion of total population above the age of 64 (1950 - 2050)
- Total Fertility Rate (1950 - 2050)
- Education attainment per capita [7, 8] (1980 - 2050)
- An indicator variable used to denote the anomalous disbursement of DAH in our DAH_d forecasts (1 for years in 2000 - 2010, 0 otherwise)
- An indicator variable used to denote the Ebola crisis in Guinea, Sierra Leone and Liberia in our DAH_r forecasts (1 for years in 2014 and 2015 for the three aforementioned countries, 0 otherwise).

Additionally, we also use the forecasted GDP and GGE per capita as covariates to predict the health expenditure variables. Section A.1 in the Tables and Figures section shows our full pathway for forecasting all our endogenous variables and how each of those variable fed into a succeeding model.

3 Ensemble Modeling

The purpose of ensemble modeling is to make sure that we capture the most out of what we have in our arsenal in terms of covariates and model specifications. We are agnostic about one model being the sole predictor of the future, and allow an ensemble of beliefs about predicting off of the past trends. 'Ensembling', in simple terms, is a way of pooling a number of sub-models, where the space of sub-models span different inclusions and combinations of predictors, and/or different econometric specifications.

3.1 Sub-model Setup

Our basic sub-model is a linear mixed effect model of the following form, for country i and time t:

$$\Delta_t Y_{i,t} = (\alpha + \alpha_i) + \left(\sum_{p=1}^{3} \rho_p \Delta_t Y_{i,t-p} \right) + \Delta_t X' \beta + \varepsilon_{i,t} \tag{1}$$

where

$$\left(\alpha, \vec{\beta}\right) \qquad \text{(Fixed effects)} \tag{2}$$

$$\alpha_i \sim N(0, \sigma_a^2) \qquad \text{(Country specific random intercept)} \tag{3}$$

$$\varepsilon_{i,t} \sim N\left(\sum_{m=1}^{3} \phi_m \varepsilon_{i,t-m}, \sigma_i^2 \right) \qquad \text{(Autocorrelated residuals as time random effects)} \tag{4}$$

'Fixed effect' is the equivalent of a non-random coefficient in a linear regression setting, and is estimated globally across all countries and time periods. The likelihood function for the data were all set to be Gaussian distributions for all of our metrics and models.

3.2 Covariates

Using our linear baseline model as defined in section 3.1, we created our set of ensemble sub-models by using all combinations of each of the covariates in Table A.2. For example, if we were predicting GDP per capita with TFR and Population as the predictors, then we would get a possible combination of four specifications to use (including one with no fixed effects). All covariates were included as fixed effects, and all specifications had the random effect on country intercepts.

3.3 Convergence to Global Growth Rates

We believe that the very-long-term trajectory of a variable forecasted for each country will gradually taper to the global growth rates. The way that we implemented this growth rate convergence is in the following manner:

1) Suppose that we are forecasting $Y_{i,t}$, which includes an intercept at the global level and the country level. Let us also assume that there were $\widetilde{T}$ time periods of Y observed in the past for each country i.

2) We start the tapering of the growth rate to start from 10 years before $\widetilde{T}$ into the future starting from the present, converging to the global value 10 years after $\widetilde{T}$. Therefore, if the present time period is H, then our decyaing scheme will start off from the year $h + \widetilde{T} - 10$ and end at $h + \widetilde{T} + 10$.

3) Our main objective is to slowly taper all the countries' random intercepts to decay to zero, so that they are all following a global intercept. In a first-difference model, since the intercepts represent the linear time trend, this scheme will slowly converge the linear time trends of all countries to decay to the global time trend.

4) Starting from the first year of decay, we will transform the country specific intercepts as such:

$$\hat{\alpha}_i = (\hat{\alpha}_i) \times \frac{20 - t}{20}$$

where t are all the time periods between $h + \widetilde{T} - 10$ and $h + \widetilde{T} + 10$.

5) By the time t reaches $h + \widetilde{T} + 10$, the value of $\hat{\alpha}_i$ will have decayed to zero, and therefore, all the countries will have reached a global linear time trend estimate.

3.4 Specifications

1) **ARIMA (Autoregressive Integrated Moving Average) terms**: We allowed up to three degrees of lags in the model (traditional auto-regressive terms), where each degree of AR term will include itself and all other lower degrees of lags. For example, a GDP per capita model with AR(3) specification (predicting log of GDP per capita) will include once, twice and thrice lagged log GDP per capita term. These were included as fixed effects.

   Additionally, in order to predict the best set of fixed effect coefficients, we test and include auto-correlated residuals in our models (traditional moving-average terms in an ARIMA setup). This basically means that we allow our models to estimate the residuals with

an autoregressive process of their own. These were included as random effects, and we allowed this to exist at the country-year level.

All of the variables we forecasted used the first-differences transformation (across time) as the dependent variable, except DAH received which was forecasted in non-differenced space, because of the presence of very high noise, thereby making the differenced series very unstable to forecast.

2) **Recency weights**: One of the other specifications we included in our sub-models was the option of weighting the recent years higher. This is particularly helpful for countries like Ethiopia and Nigeria, where they had rapid economic growth in recent years, and we believe that is a better predictor of the GDP forecasts than the further past. The weighting function was defined as such:

$$Weight = f(\tau, t) = (T - t + 1)^{\tau} \tag{5}$$

where T is the final year of in-sample data we have, and t is the year at that data point. This is an exponential decaying weighting function, where the degree of decaying is determined by τ, and we test and include a set of values of τ ranging between 0 and 0.5, where $\tau = 0$ refers to equal weights (all time periods are weighed equally at 100). Given that $\left.\frac{df(\tau, t)}{dt}\right|_{\tau>0} < 0$, this allows to weight our data with higher contributions to the likelihood from the recent past, and slowly decaying as we move further into the past towards the first observed time period. These recency weights were multiplied with the pointwise data variance parameter in order to weight the data points.

3) **Convergence Term**: We also allowed for the inclusion of a 'convergence term' in the list of sub-models. A convergence term is the one-year lag of the non-differenced dependent variable, and gets updated as each year is forecasted in the future. If a convergence term was considered in a sub-model, then we only included that sub-model if the coefficient on the convergence term was estimated to be negative (and statistically significant at 10% level).

4 Package and Architecture

4.1 Architecture

All analysis and forecasting were done on a parallel computing cluster with 20,000 nodes with a CentOS interface. We compiled R[9] version 3.5.1 from source code on a Docker based on Debian OS, which was deployed as a Singularity container with all the necessary compilers and binaries (GCC, G++ and Fortran 8.2.0).

4.2 Template Model Model

We used the R library **TMB**[10] to run our baseline mixed effects models. TMB stands for Template Model Builder, and it's a powerful method of approximating the integral of the Gaussian probability distribution function (which doesn't exist in closed form) by using a Laplace Approximation, which gives very precise results, and is relatively faster than other approximation packages which exist like Stan or Bugs. TMB uses automatic differentiation on the negative log-likelihood function in order to optimize the model parameters, and also returns a sparse joint precision matrix, from which we can simulate posterior parameter draws.

5 Inclusion and Exclusion Criteria

After we ran all possible combination of our sub-models and created a mean set of forecasts, we only want to keep the best possible set of sub-models. Hence, we implied the following set of inclusion and exclusion criteria in order to filter out the 'unrealistic' sub-models:

(1) All of the estimated coefficients must be **statistically significant at 10% level of significance**. For the fixed effects, we took the mean and the standard deviation of the posterior estimates, and filtered out the sub-model if the absolute z-score is below 1.645 (the absolute value of the one-sided 95th quantile of a standard normal distribution).

    For the random effects, we look at the whether the measure of variance is statistically significant or not. The model outputs the mean and standard deviation of the *precision of the random effects*, and therefore allowed us to exclude the specification if the precisions are not statistically significant at 10% level.

(2) If there were any estimated coefficient that **defied a prior belief we have on the direction of the value**, then we dropped that sub-model from consideration. For example, we strongly believe that as a donor country's (high income countries) income (GDP per capita) grows, they will be able to donate more DAH to lower income countries, and so, if we ran a sub-model predicting DAH_d and get a negative coefficient on GDP per capita, we dropped that sub-model from consideration.

    Our prior beliefs on the covariates for each dependent variable are listed in Table A.2.

(3) The forecasted trajectory growth **must not exceed observed growth rates**. We believe that a country will not grow faster than how much it has grown in the past trend. In order to come up with the bounds, we run a stochastic frontier analysis (SFA) of the change in the predicted variable against the level value of the predicted variable. SFA is just like an ordinary least squares specification, except with the addition of an additional 'inefficiency' term with a half-normal distribution. This allows us to estimate (for example, for GDP per capita): conditional of a country's income, how much growth rate did we see in the country's GDP. We ran this analysis across all of the observed data points, and derived a relationship binding the growth rates of GDP against the absolute values of GDP.

6 Creating the Forecasts

6.1 Ranking Sub-Models

In order to find out with sub-models would be able to predict a country's future the best, we ran out-of-sample predictive validity (OOS-PV) tests[11]. Simply put: we took each sub-model that passed the criteria in section 5, and instead of running it on all of the past data, we left out some number of recent most years. Following that, we ran the sub-model on the truncated past, and forecasted those years left out. For example, our GDP data extends from 1970 through 2017; we left out 15 years of data, and reran a sub-model from 1970 - 2007, and use the results of that sub-model to forecast GDP for the out-of-sample years (2003 - 2017).

This gave us essentially two trajectories between 2003 through 2017: the truth and the out-of-sample predictions. For each year, we computed the squared error (the difference) between these two lines, and averaged these errors for each of the neighboring years. So for example, we had squared errors for 15 data points between 2003 and 2017, and so the first mean squared error sum was just the squared error at 2003 and 2004, the second was the mean of the sum of squared errors for 2003, 2004 and 2005, and so on. We then took the square root of this new series to get the running root mean squared errors (RMSE) for a given country.

We looked at a country's 2003 RMSE values for each sub-models ran, and listed out the best 10% of the sub-models (that is, the lowest 10% RMSE values), and we did so for every year out-of-sample. For a single country, we may potentially have completely different set of sub-models for each of the OOS years. Then, for the 10% of the sub-models selected in the *first* year OOS, we only used those models to predict the *first* year of forecast for each country; the set of 10% of the sub-models selected in the *second* year OOS were used to predict the *second* year of forecast for each country, and so on, until the last year OOS model selections are used to compute the forecast the remaining years. This allowed us to narrow down every country's trajectory with the best performing OOS-PV sub-models for each year.

6.2 Uncertainty Estimation

To estimate the uncertainty intervals (UI), we reran the selected, ranked sub-models from section 6.1 and simulated draws instead of just getting a mean estimate of the future. There are four types of uncertainty we implemented in forecasting:

(i) **Model Uncertainty** : This type of uncertainty comes from having more than one

type of specification to create forecasts, and therefore we included a set of sub-models in the ensemble to incorporate for this uncertainty (which are ranked within each country-year).

(ii) **Data Uncertainty** : If our covariates themselves had draws of the future data (for example, when we forecasted GDP as a covariate to forecast PPP), then we picked randomly from the draws of the independent variable when predicting a sub-model's trajectory, if that covariate was included.

(iii) **Parameter Uncertainty** : This type of uncertainty is due to the variance for the posterior distributions. Once we have run a sub-model, we simulated from each of the estimated posterior distribution to create a set of simulated coefficients. This is done by simulating from the joint precision matrix of all the parameters estimated, and therefore produces a correlated set of coefficients.

(iv) **Fundamental Uncertainty** : The in-sample data and the fitted line in the past will never line up perfectly: there will always be errors from the model fit. We needed to reflect this level of uncertainty in our forecasts as well. By extracting these empirical residuals produced by a sub-model, we forecasted future country-specific residuals by using a random walk process, where the variance of the process is the variance of the residuals from the model fit σ_{ε}^2:

$$\hat{\varepsilon}_{i,t} \sim N(\varepsilon_{i,\hat{t}-1}, \sigma_{\varepsilon,t}^2) \tag{6}$$

A random walk is an AR(1) process with the coefficient equal to 1: in other words, the current value is independent of last year's value (except for the starting position), and will propagate forward with a random Gaussian noise of $\sigma_{\varepsilon,t}^2$ variance.

All of the above were used to simulate 1,000 forecasts (or draws), and so in order to construct our UIs, we took the mean and the values of the 2.5th and 97.5th quantiles to estimate 95% UIs.

Table B.1 shows all of the retrospective and prospective estimates of our health spending outcomes in 2018 USD and 2018 PPP in per capita space.

7 Ad-hoc Draws Correlation

7.1 Motivation

Given our current setup of compiling draws for each single year, there is no way of enforcing a temporal correlation across the draws right from the ensemble architecture. For example, the sub-models used in the first 15 years of GDP per capita forecasts were independently constructed and only depended on OOS-PV fits, while the 10th year (2032) onwards all draw from the same set of sub-models. This section details on the method used to generate the same correlation in the first 10 years of that example, drawing from the existing correlation from the 10th year onwards.

7.2 Bivariate Correlated Distributions

Using the GDP per capita example: following from uncertainty estimation, once we generated approximately 1,000 forecast draws for a country and year for any of the covariates, we used the following strategy to achieve consistent temporal correlation across all time periods in the future:

(a) For each country, we recorded country-specific Spearman's correlation coefficient across all draws between 2032 and 2050, which gave us a country-specific correlation vector.

(b) For each value of correlation in step (a), we simulated a bivariate uniform distribution for each country and year (2018 through 2032). This simulated distribution was ranked in such a way that the marginal distributions in the joint distribution were correlated with that value of correlation coefficient we supplied (this joint distribution is known as a *copula*).

(c) We recorded the ranks of the copula, and sorted our draws (within each country) using those temporal ranks, and therefore we ended up with a complete time-series data for all draws, such that each country and year will follow the same rank correlation structure that exists between 2032 and 2050.

(d) Finally, we calculated our final set of uncertainty intervals by taking the 2.5th and 97.5th percentiles of these correlated draws.

We used this method at the end of forecasting every metric, since one metric fed into the other sequentially.

8 Future Health Scenarios

We established the trajectories that our health expenditures are expected to take in the next 33 years using our ensemble models (from hereby referred to as the 'reference' case). The reference forecasts were built upon the basis of each country's past trends and expected future trends from covariates. We additionally also predicted what the possible trajectories for each country would look like if they were to follow the possible optimistic and pessimistic growth rates observed globally (referred to as 'better' and 'worse' cases, respectively).

8.1 Long-term Growth Regressions

In order to determine what the possible better and worse growth rates for each country would be, we ran long-term growth regressions with the following specification:

$$Y_{i,T} - Y_{i,t} = \alpha + \beta Y_{i.t} + \epsilon_{i,t} \tag{7}$$

where the dependent variable represented the long term growth rate of Y for country i, which was computed either as logarithmic or logistic growth rates (for fractions).

The only independent variable we used ($Y_{i,t}$) was the value of Y_i at time t, and it served as a convergence term in this regression. This allowed us to predict the long-term growth rates of Y, conditional on a country's level of Y at time t.

8.2 Forecasting Better and Worse Scenarios

In order to estimate what the future better and worse trajectories would be for each country, we followed these steps (assuming that we are forecasting from 2017 through 2050, with observed data between 1995 and 2016):

(i) We computed the 85th and 15th percentiles of the empirical residuals ϵ, as $Q_{0.85}(\hat{\epsilon_{i,t}})$ and $Q_{0.15}(\hat{\epsilon_{i,t}})$ respectively, where $Q_p(\cdot)$ is a quantile function for a percentile p.

(ii) We computed the starting annualized growth rate from the fitted scenario regression, such that, for country i:

$$\text{Better growth rate} = \exp(\hat{\alpha}) \times \exp(Q_{0.85}(\hat{\epsilon_{i,t}})) \times (Y_{i,2016}^{\hat{\beta}})^{(1/(2016-1995))} \tag{8}$$

$$\text{Worse growth rate} = \exp(\hat{\alpha}) \times \exp(Q_{0.15}(\hat{\epsilon_{i,t}})) \times (Y_{i,2016}^{\hat{\beta}})^{(1/(2016-1995))} \tag{9}$$

(iii) Finally, once we have established the growth rates as a function of the convergence term, we recursively created better and worse trajectories, conditional on the updated

growth rates every year, such that:

$$Y_{i,t+1} = Y_{i,t} \times \exp(\hat{\alpha}) \times \exp(Q_{0.85}(\hat{\epsilon_{i,t}})) \times (Y_{i,t}^{\hat{\beta}}) \tag{10}$$

where $\exp(\hat{\alpha}) \times \exp(Q_{0.85}(\hat{\epsilon_{i,t}})) \times (Y_{i,t}^{\hat{\beta}})$ was the conditional growth rate for a single year.

One condition that we imposed for the computed scenarios is: the better projection cannot be lower than the reference projection, and the worse projection cannot be higher than the reference projection. For countries with wide forecasts where this case did happen, we moved the better and worse forecasts down and up to overlap on top of the reference line respectively.

8.3 Uncertainty Estimation

The uncertainty intervals around a scenario were expected to take the same shape as the uncertainty around our reference forecasts. Therefore, once we have propagated a mean set of better and worse forecasts in section 8.2, we created the draws around our scenarios in the following way:

(i) We took our reference forecast's mean line and the 1,000 draws around that line.

(ii) We computed the deviation of the mean from each of the draws (in logarithmic or logistic transformation, depending on the space of the covariate).

(iii) We took each of the scenario mean lines and added the deviations from the previous step to the mean lines, giving us 1,000 draws of the scenario projections.

Figure set B.2 visualizes the future health scenarios of our health outcomes in per capita space (in 2018 USD).

A Tables and Figures

Table 1: Definitions of health expenditure sources

Health Expenditure Type	Definition
Development assistance for health	Financial and in-kind contributions from global health channels that aim to improve or maintain health in low- or middle-income countries.
Government health expenditure as source	Government health expenditure as source only includes domestically financed government expenditure on health, including public health spending.
Out-of-pocket expenditure	Paid by individuals for health services; considered catastrophic if exceeding 40% of a household's annual income.
Prepaid private health expenditure	Private risk pooling against catastrophic health expenditure; includes private insurance and non-governmental organizations.

A.1 State Space Diagram of Forecasting Components

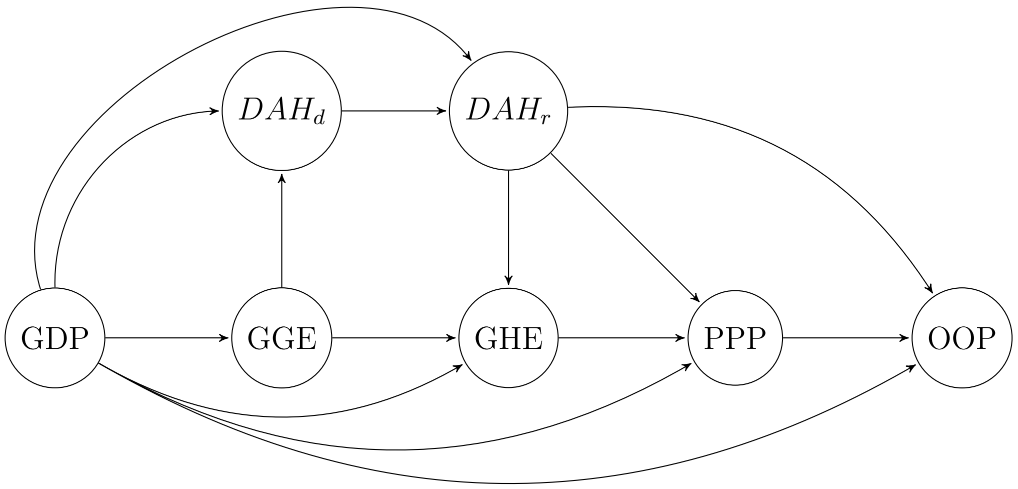

A.2 Prediction and Covariates Map with Transformations

	Predicted Variables	Covariates	Extra Specifications
1	GDP per labour (both in log)		ARIMA(2-3, 1, 0)
2	GGE per GDP (logit)	$GDPpc^{+}$, Pop $>64^{+}$, $Education^{+}$	ARIMA(0-3, 1, 0-3)
3	DAH donated per GGE (logit)	$GDPpc^{+}$, GGE/GDP^{+}, Bush Era $Dummy^{+}$	ARIMA(0-3, 1, 0-3)
4	DAH received per total DAH donated (logit)	$GDPpc^{-}$, Pop^{-}, Pop $<15^{+}$, TFR^{+}, Ebola $dummy^{+}$, total DAH envelope	ARIMA(0-3, 0, 0-3)
5	GHE per GGE (logit)	$GDPpc^{+}$, Pop $>64^{+}$, GGE/GDP^{+}, DAH/GDP^{-}	ARIMA(0-3, 1, 0-3)
6	PPP per GDP (logit)	$GDPpc^{+}$, Pop $>64^{+}$, GGE/GDP. DAH/GDP, GHE/GDP,	ARIMA(0-3, 1, 0-3)
7	OOP per GDP (logit)	$GDPpc^{-}$, Pop $>64^{+}$, GGE/GDP, DAH/GDP, GHE/GDP, PPP/GDP	ARIMA(0-3, 1, 0-3)

References

[1] Emmanuela Gakidou, Ashkan Afshin, Amanuel Alemu Abajobir, Kalkidan Hassen Abate, Abbafati, Ben Zipkin, Sanjay Zodpey, Stephen S Lim, and Christopher J L Murray. Global, regional, and national comparative risk assessment of 84 behavioural, environmental and occupational, and metabolic risks or clusters of risks, 1990-2016: a systematic analysis for the Global Burden of Disease Study 2016. *The Lancet*, 390 (10100):1345–1422, nov 2017. ISSN 0140-6736. doi: 10.1016/S0140-6736(17)32366-8. URL `http://dx.doi.org/10.1016/S0140-6736(17)32366-8`.

[2] Measuring progress and projecting attainment on the basis of past trends of the health-related sustainable development goals in 188 countries: an analysis from the global burden of disease study 2016. *The Lancet*, 390(10100):1423 – 1459, 2017. ISSN 0140-6736. doi: https://doi.org/10.1016/S0140-6736(17)32336-X. URL `http://www.sciencedirect.com/science/article/pii/S014067361732336X`.

[3] Adrian E Raftery, Leontine Alkema, and Patrick Gerland. Bayesian population projections for the united nations. *Statistical science: a review journal of the Institute of Mathematical Statistics*, 29(1):58, 2014.

[4] United Nations. United nations, department of economic and social affairs, population division (2017). world population prospects: The 2017 revision, methodology of the united nations population estimates and projections. *Working Paper No. ESA/P/WP.250*, 2017.

[5] US census bureau. `https://www.census.gov/topics/population.html`. Accessed: 2017-12-20.

[6] Spencer L James, Paul Gubbins, Christopher JL Murray, and Emmanuela Gakidou. Developing a comprehensive time series of gdp per capita for 210 countries from 1950 to 2015. *Population health metrics*, 10(1):12, 2012.

[7] Emmanuela Gakidou, Krycia Cowling, Rafael Lozano, and Christopher JL Murray. Increased educational attainment and its effect on child mortality in 175 countries between 1970 and 2009: a systematic analysis. *The Lancet*, 376(9745):959–974, 2010.

[8] Kyle J Foreman, Neal Marquez, Andrew Dolgert, Kai Fukutaki, Nancy Fullman, Madeline McGaughey, Martin A Pletcher, Amanda E Smith, Kendrick Tang, Chun-Wei Yuan,

et al. Forecasting life expectancy, years of life lost, and all-cause and cause-specific mortality for 250 causes of death: reference and alternative scenarios for 2016–40 for 195 countries and territories. *The Lancet*, 392(10159):2052–2090, 2018.

[9] R Core Team. *R: A Language and Environment for Statistical Computing*. R Foundation for Statistical Computing, Vienna, Austria, 2017. URL `https://www.R-project.org/`. Accessed: 2017-12-20.

[10] Kasper Kristensen, Anders Nielsen, Casper W Berg, Hans Skaug, and Brad Bell. Tmb: automatic differentiation and laplace approximation. *arXiv preprint arXiv:1509.00660*, 2015.

[11] Kyle J Foreman, Rafael Lozano, Alan D Lopez, and Christopher JL Murray. Modeling causes of death: an integrated approach using codem. *Population health metrics*, 10(1): 1, 2012.

B Additional Tables and Figures

The following are the list of additional tables and figures containing our data and results from our analysis.

B.1 Table: Health Spending from 1995-2050

This table shows all of our health spending outcomes for every country and year from 1995 through 2050. The variables are in per capita measures, and for currencies 2018 USD and 2018 purchasing-power-parity.

B.2 Table: Health spending by source and alternative future scenarios of government health spending, 2050

B.1 Table

		2018 US Dollars per capita					2018 PPP per capita				
Country	Year	Health spending	Government health spending	Out-of-pocket health spending	Prepaid private health spending	Development assistance for health	Health spending	Government health spending	Out-of-pocket health spending	Prepaid private health spending	Development assistance for health
Afghanistan	1995	27.7 (19.6 to 37.8)	0.9 (0.7 to 1.2)	26.5 (18.5 to 36.6)	0.1 (0.0 to 0.1)	0.3 (0.3 to 0.3)	99.6 (70.5 to 135.5)	3.3 (2.5 to 4.2)	95.1 (66.3 to 131.4)	0.2 (0.1 to 0.4)	1.0 (1.0 to 1.0)
Afghanistan	1996	27.1 (19.5 to 36.2)	0.8 (0.7 to 1.1)	25.8 (18.2 to 35.0)	0.1 (0.0 to 0.1)	0.3 (0.3 to 0.3)	97.2 (70.1 to 129.9)	3.0 (2.3 to 4.0)	92.7 (65.4 to 125.4)	0.2 (0.1 to 0.4)	1.2 (1.2 to 1.2)
Afghanistan	1997	26.9 (19.5 to 35.9)	0.8 (0.6 to 1.0)	25.4 (18.2 to 34.4)	0.0 (0.0 to 0.1)	0.6 (0.6 to 0.6)	96.3 (69.9 to 129.0)	2.8 (2.2 to 3.7)	91.2 (65.2 to 123.6)	0.2 (0.1 to 0.3)	2.2 (2.2 to 2.2)
Afghanistan	1998	26.1 (18.8 to 35.3)	0.7 (0.5 to 0.9)	25.0 (17.9 to 34.2)	0.0 (0.0 to 0.1)	0.3 (0.3 to 0.3)	93.5 (67.5 to 126.5)	2.6 (2.0 to 3.4)	89.7 (64.2 to 122.7)	0.2 (0.1 to 0.3)	1.0 (1.0 to 1.0)
Afghanistan	1999	25.6 (18.5 to 34.2)	0.7 (0.5 to 0.9)	24.4 (17.3 to 32.8)	0.0 (0.0 to 0.1)	0.5 (0.5 to 0.5)	91.9 (66.6 to 122.7)	2.4 (1.8 to 3.1)	87.5 (62.1 to 117.9)	0.2 (0.1 to 0.3)	1.7 (1.7 to 1.7)
Afghanistan	2000	24.9 (17.8 to 33.4)	0.6 (0.5 to 0.8)	23.7 (16.7 to 32.2)	0.0 (0.0 to 0.1)	0.5 (0.5 to 0.5)	89.3 (63.8 to 120.0)	2.3 (1.8 to 3.0)	85.2 (59.8 to 115.4)	0.2 (0.1 to 0.3)	1.7 (1.7 to 1.7)
Afghanistan	2001	24.1 (17.4 to 31.6)	0.6 (0.5 to 0.8)	22.8 (16.1 to 30.2)	0.0 (0.0 to 0.1)	0.7 (0.7 to 0.7)	86.6 (62.5 to 113.5)	2.2 (1.6 to 2.8)	81.6 (57.7 to 108.4)	0.2 (0.1 to 0.3)	2.7 (2.7 to 2.7)
Afghanistan	2002	28.8 (21.2 to 37.8)	0.8 (0.6 to 1.0)	26.5 (18.8 to 35.5)	0.1 (0.0 to 0.1)	1.5 (1.5 to 1.5)	103.4 (76.0 to 135.5)	2.8 (2.1 to 3.7)	95.0 (67.5 to 127.4)	0.2 (0.1 to 0.4)	5.3 (5.3 to 5.3)
Afghanistan	2003	34.0 (25.7 to 44.0)	1.5 (1.1 to 1.9)	30.3 (22.0 to 40.4)	0.1 (0.0 to 0.2)	2.1 (2.1 to 2.1)	121.9 (92.2 to 157.9)	5.3 (4.0 to 6.9)	108.8 (79.0 to 144.9)	0.3 (0.2 to 0.6)	7.5 (7.5 to 7.5)
Afghanistan	2004	37.8 (29.3 to 47.7)	1.7 (1.3 to 2.2)	30.7 (22.2 to 40.5)	0.1 (0.0 to 0.2)	5.3 (5.3 to 5.3)	135.7 (105.2 to 171.1)	6.2 (4.6 to 8.0)	110.3 (79.8 to 145.4)	0.4 (0.2 to 0.7)	18.9 (18.9 to 18.9)
Afghanistan	2005	40.3 (31.5 to 50.0)	2.0 (1.5 to 2.5)	31.7 (23.0 to 41.6)	0.1 (0.1 to 0.2)	6.5 (6.5 to 6.5)	144.5 (113.1 to 179.2)	7.0 (5.3 to 9.1)	113.9 (82.7 to 149.2)	0.4 (0.2 to 0.8)	23.2 (23.2 to 23.2)
Afghanistan	2006	40.6 (32.1 to 50.1)	2.1 (1.6 to 2.7)	32.2 (23.7 to 41.7)	0.1 (0.1 to 0.2)	6.2 (6.2 to 6.2)	145.6 (115.1 to 179.8)	7.4 (5.6 to 9.6)	115.6 (85.0 to 149.5)	0.4 (0.2 to 0.8)	22.3 (22.3 to 22.3)
Afghanistan	2007	43.4 (34.9 to 53.0)	2.3 (1.8 to 3.0)	33.4 (24.8 to 43.0)	0.1 (0.1 to 0.2)	7.5 (7.5 to 7.5)	155.8 (125.1 to 190.2)	8.3 (6.3 to 10.6)	119.9 (89.1 to 154.4)	0.4 (0.2 to 0.9)	27.1 (27.1 to 27.1)
Afghanistan	2008	44.6 (36.3 to 54.2)	2.5 (1.8 to 3.1)	34.6 (26.2 to 44.3)	0.1 (0.1 to 0.3)	7.4 (7.4 to 7.4)	160.0 (130.4 to 194.3)	8.8 (6.6 to 11.2)	124.0 (94.2 to 158.8)	0.5 (0.2 to 0.9)	26.7 (26.7 to 26.7)
Afghanistan	2009	49.7 (40.7 to 59.9)	2.7 (2.1 to 3.5)	37.1 (28.1 to 47.3)	0.1 (0.1 to 0.3)	9.7 (9.7 to 9.7)	178.3 (146.0 to 214.8)	9.7 (7.4 to 12.5)	133.2 (100.8 to 169.9)	0.5 (0.2 to 1.0)	34.9 (34.9 to 34.9)
Afghanistan	2010	50.7 (41.5 to 61.0)	2.7 (2.0 to 3.4)	37.2 (28.1 to 47.6)	0.1 (0.1 to 0.3)	10.7 (10.7 to 10.7)	181.9 (148.8 to 219.0)	9.6 (7.3 to 12.3)	133.4 (100.9 to 170.7)	0.5 (0.2 to 1.0)	38.4 (38.4 to 38.4)
Afghanistan	2011	52.7 (43.8 to 63.5)	2.6 (2.0 to 3.4)	36.8 (27.9 to 47.5)	0.1 (0.1 to 0.3)	13.1 (13.1 to 13.1)	189.0 (157.3 to 227.8)	9.4 (7.1 to 12.1)	132.1 (100.2 to 170.4)	0.5 (0.3 to 1.0)	47.0 (47.0 to 47.0)
Afghanistan	2012	51.9 (42.6 to 63.9)	2.7 (2.0 to 3.5)	38.4 (28.8 to 50.0)	0.2 (0.1 to 0.3)	10.7 (10.7 to 10.7)	186.3 (152.8 to 229.2)	9.6 (7.2 to 12.4)	137.6 (103.5 to 179.4)	0.5 (0.3 to 1.1)	38.6 (38.6 to 38.6)
Afghanistan	2013	54.4 (44.0 to 67.4)	2.8 (2.1 to 3.6)	40.6 (30.5 to 53.3)	0.2 (0.1 to 0.3)	10.9 (10.9 to 10.9)	195.2 (158.0 to 241.9)	10.1 (7.5 to 13.0)	145.5 (109.4 to 191.4)	0.6 (0.3 to 1.1)	39.0 (39.0 to 39.0)
Afghanistan	2014	56.6 (45.8 to 70.4)	3.0 (2.2 to 3.9)	43.5 (32.6 to 56.9)	0.2 (0.1 to 0.3)	10.0 (10.0 to 10.0)	203.2 (164.5 to 252.7)	10.6 (7.9 to 13.8)	156.0 (117.1 to 204.2)	0.6 (0.3 to 1.2)	36.0 (36.0 to 36.0)
Afghanistan	2015	58.8 (46.9 to 73.2)	3.1 (2.3 to 4.0)	46.1 (34.5 to 60.9)	0.2 (0.1 to 0.3)	9.4 (9.4 to 9.4)	210.9 (168.4 to 262.6)	11.0 (8.3 to 14.5)	165.5 (123.8 to 218.4)	0.7 (0.3 to 1.2)	33.6 (33.6 to 33.6)

		2018 US Dollars per capita					2018 PPP per capita				
**Country**	**Year**	**Health spending**	**Government health spending**	**Out-of-pocket health spending**	**Prepaid private health spending**	**Development assistance for health**	**Health spending**	**Government health spending**	**Out-of-pocket health spending**	**Prepaid private health spending**	**Development assistance for health**
Afghanistan	2016	55.8 (43.4 to 71.4)	3.1 (2.3 to 4.1)	47.2 (35.1 to 62.6)	0.2 (0.1 to 0.3)	5.3 (5.3 to 5.3)	200.2 (155.8 to 256.1)	11.2 (8.4 to 14.8)	169.2 (125.8 to 224.5)	0.7 (0.3 to 1.2)	19.1 (19.1 to 19.1)
Afghanistan	2017	55.6 (43.3 to 70.9)	3.0 (2.3 to 4.0)	47.4 (35.3 to 63.0)	0.2 (0.1 to 0.3)	5.0 (5.0 to 5.0)	199.6 (155.3 to 254.4)	10.9 (8.2 to 14.4)	170.1 (126.6 to 225.9)	0.7 (0.3 to 1.2)	17.9 (17.9 to 17.9)
Afghanistan	2018	56.0 (43.4 to 71.6)	3.1 (2.3 to 4.1)	48.1 (35.7 to 63.6)	0.2 (0.1 to 0.3)	4.6 (4.6 to 4.6)	200.9 (155.6 to 257.0)	11.2 (8.4 to 14.7)	172.6 (128.2 to 228.4)	0.7 (0.3 to 1.2)	16.5 (16.5 to 16.5)
Afghanistan	2019	56.7 (43.9 to 72.6)	3.2 (2.4 to 4.2)	48.8 (36.1 to 64.7)	0.2 (0.1 to 0.4)	4.5 (4.2 to 4.8)	203.6 (157.5 to 260.6)	11.4 (8.5 to 15.1)	175.2 (129.5 to 232.2)	0.7 (0.3 to 1.3)	16.3 (15.2 to 17.2)
Afghanistan	2020	57.6 (44.2 to 74.0)	3.2 (2.4 to 4.3)	49.6 (36.6 to 65.7)	0.2 (0.1 to 0.4)	4.5 (4.1 to 4.9)	206.5 (158.7 to 265.6)	11.6 (8.8 to 15.3)	178.0 (131.5 to 235.6)	0.7 (0.3 to 1.3)	16.1 (14.8 to 17.4)
Afghanistan	2021	58.4 (45.1 to 74.7)	3.3 (2.5 to 4.4)	50.5 (37.2 to 66.9)	0.2 (0.1 to 0.4)	4.5 (4.0 to 4.9)	209.7 (161.8 to 268.2)	11.9 (8.9 to 15.7)	181.1 (133.4 to 240.0)	0.7 (0.3 to 1.3)	16.0 (14.3 to 17.7)
Afghanistan	2022	59.4 (45.9 to 76.4)	3.4 (2.5 to 4.5)	51.4 (37.8 to 68.1)	0.2 (0.1 to 0.4)	4.4 (3.9 to 5.0)	213.2 (164.5 to 274.1)	12.2 (9.1 to 16.1)	184.3 (135.6 to 244.5)	0.8 (0.3 to 1.4)	15.9 (13.9 to 18.0)
Afghanistan	2023	60.5 (46.5 to 77.6)	3.5 (2.6 to 4.6)	52.4 (38.5 to 69.3)	0.2 (0.1 to 0.4)	4.4 (3.8 to 5.1)	216.9 (166.9 to 278.5)	12.5 (9.4 to 16.5)	187.9 (138.2 to 248.8)	0.8 (0.4 to 1.4)	15.7 (13.5 to 18.1)
Afghanistan	2024	61.6 (47.3 to 79.1)	3.6 (2.7 to 4.7)	53.4 (39.1 to 70.9)	0.2 (0.1 to 0.4)	4.4 (3.7 to 5.1)	221.1 (169.6 to 283.8)	12.9 (9.6 to 16.9)	191.8 (140.5 to 254.4)	0.8 (0.4 to 1.4)	15.6 (13.3 to 18.5)
Afghanistan	2025	62.9 (48.5 to 80.7)	3.7 (2.7 to 4.8)	54.7 (40.4 to 72.6)	0.2 (0.1 to 0.4)	4.3 (3.6 to 5.1)	225.7 (174.0 to 289.7)	13.2 (9.8 to 17.3)	196.1 (145.1 to 260.6)	0.8 (0.4 to 1.4)	15.6 (13.0 to 18.5)
Afghanistan	2026	64.2 (49.4 to 82.7)	3.8 (2.8 to 4.9)	55.9 (41.3 to 74.5)	0.2 (0.1 to 0.4)	4.3 (3.6 to 5.2)	230.4 (177.4 to 296.6)	13.5 (10.1 to 17.7)	200.6 (148.3 to 267.3)	0.8 (0.4 to 1.5)	15.6 (12.9 to 18.5)
Afghanistan	2027	65.6 (50.5 to 84.6)	3.9 (2.9 to 5.0)	57.2 (42.3 to 76.2)	0.2 (0.1 to 0.4)	4.3 (3.6 to 5.2)	235.3 (181.1 to 303.4)	13.8 (10.3 to 18.1)	205.1 (151.9 to 273.3)	0.8 (0.4 to 1.5)	15.5 (12.8 to 18.8)
Afghanistan	2028	67.0 (51.4 to 86.4)	3.9 (2.9 to 5.2)	58.4 (43.1 to 77.8)	0.2 (0.1 to 0.4)	4.3 (3.6 to 5.3)	240.3 (184.3 to 309.9)	14.1 (10.6 to 18.5)	209.7 (154.8 to 279.3)	0.9 (0.4 to 1.6)	15.6 (12.9 to 18.9)
Afghanistan	2029	68.3 (52.1 to 88.1)	4.0 (3.0 to 5.3)	59.7 (43.6 to 79.4)	0.2 (0.1 to 0.4)	4.4 (3.5 to 5.4)	245.2 (187.0 to 315.9)	14.5 (10.8 to 19.0)	214.2 (156.6 to 284.8)	0.9 (0.4 to 1.6)	15.6 (12.7 to 19.2)
Afghanistan	2030	69.7 (53.0 to 90.2)	4.1 (3.1 to 5.4)	60.9 (44.4 to 81.1)	0.3 (0.1 to 0.5)	4.4 (3.5 to 5.4)	250.0 (190.1 to 323.7)	14.8 (11.0 to 19.3)	218.6 (159.2 to 291.1)	0.9 (0.4 to 1.6)	15.7 (12.7 to 19.4)
Afghanistan	2031	71.0 (53.5 to 92.1)	4.2 (3.1 to 5.5)	62.2 (44.8 to 83.5)	0.3 (0.1 to 0.5)	4.4 (3.5 to 5.5)	254.9 (192.1 to 330.3)	15.1 (11.3 to 19.6)	223.2 (160.9 to 299.7)	0.9 (0.4 to 1.7)	15.7 (12.6 to 19.8)
Afghanistan	2032	72.4 (54.3 to 94.2)	4.3 (3.2 to 5.6)	63.5 (45.8 to 85.9)	0.3 (0.1 to 0.5)	4.4 (3.5 to 5.6)	259.8 (194.7 to 338.0)	15.4 (11.6 to 20.2)	227.7 (164.4 to 308.1)	0.9 (0.4 to 1.7)	15.8 (12.5 to 20.0)
Afghanistan	2033	73.8 (55.1 to 97.1)	4.4 (3.3 to 5.7)	64.7 (46.4 to 87.9)	0.3 (0.1 to 0.5)	4.4 (3.5 to 5.7)	264.7 (197.6 to 348.3)	15.7 (11.8 to 20.6)	232.2 (166.6 to 315.5)	1.0 (0.4 to 1.7)	15.9 (12.5 to 20.4)
Afghanistan	2034	75.2 (56.1 to 99.0)	4.5 (3.3 to 5.8)	66.0 (47.4 to 90.1)	0.3 (0.1 to 0.5)	4.5 (3.5 to 5.9)	269.8 (201.4 to 355.3)	16.0 (12.0 to 21.0)	236.8 (170.0 to 323.2)	1.0 (0.5 to 1.8)	16.0 (12.5 to 21.1)
Afghanistan	2035	76.6 (56.9 to 101.5)	4.6 (3.4 to 6.0)	67.3 (48.3 to 92.2)	0.3 (0.1 to 0.5)	4.5 (3.5 to 5.9)	275.0 (204.2 to 364.1)	16.4 (12.3 to 21.5)	241.5 (173.4 to 331.0)	1.0 (0.5 to 1.8)	16.1 (12.5 to 21.2)
Afghanistan	2036	78.1 (57.4 to 104.0)	4.6 (3.5 to 6.1)	68.6 (49.2 to 94.4)	0.3 (0.1 to 0.5)	4.5 (3.5 to 6.0)	280.1 (205.8 to 373.3)	16.7 (12.5 to 22.0)	246.1 (176.6 to 338.5)	1.0 (0.5 to 1.8)	16.3 (12.6 to 21.7)

		2018 US Dollars per capita					2018 PPP per capita				
Country	Year	Health spending	Government health spending	Out-of-pocket health spending	Prepaid private health spending	Development assistance for health	Health spending	Government health spending	Out-of-pocket health spending	Prepaid private health spending	Development assistance for health
Afghanistan	2037	79.5 (58.1 to 106.1)	4.7 (3.5 to 6.3)	69.9 (49.7 to 96.5)	0.3 (0.1 to 0.5)	4.6 (3.5 to 6.1)	285.1 (208.6 to 380.8)	17.0 (12.6 to 22.5)	250.6 (178.2 to 346.2)	1.0 (0.5 to 1.9)	16.4 (12.5 to 22.1)
Afghanistan	2038	80.9 (59.3 to 108.9)	4.8 (3.6 to 6.4)	71.2 (50.2 to 98.9)	0.3 (0.1 to 0.5)	4.6 (3.5 to 6.3)	290.3 (212.7 to 390.8)	17.3 (13.0 to 22.9)	255.3 (180.1 to 355.0)	1.1 (0.5 to 1.9)	16.5 (12.5 to 22.7)
Afghanistan	2039	82.3 (60.3 to 111.1)	4.9 (3.7 to 6.5)	72.5 (50.9 to 101.1)	0.3 (0.1 to 0.6)	4.6 (3.5 to 6.4)	295.4 (216.3 to 398.7)	17.7 (13.2 to 23.3)	260.0 (182.5 to 362.9)	1.1 (0.5 to 2.0)	16.7 (12.5 to 22.9)
Afghanistan	2040	83.8 (61.3 to 113.4)	5.0 (3.7 to 6.6)	73.8 (51.7 to 103.3)	0.3 (0.1 to 0.6)	4.7 (3.5 to 6.6)	300.6 (219.9 to 406.9)	18.0 (13.4 to 23.8)	264.6 (185.3 to 370.7)	1.1 (0.5 to 2.0)	16.8 (12.7 to 23.8)
Afghanistan	2041	85.2 (61.9 to 115.4)	5.1 (3.8 to 6.8)	75.0 (52.4 to 105.3)	0.3 (0.1 to 0.6)	4.7 (3.6 to 6.7)	305.6 (222.1 to 414.1)	18.3 (13.7 to 24.3)	269.0 (188.2 to 378.0)	1.1 (0.5 to 2.1)	17.0 (12.8 to 24.1)
Afghanistan	2042	86.6 (62.7 to 117.7)	5.2 (3.9 to 6.9)	76.2 (53.0 to 107.1)	0.3 (0.1 to 0.6)	4.8 (3.5 to 6.9)	310.7 (225.1 to 422.5)	18.7 (14.0 to 24.9)	273.6 (190.2 to 384.4)	1.1 (0.5 to 2.1)	17.2 (12.7 to 24.9)
Afghanistan	2043	88.0 (63.8 to 120.3)	5.3 (4.0 to 7.1)	77.5 (53.5 to 109.1)	0.3 (0.2 to 0.6)	4.9 (3.5 to 7.1)	315.9 (228.9 to 431.6)	19.1 (14.2 to 25.4)	278.2 (192.1 to 391.5)	1.2 (0.5 to 2.1)	17.4 (12.7 to 25.4)
Afghanistan	2044	89.4 (64.9 to 121.7)	5.4 (4.0 to 7.2)	78.8 (54.0 to 110.9)	0.3 (0.2 to 0.6)	4.9 (3.6 to 7.2)	321.0 (233.0 to 436.7)	19.4 (14.5 to 25.8)	282.7 (193.8 to 398.0)	1.2 (0.6 to 2.2)	17.7 (12.9 to 25.9)
Afghanistan	2045	90.9 (65.9 to 123.7)	5.5 (4.1 to 7.4)	80.1 (54.8 to 112.6)	0.3 (0.2 to 0.6)	5.0 (3.6 to 7.6)	326.1 (236.4 to 443.7)	19.8 (14.7 to 26.5)	287.3 (196.7 to 404.1)	1.2 (0.6 to 2.2)	17.9 (12.9 to 27.4)
Afghanistan	2046	92.4 (67.1 to 125.4)	5.6 (4.2 to 7.6)	81.4 (55.8 to 114.2)	0.3 (0.2 to 0.6)	5.1 (3.6 to 7.8)	331.6 (240.6 to 449.8)	20.2 (14.9 to 27.2)	292.1 (200.2 to 409.6)	1.2 (0.6 to 2.2)	18.1 (13.1 to 27.8)
Afghanistan	2047	93.9 (67.7 to 127.3)	5.7 (4.2 to 7.8)	82.6 (56.7 to 115.8)	0.4 (0.2 to 0.6)	5.1 (3.7 to 8.2)	336.8 (242.9 to 456.9)	20.6 (15.1 to 27.9)	296.6 (203.6 to 415.4)	1.3 (0.6 to 2.3)	18.4 (13.1 to 29.3)
Afghanistan	2048	95.4 (68.9 to 129.4)	5.9 (4.3 to 7.9)	84.0 (57.7 to 117.9)	0.4 (0.2 to 0.7)	5.2 (3.7 to 8.4)	342.3 (247.3 to 464.2)	21.0 (15.3 to 28.5)	301.3 (207.2 to 422.9)	1.3 (0.6 to 2.3)	18.7 (13.3 to 30.2)
Afghanistan	2049	96.9 (69.9 to 131.3)	6.0 (4.3 to 8.1)	85.3 (58.4 to 119.8)	0.4 (0.2 to 0.7)	5.3 (3.8 to 8.7)	347.7 (250.8 to 471.0)	21.4 (15.6 to 29.1)	306.0 (209.5 to 429.7)	1.3 (0.6 to 2.4)	19.0 (13.5 to 31.3)
Afghanistan	2050	98.4 (71.2 to 133.8)	6.1 (4.4 to 8.3)	86.6 (58.9 to 121.6)	0.4 (0.2 to 0.7)	5.4 (3.8 to 8.8)	353.1 (255.4 to 480.3)	21.8 (15.9 to 30.0)	310.6 (211.4 to 436.3)	1.3 (0.6 to 2.4)	19.3 (13.6 to 31.6)
Albania	1995	125.1 (106.9 to 145.2)	31.5 (25.2 to 39.3)	88.3 (71.9 to 108.5)	0.0 (0.0 to 0.0)	5.3 (5.3 to 5.3)	328.6 (280.8 to 381.4)	82.7 (66.1 to 103.4)	231.9 (188.9 to 285.1)	0.0 (0.0 to 0.1)	14.0 (14.0 to 14.0)
Albania	1996	138.2 (119.4 to 159.0)	35.5 (28.6 to 43.9)	98.0 (81.7 to 118.4)	0.0 (0.0 to 0.0)	4.7 (4.7 to 4.7)	363.2 (313.6 to 417.6)	93.3 (75.2 to 115.3)	257.4 (214.5 to 311.0)	0.0 (0.0 to 0.1)	12.5 (12.5 to 12.5)
Albania	1997	126.6 (111.3 to 144.8)	34.7 (28.1 to 42.4)	90.3 (76.5 to 106.9)	0.0 (0.0 to 0.0)	1.6 (1.6 to 1.6)	332.6 (292.4 to 380.3)	91.3 (73.9 to 111.4)	237.3 (200.8 to 280.9)	0.0 (0.0 to 0.1)	4.1 (4.1 to 4.1)
Albania	1998	136.2 (120.7 to 154.0)	39.5 (32.1 to 47.8)	89.5 (76.7 to 104.7)	0.0 (0.0 to 0.0)	7.2 (7.2 to 7.2)	357.8 (317.0 to 404.5)	103.7 (84.4 to 125.6)	235.1 (201.4 to 275.1)	0.0 (0.0 to 0.1)	19.0 (19.0 to 19.0)
Albania	1999	149.0 (133.6 to 167.8)	47.3 (38.6 to 57.2)	92.9 (79.9 to 109.0)	0.0 (0.0 to 0.0)	8.8 (8.8 to 8.8)	391.5 (350.8 to 440.8)	124.4 (101.3 to 150.2)	244.0 (210.0 to 286.3)	0.0 (0.0 to 0.1)	23.2 (23.2 to 23.2)
Albania	2000	153.0 (137.4 to 171.6)	51.0 (41.6 to 60.9)	94.1 (80.5 to 110.7)	0.0 (0.0 to 0.0)	7.9 (7.9 to 7.9)	401.9 (360.8 to 450.8)	133.9 (109.2 to 159.9)	247.1 (211.4 to 290.7)	0.0 (0.0 to 0.1)	20.9 (20.9 to 20.9)
Albania	2001	160.4 (143.9 to 178.5)	55.6 (46.1 to 65.8)	96.9 (83.2 to 112.7)	0.0 (0.0 to 0.0)	7.9 (7.9 to 7.9)	421.5 (378.1 to 469.0)	146.1 (121.2 to 172.8)	254.5 (218.5 to 296.0)	0.0 (0.0 to 0.1)	20.8 (20.8 to 20.8)

		2018 US Dollars per capita					2018 PPP per capita				
Country	Year	Health spending	Government health spending	Out-of-pocket health spending	Prepaid private health spending	Development assistance for health	Health spending	Government health spending	Out-of-pocket health spending	Prepaid private health spending	Development assistance for health
Albania	2002	163.5 (147.6 to 181.6)	58.9 (48.9 to 69.3)	98.5 (85.0 to 113.6)	0.0 (0.0 to 0.0)	6.1 (6.1 to 6.1)	429.6 (387.8 to 477.1)	154.7 (128.4 to 182.2)	258.9 (223.4 to 298.5)	0.0 (0.0 to 0.1)	16.0 (16.0 to 16.0)
Albania	2003	172.0 (155.3 to 190.6)	64.3 (53.3 to 75.5)	99.6 (86.0 to 115.4)	0.0 (0.0 to 0.0)	8.0 (8.0 to 8.0)	451.8 (407.9 to 500.7)	169.0 (140.1 to 198.3)	261.6 (225.9 to 303.3)	0.0 (0.0 to 0.1)	21.1 (21.1 to 21.1)
Albania	2004	182.1 (163.8 to 203.0)	73.1 (61.3 to 84.9)	101.1 (86.8 to 117.8)	0.0 (0.0 to 0.0)	7.8 (7.8 to 7.8)	478.4 (430.3 to 533.4)	192.1 (161.0 to 223.0)	265.7 (227.9 to 309.4)	0.0 (0.0 to 0.1)	20.5 (20.5 to 20.5)
Albania	2005	191.1 (172.7 to 210.5)	79.7 (67.5 to 92.4)	101.5 (86.7 to 117.0)	0.0 (0.0 to 0.0)	9.9 (9.9 to 9.9)	502.0 (453.7 to 552.9)	209.4 (177.3 to 242.7)	266.7 (227.6 to 307.3)	0.1 (0.0 to 0.1)	25.9 (25.9 to 25.9)
Albania	2006	197.9 (178.4 to 219.1)	85.1 (72.4 to 98.5)	102.2 (88.1 to 117.5)	0.0 (0.0 to 0.0)	10.6 (10.6 to 10.6)	519.9 (468.7 to 575.6)	223.5 (190.1 to 258.7)	268.6 (231.5 to 308.6)	0.1 (0.0 to 0.1)	27.8 (27.8 to 27.8)
Albania	2007	201.8 (181.8 to 224.0)	91.2 (77.7 to 105.1)	105.0 (90.2 to 120.5)	0.0 (0.0 to 0.0)	5.7 (5.7 to 5.7)	530.1 (477.7 to 588.4)	239.5 (204.2 to 276.2)	275.7 (237.0 to 316.7)	0.1 (0.0 to 0.1)	14.8 (14.8 to 14.8)
Albania	2008	213.6 (194.3 to 237.4)	100.5 (86.1 to 116.5)	104.4 (89.8 to 120.7)	0.0 (0.0 to 0.0)	8.7 (8.7 to 8.7)	561.2 (510.3 to 623.6)	263.9 (226.1 to 306.1)	274.4 (236.0 to 317.2)	0.1 (0.0 to 0.1)	22.9 (22.9 to 22.9)
Albania	2009	215.7 (196.1 to 238.8)	107.1 (91.6 to 124.7)	101.9 (87.7 to 118.0)	0.0 (0.0 to 0.0)	6.7 (6.7 to 6.7)	566.5 (515.3 to 627.2)	281.2 (240.7 to 327.7)	267.6 (230.3 to 310.1)	0.1 (0.0 to 0.1)	17.6 (17.6 to 17.6)
Albania	2010	223.9 (203.7 to 247.8)	110.4 (94.1 to 129.0)	107.7 (93.2 to 124.7)	0.0 (0.0 to 0.0)	5.8 (5.8 to 5.8)	588.3 (535.1 to 651.0)	289.9 (247.2 to 338.9)	283.0 (244.8 to 327.5)	0.1 (0.0 to 0.1)	15.2 (15.2 to 15.2)
Albania	2011	246.1 (224.1 to 270.2)	115.4 (99.2 to 134.4)	119.4 (103.5 to 137.7)	0.0 (0.0 to 0.0)	11.3 (11.3 to 11.3)	646.6 (588.7 to 709.9)	303.3 (260.6 to 353.1)	313.6 (271.8 to 361.8)	0.1 (0.0 to 0.1)	29.6 (29.6 to 29.6)
Albania	2012	258.7 (235.8 to 285.2)	119.6 (103.4 to 138.0)	134.7 (117.8 to 154.0)	0.0 (0.0 to 0.1)	4.3 (4.3 to 4.3)	679.6 (619.6 to 749.3)	314.3 (271.7 to 362.6)	354.0 (309.5 to 404.7)	0.1 (0.0 to 0.1)	11.3 (11.3 to 11.3)
Albania	2013	276.3 (251.6 to 304.5)	124.1 (107.0 to 142.2)	151.2 (132.4 to 173.1)	0.0 (0.0 to 0.1)	1.1 (1.1 to 1.1)	725.8 (660.9 to 799.8)	325.9 (281.2 to 373.4)	397.1 (347.9 to 454.8)	0.1 (0.0 to 0.1)	2.8 (2.8 to 2.8)
Albania	2014	305.0 (277.4 to 336.3)	131.6 (112.9 to 151.3)	168.5 (147.8 to 194.1)	0.0 (0.0 to 0.1)	4.9 (4.9 to 4.9)	801.3 (728.7 to 883.6)	345.8 (296.6 to 397.6)	442.5 (388.4 to 509.9)	0.1 (0.0 to 0.1)	12.8 (12.8 to 12.8)
Albania	2015	318.1 (286.9 to 353.0)	135.8 (115.4 to 157.3)	180.0 (156.7 to 208.2)	0.0 (0.0 to 0.1)	2.4 (2.4 to 2.4)	835.7 (753.7 to 927.3)	356.6 (303.2 to 413.1)	472.8 (411.6 to 546.9)	0.1 (0.0 to 0.1)	6.3 (6.3 to 6.3)
Albania	2016	330.0 (292.4 to 371.4)	139.5 (117.0 to 166.8)	188.9 (161.2 to 223.9)	0.0 (0.0 to 0.1)	1.6 (1.6 to 1.6)	867.0 (768.2 to 975.7)	366.6 (307.4 to 438.2)	496.1 (423.4 to 588.1)	0.1 (0.0 to 0.1)	4.2 (4.2 to 4.2)
Albania	2017	342.2 (303.8 to 385.9)	145.0 (121.6 to 173.3)	195.7 (166.3 to 231.7)	0.0 (0.0 to 0.1)	1.6 (1.6 to 1.6)	899.1 (798.0 to 1013.8)	380.8 (319.4 to 455.3)	514.0 (436.8 to 608.7)	0.1 (0.0 to 0.2)	4.2 (4.2 to 4.2)
Albania	2018	352.7 (311.4 to 398.3)	149.7 (125.5 to 178.9)	201.5 (170.6 to 240.9)	0.0 (0.0 to 0.1)	1.5 (1.4 to 1.5)	926.6 (818.1 to 1046.3)	393.3 (329.8 to 470.1)	529.4 (448.1 to 632.9)	0.1 (0.0 to 0.2)	3.8 (3.6 to 4.0)
Albania	2019	363.1 (321.3 to 408.3)	154.3 (129.5 to 184.3)	207.4 (175.3 to 247.3)	0.0 (0.0 to 0.1)	1.5 (1.3 to 1.6)	953.9 (844.1 to 1072.6)	405.2 (340.3 to 484.1)	544.7 (460.6 to 649.7)	0.1 (0.0 to 0.2)	3.9 (3.5 to 4.2)
Albania	2020	374.5 (330.9 to 425.1)	159.8 (134.0 to 191.1)	213.1 (180.0 to 254.2)	0.0 (0.0 to 0.1)	1.5 (1.3 to 1.7)	983.8 (869.4 to 1116.8)	419.9 (351.9 to 502.0)	559.9 (472.9 to 667.8)	0.1 (0.0 to 0.2)	3.9 (3.4 to 4.4)
Albania	2021	386.4 (342.6 to 435.2)	165.6 (138.9 to 197.7)	219.3 (185.2 to 261.0)	0.0 (0.0 to 0.1)	1.5 (1.3 to 1.7)	1015.1 (899.9 to 1143.2)	435.0 (364.9 to 519.3)	576.0 (486.5 to 685.7)	0.1 (0.0 to 0.2)	4.0 (3.5 to 4.5)
Albania	2022	395.2 (349.5 to 448.3)	170.6 (143.3 to 204.0)	223.0 (188.5 to 266.6)	0.0 (0.0 to 0.1)	1.6 (1.3 to 1.8)	1038.3 (918.0 to 1177.7)	448.2 (376.4 to 536.0)	585.9 (495.2 to 700.4)	0.1 (0.0 to 0.2)	4.1 (3.5 to 4.8)

Country	Year	2018 US Dollars per capita					2018 PPP per capita				
		Health spending	Government health spending	Out-of-pocket health spending	Prepaid private health spending	Development assistance for health	Health spending	Government health spending	Out-of-pocket health spending	Prepaid private health spending	Development assistance for health
Albania	2023	402.3 (357.3 to 451.3)	175.1 (146.7 to 208.3)	225.6 (190.6 to 269.2)	0.0 (0.0 to 0.1)	1.6 (1.4 to 1.9)	1056.9 (938.6 to 1185.5)	460.0 (385.3 to 547.3)	592.6 (500.7 to 707.3)	0.1 (0.0 to 0.2)	4.3 (3.6 to 5.0)
Albania	2024	409.4 (359.6 to 461.7)	179.6 (150.9 to 213.2)	228.0 (192.7 to 272.0)	0.0 (0.0 to 0.1)	1.7 (1.4 to 2.0)	1075.5 (944.8 to 1212.9)	471.9 (396.3 to 560.0)	599.1 (506.1 to 714.6)	0.1 (0.0 to 0.2)	4.4 (3.6 to 5.2)
Albania	2025	416.4 (368.8 to 469.1)	184.1 (154.1 to 217.9)	230.6 (194.6 to 274.7)	0.0 (0.0 to 0.1)	1.7 (1.4 to 2.0)	1094.0 (968.9 to 1232.3)	483.6 (404.9 to 572.4)	605.8 (511.2 to 721.7)	0.1 (0.0 to 0.2)	4.5 (3.7 to 5.4)
Albania	2026	423.8 (373.0 to 478.9)	188.6 (157.4 to 223.0)	233.3 (196.5 to 278.1)	0.0 (0.0 to 0.1)	1.8 (1.5 to 2.1)	1113.2 (980.0 to 1258.0)	495.6 (413.5 to 586.0)	612.9 (516.2 to 730.6)	0.1 (0.0 to 0.2)	4.7 (3.8 to 5.6)
Albania	2027	430.3 (380.2 to 486.9)	192.9 (161.4 to 228.3)	235.6 (197.7 to 280.5)	0.0 (0.0 to 0.1)	1.8 (1.5 to 2.2)	1130.5 (998.8 to 1279.2)	506.7 (423.9 to 599.8)	618.9 (519.3 to 736.9)	0.1 (0.0 to 0.2)	4.8 (3.9 to 5.8)
Albania	2028	437.1 (387.3 to 498.4)	197.2 (164.9 to 232.7)	238.0 (199.3 to 283.6)	0.0 (0.0 to 0.1)	1.9 (1.5 to 2.3)	1148.3 (1017.4 to 1309.3)	517.9 (433.1 to 611.2)	625.3 (523.5 to 745.0)	0.1 (0.0 to 0.2)	5.0 (4.0 to 6.2)
Albania	2029	444.6 (390.6 to 502.5)	201.8 (167.9 to 238.1)	240.9 (201.5 to 288.3)	0.0 (0.0 to 0.1)	2.0 (1.6 to 2.4)	1168.0 (1026.2 to 1320.2)	530.0 (441.1 to 625.5)	632.8 (529.4 to 757.4)	0.1 (0.0 to 0.2)	5.1 (4.1 to 6.4)
Albania	2030	452.1 (397.8 to 514.4)	206.3 (172.0 to 244.1)	243.7 (203.3 to 291.8)	0.0 (0.0 to 0.1)	2.0 (1.6 to 2.5)	1187.8 (1045.1 to 1351.3)	542.1 (451.8 to 641.3)	640.3 (534.1 to 766.4)	0.1 (0.0 to 0.2)	5.3 (4.2 to 6.7)
Albania	2031	460.5 (404.2 to 519.4)	211.3 (176.2 to 250.3)	247.1 (205.5 to 295.9)	0.0 (0.0 to 0.1)	2.1 (1.7 to 2.6)	1209.6 (1061.9 to 1364.6)	555.0 (463.0 to 657.5)	649.0 (540.0 to 777.5)	0.1 (0.0 to 0.2)	5.5 (4.3 to 7.0)
Albania	2032	468.9 (411.7 to 534.9)	216.2 (179.8 to 255.8)	250.6 (208.5 to 302.0)	0.0 (0.0 to 0.1)	2.2 (1.7 to 2.8)	1231.9 (1081.4 to 1405.1)	567.9 (472.5 to 671.9)	658.2 (547.6 to 793.2)	0.1 (0.0 to 0.3)	5.7 (4.5 to 7.2)
Albania	2033	477.3 (419.6 to 541.3)	220.9 (184.7 to 261.1)	254.1 (210.6 to 308.2)	0.0 (0.0 to 0.1)	2.2 (1.7 to 2.9)	1253.9 (1102.4 to 1421.9)	580.4 (485.2 to 685.9)	667.4 (553.2 to 809.6)	0.1 (0.0 to 0.3)	5.9 (4.5 to 7.6)
Albania	2034	486.3 (425.0 to 558.4)	226.1 (188.3 to 267.9)	257.9 (214.0 to 312.9)	0.0 (0.0 to 0.1)	2.3 (1.8 to 3.0)	1277.5 (1116.4 to 1466.8)	593.9 (494.7 to 703.7)	677.4 (562.2 to 821.9)	0.1 (0.0 to 0.3)	6.1 (4.7 to 7.9)
Albania	2035	495.4 (430.7 to 564.5)	231.2 (192.5 to 274.8)	261.7 (216.5 to 317.6)	0.0 (0.0 to 0.1)	2.4 (1.8 to 3.2)	1301.5 (1131.4 to 1483.1)	607.5 (505.7 to 721.9)	687.6 (568.9 to 834.4)	0.1 (0.0 to 0.3)	6.3 (4.8 to 8.4)
Albania	2036	504.9 (439.6 to 582.3)	236.5 (196.8 to 281.9)	265.8 (219.8 to 324.3)	0.1 (0.0 to 0.1)	2.5 (1.9 to 3.4)	1326.3 (1154.8 to 1529.6)	621.4 (517.0 to 740.5)	698.3 (577.5 to 852.1)	0.1 (0.0 to 0.3)	6.5 (4.9 to 8.9)
Albania	2037	514.5 (448.5 to 593.3)	242.0 (201.7 to 287.8)	269.9 (222.8 to 332.1)	0.1 (0.0 to 0.1)	2.6 (1.9 to 3.5)	1351.6 (1178.4 to 1558.6)	635.6 (529.9 to 756.1)	709.1 (585.4 to 872.5)	0.1 (0.0 to 0.3)	6.7 (5.1 to 9.2)
Albania	2038	523.7 (456.1 to 601.3)	247.2 (205.8 to 294.8)	273.8 (225.7 to 339.8)	0.1 (0.0 to 0.1)	2.7 (2.0 to 3.7)	1375.9 (1198.3 to 1579.6)	649.4 (540.6 to 774.5)	719.3 (592.9 to 892.6)	0.1 (0.0 to 0.3)	7.0 (5.2 to 9.6)
Albania	2039	534.0 (465.3 to 613.9)	253.0 (209.8 to 301.4)	278.2 (228.9 to 348.6)	0.1 (0.0 to 0.1)	2.8 (2.0 to 3.8)	1402.8 (1222.5 to 1612.8)	664.6 (551.3 to 791.8)	730.7 (601.3 to 915.8)	0.1 (0.0 to 0.3)	7.2 (5.4 to 10.0)
Albania	2040	544.0 (470.5 to 627.2)	258.7 (214.3 to 308.4)	282.4 (231.7 to 355.4)	0.1 (0.0 to 0.1)	2.8 (2.1 to 4.0)	1429.1 (1236.1 to 1647.6)	679.5 (563.1 to 810.3)	741.9 (608.8 to 933.8)	0.2 (0.0 to 0.3)	7.5 (5.6 to 10.4)
Albania	2041	554.5 (485.2 to 638.2)	264.5 (218.4 to 316.3)	287.0 (234.6 to 362.0)	0.1 (0.0 to 0.1)	3.0 (2.2 to 4.2)	1456.8 (1274.6 to 1676.6)	694.9 (573.8 to 830.9)	754.0 (616.4 to 951.0)	0.2 (0.0 to 0.3)	7.8 (5.7 to 11.0)
Albania	2042	565.4 (488.2 to 652.6)	270.6 (222.9 to 322.8)	291.7 (237.9 to 368.0)	0.1 (0.0 to 0.1)	3.1 (2.2 to 4.4)	1485.2 (1282.5 to 1714.4)	710.8 (585.5 to 848.1)	766.2 (624.8 to 966.7)	0.2 (0.0 to 0.4)	8.0 (5.9 to 11.6)
Albania	2043	576.7 (500.3 to 668.2)	276.9 (228.2 to 331.1)	296.5 (241.2 to 374.8)	0.1 (0.0 to 0.1)	3.2 (2.3 to 4.7)	1514.9 (1314.2 to 1755.3)	727.5 (599.4 to 869.8)	778.9 (633.6 to 984.6)	0.2 (0.0 to 0.4)	8.3 (6.0 to 12.3)

Country	Year	2018 US Dollars per capita					2018 PPP per capita				
		Health spending	Government health spending	Out-of-pocket health spending	Prepaid private health spending	Development assistance for health	Health spending	Government health spending	Out-of-pocket health spending	Prepaid private health spending	Development assistance for health
Albania	2044	587.5 (510.8 to 685.3)	283.1 (233.3 to 338.9)	301.0 (244.3 to 381.7)	0.1 (0.0 to 0.1)	3.3 (2.4 to 4.9)	1543.3 (1341.8 to 1800.2)	743.8 (612.8 to 890.4)	790.7 (641.7 to 1002.8)	0.2 (0.0 to 0.4)	8.6 (6.3 to 12.8)
Albania	2045	598.5 (519.5 to 699.4)	289.5 (238.4 to 348.0)	305.4 (247.4 to 389.4)	0.1 (0.0 to 0.1)	3.4 (2.5 to 5.2)	1572.2 (1364.8 to 1837.4)	760.6 (626.3 to 914.1)	802.4 (649.8 to 1022.8)	0.2 (0.0 to 0.4)	9.0 (6.5 to 13.6)
Albania	2046	609.2 (526.2 to 719.1)	295.8 (243.1 to 355.3)	309.8 (250.8 to 395.7)	0.1 (0.0 to 0.2)	3.5 (2.5 to 5.5)	1600.5 (1382.3 to 1889.0)	777.2 (638.8 to 933.5)	813.8 (658.8 to 1039.5)	0.2 (0.0 to 0.4)	9.3 (6.6 to 14.4)
Albania	2047	620.5 (538.4 to 721.0)	302.5 (248.5 to 364.3)	314.2 (254.1 to 402.1)	0.1 (0.0 to 0.2)	3.7 (2.6 to 5.7)	1630.0 (1414.4 to 1894.1)	794.6 (652.7 to 957.1)	825.5 (667.6 to 1056.5)	0.2 (0.0 to 0.4)	9.6 (6.9 to 14.9)
Albania	2048	631.6 (544.8 to 740.0)	309.2 (253.0 to 371.8)	318.6 (257.2 to 408.6)	0.1 (0.0 to 0.2)	3.8 (2.7 to 6.1)	1659.3 (1431.1 to 1944.0)	812.2 (664.7 to 976.8)	836.9 (675.7 to 1073.4)	0.2 (0.0 to 0.4)	10.0 (7.1 to 16.1)
Albania	2049	643.2 (558.4 to 748.1)	316.1 (258.3 to 381.0)	323.1 (260.7 to 415.2)	0.1 (0.0 to 0.2)	4.0 (2.8 to 6.3)	1689.7 (1467.0 to 1965.3)	830.4 (678.5 to 1000.8)	848.7 (684.8 to 1090.7)	0.2 (0.0 to 0.4)	10.4 (7.2 to 16.6)
Albania	2050	654.7 (562.5 to 770.7)	323.0 (262.4 to 390.1)	327.5 (264.1 to 421.8)	0.1 (0.0 to 0.2)	4.1 (2.8 to 6.7)	1719.8 (1477.7 to 2024.6)	848.5 (689.4 to 1024.8)	860.3 (693.8 to 1108.0)	0.2 (0.0 to 0.5)	10.8 (7.5 to 17.6)
Algeria	1995	106.6 (92.0 to 123.1)	75.1 (62.1 to 89.4)	28.6 (20.5 to 39.1)	2.4 (1.1 to 4.4)	0.5 (0.5 to 0.5)	369.9 (319.0 to 427.0)	260.6 (215.3 to 309.9)	99.1 (71.0 to 135.7)	8.3 (3.9 to 15.2)	1.9 (1.9 to 1.9)
Algeria	1996	107.3 (93.8 to 123.6)	76.2 (64.6 to 89.7)	28.1 (20.4 to 37.7)	2.4 (1.1 to 4.4)	0.5 (0.5 to 0.5)	372.1 (325.2 to 428.6)	264.3 (224.0 to 311.0)	97.5 (70.8 to 130.8)	8.4 (4.0 to 15.4)	1.9 (1.9 to 1.9)
Algeria	1997	108.1 (94.4 to 124.3)	75.8 (64.4 to 89.0)	29.3 (21.5 to 38.5)	2.5 (1.2 to 4.6)	0.6 (0.6 to 0.6)	375.1 (327.4 to 431.3)	263.1 (223.4 to 308.6)	101.6 (74.6 to 133.5)	8.5 (4.1 to 15.8)	1.9 (1.9 to 1.9)
Algeria	1998	109.7 (96.0 to 125.0)	77.0 (65.9 to 89.9)	30.1 (22.4 to 39.2)	2.5 (1.2 to 4.7)	0.1 (0.1 to 0.1)	380.6 (332.9 to 433.5)	267.1 (228.6 to 311.7)	104.5 (77.7 to 136.0)	8.6 (4.2 to 16.3)	0.3 (0.3 to 0.3)
Algeria	1999	111.1 (97.7 to 125.8)	78.3 (67.4 to 90.9)	30.0 (22.4 to 38.7)	2.5 (1.2 to 4.8)	0.2 (0.2 to 0.2)	385.3 (339.0 to 436.4)	271.7 (233.8 to 315.3)	104.1 (77.7 to 134.3)	8.7 (4.2 to 16.6)	0.7 (0.7 to 0.7)
Algeria	2000	117.2 (103.8 to 132.1)	84.7 (73.5 to 96.9)	29.7 (22.3 to 37.8)	2.7 (1.3 to 5.1)	0.1 (0.1 to 0.1)	406.6 (359.9 to 458.1)	293.8 (254.8 to 336.2)	103.1 (77.3 to 131.1)	9.3 (4.4 to 17.7)	0.3 (0.3 to 0.3)
Algeria	2001	119.5 (106.1 to 134.8)	87.8 (76.7 to 101.3)	28.9 (22.0 to 37.2)	2.8 (1.3 to 5.4)	0.1 (0.1 to 0.1)	414.6 (368.1 to 467.5)	304.6 (266.0 to 351.2)	100.2 (76.3 to 129.2)	9.6 (4.6 to 18.7)	0.3 (0.3 to 0.3)
Algeria	2002	122.3 (108.8 to 138.5)	90.2 (78.7 to 103.4)	29.2 (22.6 to 37.4)	2.9 (1.4 to 5.6)	0.0 (0.0 to 0.0)	424.4 (377.5 to 480.4)	312.7 (272.8 to 358.7)	101.4 (78.3 to 129.6)	10.0 (4.9 to 19.4)	0.2 (0.2 to 0.2)
Algeria	2003	126.7 (112.9 to 143.1)	93.2 (80.9 to 107.4)	30.4 (23.9 to 38.5)	3.0 (1.4 to 6.0)	0.0 (0.0 to 0.0)	439.4 (391.7 to 496.3)	323.3 (280.7 to 372.5)	105.5 (83.1 to 133.6)	10.5 (5.0 to 20.9)	0.1 (0.1 to 0.1)
Algeria	2004	127.8 (113.9 to 143.9)	91.8 (80.0 to 105.0)	32.8 (26.1 to 41.4)	3.1 (1.5 to 6.1)	0.1 (0.1 to 0.1)	443.4 (395.1 to 499.1)	318.5 (277.3 to 364.2)	113.8 (90.6 to 143.7)	10.8 (5.1 to 21.3)	0.3 (0.3 to 0.3)
Algeria	2005	132.5 (117.5 to 149.0)	93.9 (81.6 to 107.6)	35.4 (28.2 to 44.9)	3.2 (1.5 to 6.0)	0.1 (0.1 to 0.1)	459.6 (407.5 to 516.8)	325.6 (282.9 to 373.3)	122.8 (97.8 to 155.6)	10.9 (5.2 to 21.0)	0.3 (0.3 to 0.3)
Algeria	2006	139.7 (124.5 to 156.2)	98.2 (85.4 to 111.6)	38.3 (30.6 to 48.1)	3.1 (1.5 to 6.0)	0.1 (0.1 to 0.1)	484.7 (431.9 to 541.6)	340.5 (296.3 to 387.1)	132.9 (106.3 to 166.8)	10.9 (5.2 to 20.9)	0.4 (0.4 to 0.4)
Algeria	2007	153.2 (137.7 to 170.0)	107.7 (94.1 to 122.1)	42.2 (33.5 to 53.5)	3.2 (1.4 to 6.2)	0.1 (0.1 to 0.1)	531.4 (477.6 to 589.8)	373.6 (326.5 to 423.6)	146.3 (116.3 to 185.7)	11.1 (5.0 to 21.5)	0.4 (0.4 to 0.4)
Algeria	2008	173.3 (156.3 to 192.1)	122.6 (108.1 to 138.9)	47.2 (37.2 to 58.9)	3.4 (1.5 to 6.4)	0.2 (0.2 to 0.2)	601.1 (542.0 to 666.2)	425.3 (375.0 to 482.0)	163.6 (129.2 to 204.4)	11.6 (5.3 to 22.1)	0.6 (0.6 to 0.6)

Country	Year	2018 US Dollars per capita					2018 PPP per capita				
		Health spending	Government health spending	Out-of-pocket health spending	Prepaid private health spending	Development assistance for health	Health spending	Government health spending	Out-of-pocket health spending	Prepaid private health spending	Development assistance for health
Algeria	2009	190.2 (172.9 to 210.8)	133.5 (117.9 to 150.5)	53.2 (42.0 to 65.6)	3.4 (1.6 to 6.5)	0.1 (0.1 to 0.1)	659.6 (599.6 to 731.3)	463.0 (409.0 to 521.9)	184.4 (145.8 to 227.6)	12.0 (5.6 to 22.6)	0.3 (0.3 to 0.3)
Algeria	2010	209.4 (190.4 to 232.8)	147.5 (131.1 to 166.2)	58.3 (46.3 to 71.2)	3.5 (1.6 to 6.7)	0.1 (0.1 to 0.1)	726.5 (660.4 to 807.5)	511.7 (454.6 to 576.4)	202.2 (160.5 to 246.8)	12.2 (5.7 to 23.1)	0.4 (0.4 to 0.4)
Algeria	2011	226.9 (205.8 to 252.0)	161.0 (143.7 to 181.1)	62.1 (49.5 to 75.5)	3.6 (1.7 to 6.7)	0.2 (0.2 to 0.2)	787.0 (713.7 to 874.0)	558.3 (498.5 to 628.2)	215.5 (171.7 to 261.9)	12.3 (5.7 to 23.3)	0.8 (0.8 to 0.8)
Algeria	2012	247.1 (224.3 to 274.1)	176.5 (158.3 to 197.7)	66.8 (53.1 to 81.9)	3.7 (1.8 to 6.9)	0.1 (0.1 to 0.1)	857.0 (777.9 to 950.8)	612.2 (549.2 to 685.7)	231.7 (184.2 to 284.2)	12.8 (6.1 to 23.8)	0.3 (0.3 to 0.3)
Algeria	2013	261.3 (236.9 to 288.5)	185.8 (167.2 to 206.8)	71.5 (57.5 to 88.1)	3.8 (1.8 to 7.1)	0.2 (0.2 to 0.2)	906.4 (821.6 to 1000.7)	644.6 (580.1 to 717.1)	248.1 (199.6 to 305.7)	13.2 (6.4 to 24.6)	0.6 (0.6 to 0.6)
Algeria	2014	279.5 (251.8 to 308.7)	198.1 (177.2 to 221.5)	77.2 (61.6 to 95.9)	4.0 (2.0 to 7.6)	0.2 (0.2 to 0.2)	969.4 (873.4 to 1070.7)	687.1 (614.7 to 768.3)	267.9 (213.6 to 332.8)	13.9 (6.9 to 26.2)	0.5 (0.5 to 0.5)
Algeria	2015	297.7 (266.5 to 332.0)	209.0 (185.3 to 235.4)	84.3 (67.1 to 104.9)	4.3 (2.1 to 8.1)	0.1 (0.1 to 0.1)	1032.5 (924.4 to 1151.4)	724.9 (642.6 to 816.7)	292.5 (232.8 to 364.0)	14.9 (7.2 to 28.2)	0.2 (0.2 to 0.2)
Algeria	2016	304.0 (267.1 to 341.4)	210.8 (182.6 to 241.6)	88.7 (69.6 to 111.3)	4.4 (2.2 to 8.0)	0.0 (0.0 to 0.0)	1054.5 (926.3 to 1184.1)	731.2 (633.3 to 838.0)	307.7 (241.5 to 386.1)	15.4 (7.7 to 27.9)	0.2 (0.2 to 0.2)
Algeria	2017	283.0 (248.1 to 319.5)	189.8 (164.5 to 217.6)	88.7 (69.8 to 111.7)	4.4 (2.2 to 8.0)	0.0 (0.0 to 0.0)	981.5 (860.5 to 1108.3)	658.3 (570.7 to 754.8)	307.7 (241.9 to 387.3)	15.3 (7.7 to 27.6)	0.2 (0.2 to 0.2)
Algeria	2018	283.6 (249.1 to 319.7)	188.7 (163.8 to 216.8)	90.5 (70.9 to 113.9)	4.5 (2.2 to 8.1)	0.0 (0.0 to 0.0)	983.8 (864.0 to 1108.9)	654.3 (568.0 to 752.0)	313.8 (246.0 to 395.2)	15.5 (7.8 to 28.0)	0.2 (0.2 to 0.2)
Algeria	2019	286.0 (250.8 to 322.1)	189.2 (163.9 to 217.6)	92.3 (72.2 to 116.1)	4.5 (2.3 to 8.2)	0.0 (0.0 to 0.0)	992.2 (869.9 to 1117.3)	656.2 (568.6 to 754.8)	320.1 (250.5 to 402.9)	15.7 (7.9 to 28.3)	0.2 (0.1 to 0.2)
Algeria	2020	288.2 (252.5 to 325.5)	189.5 (164.1 to 217.9)	94.1 (73.6 to 118.3)	4.6 (2.3 to 8.2)	0.0 (0.0 to 0.0)	999.7 (875.9 to 1128.9)	657.4 (569.3 to 755.9)	326.3 (255.2 to 410.5)	15.8 (7.9 to 28.6)	0.2 (0.1 to 0.2)
Algeria	2021	291.1 (254.8 to 328.8)	190.5 (164.5 to 218.9)	96.0 (74.7 to 120.9)	4.6 (2.3 to 8.3)	0.0 (0.0 to 0.0)	1009.8 (883.9 to 1140.6)	660.7 (570.6 to 759.4)	332.9 (259.0 to 419.2)	16.0 (8.0 to 28.9)	0.2 (0.1 to 0.2)
Algeria	2022	295.2 (258.6 to 333.4)	192.6 (166.4 to 221.7)	97.9 (76.0 to 123.2)	4.6 (2.3 to 8.4)	0.0 (0.0 to 0.1)	1023.8 (897.0 to 1156.3)	668.0 (577.2 to 769.0)	339.5 (263.4 to 427.4)	16.1 (8.1 to 29.0)	0.2 (0.1 to 0.2)
Algeria	2023	298.1 (261.2 to 336.4)	193.8 (167.4 to 222.8)	99.6 (77.3 to 125.8)	4.7 (2.3 to 8.4)	0.0 (0.0 to 0.1)	1034.1 (906.1 to 1166.7)	672.2 (580.8 to 772.8)	345.6 (268.0 to 436.4)	16.2 (8.1 to 29.0)	0.2 (0.1 to 0.2)
Algeria	2024	301.2 (263.5 to 339.9)	195.1 (168.2 to 224.6)	101.4 (79.0 to 128.3)	4.7 (2.3 to 8.4)	0.0 (0.0 to 0.1)	1044.7 (914.0 to 1179.1)	676.6 (583.3 to 779.0)	351.8 (273.9 to 445.0)	16.2 (8.1 to 29.2)	0.2 (0.1 to 0.2)
Algeria	2025	305.2 (267.0 to 343.9)	197.1 (169.7 to 226.9)	103.4 (80.1 to 130.3)	4.7 (2.4 to 8.5)	0.0 (0.0 to 0.1)	1058.7 (926.0 to 1192.9)	683.5 (588.6 to 786.9)	358.6 (277.9 to 452.1)	16.4 (8.2 to 29.5)	0.2 (0.1 to 0.2)
Algeria	2026	309.6 (270.9 to 349.6)	199.3 (171.6 to 229.6)	105.5 (81.9 to 133.2)	4.8 (2.4 to 8.6)	0.0 (0.0 to 0.1)	1073.8 (939.8 to 1212.5)	691.3 (595.2 to 796.4)	365.8 (284.0 to 462.0)	16.5 (8.2 to 29.8)	0.2 (0.1 to 0.2)
Algeria	2027	314.8 (275.1 to 356.9)	202.2 (173.7 to 234.1)	107.8 (83.7 to 136.8)	4.8 (2.4 to 8.7)	0.1 (0.0 to 0.1)	1092.1 (954.3 to 1238.0)	701.4 (602.6 to 811.8)	373.8 (290.1 to 474.4)	16.7 (8.3 to 30.2)	0.2 (0.1 to 0.2)
Algeria	2028	320.7 (279.3 to 364.3)	205.6 (176.6 to 238.8)	110.2 (85.3 to 140.9)	4.9 (2.4 to 8.8)	0.1 (0.0 to 0.1)	1112.4 (968.7 to 1263.6)	713.2 (612.4 to 828.3)	382.1 (295.9 to 488.8)	16.9 (8.5 to 30.7)	0.2 (0.1 to 0.2)
Algeria	2029	327.2 (283.7 to 371.8)	209.5 (179.5 to 243.9)	112.7 (86.7 to 143.4)	5.0 (2.5 to 9.0)	0.1 (0.0 to 0.1)	1134.8 (984.2 to 1289.8)	726.6 (622.7 to 846.0)	390.8 (300.8 to 497.6)	17.2 (8.6 to 31.2)	0.2 (0.1 to 0.2)

Country	Year	2018 US Dollars per capita					2018 PPP per capita				
		Health spending	Government health spending	Out-of-pocket health spending	Prepaid private health spending	Development assistance for health	Health spending	Government health spending	Out-of-pocket health spending	Prepaid private health spending	Development assistance for health
Algeria	2030	334.0 (288.9 to 380.1)	213.7 (182.2 to 249.3)	115.2 (88.4 to 146.6)	5.0 (2.5 to 9.2)	0.1 (0.0 to 0.1)	1158.5 (1002.1 to 1318.3)	741.1 (632.1 to 864.6)	399.8 (306.6 to 508.3)	17.5 (8.7 to 31.8)	0.2 (0.2 to 0.2)
Algeria	2031	341.1 (295.4 to 389.5)	218.0 (186.4 to 255.2)	117.9 (90.2 to 150.0)	5.1 (2.6 to 9.3)	0.1 (0.0 to 0.1)	1183.1 (1024.6 to 1351.2)	756.3 (646.4 to 885.0)	408.8 (312.9 to 520.2)	17.8 (8.9 to 32.4)	0.2 (0.2 to 0.2)
Algeria	2032	348.4 (300.6 to 398.0)	222.6 (189.5 to 260.6)	120.5 (91.9 to 154.3)	5.2 (2.6 to 9.5)	0.1 (0.0 to 0.1)	1208.6 (1042.6 to 1380.5)	772.2 (657.3 to 903.9)	418.1 (318.8 to 535.1)	18.1 (9.0 to 33.0)	0.2 (0.2 to 0.2)
Algeria	2033	356.2 (304.4 to 407.0)	227.5 (193.6 to 267.0)	123.3 (93.5 to 159.6)	5.3 (2.7 to 9.7)	0.1 (0.0 to 0.1)	1235.5 (1055.9 to 1411.9)	789.2 (671.4 to 926.0)	427.6 (324.2 to 553.5)	18.5 (9.2 to 33.7)	0.2 (0.2 to 0.3)
Algeria	2034	364.0 (311.2 to 417.0)	232.6 (198.0 to 273.9)	126.0 (95.1 to 163.9)	5.4 (2.7 to 9.9)	0.1 (0.0 to 0.1)	1262.7 (1079.5 to 1446.4)	806.7 (686.7 to 950.1)	437.0 (329.9 to 568.5)	18.8 (9.4 to 34.4)	0.2 (0.2 to 0.3)
Algeria	2035	371.7 (317.7 to 426.4)	237.5 (201.8 to 279.9)	128.6 (96.7 to 167.6)	5.5 (2.8 to 10.1)	0.1 (0.0 to 0.1)	1289.4 (1102.0 to 1479.1)	823.8 (699.8 to 970.9)	446.2 (335.4 to 581.4)	19.2 (9.6 to 35.1)	0.2 (0.2 to 0.3)
Algeria	2036	379.1 (323.0 to 437.7)	242.2 (205.6 to 285.8)	131.2 (98.6 to 171.9)	5.6 (2.8 to 10.3)	0.1 (0.0 to 0.1)	1315.1 (1120.4 to 1518.4)	840.2 (713.0 to 991.2)	455.1 (341.9 to 596.3)	19.5 (9.7 to 35.8)	0.2 (0.2 to 0.3)
Algeria	2037	386.3 (328.0 to 446.8)	246.8 (208.1 to 292.0)	133.7 (99.9 to 176.5)	5.7 (2.8 to 10.5)	0.1 (0.0 to 0.1)	1339.9 (1137.7 to 1549.9)	856.1 (721.9 to 1012.8)	463.8 (346.6 to 612.3)	19.8 (9.9 to 36.5)	0.2 (0.2 to 0.3)
Algeria	2038	393.5 (333.6 to 455.4)	251.5 (211.8 to 297.6)	136.2 (101.5 to 180.6)	5.8 (2.9 to 10.7)	0.1 (0.0 to 0.1)	1365.0 (1157.3 to 1579.5)	872.3 (734.6 to 1032.3)	472.3 (352.0 to 626.4)	20.2 (10.0 to 37.1)	0.2 (0.2 to 0.3)
Algeria	2039	400.6 (339.5 to 464.0)	256.1 (214.9 to 302.7)	138.6 (103.1 to 183.7)	5.9 (2.9 to 10.9)	0.1 (0.0 to 0.1)	1389.6 (1177.7 to 1609.4)	888.3 (745.4 to 1050.1)	480.6 (357.5 to 637.2)	20.5 (10.2 to 37.8)	0.2 (0.2 to 0.3)
Algeria	2040	407.7 (344.0 to 471.6)	260.7 (217.4 to 309.0)	140.9 (104.5 to 187.0)	6.0 (3.0 to 11.1)	0.1 (0.0 to 0.1)	1414.0 (1193.3 to 1635.8)	904.4 (753.9 to 1071.9)	488.6 (362.3 to 648.5)	20.8 (10.3 to 38.4)	0.2 (0.2 to 0.3)
Algeria	2041	414.3 (348.7 to 481.3)	265.1 (220.2 to 314.9)	143.1 (105.7 to 190.3)	6.1 (3.0 to 11.3)	0.1 (0.0 to 0.1)	1437.0 (1209.4 to 1669.4)	919.4 (763.9 to 1092.3)	496.3 (366.8 to 660.2)	21.1 (10.5 to 39.0)	0.2 (0.2 to 0.3)
Algeria	2042	420.9 (353.5 to 489.7)	269.5 (222.8 to 319.8)	145.2 (107.1 to 193.6)	6.2 (3.1 to 11.4)	0.1 (0.1 to 0.1)	1460.0 (1226.0 to 1698.6)	934.7 (772.8 to 1109.4)	503.6 (371.3 to 671.7)	21.4 (10.6 to 39.6)	0.2 (0.2 to 0.3)
Algeria	2043	427.5 (358.8 to 497.5)	273.9 (225.9 to 325.3)	147.3 (108.2 to 196.7)	6.2 (3.1 to 11.6)	0.1 (0.1 to 0.1)	1482.7 (1244.5 to 1725.8)	950.1 (783.4 to 1128.5)	510.7 (375.3 to 682.4)	21.7 (10.7 to 40.2)	0.2 (0.2 to 0.3)
Algeria	2044	433.8 (361.7 to 508.9)	278.2 (228.8 to 331.5)	149.2 (109.4 to 199.6)	6.3 (3.1 to 11.8)	0.1 (0.1 to 0.1)	1504.7 (1254.6 to 1765.0)	965.1 (793.6 to 1149.8)	517.5 (379.4 to 692.2)	21.9 (10.8 to 40.8)	0.2 (0.2 to 0.4)
Algeria	2045	440.1 (365.9 to 515.6)	282.6 (232.1 to 338.0)	151.0 (110.5 to 202.4)	6.4 (3.2 to 11.9)	0.1 (0.1 to 0.1)	1526.4 (1269.3 to 1788.4)	980.1 (805.0 to 1172.3)	523.9 (383.4 to 702.2)	22.2 (11.0 to 41.3)	0.2 (0.2 to 0.4)
Algeria	2046	446.0 (369.8 to 524.7)	286.7 (234.4 to 344.1)	152.8 (111.7 to 205.3)	6.5 (3.2 to 12.1)	0.1 (0.1 to 0.1)	1546.9 (1282.7 to 1820.0)	994.3 (813.0 to 1193.4)	529.9 (387.3 to 712.1)	22.4 (11.1 to 41.9)	0.3 (0.2 to 0.4)
Algeria	2047	451.7 (373.9 to 532.4)	290.7 (236.8 to 350.2)	154.4 (113.1 to 208.1)	6.5 (3.2 to 12.2)	0.1 (0.1 to 0.1)	1566.7 (1296.9 to 1846.7)	1008.2 (821.3 to 1214.6)	535.6 (392.4 to 721.8)	22.7 (11.2 to 42.4)	0.3 (0.2 to 0.4)
Algeria	2048	457.5 (379.4 to 539.4)	294.8 (239.2 to 356.5)	156.0 (114.1 to 210.9)	6.6 (3.3 to 12.4)	0.1 (0.1 to 0.1)	1586.8 (1315.9 to 1870.8)	1022.4 (829.8 to 1236.5)	541.2 (395.9 to 731.6)	22.9 (11.3 to 42.9)	0.3 (0.2 to 0.4)
Algeria	2049	463.1 (383.4 to 546.7)	298.7 (241.5 to 362.9)	157.7 (115.1 to 213.5)	6.7 (3.3 to 12.5)	0.1 (0.1 to 0.1)	1606.3 (1329.9 to 1896.2)	1036.1 (837.8 to 1258.8)	546.8 (399.4 to 740.6)	23.1 (11.4 to 43.4)	0.3 (0.2 to 0.4)
Algeria	2050	468.8 (387.8 to 556.1)	302.7 (244.3 to 369.3)	159.3 (116.4 to 216.3)	6.7 (3.3 to 12.7)	0.1 (0.1 to 0.1)	1626.0 (1345.1 to 1928.9)	1049.9 (847.5 to 1281.0)	552.5 (403.6 to 750.2)	23.4 (11.5 to 43.9)	0.3 (0.2 to 0.5)

		2018 US Dollars per capita					2018 PPP per capita				
Country	Year	Health spending	Government health spending	Out-of-pocket health spending	Prepaid private health spending	Development assistance for health	Health spending	Government health spending	Out-of-pocket health spending	Prepaid private health spending	Development assistance for health
American Samoa	1995	1109.7 (886.4 to 1383.4)	1032.9 (810.2 to 1303.1)	68.8 (49.2 to 95.2)	8.1 (4.0 to 14.8)	0.0 (0.0 to 0.0)	1109.7 (886.4 to 1383.4)	1032.9 (810.2 to 1303.1)	68.8 (49.2 to 95.2)	8.1 (4.0 to 14.8)	0.0 (0.0 to 0.0)
American Samoa	1996	1097.9 (885.2 to 1359.6)	1022.5 (812.1 to 1292.5)	67.6 (47.9 to 92.9)	7.8 (3.8 to 14.2)	0.0 (0.0 to 0.0)	1097.9 (885.2 to 1359.6)	1022.5 (812.1 to 1292.5)	67.6 (47.9 to 92.9)	7.8 (3.8 to 14.2)	0.0 (0.0 to 0.0)
American Samoa	1997	1093.8 (892.9 to 1340.4)	1019.7 (822.0 to 1260.9)	66.8 (47.3 to 90.4)	7.4 (3.5 to 13.8)	0.0 (0.0 to 0.0)	1093.8 (892.9 to 1340.4)	1019.7 (822.0 to 1260.9)	66.8 (47.3 to 90.4)	7.4 (3.5 to 13.8)	0.0 (0.0 to 0.0)
American Samoa	1998	1081.5 (894.4 to 1296.1)	1009.0 (830.7 to 1218.9)	65.8 (46.1 to 89.1)	6.7 (3.3 to 12.4)	0.0 (0.0 to 0.0)	1081.5 (894.4 to 1296.1)	1009.0 (830.7 to 1218.9)	65.8 (46.1 to 89.1)	6.7 (3.3 to 12.4)	0.0 (0.0 to 0.0)
American Samoa	1999	1072.0 (911.2 to 1254.3)	1001.2 (844.6 to 1175.3)	64.9 (45.3 to 88.3)	5.8 (2.8 to 10.8)	0.0 (0.0 to 0.0)	1072.0 (911.2 to 1254.3)	1001.2 (844.6 to 1175.3)	64.9 (45.3 to 88.3)	5.8 (2.8 to 10.8)	0.0 (0.0 to 0.0)
American Samoa	2000	1073.2 (935.1 to 1221.8)	1003.8 (869.0 to 1148.5)	64.5 (45.0 to 88.9)	4.9 (2.4 to 9.2)	0.0 (0.0 to 0.0)	1073.2 (935.1 to 1221.8)	1003.8 (869.0 to 1148.5)	64.5 (45.0 to 88.9)	4.9 (2.4 to 9.2)	0.0 (0.0 to 0.0)
American Samoa	2001	1070.4 (966.7 to 1182.0)	1002.1 (896.3 to 1110.4)	64.3 (44.9 to 88.6)	3.9 (1.9 to 7.3)	0.0 (0.0 to 0.0)	1070.4 (966.7 to 1182.0)	1002.1 (896.3 to 1110.4)	64.3 (44.9 to 88.6)	3.9 (1.9 to 7.3)	0.0 (0.0 to 0.0)
American Samoa	2002	1067.2 (995.5 to 1140.5)	998.6 (927.4 to 1070.3)	64.9 (44.7 to 90.9)	3.7 (1.8 to 7.1)	0.0 (0.0 to 0.0)	1067.2 (995.5 to 1140.5)	998.6 (927.4 to 1070.3)	64.9 (44.7 to 90.9)	3.7 (1.8 to 7.1)	0.0 (0.0 to 0.0)
American Samoa	2003	1064.0 (1008.9 to 1122.1)	994.6 (945.1 to 1050.0)	65.7 (45.4 to 91.6)	3.6 (1.8 to 7.0)	0.0 (0.0 to 0.0)	1064.0 (1008.9 to 1122.1)	994.6 (945.1 to 1050.0)	65.7 (45.4 to 91.6)	3.6 (1.8 to 7.0)	0.0 (0.0 to 0.0)
American Samoa	2004	1039.8 (974.3 to 1108.8)	969.8 (905.9 to 1034.2)	66.2 (46.0 to 91.5)	3.7 (1.8 to 7.1)	0.0 (0.0 to 0.0)	1039.8 (974.3 to 1108.8)	969.8 (905.9 to 1034.2)	66.2 (46.0 to 91.5)	3.7 (1.8 to 7.1)	0.0 (0.0 to 0.0)
American Samoa	2005	1005.4 (913.7 to 1100.8)	935.5 (845.8 to 1030.1)	66.0 (46.0 to 89.9)	4.0 (1.9 to 7.7)	0.0 (0.0 to 0.0)	1005.4 (913.7 to 1100.8)	935.5 (845.8 to 1030.1)	66.0 (46.0 to 89.9)	4.0 (1.9 to 7.7)	0.0 (0.0 to 0.0)
American Samoa	2006	942.7 (839.4 to 1065.4)	873.8 (768.2 to 996.0)	64.5 (45.1 to 87.8)	4.4 (2.1 to 8.4)	0.0 (0.0 to 0.0)	942.7 (839.4 to 1065.4)	873.8 (768.2 to 996.0)	64.5 (45.1 to 87.8)	4.4 (2.1 to 8.4)	0.0 (0.0 to 0.0)
American Samoa	2007	926.7 (811.4 to 1066.0)	856.3 (740.7 to 992.8)	65.1 (45.1 to 90.4)	5.4 (2.6 to 10.1)	0.0 (0.0 to 0.0)	926.7 (811.4 to 1066.0)	856.3 (740.7 to 992.8)	65.1 (45.1 to 90.4)	5.4 (2.6 to 10.1)	0.0 (0.0 to 0.0)
American Samoa	2008	887.3 (771.2 to 1018.9)	815.3 (698.0 to 949.7)	64.8 (44.8 to 89.8)	7.2 (3.5 to 14.0)	0.0 (0.0 to 0.0)	887.3 (771.2 to 1018.9)	815.3 (698.0 to 949.7)	64.8 (44.8 to 89.8)	7.2 (3.5 to 14.0)	0.0 (0.0 to 0.0)
American Samoa	2009	835.5 (726.7 to 953.7)	763.1 (653.8 to 882.8)	63.8 (43.6 to 88.2)	8.7 (4.2 to 16.2)	0.0 (0.0 to 0.0)	835.5 (726.7 to 953.7)	763.1 (653.8 to 882.8)	63.8 (43.6 to 88.2)	8.7 (4.2 to 16.2)	0.0 (0.0 to 0.0)
American Samoa	2010	807.0 (708.3 to 912.2)	733.6 (638.9 to 837.9)	63.4 (43.6 to 87.3)	10.0 (4.9 to 18.6)	0.0 (0.0 to 0.0)	807.0 (708.3 to 912.2)	733.6 (638.9 to 837.9)	63.4 (43.6 to 87.3)	10.0 (4.9 to 18.6)	0.0 (0.0 to 0.0)
American Samoa	2011	784.2 (704.3 to 868.8)	710.4 (636.0 to 793.4)	62.7 (43.0 to 86.9)	11.1 (5.3 to 20.2)	0.0 (0.0 to 0.0)	784.2 (704.3 to 868.8)	710.4 (636.0 to 793.4)	62.7 (43.0 to 86.9)	11.1 (5.3 to 20.2)	0.0 (0.0 to 0.0)
American Samoa	2012	738.3 (679.5 to 802.0)	666.2 (614.8 to 723.5)	60.6 (41.9 to 85.4)	11.4 (5.4 to 20.9)	0.0 (0.0 to 0.0)	738.3 (679.5 to 802.0)	666.2 (614.8 to 723.5)	60.6 (41.9 to 85.4)	11.4 (5.4 to 20.9)	0.0 (0.0 to 0.0)
American Samoa	2013	702.4 (654.6 to 750.7)	632.0 (590.0 to 677.8)	58.8 (40.8 to 82.8)	11.5 (5.4 to 21.2)	0.0 (0.0 to 0.0)	702.4 (654.6 to 750.7)	632.0 (590.0 to 677.8)	58.8 (40.8 to 82.8)	11.5 (5.4 to 21.2)	0.0 (0.0 to 0.0)
American Samoa	2014	694.0 (637.9 to 750.3)	624.1 (571.8 to 676.8)	58.1 (40.7 to 81.2)	11.8 (5.5 to 21.9)	0.0 (0.0 to 0.0)	694.0 (637.9 to 750.3)	624.1 (571.8 to 676.8)	58.1 (40.7 to 81.2)	11.8 (5.5 to 21.9)	0.0 (0.0 to 0.0)
American Samoa	2015	691.8 (618.4 to 770.2)	622.6 (553.1 to 696.3)	57.5 (39.9 to 79.5)	11.8 (5.5 to 21.9)	0.0 (0.0 to 0.0)	691.8 (618.4 to 770.2)	622.6 (553.1 to 696.3)	57.5 (39.9 to 79.5)	11.8 (5.5 to 21.9)	0.0 (0.0 to 0.0)

		2018 US Dollars per capita					2018 PPP per capita				
Country	Year	Health spending	Government health spending	Out-of-pocket health spending	Prepaid private health spending	Development assistance for health	Health spending	Government health spending	Out-of-pocket health spending	Prepaid private health spending	Development assistance for health
American Samoa	2016	691.8 (603.9 to 790.6)	623.1 (535.6 to 715.3)	57.1 (39.6 to 81.4)	11.5 (5.3 to 21.9)	0.0 (0.0 to 0.0)	691.8 (603.9 to 790.6)	623.1 (535.6 to 715.3)	57.1 (39.6 to 81.4)	11.5 (5.3 to 21.9)	0.0 (0.0 to 0.0)
American Samoa	2017	689.2 (596.8 to 789.7)	620.7 (532.6 to 713.1)	57.0 (39.7 to 81.0)	11.5 (5.3 to 22.0)	0.0 (0.0 to 0.0)	689.2 (596.8 to 789.7)	620.7 (532.6 to 713.1)	57.0 (39.7 to 81.0)	11.5 (5.3 to 22.0)	0.0 (0.0 to 0.0)
American Samoa	2018	700.3 (608.6 to 800.1)	631.0 (541.6 to 727.6)	57.6 (39.8 to 82.0)	11.7 (5.4 to 22.5)	0.0 (0.0 to 0.0)	700.3 (608.6 to 800.1)	631.0 (541.6 to 727.6)	57.6 (39.8 to 82.0)	11.7 (5.4 to 22.5)	0.0 (0.0 to 0.0)
American Samoa	2019	711.4 (615.7 to 814.3)	641.2 (550.5 to 740.1)	58.2 (40.2 to 83.0)	11.9 (5.5 to 22.9)	0.0 (0.0 to 0.0)	711.4 (615.7 to 814.3)	641.2 (550.5 to 740.1)	58.2 (40.2 to 83.0)	11.9 (5.5 to 22.9)	0.0 (0.0 to 0.0)
American Samoa	2020	724.3 (630.8 to 829.4)	653.2 (560.0 to 755.0)	58.9 (40.8 to 84.0)	12.2 (5.6 to 23.3)	0.0 (0.0 to 0.0)	724.3 (630.8 to 829.4)	653.2 (560.0 to 755.0)	58.9 (40.8 to 84.0)	12.2 (5.6 to 23.3)	0.0 (0.0 to 0.0)
American Samoa	2021	738.3 (637.7 to 846.7)	666.1 (570.8 to 770.1)	59.8 (41.4 to 84.7)	12.5 (5.8 to 23.8)	0.0 (0.0 to 0.0)	738.3 (637.7 to 846.7)	666.1 (570.8 to 770.1)	59.8 (41.4 to 84.7)	12.5 (5.8 to 23.8)	0.0 (0.0 to 0.0)
American Samoa	2022	751.4 (651.7 to 859.8)	678.1 (579.3 to 784.2)	60.5 (41.9 to 86.1)	12.7 (5.9 to 24.3)	0.0 (0.0 to 0.0)	751.4 (651.7 to 859.8)	678.1 (579.3 to 784.2)	60.5 (41.9 to 86.1)	12.7 (5.9 to 24.3)	0.0 (0.0 to 0.0)
American Samoa	2023	763.3 (659.4 to 876.5)	689.1 (588.4 to 795.6)	61.2 (42.3 to 87.2)	13.0 (6.0 to 24.7)	0.0 (0.0 to 0.0)	763.3 (659.4 to 876.5)	689.1 (588.4 to 795.6)	61.2 (42.3 to 87.2)	13.0 (6.0 to 24.7)	0.0 (0.0 to 0.0)
American Samoa	2024	773.2 (670.0 to 884.8)	698.3 (596.8 to 804.8)	61.7 (42.5 to 88.0)	13.2 (6.1 to 25.0)	0.0 (0.0 to 0.0)	773.2 (670.0 to 884.8)	698.3 (596.8 to 804.8)	61.7 (42.5 to 88.0)	13.2 (6.1 to 25.0)	0.0 (0.0 to 0.0)
American Samoa	2025	782.7 (675.9 to 897.1)	707.1 (605.0 to 814.7)	62.2 (42.9 to 88.6)	13.4 (6.2 to 25.3)	0.0 (0.0 to 0.0)	782.7 (675.9 to 897.1)	707.1 (605.0 to 814.7)	62.2 (42.9 to 88.6)	13.4 (6.2 to 25.3)	0.0 (0.0 to 0.0)
American Samoa	2026	788.1 (683.3 to 903.9)	712.3 (608.3 to 821.0)	62.3 (43.1 to 88.7)	13.5 (6.2 to 25.5)	0.0 (0.0 to 0.0)	788.1 (683.3 to 903.9)	712.3 (608.3 to 821.0)	62.3 (43.1 to 88.7)	13.5 (6.2 to 25.5)	0.0 (0.0 to 0.0)
American Samoa	2027	790.8 (681.1 to 906.7)	715.1 (609.3 to 824.6)	62.2 (43.2 to 87.9)	13.6 (6.3 to 25.6)	0.0 (0.0 to 0.0)	790.8 (681.1 to 906.7)	715.1 (609.3 to 824.6)	62.2 (43.2 to 87.9)	13.6 (6.3 to 25.6)	0.0 (0.0 to 0.0)
American Samoa	2028	793.8 (685.6 to 910.2)	718.0 (610.9 to 828.1)	62.1 (43.1 to 87.3)	13.7 (6.3 to 25.7)	0.0 (0.0 to 0.0)	793.8 (685.6 to 910.2)	718.0 (610.9 to 828.1)	62.1 (43.1 to 87.3)	13.7 (6.3 to 25.7)	0.0 (0.0 to 0.0)
American Samoa	2029	797.6 (686.4 to 916.6)	721.7 (613.3 to 833.0)	62.1 (43.0 to 87.3)	13.8 (6.4 to 25.9)	0.0 (0.0 to 0.0)	797.6 (686.4 to 916.6)	721.7 (613.3 to 833.0)	62.1 (43.0 to 87.3)	13.8 (6.4 to 25.9)	0.0 (0.0 to 0.0)
American Samoa	2030	801.4 (689.1 to 919.1)	725.4 (615.3 to 837.8)	62.1 (43.0 to 87.3)	13.9 (6.4 to 26.0)	0.0 (0.0 to 0.0)	801.4 (689.1 to 919.1)	725.4 (615.3 to 837.8)	62.1 (43.0 to 87.3)	13.9 (6.4 to 26.0)	0.0 (0.0 to 0.0)
American Samoa	2031	805.3 (693.6 to 926.3)	729.2 (618.4 to 843.0)	62.1 (42.9 to 87.3)	14.0 (6.5 to 26.2)	0.0 (0.0 to 0.0)	805.3 (693.6 to 926.3)	729.2 (618.4 to 843.0)	62.1 (42.9 to 87.3)	14.0 (6.5 to 26.2)	0.0 (0.0 to 0.0)
American Samoa	2032	809.5 (692.7 to 929.4)	733.3 (621.7 to 848.5)	62.1 (42.9 to 87.5)	14.1 (6.6 to 26.5)	0.0 (0.0 to 0.0)	809.5 (692.7 to 929.4)	733.3 (621.7 to 848.5)	62.1 (42.9 to 87.5)	14.1 (6.6 to 26.5)	0.0 (0.0 to 0.0)
American Samoa	2033	813.3 (697.7 to 937.4)	737.0 (624.7 to 854.0)	62.1 (42.9 to 87.6)	14.2 (6.6 to 26.8)	0.0 (0.0 to 0.0)	813.3 (697.7 to 937.4)	737.0 (624.7 to 854.0)	62.1 (42.9 to 87.6)	14.2 (6.6 to 26.8)	0.0 (0.0 to 0.0)
American Samoa	2034	816.8 (698.4 to 939.5)	740.4 (627.1 to 858.9)	62.1 (42.9 to 87.7)	14.3 (6.7 to 26.9)	0.0 (0.0 to 0.0)	816.8 (698.4 to 939.5)	740.4 (627.1 to 858.9)	62.1 (42.9 to 87.7)	14.3 (6.7 to 26.9)	0.0 (0.0 to 0.0)
American Samoa	2035	820.6 (704.1 to 949.1)	744.2 (628.5 to 865.1)	62.0 (42.9 to 87.5)	14.4 (6.7 to 27.1)	0.0 (0.0 to 0.0)	820.6 (704.1 to 949.1)	744.2 (628.5 to 865.1)	62.0 (42.9 to 87.5)	14.4 (6.7 to 27.1)	0.0 (0.0 to 0.0)
American Samoa	2036	824.6 (703.2 to 952.7)	748.1 (630.3 to 871.1)	62.0 (42.9 to 87.2)	14.5 (6.8 to 27.2)	0.0 (0.0 to 0.0)	824.6 (703.2 to 952.7)	748.1 (630.3 to 871.1)	62.0 (42.9 to 87.2)	14.5 (6.8 to 27.2)	0.0 (0.0 to 0.0)

		2018 US Dollars per capita					2018 PPP per capita				
Country	Year	Health spending	Government health spending	Out-of-pocket health spending	Prepaid private health spending	Development assistance for health	Health spending	Government health spending	Out-of-pocket health spending	Prepaid private health spending	Development assistance for health
American Samoa	2037	828.8 (707.8 to 956.7)	752.2 (632.6 to 875.5)	62.0 (42.9 to 87.3)	14.6 (6.8 to 27.4)	0.0 (0.0 to 0.0)	828.8 (707.8 to 956.7)	752.2 (632.6 to 875.5)	62.0 (42.9 to 87.3)	14.6 (6.8 to 27.4)	0.0 (0.0 to 0.0)
American Samoa	2038	833.2 (709.9 to 967.3)	756.4 (634.5 to 880.8)	62.0 (42.9 to 87.3)	14.7 (6.9 to 27.5)	0.0 (0.0 to 0.0)	833.2 (709.9 to 967.3)	756.4 (634.5 to 880.8)	62.0 (42.9 to 87.3)	14.7 (6.9 to 27.5)	0.0 (0.0 to 0.0)
American Samoa	2039	837.6 (713.8 to 968.0)	760.7 (636.6 to 888.4)	62.0 (42.8 to 87.3)	14.9 (6.9 to 27.7)	0.0 (0.0 to 0.0)	837.6 (713.8 to 968.0)	760.7 (636.6 to 888.4)	62.0 (42.8 to 87.3)	14.9 (6.9 to 27.7)	0.0 (0.0 to 0.0)
American Samoa	2040	841.7 (714.1 to 976.0)	764.7 (638.4 to 893.5)	62.0 (42.7 to 87.3)	15.0 (7.0 to 27.8)	0.0 (0.0 to 0.0)	841.7 (714.1 to 976.0)	764.7 (638.4 to 893.5)	62.0 (42.7 to 87.3)	15.0 (7.0 to 27.8)	0.0 (0.0 to 0.0)
American Samoa	2041	845.5 (718.6 to 978.4)	768.5 (640.9 to 898.1)	62.0 (42.6 to 87.1)	15.1 (7.0 to 28.0)	0.0 (0.0 to 0.0)	845.5 (718.6 to 978.4)	768.5 (640.9 to 898.1)	62.0 (42.6 to 87.1)	15.1 (7.0 to 28.0)	0.0 (0.0 to 0.0)
American Samoa	2042	849.3 (717.1 to 987.7)	772.2 (644.0 to 903.6)	61.9 (42.5 to 86.9)	15.2 (7.1 to 28.3)	0.0 (0.0 to 0.0)	849.3 (717.1 to 987.7)	772.2 (644.0 to 903.6)	61.9 (42.5 to 86.9)	15.2 (7.1 to 28.3)	0.0 (0.0 to 0.0)
American Samoa	2043	853.1 (724.2 to 990.8)	775.9 (646.3 to 908.1)	61.9 (42.3 to 86.7)	15.3 (7.2 to 28.6)	0.0 (0.0 to 0.0)	853.1 (724.2 to 990.8)	775.9 (646.3 to 908.1)	61.9 (42.3 to 86.7)	15.3 (7.2 to 28.6)	0.0 (0.0 to 0.0)
American Samoa	2044	856.9 (722.3 to 1000.1)	779.7 (649.0 to 913.9)	61.8 (42.3 to 86.5)	15.4 (7.2 to 28.9)	0.0 (0.0 to 0.0)	856.9 (722.3 to 1000.1)	779.7 (649.0 to 913.9)	61.8 (42.3 to 86.5)	15.4 (7.2 to 28.9)	0.0 (0.0 to 0.0)
American Samoa	2045	861.2 (728.0 to 1002.3)	783.9 (652.0 to 921.8)	61.8 (42.3 to 86.6)	15.5 (7.3 to 29.1)	0.0 (0.0 to 0.0)	861.2 (728.0 to 1002.3)	783.9 (652.0 to 921.8)	61.8 (42.3 to 86.6)	15.5 (7.3 to 29.1)	0.0 (0.0 to 0.0)
American Samoa	2046	865.7 (726.6 to 1011.4)	788.3 (654.2 to 928.0)	61.8 (42.2 to 86.5)	15.6 (7.3 to 29.2)	0.0 (0.0 to 0.0)	865.7 (726.6 to 1011.4)	788.3 (654.2 to 928.0)	61.8 (42.2 to 86.5)	15.6 (7.3 to 29.2)	0.0 (0.0 to 0.0)
American Samoa	2047	870.5 (734.5 to 1014.6)	792.9 (656.5 to 934.5)	61.8 (42.2 to 86.5)	15.8 (7.4 to 29.4)	0.0 (0.0 to 0.0)	870.5 (734.5 to 1014.6)	792.9 (656.5 to 934.5)	61.8 (42.2 to 86.5)	15.8 (7.4 to 29.4)	0.0 (0.0 to 0.0)
American Samoa	2048	875.7 (731.1 to 1026.6)	798.0 (659.1 to 942.9)	61.8 (42.3 to 86.4)	15.9 (7.5 to 29.6)	0.0 (0.0 to 0.0)	875.7 (731.1 to 1026.6)	798.0 (659.1 to 942.9)	61.8 (42.3 to 86.4)	15.9 (7.5 to 29.6)	0.0 (0.0 to 0.0)
American Samoa	2049	881.0 (740.0 to 1029.5)	803.1 (661.7 to 951.3)	61.8 (42.3 to 86.6)	16.0 (7.6 to 29.8)	0.0 (0.0 to 0.0)	881.0 (740.0 to 1029.5)	803.1 (661.7 to 951.3)	61.8 (42.3 to 86.6)	16.0 (7.6 to 29.8)	0.0 (0.0 to 0.0)
American Samoa	2050	886.8 (739.5 to 1043.6)	808.7 (664.0 to 960.5)	61.9 (42.3 to 86.8)	16.2 (7.6 to 30.1)	0.0 (0.0 to 0.0)	886.8 (739.5 to 1043.6)	808.7 (664.0 to 960.5)	61.9 (42.3 to 86.8)	16.2 (7.6 to 30.1)	0.0 (0.0 to 0.0)
Andorra	1995	3342.7 (3218.7 to 3478.3)	1425.0 (1334.2 to 1525.9)	1555.4 (1486.4 to 1623.5)	362.3 (320.1 to 409.5)	0.0 (0.0 to 0.0)	6209.1 (5978.9 to 6461.0)	2646.9 (2478.3 to 2834.4)	2889.2 (2761.1 to 3015.7)	673.0 (594.6 to 760.6)	0.0 (0.0 to 0.0)
Andorra	1996	3450.6 (3350.4 to 3548.1)	1465.9 (1390.4 to 1541.8)	1597.0 (1546.2 to 1647.0)	387.7 (353.2 to 425.3)	0.0 (0.0 to 0.0)	6409.7 (6223.5 to 6590.6)	2723.0 (2582.7 to 2863.9)	2966.5 (2872.0 to 3059.4)	720.1 (656.1 to 790.0)	0.0 (0.0 to 0.0)
Andorra	1997	3671.9 (3577.1 to 3759.3)	1561.5 (1495.7 to 1635.3)	1684.6 (1632.1 to 1734.6)	425.8 (391.6 to 462.8)	0.0 (0.0 to 0.0)	6820.6 (6644.6 to 6983.0)	2900.6 (2778.3 to 3037.5)	3129.2 (3031.7 to 3222.0)	790.9 (727.5 to 859.7)	0.0 (0.0 to 0.0)
Andorra	1998	3789.2 (3692.6 to 3882.3)	1605.3 (1536.4 to 1678.3)	1733.9 (1683.9 to 1786.2)	450.0 (415.4 to 486.4)	0.0 (0.0 to 0.0)	7038.4 (6859.0 to 7211.4)	2981.8 (2854.0 to 3117.5)	3220.8 (3127.8 to 3317.9)	835.9 (771.7 to 903.4)	0.0 (0.0 to 0.0)
Andorra	1999	3785.9 (3693.6 to 3873.9)	1596.8 (1531.1 to 1668.8)	1767.8 (1717.5 to 1822.3)	421.3 (390.0 to 455.1)	0.0 (0.0 to 0.0)	7032.4 (6861.0 to 7195.8)	2966.1 (2844.0 to 3099.9)	3283.8 (3190.3 to 3385.0)	782.6 (724.5 to 845.4)	0.0 (0.0 to 0.0)
Andorra	2000	3748.0 (3661.0 to 3830.3)	1567.8 (1504.7 to 1635.1)	1783.7 (1737.1 to 1836.5)	396.4 (368.6 to 425.9)	0.0 (0.0 to 0.0)	6961.9 (6800.4 to 7114.9)	2912.3 (2795.0 to 3037.3)	3313.2 (3226.7 to 3411.4)	736.4 (684.7 to 791.1)	0.0 (0.0 to 0.0)
Andorra	2001	3816.0 (3722.3 to 3905.4)	1577.1 (1509.7 to 1643.2)	1837.6 (1790.9 to 1885.9)	401.3 (375.6 to 427.4)	0.0 (0.0 to 0.0)	7088.3 (6914.2 to 7254.5)	2929.5 (2804.4 to 3052.4)	3413.4 (3326.6 to 3503.2)	745.4 (697.7 to 793.9)	0.0 (0.0 to 0.0)

Country	Year	2018 US Dollars per capita					2018 PPP per capita				
		Health spending	Government health spending	Out-of-pocket health spending	Prepaid private health spending	Development assistance for health	Health spending	Government health spending	Out-of-pocket health spending	Prepaid private health spending	Development assistance for health
Andorra	2002	3834.7 (3742.3 to 3923.4)	1531.9 (1463.5 to 1601.0)	1890.2 (1840.3 to 1940.0)	412.6 (387.6 to 439.7)	0.0 (0.0 to 0.0)	7123.0 (6951.4 to 7287.9)	2845.5 (2718.4 to 2973.9)	3511.1 (3418.4 to 3603.5)	766.3 (720.0 to 816.8)	0.0 (0.0 to 0.0)
Andorra	2003	3876.8 (3791.6 to 3967.5)	1490.3 (1427.0 to 1558.0)	1958.2 (1908.8 to 2007.9)	428.3 (401.3 to 456.2)	0.0 (0.0 to 0.0)	7201.2 (7043.0 to 7369.8)	2768.2 (2650.7 to 2894.1)	3637.4 (3545.7 to 3729.7)	795.6 (745.4 to 847.3)	0.0 (0.0 to 0.0)
Andorra	2004	3892.9 (3808.6 to 3980.2)	1450.5 (1390.0 to 1519.1)	2002.5 (1956.0 to 2050.4)	439.9 (412.2 to 467.7)	0.0 (0.0 to 0.0)	7231.1 (7074.6 to 7393.2)	2694.4 (2581.9 to 2821.7)	3719.6 (3633.3 to 3808.7)	817.1 (765.6 to 868.7)	0.0 (0.0 to 0.0)
Andorra	2005	4040.6 (3952.0 to 4132.4)	1509.8 (1447.3 to 1580.3)	2069.6 (2022.8 to 2117.7)	461.2 (431.4 to 490.8)	0.0 (0.0 to 0.0)	7505.6 (7341.0 to 7675.9)	2804.5 (2688.4 to 2935.4)	3844.4 (3757.4 to 3933.8)	856.7 (801.3 to 911.7)	0.0 (0.0 to 0.0)
Andorra	2006	4090.5 (3994.5 to 4174.6)	1549.4 (1481.2 to 1615.6)	2076.9 (2027.3 to 2129.2)	464.1 (435.4 to 491.9)	0.0 (0.0 to 0.0)	7598.1 (7420.0 to 7754.3)	2878.1 (2751.4 to 3001.1)	3857.9 (3765.8 to 3955.0)	862.2 (808.7 to 913.8)	0.0 (0.0 to 0.0)
Andorra	2007	4027.5 (3932.4 to 4112.5)	1576.7 (1506.9 to 1647.9)	2003.2 (1954.8 to 2054.1)	447.6 (419.7 to 475.8)	0.0 (0.0 to 0.0)	7481.1 (7304.5 to 7639.0)	2928.7 (2799.1 to 3061.0)	3720.9 (3631.1 to 3815.5)	831.5 (779.5 to 883.9)	0.0 (0.0 to 0.0)
Andorra	2008	3781.2 (3694.1 to 3859.5)	1546.3 (1478.7 to 1613.2)	1830.3 (1782.2 to 1877.4)	404.6 (376.7 to 431.8)	0.0 (0.0 to 0.0)	7023.6 (6861.9 to 7169.1)	2872.3 (2746.7 to 2996.6)	3399.8 (3310.4 to 3487.3)	751.5 (699.8 to 802.1)	0.0 (0.0 to 0.0)
Andorra	2009	3741.7 (3656.2 to 3824.1)	1612.3 (1545.3 to 1679.3)	1744.1 (1700.9 to 1791.3)	385.3 (359.0 to 412.3)	0.0 (0.0 to 0.0)	6950.3 (6791.5 to 7103.4)	2994.9 (2870.5 to 3119.3)	3239.6 (3159.5 to 3327.4)	715.8 (666.9 to 765.8)	0.0 (0.0 to 0.0)
Andorra	2010	3746.9 (3664.8 to 3829.6)	1672.1 (1606.4 to 1738.4)	1699.1 (1654.3 to 1744.3)	375.7 (349.9 to 404.6)	0.0 (0.0 to 0.0)	6960.0 (6807.4 to 7113.6)	3106.0 (2984.0 to 3229.2)	3156.0 (3072.8 to 3240.1)	697.9 (650.0 to 751.5)	0.0 (0.0 to 0.0)
Andorra	2011	3859.3 (3772.5 to 3948.0)	1758.0 (1689.8 to 1829.8)	1719.7 (1676.1 to 1766.2)	381.6 (355.2 to 408.7)	0.0 (0.0 to 0.0)	7168.7 (7007.5 to 7333.6)	3265.5 (3138.8 to 3399.0)	3194.4 (3113.3 to 3280.7)	708.8 (659.8 to 759.2)	0.0 (0.0 to 0.0)
Andorra	2012	4012.2 (3926.4 to 4097.9)	1858.0 (1790.6 to 1923.6)	1764.8 (1720.1 to 1811.0)	389.4 (364.1 to 416.7)	0.0 (0.0 to 0.0)	7452.8 (7293.3 to 7612.0)	3451.3 (3326.1 to 3573.1)	3278.2 (3195.1 to 3364.0)	723.4 (676.3 to 774.0)	0.0 (0.0 to 0.0)
Andorra	2013	4113.1 (4029.3 to 4203.2)	1939.5 (1869.5 to 2008.1)	1782.4 (1736.6 to 1830.0)	391.2 (365.1 to 418.5)	0.0 (0.0 to 0.0)	7640.2 (7484.6 to 7807.6)	3602.7 (3472.6 to 3730.0)	3310.9 (3225.7 to 3399.3)	726.6 (678.3 to 777.3)	0.0 (0.0 to 0.0)
Andorra	2014	4220.1 (4136.6 to 4315.8)	2027.8 (1958.2 to 2100.9)	1799.0 (1755.8 to 1847.7)	393.3 (365.0 to 421.3)	0.0 (0.0 to 0.0)	7839.0 (7683.9 to 8016.8)	3766.7 (3637.3 to 3902.5)	3341.7 (3261.4 to 3432.2)	730.5 (678.0 to 782.5)	0.0 (0.0 to 0.0)
Andorra	2015	4263.8 (4168.3 to 4358.8)	2076.8 (1998.8 to 2151.3)	1792.6 (1744.7 to 1841.7)	394.4 (367.2 to 422.4)	0.0 (0.0 to 0.0)	7920.1 (7742.7 to 8096.6)	3857.8 (3712.8 to 3996.1)	3329.7 (3240.9 to 3421.0)	732.6 (682.1 to 784.6)	0.0 (0.0 to 0.0)
Andorra	2016	4234.2 (4106.9 to 4356.9)	2069.9 (1973.1 to 2164.3)	1772.9 (1706.1 to 1839.3)	391.4 (355.2 to 430.2)	0.0 (0.0 to 0.0)	7865.1 (7628.6 to 8093.1)	3844.9 (3665.0 to 4020.2)	3293.2 (3169.0 to 3416.6)	727.1 (659.9 to 799.0)	0.0 (0.0 to 0.0)
Andorra	2017	4333.3 (4206.1 to 4466.7)	2117.4 (2014.0 to 2224.8)	1810.2 (1743.2 to 1878.7)	405.8 (368.1 to 446.8)	0.0 (0.0 to 0.0)	8049.2 (7812.9 to 8296.9)	3933.0 (3741.1 to 4132.7)	3362.4 (3238.1 to 3489.7)	753.7 (683.8 to 830.0)	0.0 (0.0 to 0.0)
Andorra	2018	4353.5 (4223.2 to 4477.9)	2124.6 (2022.1 to 2230.9)	1817.9 (1751.2 to 1886.0)	411.0 (372.3 to 453.1)	0.0 (0.0 to 0.0)	8086.6 (7844.7 to 8317.9)	3946.5 (3756.0 to 4144.0)	3376.8 (3253.0 to 3503.4)	763.4 (691.5 to 841.7)	0.0 (0.0 to 0.0)
Andorra	2019	4376.0 (4244.8 to 4514.6)	2137.1 (2033.0 to 2249.4)	1822.9 (1756.4 to 1891.1)	416.0 (376.6 to 459.1)	0.0 (0.0 to 0.0)	8128.5 (7884.7 to 8386.0)	3969.7 (3776.4 to 4178.2)	3386.1 (3262.6 to 3512.7)	772.7 (699.5 to 852.7)	0.0 (0.0 to 0.0)
Andorra	2020	4390.9 (4253.1 to 4526.7)	2147.2 (2039.1 to 2263.1)	1822.8 (1755.3 to 1894.8)	421.0 (382.0 to 464.0)	0.0 (0.0 to 0.0)	8156.1 (7900.1 to 8408.4)	3988.4 (3787.8 to 4203.8)	3385.8 (3260.4 to 3519.6)	781.9 (709.5 to 861.8)	0.0 (0.0 to 0.0)
Andorra	2021	4408.1 (4269.3 to 4548.4)	2158.6 (2049.0 to 2273.9)	1823.6 (1755.5 to 1900.1)	425.9 (386.5 to 469.4)	0.0 (0.0 to 0.0)	8188.1 (7930.3 to 8448.8)	4009.6 (3806.1 to 4223.9)	3387.3 (3260.9 to 3529.5)	791.1 (717.9 to 871.9)	0.0 (0.0 to 0.0)
Andorra	2022	4437.3 (4295.9 to 4577.9)	2178.4 (2066.2 to 2294.0)	1827.6 (1757.6 to 1903.9)	431.3 (391.7 to 475.1)	0.0 (0.0 to 0.0)	8242.5 (7979.7 to 8503.5)	4046.4 (3838.1 to 4261.2)	3394.8 (3264.9 to 3536.6)	801.2 (727.6 to 882.5)	0.0 (0.0 to 0.0)

Country	Year	2018 US Dollars per capita					2018 PPP per capita				
		Health spending	Government health spending	Out-of-pocket health spending	Prepaid private health spending	Development assistance for health	Health spending	Government health spending	Out-of-pocket health spending	Prepaid private health spending	Development assistance for health
Andorra	2023	4486.4 (4345.0 to 4638.5)	2214.8 (2091.5 to 2335.7)	1834.5 (1762.4 to 1909.4)	437.1 (396.8 to 481.7)	0.0 (0.0 to 0.0)	8333.6 (8070.9 to 8616.1)	4114.1 (3885.0 to 4338.6)	3407.6 (3273.7 to 3546.7)	811.9 (737.0 to 894.8)	0.0 (0.0 to 0.0)
Andorra	2024	4508.6 (4352.2 to 4657.0)	2237.5 (2108.6 to 2363.6)	1830.8 (1756.3 to 1907.5)	440.3 (399.7 to 485.6)	0.0 (0.0 to 0.0)	8374.9 (8084.3 to 8650.5)	4156.2 (3916.8 to 4390.5)	3400.8 (3262.4 to 3543.2)	817.9 (742.5 to 901.9)	0.0 (0.0 to 0.0)
Andorra	2025	4531.1 (4384.5 to 4693.3)	2260.0 (2133.5 to 2395.6)	1827.6 (1753.1 to 1904.4)	443.5 (402.7 to 489.4)	0.0 (0.0 to 0.0)	8416.5 (8144.3 to 8718.0)	4198.1 (3963.0 to 4449.9)	3394.7 (3256.3 to 3537.4)	823.7 (748.0 to 909.1)	0.0 (0.0 to 0.0)
Andorra	2026	4552.6 (4386.6 to 4723.1)	2282.7 (2150.1 to 2423.6)	1823.5 (1744.0 to 1903.2)	446.4 (404.9 to 494.0)	0.0 (0.0 to 0.0)	8456.6 (8148.2 to 8773.3)	4240.2 (3993.8 to 4501.9)	3387.2 (3239.4 to 3535.3)	829.2 (752.1 to 917.6)	0.0 (0.0 to 0.0)
Andorra	2027	4568.7 (4409.9 to 4752.7)	2300.9 (2163.8 to 2450.9)	1818.5 (1735.7 to 1901.8)	449.3 (406.9 to 498.2)	0.0 (0.0 to 0.0)	8486.4 (8191.5 to 8828.3)	4274.0 (4019.4 to 4552.6)	3377.8 (3224.2 to 3532.7)	834.5 (755.9 to 925.4)	0.0 (0.0 to 0.0)
Andorra	2028	4590.0 (4412.4 to 4782.3)	2323.9 (2179.7 to 2493.0)	1814.0 (1725.6 to 1900.4)	452.1 (409.3 to 502.5)	0.0 (0.0 to 0.0)	8526.0 (8196.2 to 8883.1)	4316.7 (4048.9 to 4630.8)	3369.5 (3205.3 to 3530.0)	839.9 (760.2 to 933.3)	0.0 (0.0 to 0.0)
Andorra	2029	4608.1 (4434.6 to 4808.3)	2345.7 (2192.5 to 2517.3)	1807.8 (1713.1 to 1895.4)	454.6 (411.6 to 506.7)	0.0 (0.0 to 0.0)	8559.7 (8237.4 to 8931.5)	4357.2 (4072.7 to 4676.0)	3358.1 (3182.1 to 3520.8)	844.4 (764.6 to 941.3)	0.0 (0.0 to 0.0)
Andorra	2030	4647.1 (4453.7 to 4851.4)	2378.5 (2219.0 to 2557.4)	1809.6 (1706.6 to 1901.7)	459.0 (415.6 to 511.7)	0.0 (0.0 to 0.0)	8632.0 (8272.8 to 9011.7)	4418.1 (4121.8 to 4750.4)	3361.3 (3170.1 to 3532.4)	852.6 (772.0 to 950.4)	0.0 (0.0 to 0.0)
Andorra	2031	4691.0 (4504.7 to 4912.9)	2413.7 (2248.1 to 2601.4)	1813.3 (1699.3 to 1911.1)	464.0 (418.9 to 517.1)	0.0 (0.0 to 0.0)	8713.7 (8367.5 to 9125.7)	4483.6 (4175.9 to 4832.1)	3368.2 (3156.4 to 3550.0)	861.9 (778.1 to 960.5)	0.0 (0.0 to 0.0)
Andorra	2032	4734.0 (4511.6 to 4954.7)	2447.3 (2266.7 to 2657.7)	1817.5 (1696.8 to 1921.2)	469.2 (422.5 to 523.3)	0.0 (0.0 to 0.0)	8793.5 (8380.5 to 9203.4)	4545.9 (4210.5 to 4936.8)	3376.1 (3151.8 to 3568.7)	871.6 (784.8 to 972.1)	0.0 (0.0 to 0.0)
Andorra	2033	4776.9 (4558.4 to 5016.5)	2480.8 (2292.9 to 2680.2)	1821.6 (1690.3 to 1932.9)	474.5 (426.4 to 531.0)	0.0 (0.0 to 0.0)	8873.1 (8467.4 to 9318.2)	4608.1 (4259.1 to 4978.5)	3383.8 (3139.8 to 3590.4)	881.3 (792.0 to 986.3)	0.0 (0.0 to 0.0)
Andorra	2034	4820.3 (4570.2 to 5071.1)	2514.6 (2317.5 to 2724.7)	1825.9 (1684.5 to 1942.6)	479.8 (430.3 to 537.8)	0.0 (0.0 to 0.0)	8953.8 (8489.3 to 9419.7)	4670.9 (4304.8 to 5061.2)	3391.6 (3129.0 to 3608.4)	891.2 (799.4 to 998.9)	0.0 (0.0 to 0.0)
Andorra	2035	4861.2 (4619.8 to 5128.6)	2547.8 (2334.9 to 2763.6)	1828.7 (1677.8 to 1950.5)	484.8 (433.9 to 543.9)	0.0 (0.0 to 0.0)	9029.9 (8581.3 to 9526.5)	4732.5 (4337.1 to 5133.5)	3396.8 (3116.5 to 3623.2)	900.5 (806.0 to 1010.2)	0.0 (0.0 to 0.0)
Andorra	2036	4909.1 (4624.7 to 5179.4)	2585.3 (2372.2 to 2817.5)	1833.4 (1674.7 to 1960.2)	490.3 (438.0 to 550.3)	0.0 (0.0 to 0.0)	9118.7 (8590.5 to 9620.9)	4802.3 (4406.4 to 5233.6)	3405.7 (3110.9 to 3641.2)	910.8 (813.7 to 1022.2)	0.0 (0.0 to 0.0)
Andorra	2037	4949.3 (4694.1 to 5244.8)	2618.2 (2395.9 to 2867.8)	1835.8 (1670.0 to 1966.2)	495.3 (441.4 to 557.2)	0.0 (0.0 to 0.0)	9193.5 (8719.4 to 9742.4)	4863.4 (4450.5 to 5327.0)	3410.0 (3102.0 to 3652.3)	920.0 (819.9 to 1035.0)	0.0 (0.0 to 0.0)
Andorra	2038	4989.7 (4679.0 to 5288.0)	2651.2 (2411.6 to 2904.2)	1838.2 (1665.7 to 1971.8)	500.3 (445.2 to 562.8)	0.0 (0.0 to 0.0)	9268.5 (8691.4 to 9822.7)	4924.7 (4479.6 to 5394.7)	3414.4 (3094.0 to 3662.7)	929.4 (827.0 to 1045.5)	0.0 (0.0 to 0.0)
Andorra	2039	5032.7 (4742.5 to 5351.1)	2685.9 (2433.1 to 2960.2)	1841.2 (1662.6 to 1980.4)	505.6 (449.0 to 569.3)	0.0 (0.0 to 0.0)	9348.4 (8809.2 to 9939.7)	4989.1 (4519.6 to 5498.7)	3420.1 (3088.3 to 3678.7)	939.2 (834.1 to 1057.4)	0.0 (0.0 to 0.0)
Andorra	2040	5071.6 (4742.9 to 5390.8)	2718.4 (2462.9 to 3001.4)	1842.7 (1658.6 to 1985.9)	510.5 (452.3 to 575.6)	0.0 (0.0 to 0.0)	9420.6 (8810.0 to 10013.6)	5049.4 (4574.9 to 5575.2)	3422.9 (3080.9 to 3688.8)	948.3 (840.1 to 1069.3)	0.0 (0.0 to 0.0)
Andorra	2041	5112.0 (4788.5 to 5466.9)	2751.7 (2478.2 to 3047.7)	1844.7 (1656.5 to 1990.5)	515.6 (455.2 to 582.9)	0.0 (0.0 to 0.0)	9495.7 (8894.7 to 10154.9)	5111.4 (4603.2 to 5661.1)	3426.6 (3076.9 to 3697.5)	957.7 (845.6 to 1082.7)	0.0 (0.0 to 0.0)
Andorra	2042	5146.2 (4796.0 to 5489.9)	2779.9 (2488.4 to 3101.8)	1845.8 (1653.8 to 1994.5)	520.5 (457.9 to 590.5)	0.0 (0.0 to 0.0)	9559.2 (8908.7 to 10197.6)	5163.8 (4622.3 to 5761.6)	3428.6 (3072.0 to 3704.8)	966.9 (850.6 to 1096.8)	0.0 (0.0 to 0.0)
Andorra	2043	5185.5 (4832.0 to 5564.4)	2810.6 (2508.7 to 3146.3)	1848.8 (1653.2 to 1998.9)	526.1 (462.5 to 598.2)	0.0 (0.0 to 0.0)	9632.2 (8975.6 to 10336.0)	5220.8 (4660.0 to 5844.3)	3434.2 (3070.9 to 3713.1)	977.2 (859.1 to 1111.1)	0.0 (0.0 to 0.0)

Country	Year	2018 US Dollars per capita					2018 PPP per capita				
		Health spending	Government health spending	Out-of-pocket health spending	Prepaid private health spending	Development assistance for health	Health spending	Government health spending	Out-of-pocket health spending	Prepaid private health spending	Development assistance for health
Andorra	2044	5225.9 (4858.1 to 5615.9)	2842.2 (2536.5 to 3176.8)	1851.9 (1653.1 to 2001.2)	531.7 (466.3 to 607.0)	0.0 (0.0 to 0.0)	9707.2 (9024.1 to 10431.6)	5279.4 (4711.5 to 5900.9)	3440.0 (3070.7 to 3717.2)	987.7 (866.2 to 1127.5)	0.0 (0.0 to 0.0)
Andorra	2045	5262.6 (4883.4 to 5664.7)	2871.0 (2547.2 to 3216.4)	1854.4 (1652.0 to 2002.3)	537.3 (469.9 to 615.8)	0.0 (0.0 to 0.0)	9775.5 (9071.1 to 10522.4)	5332.9 (4731.5 to 5974.5)	3444.6 (3068.7 to 3719.4)	998.0 (872.9 to 1143.8)	0.0 (0.0 to 0.0)
Andorra	2046	5303.1 (4914.7 to 5701.8)	2902.2 (2563.8 to 3275.5)	1857.8 (1651.8 to 2010.9)	543.1 (473.8 to 624.0)	0.0 (0.0 to 0.0)	9850.7 (9129.2 to 10591.1)	5390.9 (4762.3 to 6084.3)	3450.9 (3068.3 to 3735.3)	1008.9 (880.2 to 1159.0)	0.0 (0.0 to 0.0)
Andorra	2047	5343.4 (4945.0 to 5763.9)	2931.6 (2580.1 to 3313.1)	1862.4 (1652.2 to 2019.0)	549.5 (478.2 to 632.7)	0.0 (0.0 to 0.0)	9925.5 (9185.5 to 10706.6)	5445.4 (4792.6 to 6154.2)	3459.4 (3069.1 to 3750.3)	1020.7 (888.2 to 1175.3)	0.0 (0.0 to 0.0)
Andorra	2048	5381.3 (4958.0 to 5812.8)	2959.5 (2594.5 to 3354.0)	1866.1 (1651.9 to 2022.9)	555.7 (482.5 to 641.3)	0.0 (0.0 to 0.0)	9995.8 (9209.6 to 10797.5)	5497.4 (4819.3 to 6230.2)	3466.3 (3068.4 to 3757.5)	1032.2 (896.3 to 1191.3)	0.0 (0.0 to 0.0)
Andorra	2049	5419.6 (5022.6 to 5851.9)	2987.1 (2613.8 to 3390.1)	1870.4 (1653.1 to 2029.4)	562.1 (486.8 to 649.7)	0.0 (0.0 to 0.0)	10067.0 (9329.5 to 10870.0)	5548.5 (4855.2 to 6297.2)	3474.3 (3070.7 to 3769.7)	1044.2 (904.3 to 1206.8)	0.0 (0.0 to 0.0)
Andorra	2050	5463.4 (5006.0 to 5931.2)	3017.5 (2635.2 to 3422.5)	1876.6 (1656.0 to 2039.3)	569.2 (491.5 to 659.7)	0.0 (0.0 to 0.0)	10148.3 (9298.7 to 11017.3)	5605.2 (4895.0 to 6357.3)	3485.8 (3076.1 to 3788.1)	1057.3 (912.9 to 1225.4)	0.0 (0.0 to 0.0)
Angola	1995	124.1 (103.1 to 148.7)	53.7 (42.9 to 67.2)	40.4 (30.2 to 53.7)	26.3 (14.8 to 43.5)	3.7 (3.7 to 3.7)	206.3 (171.5 to 247.3)	89.3 (71.3 to 111.8)	67.2 (50.2 to 89.3)	43.7 (24.6 to 72.3)	6.1 (6.1 to 6.1)
Angola	1996	95.6 (80.9 to 112.2)	44.0 (35.3 to 54.6)	26.5 (19.7 to 35.3)	17.9 (10.2 to 29.5)	7.1 (7.1 to 7.1)	158.9 (134.5 to 186.5)	73.3 (58.7 to 90.8)	44.0 (32.7 to 58.7)	29.8 (16.9 to 49.0)	11.9 (11.9 to 11.9)
Angola	1997	84.2 (70.8 to 98.8)	40.6 (32.8 to 50.4)	25.7 (18.9 to 33.8)	14.5 (8.1 to 23.5)	3.5 (3.5 to 3.5)	140.0 (117.7 to 164.3)	67.5 (54.5 to 83.8)	42.7 (31.5 to 56.2)	24.1 (13.5 to 39.1)	5.7 (5.7 to 5.7)
Angola	1998	81.9 (69.0 to 95.6)	39.8 (31.9 to 49.1)	27.1 (20.1 to 35.3)	13.0 (7.4 to 21.0)	2.0 (2.0 to 2.0)	136.2 (114.7 to 159.1)	66.1 (53.0 to 81.6)	45.1 (33.4 to 58.8)	21.6 (12.3 to 34.9)	3.3 (3.3 to 3.3)
Angola	1999	82.0 (69.2 to 96.2)	41.5 (33.5 to 51.3)	26.3 (19.6 to 34.0)	12.0 (6.8 to 20.0)	2.3 (2.3 to 2.3)	136.4 (115.1 to 159.9)	69.0 (55.7 to 85.3)	43.7 (32.5 to 56.5)	19.9 (11.2 to 33.2)	3.8 (3.8 to 3.8)
Angola	2000	82.2 (69.7 to 96.0)	40.8 (33.0 to 50.3)	28.7 (21.4 to 36.8)	10.4 (5.7 to 17.0)	2.3 (2.3 to 2.3)	136.7 (115.9 to 159.7)	67.9 (54.9 to 83.7)	47.6 (35.7 to 61.3)	17.3 (9.5 to 28.2)	3.9 (3.9 to 3.9)
Angola	2001	102.3 (87.0 to 119.5)	46.2 (37.8 to 56.9)	42.2 (32.2 to 54.8)	11.1 (6.1 to 18.2)	2.8 (2.8 to 2.8)	170.2 (144.6 to 198.7)	76.9 (62.9 to 94.6)	70.2 (53.5 to 91.1)	18.4 (10.2 to 30.3)	4.7 (4.7 to 4.7)
Angola	2002	101.2 (86.7 to 118.3)	43.4 (35.0 to 53.3)	45.4 (34.7 to 59.4)	10.0 (5.4 to 16.4)	2.4 (2.4 to 2.4)	168.3 (144.2 to 196.7)	72.2 (58.2 to 88.6)	75.5 (57.6 to 98.9)	16.6 (9.0 to 27.3)	4.0 (4.0 to 4.0)
Angola	2003	102.5 (87.3 to 119.5)	43.3 (35.5 to 52.6)	46.9 (35.9 to 61.2)	9.7 (5.3 to 15.6)	2.6 (2.6 to 2.6)	170.4 (145.2 to 198.8)	72.1 (59.0 to 87.5)	78.0 (59.8 to 101.7)	16.1 (8.8 to 26.0)	4.3 (4.3 to 4.3)
Angola	2004	106.9 (90.9 to 124.6)	46.8 (38.2 to 57.0)	47.0 (35.9 to 61.0)	10.1 (5.5 to 16.6)	3.1 (3.1 to 3.1)	177.8 (151.2 to 207.2)	77.8 (63.6 to 94.8)	78.1 (59.6 to 101.4)	16.8 (9.1 to 27.5)	5.2 (5.2 to 5.2)
Angola	2005	112.2 (96.1 to 130.1)	51.6 (42.4 to 62.4)	42.9 (32.5 to 55.4)	10.6 (5.8 to 17.8)	7.1 (7.1 to 7.1)	186.6 (159.8 to 216.3)	85.8 (70.5 to 103.8)	71.3 (54.1 to 92.2)	17.7 (9.7 to 29.7)	11.8 (11.8 to 11.8)
Angola	2006	115.0 (98.4 to 133.4)	61.1 (50.4 to 73.7)	38.5 (29.0 to 49.9)	12.0 (6.5 to 20.0)	3.5 (3.5 to 3.5)	191.3 (163.6 to 221.9)	101.5 (83.9 to 122.5)	64.0 (48.2 to 83.0)	20.0 (10.7 to 33.3)	5.8 (5.8 to 5.8)
Angola	2007	126.6 (109.0 to 147.1)	72.2 (59.5 to 86.8)	35.9 (26.8 to 46.5)	14.4 (8.1 to 23.7)	4.1 (4.1 to 4.1)	210.5 (181.2 to 244.6)	120.1 (99.0 to 144.4)	59.7 (44.6 to 77.4)	23.9 (13.5 to 39.4)	6.9 (6.9 to 6.9)
Angola	2008	140.3 (120.7 to 163.1)	84.5 (69.9 to 101.0)	32.9 (24.6 to 42.6)	17.4 (9.5 to 29.1)	5.4 (5.4 to 5.4)	233.3 (200.8 to 271.3)	140.6 (116.3 to 167.9)	54.7 (40.8 to 70.8)	29.0 (15.8 to 48.3)	9.0 (9.0 to 9.0)

		2018 US Dollars per capita					2018 PPP per capita				
Country	Year	Health spending	Government health spending	Out-of-pocket health spending	Prepaid private health spending	Development assistance for health	Health spending	Government health spending	Out-of-pocket health spending	Prepaid private health spending	Development assistance for health
Angola	2009	131.6 (112.3 to 153.7)	81.8 (67.6 to 99.1)	27.4 (20.4 to 35.4)	18.0 (9.7 to 30.0)	4.4 (4.4 to 4.4)	218.8 (186.7 to 255.7)	136.1 (112.4 to 164.8)	45.6 (34.0 to 58.9)	29.9 (16.1 to 49.9)	7.3 (7.3 to 7.3)
Angola	2010	125.5 (107.8 to 146.9)	78.5 (64.5 to 94.7)	25.0 (18.3 to 32.6)	17.8 (9.8 to 29.7)	4.3 (4.3 to 4.3)	208.7 (179.3 to 244.3)	130.5 (107.3 to 157.5)	41.6 (30.5 to 54.2)	29.5 (16.3 to 49.5)	7.1 (7.1 to 7.1)
Angola	2011	124.0 (106.3 to 145.8)	78.3 (65.0 to 94.5)	24.4 (17.9 to 32.0)	18.1 (10.2 to 30.0)	3.2 (3.2 to 3.2)	206.2 (176.8 to 242.4)	130.2 (108.1 to 157.1)	40.6 (29.8 to 53.2)	30.1 (16.9 to 49.9)	5.3 (5.3 to 5.3)
Angola	2012	122.5 (105.1 to 143.0)	75.0 (62.0 to 90.3)	24.7 (18.2 to 32.9)	18.1 (10.1 to 30.5)	4.8 (4.8 to 4.8)	203.7 (174.8 to 237.7)	124.7 (103.2 to 150.2)	41.1 (30.2 to 54.6)	30.0 (16.8 to 50.7)	7.9 (7.9 to 7.9)
Angola	2013	126.4 (108.4 to 148.5)	73.7 (60.8 to 88.8)	28.3 (20.8 to 37.9)	19.0 (10.4 to 32.7)	5.3 (5.3 to 5.3)	210.1 (180.3 to 246.9)	122.5 (101.0 to 147.7)	47.1 (34.5 to 63.0)	31.7 (17.3 to 54.4)	8.9 (8.9 to 8.9)
Angola	2014	123.5 (104.8 to 145.5)	68.1 (55.9 to 82.3)	32.2 (23.7 to 43.0)	19.5 (10.7 to 33.3)	3.6 (3.6 to 3.6)	205.4 (174.4 to 241.9)	113.3 (92.9 to 136.9)	53.6 (39.3 to 71.5)	32.4 (17.7 to 55.4)	6.1 (6.1 to 6.1)
Angola	2015	123.5 (103.8 to 145.9)	63.0 (50.7 to 76.3)	36.6 (26.7 to 49.0)	20.0 (10.5 to 34.0)	3.9 (3.9 to 3.9)	205.5 (172.5 to 242.6)	104.8 (84.3 to 127.0)	60.9 (44.3 to 81.4)	33.2 (17.4 to 56.6)	6.5 (6.5 to 6.5)
Angola	2016	120.8 (100.4 to 142.5)	58.3 (46.7 to 71.2)	38.3 (27.6 to 52.0)	19.9 (10.3 to 34.0)	4.3 (4.3 to 4.3)	201.0 (167.0 to 237.0)	96.9 (77.6 to 118.4)	63.8 (46.0 to 86.5)	33.1 (17.1 to 56.5)	7.2 (7.2 to 7.2)
Angola	2017	117.5 (97.6 to 138.5)	55.5 (44.5 to 67.8)	38.4 (27.9 to 52.5)	19.3 (10.0 to 32.9)	4.3 (4.3 to 4.3)	195.4 (162.3 to 230.4)	92.3 (74.1 to 112.8)	63.9 (46.3 to 87.4)	32.1 (16.6 to 54.7)	7.1 (7.1 to 7.1)
Angola	2018	118.4 (98.5 to 139.7)	55.9 (44.9 to 68.2)	39.0 (28.2 to 53.2)	19.5 (10.1 to 33.2)	4.0 (4.0 to 4.0)	196.9 (163.8 to 232.3)	93.0 (74.7 to 113.4)	64.9 (46.8 to 88.4)	32.4 (16.8 to 55.3)	6.6 (6.6 to 6.6)
Angola	2019	119.6 (99.5 to 141.0)	56.3 (45.2 to 69.0)	39.6 (28.7 to 54.1)	19.7 (10.2 to 33.5)	3.9 (3.7 to 4.2)	198.9 (165.4 to 234.5)	93.7 (75.1 to 114.7)	65.9 (47.7 to 90.0)	32.7 (16.9 to 55.7)	6.6 (6.1 to 6.9)
Angola	2020	120.9 (100.2 to 142.8)	56.8 (45.7 to 69.5)	40.3 (29.2 to 55.1)	19.9 (10.3 to 33.8)	3.9 (3.6 to 4.2)	201.0 (166.6 to 237.5)	94.5 (75.9 to 115.6)	67.0 (48.5 to 91.6)	33.0 (17.1 to 56.1)	6.5 (6.0 to 7.1)
Angola	2021	122.3 (101.1 to 144.3)	57.4 (46.0 to 70.3)	40.9 (29.6 to 56.1)	20.1 (10.4 to 34.1)	3.9 (3.5 to 4.4)	203.4 (168.2 to 239.9)	95.4 (76.5 to 116.9)	68.1 (49.2 to 93.4)	33.4 (17.3 to 56.8)	6.5 (5.9 to 7.2)
Angola	2022	124.2 (102.8 to 147.1)	58.2 (46.7 to 71.7)	41.7 (30.1 to 57.3)	20.3 (10.6 to 34.7)	4.0 (3.5 to 4.4)	206.5 (170.9 to 244.6)	96.8 (77.6 to 119.2)	69.3 (50.1 to 95.3)	33.8 (17.6 to 57.7)	6.6 (5.8 to 7.3)
Angola	2023	127.3 (105.6 to 150.8)	60.2 (48.3 to 74.0)	42.5 (30.8 to 58.5)	20.6 (10.7 to 35.1)	4.0 (3.5 to 4.5)	211.6 (175.6 to 250.8)	100.0 (80.3 to 123.1)	70.7 (51.2 to 97.2)	34.3 (17.8 to 58.4)	6.6 (5.8 to 7.5)
Angola	2024	130.2 (108.3 to 154.7)	61.9 (49.5 to 76.2)	43.4 (31.4 to 59.4)	20.9 (10.9 to 35.8)	4.0 (3.5 to 4.6)	216.4 (180.1 to 257.3)	102.9 (82.3 to 126.7)	72.1 (52.2 to 98.7)	34.8 (18.1 to 59.5)	6.7 (5.7 to 7.7)
Angola	2025	131.8 (109.6 to 156.7)	62.4 (49.9 to 77.0)	44.2 (32.0 to 60.6)	21.2 (11.1 to 36.4)	4.0 (3.4 to 4.7)	219.1 (182.2 to 260.6)	103.7 (82.9 to 128.0)	73.4 (53.3 to 100.8)	35.2 (18.4 to 60.5)	6.7 (5.7 to 7.8)
Angola	2026	133.4 (110.9 to 158.5)	62.9 (50.3 to 77.7)	45.0 (32.7 to 61.9)	21.5 (11.2 to 37.0)	4.0 (3.4 to 4.7)	221.9 (184.3 to 263.6)	104.7 (83.7 to 129.1)	74.8 (54.4 to 103.0)	35.7 (18.7 to 61.5)	6.7 (5.7 to 7.9)
Angola	2027	135.8 (113.3 to 161.5)	64.1 (51.2 to 78.9)	45.9 (33.3 to 63.6)	21.8 (11.4 to 37.4)	4.1 (3.4 to 4.8)	225.8 (188.5 to 268.6)	106.6 (85.2 to 131.3)	76.3 (55.5 to 105.7)	36.3 (18.9 to 62.3)	6.8 (5.7 to 8.0)
Angola	2028	138.5 (115.7 to 165.8)	65.5 (52.4 to 80.8)	46.8 (33.8 to 65.1)	22.1 (11.6 to 37.9)	4.1 (3.4 to 4.9)	230.4 (192.4 to 275.7)	109.0 (87.1 to 134.3)	77.8 (56.2 to 108.3)	36.8 (19.2 to 63.0)	6.8 (5.7 to 8.2)
Angola	2029	140.1 (116.9 to 168.0)	65.9 (52.7 to 81.1)	47.6 (34.3 to 66.2)	22.5 (11.7 to 38.4)	4.1 (3.4 to 5.0)	233.0 (194.4 to 279.3)	109.6 (87.6 to 134.9)	79.2 (57.0 to 110.2)	37.4 (19.5 to 63.8)	6.9 (5.7 to 8.4)

		2018 US Dollars per capita					2018 PPP per capita				
Country	Year	Health spending	Government health spending	Out-of-pocket health spending	Prepaid private health spending	Development assistance for health	Health spending	Government health spending	Out-of-pocket health spending	Prepaid private health spending	Development assistance for health
Angola	2030	142.1 (117.8 to 170.2)	66.7 (52.9 to 82.2)	48.5 (34.7 to 67.3)	22.8 (11.9 to 39.0)	4.2 (3.4 to 5.1)	236.3 (195.8 to 283.0)	110.9 (88.0 to 136.8)	80.6 (57.7 to 111.8)	37.9 (19.8 to 64.9)	6.9 (5.7 to 8.5)
Angola	2031	143.6 (119.3 to 172.5)	67.0 (53.1 to 83.3)	49.3 (35.0 to 68.3)	23.1 (12.1 to 39.7)	4.2 (3.4 to 5.3)	238.8 (198.4 to 286.8)	111.4 (88.4 to 138.5)	81.9 (58.2 to 113.6)	38.5 (20.1 to 66.1)	7.0 (5.6 to 8.7)
Angola	2032	145.0 (120.1 to 173.6)	67.2 (53.2 to 83.5)	50.1 (35.4 to 69.8)	23.5 (12.3 to 40.3)	4.2 (3.4 to 5.3)	241.2 (199.8 to 288.7)	111.8 (88.5 to 138.8)	83.3 (58.8 to 116.1)	39.0 (20.4 to 67.1)	7.0 (5.6 to 8.8)
Angola	2033	146.6 (121.2 to 174.6)	67.6 (53.6 to 83.9)	50.9 (35.7 to 71.4)	23.8 (12.4 to 40.9)	4.3 (3.4 to 5.5)	243.8 (201.5 to 290.3)	112.4 (89.1 to 139.6)	84.6 (59.3 to 118.8)	39.6 (20.7 to 68.0)	7.1 (5.6 to 9.1)
Angola	2034	148.3 (122.6 to 178.1)	68.1 (54.0 to 84.6)	51.7 (36.0 to 72.7)	24.2 (12.6 to 41.5)	4.3 (3.4 to 5.6)	246.6 (203.9 to 296.1)	113.2 (89.7 to 140.7)	86.0 (59.8 to 120.9)	40.2 (21.0 to 69.0)	7.2 (5.7 to 9.3)
Angola	2035	150.1 (123.3 to 180.8)	68.6 (54.2 to 85.0)	52.6 (36.3 to 74.1)	24.5 (12.8 to 42.1)	4.4 (3.4 to 5.7)	249.5 (205.0 to 300.6)	114.0 (90.2 to 141.4)	87.4 (60.4 to 123.2)	40.8 (21.3 to 70.0)	7.3 (5.7 to 9.5)
Angola	2036	151.7 (124.2 to 182.1)	69.0 (54.3 to 85.9)	53.4 (36.7 to 75.5)	24.9 (13.0 to 42.7)	4.4 (3.4 to 5.9)	252.2 (206.5 to 302.8)	114.7 (90.4 to 142.9)	88.7 (61.0 to 125.5)	41.4 (21.6 to 71.0)	7.4 (5.7 to 9.9)
Angola	2037	153.2 (125.9 to 183.9)	69.3 (54.6 to 86.4)	54.2 (37.1 to 77.0)	25.3 (13.2 to 43.3)	4.5 (3.4 to 6.0)	254.8 (209.3 to 305.9)	115.2 (90.8 to 143.7)	90.1 (61.7 to 128.0)	42.1 (21.9 to 72.0)	7.4 (5.7 to 10.0)
Angola	2038	154.6 (126.5 to 185.6)	69.5 (54.5 to 86.8)	54.9 (37.4 to 78.5)	25.7 (13.4 to 43.9)	4.5 (3.5 to 6.3)	257.1 (210.3 to 308.6)	115.6 (90.6 to 144.3)	91.4 (62.2 to 130.5)	42.7 (22.3 to 73.0)	7.5 (5.7 to 10.4)
Angola	2039	156.4 (128.2 to 188.4)	70.0 (54.8 to 87.6)	55.7 (37.7 to 79.8)	26.0 (13.6 to 44.6)	4.6 (3.5 to 6.4)	260.0 (213.2 to 313.3)	116.4 (91.2 to 145.7)	92.7 (62.8 to 132.7)	43.3 (22.6 to 74.2)	7.6 (5.8 to 10.6)
Angola	2040	158.0 (128.9 to 191.3)	70.4 (55.0 to 88.3)	56.5 (38.1 to 81.0)	26.4 (13.8 to 45.3)	4.7 (3.5 to 6.4)	262.7 (214.3 to 318.1)	117.0 (91.5 to 146.9)	94.0 (63.4 to 134.7)	43.9 (22.9 to 75.3)	7.7 (5.9 to 10.7)
Angola	2041	159.5 (131.0 to 193.1)	70.7 (54.9 to 88.8)	57.3 (38.6 to 82.2)	26.8 (14.0 to 46.0)	4.7 (3.6 to 6.6)	265.3 (217.9 to 321.1)	117.6 (91.4 to 147.6)	95.3 (64.1 to 136.7)	44.6 (23.2 to 76.5)	7.9 (5.9 to 10.9)
Angola	2042	161.0 (132.6 to 194.1)	71.0 (55.2 to 89.6)	58.0 (38.9 to 83.3)	27.2 (14.1 to 46.6)	4.8 (3.6 to 6.9)	267.7 (220.5 to 322.8)	118.1 (91.7 to 148.9)	96.5 (64.7 to 138.6)	45.2 (23.5 to 77.6)	8.0 (6.0 to 11.4)
Angola	2043	162.8 (133.3 to 196.9)	71.5 (55.6 to 90.4)	58.8 (39.2 to 84.8)	27.6 (14.3 to 47.3)	4.9 (3.6 to 7.1)	270.7 (221.6 to 327.4)	119.0 (92.4 to 150.4)	97.8 (65.2 to 141.0)	45.8 (23.8 to 78.6)	8.1 (6.0 to 11.7)
Angola	2044	164.5 (133.8 to 200.1)	72.1 (55.7 to 90.9)	59.6 (39.7 to 86.1)	27.9 (14.4 to 47.9)	5.0 (3.7 to 7.2)	273.6 (222.6 to 332.7)	119.8 (92.7 to 151.1)	99.0 (66.0 to 143.2)	46.5 (24.0 to 79.7)	8.3 (6.1 to 11.9)
Angola	2045	166.3 (135.6 to 202.6)	72.5 (56.0 to 91.9)	60.3 (40.1 to 87.1)	28.4 (14.6 to 48.6)	5.1 (3.6 to 7.6)	276.5 (225.6 to 336.9)	120.6 (93.2 to 152.9)	100.3 (66.8 to 144.9)	47.2 (24.3 to 80.9)	8.4 (6.1 to 12.7)
Angola	2046	168.0 (136.8 to 203.9)	73.0 (56.3 to 92.6)	61.0 (40.6 to 88.0)	28.8 (14.8 to 49.3)	5.1 (3.7 to 7.9)	279.3 (227.5 to 339.1)	121.4 (93.6 to 154.0)	101.5 (67.5 to 146.4)	47.9 (24.6 to 82.0)	8.5 (6.2 to 13.1)
Angola	2047	170.2 (139.0 to 207.6)	73.9 (56.9 to 94.0)	61.8 (41.2 to 89.2)	29.2 (15.1 to 50.1)	5.2 (3.8 to 8.3)	283.1 (231.1 to 345.3)	122.9 (94.6 to 156.4)	102.8 (68.5 to 148.3)	48.6 (25.0 to 83.3)	8.7 (6.3 to 13.7)
Angola	2048	172.3 (139.7 to 211.0)	74.6 (57.6 to 94.9)	62.6 (41.8 to 90.4)	29.7 (15.3 to 50.9)	5.3 (3.8 to 8.5)	286.5 (232.3 to 350.8)	124.0 (95.8 to 157.9)	104.2 (69.5 to 150.4)	49.4 (25.4 to 84.6)	8.9 (6.4 to 14.2)
Angola	2049	174.9 (141.9 to 215.1)	75.8 (58.3 to 96.5)	63.5 (42.4 to 91.7)	30.2 (15.5 to 51.6)	5.4 (3.9 to 8.8)	290.8 (236.0 to 357.7)	126.0 (96.9 to 160.5)	105.6 (70.6 to 152.5)	50.2 (25.8 to 85.9)	9.1 (6.5 to 14.6)
Angola	2050	177.3 (143.0 to 216.6)	76.7 (59.1 to 97.8)	64.3 (43.0 to 93.0)	30.7 (15.7 to 52.5)	5.6 (3.9 to 9.1)	294.8 (237.8 to 360.2)	127.6 (98.3 to 162.6)	107.0 (71.5 to 154.7)	51.0 (26.1 to 87.3)	9.3 (6.5 to 15.2)

		2018 US Dollars per capita					2018 PPP per capita				
Country	Year	Health spending	Government health spending	Out-of-pocket health spending	Prepaid private health spending	Development assistance for health	Health spending	Government health spending	Out-of-pocket health spending	Prepaid private health spending	Development assistance for health
Antigua and Barbuda	1995	454.4 (411.2 to 501.4)	292.8 (261.2 to 328.9)	135.7 (113.5 to 157.7)	26.0 (15.2 to 42.3)	0.0 (0.0 to 0.0)	737.5 (667.3 to 813.7)	475.2 (423.9 to 533.8)	220.2 (184.2 to 256.0)	42.1 (24.6 to 68.6)	0.0 (0.0 to 0.0)
Antigua and Barbuda	1996	461.1 (422.4 to 501.5)	297.0 (269.1 to 328.3)	137.9 (117.1 to 158.6)	26.0 (15.6 to 40.9)	0.1 (0.1 to 0.1)	748.3 (685.5 to 813.8)	482.1 (436.8 to 532.9)	223.9 (190.1 to 257.4)	42.2 (25.4 to 66.4)	0.2 (0.2 to 0.2)
Antigua and Barbuda	1997	469.9 (432.8 to 510.0)	307.5 (279.9 to 337.1)	136.4 (118.0 to 155.8)	25.9 (15.9 to 39.9)	0.1 (0.1 to 0.1)	762.6 (702.3 to 827.6)	499.0 (454.3 to 547.1)	221.4 (191.6 to 252.9)	42.0 (25.9 to 64.8)	0.2 (0.2 to 0.2)
Antigua and Barbuda	1998	492.8 (456.8 to 531.0)	316.1 (288.9 to 345.4)	133.5 (116.1 to 152.0)	25.7 (16.7 to 37.8)	17.5 (17.5 to 17.5)	799.8 (741.3 to 861.7)	513.0 (468.8 to 560.5)	216.7 (188.4 to 246.6)	41.7 (27.0 to 61.3)	28.4 (28.4 to 28.4)
Antigua and Barbuda	1999	510.7 (476.4 to 546.7)	325.1 (295.7 to 353.3)	133.5 (116.4 to 151.3)	26.9 (17.9 to 38.3)	25.2 (25.2 to 25.2)	828.8 (773.2 to 887.3)	527.6 (479.9 to 573.3)	216.7 (188.9 to 245.6)	43.6 (29.1 to 62.2)	40.9 (40.9 to 40.9)
Antigua and Barbuda	2000	534.6 (499.8 to 570.4)	345.9 (316.6 to 373.5)	142.0 (123.8 to 161.0)	30.5 (20.6 to 43.6)	16.3 (16.3 to 16.3)	867.6 (811.2 to 925.6)	561.3 (513.8 to 606.1)	230.4 (200.8 to 261.3)	49.4 (33.5 to 70.7)	26.5 (26.5 to 26.5)
Antigua and Barbuda	2001	528.6 (494.1 to 564.1)	345.6 (317.5 to 372.5)	148.6 (131.0 to 168.9)	32.9 (22.0 to 46.1)	1.5 (1.5 to 1.5)	857.8 (801.9 to 915.5)	560.8 (515.3 to 604.5)	241.2 (212.6 to 274.2)	53.3 (35.7 to 74.8)	2.4 (2.4 to 2.4)
Antigua and Barbuda	2002	543.2 (509.5 to 580.3)	353.5 (326.7 to 379.1)	154.7 (137.8 to 174.9)	35.0 (23.7 to 49.0)	0.1 (0.1 to 0.1)	881.6 (826.9 to 941.8)	573.6 (530.2 to 615.3)	251.1 (223.7 to 283.9)	56.8 (38.5 to 79.5)	0.2 (0.2 to 0.2)
Antigua and Barbuda	2003	573.2 (538.5 to 611.4)	365.1 (339.2 to 392.6)	166.7 (149.2 to 187.0)	37.9 (26.3 to 53.3)	3.6 (3.6 to 3.6)	930.3 (874.0 to 992.2)	592.5 (550.5 to 637.2)	270.5 (242.2 to 303.5)	61.5 (42.7 to 86.5)	5.8 (5.8 to 5.8)
Antigua and Barbuda	2004	597.1 (562.9 to 633.8)	374.4 (347.5 to 401.0)	177.3 (159.8 to 196.4)	39.7 (27.7 to 54.5)	5.8 (5.8 to 5.8)	969.0 (913.6 to 1028.6)	607.6 (564.0 to 650.8)	287.7 (259.3 to 318.7)	64.3 (45.0 to 88.5)	9.4 (9.4 to 9.4)
Antigua and Barbuda	2005	640.1 (603.5 to 679.3)	402.4 (376.6 to 430.4)	194.2 (176.7 to 214.2)	43.3 (31.2 to 59.0)	0.2 (0.2 to 0.2)	1038.8 (979.5 to 1102.4)	653.1 (611.2 to 698.4)	315.2 (286.8 to 347.7)	70.2 (50.7 to 95.7)	0.4 (0.4 to 0.4)
Antigua and Barbuda	2006	719.9 (682.7 to 761.0)	453.6 (426.0 to 482.7)	216.8 (197.8 to 238.4)	49.2 (36.0 to 65.3)	0.3 (0.3 to 0.3)	1168.3 (1107.9 to 1235.0)	736.2 (691.3 to 783.4)	351.8 (321.0 to 387.0)	79.9 (58.4 to 105.9)	0.4 (0.4 to 0.4)
Antigua and Barbuda	2007	790.4 (753.9 to 833.6)	505.9 (477.0 to 539.0)	230.8 (211.7 to 253.4)	53.5 (39.6 to 71.1)	0.2 (0.2 to 0.2)	1282.8 (1223.5 to 1352.9)	821.1 (774.1 to 874.7)	374.5 (343.6 to 411.3)	86.8 (64.3 to 115.4)	0.4 (0.4 to 0.4)
Antigua and Barbuda	2008	817.1 (780.0 to 860.1)	541.1 (510.6 to 575.8)	223.6 (203.8 to 245.6)	52.1 (38.0 to 69.9)	0.2 (0.2 to 0.2)	1326.0 (1265.9 to 1395.8)	878.2 (828.6 to 934.5)	362.9 (330.8 to 398.5)	84.5 (61.6 to 113.4)	0.3 (0.3 to 0.3)
Antigua and Barbuda	2009	762.7 (726.0 to 803.5)	513.3 (483.1 to 543.8)	195.2 (176.6 to 215.3)	45.1 (32.5 to 60.5)	9.1 (9.1 to 9.1)	1237.8 (1178.2 to 1304.0)	833.1 (784.1 to 882.5)	316.9 (286.6 to 349.4)	73.1 (52.7 to 98.1)	14.7 (14.7 to 14.7)
Antigua and Barbuda	2010	742.8 (704.0 to 783.6)	502.7 (472.3 to 535.4)	190.0 (171.0 to 209.8)	43.8 (32.0 to 58.9)	6.3 (6.3 to 6.3)	1205.5 (1142.5 to 1271.8)	815.9 (766.4 to 868.9)	308.3 (277.5 to 340.5)	71.0 (52.0 to 95.6)	10.3 (10.3 to 10.3)
Antigua and Barbuda	2011	743.2 (704.8 to 780.7)	498.1 (468.3 to 529.7)	191.3 (172.1 to 211.0)	44.5 (32.5 to 60.1)	9.4 (9.4 to 9.4)	1206.2 (1143.7 to 1267.1)	808.3 (760.0 to 859.7)	310.5 (279.3 to 342.5)	72.2 (52.8 to 97.6)	15.2 (15.2 to 15.2)
Antigua and Barbuda	2012	741.5 (702.9 to 778.9)	498.1 (470.0 to 529.5)	197.3 (178.0 to 217.3)	45.7 (33.6 to 61.9)	0.3 (0.3 to 0.3)	1203.4 (1140.7 to 1264.1)	808.4 (762.7 to 859.4)	320.2 (288.9 to 352.7)	74.2 (54.5 to 100.4)	0.6 (0.6 to 0.6)
Antigua and Barbuda	2013	749.2 (713.4 to 785.1)	502.5 (474.4 to 533.8)	200.2 (181.7 to 219.7)	46.3 (33.8 to 61.7)	0.3 (0.3 to 0.3)	1215.9 (1157.8 to 1274.2)	815.5 (769.9 to 866.4)	324.8 (294.9 to 356.5)	75.1 (54.9 to 100.1)	0.5 (0.5 to 0.5)
Antigua and Barbuda	2014	786.7 (750.0 to 824.9)	528.9 (498.5 to 562.2)	209.2 (190.7 to 229.2)	48.3 (35.1 to 63.9)	0.3 (0.3 to 0.3)	1276.7 (1217.1 to 1338.8)	858.4 (809.0 to 912.4)	339.5 (309.4 to 371.9)	78.4 (57.0 to 103.7)	0.5 (0.5 to 0.5)
Antigua and Barbuda	2015	769.9 (730.9 to 816.4)	512.5 (478.5 to 549.7)	209.9 (190.4 to 231.3)	47.4 (33.9 to 63.6)	0.2 (0.2 to 0.2)	1249.5 (1186.1 to 1325.0)	831.7 (776.6 to 892.1)	340.6 (309.0 to 375.4)	76.9 (55.0 to 103.2)	0.4 (0.4 to 0.4)

Country	Year	2018 US Dollars per capita					2018 PPP per capita				
		Health spending	Government health spending	Out-of-pocket health spending	Prepaid private health spending	Development assistance for health	Health spending	Government health spending	Out-of-pocket health spending	Prepaid private health spending	Development assistance for health
Antigua and Barbuda	2016	759.9 (712.4 to 811.1)	489.7 (449.6 to 531.5)	221.3 (196.1 to 247.7)	48.7 (33.9 to 67.2)	0.3 (0.3 to 0.3)	1233.3 (1156.1 to 1316.3)	794.7 (729.7 to 862.6)	359.1 (318.2 to 402.1)	79.0 (55.0 to 109.1)	0.4 (0.4 to 0.4)
Antigua and Barbuda	2017	762.8 (712.7 to 818.1)	487.6 (448.0 to 529.8)	225.0 (199.6 to 251.8)	50.0 (34.8 to 69.2)	0.2 (0.2 to 0.2)	1238.0 (1156.7 to 1327.7)	791.3 (727.0 to 859.8)	365.1 (323.9 to 408.6)	81.1 (56.5 to 112.2)	0.4 (0.4 to 0.4)
Antigua and Barbuda	2018	777.3 (727.0 to 830.2)	498.4 (456.5 to 543.7)	227.7 (202.0 to 254.9)	51.0 (35.5 to 70.2)	0.2 (0.0 to 0.2)	1261.6 (1179.9 to 1347.3)	808.9 (740.8 to 882.4)	369.6 (327.8 to 413.7)	82.7 (57.6 to 113.9)	0.3 (0.0 to 0.4)
Antigua and Barbuda	2019	789.2 (736.3 to 848.3)	506.9 (463.9 to 553.8)	230.2 (204.2 to 257.7)	52.0 (36.1 to 71.6)	0.2 (0.0 to 0.2)	1280.8 (1194.9 to 1376.7)	822.6 (752.8 to 898.8)	373.5 (331.4 to 418.2)	84.3 (58.6 to 116.3)	0.3 (0.0 to 0.4)
Antigua and Barbuda	2020	800.4 (747.6 to 853.7)	514.8 (471.4 to 562.5)	232.5 (206.0 to 260.7)	52.9 (36.7 to 72.9)	0.2 (0.0 to 0.2)	1299.0 (1213.2 to 1385.5)	835.5 (765.1 to 912.8)	377.3 (334.3 to 423.0)	85.9 (59.6 to 118.3)	0.3 (0.0 to 0.4)
Antigua and Barbuda	2021	812.6 (757.7 to 872.2)	523.8 (480.3 to 571.4)	234.7 (208.4 to 263.1)	53.9 (37.3 to 74.2)	0.2 (0.0 to 0.3)	1318.8 (1229.7 to 1415.5)	850.1 (779.5 to 927.3)	380.9 (338.2 to 426.9)	87.4 (60.6 to 120.5)	0.3 (0.0 to 0.4)
Antigua and Barbuda	2022	826.5 (771.7 to 882.3)	534.2 (490.1 to 582.2)	237.3 (209.7 to 266.3)	54.9 (38.0 to 75.7)	0.2 (0.0 to 0.3)	1341.4 (1252.3 to 1431.9)	867.0 (795.3 to 944.8)	385.0 (340.3 to 432.2)	89.0 (61.6 to 122.8)	0.3 (0.0 to 0.4)
Antigua and Barbuda	2023	838.0 (779.9 to 900.8)	542.7 (497.8 to 591.8)	239.3 (211.8 to 269.1)	55.7 (38.6 to 76.9)	0.2 (0.0 to 0.3)	1359.9 (1265.8 to 1462.0)	880.8 (807.9 to 960.4)	388.4 (343.8 to 436.7)	90.5 (62.7 to 124.8)	0.3 (0.0 to 0.5)
Antigua and Barbuda	2024	849.8 (793.4 to 909.0)	551.7 (505.5 to 601.8)	241.3 (213.2 to 271.0)	56.6 (39.3 to 78.1)	0.2 (0.0 to 0.3)	1379.2 (1287.6 to 1475.2)	895.3 (820.4 to 976.6)	391.6 (346.0 to 439.9)	91.9 (63.8 to 126.8)	0.3 (0.0 to 0.5)
Antigua and Barbuda	2025	861.1 (801.1 to 926.4)	560.3 (513.2 to 612.0)	243.1 (214.8 to 272.8)	57.5 (40.0 to 79.3)	0.2 (0.0 to 0.3)	1397.5 (1300.1 to 1503.5)	909.3 (832.8 to 993.3)	394.6 (348.6 to 442.8)	93.3 (64.9 to 128.8)	0.3 (0.0 to 0.5)
Antigua and Barbuda	2026	870.2 (811.2 to 930.9)	567.0 (519.0 to 621.1)	244.7 (216.1 to 274.5)	58.3 (40.6 to 80.6)	0.2 (0.0 to 0.3)	1412.2 (1316.5 to 1510.8)	920.3 (842.2 to 1007.9)	397.1 (350.7 to 445.5)	94.6 (65.9 to 130.8)	0.3 (0.0 to 0.5)
Antigua and Barbuda	2027	878.4 (815.2 to 944.9)	573.2 (524.1 to 628.9)	246.0 (217.4 to 276.9)	59.0 (41.2 to 81.7)	0.2 (0.0 to 0.3)	1425.6 (1322.9 to 1533.6)	930.2 (850.6 to 1020.6)	399.3 (352.8 to 449.3)	95.8 (66.9 to 132.5)	0.3 (0.0 to 0.5)
Antigua and Barbuda	2028	886.6 (823.2 to 951.0)	579.3 (529.8 to 636.9)	247.3 (218.4 to 278.4)	59.8 (41.8 to 82.6)	0.2 (0.0 to 0.3)	1438.9 (1335.9 to 1543.4)	940.2 (859.8 to 1033.6)	401.4 (354.5 to 451.8)	97.0 (67.8 to 134.0)	0.3 (0.0 to 0.6)
Antigua and Barbuda	2029	893.5 (828.7 to 964.6)	584.6 (534.0 to 643.0)	248.2 (219.1 to 279.6)	60.5 (42.3 to 83.5)	0.2 (0.0 to 0.4)	1450.1 (1345.0 to 1565.4)	948.8 (866.7 to 1043.5)	402.9 (355.7 to 453.8)	98.1 (68.7 to 135.5)	0.3 (0.0 to 0.6)
Antigua and Barbuda	2030	898.5 (832.5 to 965.1)	588.5 (536.7 to 648.1)	248.8 (218.8 to 280.8)	61.0 (42.8 to 84.4)	0.2 (0.0 to 0.4)	1458.2 (1351.0 to 1566.3)	955.1 (871.0 to 1051.8)	403.7 (355.1 to 455.8)	99.0 (69.4 to 137.0)	0.3 (0.0 to 0.6)
Antigua and Barbuda	2031	903.7 (835.8 to 977.4)	592.7 (540.3 to 654.0)	249.2 (219.1 to 282.0)	61.6 (43.1 to 85.3)	0.2 (0.0 to 0.4)	1466.7 (1356.5 to 1586.3)	962.0 (876.9 to 1061.3)	404.5 (355.6 to 457.6)	100.0 (70.0 to 138.4)	0.3 (0.0 to 0.6)
Antigua and Barbuda	2032	907.7 (839.1 to 977.5)	596.0 (542.9 to 658.8)	249.3 (219.2 to 282.7)	62.1 (43.3 to 86.1)	0.2 (0.0 to 0.4)	1473.0 (1361.7 to 1586.5)	967.3 (881.0 to 1069.3)	404.7 (355.7 to 458.8)	100.8 (70.3 to 139.7)	0.3 (0.0 to 0.6)
Antigua and Barbuda	2033	911.2 (840.7 to 987.2)	599.1 (544.7 to 663.3)	249.4 (218.6 to 283.5)	62.6 (43.5 to 86.7)	0.2 (0.0 to 0.4)	1478.8 (1364.4 to 1602.2)	972.3 (884.0 to 1076.5)	404.7 (354.8 to 460.1)	101.5 (70.6 to 140.7)	0.3 (0.0 to 0.7)
Antigua and Barbuda	2034	914.1 (842.6 to 986.9)	601.6 (546.4 to 667.9)	249.3 (217.8 to 284.0)	63.0 (43.9 to 87.4)	0.2 (0.0 to 0.4)	1483.5 (1367.4 to 1601.6)	976.3 (886.8 to 1083.9)	404.6 (353.4 to 460.9)	102.3 (71.2 to 141.9)	0.3 (0.0 to 0.7)
Antigua and Barbuda	2035	918.2 (845.8 to 997.4)	605.0 (548.6 to 673.3)	249.5 (217.5 to 285.0)	63.5 (44.3 to 88.4)	0.2 (0.0 to 0.4)	1490.2 (1372.7 to 1618.6)	981.8 (890.4 to 1092.8)	405.0 (352.9 to 462.5)	103.1 (71.8 to 143.4)	0.3 (0.0 to 0.7)
Antigua and Barbuda	2036	924.6 (852.2 to 1000.9)	609.9 (552.5 to 679.8)	250.3 (217.6 to 286.7)	64.2 (44.7 to 89.3)	0.2 (0.0 to 0.5)	1500.6 (1383.0 to 1624.4)	989.9 (896.6 to 1103.3)	406.2 (353.2 to 465.4)	104.2 (72.6 to 144.8)	0.3 (0.0 to 0.7)

		2018 US Dollars per capita					2018 PPP per capita				
Country	Year	Health spending	Government health spending	Out-of-pocket health spending	Prepaid private health spending	Development assistance for health	Health spending	Government health spending	Out-of-pocket health spending	Prepaid private health spending	Development assistance for health
Antigua and Barbuda	2037	931.1 (856.2 to 1015.8)	614.9 (555.8 to 687.1)	251.1 (218.0 to 288.5)	64.9 (45.3 to 90.0)	0.2 (0.0 to 0.5)	1511.1 (1389.5 to 1648.5)	998.0 (902.0 to 1115.1)	407.5 (353.8 to 468.2)	105.3 (73.4 to 146.0)	0.3 (0.0 to 0.8)
Antigua and Barbuda	2038	939.4 (862.5 to 1020.8)	621.1 (559.9 to 695.2)	252.3 (218.7 to 290.3)	65.7 (45.8 to 91.1)	0.2 (0.0 to 0.5)	1524.5 (1399.7 to 1656.6)	1008.0 (908.6 to 1128.2)	409.5 (354.9 to 471.2)	106.7 (74.3 to 147.9)	0.3 (0.0 to 0.8)
Antigua and Barbuda	2039	948.0 (868.4 to 1037.2)	627.5 (564.1 to 705.3)	253.8 (219.8 to 292.1)	66.6 (46.4 to 92.5)	0.2 (0.0 to 0.5)	1538.5 (1409.4 to 1683.2)	1018.3 (915.4 to 1144.6)	411.8 (356.7 to 474.1)	108.1 (75.4 to 150.1)	0.3 (0.0 to 0.9)
Antigua and Barbuda	2040	957.6 (877.2 to 1042.7)	634.4 (568.9 to 714.7)	255.4 (220.8 to 294.1)	67.6 (47.1 to 93.9)	0.2 (0.0 to 0.5)	1554.0 (1423.6 to 1692.3)	1029.6 (923.3 to 1159.9)	414.5 (358.3 to 477.4)	109.6 (76.5 to 152.4)	0.3 (0.0 to 0.9)
Antigua and Barbuda	2041	969.4 (882.4 to 1063.3)	643.0 (576.0 to 725.8)	257.6 (222.2 to 297.1)	68.6 (47.9 to 95.5)	0.2 (0.0 to 0.6)	1573.2 (1432.0 to 1725.7)	1043.5 (934.8 to 1177.8)	418.0 (360.5 to 482.2)	111.4 (77.7 to 155.0)	0.3 (0.0 to 0.9)
Antigua and Barbuda	2042	982.4 (897.7 to 1072.5)	652.4 (583.1 to 737.8)	260.0 (224.0 to 300.5)	69.8 (48.6 to 97.2)	0.2 (0.0 to 0.6)	1594.4 (1456.9 to 1740.7)	1058.7 (946.2 to 1197.3)	422.0 (363.5 to 487.7)	113.3 (78.9 to 157.8)	0.3 (0.0 to 1.0)
Antigua and Barbuda	2043	996.5 (905.6 to 1095.5)	662.4 (590.8 to 750.5)	262.8 (226.2 to 304.3)	71.1 (49.5 to 99.0)	0.2 (0.0 to 0.6)	1617.2 (1469.7 to 1777.9)	1075.0 (958.9 to 1218.1)	426.5 (367.1 to 493.9)	115.4 (80.3 to 160.7)	0.3 (0.0 to 1.0)
Antigua and Barbuda	2044	1010.4 (920.3 to 1105.8)	672.4 (597.1 to 763.5)	265.4 (228.2 to 307.5)	72.4 (50.3 to 100.7)	0.2 (0.0 to 0.6)	1639.8 (1493.6 to 1794.6)	1091.2 (969.1 to 1239.1)	430.8 (370.3 to 499.1)	117.5 (81.7 to 163.4)	0.3 (0.0 to 1.0)
Antigua and Barbuda	2045	1025.3 (930.1 to 1132.1)	683.4 (605.1 to 777.7)	268.0 (230.0 to 310.7)	73.7 (51.2 to 102.6)	0.2 (0.0 to 0.7)	1663.9 (1509.5 to 1837.2)	1109.1 (982.0 to 1262.1)	435.0 (373.3 to 504.2)	119.6 (83.1 to 166.5)	0.3 (0.0 to 1.1)
Antigua and Barbuda	2046	1041.0 (944.0 to 1143.4)	695.2 (614.0 to 793.9)	270.6 (232.2 to 314.0)	75.0 (52.0 to 104.7)	0.2 (0.0 to 0.7)	1689.4 (1532.1 to 1855.7)	1128.2 (996.5 to 1288.4)	439.2 (376.8 to 509.6)	121.7 (84.4 to 169.9)	0.3 (0.0 to 1.1)
Antigua and Barbuda	2047	1057.3 (956.9 to 1170.1)	707.7 (623.5 to 809.6)	273.1 (234.4 to 317.1)	76.3 (52.8 to 106.7)	0.2 (0.0 to 0.7)	1715.9 (1552.9 to 1899.0)	1148.5 (1011.8 to 1313.9)	443.3 (380.4 to 514.7)	123.9 (85.7 to 173.2)	0.3 (0.0 to 1.2)
Antigua and Barbuda	2048	1074.3 (968.6 to 1184.5)	720.9 (634.3 to 827.3)	275.6 (236.7 to 320.3)	77.7 (53.7 to 108.7)	0.2 (0.0 to 0.8)	1743.6 (1572.0 to 1922.3)	1169.9 (1029.4 to 1342.6)	447.3 (384.1 to 519.8)	126.0 (87.1 to 176.4)	0.3 (0.0 to 1.2)
Antigua and Barbuda	2049	1092.4 (983.8 to 1214.2)	735.0 (644.3 to 845.9)	278.2 (239.0 to 323.2)	79.0 (54.5 to 110.7)	0.2 (0.0 to 0.8)	1772.9 (1596.6 to 1970.5)	1192.8 (1045.7 to 1372.8)	451.5 (387.8 to 524.6)	128.3 (88.4 to 179.6)	0.3 (0.0 to 1.3)
Antigua and Barbuda	2050	1110.9 (1000.2 to 1228.7)	749.6 (655.4 to 866.1)	280.7 (241.2 to 326.4)	80.4 (55.2 to 112.7)	0.2 (0.0 to 0.8)	1802.9 (1623.2 to 1994.1)	1216.5 (1063.7 to 1405.6)	455.6 (391.4 to 529.7)	130.5 (89.7 to 182.9)	0.3 (0.0 to 1.3)
Argentina	1995	928.7 (869.7 to 991.1)	563.5 (523.2 to 607.1)	238.4 (208.8 to 273.7)	119.9 (91.7 to 151.6)	6.9 (6.9 to 6.9)	1400.9 (1311.8 to 1495.0)	850.0 (789.1 to 915.7)	359.6 (315.0 to 412.8)	180.9 (138.2 to 228.6)	10.4 (10.4 to 10.4)
Argentina	1996	998.7 (948.7 to 1055.8)	602.4 (568.5 to 638.8)	255.0 (227.9 to 286.2)	131.8 (105.8 to 160.8)	9.5 (9.5 to 9.5)	1506.4 (1431.0 to 1592.5)	908.6 (857.5 to 963.5)	384.7 (343.8 to 431.8)	198.8 (159.5 to 242.6)	14.3 (14.3 to 14.3)
Argentina	1997	1051.0 (1001.5 to 1107.7)	619.7 (587.4 to 653.2)	269.8 (243.8 to 298.3)	147.8 (122.8 to 176.7)	13.6 (13.6 to 13.6)	1585.3 (1510.6 to 1670.8)	934.8 (886.0 to 985.3)	406.9 (367.7 to 449.9)	223.0 (185.2 to 266.5)	20.6 (20.6 to 20.6)
Argentina	1998	1081.6 (1031.2 to 1136.2)	623.1 (588.4 to 657.9)	281.4 (254.9 to 309.5)	158.2 (132.3 to 186.7)	18.9 (18.9 to 18.9)	1631.5 (1555.4 to 1713.9)	939.8 (887.5 to 992.3)	424.5 (384.5 to 466.8)	238.6 (199.5 to 281.6)	28.6 (28.6 to 28.6)
Argentina	1999	1043.9 (995.4 to 1094.9)	590.2 (557.9 to 625.2)	282.4 (255.5 to 310.3)	159.1 (134.5 to 188.7)	12.2 (12.2 to 12.2)	1574.6 (1501.4 to 1651.5)	890.3 (841.5 to 943.1)	425.9 (385.4 to 468.0)	240.0 (202.9 to 284.6)	18.4 (18.4 to 18.4)
Argentina	2000	991.6 (944.9 to 1040.1)	549.1 (519.1 to 581.8)	278.4 (251.6 to 308.0)	156.0 (131.8 to 185.6)	8.1 (8.1 to 8.1)	1495.7 (1425.3 to 1568.9)	828.2 (783.0 to 877.5)	419.9 (379.5 to 464.6)	235.2 (198.8 to 280.0)	12.2 (12.2 to 12.2)
Argentina	2001	906.2 (859.8 to 954.5)	492.1 (463.3 to 521.8)	262.5 (237.1 to 292.5)	144.2 (121.1 to 172.8)	7.3 (7.3 to 7.3)	1366.8 (1296.9 to 1439.7)	742.2 (698.8 to 787.0)	396.0 (357.7 to 441.2)	217.5 (182.7 to 260.6)	11.0 (11.0 to 11.0)

Country	Year	2018 US Dollars per capita					2018 PPP per capita				
		Health spending	Government health spending	Out-of-pocket health spending	Prepaid private health spending	Development assistance for health	Health spending	Government health spending	Out-of-pocket health spending	Prepaid private health spending	Development assistance for health
Argentina	2002	764.0 (722.8 to 804.6)	409.5 (384.7 to 436.4)	230.1 (206.8 to 256.3)	122.1 (101.8 to 145.9)	2.3 (2.3 to 2.3)	1152.4 (1090.2 to 1213.6)	617.6 (580.2 to 658.3)	347.1 (312.0 to 386.6)	184.1 (153.5 to 220.1)	3.5 (3.5 to 3.5)
Argentina	2003	768.6 (728.5 to 810.7)	400.7 (376.1 to 427.4)	224.9 (201.4 to 251.4)	122.0 (101.5 to 145.7)	20.9 (20.9 to 20.9)	1159.3 (1098.9 to 1222.8)	604.5 (567.3 to 644.7)	339.2 (303.8 to 379.2)	184.1 (153.1 to 219.8)	31.6 (31.6 to 31.6)
Argentina	2004	763.1 (721.8 to 806.9)	403.7 (377.7 to 433.2)	231.2 (208.0 to 257.2)	122.2 (101.8 to 145.6)	6.0 (6.0 to 6.0)	1151.0 (1088.7 to 1217.0)	608.9 (569.8 to 653.4)	348.7 (313.8 to 387.9)	184.4 (153.5 to 219.6)	9.1 (9.1 to 9.1)
Argentina	2005	815.4 (772.1 to 863.5)	431.1 (404.6 to 460.5)	255.6 (230.1 to 282.1)	126.4 (106.0 to 150.3)	2.3 (2.3 to 2.3)	1229.8 (1164.6 to 1302.5)	650.3 (610.3 to 694.7)	385.5 (347.1 to 425.6)	190.6 (159.9 to 226.7)	3.5 (3.5 to 3.5)
Argentina	2006	880.6 (835.4 to 929.6)	475.3 (446.5 to 506.4)	268.9 (241.5 to 296.3)	134.3 (113.5 to 159.1)	2.0 (2.0 to 2.0)	1328.3 (1260.1 to 1402.2)	717.0 (673.5 to 763.8)	405.6 (364.3 to 446.9)	202.6 (171.3 to 239.9)	3.1 (3.1 to 3.1)
Argentina	2007	970.0 (923.8 to 1018.4)	542.9 (511.8 to 575.7)	273.8 (246.2 to 301.0)	145.2 (122.7 to 171.3)	8.0 (8.0 to 8.0)	1463.1 (1393.4 to 1536.1)	818.9 (772.0 to 868.3)	413.0 (371.4 to 454.1)	219.0 (185.0 to 258.4)	12.1 (12.1 to 12.1)
Argentina	2008	1030.4 (981.3 to 1080.5)	609.1 (577.0 to 644.2)	263.3 (238.1 to 288.6)	153.5 (128.7 to 180.2)	4.4 (4.4 to 4.4)	1554.1 (1480.1 to 1629.7)	918.8 (870.3 to 971.6)	397.1 (359.2 to 435.4)	231.5 (194.1 to 271.8)	6.7 (6.7 to 6.7)
Argentina	2009	1050.5 (1000.1 to 1098.8)	649.1 (617.9 to 683.5)	241.5 (217.5 to 264.0)	155.7 (131.6 to 182.4)	4.1 (4.1 to 4.1)	1584.5 (1508.5 to 1657.4)	979.1 (932.0 to 1030.9)	364.2 (328.0 to 398.2)	234.9 (198.5 to 275.2)	6.3 (6.3 to 6.3)
Argentina	2010	1129.2 (1077.6 to 1178.3)	735.5 (701.8 to 774.0)	225.5 (202.3 to 248.6)	163.8 (138.0 to 192.0)	4.5 (4.5 to 4.5)	1703.2 (1625.4 to 1777.2)	1109.4 (1058.5 to 1167.5)	340.1 (305.2 to 375.0)	247.0 (208.2 to 289.5)	6.7 (6.7 to 6.7)
Argentina	2011	1177.1 (1127.8 to 1230.1)	805.8 (769.6 to 843.2)	206.1 (184.1 to 229.7)	157.5 (132.8 to 184.1)	7.7 (7.7 to 7.7)	1775.5 (1701.1 to 1855.4)	1215.5 (1160.8 to 1271.9)	310.9 (277.6 to 346.5)	237.6 (200.3 to 277.7)	11.6 (11.6 to 11.6)
Argentina	2012	1156.7 (1110.2 to 1208.9)	831.5 (793.3 to 869.9)	180.9 (161.3 to 201.8)	138.1 (116.0 to 163.9)	6.3 (6.3 to 6.3)	1744.8 (1674.5 to 1823.4)	1254.2 (1196.6 to 1312.2)	272.8 (243.3 to 304.4)	208.3 (174.9 to 247.3)	9.5 (9.5 to 9.5)
Argentina	2013	1160.4 (1112.9 to 1209.5)	863.0 (825.6 to 901.2)	171.0 (152.0 to 191.3)	119.5 (100.8 to 142.2)	6.9 (6.9 to 6.9)	1750.2 (1678.7 to 1824.4)	1301.7 (1245.3 to 1359.3)	257.9 (229.3 to 288.6)	180.2 (152.0 to 214.5)	10.4 (10.4 to 10.4)
Argentina	2014	1119.1 (1074.3 to 1164.4)	856.5 (818.3 to 894.4)	162.5 (143.5 to 181.8)	100.0 (82.8 to 120.4)	0.1 (0.1 to 0.1)	1688.1 (1620.4 to 1756.3)	1291.9 (1234.3 to 1349.1)	245.1 (216.4 to 274.2)	150.9 (124.9 to 181.6)	0.2 (0.2 to 0.2)
Argentina	2015	1140.0 (1086.5 to 1190.9)	877.1 (833.6 to 918.7)	162.2 (141.2 to 184.2)	96.2 (76.8 to 118.8)	4.5 (4.5 to 4.5)	1719.5 (1638.9 to 1796.4)	1323.0 (1257.4 to 1385.7)	244.6 (213.0 to 277.8)	145.1 (115.9 to 179.2)	6.8 (6.8 to 6.8)
Argentina	2016	1071.3 (1007.7 to 1135.4)	815.7 (764.5 to 866.9)	158.4 (134.4 to 185.3)	90.1 (68.3 to 116.4)	7.1 (7.1 to 7.1)	1615.9 (1519.9 to 1712.6)	1230.4 (1153.2 to 1307.5)	238.9 (202.7 to 279.5)	135.9 (103.0 to 175.5)	10.7 (10.7 to 10.7)
Argentina	2017	1075.9 (1011.5 to 1140.9)	824.8 (772.3 to 878.3)	159.0 (134.4 to 186.2)	92.1 (69.7 to 119.3)	0.1 (0.1 to 0.1)	1622.9 (1525.7 to 1720.9)	1244.0 (1164.9 to 1324.8)	239.8 (202.8 to 280.8)	138.9 (105.1 to 179.9)	0.2 (0.2 to 0.2)
Argentina	2018	1092.3 (1029.2 to 1156.9)	839.5 (786.7 to 894.0)	159.0 (134.7 to 185.8)	93.7 (70.6 to 121.6)	0.1 (0.1 to 0.1)	1647.6 (1552.4 to 1745.1)	1266.3 (1186.6 to 1348.5)	239.8 (203.2 to 280.2)	141.3 (106.6 to 183.4)	0.2 (0.2 to 0.2)
Argentina	2019	1108.9 (1041.9 to 1176.3)	854.6 (800.5 to 910.2)	159.0 (134.7 to 185.0)	95.2 (71.9 to 123.7)	0.1 (0.1 to 0.1)	1672.6 (1571.6 to 1774.3)	1289.0 (1207.4 to 1372.9)	239.8 (203.2 to 279.1)	143.7 (108.4 to 186.5)	0.2 (0.2 to 0.2)
Argentina	2020	1125.7 (1061.4 to 1191.9)	869.8 (814.1 to 925.8)	158.9 (134.8 to 186.3)	96.8 (73.0 to 125.8)	0.1 (0.1 to 0.1)	1697.9 (1600.9 to 1797.8)	1312.0 (1228.0 to 1396.5)	239.7 (203.3 to 281.0)	146.0 (110.1 to 189.8)	0.2 (0.2 to 0.2)
Argentina	2021	1141.7 (1073.1 to 1215.3)	884.3 (827.8 to 941.5)	158.9 (134.7 to 187.0)	98.3 (74.1 to 127.6)	0.1 (0.1 to 0.1)	1722.0 (1618.6 to 1833.1)	1333.9 (1248.7 to 1420.2)	239.7 (203.2 to 282.0)	148.3 (111.7 to 192.5)	0.2 (0.2 to 0.2)
Argentina	2022	1158.8 (1088.6 to 1227.7)	899.8 (840.3 to 958.8)	159.0 (135.1 to 186.5)	99.9 (75.4 to 129.5)	0.1 (0.1 to 0.1)	1747.9 (1642.0 to 1851.8)	1357.2 (1267.4 to 1446.2)	239.8 (203.8 to 281.2)	150.7 (113.7 to 195.3)	0.2 (0.2 to 0.2)

Country	Year	2018 US Dollars per capita					2018 PPP per capita				
		Health spending	Government health spending	Out-of-pocket health spending	Prepaid private health spending	Development assistance for health	Health spending	Government health spending	Out-of-pocket health spending	Prepaid private health spending	Development assistance for health
Argentina	2023	1176.1 (1104.2 to 1248.0)	915.1 (853.4 to 975.8)	159.4 (135.5 to 186.2)	101.5 (76.5 to 131.3)	0.1 (0.1 to 0.1)	1774.0 (1665.6 to 1882.4)	1380.3 (1287.3 to 1471.9)	240.5 (204.4 to 280.9)	153.1 (115.5 to 198.1)	0.2 (0.2 to 0.2)
Argentina	2024	1194.5 (1124.6 to 1270.6)	931.1 (867.4 to 994.1)	160.0 (135.6 to 187.5)	103.2 (77.6 to 133.2)	0.1 (0.1 to 0.1)	1801.7 (1696.2 to 1916.5)	1404.5 (1308.4 to 1499.4)	241.4 (204.5 to 282.8)	155.6 (117.1 to 201.0)	0.2 (0.2 to 0.2)
Argentina	2025	1212.9 (1139.4 to 1289.4)	947.0 (880.9 to 1012.2)	161.0 (135.7 to 189.0)	104.8 (78.8 to 135.7)	0.1 (0.1 to 0.2)	1829.5 (1718.6 to 1944.9)	1428.4 (1328.7 to 1526.8)	242.8 (204.6 to 285.1)	158.1 (118.8 to 204.6)	0.2 (0.2 to 0.2)
Argentina	2026	1231.4 (1155.5 to 1308.4)	962.9 (893.9 to 1029.9)	161.9 (136.5 to 189.9)	106.5 (80.0 to 138.1)	0.1 (0.1 to 0.2)	1857.4 (1742.9 to 1973.5)	1452.3 (1348.3 to 1553.5)	244.2 (205.9 to 286.5)	160.6 (120.7 to 208.3)	0.2 (0.2 to 0.2)
Argentina	2027	1250.4 (1171.2 to 1332.4)	979.2 (907.1 to 1049.8)	162.9 (136.9 to 191.1)	108.2 (81.3 to 140.2)	0.1 (0.1 to 0.2)	1886.1 (1766.6 to 2009.7)	1477.0 (1368.2 to 1583.4)	245.7 (206.5 to 288.2)	163.2 (122.7 to 211.4)	0.2 (0.2 to 0.2)
Argentina	2028	1269.2 (1186.9 to 1351.8)	995.4 (922.6 to 1068.1)	163.8 (136.8 to 192.5)	109.9 (82.6 to 142.2)	0.1 (0.1 to 0.2)	1914.5 (1790.3 to 2039.0)	1501.4 (1391.6 to 1611.1)	247.1 (206.4 to 290.4)	165.7 (124.6 to 214.4)	0.2 (0.2 to 0.3)
Argentina	2029	1287.7 (1195.5 to 1376.3)	1011.3 (936.4 to 1085.7)	164.7 (136.6 to 193.5)	111.5 (83.9 to 144.2)	0.1 (0.1 to 0.2)	1942.3 (1803.2 to 2075.9)	1525.3 (1412.4 to 1637.7)	248.5 (206.0 to 291.8)	168.2 (126.5 to 217.4)	0.2 (0.2 to 0.3)
Argentina	2030	1307.4 (1217.2 to 1396.1)	1028.2 (950.8 to 1105.4)	165.8 (137.2 to 194.5)	113.3 (85.2 to 146.8)	0.1 (0.1 to 0.2)	1972.0 (1836.0 to 2105.8)	1550.8 (1434.1 to 1667.3)	250.1 (207.0 to 293.4)	170.9 (128.6 to 221.4)	0.2 (0.2 to 0.3)
Argentina	2031	1327.9 (1237.0 to 1423.1)	1045.6 (964.3 to 1128.0)	166.9 (137.6 to 195.9)	115.2 (86.6 to 149.2)	0.1 (0.1 to 0.2)	2002.9 (1865.8 to 2146.6)	1577.2 (1454.5 to 1701.4)	251.8 (207.6 to 295.5)	173.7 (130.7 to 225.0)	0.2 (0.2 to 0.3)
Argentina	2032	1349.1 (1255.6 to 1442.4)	1063.7 (978.7 to 1149.2)	168.2 (138.2 to 197.3)	117.1 (88.1 to 151.5)	0.1 (0.0 to 0.2)	2034.9 (1893.9 to 2175.6)	1604.5 (1476.2 to 1733.4)	253.7 (208.4 to 297.6)	176.6 (132.9 to 228.5)	0.2 (0.0 to 0.3)
Argentina	2033	1368.9 (1266.9 to 1468.5)	1080.7 (992.6 to 1171.3)	169.2 (138.4 to 199.6)	118.8 (89.5 to 153.6)	0.2 (0.0 to 0.2)	2064.9 (1910.9 to 2215.0)	1630.1 (1497.1 to 1766.7)	255.3 (208.8 to 301.1)	179.3 (135.1 to 231.6)	0.2 (0.0 to 0.3)
Argentina	2034	1391.1 (1294.3 to 1501.3)	1099.5 (1008.2 to 1195.2)	170.6 (138.9 to 201.3)	120.8 (91.0 to 156.2)	0.2 (0.0 to 0.2)	2098.2 (1952.2 to 2264.4)	1658.5 (1520.7 to 1802.8)	257.3 (209.5 to 303.6)	182.2 (137.2 to 235.5)	0.2 (0.0 to 0.3)
Argentina	2035	1412.6 (1305.8 to 1520.9)	1117.9 (1020.1 to 1218.8)	171.8 (139.5 to 203.2)	122.8 (92.4 to 158.5)	0.2 (0.0 to 0.2)	2130.8 (1969.6 to 2294.0)	1686.2 (1538.7 to 1838.4)	259.2 (210.4 to 306.5)	185.2 (139.4 to 239.1)	0.2 (0.0 to 0.3)
Argentina	2036	1434.9 (1331.0 to 1549.5)	1136.9 (1034.4 to 1243.4)	173.2 (140.2 to 204.7)	124.8 (94.0 to 161.0)	0.2 (0.0 to 0.2)	2164.4 (2007.6 to 2337.2)	1714.8 (1560.2 to 1875.5)	261.2 (211.5 to 308.8)	188.2 (141.7 to 242.8)	0.2 (0.0 to 0.3)
Argentina	2037	1457.2 (1335.2 to 1573.5)	1155.8 (1049.6 to 1267.6)	174.5 (141.1 to 206.4)	126.8 (95.6 to 163.6)	0.2 (0.0 to 0.2)	2198.0 (2014.0 to 2373.4)	1743.4 (1583.2 to 1912.0)	263.2 (212.8 to 311.3)	191.2 (144.3 to 246.8)	0.2 (0.0 to 0.4)
Argentina	2038	1479.8 (1363.8 to 1600.1)	1175.0 (1065.1 to 1292.5)	175.9 (141.9 to 208.1)	128.8 (97.3 to 166.3)	0.1 (0.0 to 0.2)	2232.1 (2057.1 to 2413.5)	1772.3 (1606.6 to 1949.5)	265.3 (214.0 to 313.8)	194.3 (146.7 to 250.8)	0.2 (0.0 to 0.4)
Argentina	2039	1499.9 (1377.2 to 1620.2)	1192.1 (1078.7 to 1315.1)	177.0 (142.8 to 209.3)	130.6 (98.7 to 168.9)	0.1 (0.0 to 0.3)	2262.3 (2077.3 to 2443.9)	1798.2 (1627.1 to 1983.6)	267.0 (215.5 to 315.7)	197.0 (148.9 to 254.7)	0.2 (0.0 to 0.4)
Argentina	2040	1520.5 (1392.8 to 1651.4)	1209.7 (1091.6 to 1337.2)	178.2 (143.9 to 210.8)	132.4 (100.3 to 171.4)	0.1 (0.0 to 0.3)	2293.4 (2100.9 to 2490.9)	1824.6 (1646.6 to 2017.0)	268.8 (217.1 to 317.9)	199.8 (151.2 to 258.5)	0.2 (0.0 to 0.4)
Argentina	2041	1539.4 (1411.2 to 1670.0)	1225.9 (1103.2 to 1356.2)	179.2 (144.9 to 212.7)	134.2 (101.7 to 173.1)	0.1 (0.0 to 0.3)	2322.0 (2128.6 to 2518.9)	1849.1 (1664.0 to 2045.6)	270.3 (218.5 to 320.9)	202.4 (153.4 to 261.1)	0.2 (0.0 to 0.4)
Argentina	2042	1558.8 (1423.4 to 1700.8)	1242.5 (1115.1 to 1375.8)	180.3 (146.0 to 214.7)	135.9 (103.2 to 176.1)	0.1 (0.0 to 0.3)	2351.3 (2146.9 to 2565.4)	1874.1 (1681.9 to 2075.2)	271.9 (220.3 to 323.8)	205.0 (155.6 to 265.7)	0.2 (0.0 to 0.4)
Argentina	2043	1576.4 (1428.6 to 1718.7)	1257.6 (1125.5 to 1395.2)	181.2 (147.0 to 216.6)	137.5 (104.2 to 178.7)	0.1 (0.0 to 0.3)	2377.8 (2154.9 to 2592.5)	1896.9 (1697.7 to 2104.5)	273.3 (221.8 to 326.7)	207.4 (157.2 to 269.5)	0.2 (0.0 to 0.4)

		2018 US Dollars per capita					2018 PPP per capita				
Country	Year	Health spending	Government health spending	Out-of-pocket health spending	Prepaid private health spending	Development assistance for health	Health spending	Government health spending	Out-of-pocket health spending	Prepaid private health spending	Development assistance for health
Argentina	2044	1596.8 (1454.1 to 1743.0)	1274.9 (1137.9 to 1418.5)	182.4 (148.6 to 218.0)	139.3 (105.6 to 181.6)	0.1 (0.0 to 0.3)	2408.5 (2193.3 to 2629.1)	1923.0 (1716.3 to 2139.5)	275.1 (224.1 to 328.9)	210.1 (159.3 to 273.8)	0.2 (0.0 to 0.5)
Argentina	2045	1617.1 (1467.0 to 1771.3)	1292.2 (1150.1 to 1440.3)	183.6 (150.3 to 221.0)	141.2 (106.7 to 184.1)	0.1 (0.0 to 0.3)	2439.2 (2212.7 to 2671.7)	1949.1 (1734.8 to 2172.5)	277.0 (226.7 to 333.4)	212.9 (160.9 to 277.7)	0.2 (0.0 to 0.5)
Argentina	2046	1637.9 (1483.2 to 1807.5)	1309.7 (1162.6 to 1463.7)	184.9 (151.8 to 223.8)	143.1 (108.2 to 186.8)	0.1 (0.0 to 0.3)	2470.5 (2237.2 to 2726.4)	1975.5 (1753.6 to 2207.8)	278.9 (229.0 to 337.6)	215.8 (163.2 to 281.7)	0.2 (0.0 to 0.5)
Argentina	2047	1658.5 (1502.8 to 1822.5)	1327.1 (1175.0 to 1487.3)	186.2 (153.2 to 226.0)	145.0 (109.4 to 189.6)	0.1 (0.0 to 0.4)	2501.6 (2266.8 to 2749.0)	2001.7 (1772.3 to 2243.3)	280.9 (231.1 to 340.9)	218.7 (165.1 to 286.0)	0.2 (0.0 to 0.5)
Argentina	2048	1680.2 (1509.2 to 1854.3)	1345.3 (1187.8 to 1511.9)	187.7 (155.2 to 227.9)	147.0 (111.0 to 192.8)	0.1 (0.0 to 0.4)	2534.3 (2276.5 to 2796.9)	2029.2 (1791.6 to 2280.5)	283.1 (234.1 to 343.8)	221.8 (167.4 to 290.9)	0.2 (0.0 to 0.6)
Argentina	2049	1700.9 (1526.5 to 1877.3)	1362.7 (1199.9 to 1535.8)	189.0 (156.3 to 230.0)	149.0 (112.5 to 196.0)	0.1 (0.0 to 0.4)	2565.5 (2302.5 to 2831.7)	2055.4 (1809.9 to 2316.6)	285.1 (235.7 to 346.9)	224.8 (169.7 to 295.6)	0.2 (0.0 to 0.6)
Argentina	2050	1722.1 (1544.4 to 1914.7)	1380.5 (1211.9 to 1560.1)	190.4 (158.0 to 232.8)	151.0 (114.1 to 199.2)	0.1 (0.0 to 0.4)	2597.5 (2329.6 to 2888.0)	2082.3 (1828.0 to 2353.2)	287.2 (238.3 to 351.1)	227.8 (172.1 to 300.5)	0.2 (0.0 to 0.6)
Armenia	1995	40.6 (33.7 to 47.8)	17.0 (13.0 to 21.5)	19.7 (14.1 to 26.2)	0.8 (0.4 to 1.6)	3.0 (3.0 to 3.0)	103.7 (86.1 to 122.3)	43.5 (33.2 to 55.1)	50.4 (36.2 to 67.0)	2.1 (0.9 to 4.0)	7.8 (7.8 to 7.8)
Armenia	1996	41.8 (34.9 to 49.4)	16.4 (12.6 to 20.6)	21.2 (15.6 to 27.9)	1.0 (0.4 to 1.8)	3.3 (3.3 to 3.3)	106.9 (89.4 to 126.3)	41.8 (32.3 to 52.7)	54.1 (39.8 to 71.4)	2.4 (1.1 to 4.6)	8.5 (8.5 to 8.5)
Armenia	1997	44.5 (37.5 to 52.3)	15.5 (12.0 to 19.5)	23.8 (18.1 to 30.6)	1.0 (0.5 to 2.0)	4.1 (4.1 to 4.1)	113.7 (96.0 to 133.8)	39.7 (30.7 to 49.9)	60.9 (46.4 to 78.4)	2.6 (1.2 to 5.0)	10.5 (10.5 to 10.5)
Armenia	1998	46.9 (39.6 to 55.5)	16.1 (12.3 to 20.3)	27.7 (21.7 to 35.3)	1.1 (0.5 to 2.2)	2.0 (2.0 to 2.0)	119.9 (101.3 to 142.0)	41.2 (31.6 to 52.0)	70.8 (55.6 to 90.3)	2.9 (1.3 to 5.7)	5.1 (5.1 to 5.1)
Armenia	1999	53.2 (45.3 to 62.2)	15.8 (12.2 to 20.0)	33.4 (26.0 to 41.6)	1.2 (0.5 to 2.2)	2.8 (2.8 to 2.8)	136.1 (115.9 to 159.1)	40.5 (31.3 to 51.1)	85.3 (66.6 to 106.5)	3.0 (1.4 to 5.6)	7.3 (7.3 to 7.3)
Armenia	2000	67.0 (57.5 to 77.9)	15.4 (12.0 to 19.2)	45.4 (36.2 to 55.5)	1.2 (0.5 to 2.3)	5.1 (5.1 to 5.1)	171.5 (147.0 to 199.1)	39.4 (30.7 to 49.1)	116.0 (92.7 to 142.0)	3.0 (1.4 to 5.8)	13.1 (13.1 to 13.1)
Armenia	2001	76.9 (65.9 to 89.1)	17.4 (13.6 to 21.8)	55.1 (44.9 to 66.5)	1.3 (0.6 to 2.5)	3.1 (3.1 to 3.1)	196.6 (168.5 to 228.0)	44.6 (34.9 to 55.9)	140.9 (114.8 to 170.1)	3.3 (1.5 to 6.3)	7.8 (7.8 to 7.8)
Armenia	2002	90.7 (78.1 to 103.6)	19.7 (15.4 to 24.6)	64.8 (53.0 to 77.3)	1.3 (0.6 to 2.6)	4.9 (4.9 to 4.9)	232.1 (199.9 to 265.0)	50.3 (39.4 to 63.0)	165.8 (135.5 to 197.8)	3.4 (1.6 to 6.5)	12.6 (12.6 to 12.6)
Armenia	2003	105.3 (91.2 to 120.8)	22.9 (18.2 to 28.5)	79.1 (65.7 to 92.9)	1.2 (0.6 to 2.5)	2.0 (2.0 to 2.0)	269.4 (233.1 to 309.0)	58.6 (46.5 to 72.9)	202.4 (168.0 to 237.7)	3.2 (1.6 to 6.3)	5.2 (5.2 to 5.2)
Armenia	2004	128.8 (111.9 to 147.2)	26.7 (21.3 to 33.2)	98.1 (82.4 to 114.0)	0.9 (0.4 to 1.8)	3.1 (3.1 to 3.1)	329.6 (286.2 to 376.5)	68.3 (54.5 to 84.9)	251.0 (210.8 to 291.5)	2.3 (1.1 to 4.5)	8.0 (8.0 to 8.0)
Armenia	2005	140.8 (122.7 to 160.3)	31.9 (25.5 to 39.9)	102.3 (86.4 to 119.0)	0.5 (0.2 to 0.9)	6.2 (6.2 to 6.2)	360.2 (313.9 to 410.1)	81.7 (65.3 to 102.0)	261.6 (221.0 to 304.3)	1.2 (0.6 to 2.2)	15.8 (15.8 to 15.8)
Armenia	2006	146.2 (127.7 to 165.6)	38.0 (30.6 to 47.2)	98.5 (83.0 to 114.9)	0.3 (0.2 to 0.6)	9.4 (9.4 to 9.4)	374.0 (326.6 to 423.5)	97.2 (78.3 to 120.8)	251.9 (212.4 to 293.9)	0.8 (0.4 to 1.6)	24.0 (24.0 to 24.0)
Armenia	2007	153.2 (133.9 to 174.0)	44.6 (35.9 to 55.3)	97.9 (82.9 to 115.8)	0.3 (0.2 to 0.6)	10.4 (10.4 to 10.4)	392.0 (342.6 to 445.1)	114.0 (91.9 to 141.3)	250.5 (212.1 to 296.1)	0.8 (0.4 to 1.6)	26.7 (26.7 to 26.7)
Armenia	2008	149.9 (131.2 to 170.7)	48.3 (39.1 to 60.3)	92.9 (78.1 to 109.5)	0.4 (0.2 to 0.8)	8.2 (8.2 to 8.2)	383.4 (335.7 to 436.5)	123.6 (99.9 to 154.3)	237.7 (199.8 to 280.0)	1.1 (0.5 to 2.0)	21.1 (21.1 to 21.1)

Country	Year	2018 US Dollars per capita					2018 PPP per capita				
		Health spending	Government health spending	Out-of-pocket health spending	Prepaid private health spending	Development assistance for health	Health spending	Government health spending	Out-of-pocket health spending	Prepaid private health spending	Development assistance for health
Armenia	2009	146.2 (128.8 to 165.4)	45.3 (36.3 to 56.4)	85.5 (71.9 to 100.6)	0.5 (0.3 to 1.0)	14.9 (14.9 to 14.9)	373.9 (329.4 to 423.0)	115.8 (92.8 to 144.4)	218.7 (184.0 to 257.4)	1.4 (0.7 to 2.6)	38.0 (38.0 to 38.0)
Armenia	2010	150.0 (131.7 to 168.9)	46.6 (37.1 to 57.2)	92.4 (78.5 to 107.8)	0.9 (0.4 to 1.8)	10.0 (10.0 to 10.0)	383.7 (336.9 to 432.0)	119.3 (94.8 to 146.2)	236.4 (200.7 to 275.7)	2.4 (1.1 to 4.6)	25.7 (25.7 to 25.7)
Armenia	2011	159.5 (141.0 to 178.9)	48.4 (38.4 to 59.3)	99.1 (85.0 to 114.8)	1.5 (0.7 to 2.8)	10.5 (10.5 to 10.5)	407.9 (360.7 to 457.7)	123.9 (98.3 to 151.8)	253.5 (217.4 to 293.8)	3.7 (1.7 to 7.2)	26.8 (26.8 to 26.8)
Armenia	2012	208.2 (186.7 to 231.3)	49.7 (39.5 to 61.0)	144.8 (126.1 to 165.4)	2.3 (1.1 to 4.4)	11.4 (11.4 to 11.4)	532.5 (477.5 to 591.6)	127.1 (101.1 to 155.9)	370.4 (322.5 to 423.1)	5.9 (2.7 to 11.2)	29.1 (29.1 to 29.1)
Armenia	2013	260.4 (235.6 to 287.6)	50.6 (40.1 to 62.7)	197.7 (175.7 to 222.5)	3.1 (1.4 to 6.0)	9.0 (9.0 to 9.0)	666.0 (602.6 to 735.8)	129.3 (102.6 to 160.3)	505.7 (449.4 to 569.2)	8.0 (3.6 to 15.2)	23.0 (23.0 to 23.0)
Armenia	2014	312.7 (284.9 to 345.6)	52.7 (41.6 to 65.8)	250.8 (225.1 to 280.9)	3.7 (1.7 to 7.2)	5.5 (5.5 to 5.5)	799.8 (728.6 to 884.1)	134.8 (106.4 to 168.4)	641.5 (575.8 to 718.6)	9.5 (4.3 to 18.4)	14.0 (14.0 to 14.0)
Armenia	2015	347.8 (312.4 to 384.9)	55.8 (44.1 to 69.8)	283.9 (251.4 to 319.1)	4.2 (1.9 to 8.0)	3.9 (3.9 to 3.9)	889.7 (799.0 to 984.6)	142.7 (112.7 to 178.6)	726.1 (643.1 to 816.2)	10.8 (4.9 to 20.4)	10.1 (10.1 to 10.1)
Armenia	2016	364.9 (323.2 to 410.8)	57.5 (45.2 to 72.5)	296.1 (256.7 to 338.5)	4.3 (2.0 to 8.1)	7.0 (7.0 to 7.0)	933.3 (826.8 to 1050.9)	147.2 (115.7 to 185.5)	757.3 (656.6 to 865.9)	11.1 (5.2 to 20.8)	17.8 (17.8 to 17.8)
Armenia	2017	386.8 (342.2 to 434.9)	60.0 (46.9 to 75.7)	317.4 (274.6 to 363.8)	4.6 (2.2 to 8.7)	4.8 (4.8 to 4.8)	989.4 (875.3 to 1112.5)	153.4 (120.1 to 193.7)	811.9 (702.4 to 930.5)	11.8 (5.5 to 22.1)	12.2 (12.2 to 12.2)
Armenia	2018	398.7 (352.2 to 448.9)	61.9 (48.3 to 78.1)	327.5 (283.4 to 375.4)	4.8 (2.2 to 8.9)	4.5 (4.5 to 4.5)	1019.9 (900.9 to 1148.2)	158.4 (123.6 to 199.7)	837.7 (724.9 to 960.2)	12.2 (5.7 to 22.8)	11.6 (11.6 to 11.6)
Armenia	2019	405.8 (359.2 to 457.2)	63.7 (49.5 to 80.3)	332.6 (289.4 to 381.1)	4.9 (2.3 to 9.1)	4.6 (4.3 to 4.9)	1037.9 (918.7 to 1169.5)	163.0 (126.5 to 205.3)	850.7 (740.3 to 974.8)	12.5 (5.8 to 23.4)	11.8 (11.0 to 12.5)
Armenia	2020	412.7 (366.8 to 465.5)	65.6 (50.5 to 83.1)	337.4 (293.0 to 386.3)	5.0 (2.3 to 9.4)	4.7 (4.3 to 5.1)	1055.5 (938.3 to 1190.7)	167.8 (129.3 to 212.6)	862.9 (749.4 to 988.0)	12.8 (6.0 to 24.0)	12.0 (11.0 to 13.0)
Armenia	2021	419.4 (371.2 to 471.8)	67.8 (52.3 to 85.4)	341.7 (296.3 to 391.4)	5.1 (2.4 to 9.6)	4.8 (4.3 to 5.3)	1072.7 (949.5 to 1206.9)	173.3 (133.8 to 218.3)	874.0 (757.9 to 1001.1)	13.1 (6.1 to 24.6)	12.4 (11.1 to 13.7)
Armenia	2022	439.4 (387.7 to 495.4)	69.9 (53.7 to 88.5)	359.3 (310.7 to 411.7)	5.2 (2.4 to 9.8)	5.0 (4.4 to 5.6)	1123.9 (991.7 to 1267.3)	178.7 (137.5 to 226.3)	919.0 (794.6 to 1053.1)	13.4 (6.2 to 25.2)	12.8 (11.3 to 14.2)
Armenia	2023	459.7 (405.6 to 518.3)	72.0 (54.9 to 91.4)	377.2 (324.8 to 432.0)	5.4 (2.5 to 10.1)	5.2 (4.5 to 5.9)	1175.9 (1037.4 to 1325.8)	184.2 (140.4 to 233.8)	964.8 (830.8 to 1105.1)	13.7 (6.4 to 25.8)	13.2 (11.5 to 15.0)
Armenia	2024	480.8 (422.6 to 543.4)	74.2 (56.7 to 93.8)	395.8 (341.7 to 453.5)	5.5 (2.5 to 10.3)	5.3 (4.6 to 6.2)	1229.8 (1081.0 to 1389.9)	189.7 (144.9 to 240.0)	1012.5 (874.1 to 1160.0)	14.0 (6.5 to 26.4)	13.7 (11.8 to 15.7)
Armenia	2025	503.4 (444.0 to 569.0)	76.4 (58.2 to 97.1)	415.9 (359.3 to 477.3)	5.6 (2.6 to 10.6)	5.5 (4.7 to 6.5)	1287.7 (1135.7 to 1455.5)	195.5 (149.0 to 248.3)	1063.8 (919.0 to 1220.8)	14.3 (6.6 to 27.1)	14.1 (12.0 to 16.5)
Armenia	2026	527.1 (464.3 to 596.2)	78.8 (60.1 to 100.4)	436.9 (377.9 to 501.1)	5.7 (2.7 to 10.9)	5.7 (4.8 to 6.7)	1348.2 (1187.5 to 1524.9)	201.5 (153.8 to 256.8)	1117.5 (966.7 to 1281.7)	14.7 (6.8 to 27.8)	14.6 (12.3 to 17.1)
Armenia	2027	552.4 (482.9 to 628.1)	81.3 (61.9 to 104.0)	459.4 (394.3 to 530.0)	5.9 (2.7 to 11.1)	5.9 (4.9 to 7.0)	1413.0 (1235.3 to 1606.6)	207.9 (158.3 to 266.0)	1175.1 (1008.5 to 1355.6)	15.0 (7.0 to 28.5)	15.1 (12.6 to 17.9)
Armenia	2028	578.3 (505.9 to 657.5)	83.8 (64.1 to 108.0)	482.4 (410.6 to 558.4)	6.0 (2.8 to 11.4)	6.1 (5.1 to 7.4)	1479.3 (1294.1 to 1681.7)	214.3 (163.9 to 276.3)	1233.9 (1050.1 to 1428.3)	15.4 (7.2 to 29.2)	15.6 (13.0 to 18.8)
Armenia	2029	605.1 (523.9 to 690.3)	86.5 (65.7 to 111.9)	506.1 (426.9 to 587.0)	6.2 (2.9 to 11.7)	6.3 (5.2 to 7.7)	1547.6 (1340.2 to 1765.6)	221.2 (168.0 to 286.3)	1294.4 (1092.1 to 1501.5)	15.9 (7.3 to 30.0)	16.1 (13.3 to 19.7)

		2018 US Dollars per capita					2018 PPP per capita				
Country	Year	Health spending	Government health spending	Out-of-pocket health spending	Prepaid private health spending	Development assistance for health	Health spending	Government health spending	Out-of-pocket health spending	Prepaid private health spending	Development assistance for health
Armenia	2030	631.7 (543.1 to 725.6)	89.0 (67.5 to 116.3)	529.7 (442.8 to 620.4)	6.4 (3.0 to 12.1)	6.5 (5.3 to 8.0)	1615.7 (1389.1 to 1856.0)	227.8 (172.6 to 297.5)	1355.0 (1132.6 to 1586.8)	16.3 (7.5 to 30.9)	16.6 (13.6 to 20.4)
Armenia	2031	659.3 (563.8 to 759.1)	91.8 (69.6 to 119.3)	554.1 (460.2 to 654.8)	6.6 (3.0 to 12.4)	6.7 (5.4 to 8.4)	1686.3 (1442.0 to 1941.6)	234.9 (178.0 to 305.2)	1417.4 (1177.2 to 1674.8)	16.8 (7.8 to 31.8)	17.2 (13.9 to 21.5)
Armenia	2032	687.8 (585.4 to 795.0)	94.7 (72.0 to 124.3)	579.4 (474.9 to 688.3)	6.8 (3.2 to 12.8)	6.9 (5.6 to 8.7)	1759.4 (1497.3 to 2033.4)	242.3 (184.2 to 317.8)	1482.0 (1214.6 to 1760.6)	17.3 (8.1 to 32.9)	17.7 (14.2 to 22.3)
Armenia	2033	716.3 (603.3 to 833.3)	97.6 (73.3 to 127.3)	604.5 (493.7 to 722.9)	7.0 (3.3 to 13.3)	7.2 (5.7 to 9.2)	1832.1 (1543.2 to 2131.5)	249.6 (187.4 to 325.7)	1546.3 (1262.9 to 1849.2)	17.9 (8.3 to 33.9)	18.3 (14.6 to 23.5)
Armenia	2034	745.1 (620.1 to 873.6)	100.5 (74.8 to 131.6)	629.9 (509.3 to 756.5)	7.2 (3.4 to 13.7)	7.4 (5.8 to 9.6)	1905.9 (1586.0 to 2234.5)	257.2 (191.4 to 336.6)	1611.3 (1302.7 to 1935.1)	18.5 (8.6 to 35.1)	19.0 (14.9 to 24.5)
Armenia	2035	774.1 (642.1 to 912.9)	103.6 (76.8 to 135.8)	655.4 (524.6 to 790.8)	7.5 (3.5 to 14.2)	7.7 (6.0 to 10.0)	1980.1 (1642.3 to 2335.0)	265.0 (196.3 to 347.4)	1676.4 (1341.9 to 2022.8)	19.1 (8.9 to 36.3)	19.6 (15.2 to 25.6)
Armenia	2036	802.8 (668.5 to 956.2)	106.8 (79.6 to 140.5)	680.5 (540.8 to 829.7)	7.7 (3.6 to 14.7)	7.9 (6.1 to 10.6)	2053.5 (1709.9 to 2445.7)	273.1 (203.6 to 359.4)	1740.5 (1383.2 to 2122.3)	19.7 (9.1 to 37.5)	20.2 (15.6 to 27.2)
Armenia	2037	830.8 (678.7 to 992.9)	109.8 (81.7 to 144.6)	704.9 (559.1 to 864.6)	7.9 (3.7 to 15.1)	8.2 (6.3 to 11.0)	2125.0 (1736.1 to 2539.7)	280.8 (209.1 to 369.8)	1802.9 (1430.0 to 2211.5)	20.3 (9.4 to 38.7)	20.9 (16.0 to 28.0)
Armenia	2038	858.2 (698.6 to 1029.8)	113.0 (83.8 to 150.0)	728.7 (574.8 to 898.9)	8.2 (3.8 to 15.6)	8.4 (6.4 to 11.7)	2195.2 (1787.0 to 2634.0)	288.9 (214.4 to 383.6)	1863.8 (1470.2 to 2299.3)	20.9 (9.7 to 39.9)	21.6 (16.4 to 30.1)
Armenia	2039	884.6 (719.0 to 1071.3)	116.0 (86.2 to 153.5)	751.4 (589.0 to 934.9)	8.4 (3.9 to 16.0)	8.7 (6.7 to 12.1)	2262.7 (1839.0 to 2740.2)	296.8 (220.4 to 392.7)	1922.1 (1506.5 to 2391.2)	21.5 (10.0 to 41.0)	22.3 (17.0 to 30.9)
Armenia	2040	910.8 (742.9 to 1101.2)	119.3 (88.2 to 157.7)	773.8 (600.8 to 965.9)	8.7 (4.0 to 16.5)	9.0 (6.8 to 12.5)	2329.7 (1900.3 to 2816.6)	305.1 (225.5 to 403.4)	1979.3 (1536.8 to 2470.5)	22.2 (10.3 to 42.2)	23.1 (17.5 to 32.0)
Armenia	2041	936.4 (754.4 to 1145.1)	122.7 (91.5 to 162.8)	795.4 (611.2 to 996.2)	8.9 (4.1 to 17.0)	9.3 (7.0 to 13.0)	2395.1 (1929.6 to 2928.9)	313.9 (233.9 to 416.4)	2034.5 (1563.4 to 2548.2)	22.9 (10.6 to 43.5)	23.9 (17.9 to 33.2)
Armenia	2042	960.6 (770.6 to 1174.6)	126.1 (93.6 to 168.1)	815.6 (620.6 to 1027.6)	9.2 (4.2 to 17.5)	9.7 (7.2 to 13.8)	2457.0 (1971.2 to 3004.4)	322.6 (239.4 to 430.0)	2086.1 (1587.4 to 2628.5)	23.5 (10.9 to 44.9)	24.8 (18.5 to 35.3)
Armenia	2043	983.6 (775.2 to 1203.5)	129.6 (95.4 to 172.7)	834.5 (634.1 to 1056.0)	9.5 (4.4 to 18.1)	10.0 (7.4 to 14.5)	2515.8 (1983.0 to 3078.3)	331.6 (243.9 to 441.7)	2134.4 (1621.9 to 2701.1)	24.2 (11.2 to 46.2)	25.7 (18.9 to 37.0)
Armenia	2044	1005.1 (797.7 to 1250.0)	133.2 (98.0 to 176.9)	851.8 (643.9 to 1083.6)	9.7 (4.5 to 18.6)	10.4 (7.6 to 15.1)	2571.0 (2040.4 to 3197.3)	340.6 (250.7 to 452.5)	2178.9 (1647.1 to 2771.6)	24.8 (11.5 to 47.6)	26.6 (19.6 to 38.5)
Armenia	2045	1025.4 (811.8 to 1271.2)	136.8 (100.7 to 182.8)	867.8 (654.1 to 1108.6)	10.0 (4.6 to 19.0)	10.8 (7.8 to 16.4)	2622.9 (2076.5 to 3251.4)	350.0 (257.5 to 467.7)	2219.7 (1673.0 to 2835.6)	25.5 (11.8 to 48.7)	27.7 (20.0 to 41.8)
Armenia	2046	1044.0 (819.5 to 1298.6)	140.6 (103.2 to 189.6)	881.9 (663.5 to 1130.4)	10.2 (4.7 to 19.5)	11.3 (8.1 to 17.3)	2670.4 (2096.1 to 3321.5)	359.8 (264.0 to 485.1)	2255.8 (1697.2 to 2891.5)	26.1 (12.0 to 49.8)	28.8 (20.8 to 44.4)
Armenia	2047	1060.2 (841.1 to 1316.3)	144.3 (105.1 to 194.4)	893.7 (671.5 to 1148.6)	10.5 (4.8 to 20.0)	11.7 (8.4 to 18.5)	2711.9 (2151.4 to 3367.0)	369.2 (268.9 to 497.2)	2285.9 (1717.6 to 2937.9)	26.8 (12.3 to 51.0)	30.0 (21.5 to 47.2)
Armenia	2048	1076.9 (847.6 to 1344.5)	148.3 (108.1 to 200.7)	905.6 (680.8 to 1168.1)	10.7 (4.9 to 20.5)	12.2 (8.7 to 19.6)	2754.4 (2167.9 to 3438.9)	379.3 (276.5 to 513.4)	2316.5 (1741.4 to 2987.8)	27.4 (12.6 to 52.3)	31.3 (22.3 to 50.2)
Armenia	2049	1091.6 (848.8 to 1365.7)	151.8 (110.9 to 205.1)	916.2 (685.8 to 1186.3)	10.9 (5.0 to 20.9)	12.7 (9.1 to 20.6)	2792.1 (2171.2 to 3493.3)	388.2 (283.6 to 524.5)	2343.4 (1754.2 to 3034.4)	27.9 (12.8 to 53.5)	32.6 (23.2 to 52.6)
Armenia	2050	1106.8 (861.6 to 1392.3)	155.5 (114.1 to 210.2)	926.8 (691.5 to 1204.8)	11.1 (5.1 to 21.4)	13.3 (9.3 to 21.7)	2830.9 (2203.9 to 3561.3)	397.8 (291.9 to 537.7)	2370.6 (1768.8 to 3081.6)	28.5 (13.1 to 54.7)	34.1 (23.8 to 55.4)

		2018 US Dollars per capita					2018 PPP per capita				
Country	Year	Health spending	Government health spending	Out-of-pocket health spending	Prepaid private health spending	Development assistance for health	Health spending	Government health spending	Out-of-pocket health spending	Prepaid private health spending	Development assistance for health
Australia	1995	2827.1 (2748.9 to 2907.1)	1872.1 (1812.1 to 1931.2)	475.7 (440.2 to 510.8)	479.3 (435.5 to 527.7)	0.0 (0.0 to 0.0)	2583.2 (2511.7 to 2656.3)	1710.6 (1655.7 to 1764.6)	434.6 (402.2 to 466.7)	437.9 (397.9 to 482.1)	0.0 (0.0 to 0.0)
Australia	1996	2950.3 (2887.6 to 3012.3)	1963.5 (1917.3 to 2012.0)	517.2 (490.1 to 547.0)	469.7 (433.8 to 507.2)	0.0 (0.0 to 0.0)	2695.8 (2638.5 to 2752.4)	1794.1 (1751.9 to 1838.4)	472.5 (447.8 to 499.8)	429.2 (396.4 to 463.4)	0.0 (0.0 to 0.0)
Australia	1997	3079.1 (3019.2 to 3144.6)	2076.8 (2031.9 to 2124.2)	566.4 (540.1 to 596.4)	435.9 (403.8 to 470.6)	0.0 (0.0 to 0.0)	2813.4 (2758.7 to 2873.3)	1897.6 (1856.6 to 1940.9)	517.5 (493.5 to 544.9)	398.3 (368.9 to 430.0)	0.0 (0.0 to 0.0)
Australia	1998	3237.6 (3173.5 to 3302.6)	2199.5 (2151.8 to 2250.9)	637.4 (608.2 to 665.7)	400.7 (369.4 to 432.3)	0.0 (0.0 to 0.0)	2958.2 (2899.7 to 3017.7)	2009.8 (1966.1 to 2056.7)	582.4 (555.8 to 608.2)	366.1 (337.5 to 395.0)	0.0 (0.0 to 0.0)
Australia	1999	3402.4 (3339.3 to 3471.1)	2334.2 (2284.8 to 2387.0)	685.5 (657.0 to 712.4)	382.7 (351.3 to 413.6)	0.0 (0.0 to 0.0)	3108.8 (3051.2 to 3171.6)	2132.8 (2087.6 to 2181.0)	626.3 (600.3 to 651.0)	349.7 (321.0 to 377.9)	0.0 (0.0 to 0.0)
Australia	2000	3529.5 (3466.8 to 3594.1)	2414.6 (2366.8 to 2461.5)	727.7 (699.1 to 754.6)	387.2 (360.9 to 414.2)	0.0 (0.0 to 0.0)	3225.0 (3167.7 to 3284.0)	2206.3 (2162.6 to 2249.1)	664.9 (638.8 to 689.5)	353.8 (329.7 to 378.4)	0.0 (0.0 to 0.0)
Australia	2001	3660.2 (3596.9 to 3720.4)	2494.8 (2447.3 to 2539.8)	748.1 (723.5 to 775.1)	417.3 (390.6 to 446.3)	0.0 (0.0 to 0.0)	3344.4 (3286.5 to 3399.4)	2279.5 (2236.1 to 2320.7)	683.6 (661.0 to 708.2)	381.3 (356.9 to 407.8)	0.0 (0.0 to 0.0)
Australia	2002	3816.6 (3754.7 to 3875.3)	2604.4 (2556.6 to 2653.7)	759.9 (734.6 to 786.9)	452.3 (424.8 to 480.8)	0.0 (0.0 to 0.0)	3487.3 (3430.8 to 3540.9)	2379.7 (2336.0 to 2424.8)	694.3 (671.2 to 719.0)	413.3 (388.1 to 439.3)	0.0 (0.0 to 0.0)
Australia	2003	3948.1 (3889.8 to 4011.2)	2691.8 (2644.7 to 2740.0)	767.0 (739.1 to 794.2)	489.3 (459.4 to 518.5)	0.0 (0.0 to 0.0)	3607.5 (3554.2 to 3665.1)	2459.6 (2416.5 to 2503.6)	700.9 (675.3 to 725.7)	447.1 (419.8 to 473.8)	0.0 (0.0 to 0.0)
Australia	2004	4077.0 (4016.3 to 4141.0)	2782.6 (2733.6 to 2829.6)	787.4 (759.6 to 816.5)	507.0 (478.3 to 537.9)	0.0 (0.0 to 0.0)	3725.2 (3669.7 to 3783.7)	2542.5 (2497.7 to 2585.5)	719.5 (694.1 to 746.1)	463.2 (437.0 to 491.5)	0.0 (0.0 to 0.0)
Australia	2005	4132.8 (4071.2 to 4196.1)	2826.7 (2780.9 to 2874.4)	804.1 (777.3 to 832.0)	502.0 (475.2 to 533.1)	0.0 (0.0 to 0.0)	3776.3 (3720.0 to 3834.1)	2582.8 (2540.9 to 2626.4)	734.7 (710.2 to 760.2)	458.7 (434.2 to 487.1)	0.0 (0.0 to 0.0)
Australia	2006	4211.0 (4151.1 to 4270.8)	2888.2 (2839.2 to 2936.8)	820.3 (793.6 to 849.0)	502.6 (476.1 to 532.5)	0.0 (0.0 to 0.0)	3847.7 (3793.0 to 3902.4)	2639.0 (2594.3 to 2683.4)	749.5 (725.1 to 775.8)	459.2 (435.0 to 486.5)	0.0 (0.0 to 0.0)
Australia	2007	4359.5 (4296.7 to 4419.3)	3008.4 (2957.6 to 3057.0)	836.5 (808.9 to 863.5)	514.7 (485.5 to 544.5)	0.0 (0.0 to 0.0)	3983.4 (3926.0 to 4038.0)	2748.8 (2702.4 to 2793.2)	764.3 (739.1 to 789.0)	470.3 (443.6 to 497.5)	0.0 (0.0 to 0.0)
Australia	2008	4496.0 (4435.6 to 4558.8)	3108.5 (3059.2 to 3156.7)	858.0 (830.2 to 885.2)	529.6 (501.3 to 558.1)	0.0 (0.0 to 0.0)	4108.1 (4052.9 to 4165.5)	2840.3 (2795.2 to 2884.3)	783.9 (758.6 to 808.8)	483.9 (458.1 to 509.9)	0.0 (0.0 to 0.0)
Australia	2009	4602.8 (4540.7 to 4670.2)	3180.4 (3127.8 to 3233.3)	883.9 (857.1 to 912.0)	538.5 (509.3 to 569.6)	0.0 (0.0 to 0.0)	4205.6 (4148.9 to 4267.3)	2906.0 (2858.0 to 2954.4)	807.6 (783.1 to 833.3)	492.0 (465.4 to 520.4)	0.0 (0.0 to 0.0)
Australia	2010	4695.3 (4634.8 to 4761.0)	3236.6 (3187.1 to 3289.5)	912.3 (883.9 to 942.1)	546.4 (514.4 to 579.4)	0.0 (0.0 to 0.0)	4290.2 (4234.9 to 4350.2)	2957.3 (2912.1 to 3005.7)	833.6 (807.7 to 860.8)	499.2 (470.0 to 529.4)	0.0 (0.0 to 0.0)
Australia	2011	4806.2 (4739.6 to 4877.5)	3297.3 (3248.3 to 3346.8)	939.1 (911.2 to 969.4)	569.8 (540.6 to 602.5)	0.0 (0.0 to 0.0)	4391.5 (4330.6 to 4456.7)	3012.8 (2968.0 to 3058.1)	858.1 (832.6 to 885.8)	520.7 (494.0 to 550.5)	0.0 (0.0 to 0.0)
Australia	2012	4920.2 (4854.0 to 4994.1)	3339.3 (3288.5 to 3394.3)	972.7 (942.0 to 1002.5)	608.2 (577.7 to 637.4)	0.0 (0.0 to 0.0)	4495.7 (4435.2 to 4563.2)	3051.2 (3004.8 to 3101.4)	888.8 (860.7 to 916.0)	555.7 (527.9 to 582.4)	0.0 (0.0 to 0.0)
Australia	2013	5050.4 (4983.9 to 5120.9)	3409.7 (3359.4 to 3461.3)	996.3 (965.4 to 1026.4)	644.3 (612.5 to 677.2)	0.0 (0.0 to 0.0)	4614.6 (4553.9 to 4679.1)	3115.5 (3069.5 to 3162.7)	910.4 (882.1 to 937.8)	588.7 (559.7 to 618.8)	0.0 (0.0 to 0.0)
Australia	2014	5235.5 (5163.3 to 5305.9)	3536.1 (3484.1 to 3591.1)	1019.7 (986.2 to 1048.9)	679.6 (647.2 to 715.2)	0.0 (0.0 to 0.0)	4783.7 (4717.8 to 4848.1)	3231.0 (3183.5 to 3281.3)	931.7 (901.1 to 958.4)	621.0 (591.4 to 653.5)	0.0 (0.0 to 0.0)
Australia	2015	5440.8 (5366.5 to 5514.4)	3698.2 (3644.4 to 3751.9)	1038.8 (1005.1 to 1069.8)	703.8 (669.6 to 737.7)	0.0 (0.0 to 0.0)	4971.3 (4903.5 to 5038.6)	3379.2 (3330.0 to 3428.2)	949.1 (918.4 to 977.5)	643.0 (611.8 to 674.0)	0.0 (0.0 to 0.0)

		2018 US Dollars per capita					2018 PPP per capita				
Country	Year	Health spending	Government health spending	Out-of-pocket health spending	Prepaid private health spending	Development assistance for health	Health spending	Government health spending	Out-of-pocket health spending	Prepaid private health spending	Development assistance for health
Australia	2016	5562.8 (5476.0 to 5649.9)	3801.9 (3730.5 to 3873.8)	1050.8 (1005.0 to 1096.4)	710.1 (665.3 to 755.5)	0.0 (0.0 to 0.0)	5082.8 (5003.5 to 5162.4)	3473.8 (3408.6 to 3539.6)	960.1 (918.3 to 1001.8)	648.9 (607.9 to 690.3)	0.0 (0.0 to 0.0)
Australia	2017	5596.2 (5505.5 to 5686.9)	3812.6 (3740.8 to 3887.2)	1063.9 (1012.5 to 1111.1)	719.8 (673.5 to 766.7)	0.0 (0.0 to 0.0)	5113.3 (5030.4 to 5196.2)	3483.6 (3418.0 to 3551.8)	972.1 (925.1 to 1015.2)	657.7 (615.4 to 700.5)	0.0 (0.0 to 0.0)
Australia	2018	5662.0 (5564.7 to 5762.1)	3855.4 (3782.2 to 3930.3)	1078.4 (1026.8 to 1129.6)	728.3 (682.6 to 774.6)	0.0 (0.0 to 0.0)	5173.5 (5084.5 to 5265.0)	3522.7 (3455.9 to 3591.2)	985.3 (938.2 to 1032.2)	665.4 (623.7 to 707.8)	0.0 (0.0 to 0.0)
Australia	2019	5728.0 (5624.4 to 5833.6)	3898.4 (3823.3 to 3982.3)	1093.1 (1037.7 to 1145.7)	736.5 (691.8 to 783.6)	0.0 (0.0 to 0.0)	5233.8 (5139.1 to 5330.3)	3562.1 (3493.4 to 3638.7)	998.8 (948.2 to 1046.8)	672.9 (632.1 to 716.0)	0.0 (0.0 to 0.0)
Australia	2020	5798.8 (5683.5 to 5908.2)	3945.2 (3862.5 to 4034.8)	1108.4 (1047.3 to 1165.6)	745.2 (700.6 to 793.6)	0.0 (0.0 to 0.0)	5298.5 (5193.1 to 5398.4)	3604.8 (3529.2 to 3686.6)	1012.8 (956.9 to 1065.0)	680.9 (640.2 to 725.1)	0.0 (0.0 to 0.0)
Australia	2021	5860.2 (5745.4 to 5973.9)	3983.1 (3895.2 to 4071.0)	1123.8 (1060.6 to 1184.2)	753.2 (706.5 to 802.3)	0.0 (0.0 to 0.0)	5354.5 (5249.7 to 5458.5)	3639.5 (3559.1 to 3719.8)	1026.8 (969.1 to 1082.0)	688.2 (645.5 to 733.1)	0.0 (0.0 to 0.0)
Australia	2022	5936.3 (5814.1 to 6050.5)	4033.2 (3944.2 to 4120.2)	1140.6 (1076.1 to 1205.4)	762.5 (716.3 to 812.1)	0.0 (0.0 to 0.0)	5424.1 (5312.4 to 5528.5)	3685.2 (3603.8 to 3764.7)	1042.1 (983.3 to 1101.4)	696.7 (654.5 to 742.0)	0.0 (0.0 to 0.0)
Australia	2023	6018.5 (5892.2 to 6139.3)	4088.1 (3995.8 to 4181.2)	1158.1 (1095.6 to 1227.7)	772.3 (724.5 to 822.5)	0.0 (0.0 to 0.0)	5499.2 (5383.8 to 5609.6)	3735.4 (3651.1 to 3820.5)	1058.2 (1001.0 to 1121.8)	705.7 (662.0 to 751.6)	0.0 (0.0 to 0.0)
Australia	2024	6105.6 (5971.1 to 6233.0)	4147.1 (4050.9 to 4249.9)	1175.9 (1102.0 to 1249.7)	782.5 (734.5 to 833.8)	0.0 (0.0 to 0.0)	5578.8 (5455.9 to 5695.2)	3789.3 (3701.4 to 3883.2)	1074.5 (1006.9 to 1141.9)	715.0 (671.2 to 761.8)	0.0 (0.0 to 0.0)
Australia	2025	6192.8 (6047.3 to 6325.6)	4206.2 (4103.7 to 4314.2)	1193.9 (1119.3 to 1273.8)	792.7 (744.5 to 844.5)	0.0 (0.0 to 0.0)	5658.5 (5525.6 to 5779.9)	3843.3 (3749.7 to 3942.0)	1090.9 (1022.8 to 1163.9)	724.3 (680.2 to 771.6)	0.0 (0.0 to 0.0)
Australia	2026	6283.3 (6125.9 to 6438.0)	4267.4 (4155.0 to 4384.0)	1212.4 (1131.0 to 1306.1)	803.5 (753.9 to 857.0)	0.0 (0.0 to 0.0)	5741.2 (5597.3 to 5882.6)	3899.2 (3796.5 to 4005.8)	1107.8 (1033.4 to 1193.4)	734.2 (688.8 to 783.1)	0.0 (0.0 to 0.0)
Australia	2027	6373.3 (6200.6 to 6549.6)	4328.0 (4205.3 to 4454.7)	1231.0 (1140.4 to 1338.6)	814.2 (762.2 to 870.1)	0.0 (0.0 to 0.0)	5823.4 (5665.6 to 5984.5)	3954.6 (3842.4 to 4070.4)	1124.8 (1042.0 to 1223.1)	744.0 (696.5 to 795.0)	0.0 (0.0 to 0.0)
Australia	2028	6465.4 (6275.8 to 6660.8)	4390.2 (4254.9 to 4525.6)	1249.9 (1145.9 to 1375.2)	825.2 (770.9 to 882.8)	0.0 (0.0 to 0.0)	5907.5 (5734.3 to 6086.1)	4011.4 (3887.7 to 4135.1)	1142.1 (1047.0 to 1256.5)	754.0 (704.3 to 806.6)	0.0 (0.0 to 0.0)
Australia	2029	6558.9 (6359.6 to 6779.4)	4453.3 (4304.4 to 4604.8)	1269.1 (1149.8 to 1412.8)	836.6 (779.7 to 895.8)	0.0 (0.0 to 0.0)	5993.0 (5810.9 to 6194.5)	4069.0 (3933.0 to 4207.5)	1159.6 (1050.6 to 1290.9)	764.4 (712.4 to 818.5)	0.0 (0.0 to 0.0)
Australia	2030	6654.5 (6434.6 to 6895.6)	4517.5 (4354.1 to 4688.4)	1288.6 (1156.0 to 1452.2)	848.4 (789.0 to 910.0)	0.0 (0.0 to 0.0)	6080.3 (5879.4 to 6300.6)	4127.7 (3978.4 to 4283.8)	1177.4 (1056.3 to 1326.9)	775.2 (720.9 to 831.5)	0.0 (0.0 to 0.0)
Australia	2031	6749.3 (6508.3 to 7012.5)	4580.5 (4402.2 to 4765.3)	1308.1 (1159.9 to 1490.3)	860.6 (798.1 to 925.0)	0.0 (0.0 to 0.0)	6167.0 (5946.7 to 6407.5)	4185.3 (4022.4 to 4354.2)	1195.3 (1059.8 to 1361.7)	786.4 (729.2 to 845.2)	0.0 (0.0 to 0.0)
Australia	2032	6847.4 (6584.9 to 7143.1)	4645.7 (4449.7 to 4845.3)	1328.1 (1163.8 to 1526.1)	873.6 (809.6 to 941.1)	0.0 (0.0 to 0.0)	6256.6 (6016.8 to 6526.8)	4244.9 (4065.8 to 4427.3)	1213.5 (1063.4 to 1394.4)	798.2 (739.7 to 859.9)	0.0 (0.0 to 0.0)
Australia	2033	6948.1 (6653.0 to 7272.2)	4712.7 (4500.7 to 4926.2)	1348.4 (1173.6 to 1564.6)	887.1 (820.3 to 957.3)	0.0 (0.0 to 0.0)	6348.6 (6079.0 to 6644.7)	4306.0 (4112.3 to 4501.2)	1232.0 (1072.4 to 1429.6)	810.5 (749.5 to 874.7)	0.0 (0.0 to 0.0)
Australia	2034	7051.4 (6742.7 to 7395.9)	4781.7 (4552.7 to 5006.9)	1368.9 (1179.3 to 1602.7)	900.8 (830.8 to 974.6)	0.0 (0.0 to 0.0)	6443.0 (6161.0 to 6757.8)	4369.2 (4159.8 to 4574.9)	1250.8 (1077.6 to 1464.4)	823.1 (759.1 to 890.5)	0.0 (0.0 to 0.0)
Australia	2035	7159.6 (6828.7 to 7517.8)	4854.7 (4610.3 to 5099.7)	1389.8 (1186.4 to 1640.7)	915.1 (841.7 to 992.2)	0.0 (0.0 to 0.0)	6541.9 (6239.5 to 6869.2)	4435.9 (4212.5 to 4659.7)	1269.9 (1084.1 to 1499.1)	836.1 (769.1 to 906.6)	0.0 (0.0 to 0.0)
Australia	2036	7266.1 (6904.1 to 7647.8)	4926.0 (4660.6 to 5182.7)	1410.7 (1195.6 to 1677.6)	929.4 (852.0 to 1008.9)	0.0 (0.0 to 0.0)	6639.2 (6308.4 to 6987.9)	4500.9 (4258.4 to 4735.5)	1289.0 (1092.4 to 1532.9)	849.2 (778.5 to 921.8)	0.0 (0.0 to 0.0)

		2018 US Dollars per capita					2018 PPP per capita				
Country	Year	Health spending	Government health spending	Out-of-pocket health spending	Prepaid private health spending	Development assistance for health	Health spending	Government health spending	Out-of-pocket health spending	Prepaid private health spending	Development assistance for health
Australia	2037	7373.5 (6987.5 to 7787.1)	4997.9 (4713.0 to 5277.1)	1431.7 (1204.6 to 1714.2)	943.9 (862.7 to 1026.3)	0.0 (0.0 to 0.0)	6737.3 (6384.6 to 7115.2)	4566.7 (4306.4 to 4821.8)	1308.1 (1100.7 to 1566.3)	862.5 (788.3 to 937.8)	0.0 (0.0 to 0.0)
Australia	2038	7485.8 (7080.8 to 7908.8)	5073.8 (4769.6 to 5371.6)	1453.0 (1214.1 to 1752.7)	959.0 (873.9 to 1045.2)	0.0 (0.0 to 0.0)	6839.9 (6469.9 to 7226.4)	4636.0 (4358.1 to 4908.1)	1327.7 (1109.4 to 1601.5)	876.3 (798.5 to 955.0)	0.0 (0.0 to 0.0)
Australia	2039	7599.2 (7178.2 to 8060.3)	5150.3 (4825.4 to 5472.4)	1474.5 (1224.3 to 1789.9)	974.4 (885.9 to 1064.9)	0.0 (0.0 to 0.0)	6943.5 (6558.9 to 7364.8)	4705.9 (4409.0 to 5000.2)	1347.3 (1118.7 to 1635.5)	890.3 (809.4 to 973.0)	0.0 (0.0 to 0.0)
Australia	2040	7715.1 (7264.3 to 8195.3)	5228.4 (4882.9 to 5568.7)	1496.3 (1235.9 to 1825.8)	990.4 (898.1 to 1085.5)	0.0 (0.0 to 0.0)	7049.4 (6637.5 to 7488.2)	4777.3 (4461.6 to 5088.2)	1367.2 (1129.2 to 1668.3)	904.9 (820.6 to 991.9)	0.0 (0.0 to 0.0)
Australia	2041	7826.4 (7359.1 to 8336.3)	5302.2 (4940.4 to 5660.9)	1517.7 (1248.5 to 1859.0)	1006.6 (910.9 to 1105.7)	0.0 (0.0 to 0.0)	7151.2 (6724.1 to 7617.1)	4844.7 (4514.2 to 5172.5)	1386.7 (1140.7 to 1698.6)	919.7 (832.3 to 1010.3)	0.0 (0.0 to 0.0)
Australia	2042	7941.0 (7437.0 to 8483.5)	5378.0 (4992.0 to 5755.2)	1539.4 (1261.9 to 1890.8)	1023.6 (923.8 to 1127.2)	0.0 (0.0 to 0.0)	7255.8 (6795.3 to 7751.5)	4914.0 (4561.3 to 5258.6)	1406.5 (1153.0 to 1727.6)	935.3 (844.1 to 1029.9)	0.0 (0.0 to 0.0)
Australia	2043	8057.8 (7541.2 to 8626.8)	5455.5 (5037.7 to 5851.8)	1561.2 (1276.6 to 1920.0)	1041.1 (936.7 to 1150.2)	0.0 (0.0 to 0.0)	7362.6 (6890.5 to 7882.5)	4984.8 (4603.0 to 5346.9)	1426.5 (1166.4 to 1754.3)	951.3 (855.9 to 1050.9)	0.0 (0.0 to 0.0)
Australia	2044	8175.6 (7655.6 to 8753.5)	5534.0 (5091.9 to 5956.4)	1583.0 (1289.7 to 1946.8)	1058.6 (950.1 to 1172.9)	0.0 (0.0 to 0.0)	7470.2 (6995.1 to 7998.2)	5056.5 (4652.5 to 5442.4)	1446.4 (1178.4 to 1778.8)	967.3 (868.1 to 1071.7)	0.0 (0.0 to 0.0)
Australia	2045	8294.9 (7755.3 to 8878.3)	5614.3 (5149.1 to 6055.1)	1604.8 (1303.8 to 1976.7)	1075.9 (962.9 to 1195.4)	0.0 (0.0 to 0.0)	7579.2 (7086.2 to 8112.3)	5129.9 (4704.8 to 5532.6)	1466.3 (1191.3 to 1806.2)	983.0 (879.8 to 1092.2)	0.0 (0.0 to 0.0)
Australia	2046	8408.7 (7860.3 to 9043.0)	5690.0 (5200.0 to 6150.9)	1626.0 (1321.1 to 2003.7)	1092.6 (975.2 to 1217.4)	0.0 (0.0 to 0.0)	7683.1 (7182.1 to 8262.8)	5199.1 (4751.3 to 5620.2)	1485.7 (1207.1 to 1830.8)	998.3 (891.1 to 1112.4)	0.0 (0.0 to 0.0)
Australia	2047	8523.9 (7932.4 to 9170.0)	5767.4 (5263.1 to 6255.6)	1647.2 (1340.5 to 2031.0)	1109.4 (987.3 to 1239.6)	0.0 (0.0 to 0.0)	7788.5 (7248.0 to 8378.8)	5269.7 (4809.0 to 5715.8)	1505.1 (1224.9 to 1855.7)	1013.7 (902.2 to 1132.6)	0.0 (0.0 to 0.0)
Australia	2048	8638.6 (8043.2 to 9318.4)	5844.3 (5314.1 to 6356.2)	1668.5 (1358.0 to 2055.7)	1125.8 (999.0 to 1261.9)	0.0 (0.0 to 0.0)	7893.2 (7349.2 to 8514.4)	5340.1 (4855.6 to 5807.8)	1524.5 (1240.8 to 1878.3)	1028.6 (912.8 to 1153.1)	0.0 (0.0 to 0.0)
Australia	2049	8756.7 (8137.1 to 9475.1)	5924.0 (5371.8 to 6464.4)	1690.2 (1375.0 to 2084.3)	1142.5 (1009.9 to 1283.8)	0.0 (0.0 to 0.0)	8001.1 (7435.1 to 8657.6)	5412.9 (4908.3 to 5906.7)	1544.4 (1256.4 to 1904.4)	1043.9 (922.8 to 1173.1)	0.0 (0.0 to 0.0)
Australia	2050	8875.4 (8221.6 to 9605.3)	6004.2 (5423.6 to 6571.8)	1712.1 (1394.3 to 2113.2)	1159.1 (1020.7 to 1305.8)	0.0 (0.0 to 0.0)	8109.6 (7512.2 to 8776.6)	5486.2 (4955.6 to 6004.8)	1564.3 (1274.0 to 1930.8)	1059.1 (932.6 to 1193.2)	0.0 (0.0 to 0.0)
Austria	1995	3668.3 (3574.4 to 3764.8)	2541.8 (2470.0 to 2611.8)	615.8 (577.1 to 655.9)	510.8 (453.8 to 567.8)	0.0 (0.0 to 0.0)	3644.5 (3551.2 to 3740.3)	2525.3 (2453.9 to 2594.9)	611.8 (573.4 to 651.6)	507.4 (450.9 to 564.1)	0.0 (0.0 to 0.0)
Austria	1996	3711.4 (3642.2 to 3780.1)	2606.7 (2558.3 to 2657.6)	635.3 (607.5 to 665.8)	469.4 (431.1 to 512.1)	0.0 (0.0 to 0.0)	3687.3 (3618.5 to 3755.6)	2589.7 (2541.6 to 2640.3)	631.2 (603.5 to 661.5)	466.3 (428.3 to 508.8)	0.0 (0.0 to 0.0)
Austria	1997	3761.5 (3694.7 to 3830.0)	2709.0 (2659.6 to 2764.6)	656.1 (629.1 to 684.9)	396.3 (365.2 to 430.1)	0.0 (0.0 to 0.0)	3737.0 (3670.7 to 3805.0)	2691.4 (2642.3 to 2746.6)	651.9 (625.0 to 680.5)	393.8 (362.8 to 427.3)	0.0 (0.0 to 0.0)
Austria	1998	3881.8 (3813.6 to 3951.9)	2841.7 (2789.7 to 2898.5)	681.2 (652.0 to 710.0)	358.8 (328.7 to 392.1)	0.0 (0.0 to 0.0)	3856.5 (3788.7 to 3926.2)	2823.2 (2771.6 to 2879.6)	676.8 (647.7 to 705.3)	356.5 (326.5 to 389.5)	0.0 (0.0 to 0.0)
Austria	1999	3989.4 (3920.0 to 4052.9)	2948.6 (2898.0 to 2999.7)	703.2 (676.2 to 728.6)	337.5 (308.3 to 368.6)	0.0 (0.0 to 0.0)	3963.4 (3894.5 to 4026.5)	2929.4 (2879.1 to 2980.2)	698.6 (671.8 to 723.9)	335.4 (306.3 to 366.2)	0.0 (0.0 to 0.0)
Austria	2000	4081.6 (4018.5 to 4142.9)	3025.1 (2976.4 to 3075.5)	729.5 (706.0 to 755.3)	327.0 (300.7 to 357.1)	0.0 (0.0 to 0.0)	4055.1 (3992.4 to 4115.9)	3005.4 (2957.1 to 3055.5)	724.8 (701.4 to 750.4)	324.9 (298.7 to 354.8)	0.0 (0.0 to 0.0)
Austria	2001	4126.5 (4066.5 to 4185.6)	3044.1 (2993.5 to 3093.9)	757.0 (734.7 to 781.6)	325.4 (299.5 to 355.8)	0.0 (0.0 to 0.0)	4099.7 (4040.0 to 4158.3)	3024.3 (2974.0 to 3073.8)	752.1 (729.9 to 776.5)	323.3 (297.5 to 353.5)	0.0 (0.0 to 0.0)

Country	Year	2018 US Dollars per capita					2018 PPP per capita				
		Health spending	Government health spending	Out-of-pocket health spending	Prepaid private health spending	Development assistance for health	Health spending	Government health spending	Out-of-pocket health spending	Prepaid private health spending	Development assistance for health
Austria	2002	4237.4 (4178.0 to 4298.6)	3110.1 (3060.8 to 3159.1)	790.5 (767.6 to 813.8)	336.8 (310.9 to 365.1)	0.0 (0.0 to 0.0)	4209.8 (4150.8 to 4270.6)	3089.9 (3040.9 to 3138.5)	785.3 (762.6 to 808.5)	334.6 (308.9 to 362.7)	0.0 (0.0 to 0.0)
Austria	2003	4313.7 (4250.9 to 4376.9)	3145.0 (3096.1 to 3191.4)	819.2 (794.9 to 844.4)	349.4 (322.1 to 376.3)	0.0 (0.0 to 0.0)	4285.6 (4223.2 to 4348.4)	3124.6 (3076.0 to 3170.6)	813.9 (789.7 to 838.9)	347.1 (320.0 to 373.9)	0.0 (0.0 to 0.0)
Austria	2004	4450.1 (4389.0 to 4514.1)	3227.3 (3179.2 to 3271.9)	852.9 (826.6 to 877.4)	370.0 (342.8 to 398.0)	0.0 (0.0 to 0.0)	4421.2 (4360.4 to 4484.7)	3206.3 (3158.5 to 3250.6)	847.3 (821.2 to 871.7)	367.6 (340.6 to 395.4)	0.0 (0.0 to 0.0)
Austria	2005	4504.0 (4444.2 to 4563.7)	3266.0 (3219.8 to 3310.3)	863.5 (840.7 to 887.3)	374.5 (348.2 to 401.4)	0.0 (0.0 to 0.0)	4474.7 (4415.3 to 4534.0)	3244.8 (3198.9 to 3288.7)	857.9 (835.2 to 881.5)	372.0 (346.0 to 398.8)	0.0 (0.0 to 0.0)
Austria	2006	4611.9 (4549.6 to 4673.7)	3355.2 (3307.0 to 3406.0)	872.8 (849.0 to 897.3)	383.9 (357.7 to 414.1)	0.0 (0.0 to 0.0)	4581.9 (4520.0 to 4643.3)	3333.3 (3285.5 to 3383.9)	867.2 (843.5 to 891.4)	381.4 (355.4 to 411.4)	0.0 (0.0 to 0.0)
Austria	2007	4767.5 (4698.7 to 4832.1)	3481.9 (3428.1 to 3535.0)	887.7 (861.9 to 911.5)	397.9 (370.2 to 427.3)	0.0 (0.0 to 0.0)	4736.5 (4668.2 to 4800.7)	3459.3 (3405.7 to 3512.0)	882.0 (856.3 to 905.6)	395.3 (367.7 to 424.6)	0.0 (0.0 to 0.0)
Austria	2008	4931.0 (4864.9 to 4996.4)	3615.4 (3563.4 to 3663.9)	900.1 (875.1 to 924.2)	415.5 (387.9 to 446.3)	0.0 (0.0 to 0.0)	4898.9 (4833.2 to 4963.9)	3591.8 (3540.2 to 3640.0)	894.3 (869.4 to 918.2)	412.8 (385.4 to 443.4)	0.0 (0.0 to 0.0)
Austria	2009	4930.8 (4868.9 to 4995.2)	3595.6 (3545.9 to 3648.0)	901.7 (875.9 to 927.5)	433.5 (405.0 to 463.1)	0.0 (0.0 to 0.0)	4898.8 (4837.2 to 4962.7)	3572.2 (3522.8 to 3624.3)	895.9 (870.2 to 921.5)	430.7 (402.4 to 460.1)	0.0 (0.0 to 0.0)
Austria	2010	5001.6 (4939.6 to 5061.8)	3634.4 (3583.8 to 3682.4)	924.4 (897.8 to 950.4)	442.8 (412.9 to 473.7)	0.0 (0.0 to 0.0)	4969.0 (4907.5 to 5028.9)	3610.7 (3560.5 to 3658.4)	918.4 (891.9 to 944.2)	439.9 (410.2 to 470.7)	0.0 (0.0 to 0.0)
Austria	2011	5085.5 (5019.0 to 5151.0)	3694.8 (3644.8 to 3749.5)	948.1 (920.7 to 974.5)	442.6 (413.0 to 474.1)	0.0 (0.0 to 0.0)	5052.4 (4986.4 to 5117.5)	3670.8 (3621.0 to 3725.1)	941.9 (914.7 to 968.1)	439.7 (410.3 to 471.0)	0.0 (0.0 to 0.0)
Austria	2012	5146.7 (5080.5 to 5214.0)	3737.0 (3687.1 to 3787.7)	967.9 (941.6 to 994.9)	441.8 (412.7 to 471.6)	0.0 (0.0 to 0.0)	5113.2 (5047.5 to 5180.1)	3712.7 (3663.1 to 3763.1)	961.6 (935.5 to 988.4)	439.0 (410.0 to 468.5)	0.0 (0.0 to 0.0)
Austria	2013	5170.7 (5106.6 to 5237.8)	3745.6 (3695.7 to 3797.0)	984.5 (958.0 to 1010.8)	440.6 (409.5 to 472.7)	0.0 (0.0 to 0.0)	5137.1 (5073.3 to 5203.8)	3721.2 (3671.6 to 3772.3)	978.1 (951.8 to 1004.2)	437.7 (406.8 to 469.6)	0.0 (0.0 to 0.0)
Austria	2014	5205.8 (5141.6 to 5271.6)	3774.1 (3722.7 to 3824.9)	993.7 (968.3 to 1019.7)	438.0 (407.9 to 471.8)	0.0 (0.0 to 0.0)	5171.9 (5108.1 to 5237.3)	3749.5 (3698.5 to 3800.0)	987.3 (962.0 to 1013.1)	435.1 (405.3 to 468.7)	0.0 (0.0 to 0.0)
Austria	2015	5237.9 (5174.4 to 5308.3)	3803.8 (3752.0 to 3852.4)	996.6 (970.7 to 1024.6)	437.5 (407.1 to 471.9)	0.0 (0.0 to 0.0)	5203.8 (5140.7 to 5273.8)	3779.0 (3727.6 to 3827.4)	990.1 (964.4 to 1018.0)	434.7 (404.4 to 468.8)	0.0 (0.0 to 0.0)
Austria	2016	5286.6 (5199.4 to 5378.7)	3837.4 (3772.8 to 3907.6)	1000.7 (961.8 to 1038.0)	448.5 (408.0 to 495.8)	0.0 (0.0 to 0.0)	5252.2 (5165.6 to 5343.7)	3812.4 (3748.3 to 3882.2)	994.2 (955.5 to 1031.2)	445.6 (405.4 to 492.5)	0.0 (0.0 to 0.0)
Austria	2017	5358.7 (5265.0 to 5456.7)	3881.3 (3809.9 to 3956.6)	1019.9 (980.5 to 1058.5)	457.6 (415.8 to 504.6)	0.0 (0.0 to 0.0)	5323.9 (5230.7 to 5421.2)	3856.1 (3785.1 to 3930.9)	1013.2 (974.1 to 1051.6)	454.6 (413.1 to 501.3)	0.0 (0.0 to 0.0)
Austria	2018	5433.2 (5331.9 to 5533.9)	3933.4 (3855.1 to 4012.7)	1034.4 (993.1 to 1078.0)	465.3 (423.6 to 512.8)	0.0 (0.0 to 0.0)	5397.8 (5297.2 to 5497.9)	3907.9 (3830.0 to 3986.6)	1027.7 (986.6 to 1071.0)	462.3 (420.8 to 509.4)	0.0 (0.0 to 0.0)
Austria	2019	5503.0 (5404.2 to 5608.6)	3982.7 (3900.1 to 4069.4)	1048.4 (1002.7 to 1096.6)	471.9 (429.5 to 520.3)	0.0 (0.0 to 0.0)	5467.2 (5369.0 to 5572.1)	3956.7 (3874.7 to 4042.9)	1041.6 (996.2 to 1089.5)	468.9 (426.7 to 516.9)	0.0 (0.0 to 0.0)
Austria	2020	5570.2 (5454.0 to 5680.2)	4029.7 (3937.8 to 4129.5)	1062.1 (1013.3 to 1114.9)	478.4 (434.6 to 527.7)	0.0 (0.0 to 0.0)	5533.9 (5418.5 to 5643.3)	4003.5 (3912.2 to 4102.6)	1055.2 (1006.7 to 1107.6)	475.2 (431.8 to 524.3)	0.0 (0.0 to 0.0)
Austria	2021	5635.0 (5507.8 to 5762.5)	4075.1 (3974.6 to 4186.5)	1075.6 (1024.7 to 1131.7)	484.3 (439.5 to 533.8)	0.0 (0.0 to 0.0)	5598.3 (5472.0 to 5725.0)	4048.6 (3948.7 to 4159.3)	1068.6 (1018.0 to 1124.4)	481.1 (436.7 to 530.4)	0.0 (0.0 to 0.0)
Austria	2022	5701.6 (5560.7 to 5846.3)	4122.2 (4010.1 to 4245.9)	1089.1 (1034.5 to 1147.3)	490.2 (444.8 to 540.1)	0.0 (0.0 to 0.0)	5664.5 (5524.5 to 5808.2)	4095.4 (3984.0 to 4218.3)	1082.1 (1027.8 to 1139.9)	487.0 (441.9 to 536.6)	0.0 (0.0 to 0.0)

Country	Year	2018 US Dollars per capita					2018 PPP per capita				
		Health spending	Government health spending	Out-of-pocket health spending	Prepaid private health spending	Development assistance for health	Health spending	Government health spending	Out-of-pocket health spending	Prepaid private health spending	Development assistance for health
Austria	2023	5768.7 (5615.6 to 5933.5)	4169.7 (4043.4 to 4311.2)	1103.1 (1043.6 to 1163.2)	495.9 (449.3 to 546.3)	0.0 (0.0 to 0.0)	5731.1 (5579.1 to 5894.9)	4142.6 (4017.1 to 4283.2)	1095.9 (1036.8 to 1155.7)	492.7 (446.4 to 542.8)	0.0 (0.0 to 0.0)
Austria	2024	5840.1 (5670.8 to 6011.2)	4221.5 (4076.9 to 4374.8)	1117.0 (1052.4 to 1180.2)	501.6 (453.8 to 553.2)	0.0 (0.0 to 0.0)	5802.1 (5633.9 to 5972.1)	4194.0 (4050.4 to 4346.3)	1109.8 (1045.5 to 1172.5)	498.3 (450.8 to 549.6)	0.0 (0.0 to 0.0)
Austria	2025	5909.5 (5728.2 to 6092.5)	4272.5 (4109.4 to 4445.2)	1130.1 (1063.8 to 1198.8)	506.9 (458.2 to 558.3)	0.0 (0.0 to 0.0)	5871.0 (5690.9 to 6052.8)	4244.7 (4082.7 to 4416.3)	1122.8 (1056.8 to 1191.0)	503.6 (455.2 to 554.6)	0.0 (0.0 to 0.0)
Austria	2026	5973.9 (5773.7 to 6177.6)	4318.9 (4141.8 to 4508.9)	1143.1 (1069.8 to 1218.0)	511.9 (462.7 to 563.6)	0.0 (0.0 to 0.0)	5935.0 (5736.2 to 6137.4)	4290.8 (4114.8 to 4479.6)	1135.6 (1062.9 to 1210.1)	508.6 (459.7 to 559.9)	0.0 (0.0 to 0.0)
Austria	2027	6037.6 (5824.8 to 6263.7)	4364.9 (4167.4 to 4567.5)	1155.9 (1073.6 to 1241.6)	516.7 (466.8 to 570.1)	0.0 (0.0 to 0.0)	5998.3 (5786.9 to 6222.9)	4336.5 (4140.2 to 4537.8)	1148.4 (1066.6 to 1233.6)	513.3 (463.8 to 566.3)	0.0 (0.0 to 0.0)
Austria	2028	6099.8 (5858.1 to 6363.3)	4409.9 (4197.8 to 4637.2)	1168.7 (1075.0 to 1264.1)	521.3 (470.8 to 576.2)	0.0 (0.0 to 0.0)	6060.1 (5819.9 to 6321.9)	4381.2 (4170.5 to 4607.0)	1161.1 (1068.0 to 1255.9)	517.9 (467.7 to 572.5)	0.0 (0.0 to 0.0)
Austria	2029	6159.2 (5893.8 to 6443.4)	4452.6 (4221.5 to 4694.1)	1181.1 (1076.2 to 1292.8)	525.5 (473.6 to 582.1)	0.0 (0.0 to 0.0)	6119.1 (5855.5 to 6401.5)	4423.6 (4194.0 to 4663.6)	1173.4 (1069.2 to 1284.4)	522.1 (470.5 to 578.3)	0.0 (0.0 to 0.0)
Austria	2030	6216.7 (5937.7 to 6538.7)	4493.7 (4242.6 to 4768.2)	1193.4 (1076.1 to 1320.5)	529.6 (477.5 to 587.9)	0.0 (0.0 to 0.0)	6176.3 (5899.1 to 6496.1)	4464.4 (4215.0 to 4737.1)	1185.6 (1069.1 to 1311.9)	526.2 (474.4 to 584.0)	0.0 (0.0 to 0.0)
Austria	2031	6273.6 (5963.5 to 6608.8)	4533.9 (4267.2 to 4823.7)	1205.9 (1078.5 to 1349.9)	533.9 (480.6 to 592.3)	0.0 (0.0 to 0.0)	6232.8 (5924.7 to 6565.8)	4504.4 (4239.5 to 4792.3)	1198.0 (1071.5 to 1341.1)	530.4 (477.5 to 588.5)	0.0 (0.0 to 0.0)
Austria	2032	6328.0 (5994.8 to 6691.6)	4571.9 (4283.1 to 4890.7)	1218.2 (1081.0 to 1376.1)	537.9 (484.9 to 596.4)	0.0 (0.0 to 0.0)	6286.8 (5955.8 to 6648.0)	4542.2 (4255.2 to 4858.9)	1210.2 (1074.0 to 1367.2)	534.4 (481.7 to 592.5)	0.0 (0.0 to 0.0)
Austria	2033	6386.0 (6055.2 to 6771.7)	4612.8 (4300.1 to 4940.3)	1230.8 (1080.8 to 1407.3)	542.3 (488.6 to 601.8)	0.0 (0.0 to 0.0)	6344.4 (6015.8 to 6727.6)	4582.8 (4272.1 to 4908.1)	1222.8 (1073.7 to 1398.1)	538.8 (485.4 to 597.9)	0.0 (0.0 to 0.0)
Austria	2034	6445.9 (6077.9 to 6851.6)	4654.7 (4325.0 to 4998.8)	1244.0 (1083.9 to 1437.6)	547.2 (492.5 to 607.4)	0.0 (0.0 to 0.0)	6403.9 (6038.3 to 6807.0)	4624.4 (4296.9 to 4966.2)	1235.9 (1076.8 to 1428.2)	543.6 (489.3 to 603.4)	0.0 (0.0 to 0.0)
Austria	2035	6507.9 (6115.9 to 6935.9)	4697.7 (4343.7 to 5072.9)	1257.6 (1084.9 to 1463.3)	552.6 (496.4 to 613.9)	0.0 (0.0 to 0.0)	6465.6 (6076.1 to 6890.8)	4667.2 (4315.5 to 5039.9)	1249.4 (1077.8 to 1453.8)	549.0 (493.2 to 609.9)	0.0 (0.0 to 0.0)
Austria	2036	6572.4 (6151.8 to 7031.3)	4742.0 (4378.2 to 5136.6)	1271.8 (1089.6 to 1491.1)	558.5 (500.8 to 622.2)	0.0 (0.0 to 0.0)	6529.6 (6111.8 to 6985.6)	4711.2 (4349.7 to 5103.2)	1263.5 (1082.5 to 1481.4)	554.9 (497.6 to 618.2)	0.0 (0.0 to 0.0)
Austria	2037	6640.0 (6183.3 to 7108.4)	4788.2 (4410.2 to 5207.4)	1286.6 (1095.6 to 1518.7)	565.1 (505.8 to 631.3)	0.0 (0.0 to 0.0)	6596.8 (6143.1 to 7062.1)	4757.1 (4381.5 to 5173.5)	1278.2 (1088.5 to 1508.9)	561.4 (502.5 to 627.2)	0.0 (0.0 to 0.0)
Austria	2038	6711.2 (6238.6 to 7209.1)	4836.7 (4449.7 to 5274.6)	1302.1 (1102.9 to 1545.5)	572.4 (511.2 to 640.7)	0.0 (0.0 to 0.0)	6667.5 (6198.0 to 7162.2)	4805.2 (4420.7 to 5240.2)	1293.6 (1095.7 to 1535.4)	568.6 (507.9 to 636.5)	0.0 (0.0 to 0.0)
Austria	2039	6784.2 (6294.2 to 7293.7)	4886.3 (4479.8 to 5333.2)	1317.9 (1109.7 to 1575.4)	580.0 (517.1 to 649.4)	0.0 (0.0 to 0.0)	6740.1 (6253.2 to 7246.2)	4854.5 (4450.7 to 5298.5)	1309.4 (1102.5 to 1565.1)	576.2 (513.7 to 645.2)	0.0 (0.0 to 0.0)
Austria	2040	6860.6 (6343.7 to 7372.2)	4938.5 (4515.1 to 5410.6)	1334.2 (1116.5 to 1604.9)	588.0 (523.2 to 658.6)	0.0 (0.0 to 0.0)	6816.0 (6302.4 to 7324.2)	4906.4 (4485.7 to 5375.4)	1325.5 (1109.2 to 1594.5)	584.1 (519.8 to 654.3)	0.0 (0.0 to 0.0)
Austria	2041	6937.2 (6417.4 to 7492.3)	4990.0 (4551.4 to 5480.8)	1350.8 (1125.2 to 1633.9)	596.4 (529.3 to 668.6)	0.0 (0.0 to 0.0)	6892.0 (6375.7 to 7443.6)	4957.6 (4521.8 to 5445.2)	1342.0 (1117.9 to 1623.3)	592.5 (525.9 to 664.2)	0.0 (0.0 to 0.0)
Austria	2042	7016.2 (6488.6 to 7628.6)	5043.1 (4586.7 to 5540.5)	1367.8 (1136.0 to 1662.4)	605.3 (536.5 to 680.7)	0.0 (0.0 to 0.0)	6970.5 (6446.4 to 7579.0)	5010.3 (4556.8 to 5504.4)	1358.9 (1128.6 to 1651.6)	601.3 (533.1 to 676.3)	0.0 (0.0 to 0.0)
Austria	2043	7099.7 (6544.4 to 7730.0)	5099.7 (4624.9 to 5613.4)	1385.4 (1148.1 to 1684.3)	614.7 (543.9 to 692.5)	0.0 (0.0 to 0.0)	7053.5 (6501.8 to 7679.7)	5066.5 (4594.9 to 5576.9)	1376.4 (1140.6 to 1673.4)	610.7 (540.4 to 688.0)	0.0 (0.0 to 0.0)

		2018 US Dollars per capita					2018 PPP per capita				
Country	Year	Health spending	Government health spending	Out-of-pocket health spending	Prepaid private health spending	Development assistance for health	Health spending	Government health spending	Out-of-pocket health spending	Prepaid private health spending	Development assistance for health
Austria	2044	7181.9 (6615.3 to 7820.6)	5155.3 (4664.7 to 5697.5)	1402.7 (1160.8 to 1703.5)	623.9 (550.9 to 704.0)	0.0 (0.0 to 0.0)	7135.2 (6572.3 to 7769.8)	5121.7 (4634.3 to 5660.4)	1393.6 (1153.3 to 1692.4)	619.8 (547.3 to 699.4)	0.0 (0.0 to 0.0)
Austria	2045	7265.0 (6669.3 to 7905.4)	5212.2 (4710.1 to 5768.3)	1419.9 (1174.1 to 1720.6)	632.9 (557.0 to 716.5)	0.0 (0.0 to 0.0)	7217.8 (6625.9 to 7854.0)	5178.3 (4679.4 to 5730.8)	1410.7 (1166.5 to 1709.4)	628.8 (553.4 to 711.8)	0.0 (0.0 to 0.0)
Austria	2046	7344.5 (6765.2 to 8036.4)	5265.8 (4738.3 to 5848.3)	1436.8 (1188.4 to 1743.4)	641.9 (563.4 to 728.3)	0.0 (0.0 to 0.0)	7296.7 (6721.1 to 7984.1)	5231.6 (4707.4 to 5810.2)	1427.5 (1180.7 to 1732.0)	637.7 (559.7 to 723.6)	0.0 (0.0 to 0.0)
Austria	2047	7423.5 (6819.7 to 8128.9)	5319.2 (4766.5 to 5920.4)	1453.6 (1203.4 to 1765.6)	650.7 (569.5 to 741.9)	0.0 (0.0 to 0.0)	7375.2 (6775.3 to 8076.0)	5284.6 (4735.4 to 5881.9)	1444.2 (1195.5 to 1754.1)	646.4 (565.8 to 737.1)	0.0 (0.0 to 0.0)
Austria	2048	7502.5 (6896.3 to 8217.7)	5372.8 (4800.0 to 6003.0)	1470.4 (1218.9 to 1788.5)	659.3 (575.5 to 753.2)	0.0 (0.0 to 0.0)	7453.7 (6851.4 to 8164.2)	5337.9 (4768.8 to 5963.9)	1460.8 (1210.9 to 1776.9)	655.0 (571.8 to 748.3)	0.0 (0.0 to 0.0)
Austria	2049	7584.9 (6969.1 to 8338.6)	5429.3 (4839.0 to 6071.3)	1487.4 (1234.2 to 1802.4)	668.2 (581.5 to 766.1)	0.0 (0.0 to 0.0)	7535.5 (6923.8 to 8284.3)	5394.0 (4807.5 to 6031.8)	1477.7 (1226.2 to 1790.7)	663.8 (577.7 to 761.2)	0.0 (0.0 to 0.0)
Austria	2050	7665.7 (7012.1 to 8382.2)	5484.4 (4874.5 to 6154.4)	1504.4 (1248.5 to 1823.5)	677.0 (586.8 to 779.0)	0.0 (0.0 to 0.0)	7615.9 (6966.5 to 8327.7)	5448.7 (4842.8 to 6114.4)	1494.6 (1240.4 to 1811.6)	672.6 (583.0 to 773.9)	0.0 (0.0 to 0.0)
Azerbaijan	1995	48.7 (40.0 to 58.9)	13.4 (10.1 to 17.6)	35.3 (27.4 to 45.0)	0.0 (0.0 to 0.0)	0.0 (0.0 to 0.0)	195.7 (160.6 to 236.6)	54.0 (40.6 to 70.7)	141.7 (110.3 to 180.7)	0.0 (0.0 to 0.0)	0.0 (0.0 to 0.0)
Azerbaijan	1996	49.5 (41.0 to 59.5)	12.8 (9.7 to 16.6)	36.7 (28.8 to 46.1)	0.0 (0.0 to 0.1)	0.1 (0.1 to 0.1)	199.1 (164.8 to 239.1)	51.2 (38.9 to 66.6)	147.4 (115.8 to 185.3)	0.1 (0.1 to 0.2)	0.3 (0.3 to 0.3)
Azerbaijan	1997	49.8 (41.4 to 59.5)	11.8 (9.0 to 15.1)	36.8 (29.3 to 46.3)	0.1 (0.0 to 0.2)	1.2 (1.2 to 1.2)	200.2 (166.3 to 239.1)	47.3 (36.1 to 60.8)	147.9 (117.7 to 186.1)	0.4 (0.2 to 0.8)	4.7 (4.7 to 4.7)
Azerbaijan	1998	48.8 (40.7 to 58.3)	10.7 (8.3 to 13.6)	37.8 (30.3 to 46.9)	0.2 (0.1 to 0.4)	0.1 (0.1 to 0.1)	196.0 (163.6 to 234.4)	42.9 (33.2 to 54.4)	152.0 (121.5 to 188.6)	0.8 (0.3 to 1.5)	0.4 (0.4 to 0.4)
Azerbaijan	1999	53.5 (45.5 to 63.3)	10.7 (8.2 to 13.6)	39.4 (31.8 to 48.4)	0.3 (0.1 to 0.5)	3.1 (3.1 to 3.1)	214.8 (182.8 to 254.2)	43.0 (33.1 to 54.6)	158.2 (127.8 to 194.5)	1.1 (0.5 to 2.0)	12.5 (12.5 to 12.5)
Azerbaijan	2000	54.3 (46.4 to 63.5)	10.9 (8.5 to 14.0)	40.8 (33.5 to 50.0)	0.3 (0.1 to 0.5)	2.3 (2.3 to 2.3)	218.1 (186.4 to 255.3)	43.8 (34.0 to 56.2)	164.0 (134.5 to 200.7)	1.1 (0.5 to 2.1)	9.3 (9.3 to 9.3)
Azerbaijan	2001	57.3 (49.0 to 66.9)	11.4 (8.8 to 14.6)	45.2 (37.4 to 54.6)	0.3 (0.1 to 0.5)	0.4 (0.4 to 0.4)	230.2 (196.9 to 268.6)	45.7 (35.3 to 58.7)	181.8 (150.1 to 219.2)	1.1 (0.5 to 2.2)	1.8 (1.8 to 1.8)
Azerbaijan	2002	67.6 (58.0 to 78.4)	12.0 (9.4 to 15.5)	54.6 (45.5 to 64.9)	0.3 (0.1 to 0.5)	0.8 (0.8 to 0.8)	271.7 (232.9 to 315.0)	48.4 (37.7 to 62.2)	219.2 (182.8 to 260.6)	1.1 (0.5 to 2.2)	3.0 (3.0 to 3.0)
Azerbaijan	2003	89.2 (77.0 to 102.7)	13.6 (10.5 to 17.4)	74.9 (62.9 to 88.1)	0.3 (0.1 to 0.6)	0.5 (0.5 to 0.5)	358.4 (309.3 to 412.5)	54.5 (42.2 to 69.9)	300.9 (252.7 to 354.1)	1.2 (0.5 to 2.3)	1.8 (1.8 to 1.8)
Azerbaijan	2004	112.5 (98.2 to 128.3)	15.5 (12.0 to 20.0)	96.2 (82.5 to 111.4)	0.4 (0.2 to 0.7)	0.4 (0.4 to 0.4)	452.0 (394.5 to 515.3)	62.5 (48.3 to 80.3)	386.5 (331.3 to 447.4)	1.5 (0.7 to 2.9)	1.6 (1.6 to 1.6)
Azerbaijan	2005	139.4 (123.0 to 157.1)	19.3 (15.1 to 24.9)	118.3 (102.7 to 135.7)	0.5 (0.2 to 0.9)	1.3 (1.3 to 1.3)	559.9 (494.2 to 631.1)	77.6 (60.8 to 100.0)	475.3 (412.5 to 545.1)	1.9 (0.9 to 3.6)	5.1 (5.1 to 5.1)
Azerbaijan	2006	161.1 (143.5 to 180.2)	25.5 (20.1 to 32.5)	133.2 (116.2 to 151.4)	0.6 (0.3 to 1.1)	1.8 (1.8 to 1.8)	647.4 (576.7 to 723.8)	102.5 (80.6 to 130.4)	535.2 (467.0 to 608.2)	2.5 (1.1 to 4.5)	7.2 (7.2 to 7.2)
Azerbaijan	2007	176.0 (157.8 to 195.7)	32.8 (26.0 to 41.3)	140.9 (123.8 to 159.5)	0.8 (0.4 to 1.6)	1.5 (1.5 to 1.5)	707.1 (634.0 to 786.4)	131.8 (104.7 to 166.1)	566.0 (497.2 to 640.7)	3.4 (1.6 to 6.2)	6.0 (6.0 to 6.0)
Azerbaijan	2008	184.9 (166.3 to 205.7)	38.1 (30.8 to 47.9)	143.9 (126.5 to 163.1)	1.0 (0.5 to 1.9)	1.8 (1.8 to 1.8)	742.9 (668.1 to 826.5)	153.1 (123.6 to 192.3)	578.3 (508.2 to 655.1)	4.1 (1.9 to 7.4)	7.4 (7.4 to 7.4)

Country	Year	2018 US Dollars per capita					2018 PPP per capita				
		Health spending	Government health spending	Out-of-pocket health spending	Prepaid private health spending	Development assistance for health	Health spending	Government health spending	Out-of-pocket health spending	Prepaid private health spending	Development assistance for health
Azerbaijan	2009	204.2 (183.3 to 228.0)	44.8 (36.3 to 56.2)	155.6 (136.5 to 176.2)	1.3 (0.6 to 2.3)	2.6 (2.6 to 2.6)	820.2 (736.5 to 916.1)	179.8 (145.9 to 225.8)	625.0 (548.2 to 707.9)	5.0 (2.3 to 9.2)	10.3 (10.3 to 10.3)
Azerbaijan	2010	212.6 (190.8 to 236.6)	48.8 (39.4 to 60.8)	159.4 (140.4 to 181.1)	1.3 (0.6 to 2.4)	3.0 (3.0 to 3.0)	854.0 (766.5 to 950.6)	196.1 (158.1 to 244.2)	640.6 (564.2 to 727.7)	5.4 (2.4 to 9.7)	11.9 (11.9 to 11.9)
Azerbaijan	2011	217.4 (194.7 to 240.6)	50.8 (41.0 to 62.7)	160.8 (142.1 to 181.9)	1.3 (0.6 to 2.4)	4.6 (4.6 to 4.6)	873.6 (782.2 to 966.8)	204.0 (164.8 to 251.8)	645.9 (570.7 to 730.9)	5.3 (2.4 to 9.7)	18.3 (18.3 to 18.3)
Azerbaijan	2012	224.2 (201.1 to 247.6)	52.7 (42.5 to 65.4)	167.7 (147.8 to 188.4)	1.4 (0.6 to 2.5)	2.4 (2.4 to 2.4)	900.7 (808.0 to 994.6)	211.6 (170.9 to 262.8)	673.8 (594.0 to 757.1)	5.5 (2.4 to 9.9)	9.8 (9.8 to 9.8)
Azerbaijan	2013	240.4 (215.4 to 266.2)	54.8 (44.1 to 68.0)	181.0 (159.9 to 201.7)	1.5 (0.7 to 2.8)	3.0 (3.0 to 3.0)	965.6 (865.5 to 1069.5)	220.2 (177.1 to 273.4)	727.3 (642.3 to 810.4)	6.0 (2.7 to 11.2)	12.2 (12.2 to 12.2)
Azerbaijan	2014	258.3 (230.9 to 286.0)	57.0 (45.8 to 71.3)	197.6 (174.0 to 220.8)	1.7 (0.7 to 3.1)	1.9 (1.9 to 1.9)	1037.6 (927.7 to 1149.2)	229.2 (183.9 to 286.5)	793.8 (699.2 to 887.1)	6.8 (3.0 to 12.6)	7.8 (7.8 to 7.8)
Azerbaijan	2015	285.7 (253.2 to 317.9)	60.7 (48.6 to 76.1)	222.0 (193.6 to 251.3)	2.0 (0.9 to 3.7)	1.0 (1.0 to 1.0)	1147.8 (1017.2 to 1277.2)	243.9 (195.1 to 305.9)	891.8 (777.9 to 1009.5)	8.1 (3.6 to 14.8)	4.0 (4.0 to 4.0)
Azerbaijan	2016	296.7 (260.9 to 335.2)	61.2 (48.5 to 77.1)	232.5 (199.3 to 267.6)	2.2 (1.0 to 4.0)	0.9 (0.9 to 0.9)	1192.2 (1048.3 to 1346.8)	245.7 (194.8 to 309.6)	934.1 (800.9 to 1075.2)	8.7 (4.0 to 16.1)	3.7 (3.7 to 3.7)
Azerbaijan	2017	307.8 (269.7 to 350.5)	62.1 (49.0 to 77.9)	242.1 (208.1 to 279.4)	2.2 (1.0 to 4.0)	1.3 (1.3 to 1.3)	1236.4 (1083.6 to 1408.2)	249.6 (197.0 to 313.0)	972.7 (836.1 to 1122.7)	8.7 (4.0 to 16.0)	5.3 (5.3 to 5.3)
Azerbaijan	2018	321.1 (281.3 to 365.9)	63.9 (50.3 to 80.7)	253.7 (217.3 to 293.6)	2.2 (1.0 to 4.1)	1.2 (1.2 to 1.2)	1290.0 (1130.3 to 1470.2)	256.8 (201.9 to 324.0)	1019.5 (873.1 to 1179.7)	8.9 (4.1 to 16.4)	4.8 (4.7 to 4.9)
Azerbaijan	2019	335.2 (292.0 to 383.3)	65.9 (51.9 to 82.8)	265.9 (227.9 to 308.0)	2.3 (1.0 to 4.2)	1.2 (1.1 to 1.3)	1346.9 (1173.1 to 1539.8)	264.7 (208.6 to 332.7)	1068.2 (915.6 to 1237.6)	9.1 (4.1 to 16.8)	4.8 (4.5 to 5.2)
Azerbaijan	2020	349.7 (304.4 to 398.8)	67.8 (53.8 to 85.4)	278.3 (237.3 to 321.4)	2.3 (1.0 to 4.3)	1.2 (1.1 to 1.3)	1405.0 (1223.1 to 1602.4)	272.5 (216.0 to 343.2)	1118.3 (953.2 to 1291.1)	9.3 (4.2 to 17.1)	4.9 (4.5 to 5.4)
Azerbaijan	2021	362.5 (315.0 to 415.2)	70.1 (55.2 to 87.9)	288.8 (244.7 to 335.9)	2.4 (1.1 to 4.3)	1.3 (1.1 to 1.4)	1456.6 (1265.7 to 1668.3)	281.7 (221.9 to 353.2)	1160.3 (983.1 to 1349.5)	9.5 (4.3 to 17.5)	5.0 (4.5 to 5.6)
Azerbaijan	2022	368.7 (321.0 to 419.2)	72.1 (56.6 to 90.2)	292.9 (247.2 to 340.3)	2.4 (1.1 to 4.4)	1.3 (1.2 to 1.5)	1481.2 (1289.5 to 1684.3)	289.6 (227.2 to 362.2)	1176.7 (993.1 to 1367.2)	9.7 (4.4 to 17.7)	5.3 (4.6 to 5.9)
Azerbaijan	2023	370.5 (321.6 to 423.0)	73.9 (58.0 to 93.2)	292.8 (247.0 to 340.8)	2.4 (1.1 to 4.5)	1.4 (1.2 to 1.5)	1488.7 (1291.9 to 1699.6)	296.9 (233.1 to 374.5)	1176.5 (992.2 to 1369.3)	9.8 (4.4 to 17.9)	5.4 (4.7 to 6.2)
Azerbaijan	2024	371.9 (323.2 to 423.2)	75.7 (59.5 to 95.6)	292.3 (246.1 to 340.3)	2.5 (1.1 to 4.5)	1.4 (1.2 to 1.6)	1494.1 (1298.5 to 1700.3)	304.2 (238.9 to 384.3)	1174.3 (988.7 to 1367.2)	9.9 (4.5 to 18.2)	5.6 (4.8 to 6.4)
Azerbaijan	2025	382.3 (328.7 to 438.2)	76.4 (60.0 to 96.6)	302.0 (252.3 to 354.1)	2.5 (1.1 to 4.6)	1.4 (1.2 to 1.7)	1535.8 (1320.5 to 1760.7)	306.8 (241.0 to 387.9)	1213.3 (1013.8 to 1422.6)	10.0 (4.5 to 18.4)	5.8 (4.9 to 6.8)
Azerbaijan	2026	392.8 (332.9 to 454.7)	76.9 (60.3 to 97.2)	311.9 (255.6 to 367.9)	2.5 (1.1 to 4.6)	1.5 (1.2 to 1.7)	1578.0 (1337.6 to 1826.8)	309.0 (242.1 to 390.6)	1253.0 (1026.8 to 1478.2)	10.1 (4.5 to 18.5)	5.9 (5.0 to 7.0)
Azerbaijan	2027	404.0 (335.5 to 471.6)	77.6 (60.7 to 98.1)	322.4 (256.6 to 384.6)	2.5 (1.1 to 4.6)	1.5 (1.3 to 1.8)	1623.3 (1347.9 to 1894.8)	311.7 (243.8 to 394.1)	1295.3 (1030.8 to 1545.0)	10.1 (4.6 to 18.7)	6.1 (5.1 to 7.3)
Azerbaijan	2028	415.8 (334.8 to 489.1)	78.4 (61.3 to 99.3)	333.3 (256.5 to 402.8)	2.5 (1.2 to 4.7)	1.6 (1.3 to 1.9)	1670.7 (1345.1 to 1965.2)	315.0 (246.2 to 399.1)	1339.1 (1030.6 to 1618.2)	10.2 (4.6 to 18.9)	6.3 (5.3 to 7.7)
Azerbaijan	2029	428.3 (340.6 to 509.3)	79.4 (61.8 to 101.2)	344.6 (257.0 to 422.5)	2.6 (1.2 to 4.8)	1.6 (1.3 to 2.0)	1720.5 (1368.5 to 2046.3)	319.0 (248.4 to 406.7)	1384.6 (1032.5 to 1697.4)	10.4 (4.7 to 19.2)	6.5 (5.4 to 8.0)

		2018 US Dollars per capita					2018 PPP per capita				
Country	Year	Health spending	Government health spending	Out-of-pocket health spending	Prepaid private health spending	Development assistance for health	Health spending	Government health spending	Out-of-pocket health spending	Prepaid private health spending	Development assistance for health
Azerbaijan	2030	440.7 (336.8 to 528.9)	80.5 (62.5 to 103.2)	355.9 (259.1 to 442.8)	2.6 (1.2 to 4.9)	1.7 (1.4 to 2.1)	1770.7 (1353.2 to 2125.0)	323.4 (250.9 to 414.4)	1430.0 (1040.9 to 1779.0)	10.5 (4.8 to 19.5)	6.7 (5.5 to 8.3)
Azerbaijan	2031	453.6 (341.9 to 552.6)	81.8 (63.4 to 105.1)	367.5 (260.0 to 462.5)	2.7 (1.2 to 4.9)	1.7 (1.4 to 2.2)	1822.6 (1373.4 to 2220.3)	328.5 (254.7 to 422.3)	1476.5 (1044.6 to 1858.3)	10.7 (4.9 to 19.9)	7.0 (5.6 to 8.8)
Azerbaijan	2032	467.0 (344.5 to 574.9)	83.2 (64.6 to 106.8)	379.3 (262.2 to 482.6)	2.7 (1.2 to 5.1)	1.8 (1.4 to 2.3)	1876.2 (1383.9 to 2309.6)	334.2 (259.6 to 429.1)	1523.9 (1053.4 to 1939.0)	10.8 (5.0 to 20.3)	7.2 (5.7 to 9.1)
Azerbaijan	2033	481.2 (349.3 to 595.9)	85.0 (65.7 to 109.4)	391.6 (265.8 to 503.1)	2.8 (1.3 to 5.2)	1.8 (1.4 to 2.4)	1933.3 (1403.5 to 2394.2)	341.3 (264.0 to 439.4)	1573.5 (1068.0 to 2021.2)	11.1 (5.0 to 20.8)	7.4 (5.8 to 9.5)
Azerbaijan	2034	495.4 (351.9 to 620.5)	86.8 (66.8 to 111.8)	403.9 (268.3 to 525.3)	2.8 (1.3 to 5.3)	1.9 (1.5 to 2.5)	1990.2 (1413.9 to 2492.8)	348.6 (268.6 to 449.2)	1622.7 (1078.1 to 2110.6)	11.3 (5.1 to 21.2)	7.6 (5.9 to 9.9)
Azerbaijan	2035	509.0 (358.7 to 643.5)	88.5 (68.2 to 114.2)	415.6 (270.3 to 547.0)	2.9 (1.3 to 5.4)	2.0 (1.5 to 2.5)	2044.8 (1441.0 to 2585.3)	355.6 (273.9 to 458.6)	1669.9 (1085.8 to 2197.6)	11.5 (5.2 to 21.6)	7.8 (6.1 to 10.2)
Azerbaijan	2036	522.0 (362.4 to 664.3)	90.2 (69.4 to 116.6)	426.8 (272.3 to 566.2)	2.9 (1.3 to 5.5)	2.0 (1.6 to 2.7)	2097.1 (1456.0 to 2668.7)	362.4 (279.0 to 468.6)	1714.9 (1094.1 to 2274.9)	11.7 (5.4 to 22.1)	8.1 (6.3 to 10.9)
Azerbaijan	2037	535.1 (367.6 to 687.2)	92.0 (70.6 to 118.4)	438.0 (274.0 to 586.1)	3.0 (1.4 to 5.6)	2.1 (1.6 to 2.8)	2149.7 (1477.0 to 2761.0)	369.6 (283.8 to 475.7)	1759.7 (1100.9 to 2354.9)	12.0 (5.5 to 22.6)	8.3 (6.4 to 11.3)
Azerbaijan	2038	548.1 (371.7 to 707.7)	93.9 (72.0 to 120.9)	449.0 (277.3 to 606.3)	3.0 (1.4 to 5.7)	2.1 (1.6 to 3.0)	2202.1 (1493.2 to 2843.2)	377.3 (289.2 to 485.6)	1804.0 (1114.0 to 2436.0)	12.2 (5.6 to 23.1)	8.6 (6.5 to 11.9)
Azerbaijan	2039	560.4 (373.8 to 727.5)	95.7 (73.4 to 123.7)	459.4 (279.6 to 624.1)	3.1 (1.4 to 5.9)	2.2 (1.7 to 3.1)	2251.3 (1501.8 to 2922.7)	384.5 (294.8 to 496.9)	1845.5 (1123.4 to 2507.5)	12.5 (5.7 to 23.5)	8.8 (6.7 to 12.3)
Azerbaijan	2040	572.5 (380.3 to 750.4)	97.6 (75.0 to 126.6)	469.5 (282.1 to 642.3)	3.2 (1.4 to 6.0)	2.3 (1.7 to 3.1)	2300.2 (1527.9 to 3014.7)	392.1 (301.2 to 508.5)	1886.3 (1133.3 to 2580.3)	12.7 (5.8 to 24.0)	9.1 (6.9 to 12.6)
Azerbaijan	2041	583.8 (384.0 to 766.0)	99.4 (76.2 to 129.1)	478.9 (284.6 to 659.1)	3.2 (1.5 to 6.1)	2.3 (1.8 to 3.3)	2345.6 (1542.7 to 3077.4)	399.4 (306.2 to 518.5)	1923.8 (1143.6 to 2648.2)	12.9 (5.9 to 24.4)	9.4 (7.0 to 13.2)
Azerbaijan	2042	595.0 (390.6 to 787.2)	101.3 (77.7 to 131.5)	488.0 (288.1 to 676.0)	3.3 (1.5 to 6.2)	2.4 (1.8 to 3.5)	2390.3 (1569.3 to 3162.6)	407.1 (312.1 to 528.5)	1960.4 (1157.6 to 2715.9)	13.2 (6.1 to 24.8)	9.7 (7.2 to 14.1)
Azerbaijan	2043	605.2 (397.1 to 803.4)	103.1 (79.1 to 133.5)	496.2 (290.5 to 690.4)	3.3 (1.5 to 6.3)	2.5 (1.8 to 3.6)	2431.4 (1595.3 to 3227.9)	414.3 (317.7 to 536.5)	1993.7 (1167.3 to 2773.9)	13.4 (6.2 to 25.2)	10.0 (7.4 to 14.6)
Azerbaijan	2044	614.9 (401.6 to 821.3)	104.9 (80.3 to 136.0)	504.0 (293.0 to 704.7)	3.4 (1.6 to 6.4)	2.6 (1.9 to 3.8)	2470.4 (1613.3 to 3299.5)	421.4 (322.5 to 546.5)	2025.0 (1177.1 to 2831.1)	13.6 (6.3 to 25.6)	10.3 (7.6 to 15.1)
Azerbaijan	2045	624.1 (402.7 to 834.3)	106.7 (81.3 to 138.5)	511.3 (295.5 to 718.6)	3.5 (1.6 to 6.5)	2.7 (1.9 to 4.0)	2507.5 (1618.0 to 3351.7)	428.5 (326.8 to 556.3)	2054.4 (1187.0 to 2886.9)	13.9 (6.4 to 26.1)	10.7 (7.7 to 16.1)
Azerbaijan	2046	632.1 (405.3 to 851.7)	108.2 (82.2 to 140.6)	517.6 (297.3 to 730.2)	3.5 (1.6 to 6.6)	2.7 (2.0 to 4.2)	2539.4 (1628.2 to 3421.7)	434.7 (330.2 to 564.8)	2079.6 (1194.6 to 2933.6)	14.1 (6.5 to 26.5)	11.0 (7.9 to 17.0)
Azerbaijan	2047	639.1 (407.0 to 856.4)	109.6 (83.1 to 143.1)	523.1 (298.0 to 741.0)	3.6 (1.7 to 6.7)	2.8 (2.0 to 4.4)	2567.7 (1635.1 to 3440.5)	440.4 (333.8 to 575.0)	2101.7 (1197.2 to 2977.1)	14.3 (6.7 to 26.9)	11.4 (8.0 to 17.8)
Azerbaijan	2048	646.0 (408.3 to 871.3)	111.0 (84.3 to 144.7)	528.5 (298.4 to 751.7)	3.6 (1.7 to 6.8)	2.9 (2.1 to 4.8)	2595.4 (1640.4 to 3500.6)	445.9 (338.6 to 581.5)	2123.3 (1198.8 to 3019.9)	14.5 (6.7 to 27.3)	11.8 (8.3 to 19.2)
Azerbaijan	2049	653.1 (411.0 to 882.2)	112.4 (85.5 to 146.4)	534.0 (298.8 to 761.3)	3.6 (1.7 to 6.9)	3.0 (2.2 to 5.0)	2623.8 (1651.4 to 3544.2)	451.5 (343.5 to 588.3)	2145.5 (1200.6 to 3058.8)	14.6 (6.8 to 27.6)	12.2 (8.7 to 20.0)
Azerbaijan	2050	659.9 (413.9 to 894.6)	113.6 (86.6 to 148.2)	539.5 (299.5 to 773.2)	3.7 (1.7 to 7.0)	3.2 (2.2 to 5.2)	2651.4 (1662.9 to 3594.2)	456.5 (348.0 to 595.4)	2167.3 (1203.1 to 3106.6)	14.8 (6.9 to 27.9)	12.7 (8.8 to 21.1)

		2018 US Dollars per capita					2018 PPP per capita				
Country	Year	Health spending	Government health spending	Out-of-pocket health spending	Prepaid private health spending	Development assistance for health	Health spending	Government health spending	Out-of-pocket health spending	Prepaid private health spending	Development assistance for health
Bahrain	1995	948.4 (878.4 to 1024.6)	625.9 (576.2 to 682.3)	254.0 (220.8 to 291.6)	68.6 (48.4 to 94.7)	0.0 (0.0 to 0.0)	1918.2 (1776.6 to 2072.3)	1265.8 (1165.4 to 1380.0)	513.7 (446.5 to 589.8)	138.7 (98.0 to 191.5)	0.0 (0.0 to 0.0)
Bahrain	1996	955.5 (899.5 to 1019.4)	631.3 (589.1 to 676.3)	254.4 (225.5 to 285.2)	69.8 (50.7 to 93.7)	0.0 (0.0 to 0.0)	1932.6 (1819.3 to 2061.8)	1276.9 (1191.5 to 1367.9)	514.6 (456.0 to 576.8)	141.1 (102.5 to 189.5)	0.0 (0.0 to 0.0)
Bahrain	1997	973.4 (923.8 to 1028.8)	645.5 (606.7 to 685.8)	256.0 (229.8 to 284.6)	72.0 (54.3 to 93.4)	0.0 (0.0 to 0.0)	1968.8 (1868.5 to 2080.8)	1305.5 (1227.0 to 1387.0)	517.7 (464.7 to 575.6)	145.5 (109.8 to 188.8)	0.0 (0.0 to 0.0)
Bahrain	1998	968.5 (921.3 to 1020.9)	639.9 (603.9 to 681.3)	255.3 (230.1 to 282.9)	73.2 (57.2 to 92.8)	0.1 (0.1 to 0.1)	1958.8 (1863.3 to 2064.9)	1294.2 (1221.3 to 1377.9)	516.3 (465.4 to 572.2)	148.0 (115.6 to 187.6)	0.2 (0.2 to 0.2)
Bahrain	1999	967.2 (920.8 to 1015.3)	639.7 (606.8 to 676.8)	253.2 (229.3 to 279.8)	74.2 (58.6 to 92.2)	0.0 (0.0 to 0.0)	1956.2 (1862.3 to 2053.5)	1293.9 (1227.3 to 1368.9)	512.2 (463.8 to 565.8)	150.1 (118.6 to 186.5)	0.1 (0.1 to 0.1)
Bahrain	2000	978.6 (934.5 to 1022.8)	650.3 (617.6 to 687.5)	252.5 (228.9 to 277.9)	75.7 (60.3 to 93.6)	0.0 (0.0 to 0.0)	1979.3 (1890.1 to 2068.6)	1315.3 (1249.1 to 1390.5)	510.7 (462.9 to 562.0)	153.2 (122.0 to 189.3)	0.1 (0.1 to 0.1)
Bahrain	2001	967.5 (926.3 to 1008.8)	640.2 (606.6 to 673.3)	255.0 (232.6 to 279.7)	72.4 (58.0 to 89.8)	0.0 (0.0 to 0.0)	1956.8 (1873.4 to 2040.2)	1294.7 (1226.9 to 1361.7)	515.7 (470.4 to 565.7)	146.4 (117.2 to 181.5)	0.0 (0.0 to 0.0)
Bahrain	2002	940.0 (899.2 to 982.0)	615.0 (585.6 to 646.2)	260.5 (238.3 to 284.2)	64.5 (51.5 to 80.0)	0.0 (0.0 to 0.0)	1901.1 (1818.7 to 1986.0)	1243.9 (1184.3 to 1307.0)	526.8 (481.9 to 574.7)	130.5 (104.1 to 161.8)	0.0 (0.0 to 0.0)
Bahrain	2003	925.6 (884.8 to 968.4)	598.8 (567.6 to 630.3)	271.9 (249.7 to 295.9)	54.9 (43.6 to 68.9)	0.0 (0.0 to 0.0)	1872.1 (1789.6 to 1958.5)	1211.0 (1148.0 to 1274.9)	549.9 (505.1 to 598.5)	111.1 (88.1 to 139.3)	0.0 (0.0 to 0.0)
Bahrain	2004	861.0 (823.5 to 902.4)	562.9 (532.8 to 596.0)	255.2 (233.1 to 276.8)	42.9 (33.6 to 54.4)	0.0 (0.0 to 0.0)	1741.5 (1665.5 to 1825.0)	1138.5 (1077.6 to 1205.3)	516.2 (471.5 to 559.9)	86.7 (68.0 to 110.0)	0.0 (0.0 to 0.0)
Bahrain	2005	822.4 (782.6 to 862.2)	547.1 (515.9 to 579.6)	236.9 (216.3 to 258.3)	38.3 (29.7 to 48.7)	0.0 (0.0 to 0.0)	1663.2 (1582.8 to 1743.9)	1106.6 (1043.5 to 1172.3)	479.2 (437.4 to 522.4)	77.5 (60.0 to 98.6)	0.0 (0.0 to 0.0)
Bahrain	2006	781.3 (743.5 to 818.9)	521.9 (490.6 to 553.8)	223.3 (204.1 to 244.5)	36.1 (27.9 to 45.4)	0.0 (0.0 to 0.0)	1580.2 (1503.8 to 1656.2)	1055.5 (992.2 to 1120.0)	451.7 (412.8 to 494.6)	72.9 (56.4 to 91.8)	0.0 (0.0 to 0.0)
Bahrain	2007	795.2 (757.1 to 834.7)	524.7 (495.0 to 555.6)	229.3 (210.2 to 251.6)	41.2 (32.2 to 52.1)	0.0 (0.0 to 0.0)	1608.2 (1531.2 to 1688.1)	1061.3 (1001.2 to 1123.6)	463.7 (425.1 to 508.8)	83.3 (65.1 to 105.3)	0.0 (0.0 to 0.0)
Bahrain	2008	823.0 (783.9 to 862.7)	538.7 (508.9 to 573.1)	232.6 (213.7 to 253.2)	51.7 (41.1 to 64.1)	0.0 (0.0 to 0.0)	1664.6 (1585.6 to 1744.7)	1089.4 (1029.4 to 1159.2)	470.5 (432.2 to 512.0)	104.6 (83.2 to 129.6)	0.0 (0.0 to 0.0)
Bahrain	2009	827.5 (787.2 to 867.2)	528.2 (499.9 to 559.8)	238.1 (218.9 to 260.2)	61.2 (49.5 to 76.4)	0.0 (0.0 to 0.0)	1673.7 (1592.0 to 1753.9)	1068.3 (1011.0 to 1132.3)	481.6 (442.7 to 526.2)	123.8 (100.0 to 154.4)	0.0 (0.0 to 0.0)
Bahrain	2010	840.9 (801.8 to 880.1)	534.5 (505.3 to 565.8)	238.7 (217.9 to 259.8)	67.7 (55.7 to 82.9)	0.0 (0.0 to 0.0)	1700.7 (1621.7 to 1780.0)	1081.0 (1022.0 to 1144.4)	482.8 (440.7 to 525.4)	136.9 (112.7 to 167.7)	0.0 (0.0 to 0.0)
Bahrain	2011	860.0 (820.0 to 899.4)	549.8 (517.9 to 581.3)	238.7 (217.8 to 259.5)	71.5 (58.5 to 86.7)	0.0 (0.0 to 0.0)	1739.5 (1658.4 to 1819.0)	1112.0 (1047.4 to 1175.6)	482.7 (440.4 to 524.9)	144.7 (118.2 to 175.4)	0.0 (0.0 to 0.0)
Bahrain	2012	918.2 (876.4 to 956.9)	592.1 (559.7 to 624.3)	249.4 (229.9 to 270.6)	76.7 (64.2 to 91.8)	0.0 (0.0 to 0.0)	1857.1 (1772.5 to 1935.3)	1197.5 (1132.1 to 1262.7)	504.5 (465.1 to 547.3)	155.1 (129.9 to 185.7)	0.0 (0.0 to 0.0)
Bahrain	2013	989.4 (946.1 to 1031.0)	640.0 (607.0 to 675.5)	264.8 (244.8 to 287.1)	84.6 (70.9 to 100.9)	0.0 (0.0 to 0.0)	2001.1 (1913.4 to 2085.3)	1294.5 (1227.7 to 1366.2)	535.5 (495.1 to 580.6)	171.1 (143.4 to 204.1)	0.0 (0.0 to 0.0)
Bahrain	2014	1060.0 (1014.6 to 1106.1)	684.4 (651.5 to 719.8)	280.0 (258.1 to 303.3)	95.6 (80.1 to 113.8)	0.0 (0.0 to 0.0)	2143.8 (2052.1 to 2237.2)	1384.3 (1317.6 to 1455.8)	566.3 (522.0 to 613.4)	193.3 (162.1 to 230.1)	0.0 (0.0 to 0.0)
Bahrain	2015	1131.6 (1083.4 to 1181.1)	723.1 (687.0 to 762.5)	300.2 (276.0 to 325.1)	108.3 (90.3 to 130.7)	0.0 (0.0 to 0.0)	2288.8 (2191.3 to 2388.9)	1462.6 (1389.4 to 1542.3)	607.2 (558.1 to 657.5)	219.0 (182.6 to 264.3)	0.0 (0.0 to 0.0)

		2018 US Dollars per capita					2018 PPP per capita				
Country	Year	Health spending	Government health spending	Out-of-pocket health spending	Prepaid private health spending	Development assistance for health	Health spending	Government health spending	Out-of-pocket health spending	Prepaid private health spending	Development assistance for health
Bahrain	2016	1169.3 (1108.8 to 1232.9)	733.6 (687.0 to 783.9)	317.3 (286.6 to 350.1)	118.5 (96.2 to 146.8)	0.0 (0.0 to 0.0)	2365.0 (2242.6 to 2493.6)	1483.7 (1389.5 to 1585.5)	641.7 (579.6 to 708.2)	239.6 (194.6 to 297.0)	0.0 (0.0 to 0.0)
Bahrain	2017	1138.6 (1080.1 to 1198.7)	705.0 (660.6 to 755.1)	317.7 (286.2 to 352.4)	115.9 (94.1 to 143.6)	0.0 (0.0 to 0.0)	2302.9 (2184.5 to 2424.4)	1425.8 (1336.2 to 1527.2)	642.6 (578.9 to 712.8)	234.4 (190.3 to 290.5)	0.0 (0.0 to 0.0)
Bahrain	2018	1148.6 (1088.8 to 1209.5)	709.6 (665.4 to 759.1)	321.2 (289.1 to 355.5)	117.8 (95.6 to 145.9)	0.0 (0.0 to 0.0)	2323.0 (2202.1 to 2446.3)	1435.2 (1345.7 to 1535.3)	649.5 (584.6 to 718.9)	238.2 (193.3 to 295.1)	0.0 (0.0 to 0.0)
Bahrain	2019	1163.4 (1102.5 to 1227.7)	719.4 (674.3 to 773.5)	324.7 (292.4 to 360.3)	119.3 (96.6 to 147.5)	0.0 (0.0 to 0.0)	2352.9 (2229.8 to 2483.0)	1455.0 (1363.7 to 1564.4)	656.7 (591.4 to 728.7)	241.2 (195.4 to 298.4)	0.0 (0.0 to 0.0)
Bahrain	2020	1181.9 (1116.5 to 1247.0)	732.4 (681.1 to 787.8)	328.6 (294.5 to 365.1)	120.9 (98.1 to 149.5)	0.0 (0.0 to 0.0)	2390.4 (2258.0 to 2522.1)	1481.2 (1377.6 to 1593.4)	664.6 (595.6 to 738.4)	244.6 (198.4 to 302.4)	0.0 (0.0 to 0.0)
Bahrain	2021	1199.8 (1130.8 to 1272.1)	746.1 (689.3 to 806.1)	331.9 (297.5 to 369.8)	121.7 (98.9 to 150.3)	0.0 (0.0 to 0.0)	2426.7 (2287.0 to 2572.8)	1509.1 (1394.0 to 1630.3)	671.4 (601.7 to 748.0)	246.2 (200.1 to 303.9)	0.0 (0.0 to 0.0)
Bahrain	2022	1222.4 (1146.7 to 1302.7)	763.5 (699.2 to 835.6)	336.0 (301.0 to 376.7)	122.9 (99.8 to 151.5)	0.0 (0.0 to 0.0)	2472.4 (2319.3 to 2634.7)	1544.2 (1414.2 to 1690.1)	679.6 (608.8 to 761.9)	248.5 (201.8 to 306.4)	0.0 (0.0 to 0.0)
Bahrain	2023	1247.5 (1161.4 to 1336.8)	783.4 (709.5 to 862.8)	339.9 (302.3 to 379.9)	124.2 (100.8 to 152.7)	0.0 (0.0 to 0.0)	2523.0 (2349.0 to 2703.7)	1584.5 (1435.0 to 1745.0)	687.4 (611.5 to 768.3)	251.2 (203.9 to 308.9)	0.0 (0.0 to 0.0)
Bahrain	2024	1271.8 (1181.1 to 1368.6)	802.7 (724.5 to 892.2)	343.5 (305.7 to 383.8)	125.5 (101.7 to 154.6)	0.0 (0.0 to 0.0)	2572.2 (2388.8 to 2768.1)	1623.5 (1465.2 to 1804.5)	694.8 (618.3 to 776.2)	253.9 (205.6 to 312.8)	0.0 (0.0 to 0.0)
Bahrain	2025	1297.9 (1202.1 to 1403.3)	823.3 (737.7 to 916.4)	347.5 (309.2 to 390.4)	127.1 (102.8 to 156.7)	0.0 (0.0 to 0.0)	2625.0 (2431.2 to 2838.2)	1665.1 (1492.1 to 1853.5)	702.8 (625.4 to 789.5)	257.1 (207.9 to 317.0)	0.0 (0.0 to 0.0)
Bahrain	2026	1316.2 (1213.6 to 1426.7)	837.9 (746.8 to 938.4)	350.3 (309.3 to 394.3)	128.0 (103.7 to 157.6)	0.0 (0.0 to 0.0)	2662.1 (2454.4 to 2885.6)	1694.7 (1510.4 to 1897.9)	708.4 (625.6 to 797.5)	258.9 (209.7 to 318.7)	0.0 (0.0 to 0.0)
Bahrain	2027	1339.1 (1226.4 to 1459.6)	856.2 (755.0 to 965.4)	353.6 (309.1 to 401.4)	129.3 (105.0 to 159.5)	0.0 (0.0 to 0.0)	2708.4 (2480.4 to 2952.0)	1731.7 (1526.9 to 1952.6)	715.2 (625.2 to 811.8)	261.5 (212.3 to 322.6)	0.0 (0.0 to 0.0)
Bahrain	2028	1363.9 (1241.4 to 1496.7)	875.9 (765.9 to 992.6)	357.3 (309.5 to 409.7)	130.8 (106.1 to 161.1)	0.0 (0.0 to 0.0)	2758.5 (2510.8 to 3027.0)	1771.4 (1549.1 to 2007.5)	722.6 (626.1 to 828.6)	264.5 (214.6 to 325.9)	0.0 (0.0 to 0.0)
Bahrain	2029	1390.1 (1259.5 to 1529.8)	896.5 (779.4 to 1022.6)	361.3 (310.2 to 418.3)	132.4 (107.6 to 163.0)	0.0 (0.0 to 0.0)	2811.6 (2547.4 to 3094.1)	1813.2 (1576.3 to 2068.2)	730.7 (627.4 to 846.0)	267.7 (217.7 to 329.6)	0.0 (0.0 to 0.0)
Bahrain	2030	1417.1 (1272.8 to 1564.3)	917.7 (788.8 to 1053.4)	365.3 (311.0 to 427.0)	134.0 (109.1 to 165.0)	0.0 (0.0 to 0.0)	2866.1 (2574.3 to 3163.9)	1856.1 (1595.3 to 2130.5)	738.9 (628.9 to 863.6)	271.1 (220.6 to 333.6)	0.0 (0.0 to 0.0)
Bahrain	2031	1439.1 (1286.7 to 1599.4)	935.3 (799.7 to 1079.5)	368.6 (311.3 to 434.2)	135.2 (110.0 to 166.3)	0.0 (0.0 to 0.0)	2910.5 (2602.3 to 3234.9)	1891.6 (1617.3 to 2183.4)	745.4 (629.6 to 878.1)	273.4 (222.4 to 336.4)	0.0 (0.0 to 0.0)
Bahrain	2032	1463.0 (1294.3 to 1634.2)	954.4 (810.5 to 1110.6)	372.1 (311.2 to 443.8)	136.5 (110.9 to 167.8)	0.0 (0.0 to 0.0)	2958.9 (2617.8 to 3305.1)	1930.3 (1639.2 to 2246.2)	752.5 (629.3 to 897.6)	276.1 (224.2 to 339.4)	0.0 (0.0 to 0.0)
Bahrain	2033	1487.8 (1314.5 to 1669.1)	974.2 (816.8 to 1138.7)	375.7 (312.4 to 453.7)	137.9 (111.7 to 169.9)	0.0 (0.0 to 0.0)	3009.2 (2658.6 to 3375.9)	1970.4 (1651.9 to 2303.1)	759.9 (631.9 to 917.5)	278.9 (226.0 to 343.6)	0.0 (0.0 to 0.0)
Bahrain	2034	1512.4 (1323.5 to 1705.9)	993.6 (828.9 to 1171.2)	379.5 (313.1 to 464.3)	139.3 (112.6 to 171.9)	0.0 (0.0 to 0.0)	3058.8 (2676.9 to 3450.3)	2009.6 (1676.6 to 2368.7)	767.4 (633.3 to 939.1)	281.7 (227.7 to 347.7)	0.0 (0.0 to 0.0)
Bahrain	2035	1537.5 (1337.7 to 1743.5)	1013.5 (837.8 to 1198.2)	383.3 (313.5 to 474.6)	140.8 (113.6 to 173.6)	0.0 (0.0 to 0.0)	3109.6 (2705.6 to 3526.3)	2049.8 (1694.5 to 2423.4)	775.1 (634.0 to 959.9)	284.7 (229.8 to 351.0)	0.0 (0.0 to 0.0)
Bahrain	2036	1557.7 (1348.0 to 1783.0)	1029.7 (845.8 to 1223.6)	386.2 (312.5 to 483.8)	141.7 (114.3 to 175.0)	0.0 (0.0 to 0.0)	3150.5 (2726.3 to 3606.2)	2082.6 (1710.6 to 2474.7)	781.2 (632.0 to 978.5)	286.7 (231.3 to 354.0)	0.0 (0.0 to 0.0)

Country	Year	2018 US Dollars per capita					2018 PPP per capita				
		Health spending	Government health spending	Out-of-pocket health spending	Prepaid private health spending	Development assistance for health	Health spending	Government health spending	Out-of-pocket health spending	Prepaid private health spending	Development assistance for health
Bahrain	2037	1580.5 (1364.7 to 1809.5)	1047.7 (854.8 to 1257.8)	389.7 (312.1 to 493.3)	143.0 (115.2 to 176.6)	0.0 (0.0 to 0.0)	3196.5 (2760.2 to 3659.8)	2119.1 (1728.9 to 2544.0)	788.2 (631.2 to 997.7)	289.2 (233.0 to 357.1)	0.0 (0.0 to 0.0)
Bahrain	2038	1605.0 (1377.5 to 1853.5)	1067.3 (860.0 to 1289.0)	393.4 (312.1 to 502.6)	144.4 (116.3 to 178.4)	0.0 (0.0 to 0.0)	3246.2 (2785.9 to 3748.7)	2158.6 (1739.3 to 2607.0)	795.6 (631.2 to 1016.6)	292.0 (235.3 to 360.8)	0.0 (0.0 to 0.0)
Bahrain	2039	1628.5 (1391.2 to 1888.5)	1085.8 (868.0 to 1323.5)	397.0 (311.7 to 512.3)	145.7 (117.3 to 180.2)	0.0 (0.0 to 0.0)	3293.7 (2813.8 to 3819.5)	2196.1 (1755.5 to 2676.9)	802.9 (630.4 to 1036.2)	294.7 (237.3 to 364.5)	0.0 (0.0 to 0.0)
Bahrain	2040	1653.8 (1398.8 to 1925.0)	1105.9 (878.9 to 1355.5)	400.8 (311.5 to 521.5)	147.2 (118.5 to 182.5)	0.0 (0.0 to 0.0)	3344.9 (2829.1 to 3893.3)	2236.6 (1777.5 to 2741.5)	810.7 (630.0 to 1054.8)	297.6 (239.7 to 369.0)	0.0 (0.0 to 0.0)
Bahrain	2041	1673.7 (1409.1 to 1963.5)	1121.6 (883.2 to 1385.4)	403.9 (311.6 to 528.9)	148.2 (119.3 to 183.8)	0.0 (0.0 to 0.0)	3385.1 (2849.8 to 3971.1)	2268.4 (1786.4 to 2801.9)	816.9 (630.3 to 1069.7)	299.7 (241.3 to 371.7)	0.0 (0.0 to 0.0)
Bahrain	2042	1693.3 (1428.1 to 1992.8)	1136.8 (893.2 to 1404.2)	407.1 (311.4 to 537.5)	149.3 (120.0 to 185.7)	0.0 (0.0 to 0.0)	3424.7 (2888.4 to 4030.5)	2299.3 (1806.4 to 2840.1)	823.4 (629.8 to 1087.1)	302.0 (242.7 to 375.6)	0.0 (0.0 to 0.0)
Bahrain	2043	1713.0 (1438.7 to 2019.3)	1152.1 (899.3 to 1433.8)	410.4 (310.9 to 544.6)	150.5 (120.9 to 187.7)	0.0 (0.0 to 0.0)	3464.5 (2909.9 to 4084.1)	2330.1 (1818.9 to 2899.9)	830.0 (628.7 to 1101.5)	304.4 (244.5 to 379.5)	0.0 (0.0 to 0.0)
Bahrain	2044	1732.6 (1454.8 to 2050.8)	1167.0 (904.5 to 1465.6)	413.8 (311.5 to 551.8)	151.8 (121.6 to 189.2)	0.0 (0.0 to 0.0)	3504.1 (2942.4 to 4147.7)	2360.2 (1829.4 to 2964.2)	836.9 (630.1 to 1116.1)	307.1 (246.0 to 382.7)	0.0 (0.0 to 0.0)
Bahrain	2045	1753.3 (1456.1 to 2082.0)	1182.5 (909.7 to 1492.6)	417.5 (311.5 to 559.1)	153.3 (122.6 to 191.5)	0.0 (0.0 to 0.0)	3546.1 (2945.1 to 4210.9)	2391.6 (1839.9 to 3018.7)	844.3 (630.0 to 1130.7)	310.1 (247.9 to 387.4)	0.0 (0.0 to 0.0)
Bahrain	2046	1772.3 (1474.0 to 2113.3)	1196.9 (916.1 to 1504.1)	420.8 (311.0 to 566.0)	154.6 (123.3 to 193.7)	0.0 (0.0 to 0.0)	3584.5 (2981.2 to 4274.2)	2420.8 (1852.8 to 3042.1)	851.0 (628.9 to 1144.7)	312.7 (249.4 to 391.7)	0.0 (0.0 to 0.0)
Bahrain	2047	1790.7 (1481.3 to 2138.1)	1210.8 (926.1 to 1532.8)	424.0 (312.5 to 572.5)	155.8 (124.0 to 195.2)	0.0 (0.0 to 0.0)	3621.7 (2996.0 to 4324.4)	2448.9 (1873.1 to 3100.2)	857.6 (632.1 to 1158.0)	315.1 (250.8 to 394.9)	0.0 (0.0 to 0.0)
Bahrain	2048	1810.7 (1490.5 to 2175.0)	1225.9 (929.7 to 1554.8)	427.6 (312.6 to 579.2)	157.2 (124.9 to 197.3)	0.0 (0.0 to 0.0)	3662.2 (3014.5 to 4398.9)	2479.5 (1880.3 to 3144.6)	864.8 (632.2 to 1171.5)	317.9 (252.6 to 399.1)	0.0 (0.0 to 0.0)
Bahrain	2049	1830.3 (1506.2 to 2201.7)	1240.4 (937.2 to 1579.9)	431.2 (313.0 to 585.9)	158.6 (125.9 to 198.9)	0.0 (0.0 to 0.0)	3701.7 (3046.3 to 4453.1)	2508.8 (1895.5 to 3195.3)	872.1 (633.1 to 1185.0)	320.8 (254.7 to 402.3)	0.0 (0.0 to 0.0)
Bahrain	2050	1851.1 (1519.1 to 2230.9)	1255.8 (947.5 to 1598.5)	435.0 (313.2 to 593.0)	160.3 (126.7 to 200.6)	0.0 (0.0 to 0.0)	3743.8 (3072.3 to 4512.1)	2539.8 (1916.3 to 3232.9)	879.9 (633.4 to 1199.4)	324.1 (256.3 to 405.7)	0.0 (0.0 to 0.0)
Bangladesh	1995	16.9 (13.3 to 21.1)	4.3 (3.3 to 5.6)	11.1 (7.7 to 15.3)	0.6 (0.3 to 1.1)	1.0 (1.0 to 1.0)	45.4 (35.7 to 56.6)	11.6 (8.9 to 15.1)	29.7 (20.7 to 41.1)	1.5 (0.7 to 2.8)	2.6 (2.6 to 2.6)
Bangladesh	1996	16.1 (12.7 to 20.1)	4.1 (3.2 to 5.4)	10.5 (7.3 to 14.4)	0.6 (0.3 to 1.1)	0.9 (0.9 to 0.9)	43.1 (34.0 to 54.0)	11.1 (8.5 to 14.4)	28.1 (19.6 to 38.5)	1.6 (0.7 to 2.9)	2.4 (2.4 to 2.4)
Bangladesh	1997	15.0 (12.1 to 18.8)	4.0 (3.0 to 5.2)	9.4 (6.7 to 12.7)	0.6 (0.3 to 1.1)	1.1 (1.1 to 1.1)	40.3 (32.3 to 50.4)	10.6 (8.1 to 13.8)	25.2 (17.9 to 34.1)	1.5 (0.7 to 2.9)	3.0 (3.0 to 3.0)
Bangladesh	1998	15.8 (12.9 to 19.3)	3.8 (2.9 to 4.9)	9.1 (6.6 to 12.4)	0.6 (0.3 to 1.1)	2.3 (2.3 to 2.3)	42.2 (34.5 to 51.7)	10.2 (7.7 to 13.1)	24.5 (17.6 to 33.3)	1.5 (0.7 to 2.9)	6.0 (6.0 to 6.0)
Bangladesh	1999	15.9 (13.1 to 19.5)	3.8 (2.9 to 4.9)	9.3 (6.7 to 12.8)	0.6 (0.3 to 1.1)	2.2 (2.2 to 2.2)	42.7 (35.1 to 52.4)	10.1 (7.7 to 13.1)	25.0 (18.0 to 34.2)	1.5 (0.7 to 2.9)	6.0 (6.0 to 6.0)
Bangladesh	2000	15.9 (13.1 to 19.8)	4.0 (3.0 to 5.1)	9.7 (7.0 to 13.3)	0.5 (0.2 to 0.9)	1.8 (1.8 to 1.8)	42.7 (35.1 to 53.2)	10.6 (8.1 to 13.7)	26.0 (18.8 to 35.6)	1.3 (0.6 to 2.5)	4.8 (4.8 to 4.8)
Bangladesh	2001	16.2 (13.3 to 20.2)	3.9 (3.0 to 5.0)	10.2 (7.4 to 14.0)	0.5 (0.2 to 0.9)	1.6 (1.6 to 1.6)	43.5 (35.7 to 54.2)	10.6 (8.1 to 13.5)	27.2 (19.9 to 37.5)	1.3 (0.6 to 2.5)	4.3 (4.3 to 4.3)

Country	Year	2018 US Dollars per capita					2018 PPP per capita				
		Health spending	Government health spending	Out-of-pocket health spending	Prepaid private health spending	Development assistance for health	Health spending	Government health spending	Out-of-pocket health spending	Prepaid private health spending	Development assistance for health
Bangladesh	2002	16.8 (13.6 to 21.0)	4.2 (3.2 to 5.4)	10.9 (7.9 to 14.9)	0.5 (0.2 to 1.0)	1.2 (1.2 to 1.2)	45.0 (36.5 to 56.3)	11.3 (8.5 to 14.4)	29.2 (21.1 to 40.0)	1.4 (0.7 to 2.7)	3.1 (3.1 to 3.1)
Bangladesh	2003	17.7 (14.3 to 22.5)	4.4 (3.4 to 5.7)	11.6 (8.4 to 16.0)	0.6 (0.3 to 1.1)	1.2 (1.2 to 1.2)	47.5 (38.3 to 60.3)	11.9 (9.1 to 15.4)	31.1 (22.5 to 42.8)	1.5 (0.7 to 2.9)	3.1 (3.1 to 3.1)
Bangladesh	2004	18.8 (15.1 to 24.1)	4.6 (3.5 to 6.0)	12.4 (9.0 to 17.5)	0.6 (0.3 to 1.2)	1.2 (1.2 to 1.2)	50.5 (40.6 to 64.5)	12.3 (9.4 to 16.1)	33.2 (24.1 to 47.0)	1.6 (0.8 to 3.1)	3.3 (3.3 to 3.3)
Bangladesh	2005	20.5 (16.6 to 26.1)	4.8 (3.6 to 6.1)	13.4 (9.8 to 18.9)	0.7 (0.3 to 1.3)	1.6 (1.6 to 1.6)	54.9 (44.6 to 70.0)	12.8 (9.7 to 16.5)	36.0 (26.2 to 50.7)	1.8 (0.8 to 3.5)	4.3 (4.3 to 4.3)
Bangladesh	2006	21.9 (17.6 to 27.7)	5.1 (3.8 to 6.6)	14.7 (10.6 to 20.4)	0.8 (0.4 to 1.4)	1.4 (1.4 to 1.4)	58.8 (47.2 to 74.3)	13.6 (10.3 to 17.7)	39.3 (28.4 to 54.8)	2.0 (0.9 to 3.8)	3.8 (3.8 to 3.8)
Bangladesh	2007	23.0 (18.2 to 29.4)	5.4 (4.0 to 6.9)	15.9 (11.4 to 22.2)	0.8 (0.4 to 1.5)	0.9 (0.9 to 0.9)	61.6 (48.9 to 79.0)	14.4 (10.8 to 18.6)	42.6 (30.5 to 59.5)	2.1 (1.0 to 4.0)	2.5 (2.5 to 2.5)
Bangladesh	2008	24.6 (19.4 to 31.2)	5.5 (4.1 to 7.1)	16.8 (12.1 to 23.4)	0.8 (0.4 to 1.5)	1.5 (1.5 to 1.5)	66.0 (52.1 to 83.8)	14.7 (11.1 to 19.1)	45.2 (32.3 to 62.7)	2.1 (1.0 to 4.0)	4.1 (4.1 to 4.1)
Bangladesh	2009	26.2 (20.8 to 33.1)	5.7 (4.2 to 7.4)	18.0 (12.8 to 25.1)	0.8 (0.4 to 1.5)	1.7 (1.7 to 1.7)	70.2 (55.9 to 88.8)	15.2 (11.4 to 19.9)	48.2 (34.3 to 67.2)	2.2 (1.0 to 4.1)	4.6 (4.6 to 4.6)
Bangladesh	2010	28.1 (22.2 to 35.4)	6.0 (4.5 to 7.9)	19.4 (13.9 to 26.6)	0.8 (0.4 to 1.6)	1.8 (1.8 to 1.8)	75.4 (59.5 to 95.1)	16.2 (12.1 to 21.3)	52.1 (37.2 to 71.5)	2.3 (1.0 to 4.4)	4.8 (4.8 to 4.8)
Bangladesh	2011	29.9 (23.4 to 37.8)	6.4 (4.8 to 8.4)	21.0 (14.8 to 28.3)	0.9 (0.4 to 1.7)	1.5 (1.5 to 1.5)	80.1 (62.7 to 101.5)	17.2 (12.8 to 22.6)	56.3 (39.6 to 76.0)	2.4 (1.1 to 4.6)	4.1 (4.1 to 4.1)
Bangladesh	2012	32.0 (25.4 to 39.9)	6.6 (4.9 to 8.6)	22.2 (15.8 to 30.5)	0.9 (0.4 to 1.7)	2.3 (2.3 to 2.3)	85.8 (68.1 to 107.1)	17.8 (13.3 to 23.1)	59.5 (42.3 to 81.9)	2.5 (1.1 to 4.7)	6.1 (6.1 to 6.1)
Bangladesh	2013	33.8 (26.7 to 42.7)	6.7 (5.0 to 8.7)	23.2 (16.4 to 32.3)	0.9 (0.4 to 1.7)	3.0 (3.0 to 3.0)	90.7 (71.7 to 114.6)	18.0 (13.5 to 23.3)	62.2 (44.0 to 86.5)	2.5 (1.1 to 4.7)	8.0 (8.0 to 8.0)
Bangladesh	2014	34.3 (26.8 to 44.3)	6.7 (5.2 to 8.8)	24.3 (17.1 to 34.0)	0.9 (0.4 to 1.8)	2.3 (2.3 to 2.3)	92.1 (71.7 to 118.9)	18.1 (13.8 to 23.5)	65.2 (46.0 to 91.2)	2.5 (1.2 to 4.8)	6.2 (6.2 to 6.2)
Bangladesh	2015	35.7 (27.8 to 46.2)	6.8 (5.1 to 8.7)	25.6 (18.0 to 35.8)	1.0 (0.5 to 1.8)	2.3 (2.3 to 2.3)	95.7 (74.6 to 124.0)	18.3 (13.8 to 23.3)	68.6 (48.2 to 96.0)	2.6 (1.2 to 4.8)	6.2 (6.2 to 6.2)
Bangladesh	2016	37.1 (29.2 to 47.7)	7.0 (5.3 to 9.1)	26.6 (18.6 to 37.2)	1.0 (0.5 to 1.9)	2.4 (2.4 to 2.4)	99.5 (78.3 to 127.8)	18.9 (14.2 to 24.4)	71.4 (50.0 to 99.9)	2.7 (1.3 to 5.1)	6.6 (6.6 to 6.6)
Bangladesh	2017	39.3 (31.1 to 50.3)	7.5 (5.6 to 9.7)	28.1 (19.7 to 39.2)	1.1 (0.5 to 2.1)	2.7 (2.7 to 2.7)	105.5 (83.4 to 134.8)	20.2 (15.1 to 26.1)	75.3 (52.7 to 105.1)	2.9 (1.4 to 5.5)	7.2 (7.2 to 7.2)
Bangladesh	2018	40.6 (31.8 to 52.0)	7.9 (5.9 to 10.1)	29.2 (20.4 to 40.6)	1.1 (0.5 to 2.1)	2.4 (2.3 to 2.4)	108.8 (85.4 to 139.5)	21.1 (15.8 to 27.2)	78.4 (54.7 to 108.9)	3.0 (1.4 to 5.8)	6.3 (6.3 to 6.4)
Bangladesh	2019	42.1 (33.1 to 53.8)	8.2 (6.1 to 10.5)	30.4 (21.2 to 42.4)	1.2 (0.5 to 2.2)	2.3 (2.2 to 2.5)	112.9 (88.8 to 144.3)	21.9 (16.4 to 28.3)	81.6 (56.9 to 113.8)	3.1 (1.5 to 6.0)	6.3 (5.8 to 6.7)
Bangladesh	2020	43.7 (34.1 to 56.5)	8.5 (6.4 to 11.0)	31.7 (22.2 to 44.2)	1.2 (0.6 to 2.3)	2.3 (2.1 to 2.5)	117.3 (91.5 to 151.6)	22.8 (17.1 to 29.4)	85.0 (59.6 to 118.5)	3.3 (1.5 to 6.2)	6.2 (5.7 to 6.7)
Bangladesh	2021	45.5 (35.2 to 59.0)	8.8 (6.6 to 11.4)	33.1 (23.2 to 46.1)	1.3 (0.6 to 2.4)	2.3 (2.1 to 2.5)	121.9 (94.5 to 158.3)	23.7 (17.8 to 30.7)	88.6 (62.3 to 123.6)	3.4 (1.6 to 6.5)	6.2 (5.5 to 6.8)
Bangladesh	2022	47.3 (36.8 to 60.8)	9.2 (6.9 to 11.9)	34.5 (24.2 to 48.4)	1.3 (0.6 to 2.5)	2.3 (2.1 to 2.6)	126.9 (98.7 to 163.1)	24.7 (18.5 to 32.0)	92.4 (64.9 to 129.8)	3.5 (1.7 to 6.7)	6.2 (5.5 to 7.0)

		2018 US Dollars per capita					2018 PPP per capita				
Country	Year	Health spending	Government health spending	Out-of-pocket health spending	Prepaid private health spending	Development assistance for health	Health spending	Government health spending	Out-of-pocket health spending	Prepaid private health spending	Development assistance for health
Bangladesh	2023	49.2 (38.0 to 63.6)	9.6 (7.2 to 12.4)	35.9 (25.1 to 50.3)	1.4 (0.6 to 2.6)	2.4 (2.0 to 2.7)	132.0 (102.0 to 170.6)	25.7 (19.2 to 33.3)	96.3 (67.4 to 134.8)	3.7 (1.7 to 7.0)	6.3 (5.5 to 7.2)
Bangladesh	2024	51.2 (39.7 to 66.2)	10.0 (7.4 to 13.0)	37.4 (26.3 to 52.3)	1.4 (0.7 to 2.7)	2.4 (2.1 to 2.8)	137.4 (106.5 to 177.5)	26.8 (20.0 to 34.8)	100.3 (70.6 to 140.2)	3.8 (1.8 to 7.3)	6.4 (5.5 to 7.5)
Bangladesh	2025	53.2 (41.1 to 68.4)	10.4 (7.7 to 13.6)	38.9 (27.4 to 54.6)	1.5 (0.7 to 2.8)	2.4 (2.1 to 2.9)	142.8 (110.1 to 183.4)	27.9 (20.7 to 36.3)	104.4 (73.4 to 146.4)	4.0 (1.8 to 7.6)	6.6 (5.6 to 7.7)
Bangladesh	2026	55.4 (43.3 to 72.3)	10.9 (8.0 to 14.3)	40.5 (28.5 to 57.0)	1.5 (0.7 to 3.0)	2.5 (2.1 to 2.9)	148.6 (116.0 to 194.0)	29.1 (21.5 to 38.3)	108.7 (76.5 to 152.8)	4.1 (1.9 to 7.9)	6.7 (5.6 to 7.9)
Bangladesh	2027	57.7 (44.5 to 74.8)	11.3 (8.4 to 14.9)	42.2 (29.6 to 59.1)	1.6 (0.7 to 3.1)	2.5 (2.1 to 3.1)	154.6 (119.3 to 200.7)	30.4 (22.4 to 39.9)	113.2 (79.4 to 158.6)	4.3 (2.0 to 8.2)	6.8 (5.7 to 8.2)
Bangladesh	2028	60.0 (46.5 to 78.0)	11.8 (8.7 to 15.6)	43.9 (30.6 to 61.9)	1.7 (0.8 to 3.2)	2.6 (2.2 to 3.2)	160.8 (124.6 to 209.1)	31.8 (23.4 to 41.8)	117.7 (82.2 to 165.9)	4.4 (2.1 to 8.6)	7.0 (5.8 to 8.5)
Bangladesh	2029	62.3 (47.9 to 81.5)	12.4 (9.0 to 16.3)	45.6 (31.8 to 64.9)	1.7 (0.8 to 3.3)	2.7 (2.2 to 3.3)	167.2 (128.5 to 218.6)	33.2 (24.2 to 43.6)	122.3 (85.3 to 174.0)	4.6 (2.1 to 8.9)	7.1 (5.8 to 8.8)
Bangladesh	2030	64.7 (49.9 to 83.8)	12.9 (9.4 to 17.0)	47.3 (33.2 to 67.5)	1.8 (0.8 to 3.4)	2.7 (2.2 to 3.4)	173.6 (133.9 to 224.8)	34.6 (25.1 to 45.7)	127.0 (89.1 to 181.1)	4.8 (2.2 to 9.2)	7.3 (5.9 to 9.1)
Bangladesh	2031	67.2 (51.4 to 88.0)	13.4 (9.7 to 17.8)	49.1 (34.2 to 70.1)	1.8 (0.9 to 3.5)	2.8 (2.2 to 3.5)	180.1 (137.9 to 236.0)	36.0 (26.1 to 47.8)	131.7 (91.7 to 188.0)	4.9 (2.3 to 9.5)	7.5 (5.9 to 9.4)
Bangladesh	2032	69.6 (53.1 to 91.0)	14.0 (10.1 to 18.6)	50.9 (35.2 to 72.9)	1.9 (0.9 to 3.7)	2.8 (2.2 to 3.6)	186.7 (142.4 to 244.2)	37.4 (27.0 to 49.9)	136.5 (94.4 to 195.6)	5.1 (2.4 to 9.8)	7.6 (6.0 to 9.7)
Bangladesh	2033	72.1 (55.2 to 94.4)	14.5 (10.5 to 19.5)	52.7 (36.2 to 75.6)	2.0 (0.9 to 3.8)	2.9 (2.2 to 3.8)	193.4 (148.0 to 253.2)	38.9 (28.1 to 52.2)	141.4 (97.0 to 202.8)	5.3 (2.5 to 10.2)	7.8 (6.0 to 10.2)
Bangladesh	2034	74.6 (56.5 to 98.0)	15.1 (10.9 to 20.3)	54.5 (37.1 to 78.4)	2.0 (0.9 to 4.0)	3.0 (2.3 to 3.9)	200.2 (151.5 to 262.8)	40.4 (29.1 to 54.4)	146.2 (99.5 to 210.3)	5.5 (2.5 to 10.6)	8.0 (6.0 to 10.5)
Bangladesh	2035	77.2 (58.3 to 103.5)	15.6 (11.3 to 21.2)	56.4 (38.2 to 81.2)	2.1 (1.0 to 4.1)	3.1 (2.3 to 4.0)	207.1 (156.4 to 277.6)	42.0 (30.3 to 56.7)	151.2 (102.6 to 217.7)	5.7 (2.6 to 10.9)	8.3 (6.1 to 10.8)
Bangladesh	2036	79.8 (59.6 to 106.1)	16.2 (11.7 to 22.2)	58.2 (39.3 to 84.5)	2.2 (1.0 to 4.2)	3.2 (2.3 to 4.3)	214.1 (159.8 to 284.6)	43.6 (31.4 to 59.4)	156.1 (105.3 to 226.6)	5.9 (2.7 to 11.4)	8.5 (6.2 to 11.4)
Bangladesh	2037	82.5 (62.4 to 109.2)	16.9 (12.2 to 23.1)	60.1 (40.3 to 87.4)	2.3 (1.1 to 4.4)	3.2 (2.3 to 4.4)	221.2 (167.3 to 292.9)	45.2 (32.6 to 62.1)	161.1 (108.2 to 234.3)	6.1 (2.8 to 11.8)	8.7 (6.3 to 11.9)
Bangladesh	2038	85.1 (63.6 to 113.6)	17.5 (12.6 to 24.1)	62.0 (41.5 to 90.3)	2.4 (1.1 to 4.6)	3.3 (2.4 to 4.7)	228.3 (170.6 to 304.5)	46.9 (33.9 to 64.6)	166.2 (111.3 to 242.1)	6.3 (2.9 to 12.2)	8.9 (6.4 to 12.5)
Bangladesh	2039	87.8 (65.3 to 117.6)	18.2 (13.0 to 25.0)	63.8 (42.7 to 93.2)	2.4 (1.1 to 4.7)	3.4 (2.4 to 4.9)	235.6 (175.3 to 315.4)	48.7 (34.9 to 67.0)	171.2 (114.5 to 249.8)	6.5 (3.0 to 12.6)	9.2 (6.4 to 13.2)
Bangladesh	2040	90.6 (66.7 to 119.9)	18.8 (13.4 to 26.1)	65.7 (43.9 to 96.1)	2.5 (1.2 to 4.9)	3.5 (2.5 to 5.0)	242.9 (178.9 to 321.7)	50.5 (35.9 to 70.0)	176.2 (117.7 to 257.6)	6.8 (3.2 to 13.0)	9.4 (6.6 to 13.5)
Bangladesh	2041	93.3 (68.6 to 125.6)	19.5 (13.8 to 27.2)	67.6 (45.1 to 99.0)	2.6 (1.2 to 5.0)	3.6 (2.5 to 5.2)	250.2 (183.9 to 336.8)	52.4 (37.0 to 73.0)	181.2 (120.9 to 265.4)	7.0 (3.3 to 13.4)	9.7 (6.8 to 14.1)
Bangladesh	2042	96.1 (70.2 to 129.7)	20.2 (14.2 to 28.5)	69.4 (46.4 to 102.3)	2.7 (1.3 to 5.2)	3.7 (2.5 to 5.5)	257.7 (188.2 to 347.9)	54.3 (38.1 to 76.3)	186.2 (124.5 to 274.4)	7.2 (3.4 to 13.9)	10.0 (6.7 to 14.8)
Bangladesh	2043	98.9 (73.8 to 133.3)	21.0 (14.7 to 29.7)	71.3 (47.7 to 105.1)	2.8 (1.3 to 5.3)	3.8 (2.6 to 5.6)	265.2 (197.8 to 357.5)	56.3 (39.5 to 79.5)	191.1 (128.0 to 282.0)	7.5 (3.5 to 14.3)	10.3 (6.9 to 15.1)

		2018 US Dollars per capita					2018 PPP per capita				
Country	Year	Health spending	Government health spending	Out-of-pocket health spending	Prepaid private health spending	Development assistance for health	Health spending	Government health spending	Out-of-pocket health spending	Prepaid private health spending	Development assistance for health
Bangladesh	2044	101.7 (76.1 to 137.5)	21.7 (15.2 to 31.0)	73.1 (48.9 to 107.8)	2.9 (1.3 to 5.5)	4.0 (2.6 to 5.9)	272.8 (204.0 to 368.8)	58.3 (40.7 to 83.1)	196.1 (131.3 to 289.2)	7.7 (3.6 to 14.8)	10.7 (6.9 to 15.8)
Bangladesh	2045	104.5 (78.1 to 141.5)	22.5 (15.6 to 32.3)	74.9 (50.1 to 110.5)	3.0 (1.4 to 5.7)	4.1 (2.7 to 6.3)	280.3 (209.5 to 379.4)	60.4 (41.9 to 86.7)	200.9 (134.4 to 296.4)	8.0 (3.7 to 15.2)	11.0 (7.2 to 16.9)
Bangladesh	2046	107.3 (80.2 to 144.7)	23.3 (16.0 to 33.7)	76.7 (51.3 to 113.1)	3.1 (1.4 to 5.9)	4.2 (2.7 to 6.7)	287.7 (215.2 to 388.0)	62.5 (43.0 to 90.3)	205.6 (137.6 to 303.2)	8.2 (3.8 to 15.7)	11.4 (7.3 to 18.1)
Bangladesh	2047	110.1 (82.0 to 147.6)	24.1 (16.5 to 35.2)	78.4 (52.5 to 116.0)	3.2 (1.5 to 6.0)	4.4 (2.8 to 7.2)	295.2 (219.8 to 395.9)	64.7 (44.2 to 94.5)	210.2 (140.8 to 311.2)	8.5 (4.0 to 16.2)	11.8 (7.5 to 19.3)
Bangladesh	2048	112.9 (83.4 to 153.9)	25.0 (16.9 to 36.9)	80.1 (53.7 to 118.8)	3.3 (1.5 to 6.2)	4.6 (2.9 to 7.3)	302.9 (223.6 to 412.8)	66.9 (45.3 to 98.9)	214.9 (144.1 to 318.6)	8.8 (4.1 to 16.6)	12.3 (7.7 to 19.7)
Bangladesh	2049	115.8 (86.1 to 158.1)	25.8 (17.3 to 38.4)	81.9 (54.9 to 121.6)	3.4 (1.6 to 6.4)	4.8 (3.0 to 8.0)	310.6 (231.0 to 424.0)	69.2 (46.4 to 103.0)	219.6 (147.3 to 326.1)	9.0 (4.2 to 17.1)	12.8 (8.0 to 21.4)
Bangladesh	2050	118.7 (87.3 to 161.3)	26.6 (17.7 to 40.1)	83.7 (56.0 to 124.4)	3.5 (1.6 to 6.6)	5.0 (3.0 to 8.5)	318.5 (234.2 to 432.5)	71.5 (47.4 to 107.6)	224.4 (150.3 to 333.6)	9.3 (4.3 to 17.7)	13.4 (8.1 to 22.9)
Barbados	1995	807.6 (749.4 to 867.7)	472.0 (426.6 to 522.3)	269.8 (242.4 to 301.1)	64.8 (44.6 to 90.6)	1.1 (1.1 to 1.1)	845.9 (784.9 to 908.9)	494.3 (446.8 to 547.0)	282.6 (253.9 to 315.4)	67.9 (46.7 to 94.9)	1.1 (1.1 to 1.1)
Barbados	1996	847.9 (796.2 to 903.5)	496.1 (457.6 to 538.2)	283.1 (259.3 to 309.5)	67.8 (49.3 to 91.3)	0.8 (0.8 to 0.8)	888.1 (834.0 to 946.3)	519.7 (479.4 to 563.7)	296.6 (271.6 to 324.2)	71.0 (51.7 to 95.6)	0.9 (0.9 to 0.9)
Barbados	1997	872.6 (825.3 to 924.2)	501.7 (466.9 to 539.7)	300.1 (278.1 to 325.0)	69.9 (51.0 to 92.2)	0.8 (0.8 to 0.8)	913.9 (864.4 to 968.0)	525.5 (489.0 to 565.3)	314.3 (291.3 to 340.4)	73.3 (53.4 to 96.6)	0.9 (0.9 to 0.9)
Barbados	1998	870.8 (825.8 to 918.2)	492.5 (459.8 to 528.1)	307.6 (286.7 to 331.1)	69.9 (52.2 to 90.3)	0.8 (0.8 to 0.8)	912.1 (865.0 to 961.7)	515.9 (481.6 to 553.1)	322.2 (300.3 to 346.8)	73.2 (54.7 to 94.6)	0.8 (0.8 to 0.8)
Barbados	1999	870.4 (827.0 to 913.9)	483.9 (451.6 to 520.1)	315.8 (295.2 to 337.8)	70.0 (53.2 to 89.1)	0.8 (0.8 to 0.8)	911.7 (866.3 to 957.2)	506.8 (473.0 to 544.8)	330.7 (309.2 to 353.8)	73.3 (55.7 to 93.3)	0.8 (0.8 to 0.8)
Barbados	2000	877.8 (835.4 to 921.1)	481.0 (448.5 to 516.7)	326.0 (306.3 to 346.5)	70.9 (54.8 to 89.5)	0.0 (0.0 to 0.0)	919.4 (875.1 to 964.8)	503.8 (469.8 to 541.2)	341.4 (320.8 to 363.0)	74.2 (57.5 to 93.8)	0.0 (0.0 to 0.0)
Barbados	2001	874.5 (833.3 to 918.6)	477.8 (443.6 to 510.1)	326.5 (307.3 to 346.4)	68.8 (53.6 to 86.9)	1.4 (1.4 to 1.4)	915.9 (872.8 to 962.1)	500.5 (464.6 to 534.3)	342.0 (321.8 to 362.9)	72.0 (56.1 to 91.0)	1.5 (1.5 to 1.5)
Barbados	2002	915.1 (873.4 to 959.8)	508.6 (474.0 to 542.7)	338.3 (317.8 to 357.8)	68.1 (53.4 to 86.2)	0.0 (0.0 to 0.0)	958.5 (914.8 to 1005.3)	532.7 (496.5 to 568.4)	354.4 (332.9 to 374.8)	71.4 (55.9 to 90.3)	0.0 (0.0 to 0.0)
Barbados	2003	1011.7 (966.0 to 1055.9)	564.0 (526.6 to 600.8)	361.1 (340.4 to 382.5)	69.0 (53.1 to 87.5)	17.5 (17.5 to 17.5)	1059.7 (1011.8 to 1105.9)	590.8 (551.5 to 629.3)	378.2 (356.6 to 400.6)	72.3 (55.6 to 91.6)	18.4 (18.4 to 18.4)
Barbados	2004	1080.5 (1033.2 to 1130.1)	609.1 (570.9 to 649.9)	376.1 (353.9 to 399.1)	71.5 (55.7 to 88.9)	23.9 (23.9 to 23.9)	1131.8 (1082.2 to 1183.7)	638.0 (597.9 to 680.7)	394.0 (370.7 to 418.0)	74.9 (58.3 to 93.2)	25.0 (25.0 to 25.0)
Barbados	2005	1080.1 (1034.4 to 1127.9)	617.6 (580.2 to 656.0)	381.9 (360.5 to 403.9)	72.1 (56.4 to 89.7)	8.6 (8.6 to 8.6)	1131.4 (1083.5 to 1181.4)	646.9 (607.7 to 687.1)	400.0 (377.6 to 423.1)	75.5 (59.1 to 94.0)	9.0 (9.0 to 9.0)
Barbados	2006	1085.6 (1043.6 to 1133.1)	623.3 (589.5 to 662.1)	389.4 (367.5 to 410.7)	72.9 (56.9 to 91.9)	0.0 (0.0 to 0.0)	1137.0 (1093.1 to 1186.8)	652.8 (617.4 to 693.5)	407.9 (384.9 to 430.2)	76.3 (59.6 to 96.3)	0.0 (0.0 to 0.0)
Barbados	2007	1090.9 (1047.3 to 1136.9)	622.7 (589.9 to 661.0)	396.5 (375.8 to 417.0)	71.7 (57.7 to 89.7)	0.0 (0.0 to 0.0)	1142.7 (1097.0 to 1190.8)	652.2 (617.9 to 692.3)	415.3 (393.6 to 436.8)	75.1 (60.4 to 94.0)	0.0 (0.0 to 0.0)
Barbados	2008	1107.1 (1060.6 to 1150.7)	629.0 (595.1 to 664.5)	407.2 (387.1 to 428.6)	70.8 (56.2 to 87.1)	0.0 (0.0 to 0.0)	1159.6 (1110.9 to 1205.3)	658.9 (623.3 to 696.0)	426.5 (405.4 to 449.0)	74.1 (58.9 to 91.2)	0.0 (0.0 to 0.0)

Country	Year	2018 US Dollars per capita					2018 PPP per capita				
		Health spending	Government health spending	Out-of-pocket health spending	Prepaid private health spending	Development assistance for health	Health spending	Government health spending	Out-of-pocket health spending	Prepaid private health spending	Development assistance for health
Barbados	2009	1110.0 (1062.2 to 1156.0)	622.7 (588.2 to 658.9)	418.5 (398.9 to 440.9)	68.7 (55.1 to 85.0)	0.0 (0.0 to 0.0)	1162.6 (1112.6 to 1210.8)	652.3 (616.1 to 690.2)	438.4 (417.8 to 461.8)	72.0 (57.8 to 89.0)	0.0 (0.0 to 0.0)
Barbados	2010	1135.6 (1091.6 to 1182.0)	622.2 (585.1 to 659.4)	443.0 (422.3 to 465.9)	70.4 (56.9 to 85.9)	0.0 (0.0 to 0.0)	1189.5 (1143.4 to 1238.1)	651.8 (612.8 to 690.6)	464.0 (442.4 to 488.0)	73.7 (59.6 to 89.9)	0.0 (0.0 to 0.0)
Barbados	2011	1185.1 (1142.9 to 1231.5)	628.7 (594.3 to 664.7)	482.8 (460.8 to 504.6)	73.6 (59.6 to 89.3)	0.0 (0.0 to 0.0)	1241.4 (1197.1 to 1289.9)	658.5 (622.4 to 696.2)	505.8 (482.6 to 528.5)	77.1 (62.5 to 93.6)	0.0 (0.0 to 0.0)
Barbados	2012	1251.3 (1208.1 to 1298.6)	655.5 (620.9 to 694.4)	516.7 (494.8 to 539.7)	79.0 (65.2 to 95.6)	0.0 (0.0 to 0.0)	1310.7 (1265.4 to 1360.2)	686.6 (650.3 to 727.3)	541.2 (518.2 to 565.3)	82.8 (68.2 to 100.1)	0.0 (0.0 to 0.0)
Barbados	2013	1254.2 (1208.8 to 1301.6)	634.4 (598.0 to 671.8)	536.9 (513.3 to 561.3)	82.9 (67.7 to 99.1)	0.0 (0.0 to 0.0)	1313.7 (1266.2 to 1363.3)	664.5 (626.4 to 703.6)	562.4 (537.7 to 588.0)	86.8 (71.0 to 103.8)	0.0 (0.0 to 0.0)
Barbados	2014	1216.4 (1168.0 to 1265.0)	591.5 (554.1 to 631.7)	541.0 (518.0 to 566.5)	83.9 (68.0 to 101.8)	0.0 (0.0 to 0.0)	1274.1 (1223.4 to 1325.0)	619.6 (580.4 to 661.6)	566.6 (542.6 to 593.3)	87.8 (71.3 to 106.7)	0.0 (0.0 to 0.0)
Barbados	2015	1195.2 (1142.2 to 1248.7)	567.3 (528.7 to 610.3)	542.6 (518.2 to 569.8)	85.2 (67.0 to 106.4)	0.0 (0.0 to 0.0)	1251.8 (1196.4 to 1307.9)	594.2 (553.8 to 639.3)	568.4 (542.8 to 596.8)	89.3 (70.2 to 111.4)	0.0 (0.0 to 0.0)
Barbados	2016	1187.6 (1123.5 to 1256.8)	556.8 (508.9 to 607.2)	543.8 (510.9 to 578.8)	86.9 (63.9 to 112.8)	0.0 (0.0 to 0.0)	1243.9 (1176.8 to 1316.4)	583.3 (533.0 to 636.0)	569.6 (535.1 to 606.2)	91.0 (67.0 to 118.1)	0.0 (0.0 to 0.0)
Barbados	2017	1201.2 (1135.9 to 1270.5)	560.0 (511.9 to 611.9)	553.1 (519.3 to 589.3)	88.1 (64.8 to 114.4)	0.0 (0.0 to 0.0)	1258.2 (1189.7 to 1330.7)	586.6 (536.2 to 640.9)	579.4 (543.9 to 617.2)	92.3 (67.9 to 119.8)	0.0 (0.0 to 0.0)
Barbados	2018	1210.6 (1144.8 to 1280.1)	560.8 (512.0 to 612.0)	560.7 (524.6 to 597.4)	89.1 (65.6 to 115.7)	0.0 (0.0 to 0.0)	1268.1 (1199.1 to 1340.8)	587.4 (536.3 to 641.0)	587.3 (549.5 to 625.8)	93.3 (68.7 to 121.1)	0.0 (0.0 to 0.0)
Barbados	2019	1222.3 (1155.7 to 1294.0)	563.9 (514.6 to 620.6)	568.4 (532.5 to 607.3)	90.0 (66.2 to 117.0)	0.0 (0.0 to 0.0)	1280.3 (1210.5 to 1355.4)	590.6 (539.0 to 650.1)	595.4 (557.8 to 636.1)	94.3 (69.3 to 122.6)	0.0 (0.0 to 0.0)
Barbados	2020	1232.7 (1162.5 to 1309.1)	565.5 (515.2 to 625.1)	576.3 (539.1 to 615.7)	90.8 (67.0 to 118.1)	0.0 (0.0 to 0.0)	1291.1 (1217.6 to 1371.2)	592.4 (539.6 to 654.7)	603.6 (564.7 to 644.9)	95.2 (70.1 to 123.7)	0.0 (0.0 to 0.0)
Barbados	2021	1244.4 (1172.4 to 1324.1)	568.4 (517.2 to 628.2)	584.5 (545.1 to 625.7)	91.6 (67.5 to 119.3)	0.0 (0.0 to 0.0)	1303.5 (1228.0 to 1386.9)	595.3 (541.7 to 658.0)	612.2 (571.0 to 655.4)	95.9 (70.7 to 124.9)	0.0 (0.0 to 0.0)
Barbados	2022	1257.5 (1184.2 to 1340.9)	572.3 (519.6 to 635.1)	592.8 (551.9 to 636.0)	92.4 (68.1 to 120.5)	0.0 (0.0 to 0.0)	1317.1 (1240.4 to 1404.5)	599.4 (544.2 to 665.2)	621.0 (578.1 to 666.2)	96.8 (71.3 to 126.2)	0.0 (0.0 to 0.0)
Barbados	2023	1271.8 (1196.6 to 1356.1)	577.4 (524.3 to 640.9)	601.2 (559.2 to 647.4)	93.2 (68.7 to 121.5)	0.0 (0.0 to 0.0)	1332.1 (1253.4 to 1420.5)	604.8 (549.2 to 671.2)	629.8 (585.7 to 678.1)	97.6 (72.0 to 127.3)	0.0 (0.0 to 0.0)
Barbados	2024	1284.7 (1205.8 to 1371.3)	581.4 (527.5 to 647.0)	609.3 (563.1 to 660.1)	94.0 (69.3 to 122.6)	0.0 (0.0 to 0.0)	1345.6 (1263.0 to 1436.3)	609.0 (552.5 to 677.7)	638.2 (589.8 to 691.4)	98.4 (72.6 to 128.4)	0.0 (0.0 to 0.0)
Barbados	2025	1297.2 (1218.6 to 1383.9)	585.2 (531.1 to 650.0)	617.4 (570.1 to 670.2)	94.7 (69.8 to 123.6)	0.0 (0.0 to 0.0)	1358.8 (1276.4 to 1449.5)	613.0 (556.2 to 680.8)	646.6 (597.1 to 701.9)	99.1 (73.1 to 129.4)	0.0 (0.0 to 0.0)
Barbados	2026	1311.6 (1230.1 to 1399.0)	590.2 (535.6 to 655.3)	625.8 (572.5 to 682.5)	95.5 (70.2 to 124.7)	0.0 (0.0 to 0.0)	1373.8 (1288.4 to 1465.4)	618.2 (561.0 to 686.4)	655.5 (599.7 to 714.9)	100.1 (73.6 to 130.6)	0.0 (0.0 to 0.0)
Barbados	2027	1325.7 (1243.6 to 1421.4)	595.0 (539.8 to 661.9)	634.3 (574.5 to 698.0)	96.4 (70.7 to 125.6)	0.0 (0.0 to 0.0)	1388.5 (1302.5 to 1488.8)	623.2 (565.4 to 693.2)	664.4 (601.7 to 731.1)	100.9 (74.1 to 131.6)	0.0 (0.0 to 0.0)
Barbados	2028	1339.6 (1255.3 to 1440.6)	599.7 (543.8 to 667.0)	642.7 (576.7 to 714.3)	97.2 (71.5 to 126.6)	0.0 (0.0 to 0.0)	1403.1 (1314.9 to 1509.0)	628.1 (569.6 to 698.7)	673.2 (604.1 to 748.2)	101.8 (74.9 to 132.6)	0.0 (0.0 to 0.0)
Barbados	2029	1353.8 (1266.8 to 1461.2)	604.5 (548.0 to 672.9)	651.2 (578.0 to 730.9)	98.1 (72.3 to 127.6)	0.0 (0.0 to 0.0)	1418.0 (1326.9 to 1530.5)	633.2 (574.0 to 704.8)	682.1 (605.5 to 765.6)	102.7 (75.8 to 133.6)	0.0 (0.0 to 0.0)

		2018 US Dollars per capita					2018 PPP per capita				
Country	Year	Health spending	Government health spending	Out-of-pocket health spending	Prepaid private health spending	Development assistance for health	Health spending	Government health spending	Out-of-pocket health spending	Prepaid private health spending	Development assistance for health
Barbados	2030	1368.5 (1274.9 to 1486.7)	609.8 (551.9 to 678.6)	659.7 (580.0 to 746.0)	99.0 (72.8 to 128.6)	0.0 (0.0 to 0.0)	1433.4 (1335.3 to 1557.2)	638.7 (578.0 to 710.8)	691.0 (607.5 to 781.4)	103.7 (76.3 to 134.7)	0.0 (0.0 to 0.0)
Barbados	2031	1384.9 (1285.5 to 1507.3)	616.2 (558.0 to 685.2)	668.6 (582.8 to 762.4)	100.1 (73.6 to 130.0)	0.0 (0.0 to 0.0)	1450.6 (1346.5 to 1578.7)	645.4 (584.4 to 717.7)	700.3 (610.4 to 798.5)	104.9 (77.1 to 136.1)	0.0 (0.0 to 0.0)
Barbados	2032	1401.8 (1297.2 to 1532.9)	622.9 (563.7 to 692.2)	677.6 (585.3 to 780.9)	101.3 (74.5 to 131.4)	0.0 (0.0 to 0.0)	1468.3 (1358.8 to 1605.6)	652.5 (590.5 to 725.0)	709.7 (613.0 to 817.9)	106.1 (78.1 to 137.6)	0.0 (0.0 to 0.0)
Barbados	2033	1418.7 (1307.8 to 1558.4)	629.7 (569.6 to 700.8)	686.4 (587.3 to 798.7)	102.5 (75.6 to 132.7)	0.0 (0.0 to 0.0)	1485.9 (1369.8 to 1632.3)	659.6 (596.6 to 734.0)	719.0 (615.1 to 836.6)	107.4 (79.1 to 139.0)	0.0 (0.0 to 0.0)
Barbados	2034	1435.9 (1317.3 to 1583.3)	636.7 (575.1 to 708.0)	695.4 (590.1 to 817.0)	103.8 (76.5 to 134.8)	0.0 (0.0 to 0.0)	1504.0 (1379.8 to 1658.4)	666.9 (602.4 to 741.5)	728.4 (618.1 to 855.7)	108.7 (80.1 to 141.2)	0.0 (0.0 to 0.0)
Barbados	2035	1452.6 (1330.1 to 1608.8)	643.4 (579.4 to 716.4)	704.2 (594.3 to 834.7)	105.0 (77.4 to 136.4)	0.0 (0.0 to 0.0)	1521.5 (1393.2 to 1685.1)	674.0 (606.8 to 750.4)	737.6 (622.5 to 874.3)	109.9 (81.0 to 142.8)	0.0 (0.0 to 0.0)
Barbados	2036	1470.4 (1346.2 to 1630.8)	650.9 (584.6 to 726.9)	713.3 (598.4 to 851.0)	106.3 (78.4 to 137.7)	0.0 (0.0 to 0.0)	1540.1 (1410.0 to 1708.2)	681.7 (612.3 to 761.3)	747.1 (626.7 to 891.4)	111.3 (82.1 to 144.2)	0.0 (0.0 to 0.0)
Barbados	2037	1487.5 (1358.3 to 1661.7)	657.9 (589.4 to 735.9)	722.1 (600.7 to 866.8)	107.5 (79.3 to 139.5)	0.0 (0.0 to 0.0)	1558.1 (1422.7 to 1740.5)	689.1 (617.4 to 770.8)	756.4 (629.2 to 907.9)	112.6 (83.1 to 146.1)	0.0 (0.0 to 0.0)
Barbados	2038	1505.3 (1366.7 to 1689.1)	665.3 (594.6 to 744.7)	731.1 (605.0 to 884.1)	108.8 (80.4 to 141.7)	0.0 (0.0 to 0.0)	1576.7 (1431.5 to 1769.2)	696.9 (622.8 to 780.0)	765.8 (633.7 to 926.0)	114.0 (84.2 to 148.4)	0.0 (0.0 to 0.0)
Barbados	2039	1522.9 (1375.3 to 1712.5)	672.7 (600.0 to 753.4)	740.0 (608.8 to 899.0)	110.2 (81.1 to 143.0)	0.0 (0.0 to 0.0)	1595.1 (1440.5 to 1793.7)	704.6 (628.4 to 789.2)	775.1 (637.7 to 941.7)	115.4 (85.0 to 149.8)	0.0 (0.0 to 0.0)
Barbados	2040	1541.3 (1391.4 to 1737.8)	680.7 (607.0 to 764.1)	749.0 (613.1 to 913.5)	111.6 (82.2 to 144.9)	0.0 (0.0 to 0.0)	1614.4 (1457.4 to 1820.2)	713.0 (635.8 to 800.3)	784.5 (642.2 to 956.9)	116.9 (86.1 to 151.8)	0.0 (0.0 to 0.0)
Barbados	2041	1561.4 (1401.0 to 1755.6)	689.9 (612.5 to 776.1)	758.4 (618.1 to 928.2)	113.2 (83.6 to 147.3)	0.0 (0.0 to 0.0)	1635.5 (1467.5 to 1838.8)	722.6 (641.6 to 812.9)	794.3 (647.4 to 972.2)	118.5 (87.6 to 154.3)	0.0 (0.0 to 0.0)
Barbados	2042	1582.1 (1420.8 to 1789.3)	699.5 (621.0 to 787.0)	767.8 (624.1 to 944.2)	114.9 (85.0 to 149.5)	0.0 (0.0 to 0.0)	1657.2 (1488.2 to 1874.2)	732.7 (650.4 to 824.3)	804.2 (653.7 to 988.9)	120.3 (89.1 to 156.5)	0.0 (0.0 to 0.0)
Barbados	2043	1602.7 (1433.5 to 1813.7)	709.1 (627.7 to 798.2)	777.0 (629.8 to 959.7)	116.6 (86.3 to 151.8)	0.0 (0.0 to 0.0)	1678.7 (1501.5 to 1899.7)	742.7 (657.5 to 836.0)	813.9 (659.7 to 1005.2)	122.1 (90.4 to 159.0)	0.0 (0.0 to 0.0)
Barbados	2044	1623.8 (1448.3 to 1841.4)	719.0 (634.6 to 809.8)	786.4 (635.5 to 974.9)	118.3 (87.5 to 154.6)	0.0 (0.0 to 0.0)	1700.8 (1517.0 to 1928.7)	753.1 (664.8 to 848.2)	823.7 (665.6 to 1021.2)	123.9 (91.7 to 161.9)	0.0 (0.0 to 0.0)
Barbados	2045	1644.1 (1464.4 to 1866.7)	728.5 (641.1 to 822.7)	795.6 (641.6 to 988.8)	120.0 (88.6 to 157.3)	0.0 (0.0 to 0.0)	1722.0 (1533.9 to 1955.2)	763.0 (671.5 to 861.8)	833.3 (672.0 to 1035.7)	125.7 (92.8 to 164.8)	0.0 (0.0 to 0.0)
Barbados	2046	1665.3 (1479.6 to 1888.0)	738.5 (648.8 to 837.5)	805.0 (647.9 to 1000.5)	121.8 (89.9 to 160.1)	0.0 (0.0 to 0.0)	1744.3 (1549.8 to 1977.6)	773.5 (679.6 to 877.2)	843.2 (678.7 to 1048.0)	127.6 (94.2 to 167.6)	0.0 (0.0 to 0.0)
Barbados	2047	1686.7 (1502.1 to 1919.1)	748.7 (656.9 to 849.7)	814.4 (654.9 to 1014.4)	123.6 (90.9 to 163.0)	0.0 (0.0 to 0.0)	1766.7 (1573.3 to 2010.1)	784.2 (688.1 to 890.0)	853.0 (686.0 to 1062.5)	129.5 (95.2 to 170.7)	0.0 (0.0 to 0.0)
Barbados	2048	1707.9 (1520.1 to 1936.0)	758.7 (664.8 to 862.8)	823.8 (660.6 to 1028.3)	125.4 (91.9 to 165.5)	0.0 (0.0 to 0.0)	1788.9 (1592.2 to 2027.8)	794.6 (696.3 to 903.7)	862.9 (691.9 to 1077.1)	131.4 (96.3 to 173.3)	0.0 (0.0 to 0.0)
Barbados	2049	1728.5 (1536.6 to 1971.4)	768.2 (671.7 to 878.0)	833.1 (668.8 to 1042.1)	127.2 (93.1 to 167.9)	0.0 (0.0 to 0.0)	1810.5 (1609.5 to 2064.9)	804.7 (703.5 to 919.7)	872.6 (700.5 to 1091.5)	133.2 (97.5 to 175.8)	0.0 (0.0 to 0.0)
Barbados	2050	1750.7 (1559.6 to 1998.8)	778.7 (679.3 to 891.0)	842.8 (675.4 to 1056.7)	129.1 (94.5 to 170.5)	0.0 (0.0 to 0.0)	1833.8 (1633.5 to 2093.6)	815.7 (711.5 to 933.2)	882.8 (707.4 to 1106.8)	135.3 (99.0 to 178.6)	0.0 (0.0 to 0.0)

Country	Year	2018 US Dollars per capita					2018 PPP per capita				
		Health spending	Government health spending	Out-of-pocket health spending	Prepaid private health spending	Development assistance for health	Health spending	Government health spending	Out-of-pocket health spending	Prepaid private health spending	Development assistance for health
Belarus	1995	105.8 (90.7 to 122.6)	79.7 (66.9 to 94.4)	19.2 (13.9 to 25.3)	6.9 (3.1 to 13.9)	0.0 (0.0 to 0.0)	349.5 (299.6 to 405.1)	263.3 (221.2 to 311.8)	63.3 (46.0 to 83.6)	22.9 (10.4 to 45.9)	0.0 (0.0 to 0.0)
Belarus	1996	107.8 (93.5 to 124.0)	83.4 (71.2 to 97.3)	17.2 (12.6 to 22.5)	7.2 (3.4 to 14.3)	0.0 (0.0 to 0.0)	356.2 (309.0 to 409.9)	275.6 (235.4 to 321.4)	56.7 (41.8 to 74.5)	23.9 (11.1 to 47.3)	0.0 (0.0 to 0.0)
Belarus	1997	117.0 (101.4 to 133.9)	92.7 (79.1 to 107.8)	16.3 (12.2 to 21.4)	7.9 (3.8 to 15.4)	0.0 (0.0 to 0.0)	386.5 (335.1 to 442.4)	306.3 (261.3 to 356.3)	53.9 (40.3 to 70.9)	26.2 (12.7 to 50.9)	0.0 (0.0 to 0.0)
Belarus	1998	123.4 (108.4 to 140.4)	96.8 (83.3 to 111.1)	18.4 (13.9 to 23.9)	8.3 (4.0 to 16.2)	0.0 (0.0 to 0.0)	407.9 (358.1 to 463.9)	319.7 (275.4 to 367.2)	60.7 (46.0 to 79.0)	27.4 (13.1 to 53.6)	0.0 (0.0 to 0.0)
Belarus	1999	128.1 (113.2 to 144.2)	99.5 (86.0 to 113.7)	20.4 (15.6 to 26.0)	8.1 (3.9 to 15.7)	0.0 (0.0 to 0.0)	423.2 (374.0 to 476.5)	328.9 (284.2 to 375.7)	67.3 (51.7 to 85.9)	26.9 (12.9 to 51.8)	0.1 (0.1 to 0.1)
Belarus	2000	136.2 (121.4 to 153.1)	104.7 (91.0 to 119.3)	23.5 (18.1 to 30.0)	7.9 (3.8 to 14.6)	0.0 (0.0 to 0.0)	450.0 (401.3 to 506.0)	346.1 (300.6 to 394.1)	77.7 (59.8 to 99.1)	26.1 (12.6 to 48.4)	0.1 (0.1 to 0.1)
Belarus	2001	148.0 (132.3 to 166.1)	111.7 (97.5 to 126.5)	28.6 (22.4 to 35.9)	7.6 (3.7 to 14.5)	0.1 (0.1 to 0.1)	489.1 (437.2 to 549.0)	369.3 (322.2 to 417.9)	94.5 (74.0 to 118.8)	25.1 (12.3 to 47.9)	0.2 (0.2 to 0.2)
Belarus	2002	159.0 (142.2 to 177.3)	118.7 (104.0 to 133.7)	33.0 (26.0 to 41.1)	7.3 (3.5 to 13.9)	0.1 (0.1 to 0.1)	525.3 (469.8 to 585.9)	392.2 (343.5 to 442.0)	108.9 (86.0 to 136.0)	24.0 (11.6 to 45.9)	0.2 (0.2 to 0.2)
Belarus	2003	174.2 (156.9 to 193.5)	130.1 (115.1 to 145.5)	36.5 (29.1 to 44.9)	7.5 (3.7 to 14.3)	0.1 (0.1 to 0.1)	575.5 (518.5 to 639.3)	429.9 (380.2 to 480.9)	120.5 (96.1 to 148.3)	24.8 (12.1 to 47.1)	0.3 (0.3 to 0.3)
Belarus	2004	195.4 (176.7 to 216.0)	145.4 (129.3 to 162.2)	41.5 (33.7 to 51.2)	8.3 (4.1 to 15.6)	0.2 (0.2 to 0.2)	645.8 (583.9 to 713.7)	480.3 (427.2 to 536.1)	137.2 (111.4 to 169.2)	27.6 (13.6 to 51.6)	0.7 (0.7 to 0.7)
Belarus	2005	217.9 (197.2 to 240.5)	158.9 (141.5 to 176.5)	49.1 (40.4 to 60.4)	9.1 (4.5 to 16.7)	0.8 (0.8 to 0.8)	720.2 (651.8 to 794.8)	525.1 (467.7 to 583.1)	162.3 (133.4 to 199.4)	29.9 (14.9 to 55.3)	2.7 (2.7 to 2.7)
Belarus	2006	236.0 (214.1 to 259.0)	168.5 (150.4 to 187.3)	57.1 (46.9 to 70.4)	9.7 (4.8 to 17.6)	0.6 (0.6 to 0.6)	779.8 (707.3 to 855.9)	556.8 (496.9 to 619.0)	188.8 (155.0 to 232.6)	32.0 (15.8 to 58.2)	2.1 (2.1 to 2.1)
Belarus	2007	254.2 (231.0 to 278.4)	176.7 (157.7 to 195.1)	66.3 (55.0 to 81.1)	10.2 (5.1 to 18.5)	1.0 (1.0 to 1.0)	840.1 (763.4 to 920.0)	584.0 (521.1 to 644.5)	218.9 (181.8 to 268.1)	33.9 (16.8 to 61.3)	3.3 (3.3 to 3.3)
Belarus	2008	273.5 (248.3 to 298.7)	184.9 (165.0 to 205.1)	76.3 (63.8 to 92.8)	10.7 (5.3 to 18.6)	1.6 (1.6 to 1.6)	903.8 (820.6 to 987.0)	611.0 (545.3 to 677.8)	252.2 (210.7 to 306.8)	35.2 (17.6 to 61.6)	5.4 (5.4 to 5.4)
Belarus	2009	273.4 (248.6 to 298.4)	180.2 (160.5 to 200.3)	81.5 (68.4 to 97.5)	10.0 (5.1 to 17.9)	1.7 (1.7 to 1.7)	903.5 (821.5 to 986.0)	595.5 (530.5 to 661.7)	269.3 (226.1 to 322.0)	33.0 (16.7 to 59.0)	5.7 (5.7 to 5.7)
Belarus	2010	289.1 (263.2 to 314.3)	189.6 (169.5 to 211.0)	88.3 (74.3 to 104.2)	9.2 (4.7 to 16.7)	2.0 (2.0 to 2.0)	955.3 (869.6 to 1038.6)	626.6 (560.1 to 697.2)	291.7 (245.4 to 344.4)	30.3 (15.5 to 55.1)	6.7 (6.7 to 6.7)
Belarus	2011	299.8 (272.3 to 326.4)	194.4 (173.2 to 216.4)	94.7 (79.5 to 111.7)	9.1 (4.6 to 16.8)	1.6 (1.6 to 1.6)	990.6 (899.9 to 1078.6)	642.3 (572.5 to 715.1)	312.9 (262.6 to 369.0)	30.0 (15.4 to 55.6)	5.4 (5.4 to 5.4)
Belarus	2012	313.4 (284.5 to 341.7)	201.4 (179.8 to 223.5)	100.9 (84.7 to 118.8)	9.5 (4.8 to 17.6)	1.6 (1.6 to 1.6)	1035.7 (940.2 to 1129.0)	665.5 (594.0 to 738.7)	333.5 (279.8 to 392.7)	31.4 (15.8 to 58.3)	5.3 (5.3 to 5.3)
Belarus	2013	325.4 (296.4 to 355.3)	204.9 (183.6 to 227.5)	109.4 (92.0 to 129.1)	9.7 (4.7 to 18.0)	1.5 (1.5 to 1.5)	1075.4 (979.4 to 1173.9)	677.0 (606.6 to 751.8)	361.4 (304.1 to 426.7)	32.0 (15.5 to 59.6)	5.0 (5.0 to 5.0)
Belarus	2014	336.3 (306.5 to 368.7)	209.8 (187.3 to 234.7)	116.0 (96.8 to 138.0)	9.2 (4.5 to 17.0)	1.3 (1.3 to 1.3)	1111.3 (1012.7 to 1218.4)	693.3 (618.9 to 775.5)	383.2 (320.0 to 456.1)	30.3 (14.8 to 56.2)	4.5 (4.5 to 4.5)
Belarus	2015	345.3 (312.5 to 381.0)	212.2 (189.1 to 238.2)	122.9 (101.2 to 148.7)	9.5 (4.5 to 17.3)	0.7 (0.7 to 0.7)	1141.1 (1032.6 to 1259.1)	701.1 (624.9 to 787.0)	406.2 (334.3 to 491.2)	31.3 (15.0 to 57.3)	2.5 (2.5 to 2.5)

		2018 US Dollars per capita					2018 PPP per capita				
Country	Year	Health spending	Government health spending	Out-of-pocket health spending	Prepaid private health spending	Development assistance for health	Health spending	Government health spending	Out-of-pocket health spending	Prepaid private health spending	Development assistance for health
Belarus	2016	354.0 (318.1 to 395.8)	216.1 (190.2 to 246.6)	127.2 (101.8 to 156.7)	9.6 (4.6 to 17.7)	1.1 (1.1 to 1.1)	1169.9 (1051.2 to 1308.0)	714.2 (628.4 to 814.9)	420.4 (336.4 to 517.8)	31.8 (15.1 to 58.4)	3.5 (3.5 to 3.5)
Belarus	2017	356.8 (319.6 to 401.4)	214.0 (188.2 to 244.4)	131.9 (105.4 to 162.6)	9.7 (4.6 to 17.9)	1.2 (1.2 to 1.2)	1179.0 (1056.1 to 1326.5)	707.1 (622.0 to 807.8)	435.7 (348.4 to 537.3)	32.2 (15.3 to 59.2)	4.0 (4.0 to 4.0)
Belarus	2018	367.5 (326.9 to 411.9)	218.0 (191.1 to 249.1)	138.4 (110.3 to 170.6)	10.0 (4.7 to 18.3)	1.1 (1.1 to 1.1)	1214.5 (1080.2 to 1361.1)	720.5 (631.4 to 823.1)	457.4 (364.5 to 563.8)	32.9 (15.6 to 60.6)	3.6 (3.6 to 3.7)
Belarus	2019	377.8 (336.8 to 424.2)	221.3 (193.5 to 252.7)	145.2 (115.4 to 179.8)	10.1 (4.8 to 18.6)	1.1 (1.1 to 1.2)	1248.3 (1112.8 to 1401.9)	731.4 (639.5 to 834.9)	479.8 (381.5 to 594.2)	33.4 (15.9 to 61.6)	3.8 (3.5 to 4.0)
Belarus	2020	388.1 (345.7 to 438.3)	224.5 (196.0 to 256.0)	152.2 (121.2 to 188.9)	10.3 (4.9 to 18.9)	1.2 (1.1 to 1.3)	1282.5 (1142.2 to 1448.3)	741.9 (647.8 to 845.9)	502.8 (400.6 to 624.4)	33.9 (16.1 to 62.5)	3.9 (3.6 to 4.3)
Belarus	2021	397.7 (351.9 to 449.9)	226.8 (198.0 to 258.4)	159.3 (127.2 to 197.0)	10.4 (4.9 to 19.1)	1.3 (1.1 to 1.4)	1314.3 (1162.8 to 1486.7)	749.3 (654.3 to 853.8)	526.5 (420.4 to 651.1)	34.3 (16.2 to 63.1)	4.2 (3.7 to 4.6)
Belarus	2022	407.1 (360.7 to 458.8)	228.7 (199.7 to 260.4)	166.7 (132.5 to 206.1)	10.5 (5.0 to 19.3)	1.3 (1.1 to 1.5)	1345.3 (1191.8 to 1516.1)	755.6 (659.9 to 860.6)	550.7 (438.0 to 681.2)	34.6 (16.4 to 63.8)	4.3 (3.8 to 4.9)
Belarus	2023	416.7 (368.8 to 471.7)	230.5 (201.5 to 262.9)	174.3 (137.9 to 215.9)	10.6 (5.0 to 19.5)	1.4 (1.2 to 1.5)	1377.0 (1218.7 to 1558.8)	761.7 (665.9 to 868.6)	575.9 (455.5 to 713.5)	35.0 (16.5 to 64.5)	4.5 (3.9 to 5.1)
Belarus	2024	426.7 (377.6 to 481.3)	232.4 (203.7 to 264.7)	182.2 (144.1 to 226.0)	10.7 (5.0 to 19.7)	1.4 (1.2 to 1.6)	1409.9 (1247.7 to 1590.3)	768.0 (673.1 to 874.8)	601.9 (476.3 to 746.7)	35.4 (16.7 to 65.2)	4.6 (4.0 to 5.4)
Belarus	2025	437.5 (387.8 to 496.4)	234.7 (205.7 to 267.8)	190.5 (151.1 to 237.2)	10.8 (5.1 to 20.0)	1.5 (1.2 to 1.7)	1445.6 (1281.4 to 1640.2)	775.5 (679.7 to 884.9)	629.5 (499.4 to 783.8)	35.8 (16.9 to 66.0)	4.8 (4.1 to 5.7)
Belarus	2026	448.0 (394.5 to 507.3)	236.3 (206.9 to 269.9)	199.2 (158.1 to 247.6)	10.9 (5.2 to 20.2)	1.5 (1.3 to 1.8)	1480.3 (1303.5 to 1676.5)	780.8 (683.7 to 891.8)	658.4 (522.5 to 818.2)	36.1 (17.0 to 66.8)	5.0 (4.2 to 5.9)
Belarus	2027	459.4 (403.3 to 520.7)	238.3 (208.4 to 272.1)	208.5 (165.6 to 260.3)	11.1 (5.2 to 20.5)	1.6 (1.3 to 1.9)	1517.9 (1332.5 to 1720.7)	787.3 (688.7 to 899.1)	688.9 (547.1 to 860.2)	36.6 (17.2 to 67.7)	5.2 (4.3 to 6.2)
Belarus	2028	472.2 (413.8 to 536.9)	241.3 (211.0 to 275.4)	218.0 (172.7 to 274.3)	11.2 (5.3 to 20.8)	1.6 (1.4 to 2.0)	1560.2 (1367.2 to 1774.0)	797.4 (697.3 to 910.1)	720.3 (570.8 to 906.5)	37.1 (17.5 to 68.8)	5.4 (4.5 to 6.5)
Belarus	2029	485.9 (426.4 to 551.4)	245.0 (214.4 to 279.5)	227.8 (179.7 to 288.1)	11.4 (5.4 to 21.2)	1.7 (1.4 to 2.1)	1605.7 (1409.1 to 1822.1)	809.7 (708.5 to 923.4)	752.6 (593.9 to 952.1)	37.8 (17.8 to 70.0)	5.6 (4.6 to 6.9)
Belarus	2030	500.3 (437.9 to 570.0)	249.2 (217.7 to 284.3)	237.7 (186.4 to 301.1)	11.6 (5.5 to 21.6)	1.8 (1.4 to 2.2)	1653.2 (1447.2 to 1883.7)	823.4 (719.3 to 939.4)	785.6 (616.0 to 994.9)	38.5 (18.1 to 71.3)	5.8 (4.7 to 7.1)
Belarus	2031	514.9 (448.5 to 589.4)	253.5 (221.4 to 289.0)	247.7 (192.6 to 316.0)	11.9 (5.6 to 22.0)	1.8 (1.5 to 2.3)	1701.3 (1482.2 to 1947.7)	837.6 (731.7 to 954.9)	818.6 (636.3 to 1044.1)	39.2 (18.4 to 72.7)	6.0 (4.8 to 7.6)
Belarus	2032	529.9 (462.2 to 612.6)	258.1 (225.5 to 294.5)	257.8 (199.3 to 330.0)	12.1 (5.7 to 22.4)	1.9 (1.5 to 2.4)	1751.0 (1527.2 to 2024.4)	852.9 (745.1 to 973.2)	851.9 (658.4 to 1090.5)	40.0 (18.8 to 74.1)	6.2 (5.0 to 7.8)
Belarus	2033	545.7 (474.1 to 631.6)	263.4 (229.6 to 300.9)	268.1 (205.7 to 346.9)	12.4 (5.8 to 22.9)	1.9 (1.6 to 2.5)	1803.4 (1566.7 to 2087.3)	870.2 (758.8 to 994.4)	885.9 (679.8 to 1146.4)	40.8 (19.2 to 75.7)	6.4 (5.1 to 8.3)
Belarus	2034	561.3 (484.8 to 647.0)	268.5 (233.4 to 307.0)	278.1 (212.6 to 362.3)	12.6 (5.9 to 23.4)	2.0 (1.6 to 2.6)	1854.7 (1602.0 to 2138.0)	887.2 (771.3 to 1014.5)	919.1 (702.5 to 1197.1)	41.7 (19.5 to 77.2)	6.7 (5.2 to 8.7)
Belarus	2035	577.1 (497.5 to 666.3)	273.9 (237.6 to 313.7)	288.2 (218.9 to 375.9)	12.9 (6.0 to 23.9)	2.1 (1.6 to 2.8)	1906.9 (1644.1 to 2201.8)	905.1 (785.1 to 1036.6)	952.3 (723.3 to 1242.0)	42.6 (19.9 to 78.9)	6.9 (5.4 to 9.1)
Belarus	2036	592.8 (509.4 to 690.0)	279.4 (242.2 to 320.2)	298.1 (226.6 to 388.8)	13.2 (6.1 to 24.4)	2.2 (1.7 to 2.9)	1958.9 (1683.2 to 2280.2)	923.2 (800.4 to 1058.0)	985.0 (748.7 to 1284.8)	43.5 (20.3 to 80.5)	7.2 (5.6 to 9.6)

		2018 US Dollars per capita					2018 PPP per capita				
Country	Year	Health spending	Government health spending	Out-of-pocket health spending	Prepaid private health spending	Development assistance for health	Health spending	Government health spending	Out-of-pocket health spending	Prepaid private health spending	Development assistance for health
Belarus	2037	608.3 (523.9 to 709.3)	284.9 (246.4 to 326.5)	307.7 (233.4 to 404.0)	13.5 (6.3 to 24.9)	2.2 (1.7 to 3.0)	2010.0 (1731.3 to 2343.7)	941.4 (814.1 to 1078.8)	1016.8 (771.3 to 1335.1)	44.5 (20.7 to 82.2)	7.4 (5.7 to 10.0)
Belarus	2038	623.5 (531.0 to 731.1)	290.4 (250.5 to 333.7)	317.0 (239.9 to 416.3)	13.7 (6.4 to 25.4)	2.3 (1.8 to 3.2)	2060.3 (1754.8 to 2416.0)	959.6 (827.7 to 1102.9)	1047.6 (792.7 to 1375.6)	45.4 (21.1 to 83.9)	7.7 (5.8 to 10.6)
Belarus	2039	638.3 (544.6 to 751.3)	295.9 (254.9 to 340.4)	326.0 (246.2 to 428.0)	14.0 (6.5 to 25.9)	2.4 (1.8 to 3.3)	2109.1 (1799.6 to 2482.6)	977.7 (842.4 to 1124.9)	1077.2 (813.6 to 1414.4)	46.3 (21.6 to 85.7)	8.0 (6.1 to 10.9)
Belarus	2040	653.2 (555.4 to 769.3)	301.6 (259.3 to 347.6)	334.7 (250.9 to 440.7)	14.3 (6.7 to 26.5)	2.5 (1.9 to 3.5)	2158.3 (1835.5 to 2542.2)	996.7 (856.9 to 1148.6)	1106.1 (829.0 to 1456.4)	47.3 (22.0 to 87.7)	8.2 (6.3 to 11.5)
Belarus	2041	667.4 (566.1 to 783.6)	307.2 (263.9 to 354.9)	342.9 (255.8 to 453.4)	14.6 (6.8 to 27.1)	2.6 (2.0 to 3.6)	2205.3 (1870.6 to 2589.4)	1015.3 (872.0 to 1172.8)	1133.2 (845.2 to 1498.1)	48.2 (22.4 to 89.7)	8.5 (6.5 to 12.0)
Belarus	2042	681.6 (574.4 to 801.7)	313.1 (268.2 to 362.2)	350.9 (260.6 to 465.2)	14.9 (6.9 to 27.7)	2.7 (2.0 to 3.9)	2252.3 (1898.1 to 2649.3)	1034.8 (886.2 to 1196.9)	1159.4 (861.0 to 1537.4)	49.3 (22.8 to 91.5)	8.9 (6.6 to 12.7)
Belarus	2043	695.3 (589.4 to 819.0)	319.0 (272.5 to 369.4)	358.3 (265.8 to 475.5)	15.2 (7.0 to 28.2)	2.8 (2.1 to 4.0)	2297.7 (1947.7 to 2706.3)	1054.3 (900.5 to 1220.7)	1184.0 (878.4 to 1571.1)	50.3 (23.2 to 93.1)	9.2 (6.8 to 13.4)
Belarus	2044	709.1 (598.7 to 831.6)	325.3 (277.3 to 377.2)	365.4 (270.7 to 484.2)	15.5 (7.2 to 28.7)	2.9 (2.1 to 4.2)	2343.1 (1978.4 to 2747.9)	1074.9 (916.3 to 1246.4)	1207.4 (894.4 to 1600.0)	51.3 (23.7 to 94.9)	9.5 (7.0 to 14.0)
Belarus	2045	721.4 (609.8 to 851.5)	330.9 (281.8 to 385.1)	371.6 (274.9 to 493.3)	15.8 (7.3 to 29.3)	3.0 (2.2 to 4.5)	2383.7 (2015.0 to 2813.8)	1093.6 (931.3 to 1272.7)	1227.9 (908.4 to 1630.2)	52.3 (24.0 to 96.7)	9.9 (7.2 to 15.0)
Belarus	2046	732.3 (616.0 to 870.5)	336.1 (285.0 to 392.2)	376.9 (279.2 to 501.3)	16.1 (7.4 to 29.9)	3.1 (2.3 to 4.8)	2419.8 (2035.7 to 2876.6)	1110.8 (941.8 to 1295.9)	1245.6 (922.5 to 1656.4)	53.2 (24.4 to 98.7)	10.3 (7.5 to 15.8)
Belarus	2047	742.8 (627.0 to 882.5)	341.5 (288.9 to 399.4)	381.7 (282.2 to 508.1)	16.4 (7.5 to 30.5)	3.2 (2.3 to 5.2)	2454.7 (2071.8 to 2916.2)	1128.4 (954.8 to 1319.7)	1261.5 (932.4 to 1679.1)	54.1 (24.8 to 100.7)	10.7 (7.7 to 17.1)
Belarus	2048	753.0 (636.9 to 897.8)	346.5 (291.9 to 406.4)	386.4 (285.8 to 515.1)	16.7 (7.6 to 31.0)	3.4 (2.4 to 5.3)	2488.2 (2104.7 to 2966.8)	1145.1 (964.7 to 1342.9)	1276.9 (944.4 to 1702.1)	55.0 (25.2 to 102.6)	11.1 (8.0 to 17.6)
Belarus	2049	763.1 (649.4 to 902.9)	351.6 (295.1 to 413.6)	391.1 (289.4 to 521.9)	16.9 (7.7 to 31.6)	3.5 (2.5 to 5.8)	2521.6 (2146.0 to 2983.7)	1161.8 (975.0 to 1366.6)	1292.3 (956.4 to 1724.5)	55.9 (25.6 to 104.5)	11.6 (8.3 to 19.0)
Belarus	2050	772.9 (656.8 to 918.2)	356.4 (298.6 to 420.0)	395.7 (292.7 to 528.6)	17.2 (7.9 to 32.2)	3.6 (2.6 to 6.0)	2554.2 (2170.4 to 3034.1)	1177.9 (986.7 to 1388.0)	1307.5 (967.3 to 1746.7)	56.8 (25.9 to 106.4)	12.0 (8.4 to 19.9)
Belgium	1995	2919.1 (2813.8 to 3025.4)	2276.8 (2182.3 to 2370.0)	538.3 (497.7 to 584.2)	104.0 (81.6 to 130.9)	0.0 (0.0 to 0.0)	2938.8 (2832.8 to 3045.8)	2292.1 (2197.0 to 2386.0)	541.9 (501.0 to 588.1)	104.7 (82.1 to 131.8)	0.0 (0.0 to 0.0)
Belgium	1996	3003.5 (2923.5 to 3084.2)	2330.8 (2263.6 to 2402.8)	559.5 (526.2 to 596.2)	113.1 (92.7 to 136.3)	0.0 (0.0 to 0.0)	3023.8 (2943.2 to 3105.1)	2346.6 (2278.9 to 2419.0)	563.3 (529.8 to 600.3)	113.9 (93.3 to 137.2)	0.0 (0.0 to 0.0)
Belgium	1997	3109.0 (3035.0 to 3187.0)	2384.0 (2319.1 to 2449.4)	596.2 (565.0 to 631.3)	128.8 (109.2 to 151.8)	0.0 (0.0 to 0.0)	3130.0 (3055.5 to 3208.5)	2400.1 (2334.8 to 2466.0)	600.3 (568.8 to 635.6)	129.7 (109.9 to 152.8)	0.0 (0.0 to 0.0)
Belgium	1998	3191.6 (3116.1 to 3269.8)	2423.8 (2354.0 to 2487.7)	620.7 (590.5 to 655.3)	147.1 (128.2 to 168.6)	0.0 (0.0 to 0.0)	3213.1 (3137.1 to 3291.8)	2440.2 (2369.9 to 2504.5)	624.9 (594.5 to 659.8)	148.1 (129.1 to 169.7)	0.0 (0.0 to 0.0)
Belgium	1999	3324.0 (3243.9 to 3406.3)	2512.8 (2445.5 to 2583.9)	642.8 (613.9 to 674.0)	168.4 (148.3 to 192.1)	0.0 (0.0 to 0.0)	3346.4 (3265.8 to 3429.3)	2529.8 (2462.0 to 2601.3)	647.1 (618.1 to 678.5)	169.5 (149.4 to 193.4)	0.0 (0.0 to 0.0)
Belgium	2000	3479.6 (3400.8 to 3556.5)	2631.0 (2563.8 to 2700.4)	661.5 (633.5 to 691.7)	187.1 (166.7 to 208.2)	0.0 (0.0 to 0.0)	3503.1 (3423.8 to 3580.5)	2648.8 (2581.1 to 2718.7)	666.0 (637.7 to 696.4)	188.4 (167.8 to 209.6)	0.0 (0.0 to 0.0)
Belgium	2001	3583.3 (3503.8 to 3656.4)	2708.0 (2635.7 to 2775.3)	676.9 (649.6 to 705.4)	198.4 (178.6 to 218.3)	0.0 (0.0 to 0.0)	3607.5 (3527.5 to 3681.1)	2726.3 (2653.5 to 2794.1)	681.5 (654.0 to 710.1)	199.7 (179.8 to 219.8)	0.0 (0.0 to 0.0)

		2018 US Dollars per capita					2018 PPP per capita				
Country	Year	Health spending	Government health spending	Out-of-pocket health spending	Prepaid private health spending	Development assistance for health	Health spending	Government health spending	Out-of-pocket health spending	Prepaid private health spending	Development assistance for health
Belgium	2002	3773.2 (3696.3 to 3856.8)	2847.2 (2783.5 to 2918.3)	713.4 (687.1 to 741.8)	212.6 (193.6 to 233.1)	0.0 (0.0 to 0.0)	3798.7 (3721.3 to 3882.9)	2866.4 (2802.3 to 2938.0)	718.2 (691.8 to 746.8)	214.1 (194.9 to 234.7)	0.0 (0.0 to 0.0)
Belgium	2003	3979.9 (3903.4 to 4062.2)	3009.2 (2935.4 to 3082.8)	737.6 (709.4 to 766.8)	233.2 (214.1 to 254.1)	0.0 (0.0 to 0.0)	4006.8 (3929.8 to 4089.7)	3029.5 (2955.3 to 3103.6)	742.6 (714.2 to 772.0)	234.8 (215.5 to 255.8)	0.0 (0.0 to 0.0)
Belgium	2004	4180.4 (4103.2 to 4255.7)	3195.2 (3123.6 to 3263.6)	734.6 (705.2 to 762.7)	250.6 (228.3 to 275.0)	0.0 (0.0 to 0.0)	4208.6 (4130.9 to 4284.4)	3216.8 (3144.7 to 3285.7)	739.5 (710.0 to 767.8)	252.3 (229.9 to 276.9)	0.0 (0.0 to 0.0)
Belgium	2005	4240.9 (4164.0 to 4312.9)	3260.0 (3191.8 to 3332.2)	724.1 (696.2 to 752.6)	256.8 (234.2 to 280.9)	0.0 (0.0 to 0.0)	4269.6 (4192.2 to 4342.0)	3282.0 (3213.4 to 3354.7)	728.9 (700.9 to 757.7)	258.6 (235.8 to 282.8)	0.0 (0.0 to 0.0)
Belgium	2006	4282.8 (4205.7 to 4364.0)	3295.2 (3222.7 to 3368.4)	732.5 (705.4 to 760.3)	255.1 (234.2 to 278.9)	0.0 (0.0 to 0.0)	4311.8 (4234.1 to 4393.5)	3317.5 (3244.5 to 3391.2)	737.5 (710.2 to 765.5)	256.9 (235.8 to 280.8)	0.0 (0.0 to 0.0)
Belgium	2007	4415.8 (4335.5 to 4495.9)	3406.7 (3338.2 to 3484.4)	757.5 (729.8 to 787.3)	251.6 (230.4 to 273.3)	0.0 (0.0 to 0.0)	4445.7 (4364.8 to 4526.3)	3429.7 (3360.7 to 3508.0)	762.6 (734.7 to 792.7)	253.3 (232.0 to 275.1)	0.0 (0.0 to 0.0)
Belgium	2008	4563.8 (4479.9 to 4644.3)	3548.4 (3475.7 to 3617.7)	776.4 (746.9 to 805.3)	239.0 (217.9 to 259.8)	0.0 (0.0 to 0.0)	4594.6 (4510.2 to 4675.6)	3572.3 (3499.2 to 3642.1)	781.6 (752.0 to 810.7)	240.7 (219.4 to 261.6)	0.0 (0.0 to 0.0)
Belgium	2009	4642.9 (4561.0 to 4725.8)	3626.3 (3556.8 to 3696.0)	788.0 (760.0 to 817.1)	228.6 (208.4 to 248.1)	0.0 (0.0 to 0.0)	4674.2 (4591.8 to 4757.8)	3650.8 (3580.8 to 3721.0)	793.3 (765.1 to 822.6)	230.2 (209.8 to 249.8)	0.0 (0.0 to 0.0)
Belgium	2010	4789.0 (4706.9 to 4873.9)	3754.5 (3683.8 to 3827.0)	804.9 (775.0 to 834.9)	229.6 (209.7 to 250.1)	0.0 (0.0 to 0.0)	4821.3 (4738.7 to 4906.8)	3779.9 (3708.7 to 3852.9)	810.3 (780.3 to 840.5)	231.1 (211.1 to 251.8)	0.0 (0.0 to 0.0)
Belgium	2011	4872.9 (4789.5 to 4954.1)	3821.5 (3747.7 to 3893.8)	816.5 (785.0 to 845.5)	234.9 (213.1 to 256.4)	0.0 (0.0 to 0.0)	4905.8 (4821.9 to 4987.6)	3847.3 (3773.0 to 3920.1)	822.0 (790.3 to 851.2)	236.4 (214.5 to 258.2)	0.0 (0.0 to 0.0)
Belgium	2012	4923.0 (4845.2 to 5003.6)	3863.8 (3791.3 to 3939.3)	819.3 (789.7 to 846.6)	239.9 (218.4 to 262.9)	0.0 (0.0 to 0.0)	4956.3 (4878.0 to 5037.4)	3889.9 (3816.9 to 3965.9)	824.8 (795.0 to 852.3)	241.6 (219.9 to 264.7)	0.0 (0.0 to 0.0)
Belgium	2013	4955.6 (4870.5 to 5038.4)	3887.4 (3811.0 to 3961.0)	818.9 (789.0 to 847.6)	249.2 (228.4 to 274.4)	0.0 (0.0 to 0.0)	4989.0 (4903.4 to 5072.4)	3913.7 (3836.7 to 3987.8)	824.4 (794.3 to 853.3)	250.9 (229.9 to 276.3)	0.0 (0.0 to 0.0)
Belgium	2014	5008.4 (4928.0 to 5094.5)	3936.6 (3858.5 to 4012.7)	809.1 (779.9 to 840.1)	262.6 (241.5 to 288.7)	0.0 (0.0 to 0.0)	5042.2 (4961.3 to 5128.9)	3963.2 (3884.6 to 4039.8)	814.6 (785.1 to 845.8)	264.4 (243.1 to 290.6)	0.0 (0.0 to 0.0)
Belgium	2015	5022.1 (4941.2 to 5103.7)	3959.1 (3882.8 to 4032.9)	783.6 (752.9 to 817.8)	279.4 (254.0 to 307.1)	0.0 (0.0 to 0.0)	5056.0 (4974.6 to 5138.1)	3985.8 (3909.1 to 4060.2)	788.9 (758.0 to 823.3)	281.3 (255.8 to 309.2)	0.0 (0.0 to 0.0)
Belgium	2016	5014.3 (4893.7 to 5134.5)	3964.9 (3861.7 to 4073.2)	757.9 (717.5 to 801.3)	291.6 (257.1 to 325.6)	0.0 (0.0 to 0.0)	5048.2 (4926.8 to 5169.2)	3991.7 (3887.8 to 4100.7)	763.0 (722.3 to 806.7)	293.5 (258.8 to 327.8)	0.0 (0.0 to 0.0)
Belgium	2017	5045.6 (4924.1 to 5171.2)	3985.3 (3876.3 to 4094.4)	763.6 (722.5 to 809.8)	296.7 (261.4 to 331.6)	0.0 (0.0 to 0.0)	5079.7 (4957.4 to 5206.1)	4012.2 (3902.5 to 4122.1)	768.8 (727.3 to 815.2)	298.7 (263.2 to 333.9)	0.0 (0.0 to 0.0)
Belgium	2018	5093.9 (4970.6 to 5218.3)	4024.7 (3924.2 to 4132.6)	768.3 (724.8 to 815.7)	300.9 (265.2 to 336.4)	0.0 (0.0 to 0.0)	5128.4 (5004.1 to 5253.6)	4051.9 (3950.7 to 4160.5)	773.5 (729.7 to 821.2)	302.9 (267.0 to 338.7)	0.0 (0.0 to 0.0)
Belgium	2019	5138.4 (5010.0 to 5271.1)	4061.1 (3955.7 to 4174.4)	772.6 (726.6 to 821.1)	304.7 (268.5 to 340.3)	0.0 (0.0 to 0.0)	5173.1 (5043.9 to 5306.7)	4088.5 (3982.4 to 4202.6)	777.9 (731.5 to 826.7)	306.7 (270.4 to 342.6)	0.0 (0.0 to 0.0)
Belgium	2020	5183.2 (5051.7 to 5315.8)	4097.6 (3986.4 to 4217.2)	777.1 (725.6 to 827.4)	308.5 (271.8 to 345.4)	0.0 (0.0 to 0.0)	5218.2 (5085.8 to 5351.7)	4125.2 (4013.4 to 4245.7)	782.3 (730.5 to 833.0)	310.6 (273.7 to 347.7)	0.0 (0.0 to 0.0)
Belgium	2021	5224.4 (5088.0 to 5364.9)	4130.7 (4015.8 to 4249.5)	781.4 (729.0 to 834.2)	312.2 (275.4 to 349.0)	0.0 (0.0 to 0.0)	5259.7 (5122.4 to 5401.2)	4158.7 (4042.9 to 4278.3)	786.7 (733.9 to 839.8)	314.3 (277.3 to 351.4)	0.0 (0.0 to 0.0)
Belgium	2022	5267.8 (5129.5 to 5404.2)	4166.0 (4049.6 to 4289.4)	785.9 (730.1 to 840.3)	315.9 (278.4 to 353.3)	0.0 (0.0 to 0.0)	5303.3 (5164.2 to 5440.7)	4194.1 (4077.0 to 4318.4)	791.2 (735.0 to 846.0)	318.0 (280.3 to 355.7)	0.0 (0.0 to 0.0)

		2018 US Dollars per capita					2018 PPP per capita				
Country	Year	Health spending	Government health spending	Out-of-pocket health spending	Prepaid private health spending	Development assistance for health	Health spending	Government health spending	Out-of-pocket health spending	Prepaid private health spending	Development assistance for health
Belgium	2023	5309.5 (5164.2 to 5458.4)	4199.6 (4076.4 to 4330.6)	790.4 (728.9 to 847.4)	319.6 (281.7 to 357.7)	0.0 (0.0 to 0.0)	5345.4 (5199.1 to 5495.3)	4228.0 (4104.0 to 4359.9)	795.7 (733.9 to 853.1)	321.7 (283.6 to 360.1)	0.0 (0.0 to 0.0)
Belgium	2024	5353.0 (5199.6 to 5503.1)	4235.0 (4107.6 to 4373.2)	794.6 (733.3 to 855.9)	323.4 (284.2 to 362.4)	0.0 (0.0 to 0.0)	5389.2 (5234.7 to 5540.3)	4263.6 (4135.4 to 4402.7)	800.0 (738.3 to 861.7)	325.6 (286.2 to 364.9)	0.0 (0.0 to 0.0)
Belgium	2025	5391.6 (5230.1 to 5556.7)	4268.3 (4133.0 to 4413.7)	796.3 (734.0 to 856.8)	327.0 (287.5 to 366.4)	0.0 (0.0 to 0.0)	5428.0 (5265.4 to 5594.2)	4297.1 (4160.9 to 4443.5)	801.7 (739.0 to 862.6)	329.2 (289.4 to 368.8)	0.0 (0.0 to 0.0)
Belgium	2026	5428.5 (5258.3 to 5595.7)	4300.3 (4155.0 to 4453.0)	797.7 (733.9 to 860.6)	330.5 (291.2 to 369.7)	0.0 (0.0 to 0.0)	5465.2 (5293.8 to 5633.5)	4329.4 (4183.1 to 4483.0)	803.1 (738.9 to 866.4)	332.7 (293.1 to 372.2)	0.0 (0.0 to 0.0)
Belgium	2027	5461.5 (5283.1 to 5647.3)	4329.2 (4172.5 to 4493.4)	798.5 (734.3 to 863.8)	333.8 (293.5 to 373.8)	0.0 (0.0 to 0.0)	5498.4 (5318.8 to 5685.5)	4358.5 (4200.6 to 4523.7)	803.9 (739.3 to 869.6)	336.0 (295.5 to 376.4)	0.0 (0.0 to 0.0)
Belgium	2028	5490.4 (5299.1 to 5682.9)	4354.8 (4188.8 to 4529.4)	798.7 (732.3 to 867.3)	336.8 (296.0 to 377.9)	0.0 (0.0 to 0.0)	5527.5 (5334.9 to 5721.2)	4384.3 (4217.1 to 4560.0)	804.1 (737.3 to 873.2)	339.1 (298.0 to 380.5)	0.0 (0.0 to 0.0)
Belgium	2029	5519.0 (5316.9 to 5729.0)	4380.2 (4204.9 to 4564.3)	798.8 (730.3 to 871.5)	339.9 (298.5 to 382.1)	0.0 (0.0 to 0.0)	5556.3 (5352.8 to 5767.7)	4409.8 (4233.3 to 4595.1)	804.2 (735.3 to 877.4)	342.2 (300.5 to 384.7)	0.0 (0.0 to 0.0)
Belgium	2030	5549.7 (5339.4 to 5762.1)	4407.3 (4220.1 to 4601.2)	799.3 (729.0 to 874.3)	343.1 (301.1 to 386.0)	0.0 (0.0 to 0.0)	5587.2 (5375.5 to 5801.1)	4437.1 (4248.6 to 4632.3)	804.7 (734.0 to 880.2)	345.4 (303.1 to 388.6)	0.0 (0.0 to 0.0)
Belgium	2031	5584.7 (5360.2 to 5819.5)	4437.7 (4233.2 to 4646.5)	800.4 (729.7 to 877.4)	346.6 (304.1 to 389.9)	0.0 (0.0 to 0.0)	5622.4 (5396.5 to 5858.8)	4467.7 (4261.8 to 4677.9)	805.8 (734.6 to 883.3)	348.9 (306.2 to 392.5)	0.0 (0.0 to 0.0)
Belgium	2032	5621.2 (5388.9 to 5860.0)	4469.4 (4253.3 to 4693.9)	801.6 (730.3 to 881.2)	350.2 (307.2 to 394.8)	0.0 (0.0 to 0.0)	5659.2 (5425.3 to 5899.6)	4499.6 (4282.0 to 4725.6)	807.0 (735.2 to 887.2)	352.6 (309.3 to 397.5)	0.0 (0.0 to 0.0)
Belgium	2033	5661.0 (5412.2 to 5921.6)	4503.7 (4275.9 to 4743.2)	803.3 (729.9 to 884.1)	354.0 (310.4 to 400.0)	0.0 (0.0 to 0.0)	5699.2 (5448.8 to 5961.6)	4534.1 (4304.8 to 4775.2)	808.7 (734.8 to 890.1)	356.4 (312.5 to 402.7)	0.0 (0.0 to 0.0)
Belgium	2034	5703.5 (5446.9 to 5978.1)	4540.1 (4300.2 to 4796.5)	805.3 (730.0 to 888.2)	358.1 (313.4 to 405.5)	0.0 (0.0 to 0.0)	5742.0 (5483.7 to 6018.5)	4570.8 (4329.3 to 4828.9)	810.7 (734.9 to 894.2)	360.5 (315.6 to 408.2)	0.0 (0.0 to 0.0)
Belgium	2035	5745.9 (5466.0 to 6034.4)	4576.5 (4322.1 to 4850.0)	807.3 (729.1 to 891.6)	362.1 (316.5 to 411.7)	0.0 (0.0 to 0.0)	5784.7 (5502.9 to 6075.2)	4607.4 (4351.3 to 4882.7)	812.7 (734.0 to 897.6)	364.6 (318.6 to 414.5)	0.0 (0.0 to 0.0)
Belgium	2036	5794.8 (5504.8 to 6101.2)	4618.1 (4350.3 to 4905.7)	810.1 (730.8 to 896.0)	366.6 (319.8 to 418.5)	0.0 (0.0 to 0.0)	5833.9 (5542.0 to 6142.4)	4649.3 (4379.7 to 4938.8)	815.6 (735.7 to 902.1)	369.1 (321.9 to 421.3)	0.0 (0.0 to 0.0)
Belgium	2037	5845.8 (5535.4 to 6171.2)	4661.4 (4376.5 to 4968.7)	813.2 (732.5 to 900.6)	371.2 (324.1 to 424.0)	0.0 (0.0 to 0.0)	5885.3 (5572.8 to 6212.9)	4692.9 (4406.1 to 5002.3)	818.7 (737.4 to 906.7)	373.8 (326.2 to 426.9)	0.0 (0.0 to 0.0)
Belgium	2038	5896.0 (5575.4 to 6238.4)	4704.0 (4402.1 to 5031.6)	816.1 (733.7 to 905.0)	375.9 (327.8 to 430.5)	0.0 (0.0 to 0.0)	5935.9 (5613.1 to 6280.5)	4735.8 (4431.9 to 5065.6)	821.6 (738.7 to 911.2)	378.4 (330.0 to 433.4)	0.0 (0.0 to 0.0)
Belgium	2039	5949.8 (5607.9 to 6315.1)	4749.6 (4434.1 to 5096.2)	819.5 (735.5 to 910.0)	380.8 (332.0 to 436.6)	0.0 (0.0 to 0.0)	5990.0 (5645.8 to 6357.8)	4781.7 (4464.1 to 5130.6)	825.0 (740.4 to 916.1)	383.3 (334.2 to 439.6)	0.0 (0.0 to 0.0)
Belgium	2040	6004.2 (5646.4 to 6390.3)	4795.6 (4467.9 to 5161.4)	822.9 (737.3 to 915.0)	385.7 (336.2 to 442.4)	0.0 (0.0 to 0.0)	6044.7 (5684.6 to 6433.5)	4828.0 (4498.1 to 5196.3)	828.4 (742.3 to 921.2)	388.3 (338.5 to 445.4)	0.0 (0.0 to 0.0)
Belgium	2041	6061.5 (5678.6 to 6462.7)	4844.0 (4501.8 to 5228.8)	826.6 (739.4 to 920.5)	390.9 (339.8 to 449.3)	0.0 (0.0 to 0.0)	6102.4 (5716.9 to 6506.3)	4876.7 (4532.2 to 5264.2)	832.2 (744.4 to 926.7)	393.5 (342.1 to 452.3)	0.0 (0.0 to 0.0)
Belgium	2042	6120.4 (5727.2 to 6541.3)	4893.6 (4529.0 to 5297.8)	830.5 (741.8 to 926.7)	396.2 (344.7 to 456.4)	0.0 (0.0 to 0.0)	6161.8 (5765.9 to 6585.5)	4926.7 (4559.6 to 5333.6)	836.2 (746.8 to 933.0)	398.9 (347.0 to 459.5)	0.0 (0.0 to 0.0)
Belgium	2043	6181.5 (5771.3 to 6619.4)	4945.1 (4558.0 to 5369.0)	834.7 (743.8 to 933.3)	401.7 (348.7 to 464.3)	0.0 (0.0 to 0.0)	6223.2 (5810.3 to 6664.1)	4978.5 (4588.8 to 5405.3)	840.3 (748.8 to 939.6)	404.4 (351.0 to 467.5)	0.0 (0.0 to 0.0)

		2018 US Dollars per capita					2018 PPP per capita				
Country	Year	Health spending	Government health spending	Out-of-pocket health spending	Prepaid private health spending	Development assistance for health	Health spending	Government health spending	Out-of-pocket health spending	Prepaid private health spending	Development assistance for health
Belgium	2044	6243.2 (5813.9 to 6708.4)	4997.2 (4593.2 to 5441.2)	838.8 (745.7 to 938.9)	407.3 (352.9 to 471.1)	0.0 (0.0 to 0.0)	6285.4 (5853.2 to 6753.7)	5030.9 (4624.2 to 5477.9)	844.5 (750.8 to 945.2)	410.0 (355.3 to 474.3)	0.0 (0.0 to 0.0)
Belgium	2045	6304.0 (5856.8 to 6781.5)	5048.4 (4626.1 to 5512.9)	842.8 (747.7 to 944.1)	412.8 (357.9 to 479.0)	0.0 (0.0 to 0.0)	6346.6 (5896.4 to 6827.3)	5082.6 (4657.3 to 5550.1)	848.5 (752.7 to 950.5)	415.6 (360.3 to 482.3)	0.0 (0.0 to 0.0)
Belgium	2046	6365.7 (5898.5 to 6875.3)	5100.5 (4658.1 to 5590.2)	846.8 (749.8 to 948.7)	418.4 (362.5 to 487.2)	0.0 (0.0 to 0.0)	6408.7 (5938.3 to 6921.8)	5135.0 (4689.6 to 5628.0)	852.5 (754.8 to 955.1)	421.2 (364.9 to 490.5)	0.0 (0.0 to 0.0)
Belgium	2047	6429.9 (5948.6 to 6954.9)	5154.6 (4694.9 to 5667.8)	851.0 (752.3 to 954.3)	424.2 (367.6 to 495.3)	0.0 (0.0 to 0.0)	6473.3 (5988.8 to 7001.9)	5189.4 (4726.6 to 5706.1)	856.8 (757.4 to 960.8)	427.1 (370.1 to 498.6)	0.0 (0.0 to 0.0)
Belgium	2048	6491.0 (5986.7 to 7049.0)	5206.3 (4729.3 to 5742.0)	854.8 (755.7 to 959.4)	429.9 (371.4 to 502.8)	0.0 (0.0 to 0.0)	6534.8 (6027.1 to 7096.6)	5241.5 (4761.2 to 5780.8)	860.6 (760.8 to 965.9)	432.8 (373.9 to 506.2)	0.0 (0.0 to 0.0)
Belgium	2049	6553.1 (6034.9 to 7123.9)	5258.9 (4762.7 to 5817.4)	858.6 (759.8 to 964.6)	435.6 (375.3 to 511.5)	0.0 (0.0 to 0.0)	6597.4 (6075.6 to 7172.0)	5294.4 (4794.9 to 5856.7)	864.4 (764.9 to 971.1)	438.5 (377.8 to 515.0)	0.0 (0.0 to 0.0)
Belgium	2050	6613.3 (6062.9 to 7218.9)	5309.9 (4794.7 to 5890.4)	862.1 (761.8 to 969.4)	441.2 (378.9 to 520.2)	0.0 (0.0 to 0.0)	6657.9 (6103.9 to 7267.6)	5345.8 (4827.1 to 5930.2)	868.0 (767.0 to 976.0)	444.2 (381.4 to 523.7)	0.0 (0.0 to 0.0)
Belize	1995	141.3 (120.7 to 163.1)	91.7 (76.3 to 108.6)	45.7 (34.6 to 59.6)	1.8 (0.8 to 3.3)	2.1 (2.1 to 2.1)	255.1 (218.0 to 294.5)	165.6 (137.7 to 196.1)	82.4 (62.5 to 107.6)	3.2 (1.5 to 6.0)	3.8 (3.8 to 3.8)
Belize	1996	131.8 (113.9 to 150.8)	83.3 (70.6 to 97.0)	44.3 (34.0 to 57.0)	1.8 (0.8 to 3.3)	2.4 (2.4 to 2.4)	238.0 (205.7 to 272.3)	150.5 (127.4 to 175.2)	80.0 (61.3 to 102.8)	3.2 (1.5 to 5.9)	4.3 (4.3 to 4.3)
Belize	1997	134.3 (117.6 to 152.1)	82.8 (70.1 to 95.5)	45.7 (35.2 to 58.4)	1.9 (0.8 to 3.5)	3.9 (3.9 to 3.9)	242.4 (212.3 to 274.7)	149.5 (126.6 to 172.5)	82.5 (63.6 to 105.4)	3.4 (1.5 to 6.3)	7.1 (7.1 to 7.1)
Belize	1998	134.4 (118.4 to 151.2)	82.5 (71.1 to 94.7)	47.5 (37.0 to 59.6)	2.0 (0.9 to 3.7)	2.4 (2.4 to 2.4)	242.6 (213.8 to 273.1)	148.9 (128.4 to 171.0)	85.8 (66.7 to 107.5)	3.6 (1.7 to 6.7)	4.3 (4.3 to 4.3)
Belize	1999	146.0 (129.1 to 163.5)	85.5 (74.3 to 97.8)	52.2 (40.7 to 65.2)	2.3 (1.0 to 4.2)	6.1 (6.1 to 6.1)	263.7 (233.2 to 295.2)	154.3 (134.1 to 176.5)	94.2 (73.4 to 117.8)	4.1 (1.9 to 7.6)	11.0 (11.0 to 11.0)
Belize	2000	172.9 (154.5 to 193.4)	94.5 (82.2 to 108.1)	62.0 (48.4 to 77.5)	2.7 (1.2 to 5.0)	13.7 (13.7 to 13.7)	312.2 (278.9 to 349.2)	170.6 (148.4 to 195.2)	112.0 (87.4 to 140.0)	4.9 (2.2 to 9.0)	24.8 (24.8 to 24.8)
Belize	2001	180.3 (161.3 to 201.2)	99.3 (86.7 to 112.9)	66.9 (52.9 to 83.7)	3.0 (1.4 to 5.4)	11.1 (11.1 to 11.1)	325.5 (291.2 to 363.3)	179.3 (156.5 to 203.9)	120.8 (95.4 to 151.1)	5.3 (2.6 to 9.7)	20.0 (20.0 to 20.0)
Belize	2002	181.9 (161.9 to 203.1)	100.7 (87.9 to 114.1)	69.4 (54.8 to 86.5)	3.3 (1.6 to 6.0)	8.6 (8.6 to 8.6)	328.3 (292.2 to 366.7)	181.7 (158.7 to 206.0)	125.2 (99.0 to 156.1)	5.9 (2.9 to 10.8)	15.5 (15.5 to 15.5)
Belize	2003	194.4 (173.3 to 216.4)	109.4 (96.2 to 124.0)	72.6 (57.7 to 90.6)	4.3 (2.1 to 7.8)	8.1 (8.1 to 8.1)	350.9 (313.0 to 390.7)	197.5 (173.8 to 223.8)	131.0 (104.1 to 163.6)	7.7 (3.9 to 14.1)	14.7 (14.7 to 14.7)
Belize	2004	197.8 (176.8 to 220.3)	112.9 (99.9 to 127.9)	71.7 (57.0 to 89.2)	4.9 (2.4 to 8.7)	8.3 (8.3 to 8.3)	357.1 (319.2 to 397.7)	203.9 (180.3 to 231.0)	129.4 (102.8 to 161.1)	8.8 (4.4 to 15.8)	15.1 (15.1 to 15.1)
Belize	2005	198.5 (178.3 to 220.8)	116.3 (102.9 to 131.2)	68.7 (54.5 to 85.3)	5.5 (2.8 to 10.0)	7.9 (7.9 to 7.9)	358.4 (321.9 to 398.7)	210.1 (185.8 to 236.9)	124.1 (98.4 to 154.1)	10.0 (5.0 to 18.0)	14.3 (14.3 to 14.3)
Belize	2006	208.4 (187.2 to 230.7)	125.0 (110.1 to 141.7)	67.0 (53.9 to 83.5)	7.3 (3.7 to 13.0)	9.2 (9.2 to 9.2)	376.3 (337.9 to 416.6)	225.6 (198.7 to 255.8)	120.9 (97.3 to 150.8)	13.2 (6.7 to 23.5)	16.6 (16.6 to 16.6)
Belize	2007	216.7 (194.2 to 239.3)	134.6 (117.7 to 151.5)	65.6 (52.9 to 81.3)	9.0 (4.6 to 16.2)	7.4 (7.4 to 7.4)	391.2 (350.7 to 432.1)	243.0 (212.5 to 273.6)	118.5 (95.5 to 146.8)	16.2 (8.3 to 29.2)	13.4 (13.4 to 13.4)
Belize	2008	226.8 (203.3 to 250.5)	144.2 (126.8 to 162.0)	65.2 (52.5 to 80.7)	10.6 (5.5 to 19.3)	6.9 (6.9 to 6.9)	409.5 (367.0 to 452.3)	260.3 (229.0 to 292.5)	117.6 (94.8 to 145.8)	19.2 (9.9 to 34.9)	12.4 (12.4 to 12.4)

Country	Year	2018 US Dollars per capita					2018 PPP per capita				
		Health spending	Government health spending	Out-of-pocket health spending	Prepaid private health spending	Development assistance for health	Health spending	Government health spending	Out-of-pocket health spending	Prepaid private health spending	Development assistance for health
Belize	2009	241.2 (217.1 to 266.8)	156.2 (138.0 to 175.9)	65.6 (52.8 to 81.3)	12.5 (6.5 to 22.6)	6.9 (6.9 to 6.9)	435.5 (391.9 to 481.8)	282.1 (249.2 to 317.6)	118.4 (95.3 to 146.9)	22.6 (11.8 to 40.8)	12.5 (12.5 to 12.5)
Belize	2010	262.0 (236.4 to 290.7)	170.5 (151.2 to 191.7)	66.0 (52.8 to 81.8)	14.6 (7.6 to 25.7)	10.9 (10.9 to 10.9)	473.1 (426.9 to 524.9)	307.8 (273.0 to 346.1)	119.2 (95.3 to 147.8)	26.4 (13.8 to 46.3)	19.7 (19.7 to 19.7)
Belize	2011	255.0 (229.9 to 282.0)	170.3 (151.6 to 192.0)	63.8 (50.7 to 79.5)	14.9 (7.9 to 26.1)	6.1 (6.1 to 6.1)	460.5 (415.1 to 509.2)	307.5 (273.7 to 346.7)	115.1 (91.6 to 143.5)	26.9 (14.2 to 47.1)	10.9 (10.9 to 10.9)
Belize	2012	259.2 (233.4 to 285.6)	171.3 (152.7 to 191.9)	63.6 (50.6 to 78.8)	15.5 (8.1 to 26.8)	8.8 (8.8 to 8.8)	468.0 (421.4 to 515.6)	309.4 (275.7 to 346.5)	114.8 (91.3 to 142.3)	27.9 (14.5 to 48.4)	15.9 (15.9 to 15.9)
Belize	2013	285.3 (258.5 to 313.1)	170.5 (152.0 to 191.1)	64.0 (50.7 to 79.6)	15.8 (8.2 to 27.0)	35.0 (35.0 to 35.0)	515.1 (466.7 to 565.3)	307.9 (274.4 to 345.0)	115.6 (91.5 to 143.7)	28.5 (14.9 to 48.8)	63.1 (63.1 to 63.1)
Belize	2014	262.1 (235.2 to 290.4)	173.7 (153.5 to 195.1)	64.7 (50.5 to 81.1)	16.2 (8.4 to 27.8)	7.5 (7.5 to 7.5)	473.2 (424.7 to 524.3)	313.7 (277.1 to 352.2)	116.7 (91.2 to 146.5)	29.3 (15.1 to 50.2)	13.5 (13.5 to 13.5)
Belize	2015	283.7 (254.0 to 317.2)	185.5 (162.5 to 212.0)	66.2 (51.6 to 83.1)	17.7 (9.1 to 30.6)	14.4 (14.4 to 14.4)	512.3 (458.6 to 572.7)	335.0 (293.4 to 382.8)	119.5 (93.2 to 150.0)	32.0 (16.5 to 55.2)	25.9 (25.9 to 25.9)
Belize	2016	283.0 (248.6 to 317.2)	187.6 (161.5 to 215.9)	66.3 (50.5 to 84.8)	19.3 (9.6 to 33.5)	9.8 (9.8 to 9.8)	511.0 (448.9 to 572.7)	338.8 (291.6 to 389.7)	119.8 (91.2 to 153.1)	34.8 (17.3 to 60.4)	17.7 (17.7 to 17.7)
Belize	2017	280.1 (247.5 to 315.2)	189.4 (162.7 to 219.0)	65.4 (49.8 to 83.8)	18.9 (9.4 to 32.9)	6.4 (6.4 to 6.4)	505.7 (446.9 to 569.1)	341.9 (293.7 to 395.4)	118.0 (89.8 to 151.3)	34.2 (17.0 to 59.5)	11.6 (11.6 to 11.6)
Belize	2018	282.6 (247.4 to 317.2)	191.5 (164.4 to 222.4)	65.9 (50.1 to 84.4)	19.2 (9.5 to 33.4)	6.0 (6.0 to 6.0)	510.3 (446.7 to 572.7)	345.8 (296.9 to 401.6)	118.9 (90.5 to 152.5)	34.7 (17.2 to 60.2)	10.9 (10.9 to 10.9)
Belize	2019	286.3 (252.0 to 322.6)	194.2 (166.6 to 226.2)	66.5 (50.7 to 85.3)	19.5 (9.7 to 34.0)	6.0 (5.6 to 6.4)	516.9 (455.0 to 582.4)	350.6 (300.8 to 408.3)	120.1 (91.5 to 154.0)	35.3 (17.5 to 61.3)	10.9 (10.2 to 11.5)
Belize	2020	289.9 (252.6 to 325.3)	196.8 (167.9 to 229.7)	67.1 (51.2 to 86.1)	19.9 (9.9 to 34.4)	6.1 (5.6 to 6.6)	523.4 (456.1 to 587.4)	355.3 (303.1 to 414.7)	121.2 (92.5 to 155.5)	35.9 (17.8 to 62.2)	11.0 (10.1 to 11.9)
Belize	2021	293.6 (258.1 to 331.9)	199.6 (170.9 to 232.9)	67.7 (51.6 to 86.8)	20.2 (10.0 to 34.9)	6.2 (5.5 to 6.8)	530.2 (466.1 to 599.3)	360.4 (308.5 to 420.5)	122.3 (93.2 to 156.8)	36.4 (18.1 to 63.0)	11.1 (10.0 to 12.3)
Belize	2022	299.2 (260.9 to 337.7)	203.7 (174.0 to 239.2)	68.7 (52.3 to 88.1)	20.6 (10.3 to 35.7)	6.2 (5.5 to 7.0)	540.2 (471.0 to 609.8)	367.8 (314.2 to 431.8)	124.0 (94.5 to 159.1)	37.2 (18.5 to 64.4)	11.2 (9.9 to 12.6)
Belize	2023	304.9 (267.8 to 345.6)	208.0 (176.5 to 244.1)	69.6 (53.0 to 89.4)	21.0 (10.5 to 36.5)	6.3 (5.4 to 7.1)	550.6 (483.5 to 624.0)	375.5 (318.6 to 440.8)	125.7 (95.7 to 161.4)	38.0 (19.0 to 65.9)	11.3 (9.8 to 12.8)
Belize	2024	310.5 (269.6 to 351.2)	212.2 (179.6 to 248.8)	70.5 (53.6 to 90.5)	21.5 (10.7 to 37.2)	6.3 (5.4 to 7.3)	560.6 (486.8 to 634.2)	383.1 (324.3 to 449.2)	127.3 (96.8 to 163.5)	38.8 (19.3 to 67.2)	11.4 (9.8 to 13.2)
Belize	2025	315.7 (276.4 to 357.9)	216.1 (182.7 to 253.3)	71.3 (54.3 to 91.6)	21.9 (10.9 to 38.0)	6.4 (5.5 to 7.5)	570.0 (499.0 to 646.1)	390.1 (329.8 to 457.3)	128.8 (98.0 to 165.4)	39.5 (19.6 to 68.6)	11.6 (9.8 to 13.5)
Belize	2026	320.6 (278.2 to 363.4)	219.8 (185.3 to 258.4)	72.1 (54.8 to 92.6)	22.3 (11.0 to 38.7)	6.5 (5.5 to 7.7)	578.9 (502.3 to 656.1)	396.8 (334.6 to 466.6)	130.1 (99.0 to 167.3)	40.2 (19.9 to 69.8)	11.8 (9.9 to 13.8)
Belize	2027	325.7 (284.3 to 370.5)	223.7 (188.8 to 263.6)	72.7 (55.3 to 93.6)	22.7 (11.2 to 39.4)	6.6 (5.5 to 8.0)	588.2 (513.4 to 669.0)	404.0 (340.9 to 476.0)	131.3 (99.9 to 169.0)	40.9 (20.3 to 71.1)	11.9 (10.0 to 14.4)
Belize	2028	330.8 (286.3 to 376.8)	227.7 (192.1 to 268.9)	73.4 (55.8 to 94.4)	23.0 (11.5 to 40.0)	6.7 (5.6 to 8.2)	597.2 (516.9 to 680.4)	411.1 (346.8 to 485.4)	132.5 (100.8 to 170.5)	41.6 (20.7 to 72.3)	12.1 (10.2 to 14.8)
Belize	2029	337.1 (294.0 to 383.9)	232.8 (196.1 to 274.6)	74.0 (56.3 to 95.3)	23.5 (11.7 to 40.8)	6.8 (5.6 to 8.4)	608.6 (530.8 to 693.1)	420.3 (354.1 to 495.8)	133.7 (101.7 to 172.0)	42.4 (21.1 to 73.7)	12.3 (10.2 to 15.2)

Country	Year	2018 US Dollars per capita					2018 PPP per capita				
		Health spending	Government health spending	Out-of-pocket health spending	Prepaid private health spending	Development assistance for health	Health spending	Government health spending	Out-of-pocket health spending	Prepaid private health spending	Development assistance for health
Belize	2030	343.4 (295.8 to 391.3)	237.8 (199.6 to 280.3)	74.7 (56.8 to 96.1)	23.9 (11.9 to 41.7)	7.0 (5.7 to 8.6)	620.1 (534.0 to 706.6)	429.4 (360.4 to 506.0)	134.9 (102.6 to 173.5)	43.1 (21.5 to 75.2)	12.6 (10.2 to 15.4)
Belize	2031	349.8 (303.5 to 400.5)	243.0 (203.9 to 286.7)	75.4 (57.4 to 97.0)	24.3 (12.1 to 42.6)	7.1 (5.7 to 8.8)	631.7 (547.9 to 723.1)	438.8 (368.1 to 517.7)	136.2 (103.6 to 175.1)	43.9 (21.9 to 76.8)	12.8 (10.3 to 15.9)
Belize	2032	356.1 (305.5 to 407.1)	248.1 (207.1 to 294.9)	76.1 (57.9 to 97.8)	24.8 (12.4 to 43.3)	7.2 (5.8 to 9.1)	643.0 (551.6 to 735.1)	447.9 (373.9 to 532.4)	137.4 (104.5 to 176.5)	44.7 (22.3 to 78.3)	13.0 (10.4 to 16.4)
Belize	2033	362.7 (313.9 to 416.4)	253.3 (210.4 to 302.6)	76.8 (58.4 to 98.6)	25.2 (12.6 to 44.2)	7.3 (5.8 to 9.4)	654.9 (566.8 to 751.8)	457.4 (379.9 to 546.3)	138.7 (105.5 to 178.0)	45.5 (22.7 to 79.8)	13.2 (10.5 to 17.0)
Belize	2034	369.6 (315.0 to 423.8)	258.9 (214.2 to 310.7)	77.6 (59.0 to 99.5)	25.7 (12.8 to 45.1)	7.5 (5.9 to 9.6)	667.3 (568.7 to 765.2)	467.4 (386.8 to 561.1)	140.0 (106.5 to 179.7)	46.4 (23.2 to 81.4)	13.5 (10.6 to 17.4)
Belize	2035	376.4 (323.8 to 435.3)	264.3 (217.8 to 317.4)	78.3 (59.5 to 100.4)	26.1 (13.1 to 45.8)	7.6 (5.9 to 10.0)	679.6 (584.6 to 785.9)	477.3 (393.2 to 573.1)	141.4 (107.4 to 181.3)	47.2 (23.6 to 82.6)	13.8 (10.7 to 18.1)
Belize	2036	383.0 (324.7 to 442.5)	269.7 (221.1 to 325.6)	79.0 (60.0 to 101.2)	26.6 (13.3 to 46.5)	7.8 (6.0 to 10.5)	691.6 (586.3 to 799.0)	486.9 (399.1 to 587.9)	142.6 (108.3 to 182.7)	48.0 (23.9 to 83.9)	14.1 (10.8 to 18.9)
Belize	2037	390.0 (333.5 to 453.2)	275.3 (224.8 to 333.6)	79.7 (60.6 to 102.1)	27.1 (13.5 to 47.3)	7.9 (6.2 to 10.7)	704.1 (602.2 to 818.3)	497.1 (406.0 to 602.3)	143.9 (109.4 to 184.4)	48.9 (24.3 to 85.4)	14.3 (11.1 to 19.3)
Belize	2038	397.0 (335.4 to 459.4)	281.0 (228.6 to 340.6)	80.4 (61.0 to 103.1)	27.6 (13.7 to 48.0)	8.1 (6.2 to 11.1)	716.8 (605.7 to 829.5)	507.3 (412.7 to 615.0)	145.2 (110.2 to 186.1)	49.7 (24.7 to 86.7)	14.6 (11.2 to 20.0)
Belize	2039	404.2 (343.3 to 472.3)	286.7 (232.7 to 347.7)	81.1 (61.6 to 104.1)	28.0 (13.9 to 48.9)	8.3 (6.3 to 11.3)	729.7 (619.8 to 852.8)	517.7 (420.1 to 627.7)	146.5 (111.2 to 187.9)	50.6 (25.2 to 88.3)	14.9 (11.3 to 20.5)
Belize	2040	411.9 (346.0 to 477.5)	292.9 (237.0 to 357.7)	82.0 (62.2 to 105.2)	28.6 (14.2 to 49.9)	8.4 (6.4 to 11.6)	743.6 (624.7 to 862.1)	528.8 (428.0 to 645.8)	148.0 (112.4 to 190.0)	51.6 (25.7 to 90.1)	15.2 (11.6 to 20.9)
Belize	2041	419.1 (354.3 to 493.9)	298.7 (240.7 to 365.5)	82.8 (62.8 to 106.2)	29.1 (14.5 to 50.9)	8.6 (6.5 to 12.2)	756.7 (639.8 to 891.7)	539.2 (434.7 to 660.0)	149.4 (113.4 to 191.8)	52.5 (26.1 to 91.8)	15.6 (11.7 to 22.0)
Belize	2042	426.6 (357.1 to 498.6)	304.6 (244.3 to 374.7)	83.6 (63.4 to 107.3)	29.6 (14.7 to 51.9)	8.8 (6.6 to 12.8)	770.2 (644.7 to 900.3)	549.9 (441.1 to 676.6)	150.9 (114.4 to 193.8)	53.5 (26.6 to 93.7)	15.9 (11.9 to 23.1)
Belize	2043	434.2 (365.8 to 513.8)	310.6 (248.3 to 382.5)	84.4 (64.0 to 108.4)	30.2 (15.0 to 52.9)	9.0 (6.6 to 13.2)	783.9 (660.6 to 927.8)	560.8 (448.4 to 690.7)	152.4 (115.5 to 195.8)	54.4 (27.1 to 95.6)	16.3 (12.0 to 23.8)
Belize	2044	441.8 (369.1 to 517.1)	316.6 (252.0 to 392.9)	85.3 (64.6 to 109.6)	30.7 (15.3 to 53.9)	9.2 (6.8 to 13.6)	797.7 (666.4 to 933.6)	571.7 (455.0 to 709.4)	154.0 (116.6 to 197.9)	55.4 (27.6 to 97.4)	16.7 (12.3 to 24.5)
Belize	2045	449.8 (377.7 to 536.1)	323.0 (257.1 to 400.4)	86.1 (65.2 to 110.7)	31.3 (15.6 to 55.0)	9.4 (6.8 to 14.3)	812.2 (681.9 to 967.9)	583.2 (464.1 to 723.0)	155.5 (117.7 to 199.9)	56.5 (28.1 to 99.2)	17.0 (12.3 to 25.7)
Belize	2046	457.6 (379.6 to 538.9)	329.3 (261.2 to 410.3)	86.9 (65.7 to 111.8)	31.8 (15.9 to 55.9)	9.7 (7.0 to 14.7)	826.2 (685.5 to 973.0)	594.5 (471.6 to 740.8)	156.8 (118.7 to 201.8)	57.4 (28.6 to 101.0)	17.5 (12.6 to 26.5)
Belize	2047	465.7 (389.1 to 559.2)	335.8 (265.2 to 422.8)	87.6 (66.3 to 112.9)	32.4 (16.1 to 57.0)	9.9 (7.2 to 15.5)	840.8 (702.6 to 1009.7)	606.3 (478.9 to 763.4)	158.2 (119.7 to 203.8)	58.4 (29.1 to 102.9)	17.9 (13.0 to 27.9)
Belize	2048	473.7 (390.5 to 566.0)	342.2 (269.3 to 434.2)	88.4 (66.8 to 113.9)	32.9 (16.4 to 58.0)	10.2 (7.3 to 16.2)	855.3 (705.0 to 1021.9)	617.8 (486.3 to 783.9)	159.6 (120.7 to 205.7)	59.4 (29.6 to 104.7)	18.4 (13.2 to 29.3)
Belize	2049	481.9 (399.9 to 582.8)	348.8 (273.8 to 443.0)	89.1 (67.4 to 114.8)	33.5 (16.6 to 59.0)	10.5 (7.5 to 16.7)	870.0 (722.0 to 1052.2)	629.8 (494.4 to 799.8)	160.9 (121.7 to 207.2)	60.4 (30.0 to 106.5)	18.9 (13.5 to 30.2)
Belize	2050	490.2 (401.6 to 588.5)	355.5 (277.6 to 453.2)	89.9 (67.9 to 115.8)	34.0 (16.9 to 60.0)	10.8 (7.6 to 17.6)	885.1 (725.2 to 1062.6)	641.9 (501.2 to 818.3)	162.3 (122.6 to 209.0)	61.5 (30.5 to 108.4)	19.4 (13.8 to 31.8)

		2018 US Dollars per capita					2018 PPP per capita				
Country	Year	Health spending	Government health spending	Out-of-pocket health spending	Prepaid private health spending	Development assistance for health	Health spending	Government health spending	Out-of-pocket health spending	Prepaid private health spending	Development assistance for health
Benin	1995	28.3 (22.7 to 34.8)	7.5 (5.6 to 9.7)	16.2 (11.3 to 22.6)	1.8 (0.8 to 3.4)	2.8 (2.8 to 2.8)	73.6 (59.2 to 90.6)	19.4 (14.7 to 25.3)	42.3 (29.5 to 59.0)	4.6 (2.2 to 8.9)	7.3 (7.3 to 7.3)
Benin	1996	28.9 (23.5 to 35.4)	7.4 (5.6 to 9.6)	16.1 (11.3 to 22.3)	1.7 (0.8 to 3.4)	3.6 (3.6 to 3.6)	75.3 (61.1 to 92.3)	19.3 (14.6 to 25.1)	42.0 (29.4 to 58.2)	4.5 (2.2 to 8.8)	9.5 (9.5 to 9.5)
Benin	1997	27.8 (22.2 to 34.2)	7.4 (5.6 to 9.6)	16.1 (11.3 to 22.4)	1.7 (0.8 to 3.3)	2.6 (2.6 to 2.6)	72.5 (57.8 to 89.0)	19.3 (14.7 to 25.0)	42.1 (29.4 to 58.3)	4.5 (2.1 to 8.7)	6.7 (6.7 to 6.7)
Benin	1998	31.0 (25.5 to 37.4)	7.4 (5.6 to 9.7)	16.1 (11.2 to 22.4)	1.7 (0.8 to 3.2)	5.7 (5.7 to 5.7)	80.7 (66.5 to 97.5)	19.3 (14.5 to 25.3)	42.1 (29.1 to 58.4)	4.4 (2.2 to 8.4)	14.9 (14.9 to 14.9)
Benin	1999	29.4 (23.7 to 35.9)	7.6 (5.8 to 10.1)	16.5 (11.5 to 22.9)	1.7 (0.8 to 3.2)	3.5 (3.5 to 3.5)	76.5 (61.8 to 93.4)	19.9 (15.2 to 26.2)	42.9 (29.8 to 59.6)	4.4 (2.1 to 8.3)	9.2 (9.2 to 9.2)
Benin	2000	31.0 (25.2 to 37.8)	8.0 (6.1 to 10.5)	16.9 (11.9 to 23.4)	1.7 (0.8 to 3.2)	4.5 (4.5 to 4.5)	80.8 (65.7 to 98.4)	20.8 (15.9 to 27.3)	43.9 (30.9 to 60.9)	4.4 (2.1 to 8.3)	11.7 (11.7 to 11.7)
Benin	2001	30.8 (25.2 to 38.3)	8.2 (6.2 to 10.8)	17.1 (12.2 to 23.6)	1.7 (0.8 to 3.2)	3.9 (3.9 to 3.9)	80.3 (65.7 to 99.7)	21.3 (16.2 to 28.0)	44.5 (31.7 to 61.6)	4.4 (2.1 to 8.2)	10.1 (10.1 to 10.1)
Benin	2002	30.1 (24.7 to 37.5)	7.8 (6.0 to 10.1)	17.0 (12.0 to 23.7)	1.7 (0.8 to 3.1)	3.6 (3.6 to 3.6)	78.5 (64.5 to 97.6)	20.3 (15.5 to 26.4)	44.3 (31.2 to 61.7)	4.4 (2.1 to 8.2)	9.5 (9.5 to 9.5)
Benin	2003	31.4 (26.1 to 38.4)	7.5 (5.7 to 9.7)	16.8 (11.8 to 23.4)	1.7 (0.8 to 3.1)	5.5 (5.5 to 5.5)	81.9 (67.9 to 100.1)	19.5 (14.8 to 25.2)	43.7 (30.8 to 60.9)	4.4 (2.0 to 8.2)	14.3 (14.3 to 14.3)
Benin	2004	33.3 (28.1 to 39.9)	7.5 (5.7 to 9.6)	16.6 (11.8 to 22.6)	1.7 (0.8 to 3.3)	7.4 (7.4 to 7.4)	86.6 (73.2 to 104.0)	19.6 (15.0 to 25.0)	43.3 (30.8 to 58.8)	4.4 (2.1 to 8.5)	19.3 (19.3 to 19.3)
Benin	2005	33.2 (28.3 to 39.5)	7.3 (5.6 to 9.4)	16.0 (11.4 to 21.9)	1.7 (0.8 to 3.3)	8.2 (8.2 to 8.2)	86.6 (73.8 to 103.0)	19.0 (14.5 to 24.4)	41.7 (29.8 to 57.1)	4.4 (2.1 to 8.5)	21.4 (21.4 to 21.4)
Benin	2006	32.2 (27.3 to 38.3)	7.2 (5.5 to 9.3)	15.7 (11.3 to 21.3)	1.7 (0.8 to 3.2)	7.6 (7.6 to 7.6)	83.9 (71.2 to 99.7)	18.8 (14.3 to 24.2)	40.8 (29.3 to 55.5)	4.4 (2.1 to 8.4)	19.8 (19.8 to 19.8)
Benin	2007	31.2 (26.3 to 37.2)	7.1 (5.3 to 9.1)	15.2 (10.7 to 20.8)	1.7 (0.8 to 3.3)	7.2 (7.2 to 7.2)	81.3 (68.6 to 96.8)	18.4 (13.9 to 23.7)	39.7 (27.9 to 54.1)	4.4 (2.0 to 8.6)	18.7 (18.7 to 18.7)
Benin	2008	32.6 (27.6 to 38.3)	7.2 (5.5 to 9.1)	14.8 (10.5 to 20.3)	1.7 (0.8 to 3.4)	8.8 (8.8 to 8.8)	84.8 (71.9 to 99.8)	18.8 (14.3 to 23.8)	38.6 (27.4 to 53.0)	4.5 (2.0 to 8.9)	23.0 (23.0 to 23.0)
Benin	2009	35.4 (30.4 to 41.3)	7.7 (5.9 to 9.7)	14.7 (10.4 to 20.2)	1.8 (0.8 to 3.4)	11.1 (11.1 to 11.1)	92.1 (79.1 to 107.6)	20.1 (15.3 to 25.4)	38.4 (27.1 to 52.7)	4.6 (2.2 to 8.8)	29.0 (29.0 to 29.0)
Benin	2010	34.2 (29.0 to 40.2)	7.8 (6.0 to 9.9)	14.6 (10.2 to 20.0)	1.8 (0.8 to 3.3)	10.0 (10.0 to 10.0)	89.2 (75.6 to 104.7)	20.4 (15.6 to 25.9)	38.0 (26.5 to 52.1)	4.6 (2.1 to 8.7)	26.1 (26.1 to 26.1)
Benin	2011	35.9 (30.7 to 41.9)	7.9 (6.1 to 10.1)	14.4 (10.2 to 19.8)	1.8 (0.8 to 3.4)	11.8 (11.8 to 11.8)	93.5 (80.0 to 109.1)	20.6 (15.9 to 26.3)	37.5 (26.5 to 51.5)	4.6 (2.1 to 8.8)	30.7 (30.7 to 30.7)
Benin	2012	32.0 (26.9 to 37.9)	7.9 (6.0 to 10.1)	14.2 (10.0 to 19.7)	1.8 (0.8 to 3.5)	8.1 (8.1 to 8.1)	83.3 (70.0 to 98.7)	20.6 (15.6 to 26.3)	36.9 (25.9 to 51.3)	4.7 (2.2 to 9.0)	21.1 (21.1 to 21.1)
Benin	2013	31.2 (26.2 to 37.1)	7.9 (6.0 to 10.0)	14.1 (9.8 to 19.7)	1.9 (0.8 to 3.5)	7.3 (7.3 to 7.3)	81.2 (68.2 to 96.5)	20.5 (15.6 to 26.1)	36.9 (25.5 to 51.3)	4.9 (2.2 to 9.1)	18.9 (18.9 to 18.9)
Benin	2014	33.6 (28.5 to 39.7)	7.5 (5.6 to 9.5)	14.1 (9.8 to 19.5)	1.9 (0.9 to 3.5)	10.1 (10.1 to 10.1)	87.4 (74.3 to 103.5)	19.4 (14.7 to 24.7)	36.7 (25.5 to 50.8)	4.9 (2.3 to 9.1)	26.4 (26.4 to 26.4)
Benin	2015	30.9 (25.8 to 37.2)	7.2 (5.4 to 9.1)	14.0 (9.7 to 19.3)	1.8 (0.9 to 3.5)	7.9 (7.9 to 7.9)	80.6 (67.3 to 97.0)	18.6 (14.1 to 23.7)	36.5 (25.2 to 50.3)	4.8 (2.3 to 9.1)	20.7 (20.7 to 20.7)

Country	Year	2018 US Dollars per capita					2018 PPP per capita				
		Health spending	Government health spending	Out-of-pocket health spending	Prepaid private health spending	Development assistance for health	Health spending	Government health spending	Out-of-pocket health spending	Prepaid private health spending	Development assistance for health
Benin	2016	31.7 (26.8 to 37.7)	7.1 (5.3 to 9.0)	14.1 (9.8 to 19.5)	1.9 (0.9 to 3.5)	8.7 (8.7 to 8.7)	82.6 (69.7 to 98.2)	18.4 (13.8 to 23.5)	36.8 (25.4 to 50.7)	4.9 (2.3 to 9.2)	22.5 (22.5 to 22.5)
Benin	2017	36.4 (31.4 to 42.7)	7.9 (6.0 to 10.2)	14.6 (10.1 to 20.2)	2.0 (1.0 to 3.8)	11.8 (11.8 to 11.8)	94.8 (81.7 to 111.3)	20.7 (15.5 to 26.5)	38.1 (26.3 to 52.5)	5.2 (2.5 to 9.8)	30.8 (30.8 to 30.8)
Benin	2018	35.5 (30.1 to 41.6)	8.1 (6.1 to 10.4)	14.8 (10.2 to 20.5)	2.0 (1.0 to 3.8)	10.6 (10.5 to 10.7)	92.4 (78.4 to 108.3)	21.1 (15.9 to 27.0)	38.5 (26.6 to 53.5)	5.3 (2.5 to 9.9)	27.6 (27.4 to 27.8)
Benin	2019	35.7 (30.4 to 42.0)	8.3 (6.2 to 10.6)	14.9 (10.3 to 20.7)	2.1 (1.0 to 3.9)	10.5 (9.7 to 11.1)	93.0 (79.1 to 109.3)	21.5 (16.1 to 27.7)	38.9 (26.9 to 53.9)	5.4 (2.6 to 10.2)	27.2 (25.4 to 28.9)
Benin	2020	36.0 (30.3 to 42.4)	8.4 (6.3 to 10.8)	15.1 (10.5 to 20.9)	2.1 (1.0 to 4.0)	10.4 (9.5 to 11.3)	93.8 (78.9 to 110.4)	21.9 (16.5 to 28.2)	39.3 (27.2 to 54.5)	5.5 (2.6 to 10.4)	27.1 (24.8 to 29.4)
Benin	2021	36.3 (30.8 to 42.9)	8.5 (6.4 to 11.0)	15.2 (10.6 to 21.1)	2.1 (1.0 to 4.1)	10.4 (9.3 to 11.5)	94.6 (80.3 to 111.8)	22.2 (16.7 to 28.6)	39.7 (27.5 to 55.1)	5.6 (2.7 to 10.6)	27.1 (24.3 to 30.0)
Benin	2022	36.7 (30.8 to 43.3)	8.7 (6.5 to 11.2)	15.4 (10.7 to 21.4)	2.2 (1.0 to 4.1)	10.5 (9.3 to 11.8)	95.7 (80.3 to 112.8)	22.6 (17.0 to 29.1)	40.1 (27.8 to 55.7)	5.7 (2.7 to 10.8)	27.3 (24.2 to 30.6)
Benin	2023	37.2 (31.4 to 44.1)	8.9 (6.7 to 11.4)	15.6 (10.8 to 21.7)	2.2 (1.1 to 4.2)	10.5 (9.1 to 12.0)	96.8 (81.9 to 114.9)	23.1 (17.3 to 29.6)	40.6 (28.1 to 56.4)	5.8 (2.8 to 11.0)	27.4 (23.7 to 31.2)
Benin	2024	37.6 (31.4 to 44.6)	9.0 (6.8 to 11.6)	15.7 (10.9 to 21.9)	2.3 (1.1 to 4.3)	10.6 (9.1 to 12.2)	98.0 (81.9 to 116.1)	23.5 (17.6 to 30.3)	41.0 (28.3 to 57.0)	5.9 (2.8 to 11.1)	27.6 (23.7 to 31.7)
Benin	2025	38.0 (32.1 to 45.1)	9.2 (6.9 to 11.9)	15.9 (11.0 to 22.2)	2.3 (1.1 to 4.4)	10.6 (9.0 to 12.4)	99.0 (83.6 to 117.5)	23.9 (17.9 to 30.9)	41.4 (28.7 to 57.7)	6.0 (2.9 to 11.4)	27.7 (23.5 to 32.3)
Benin	2026	38.4 (32.0 to 45.3)	9.3 (7.0 to 12.1)	16.0 (11.1 to 22.3)	2.4 (1.1 to 4.4)	10.7 (9.0 to 12.5)	100.1 (83.5 to 118.0)	24.3 (18.2 to 31.4)	41.8 (28.9 to 58.2)	6.1 (2.9 to 11.6)	27.8 (23.4 to 32.5)
Benin	2027	38.9 (32.6 to 46.0)	9.5 (7.1 to 12.3)	16.2 (11.2 to 22.6)	2.4 (1.1 to 4.5)	10.7 (9.0 to 12.7)	101.2 (84.8 to 119.8)	24.8 (18.5 to 32.1)	42.2 (29.1 to 58.8)	6.2 (3.0 to 11.8)	28.0 (23.4 to 33.2)
Benin	2028	39.4 (32.5 to 46.8)	9.7 (7.2 to 12.6)	16.4 (11.3 to 22.8)	2.5 (1.2 to 4.6)	10.8 (9.0 to 13.0)	102.7 (84.7 to 122.0)	25.4 (18.9 to 32.9)	42.8 (29.5 to 59.4)	6.4 (3.1 to 12.1)	28.1 (23.4 to 34.0)
Benin	2029	40.0 (33.4 to 47.4)	10.0 (7.4 to 13.0)	16.7 (11.5 to 23.0)	2.5 (1.2 to 4.8)	10.9 (8.9 to 13.3)	104.1 (87.1 to 123.5)	25.9 (19.2 to 33.8)	43.4 (30.0 to 59.9)	6.5 (3.1 to 12.4)	28.3 (23.3 to 34.5)
Benin	2030	40.6 (33.5 to 48.2)	10.2 (7.5 to 13.2)	16.9 (11.7 to 23.3)	2.6 (1.2 to 4.9)	10.9 (8.9 to 13.4)	105.6 (87.2 to 125.5)	26.5 (19.6 to 34.5)	44.0 (30.4 to 60.6)	6.7 (3.2 to 12.7)	28.4 (23.3 to 34.9)
Benin	2031	41.2 (34.5 to 49.1)	10.4 (7.7 to 13.6)	17.1 (11.8 to 23.7)	2.6 (1.3 to 5.0)	11.0 (8.9 to 13.7)	107.3 (89.7 to 127.8)	27.2 (20.1 to 35.5)	44.6 (30.8 to 61.8)	6.8 (3.3 to 13.0)	28.6 (23.1 to 35.8)
Benin	2032	41.8 (34.7 to 50.0)	10.7 (7.9 to 14.0)	17.4 (12.0 to 24.2)	2.7 (1.3 to 5.1)	11.1 (8.9 to 14.0)	109.0 (90.5 to 130.2)	27.9 (20.6 to 36.4)	45.3 (31.2 to 63.0)	7.0 (3.3 to 13.3)	28.8 (23.1 to 36.5)
Benin	2033	42.5 (35.7 to 50.5)	11.0 (8.1 to 14.4)	17.7 (12.1 to 24.5)	2.7 (1.3 to 5.2)	11.2 (8.8 to 14.4)	110.8 (93.1 to 131.6)	28.6 (21.2 to 37.5)	46.0 (31.6 to 63.8)	7.2 (3.4 to 13.7)	29.1 (23.0 to 37.5)
Benin	2034	43.2 (36.1 to 51.5)	11.2 (8.3 to 14.7)	17.9 (12.3 to 24.9)	2.8 (1.3 to 5.4)	11.3 (8.9 to 14.6)	112.6 (94.1 to 134.1)	29.3 (21.6 to 38.2)	46.6 (32.1 to 64.9)	7.3 (3.5 to 14.0)	29.4 (23.1 to 38.0)
Benin	2035	44.0 (36.3 to 52.2)	11.5 (8.5 to 15.1)	18.2 (12.5 to 25.4)	2.9 (1.4 to 5.5)	11.4 (8.9 to 14.9)	114.5 (94.5 to 136.0)	30.0 (22.1 to 39.3)	47.3 (32.6 to 66.2)	7.5 (3.6 to 14.3)	29.7 (23.1 to 38.7)
Benin	2036	44.7 (37.1 to 52.9)	11.8 (8.7 to 15.5)	18.4 (12.7 to 25.9)	2.9 (1.4 to 5.6)	11.5 (8.9 to 15.5)	116.5 (96.7 to 137.8)	30.8 (22.6 to 40.3)	48.0 (33.1 to 67.5)	7.7 (3.6 to 14.7)	29.9 (23.2 to 40.3)

Country	Year	2018 US Dollars per capita					2018 PPP per capita				
		Health spending	Government health spending	Out-of-pocket health spending	Prepaid private health spending	Development assistance for health	Health spending	Government health spending	Out-of-pocket health spending	Prepaid private health spending	Development assistance for health
Benin	2037	45.5 (37.8 to 54.3)	12.1 (8.9 to 15.9)	18.7 (12.9 to 26.4)	3.0 (1.4 to 5.8)	11.6 (8.9 to 15.6)	118.4 (98.5 to 141.4)	31.6 (23.2 to 41.4)	48.7 (33.7 to 68.7)	7.8 (3.7 to 15.0)	30.2 (23.2 to 40.6)
Benin	2038	46.2 (38.1 to 55.5)	12.4 (9.1 to 16.3)	19.0 (13.1 to 26.8)	3.1 (1.5 to 5.9)	11.7 (8.9 to 16.1)	120.4 (99.1 to 144.7)	32.4 (23.7 to 42.4)	49.5 (34.2 to 69.9)	8.0 (3.8 to 15.4)	30.5 (23.3 to 42.0)
Benin	2039	47.0 (38.7 to 57.3)	12.8 (9.3 to 16.8)	19.3 (13.3 to 27.2)	3.2 (1.5 to 6.0)	11.8 (9.0 to 16.2)	122.5 (100.9 to 149.2)	33.2 (24.3 to 43.7)	50.2 (34.7 to 70.9)	8.2 (3.9 to 15.7)	30.8 (23.5 to 42.2)
Benin	2040	47.9 (39.0 to 57.5)	13.1 (9.6 to 17.2)	19.6 (13.5 to 27.6)	3.2 (1.5 to 6.2)	12.0 (9.1 to 16.4)	124.7 (101.6 to 149.9)	34.1 (24.9 to 44.8)	50.9 (35.2 to 71.9)	8.4 (4.0 to 16.1)	31.2 (23.7 to 42.7)
Benin	2041	48.7 (40.0 to 58.9)	13.4 (9.7 to 17.6)	19.8 (13.7 to 28.0)	3.3 (1.6 to 6.3)	12.1 (9.1 to 17.0)	126.8 (104.1 to 153.4)	35.0 (25.4 to 45.9)	51.7 (35.8 to 72.8)	8.6 (4.1 to 16.5)	31.6 (23.7 to 44.4)
Benin	2042	49.6 (40.4 to 59.8)	13.8 (10.0 to 18.1)	20.1 (13.9 to 28.3)	3.4 (1.6 to 6.5)	12.3 (9.1 to 17.7)	129.1 (105.1 to 155.9)	35.9 (26.0 to 47.1)	52.4 (36.3 to 73.8)	8.8 (4.2 to 16.9)	32.0 (23.7 to 46.2)
Benin	2043	50.5 (41.1 to 61.0)	14.1 (10.3 to 18.6)	20.4 (14.2 to 28.7)	3.5 (1.6 to 6.6)	12.5 (9.3 to 18.3)	131.5 (107.0 to 158.9)	36.8 (26.8 to 48.4)	53.2 (36.9 to 74.8)	9.0 (4.3 to 17.3)	32.5 (24.1 to 47.8)
Benin	2044	51.4 (41.6 to 62.2)	14.5 (10.6 to 19.1)	20.7 (14.4 to 29.1)	3.5 (1.7 to 6.8)	12.6 (9.3 to 18.6)	134.0 (108.3 to 162.1)	37.8 (27.6 to 49.8)	54.0 (37.5 to 75.9)	9.2 (4.4 to 17.7)	32.9 (24.2 to 48.5)
Benin	2045	52.4 (42.4 to 63.5)	14.9 (10.9 to 19.6)	21.0 (14.6 to 29.5)	3.6 (1.7 to 6.9)	12.8 (9.3 to 19.4)	136.5 (110.5 to 165.5)	38.8 (28.3 to 51.2)	54.8 (38.0 to 76.9)	9.5 (4.5 to 18.1)	33.4 (24.3 to 50.7)
Benin	2046	53.4 (43.0 to 64.8)	15.3 (11.1 to 20.1)	21.3 (14.8 to 30.0)	3.7 (1.8 to 7.1)	13.0 (9.4 to 19.7)	139.0 (112.0 to 168.7)	39.8 (29.0 to 52.5)	55.6 (38.4 to 78.0)	9.7 (4.6 to 18.5)	34.0 (24.4 to 51.4)
Benin	2047	54.4 (44.1 to 65.9)	15.6 (11.4 to 20.7)	21.7 (14.9 to 30.4)	3.8 (1.8 to 7.3)	13.3 (9.5 to 20.9)	141.6 (114.7 to 171.6)	40.8 (29.7 to 54.0)	56.4 (38.9 to 79.1)	9.9 (4.7 to 18.9)	34.5 (24.8 to 54.4)
Benin	2048	55.4 (44.6 to 68.3)	16.0 (11.6 to 21.3)	22.0 (15.1 to 30.8)	3.9 (1.8 to 7.4)	13.5 (9.7 to 21.6)	144.3 (116.1 to 177.8)	41.8 (30.3 to 55.5)	57.2 (39.4 to 80.3)	10.1 (4.8 to 19.4)	35.2 (25.2 to 56.4)
Benin	2049	56.4 (46.1 to 69.0)	16.5 (11.9 to 21.9)	22.3 (15.4 to 31.3)	4.0 (1.9 to 7.6)	13.7 (9.7 to 22.0)	147.0 (120.1 to 179.6)	42.8 (30.9 to 56.9)	58.1 (40.0 to 81.6)	10.4 (4.9 to 19.8)	35.7 (25.2 to 57.2)
Benin	2050	57.5 (46.5 to 71.4)	16.9 (12.1 to 22.5)	22.6 (15.6 to 31.8)	4.1 (1.9 to 7.8)	14.0 (9.9 to 22.6)	149.9 (121.0 to 186.0)	43.9 (31.6 to 58.5)	58.9 (40.7 to 82.8)	10.6 (5.0 to 20.3)	36.4 (25.7 to 58.8)
Bermuda	1995	7317.6 (5490.1 to 10191.9)	2449.4 (1869.0 to 3130.7)	1791.3 (1277.1 to 2453.6)	3077.0 (1498.4 to 5854.9)	0.0 (0.0 to 0.0)	4729.5 (3548.4 to 6587.2)	1583.1 (1208.0 to 2023.4)	1157.7 (825.4 to 1585.8)	1988.7 (968.4 to 3784.1)	0.0 (0.0 to 0.0)
Bermuda	1996	6971.6 (5254.6 to 9516.7)	2345.6 (1794.4 to 2972.1)	1879.2 (1336.6 to 2538.6)	2746.9 (1346.3 to 5110.8)	0.0 (0.0 to 0.0)	4505.9 (3396.1 to 6150.8)	1516.0 (1159.7 to 1920.9)	1214.5 (863.8 to 1640.8)	1775.4 (870.1 to 3303.2)	0.0 (0.0 to 0.0)
Bermuda	1997	6674.3 (5123.8 to 8729.1)	2269.2 (1758.5 to 2855.2)	2073.0 (1471.4 to 2801.6)	2332.1 (1149.0 to 4220.7)	0.0 (0.0 to 0.0)	4313.7 (3311.6 to 5641.7)	1466.6 (1136.5 to 1845.4)	1339.8 (951.0 to 1810.7)	1507.3 (742.6 to 2727.9)	0.0 (0.0 to 0.0)
Bermuda	1998	6498.2 (5173.9 to 8237.3)	2178.2 (1713.4 to 2711.3)	2382.7 (1720.7 to 3183.0)	1937.2 (963.1 to 3421.0)	0.0 (0.0 to 0.0)	4199.9 (3344.0 to 5323.9)	1407.8 (1107.4 to 1752.4)	1540.0 (1112.1 to 2057.2)	1252.1 (622.5 to 2211.1)	0.0 (0.0 to 0.0)
Bermuda	1999	6640.1 (5372.2 to 8248.5)	2117.0 (1690.8 to 2603.7)	2851.5 (2123.5 to 3741.5)	1671.6 (857.4 to 2872.1)	0.0 (0.0 to 0.0)	4291.6 (3472.2 to 5331.2)	1368.2 (1092.8 to 1682.8)	1843.0 (1372.5 to 2418.2)	1080.4 (554.1 to 1856.3)	0.0 (0.0 to 0.0)
Bermuda	2000	7150.0 (5926.0 to 8725.8)	2135.8 (1744.3 to 2589.7)	3461.9 (2649.8 to 4474.2)	1552.3 (845.0 to 2572.8)	0.0 (0.0 to 0.0)	4621.2 (3830.1 to 5639.6)	1380.4 (1127.4 to 1673.8)	2237.5 (1712.6 to 2891.8)	1003.3 (546.1 to 1662.9)	0.0 (0.0 to 0.0)
Bermuda	2001	7895.8 (6665.9 to 9400.9)	2231.4 (1867.0 to 2649.6)	4139.2 (3277.0 to 5217.0)	1525.2 (862.8 to 2422.8)	0.0 (0.0 to 0.0)	5103.2 (4308.3 to 6076.0)	1442.2 (1206.7 to 1712.5)	2675.2 (2118.0 to 3371.8)	985.8 (557.7 to 1565.9)	0.0 (0.0 to 0.0)

		2018 US Dollars per capita					2018 PPP per capita				
Country	Year	Health spending	Government health spending	Out-of-pocket health spending	Prepaid private health spending	Development assistance for health	Health spending	Government health spending	Out-of-pocket health spending	Prepaid private health spending	Development assistance for health
Bermuda	2002	8347.5 (7222.8 to 9676.0)	2223.6 (1921.2 to 2571.7)	4603.2 (3769.5 to 5575.9)	1520.7 (925.6 to 2343.0)	0.0 (0.0 to 0.0)	5395.1 (4668.2 to 6253.8)	1437.1 (1241.7 to 1662.1)	2975.1 (2436.3 to 3603.8)	982.9 (598.3 to 1514.3)	0.0 (0.0 to 0.0)
Bermuda	2003	9007.2 (7989.5 to 10181.1)	2315.2 (2070.8 to 2587.0)	5078.9 (4339.9 to 5927.6)	1613.1 (1049.5 to 2347.4)	0.0 (0.0 to 0.0)	5821.5 (5163.8 to 6580.2)	1496.4 (1338.4 to 1672.0)	3282.6 (2805.0 to 3831.1)	1042.6 (678.3 to 1517.1)	0.0 (0.0 to 0.0)
Bermuda	2004	9573.8 (8662.7 to 10575.9)	2396.7 (2195.8 to 2619.7)	5456.2 (4793.2 to 6155.9)	1720.9 (1199.3 to 2372.1)	0.0 (0.0 to 0.0)	6187.7 (5598.9 to 6835.4)	1549.0 (1419.2 to 1693.1)	3526.5 (3097.9 to 3978.7)	1112.2 (775.1 to 1533.1)	0.0 (0.0 to 0.0)
Bermuda	2005	9438.1 (8676.5 to 10254.4)	2433.4 (2267.4 to 2598.0)	5048.4 (4505.9 to 5616.5)	1956.4 (1457.5 to 2550.3)	0.0 (0.0 to 0.0)	6100.0 (5607.8 to 6627.6)	1572.7 (1465.5 to 1679.1)	3262.8 (2912.2 to 3630.1)	1264.5 (942.0 to 1648.3)	0.0 (0.0 to 0.0)
Bermuda	2006	9176.9 (8472.4 to 9957.8)	2516.9 (2357.8 to 2676.1)	4112.8 (3646.3 to 4574.5)	2547.1 (2070.0 to 3087.3)	0.0 (0.0 to 0.0)	5931.2 (5475.9 to 6435.9)	1626.7 (1523.9 to 1729.6)	2658.2 (2356.6 to 2956.6)	1646.2 (1337.9 to 1995.4)	0.0 (0.0 to 0.0)
Bermuda	2007	9097.3 (8382.5 to 9884.6)	2552.0 (2397.4 to 2703.1)	2561.2 (2211.3 to 2942.9)	3984.0 (3422.2 to 4637.4)	0.0 (0.0 to 0.0)	5879.8 (5417.8 to 6388.6)	1649.4 (1549.5 to 1747.1)	1655.4 (1429.2 to 1902.0)	2575.0 (2211.8 to 2997.3)	0.0 (0.0 to 0.0)
Bermuda	2008	9829.3 (9155.6 to 10540.6)	2710.2 (2553.8 to 2872.4)	2041.5 (1723.5 to 2389.2)	5077.5 (4471.1 to 5732.0)	0.0 (0.0 to 0.0)	6352.8 (5917.5 to 6812.6)	1751.6 (1650.6 to 1856.5)	1319.5 (1114.0 to 1544.2)	3281.7 (2889.8 to 3704.7)	0.0 (0.0 to 0.0)
Bermuda	2009	10447.0 (9736.4 to 11138.6)	2859.0 (2714.7 to 3021.6)	1790.1 (1482.3 to 2124.5)	5798.0 (5203.6 to 6420.0)	0.0 (0.0 to 0.0)	6752.1 (6292.8 to 7199.1)	1847.8 (1754.5 to 1952.9)	1157.0 (958.0 to 1373.1)	3747.3 (3363.2 to 4149.4)	0.0 (0.0 to 0.0)
Bermuda	2010	11340.5 (10603.1 to 12039.9)	3221.5 (3074.4 to 3396.6)	1711.2 (1398.5 to 2073.2)	6407.9 (5787.9 to 7051.3)	0.0 (0.0 to 0.0)	7329.6 (6853.0 to 7781.6)	2082.1 (1987.1 to 2195.3)	1106.0 (903.9 to 1340.0)	4141.5 (3740.8 to 4557.4)	0.0 (0.0 to 0.0)
Bermuda	2011	11907.0 (11168.9 to 12724.7)	3496.6 (3332.9 to 3666.3)	1650.9 (1329.7 to 2017.6)	6759.4 (6117.6 to 7444.2)	0.0 (0.0 to 0.0)	7695.7 (7218.6 to 8224.2)	2259.9 (2154.1 to 2369.6)	1067.0 (859.4 to 1304.0)	4368.7 (3953.9 to 4811.3)	0.0 (0.0 to 0.0)
Bermuda	2012	11627.9 (10906.4 to 12435.5)	3451.4 (3285.6 to 3615.3)	1544.7 (1230.8 to 1909.8)	6631.8 (6021.5 to 7314.2)	0.0 (0.0 to 0.0)	7515.3 (7049.0 to 8037.3)	2230.7 (2123.5 to 2336.6)	998.3 (795.5 to 1234.3)	4286.2 (3891.8 to 4727.3)	0.0 (0.0 to 0.0)
Bermuda	2013	11427.9 (10723.4 to 12185.5)	3387.8 (3226.6 to 3553.4)	1389.3 (1086.4 to 1742.8)	6650.8 (6055.9 to 7312.8)	0.0 (0.0 to 0.0)	7386.0 (6930.7 to 7875.7)	2189.6 (2085.4 to 2296.6)	897.9 (702.2 to 1126.4)	4298.5 (3914.0 to 4726.4)	0.0 (0.0 to 0.0)
Bermuda	2014	11285.8 (10558.9 to 12077.3)	3319.7 (3137.8 to 3492.7)	1226.7 (950.9 to 1570.5)	6739.4 (6135.7 to 7411.4)	0.0 (0.0 to 0.0)	7294.2 (6824.4 to 7805.8)	2145.6 (2028.0 to 2257.4)	792.8 (614.6 to 1015.0)	4355.8 (3965.6 to 4790.1)	0.0 (0.0 to 0.0)
Bermuda	2015	11116.3 (10217.7 to 12047.9)	3211.4 (2992.0 to 3436.7)	1118.6 (850.8 to 1442.6)	6786.2 (5960.1 to 7652.5)	0.0 (0.0 to 0.0)	7184.7 (6603.9 to 7786.8)	2075.6 (1933.8 to 2221.2)	723.0 (549.9 to 932.4)	4386.1 (3852.1 to 4945.9)	0.0 (0.0 to 0.0)
Bermuda	2016	10802.2 (9469.4 to 12351.7)	3134.5 (2839.8 to 3464.6)	1097.3 (829.2 to 1430.6)	6570.4 (5361.2 to 8035.7)	0.0 (0.0 to 0.0)	6981.7 (6120.3 to 7983.1)	2025.9 (1835.4 to 2239.2)	709.2 (535.9 to 924.7)	4246.6 (3465.1 to 5193.6)	0.0 (0.0 to 0.0)
Bermuda	2017	11094.8 (9790.7 to 12639.0)	3211.5 (2915.3 to 3549.0)	1105.7 (834.3 to 1453.5)	6777.5 (5521.4 to 8289.6)	0.0 (0.0 to 0.0)	7170.8 (6327.9 to 8168.8)	2075.7 (1884.2 to 2293.8)	714.7 (539.2 to 939.4)	4380.4 (3568.6 to 5357.7)	0.0 (0.0 to 0.0)
Bermuda	2018	11195.8 (9833.6 to 12743.4)	3262.1 (2955.2 to 3606.7)	1103.8 (833.2 to 1448.4)	6829.9 (5570.6 to 8350.8)	0.0 (0.0 to 0.0)	7236.0 (6355.7 to 8236.3)	2108.4 (1910.0 to 2331.1)	713.4 (538.5 to 936.1)	4414.3 (3600.4 to 5397.3)	0.0 (0.0 to 0.0)
Bermuda	2019	11277.2 (9923.7 to 12837.7)	3305.2 (2986.2 to 3659.5)	1099.8 (830.3 to 1442.9)	6872.2 (5607.6 to 8403.0)	0.0 (0.0 to 0.0)	7288.6 (6413.9 to 8297.2)	2136.2 (1930.1 to 2365.2)	710.8 (536.6 to 932.6)	4441.6 (3624.3 to 5431.0)	0.0 (0.0 to 0.0)
Bermuda	2020	11355.3 (9954.2 to 12946.1)	3348.5 (3020.5 to 3713.7)	1095.5 (824.9 to 1439.0)	6911.2 (5639.3 to 8456.4)	0.0 (0.0 to 0.0)	7339.1 (6433.6 to 8367.3)	2164.2 (1952.2 to 2400.3)	708.1 (533.1 to 930.0)	4466.8 (3644.8 to 5465.5)	0.0 (0.0 to 0.0)
Bermuda	2021	11424.5 (10049.1 to 13038.2)	3389.2 (3060.3 to 3771.3)	1090.6 (821.2 to 1431.4)	6944.7 (5669.1 to 8506.5)	0.0 (0.0 to 0.0)	7383.8 (6494.9 to 8426.8)	2190.5 (1977.9 to 2437.5)	704.9 (530.7 to 925.2)	4488.5 (3664.0 to 5497.9)	0.0 (0.0 to 0.0)
Bermuda	2022	11489.3 (10161.0 to 13106.8)	3429.1 (3085.6 to 3807.7)	1085.1 (817.0 to 1422.1)	6975.1 (5697.5 to 8545.7)	0.0 (0.0 to 0.0)	7425.7 (6567.2 to 8471.2)	2216.3 (1994.3 to 2461.0)	701.3 (528.0 to 919.1)	4508.1 (3682.4 to 5523.2)	0.0 (0.0 to 0.0)

Country	Year	2018 US Dollars per capita					2018 PPP per capita				
		Health spending	Government health spending	Out-of-pocket health spending	Prepaid private health spending	Development assistance for health	Health spending	Government health spending	Out-of-pocket health spending	Prepaid private health spending	Development assistance for health
Bermuda	2023	11555.6 (10163.7 to 13100.5)	3470.5 (3109.9 to 3867.5)	1079.5 (812.8 to 1414.0)	7005.6 (5725.9 to 8586.9)	0.0 (0.0 to 0.0)	7468.6 (6569.0 to 8467.1)	2243.0 (2010.0 to 2499.6)	697.7 (525.3 to 913.9)	4527.8 (3700.8 to 5549.9)	0.0 (0.0 to 0.0)
Bermuda	2024	11608.8 (10195.3 to 13263.5)	3508.2 (3133.2 to 3911.7)	1072.7 (807.8 to 1405.8)	7027.9 (5748.0 to 8619.8)	0.0 (0.0 to 0.0)	7503.0 (6589.4 to 8572.4)	2267.4 (2025.0 to 2528.2)	693.3 (522.1 to 908.6)	4542.3 (3715.0 to 5571.1)	0.0 (0.0 to 0.0)
Bermuda	2025	11657.5 (10181.5 to 13282.7)	3544.5 (3161.3 to 3961.7)	1065.6 (802.5 to 1397.2)	7047.4 (5767.3 to 8634.2)	0.0 (0.0 to 0.0)	7534.4 (6580.5 to 8584.8)	2290.9 (2043.2 to 2560.5)	688.7 (518.6 to 903.1)	4554.9 (3727.5 to 5580.5)	0.0 (0.0 to 0.0)
Bermuda	2026	11692.4 (10263.7 to 13397.6)	3575.8 (3167.9 to 4014.4)	1057.5 (796.5 to 1387.3)	7059.1 (5780.3 to 8652.7)	0.0 (0.0 to 0.0)	7557.0 (6633.6 to 8659.1)	2311.1 (2047.5 to 2594.6)	683.5 (514.8 to 896.7)	4562.4 (3735.9 to 5592.4)	0.0 (0.0 to 0.0)
Bermuda	2027	11707.2 (10269.5 to 13295.3)	3600.5 (3180.7 to 4047.8)	1047.7 (789.2 to 1374.2)	7059.0 (5769.6 to 8626.8)	0.0 (0.0 to 0.0)	7566.6 (6637.4 to 8593.0)	2327.1 (2055.7 to 2616.2)	677.2 (510.1 to 888.2)	4562.3 (3729.0 to 5575.6)	0.0 (0.0 to 0.0)
Bermuda	2028	11695.6 (10228.7 to 13307.9)	3615.6 (3170.8 to 4090.8)	1036.2 (781.1 to 1358.7)	7043.9 (5744.9 to 8609.5)	0.0 (0.0 to 0.0)	7559.1 (6611.0 to 8601.1)	2336.8 (2049.4 to 2644.0)	669.7 (504.8 to 878.1)	4552.6 (3713.0 to 5564.4)	0.0 (0.0 to 0.0)
Bermuda	2029	11694.6 (10241.9 to 13316.2)	3631.6 (3179.0 to 4108.7)	1026.3 (773.7 to 1345.4)	7036.8 (5727.0 to 8608.4)	0.0 (0.0 to 0.0)	7558.4 (6619.5 to 8606.5)	2347.1 (2054.6 to 2655.5)	663.3 (500.0 to 869.5)	4548.0 (3701.5 to 5563.7)	0.0 (0.0 to 0.0)
Bermuda	2030	11717.9 (10327.0 to 13300.3)	3652.2 (3202.0 to 4163.5)	1019.3 (768.5 to 1335.9)	7046.4 (5733.7 to 8628.0)	0.0 (0.0 to 0.0)	7573.5 (6674.5 to 8596.2)	2360.5 (2069.5 to 2690.9)	658.8 (496.7 to 863.4)	4554.2 (3705.8 to 5576.4)	0.0 (0.0 to 0.0)
Bermuda	2031	11764.0 (10288.8 to 13342.9)	3676.7 (3199.9 to 4201.6)	1015.2 (765.5 to 1330.2)	7072.2 (5760.2 to 8668.0)	0.0 (0.0 to 0.0)	7603.3 (6649.9 to 8623.8)	2376.3 (2068.1 to 2715.5)	656.1 (494.8 to 859.7)	4570.9 (3722.9 to 5602.3)	0.0 (0.0 to 0.0)
Bermuda	2032	11820.3 (10290.5 to 13437.7)	3700.5 (3206.1 to 4216.0)	1012.9 (763.9 to 1326.9)	7106.8 (5774.3 to 8719.4)	0.0 (0.0 to 0.0)	7639.6 (6651.0 to 8685.0)	2391.7 (2072.2 to 2724.9)	654.7 (493.7 to 857.6)	4593.3 (3732.0 to 5635.5)	0.0 (0.0 to 0.0)
Bermuda	2033	11916.5 (10446.0 to 13597.1)	3732.9 (3230.6 to 4280.0)	1015.0 (765.6 to 1329.3)	7168.6 (5812.2 to 8803.0)	0.0 (0.0 to 0.0)	7701.9 (6751.4 to 8788.0)	2412.7 (2088.0 to 2766.3)	656.0 (494.8 to 859.1)	4633.2 (3756.6 to 5689.5)	0.0 (0.0 to 0.0)
Bermuda	2034	12032.0 (10558.1 to 13763.4)	3768.4 (3262.0 to 4318.5)	1019.4 (769.0 to 1334.6)	7244.1 (5872.3 to 8889.8)	0.0 (0.0 to 0.0)	7776.5 (6823.9 to 8895.5)	2435.6 (2108.3 to 2791.1)	658.9 (497.0 to 862.6)	4682.0 (3795.4 to 5745.7)	0.0 (0.0 to 0.0)
Bermuda	2035	12168.2 (10625.6 to 13945.4)	3807.8 (3302.5 to 4370.9)	1026.1 (774.1 to 1343.0)	7334.3 (5944.1 to 8988.6)	0.0 (0.0 to 0.0)	7864.5 (6867.5 to 9013.1)	2461.1 (2134.5 to 2825.0)	663.2 (500.3 to 868.0)	4740.3 (3841.8 to 5809.5)	0.0 (0.0 to 0.0)
Bermuda	2036	12314.8 (10765.5 to 14034.3)	3848.8 (3336.8 to 4423.4)	1033.9 (779.6 to 1353.8)	7432.1 (6022.0 to 9123.3)	0.0 (0.0 to 0.0)	7959.3 (6957.9 to 9070.6)	2487.5 (2156.6 to 2858.9)	668.2 (503.9 to 875.0)	4803.5 (3892.1 to 5896.5)	0.0 (0.0 to 0.0)
Bermuda	2037	12482.6 (10816.2 to 14283.7)	3894.6 (3389.5 to 4476.2)	1043.8 (786.2 to 1368.1)	7544.2 (6107.0 to 9260.4)	0.0 (0.0 to 0.0)	8067.8 (6990.7 to 9231.8)	2517.1 (2190.7 to 2893.0)	674.7 (508.1 to 884.2)	4876.0 (3947.1 to 5985.2)	0.0 (0.0 to 0.0)
Bermuda	2038	12666.6 (11005.9 to 14407.7)	3943.7 (3425.2 to 4540.1)	1055.4 (793.8 to 1384.5)	7667.5 (6202.0 to 9417.8)	0.0 (0.0 to 0.0)	8186.6 (7113.3 to 9312.0)	2548.9 (2213.8 to 2934.4)	682.1 (513.1 to 894.8)	4955.6 (4008.5 to 6086.9)	0.0 (0.0 to 0.0)
Bermuda	2039	12868.6 (11151.4 to 14751.1)	3997.3 (3477.8 to 4603.6)	1068.5 (803.0 to 1403.3)	7802.8 (6302.1 to 9584.2)	0.0 (0.0 to 0.0)	8317.2 (7207.3 to 9533.9)	2583.5 (2247.8 to 2975.4)	690.6 (519.0 to 907.0)	5043.1 (4073.1 to 6194.4)	0.0 (0.0 to 0.0)
Bermuda	2040	13074.2 (11391.0 to 14981.8)	4050.4 (3528.9 to 4672.7)	1082.2 (813.1 to 1422.1)	7941.7 (6402.4 to 9768.1)	0.0 (0.0 to 0.0)	8450.1 (7362.2 to 9683.0)	2617.8 (2280.8 to 3020.0)	699.4 (525.5 to 919.1)	5132.8 (4138.0 to 6313.3)	0.0 (0.0 to 0.0)
Bermuda	2041	13299.1 (11615.5 to 15317.9)	4108.3 (3565.5 to 4738.8)	1097.5 (824.3 to 1442.0)	8093.3 (6512.3 to 9958.0)	0.0 (0.0 to 0.0)	8595.4 (7507.3 to 9900.2)	2655.2 (2304.4 to 3062.8)	709.3 (532.8 to 932.0)	5230.9 (4209.0 to 6436.0)	0.0 (0.0 to 0.0)
Bermuda	2042	13529.7 (11705.0 to 15447.0)	4166.3 (3598.4 to 4806.0)	1113.5 (836.1 to 1462.7)	8249.9 (6625.4 to 10169.0)	0.0 (0.0 to 0.0)	8744.5 (7565.2 to 9983.7)	2692.8 (2325.7 to 3106.2)	719.7 (540.4 to 945.4)	5332.1 (4282.1 to 6572.4)	0.0 (0.0 to 0.0)
Bermuda	2043	13787.3 (11981.7 to 15844.4)	4230.6 (3651.5 to 4882.7)	1132.0 (849.8 to 1486.8)	8424.7 (6753.2 to 10411.9)	0.0 (0.0 to 0.0)	8911.0 (7744.0 to 10240.5)	2734.3 (2360.1 to 3155.8)	731.6 (549.2 to 960.9)	5445.1 (4364.7 to 6729.4)	0.0 (0.0 to 0.0)

Country	Year	2018 US Dollars per capita					2018 PPP per capita				
		Health spending	Government health spending	Out-of-pocket health spending	Prepaid private health spending	Development assistance for health	Health spending	Government health spending	Out-of-pocket health spending	Prepaid private health spending	Development assistance for health
Bermuda	2044	14041.2 (12211.6 to 16173.5)	4294.6 (3714.8 to 4968.2)	1149.7 (862.4 to 1510.0)	8596.9 (6878.7 to 10629.9)	0.0 (0.0 to 0.0)	9075.1 (7892.6 to 10453.2)	2775.7 (2400.9 to 3211.0)	743.1 (557.4 to 975.9)	5556.3 (4445.8 to 6870.3)	0.0 (0.0 to 0.0)
Bermuda	2045	14302.5 (12488.0 to 16461.7)	4359.2 (3762.9 to 5055.5)	1168.2 (875.2 to 1535.3)	8775.1 (7008.1 to 10857.9)	0.0 (0.0 to 0.0)	9244.0 (8071.2 to 10639.5)	2817.4 (2432.0 to 3267.4)	755.0 (565.7 to 992.3)	5671.5 (4529.5 to 7017.6)	0.0 (0.0 to 0.0)
Bermuda	2046	14553.4 (12620.4 to 16714.4)	4421.2 (3821.0 to 5149.1)	1185.5 (887.1 to 1558.9)	8946.7 (7140.3 to 11103.5)	0.0 (0.0 to 0.0)	9406.1 (8156.8 to 10802.8)	2857.5 (2469.6 to 3328.0)	766.2 (573.4 to 1007.6)	5782.4 (4614.9 to 7176.4)	0.0 (0.0 to 0.0)
Bermuda	2047	14810.5 (12797.9 to 17174.4)	4485.4 (3856.5 to 5219.9)	1203.0 (899.6 to 1582.9)	9122.1 (7261.5 to 11333.0)	0.0 (0.0 to 0.0)	9572.3 (8271.5 to 11100.1)	2899.0 (2492.5 to 3373.7)	777.5 (581.4 to 1023.1)	5895.8 (4693.2 to 7324.7)	0.0 (0.0 to 0.0)
Bermuda	2048	15063.5 (13058.6 to 17396.0)	4549.2 (3899.5 to 5309.7)	1219.7 (912.5 to 1605.9)	9294.6 (7394.7 to 11553.5)	0.0 (0.0 to 0.0)	9735.8 (8440.0 to 11243.3)	2940.2 (2520.3 to 3431.7)	788.3 (589.8 to 1037.9)	6007.3 (4779.3 to 7467.2)	0.0 (0.0 to 0.0)
Bermuda	2049	15314.6 (13139.3 to 17764.8)	4613.8 (3953.2 to 5406.0)	1235.7 (924.9 to 1626.2)	9465.2 (7527.9 to 11768.0)	0.0 (0.0 to 0.0)	9898.1 (8492.2 to 11481.7)	2982.0 (2555.0 to 3494.0)	798.6 (597.8 to 1051.1)	6117.5 (4865.4 to 7605.9)	0.0 (0.0 to 0.0)
Bermuda	2050	15572.0 (13383.5 to 18169.5)	4680.5 (3999.3 to 5478.8)	1251.9 (937.5 to 1648.7)	9639.6 (7662.1 to 11990.4)	0.0 (0.0 to 0.0)	10064.5 (8650.0 to 11743.3)	3025.1 (2584.8 to 3541.0)	809.2 (605.9 to 1065.6)	6230.2 (4952.1 to 7749.6)	0.0 (0.0 to 0.0)
Bhutan	1995	48.7 (39.9 to 58.8)	38.1 (29.6 to 48.0)	8.5 (6.1 to 11.6)	0.4 (0.2 to 0.8)	1.6 (1.6 to 1.6)	149.9 (122.9 to 181.1)	117.5 (91.3 to 147.7)	26.1 (18.8 to 35.7)	1.3 (0.6 to 2.5)	5.0 (5.0 to 5.0)
Bhutan	1996	50.1 (41.3 to 60.4)	40.2 (31.4 to 50.5)	9.1 (6.5 to 12.6)	0.4 (0.2 to 0.8)	0.4 (0.4 to 0.4)	154.4 (127.1 to 185.8)	123.7 (96.6 to 155.5)	28.0 (20.0 to 38.8)	1.4 (0.7 to 2.6)	1.3 (1.3 to 1.3)
Bhutan	1997	72.2 (63.2 to 82.9)	40.9 (32.0 to 51.4)	9.7 (7.0 to 13.3)	0.5 (0.2 to 0.8)	21.2 (21.2 to 21.2)	222.3 (194.7 to 255.3)	125.8 (98.6 to 158.2)	30.0 (21.4 to 41.0)	1.4 (0.7 to 2.6)	65.1 (65.1 to 65.1)
Bhutan	1998	64.7 (55.7 to 75.8)	41.0 (32.7 to 51.6)	9.8 (6.9 to 13.5)	0.5 (0.2 to 0.8)	13.4 (13.4 to 13.4)	199.1 (171.6 to 233.3)	126.4 (100.7 to 158.9)	30.1 (21.2 to 41.5)	1.4 (0.7 to 2.6)	41.3 (41.3 to 41.3)
Bhutan	1999	52.5 (43.7 to 63.4)	41.5 (33.3 to 52.2)	9.1 (6.5 to 12.4)	0.5 (0.2 to 0.8)	1.4 (1.4 to 1.4)	161.8 (134.6 to 195.3)	127.9 (102.4 to 160.6)	28.1 (19.9 to 38.3)	1.4 (0.7 to 2.6)	4.4 (4.4 to 4.4)
Bhutan	2000	55.5 (47.0 to 65.9)	41.3 (33.1 to 51.9)	7.4 (5.3 to 10.0)	0.4 (0.2 to 0.8)	6.3 (6.3 to 6.3)	170.9 (144.7 to 202.8)	127.3 (101.8 to 159.8)	22.8 (16.4 to 30.9)	1.4 (0.6 to 2.6)	19.5 (19.5 to 19.5)
Bhutan	2001	52.6 (44.4 to 63.5)	40.9 (32.8 to 51.0)	6.7 (4.7 to 9.1)	0.5 (0.2 to 0.8)	4.4 (4.4 to 4.4)	161.8 (136.6 to 195.5)	126.0 (101.1 to 156.9)	20.7 (14.6 to 28.0)	1.4 (0.7 to 2.6)	13.7 (13.7 to 13.7)
Bhutan	2002	53.8 (45.4 to 64.5)	41.4 (33.0 to 51.7)	7.4 (5.3 to 10.1)	0.4 (0.2 to 0.7)	4.6 (4.6 to 4.6)	165.7 (139.9 to 198.6)	127.6 (101.7 to 159.2)	22.8 (16.3 to 31.1)	1.2 (0.5 to 2.1)	14.2 (14.2 to 14.2)
Bhutan	2003	58.7 (50.2 to 69.4)	40.7 (32.7 to 50.6)	8.8 (6.3 to 11.9)	0.3 (0.1 to 0.5)	8.9 (8.9 to 8.9)	180.8 (154.5 to 213.6)	125.4 (100.6 to 155.9)	27.2 (19.4 to 36.8)	0.8 (0.4 to 1.5)	27.5 (27.5 to 27.5)
Bhutan	2004	57.4 (48.9 to 67.8)	40.7 (32.7 to 50.6)	9.6 (6.9 to 12.9)	0.2 (0.1 to 0.4)	6.9 (6.9 to 6.9)	176.7 (150.6 to 208.7)	125.4 (100.7 to 155.8)	29.5 (21.2 to 39.8)	0.7 (0.3 to 1.3)	21.1 (21.1 to 21.1)
Bhutan	2005	62.7 (54.0 to 72.3)	40.0 (32.0 to 49.9)	9.8 (7.0 to 13.2)	0.2 (0.1 to 0.3)	12.7 (12.7 to 12.7)	192.9 (166.2 to 222.7)	123.1 (98.4 to 153.7)	30.1 (21.4 to 40.7)	0.6 (0.3 to 1.1)	39.2 (39.2 to 39.2)
Bhutan	2006	62.0 (53.1 to 71.4)	40.0 (32.1 to 49.2)	9.8 (7.1 to 13.3)	0.2 (0.1 to 0.3)	12.0 (12.0 to 12.0)	190.8 (163.4 to 219.8)	123.2 (98.8 to 151.6)	30.2 (21.8 to 41.0)	0.5 (0.3 to 0.9)	36.9 (36.9 to 36.9)
Bhutan	2007	63.5 (53.8 to 73.7)	43.9 (35.0 to 54.2)	10.2 (7.3 to 13.8)	0.2 (0.1 to 0.3)	9.2 (9.2 to 9.2)	195.4 (165.5 to 227.0)	135.2 (107.7 to 166.9)	31.4 (22.6 to 42.5)	0.6 (0.3 to 1.0)	28.3 (28.3 to 28.3)
Bhutan	2008	60.8 (51.4 to 71.4)	43.9 (35.2 to 54.6)	10.2 (7.4 to 13.9)	0.2 (0.1 to 0.4)	6.4 (6.4 to 6.4)	187.1 (158.2 to 219.8)	135.3 (108.4 to 168.0)	31.4 (22.8 to 42.9)	0.7 (0.3 to 1.3)	19.7 (19.7 to 19.7)

		2018 US Dollars per capita					2018 PPP per capita				
Country	Year	Health spending	Government health spending	Out-of-pocket health spending	Prepaid private health spending	Development assistance for health	Health spending	Government health spending	Out-of-pocket health spending	Prepaid private health spending	Development assistance for health
Bhutan	2009	61.6 (51.8 to 73.4)	46.0 (37.1 to 57.3)	10.8 (7.7 to 14.8)	0.3 (0.1 to 0.5)	4.5 (4.5 to 4.5)	189.6 (159.4 to 226.0)	141.5 (114.1 to 176.3)	33.2 (23.7 to 45.4)	0.9 (0.4 to 1.7)	14.0 (14.0 to 14.0)
Bhutan	2010	64.7 (54.9 to 77.3)	48.5 (39.1 to 60.7)	11.5 (8.2 to 15.8)	0.3 (0.2 to 0.7)	4.4 (4.4 to 4.4)	199.4 (169.0 to 238.1)	149.2 (120.3 to 187.1)	35.4 (25.1 to 48.6)	1.1 (0.5 to 2.0)	13.7 (13.7 to 13.7)
Bhutan	2011	66.4 (56.0 to 79.4)	50.0 (40.2 to 62.6)	12.3 (8.8 to 16.9)	0.4 (0.2 to 0.8)	3.8 (3.8 to 3.8)	204.5 (172.5 to 244.4)	153.8 (123.6 to 192.8)	37.8 (27.1 to 51.9)	1.4 (0.6 to 2.6)	11.6 (11.6 to 11.6)
Bhutan	2012	71.5 (59.9 to 85.0)	51.4 (40.9 to 63.7)	13.9 (9.8 to 19.4)	0.6 (0.3 to 1.2)	5.6 (5.6 to 5.6)	220.2 (184.4 to 261.7)	158.1 (125.9 to 196.0)	42.8 (30.1 to 59.7)	2.0 (0.9 to 3.8)	17.2 (17.2 to 17.2)
Bhutan	2013	70.9 (58.7 to 84.5)	52.0 (41.7 to 64.2)	14.8 (10.4 to 20.7)	0.8 (0.4 to 1.5)	3.3 (3.3 to 3.3)	218.2 (180.8 to 260.1)	160.1 (128.4 to 197.8)	45.6 (32.1 to 63.9)	2.3 (1.1 to 4.6)	10.2 (10.2 to 10.2)
Bhutan	2014	74.1 (61.3 to 88.0)	53.9 (42.8 to 66.9)	15.5 (10.9 to 21.5)	0.8 (0.4 to 1.6)	3.9 (3.9 to 3.9)	228.1 (188.9 to 270.9)	165.9 (131.8 to 205.9)	47.6 (33.6 to 66.1)	2.5 (1.2 to 4.9)	12.0 (12.0 to 12.0)
Bhutan	2015	81.3 (68.2 to 96.3)	58.0 (46.0 to 72.1)	16.2 (11.7 to 22.4)	0.9 (0.4 to 1.6)	6.2 (6.2 to 6.2)	250.3 (210.0 to 296.4)	178.7 (141.5 to 222.0)	49.8 (35.9 to 68.9)	2.7 (1.2 to 5.1)	19.2 (19.2 to 19.2)
Bhutan	2016	83.7 (69.3 to 99.5)	60.9 (48.0 to 76.0)	16.7 (11.8 to 23.1)	1.0 (0.4 to 1.9)	5.1 (5.1 to 5.1)	257.7 (213.3 to 306.4)	187.6 (147.9 to 234.1)	51.5 (36.3 to 71.1)	3.0 (1.4 to 5.7)	15.6 (15.6 to 15.6)
Bhutan	2017	90.9 (74.7 to 109.4)	69.2 (54.4 to 86.1)	17.6 (12.4 to 24.2)	1.0 (0.5 to 2.0)	3.1 (3.1 to 3.1)	279.8 (230.1 to 336.8)	213.0 (167.5 to 265.1)	54.1 (38.2 to 74.6)	3.1 (1.4 to 6.1)	9.6 (9.6 to 9.6)
Bhutan	2018	94.5 (77.5 to 113.1)	72.3 (57.0 to 90.0)	18.2 (12.8 to 25.2)	1.1 (0.5 to 2.1)	2.9 (2.9 to 2.9)	290.8 (238.6 to 348.4)	222.6 (175.4 to 277.1)	55.9 (39.5 to 77.5)	3.3 (1.5 to 6.3)	9.0 (9.0 to 9.0)
Bhutan	2019	98.0 (80.3 to 117.4)	75.2 (59.3 to 94.2)	18.8 (13.3 to 26.0)	1.1 (0.5 to 2.1)	2.9 (2.7 to 3.1)	301.8 (247.3 to 361.4)	231.6 (182.5 to 290.1)	57.8 (40.8 to 80.1)	3.4 (1.6 to 6.6)	9.0 (8.4 to 9.5)
Bhutan	2020	101.6 (83.3 to 121.5)	78.1 (61.5 to 97.7)	19.4 (13.5 to 26.7)	1.1 (0.5 to 2.2)	3.0 (2.7 to 3.2)	312.8 (256.6 to 374.2)	240.5 (189.4 to 300.8)	59.7 (41.7 to 82.3)	3.5 (1.6 to 6.9)	9.1 (8.3 to 9.8)
Bhutan	2021	105.1 (85.9 to 126.0)	81.0 (63.7 to 101.1)	20.0 (13.9 to 27.6)	1.2 (0.5 to 2.3)	3.0 (2.7 to 3.3)	323.7 (264.6 to 388.0)	249.3 (196.2 to 311.4)	61.6 (42.9 to 84.9)	3.6 (1.7 to 7.1)	9.2 (8.3 to 10.2)
Bhutan	2022	108.8 (88.8 to 130.1)	83.9 (65.9 to 104.7)	20.6 (14.4 to 28.6)	1.2 (0.6 to 2.4)	3.1 (2.7 to 3.4)	335.0 (273.5 to 400.6)	258.2 (203.0 to 322.4)	63.6 (44.4 to 88.1)	3.8 (1.7 to 7.3)	9.4 (8.3 to 10.5)
Bhutan	2023	112.5 (91.6 to 134.6)	86.8 (68.1 to 108.6)	21.2 (14.8 to 29.4)	1.3 (0.6 to 2.5)	3.1 (2.7 to 3.5)	346.3 (281.9 to 414.5)	267.4 (209.7 to 334.4)	65.4 (45.6 to 90.6)	3.9 (1.8 to 7.6)	9.6 (8.3 to 10.9)
Bhutan	2024	116.2 (94.8 to 139.2)	89.8 (70.3 to 112.3)	21.9 (15.2 to 30.3)	1.3 (0.6 to 2.6)	3.2 (2.7 to 3.6)	357.8 (291.7 to 428.7)	276.6 (216.5 to 345.9)	67.3 (46.9 to 93.3)	4.1 (1.9 to 7.9)	9.8 (8.4 to 11.2)
Bhutan	2025	119.9 (98.2 to 144.0)	92.8 (73.0 to 115.8)	22.5 (15.7 to 31.2)	1.4 (0.6 to 2.7)	3.2 (2.8 to 3.8)	369.3 (302.4 to 443.3)	285.9 (224.9 to 356.5)	69.3 (48.3 to 96.1)	4.2 (2.0 to 8.2)	9.9 (8.5 to 11.6)
Bhutan	2026	123.9 (101.4 to 149.0)	96.0 (75.5 to 119.7)	23.2 (16.1 to 32.3)	1.4 (0.7 to 2.8)	3.3 (2.8 to 3.9)	381.4 (312.3 to 458.9)	295.6 (232.3 to 368.5)	71.4 (49.7 to 99.4)	4.4 (2.0 to 8.5)	10.1 (8.5 to 11.9)
Bhutan	2027	127.9 (104.6 to 153.7)	99.2 (77.9 to 123.7)	23.9 (16.6 to 33.3)	1.5 (0.7 to 2.9)	3.3 (2.8 to 4.0)	393.9 (322.1 to 473.3)	305.6 (239.8 to 381.0)	73.5 (51.2 to 102.5)	4.5 (2.1 to 8.8)	10.3 (8.6 to 12.2)
Bhutan	2028	132.1 (107.9 to 158.8)	102.6 (80.5 to 127.9)	24.6 (17.1 to 34.3)	1.5 (0.7 to 3.0)	3.4 (2.9 to 4.1)	406.7 (332.1 to 488.8)	315.8 (247.8 to 393.9)	75.6 (52.7 to 105.5)	4.7 (2.2 to 9.1)	10.5 (8.8 to 12.7)
Bhutan	2029	136.4 (111.7 to 164.6)	106.0 (83.6 to 132.2)	25.3 (17.6 to 35.2)	1.6 (0.7 to 3.1)	3.5 (2.9 to 4.3)	419.9 (344.0 to 506.7)	326.5 (257.5 to 407.2)	77.8 (54.2 to 108.5)	4.9 (2.3 to 9.4)	10.7 (8.8 to 13.1)

Country	Year	2018 US Dollars per capita					2018 PPP per capita				
		Health spending	Government health spending	Out-of-pocket health spending	Prepaid private health spending	Development assistance for health	Health spending	Government health spending	Out-of-pocket health spending	Prepaid private health spending	Development assistance for health
Bhutan	2030	140.6 (115.1 to 169.9)	109.5 (86.2 to 137.4)	26.0 (18.1 to 36.2)	1.6 (0.8 to 3.2)	3.6 (2.9 to 4.4)	433.0 (354.4 to 523.2)	337.0 (265.3 to 423.1)	80.0 (55.7 to 111.4)	5.0 (2.3 to 9.8)	10.9 (9.0 to 13.4)
Bhutan	2031	145.0 (118.6 to 174.7)	113.0 (88.6 to 142.2)	26.7 (18.6 to 37.2)	1.7 (0.8 to 3.3)	3.6 (2.9 to 4.6)	446.6 (365.0 to 538.0)	348.0 (272.8 to 437.9)	82.1 (57.2 to 114.6)	5.2 (2.4 to 10.2)	11.2 (9.0 to 14.1)
Bhutan	2032	149.4 (122.0 to 181.3)	116.6 (91.3 to 146.9)	27.4 (19.1 to 38.3)	1.8 (0.8 to 3.4)	3.7 (3.0 to 4.7)	460.1 (375.8 to 558.2)	359.0 (281.1 to 452.3)	84.3 (58.7 to 117.8)	5.4 (2.5 to 10.5)	11.4 (9.2 to 14.5)
Bhutan	2033	154.0 (125.6 to 187.5)	120.3 (93.7 to 152.1)	28.1 (19.6 to 39.3)	1.8 (0.8 to 3.5)	3.8 (3.0 to 4.9)	474.0 (386.7 to 577.2)	370.3 (288.6 to 468.4)	86.5 (60.2 to 120.9)	5.6 (2.6 to 10.9)	11.7 (9.2 to 15.0)
Bhutan	2034	158.6 (129.4 to 192.6)	124.0 (96.5 to 157.6)	28.8 (20.1 to 40.3)	1.9 (0.9 to 3.6)	3.9 (3.0 to 5.0)	488.3 (398.4 to 592.9)	381.8 (297.2 to 485.3)	88.7 (61.8 to 124.1)	5.8 (2.7 to 11.2)	12.0 (9.4 to 15.4)
Bhutan	2035	163.4 (132.9 to 198.8)	127.9 (99.5 to 163.7)	29.6 (20.6 to 41.4)	1.9 (0.9 to 3.7)	4.0 (3.1 to 5.2)	503.3 (409.1 to 612.1)	393.9 (306.2 to 504.2)	91.1 (63.5 to 127.5)	6.0 (2.7 to 11.5)	12.2 (9.5 to 15.9)
Bhutan	2036	168.4 (136.3 to 205.6)	132.0 (102.4 to 169.7)	30.4 (21.2 to 42.5)	2.0 (0.9 to 3.9)	4.1 (3.2 to 5.5)	518.6 (419.6 to 632.9)	406.3 (315.2 to 522.6)	93.6 (65.1 to 131.0)	6.2 (2.8 to 11.9)	12.5 (9.7 to 16.8)
Bhutan	2037	173.6 (140.2 to 214.6)	136.1 (105.4 to 175.4)	31.2 (21.7 to 43.7)	2.1 (1.0 to 4.0)	4.2 (3.2 to 5.6)	534.4 (431.8 to 660.9)	419.1 (324.4 to 540.1)	96.1 (66.9 to 134.6)	6.4 (2.9 to 12.3)	12.8 (9.8 to 17.3)
Bhutan	2038	178.9 (144.5 to 220.7)	140.4 (108.5 to 180.7)	32.1 (22.3 to 45.0)	2.1 (1.0 to 4.1)	4.3 (3.2 to 5.9)	550.8 (445.1 to 679.6)	432.4 (334.0 to 556.3)	98.7 (68.7 to 138.6)	6.6 (3.0 to 12.6)	13.1 (9.9 to 18.2)
Bhutan	2039	184.4 (149.5 to 229.1)	144.9 (111.7 to 186.8)	32.9 (22.9 to 46.4)	2.2 (1.0 to 4.3)	4.3 (3.3 to 6.0)	567.8 (460.4 to 705.3)	446.2 (344.1 to 575.3)	101.4 (70.6 to 143.0)	6.8 (3.1 to 13.1)	13.4 (10.2 to 18.6)
Bhutan	2040	189.8 (153.3 to 235.8)	149.3 (115.1 to 193.5)	33.8 (23.6 to 47.8)	2.3 (1.1 to 4.4)	4.4 (3.4 to 6.2)	584.5 (472.0 to 725.9)	459.7 (354.4 to 595.8)	104.0 (72.5 to 147.2)	7.1 (3.2 to 13.6)	13.7 (10.4 to 19.1)
Bhutan	2041	195.3 (156.7 to 243.5)	153.7 (118.1 to 199.9)	34.6 (24.2 to 49.1)	2.4 (1.1 to 4.6)	4.6 (3.4 to 6.4)	601.4 (482.4 to 749.7)	473.4 (363.8 to 615.6)	106.6 (74.5 to 151.3)	7.3 (3.4 to 14.1)	14.0 (10.5 to 19.6)
Bhutan	2042	200.8 (161.1 to 252.2)	158.2 (121.3 to 205.3)	35.5 (24.8 to 50.4)	2.4 (1.1 to 4.7)	4.7 (3.5 to 6.7)	618.3 (496.0 to 776.4)	487.1 (373.5 to 632.1)	109.3 (76.4 to 155.1)	7.5 (3.5 to 14.6)	14.4 (10.7 to 20.7)
Bhutan	2043	206.4 (166.6 to 259.8)	162.8 (124.7 to 214.3)	36.4 (25.4 to 51.5)	2.5 (1.2 to 4.9)	4.8 (3.5 to 7.0)	635.7 (512.8 to 799.8)	501.2 (384.1 to 659.9)	111.9 (78.2 to 158.7)	7.8 (3.6 to 15.1)	14.8 (10.9 to 21.4)
Bhutan	2044	212.0 (170.9 to 268.1)	167.3 (127.8 to 223.5)	37.2 (26.0 to 52.7)	2.6 (1.2 to 5.1)	4.9 (3.6 to 7.1)	652.9 (526.2 to 825.6)	515.2 (393.5 to 688.2)	114.6 (80.1 to 162.3)	8.0 (3.7 to 15.6)	15.2 (11.1 to 21.9)
Bhutan	2045	217.6 (174.4 to 277.5)	171.8 (131.1 to 230.5)	38.0 (26.6 to 53.8)	2.7 (1.2 to 5.2)	5.1 (3.6 to 7.6)	670.0 (536.9 to 854.5)	529.0 (403.5 to 709.8)	117.1 (81.9 to 165.7)	8.3 (3.8 to 16.0)	15.6 (11.2 to 23.4)
Bhutan	2046	223.0 (178.0 to 286.1)	176.2 (134.3 to 239.3)	38.8 (27.2 to 54.9)	2.8 (1.3 to 5.3)	5.2 (3.7 to 8.0)	686.8 (548.1 to 881.0)	542.6 (413.6 to 736.8)	119.6 (83.7 to 169.0)	8.5 (3.9 to 16.4)	16.0 (11.5 to 24.7)
Bhutan	2047	228.7 (182.3 to 295.4)	180.8 (137.7 to 248.1)	39.6 (27.8 to 56.1)	2.8 (1.3 to 5.5)	5.3 (3.8 to 8.4)	704.1 (561.3 to 909.4)	556.8 (423.9 to 763.9)	122.1 (85.5 to 172.6)	8.8 (4.0 to 16.9)	16.5 (11.8 to 25.9)
Bhutan	2048	234.2 (187.1 to 306.3)	185.3 (140.7 to 258.0)	40.4 (28.4 to 57.3)	2.9 (1.3 to 5.7)	5.5 (3.9 to 8.9)	721.1 (576.1 to 943.1)	570.6 (433.2 to 794.5)	124.5 (87.3 to 176.3)	9.0 (4.1 to 17.4)	17.0 (12.0 to 27.4)
Bhutan	2049	239.4 (189.5 to 317.2)	189.6 (143.5 to 266.5)	41.2 (28.9 to 58.3)	3.0 (1.4 to 5.8)	5.7 (4.0 to 9.2)	737.2 (583.6 to 976.7)	583.7 (441.8 to 820.7)	126.8 (89.0 to 179.6)	9.2 (4.2 to 17.9)	17.5 (12.4 to 28.4)
Bhutan	2050	244.8 (192.9 to 327.6)	193.9 (146.3 to 276.5)	41.9 (29.5 to 59.2)	3.1 (1.4 to 5.9)	5.9 (4.1 to 9.5)	753.7 (593.9 to 1008.8)	597.1 (450.6 to 851.5)	129.0 (90.7 to 182.4)	9.5 (4.4 to 18.2)	18.1 (12.6 to 29.3)

Country	Year	2018 US Dollars per capita					2018 PPP per capita				
		Health spending	Government health spending	Out-of-pocket health spending	Prepaid private health spending	Development assistance for health	Health spending	Government health spending	Out-of-pocket health spending	Prepaid private health spending	Development assistance for health
Bolivia	1995	77.8 (65.8 to 90.8)	37.7 (30.2 to 46.0)	29.3 (21.7 to 39.3)	4.6 (2.1 to 8.7)	6.2 (6.2 to 6.2)	176.5 (149.2 to 206.0)	85.6 (68.5 to 104.4)	66.5 (49.1 to 89.1)	10.4 (4.8 to 19.8)	14.0 (14.0 to 14.0)
Bolivia	1996	88.2 (75.6 to 101.7)	43.9 (35.6 to 53.3)	31.1 (23.3 to 41.3)	5.0 (2.4 to 9.3)	8.2 (8.2 to 8.2)	200.1 (171.5 to 230.8)	99.7 (80.7 to 120.8)	70.6 (52.8 to 93.7)	11.3 (5.4 to 21.0)	18.6 (18.6 to 18.6)
Bolivia	1997	92.6 (79.0 to 106.3)	47.5 (38.8 to 57.3)	32.6 (24.5 to 42.8)	5.0 (2.5 to 9.7)	7.5 (7.5 to 7.5)	210.0 (179.3 to 241.2)	107.7 (88.0 to 130.1)	73.9 (55.6 to 97.0)	11.4 (5.6 to 22.0)	17.0 (17.0 to 17.0)
Bolivia	1998	103.4 (88.8 to 118.6)	53.0 (43.6 to 63.4)	35.6 (26.8 to 46.5)	5.2 (2.6 to 9.8)	9.7 (9.7 to 9.7)	234.7 (201.5 to 269.0)	120.2 (98.9 to 144.0)	80.7 (60.9 to 105.5)	11.9 (5.9 to 22.2)	21.9 (21.9 to 21.9)
Bolivia	1999	107.4 (92.3 to 122.7)	56.3 (46.7 to 67.4)	37.3 (28.3 to 48.5)	5.2 (2.6 to 9.8)	8.6 (8.6 to 8.6)	243.6 (209.5 to 278.4)	127.7 (106.0 to 153.0)	84.6 (64.2 to 110.1)	11.9 (5.9 to 22.2)	19.4 (19.4 to 19.4)
Bolivia	2000	109.5 (95.5 to 125.2)	57.3 (47.8 to 68.6)	35.0 (26.6 to 45.3)	5.1 (2.5 to 9.7)	12.0 (12.0 to 12.0)	248.4 (216.7 to 284.1)	130.0 (108.6 to 155.6)	79.5 (60.3 to 102.8)	11.6 (5.7 to 21.9)	27.3 (27.3 to 27.3)
Bolivia	2001	108.6 (94.4 to 124.5)	58.9 (49.1 to 70.1)	34.8 (26.8 to 44.7)	5.2 (2.6 to 9.8)	9.6 (9.6 to 9.6)	246.4 (214.3 to 282.4)	133.7 (111.5 to 159.0)	78.9 (60.9 to 101.5)	11.9 (5.9 to 22.2)	21.9 (21.9 to 21.9)
Bolivia	2002	107.1 (92.8 to 122.5)	59.8 (50.2 to 71.3)	34.8 (26.6 to 45.0)	5.2 (2.6 to 9.8)	7.2 (7.2 to 7.2)	243.1 (210.5 to 277.9)	135.8 (113.9 to 161.9)	79.1 (60.5 to 102.0)	11.9 (5.9 to 22.2)	16.4 (16.4 to 16.4)
Bolivia	2003	112.2 (98.0 to 127.8)	61.1 (51.1 to 72.2)	34.8 (26.3 to 44.9)	5.3 (2.6 to 9.9)	11.0 (11.0 to 11.0)	254.5 (222.3 to 289.9)	138.5 (115.9 to 163.8)	79.0 (59.7 to 102.0)	12.1 (6.0 to 22.4)	24.9 (24.9 to 24.9)
Bolivia	2004	112.0 (97.6 to 127.5)	62.0 (52.1 to 72.8)	34.5 (26.3 to 44.7)	5.2 (2.5 to 9.5)	10.3 (10.3 to 10.3)	254.2 (221.5 to 289.3)	140.7 (118.1 to 165.2)	78.4 (59.6 to 101.5)	11.9 (5.8 to 21.6)	23.3 (23.3 to 23.3)
Bolivia	2005	111.5 (97.6 to 127.5)	64.1 (54.2 to 75.1)	34.6 (26.1 to 45.2)	5.2 (2.5 to 9.5)	7.5 (7.5 to 7.5)	253.0 (221.5 to 289.4)	145.5 (123.1 to 170.4)	78.5 (59.3 to 102.6)	11.9 (5.7 to 21.6)	17.1 (17.1 to 17.1)
Bolivia	2006	115.2 (101.1 to 130.9)	66.3 (56.8 to 77.7)	34.6 (26.2 to 45.2)	5.1 (2.5 to 9.2)	9.2 (9.2 to 9.2)	261.5 (229.3 to 297.1)	150.5 (128.9 to 176.3)	78.4 (59.5 to 102.5)	11.6 (5.6 to 20.8)	21.0 (21.0 to 21.0)
Bolivia	2007	117.4 (103.1 to 133.2)	69.3 (59.2 to 80.3)	34.8 (26.3 to 45.5)	5.0 (2.4 to 9.0)	8.3 (8.3 to 8.3)	266.4 (234.0 to 302.2)	157.4 (134.4 to 182.3)	79.0 (59.6 to 103.2)	11.2 (5.4 to 20.4)	18.8 (18.8 to 18.8)
Bolivia	2008	123.5 (108.8 to 139.9)	74.1 (62.8 to 85.6)	36.3 (27.4 to 47.5)	4.9 (2.4 to 9.1)	8.2 (8.2 to 8.2)	280.3 (246.9 to 317.5)	168.1 (142.6 to 194.3)	82.4 (62.2 to 107.8)	11.2 (5.4 to 20.8)	18.6 (18.6 to 18.6)
Bolivia	2009	128.6 (112.5 to 145.8)	78.4 (66.7 to 91.0)	38.7 (29.4 to 49.8)	5.0 (2.4 to 9.6)	6.5 (6.5 to 6.5)	291.9 (255.3 to 330.9)	178.0 (151.3 to 206.4)	87.9 (66.8 to 113.1)	11.3 (5.4 to 21.7)	14.8 (14.8 to 14.8)
Bolivia	2010	136.2 (120.6 to 154.0)	82.4 (70.9 to 95.3)	41.7 (31.7 to 53.8)	5.0 (2.4 to 9.1)	7.1 (7.1 to 7.1)	309.1 (273.6 to 349.6)	187.0 (160.8 to 216.3)	94.7 (72.0 to 122.1)	11.2 (5.3 to 20.7)	16.2 (16.2 to 16.2)
Bolivia	2011	143.4 (127.6 to 162.2)	86.8 (74.8 to 100.4)	44.2 (33.4 to 57.3)	5.0 (2.4 to 9.2)	7.4 (7.4 to 7.4)	325.5 (289.5 to 368.0)	197.0 (169.7 to 227.9)	100.3 (75.8 to 130.0)	11.4 (5.5 to 21.0)	16.8 (16.8 to 16.8)
Bolivia	2012	151.0 (133.6 to 171.9)	92.4 (79.9 to 106.6)	47.3 (36.0 to 61.4)	5.1 (2.5 to 9.5)	6.2 (6.2 to 6.2)	342.7 (303.2 to 390.1)	209.7 (181.2 to 242.0)	107.3 (81.7 to 139.4)	11.6 (5.6 to 21.5)	14.1 (14.1 to 14.1)
Bolivia	2013	166.6 (147.0 to 189.8)	103.0 (88.7 to 118.6)	51.3 (38.8 to 66.7)	5.4 (2.6 to 10.1)	6.9 (6.9 to 6.9)	378.0 (333.5 to 430.7)	233.7 (201.2 to 269.1)	116.3 (88.1 to 151.4)	12.3 (5.8 to 22.8)	15.7 (15.7 to 15.7)
Bolivia	2014	180.6 (158.8 to 206.3)	115.8 (99.3 to 134.0)	53.4 (40.3 to 69.8)	5.9 (2.8 to 11.0)	5.5 (5.5 to 5.5)	409.7 (360.3 to 468.2)	262.7 (225.3 to 304.1)	121.1 (91.5 to 158.4)	13.4 (6.3 to 25.0)	12.6 (12.6 to 12.6)
Bolivia	2015	199.1 (173.5 to 228.2)	131.4 (111.2 to 153.9)	56.1 (42.3 to 73.1)	6.6 (3.1 to 12.3)	5.0 (5.0 to 5.0)	451.8 (393.7 to 517.8)	298.2 (252.3 to 349.3)	127.3 (96.0 to 165.9)	14.9 (7.1 to 27.8)	11.4 (11.4 to 11.4)

Country	Year	2018 US Dollars per capita					2018 PPP per capita				
		Health spending	Government health spending	Out-of-pocket health spending	Prepaid private health spending	Development assistance for health	Health spending	Government health spending	Out-of-pocket health spending	Prepaid private health spending	Development assistance for health
Bolivia	2016	214.0 (185.3 to 245.9)	142.7 (120.0 to 169.8)	60.2 (45.0 to 78.7)	7.1 (3.3 to 13.1)	3.9 (3.9 to 3.9)	485.6 (420.5 to 558.0)	323.8 (272.3 to 385.3)	136.7 (102.1 to 178.6)	16.2 (7.6 to 29.7)	8.9 (8.9 to 8.9)
Bolivia	2017	218.8 (189.7 to 253.7)	144.7 (121.5 to 172.4)	61.5 (45.9 to 79.9)	7.3 (3.4 to 13.4)	5.2 (5.2 to 5.2)	496.5 (430.4 to 575.6)	328.4 (275.8 to 391.1)	139.6 (104.2 to 181.4)	16.6 (7.8 to 30.4)	11.9 (11.9 to 11.9)
Bolivia	2018	220.7 (191.1 to 253.8)	146.2 (122.5 to 174.1)	62.2 (46.3 to 81.0)	7.5 (3.5 to 13.7)	4.9 (4.9 to 4.9)	500.9 (433.6 to 575.9)	331.7 (278.0 to 395.1)	141.1 (105.0 to 183.8)	17.0 (7.9 to 31.1)	11.1 (11.1 to 11.1)
Bolivia	2019	224.5 (194.0 to 261.3)	149.0 (125.0 to 177.3)	62.9 (46.7 to 81.9)	7.7 (3.6 to 14.0)	4.9 (4.6 to 5.2)	509.3 (440.1 to 593.0)	338.1 (283.6 to 402.3)	142.8 (106.0 to 185.8)	17.4 (8.1 to 31.7)	11.1 (10.4 to 11.8)
Bolivia	2020	226.8 (195.9 to 260.2)	150.5 (126.3 to 179.0)	63.5 (47.2 to 82.8)	7.8 (3.6 to 14.3)	4.9 (4.5 to 5.3)	514.7 (444.6 to 590.5)	341.6 (286.6 to 406.3)	144.1 (107.0 to 187.9)	17.8 (8.3 to 32.4)	11.2 (10.3 to 12.1)
Bolivia	2021	230.3 (197.9 to 268.2)	153.1 (127.9 to 183.0)	64.2 (47.7 to 83.7)	8.0 (3.7 to 14.6)	5.0 (4.5 to 5.5)	522.5 (449.2 to 608.6)	347.3 (290.2 to 415.2)	145.8 (108.3 to 190.0)	18.1 (8.4 to 33.2)	11.3 (10.2 to 12.5)
Bolivia	2022	234.4 (202.1 to 268.7)	156.1 (130.3 to 186.9)	65.1 (48.3 to 84.9)	8.2 (3.8 to 14.9)	5.1 (4.5 to 5.7)	531.8 (458.5 to 609.7)	354.1 (295.7 to 424.2)	147.6 (109.5 to 192.6)	18.5 (8.6 to 33.8)	11.5 (10.2 to 12.8)
Bolivia	2023	238.7 (205.7 to 277.4)	159.3 (133.0 to 190.9)	65.9 (49.1 to 86.0)	8.4 (3.9 to 15.3)	5.1 (4.5 to 5.8)	541.7 (466.7 to 629.4)	361.5 (301.7 to 433.1)	149.6 (111.4 to 195.3)	19.0 (8.8 to 34.6)	11.7 (10.2 to 13.2)
Bolivia	2024	243.1 (210.0 to 278.4)	162.6 (135.6 to 195.0)	66.8 (49.7 to 87.4)	8.5 (4.0 to 15.6)	5.2 (4.5 to 6.0)	551.7 (476.5 to 631.6)	368.9 (307.7 to 442.6)	151.6 (112.9 to 198.4)	19.4 (9.0 to 35.4)	11.9 (10.2 to 13.6)
Bolivia	2025	247.5 (213.3 to 287.5)	165.8 (138.2 to 198.7)	67.7 (50.4 to 88.4)	8.7 (4.0 to 16.0)	5.3 (4.5 to 6.2)	561.7 (484.0 to 652.5)	376.3 (313.7 to 450.9)	153.5 (114.4 to 200.7)	19.8 (9.2 to 36.2)	12.0 (10.3 to 14.1)
Bolivia	2026	251.9 (216.7 to 288.9)	169.1 (140.6 to 202.6)	68.5 (51.0 to 89.5)	8.9 (4.1 to 16.4)	5.4 (4.5 to 6.3)	571.7 (491.8 to 655.6)	383.7 (318.9 to 459.7)	155.5 (115.7 to 203.0)	20.3 (9.4 to 37.2)	12.2 (10.3 to 14.3)
Bolivia	2027	256.3 (221.6 to 296.3)	172.4 (143.0 to 205.8)	69.3 (51.6 to 90.3)	9.1 (4.2 to 16.7)	5.5 (4.6 to 6.5)	581.6 (502.8 to 672.4)	391.2 (324.5 to 467.0)	157.3 (117.1 to 204.9)	20.7 (9.6 to 37.9)	12.4 (10.4 to 14.7)
Bolivia	2028	260.9 (224.3 to 300.5)	175.8 (146.3 to 210.1)	70.2 (52.2 to 91.4)	9.3 (4.3 to 17.0)	5.6 (4.7 to 6.7)	591.9 (508.9 to 681.9)	398.9 (332.1 to 476.7)	159.2 (118.5 to 207.3)	21.1 (9.7 to 38.7)	12.6 (10.6 to 15.2)
Bolivia	2029	265.4 (229.4 to 307.9)	179.3 (149.0 to 214.2)	71.0 (52.9 to 92.7)	9.5 (4.4 to 17.4)	5.7 (4.7 to 6.9)	602.3 (520.5 to 698.7)	406.8 (338.0 to 486.1)	161.1 (120.0 to 210.2)	21.6 (9.9 to 39.5)	12.8 (10.6 to 15.7)
Bolivia	2030	270.0 (232.4 to 310.8)	182.7 (151.6 to 218.3)	71.8 (53.5 to 93.8)	9.7 (4.5 to 17.8)	5.8 (4.7 to 7.1)	612.6 (527.4 to 705.2)	414.6 (344.0 to 495.4)	162.9 (121.4 to 212.8)	22.0 (10.2 to 40.3)	13.1 (10.7 to 16.0)
Bolivia	2031	274.5 (236.1 to 320.6)	186.1 (154.2 to 222.8)	72.6 (54.1 to 94.9)	9.9 (4.6 to 18.1)	5.9 (4.7 to 7.3)	622.8 (535.8 to 727.5)	422.3 (350.0 to 505.6)	164.8 (122.8 to 215.4)	22.5 (10.4 to 41.1)	13.3 (10.8 to 16.7)
Bolivia	2032	279.0 (239.8 to 322.1)	189.6 (156.8 to 227.1)	73.4 (54.7 to 96.1)	10.1 (4.7 to 18.5)	6.0 (4.8 to 7.5)	633.1 (544.1 to 730.9)	430.2 (355.8 to 515.2)	166.5 (124.0 to 218.2)	22.9 (10.6 to 42.0)	13.5 (10.9 to 17.0)
Bolivia	2033	283.7 (243.0 to 330.7)	193.1 (159.5 to 231.5)	74.2 (55.3 to 97.3)	10.3 (4.8 to 18.9)	6.1 (4.8 to 7.8)	643.7 (551.4 to 750.4)	438.2 (361.9 to 525.3)	168.3 (125.5 to 220.7)	23.4 (10.8 to 42.8)	13.8 (10.9 to 17.7)
Bolivia	2034	288.5 (247.4 to 334.0)	196.8 (162.1 to 236.3)	75.0 (55.9 to 98.4)	10.5 (4.9 to 19.2)	6.2 (4.9 to 8.0)	654.6 (561.4 to 757.8)	446.5 (367.8 to 536.2)	170.2 (126.9 to 223.3)	23.8 (11.1 to 43.6)	14.1 (11.1 to 18.2)
Bolivia	2035	293.4 (250.6 to 342.3)	200.5 (165.0 to 241.4)	75.8 (56.3 to 99.3)	10.7 (5.0 to 19.6)	6.3 (4.9 to 8.3)	665.7 (568.6 to 776.6)	455.1 (374.5 to 547.7)	172.0 (127.9 to 225.3)	24.3 (11.3 to 44.5)	14.4 (11.2 to 18.7)
Bolivia	2036	298.4 (254.1 to 346.8)	204.4 (167.8 to 246.2)	76.6 (56.8 to 100.2)	10.9 (5.1 to 20.0)	6.5 (5.0 to 8.7)	677.1 (576.5 to 786.9)	463.8 (380.8 to 558.6)	173.9 (128.9 to 227.3)	24.8 (11.5 to 45.3)	14.6 (11.3 to 19.7)

Country	Year	2018 US Dollars per capita					2018 PPP per capita				
		Health spending	Government health spending	Out-of-pocket health spending	Prepaid private health spending	Development assistance for health	Health spending	Government health spending	Out-of-pocket health spending	Prepaid private health spending	Development assistance for health
Bolivia	2037	303.5 (259.1 to 354.4)	208.3 (170.9 to 251.1)	77.5 (57.3 to 101.2)	11.1 (5.2 to 20.3)	6.6 (5.1 to 8.8)	688.7 (587.9 to 804.3)	472.7 (387.8 to 569.9)	175.8 (130.0 to 229.7)	25.3 (11.7 to 46.1)	14.9 (11.5 to 20.1)
Bolivia	2038	308.7 (262.8 to 358.7)	212.3 (174.3 to 256.3)	78.3 (57.8 to 102.4)	11.4 (5.3 to 20.7)	6.7 (5.1 to 9.3)	700.5 (596.4 to 813.9)	481.8 (395.5 to 581.5)	177.7 (131.1 to 232.3)	25.8 (11.9 to 47.0)	15.2 (11.6 to 21.2)
Bolivia	2039	313.9 (267.4 to 366.3)	216.4 (177.5 to 262.1)	79.1 (58.3 to 103.5)	11.6 (5.4 to 21.1)	6.8 (5.2 to 9.5)	712.4 (606.7 to 831.2)	490.9 (402.8 to 594.6)	179.6 (132.2 to 234.9)	26.3 (12.2 to 47.9)	15.5 (11.9 to 21.6)
Bolivia	2040	319.2 (271.5 to 371.6)	220.4 (180.6 to 266.6)	80.0 (58.9 to 104.8)	11.8 (5.5 to 21.5)	7.0 (5.3 to 9.7)	724.3 (616.1 to 843.2)	500.2 (409.7 to 604.9)	181.4 (133.6 to 237.9)	26.8 (12.4 to 48.7)	15.9 (12.0 to 22.0)
Bolivia	2041	324.5 (276.1 to 381.3)	224.6 (183.4 to 271.8)	80.8 (59.4 to 106.0)	12.0 (5.6 to 21.9)	7.1 (5.4 to 9.9)	736.4 (626.5 to 865.3)	509.6 (416.1 to 616.6)	183.3 (134.9 to 240.6)	27.3 (12.6 to 49.7)	16.2 (12.2 to 22.5)
Bolivia	2042	330.0 (278.4 to 385.4)	228.8 (186.4 to 277.2)	81.6 (60.1 to 107.0)	12.3 (5.7 to 22.3)	7.3 (5.5 to 10.4)	748.7 (631.6 to 874.4)	519.2 (423.0 to 628.9)	185.2 (136.3 to 242.8)	27.8 (12.8 to 50.6)	16.6 (12.4 to 23.6)
Bolivia	2043	335.4 (283.9 to 396.9)	233.0 (189.3 to 283.2)	82.4 (60.6 to 108.0)	12.5 (5.7 to 22.8)	7.5 (5.5 to 10.8)	761.1 (644.1 to 900.6)	528.8 (429.5 to 642.6)	187.0 (137.6 to 245.0)	28.3 (13.0 to 51.7)	16.9 (12.6 to 24.5)
Bolivia	2044	340.9 (286.5 to 399.4)	237.3 (192.5 to 288.2)	83.2 (61.1 to 109.1)	12.7 (5.8 to 23.3)	7.6 (5.6 to 11.1)	773.6 (650.1 to 906.4)	538.6 (436.8 to 654.0)	188.8 (138.7 to 247.5)	28.9 (13.3 to 52.8)	17.3 (12.8 to 25.1)
Bolivia	2045	346.5 (293.0 to 410.3)	241.7 (194.9 to 295.1)	84.0 (61.6 to 110.3)	13.0 (6.0 to 23.8)	7.8 (5.6 to 11.8)	786.2 (664.7 to 931.0)	548.5 (442.3 to 669.5)	190.6 (139.9 to 250.3)	29.4 (13.5 to 54.0)	17.8 (12.8 to 26.8)
Bolivia	2046	352.1 (294.5 to 412.4)	246.1 (197.7 to 301.4)	84.8 (62.1 to 111.6)	13.2 (6.1 to 24.3)	8.0 (5.8 to 12.3)	798.9 (668.3 to 935.8)	558.4 (448.6 to 684.0)	192.4 (141.0 to 253.2)	29.9 (13.7 to 55.1)	18.2 (13.1 to 28.0)
Bolivia	2047	357.7 (301.8 to 425.2)	250.5 (200.5 to 306.8)	85.6 (62.7 to 112.8)	13.4 (6.2 to 24.7)	8.2 (5.9 to 12.9)	811.7 (684.9 to 964.8)	568.3 (455.0 to 696.1)	194.2 (142.2 to 255.8)	30.5 (14.0 to 56.1)	18.6 (13.4 to 29.4)
Bolivia	2048	363.3 (301.6 to 428.7)	254.9 (203.2 to 312.8)	86.4 (63.3 to 113.8)	13.7 (6.3 to 25.2)	8.4 (6.0 to 13.5)	824.5 (684.4 to 972.8)	578.3 (461.0 to 709.8)	196.0 (143.5 to 258.2)	31.0 (14.2 to 57.2)	19.1 (13.6 to 30.7)
Bolivia	2049	369.1 (309.2 to 438.4)	259.3 (206.4 to 319.4)	87.2 (63.8 to 114.9)	13.9 (6.4 to 25.7)	8.6 (6.2 to 13.9)	837.5 (701.5 to 994.9)	588.4 (468.4 to 724.8)	197.8 (144.9 to 260.7)	31.6 (14.5 to 58.2)	19.6 (14.0 to 31.6)
Bolivia	2050	375.0 (309.9 to 442.9)	263.9 (209.4 to 326.1)	88.0 (64.4 to 116.0)	14.2 (6.5 to 26.1)	8.9 (6.2 to 14.5)	850.9 (703.1 to 1005.1)	598.9 (475.2 to 740.0)	199.7 (146.1 to 263.3)	32.2 (14.7 to 59.3)	20.2 (14.1 to 32.8)
Bosnia and Herzegovina	1995	93.7 (80.7 to 108.8)	36.2 (30.1 to 42.9)	56.6 (45.6 to 69.8)	0.6 (0.3 to 1.1)	0.4 (0.4 to 0.4)	226.8 (195.4 to 263.3)	87.6 (72.8 to 103.8)	136.9 (110.3 to 168.9)	1.4 (0.7 to 2.6)	0.9 (0.9 to 0.9)
Bosnia and Herzegovina	1996	153.9 (135.5 to 175.0)	62.5 (53.1 to 73.1)	85.3 (70.1 to 103.5)	1.0 (0.5 to 1.9)	5.1 (5.1 to 5.1)	372.5 (327.8 to 423.4)	151.3 (128.4 to 176.8)	206.4 (169.6 to 250.4)	2.4 (1.2 to 4.5)	12.4 (12.4 to 12.4)
Bosnia and Herzegovina	1997	182.5 (160.3 to 206.7)	77.1 (67.0 to 89.8)	102.9 (85.3 to 122.8)	1.3 (0.6 to 2.5)	1.2 (1.2 to 1.2)	441.7 (387.8 to 500.2)	186.5 (162.1 to 217.4)	249.1 (206.4 to 297.2)	3.2 (1.5 to 6.0)	3.0 (3.0 to 3.0)
Bosnia and Herzegovina	1998	213.6 (190.6 to 238.3)	88.1 (77.2 to 100.7)	113.5 (94.9 to 134.3)	1.6 (0.7 to 3.0)	10.5 (10.5 to 10.5)	516.9 (461.2 to 576.7)	213.1 (186.8 to 243.7)	274.6 (229.8 to 325.0)	3.8 (1.8 to 7.2)	25.4 (25.4 to 25.4)
Bosnia and Herzegovina	1999	243.2 (221.2 to 268.2)	106.7 (94.5 to 120.6)	112.9 (95.0 to 133.0)	1.9 (0.9 to 3.7)	21.8 (21.8 to 21.8)	588.6 (535.2 to 649.1)	258.2 (228.6 to 291.7)	273.1 (229.9 to 321.8)	4.6 (2.2 to 8.9)	52.7 (52.7 to 52.7)
Bosnia and Herzegovina	2000	222.0 (202.3 to 243.7)	116.0 (103.3 to 129.7)	98.8 (83.0 to 117.0)	2.1 (1.0 to 4.3)	5.1 (5.1 to 5.1)	537.1 (489.5 to 589.6)	280.6 (249.9 to 314.0)	239.2 (200.8 to 283.2)	5.2 (2.5 to 10.3)	12.2 (12.2 to 12.2)
Bosnia and Herzegovina	2001	230.6 (211.6 to 252.2)	127.0 (113.9 to 141.2)	92.9 (78.2 to 109.1)	2.5 (1.2 to 4.9)	8.2 (8.2 to 8.2)	558.0 (512.1 to 610.3)	307.3 (275.5 to 341.6)	224.7 (189.2 to 263.9)	6.0 (2.9 to 11.9)	19.9 (19.9 to 19.9)

Country	Year	2018 US Dollars per capita					2018 PPP per capita				
		Health spending	Government health spending	Out-of-pocket health spending	Prepaid private health spending	Development assistance for health	Health spending	Government health spending	Out-of-pocket health spending	Prepaid private health spending	Development assistance for health
Bosnia and Herzegovina	2002	242.9 (223.3 to 265.6)	142.0 (127.4 to 157.2)	93.2 (78.5 to 109.3)	3.0 (1.4 to 5.6)	4.8 (4.8 to 4.8)	587.8 (540.3 to 642.8)	343.6 (308.2 to 380.4)	225.5 (190.0 to 264.5)	7.2 (3.5 to 13.6)	11.6 (11.6 to 11.6)
Bosnia and Herzegovina	2003	268.0 (247.0 to 291.2)	158.9 (143.3 to 175.3)	100.5 (85.2 to 116.4)	3.6 (1.7 to 6.6)	5.1 (5.1 to 5.1)	648.5 (597.7 to 704.7)	384.5 (346.9 to 424.1)	243.1 (206.1 to 281.7)	8.6 (4.2 to 15.9)	12.3 (12.3 to 12.3)
Bosnia and Herzegovina	2004	298.1 (274.2 to 322.5)	170.1 (154.1 to 186.8)	120.9 (103.7 to 140.0)	4.0 (1.9 to 7.6)	3.1 (3.1 to 3.1)	721.3 (663.5 to 780.3)	411.6 (373.0 to 452.0)	292.5 (251.0 to 338.9)	9.8 (4.6 to 18.4)	7.5 (7.5 to 7.5)
Bosnia and Herzegovina	2005	326.6 (300.3 to 353.8)	180.4 (163.8 to 196.7)	137.7 (118.7 to 160.1)	4.5 (2.1 to 8.5)	4.0 (4.0 to 4.0)	790.4 (726.6 to 856.1)	436.6 (396.5 to 476.1)	333.3 (287.3 to 387.4)	10.8 (5.1 to 20.5)	9.8 (9.8 to 9.8)
Bosnia and Herzegovina	2006	355.5 (326.8 to 384.6)	195.7 (177.8 to 214.0)	150.8 (130.5 to 173.2)	4.9 (2.3 to 9.1)	4.1 (4.1 to 4.1)	860.2 (790.7 to 930.6)	473.6 (430.2 to 517.8)	364.8 (315.7 to 419.1)	11.9 (5.5 to 22.1)	9.9 (9.9 to 9.9)
Bosnia and Herzegovina	2007	384.7 (354.9 to 414.7)	215.8 (196.0 to 235.9)	159.0 (137.9 to 182.1)	5.4 (2.5 to 9.9)	4.4 (4.4 to 4.4)	931.0 (858.8 to 1003.6)	522.3 (474.2 to 570.8)	384.8 (333.6 to 440.5)	13.1 (6.1 to 23.9)	10.7 (10.7 to 10.7)
Bosnia and Herzegovina	2008	422.7 (393.2 to 454.1)	240.3 (220.2 to 261.6)	159.0 (137.6 to 182.6)	6.0 (2.8 to 11.1)	17.4 (17.4 to 17.4)	1022.9 (951.6 to 1098.8)	581.4 (532.7 to 633.0)	384.8 (333.0 to 441.8)	14.5 (6.8 to 26.8)	42.1 (42.1 to 42.1)
Bosnia and Herzegovina	2009	412.8 (383.4 to 442.0)	257.5 (236.3 to 279.5)	140.8 (123.0 to 162.1)	6.4 (3.0 to 11.8)	8.1 (8.1 to 8.1)	999.0 (927.8 to 1069.6)	623.2 (571.8 to 676.4)	340.7 (297.7 to 392.2)	15.5 (7.3 to 28.6)	19.6 (19.6 to 19.6)
Bosnia and Herzegovina	2010	427.4 (397.3 to 456.8)	273.2 (250.1 to 295.8)	133.0 (115.6 to 153.2)	6.8 (3.2 to 12.2)	14.4 (14.4 to 14.4)	1034.3 (961.3 to 1105.3)	661.0 (605.2 to 715.7)	321.9 (279.6 to 370.7)	16.4 (7.8 to 29.6)	34.9 (34.9 to 34.9)
Bosnia and Herzegovina	2011	435.0 (404.1 to 464.4)	288.1 (264.9 to 311.6)	130.5 (113.2 to 150.8)	7.2 (3.4 to 13.1)	9.2 (9.2 to 9.2)	1052.6 (977.9 to 1123.9)	697.2 (641.0 to 754.1)	315.8 (273.8 to 364.8)	17.4 (8.3 to 31.7)	22.1 (22.1 to 22.1)
Bosnia and Herzegovina	2012	445.6 (416.2 to 476.3)	299.7 (277.2 to 323.4)	130.3 (112.5 to 149.5)	7.6 (3.6 to 13.9)	8.1 (8.1 to 8.1)	1078.3 (1007.2 to 1152.6)	725.1 (670.7 to 782.5)	315.3 (272.1 to 361.7)	18.4 (8.7 to 33.7)	19.5 (19.5 to 19.5)
Bosnia and Herzegovina	2013	462.5 (430.7 to 494.7)	315.3 (292.7 to 340.0)	132.7 (113.2 to 153.2)	8.1 (3.8 to 15.4)	6.3 (6.3 to 6.3)	1119.1 (1042.2 to 1197.2)	763.0 (708.2 to 822.7)	321.1 (274.0 to 370.6)	19.7 (9.3 to 37.3)	15.3 (15.3 to 15.3)
Bosnia and Herzegovina	2014	478.4 (445.1 to 513.7)	326.7 (302.8 to 354.4)	135.1 (114.8 to 156.3)	8.6 (4.1 to 16.3)	8.0 (8.0 to 8.0)	1157.5 (1077.1 to 1243.0)	790.5 (732.7 to 857.5)	326.9 (277.8 to 378.2)	20.7 (10.0 to 39.5)	19.4 (19.4 to 19.4)
Bosnia and Herzegovina	2015	491.9 (455.1 to 531.6)	341.6 (312.7 to 372.8)	139.0 (117.2 to 163.1)	9.1 (4.4 to 17.3)	2.2 (2.2 to 2.2)	1190.2 (1101.3 to 1286.3)	826.5 (756.7 to 902.0)	336.3 (283.7 to 394.7)	22.1 (10.6 to 41.8)	5.3 (5.3 to 5.3)
Bosnia and Herzegovina	2016	517.1 (472.8 to 568.7)	354.4 (318.6 to 394.7)	142.8 (117.0 to 170.4)	9.7 (4.6 to 18.3)	10.2 (10.2 to 10.2)	1251.3 (1144.0 to 1376.1)	857.6 (771.0 to 955.1)	345.6 (283.2 to 412.3)	23.4 (11.0 to 44.3)	24.7 (24.7 to 24.7)
Bosnia and Herzegovina	2017	523.6 (477.1 to 573.5)	359.8 (323.3 to 401.1)	146.4 (119.8 to 175.6)	9.9 (4.7 to 18.9)	7.6 (7.6 to 7.6)	1267.1 (1154.6 to 1387.8)	870.5 (782.2 to 970.6)	354.2 (290.0 to 425.0)	24.0 (11.3 to 45.7)	18.3 (18.3 to 18.3)
Bosnia and Herzegovina	2018	534.9 (488.9 to 587.1)	368.4 (331.2 to 410.8)	149.4 (122.5 to 179.3)	10.2 (4.8 to 19.4)	6.9 (6.8 to 6.9)	1294.4 (1183.0 to 1420.7)	891.4 (801.4 to 994.2)	361.6 (296.4 to 433.9)	24.7 (11.7 to 47.0)	16.6 (16.4 to 16.8)
Bosnia and Herzegovina	2019	546.2 (499.0 to 597.3)	376.4 (338.4 to 419.5)	152.3 (124.8 to 182.7)	10.5 (4.9 to 19.9)	7.0 (6.5 to 7.4)	1321.6 (1207.4 to 1445.2)	910.7 (818.9 to 1015.2)	368.6 (302.0 to 442.1)	25.4 (12.0 to 48.2)	16.9 (15.7 to 17.9)
Bosnia and Herzegovina	2020	557.6 (509.4 to 614.0)	384.5 (345.5 to 429.4)	155.3 (127.2 to 186.0)	10.8 (5.1 to 20.3)	7.1 (6.5 to 7.7)	1349.4 (1232.6 to 1485.8)	930.3 (836.1 to 1039.1)	375.8 (307.9 to 450.2)	26.0 (12.3 to 49.2)	17.3 (15.7 to 18.7)
Bosnia and Herzegovina	2021	568.2 (519.4 to 621.5)	391.9 (352.0 to 438.0)	158.0 (129.5 to 189.1)	11.0 (5.2 to 20.8)	7.3 (6.6 to 8.1)	1374.9 (1256.9 to 1504.0)	948.2 (851.8 to 1060.0)	382.3 (313.4 to 457.5)	26.6 (12.5 to 50.2)	17.7 (16.0 to 19.6)
Bosnia and Herzegovina	2022	581.1 (531.6 to 640.6)	401.6 (361.6 to 448.8)	160.6 (131.7 to 192.4)	11.3 (5.3 to 21.3)	7.6 (6.7 to 8.5)	1406.1 (1286.4 to 1550.2)	971.7 (874.9 to 1086.0)	388.7 (318.8 to 465.6)	27.3 (12.8 to 51.6)	18.3 (16.3 to 20.5)

		2018 US Dollars per capita					2018 PPP per capita				
Country	Year	Health spending	Government health spending	Out-of-pocket health spending	Prepaid private health spending	Development assistance for health	Health spending	Government health spending	Out-of-pocket health spending	Prepaid private health spending	Development assistance for health
Bosnia and Herzegovina	2023	595.0 (543.2 to 652.5)	412.3 (370.5 to 460.4)	163.3 (133.9 to 195.7)	11.6 (5.4 to 21.8)	7.8 (6.8 to 8.9)	1439.7 (1314.3 to 1578.9)	997.6 (896.5 to 1114.1)	395.2 (323.9 to 473.6)	28.0 (13.1 to 52.7)	18.9 (16.4 to 21.4)
Bosnia and Herzegovina	2024	608.9 (557.9 to 671.4)	423.1 (379.8 to 472.4)	165.9 (135.9 to 198.9)	11.9 (5.6 to 22.3)	8.1 (7.0 to 9.3)	1473.5 (1350.0 to 1624.8)	1023.7 (919.0 to 1143.0)	401.5 (328.9 to 481.4)	28.7 (13.5 to 54.0)	19.6 (17.0 to 22.5)
Bosnia and Herzegovina	2025	623.4 (569.2 to 684.8)	434.3 (389.5 to 484.5)	168.6 (138.1 to 202.2)	12.2 (5.7 to 22.9)	8.4 (7.2 to 9.9)	1508.5 (1377.3 to 1657.0)	1050.8 (942.6 to 1172.4)	407.9 (334.3 to 489.4)	29.4 (13.8 to 55.5)	20.3 (17.3 to 23.9)
Bosnia and Herzegovina	2026	638.0 (584.2 to 703.1)	445.8 (399.1 to 497.6)	171.1 (140.4 to 205.1)	12.5 (5.8 to 23.5)	8.7 (7.3 to 10.2)	1543.9 (1413.7 to 1701.4)	1078.8 (965.8 to 1204.1)	414.0 (339.6 to 496.3)	30.2 (14.1 to 56.9)	21.0 (17.7 to 24.8)
Bosnia and Herzegovina	2027	652.2 (592.8 to 716.7)	456.8 (408.9 to 509.5)	173.7 (142.5 to 208.1)	12.8 (6.0 to 24.1)	9.0 (7.5 to 10.7)	1578.1 (1434.5 to 1734.3)	1105.3 (989.5 to 1232.9)	420.2 (344.9 to 503.5)	30.9 (14.4 to 58.4)	21.7 (18.2 to 25.9)
Bosnia and Herzegovina	2028	666.6 (607.7 to 735.7)	468.0 (419.0 to 522.5)	176.3 (144.7 to 211.1)	13.1 (6.1 to 24.8)	9.3 (7.8 to 11.3)	1613.0 (1470.6 to 1780.2)	1132.3 (1013.8 to 1264.3)	426.5 (350.1 to 510.8)	31.6 (14.8 to 59.9)	22.5 (19.0 to 27.3)
Bosnia and Herzegovina	2029	681.4 (620.8 to 752.2)	479.3 (428.9 to 536.5)	179.1 (147.0 to 214.5)	13.4 (6.2 to 25.4)	9.7 (8.0 to 11.9)	1648.8 (1502.1 to 1820.1)	1159.8 (1037.9 to 1298.1)	433.3 (355.8 to 519.0)	32.4 (15.1 to 61.5)	23.4 (19.2 to 28.7)
Bosnia and Herzegovina	2030	695.6 (631.4 to 766.0)	489.8 (436.7 to 547.6)	182.0 (149.3 to 218.2)	13.7 (6.4 to 26.1)	10.0 (8.2 to 12.3)	1683.1 (1528.0 to 1853.5)	1185.2 (1056.6 to 1325.1)	440.5 (361.4 to 527.9)	33.2 (15.5 to 63.1)	24.2 (19.9 to 29.7)
Bosnia and Herzegovina	2031	710.1 (645.4 to 783.4)	500.5 (445.6 to 559.5)	185.2 (151.9 to 221.8)	14.0 (6.6 to 26.8)	10.4 (8.4 to 13.1)	1718.2 (1561.7 to 1895.7)	1211.0 (1078.2 to 1353.9)	448.1 (367.5 to 536.8)	34.0 (15.9 to 64.8)	25.1 (20.3 to 31.6)
Bosnia and Herzegovina	2032	725.0 (660.0 to 801.7)	511.3 (455.1 to 574.3)	188.6 (154.7 to 225.9)	14.4 (6.7 to 27.4)	10.7 (8.6 to 13.5)	1754.4 (1597.0 to 1939.9)	1237.3 (1101.2 to 1389.6)	456.3 (374.3 to 546.6)	34.8 (16.3 to 66.3)	26.0 (20.9 to 32.7)
Bosnia and Herzegovina	2033	740.0 (670.5 to 821.1)	522.0 (465.1 to 585.5)	192.1 (157.4 to 230.0)	14.8 (6.9 to 28.1)	11.1 (8.9 to 14.3)	1790.6 (1622.4 to 1986.9)	1263.2 (1125.4 to 1416.8)	464.8 (380.9 to 556.7)	35.7 (16.7 to 67.9)	26.9 (21.4 to 34.6)
Bosnia and Herzegovina	2034	755.2 (684.5 to 835.3)	532.9 (474.0 to 599.8)	195.6 (160.0 to 234.4)	15.1 (7.1 to 28.7)	11.6 (9.1 to 15.0)	1827.4 (1656.3 to 2021.3)	1289.4 (1147.1 to 1451.4)	473.4 (387.2 to 567.1)	36.6 (17.1 to 69.6)	28.0 (22.0 to 36.2)
Bosnia and Herzegovina	2035	770.8 (697.5 to 854.6)	544.1 (483.8 to 611.9)	199.2 (163.0 to 238.7)	15.5 (7.3 to 29.4)	12.0 (9.3 to 15.5)	1865.2 (1687.8 to 2067.9)	1316.6 (1170.8 to 1480.8)	482.1 (394.5 to 577.6)	37.5 (17.6 to 71.2)	29.0 (22.5 to 37.6)
Bosnia and Herzegovina	2036	786.7 (709.9 to 868.8)	555.6 (492.5 to 627.1)	202.8 (165.9 to 243.0)	15.9 (7.4 to 30.1)	12.5 (9.7 to 16.6)	1903.7 (1717.9 to 2102.2)	1344.4 (1191.7 to 1517.5)	490.7 (401.5 to 588.0)	38.5 (18.0 to 72.9)	30.1 (23.4 to 40.2)
Bosnia and Herzegovina	2037	803.4 (726.5 to 887.6)	567.7 (501.5 to 641.5)	206.5 (169.0 to 247.6)	16.3 (7.6 to 30.9)	12.9 (10.0 to 17.3)	1944.0 (1757.9 to 2147.8)	1373.6 (1213.4 to 1552.4)	499.7 (409.0 to 599.1)	39.5 (18.4 to 74.7)	31.2 (24.2 to 41.9)
Bosnia and Herzegovina	2038	820.0 (737.8 to 906.3)	579.7 (513.6 to 656.8)	210.2 (172.1 to 252.2)	16.7 (7.8 to 31.7)	13.4 (10.2 to 18.6)	1984.2 (1785.3 to 2193.0)	1402.8 (1242.8 to 1589.4)	508.5 (416.4 to 610.4)	40.4 (18.8 to 76.6)	32.4 (24.6 to 45.0)
Bosnia and Herzegovina	2039	836.9 (754.6 to 930.2)	592.0 (521.5 to 670.7)	213.9 (175.2 to 256.7)	17.1 (7.9 to 32.5)	13.9 (10.6 to 19.4)	2025.1 (1826.0 to 2250.8)	1432.5 (1261.8 to 1623.0)	517.5 (424.1 to 621.3)	41.4 (19.2 to 78.6)	33.7 (25.7 to 47.0)
Bosnia and Herzegovina	2040	854.6 (769.2 to 944.9)	604.9 (535.2 to 684.5)	217.8 (178.6 to 261.7)	17.6 (8.1 to 33.3)	14.4 (11.0 to 20.1)	2068.1 (1861.4 to 2286.4)	1463.6 (1295.1 to 1656.4)	527.0 (432.1 to 633.3)	42.5 (19.7 to 80.7)	35.0 (26.6 to 48.7)
Bosnia and Herzegovina	2041	871.9 (784.8 to 964.2)	617.3 (543.3 to 701.5)	221.7 (181.9 to 266.7)	18.0 (8.3 to 34.2)	15.0 (11.2 to 20.9)	2109.9 (1898.9 to 2333.2)	1493.7 (1314.7 to 1697.4)	536.4 (440.2 to 645.3)	43.5 (20.2 to 82.8)	36.3 (27.2 to 50.7)
Bosnia and Herzegovina	2042	890.7 (803.0 to 986.7)	630.7 (553.5 to 718.1)	225.9 (185.4 to 272.0)	18.5 (8.6 to 35.1)	15.6 (11.6 to 22.4)	2155.3 (1943.1 to 2387.6)	1526.2 (1339.3 to 1737.7)	546.6 (448.5 to 658.3)	44.7 (20.7 to 85.0)	37.8 (28.2 to 54.2)
Bosnia and Herzegovina	2043	910.2 (815.2 to 1009.8)	644.7 (564.2 to 735.5)	230.3 (189.3 to 277.6)	18.9 (8.8 to 36.1)	16.2 (12.0 to 23.5)	2202.5 (1972.5 to 2443.4)	1560.1 (1365.3 to 1779.8)	557.4 (458.2 to 671.8)	45.8 (21.3 to 87.3)	39.3 (29.0 to 56.9)

Country	Year	2018 US Dollars per capita					2018 PPP per capita				
		Health spending	Government health spending	Out-of-pocket health spending	Prepaid private health spending	Development assistance for health	Health spending	Government health spending	Out-of-pocket health spending	Prepaid private health spending	Development assistance for health
Bosnia and Herzegovina	2044	930.1 (831.3 to 1031.3)	658.9 (574.1 to 751.7)	234.9 (192.9 to 283.2)	19.4 (9.0 to 37.0)	16.9 (12.4 to 24.5)	2250.7 (2011.7 to 2495.5)	1594.5 (1389.2 to 1819.0)	568.3 (466.8 to 685.2)	47.1 (21.9 to 89.6)	40.8 (29.9 to 59.3)
Bosnia and Herzegovina	2045	950.0 (850.4 to 1057.3)	673.2 (584.9 to 770.3)	239.4 (196.3 to 288.7)	19.9 (9.3 to 38.0)	17.6 (12.6 to 26.6)	2298.9 (2057.7 to 2558.6)	1628.9 (1415.4 to 1863.9)	579.2 (475.1 to 698.6)	48.3 (22.5 to 91.9)	42.5 (30.6 to 64.5)
Bosnia and Herzegovina	2046	970.8 (867.5 to 1081.9)	688.1 (598.3 to 787.0)	243.9 (200.0 to 294.3)	20.5 (9.6 to 38.9)	18.3 (13.1 to 28.2)	2349.1 (2099.3 to 2618.0)	1665.1 (1447.9 to 1904.5)	590.3 (484.0 to 712.2)	49.5 (23.1 to 94.2)	44.2 (31.7 to 68.1)
Bosnia and Herzegovina	2047	991.4 (885.9 to 1104.9)	703.0 (608.1 to 805.3)	248.4 (203.7 to 300.4)	21.0 (9.8 to 39.8)	19.0 (13.6 to 30.0)	2399.0 (2143.7 to 2673.6)	1701.1 (1471.5 to 1948.7)	601.1 (492.9 to 726.9)	50.8 (23.7 to 96.4)	46.0 (33.0 to 72.6)
Bosnia and Herzegovina	2048	1012.0 (900.5 to 1132.7)	717.9 (618.5 to 824.2)	252.8 (207.4 to 306.4)	21.5 (10.1 to 40.9)	19.8 (14.1 to 32.1)	2448.9 (2178.9 to 2740.8)	1737.1 (1496.6 to 1994.5)	611.8 (501.9 to 741.5)	52.1 (24.4 to 99.0)	48.0 (34.2 to 77.7)
Bosnia and Herzegovina	2049	1032.6 (917.5 to 1156.0)	732.8 (631.4 to 845.4)	257.3 (210.8 to 312.1)	22.0 (10.3 to 41.9)	20.5 (14.5 to 33.6)	2498.7 (2220.2 to 2797.2)	1773.2 (1527.7 to 2045.7)	622.5 (510.0 to 755.2)	53.3 (25.0 to 101.4)	49.7 (35.0 to 81.3)
Bosnia and Herzegovina	2050	1053.6 (937.8 to 1181.5)	748.1 (643.8 to 866.3)	261.6 (214.5 to 317.7)	22.6 (10.6 to 42.7)	21.4 (14.8 to 35.2)	2549.6 (2269.4 to 2858.9)	1810.2 (1557.9 to 2096.2)	633.0 (519.0 to 768.7)	54.6 (25.6 to 103.4)	51.8 (35.7 to 85.2)
Botswana	1995	292.8 (261.0 to 327.4)	198.7 (172.4 to 227.6)	29.1 (20.6 to 40.1)	51.1 (34.9 to 71.4)	13.8 (13.8 to 13.8)	685.1 (610.8 to 766.1)	465.0 (403.4 to 532.5)	68.2 (48.2 to 93.8)	119.7 (81.7 to 167.1)	32.2 (32.2 to 32.2)
Botswana	1996	288.5 (257.8 to 320.9)	206.1 (182.3 to 232.2)	29.2 (21.0 to 40.0)	52.7 (37.9 to 70.1)	0.6 (0.6 to 0.6)	675.0 (603.4 to 750.9)	482.2 (426.7 to 543.4)	68.3 (49.1 to 93.5)	123.3 (88.6 to 164.1)	1.3 (1.3 to 1.3)
Botswana	1997	295.4 (267.7 to 324.9)	211.8 (188.0 to 236.6)	29.5 (21.0 to 40.1)	53.0 (39.3 to 69.2)	1.1 (1.1 to 1.1)	691.3 (626.5 to 760.3)	495.7 (439.8 to 553.6)	69.1 (49.1 to 93.9)	123.9 (92.0 to 162.0)	2.6 (2.6 to 2.6)
Botswana	1998	285.2 (259.7 to 313.7)	205.9 (184.3 to 229.4)	28.8 (20.5 to 38.6)	49.6 (37.8 to 65.3)	0.9 (0.9 to 0.9)	667.4 (607.7 to 734.0)	481.8 (431.2 to 536.9)	67.4 (47.9 to 90.4)	116.2 (88.5 to 152.7)	2.0 (2.0 to 2.0)
Botswana	1999	297.7 (270.7 to 326.3)	216.6 (195.1 to 240.1)	29.8 (21.3 to 40.4)	50.8 (38.8 to 66.1)	0.5 (0.5 to 0.5)	696.7 (633.5 to 763.5)	506.8 (456.6 to 561.8)	69.8 (49.7 to 94.6)	119.0 (90.9 to 154.6)	1.1 (1.1 to 1.1)
Botswana	2000	300.1 (273.2 to 329.3)	217.5 (195.2 to 240.7)	30.6 (22.0 to 41.7)	50.9 (39.6 to 66.8)	1.1 (1.1 to 1.1)	702.2 (639.3 to 770.5)	508.9 (456.9 to 563.2)	71.7 (51.4 to 97.5)	119.1 (92.7 to 156.2)	2.5 (2.5 to 2.5)
Botswana	2001	293.9 (267.5 to 323.7)	212.0 (190.1 to 235.9)	29.5 (21.2 to 39.2)	51.2 (39.8 to 67.4)	1.1 (1.1 to 1.1)	687.7 (626.0 to 757.5)	496.2 (444.9 to 552.0)	69.0 (49.7 to 91.8)	119.8 (93.1 to 157.6)	2.7 (2.7 to 2.7)
Botswana	2002	285.9 (261.2 to 315.0)	198.4 (178.4 to 220.6)	27.5 (20.0 to 37.3)	52.1 (40.2 to 68.0)	7.8 (7.8 to 7.8)	669.1 (611.3 to 737.0)	464.3 (417.6 to 516.1)	64.4 (46.7 to 87.2)	122.0 (94.0 to 159.2)	18.3 (18.3 to 18.3)
Botswana	2003	305.7 (279.9 to 334.3)	207.6 (187.2 to 229.8)	25.6 (18.5 to 34.7)	56.0 (43.0 to 72.6)	16.5 (16.5 to 16.5)	715.3 (654.9 to 782.3)	485.7 (438.1 to 537.8)	59.8 (43.4 to 81.2)	131.1 (100.7 to 169.9)	38.7 (38.7 to 38.7)
Botswana	2004	316.2 (290.0 to 343.8)	213.9 (192.4 to 236.7)	22.9 (16.6 to 31.5)	56.9 (43.2 to 73.4)	22.5 (22.5 to 22.5)	739.8 (678.7 to 804.5)	500.6 (450.2 to 553.8)	53.5 (38.8 to 73.6)	133.2 (101.1 to 171.8)	52.6 (52.6 to 52.6)
Botswana	2005	306.5 (280.9 to 333.6)	214.5 (193.0 to 237.8)	20.2 (14.7 to 27.6)	57.4 (44.0 to 73.7)	14.4 (14.4 to 14.4)	717.2 (657.3 to 780.6)	501.9 (451.5 to 556.6)	47.3 (34.3 to 64.5)	134.3 (103.0 to 172.5)	33.7 (33.7 to 33.7)
Botswana	2006	323.2 (297.0 to 351.2)	219.8 (197.3 to 244.1)	18.4 (13.4 to 25.0)	58.0 (45.5 to 74.1)	27.0 (27.0 to 27.0)	756.3 (695.1 to 821.9)	514.4 (461.7 to 571.3)	43.1 (31.3 to 58.5)	135.7 (106.5 to 173.5)	63.2 (63.2 to 63.2)
Botswana	2007	339.3 (312.7 to 368.4)	231.5 (207.7 to 258.0)	17.6 (12.9 to 23.6)	60.6 (47.3 to 77.1)	29.7 (29.7 to 29.7)	794.1 (731.8 to 862.2)	541.7 (486.1 to 603.6)	41.2 (30.2 to 55.2)	141.8 (110.7 to 180.4)	69.4 (69.4 to 69.4)
Botswana	2008	476.9 (445.6 to 509.0)	248.7 (223.4 to 276.8)	18.2 (13.3 to 24.4)	68.2 (52.8 to 86.2)	141.7 (141.7 to 141.7)	1116.1 (1042.9 to 1191.0)	582.1 (522.9 to 647.8)	42.6 (31.0 to 57.0)	159.7 (123.4 to 201.6)	331.7 (331.7 to 331.7)

		2018 US Dollars per capita					2018 PPP per capita				
**Country**	**Year**	**Health spending**	**Government health spending**	**Out-of-pocket health spending**	**Prepaid private health spending**	**Development assistance for health**	**Health spending**	**Government health spending**	**Out-of-pocket health spending**	**Prepaid private health spending**	**Development assistance for health**
Botswana	2009	464.0 (433.5 to 495.6)	237.4 (214.2 to 264.5)	18.6 (13.5 to 24.7)	77.7 (60.6 to 96.8)	130.3 (130.3 to 130.3)	1085.9 (1014.3 to 1159.9)	555.6 (501.2 to 618.9)	43.5 (31.7 to 57.8)	181.9 (141.8 to 226.5)	304.9 (304.9 to 304.9)
Botswana	2010	401.0 (367.9 to 434.0)	240.8 (217.6 to 268.5)	19.5 (14.2 to 25.7)	90.9 (71.4 to 113.5)	49.8 (49.8 to 49.8)	938.3 (860.9 to 1015.5)	563.5 (509.3 to 628.2)	45.7 (33.1 to 60.1)	212.6 (167.0 to 265.5)	116.5 (116.5 to 116.5)
Botswana	2011	420.2 (386.7 to 456.9)	246.1 (221.7 to 273.3)	20.2 (14.5 to 26.6)	101.7 (81.1 to 125.8)	52.2 (52.2 to 52.2)	983.3 (905.0 to 1069.3)	575.8 (518.8 to 639.6)	47.4 (34.0 to 62.3)	238.1 (189.7 to 294.3)	122.1 (122.1 to 122.1)
Botswana	2012	406.7 (372.9 to 443.4)	234.4 (211.1 to 259.7)	20.9 (15.2 to 28.2)	108.4 (86.7 to 132.5)	42.9 (42.9 to 42.9)	951.8 (872.6 to 1037.6)	548.6 (493.9 to 607.7)	49.0 (35.6 to 65.9)	253.7 (203.0 to 310.2)	100.5 (100.5 to 100.5)
Botswana	2013	415.1 (381.0 to 452.1)	228.8 (205.4 to 254.5)	21.6 (15.7 to 29.0)	118.5 (97.4 to 145.7)	46.3 (46.3 to 46.3)	971.4 (891.7 to 1058.1)	535.5 (480.7 to 595.5)	50.4 (36.8 to 67.8)	277.2 (227.8 to 340.9)	108.3 (108.3 to 108.3)
Botswana	2014	415.6 (380.4 to 454.5)	232.1 (206.8 to 259.7)	22.2 (16.1 to 30.3)	126.1 (102.7 to 153.6)	35.2 (35.2 to 35.2)	972.6 (890.2 to 1063.5)	543.2 (483.8 to 607.7)	51.9 (37.6 to 70.9)	295.1 (240.2 to 359.4)	82.4 (82.4 to 82.4)
Botswana	2015	411.3 (372.8 to 451.1)	229.7 (204.1 to 258.7)	22.4 (16.2 to 30.6)	131.9 (105.7 to 163.9)	27.2 (27.2 to 27.2)	962.4 (872.4 to 1055.7)	537.5 (477.6 to 605.3)	52.5 (38.0 to 71.6)	308.8 (247.4 to 383.5)	63.7 (63.7 to 63.7)
Botswana	2016	427.3 (380.5 to 478.0)	232.8 (203.6 to 266.3)	22.5 (16.3 to 31.0)	136.2 (106.1 to 175.8)	35.9 (35.9 to 35.9)	1000.0 (890.3 to 1118.6)	544.7 (476.4 to 623.2)	52.6 (38.1 to 72.5)	318.7 (248.3 to 411.4)	84.0 (84.0 to 84.0)
Botswana	2017	423.0 (375.5 to 474.0)	230.9 (201.3 to 264.2)	22.6 (16.3 to 31.1)	137.2 (107.1 to 176.8)	32.3 (32.3 to 32.3)	990.0 (878.8 to 1109.2)	540.3 (471.1 to 618.3)	52.9 (38.1 to 72.7)	321.0 (250.5 to 413.8)	75.7 (75.7 to 75.7)
Botswana	2018	429.3 (380.2 to 481.6)	236.8 (207.5 to 270.1)	22.9 (16.6 to 31.3)	140.8 (109.7 to 181.2)	28.9 (28.6 to 29.1)	1004.6 (889.8 to 1127.1)	554.1 (485.6 to 632.1)	53.6 (38.8 to 73.3)	329.4 (256.7 to 423.9)	67.6 (66.9 to 68.1)
Botswana	2019	439.4 (388.5 to 493.6)	243.0 (212.2 to 277.6)	23.2 (16.7 to 31.4)	144.4 (112.3 to 186.0)	28.8 (26.8 to 30.8)	1028.1 (909.0 to 1155.0)	568.5 (496.6 to 649.5)	54.2 (39.1 to 73.6)	337.9 (262.9 to 435.3)	67.5 (62.7 to 72.0)
Botswana	2020	449.2 (398.3 to 504.3)	248.7 (216.6 to 284.2)	23.6 (16.9 to 32.0)	147.9 (114.8 to 190.4)	29.0 (26.4 to 31.5)	1051.3 (932.2 to 1180.0)	582.1 (506.9 to 665.1)	55.1 (39.6 to 74.9)	346.2 (268.6 to 445.6)	67.9 (61.7 to 73.7)
Botswana	2021	459.2 (405.0 to 514.9)	254.5 (221.5 to 291.1)	23.9 (17.2 to 32.4)	151.5 (117.8 to 194.8)	29.3 (26.2 to 32.6)	1074.5 (947.7 to 1205.0)	595.5 (518.4 to 681.1)	56.0 (40.3 to 75.9)	354.4 (275.6 to 455.9)	68.6 (61.4 to 76.4)
Botswana	2022	468.5 (414.2 to 526.1)	259.7 (225.5 to 297.4)	24.2 (17.5 to 33.0)	154.8 (120.4 to 199.1)	29.8 (26.3 to 33.5)	1096.4 (969.4 to 1231.1)	607.7 (527.7 to 695.9)	56.7 (40.9 to 77.2)	362.3 (281.6 to 465.9)	69.6 (61.7 to 78.5)
Botswana	2023	477.9 (420.9 to 535.1)	265.0 (229.3 to 303.8)	24.6 (17.7 to 33.3)	158.1 (122.6 to 203.4)	30.2 (26.1 to 34.9)	1118.2 (985.0 to 1252.2)	620.1 (536.7 to 710.9)	57.5 (41.3 to 78.0)	370.0 (286.9 to 476.0)	70.7 (61.0 to 81.6)
Botswana	2024	487.6 (429.6 to 547.7)	270.4 (233.6 to 309.8)	24.9 (17.9 to 33.7)	161.5 (124.8 to 208.3)	30.8 (26.5 to 35.7)	1141.0 (1005.4 to 1281.8)	632.8 (546.6 to 724.9)	58.2 (41.8 to 78.9)	377.8 (292.0 to 487.5)	72.1 (62.0 to 83.6)
Botswana	2025	497.7 (437.3 to 560.3)	276.0 (238.2 to 317.4)	25.2 (18.1 to 34.3)	165.0 (127.2 to 212.2)	31.4 (26.7 to 36.8)	1164.6 (1023.3 to 1311.3)	646.0 (557.4 to 742.7)	59.1 (42.4 to 80.3)	386.1 (297.6 to 496.5)	73.5 (62.5 to 86.2)
Botswana	2026	507.5 (446.1 to 574.1)	281.3 (242.6 to 324.2)	25.6 (18.4 to 34.9)	168.6 (130.0 to 216.6)	32.0 (26.9 to 37.6)	1187.6 (1044.0 to 1343.4)	658.4 (567.7 to 758.5)	59.9 (43.0 to 81.7)	394.4 (304.2 to 506.8)	74.8 (62.9 to 88.0)
Botswana	2027	517.6 (454.9 to 585.6)	286.8 (246.5 to 331.3)	26.0 (18.6 to 35.3)	172.3 (132.9 to 221.3)	32.6 (27.2 to 38.8)	1211.3 (1064.6 to 1370.4)	671.1 (576.9 to 775.2)	60.8 (43.4 to 82.6)	403.1 (311.0 to 517.9)	76.3 (63.7 to 90.8)
Botswana	2028	528.4 (464.1 to 598.4)	292.4 (250.6 to 339.0)	26.4 (18.9 to 35.8)	176.3 (136.1 to 226.6)	33.3 (27.8 to 40.2)	1236.5 (1086.1 to 1400.3)	684.3 (586.4 to 793.2)	61.8 (44.2 to 83.9)	412.5 (318.4 to 530.3)	77.9 (65.0 to 94.2)
Botswana	2029	539.5 (474.2 to 611.2)	298.3 (255.4 to 346.1)	26.8 (19.3 to 36.6)	180.4 (139.3 to 232.6)	34.0 (28.0 to 41.6)	1262.5 (1109.7 to 1430.4)	698.1 (597.6 to 809.9)	62.7 (45.2 to 85.8)	422.1 (326.0 to 544.3)	79.5 (65.4 to 97.4)

		2018 US Dollars per capita					2018 PPP per capita				
Country	Year	Health spending	Government health spending	Out-of-pocket health spending	Prepaid private health spending	Development assistance for health	Health spending	Government health spending	Out-of-pocket health spending	Prepaid private health spending	Development assistance for health
Botswana	2030	550.7 (482.9 to 628.1)	304.2 (259.2 to 353.1)	27.2 (19.6 to 37.4)	184.6 (142.6 to 238.5)	34.7 (28.4 to 42.8)	1288.8 (1130.1 to 1469.8)	712.0 (606.4 to 826.3)	63.7 (45.9 to 87.5)	432.0 (333.8 to 558.1)	81.1 (66.5 to 100.1)
Botswana	2031	562.6 (492.5 to 638.8)	310.5 (263.2 to 361.6)	27.7 (19.9 to 37.9)	189.0 (146.0 to 245.0)	35.4 (28.4 to 44.5)	1316.5 (1152.5 to 1494.8)	726.7 (615.9 to 846.2)	64.8 (46.7 to 88.7)	442.3 (341.8 to 573.2)	82.8 (66.3 to 104.1)
Botswana	2032	575.0 (501.4 to 653.7)	317.1 (268.4 to 368.4)	28.2 (20.3 to 38.7)	193.6 (149.7 to 251.2)	36.1 (28.8 to 45.8)	1345.6 (1173.4 to 1529.8)	742.1 (628.0 to 862.1)	65.9 (47.6 to 90.5)	453.0 (350.3 to 587.8)	84.5 (67.5 to 107.2)
Botswana	2033	588.0 (511.5 to 667.5)	324.1 (273.7 to 377.3)	28.7 (20.7 to 39.5)	198.4 (153.1 to 257.2)	36.9 (29.2 to 47.4)	1375.9 (1196.9 to 1562.0)	758.3 (640.5 to 883.0)	67.1 (48.4 to 92.5)	464.2 (358.3 to 601.8)	86.3 (68.3 to 110.8)
Botswana	2034	601.3 (521.0 to 683.8)	331.1 (279.0 to 387.6)	29.2 (21.1 to 40.2)	203.3 (156.6 to 263.3)	37.7 (29.6 to 48.8)	1407.2 (1219.2 to 1600.1)	774.7 (653.0 to 907.1)	68.3 (49.3 to 94.2)	475.8 (366.5 to 616.2)	88.3 (69.2 to 114.3)
Botswana	2035	614.8 (532.0 to 702.5)	338.2 (283.9 to 397.1)	29.7 (21.3 to 41.1)	208.3 (160.6 to 270.3)	38.5 (29.8 to 50.6)	1438.6 (1244.9 to 1644.0)	791.5 (664.3 to 929.3)	69.5 (49.9 to 96.2)	487.5 (375.9 to 632.6)	90.2 (69.7 to 118.5)
Botswana	2036	628.1 (542.1 to 716.0)	345.4 (288.3 to 406.4)	30.2 (21.7 to 42.0)	213.2 (164.3 to 277.1)	39.3 (30.4 to 53.3)	1469.8 (1268.5 to 1675.6)	808.2 (674.7 to 951.1)	70.6 (50.7 to 98.2)	499.0 (384.5 to 648.3)	92.0 (71.2 to 124.8)
Botswana	2037	641.0 (552.5 to 729.7)	352.6 (292.9 to 417.3)	30.7 (22.0 to 42.8)	218.2 (167.9 to 283.3)	39.6 (29.1 to 54.6)	1500.1 (1292.8 to 1707.5)	825.2 (685.3 to 976.5)	71.8 (51.4 to 100.2)	510.5 (392.8 to 663.0)	92.6 (68.0 to 127.7)
Botswana	2038	654.4 (560.1 to 750.5)	360.2 (297.5 to 427.9)	31.2 (22.3 to 43.6)	223.2 (171.8 to 290.4)	39.7 (0.0 to 57.1)	1531.3 (1310.6 to 1756.2)	842.9 (696.1 to 1001.2)	73.0 (52.1 to 102.0)	522.4 (402.0 to 679.6)	93.0 (0.0 to 133.7)
Botswana	2039	668.3 (569.9 to 765.3)	367.9 (301.6 to 438.9)	31.7 (22.6 to 44.5)	228.3 (175.2 to 297.2)	40.4 (0.0 to 58.4)	1564.0 (1333.6 to 1790.9)	860.9 (705.8 to 1027.1)	74.2 (52.9 to 104.0)	534.3 (409.9 to 695.4)	94.6 (0.0 to 136.7)
Botswana	2040	681.8 (580.2 to 783.1)	375.9 (305.8 to 453.9)	32.2 (22.9 to 45.2)	233.4 (178.9 to 304.5)	40.4 (0.0 to 59.6)	1595.5 (1357.8 to 1832.5)	879.6 (715.6 to 1062.2)	75.4 (53.5 to 105.9)	546.1 (418.7 to 712.6)	94.5 (0.0 to 139.5)
Botswana	2041	695.3 (587.4 to 798.9)	383.8 (310.4 to 466.0)	32.7 (23.2 to 45.9)	238.4 (182.9 to 311.8)	40.4 (0.0 to 61.1)	1627.0 (1374.7 to 1869.5)	898.1 (726.3 to 1090.4)	76.5 (54.2 to 107.5)	557.8 (428.0 to 729.6)	94.6 (0.0 to 143.1)
Botswana	2042	708.9 (593.7 to 825.0)	391.9 (315.3 to 480.2)	33.2 (23.5 to 46.7)	243.5 (186.9 to 318.7)	40.3 (0.0 to 64.7)	1658.9 (1389.3 to 1930.6)	917.0 (737.7 to 1123.7)	77.7 (54.9 to 109.3)	569.8 (437.5 to 745.8)	94.4 (0.0 to 151.4)
Botswana	2043	722.1 (600.1 to 842.0)	400.0 (319.5 to 496.1)	33.7 (23.7 to 47.4)	248.5 (190.6 to 325.5)	40.0 (0.0 to 66.9)	1689.8 (1404.3 to 1970.4)	935.9 (747.7 to 1160.9)	78.8 (55.5 to 111.0)	581.5 (446.1 to 761.6)	93.6 (0.0 to 156.5)
Botswana	2044	735.3 (610.6 to 862.3)	407.9 (323.2 to 510.7)	34.1 (24.0 to 48.2)	253.4 (194.4 to 332.1)	39.8 (0.0 to 68.4)	1720.6 (1429.0 to 2017.9)	954.4 (756.3 to 1195.0)	79.9 (56.2 to 112.8)	593.0 (455.0 to 777.2)	93.2 (0.0 to 160.1)
Botswana	2045	748.6 (615.9 to 881.7)	416.1 (327.0 to 526.5)	34.6 (24.3 to 48.9)	258.3 (198.0 to 338.7)	39.6 (0.0 to 72.9)	1751.8 (1441.2 to 2063.3)	973.7 (765.2 to 1232.2)	81.0 (57.0 to 114.5)	604.5 (463.3 to 792.7)	92.6 (0.0 to 170.6)
Botswana	2046	761.8 (622.9 to 903.6)	424.3 (330.9 to 543.2)	35.1 (24.6 to 49.7)	263.1 (201.4 to 345.2)	39.3 (0.0 to 75.4)	1782.6 (1457.6 to 2114.5)	992.9 (774.4 to 1271.1)	82.1 (57.5 to 116.2)	615.7 (471.4 to 807.9)	91.9 (0.0 to 176.5)
Botswana	2047	775.3 (634.2 to 922.9)	432.7 (334.7 to 560.8)	35.5 (24.8 to 50.3)	268.0 (205.1 to 351.8)	39.0 (0.0 to 78.5)	1814.2 (1484.0 to 2159.7)	1012.6 (783.3 to 1312.2)	83.1 (58.1 to 117.6)	627.1 (479.9 to 823.3)	91.3 (0.0 to 183.6)
Botswana	2048	788.6 (637.8 to 941.6)	440.4 (338.0 to 578.1)	35.9 (25.0 to 50.9)	272.6 (208.0 to 358.2)	39.6 (0.0 to 82.7)	1845.3 (1492.6 to 2203.5)	1030.5 (790.9 to 1352.8)	84.1 (58.6 to 119.1)	638.0 (486.7 to 838.2)	92.8 (0.0 to 193.5)
Botswana	2049	801.8 (644.7 to 964.5)	448.0 (341.2 to 591.5)	36.4 (25.2 to 51.6)	277.4 (211.3 to 364.6)	40.0 (0.0 to 85.0)	1876.2 (1508.7 to 2256.9)	1048.4 (798.4 to 1384.1)	85.1 (59.0 to 120.7)	649.1 (494.5 to 853.2)	93.6 (0.0 to 199.0)
Botswana	2050	815.1 (655.6 to 990.9)	456.1 (344.9 to 608.7)	36.8 (25.5 to 52.2)	282.4 (215.9 to 371.9)	39.8 (0.0 to 89.5)	1907.5 (1534.2 to 2318.7)	1067.4 (807.1 to 1424.5)	86.1 (59.7 to 122.3)	660.9 (505.2 to 870.2)	93.1 (0.0 to 209.5)

		2018 US Dollars per capita					2018 PPP per capita				
**Country**	**Year**	**Health spending**	**Government health spending**	**Out-of-pocket health spending**	**Prepaid private health spending**	**Development assistance for health**	**Health spending**	**Government health spending**	**Out-of-pocket health spending**	**Prepaid private health spending**	**Development assistance for health**
Brazil	1995	558.3 (509.4 to 610.7)	153.8 (130.3 to 177.9)	286.1 (251.4 to 323.2)	117.3 (94.8 to 144.5)	1.1 (1.1 to 1.1)	934.0 (852.1 to 1021.7)	257.3 (218.0 to 297.7)	478.6 (420.6 to 540.7)	196.2 (158.6 to 241.8)	1.9 (1.9 to 1.9)
Brazil	1996	582.7 (540.8 to 625.1)	163.3 (141.0 to 187.4)	294.1 (263.4 to 323.4)	123.8 (103.5 to 145.1)	1.5 (1.5 to 1.5)	974.8 (904.6 to 1045.8)	273.2 (235.9 to 313.6)	491.9 (440.7 to 541.1)	207.1 (173.1 to 242.7)	2.5 (2.5 to 2.5)
Brazil	1997	595.2 (555.7 to 634.6)	170.5 (149.9 to 193.4)	294.9 (268.8 to 321.3)	128.0 (109.5 to 147.8)	1.9 (1.9 to 1.9)	995.7 (929.5 to 1061.6)	285.3 (250.7 to 323.5)	493.3 (449.7 to 537.5)	214.0 (183.1 to 247.2)	3.1 (3.1 to 3.1)
Brazil	1998	588.3 (550.6 to 626.6)	170.4 (151.4 to 191.4)	288.2 (263.1 to 314.7)	128.2 (110.6 to 148.7)	1.4 (1.4 to 1.4)	984.1 (921.1 to 1048.2)	285.0 (253.3 to 320.1)	482.2 (440.2 to 526.4)	214.5 (185.0 to 248.7)	2.4 (2.4 to 2.4)
Brazil	1999	576.9 (540.3 to 614.0)	169.3 (151.0 to 188.7)	275.6 (250.1 to 302.3)	129.3 (111.1 to 150.2)	2.8 (2.8 to 2.8)	965.1 (903.8 to 1027.1)	283.2 (252.5 to 315.7)	461.0 (418.3 to 505.7)	216.3 (185.8 to 251.2)	4.6 (4.6 to 4.6)
Brazil	2000	552.4 (517.0 to 587.4)	166.7 (148.9 to 185.0)	253.8 (230.9 to 278.2)	130.4 (112.3 to 151.5)	1.5 (1.5 to 1.5)	924.0 (864.8 to 982.6)	278.8 (249.1 to 309.5)	424.6 (386.2 to 465.3)	218.1 (187.9 to 253.4)	2.5 (2.5 to 2.5)
Brazil	2001	555.8 (521.3 to 590.3)	178.0 (159.8 to 197.0)	244.3 (221.6 to 268.0)	131.7 (112.8 to 150.8)	1.7 (1.7 to 1.7)	929.7 (872.0 to 987.5)	297.8 (267.4 to 329.5)	408.7 (370.6 to 448.3)	220.3 (188.7 to 252.2)	2.9 (2.9 to 2.9)
Brazil	2002	608.3 (570.3 to 647.7)	220.3 (199.1 to 243.3)	243.6 (220.4 to 268.6)	143.2 (123.1 to 163.4)	1.2 (1.2 to 1.2)	1017.6 (954.0 to 1083.5)	368.6 (333.0 to 407.1)	407.6 (368.7 to 449.4)	239.5 (206.0 to 273.4)	2.0 (2.0 to 2.0)
Brazil	2003	626.2 (588.7 to 667.5)	245.7 (223.3 to 269.9)	234.9 (212.0 to 258.5)	143.0 (122.7 to 163.6)	2.6 (2.6 to 2.6)	1047.5 (984.8 to 1116.6)	411.1 (373.6 to 451.4)	392.9 (354.6 to 432.5)	239.2 (205.3 to 273.6)	4.3 (4.3 to 4.3)
Brazil	2004	660.8 (623.6 to 701.6)	275.7 (251.9 to 299.7)	235.8 (213.2 to 261.7)	148.6 (127.7 to 171.0)	0.8 (0.8 to 0.8)	1105.5 (1043.2 to 1173.6)	461.1 (421.3 to 501.3)	394.4 (356.7 to 437.8)	248.6 (213.6 to 286.1)	1.4 (1.4 to 1.4)
Brazil	2005	683.9 (646.5 to 724.9)	293.7 (269.3 to 320.1)	236.0 (212.6 to 262.2)	153.3 (133.4 to 175.7)	0.9 (0.9 to 0.9)	1144.1 (1081.5 to 1212.7)	491.3 (450.5 to 535.5)	394.8 (355.7 to 438.5)	256.4 (223.1 to 293.9)	1.5 (1.5 to 1.5)
Brazil	2006	710.7 (671.0 to 752.8)	309.2 (283.6 to 338.0)	239.0 (215.1 to 263.9)	161.7 (139.9 to 183.7)	0.7 (0.7 to 0.7)	1188.9 (1122.4 to 1259.3)	517.3 (474.4 to 565.4)	399.8 (359.8 to 441.5)	270.5 (234.0 to 307.3)	1.2 (1.2 to 1.2)
Brazil	2007	745.5 (706.5 to 787.8)	323.9 (297.5 to 354.3)	246.7 (223.3 to 270.0)	174.4 (151.7 to 197.0)	0.6 (0.6 to 0.6)	1247.2 (1181.8 to 1317.8)	541.8 (497.7 to 592.6)	412.7 (373.5 to 451.7)	291.7 (253.7 to 329.6)	1.0 (1.0 to 1.0)
Brazil	2008	783.2 (742.1 to 826.3)	336.0 (308.9 to 367.1)	261.0 (236.7 to 283.9)	185.5 (161.5 to 211.0)	0.7 (0.7 to 0.7)	1310.2 (1241.4 to 1382.3)	562.1 (516.7 to 614.1)	436.6 (396.0 to 474.8)	310.3 (270.2 to 352.9)	1.2 (1.2 to 1.2)
Brazil	2009	809.6 (766.6 to 851.3)	331.8 (305.9 to 359.5)	289.7 (265.5 to 313.9)	187.0 (162.8 to 212.0)	1.0 (1.0 to 1.0)	1354.3 (1282.4 to 1424.1)	555.1 (511.7 to 601.5)	484.7 (444.2 to 525.1)	312.9 (272.4 to 354.6)	1.7 (1.7 to 1.7)
Brazil	2010	904.7 (857.5 to 954.8)	343.1 (318.5 to 371.5)	363.6 (336.6 to 392.1)	196.1 (170.5 to 223.2)	1.8 (1.8 to 1.8)	1513.3 (1434.5 to 1597.2)	574.0 (532.8 to 621.4)	608.2 (563.1 to 655.9)	328.1 (285.2 to 373.4)	3.0 (3.0 to 3.0)
Brazil	2011	974.0 (923.9 to 1027.4)	353.8 (328.3 to 383.2)	414.2 (382.6 to 444.6)	205.0 (180.2 to 232.1)	1.0 (1.0 to 1.0)	1629.4 (1545.5 to 1718.6)	591.8 (549.2 to 641.0)	692.9 (640.0 to 743.8)	343.0 (301.4 to 388.3)	1.7 (1.7 to 1.7)
Brazil	2012	1018.1 (966.8 to 1074.1)	357.8 (330.8 to 387.6)	447.0 (412.8 to 480.4)	212.4 (187.0 to 241.0)	0.9 (0.9 to 0.9)	1703.1 (1617.3 to 1796.8)	598.6 (553.4 to 648.3)	747.8 (690.5 to 803.6)	355.2 (312.7 to 403.2)	1.5 (1.5 to 1.5)
Brazil	2013	1073.2 (1023.2 to 1131.1)	369.9 (340.8 to 398.8)	475.7 (441.6 to 511.3)	226.2 (200.4 to 255.6)	1.4 (1.4 to 1.4)	1795.4 (1711.6 to 1892.2)	618.8 (570.1 to 667.2)	795.8 (738.7 to 855.3)	378.4 (335.3 to 427.6)	2.4 (2.4 to 2.4)
Brazil	2014	1113.7 (1062.4 to 1170.4)	377.5 (347.2 to 409.7)	495.8 (461.4 to 533.5)	239.1 (212.0 to 267.3)	1.3 (1.3 to 1.3)	1863.0 (1777.2 to 1957.9)	631.5 (580.8 to 685.4)	829.4 (771.8 to 892.5)	400.0 (354.6 to 447.1)	2.1 (2.1 to 2.1)
Brazil	2015	1118.0 (1061.0 to 1181.4)	373.8 (340.0 to 407.6)	497.6 (459.6 to 537.2)	246.4 (215.9 to 280.4)	0.3 (0.3 to 0.3)	1870.3 (1774.9 to 1976.3)	625.2 (568.7 to 681.8)	832.3 (768.8 to 898.6)	412.1 (361.2 to 469.1)	0.6 (0.6 to 0.6)

Country	Year	2018 US Dollars per capita					2018 PPP per capita				
		Health spending	Government health spending	Out-of-pocket health spending	Prepaid private health spending	Development assistance for health	Health spending	Government health spending	Out-of-pocket health spending	Prepaid private health spending	Development assistance for health
Brazil	2016	1114.2 (1039.6 to 1195.5)	370.5 (331.0 to 407.4)	489.6 (443.0 to 541.6)	253.3 (213.3 to 297.4)	0.7 (0.7 to 0.7)	1863.9 (1739.1 to 1999.9)	619.8 (553.7 to 681.5)	819.1 (741.0 to 905.9)	423.8 (356.8 to 497.5)	1.2 (1.2 to 1.2)
Brazil	2017	1109.7 (1033.6 to 1188.6)	366.6 (326.9 to 402.9)	489.0 (442.8 to 542.0)	253.4 (212.9 to 297.7)	0.7 (0.7 to 0.7)	1856.4 (1729.1 to 1988.3)	613.3 (546.9 to 673.9)	817.9 (740.8 to 906.7)	423.9 (356.1 to 498.0)	1.2 (1.2 to 1.2)
Brazil	2018	1127.5 (1052.2 to 1209.1)	375.8 (335.2 to 413.1)	493.0 (446.6 to 546.4)	258.0 (216.9 to 302.9)	0.6 (0.6 to 0.7)	1886.1 (1760.1 to 2022.6)	628.7 (560.8 to 691.0)	824.7 (747.1 to 914.0)	431.6 (362.8 to 506.7)	1.1 (1.1 to 1.1)
Brazil	2019	1143.2 (1063.6 to 1224.1)	382.1 (340.7 to 420.2)	497.7 (451.2 to 551.6)	262.7 (220.9 to 307.8)	0.7 (0.6 to 0.7)	1912.3 (1779.2 to 2047.6)	639.3 (569.9 to 702.9)	832.6 (754.8 to 922.8)	439.4 (369.6 to 514.9)	1.1 (1.0 to 1.2)
Brazil	2020	1157.7 (1079.6 to 1240.8)	388.2 (347.2 to 426.4)	501.8 (454.4 to 556.1)	267.0 (224.3 to 313.6)	0.7 (0.6 to 0.7)	1936.6 (1806.1 to 2075.6)	649.4 (580.8 to 713.4)	839.5 (760.2 to 930.2)	446.6 (375.2 to 524.5)	1.1 (1.0 to 1.2)
Brazil	2021	1171.7 (1089.1 to 1253.4)	394.2 (352.0 to 433.1)	505.6 (457.1 to 560.8)	271.2 (227.5 to 318.5)	0.7 (0.6 to 0.7)	1960.0 (1821.9 to 2096.8)	659.4 (588.9 to 724.5)	845.8 (764.6 to 938.1)	453.6 (380.5 to 532.8)	1.1 (1.0 to 1.3)
Brazil	2022	1185.1 (1104.6 to 1270.1)	400.0 (356.8 to 439.8)	509.2 (460.1 to 564.3)	275.3 (231.3 to 323.4)	0.7 (0.6 to 0.8)	1982.6 (1847.9 to 2124.6)	669.1 (596.9 to 735.8)	851.8 (769.7 to 944.0)	460.5 (386.9 to 540.9)	1.1 (1.0 to 1.3)
Brazil	2023	1197.6 (1113.0 to 1282.0)	405.5 (361.3 to 446.4)	512.2 (462.9 to 568.5)	279.2 (234.2 to 328.6)	0.7 (0.6 to 0.8)	2003.4 (1861.9 to 2144.6)	678.3 (604.4 to 746.8)	856.9 (774.3 to 951.0)	467.1 (391.7 to 549.7)	1.2 (1.0 to 1.3)
Brazil	2024	1209.7 (1127.3 to 1296.4)	410.9 (365.3 to 452.7)	515.1 (465.5 to 570.5)	283.0 (237.0 to 332.7)	0.7 (0.6 to 0.8)	2023.7 (1885.8 to 2168.6)	687.3 (611.1 to 757.4)	861.7 (778.7 to 954.3)	473.5 (396.4 to 556.5)	1.2 (1.0 to 1.4)
Brazil	2025	1220.7 (1134.0 to 1306.3)	415.9 (369.7 to 458.3)	517.4 (467.2 to 571.8)	286.6 (240.4 to 336.9)	0.7 (0.6 to 0.9)	2042.0 (1896.9 to 2185.2)	695.7 (618.5 to 766.6)	865.6 (781.5 to 956.5)	479.5 (402.2 to 563.6)	1.2 (1.0 to 1.5)
Brazil	2026	1231.4 (1147.1 to 1321.7)	420.8 (374.0 to 464.2)	519.6 (469.2 to 573.0)	290.2 (244.0 to 340.3)	0.8 (0.6 to 0.9)	2059.9 (1919.0 to 2210.9)	704.0 (625.7 to 776.5)	869.2 (784.9 to 958.5)	485.5 (408.1 to 569.3)	1.3 (1.1 to 1.5)
Brazil	2027	1241.4 (1152.0 to 1329.2)	425.5 (378.6 to 470.3)	521.5 (470.5 to 574.6)	293.6 (246.7 to 345.0)	0.8 (0.6 to 0.9)	2076.7 (1927.0 to 2223.5)	711.9 (633.4 to 786.7)	872.3 (787.1 to 961.3)	491.2 (412.7 to 577.1)	1.3 (1.1 to 1.6)
Brazil	2028	1250.6 (1164.5 to 1342.3)	430.0 (383.0 to 475.2)	522.9 (471.3 to 577.0)	296.9 (249.0 to 349.5)	0.8 (0.7 to 1.0)	2092.0 (1948.0 to 2245.5)	719.3 (640.8 to 794.9)	874.8 (788.5 to 965.2)	496.6 (416.6 to 584.6)	1.3 (1.1 to 1.6)
Brazil	2029	1260.0 (1167.8 to 1351.3)	434.5 (386.7 to 480.9)	524.5 (472.6 to 578.7)	300.2 (251.5 to 353.7)	0.8 (0.7 to 1.0)	2107.8 (1953.5 to 2260.6)	726.9 (646.9 to 804.4)	877.4 (790.5 to 968.1)	502.2 (420.7 to 591.7)	1.4 (1.1 to 1.7)
Brazil	2030	1269.8 (1180.9 to 1362.8)	439.2 (390.0 to 486.9)	526.2 (473.0 to 580.4)	303.6 (253.9 to 357.5)	0.8 (0.7 to 1.1)	2124.2 (1975.4 to 2279.7)	734.7 (652.3 to 814.5)	880.2 (791.2 to 971.0)	507.9 (424.7 to 598.1)	1.4 (1.1 to 1.8)
Brazil	2031	1279.6 (1184.9 to 1373.8)	443.9 (393.2 to 493.2)	527.8 (473.9 to 583.0)	307.0 (256.3 to 361.4)	0.9 (0.0 to 1.1)	2140.6 (1982.1 to 2298.1)	742.6 (657.8 to 825.1)	883.0 (792.8 to 975.3)	513.6 (428.8 to 604.6)	1.4 (0.0 to 1.8)
Brazil	2032	1290.3 (1198.2 to 1388.0)	448.9 (398.0 to 500.8)	529.8 (475.6 to 586.6)	310.7 (259.3 to 365.5)	0.9 (0.0 to 1.1)	2158.5 (2004.4 to 2321.8)	751.0 (665.8 to 837.7)	886.3 (795.6 to 981.3)	519.8 (433.7 to 611.4)	1.5 (0.0 to 1.9)
Brazil	2033	1301.3 (1201.2 to 1398.9)	454.0 (402.9 to 507.8)	531.9 (477.1 to 590.7)	314.5 (262.3 to 369.7)	0.9 (0.0 to 1.2)	2176.8 (2009.4 to 2340.2)	759.6 (674.0 to 849.5)	889.7 (798.1 to 988.1)	526.0 (438.8 to 618.5)	1.5 (0.0 to 2.0)
Brazil	2034	1312.1 (1215.9 to 1413.4)	459.1 (407.1 to 515.5)	533.8 (477.3 to 593.7)	318.2 (265.5 to 374.9)	0.9 (0.0 to 1.2)	2194.9 (2034.1 to 2364.3)	768.1 (681.1 to 862.3)	893.0 (798.4 to 993.2)	532.3 (444.1 to 627.1)	1.5 (0.0 to 2.1)
Brazil	2035	1322.8 (1218.4 to 1423.9)	464.2 (410.6 to 523.1)	535.7 (478.1 to 596.5)	321.9 (268.4 to 379.4)	0.9 (0.0 to 1.3)	2212.9 (2038.1 to 2382.0)	776.6 (686.8 to 875.0)	896.2 (799.8 to 997.8)	538.5 (449.0 to 634.7)	1.5 (0.0 to 2.2)
Brazil	2036	1334.1 (1235.5 to 1441.2)	469.6 (414.2 to 530.9)	537.8 (479.6 to 599.6)	325.8 (271.5 to 384.4)	0.9 (0.0 to 1.4)	2231.7 (2066.8 to 2410.9)	785.5 (692.8 to 888.2)	899.6 (802.3 to 1003.1)	545.0 (454.2 to 643.0)	1.5 (0.0 to 2.3)

		2018 US Dollars per capita					2018 PPP per capita				
Country	Year	Health spending	Government health spending	Out-of-pocket health spending	Prepaid private health spending	Development assistance for health	Health spending	Government health spending	Out-of-pocket health spending	Prepaid private health spending	Development assistance for health
Brazil	2037	1345.7 (1237.9 to 1451.6)	475.0 (417.9 to 537.7)	539.9 (480.9 to 603.1)	329.8 (274.6 to 390.1)	0.9 (0.0 to 1.4)	2251.1 (2070.9 to 2428.3)	794.6 (699.0 to 899.4)	903.2 (804.4 to 1008.8)	551.7 (459.3 to 652.6)	1.6 (0.0 to 2.3)
Brazil	2038	1357.3 (1254.8 to 1468.3)	480.5 (422.3 to 545.9)	542.1 (482.4 to 606.1)	333.8 (278.0 to 395.8)	0.9 (0.0 to 1.4)	2270.6 (2099.1 to 2456.2)	803.8 (706.4 to 913.2)	906.8 (807.0 to 1013.9)	558.4 (465.1 to 662.1)	1.6 (0.0 to 2.4)
Brazil	2039	1368.0 (1257.1 to 1477.0)	485.6 (425.1 to 553.7)	543.8 (483.7 to 608.3)	337.6 (281.1 to 401.2)	0.9 (0.0 to 1.5)	2288.4 (2103.0 to 2470.7)	812.4 (711.1 to 926.2)	909.7 (809.2 to 1017.5)	564.8 (470.2 to 671.2)	1.6 (0.0 to 2.5)
Brazil	2040	1377.9 (1270.6 to 1492.0)	490.5 (428.0 to 560.1)	545.2 (485.1 to 610.1)	341.2 (284.2 to 405.3)	0.9 (0.0 to 1.6)	2305.0 (2125.6 to 2495.9)	820.6 (716.0 to 937.0)	912.0 (811.5 to 1020.5)	570.8 (475.4 to 678.1)	1.6 (0.0 to 2.6)
Brazil	2041	1388.3 (1272.1 to 1502.2)	495.6 (431.2 to 566.8)	546.7 (486.2 to 612.1)	345.0 (287.1 to 410.8)	0.9 (0.0 to 1.7)	2322.4 (2128.0 to 2512.9)	829.1 (721.3 to 948.1)	914.6 (813.3 to 1023.9)	577.1 (480.3 to 687.2)	1.6 (0.0 to 2.8)
Brazil	2042	1398.2 (1286.0 to 1515.9)	500.5 (434.5 to 573.2)	548.0 (486.6 to 613.9)	348.7 (290.0 to 416.1)	0.9 (0.0 to 1.7)	2338.9 (2151.3 to 2535.8)	837.3 (726.8 to 958.9)	916.8 (814.0 to 1026.9)	583.2 (485.1 to 696.1)	1.6 (0.0 to 2.9)
Brazil	2043	1408.1 (1286.2 to 1526.2)	505.5 (437.6 to 579.8)	549.3 (486.9 to 616.1)	352.3 (292.5 to 420.5)	0.9 (0.0 to 1.8)	2355.6 (2151.6 to 2553.1)	845.7 (732.0 to 969.9)	918.9 (814.6 to 1030.6)	589.4 (489.2 to 703.5)	1.6 (0.0 to 3.0)
Brazil	2044	1417.3 (1299.7 to 1539.3)	510.2 (440.5 to 586.2)	550.3 (487.4 to 617.9)	355.8 (294.5 to 425.4)	0.9 (0.0 to 1.9)	2370.9 (2174.1 to 2574.9)	853.5 (736.8 to 980.6)	920.5 (815.3 to 1033.7)	595.3 (492.7 to 711.6)	1.6 (0.0 to 3.1)
Brazil	2045	1425.2 (1299.1 to 1547.9)	514.4 (443.2 to 593.4)	550.8 (487.5 to 619.3)	359.1 (296.3 to 429.9)	1.0 (0.0 to 2.0)	2384.2 (2173.1 to 2589.4)	860.5 (741.5 to 992.6)	921.4 (815.5 to 1036.0)	600.7 (495.7 to 719.1)	1.6 (0.0 to 3.3)
Brazil	2046	1433.9 (1311.7 to 1560.8)	518.9 (445.6 to 601.4)	551.5 (487.8 to 620.9)	362.5 (298.2 to 434.6)	1.0 (0.0 to 2.0)	2398.7 (2194.3 to 2610.9)	868.1 (745.4 to 1006.0)	922.6 (816.0 to 1038.7)	606.4 (498.9 to 727.1)	1.6 (0.0 to 3.4)
Brazil	2047	1441.2 (1311.8 to 1567.0)	522.9 (447.8 to 608.9)	551.8 (486.9 to 622.0)	365.6 (299.9 to 438.9)	1.0 (0.0 to 2.2)	2411.0 (2194.5 to 2621.4)	874.8 (749.1 to 1018.6)	923.0 (814.4 to 1040.5)	611.6 (501.7 to 734.3)	1.6 (0.0 to 3.6)
Brazil	2048	1448.9 (1323.8 to 1579.3)	527.0 (450.1 to 616.6)	552.1 (486.7 to 622.1)	368.8 (301.7 to 442.8)	1.0 (0.0 to 2.2)	2423.9 (2214.5 to 2642.0)	881.7 (753.0 to 1031.5)	923.6 (814.2 to 1040.7)	617.0 (504.6 to 740.8)	1.6 (0.0 to 3.7)
Brazil	2049	1456.2 (1322.6 to 1587.7)	531.0 (452.0 to 624.1)	552.3 (486.4 to 622.6)	371.9 (303.6 to 446.7)	1.0 (0.0 to 2.3)	2435.9 (2212.4 to 2655.9)	888.2 (756.1 to 1044.0)	923.9 (813.6 to 1041.6)	622.2 (507.9 to 747.3)	1.7 (0.0 to 3.9)
Brazil	2050	1463.3 (1333.8 to 1598.6)	534.8 (453.8 to 631.6)	552.4 (486.0 to 624.0)	375.0 (305.0 to 451.0)	1.0 (0.0 to 2.5)	2447.9 (2231.2 to 2674.2)	894.7 (759.1 to 1056.6)	924.1 (813.0 to 1043.9)	627.4 (510.2 to 754.4)	1.7 (0.0 to 4.1)
Brunei	1995	1203.8 (1110.7 to 1299.5)	902.8 (820.3 to 985.3)	276.2 (242.9 to 314.4)	24.8 (11.1 to 46.4)	0.0 (0.0 to 0.0)	2994.0 (2762.4 to 3231.9)	2245.2 (2040.0 to 2450.5)	687.0 (604.1 to 781.9)	61.8 (27.7 to 115.4)	0.0 (0.0 to 0.0)
Brunei	1996	1363.4 (1282.4 to 1446.4)	1048.2 (976.1 to 1125.2)	287.7 (257.2 to 319.5)	27.6 (12.6 to 50.5)	0.0 (0.0 to 0.0)	3390.8 (3189.5 to 3597.3)	2606.9 (2427.6 to 2798.5)	715.4 (639.6 to 794.6)	68.5 (31.4 to 125.7)	0.0 (0.0 to 0.0)
Brunei	1997	1361.8 (1290.2 to 1438.7)	1060.3 (994.4 to 1127.0)	274.4 (248.3 to 300.6)	27.1 (12.3 to 50.6)	0.0 (0.0 to 0.0)	3386.9 (3208.9 to 3578.2)	2637.0 (2473.1 to 2803.0)	682.4 (617.6 to 747.5)	67.5 (30.6 to 125.9)	0.0 (0.0 to 0.0)
Brunei	1998	1357.5 (1289.1 to 1429.3)	1075.1 (1010.9 to 1141.4)	255.4 (231.5 to 279.7)	27.0 (12.4 to 48.9)	0.0 (0.0 to 0.0)	3376.1 (3206.1 to 3554.7)	2673.8 (2514.1 to 2838.7)	635.2 (575.7 to 695.6)	67.1 (30.7 to 121.6)	0.0 (0.0 to 0.0)
Brunei	1999	1283.1 (1212.3 to 1351.4)	1032.3 (969.7 to 1093.3)	225.0 (204.1 to 246.2)	25.9 (12.1 to 47.3)	0.0 (0.0 to 0.0)	3191.1 (3015.0 to 3361.0)	2567.3 (2411.7 to 2719.1)	559.5 (507.5 to 612.3)	64.3 (30.2 to 117.6)	0.0 (0.0 to 0.0)
Brunei	2000	1173.7 (1112.2 to 1234.8)	957.6 (898.7 to 1015.9)	191.9 (174.9 to 208.9)	24.3 (11.2 to 45.0)	0.0 (0.0 to 0.0)	2919.0 (2766.1 to 3070.9)	2381.5 (2235.1 to 2526.6)	477.1 (435.0 to 519.5)	60.4 (28.0 to 111.8)	0.0 (0.0 to 0.0)
Brunei	2001	1116.5 (1056.3 to 1183.0)	910.7 (852.9 to 972.1)	181.7 (166.2 to 197.5)	24.2 (11.2 to 44.7)	0.0 (0.0 to 0.0)	2776.8 (2627.2 to 2942.2)	2264.9 (2121.1 to 2417.7)	451.8 (413.3 to 491.3)	60.2 (27.9 to 111.2)	0.0 (0.0 to 0.0)

Country	Year	2018 US Dollars per capita					2018 PPP per capita				
		Health spending	Government health spending	Out-of-pocket health spending	Prepaid private health spending	Development assistance for health	Health spending	Government health spending	Out-of-pocket health spending	Prepaid private health spending	Development assistance for health
Brunei	2002	1089.5 (1031.3 to 1153.3)	887.4 (833.7 to 946.1)	177.4 (162.7 to 193.7)	24.7 (11.5 to 45.7)	0.0 (0.0 to 0.0)	2709.5 (2564.9 to 2868.2)	2207.0 (2073.4 to 2352.9)	441.1 (404.5 to 481.7)	61.4 (28.7 to 113.7)	0.0 (0.0 to 0.0)
Brunei	2003	1075.5 (1015.0 to 1139.6)	873.6 (818.9 to 931.6)	176.2 (161.2 to 192.6)	25.7 (12.1 to 47.5)	0.0 (0.0 to 0.0)	2674.8 (2524.4 to 2834.3)	2172.7 (2036.7 to 2316.9)	438.3 (400.9 to 479.0)	63.8 (30.1 to 118.0)	0.0 (0.0 to 0.0)
Brunei	2004	1023.2 (963.7 to 1083.2)	832.3 (777.8 to 886.6)	165.2 (151.0 to 179.4)	25.7 (12.3 to 47.1)	0.0 (0.0 to 0.0)	2544.8 (2396.8 to 2693.9)	2070.0 (1934.4 to 2205.1)	410.8 (375.6 to 446.2)	64.0 (30.5 to 117.1)	0.0 (0.0 to 0.0)
Brunei	2005	950.9 (893.0 to 1011.2)	780.9 (725.3 to 837.5)	144.6 (131.8 to 158.6)	25.4 (12.1 to 46.1)	0.0 (0.0 to 0.0)	2364.9 (2220.8 to 2514.8)	1942.0 (1803.8 to 2082.8)	359.6 (327.8 to 394.5)	63.3 (30.2 to 114.8)	0.0 (0.0 to 0.0)
Brunei	2006	900.3 (845.3 to 960.3)	751.3 (698.7 to 807.1)	123.3 (111.2 to 135.8)	25.7 (12.1 to 47.8)	0.0 (0.0 to 0.0)	2239.2 (2102.4 to 2388.2)	1868.6 (1737.7 to 2007.3)	306.7 (276.5 to 337.7)	63.9 (30.1 to 118.8)	0.0 (0.0 to 0.0)
Brunei	2007	850.7 (793.8 to 906.7)	721.4 (669.0 to 773.3)	103.6 (92.7 to 114.7)	25.6 (11.8 to 49.3)	0.0 (0.0 to 0.0)	2115.7 (1974.3 to 2255.0)	1794.2 (1663.7 to 1923.1)	257.7 (230.5 to 285.4)	63.8 (29.4 to 122.7)	0.0 (0.0 to 0.0)
Brunei	2008	862.4 (802.5 to 920.5)	745.6 (688.7 to 803.0)	89.6 (79.4 to 100.1)	27.2 (12.8 to 52.4)	0.0 (0.0 to 0.0)	2144.8 (1995.9 to 2289.4)	1854.2 (1712.8 to 1997.2)	222.8 (197.6 to 249.0)	67.8 (31.8 to 130.3)	0.0 (0.0 to 0.0)
Brunei	2009	822.0 (766.6 to 881.0)	715.9 (663.0 to 771.1)	79.3 (69.4 to 89.9)	26.8 (12.6 to 50.7)	0.0 (0.0 to 0.0)	2044.2 (1906.6 to 2191.1)	1780.5 (1649.0 to 1917.6)	197.1 (172.7 to 223.5)	66.6 (31.4 to 126.1)	0.0 (0.0 to 0.0)
Brunei	2010	788.6 (734.7 to 848.7)	693.2 (642.4 to 749.7)	68.8 (60.0 to 78.9)	26.6 (12.8 to 50.0)	0.0 (0.0 to 0.0)	1961.3 (1827.3 to 2110.8)	1724.0 (1597.7 to 1864.5)	171.0 (149.3 to 196.1)	66.2 (31.8 to 124.5)	0.0 (0.0 to 0.0)
Brunei	2011	763.2 (707.4 to 820.0)	674.0 (624.9 to 726.1)	62.2 (53.9 to 72.1)	26.9 (13.0 to 50.1)	0.0 (0.0 to 0.0)	1898.0 (1759.4 to 2039.4)	1676.3 (1554.0 to 1805.8)	154.8 (134.0 to 179.4)	66.9 (32.4 to 124.5)	0.0 (0.0 to 0.0)
Brunei	2012	732.4 (676.1 to 788.8)	647.4 (598.3 to 699.5)	58.1 (49.7 to 67.7)	26.8 (12.9 to 50.5)	0.0 (0.0 to 0.0)	1821.4 (1681.5 to 1961.8)	1610.1 (1487.9 to 1739.7)	144.6 (123.7 to 168.3)	66.7 (32.0 to 125.7)	0.0 (0.0 to 0.0)
Brunei	2013	706.7 (656.0 to 762.6)	626.0 (577.3 to 676.3)	53.9 (45.7 to 63.8)	26.8 (12.8 to 50.3)	0.0 (0.0 to 0.0)	1757.7 (1631.5 to 1896.5)	1556.8 (1435.8 to 1682.0)	134.1 (113.7 to 158.7)	66.7 (31.9 to 125.2)	0.0 (0.0 to 0.0)
Brunei	2014	708.2 (654.2 to 763.4)	632.9 (582.8 to 682.5)	47.5 (39.9 to 57.0)	27.8 (13.6 to 51.6)	0.0 (0.0 to 0.0)	1761.3 (1626.9 to 1898.5)	1573.9 (1449.5 to 1697.4)	118.2 (99.4 to 141.8)	69.2 (33.8 to 128.4)	0.0 (0.0 to 0.0)
Brunei	2015	759.5 (697.1 to 828.1)	685.5 (624.0 to 748.0)	43.4 (35.7 to 53.0)	30.6 (14.9 to 56.7)	0.0 (0.0 to 0.0)	1888.8 (1733.6 to 2059.5)	1704.8 (1552.0 to 1860.3)	107.9 (88.7 to 131.8)	76.1 (37.0 to 141.1)	0.0 (0.0 to 0.0)
Brunei	2016	769.6 (693.4 to 849.0)	696.7 (625.5 to 772.1)	40.9 (32.8 to 50.7)	31.9 (15.7 to 60.1)	0.0 (0.0 to 0.0)	1914.0 (1724.5 to 2111.4)	1732.8 (1555.5 to 1920.2)	101.8 (81.6 to 126.1)	79.4 (38.9 to 149.4)	0.0 (0.0 to 0.0)
Brunei	2017	680.0 (612.9 to 749.5)	610.4 (547.8 to 676.8)	39.4 (31.7 to 48.9)	30.2 (14.7 to 56.8)	0.0 (0.0 to 0.0)	1691.2 (1524.3 to 1864.1)	1518.2 (1362.5 to 1683.1)	97.9 (78.8 to 121.6)	75.1 (36.7 to 141.2)	0.0 (0.0 to 0.0)
Brunei	2018	681.9 (613.8 to 751.5)	613.0 (549.8 to 679.3)	38.4 (30.7 to 47.6)	30.6 (14.9 to 57.6)	0.0 (0.0 to 0.0)	1696.0 (1526.6 to 1868.9)	1524.4 (1367.5 to 1689.5)	95.5 (76.5 to 118.3)	76.0 (37.2 to 143.2)	0.0 (0.0 to 0.0)
Brunei	2019	682.5 (617.0 to 752.5)	614.2 (550.9 to 680.7)	37.5 (29.9 to 46.8)	30.8 (15.1 to 57.9)	0.0 (0.0 to 0.0)	1697.3 (1534.4 to 1871.4)	1527.5 (1370.2 to 1693.0)	93.2 (74.4 to 116.5)	76.6 (37.4 to 144.0)	0.0 (0.0 to 0.0)
Brunei	2020	682.3 (616.1 to 752.4)	614.7 (550.6 to 682.0)	36.5 (29.2 to 45.6)	31.0 (15.2 to 58.2)	0.0 (0.0 to 0.0)	1696.8 (1532.3 to 1871.2)	1528.8 (1369.4 to 1696.1)	90.9 (72.7 to 113.4)	77.1 (37.7 to 144.7)	0.0 (0.0 to 0.0)
Brunei	2021	680.8 (611.2 to 752.7)	613.9 (550.4 to 681.2)	35.6 (28.7 to 44.7)	31.2 (15.2 to 58.6)	0.0 (0.0 to 0.0)	1693.1 (1520.0 to 1871.9)	1526.9 (1368.8 to 1694.2)	88.6 (71.3 to 111.1)	77.6 (37.9 to 145.7)	0.0 (0.0 to 0.0)
Brunei	2022	678.7 (611.7 to 749.4)	612.7 (549.2 to 680.6)	34.7 (27.9 to 43.4)	31.3 (15.3 to 59.0)	0.0 (0.0 to 0.0)	1687.9 (1521.4 to 1863.8)	1523.7 (1366.0 to 1692.6)	86.3 (69.4 to 107.8)	77.9 (38.1 to 146.7)	0.0 (0.0 to 0.0)

Country	Year	2018 US Dollars per capita					2018 PPP per capita				
		Health spending	Government health spending	Out-of-pocket health spending	Prepaid private health spending	Development assistance for health	Health spending	Government health spending	Out-of-pocket health spending	Prepaid private health spending	Development assistance for health
Brunei	2023	676.8 (608.0 to 748.4)	611.6 (547.6 to 680.2)	33.8 (27.2 to 42.3)	31.4 (15.4 to 59.0)	0.0 (0.0 to 0.0)	1683.2 (1512.1 to 1861.2)	1521.0 (1361.9 to 1691.7)	84.0 (67.5 to 105.2)	78.1 (38.2 to 146.8)	0.0 (0.0 to 0.0)
Brunei	2024	675.9 (606.2 to 749.9)	611.6 (547.3 to 680.9)	32.9 (26.3 to 41.1)	31.5 (15.4 to 59.0)	0.0 (0.0 to 0.0)	1681.0 (1507.5 to 1865.0)	1521.0 (1361.2 to 1693.5)	81.7 (65.4 to 102.3)	78.3 (38.3 to 146.7)	0.0 (0.0 to 0.0)
Brunei	2025	673.9 (604.9 to 745.0)	610.4 (545.3 to 680.4)	32.0 (25.6 to 40.1)	31.5 (15.4 to 58.9)	0.0 (0.0 to 0.0)	1676.0 (1504.4 to 1852.9)	1518.1 (1356.3 to 1692.2)	79.5 (63.6 to 99.7)	78.4 (38.3 to 146.6)	0.0 (0.0 to 0.0)
Brunei	2026	673.3 (603.9 to 746.5)	610.7 (545.8 to 681.4)	31.1 (24.7 to 39.0)	31.6 (15.4 to 58.9)	0.0 (0.0 to 0.0)	1674.6 (1501.9 to 1856.5)	1518.7 (1357.5 to 1694.7)	77.3 (61.5 to 97.1)	78.6 (38.4 to 146.4)	0.0 (0.0 to 0.0)
Brunei	2027	671.5 (599.3 to 747.7)	609.7 (545.4 to 680.6)	30.2 (23.9 to 37.8)	31.6 (15.4 to 58.8)	0.0 (0.0 to 0.0)	1670.0 (1490.4 to 1859.6)	1516.2 (1356.5 to 1692.6)	75.1 (59.5 to 93.9)	78.6 (38.4 to 146.1)	0.0 (0.0 to 0.0)
Brunei	2028	668.5 (598.2 to 741.2)	607.4 (543.6 to 678.5)	29.4 (23.1 to 36.9)	31.6 (15.5 to 58.7)	0.0 (0.0 to 0.0)	1662.6 (1487.9 to 1843.5)	1510.7 (1352.0 to 1687.3)	73.2 (57.5 to 91.9)	78.7 (38.4 to 145.9)	0.0 (0.0 to 0.0)
Brunei	2029	665.9 (595.4 to 742.6)	605.5 (541.5 to 678.1)	28.7 (22.4 to 36.6)	31.7 (15.5 to 58.6)	0.0 (0.0 to 0.0)	1656.1 (1480.9 to 1846.8)	1505.9 (1346.6 to 1686.3)	71.4 (55.7 to 91.0)	78.7 (38.5 to 145.6)	0.0 (0.0 to 0.0)
Brunei	2030	662.2 (591.2 to 738.6)	602.4 (537.4 to 676.7)	28.1 (21.8 to 35.9)	31.7 (15.5 to 58.5)	0.0 (0.0 to 0.0)	1646.9 (1470.2 to 1836.8)	1498.3 (1336.5 to 1683.0)	69.8 (54.1 to 89.4)	78.8 (38.5 to 145.5)	0.0 (0.0 to 0.0)
Brunei	2031	659.2 (586.6 to 737.3)	600.0 (534.3 to 674.1)	27.5 (21.1 to 35.3)	31.7 (15.5 to 58.5)	0.0 (0.0 to 0.0)	1639.5 (1458.9 to 1833.6)	1492.3 (1328.8 to 1676.6)	68.4 (52.6 to 87.7)	78.8 (38.5 to 145.5)	0.0 (0.0 to 0.0)
Brunei	2032	656.2 (583.0 to 734.4)	597.5 (531.0 to 673.0)	27.0 (20.5 to 34.8)	31.7 (15.5 to 58.5)	0.0 (0.0 to 0.0)	1632.1 (1449.9 to 1826.5)	1486.1 (1320.7 to 1673.7)	67.1 (51.0 to 86.6)	78.9 (38.5 to 145.5)	0.0 (0.0 to 0.0)
Brunei	2033	653.4 (580.9 to 733.0)	595.1 (528.0 to 671.9)	26.5 (20.0 to 34.4)	31.7 (15.5 to 58.5)	0.0 (0.0 to 0.0)	1624.9 (1444.7 to 1822.9)	1479.9 (1313.1 to 1671.1)	66.0 (49.8 to 85.5)	78.9 (38.6 to 145.5)	0.0 (0.0 to 0.0)
Brunei	2034	650.5 (575.9 to 732.8)	592.5 (524.6 to 670.9)	26.2 (19.5 to 34.0)	31.8 (15.5 to 58.6)	0.0 (0.0 to 0.0)	1617.7 (1432.2 to 1822.4)	1473.7 (1304.7 to 1668.5)	65.0 (48.6 to 84.6)	79.0 (38.5 to 145.6)	0.0 (0.0 to 0.0)
Brunei	2035	647.9 (574.2 to 730.3)	590.3 (520.7 to 669.4)	25.8 (19.1 to 33.9)	31.8 (15.5 to 58.7)	0.0 (0.0 to 0.0)	1611.4 (1428.1 to 1816.2)	1468.1 (1294.9 to 1664.7)	64.2 (47.6 to 84.3)	79.1 (38.6 to 145.9)	0.0 (0.0 to 0.0)
Brunei	2036	645.2 (569.4 to 730.1)	587.8 (517.1 to 668.4)	25.5 (18.8 to 33.7)	31.8 (15.5 to 58.6)	0.0 (0.0 to 0.0)	1604.5 (1416.2 to 1815.7)	1461.9 (1286.1 to 1662.2)	63.5 (46.8 to 83.9)	79.1 (38.6 to 145.7)	0.0 (0.0 to 0.0)
Brunei	2037	642.9 (564.5 to 726.6)	585.7 (514.5 to 668.1)	25.3 (18.5 to 33.6)	31.9 (15.5 to 58.6)	0.0 (0.0 to 0.0)	1598.9 (1403.8 to 1807.0)	1456.7 (1279.7 to 1661.6)	62.9 (46.1 to 83.5)	79.2 (38.6 to 145.8)	0.0 (0.0 to 0.0)
Brunei	2038	639.9 (561.3 to 725.7)	582.9 (509.5 to 666.7)	25.1 (18.2 to 33.4)	31.9 (15.5 to 58.6)	0.0 (0.0 to 0.0)	1591.4 (1396.1 to 1804.9)	1449.7 (1267.2 to 1658.2)	62.5 (45.3 to 83.2)	79.2 (38.6 to 145.8)	0.0 (0.0 to 0.0)
Brunei	2039	637.1 (558.1 to 723.8)	580.2 (505.6 to 666.1)	25.0 (17.9 to 33.5)	31.9 (15.5 to 58.9)	0.0 (0.0 to 0.0)	1584.4 (1388.1 to 1800.2)	1443.0 (1257.5 to 1656.6)	62.1 (44.5 to 83.4)	79.3 (38.6 to 146.4)	0.0 (0.0 to 0.0)
Brunei	2040	634.2 (555.8 to 721.6)	577.5 (501.8 to 664.6)	24.9 (17.7 to 33.5)	31.9 (15.5 to 58.8)	0.0 (0.0 to 0.0)	1577.3 (1382.3 to 1794.7)	1436.2 (1248.0 to 1653.0)	61.9 (44.0 to 83.3)	79.2 (38.6 to 146.2)	0.0 (0.0 to 0.0)
Brunei	2041	631.9 (552.4 to 722.2)	575.2 (498.5 to 663.5)	24.8 (17.6 to 33.7)	31.9 (15.5 to 58.9)	0.0 (0.0 to 0.0)	1571.5 (1373.8 to 1796.2)	1430.5 (1239.7 to 1650.2)	61.8 (43.7 to 83.7)	79.3 (38.6 to 146.6)	0.0 (0.0 to 0.0)
Brunei	2042	629.7 (547.7 to 719.6)	573.0 (495.5 to 663.2)	24.8 (17.5 to 33.7)	31.9 (15.5 to 59.1)	0.0 (0.0 to 0.0)	1566.1 (1362.1 to 1789.7)	1425.1 (1232.3 to 1649.4)	61.8 (43.6 to 83.9)	79.3 (38.6 to 147.0)	0.0 (0.0 to 0.0)
Brunei	2043	626.9 (541.1 to 719.8)	570.1 (491.3 to 662.1)	24.9 (17.5 to 34.0)	31.9 (15.5 to 59.2)	0.0 (0.0 to 0.0)	1559.0 (1345.7 to 1790.1)	1417.9 (1221.9 to 1646.6)	61.9 (43.4 to 84.6)	79.3 (38.6 to 147.2)	0.0 (0.0 to 0.0)

		2018 US Dollars per capita					2018 PPP per capita				
Country	Year	Health spending	Government health spending	Out-of-pocket health spending	Prepaid private health spending	Development assistance for health	Health spending	Government health spending	Out-of-pocket health spending	Prepaid private health spending	Development assistance for health
Brunei	2044	624.2 (537.5 to 714.4)	567.4 (487.1 to 661.1)	25.0 (17.5 to 34.5)	31.9 (15.5 to 59.1)	0.0 (0.0 to 0.0)	1552.4 (1336.8 to 1776.8)	1411.0 (1211.5 to 1644.2)	62.1 (43.4 to 85.8)	79.3 (38.6 to 147.0)	0.0 (0.0 to 0.0)
Brunei	2045	621.3 (535.8 to 716.2)	564.4 (482.3 to 659.4)	25.1 (17.5 to 35.1)	31.9 (15.5 to 59.0)	0.0 (0.0 to 0.0)	1545.3 (1332.5 to 1781.2)	1403.6 (1199.5 to 1639.9)	62.4 (43.6 to 87.3)	79.3 (38.6 to 146.7)	0.0 (0.0 to 0.0)
Brunei	2046	618.9 (530.5 to 713.3)	561.8 (478.3 to 658.5)	25.3 (17.6 to 35.8)	31.9 (15.5 to 58.8)	0.0 (0.0 to 0.0)	1539.3 (1319.4 to 1774.1)	1397.1 (1189.4 to 1637.8)	62.9 (43.8 to 88.9)	79.3 (38.5 to 146.3)	0.0 (0.0 to 0.0)
Brunei	2047	616.6 (529.9 to 712.6)	559.2 (474.1 to 657.7)	25.5 (17.7 to 36.6)	31.9 (15.5 to 59.0)	0.0 (0.0 to 0.0)	1533.5 (1317.9 to 1772.2)	1390.8 (1179.1 to 1635.7)	63.5 (44.0 to 91.0)	79.2 (38.5 to 146.8)	0.0 (0.0 to 0.0)
Brunei	2048	614.6 (523.7 to 711.0)	557.0 (471.5 to 657.2)	25.8 (17.8 to 37.0)	31.9 (15.5 to 59.2)	0.0 (0.0 to 0.0)	1528.5 (1302.5 to 1768.2)	1385.2 (1172.6 to 1634.6)	64.1 (44.4 to 92.1)	79.2 (38.5 to 147.3)	0.0 (0.0 to 0.0)
Brunei	2049	612.6 (519.8 to 712.9)	554.7 (468.8 to 656.6)	26.0 (18.0 to 37.8)	31.9 (15.5 to 59.4)	0.0 (0.0 to 0.0)	1523.4 (1292.8 to 1773.1)	1379.5 (1166.0 to 1632.9)	64.7 (44.8 to 94.1)	79.2 (38.5 to 147.8)	0.0 (0.0 to 0.0)
Brunei	2050	610.8 (519.8 to 711.0)	552.7 (466.2 to 656.1)	26.2 (18.2 to 38.6)	31.9 (15.5 to 59.5)	0.0 (0.0 to 0.0)	1519.0 (1292.7 to 1768.2)	1374.5 (1159.4 to 1631.7)	65.3 (45.2 to 96.1)	79.2 (38.5 to 148.1)	0.0 (0.0 to 0.0)
Bulgaria	1995	186.1 (166.0 to 211.3)	133.0 (114.6 to 154.0)	52.5 (42.4 to 64.7)	0.5 (0.2 to 1.0)	0.1 (0.1 to 0.1)	488.0 (435.3 to 554.3)	348.7 (300.6 to 404.0)	137.8 (111.2 to 169.6)	1.3 (0.6 to 2.5)	0.1 (0.1 to 0.1)
Bulgaria	1996	174.4 (157.3 to 195.1)	121.3 (105.7 to 138.8)	52.6 (43.2 to 63.5)	0.4 (0.2 to 0.8)	0.0 (0.0 to 0.0)	457.4 (412.6 to 511.7)	318.3 (277.3 to 364.1)	137.9 (113.3 to 166.5)	1.2 (0.5 to 2.2)	0.1 (0.1 to 0.1)
Bulgaria	1997	175.8 (159.6 to 195.1)	120.3 (106.2 to 136.5)	54.7 (45.7 to 65.1)	0.4 (0.2 to 0.8)	0.3 (0.3 to 0.3)	460.9 (418.6 to 511.6)	315.6 (278.6 to 358.0)	143.5 (119.9 to 170.7)	1.1 (0.5 to 2.0)	0.7 (0.7 to 0.7)
Bulgaria	1998	197.2 (181.1 to 215.9)	129.1 (115.5 to 145.3)	62.9 (53.4 to 73.7)	0.4 (0.2 to 0.8)	4.7 (4.7 to 4.7)	517.1 (475.0 to 566.3)	338.7 (302.9 to 381.0)	165.0 (139.9 to 193.2)	1.1 (0.5 to 2.1)	12.3 (12.3 to 12.3)
Bulgaria	1999	214.9 (198.3 to 234.3)	137.9 (124.1 to 153.5)	76.3 (65.6 to 88.4)	0.5 (0.2 to 0.8)	0.3 (0.3 to 0.3)	563.7 (520.1 to 614.5)	361.7 (325.3 to 402.7)	200.1 (171.9 to 231.7)	1.2 (0.6 to 2.1)	0.7 (0.7 to 0.7)
Bulgaria	2000	248.5 (230.4 to 267.9)	152.2 (137.3 to 168.1)	94.0 (81.5 to 108.0)	0.5 (0.2 to 0.9)	1.8 (1.8 to 1.8)	651.8 (604.3 to 702.7)	399.1 (360.0 to 440.7)	246.6 (213.8 to 283.2)	1.3 (0.6 to 2.4)	4.8 (4.8 to 4.8)
Bulgaria	2001	289.0 (268.2 to 310.2)	171.1 (154.7 to 187.5)	115.5 (102.1 to 131.9)	1.1 (0.5 to 1.9)	1.4 (1.4 to 1.4)	758.0 (703.4 to 813.5)	448.8 (405.7 to 491.7)	302.8 (267.8 to 346.0)	2.8 (1.3 to 5.0)	3.6 (3.6 to 3.6)
Bulgaria	2002	327.6 (304.4 to 352.2)	193.0 (175.4 to 211.3)	130.6 (115.9 to 147.2)	2.3 (1.1 to 4.1)	1.8 (1.8 to 1.8)	859.2 (798.3 to 923.8)	506.1 (460.1 to 554.1)	342.4 (304.0 to 386.2)	5.9 (2.8 to 10.8)	4.7 (4.7 to 4.7)
Bulgaria	2003	351.5 (326.5 to 377.6)	207.2 (189.7 to 227.2)	139.5 (124.2 to 156.3)	3.6 (1.7 to 6.7)	1.1 (1.1 to 1.1)	921.8 (856.4 to 990.4)	543.5 (497.5 to 595.9)	365.8 (325.7 to 409.9)	9.5 (4.5 to 17.5)	2.9 (2.9 to 2.9)
Bulgaria	2004	372.6 (346.6 to 400.0)	216.9 (198.5 to 236.9)	147.3 (130.5 to 164.6)	4.8 (2.2 to 8.7)	3.7 (3.7 to 3.7)	977.3 (909.1 to 1049.1)	568.8 (520.7 to 621.3)	386.2 (342.4 to 431.6)	12.6 (5.9 to 22.7)	9.7 (9.7 to 9.7)
Bulgaria	2005	389.3 (363.1 to 417.6)	223.1 (204.1 to 244.2)	155.4 (139.5 to 173.0)	5.8 (2.7 to 10.5)	5.0 (5.0 to 5.0)	1021.0 (952.4 to 1095.3)	585.2 (535.3 to 640.5)	407.5 (365.8 to 453.7)	15.3 (7.2 to 27.4)	13.0 (13.0 to 13.0)
Bulgaria	2006	399.4 (373.0 to 427.5)	226.1 (206.6 to 246.1)	165.1 (148.7 to 183.2)	6.6 (3.2 to 12.0)	1.6 (1.6 to 1.6)	1047.6 (978.3 to 1121.3)	593.0 (541.9 to 645.4)	433.1 (390.0 to 480.4)	17.3 (8.3 to 31.6)	4.1 (4.1 to 4.1)
Bulgaria	2007	429.6 (403.0 to 458.7)	236.2 (216.9 to 257.9)	175.9 (158.0 to 195.0)	7.3 (3.5 to 13.5)	10.3 (10.3 to 10.3)	1126.8 (1056.8 to 1203.0)	619.4 (568.8 to 676.5)	461.2 (414.5 to 511.5)	19.3 (9.1 to 35.3)	26.9 (26.9 to 26.9)
Bulgaria	2008	451.6 (423.1 to 483.3)	249.5 (229.5 to 271.2)	190.3 (172.8 to 211.1)	8.3 (3.9 to 15.4)	3.5 (3.5 to 3.5)	1184.4 (1109.7 to 1267.6)	654.4 (601.8 to 711.2)	499.0 (453.1 to 553.5)	21.8 (10.3 to 40.3)	9.2 (9.2 to 9.2)

		2018 US Dollars per capita					2018 PPP per capita				
Country	Year	Health spending	Government health spending	Out-of-pocket health spending	Prepaid private health spending	Development assistance for health	Health spending	Government health spending	Out-of-pocket health spending	Prepaid private health spending	Development assistance for health
Bulgaria	2009	477.9 (449.8 to 509.4)	250.6 (231.3 to 272.5)	200.4 (182.6 to 221.1)	8.4 (4.0 to 15.5)	18.5 (18.5 to 18.5)	1253.3 (1179.7 to 1336.0)	657.2 (606.6 to 714.6)	525.5 (478.8 to 579.9)	22.0 (10.4 to 40.8)	48.6 (48.6 to 48.6)
Bulgaria	2010	489.2 (459.1 to 520.9)	263.0 (243.1 to 286.1)	215.5 (195.6 to 236.3)	8.5 (4.0 to 15.8)	2.3 (2.3 to 2.3)	1283.0 (1204.0 to 1366.0)	689.8 (637.5 to 750.3)	565.1 (513.1 to 619.8)	22.2 (10.5 to 41.6)	5.9 (5.9 to 5.9)
Bulgaria	2011	519.7 (488.8 to 554.0)	275.7 (254.1 to 300.0)	233.8 (212.6 to 255.3)	8.4 (4.1 to 15.7)	1.8 (1.8 to 1.8)	1362.9 (1282.0 to 1453.0)	723.0 (666.5 to 786.9)	613.1 (557.5 to 669.6)	22.1 (10.8 to 41.3)	4.7 (4.7 to 4.7)
Bulgaria	2012	549.6 (517.1 to 583.8)	285.1 (263.5 to 309.6)	254.8 (231.8 to 277.0)	8.6 (4.3 to 15.9)	1.0 (1.0 to 1.0)	1441.4 (1356.2 to 1531.1)	747.8 (691.0 to 812.0)	668.3 (607.9 to 726.5)	22.6 (11.2 to 41.6)	2.6 (2.6 to 2.6)
Bulgaria	2013	584.1 (552.7 to 620.1)	300.7 (278.7 to 325.0)	273.7 (248.6 to 297.4)	9.0 (4.5 to 16.1)	0.6 (0.6 to 0.6)	1531.8 (1449.5 to 1626.2)	788.8 (730.9 to 852.3)	717.8 (652.0 to 780.0)	23.6 (11.8 to 42.3)	1.7 (1.7 to 1.7)
Bulgaria	2014	623.0 (586.9 to 659.7)	319.9 (295.6 to 345.4)	292.7 (266.9 to 318.3)	9.5 (4.8 to 17.4)	1.0 (1.0 to 1.0)	1634.0 (1539.2 to 1730.1)	839.0 (775.3 to 906.0)	767.6 (700.1 to 834.8)	24.9 (12.6 to 45.5)	2.5 (2.5 to 2.5)
Bulgaria	2015	652.6 (609.1 to 692.0)	332.9 (305.7 to 363.6)	308.7 (279.6 to 338.8)	10.0 (4.9 to 18.4)	1.0 (1.0 to 1.0)	1711.5 (1597.5 to 1815.0)	873.1 (801.7 to 953.7)	809.6 (733.3 to 888.6)	26.3 (12.9 to 48.2)	2.6 (2.6 to 2.6)
Bulgaria	2016	680.8 (630.3 to 733.0)	346.3 (312.1 to 383.0)	323.0 (286.5 to 367.0)	10.6 (5.2 to 19.2)	1.0 (1.0 to 1.0)	1785.5 (1653.0 to 1922.4)	908.2 (818.6 to 1004.4)	847.1 (751.5 to 962.6)	27.7 (13.5 to 50.3)	2.5 (2.5 to 2.5)
Bulgaria	2017	714.6 (661.3 to 774.9)	363.9 (327.2 to 403.8)	338.9 (299.8 to 384.2)	11.1 (5.4 to 20.1)	0.7 (0.7 to 0.7)	1874.1 (1734.3 to 2032.2)	954.3 (858.2 to 1059.1)	888.9 (786.4 to 1007.6)	29.0 (14.2 to 52.8)	1.8 (1.8 to 1.8)
Bulgaria	2018	734.9 (678.8 to 793.6)	371.4 (333.3 to 411.3)	351.5 (310.8 to 400.5)	11.3 (5.6 to 20.5)	0.7 (0.7 to 0.7)	1927.4 (1780.2 to 2081.5)	974.1 (874.3 to 1078.8)	921.9 (815.1 to 1050.4)	29.7 (14.6 to 53.9)	1.7 (1.7 to 1.7)
Bulgaria	2019	754.6 (698.2 to 813.0)	378.1 (339.4 to 418.9)	364.3 (322.0 to 413.1)	11.6 (5.7 to 20.9)	0.7 (0.6 to 0.7)	1979.1 (1831.1 to 2132.2)	991.6 (890.0 to 1098.7)	955.4 (844.5 to 1083.4)	30.3 (14.9 to 54.9)	1.8 (1.6 to 1.9)
Bulgaria	2020	775.0 (717.3 to 839.0)	384.7 (344.8 to 426.6)	377.8 (335.0 to 427.7)	11.8 (5.8 to 21.4)	0.7 (0.6 to 0.7)	2032.5 (1881.2 to 2200.5)	1008.9 (904.3 to 1118.8)	990.8 (878.7 to 1121.7)	31.0 (15.2 to 56.1)	1.8 (1.7 to 2.0)
Bulgaria	2021	796.1 (737.8 to 859.7)	391.7 (352.0 to 434.4)	391.6 (346.2 to 441.8)	12.1 (5.9 to 21.8)	0.7 (0.6 to 0.8)	2088.0 (1935.0 to 2254.7)	1027.4 (923.2 to 1139.2)	1027.1 (908.1 to 1158.8)	31.6 (15.5 to 57.3)	1.9 (1.7 to 2.1)
Bulgaria	2022	819.2 (758.1 to 884.1)	399.8 (359.7 to 442.7)	406.3 (359.2 to 458.0)	12.3 (6.0 to 22.3)	0.7 (0.7 to 0.8)	2148.4 (1988.3 to 2318.8)	1048.5 (943.5 to 1161.1)	1065.7 (942.0 to 1201.1)	32.3 (15.9 to 58.6)	2.0 (1.7 to 2.2)
Bulgaria	2023	843.8 (777.9 to 912.5)	408.6 (367.4 to 452.6)	421.8 (372.8 to 477.4)	12.6 (6.2 to 22.9)	0.8 (0.7 to 0.9)	2213.0 (2040.3 to 2393.3)	1071.6 (963.7 to 1187.2)	1106.3 (977.7 to 1252.1)	33.1 (16.2 to 60.1)	2.0 (1.8 to 2.3)
Bulgaria	2024	869.2 (804.8 to 945.2)	417.6 (375.4 to 463.2)	437.9 (387.0 to 496.3)	12.9 (6.3 to 23.5)	0.8 (0.7 to 0.9)	2279.7 (2110.7 to 2479.0)	1095.2 (984.5 to 1214.9)	1148.4 (1015.0 to 1301.6)	33.9 (16.6 to 61.5)	2.1 (1.8 to 2.4)
Bulgaria	2025	896.1 (826.8 to 974.9)	427.2 (383.9 to 474.1)	454.8 (400.9 to 517.5)	13.3 (6.5 to 24.0)	0.8 (0.7 to 1.0)	2350.1 (2168.6 to 2556.9)	1120.3 (1006.8 to 1243.5)	1192.8 (1051.4 to 1357.3)	34.8 (17.0 to 63.0)	2.2 (1.9 to 2.6)
Bulgaria	2026	923.6 (854.8 to 1004.9)	436.8 (392.3 to 484.9)	472.3 (414.8 to 540.5)	13.6 (6.7 to 24.6)	0.9 (0.7 to 1.0)	2422.3 (2241.9 to 2635.5)	1145.5 (1028.9 to 1271.7)	1238.8 (1087.9 to 1417.7)	35.7 (17.5 to 64.5)	2.3 (1.9 to 2.7)
Bulgaria	2027	952.8 (879.2 to 1040.6)	447.1 (402.6 to 496.5)	490.8 (430.6 to 564.8)	14.0 (6.8 to 25.3)	0.9 (0.8 to 1.1)	2498.9 (2305.9 to 2729.1)	1172.6 (1055.9 to 1302.1)	1287.2 (1129.2 to 1481.4)	36.7 (17.8 to 66.4)	2.4 (2.0 to 2.8)
Bulgaria	2028	983.1 (905.0 to 1077.1)	458.1 (411.8 to 508.7)	509.7 (444.5 to 592.2)	14.4 (7.0 to 26.1)	0.9 (0.8 to 1.1)	2578.4 (2373.6 to 2825.0)	1201.5 (1079.9 to 1334.2)	1336.8 (1165.8 to 1553.2)	37.7 (18.3 to 68.3)	2.4 (2.0 to 3.0)
Bulgaria	2029	1014.4 (924.2 to 1116.5)	469.9 (421.4 to 522.9)	528.8 (456.5 to 617.1)	14.8 (7.2 to 26.8)	1.0 (0.8 to 1.2)	2660.4 (2424.0 to 2928.2)	1232.3 (1105.2 to 1371.5)	1386.9 (1197.3 to 1618.4)	38.8 (18.8 to 70.4)	2.5 (2.1 to 3.1)

Country	Year	2018 US Dollars per capita					2018 PPP per capita				
		Health spending	Government health spending	Out-of-pocket health spending	Prepaid private health spending	Development assistance for health	Health spending	Government health spending	Out-of-pocket health spending	Prepaid private health spending	Development assistance for health
Bulgaria	2030	1046.1 (952.7 to 1157.2)	481.8 (431.2 to 535.8)	548.1 (470.9 to 643.8)	15.2 (7.4 to 27.6)	1.0 (0.8 to 1.2)	2743.6 (2498.7 to 3035.0)	1263.7 (1130.8 to 1405.4)	1437.4 (1235.0 to 1688.4)	39.9 (19.3 to 72.5)	2.6 (2.2 to 3.2)
Bulgaria	2031	1077.1 (976.6 to 1191.0)	493.3 (441.5 to 550.0)	567.2 (482.8 to 671.4)	15.6 (7.6 to 28.4)	1.0 (0.8 to 1.3)	2824.9 (2561.2 to 3123.7)	1293.7 (1157.9 to 1442.5)	1487.6 (1266.3 to 1761.0)	40.9 (19.8 to 74.6)	2.7 (2.2 to 3.4)
Bulgaria	2032	1108.4 (1005.5 to 1230.3)	505.0 (452.1 to 564.5)	586.3 (497.7 to 699.8)	16.0 (7.8 to 29.2)	1.1 (0.9 to 1.4)	2907.1 (2637.2 to 3226.7)	1324.5 (1185.7 to 1480.6)	1537.8 (1305.2 to 1835.5)	42.0 (20.3 to 76.7)	2.8 (2.2 to 3.6)
Bulgaria	2033	1140.1 (1029.5 to 1267.9)	517.1 (462.3 to 579.5)	605.5 (510.0 to 729.2)	16.5 (7.9 to 30.0)	1.1 (0.9 to 1.4)	2990.3 (2700.1 to 3325.2)	1356.2 (1212.6 to 1519.9)	1588.0 (1337.5 to 1912.3)	43.2 (20.8 to 78.6)	2.9 (2.3 to 3.8)
Bulgaria	2034	1171.4 (1053.1 to 1309.9)	529.0 (472.3 to 594.4)	624.4 (521.2 to 757.8)	16.9 (8.1 to 30.7)	1.1 (0.0 to 1.5)	3072.2 (2762.0 to 3435.4)	1387.3 (1238.6 to 1558.9)	1637.6 (1367.0 to 1987.5)	44.3 (21.4 to 80.6)	3.0 (0.0 to 3.9)
Bulgaria	2035	1203.0 (1073.6 to 1354.5)	541.3 (482.6 to 609.9)	643.3 (532.3 to 785.0)	17.3 (8.3 to 31.5)	1.1 (0.0 to 1.6)	3155.2 (2815.8 to 3552.4)	1419.6 (1265.7 to 1599.6)	1687.2 (1396.0 to 2058.9)	45.5 (21.9 to 82.5)	3.0 (0.0 to 4.1)
Bulgaria	2036	1233.8 (1093.2 to 1398.5)	553.2 (493.8 to 624.5)	661.7 (543.5 to 813.8)	17.8 (8.5 to 32.2)	1.2 (0.0 to 1.7)	3236.0 (2867.0 to 3667.9)	1450.9 (1295.2 to 1637.8)	1735.4 (1425.3 to 2134.2)	46.6 (22.4 to 84.4)	3.0 (0.0 to 4.4)
Bulgaria	2037	1264.1 (1118.3 to 1441.2)	565.0 (503.4 to 638.7)	679.7 (555.0 to 841.2)	18.2 (8.7 to 32.9)	1.2 (0.0 to 1.7)	3315.3 (2933.0 to 3779.8)	1481.9 (1320.3 to 1675.1)	1782.7 (1455.5 to 2206.2)	47.7 (22.9 to 86.3)	3.1 (0.0 to 4.6)
Bulgaria	2038	1295.1 (1138.7 to 1476.5)	577.5 (512.8 to 654.1)	697.8 (565.8 to 868.3)	18.6 (8.9 to 33.8)	1.2 (0.0 to 1.8)	3396.7 (2986.4 to 3872.4)	1514.7 (1345.0 to 1715.4)	1830.0 (1484.0 to 2277.3)	48.9 (23.4 to 88.5)	3.1 (0.0 to 4.8)
Bulgaria	2039	1325.9 (1167.5 to 1511.8)	590.2 (523.4 to 670.1)	715.5 (577.2 to 895.0)	19.1 (9.1 to 34.5)	1.2 (0.0 to 1.9)	3477.6 (3062.0 to 3965.0)	1547.9 (1372.6 to 1757.4)	1876.4 (1513.8 to 2347.3)	50.1 (23.9 to 90.5)	3.1 (0.0 to 5.1)
Bulgaria	2040	1356.6 (1190.1 to 1558.3)	603.1 (535.2 to 686.6)	732.8 (588.5 to 921.2)	19.6 (9.3 to 35.4)	1.2 (0.0 to 2.0)	3557.9 (3121.4 to 4086.9)	1581.6 (1403.7 to 1800.7)	1921.8 (1543.6 to 2415.9)	51.3 (24.5 to 92.8)	3.1 (0.0 to 5.3)
Bulgaria	2041	1385.9 (1213.5 to 1592.2)	615.5 (543.9 to 701.9)	749.3 (598.5 to 946.2)	20.0 (9.5 to 36.3)	1.2 (0.0 to 2.1)	3634.9 (3182.7 to 4175.9)	1614.3 (1426.6 to 1841.0)	1965.1 (1569.6 to 2481.7)	52.5 (25.0 to 95.2)	3.0 (0.0 to 5.5)
Bulgaria	2042	1415.3 (1234.5 to 1633.9)	628.3 (555.5 to 718.3)	765.4 (609.1 to 969.9)	20.5 (9.7 to 37.2)	1.1 (0.0 to 2.2)	3712.0 (3237.8 to 4285.2)	1647.8 (1457.0 to 1883.8)	2007.5 (1597.4 to 2543.8)	53.7 (25.5 to 97.5)	3.0 (0.0 to 5.8)
Bulgaria	2043	1445.3 (1264.1 to 1667.6)	641.8 (568.5 to 735.0)	781.4 (619.8 to 993.2)	21.0 (10.0 to 38.0)	1.1 (0.0 to 2.3)	3790.6 (3315.3 to 4373.6)	1683.3 (1491.1 to 1927.7)	2049.4 (1625.5 to 2604.8)	55.0 (26.1 to 99.6)	2.9 (0.0 to 6.1)
Bulgaria	2044	1475.7 (1281.1 to 1703.9)	656.1 (580.0 to 753.1)	797.1 (630.2 to 1014.1)	21.5 (10.2 to 38.8)	1.1 (0.0 to 2.5)	3870.3 (3360.1 to 4468.8)	1720.7 (1521.3 to 1975.1)	2090.5 (1652.9 to 2659.6)	56.3 (26.7 to 101.7)	2.8 (0.0 to 6.4)
Bulgaria	2045	1504.7 (1307.3 to 1747.4)	669.9 (590.2 to 771.1)	811.8 (638.1 to 1032.4)	22.0 (10.4 to 39.7)	1.1 (0.0 to 2.5)	3946.4 (3428.6 to 4582.8)	1756.9 (1547.8 to 2022.4)	2129.0 (1673.6 to 2707.7)	57.7 (27.3 to 104.0)	2.8 (0.0 to 6.7)
Bulgaria	2046	1532.8 (1335.2 to 1779.9)	683.6 (600.0 to 787.5)	825.6 (649.5 to 1051.3)	22.5 (10.6 to 40.7)	1.0 (0.0 to 2.7)	4020.1 (3501.9 to 4668.2)	1792.9 (1573.6 to 2065.4)	2165.4 (1703.4 to 2757.3)	59.0 (27.9 to 106.7)	2.7 (0.0 to 7.1)
Bulgaria	2047	1561.3 (1352.1 to 1817.5)	698.1 (609.5 to 805.5)	839.1 (660.7 to 1071.4)	23.0 (10.9 to 41.8)	1.0 (0.0 to 2.9)	4094.8 (3546.1 to 4766.7)	1831.0 (1598.5 to 2112.5)	2200.7 (1732.7 to 2810.0)	60.4 (28.6 to 109.6)	2.6 (0.0 to 7.5)
Bulgaria	2048	1590.5 (1370.6 to 1855.2)	713.1 (621.3 to 824.6)	852.8 (670.6 to 1087.6)	23.6 (11.2 to 42.9)	1.0 (0.0 to 3.0)	4171.3 (3594.7 to 4865.7)	1870.3 (1629.5 to 2162.6)	2236.6 (1758.8 to 2852.5)	61.9 (29.3 to 112.5)	2.5 (0.0 to 7.8)
Bulgaria	2049	1619.3 (1401.7 to 1883.8)	727.9 (632.2 to 843.4)	866.3 (679.6 to 1109.2)	24.1 (11.5 to 44.0)	0.9 (0.0 to 3.2)	4247.0 (3676.3 to 4940.7)	1909.0 (1658.0 to 2212.0)	2272.1 (1782.3 to 2909.2)	63.3 (30.0 to 115.5)	2.5 (0.0 to 8.3)
Bulgaria	2050	1647.1 (1425.8 to 1915.8)	741.9 (643.3 to 861.4)	879.6 (688.0 to 1130.4)	24.7 (11.7 to 45.1)	0.9 (0.0 to 3.3)	4319.8 (3739.5 to 5024.7)	1945.8 (1687.1 to 2259.1)	2306.8 (1804.5 to 2964.8)	64.7 (30.7 to 118.3)	2.4 (0.0 to 8.7)

		2018 US Dollars per capita					2018 PPP per capita				
Country	Year	Health spending	Government health spending	Out-of-pocket health spending	Prepaid private health spending	Development assistance for health	Health spending	Government health spending	Out-of-pocket health spending	Prepaid private health spending	Development assistance for health
Burkina Faso	1995	18.0 (15.0 to 21.7)	4.7 (3.5 to 6.2)	8.6 (6.0 to 11.9)	1.0 (0.4 to 1.8)	3.7 (3.7 to 3.7)	50.1 (41.7 to 60.3)	13.2 (9.9 to 17.2)	23.9 (16.8 to 33.1)	2.7 (1.2 to 5.1)	10.3 (10.3 to 10.3)
Burkina Faso	1996	16.7 (13.7 to 20.4)	5.2 (3.9 to 6.7)	8.7 (6.1 to 12.0)	1.0 (0.5 to 2.0)	1.8 (1.8 to 1.8)	46.4 (38.1 to 56.7)	14.3 (10.7 to 18.6)	24.2 (17.0 to 33.3)	2.8 (1.3 to 5.5)	5.0 (5.0 to 5.0)
Burkina Faso	1997	18.5 (15.4 to 22.2)	5.3 (4.0 to 6.9)	8.6 (6.0 to 11.9)	1.0 (0.5 to 2.0)	3.6 (3.6 to 3.6)	51.4 (42.7 to 61.6)	14.8 (11.1 to 19.1)	23.8 (16.5 to 33.0)	2.8 (1.3 to 5.4)	10.0 (10.0 to 10.0)
Burkina Faso	1998	18.6 (15.5 to 22.2)	5.5 (4.2 to 7.1)	8.4 (5.8 to 11.6)	1.0 (0.5 to 1.9)	3.8 (3.8 to 3.8)	51.6 (43.1 to 61.5)	15.2 (11.5 to 19.6)	23.2 (16.2 to 32.2)	2.7 (1.3 to 5.2)	10.5 (10.5 to 10.5)
Burkina Faso	1999	17.0 (14.1 to 20.3)	5.6 (4.3 to 7.3)	8.0 (5.6 to 11.1)	0.9 (0.4 to 1.8)	2.6 (2.6 to 2.6)	47.3 (39.1 to 56.3)	15.6 (11.8 to 20.4)	22.1 (15.5 to 30.9)	2.5 (1.2 to 4.9)	7.1 (7.1 to 7.1)
Burkina Faso	2000	15.6 (13.1 to 18.6)	5.3 (4.0 to 7.0)	7.0 (4.9 to 9.7)	0.7 (0.3 to 1.4)	2.6 (2.6 to 2.6)	43.4 (36.2 to 51.7)	14.7 (11.2 to 19.3)	19.4 (13.7 to 26.9)	2.0 (0.9 to 3.9)	7.3 (7.3 to 7.3)
Burkina Faso	2001	16.6 (13.9 to 19.5)	5.5 (4.1 to 7.1)	6.7 (4.8 to 9.4)	0.7 (0.3 to 1.3)	3.8 (3.8 to 3.8)	46.0 (38.7 to 54.1)	15.2 (11.5 to 19.8)	18.5 (13.3 to 26.2)	1.9 (0.8 to 3.6)	10.5 (10.5 to 10.5)
Burkina Faso	2002	16.2 (13.5 to 19.1)	5.7 (4.3 to 7.5)	6.5 (4.5 to 8.9)	0.7 (0.3 to 1.2)	3.3 (3.3 to 3.3)	44.8 (37.4 to 53.0)	16.0 (12.1 to 20.8)	17.9 (12.5 to 24.6)	1.8 (0.8 to 3.4)	9.2 (9.2 to 9.2)
Burkina Faso	2003	19.0 (16.2 to 22.1)	6.3 (4.8 to 8.2)	6.6 (4.6 to 8.9)	0.7 (0.3 to 1.3)	5.4 (5.4 to 5.4)	52.6 (45.0 to 61.3)	17.5 (13.2 to 22.7)	18.2 (12.7 to 24.8)	1.8 (0.8 to 3.5)	15.1 (15.1 to 15.1)
Burkina Faso	2004	21.5 (18.4 to 24.9)	7.3 (5.5 to 9.4)	7.1 (5.0 to 9.8)	0.7 (0.3 to 1.3)	6.3 (6.3 to 6.3)	59.6 (51.1 to 69.2)	20.4 (15.4 to 26.1)	19.8 (13.8 to 27.1)	1.9 (0.9 to 3.7)	17.5 (17.5 to 17.5)
Burkina Faso	2005	23.4 (20.2 to 27.0)	7.8 (5.8 to 9.9)	7.6 (5.3 to 10.4)	0.7 (0.3 to 1.4)	7.4 (7.4 to 7.4)	65.0 (56.2 to 74.9)	21.5 (16.2 to 27.4)	21.0 (14.8 to 28.9)	2.0 (0.9 to 3.8)	20.4 (20.4 to 20.4)
Burkina Faso	2006	24.7 (21.3 to 28.4)	8.4 (6.4 to 10.6)	8.2 (5.8 to 11.4)	0.8 (0.3 to 1.4)	7.3 (7.3 to 7.3)	68.5 (59.1 to 78.7)	23.2 (17.7 to 29.5)	22.9 (16.2 to 31.8)	2.1 (1.0 to 4.0)	20.3 (20.3 to 20.3)
Burkina Faso	2007	25.1 (21.6 to 29.0)	8.6 (6.6 to 11.0)	8.7 (6.3 to 12.1)	0.9 (0.4 to 1.6)	6.8 (6.8 to 6.8)	69.5 (60.0 to 80.4)	23.9 (18.2 to 30.5)	24.3 (17.4 to 33.5)	2.4 (1.1 to 4.6)	19.0 (19.0 to 19.0)
Burkina Faso	2008	28.0 (24.4 to 32.1)	8.9 (6.7 to 11.3)	9.2 (6.6 to 12.7)	1.0 (0.5 to 2.0)	8.8 (8.8 to 8.8)	77.7 (67.8 to 89.0)	24.6 (18.6 to 31.3)	25.7 (18.4 to 35.3)	2.9 (1.3 to 5.5)	24.5 (24.5 to 24.5)
Burkina Faso	2009	28.7 (25.0 to 33.0)	9.1 (6.9 to 11.5)	9.8 (6.9 to 13.5)	1.3 (0.6 to 2.5)	8.6 (8.6 to 8.6)	79.6 (69.4 to 91.7)	25.2 (19.1 to 32.1)	27.1 (19.2 to 37.5)	3.5 (1.7 to 7.0)	23.8 (23.8 to 23.8)
Burkina Faso	2010	31.2 (27.3 to 35.6)	9.1 (6.8 to 11.6)	10.2 (7.2 to 13.9)	1.3 (0.6 to 2.6)	10.6 (10.6 to 10.6)	86.6 (75.8 to 98.9)	25.2 (19.0 to 32.1)	28.3 (19.9 to 38.6)	3.7 (1.7 to 7.2)	29.5 (29.5 to 29.5)
Burkina Faso	2011	27.2 (23.2 to 32.0)	9.2 (6.9 to 11.8)	10.5 (7.3 to 14.3)	1.4 (0.7 to 2.8)	6.1 (6.1 to 6.1)	75.6 (64.3 to 88.7)	25.6 (19.2 to 32.9)	29.2 (20.4 to 39.6)	3.9 (1.8 to 7.6)	17.0 (17.0 to 17.0)
Burkina Faso	2012	30.7 (26.5 to 35.6)	9.3 (7.0 to 12.1)	11.0 (7.7 to 14.9)	1.5 (0.7 to 2.9)	8.9 (8.9 to 8.9)	85.1 (73.6 to 98.8)	25.9 (19.5 to 33.6)	30.4 (21.4 to 41.3)	4.1 (1.9 to 7.9)	24.6 (24.6 to 24.6)
Burkina Faso	2013	32.1 (27.5 to 37.5)	10.4 (7.8 to 13.5)	12.0 (8.5 to 16.5)	1.8 (0.8 to 3.4)	7.9 (7.9 to 7.9)	89.0 (76.3 to 104.2)	28.8 (21.7 to 37.5)	33.4 (23.5 to 45.7)	5.0 (2.4 to 9.6)	21.8 (21.8 to 21.8)
Burkina Faso	2014	33.4 (28.7 to 39.4)	11.1 (8.5 to 14.4)	12.4 (8.7 to 17.2)	2.1 (1.0 to 4.0)	7.8 (7.8 to 7.8)	92.8 (79.6 to 109.4)	30.8 (23.5 to 39.8)	34.4 (24.2 to 47.7)	5.8 (2.7 to 11.1)	21.7 (21.7 to 21.7)
Burkina Faso	2015	35.0 (30.1 to 41.3)	11.5 (8.6 to 14.8)	12.6 (9.0 to 17.7)	2.2 (1.0 to 4.1)	8.8 (8.8 to 8.8)	97.2 (83.5 to 114.7)	31.8 (23.9 to 41.2)	35.0 (25.0 to 49.0)	6.0 (2.7 to 11.4)	24.3 (24.3 to 24.3)

Country	Year	2018 US Dollars per capita					2018 PPP per capita				
		Health spending	Government health spending	Out-of-pocket health spending	Prepaid private health spending	Development assistance for health	Health spending	Government health spending	Out-of-pocket health spending	Prepaid private health spending	Development assistance for health
Burkina Faso	2016	37.2 (31.8 to 43.6)	13.4 (10.2 to 17.4)	13.2 (9.5 to 18.6)	2.4 (1.1 to 4.5)	8.2 (8.2 to 8.2)	103.3 (88.3 to 120.9)	37.1 (28.4 to 48.4)	36.7 (26.2 to 51.7)	6.6 (3.0 to 12.4)	22.9 (22.9 to 22.9)
Burkina Faso	2017	40.7 (34.7 to 48.2)	16.0 (12.2 to 20.9)	13.7 (9.8 to 19.2)	2.6 (1.2 to 4.9)	8.4 (8.4 to 8.4)	112.9 (96.3 to 133.9)	44.4 (34.0 to 57.9)	38.0 (27.1 to 53.3)	7.2 (3.2 to 13.6)	23.3 (23.3 to 23.3)
Burkina Faso	2018	40.7 (34.6 to 48.3)	16.5 (12.6 to 21.5)	14.0 (10.0 to 19.6)	2.6 (1.2 to 5.0)	7.6 (7.5 to 7.7)	113.1 (95.9 to 134.2)	45.7 (35.1 to 59.8)	38.9 (27.7 to 54.4)	7.4 (3.3 to 13.9)	21.1 (20.7 to 21.4)
Burkina Faso	2019	41.5 (35.3 to 49.2)	17.0 (13.0 to 22.2)	14.3 (10.2 to 20.0)	2.7 (1.2 to 5.2)	7.5 (7.0 to 8.0)	115.2 (98.1 to 136.6)	47.1 (36.2 to 61.6)	39.8 (28.2 to 55.5)	7.6 (3.4 to 14.3)	20.7 (19.3 to 22.1)
Burkina Faso	2020	42.4 (36.1 to 50.1)	17.5 (13.5 to 22.9)	14.7 (10.4 to 20.5)	2.8 (1.3 to 5.3)	7.4 (6.8 to 8.1)	117.7 (100.2 to 139.0)	48.6 (37.4 to 63.5)	40.7 (28.9 to 57.0)	7.8 (3.5 to 14.7)	20.6 (18.8 to 22.4)
Burkina Faso	2021	43.3 (36.8 to 51.3)	18.0 (13.8 to 23.6)	15.0 (10.6 to 21.1)	2.9 (1.3 to 5.5)	7.4 (6.6 to 8.2)	120.2 (102.1 to 142.3)	50.0 (38.4 to 65.6)	41.6 (29.5 to 58.5)	8.0 (3.6 to 15.2)	20.6 (18.4 to 22.7)
Burkina Faso	2022	44.3 (37.4 to 52.2)	18.6 (14.1 to 24.3)	15.3 (10.9 to 21.6)	3.0 (1.3 to 5.6)	7.4 (6.6 to 8.4)	122.9 (103.8 to 144.7)	51.5 (39.3 to 67.5)	42.5 (30.2 to 59.8)	8.2 (3.7 to 15.7)	20.6 (18.2 to 23.2)
Burkina Faso	2023	45.3 (38.3 to 53.3)	19.1 (14.6 to 25.0)	15.7 (11.1 to 22.0)	3.0 (1.4 to 5.8)	7.4 (6.4 to 8.5)	125.7 (106.4 to 148.0)	53.1 (40.6 to 69.3)	43.5 (30.7 to 61.1)	8.4 (3.8 to 16.1)	20.6 (17.9 to 23.6)
Burkina Faso	2024	46.4 (38.8 to 54.9)	19.8 (15.1 to 25.8)	16.1 (11.3 to 22.6)	3.1 (1.4 to 6.0)	7.4 (6.4 to 8.7)	128.7 (107.8 to 152.4)	54.8 (42.0 to 71.6)	44.6 (31.5 to 62.6)	8.7 (3.9 to 16.6)	20.6 (17.7 to 24.1)
Burkina Faso	2025	47.5 (39.7 to 56.5)	20.4 (15.6 to 26.7)	16.4 (11.6 to 23.0)	3.2 (1.4 to 6.2)	7.4 (6.3 to 8.8)	131.7 (110.0 to 156.9)	56.6 (43.4 to 74.0)	45.6 (32.2 to 63.7)	9.0 (4.0 to 17.1)	20.6 (17.5 to 24.4)
Burkina Faso	2026	48.6 (40.3 to 57.7)	21.1 (16.2 to 27.5)	16.8 (11.8 to 23.6)	3.3 (1.5 to 6.4)	7.4 (6.2 to 8.7)	134.9 (111.9 to 160.1)	58.4 (44.8 to 76.4)	46.7 (32.8 to 65.6)	9.2 (4.1 to 17.7)	20.5 (17.2 to 24.2)
Burkina Faso	2027	49.8 (41.7 to 59.4)	21.8 (16.7 to 28.5)	17.2 (12.1 to 24.3)	3.4 (1.5 to 6.6)	7.4 (6.1 to 8.8)	138.2 (115.7 to 164.9)	60.4 (46.4 to 79.0)	47.8 (33.5 to 67.5)	9.5 (4.2 to 18.2)	20.5 (17.0 to 24.5)
Burkina Faso	2028	51.0 (42.6 to 61.3)	22.5 (17.2 to 29.4)	17.6 (12.3 to 25.0)	3.5 (1.6 to 6.8)	7.4 (6.1 to 9.0)	141.6 (118.2 to 170.0)	62.4 (47.8 to 81.6)	48.9 (34.2 to 69.4)	9.8 (4.4 to 18.8)	20.5 (17.0 to 25.1)
Burkina Faso	2029	52.3 (43.7 to 62.6)	23.2 (17.8 to 30.3)	18.1 (12.6 to 25.7)	3.6 (1.6 to 7.0)	7.4 (6.0 to 9.1)	145.2 (121.3 to 173.6)	64.5 (49.3 to 84.2)	50.1 (34.8 to 71.2)	10.1 (4.5 to 19.4)	20.5 (16.8 to 25.2)
Burkina Faso	2030	53.5 (44.1 to 64.2)	23.9 (18.3 to 31.2)	18.5 (12.8 to 26.3)	3.7 (1.7 to 7.2)	7.4 (6.0 to 9.1)	148.6 (122.4 to 178.1)	66.4 (50.7 to 86.6)	51.3 (35.5 to 73.0)	10.4 (4.6 to 20.0)	20.5 (16.6 to 25.4)
Burkina Faso	2031	54.9 (45.2 to 66.4)	24.7 (18.9 to 32.2)	18.9 (13.0 to 27.0)	3.9 (1.7 to 7.4)	7.4 (6.0 to 9.3)	152.3 (125.5 to 184.3)	68.6 (52.5 to 89.4)	52.5 (36.1 to 75.0)	10.7 (4.8 to 20.6)	20.5 (16.5 to 25.9)
Burkina Faso	2032	56.3 (46.3 to 68.1)	25.5 (19.6 to 33.3)	19.4 (13.3 to 27.8)	4.0 (1.8 to 7.6)	7.4 (5.9 to 9.4)	156.2 (128.5 to 188.9)	70.9 (54.3 to 92.3)	53.8 (36.8 to 77.1)	11.0 (4.9 to 21.2)	20.5 (16.4 to 26.2)
Burkina Faso	2033	57.7 (47.9 to 69.9)	26.4 (20.2 to 34.4)	19.8 (13.6 to 28.5)	4.1 (1.8 to 7.9)	7.4 (5.9 to 9.5)	160.2 (133.0 to 194.0)	73.2 (56.0 to 95.5)	55.0 (37.7 to 79.2)	11.4 (5.1 to 21.9)	20.5 (16.3 to 26.3)
Burkina Faso	2034	59.2 (48.6 to 70.9)	27.3 (20.8 to 35.5)	20.3 (13.9 to 29.2)	4.2 (1.9 to 8.1)	7.4 (5.8 to 9.7)	164.3 (134.9 to 196.8)	75.7 (57.6 to 98.6)	56.3 (38.5 to 81.1)	11.7 (5.2 to 22.5)	20.6 (16.2 to 26.9)
Burkina Faso	2035	60.7 (49.9 to 73.3)	28.2 (21.4 to 36.7)	20.8 (14.1 to 30.0)	4.4 (1.9 to 8.4)	7.5 (5.8 to 9.8)	168.5 (138.4 to 203.4)	78.2 (59.4 to 101.9)	57.6 (39.2 to 83.3)	12.1 (5.4 to 23.2)	20.7 (16.1 to 27.3)
Burkina Faso	2036	62.3 (51.4 to 75.1)	29.1 (22.1 to 37.9)	21.2 (14.3 to 30.8)	4.5 (2.0 to 8.6)	7.5 (5.8 to 10.0)	172.8 (142.7 to 208.3)	80.7 (61.3 to 105.1)	58.9 (39.7 to 85.4)	12.4 (5.5 to 23.9)	20.8 (16.0 to 27.9)

		2018 US Dollars per capita					2018 PPP per capita				
Country	Year	Health spending	Government health spending	Out-of-pocket health spending	Prepaid private health spending	Development assistance for health	Health spending	Government health spending	Out-of-pocket health spending	Prepaid private health spending	Development assistance for health
Burkina Faso	2037	63.9 (52.6 to 77.5)	30.0 (22.8 to 39.0)	21.7 (14.6 to 31.5)	4.6 (2.1 to 8.9)	7.5 (5.7 to 10.2)	177.2 (146.0 to 214.9)	83.4 (63.3 to 108.4)	60.2 (40.5 to 87.4)	12.8 (5.7 to 24.7)	20.8 (15.9 to 28.2)
Burkina Faso	2038	65.5 (53.9 to 79.6)	31.0 (23.5 to 40.3)	22.2 (14.9 to 32.3)	4.8 (2.1 to 9.2)	7.5 (5.7 to 10.3)	181.8 (149.5 to 220.9)	86.1 (65.3 to 111.8)	61.6 (41.2 to 89.7)	13.2 (5.9 to 25.4)	20.9 (15.8 to 28.7)
Burkina Faso	2039	67.2 (54.8 to 82.2)	32.0 (24.2 to 41.6)	22.7 (15.1 to 33.0)	4.9 (2.2 to 9.4)	7.6 (5.7 to 10.3)	186.5 (152.2 to 228.0)	88.9 (67.3 to 115.6)	62.9 (42.0 to 91.7)	13.6 (6.0 to 26.2)	21.0 (15.8 to 28.6)
Burkina Faso	2040	68.9 (56.5 to 83.1)	33.1 (25.0 to 43.1)	23.2 (15.4 to 33.8)	5.1 (2.2 to 9.7)	7.6 (5.7 to 10.6)	191.3 (156.9 to 230.5)	91.8 (69.3 to 119.5)	64.3 (42.8 to 93.7)	14.0 (6.2 to 27.0)	21.1 (15.8 to 29.3)
Burkina Faso	2041	70.7 (57.7 to 85.6)	34.2 (25.7 to 44.5)	23.7 (15.7 to 34.6)	5.2 (2.3 to 10.0)	7.7 (5.7 to 10.9)	196.2 (160.2 to 237.4)	94.8 (71.4 to 123.6)	65.7 (43.5 to 96.0)	14.4 (6.4 to 27.8)	21.3 (15.8 to 30.2)
Burkina Faso	2042	72.5 (58.9 to 88.1)	35.3 (26.6 to 46.0)	24.2 (16.0 to 35.4)	5.4 (2.4 to 10.3)	7.7 (5.6 to 11.2)	201.3 (163.5 to 244.5)	97.8 (73.7 to 127.8)	67.1 (44.4 to 98.1)	14.9 (6.6 to 28.6)	21.4 (15.6 to 31.0)
Burkina Faso	2043	74.4 (60.4 to 90.9)	36.4 (27.4 to 47.6)	24.7 (16.3 to 36.1)	5.5 (2.4 to 10.6)	7.8 (5.7 to 11.5)	206.5 (167.6 to 252.1)	101.0 (76.0 to 132.1)	68.6 (45.2 to 100.2)	15.3 (6.8 to 29.5)	21.6 (15.8 to 31.9)
Burkina Faso	2044	76.4 (62.7 to 93.4)	37.6 (28.2 to 49.3)	25.2 (16.6 to 37.0)	5.7 (2.5 to 10.9)	7.9 (5.7 to 11.5)	211.9 (173.9 to 259.1)	104.3 (78.4 to 136.7)	70.0 (46.1 to 102.6)	15.8 (7.0 to 30.4)	21.8 (15.8 to 31.9)
Burkina Faso	2045	78.3 (63.3 to 95.5)	38.8 (29.1 to 50.9)	25.8 (16.9 to 37.8)	5.9 (2.6 to 11.3)	7.9 (5.7 to 12.1)	217.4 (175.7 to 265.1)	107.7 (80.7 to 141.2)	71.5 (46.9 to 104.9)	16.3 (7.2 to 31.3)	22.0 (15.8 to 33.6)
Burkina Faso	2046	80.4 (64.4 to 98.8)	40.0 (29.9 to 52.6)	26.3 (17.3 to 38.5)	6.0 (2.7 to 11.6)	8.0 (5.7 to 12.2)	223.0 (178.7 to 274.3)	111.1 (83.1 to 145.9)	73.0 (47.9 to 106.9)	16.7 (7.5 to 32.2)	22.2 (15.9 to 33.9)
Burkina Faso	2047	82.4 (66.2 to 99.6)	41.3 (30.8 to 54.3)	26.8 (17.6 to 39.2)	6.2 (2.8 to 11.9)	8.1 (5.8 to 12.7)	228.7 (183.6 to 276.3)	114.5 (85.6 to 150.8)	74.4 (48.8 to 108.8)	17.2 (7.7 to 33.2)	22.5 (16.1 to 35.3)
Burkina Faso	2048	84.5 (69.1 to 102.9)	42.5 (31.8 to 56.1)	27.4 (17.9 to 39.9)	6.4 (2.9 to 12.3)	8.2 (5.8 to 13.2)	234.5 (191.6 to 285.5)	118.0 (88.1 to 155.6)	75.9 (49.8 to 110.8)	17.7 (7.9 to 34.2)	22.8 (16.0 to 36.6)
Burkina Faso	2049	86.6 (70.0 to 105.9)	43.8 (32.6 to 57.7)	27.9 (18.3 to 40.7)	6.6 (2.9 to 12.7)	8.3 (5.7 to 13.4)	240.4 (194.3 to 293.8)	121.6 (90.5 to 160.2)	77.5 (50.7 to 113.0)	18.2 (8.2 to 35.2)	23.1 (15.9 to 37.0)
Burkina Faso	2050	88.8 (71.7 to 108.5)	45.1 (33.5 to 59.3)	28.5 (18.6 to 41.6)	6.8 (3.0 to 13.0)	8.4 (5.9 to 13.6)	246.5 (199.0 to 301.1)	125.3 (93.0 to 164.7)	79.0 (51.6 to 115.4)	18.8 (8.4 to 36.2)	23.4 (16.3 to 37.8)
Burundi	1995	23.3 (19.2 to 28.4)	7.4 (5.6 to 9.6)	9.8 (6.9 to 13.7)	3.9 (1.9 to 7.3)	2.2 (2.2 to 2.2)	50.9 (42.0 to 62.0)	16.1 (12.3 to 21.1)	21.4 (15.0 to 29.9)	8.5 (4.1 to 15.9)	4.9 (4.9 to 4.9)
Burundi	1996	21.3 (17.5 to 26.3)	7.0 (5.4 to 9.2)	9.3 (6.5 to 12.8)	3.9 (1.9 to 7.0)	1.2 (1.2 to 1.2)	46.6 (38.2 to 57.6)	15.3 (11.7 to 20.2)	20.3 (14.3 to 28.0)	8.5 (4.1 to 15.3)	2.5 (2.5 to 2.5)
Burundi	1997	21.2 (17.4 to 26.0)	6.9 (5.2 to 8.9)	9.2 (6.4 to 12.8)	3.9 (1.8 to 7.1)	1.3 (1.3 to 1.3)	46.4 (38.0 to 56.9)	15.0 (11.4 to 19.5)	20.1 (14.0 to 27.9)	8.4 (3.9 to 15.6)	2.8 (2.8 to 2.8)
Burundi	1998	22.0 (18.0 to 27.2)	7.0 (5.2 to 9.1)	9.5 (6.6 to 13.3)	4.2 (2.0 to 7.9)	1.5 (1.5 to 1.5)	48.2 (39.3 to 59.5)	15.2 (11.4 to 19.9)	20.7 (14.4 to 29.0)	9.1 (4.4 to 17.2)	3.2 (3.2 to 3.2)
Burundi	1999	22.2 (18.0 to 27.6)	6.7 (5.1 to 8.8)	9.6 (6.6 to 13.5)	4.5 (2.2 to 8.4)	1.5 (1.5 to 1.5)	48.6 (39.4 to 60.3)	14.6 (11.0 to 19.2)	20.9 (14.5 to 29.4)	9.7 (4.8 to 18.4)	3.4 (3.4 to 3.4)
Burundi	2000	23.1 (18.6 to 28.6)	6.4 (4.8 to 8.3)	9.9 (7.0 to 13.7)	5.0 (2.4 to 9.3)	1.9 (1.9 to 1.9)	50.4 (40.6 to 62.5)	13.9 (10.6 to 18.1)	21.6 (15.4 to 30.0)	10.9 (5.2 to 20.3)	4.1 (4.1 to 4.1)
Burundi	2001	22.5 (18.3 to 27.7)	6.4 (5.0 to 8.4)	10.1 (7.2 to 13.8)	4.4 (2.1 to 8.1)	1.6 (1.6 to 1.6)	49.3 (40.0 to 60.7)	14.1 (10.9 to 18.3)	22.0 (15.7 to 30.1)	9.7 (4.6 to 17.6)	3.4 (3.4 to 3.4)

Country	Year	2018 US Dollars per capita					2018 PPP per capita				
		Health spending	Government health spending	Out-of-pocket health spending	Prepaid private health spending	Development assistance for health	Health spending	Government health spending	Out-of-pocket health spending	Prepaid private health spending	Development assistance for health
Burundi	2002	23.3 (18.9 to 28.6)	6.5 (5.0 to 8.4)	10.3 (7.3 to 14.3)	4.4 (2.1 to 8.3)	2.0 (2.0 to 2.0)	50.9 (41.4 to 62.6)	14.2 (10.8 to 18.4)	22.5 (15.9 to 31.2)	9.7 (4.7 to 18.1)	4.5 (4.5 to 4.5)
Burundi	2003	23.7 (19.6 to 28.9)	6.5 (4.9 to 8.4)	10.3 (7.2 to 14.1)	4.1 (2.0 to 7.6)	2.8 (2.8 to 2.8)	51.9 (42.8 to 63.1)	14.2 (10.7 to 18.4)	22.5 (15.8 to 30.8)	9.1 (4.3 to 16.6)	6.2 (6.2 to 6.2)
Burundi	2004	26.5 (22.1 to 31.8)	7.5 (5.7 to 9.7)	10.9 (7.6 to 14.8)	4.1 (1.9 to 7.5)	4.0 (4.0 to 4.0)	57.9 (48.2 to 69.5)	16.4 (12.5 to 21.3)	23.8 (16.6 to 32.3)	8.9 (4.2 to 16.5)	8.8 (8.8 to 8.8)
Burundi	2005	29.0 (24.3 to 34.7)	7.8 (5.8 to 10.1)	11.4 (8.0 to 15.6)	4.3 (2.0 to 7.9)	5.5 (5.5 to 5.5)	63.4 (53.1 to 75.8)	16.9 (12.8 to 22.1)	25.0 (17.5 to 34.1)	9.4 (4.4 to 17.3)	12.1 (12.1 to 12.1)
Burundi	2006	31.5 (26.4 to 37.7)	8.1 (6.1 to 10.7)	12.4 (8.7 to 17.1)	4.8 (2.3 to 8.7)	6.2 (6.2 to 6.2)	68.8 (57.6 to 82.5)	17.8 (13.4 to 23.3)	27.1 (18.9 to 37.4)	10.4 (4.9 to 19.1)	13.5 (13.5 to 13.5)
Burundi	2007	31.1 (25.9 to 37.1)	8.1 (6.1 to 10.6)	12.8 (9.0 to 17.5)	4.7 (2.3 to 8.5)	5.5 (5.5 to 5.5)	68.0 (56.6 to 81.1)	17.7 (13.3 to 23.3)	28.0 (19.7 to 38.2)	10.2 (4.9 to 18.6)	12.1 (12.1 to 12.1)
Burundi	2008	31.1 (26.3 to 36.6)	7.5 (5.6 to 9.9)	12.2 (8.5 to 16.7)	3.7 (1.8 to 7.0)	7.7 (7.7 to 7.7)	68.0 (57.4 to 79.9)	16.4 (12.3 to 21.6)	26.6 (18.6 to 36.6)	8.2 (3.9 to 15.2)	16.8 (16.8 to 16.8)
Burundi	2009	30.5 (26.1 to 35.4)	7.9 (6.0 to 10.3)	11.7 (8.1 to 16.3)	2.5 (1.2 to 4.7)	8.5 (8.5 to 8.5)	66.7 (57.1 to 77.4)	17.2 (13.1 to 22.6)	25.5 (17.7 to 35.6)	5.4 (2.7 to 10.3)	18.6 (18.6 to 18.6)
Burundi	2010	31.1 (27.0 to 35.9)	8.2 (6.3 to 10.8)	10.6 (7.4 to 14.8)	1.1 (0.6 to 2.1)	11.1 (11.1 to 11.1)	67.9 (59.0 to 78.4)	18.0 (13.8 to 23.5)	23.2 (16.3 to 32.3)	2.4 (1.2 to 4.6)	24.3 (24.3 to 24.3)
Burundi	2011	30.5 (26.7 to 35.0)	8.9 (6.8 to 11.6)	9.4 (6.5 to 13.1)	0.9 (0.5 to 1.7)	11.3 (11.3 to 11.3)	66.7 (58.4 to 76.5)	19.4 (14.8 to 25.4)	20.5 (14.2 to 28.5)	2.0 (1.0 to 3.8)	24.8 (24.8 to 24.8)
Burundi	2012	27.0 (23.5 to 31.0)	8.6 (6.5 to 11.3)	8.1 (5.6 to 11.2)	0.8 (0.4 to 1.5)	9.6 (9.6 to 9.6)	59.1 (51.4 to 67.8)	18.8 (14.3 to 24.6)	17.7 (12.3 to 24.5)	1.7 (0.8 to 3.2)	20.9 (20.9 to 20.9)
Burundi	2013	26.3 (23.2 to 30.0)	7.7 (5.9 to 10.2)	7.3 (5.1 to 10.1)	0.6 (0.3 to 1.1)	10.7 (10.7 to 10.7)	57.6 (50.7 to 65.7)	16.9 (12.9 to 22.2)	16.0 (11.1 to 22.0)	1.3 (0.6 to 2.5)	23.3 (23.3 to 23.3)
Burundi	2014	27.1 (23.8 to 31.0)	8.4 (6.4 to 11.2)	7.6 (5.2 to 10.4)	0.6 (0.3 to 1.1)	10.5 (10.5 to 10.5)	59.3 (52.1 to 67.8)	18.5 (14.1 to 24.4)	16.5 (11.5 to 22.7)	1.2 (0.6 to 2.4)	23.0 (23.0 to 23.0)
Burundi	2015	25.0 (22.0 to 28.9)	8.1 (6.2 to 10.7)	7.3 (5.1 to 10.0)	0.5 (0.2 to 0.9)	9.1 (9.1 to 9.1)	54.7 (48.0 to 63.3)	17.6 (13.6 to 23.4)	16.0 (11.2 to 21.9)	1.1 (0.5 to 2.1)	19.9 (19.9 to 19.9)
Burundi	2016	28.0 (25.2 to 31.4)	7.4 (5.6 to 9.6)	7.0 (4.9 to 9.8)	0.5 (0.2 to 0.9)	13.2 (13.2 to 13.2)	61.2 (55.1 to 68.6)	16.1 (12.3 to 21.1)	15.3 (10.7 to 21.4)	1.0 (0.5 to 1.9)	28.8 (28.8 to 28.8)
Burundi	2017	27.6 (24.7 to 30.9)	7.5 (5.8 to 9.9)	6.9 (4.9 to 9.7)	0.5 (0.2 to 0.9)	12.7 (12.7 to 12.7)	60.4 (54.1 to 67.6)	16.5 (12.6 to 21.5)	15.1 (10.7 to 21.1)	1.0 (0.5 to 1.9)	27.8 (27.8 to 27.8)
Burundi	2018	26.3 (23.6 to 29.7)	7.5 (5.7 to 9.7)	6.8 (4.8 to 9.5)	0.4 (0.2 to 0.9)	11.6 (11.5 to 11.6)	57.6 (51.5 to 65.0)	16.3 (12.5 to 21.3)	15.0 (10.5 to 20.9)	1.0 (0.5 to 1.9)	25.3 (25.2 to 25.4)
Burundi	2019	26.3 (23.4 to 29.7)	7.4 (5.7 to 9.7)	6.8 (4.8 to 9.5)	0.4 (0.2 to 0.9)	11.6 (10.8 to 12.3)	57.5 (51.1 to 65.0)	16.3 (12.5 to 21.3)	14.8 (10.4 to 20.7)	1.0 (0.5 to 1.9)	25.5 (23.7 to 27.0)
Burundi	2020	26.4 (23.5 to 30.0)	7.4 (5.6 to 9.8)	6.7 (4.7 to 9.4)	0.4 (0.2 to 0.9)	11.8 (10.8 to 12.8)	57.8 (51.3 to 65.5)	16.2 (12.3 to 21.3)	14.7 (10.3 to 20.5)	1.0 (0.5 to 1.9)	25.9 (23.7 to 27.9)
Burundi	2021	26.6 (23.7 to 30.0)	7.4 (5.7 to 9.7)	6.7 (4.7 to 9.3)	0.4 (0.2 to 0.9)	12.1 (10.8 to 13.4)	58.1 (51.7 to 65.6)	16.2 (12.4 to 21.2)	14.6 (10.2 to 20.3)	1.0 (0.5 to 1.9)	26.4 (23.7 to 29.2)
Burundi	2022	26.9 (23.8 to 30.5)	7.4 (5.6 to 9.7)	6.6 (4.6 to 9.2)	0.4 (0.2 to 0.9)	12.4 (11.0 to 13.9)	58.7 (52.0 to 66.8)	16.2 (12.4 to 21.2)	14.4 (10.1 to 20.1)	1.0 (0.5 to 1.9)	27.2 (24.1 to 30.4)

		2018 US Dollars per capita					2018 PPP per capita				
Country	Year	Health spending	Government health spending	Out-of-pocket health spending	Prepaid private health spending	Development assistance for health	Health spending	Government health spending	Out-of-pocket health spending	Prepaid private health spending	Development assistance for health
Burundi	2023	27.1 (23.9 to 30.7)	7.4 (5.6 to 9.6)	6.5 (4.6 to 9.1)	0.4 (0.2 to 0.9)	12.7 (11.1 to 14.5)	59.2 (52.2 to 67.2)	16.1 (12.3 to 21.0)	14.3 (10.1 to 19.9)	1.0 (0.5 to 1.9)	27.8 (24.2 to 31.7)
Burundi	2024	27.3 (23.9 to 31.4)	7.3 (5.6 to 9.6)	6.5 (4.6 to 9.1)	0.4 (0.2 to 0.8)	13.1 (11.3 to 15.1)	59.7 (52.4 to 68.6)	16.0 (12.2 to 20.9)	14.2 (10.0 to 19.8)	1.0 (0.4 to 1.9)	28.5 (24.7 to 32.9)
Burundi	2025	27.6 (24.2 to 31.3)	7.3 (5.6 to 9.5)	6.5 (4.5 to 9.0)	0.5 (0.2 to 0.8)	13.3 (11.4 to 15.6)	60.2 (53.0 to 68.5)	16.0 (12.2 to 20.9)	14.1 (9.9 to 19.7)	1.0 (0.4 to 1.9)	29.2 (24.8 to 34.1)
Burundi	2026	27.8 (24.3 to 31.9)	7.3 (5.5 to 9.5)	6.4 (4.5 to 9.0)	0.5 (0.2 to 0.9)	13.6 (11.5 to 15.9)	60.7 (53.1 to 69.7)	15.9 (12.1 to 20.8)	14.0 (9.8 to 19.6)	1.0 (0.4 to 1.9)	29.8 (25.1 to 34.8)
Burundi	2027	28.0 (24.5 to 32.2)	7.3 (5.5 to 9.5)	6.4 (4.5 to 8.9)	0.5 (0.2 to 0.9)	13.9 (11.6 to 16.5)	61.3 (53.6 to 70.4)	15.9 (12.1 to 20.8)	13.9 (9.8 to 19.5)	1.0 (0.4 to 1.9)	30.4 (25.4 to 36.2)
Burundi	2028	28.3 (24.7 to 32.8)	7.3 (5.5 to 9.5)	6.3 (4.4 to 8.9)	0.5 (0.2 to 0.9)	14.2 (11.8 to 17.2)	61.8 (54.1 to 71.8)	15.9 (12.1 to 20.7)	13.9 (9.7 to 19.4)	1.0 (0.5 to 1.9)	31.1 (25.9 to 37.5)
Burundi	2029	28.5 (24.8 to 32.9)	7.3 (5.5 to 9.5)	6.3 (4.4 to 8.8)	0.5 (0.2 to 0.9)	14.5 (11.9 to 17.8)	62.4 (54.2 to 72.0)	15.9 (12.1 to 20.7)	13.8 (9.7 to 19.3)	1.0 (0.5 to 1.9)	31.7 (26.1 to 39.0)
Burundi	2030	28.8 (24.9 to 33.7)	7.3 (5.5 to 9.5)	6.3 (4.4 to 8.8)	0.5 (0.2 to 0.9)	14.8 (12.1 to 18.1)	63.0 (54.6 to 73.7)	15.9 (12.1 to 20.8)	13.8 (9.6 to 19.2)	1.0 (0.5 to 1.9)	32.3 (26.4 to 39.5)
Burundi	2031	29.1 (24.9 to 34.0)	7.3 (5.5 to 9.5)	6.3 (4.4 to 8.7)	0.5 (0.2 to 0.9)	15.1 (12.2 to 19.1)	63.7 (54.4 to 74.4)	16.0 (12.1 to 20.8)	13.7 (9.6 to 19.1)	1.0 (0.5 to 1.9)	33.0 (26.6 to 41.7)
Burundi	2032	29.4 (25.3 to 34.5)	7.3 (5.6 to 9.6)	6.2 (4.4 to 8.7)	0.5 (0.2 to 0.9)	15.4 (12.2 to 19.6)	64.3 (55.2 to 75.5)	16.1 (12.2 to 20.9)	13.7 (9.5 to 19.0)	1.0 (0.5 to 1.9)	33.6 (26.7 to 42.9)
Burundi	2033	29.7 (25.2 to 35.1)	7.4 (5.6 to 9.6)	6.2 (4.3 to 8.7)	0.5 (0.2 to 0.9)	15.7 (12.3 to 20.4)	65.0 (55.0 to 76.7)	16.1 (12.2 to 21.0)	13.6 (9.5 to 19.0)	1.0 (0.5 to 1.9)	34.2 (26.9 to 44.7)
Burundi	2034	30.1 (25.6 to 35.8)	7.4 (5.6 to 9.7)	6.2 (4.3 to 8.6)	0.5 (0.2 to 0.9)	16.0 (12.4 to 21.1)	65.8 (55.9 to 78.3)	16.2 (12.3 to 21.2)	13.6 (9.5 to 18.9)	1.0 (0.5 to 1.9)	35.0 (27.0 to 46.0)
Burundi	2035	30.4 (25.4 to 36.3)	7.5 (5.6 to 9.8)	6.2 (4.3 to 8.6)	0.5 (0.2 to 0.9)	16.3 (12.4 to 21.5)	66.5 (55.6 to 79.4)	16.3 (12.3 to 21.4)	13.6 (9.4 to 18.9)	1.0 (0.5 to 1.9)	35.6 (27.1 to 46.9)
Burundi	2036	30.8 (25.6 to 37.3)	7.5 (5.7 to 9.8)	6.2 (4.3 to 8.6)	0.5 (0.2 to 0.9)	16.6 (12.5 to 22.1)	67.3 (55.9 to 81.5)	16.4 (12.4 to 21.4)	13.5 (9.4 to 18.9)	1.0 (0.5 to 2.0)	36.3 (27.3 to 48.3)
Burundi	2037	31.1 (25.6 to 37.5)	7.5 (5.7 to 9.9)	6.2 (4.3 to 8.6)	0.5 (0.2 to 0.9)	16.9 (12.6 to 22.8)	67.9 (55.9 to 82.0)	16.5 (12.4 to 21.6)	13.5 (9.4 to 18.9)	1.0 (0.5 to 2.0)	36.9 (27.6 to 49.8)
Burundi	2038	31.4 (25.8 to 38.3)	7.6 (5.7 to 9.9)	6.2 (4.3 to 8.6)	0.5 (0.2 to 0.9)	17.1 (12.6 to 24.0)	68.6 (56.5 to 83.8)	16.6 (12.5 to 21.7)	13.5 (9.4 to 18.9)	1.1 (0.5 to 2.0)	37.5 (27.6 to 52.4)
Burundi	2039	31.7 (25.9 to 39.1)	7.6 (5.7 to 10.0)	6.2 (4.3 to 8.6)	0.5 (0.2 to 0.9)	17.4 (12.7 to 23.9)	69.3 (56.7 to 85.5)	16.7 (12.5 to 21.8)	13.5 (9.4 to 18.9)	1.1 (0.5 to 2.0)	38.0 (27.8 to 52.3)
Burundi	2040	32.0 (26.4 to 40.0)	7.7 (5.8 to 10.0)	6.2 (4.3 to 8.6)	0.5 (0.2 to 0.9)	17.7 (12.9 to 25.2)	70.1 (57.7 to 87.5)	16.8 (12.6 to 21.9)	13.5 (9.4 to 18.9)	1.1 (0.5 to 2.0)	38.7 (28.2 to 55.2)
Burundi	2041	32.4 (26.1 to 40.6)	7.7 (5.8 to 10.1)	6.2 (4.3 to 8.6)	0.5 (0.2 to 0.9)	18.0 (13.0 to 25.9)	70.9 (57.1 to 88.9)	16.9 (12.7 to 22.0)	13.5 (9.4 to 18.8)	1.1 (0.5 to 2.0)	39.5 (28.4 to 56.6)
Burundi	2042	32.8 (26.3 to 42.1)	7.8 (5.8 to 10.2)	6.1 (4.3 to 8.6)	0.5 (0.2 to 0.9)	18.4 (12.8 to 27.0)	71.8 (57.5 to 92.0)	17.0 (12.8 to 22.2)	13.4 (9.3 to 18.8)	1.1 (0.5 to 2.0)	40.3 (28.1 to 59.1)
Burundi	2043	33.2 (26.6 to 42.8)	7.8 (5.9 to 10.3)	6.1 (4.3 to 8.6)	0.5 (0.2 to 0.9)	18.8 (13.0 to 28.4)	72.7 (58.2 to 93.7)	17.1 (12.8 to 22.4)	13.4 (9.3 to 18.7)	1.1 (0.5 to 2.1)	41.1 (28.4 to 62.0)

		2018 US Dollars per capita					2018 PPP per capita				
Country	Year	Health spending	Government health spending	Out-of-pocket health spending	Prepaid private health spending	Development assistance for health	Health spending	Government health spending	Out-of-pocket health spending	Prepaid private health spending	Development assistance for health
Burundi	2044	33.7 (26.7 to 43.9)	7.9 (5.9 to 10.3)	6.1 (4.3 to 8.5)	0.5 (0.2 to 0.9)	19.2 (13.1 to 29.5)	73.7 (58.4 to 96.0)	17.2 (12.9 to 22.6)	13.4 (9.3 to 18.6)	1.1 (0.5 to 2.1)	42.0 (28.7 to 64.6)
Burundi	2045	34.2 (27.0 to 45.7)	7.9 (5.9 to 10.4)	6.1 (4.2 to 8.5)	0.5 (0.2 to 1.0)	19.7 (13.4 to 30.7)	74.7 (59.0 to 100.0)	17.3 (12.9 to 22.8)	13.4 (9.3 to 18.6)	1.1 (0.5 to 2.1)	43.0 (29.3 to 67.2)
Burundi	2046	34.7 (27.2 to 47.2)	7.9 (5.9 to 10.4)	6.1 (4.2 to 8.5)	0.5 (0.2 to 1.0)	20.1 (13.5 to 32.7)	75.9 (59.5 to 103.2)	17.4 (12.9 to 22.8)	13.3 (9.3 to 18.6)	1.1 (0.5 to 2.1)	44.1 (29.6 to 71.6)
Burundi	2047	35.3 (27.4 to 48.4)	8.0 (5.9 to 10.5)	6.1 (4.2 to 8.5)	0.5 (0.2 to 1.0)	20.7 (13.7 to 33.7)	77.1 (60.0 to 105.9)	17.4 (12.9 to 22.9)	13.3 (9.2 to 18.6)	1.1 (0.5 to 2.1)	45.2 (30.1 to 73.6)
Burundi	2048	35.9 (27.9 to 49.9)	8.0 (6.0 to 10.6)	6.1 (4.2 to 8.5)	0.5 (0.2 to 1.0)	21.3 (14.2 to 35.8)	78.5 (61.0 to 109.2)	17.5 (13.0 to 23.1)	13.3 (9.2 to 18.5)	1.1 (0.5 to 2.1)	46.5 (31.1 to 78.4)
Burundi	2049	36.5 (28.3 to 51.7)	8.1 (6.0 to 10.6)	6.1 (4.2 to 8.5)	0.5 (0.2 to 1.0)	21.9 (14.4 to 36.8)	79.8 (61.8 to 113.0)	17.6 (13.1 to 23.3)	13.2 (9.2 to 18.5)	1.1 (0.5 to 2.1)	47.8 (31.4 to 80.4)
Burundi	2050	37.2 (28.6 to 53.6)	8.1 (6.0 to 10.7)	6.0 (4.2 to 8.5)	0.5 (0.2 to 1.0)	22.5 (14.4 to 38.8)	81.4 (62.5 to 117.3)	17.7 (13.2 to 23.4)	13.2 (9.2 to 18.5)	1.1 (0.5 to 2.2)	49.3 (31.5 to 84.9)
Cambodia	1995	41.3 (35.8 to 48.2)	8.5 (6.4 to 10.8)	18.7 (13.4 to 25.2)	0.4 (0.2 to 0.7)	13.8 (13.8 to 13.8)	123.0 (106.5 to 143.3)	25.2 (19.1 to 32.3)	55.6 (40.0 to 75.0)	1.1 (0.5 to 2.1)	41.1 (41.1 to 41.1)
Cambodia	1996	36.3 (30.3 to 43.1)	9.1 (7.0 to 11.8)	19.6 (14.3 to 26.4)	0.4 (0.2 to 0.7)	7.1 (7.1 to 7.1)	107.9 (90.3 to 128.2)	27.0 (20.7 to 35.0)	58.4 (42.7 to 78.4)	1.2 (0.5 to 2.2)	21.3 (21.3 to 21.3)
Cambodia	1997	35.3 (29.3 to 42.5)	9.1 (7.0 to 11.9)	19.9 (14.6 to 26.9)	0.4 (0.2 to 0.7)	5.9 (5.9 to 5.9)	105.0 (87.0 to 126.5)	27.1 (20.8 to 35.4)	59.3 (43.5 to 80.0)	1.1 (0.5 to 2.2)	17.4 (17.4 to 17.4)
Cambodia	1998	33.5 (27.3 to 40.7)	8.7 (6.8 to 11.5)	20.5 (15.0 to 27.6)	0.4 (0.2 to 0.7)	3.8 (3.8 to 3.8)	99.5 (81.2 to 121.1)	26.0 (20.1 to 34.1)	61.0 (44.7 to 82.0)	1.1 (0.5 to 2.1)	11.4 (11.4 to 11.4)
Cambodia	1999	33.8 (27.5 to 41.8)	8.5 (6.6 to 11.2)	21.6 (15.8 to 29.0)	0.4 (0.2 to 0.7)	3.4 (3.4 to 3.4)	100.7 (81.9 to 124.5)	25.4 (19.7 to 33.2)	64.2 (46.9 to 86.3)	1.1 (0.5 to 2.1)	10.0 (10.0 to 10.0)
Cambodia	2000	34.5 (28.1 to 42.7)	7.7 (6.0 to 10.0)	22.2 (16.1 to 30.4)	0.4 (0.2 to 0.7)	4.2 (4.2 to 4.2)	102.6 (83.6 to 126.9)	23.0 (17.8 to 29.7)	66.1 (48.0 to 90.5)	1.1 (0.5 to 2.0)	12.4 (12.4 to 12.4)
Cambodia	2001	36.4 (29.8 to 44.7)	7.7 (5.9 to 9.7)	23.2 (16.9 to 31.6)	0.4 (0.2 to 0.7)	5.2 (5.2 to 5.2)	108.2 (88.7 to 133.1)	22.8 (17.6 to 29.0)	68.9 (50.2 to 94.2)	1.1 (0.5 to 2.0)	15.4 (15.4 to 15.4)
Cambodia	2002	36.3 (29.3 to 44.9)	8.1 (6.2 to 10.3)	24.4 (18.0 to 32.7)	0.4 (0.2 to 0.7)	3.5 (3.5 to 3.5)	108.1 (87.3 to 133.5)	24.0 (18.3 to 30.6)	72.6 (53.7 to 97.4)	1.2 (0.5 to 2.2)	10.3 (10.3 to 10.3)
Cambodia	2003	40.2 (32.9 to 49.0)	8.4 (6.4 to 10.7)	25.4 (18.8 to 34.0)	0.4 (0.2 to 0.8)	6.0 (6.0 to 6.0)	119.7 (97.8 to 145.7)	24.9 (19.1 to 31.8)	75.7 (55.9 to 101.3)	1.3 (0.6 to 2.3)	17.8 (17.8 to 17.8)
Cambodia	2004	41.9 (34.1 to 50.7)	8.5 (6.5 to 11.0)	26.3 (19.1 to 34.7)	0.4 (0.2 to 0.8)	6.7 (6.7 to 6.7)	124.8 (101.4 to 150.8)	25.4 (19.4 to 32.8)	78.1 (56.9 to 103.3)	1.3 (0.6 to 2.5)	20.0 (20.0 to 20.0)
Cambodia	2005	46.0 (38.1 to 54.9)	8.7 (6.7 to 11.3)	27.3 (19.9 to 35.6)	0.5 (0.2 to 0.9)	9.4 (9.4 to 9.4)	136.8 (113.4 to 163.3)	26.0 (19.9 to 33.6)	81.4 (59.3 to 105.8)	1.4 (0.7 to 2.7)	28.0 (28.0 to 28.0)
Cambodia	2006	44.9 (37.3 to 53.0)	8.7 (6.7 to 11.2)	26.5 (19.3 to 34.4)	0.5 (0.2 to 0.9)	9.2 (9.2 to 9.2)	133.7 (110.9 to 157.7)	25.9 (19.8 to 33.3)	79.0 (57.5 to 102.5)	1.5 (0.7 to 2.8)	27.3 (27.3 to 27.3)
Cambodia	2007	46.4 (38.8 to 54.7)	9.2 (7.1 to 11.9)	26.6 (19.5 to 34.8)	0.5 (0.2 to 0.9)	10.0 (10.0 to 10.0)	138.0 (115.3 to 162.9)	27.5 (21.1 to 35.4)	79.2 (58.1 to 103.6)	1.5 (0.7 to 2.8)	29.7 (29.7 to 29.7)
Cambodia	2008	51.6 (43.0 to 61.0)	10.2 (7.8 to 13.0)	30.4 (22.4 to 39.9)	0.5 (0.3 to 1.0)	10.4 (10.4 to 10.4)	153.5 (128.0 to 181.5)	30.3 (23.1 to 38.6)	90.6 (66.5 to 118.9)	1.6 (0.8 to 2.9)	31.0 (31.0 to 31.0)

		2018 US Dollars per capita					2018 PPP per capita				
Country	Year	Health spending	Government health spending	Out-of-pocket health spending	Prepaid private health spending	Development assistance for health	Health spending	Government health spending	Out-of-pocket health spending	Prepaid private health spending	Development assistance for health
Cambodia	2009	56.2 (46.9 to 66.3)	11.0 (8.4 to 14.0)	33.5 (25.0 to 43.7)	0.5 (0.3 to 1.0)	11.2 (11.2 to 11.2)	167.1 (139.6 to 197.4)	32.6 (25.0 to 41.8)	99.7 (74.3 to 130.1)	1.6 (0.8 to 2.9)	33.2 (33.2 to 33.2)
Cambodia	2010	62.2 (52.6 to 73.6)	12.1 (9.2 to 15.6)	36.7 (27.3 to 48.1)	0.5 (0.2 to 1.0)	12.8 (12.8 to 12.8)	185.0 (156.5 to 218.9)	36.1 (27.5 to 46.3)	109.2 (81.3 to 143.0)	1.6 (0.7 to 2.8)	38.1 (38.1 to 38.1)
Cambodia	2011	67.8 (56.5 to 80.3)	13.1 (10.1 to 16.8)	41.2 (31.1 to 54.2)	0.5 (0.2 to 0.8)	13.0 (13.0 to 13.0)	201.7 (168.0 to 238.9)	38.9 (29.9 to 49.9)	122.6 (92.5 to 161.2)	1.4 (0.6 to 2.5)	38.8 (38.8 to 38.8)
Cambodia	2012	67.9 (56.4 to 82.0)	14.2 (10.9 to 18.3)	44.1 (33.2 to 58.0)	0.4 (0.2 to 0.7)	9.3 (9.3 to 9.3)	202.2 (167.8 to 243.9)	42.3 (32.5 to 54.4)	131.3 (98.6 to 172.6)	1.1 (0.5 to 1.9)	27.5 (27.5 to 27.5)
Cambodia	2013	70.7 (58.3 to 85.8)	15.1 (11.7 to 19.3)	45.2 (33.6 to 59.6)	0.2 (0.1 to 0.4)	10.3 (10.3 to 10.3)	210.5 (173.6 to 255.4)	44.9 (34.7 to 57.3)	134.4 (100.1 to 177.3)	0.6 (0.3 to 1.1)	30.6 (30.6 to 30.6)
Cambodia	2014	72.0 (59.4 to 86.8)	15.5 (11.9 to 19.7)	45.4 (34.1 to 59.6)	0.2 (0.1 to 0.4)	10.9 (10.9 to 10.9)	214.2 (176.8 to 258.2)	46.0 (35.4 to 58.6)	135.1 (101.6 to 177.3)	0.6 (0.3 to 1.2)	32.4 (32.4 to 32.4)
Cambodia	2015	73.6 (61.1 to 89.1)	16.5 (12.8 to 21.2)	46.3 (35.0 to 61.4)	0.3 (0.1 to 0.6)	10.4 (10.4 to 10.4)	219.0 (181.8 to 265.2)	49.2 (38.0 to 63.2)	137.9 (104.1 to 182.8)	0.9 (0.4 to 1.7)	30.9 (30.9 to 30.9)
Cambodia	2016	75.7 (62.5 to 93.0)	17.6 (13.7 to 22.7)	48.1 (36.0 to 65.2)	0.4 (0.2 to 0.8)	9.6 (9.6 to 9.6)	225.2 (185.9 to 276.7)	52.4 (40.7 to 67.4)	143.0 (107.2 to 194.1)	1.2 (0.6 to 2.3)	28.6 (28.6 to 28.6)
Cambodia	2017	79.4 (66.1 to 97.1)	19.5 (15.1 to 25.2)	50.8 (37.9 to 69.0)	0.4 (0.2 to 0.8)	8.7 (8.7 to 8.7)	236.1 (196.6 to 288.9)	58.0 (45.1 to 74.9)	151.1 (112.7 to 205.3)	1.3 (0.6 to 2.3)	25.8 (25.8 to 25.8)
Cambodia	2018	81.4 (66.9 to 100.1)	20.2 (15.7 to 26.1)	52.6 (39.2 to 71.6)	0.4 (0.2 to 0.8)	8.1 (8.1 to 8.1)	242.1 (199.0 to 297.7)	60.2 (46.7 to 77.8)	156.5 (116.6 to 213.0)	1.3 (0.6 to 2.4)	24.1 (24.0 to 24.1)
Cambodia	2019	83.5 (68.7 to 103.0)	20.9 (16.2 to 27.0)	54.0 (40.1 to 73.4)	0.5 (0.2 to 0.8)	8.1 (7.5 to 8.6)	248.4 (204.4 to 306.5)	62.2 (48.1 to 80.3)	160.7 (119.3 to 218.3)	1.4 (0.6 to 2.5)	24.1 (22.4 to 25.5)
Cambodia	2020	85.7 (70.2 to 105.3)	21.6 (16.7 to 27.9)	55.4 (41.2 to 74.9)	0.5 (0.2 to 0.9)	8.2 (7.5 to 8.8)	254.9 (208.8 to 313.2)	64.3 (49.6 to 82.9)	164.9 (122.7 to 222.8)	1.4 (0.7 to 2.6)	24.3 (22.2 to 26.2)
Cambodia	2021	87.9 (71.8 to 108.3)	22.3 (17.2 to 28.7)	56.9 (42.2 to 76.8)	0.5 (0.2 to 0.9)	8.3 (7.4 to 9.1)	261.6 (213.5 to 322.1)	66.3 (51.2 to 85.4)	169.2 (125.6 to 228.4)	1.5 (0.7 to 2.6)	24.6 (22.1 to 27.2)
Cambodia	2022	90.2 (74.0 to 110.6)	23.0 (17.8 to 29.6)	58.3 (43.2 to 78.5)	0.5 (0.2 to 0.9)	8.4 (7.5 to 9.4)	268.4 (220.1 to 329.2)	68.4 (52.9 to 88.0)	173.4 (128.6 to 233.7)	1.5 (0.7 to 2.7)	25.0 (22.2 to 27.9)
Cambodia	2023	92.7 (76.7 to 113.9)	23.8 (18.5 to 30.7)	59.8 (44.3 to 80.7)	0.5 (0.2 to 0.9)	8.5 (7.4 to 9.7)	275.9 (228.1 to 338.9)	71.0 (55.0 to 91.3)	178.0 (131.9 to 240.0)	1.6 (0.7 to 2.8)	25.4 (22.1 to 28.9)
Cambodia	2024	95.2 (78.2 to 117.3)	24.7 (19.2 to 31.7)	61.3 (45.8 to 83.6)	0.5 (0.3 to 1.0)	8.7 (7.5 to 10.0)	283.3 (232.6 to 349.1)	73.4 (57.0 to 94.3)	182.4 (136.2 to 248.8)	1.6 (0.8 to 2.9)	25.9 (22.4 to 29.8)
Cambodia	2025	97.9 (80.6 to 119.8)	25.6 (19.8 to 32.8)	62.9 (46.9 to 85.7)	0.6 (0.3 to 1.0)	8.9 (7.6 to 10.4)	291.2 (240.0 to 356.3)	76.1 (59.0 to 97.6)	187.0 (139.6 to 254.9)	1.7 (0.8 to 3.0)	26.4 (22.6 to 30.9)
Cambodia	2026	100.6 (82.4 to 124.0)	26.5 (20.5 to 34.1)	64.5 (48.1 to 87.6)	0.6 (0.3 to 1.0)	9.0 (7.6 to 10.6)	299.4 (245.3 to 369.0)	78.9 (61.1 to 101.4)	191.9 (143.2 to 260.7)	1.7 (0.8 to 3.1)	26.8 (22.6 to 31.4)
Cambodia	2027	103.4 (85.5 to 126.7)	27.5 (21.2 to 35.3)	66.2 (49.3 to 89.8)	0.6 (0.3 to 1.1)	9.2 (7.7 to 10.9)	307.8 (254.3 to 377.1)	81.8 (63.2 to 105.0)	196.9 (146.7 to 267.1)	1.8 (0.8 to 3.2)	27.3 (23.0 to 32.4)
Cambodia	2028	106.4 (87.1 to 130.4)	28.5 (22.0 to 36.6)	67.9 (50.7 to 92.0)	0.6 (0.3 to 1.1)	9.4 (7.9 to 11.3)	316.5 (259.2 to 388.0)	84.8 (65.6 to 108.8)	201.9 (150.9 to 273.8)	1.8 (0.9 to 3.3)	27.9 (23.4 to 33.7)
Cambodia	2029	109.3 (90.2 to 134.3)	29.5 (22.7 to 37.8)	69.6 (52.1 to 94.9)	0.6 (0.3 to 1.1)	9.6 (7.9 to 11.7)	325.2 (268.5 to 399.6)	87.8 (67.7 to 112.5)	207.0 (155.0 to 282.3)	1.9 (0.9 to 3.4)	28.5 (23.5 to 34.9)

		2018 US Dollars per capita					2018 PPP per capita				
Country	Year	Health spending	Government health spending	Out-of-pocket health spending	Prepaid private health spending	Development assistance for health	Health spending	Government health spending	Out-of-pocket health spending	Prepaid private health spending	Development assistance for health
Cambodia	2030	112.4 (91.9 to 138.7)	30.6 (23.5 to 39.2)	71.4 (53.4 to 97.3)	0.7 (0.3 to 1.2)	9.8 (8.0 to 12.0)	334.4 (273.4 to 412.7)	90.9 (69.9 to 116.5)	212.4 (158.8 to 289.5)	2.0 (0.9 to 3.5)	29.1 (23.8 to 35.7)
Cambodia	2031	115.6 (94.8 to 143.5)	31.7 (24.2 to 40.6)	73.3 (54.5 to 100.0)	0.7 (0.3 to 1.2)	10.0 (8.1 to 12.6)	344.0 (282.0 to 427.1)	94.2 (72.1 to 120.9)	218.0 (162.2 to 297.4)	2.0 (1.0 to 3.7)	29.7 (24.1 to 37.4)
Cambodia	2032	119.0 (97.6 to 146.7)	32.8 (25.1 to 42.2)	75.2 (56.0 to 102.9)	0.7 (0.3 to 1.3)	10.2 (8.2 to 12.9)	354.0 (290.4 to 436.6)	97.7 (74.6 to 125.6)	223.9 (166.5 to 306.1)	2.1 (1.0 to 3.8)	30.3 (24.4 to 38.4)
Cambodia	2033	122.5 (100.2 to 150.3)	34.1 (26.0 to 43.9)	77.3 (57.5 to 105.9)	0.7 (0.3 to 1.3)	10.4 (8.2 to 13.4)	364.6 (298.0 to 447.2)	101.4 (77.5 to 130.6)	230.1 (171.1 to 315.0)	2.2 (1.0 to 4.0)	31.0 (24.5 to 39.8)
Cambodia	2034	126.3 (103.1 to 155.7)	35.4 (27.0 to 45.7)	79.5 (59.1 to 108.9)	0.8 (0.4 to 1.4)	10.7 (8.4 to 13.7)	375.7 (306.8 to 463.4)	105.2 (80.4 to 136.1)	236.5 (175.8 to 323.9)	2.3 (1.1 to 4.1)	31.7 (24.9 to 40.8)
Cambodia	2035	130.0 (106.2 to 161.3)	36.7 (28.0 to 47.6)	81.6 (60.7 to 111.7)	0.8 (0.4 to 1.4)	10.9 (8.4 to 14.2)	386.9 (315.9 to 479.9)	109.2 (83.2 to 141.6)	242.9 (180.5 to 332.3)	2.3 (1.1 to 4.2)	32.4 (25.1 to 42.1)
Cambodia	2036	133.8 (109.7 to 167.3)	38.0 (28.9 to 49.4)	83.8 (62.1 to 114.9)	0.8 (0.4 to 1.5)	11.1 (8.6 to 14.9)	398.2 (326.3 to 497.8)	113.2 (86.0 to 147.0)	249.4 (184.8 to 341.8)	2.4 (1.1 to 4.4)	33.1 (25.7 to 44.5)
Cambodia	2037	137.8 (111.9 to 169.3)	39.5 (29.9 to 51.3)	86.1 (63.5 to 118.5)	0.8 (0.4 to 1.5)	11.4 (8.7 to 15.3)	410.1 (332.9 to 503.9)	117.5 (89.0 to 152.7)	256.3 (189.0 to 352.7)	2.5 (1.2 to 4.6)	33.8 (26.0 to 45.7)
Cambodia	2038	142.0 (115.0 to 177.4)	41.0 (31.0 to 53.3)	88.5 (65.0 to 122.3)	0.9 (0.4 to 1.6)	11.6 (8.8 to 16.1)	422.4 (342.3 to 527.8)	121.9 (92.4 to 158.6)	263.3 (193.5 to 363.8)	2.6 (1.2 to 4.7)	34.6 (26.3 to 48.0)
Cambodia	2039	146.3 (119.6 to 180.7)	42.6 (32.2 to 55.4)	91.0 (66.9 to 125.6)	0.9 (0.4 to 1.7)	11.9 (9.0 to 16.5)	435.5 (355.9 to 537.8)	126.7 (95.8 to 164.7)	270.8 (198.9 to 373.8)	2.7 (1.3 to 4.9)	35.3 (26.9 to 49.0)
Cambodia	2040	151.2 (123.2 to 189.1)	44.3 (33.5 to 57.8)	93.8 (68.9 to 129.3)	0.9 (0.4 to 1.7)	12.1 (9.2 to 16.9)	449.8 (366.6 to 562.8)	131.9 (99.7 to 172.1)	279.0 (204.9 to 384.8)	2.8 (1.3 to 5.1)	36.0 (27.3 to 50.3)
Cambodia	2041	156.2 (127.5 to 194.7)	46.2 (34.9 to 60.3)	96.7 (71.1 to 133.2)	1.0 (0.5 to 1.8)	12.4 (9.3 to 17.3)	464.7 (379.4 to 579.2)	137.4 (103.8 to 179.4)	287.6 (211.4 to 396.2)	2.9 (1.4 to 5.3)	36.8 (27.6 to 51.3)
Cambodia	2042	161.6 (131.8 to 201.4)	48.2 (36.4 to 63.0)	99.8 (73.3 to 137.4)	1.0 (0.5 to 1.9)	12.6 (9.4 to 18.2)	480.8 (392.2 to 599.3)	143.3 (108.2 to 187.4)	296.8 (218.1 to 408.7)	3.0 (1.4 to 5.6)	37.6 (27.8 to 54.1)
Cambodia	2043	167.1 (135.7 to 208.7)	50.2 (37.9 to 65.8)	102.9 (75.5 to 141.9)	1.1 (0.5 to 1.9)	12.9 (9.5 to 18.7)	497.3 (403.9 to 620.9)	149.5 (112.8 to 195.9)	306.3 (224.7 to 422.2)	3.2 (1.5 to 5.8)	38.3 (28.3 to 55.7)
Cambodia	2044	172.3 (138.8 to 214.7)	52.2 (39.4 to 68.6)	105.9 (77.6 to 146.0)	1.1 (0.5 to 2.0)	13.2 (9.6 to 19.1)	512.8 (413.0 to 638.8)	155.3 (117.2 to 204.0)	315.0 (231.0 to 434.3)	3.3 (1.5 to 6.0)	39.2 (28.6 to 56.8)
Cambodia	2045	177.1 (144.3 to 219.9)	54.0 (40.7 to 71.2)	108.5 (79.6 to 149.4)	1.1 (0.5 to 2.1)	13.5 (9.7 to 20.3)	527.0 (429.4 to 654.4)	160.7 (121.2 to 211.8)	322.7 (236.8 to 444.4)	3.4 (1.6 to 6.2)	40.2 (28.9 to 60.3)
Cambodia	2046	181.4 (146.2 to 226.6)	55.7 (42.0 to 73.5)	110.7 (81.3 to 152.1)	1.2 (0.6 to 2.1)	13.8 (10.0 to 21.4)	539.8 (434.9 to 674.2)	165.7 (124.9 to 218.8)	329.5 (241.8 to 452.6)	3.5 (1.6 to 6.4)	41.2 (29.7 to 63.6)
Cambodia	2047	185.1 (150.1 to 231.7)	57.1 (43.0 to 75.6)	112.6 (82.6 to 155.1)	1.2 (0.6 to 2.2)	14.2 (10.1 to 22.3)	550.9 (446.5 to 689.4)	170.0 (128.1 to 224.9)	335.0 (245.9 to 461.6)	3.6 (1.7 to 6.5)	42.3 (30.2 to 66.5)
Cambodia	2048	188.7 (152.8 to 235.1)	58.5 (44.1 to 77.6)	114.3 (83.9 to 158.0)	1.2 (0.6 to 2.2)	14.6 (10.4 to 23.6)	561.5 (454.7 to 699.6)	174.1 (131.2 to 231.0)	340.1 (249.6 to 470.1)	3.7 (1.7 to 6.7)	43.6 (30.9 to 70.3)
Cambodia	2049	192.2 (154.3 to 240.4)	59.9 (45.1 to 79.6)	116.0 (84.9 to 160.3)	1.3 (0.6 to 2.3)	15.1 (10.7 to 24.4)	572.0 (459.1 to 715.3)	178.2 (134.2 to 236.9)	345.2 (252.6 to 477.0)	3.8 (1.7 to 6.8)	44.8 (31.8 to 72.7)
Cambodia	2050	196.4 (159.2 to 245.0)	61.5 (46.2 to 82.0)	118.1 (86.2 to 163.2)	1.3 (0.6 to 2.4)	15.5 (10.8 to 25.1)	584.4 (473.8 to 728.9)	183.1 (137.5 to 243.9)	351.3 (256.6 to 485.7)	3.9 (1.8 to 7.0)	46.2 (32.2 to 74.8)

		2018 US Dollars per capita					2018 PPP per capita				
Country	Year	Health spending	Government health spending	Out-of-pocket health spending	Prepaid private health spending	Development assistance for health	Health spending	Government health spending	Out-of-pocket health spending	Prepaid private health spending	Development assistance for health
Cameroon	1995	42.3 (32.5 to 52.9)	7.5 (5.6 to 9.7)	33.6 (23.6 to 44.1)	0.9 (0.5 to 1.7)	0.3 (0.3 to 0.3)	107.2 (82.4 to 134.1)	19.1 (14.2 to 24.6)	85.2 (59.7 to 111.7)	2.3 (1.1 to 4.4)	0.6 (0.6 to 0.6)
Cameroon	1996	48.5 (37.7 to 59.6)	7.3 (5.4 to 9.3)	36.3 (25.8 to 47.7)	0.9 (0.4 to 1.6)	4.1 (4.1 to 4.1)	122.8 (95.6 to 150.8)	18.4 (13.7 to 23.7)	91.8 (65.3 to 120.7)	2.2 (1.1 to 4.2)	10.5 (10.5 to 10.5)
Cameroon	1997	46.8 (36.3 to 58.1)	7.7 (5.8 to 9.9)	36.7 (26.5 to 47.8)	0.9 (0.4 to 1.7)	1.6 (1.6 to 1.6)	118.5 (92.0 to 147.2)	19.5 (14.7 to 25.2)	92.8 (67.2 to 121.1)	2.2 (1.1 to 4.3)	3.9 (3.9 to 3.9)
Cameroon	1998	47.4 (37.3 to 58.2)	7.8 (5.8 to 10.1)	37.0 (27.1 to 47.8)	0.9 (0.4 to 1.7)	1.7 (1.7 to 1.7)	120.0 (94.3 to 147.4)	19.8 (14.7 to 25.7)	93.6 (68.7 to 121.1)	2.2 (1.1 to 4.2)	4.4 (4.4 to 4.4)
Cameroon	1999	48.1 (38.0 to 59.5)	8.3 (6.2 to 10.7)	37.8 (28.1 to 48.8)	0.9 (0.4 to 1.7)	1.1 (1.1 to 1.1)	121.7 (96.3 to 150.8)	21.0 (15.6 to 27.0)	95.7 (71.1 to 123.6)	2.3 (1.1 to 4.2)	2.9 (2.9 to 2.9)
Cameroon	2000	50.1 (39.4 to 62.5)	9.0 (6.8 to 11.7)	39.3 (29.0 to 51.5)	0.9 (0.5 to 1.8)	0.9 (0.9 to 0.9)	126.9 (99.8 to 158.2)	22.9 (17.1 to 29.5)	99.5 (73.4 to 130.4)	2.4 (1.2 to 4.5)	2.2 (2.2 to 2.2)
Cameroon	2001	52.6 (42.0 to 65.6)	9.9 (7.4 to 12.9)	40.3 (29.7 to 53.4)	1.0 (0.5 to 1.9)	1.4 (1.4 to 1.4)	133.3 (106.3 to 166.1)	25.1 (18.9 to 32.7)	102.2 (75.1 to 135.3)	2.5 (1.2 to 4.9)	3.5 (3.5 to 3.5)
Cameroon	2002	53.4 (42.4 to 66.9)	10.5 (7.9 to 13.8)	41.0 (30.6 to 54.1)	1.0 (0.5 to 2.0)	0.9 (0.9 to 0.9)	135.3 (107.3 to 169.4)	26.7 (20.1 to 34.9)	103.7 (77.5 to 137.0)	2.6 (1.2 to 5.1)	2.2 (2.2 to 2.2)
Cameroon	2003	54.6 (43.4 to 67.7)	10.5 (7.9 to 13.6)	41.2 (30.6 to 54.1)	1.0 (0.5 to 2.0)	1.9 (1.9 to 1.9)	138.3 (110.0 to 171.6)	26.6 (20.1 to 34.6)	104.3 (77.6 to 136.9)	2.6 (1.2 to 5.1)	4.8 (4.8 to 4.8)
Cameroon	2004	54.4 (43.3 to 67.1)	9.8 (7.4 to 12.7)	40.6 (30.1 to 53.0)	1.0 (0.5 to 1.9)	3.1 (3.1 to 3.1)	137.8 (109.6 to 169.9)	24.8 (18.6 to 32.1)	102.7 (76.2 to 134.2)	2.5 (1.2 to 4.8)	7.8 (7.8 to 7.8)
Cameroon	2005	52.9 (42.1 to 65.4)	9.0 (6.8 to 11.7)	39.7 (29.6 to 52.3)	1.0 (0.4 to 1.8)	3.2 (3.2 to 3.2)	134.0 (106.7 to 165.7)	22.9 (17.3 to 29.7)	100.6 (75.0 to 132.4)	2.4 (1.1 to 4.6)	8.1 (8.1 to 8.1)
Cameroon	2006	53.3 (42.8 to 65.7)	8.6 (6.5 to 11.2)	39.7 (29.8 to 52.2)	0.9 (0.4 to 1.8)	4.0 (4.0 to 4.0)	134.9 (108.4 to 166.4)	21.9 (16.5 to 28.4)	100.5 (75.3 to 132.1)	2.4 (1.1 to 4.5)	10.1 (10.1 to 10.1)
Cameroon	2007	53.0 (42.5 to 65.1)	8.3 (6.2 to 10.8)	39.8 (29.6 to 51.9)	0.9 (0.4 to 1.7)	3.9 (3.9 to 3.9)	134.1 (107.7 to 165.0)	21.0 (15.8 to 27.4)	100.8 (75.0 to 131.4)	2.4 (1.1 to 4.4)	10.0 (10.0 to 10.0)
Cameroon	2008	53.5 (43.2 to 65.5)	8.3 (6.3 to 10.9)	40.2 (30.0 to 51.6)	1.0 (0.5 to 1.8)	4.0 (4.0 to 4.0)	135.5 (109.4 to 165.8)	21.1 (16.0 to 27.5)	101.8 (75.9 to 130.7)	2.5 (1.2 to 4.5)	10.2 (10.2 to 10.2)
Cameroon	2009	53.1 (42.7 to 64.8)	8.9 (6.7 to 11.6)	39.0 (28.9 to 50.5)	1.1 (0.5 to 2.0)	4.1 (4.1 to 4.1)	134.5 (108.0 to 164.0)	22.6 (16.9 to 29.4)	98.8 (73.2 to 127.8)	2.7 (1.3 to 5.0)	10.5 (10.5 to 10.5)
Cameroon	2010	51.0 (41.0 to 62.3)	9.5 (7.2 to 12.3)	37.2 (27.7 to 48.3)	1.2 (0.5 to 2.2)	3.1 (3.1 to 3.1)	129.1 (103.7 to 157.7)	24.2 (18.1 to 31.2)	94.2 (70.2 to 122.4)	2.9 (1.4 to 5.6)	7.9 (7.9 to 7.9)
Cameroon	2011	51.9 (42.4 to 62.1)	10.0 (7.5 to 13.0)	33.7 (25.0 to 43.9)	1.2 (0.6 to 2.4)	6.9 (6.9 to 6.9)	131.4 (107.3 to 157.4)	25.3 (19.0 to 32.9)	85.5 (63.2 to 111.3)	3.2 (1.5 to 6.1)	17.5 (17.5 to 17.5)
Cameroon	2012	51.7 (41.2 to 62.7)	9.0 (6.8 to 11.7)	36.6 (26.7 to 47.2)	1.2 (0.5 to 2.3)	4.9 (4.9 to 4.9)	130.8 (104.5 to 158.9)	22.8 (17.1 to 29.7)	92.7 (67.6 to 119.4)	3.0 (1.4 to 5.9)	12.3 (12.3 to 12.3)
Cameroon	2013	54.6 (44.1 to 66.1)	8.5 (6.4 to 11.2)	38.8 (28.7 to 50.3)	1.2 (0.5 to 2.3)	6.2 (6.2 to 6.2)	138.4 (111.7 to 167.5)	21.5 (16.1 to 28.2)	98.2 (72.7 to 127.3)	3.1 (1.4 to 5.9)	15.6 (15.6 to 15.6)
Cameroon	2014	54.6 (43.9 to 67.4)	8.2 (6.2 to 10.9)	40.3 (29.9 to 53.1)	1.3 (0.6 to 2.4)	4.8 (4.8 to 4.8)	138.3 (111.2 to 170.6)	20.7 (15.7 to 27.5)	102.2 (75.8 to 134.4)	3.2 (1.4 to 6.2)	12.2 (12.2 to 12.2)
Cameroon	2015	58.9 (47.5 to 73.4)	8.6 (6.5 to 11.4)	42.3 (31.0 to 56.3)	1.4 (0.6 to 2.7)	6.6 (6.6 to 6.6)	149.2 (120.2 to 185.8)	21.8 (16.4 to 28.8)	107.1 (78.4 to 142.6)	3.5 (1.6 to 6.9)	16.7 (16.7 to 16.7)

		2018 US Dollars per capita					2018 PPP per capita				
Country	Year	Health spending	Government health spending	Out-of-pocket health spending	Prepaid private health spending	Development assistance for health	Health spending	Government health spending	Out-of-pocket health spending	Prepaid private health spending	Development assistance for health
Cameroon	2016	58.4 (46.5 to 74.0)	8.7 (6.6 to 11.4)	43.0 (31.5 to 58.4)	1.4 (0.6 to 2.9)	5.3 (5.3 to 5.3)	148.0 (117.7 to 187.3)	22.0 (16.7 to 29.0)	108.9 (79.7 to 147.8)	3.7 (1.6 to 7.4)	13.4 (13.4 to 13.4)
Cameroon	2017	62.0 (50.2 to 77.4)	8.2 (6.2 to 10.7)	42.6 (31.2 to 57.9)	1.4 (0.6 to 2.9)	9.8 (9.8 to 9.8)	157.1 (127.2 to 195.9)	20.7 (15.6 to 27.2)	107.9 (78.9 to 146.5)	3.6 (1.6 to 7.3)	24.8 (24.8 to 24.8)
Cameroon	2018	61.9 (49.9 to 77.5)	8.3 (6.2 to 10.9)	43.0 (31.3 to 58.4)	1.5 (0.6 to 2.9)	9.1 (9.1 to 9.2)	156.7 (126.2 to 196.1)	21.0 (15.8 to 27.6)	108.9 (79.2 to 147.9)	3.7 (1.6 to 7.4)	23.2 (23.1 to 23.2)
Cameroon	2019	62.4 (50.1 to 78.2)	8.4 (6.3 to 11.0)	43.4 (31.6 to 59.1)	1.5 (0.6 to 3.0)	9.1 (8.5 to 9.6)	158.0 (126.8 to 198.1)	21.2 (16.0 to 27.7)	110.0 (80.1 to 149.6)	3.8 (1.6 to 7.6)	23.1 (21.5 to 24.4)
Cameroon	2020	63.0 (50.7 to 78.9)	8.4 (6.4 to 11.1)	43.9 (32.0 to 59.7)	1.5 (0.7 to 3.0)	9.1 (8.4 to 9.9)	159.5 (128.3 to 199.9)	21.4 (16.1 to 28.0)	111.1 (81.0 to 151.3)	3.8 (1.7 to 7.7)	23.1 (21.2 to 25.0)
Cameroon	2021	63.6 (51.5 to 79.7)	8.5 (6.4 to 11.2)	44.3 (32.3 to 60.3)	1.5 (0.7 to 3.1)	9.2 (8.3 to 10.2)	161.1 (130.5 to 201.8)	21.6 (16.3 to 28.3)	112.3 (81.8 to 152.8)	3.9 (1.7 to 7.9)	23.3 (20.9 to 25.7)
Cameroon	2022	64.3 (51.9 to 80.5)	8.7 (6.5 to 11.3)	44.8 (32.7 to 60.9)	1.6 (0.7 to 3.2)	9.3 (8.2 to 10.4)	162.9 (131.5 to 203.8)	22.0 (16.5 to 28.7)	113.4 (82.7 to 154.3)	4.0 (1.7 to 8.0)	23.5 (20.8 to 26.2)
Cameroon	2023	65.0 (52.2 to 81.2)	8.8 (6.6 to 11.5)	45.2 (33.0 to 61.5)	1.6 (0.7 to 3.2)	9.4 (8.1 to 10.7)	164.5 (132.3 to 205.7)	22.3 (16.7 to 29.1)	114.5 (83.5 to 155.8)	4.0 (1.8 to 8.2)	23.7 (20.6 to 27.0)
Cameroon	2024	65.7 (52.7 to 82.3)	8.9 (6.7 to 11.7)	45.6 (33.3 to 62.4)	1.6 (0.7 to 3.3)	9.5 (8.2 to 10.9)	166.3 (133.5 to 208.4)	22.6 (17.0 to 29.6)	115.6 (84.3 to 158.0)	4.1 (1.8 to 8.3)	24.0 (20.7 to 27.7)
Cameroon	2025	66.4 (53.2 to 82.4)	9.1 (6.8 to 11.8)	46.1 (33.6 to 63.0)	1.7 (0.7 to 3.4)	9.6 (8.1 to 11.2)	168.2 (134.8 to 208.6)	23.0 (17.2 to 30.0)	116.8 (85.1 to 159.5)	4.2 (1.8 to 8.5)	24.2 (20.6 to 28.4)
Cameroon	2026	67.2 (53.9 to 84.2)	9.2 (6.9 to 12.1)	46.6 (33.9 to 63.5)	1.7 (0.7 to 3.4)	9.7 (8.2 to 11.3)	170.2 (136.4 to 213.1)	23.4 (17.4 to 30.5)	118.1 (85.9 to 160.9)	4.3 (1.9 to 8.6)	24.5 (20.7 to 28.7)
Cameroon	2027	68.1 (54.5 to 84.5)	9.4 (7.0 to 12.3)	47.2 (34.3 to 64.2)	1.7 (0.7 to 3.5)	9.8 (8.2 to 11.7)	172.4 (137.9 to 214.0)	23.8 (17.7 to 31.1)	119.4 (86.8 to 162.7)	4.4 (1.9 to 8.8)	24.8 (20.8 to 29.7)
Cameroon	2028	69.0 (55.2 to 86.6)	9.6 (7.1 to 12.5)	47.7 (34.7 to 65.0)	1.8 (0.8 to 3.6)	9.9 (8.3 to 12.0)	174.7 (139.7 to 219.4)	24.2 (18.1 to 31.7)	120.8 (87.9 to 164.6)	4.5 (1.9 to 9.0)	25.2 (21.0 to 30.3)
Cameroon	2029	69.9 (55.9 to 87.5)	9.8 (7.3 to 12.8)	48.3 (35.0 to 65.8)	1.8 (0.8 to 3.6)	10.1 (8.3 to 12.4)	177.1 (141.5 to 221.6)	24.7 (18.4 to 32.3)	122.3 (88.6 to 166.6)	4.6 (2.0 to 9.2)	25.5 (20.9 to 31.4)
Cameroon	2030	70.9 (57.0 to 88.6)	9.9 (7.4 to 13.0)	48.9 (35.4 to 66.9)	1.8 (0.8 to 3.7)	10.2 (8.3 to 12.5)	179.5 (144.3 to 224.3)	25.2 (18.7 to 33.0)	123.9 (89.6 to 169.5)	4.7 (2.0 to 9.4)	25.8 (21.1 to 31.7)
Cameroon	2031	71.9 (57.2 to 90.1)	10.1 (7.5 to 13.3)	49.5 (35.7 to 67.7)	1.9 (0.8 to 3.8)	10.3 (8.3 to 13.0)	182.0 (144.8 to 228.0)	25.7 (19.1 to 33.7)	125.4 (90.4 to 171.5)	4.8 (2.1 to 9.5)	26.2 (21.0 to 32.9)
Cameroon	2032	72.8 (58.2 to 90.7)	10.4 (7.7 to 13.6)	50.1 (36.1 to 68.4)	1.9 (0.8 to 3.8)	10.5 (8.4 to 13.1)	184.5 (147.4 to 229.8)	26.2 (19.4 to 34.4)	126.9 (91.4 to 173.3)	4.9 (2.1 to 9.7)	26.5 (21.2 to 33.1)
Cameroon	2033	73.9 (58.7 to 92.9)	10.6 (7.8 to 13.9)	50.7 (36.5 to 69.6)	2.0 (0.9 to 3.9)	10.6 (8.4 to 13.7)	187.1 (148.7 to 235.2)	26.8 (19.8 to 35.2)	128.5 (92.5 to 176.1)	5.0 (2.2 to 9.9)	26.9 (21.3 to 34.6)
Cameroon	2034	74.9 (59.5 to 94.2)	10.8 (8.0 to 14.2)	51.3 (36.9 to 70.4)	2.0 (0.9 to 4.0)	10.8 (8.5 to 14.2)	189.8 (150.7 to 238.6)	27.3 (20.2 to 35.9)	130.0 (93.5 to 178.2)	5.1 (2.2 to 10.1)	27.4 (21.4 to 35.9)
Cameroon	2035	76.0 (60.1 to 95.4)	11.0 (8.1 to 14.5)	52.0 (37.3 to 71.3)	2.0 (0.9 to 4.1)	11.0 (8.4 to 14.3)	192.5 (152.3 to 241.5)	27.9 (20.6 to 36.8)	131.6 (94.5 to 180.4)	5.2 (2.3 to 10.3)	27.8 (21.3 to 36.1)
Cameroon	2036	77.1 (61.2 to 96.8)	11.3 (8.3 to 14.8)	52.6 (37.7 to 72.2)	2.1 (0.9 to 4.2)	11.2 (8.6 to 14.8)	195.3 (155.0 to 245.0)	28.5 (21.0 to 37.6)	133.2 (95.5 to 182.8)	5.3 (2.3 to 10.5)	28.3 (21.8 to 37.6)

		2018 US Dollars per capita					2018 PPP per capita				
Country	Year	Health spending	Government health spending	Out-of-pocket health spending	Prepaid private health spending	Development assistance for health	Health spending	Government health spending	Out-of-pocket health spending	Prepaid private health spending	Development assistance for health
Cameroon	2037	78.3 (62.4 to 99.1)	11.5 (8.4 to 15.2)	53.3 (38.2 to 73.2)	2.1 (0.9 to 4.3)	11.3 (8.6 to 15.3)	198.2 (158.1 to 250.8)	29.2 (21.4 to 38.5)	134.9 (96.7 to 185.3)	5.4 (2.4 to 10.8)	28.7 (21.7 to 38.8)
Cameroon	2038	79.4 (63.1 to 99.4)	11.8 (8.6 to 15.6)	53.9 (38.5 to 74.2)	2.2 (0.9 to 4.3)	11.5 (8.6 to 15.9)	201.1 (159.8 to 251.7)	29.8 (21.8 to 39.4)	136.5 (97.6 to 187.8)	5.5 (2.4 to 11.0)	29.2 (21.8 to 40.2)
Cameroon	2039	80.5 (63.7 to 101.8)	12.0 (8.8 to 16.0)	54.5 (38.9 to 75.1)	2.2 (1.0 to 4.4)	11.7 (8.7 to 16.5)	203.9 (161.4 to 257.8)	30.5 (22.2 to 40.4)	138.1 (98.4 to 190.2)	5.6 (2.5 to 11.2)	29.7 (22.1 to 41.7)
Cameroon	2040	81.7 (64.6 to 103.0)	12.3 (8.9 to 16.4)	55.2 (39.3 to 76.0)	2.3 (1.0 to 4.5)	12.0 (8.7 to 17.1)	206.9 (163.7 to 260.8)	31.2 (22.6 to 41.4)	139.7 (99.5 to 192.5)	5.7 (2.5 to 11.4)	30.3 (22.1 to 43.3)
Cameroon	2041	82.9 (66.1 to 105.1)	12.6 (9.1 to 16.8)	55.8 (39.7 to 77.0)	2.3 (1.0 to 4.6)	12.2 (8.8 to 17.7)	210.0 (167.5 to 266.1)	31.9 (23.1 to 42.5)	141.3 (100.6 to 195.0)	5.9 (2.5 to 11.6)	30.9 (22.3 to 44.9)
Cameroon	2042	84.2 (66.6 to 106.5)	12.9 (9.3 to 17.2)	56.5 (40.2 to 78.0)	2.4 (1.0 to 4.7)	12.5 (8.8 to 18.4)	213.3 (168.7 to 269.8)	32.6 (23.6 to 43.6)	143.0 (101.8 to 197.5)	6.0 (2.6 to 11.8)	31.6 (22.4 to 46.6)
Cameroon	2043	85.5 (67.5 to 107.5)	13.2 (9.5 to 17.6)	57.1 (40.6 to 79.0)	2.4 (1.0 to 4.8)	12.8 (8.9 to 19.5)	216.5 (170.8 to 272.2)	33.4 (24.0 to 44.6)	144.7 (102.9 to 200.0)	6.1 (2.6 to 12.1)	32.3 (22.6 to 49.4)
Cameroon	2044	86.8 (68.5 to 110.7)	13.5 (9.7 to 18.0)	57.8 (41.1 to 80.0)	2.5 (1.1 to 4.9)	13.1 (9.1 to 19.8)	219.9 (173.6 to 280.3)	34.2 (24.6 to 45.7)	146.4 (104.1 to 202.5)	6.2 (2.7 to 12.3)	33.1 (23.0 to 50.1)
Cameroon	2045	88.2 (69.6 to 111.6)	13.8 (9.9 to 18.5)	58.5 (41.6 to 81.0)	2.5 (1.1 to 5.0)	13.4 (9.3 to 20.6)	223.4 (176.4 to 282.5)	35.0 (25.1 to 46.9)	148.1 (105.3 to 205.1)	6.4 (2.7 to 12.6)	33.9 (23.4 to 52.2)
Cameroon	2046	89.6 (70.7 to 113.5)	14.2 (10.1 to 19.0)	59.2 (42.1 to 82.0)	2.6 (1.1 to 5.1)	13.7 (9.3 to 21.7)	226.9 (178.9 to 287.4)	35.9 (25.6 to 48.1)	149.8 (106.5 to 207.7)	6.5 (2.8 to 12.9)	34.7 (23.7 to 55.0)
Cameroon	2047	91.0 (72.1 to 113.2)	14.5 (10.4 to 19.5)	59.8 (42.6 to 82.9)	2.6 (1.1 to 5.2)	14.1 (9.5 to 23.2)	230.5 (182.6 to 286.7)	36.8 (26.3 to 49.3)	151.5 (107.8 to 209.9)	6.6 (2.8 to 13.1)	35.6 (24.0 to 58.7)
Cameroon	2048	92.6 (72.6 to 117.9)	14.9 (10.6 to 20.0)	60.5 (43.1 to 83.9)	2.7 (1.1 to 5.3)	14.5 (9.6 to 24.2)	234.4 (183.7 to 298.6)	37.7 (26.9 to 50.6)	153.3 (109.2 to 212.4)	6.7 (2.9 to 13.3)	36.6 (24.3 to 61.4)
Cameroon	2049	94.1 (74.2 to 116.9)	15.3 (10.9 to 20.5)	61.3 (43.7 to 84.9)	2.7 (1.2 to 5.4)	14.9 (9.7 to 25.0)	238.3 (187.8 to 296.2)	38.7 (27.5 to 51.8)	155.1 (110.6 to 215.0)	6.9 (3.0 to 13.6)	37.6 (24.7 to 63.2)
Cameroon	2050	95.7 (75.2 to 122.4)	15.7 (11.2 to 21.0)	62.0 (44.2 to 86.0)	2.8 (1.2 to 5.5)	15.3 (9.9 to 25.9)	242.4 (190.4 to 309.8)	39.7 (28.4 to 53.1)	157.0 (111.8 to 217.7)	7.0 (3.0 to 13.9)	38.7 (25.1 to 65.6)
Canada	1995	2937.2 (2836.1 to 3040.4)	2153.5 (2068.8 to 2243.3)	486.4 (448.5 to 529.8)	297.3 (266.0 to 331.6)	0.0 (0.0 to 0.0)	3143.3 (3035.1 to 3253.8)	2304.6 (2213.9 to 2400.7)	520.5 (480.0 to 567.0)	318.1 (284.6 to 354.9)	0.0 (0.0 to 0.0)
Canada	1996	2924.9 (2852.2 to 3007.4)	2132.3 (2071.2 to 2197.4)	492.3 (462.2 to 523.4)	300.4 (276.1 to 326.7)	0.0 (0.0 to 0.0)	3130.2 (3052.3 to 3218.4)	2281.9 (2216.5 to 2351.6)	526.8 (494.6 to 560.2)	321.5 (295.5 to 349.6)	0.0 (0.0 to 0.0)
Canada	1997	3002.8 (2930.4 to 3077.4)	2178.8 (2116.9 to 2243.8)	511.6 (483.6 to 539.3)	312.4 (288.8 to 339.0)	0.0 (0.0 to 0.0)	3213.5 (3136.0 to 3293.3)	2331.7 (2265.5 to 2401.3)	547.5 (517.5 to 577.1)	334.3 (309.1 to 362.7)	0.0 (0.0 to 0.0)
Canada	1998	3095.5 (3023.2 to 3166.2)	2243.1 (2181.8 to 2306.4)	526.4 (500.3 to 553.8)	326.0 (302.6 to 352.0)	0.0 (0.0 to 0.0)	3312.8 (3235.3 to 3388.3)	2400.5 (2335.0 to 2468.2)	563.4 (535.4 to 592.6)	348.9 (323.9 to 376.7)	0.0 (0.0 to 0.0)
Canada	1999	3218.1 (3146.3 to 3288.4)	2336.1 (2275.5 to 2398.7)	541.9 (517.0 to 567.1)	340.2 (315.2 to 364.2)	0.0 (0.0 to 0.0)	3444.0 (3367.1 to 3519.2)	2500.0 (2435.1 to 2567.0)	579.9 (553.3 to 606.9)	364.0 (337.3 to 389.7)	0.0 (0.0 to 0.0)
Canada	2000	3375.7 (3303.1 to 3448.6)	2459.0 (2397.1 to 2520.3)	554.9 (531.3 to 578.8)	361.8 (339.4 to 385.2)	0.0 (0.0 to 0.0)	3612.6 (3534.8 to 3690.6)	2631.6 (2565.3 to 2697.2)	593.8 (568.5 to 619.4)	387.2 (363.2 to 412.2)	0.0 (0.0 to 0.0)
Canada	2001	3500.6 (3429.9 to 3567.1)	2546.9 (2487.7 to 2607.7)	562.5 (539.5 to 585.2)	391.3 (369.4 to 414.1)	0.0 (0.0 to 0.0)	3746.3 (3670.6 to 3817.4)	2725.6 (2662.3 to 2790.6)	601.9 (577.3 to 626.2)	418.8 (395.3 to 443.2)	0.0 (0.0 to 0.0)

Country	Year	2018 US Dollars per capita					2018 PPP per capita				
		Health spending	Government health spending	Out-of-pocket health spending	Prepaid private health spending	Development assistance for health	Health spending	Government health spending	Out-of-pocket health spending	Prepaid private health spending	Development assistance for health
Canada	2002	3654.1 (3586.5 to 3725.9)	2657.2 (2595.2 to 2721.8)	574.8 (552.5 to 598.1)	422.1 (400.7 to 445.2)	0.0 (0.0 to 0.0)	3910.6 (3838.2 to 3987.4)	2843.7 (2777.3 to 2912.8)	615.2 (591.3 to 640.0)	451.7 (428.8 to 476.4)	0.0 (0.0 to 0.0)
Canada	2003	3767.6 (3695.9 to 3837.3)	2746.6 (2681.6 to 2811.4)	581.0 (559.1 to 603.3)	440.0 (417.7 to 463.5)	0.0 (0.0 to 0.0)	4032.0 (3955.2 to 4106.6)	2939.3 (2869.8 to 3008.7)	621.7 (598.3 to 645.6)	470.9 (447.0 to 496.0)	0.0 (0.0 to 0.0)
Canada	2004	3870.1 (3792.6 to 3943.5)	2824.6 (2759.1 to 2890.6)	593.8 (570.1 to 616.4)	451.7 (428.4 to 474.8)	0.0 (0.0 to 0.0)	4141.7 (4058.7 to 4220.2)	3022.8 (2952.7 to 3093.5)	635.4 (610.1 to 659.6)	483.4 (458.5 to 508.1)	0.0 (0.0 to 0.0)
Canada	2005	3954.4 (3887.0 to 4030.0)	2885.3 (2824.5 to 2950.6)	610.4 (587.7 to 633.6)	458.7 (436.4 to 481.6)	0.0 (0.0 to 0.0)	4231.9 (4159.7 to 4312.8)	3087.7 (3022.7 to 3157.6)	653.3 (629.0 to 678.1)	490.9 (467.1 to 515.4)	0.0 (0.0 to 0.0)
Canada	2006	4042.3 (3963.3 to 4116.0)	2945.1 (2878.7 to 3010.2)	631.4 (608.3 to 654.0)	465.8 (442.4 to 489.0)	0.0 (0.0 to 0.0)	4325.9 (4241.4 to 4404.9)	3151.8 (3080.7 to 3221.4)	675.7 (651.0 to 699.9)	498.5 (473.4 to 523.4)	0.0 (0.0 to 0.0)
Canada	2007	4157.5 (4082.4 to 4230.8)	3032.4 (2964.0 to 3096.6)	647.1 (624.3 to 672.3)	478.0 (454.2 to 502.1)	0.0 (0.0 to 0.0)	4449.2 (4368.9 to 4527.7)	3245.2 (3171.9 to 3313.9)	692.5 (668.1 to 719.5)	511.6 (486.1 to 537.3)	0.0 (0.0 to 0.0)
Canada	2008	4328.9 (4253.7 to 4405.6)	3164.8 (3099.7 to 3230.9)	665.1 (641.7 to 688.4)	499.0 (474.8 to 524.6)	0.0 (0.0 to 0.0)	4632.7 (4552.2 to 4714.8)	3386.9 (3317.2 to 3457.6)	711.8 (686.7 to 736.7)	534.0 (508.1 to 561.4)	0.0 (0.0 to 0.0)
Canada	2009	4407.4 (4335.7 to 4482.2)	3217.8 (3154.0 to 3278.9)	675.7 (652.5 to 700.5)	514.0 (489.0 to 538.3)	0.0 (0.0 to 0.0)	4716.7 (4640.0 to 4796.7)	3443.6 (3375.3 to 3509.0)	723.1 (698.2 to 749.6)	550.0 (523.3 to 576.1)	0.0 (0.0 to 0.0)
Canada	2010	4565.9 (4491.9 to 4638.8)	3343.9 (3278.1 to 3407.0)	690.7 (665.1 to 716.3)	531.3 (506.1 to 554.5)	0.0 (0.0 to 0.0)	4886.2 (4807.1 to 4964.4)	3578.5 (3508.1 to 3646.1)	739.2 (711.8 to 766.5)	568.5 (541.6 to 593.5)	0.0 (0.0 to 0.0)
Canada	2011	4596.3 (4522.3 to 4676.1)	3382.6 (3316.0 to 3455.9)	679.3 (655.5 to 702.3)	534.4 (507.8 to 558.5)	0.0 (0.0 to 0.0)	4918.8 (4839.6 to 5004.2)	3619.9 (3548.7 to 3698.4)	726.9 (701.5 to 751.5)	571.9 (543.5 to 597.7)	0.0 (0.0 to 0.0)
Canada	2012	4570.0 (4496.8 to 4651.5)	3365.9 (3298.3 to 3435.2)	666.6 (642.3 to 690.6)	537.5 (512.7 to 566.5)	0.0 (0.0 to 0.0)	4890.7 (4812.3 to 4977.9)	3602.1 (3529.8 to 3676.3)	713.3 (687.3 to 739.1)	575.2 (548.6 to 606.3)	0.0 (0.0 to 0.0)
Canada	2013	4583.3 (4504.0 to 4662.3)	3373.8 (3300.4 to 3444.0)	663.9 (639.8 to 688.0)	545.5 (520.0 to 572.7)	0.0 (0.0 to 0.0)	4904.9 (4820.1 to 4989.5)	3610.5 (3532.0 to 3685.7)	710.5 (684.7 to 736.3)	583.8 (556.5 to 612.9)	0.0 (0.0 to 0.0)
Canada	2014	4647.9 (4572.9 to 4724.9)	3420.9 (3354.0 to 3486.7)	672.9 (649.4 to 697.5)	554.1 (528.2 to 581.3)	0.0 (0.0 to 0.0)	4974.0 (4893.8 to 5056.5)	3660.9 (3589.3 to 3731.3)	720.1 (695.0 to 746.4)	593.0 (565.3 to 622.1)	0.0 (0.0 to 0.0)
Canada	2015	4766.1 (4686.6 to 4848.5)	3505.1 (3437.4 to 3575.5)	691.7 (663.8 to 718.0)	569.3 (542.7 to 596.6)	0.0 (0.0 to 0.0)	5100.6 (5015.4 to 5188.8)	3751.0 (3678.6 to 3826.4)	740.2 (710.4 to 768.4)	609.3 (580.8 to 638.4)	0.0 (0.0 to 0.0)
Canada	2016	4874.5 (4772.7 to 4991.0)	3583.1 (3496.8 to 3682.7)	710.6 (673.6 to 747.4)	580.8 (543.8 to 617.9)	0.0 (0.0 to 0.0)	5216.6 (5107.6 to 5341.3)	3834.5 (3742.2 to 3941.1)	760.4 (720.8 to 799.8)	621.6 (581.9 to 661.2)	0.0 (0.0 to 0.0)
Canada	2017	4968.1 (4860.1 to 5081.6)	3652.6 (3559.3 to 3756.1)	722.3 (681.9 to 759.8)	593.3 (556.6 to 631.4)	0.0 (0.0 to 0.0)	5316.7 (5201.1 to 5438.2)	3908.9 (3809.0 to 4019.7)	772.9 (729.7 to 813.1)	634.9 (595.7 to 675.7)	0.0 (0.0 to 0.0)
Canada	2018	5030.9 (4916.8 to 5149.4)	3699.5 (3602.3 to 3805.3)	730.5 (689.4 to 770.4)	600.9 (563.7 to 639.4)	0.0 (0.0 to 0.0)	5383.9 (5261.8 to 5510.8)	3959.1 (3855.1 to 4072.3)	781.7 (737.8 to 824.4)	643.1 (603.3 to 684.3)	0.0 (0.0 to 0.0)
Canada	2019	5088.8 (4970.9 to 5212.1)	3743.2 (3637.7 to 3858.7)	738.3 (694.4 to 778.8)	607.3 (569.7 to 646.4)	0.0 (0.0 to 0.0)	5445.9 (5319.8 to 5577.8)	4005.9 (3892.9 to 4129.4)	790.1 (743.1 to 833.5)	650.0 (609.6 to 691.7)	0.0 (0.0 to 0.0)
Canada	2020	5146.8 (5014.1 to 5291.2)	3787.1 (3671.0 to 3912.2)	746.1 (700.3 to 789.4)	613.6 (575.5 to 653.1)	0.0 (0.0 to 0.0)	5508.0 (5365.9 to 5662.5)	4052.8 (3928.6 to 4186.7)	798.5 (749.5 to 844.8)	656.7 (615.9 to 699.0)	0.0 (0.0 to 0.0)
Canada	2021	5197.8 (5053.6 to 5346.5)	3825.7 (3692.5 to 3964.3)	752.9 (703.8 to 800.0)	619.3 (580.6 to 658.8)	0.0 (0.0 to 0.0)	5562.5 (5408.2 to 5721.7)	4094.1 (3951.7 to 4242.5)	805.7 (753.2 to 856.2)	662.7 (621.4 to 705.0)	0.0 (0.0 to 0.0)
Canada	2022	5244.4 (5090.5 to 5409.0)	3865.4 (3719.1 to 4016.5)	754.1 (705.2 to 802.3)	624.9 (585.6 to 665.0)	0.0 (0.0 to 0.0)	5612.4 (5447.7 to 5788.5)	4136.6 (3980.1 to 4298.3)	807.0 (754.7 to 858.6)	668.8 (626.7 to 711.6)	0.0 (0.0 to 0.0)

Country	Year	2018 US Dollars per capita					2018 PPP per capita				
		Health spending	Government health spending	Out-of-pocket health spending	Prepaid private health spending	Development assistance for health	Health spending	Government health spending	Out-of-pocket health spending	Prepaid private health spending	Development assistance for health
Canada	2023	5296.9 (5111.3 to 5475.5)	3910.8 (3748.0 to 4078.8)	755.2 (706.4 to 802.7)	630.9 (590.7 to 672.2)	0.0 (0.0 to 0.0)	5668.6 (5470.0 to 5859.8)	4185.3 (4011.0 to 4365.0)	808.2 (756.0 to 859.1)	675.1 (632.1 to 719.4)	0.0 (0.0 to 0.0)
Canada	2024	5343.3 (5152.6 to 5539.3)	3952.1 (3773.3 to 4134.6)	755.3 (704.4 to 802.6)	636.0 (594.9 to 678.1)	0.0 (0.0 to 0.0)	5718.3 (5514.2 to 5928.0)	4229.4 (4038.1 to 4424.7)	808.3 (753.8 to 858.9)	680.6 (636.7 to 725.7)	0.0 (0.0 to 0.0)
Canada	2025	5388.9 (5190.3 to 5593.9)	3992.1 (3801.3 to 4193.1)	756.0 (707.3 to 806.7)	640.8 (599.9 to 684.2)	0.0 (0.0 to 0.0)	5767.0 (5554.5 to 5986.5)	4272.2 (4068.0 to 4487.3)	809.0 (756.9 to 863.3)	685.8 (642.0 to 732.2)	0.0 (0.0 to 0.0)
Canada	2026	5435.4 (5221.9 to 5664.4)	4032.3 (3823.5 to 4244.1)	757.1 (706.6 to 807.0)	646.0 (604.8 to 690.5)	0.0 (0.0 to 0.0)	5816.8 (5588.3 to 6061.8)	4315.2 (4091.9 to 4542.0)	810.2 (756.2 to 863.6)	691.3 (647.3 to 739.0)	0.0 (0.0 to 0.0)
Canada	2027	5488.3 (5251.2 to 5734.5)	4077.3 (3853.5 to 4310.4)	759.0 (706.8 to 815.1)	652.0 (609.1 to 698.2)	0.0 (0.0 to 0.0)	5873.5 (5619.7 to 6136.9)	4363.4 (4123.9 to 4612.9)	812.3 (756.4 to 872.3)	697.8 (651.9 to 747.2)	0.0 (0.0 to 0.0)
Canada	2028	5542.8 (5292.4 to 5810.2)	4122.8 (3881.4 to 4375.2)	761.5 (705.3 to 822.3)	658.4 (615.0 to 706.9)	0.0 (0.0 to 0.0)	5931.7 (5663.8 to 6217.9)	4412.2 (4153.8 to 4682.3)	814.9 (754.8 to 880.0)	704.6 (658.2 to 756.5)	0.0 (0.0 to 0.0)
Canada	2029	5597.3 (5327.8 to 5885.4)	4167.8 (3909.2 to 4437.9)	764.3 (705.7 to 832.2)	665.2 (620.0 to 715.5)	0.0 (0.0 to 0.0)	5990.1 (5701.7 to 6298.4)	4460.2 (4183.5 to 4749.3)	817.9 (755.2 to 890.6)	711.9 (663.5 to 765.7)	0.0 (0.0 to 0.0)
Canada	2030	5657.0 (5372.1 to 5952.4)	4216.5 (3938.1 to 4511.9)	767.8 (704.5 to 845.3)	672.7 (625.4 to 725.4)	0.0 (0.0 to 0.0)	6054.0 (5749.1 to 6370.1)	4512.3 (4214.4 to 4828.5)	821.7 (754.0 to 904.7)	719.9 (669.3 to 776.3)	0.0 (0.0 to 0.0)
Canada	2031	5715.0 (5405.3 to 6035.5)	4262.3 (3970.9 to 4569.1)	771.9 (706.2 to 859.4)	680.8 (631.4 to 736.0)	0.0 (0.0 to 0.0)	6116.1 (5784.6 to 6459.0)	4561.4 (4249.5 to 4889.7)	826.1 (755.7 to 919.7)	728.5 (675.7 to 787.6)	0.0 (0.0 to 0.0)
Canada	2032	5776.3 (5465.7 to 6107.4)	4310.0 (4013.7 to 4629.9)	776.8 (709.3 to 871.5)	689.5 (639.1 to 747.9)	0.0 (0.0 to 0.0)	6181.7 (5849.2 to 6536.0)	4612.5 (4295.3 to 4954.8)	831.3 (759.1 to 932.6)	737.9 (684.0 to 800.4)	0.0 (0.0 to 0.0)
Canada	2033	5840.5 (5499.3 to 6165.9)	4359.3 (4047.4 to 4690.5)	782.2 (712.9 to 883.6)	698.9 (646.1 to 759.4)	0.0 (0.0 to 0.0)	6250.3 (5885.2 to 6598.6)	4665.2 (4331.4 to 5019.6)	837.1 (762.9 to 945.6)	748.0 (691.4 to 812.7)	0.0 (0.0 to 0.0)
Canada	2034	5906.6 (5562.6 to 6263.4)	4409.9 (4084.9 to 4752.5)	787.9 (716.1 to 898.6)	708.7 (653.7 to 772.0)	0.0 (0.0 to 0.0)	6321.0 (5952.9 to 6702.9)	4719.4 (4371.6 to 5086.0)	843.2 (766.4 to 961.6)	758.4 (699.6 to 826.1)	0.0 (0.0 to 0.0)
Canada	2035	5974.8 (5625.9 to 6352.3)	4462.4 (4122.0 to 4819.1)	793.8 (719.4 to 914.6)	718.7 (661.4 to 784.3)	0.0 (0.0 to 0.0)	6394.1 (6020.7 to 6798.0)	4775.5 (4411.2 to 5157.3)	849.5 (769.9 to 978.8)	769.1 (707.8 to 839.3)	0.0 (0.0 to 0.0)
Canada	2036	6040.5 (5648.6 to 6436.4)	4511.8 (4154.3 to 4878.9)	799.8 (723.0 to 930.7)	728.9 (668.7 to 797.8)	0.0 (0.0 to 0.0)	6464.4 (6044.9 to 6888.1)	4828.4 (4445.8 to 5221.2)	855.9 (773.7 to 996.0)	780.0 (715.6 to 853.8)	0.0 (0.0 to 0.0)
Canada	2037	6108.0 (5725.2 to 6513.4)	4562.5 (4195.8 to 4941.0)	806.1 (725.9 to 946.8)	739.4 (676.8 to 811.8)	0.0 (0.0 to 0.0)	6536.6 (6127.0 to 6970.4)	4882.7 (4490.3 to 5287.7)	862.6 (776.8 to 1013.3)	791.3 (724.3 to 868.8)	0.0 (0.0 to 0.0)
Canada	2038	6176.4 (5763.3 to 6601.2)	4613.8 (4239.1 to 5000.1)	812.4 (728.7 to 960.5)	750.2 (685.4 to 826.1)	0.0 (0.0 to 0.0)	6609.8 (6167.7 to 7064.4)	4937.6 (4536.6 to 5351.0)	869.4 (779.8 to 1027.9)	802.8 (733.5 to 884.1)	0.0 (0.0 to 0.0)
Canada	2039	6246.0 (5846.8 to 6662.4)	4666.2 (4275.8 to 5066.1)	818.8 (732.3 to 977.0)	761.1 (693.6 to 840.7)	0.0 (0.0 to 0.0)	6684.3 (6257.0 to 7129.9)	4993.6 (4575.9 to 5421.6)	876.2 (783.7 to 1045.6)	814.5 (742.3 to 899.7)	0.0 (0.0 to 0.0)
Canada	2040	6316.4 (5875.9 to 6768.5)	4719.3 (4316.0 to 5136.7)	825.1 (735.7 to 992.8)	772.0 (701.0 to 855.2)	0.0 (0.0 to 0.0)	6759.6 (6288.2 to 7243.5)	5050.4 (4618.9 to 5497.2)	883.0 (787.4 to 1062.5)	826.2 (750.2 to 915.2)	0.0 (0.0 to 0.0)
Canada	2041	6384.3 (5958.2 to 6865.8)	4769.5 (4349.7 to 5198.9)	831.6 (739.5 to 1009.2)	783.2 (708.6 to 868.6)	0.0 (0.0 to 0.0)	6832.3 (6376.3 to 7347.6)	5104.2 (4654.9 to 5563.7)	889.9 (791.4 to 1080.0)	838.1 (758.4 to 929.6)	0.0 (0.0 to 0.0)
Canada	2042	6455.7 (5997.4 to 6942.6)	4822.5 (4387.9 to 5263.1)	838.4 (743.7 to 1025.1)	794.8 (716.7 to 883.4)	0.0 (0.0 to 0.0)	6908.7 (6418.3 to 7429.8)	5160.9 (4695.8 to 5632.4)	897.2 (795.9 to 1097.0)	850.6 (767.0 to 945.4)	0.0 (0.0 to 0.0)
Canada	2043	6528.4 (6042.2 to 7039.7)	4876.5 (4431.1 to 5349.5)	845.2 (747.7 to 1040.3)	806.7 (726.1 to 898.9)	0.0 (0.0 to 0.0)	6986.5 (6466.2 to 7533.7)	5218.7 (4742.0 to 5724.8)	904.5 (800.2 to 1113.3)	863.3 (777.1 to 962.0)	0.0 (0.0 to 0.0)

		2018 US Dollars per capita					2018 PPP per capita				
Country	Year	Health spending	Government health spending	Out-of-pocket health spending	Prepaid private health spending	Development assistance for health	Health spending	Government health spending	Out-of-pocket health spending	Prepaid private health spending	Development assistance for health
Canada	2044	6602.4 (6094.3 to 7122.5)	4931.7 (4469.4 to 5427.2)	852.0 (752.6 to 1055.2)	818.6 (735.7 to 915.5)	0.0 (0.0 to 0.0)	7065.7 (6522.0 to 7622.3)	5277.8 (4783.0 to 5808.0)	911.8 (805.4 to 1129.3)	876.1 (787.4 to 979.7)	0.0 (0.0 to 0.0)
Canada	2045	6677.3 (6154.1 to 7241.3)	4988.2 (4507.6 to 5503.5)	858.6 (757.0 to 1069.6)	830.5 (743.4 to 930.4)	0.0 (0.0 to 0.0)	7145.8 (6585.9 to 7749.5)	5338.2 (4823.9 to 5889.7)	918.8 (810.1 to 1144.7)	888.8 (795.5 to 995.6)	0.0 (0.0 to 0.0)
Canada	2046	6748.0 (6206.9 to 7312.0)	5040.8 (4532.6 to 5586.4)	865.0 (761.6 to 1083.7)	842.2 (751.4 to 945.1)	0.0 (0.0 to 0.0)	7221.6 (6642.4 to 7825.1)	5394.5 (4850.6 to 5978.5)	925.7 (815.1 to 1159.7)	901.3 (804.1 to 1011.4)	0.0 (0.0 to 0.0)
Canada	2047	6819.2 (6287.8 to 7418.7)	5093.9 (4566.4 to 5660.0)	871.3 (766.5 to 1097.3)	854.0 (759.4 to 961.6)	0.0 (0.0 to 0.0)	7297.7 (6729.0 to 7939.3)	5451.4 (4886.8 to 6057.2)	932.4 (820.3 to 1174.3)	913.9 (812.7 to 1029.0)	0.0 (0.0 to 0.0)
Canada	2048	6892.3 (6327.2 to 7511.9)	5149.0 (4599.7 to 5732.6)	877.5 (771.0 to 1111.0)	865.9 (767.4 to 977.2)	0.0 (0.0 to 0.0)	7376.0 (6771.2 to 8039.0)	5510.3 (4922.5 to 6134.9)	939.0 (825.1 to 1189.0)	926.7 (821.3 to 1045.7)	0.0 (0.0 to 0.0)
Canada	2049	6965.5 (6376.9 to 7638.2)	5204.2 (4633.5 to 5800.6)	883.6 (775.3 to 1124.5)	877.8 (775.5 to 994.0)	0.0 (0.0 to 0.0)	7454.3 (6824.4 to 8174.1)	5569.3 (4958.6 to 6207.7)	945.6 (829.7 to 1203.4)	939.4 (829.9 to 1063.8)	0.0 (0.0 to 0.0)
Canada	2050	7041.3 (6432.1 to 7723.4)	5261.6 (4677.4 to 5879.4)	889.8 (779.8 to 1138.5)	889.9 (784.1 to 1012.0)	0.0 (0.0 to 0.0)	7535.4 (6883.4 to 8265.4)	5630.8 (5005.6 to 6292.0)	952.3 (834.5 to 1218.4)	952.3 (839.1 to 1083.0)	0.0 (0.0 to 0.0)
Cape Verde	1995	80.2 (67.4 to 96.2)	67.8 (55.3 to 83.3)	10.5 (7.5 to 14.1)	1.1 (0.5 to 2.1)	0.9 (0.9 to 0.9)	168.8 (141.8 to 202.5)	142.6 (116.3 to 175.3)	22.1 (15.8 to 29.7)	2.3 (1.1 to 4.3)	1.8 (1.8 to 1.8)
Cape Verde	1996	80.8 (68.0 to 97.1)	68.8 (56.5 to 84.4)	10.9 (7.8 to 14.5)	1.1 (0.5 to 2.1)	0.0 (0.0 to 0.0)	170.0 (143.2 to 204.3)	144.8 (118.9 to 177.6)	22.9 (16.4 to 30.5)	2.3 (1.1 to 4.5)	0.0 (0.0 to 0.0)
Cape Verde	1997	91.1 (78.1 to 107.0)	68.1 (56.3 to 82.6)	12.3 (8.9 to 16.5)	1.1 (0.5 to 2.0)	9.6 (9.6 to 9.6)	191.7 (164.4 to 225.1)	143.3 (118.4 to 173.8)	26.0 (18.8 to 34.7)	2.3 (1.1 to 4.3)	20.1 (20.1 to 20.1)
Cape Verde	1998	90.6 (77.5 to 106.3)	69.2 (57.3 to 83.3)	16.1 (11.6 to 21.6)	1.1 (0.5 to 2.0)	4.2 (4.2 to 4.2)	190.6 (163.0 to 223.6)	145.6 (120.6 to 175.4)	33.9 (24.4 to 45.5)	2.2 (1.0 to 4.2)	8.9 (8.9 to 8.9)
Cape Verde	1999	99.0 (85.7 to 114.4)	70.6 (58.9 to 85.4)	18.9 (13.4 to 25.5)	1.0 (0.5 to 1.9)	8.5 (8.5 to 8.5)	208.3 (180.4 to 240.7)	148.5 (123.9 to 179.7)	39.8 (28.2 to 53.7)	2.2 (1.0 to 4.1)	17.8 (17.8 to 17.8)
Cape Verde	2000	98.2 (85.2 to 114.0)	69.7 (58.6 to 83.9)	21.1 (15.0 to 28.4)	0.9 (0.4 to 1.7)	6.5 (6.5 to 6.5)	206.7 (179.4 to 239.8)	146.6 (123.3 to 176.6)	44.4 (31.6 to 59.8)	2.0 (0.9 to 3.7)	13.7 (13.7 to 13.7)
Cape Verde	2001	115.6 (102.1 to 131.1)	70.6 (59.0 to 84.4)	22.6 (15.9 to 30.3)	0.9 (0.4 to 1.7)	21.5 (21.5 to 21.5)	243.3 (214.9 to 275.9)	148.5 (124.1 to 177.5)	47.5 (33.5 to 63.9)	1.9 (0.9 to 3.6)	45.3 (45.3 to 45.3)
Cape Verde	2002	102.2 (88.5 to 117.0)	71.8 (60.0 to 85.6)	23.7 (16.8 to 31.4)	0.9 (0.4 to 1.8)	5.8 (5.8 to 5.8)	215.1 (186.1 to 246.2)	151.1 (126.4 to 180.2)	49.9 (35.4 to 66.1)	2.0 (0.9 to 3.7)	12.2 (12.2 to 12.2)
Cape Verde	2003	117.9 (103.6 to 134.0)	73.4 (61.1 to 87.4)	24.7 (17.6 to 32.9)	1.0 (0.5 to 1.8)	18.9 (18.9 to 18.9)	248.1 (217.9 to 282.0)	154.4 (128.5 to 183.8)	51.9 (37.0 to 69.3)	2.0 (1.0 to 3.9)	39.7 (39.7 to 39.7)
Cape Verde	2004	122.8 (108.0 to 138.8)	74.8 (62.6 to 88.3)	25.6 (18.5 to 34.1)	1.0 (0.5 to 1.9)	21.4 (21.4 to 21.4)	258.3 (227.3 to 292.1)	157.3 (131.7 to 185.8)	53.8 (38.9 to 71.8)	2.1 (1.0 to 4.1)	45.0 (45.0 to 45.0)
Cape Verde	2005	131.3 (116.2 to 147.6)	75.4 (63.0 to 89.1)	26.6 (19.5 to 35.2)	1.0 (0.5 to 2.0)	28.3 (28.3 to 28.3)	276.3 (244.6 to 310.6)	158.6 (132.5 to 187.4)	55.9 (41.0 to 74.0)	2.2 (1.0 to 4.2)	59.6 (59.6 to 59.6)
Cape Verde	2006	134.9 (119.3 to 151.1)	78.7 (66.1 to 92.8)	28.0 (20.6 to 37.2)	1.1 (0.5 to 2.1)	27.0 (27.0 to 27.0)	283.7 (251.1 to 318.0)	165.6 (139.0 to 195.3)	59.0 (43.4 to 78.4)	2.4 (1.1 to 4.4)	56.8 (56.8 to 56.8)
Cape Verde	2007	138.8 (122.4 to 155.8)	84.2 (70.9 to 99.1)	29.3 (21.5 to 38.9)	1.3 (0.6 to 2.3)	24.0 (24.0 to 24.0)	292.0 (257.5 to 327.9)	177.2 (149.3 to 208.4)	61.7 (45.3 to 81.9)	2.7 (1.3 to 4.8)	50.5 (50.5 to 50.5)
Cape Verde	2008	142.5 (126.0 to 159.5)	85.4 (72.4 to 100.2)	30.2 (22.1 to 40.0)	1.4 (0.7 to 2.7)	25.4 (25.4 to 25.4)	299.8 (265.2 to 335.7)	179.8 (152.3 to 210.9)	63.5 (46.6 to 84.1)	3.0 (1.4 to 5.7)	53.5 (53.5 to 53.5)

		2018 US Dollars per capita					2018 PPP per capita				
Country	Year	Health spending	Government health spending	Out-of-pocket health spending	Prepaid private health spending	Development assistance for health	Health spending	Government health spending	Out-of-pocket health spending	Prepaid private health spending	Development assistance for health
Cape Verde	2009	131.6 (115.5 to 149.4)	86.9 (73.3 to 102.1)	31.9 (23.7 to 42.0)	1.9 (0.9 to 3.5)	11.0 (11.0 to 11.0)	276.9 (243.1 to 314.3)	182.8 (154.3 to 214.8)	67.2 (49.9 to 88.4)	3.9 (1.8 to 7.3)	23.1 (23.1 to 23.1)
Cape Verde	2010	151.8 (134.9 to 169.7)	90.1 (76.2 to 105.7)	35.7 (26.5 to 46.5)	2.8 (1.3 to 5.2)	23.2 (23.2 to 23.2)	319.3 (283.9 to 357.0)	189.5 (160.4 to 222.5)	75.1 (55.7 to 97.9)	5.9 (2.8 to 10.9)	48.9 (48.9 to 48.9)
Cape Verde	2011	176.6 (159.7 to 195.7)	94.3 (79.9 to 111.1)	37.6 (27.8 to 49.3)	3.6 (1.7 to 6.5)	41.2 (41.2 to 41.2)	371.7 (336.0 to 411.7)	198.4 (168.1 to 233.9)	79.1 (58.5 to 103.8)	7.5 (3.6 to 13.7)	86.6 (86.6 to 86.6)
Cape Verde	2012	166.4 (148.7 to 186.2)	96.5 (82.2 to 113.5)	39.7 (29.6 to 52.2)	4.1 (2.0 to 7.7)	26.1 (26.1 to 26.1)	350.2 (312.8 to 391.8)	203.0 (173.0 to 238.9)	83.6 (62.2 to 109.8)	8.7 (4.2 to 16.2)	54.9 (54.9 to 54.9)
Cape Verde	2013	180.0 (160.5 to 200.3)	96.0 (81.8 to 112.8)	40.6 (30.1 to 53.5)	4.3 (2.1 to 7.9)	39.1 (39.1 to 39.1)	378.7 (337.6 to 421.4)	201.9 (172.1 to 237.4)	85.5 (63.3 to 112.6)	9.1 (4.5 to 16.7)	82.2 (82.2 to 82.2)
Cape Verde	2014	173.9 (152.8 to 195.5)	98.6 (82.7 to 116.8)	41.8 (30.9 to 55.5)	4.1 (2.0 to 7.8)	29.4 (29.4 to 29.4)	366.0 (321.6 to 411.5)	207.4 (174.1 to 245.9)	88.0 (65.0 to 116.8)	8.7 (4.3 to 16.5)	61.8 (61.8 to 61.8)
Cape Verde	2015	169.6 (148.0 to 192.2)	102.1 (84.3 to 121.7)	41.9 (30.9 to 55.7)	4.0 (1.9 to 7.4)	21.6 (21.6 to 21.6)	356.9 (311.4 to 404.5)	214.8 (177.4 to 256.2)	88.2 (65.0 to 117.2)	8.3 (4.1 to 15.7)	45.5 (45.5 to 45.5)
Cape Verde	2016	156.6 (134.0 to 182.1)	101.5 (83.3 to 122.0)	42.9 (31.1 to 57.3)	3.9 (1.9 to 7.1)	8.4 (8.4 to 8.4)	329.6 (281.9 to 383.1)	213.6 (175.2 to 256.7)	90.2 (65.4 to 120.5)	8.2 (3.9 to 14.8)	17.6 (17.6 to 17.6)
Cape Verde	2017	161.9 (138.1 to 188.9)	107.6 (88.1 to 129.1)	44.3 (32.1 to 58.9)	4.0 (1.9 to 7.4)	6.0 (6.0 to 6.0)	340.8 (290.6 to 397.4)	226.4 (185.4 to 271.6)	93.3 (67.6 to 124.0)	8.5 (4.1 to 15.5)	12.6 (12.6 to 12.6)
Cape Verde	2018	165.7 (141.0 to 192.9)	110.3 (90.2 to 132.3)	45.6 (33.0 to 60.6)	4.1 (2.0 to 7.5)	5.7 (5.6 to 5.7)	348.6 (296.7 to 405.9)	232.1 (189.7 to 278.3)	95.9 (69.5 to 127.6)	8.7 (4.2 to 15.8)	11.9 (11.9 to 11.9)
Cape Verde	2019	170.2 (145.1 to 199.1)	113.3 (91.6 to 136.3)	47.0 (34.1 to 62.6)	4.2 (2.0 to 7.7)	5.7 (5.3 to 6.0)	358.2 (305.4 to 418.9)	238.3 (192.7 to 286.7)	98.9 (71.8 to 131.7)	8.9 (4.3 to 16.3)	12.0 (11.2 to 12.7)
Cape Verde	2020	175.2 (149.7 to 205.4)	116.4 (94.6 to 140.8)	48.6 (35.2 to 64.7)	4.4 (2.1 to 7.9)	5.8 (5.3 to 6.2)	368.6 (315.0 to 432.1)	244.9 (199.0 to 296.3)	102.3 (74.1 to 136.1)	9.2 (4.4 to 16.7)	12.1 (11.1 to 13.1)
Cape Verde	2021	180.3 (153.4 to 210.3)	119.6 (96.9 to 144.2)	50.3 (36.5 to 67.1)	4.5 (2.1 to 8.2)	5.9 (5.3 to 6.5)	379.3 (322.8 to 442.5)	251.6 (203.9 to 303.5)	105.9 (76.8 to 141.2)	9.4 (4.5 to 17.2)	12.4 (11.1 to 13.7)
Cape Verde	2022	185.5 (157.9 to 217.4)	122.8 (99.3 to 148.9)	52.1 (37.7 to 69.3)	4.6 (2.2 to 8.4)	6.0 (5.3 to 6.7)	390.3 (332.3 to 457.4)	258.4 (208.9 to 313.4)	109.6 (79.3 to 145.8)	9.7 (4.6 to 17.7)	12.6 (11.2 to 14.1)
Cape Verde	2023	190.5 (162.0 to 223.6)	125.8 (101.4 to 152.6)	53.9 (39.1 to 72.0)	4.7 (2.3 to 8.6)	6.1 (5.3 to 6.9)	400.9 (340.9 to 470.4)	264.8 (213.4 to 321.2)	113.4 (82.2 to 151.4)	9.9 (4.8 to 18.1)	12.8 (11.2 to 14.6)
Cape Verde	2024	195.3 (166.5 to 228.7)	128.5 (103.3 to 155.8)	55.7 (40.3 to 74.1)	4.8 (2.3 to 8.8)	6.2 (5.4 to 7.2)	410.9 (350.4 to 481.1)	270.4 (217.4 to 327.9)	117.2 (84.9 to 155.9)	10.1 (4.9 to 18.5)	13.1 (11.3 to 15.1)
Cape Verde	2025	199.7 (169.6 to 234.1)	130.8 (105.1 to 158.8)	57.6 (41.6 to 76.8)	4.9 (2.4 to 9.0)	6.4 (5.4 to 7.5)	420.2 (356.9 to 492.6)	275.3 (221.2 to 334.1)	121.1 (87.5 to 161.7)	10.4 (5.0 to 18.9)	13.4 (11.5 to 15.7)
Cape Verde	2026	204.2 (173.4 to 239.9)	133.2 (107.0 to 162.2)	59.5 (42.9 to 79.6)	5.0 (2.4 to 9.2)	6.5 (5.5 to 7.6)	429.7 (364.9 to 504.7)	280.3 (225.1 to 341.2)	125.2 (90.2 to 167.5)	10.6 (5.1 to 19.3)	13.7 (11.5 to 16.0)
Cape Verde	2027	208.7 (177.0 to 245.8)	135.5 (108.7 to 164.7)	61.4 (44.1 to 82.3)	5.1 (2.5 to 9.4)	6.6 (5.6 to 7.9)	439.1 (372.4 to 517.3)	285.1 (228.7 to 346.5)	129.3 (92.7 to 173.3)	10.8 (5.2 to 19.7)	13.9 (11.7 to 16.6)
Cape Verde	2028	213.3 (181.4 to 250.9)	137.9 (110.5 to 167.7)	63.4 (45.5 to 85.0)	5.2 (2.5 to 9.6)	6.8 (5.7 to 8.2)	448.8 (381.7 to 527.9)	290.1 (232.6 to 352.8)	133.4 (95.7 to 178.8)	11.0 (5.3 to 20.2)	14.3 (11.9 to 17.2)
Cape Verde	2029	217.9 (185.1 to 255.9)	140.2 (112.5 to 170.7)	65.4 (46.7 to 88.0)	5.3 (2.6 to 9.8)	6.9 (5.7 to 8.5)	458.4 (389.4 to 538.4)	295.1 (236.7 to 359.2)	137.6 (98.3 to 185.1)	11.2 (5.4 to 20.6)	14.6 (12.0 to 17.8)

Country	Year	2018 US Dollars per capita					2018 PPP per capita				
		Health spending	Government health spending	Out-of-pocket health spending	Prepaid private health spending	Development assistance for health	Health spending	Government health spending	Out-of-pocket health spending	Prepaid private health spending	Development assistance for health
Cape Verde	2030	222.7 (189.9 to 262.6)	142.8 (114.7 to 173.8)	67.4 (47.7 to 90.9)	5.5 (2.6 to 10.0)	7.1 (5.8 to 8.7)	468.6 (399.5 to 552.6)	300.5 (241.3 to 365.8)	141.7 (100.3 to 191.3)	11.5 (5.5 to 20.9)	14.9 (12.2 to 18.2)
Cape Verde	2031	227.2 (192.3 to 266.9)	145.1 (116.7 to 176.4)	69.3 (48.8 to 94.4)	5.6 (2.7 to 10.1)	7.2 (5.8 to 9.0)	478.1 (404.5 to 561.7)	305.4 (245.6 to 371.2)	145.8 (102.6 to 198.7)	11.7 (5.6 to 21.4)	15.2 (12.3 to 19.0)
Cape Verde	2032	232.1 (196.2 to 273.5)	147.8 (118.9 to 180.0)	71.3 (50.0 to 97.5)	5.7 (2.7 to 10.4)	7.4 (5.9 to 9.3)	488.3 (412.9 to 575.4)	310.9 (250.3 to 378.8)	150.0 (105.2 to 205.2)	11.9 (5.8 to 21.8)	15.5 (12.5 to 19.5)
Cape Verde	2033	236.9 (200.3 to 279.6)	150.3 (120.7 to 183.3)	73.3 (51.4 to 100.4)	5.8 (2.8 to 10.6)	7.5 (6.0 to 9.7)	498.4 (421.4 to 588.3)	316.2 (253.9 to 385.7)	154.1 (108.1 to 211.3)	12.2 (5.9 to 22.2)	15.8 (12.6 to 20.3)
Cape Verde	2034	241.9 (203.1 to 285.1)	153.0 (122.4 to 186.7)	75.2 (52.6 to 103.6)	5.9 (2.9 to 10.8)	7.7 (6.1 to 9.9)	508.9 (427.4 to 600.0)	322.0 (257.6 to 392.9)	158.3 (110.7 to 217.9)	12.4 (6.1 to 22.7)	16.2 (12.8 to 20.9)
Cape Verde	2035	246.9 (206.8 to 291.9)	155.8 (124.4 to 190.2)	77.2 (53.8 to 106.5)	6.0 (2.9 to 11.0)	7.9 (6.1 to 10.3)	519.6 (435.1 to 614.1)	327.8 (261.8 to 400.2)	162.5 (113.1 to 224.0)	12.7 (6.2 to 23.2)	16.6 (12.9 to 21.6)
Cape Verde	2036	251.8 (210.0 to 299.8)	158.4 (126.5 to 193.5)	79.1 (54.7 to 109.3)	6.2 (3.0 to 11.2)	8.1 (6.2 to 10.8)	529.8 (442.0 to 630.9)	333.4 (266.2 to 407.1)	166.5 (115.2 to 230.0)	12.9 (6.3 to 23.6)	16.9 (13.1 to 22.8)
Cape Verde	2037	256.8 (212.5 to 304.4)	161.2 (128.7 to 197.0)	81.1 (55.8 to 112.4)	6.3 (3.1 to 11.5)	8.2 (6.3 to 11.0)	540.3 (447.2 to 640.6)	339.2 (270.7 to 414.5)	170.6 (117.3 to 236.5)	13.2 (6.5 to 24.1)	17.3 (13.3 to 23.2)
Cape Verde	2038	261.7 (217.5 to 310.6)	163.9 (130.7 to 200.3)	83.0 (56.9 to 115.2)	6.4 (3.1 to 11.7)	8.4 (6.4 to 11.7)	550.8 (457.7 to 653.5)	344.9 (275.0 to 421.5)	174.7 (119.6 to 242.4)	13.5 (6.6 to 24.6)	17.7 (13.5 to 24.6)
Cape Verde	2039	266.9 (221.4 to 315.6)	166.9 (132.7 to 204.0)	84.9 (58.0 to 118.1)	6.5 (3.2 to 11.9)	8.6 (6.5 to 11.9)	561.7 (465.8 to 664.1)	351.2 (279.3 to 429.3)	178.7 (122.0 to 248.6)	13.8 (6.7 to 25.1)	18.0 (13.8 to 25.0)
Cape Verde	2040	271.8 (224.0 to 322.9)	169.6 (134.6 to 207.6)	86.8 (59.2 to 121.0)	6.7 (3.3 to 12.2)	8.8 (6.7 to 12.1)	572.0 (471.4 to 679.4)	356.9 (283.2 to 436.8)	182.6 (124.5 to 254.7)	14.1 (6.8 to 25.7)	18.4 (14.0 to 25.5)
Cape Verde	2041	277.1 (229.4 to 328.0)	172.7 (136.8 to 211.4)	88.7 (60.3 to 123.8)	6.8 (3.3 to 12.5)	9.0 (6.7 to 12.4)	583.1 (482.7 to 690.2)	363.4 (287.8 to 444.8)	186.5 (127.0 to 260.4)	14.4 (7.0 to 26.2)	18.8 (14.2 to 26.2)
Cape Verde	2042	282.4 (232.8 to 335.9)	175.8 (139.0 to 215.7)	90.5 (61.4 to 126.3)	7.0 (3.4 to 12.7)	9.2 (6.9 to 13.0)	594.2 (489.8 to 706.8)	369.9 (292.5 to 453.8)	190.4 (129.2 to 265.8)	14.6 (7.1 to 26.7)	19.3 (14.4 to 27.4)
Cape Verde	2043	287.6 (237.5 to 344.6)	178.9 (140.9 to 220.0)	92.3 (62.4 to 128.8)	7.1 (3.4 to 12.9)	9.4 (6.9 to 13.5)	605.2 (499.6 to 725.0)	376.4 (296.5 to 463.0)	194.1 (131.4 to 271.1)	14.9 (7.2 to 27.2)	19.7 (14.6 to 28.4)
Cape Verde	2044	293.0 (240.2 to 348.8)	182.1 (143.1 to 224.7)	94.0 (63.5 to 131.3)	7.2 (3.5 to 13.2)	9.6 (7.1 to 13.9)	616.5 (505.4 to 733.9)	383.2 (301.1 to 472.9)	197.8 (133.6 to 276.4)	15.3 (7.4 to 27.8)	20.2 (14.9 to 29.2)
Cape Verde	2045	298.1 (245.3 to 354.8)	185.2 (145.1 to 228.7)	95.7 (64.6 to 133.8)	7.4 (3.6 to 13.5)	9.8 (7.1 to 14.8)	627.2 (516.1 to 746.6)	389.7 (305.4 to 481.3)	201.3 (135.9 to 281.6)	15.5 (7.5 to 28.3)	20.7 (14.9 to 31.2)
Cape Verde	2046	302.8 (248.1 to 360.4)	188.0 (146.9 to 232.9)	97.3 (65.7 to 136.3)	7.5 (3.6 to 13.7)	10.0 (7.3 to 15.5)	637.2 (522.1 to 758.3)	395.6 (309.2 to 490.0)	204.7 (138.3 to 286.7)	15.8 (7.6 to 28.8)	21.1 (15.3 to 32.6)
Cape Verde	2047	307.5 (255.2 to 369.0)	190.7 (148.7 to 236.7)	98.8 (66.8 to 138.6)	7.7 (3.7 to 13.9)	10.3 (7.4 to 16.2)	647.0 (537.0 to 776.5)	401.4 (312.8 to 498.1)	207.8 (140.5 to 291.5)	16.1 (7.8 to 29.3)	21.7 (15.6 to 34.2)
Cape Verde	2048	312.0 (258.2 to 373.5)	193.4 (150.3 to 240.2)	100.3 (67.9 to 140.9)	7.8 (3.7 to 14.1)	10.6 (7.5 to 17.0)	656.5 (543.2 to 785.9)	406.9 (316.4 to 505.3)	211.0 (142.8 to 296.5)	16.4 (7.9 to 29.7)	22.3 (15.9 to 35.7)
Cape Verde	2049	316.5 (260.1 to 382.8)	195.9 (152.5 to 243.3)	101.8 (69.0 to 143.4)	7.9 (3.8 to 14.4)	10.9 (7.7 to 17.5)	665.9 (547.3 to 805.6)	412.2 (320.8 to 512.0)	214.2 (145.1 to 301.7)	16.7 (8.0 to 30.2)	22.9 (16.3 to 36.8)
Cape Verde	2050	320.9 (265.0 to 385.6)	198.4 (154.6 to 246.6)	103.3 (70.0 to 145.4)	8.0 (3.9 to 14.6)	11.2 (7.8 to 18.2)	675.3 (557.6 to 811.3)	417.5 (325.2 to 518.9)	217.3 (147.4 to 306.0)	16.9 (8.1 to 30.7)	23.5 (16.5 to 38.3)

		2018 US Dollars per capita					2018 PPP per capita				
Country	Year	Health spending	Government health spending	Out-of-pocket health spending	Prepaid private health spending	Development assistance for health	Health spending	Government health spending	Out-of-pocket health spending	Prepaid private health spending	Development assistance for health
Central African Republic	1995	24.3 (20.0 to 29.3)	8.1 (6.2 to 10.7)	12.1 (8.1 to 16.9)	0.3 (0.1 to 0.5)	3.8 (3.8 to 3.8)	42.0 (34.5 to 50.7)	14.1 (10.7 to 18.6)	20.9 (14.0 to 29.2)	0.5 (0.2 to 0.9)	6.5 (6.5 to 6.5)
Central African Republic	1996	19.9 (15.8 to 24.7)	7.6 (5.7 to 9.9)	11.7 (7.9 to 16.4)	0.3 (0.1 to 0.5)	0.4 (0.4 to 0.4)	34.4 (27.4 to 42.7)	13.1 (9.9 to 17.1)	20.1 (13.6 to 28.3)	0.5 (0.2 to 0.9)	0.7 (0.7 to 0.7)
Central African Republic	1997	20.5 (16.4 to 25.3)	7.0 (5.2 to 9.1)	11.5 (7.7 to 16.1)	0.3 (0.1 to 0.5)	1.8 (1.8 to 1.8)	35.4 (28.3 to 43.6)	12.0 (9.0 to 15.7)	19.9 (13.3 to 27.8)	0.4 (0.2 to 0.8)	3.0 (3.0 to 3.0)
Central African Republic	1998	25.0 (21.1 to 29.6)	6.6 (5.0 to 8.6)	11.3 (7.6 to 15.7)	0.2 (0.1 to 0.5)	6.9 (6.9 to 6.9)	43.2 (36.4 to 51.1)	11.4 (8.6 to 14.8)	19.5 (13.1 to 27.2)	0.4 (0.2 to 0.8)	11.9 (11.9 to 11.9)
Central African Republic	1999	23.7 (19.5 to 28.5)	8.0 (6.0 to 10.3)	11.6 (7.8 to 16.1)	0.3 (0.1 to 0.5)	3.9 (3.9 to 3.9)	41.0 (33.6 to 49.2)	13.7 (10.3 to 17.8)	20.0 (13.5 to 27.8)	0.4 (0.2 to 0.8)	6.8 (6.8 to 6.8)
Central African Republic	2000	22.3 (18.0 to 27.3)	9.0 (6.7 to 11.5)	11.5 (7.8 to 16.1)	0.3 (0.1 to 0.5)	1.6 (1.6 to 1.6)	38.5 (31.1 to 47.1)	15.5 (11.6 to 19.8)	19.9 (13.5 to 27.8)	0.4 (0.2 to 0.8)	2.7 (2.7 to 2.7)
Central African Republic	2001	23.2 (18.9 to 28.3)	9.4 (7.1 to 11.9)	11.4 (7.9 to 15.9)	0.3 (0.1 to 0.5)	2.1 (2.1 to 2.1)	40.0 (32.6 to 48.9)	16.2 (12.2 to 20.5)	19.8 (13.6 to 27.4)	0.4 (0.2 to 0.8)	3.5 (3.5 to 3.5)
Central African Republic	2002	24.5 (20.1 to 30.0)	9.7 (7.3 to 12.3)	11.5 (7.7 to 15.9)	0.3 (0.1 to 0.5)	3.0 (3.0 to 3.0)	42.3 (34.8 to 51.8)	16.8 (12.7 to 21.2)	19.8 (13.4 to 27.5)	0.4 (0.2 to 0.8)	5.2 (5.2 to 5.2)
Central African Republic	2003	22.7 (18.5 to 28.3)	9.2 (7.0 to 11.6)	11.1 (7.6 to 15.7)	0.2 (0.1 to 0.4)	2.2 (2.2 to 2.2)	39.2 (31.9 to 48.9)	15.8 (12.0 to 20.0)	19.2 (13.1 to 27.2)	0.4 (0.2 to 0.8)	3.7 (3.7 to 3.7)
Central African Republic	2004	23.8 (19.7 to 29.5)	9.1 (6.8 to 11.6)	11.2 (7.7 to 15.9)	0.2 (0.1 to 0.5)	3.3 (3.3 to 3.3)	41.1 (34.1 to 50.9)	15.6 (11.8 to 20.0)	19.3 (13.4 to 27.4)	0.4 (0.2 to 0.8)	5.7 (5.7 to 5.7)
Central African Republic	2005	24.4 (20.4 to 29.9)	9.0 (6.7 to 11.5)	11.0 (7.7 to 15.3)	0.3 (0.1 to 0.5)	4.2 (4.2 to 4.2)	42.2 (35.2 to 51.6)	15.5 (11.6 to 19.9)	19.0 (13.3 to 26.4)	0.4 (0.2 to 0.8)	7.3 (7.3 to 7.3)
Central African Republic	2006	25.0 (21.1 to 30.1)	8.9 (6.7 to 11.5)	10.9 (7.7 to 15.3)	0.3 (0.1 to 0.5)	5.0 (5.0 to 5.0)	43.3 (36.5 to 51.9)	15.3 (11.7 to 19.8)	18.8 (13.2 to 26.4)	0.5 (0.2 to 0.9)	8.7 (8.7 to 8.7)
Central African Republic	2007	22.5 (18.7 to 27.4)	8.6 (6.5 to 11.3)	10.8 (7.5 to 15.1)	0.3 (0.1 to 0.5)	2.7 (2.7 to 2.7)	38.8 (32.3 to 47.4)	14.9 (11.3 to 19.5)	18.7 (13.0 to 26.0)	0.5 (0.2 to 0.9)	4.7 (4.7 to 4.7)
Central African Republic	2008	26.5 (23.1 to 31.5)	7.5 (5.6 to 9.8)	10.4 (7.3 to 14.6)	0.3 (0.1 to 0.5)	8.3 (8.3 to 8.3)	45.8 (39.8 to 54.5)	12.9 (9.8 to 17.0)	18.0 (12.6 to 25.3)	0.5 (0.2 to 0.9)	14.4 (14.4 to 14.4)
Central African Republic	2009	19.8 (16.5 to 24.6)	6.2 (4.7 to 8.1)	10.1 (7.1 to 14.5)	0.3 (0.1 to 0.5)	3.2 (3.2 to 3.2)	34.3 (28.5 to 42.5)	10.7 (8.2 to 13.9)	17.5 (12.2 to 25.1)	0.5 (0.2 to 0.9)	5.5 (5.5 to 5.5)
Central African Republic	2010	21.7 (18.2 to 26.5)	6.1 (4.6 to 7.9)	10.3 (7.2 to 14.9)	0.3 (0.1 to 0.6)	5.0 (5.0 to 5.0)	37.5 (31.5 to 45.8)	10.5 (7.9 to 13.7)	17.8 (12.4 to 25.8)	0.5 (0.3 to 1.0)	8.6 (8.6 to 8.6)
Central African Republic	2011	21.5 (18.0 to 26.5)	5.7 (4.3 to 7.3)	10.3 (7.1 to 14.8)	0.3 (0.1 to 0.6)	5.2 (5.2 to 5.2)	37.1 (31.2 to 45.8)	9.8 (7.4 to 12.7)	17.7 (12.2 to 25.6)	0.6 (0.3 to 1.0)	9.0 (9.0 to 9.0)
Central African Republic	2012	21.4 (17.9 to 26.4)	5.4 (4.1 to 7.1)	10.5 (7.2 to 15.1)	0.3 (0.1 to 0.6)	5.1 (5.1 to 5.1)	36.9 (30.9 to 45.6)	9.4 (7.0 to 12.2)	18.1 (12.5 to 26.0)	0.6 (0.3 to 1.0)	8.9 (8.9 to 8.9)
Central African Republic	2013	18.2 (15.5 to 22.0)	3.3 (2.5 to 4.2)	8.5 (5.9 to 12.0)	0.2 (0.1 to 0.4)	6.2 (6.2 to 6.2)	31.5 (26.8 to 38.0)	5.7 (4.3 to 7.3)	14.6 (10.2 to 20.8)	0.4 (0.2 to 0.7)	10.8 (10.8 to 10.8)
Central African Republic	2014	20.6 (18.0 to 24.1)	3.2 (2.4 to 4.1)	8.2 (5.8 to 11.7)	0.2 (0.1 to 0.4)	8.9 (8.9 to 8.9)	35.6 (31.1 to 41.6)	5.6 (4.1 to 7.1)	14.2 (9.9 to 20.2)	0.4 (0.2 to 0.7)	15.4 (15.4 to 15.4)
Central African Republic	2015	15.9 (13.3 to 19.3)	3.0 (2.2 to 3.9)	8.0 (5.5 to 11.3)	0.2 (0.1 to 0.4)	4.7 (4.7 to 4.7)	27.5 (22.9 to 33.3)	5.2 (3.9 to 6.7)	13.8 (9.6 to 19.5)	0.4 (0.2 to 0.7)	8.2 (8.2 to 8.2)

Country	Year	2018 US Dollars per capita					2018 PPP per capita				
		Health spending	Government health spending	Out-of-pocket health spending	Prepaid private health spending	Development assistance for health	Health spending	Government health spending	Out-of-pocket health spending	Prepaid private health spending	Development assistance for health
Central African Republic	2016	21.6 (19.0 to 24.7)	2.9 (2.2 to 3.8)	7.9 (5.5 to 10.8)	0.2 (0.1 to 0.4)	10.6 (10.6 to 10.6)	37.3 (32.9 to 42.6)	5.0 (3.8 to 6.5)	13.6 (9.4 to 18.6)	0.4 (0.2 to 0.7)	18.3 (18.3 to 18.3)
Central African Republic	2017	21.8 (19.1 to 25.1)	3.3 (2.4 to 4.2)	8.2 (5.7 to 11.2)	0.2 (0.1 to 0.4)	10.2 (10.2 to 10.2)	37.7 (33.0 to 43.4)	5.7 (4.2 to 7.3)	14.1 (9.8 to 19.4)	0.4 (0.2 to 0.7)	17.5 (17.5 to 17.5)
Central African Republic	2018	21.0 (18.3 to 24.2)	3.3 (2.5 to 4.3)	8.2 (5.7 to 11.3)	0.2 (0.1 to 0.4)	9.3 (9.2 to 9.3)	36.3 (31.5 to 41.8)	5.7 (4.3 to 7.4)	14.2 (9.8 to 19.5)	0.4 (0.2 to 0.7)	16.0 (15.9 to 16.1)
Central African Republic	2019	21.2 (18.4 to 24.7)	3.4 (2.5 to 4.3)	8.2 (5.7 to 11.3)	0.2 (0.1 to 0.4)	9.4 (8.8 to 9.9)	36.7 (31.9 to 42.7)	5.8 (4.3 to 7.5)	14.2 (9.9 to 19.5)	0.4 (0.2 to 0.8)	16.2 (15.2 to 17.1)
Central African Republic	2020	21.5 (18.7 to 24.9)	3.4 (2.5 to 4.4)	8.3 (5.7 to 11.3)	0.2 (0.1 to 0.4)	9.6 (8.8 to 10.4)	37.2 (32.3 to 43.0)	5.9 (4.4 to 7.6)	14.3 (9.9 to 19.5)	0.4 (0.2 to 0.8)	16.6 (15.3 to 18.0)
Central African Republic	2021	21.8 (18.7 to 25.3)	3.5 (2.6 to 4.5)	8.3 (5.7 to 11.4)	0.2 (0.1 to 0.4)	9.8 (8.8 to 10.8)	37.6 (32.4 to 43.6)	6.0 (4.4 to 7.7)	14.3 (9.8 to 19.7)	0.4 (0.2 to 0.8)	16.9 (15.2 to 18.7)
Central African Republic	2022	22.1 (19.0 to 25.4)	3.5 (2.6 to 4.5)	8.3 (5.7 to 11.4)	0.2 (0.1 to 0.5)	10.1 (8.9 to 11.3)	38.2 (32.8 to 43.9)	6.0 (4.5 to 7.8)	14.3 (9.8 to 19.7)	0.4 (0.2 to 0.8)	17.4 (15.4 to 19.5)
Central African Republic	2023	22.4 (19.2 to 26.0)	3.5 (2.6 to 4.6)	8.3 (5.7 to 11.4)	0.2 (0.1 to 0.5)	10.3 (8.9 to 11.8)	38.7 (33.2 to 44.9)	6.1 (4.6 to 8.0)	14.3 (9.9 to 19.8)	0.4 (0.2 to 0.8)	17.8 (15.4 to 20.4)
Central African Republic	2024	22.7 (19.5 to 26.6)	3.6 (2.7 to 4.6)	8.3 (5.7 to 11.4)	0.2 (0.1 to 0.5)	10.6 (9.0 to 12.3)	39.2 (33.6 to 46.0)	6.2 (4.6 to 8.0)	14.3 (9.9 to 19.8)	0.4 (0.2 to 0.8)	18.3 (15.6 to 21.2)
Central African Republic	2025	23.0 (19.5 to 26.9)	3.6 (2.7 to 4.7)	8.3 (5.8 to 11.5)	0.2 (0.1 to 0.5)	10.9 (9.2 to 12.7)	39.7 (33.6 to 46.4)	6.2 (4.6 to 8.1)	14.3 (10.0 to 19.8)	0.4 (0.2 to 0.8)	18.7 (15.9 to 22.0)
Central African Republic	2026	23.3 (19.9 to 27.3)	3.6 (2.7 to 4.7)	8.3 (5.8 to 11.5)	0.3 (0.1 to 0.5)	11.1 (9.3 to 13.0)	40.2 (34.4 to 47.1)	6.3 (4.6 to 8.2)	14.4 (10.0 to 19.8)	0.4 (0.2 to 0.8)	19.1 (16.0 to 22.5)
Central African Republic	2027	23.6 (20.1 to 28.0)	3.7 (2.7 to 4.8)	8.4 (5.8 to 11.5)	0.3 (0.1 to 0.5)	11.3 (9.5 to 13.4)	40.8 (34.7 to 48.3)	6.4 (4.7 to 8.3)	14.4 (10.0 to 19.9)	0.4 (0.2 to 0.8)	19.6 (16.3 to 23.2)
Central African Republic	2028	23.9 (20.4 to 28.3)	3.7 (2.8 to 4.8)	8.4 (5.8 to 11.6)	0.3 (0.1 to 0.5)	11.6 (9.7 to 14.0)	41.4 (35.2 to 48.8)	6.4 (4.8 to 8.4)	14.5 (10.0 to 20.1)	0.4 (0.2 to 0.9)	20.0 (16.7 to 24.1)
Central African Republic	2029	24.3 (20.7 to 28.8)	3.8 (2.8 to 4.9)	8.4 (5.8 to 11.7)	0.3 (0.1 to 0.5)	11.9 (9.7 to 14.6)	41.9 (35.7 to 49.7)	6.5 (4.8 to 8.4)	14.5 (10.0 to 20.2)	0.5 (0.2 to 0.9)	20.5 (16.8 to 25.1)
Central African Republic	2030	24.6 (20.9 to 28.9)	3.8 (2.8 to 4.9)	8.4 (5.8 to 11.6)	0.3 (0.1 to 0.5)	12.1 (9.8 to 15.2)	42.5 (36.0 to 50.0)	6.6 (4.8 to 8.5)	14.5 (10.0 to 20.1)	0.5 (0.2 to 0.9)	20.9 (17.0 to 26.2)
Central African Republic	2031	25.0 (20.8 to 29.6)	3.8 (2.8 to 5.0)	8.5 (5.8 to 11.7)	0.3 (0.1 to 0.5)	12.4 (9.9 to 15.7)	43.1 (36.0 to 51.2)	6.6 (4.9 to 8.6)	14.6 (10.0 to 20.2)	0.5 (0.2 to 0.9)	21.4 (17.1 to 27.1)
Central African Republic	2032	25.4 (21.1 to 30.3)	3.9 (2.8 to 5.0)	8.5 (5.8 to 11.8)	0.3 (0.1 to 0.5)	12.7 (10.1 to 16.3)	43.8 (36.5 to 52.3)	6.7 (4.9 to 8.7)	14.7 (10.0 to 20.3)	0.5 (0.2 to 0.9)	22.0 (17.5 to 28.1)
Central African Republic	2033	25.8 (21.4 to 30.8)	3.9 (2.9 to 5.1)	8.5 (5.8 to 11.9)	0.3 (0.1 to 0.5)	13.0 (10.2 to 17.1)	44.5 (37.0 to 53.2)	6.7 (4.9 to 8.8)	14.7 (10.0 to 20.6)	0.5 (0.2 to 0.9)	22.5 (17.6 to 29.5)
Central African Republic	2034	26.2 (21.6 to 31.8)	3.9 (2.9 to 5.1)	8.6 (5.8 to 12.0)	0.3 (0.1 to 0.5)	13.4 (10.4 to 17.8)	45.3 (37.4 to 54.9)	6.8 (5.0 to 8.9)	14.8 (10.0 to 20.7)	0.5 (0.2 to 0.9)	23.2 (18.0 to 30.8)
Central African Republic	2035	26.7 (22.1 to 32.5)	4.0 (2.9 to 5.2)	8.6 (5.8 to 12.1)	0.3 (0.1 to 0.5)	13.9 (10.7 to 18.3)	46.1 (38.1 to 56.1)	6.8 (5.0 to 8.9)	14.9 (10.0 to 20.9)	0.5 (0.2 to 0.9)	24.0 (18.4 to 31.6)
Central African Republic	2036	27.2 (22.2 to 33.3)	4.0 (2.9 to 5.2)	8.7 (5.8 to 12.2)	0.3 (0.1 to 0.6)	14.3 (10.8 to 19.5)	47.1 (38.4 to 57.5)	6.9 (5.0 to 9.0)	14.9 (10.0 to 21.0)	0.5 (0.2 to 1.0)	24.7 (18.7 to 33.6)

Country	Year	2018 US Dollars per capita					2018 PPP per capita				
		Health spending	Government health spending	Out-of-pocket health spending	Prepaid private health spending	Development assistance for health	Health spending	Government health spending	Out-of-pocket health spending	Prepaid private health spending	Development assistance for health
Central African Republic	2037	27.8 (22.7 to 34.5)	4.0 (2.9 to 5.3)	8.7 (5.8 to 12.2)	0.3 (0.1 to 0.6)	14.7 (11.0 to 20.1)	48.0 (39.2 to 59.6)	6.9 (5.1 to 9.1)	15.0 (10.1 to 21.0)	0.5 (0.2 to 1.0)	25.5 (19.0 to 34.7)
Central African Republic	2038	28.4 (22.9 to 35.0)	4.1 (2.9 to 5.3)	8.8 (5.9 to 12.3)	0.3 (0.1 to 0.6)	15.2 (11.1 to 21.3)	49.0 (39.6 to 60.4)	7.0 (5.1 to 9.2)	15.1 (10.1 to 21.3)	0.5 (0.2 to 1.0)	26.3 (19.2 to 36.8)
Central African Republic	2039	28.9 (23.6 to 36.2)	4.1 (3.0 to 5.4)	8.8 (5.9 to 12.4)	0.3 (0.1 to 0.6)	15.7 (11.6 to 22.2)	50.0 (40.7 to 62.5)	7.1 (5.1 to 9.3)	15.2 (10.2 to 21.5)	0.5 (0.2 to 1.0)	27.2 (20.0 to 38.3)
Central African Republic	2040	29.6 (23.8 to 37.3)	4.1 (3.0 to 5.4)	8.9 (5.9 to 12.6)	0.3 (0.1 to 0.6)	16.3 (11.9 to 23.4)	51.1 (41.1 to 64.4)	7.1 (5.1 to 9.4)	15.3 (10.2 to 21.7)	0.5 (0.3 to 1.0)	28.1 (20.5 to 40.3)
Central African Republic	2041	30.3 (24.1 to 38.6)	4.2 (3.0 to 5.5)	8.9 (5.9 to 12.7)	0.3 (0.1 to 0.6)	16.9 (12.2 to 24.6)	52.3 (41.6 to 66.7)	7.2 (5.2 to 9.5)	15.5 (10.2 to 21.9)	0.5 (0.3 to 1.0)	29.1 (21.0 to 42.5)
Central African Republic	2042	31.0 (24.5 to 39.6)	4.2 (3.0 to 5.6)	9.0 (6.0 to 12.9)	0.3 (0.1 to 0.6)	17.5 (12.3 to 25.9)	53.5 (42.3 to 68.4)	7.2 (5.2 to 9.7)	15.6 (10.3 to 22.2)	0.5 (0.3 to 1.0)	30.2 (21.2 to 44.7)
Central African Republic	2043	31.7 (24.7 to 41.5)	4.2 (3.0 to 5.7)	9.1 (6.0 to 13.0)	0.3 (0.2 to 0.6)	18.1 (12.6 to 27.2)	54.8 (42.7 to 71.7)	7.3 (5.3 to 9.8)	15.7 (10.4 to 22.5)	0.5 (0.3 to 1.0)	31.2 (21.7 to 46.9)
Central African Republic	2044	32.5 (25.4 to 42.7)	4.3 (3.1 to 5.7)	9.2 (6.1 to 13.2)	0.3 (0.2 to 0.6)	18.7 (12.8 to 28.3)	56.2 (43.8 to 73.8)	7.4 (5.3 to 9.8)	15.9 (10.5 to 22.7)	0.6 (0.3 to 1.1)	32.3 (22.2 to 48.8)
Central African Republic	2045	33.3 (26.2 to 44.7)	4.3 (3.1 to 5.8)	9.3 (6.1 to 13.3)	0.3 (0.2 to 0.6)	19.4 (13.2 to 30.1)	57.6 (45.2 to 77.2)	7.5 (5.4 to 10.0)	16.1 (10.6 to 23.0)	0.6 (0.3 to 1.1)	33.5 (22.9 to 51.9)
Central African Republic	2046	34.2 (26.4 to 46.5)	4.4 (3.2 to 5.9)	9.4 (6.2 to 13.5)	0.3 (0.2 to 0.6)	20.0 (13.5 to 31.9)	59.1 (45.5 to 80.2)	7.6 (5.4 to 10.2)	16.3 (10.7 to 23.3)	0.6 (0.3 to 1.1)	34.6 (23.3 to 55.1)
Central African Republic	2047	35.1 (27.0 to 48.5)	4.5 (3.2 to 6.1)	9.5 (6.3 to 13.6)	0.3 (0.2 to 0.6)	20.8 (13.8 to 33.8)	60.7 (46.6 to 83.7)	7.8 (5.6 to 10.5)	16.5 (10.9 to 23.6)	0.6 (0.3 to 1.1)	35.9 (23.9 to 58.3)
Central African Republic	2048	36.2 (27.6 to 50.2)	4.6 (3.3 to 6.2)	9.7 (6.4 to 13.8)	0.3 (0.2 to 0.7)	21.5 (14.2 to 35.6)	62.5 (47.7 to 86.6)	8.0 (5.7 to 10.7)	16.7 (11.0 to 23.9)	0.6 (0.3 to 1.1)	37.2 (24.5 to 61.5)
Central African Republic	2049	37.2 (28.3 to 53.2)	4.7 (3.4 to 6.4)	9.8 (6.5 to 14.0)	0.3 (0.2 to 0.7)	22.3 (14.5 to 38.6)	64.2 (48.9 to 92.0)	8.2 (5.8 to 11.0)	17.0 (11.1 to 24.2)	0.6 (0.3 to 1.2)	38.5 (25.0 to 66.7)
Central African Republic	2050	38.3 (28.5 to 55.4)	4.9 (3.4 to 6.6)	10.0 (6.5 to 14.2)	0.3 (0.2 to 0.7)	23.2 (14.6 to 39.8)	66.2 (49.3 to 95.6)	8.4 (6.0 to 11.4)	17.2 (11.3 to 24.6)	0.6 (0.3 to 1.2)	40.0 (25.2 to 68.7)
Chad	1995	34.6 (27.9 to 42.6)	7.5 (5.7 to 9.5)	20.2 (14.2 to 28.3)	1.5 (0.7 to 2.9)	5.4 (5.4 to 5.4)	95.1 (76.7 to 117.3)	20.6 (15.6 to 26.2)	55.5 (39.1 to 77.8)	4.2 (2.0 to 7.9)	14.9 (14.9 to 14.9)
Chad	1996	31.7 (25.3 to 39.6)	7.5 (5.7 to 9.6)	19.8 (14.0 to 27.6)	1.5 (0.7 to 2.8)	2.9 (2.9 to 2.9)	87.1 (69.5 to 109.1)	20.6 (15.8 to 26.4)	54.5 (38.6 to 75.9)	4.1 (1.9 to 7.7)	7.9 (7.9 to 7.9)
Chad	1997	33.4 (27.1 to 41.3)	7.8 (5.9 to 10.1)	20.0 (14.0 to 27.4)	1.5 (0.7 to 2.7)	4.2 (4.2 to 4.2)	91.9 (74.6 to 113.7)	21.5 (16.3 to 27.7)	54.9 (38.6 to 75.4)	4.0 (1.9 to 7.5)	11.5 (11.5 to 11.5)
Chad	1998	32.2 (25.9 to 39.9)	8.0 (6.0 to 10.3)	19.8 (13.8 to 26.8)	1.4 (0.7 to 2.6)	3.1 (3.1 to 3.1)	88.7 (71.2 to 109.7)	22.0 (16.6 to 28.5)	54.4 (37.8 to 73.8)	3.8 (1.8 to 7.1)	8.4 (8.4 to 8.4)
Chad	1999	33.1 (26.7 to 40.6)	8.5 (6.4 to 10.9)	19.2 (13.5 to 26.4)	1.3 (0.6 to 2.4)	4.2 (4.2 to 4.2)	91.2 (73.5 to 111.8)	23.3 (17.7 to 30.1)	52.8 (37.1 to 72.6)	3.5 (1.7 to 6.5)	11.5 (11.5 to 11.5)
Chad	2000	30.8 (25.0 to 37.7)	9.0 (6.8 to 11.6)	17.7 (12.5 to 24.3)	1.1 (0.5 to 1.9)	3.1 (3.1 to 3.1)	84.8 (68.7 to 103.7)	24.6 (18.8 to 31.8)	48.6 (34.3 to 66.8)	2.9 (1.3 to 5.3)	8.7 (8.7 to 8.7)
Chad	2001	31.7 (25.6 to 39.2)	9.7 (7.4 to 12.6)	18.4 (12.8 to 25.5)	1.1 (0.5 to 2.1)	2.5 (2.5 to 2.5)	87.2 (70.5 to 107.7)	26.6 (20.4 to 34.6)	50.5 (35.3 to 70.1)	3.2 (1.5 to 5.7)	6.9 (6.9 to 6.9)

		2018 US Dollars per capita					2018 PPP per capita				
Country	Year	Health spending	Government health spending	Out-of-pocket health spending	Prepaid private health spending	Development assistance for health	Health spending	Government health spending	Out-of-pocket health spending	Prepaid private health spending	Development assistance for health
Chad	2002	33.6 (27.2 to 41.1)	9.7 (7.5 to 12.5)	19.6 (13.8 to 26.7)	1.3 (0.6 to 2.3)	3.0 (3.0 to 3.0)	92.5 (74.8 to 113.2)	26.8 (20.6 to 34.4)	54.0 (38.0 to 73.6)	3.5 (1.7 to 6.4)	8.2 (8.2 to 8.2)
Chad	2003	34.4 (28.2 to 41.6)	10.0 (7.7 to 12.8)	18.9 (13.4 to 25.8)	1.4 (0.7 to 2.6)	4.2 (4.2 to 4.2)	94.7 (77.5 to 114.4)	27.5 (21.2 to 35.1)	51.9 (36.8 to 70.9)	3.9 (1.9 to 7.2)	11.5 (11.5 to 11.5)
Chad	2004	39.3 (32.1 to 47.9)	11.8 (9.1 to 15.1)	21.7 (15.7 to 29.8)	1.7 (0.9 to 3.2)	4.0 (4.0 to 4.0)	108.0 (88.2 to 131.8)	32.5 (24.9 to 41.5)	59.8 (43.2 to 82.1)	4.8 (2.4 to 8.8)	11.0 (11.0 to 11.0)
Chad	2005	39.7 (32.5 to 48.3)	10.8 (8.3 to 13.9)	22.4 (16.0 to 30.7)	1.8 (0.9 to 3.4)	4.7 (4.7 to 4.7)	109.3 (89.3 to 133.0)	29.8 (22.9 to 38.1)	61.6 (44.1 to 84.4)	5.0 (2.4 to 9.4)	12.8 (12.8 to 12.8)
Chad	2006	35.6 (28.9 to 43.9)	8.6 (6.7 to 11.1)	21.9 (15.4 to 29.7)	1.7 (0.8 to 3.3)	3.3 (3.3 to 3.3)	97.9 (79.4 to 120.8)	23.8 (18.3 to 30.6)	60.2 (42.5 to 81.6)	4.7 (2.2 to 9.0)	9.2 (9.2 to 9.2)
Chad	2007	33.3 (26.4 to 41.3)	7.8 (6.0 to 10.1)	21.5 (14.9 to 29.3)	1.7 (0.8 to 3.3)	2.3 (2.3 to 2.3)	91.6 (72.6 to 113.5)	21.4 (16.4 to 27.8)	59.1 (41.1 to 80.6)	4.8 (2.3 to 9.1)	6.3 (6.3 to 6.3)
Chad	2008	33.0 (26.2 to 40.6)	7.0 (5.3 to 9.2)	21.0 (14.8 to 28.5)	1.7 (0.8 to 3.4)	3.2 (3.2 to 3.2)	90.8 (72.1 to 111.8)	19.4 (14.7 to 25.4)	57.9 (40.8 to 78.4)	4.7 (2.3 to 9.2)	8.8 (8.8 to 8.8)
Chad	2009	32.2 (25.8 to 40.2)	6.8 (5.2 to 9.0)	20.8 (14.8 to 28.6)	1.8 (0.8 to 3.4)	2.8 (2.8 to 2.8)	88.7 (71.0 to 110.5)	18.7 (14.3 to 24.8)	57.3 (40.6 to 78.7)	4.8 (2.3 to 9.3)	7.8 (7.8 to 7.8)
Chad	2010	35.7 (29.2 to 44.1)	7.4 (5.6 to 9.8)	21.5 (15.6 to 29.6)	1.9 (0.9 to 3.5)	5.0 (5.0 to 5.0)	98.3 (80.4 to 121.5)	20.4 (15.4 to 26.9)	59.1 (43.0 to 81.5)	5.1 (2.4 to 9.7)	13.6 (13.6 to 13.6)
Chad	2011	32.8 (26.7 to 40.4)	7.2 (5.4 to 9.4)	19.9 (14.2 to 27.3)	1.7 (0.8 to 3.2)	4.1 (4.1 to 4.1)	90.4 (73.5 to 111.2)	19.7 (14.9 to 25.8)	54.7 (39.1 to 75.0)	4.7 (2.2 to 8.9)	11.3 (11.3 to 11.3)
Chad	2012	32.6 (26.6 to 40.6)	7.8 (5.9 to 10.2)	20.0 (14.3 to 27.3)	1.8 (0.8 to 3.4)	3.1 (3.1 to 3.1)	89.8 (73.1 to 111.7)	21.5 (16.3 to 28.2)	54.9 (39.4 to 75.1)	4.8 (2.3 to 9.4)	8.6 (8.6 to 8.6)
Chad	2013	40.1 (33.6 to 48.5)	10.0 (7.6 to 13.0)	21.3 (15.2 to 29.1)	2.0 (1.0 to 3.9)	6.8 (6.8 to 6.8)	110.4 (92.4 to 133.6)	27.5 (20.9 to 35.8)	58.6 (41.9 to 80.1)	5.6 (2.7 to 10.8)	18.8 (18.8 to 18.8)
Chad	2014	38.4 (31.6 to 46.7)	10.0 (7.5 to 13.0)	21.6 (15.5 to 29.3)	2.1 (1.0 to 3.9)	4.8 (4.8 to 4.8)	105.7 (87.0 to 128.4)	27.5 (20.7 to 35.7)	59.5 (42.6 to 80.8)	5.7 (2.7 to 10.7)	13.1 (13.1 to 13.1)
Chad	2015	35.8 (28.9 to 44.0)	9.2 (7.0 to 11.9)	21.8 (15.5 to 29.5)	2.0 (1.0 to 3.9)	2.8 (2.8 to 2.8)	98.6 (79.5 to 121.2)	25.3 (19.2 to 32.8)	59.9 (42.7 to 81.3)	5.6 (2.6 to 10.7)	7.7 (7.7 to 7.7)
Chad	2016	35.9 (29.3 to 43.5)	7.8 (6.0 to 10.2)	20.9 (14.8 to 28.5)	1.9 (0.8 to 3.6)	5.3 (5.3 to 5.3)	98.8 (80.5 to 119.7)	21.5 (16.6 to 28.0)	57.6 (40.7 to 78.4)	5.1 (2.3 to 9.8)	14.5 (14.5 to 14.5)
Chad	2017	32.9 (26.4 to 40.1)	7.5 (5.8 to 9.8)	19.8 (13.9 to 27.0)	1.8 (0.8 to 3.4)	3.8 (3.8 to 3.8)	90.5 (72.8 to 110.2)	20.7 (16.0 to 27.0)	54.5 (38.4 to 74.2)	4.9 (2.2 to 9.3)	10.4 (10.4 to 10.4)
Chad	2018	32.3 (25.9 to 40.1)	7.5 (5.8 to 9.7)	19.7 (13.8 to 26.8)	1.8 (0.8 to 3.4)	3.4 (3.4 to 3.4)	89.0 (71.3 to 110.4)	20.6 (15.9 to 26.8)	54.1 (38.0 to 73.6)	4.9 (2.2 to 9.4)	9.4 (9.3 to 9.5)
Chad	2019	32.3 (26.1 to 39.7)	7.5 (5.8 to 9.7)	19.6 (13.8 to 26.6)	1.8 (0.8 to 3.4)	3.4 (3.1 to 3.6)	88.8 (71.7 to 109.2)	20.7 (15.9 to 26.7)	53.9 (37.9 to 73.1)	4.9 (2.3 to 9.4)	9.2 (8.6 to 9.8)
Chad	2020	32.2 (26.0 to 39.7)	7.5 (5.8 to 9.7)	19.5 (13.7 to 26.6)	1.8 (0.8 to 3.4)	3.3 (3.0 to 3.6)	88.6 (71.4 to 109.2)	20.7 (15.9 to 26.6)	53.8 (37.7 to 73.2)	5.0 (2.3 to 9.5)	9.2 (8.4 to 10.0)
Chad	2021	32.2 (26.0 to 40.0)	7.5 (5.8 to 9.7)	19.5 (13.6 to 26.5)	1.8 (0.8 to 3.5)	3.3 (3.0 to 3.7)	88.5 (71.5 to 110.0)	20.7 (15.9 to 26.7)	53.6 (37.5 to 72.9)	5.0 (2.3 to 9.5)	9.2 (8.2 to 10.2)
Chad	2022	32.2 (25.8 to 39.6)	7.5 (5.8 to 9.7)	19.4 (13.6 to 26.4)	1.8 (0.8 to 3.5)	3.4 (3.0 to 3.8)	88.5 (71.1 to 108.9)	20.7 (16.0 to 26.8)	53.5 (37.4 to 72.7)	5.0 (2.3 to 9.6)	9.2 (8.1 to 10.4)

Country	Year	2018 US Dollars per capita					2018 PPP per capita				
		Health spending	Government health spending	Out-of-pocket health spending	Prepaid private health spending	Development assistance for health	Health spending	Government health spending	Out-of-pocket health spending	Prepaid private health spending	Development assistance for health
Chad	2023	32.2 (25.7 to 39.2)	7.5 (5.8 to 9.7)	19.4 (13.6 to 26.2)	1.8 (0.8 to 3.5)	3.4 (2.9 to 3.9)	88.5 (70.7 to 107.8)	20.8 (16.0 to 26.8)	53.4 (37.4 to 72.1)	5.0 (2.3 to 9.7)	9.3 (8.1 to 10.7)
Chad	2024	32.1 (25.9 to 39.2)	7.5 (5.8 to 9.7)	19.4 (13.6 to 26.2)	1.8 (0.8 to 3.5)	3.4 (2.9 to 4.0)	88.4 (71.3 to 107.7)	20.7 (15.9 to 26.7)	53.2 (37.4 to 72.1)	5.1 (2.3 to 9.7)	9.4 (8.1 to 10.9)
Chad	2025	32.1 (25.8 to 39.6)	7.5 (5.7 to 9.7)	19.3 (13.5 to 26.0)	1.8 (0.8 to 3.5)	3.4 (2.9 to 4.0)	88.2 (71.0 to 108.9)	20.6 (15.8 to 26.6)	53.1 (37.2 to 71.6)	5.1 (2.3 to 9.7)	9.5 (8.0 to 11.0)
Chad	2026	32.0 (25.8 to 39.5)	7.5 (5.7 to 9.7)	19.2 (13.5 to 26.0)	1.9 (0.8 to 3.6)	3.4 (2.9 to 4.0)	88.1 (71.0 to 108.8)	20.6 (15.8 to 26.6)	52.9 (37.1 to 71.5)	5.1 (2.3 to 9.8)	9.5 (7.9 to 11.1)
Chad	2027	32.0 (25.7 to 39.3)	7.5 (5.7 to 9.6)	19.2 (13.5 to 26.0)	1.9 (0.9 to 3.6)	3.5 (2.9 to 4.1)	87.9 (70.6 to 108.1)	20.5 (15.7 to 26.5)	52.8 (37.2 to 71.4)	5.1 (2.3 to 9.8)	9.5 (7.9 to 11.4)
Chad	2028	32.0 (25.8 to 38.9)	7.5 (5.7 to 9.6)	19.1 (13.5 to 26.0)	1.9 (0.9 to 3.6)	3.5 (2.9 to 4.3)	87.9 (71.1 to 107.2)	20.5 (15.7 to 26.5)	52.7 (37.1 to 71.4)	5.2 (2.4 to 9.9)	9.6 (7.9 to 11.7)
Chad	2029	32.0 (25.7 to 39.2)	7.5 (5.7 to 9.6)	19.1 (13.4 to 25.9)	1.9 (0.9 to 3.6)	3.5 (2.8 to 4.3)	88.0 (70.8 to 107.9)	20.6 (15.8 to 26.5)	52.6 (36.9 to 71.1)	5.2 (2.4 to 10.0)	9.6 (7.8 to 11.9)
Chad	2030	32.1 (26.0 to 39.2)	7.5 (5.8 to 9.7)	19.1 (13.4 to 25.8)	1.9 (0.9 to 3.7)	3.5 (2.8 to 4.4)	88.3 (71.5 to 107.7)	20.7 (15.8 to 26.7)	52.6 (36.9 to 71.1)	5.2 (2.4 to 10.1)	9.7 (7.8 to 12.0)
Chad	2031	32.1 (26.0 to 39.4)	7.5 (5.8 to 9.7)	19.1 (13.4 to 25.9)	1.9 (0.9 to 3.7)	3.5 (2.8 to 4.5)	88.3 (71.6 to 108.3)	20.8 (15.8 to 26.7)	52.6 (36.8 to 71.2)	5.3 (2.4 to 10.2)	9.7 (7.7 to 12.3)
Chad	2032	32.1 (25.7 to 39.6)	7.6 (5.7 to 9.8)	19.1 (13.3 to 25.9)	1.9 (0.9 to 3.7)	3.5 (2.8 to 4.6)	88.4 (70.7 to 109.0)	20.8 (15.8 to 26.8)	52.5 (36.7 to 71.3)	5.3 (2.4 to 10.2)	9.8 (7.6 to 12.6)
Chad	2033	32.2 (25.9 to 39.6)	7.6 (5.7 to 9.8)	19.1 (13.3 to 25.9)	1.9 (0.9 to 3.8)	3.6 (2.7 to 4.7)	88.5 (71.4 to 109.0)	20.8 (15.8 to 26.9)	52.5 (36.6 to 71.4)	5.4 (2.4 to 10.3)	9.8 (7.5 to 12.8)
Chad	2034	32.2 (26.1 to 39.7)	7.6 (5.7 to 9.8)	19.1 (13.3 to 26.0)	2.0 (0.9 to 3.8)	3.6 (2.7 to 4.8)	88.6 (71.7 to 109.2)	20.9 (15.8 to 27.0)	52.4 (36.5 to 71.4)	5.4 (2.5 to 10.4)	9.9 (7.5 to 13.1)
Chad	2035	32.3 (26.0 to 39.8)	7.6 (5.7 to 9.8)	19.0 (13.3 to 25.9)	2.0 (0.9 to 3.8)	3.6 (2.7 to 4.9)	88.8 (71.5 to 109.5)	21.0 (15.8 to 27.1)	52.4 (36.5 to 71.4)	5.4 (2.5 to 10.5)	10.0 (7.4 to 13.5)
Chad	2036	32.3 (26.1 to 40.4)	7.6 (5.8 to 9.9)	19.0 (13.3 to 25.9)	2.0 (0.9 to 3.8)	3.7 (2.7 to 5.1)	88.9 (71.7 to 111.1)	21.0 (15.8 to 27.2)	52.4 (36.5 to 71.3)	5.5 (2.5 to 10.6)	10.1 (7.4 to 14.0)
Chad	2037	32.4 (25.9 to 39.8)	7.7 (5.8 to 9.9)	19.0 (13.3 to 26.0)	2.0 (0.9 to 3.9)	3.7 (2.7 to 5.1)	89.1 (71.3 to 109.6)	21.2 (15.9 to 27.4)	52.4 (36.5 to 71.5)	5.5 (2.5 to 10.7)	10.1 (7.4 to 14.1)
Chad	2038	32.5 (26.0 to 40.2)	7.7 (5.8 to 10.0)	19.0 (13.3 to 25.9)	2.0 (0.9 to 3.9)	3.7 (2.7 to 5.3)	89.3 (71.7 to 110.6)	21.3 (16.0 to 27.5)	52.3 (36.5 to 71.3)	5.6 (2.5 to 10.8)	10.2 (7.3 to 14.5)
Chad	2039	32.5 (26.1 to 40.3)	7.8 (5.8 to 10.1)	19.0 (13.3 to 25.9)	2.0 (0.9 to 4.0)	3.7 (2.6 to 5.4)	89.5 (71.8 to 110.9)	21.3 (16.0 to 27.7)	52.3 (36.6 to 71.2)	5.6 (2.5 to 10.9)	10.3 (7.3 to 14.9)
Chad	2040	32.6 (26.3 to 40.0)	7.8 (5.8 to 10.1)	19.0 (13.3 to 25.9)	2.1 (0.9 to 4.0)	3.8 (2.7 to 5.5)	89.7 (72.3 to 110.1)	21.4 (16.1 to 27.8)	52.3 (36.5 to 71.2)	5.6 (2.6 to 11.0)	10.3 (7.3 to 15.1)
Chad	2041	32.7 (26.2 to 40.2)	7.8 (5.9 to 10.2)	19.0 (13.2 to 25.9)	2.1 (0.9 to 4.0)	3.8 (2.6 to 5.6)	89.9 (72.2 to 110.6)	21.6 (16.2 to 27.9)	52.2 (36.3 to 71.1)	5.7 (2.6 to 11.1)	10.4 (7.3 to 15.4)
Chad	2042	32.8 (26.2 to 40.2)	7.9 (5.9 to 10.2)	19.0 (13.2 to 25.9)	2.1 (0.9 to 4.1)	3.8 (2.6 to 5.7)	90.2 (72.2 to 110.7)	21.7 (16.3 to 28.2)	52.2 (36.3 to 71.2)	5.7 (2.6 to 11.2)	10.6 (7.2 to 15.8)
Chad	2043	32.9 (26.4 to 40.3)	7.9 (6.0 to 10.3)	19.0 (13.2 to 25.9)	2.1 (1.0 to 4.1)	3.9 (2.6 to 6.0)	90.5 (72.7 to 110.9)	21.9 (16.4 to 28.4)	52.2 (36.3 to 71.3)	5.8 (2.6 to 11.3)	10.7 (7.2 to 16.5)

		2018 US Dollars per capita					2018 PPP per capita				
Country	Year	Health spending	Government health spending	Out-of-pocket health spending	Prepaid private health spending	Development assistance for health	Health spending	Government health spending	Out-of-pocket health spending	Prepaid private health spending	Development assistance for health
Chad	2044	33.0 (26.6 to 40.8)	8.0 (6.0 to 10.4)	19.0 (13.2 to 25.9)	2.1 (1.0 to 4.1)	3.9 (2.6 to 6.2)	90.9 (73.2 to 112.2)	22.0 (16.4 to 28.6)	52.2 (36.4 to 71.3)	5.8 (2.6 to 11.4)	10.8 (7.1 to 17.0)
Chad	2045	33.2 (26.6 to 41.1)	8.1 (6.0 to 10.5)	19.0 (13.2 to 26.0)	2.1 (1.0 to 4.2)	4.0 (2.6 to 6.4)	91.4 (73.3 to 113.0)	22.3 (16.6 to 29.0)	52.3 (36.4 to 71.4)	5.9 (2.7 to 11.5)	10.9 (7.1 to 17.6)
Chad	2046	33.5 (27.0 to 41.4)	8.2 (6.1 to 10.7)	19.1 (13.3 to 26.0)	2.2 (1.0 to 4.2)	4.0 (2.6 to 6.6)	92.1 (74.2 to 113.9)	22.7 (16.9 to 29.5)	52.4 (36.5 to 71.6)	6.0 (2.7 to 11.7)	11.1 (7.1 to 18.1)
Chad	2047	33.8 (27.2 to 41.9)	8.4 (6.2 to 11.0)	19.1 (13.3 to 26.1)	2.2 (1.0 to 4.3)	4.1 (2.6 to 6.6)	93.0 (74.8 to 115.4)	23.1 (17.2 to 30.3)	52.7 (36.7 to 71.9)	6.0 (2.7 to 11.8)	11.2 (7.2 to 18.1)
Chad	2048	34.2 (27.3 to 42.4)	8.6 (6.4 to 11.3)	19.2 (13.4 to 26.2)	2.2 (1.0 to 4.4)	4.1 (2.6 to 6.9)	94.1 (75.0 to 116.7)	23.6 (17.5 to 31.1)	52.9 (36.9 to 72.2)	6.1 (2.8 to 12.0)	11.4 (7.1 to 19.0)
Chad	2049	34.6 (27.7 to 42.9)	8.8 (6.5 to 11.6)	19.4 (13.5 to 26.4)	2.3 (1.0 to 4.4)	4.2 (2.6 to 7.2)	95.3 (76.2 to 118.1)	24.3 (17.9 to 31.9)	53.2 (37.1 to 72.5)	6.2 (2.8 to 12.2)	11.5 (7.1 to 19.7)
Chad	2050	35.1 (28.2 to 43.6)	9.1 (6.7 to 12.0)	19.5 (13.5 to 26.5)	2.3 (1.0 to 4.5)	4.3 (2.6 to 7.5)	96.7 (77.6 to 120.0)	25.0 (18.5 to 33.0)	53.6 (37.3 to 72.9)	6.3 (2.8 to 12.4)	11.8 (7.2 to 20.5)
Chile	1995	488.7 (453.2 to 526.0)	267.8 (243.8 to 293.4)	194.6 (171.1 to 220.5)	24.2 (14.9 to 37.7)	2.1 (2.1 to 2.1)	864.1 (801.3 to 930.1)	473.5 (431.1 to 518.9)	344.2 (302.6 to 389.8)	42.9 (26.3 to 66.7)	3.6 (3.6 to 3.6)
Chile	1996	544.7 (511.4 to 579.3)	291.5 (270.9 to 313.7)	223.4 (200.4 to 249.3)	27.9 (17.8 to 41.3)	2.0 (2.0 to 2.0)	963.2 (904.2 to 1024.3)	515.4 (479.0 to 554.6)	395.0 (354.3 to 440.8)	49.3 (31.6 to 73.0)	3.5 (3.5 to 3.5)
Chile	1997	607.6 (574.6 to 642.7)	317.5 (298.2 to 338.5)	255.5 (232.3 to 279.8)	30.9 (20.2 to 44.3)	3.7 (3.7 to 3.7)	1074.5 (1016.1 to 1136.5)	561.4 (527.4 to 598.5)	451.8 (410.8 to 494.7)	54.6 (35.7 to 78.3)	6.6 (6.6 to 6.6)
Chile	1998	654.3 (620.2 to 691.2)	340.9 (321.3 to 361.3)	279.1 (255.8 to 303.1)	33.9 (22.7 to 47.6)	0.3 (0.3 to 0.3)	1156.9 (1096.7 to 1222.3)	602.9 (568.1 to 638.8)	493.5 (452.4 to 535.9)	60.0 (40.2 to 84.2)	0.6 (0.6 to 0.6)
Chile	1999	663.9 (631.1 to 697.6)	346.7 (326.7 to 366.2)	283.9 (260.3 to 306.8)	33.1 (22.1 to 46.3)	0.2 (0.2 to 0.2)	1174.0 (1115.9 to 1233.5)	613.1 (577.8 to 647.6)	501.9 (460.3 to 542.5)	58.6 (39.0 to 81.9)	0.4 (0.4 to 0.4)
Chile	2000	673.4 (640.0 to 708.0)	355.5 (335.4 to 375.9)	286.5 (263.7 to 309.8)	31.2 (21.1 to 43.6)	0.2 (0.2 to 0.2)	1190.7 (1131.7 to 1251.9)	628.6 (593.0 to 664.7)	506.6 (466.4 to 547.7)	55.1 (37.3 to 77.2)	0.4 (0.4 to 0.4)
Chile	2001	682.5 (651.3 to 717.8)	359.3 (338.9 to 380.2)	290.8 (267.8 to 314.3)	32.1 (21.9 to 44.1)	0.3 (0.3 to 0.3)	1206.8 (1151.6 to 1269.3)	635.3 (599.3 to 672.2)	514.2 (473.6 to 555.8)	56.8 (38.8 to 78.0)	0.5 (0.5 to 0.5)
Chile	2002	691.6 (657.9 to 726.3)	362.6 (341.4 to 384.5)	295.4 (272.8 to 319.7)	33.3 (23.2 to 45.5)	0.2 (0.2 to 0.2)	1222.9 (1163.3 to 1284.2)	641.3 (603.7 to 679.8)	522.4 (482.3 to 565.3)	58.9 (41.0 to 80.5)	0.4 (0.4 to 0.4)
Chile	2003	700.2 (668.2 to 733.8)	365.7 (345.0 to 387.1)	298.9 (276.5 to 323.3)	34.9 (24.6 to 47.9)	0.7 (0.7 to 0.7)	1238.1 (1181.6 to 1297.5)	646.7 (610.0 to 684.4)	528.6 (489.0 to 571.7)	61.6 (43.6 to 84.8)	1.2 (1.2 to 1.2)
Chile	2004	710.9 (679.0 to 743.3)	373.6 (351.8 to 395.4)	299.3 (277.5 to 323.4)	36.1 (25.5 to 49.4)	1.9 (1.9 to 1.9)	1257.0 (1200.6 to 1314.3)	660.6 (622.0 to 699.1)	529.3 (490.7 to 571.9)	63.8 (45.1 to 87.4)	3.4 (3.4 to 3.4)
Chile	2005	708.1 (677.4 to 741.7)	375.9 (356.0 to 397.3)	295.0 (273.5 to 318.1)	36.1 (26.0 to 49.9)	1.1 (1.1 to 1.1)	1252.0 (1197.9 to 1311.5)	664.6 (629.4 to 702.5)	521.7 (483.5 to 562.5)	63.8 (45.9 to 88.2)	1.9 (1.9 to 1.9)
Chile	2006	721.6 (689.3 to 757.9)	391.6 (370.9 to 413.5)	290.8 (269.8 to 314.0)	38.7 (27.0 to 53.0)	0.4 (0.4 to 0.4)	1276.0 (1218.9 to 1340.1)	692.5 (655.9 to 731.1)	514.3 (477.2 to 555.3)	68.4 (47.8 to 93.8)	0.8 (0.8 to 0.8)
Chile	2007	756.4 (723.7 to 790.5)	418.8 (396.8 to 441.7)	293.2 (272.0 to 317.9)	43.8 (30.8 to 59.6)	0.5 (0.5 to 0.5)	1337.4 (1279.7 to 1397.8)	740.6 (701.6 to 781.1)	518.4 (480.9 to 562.2)	77.5 (54.4 to 105.4)	0.9 (0.9 to 0.9)
Chile	2008	799.6 (764.7 to 837.1)	451.8 (428.2 to 476.5)	297.6 (275.0 to 321.1)	49.9 (35.7 to 66.9)	0.3 (0.3 to 0.3)	1413.9 (1352.2 to 1480.3)	798.8 (757.1 to 842.5)	526.2 (486.3 to 567.9)	88.3 (63.1 to 118.4)	0.6 (0.6 to 0.6)

Country	Year	2018 US Dollars per capita					2018 PPP per capita				
		Health spending	Government health spending	Out-of-pocket health spending	Prepaid private health spending	Development assistance for health	Health spending	Government health spending	Out-of-pocket health spending	Prepaid private health spending	Development assistance for health
Chile	2009	819.7 (785.6 to 857.8)	472.0 (447.9 to 498.2)	293.2 (271.4 to 316.4)	54.2 (39.2 to 72.8)	0.3 (0.3 to 0.3)	1449.4 (1389.2 to 1516.7)	834.7 (792.0 to 880.9)	518.4 (480.0 to 559.5)	95.9 (69.3 to 128.7)	0.5 (0.5 to 0.5)
Chile	2010	862.1 (826.3 to 901.9)	507.8 (483.1 to 535.4)	297.2 (275.8 to 320.4)	56.9 (41.7 to 74.7)	0.3 (0.3 to 0.3)	1524.4 (1461.2 to 1594.9)	897.9 (854.2 to 946.7)	525.5 (487.6 to 566.6)	100.5 (73.8 to 132.0)	0.5 (0.5 to 0.5)
Chile	2011	908.8 (873.3 to 948.3)	541.5 (518.1 to 566.6)	307.8 (285.9 to 331.0)	59.4 (44.9 to 77.5)	0.2 (0.2 to 0.2)	1607.0 (1544.2 to 1676.8)	957.5 (916.1 to 1001.9)	544.2 (505.6 to 585.3)	105.0 (79.4 to 137.1)	0.3 (0.3 to 0.3)
Chile	2012	966.9 (932.0 to 1001.1)	577.4 (554.0 to 600.7)	324.8 (303.8 to 347.3)	64.6 (50.1 to 82.1)	0.0 (0.0 to 0.0)	1709.7 (1648.1 to 1770.3)	1021.0 (979.6 to 1062.2)	574.4 (537.2 to 614.1)	114.2 (88.5 to 145.2)	0.0 (0.0 to 0.0)
Chile	2013	1039.3 (1005.4 to 1073.6)	617.5 (595.1 to 640.7)	350.9 (330.9 to 373.2)	70.9 (55.7 to 89.0)	0.0 (0.0 to 0.0)	1837.8 (1777.9 to 1898.3)	1091.9 (1052.2 to 1133.0)	620.4 (585.1 to 659.9)	125.4 (98.5 to 157.3)	0.1 (0.1 to 0.1)
Chile	2014	1106.5 (1070.0 to 1143.3)	652.7 (629.5 to 678.0)	378.3 (357.7 to 399.2)	75.5 (60.2 to 94.5)	0.0 (0.0 to 0.0)	1956.5 (1892.0 to 2021.6)	1154.1 (1113.0 to 1198.9)	668.9 (632.5 to 705.8)	133.5 (106.5 to 167.1)	0.1 (0.1 to 0.1)
Chile	2015	1184.8 (1147.5 to 1228.2)	695.0 (670.8 to 720.7)	408.7 (385.7 to 434.2)	81.1 (64.0 to 103.7)	0.0 (0.0 to 0.0)	2095.1 (2029.0 to 2171.8)	1229.0 (1186.2 to 1274.4)	722.8 (682.0 to 767.8)	143.3 (113.2 to 183.3)	0.0 (0.0 to 0.0)
Chile	2016	1243.7 (1192.8 to 1293.9)	726.9 (694.0 to 764.8)	431.3 (399.3 to 465.9)	85.5 (64.3 to 113.3)	0.0 (0.0 to 0.0)	2199.2 (2109.2 to 2287.9)	1285.3 (1227.1 to 1352.3)	762.6 (706.1 to 823.8)	151.2 (113.7 to 200.3)	0.0 (0.0 to 0.0)
Chile	2017	1262.9 (1210.9 to 1318.2)	738.7 (705.4 to 776.8)	437.9 (404.1 to 473.0)	86.2 (64.8 to 114.1)	0.0 (0.0 to 0.0)	2233.1 (2141.2 to 2331.0)	1306.3 (1247.4 to 1373.6)	774.4 (714.6 to 836.4)	152.4 (114.6 to 201.8)	0.0 (0.0 to 0.0)
Chile	2018	1282.6 (1227.8 to 1340.0)	750.1 (716.1 to 790.5)	444.7 (411.1 to 482.2)	87.7 (66.0 to 116.3)	0.0 (0.0 to 0.0)	2267.9 (2171.1 to 2369.5)	1326.5 (1266.2 to 1397.8)	786.4 (727.0 to 852.7)	155.1 (116.8 to 205.6)	0.0 (0.0 to 0.0)
Chile	2019	1301.8 (1245.7 to 1363.1)	761.3 (727.3 to 802.4)	451.4 (416.2 to 490.3)	89.1 (67.2 to 118.2)	0.0 (0.0 to 0.0)	2301.9 (2202.8 to 2410.3)	1346.1 (1286.0 to 1418.8)	798.2 (735.9 to 866.9)	157.6 (118.8 to 209.0)	0.0 (0.0 to 0.0)
Chile	2020	1321.8 (1264.3 to 1381.2)	772.1 (737.6 to 813.9)	459.2 (422.0 to 499.8)	90.6 (68.0 to 120.1)	0.0 (0.0 to 0.0)	2337.3 (2235.5 to 2442.3)	1365.2 (1304.2 to 1439.1)	812.0 (746.1 to 883.8)	160.2 (120.3 to 212.4)	0.0 (0.0 to 0.0)
Chile	2021	1341.2 (1280.8 to 1408.6)	781.9 (746.3 to 823.9)	467.3 (427.1 to 510.3)	92.0 (69.0 to 122.0)	0.0 (0.0 to 0.0)	2371.7 (2264.8 to 2490.7)	1382.7 (1319.6 to 1456.9)	826.4 (755.2 to 902.4)	162.6 (122.0 to 215.7)	0.0 (0.0 to 0.0)
Chile	2022	1361.2 (1297.8 to 1427.2)	792.4 (755.5 to 836.3)	475.4 (432.9 to 520.2)	93.4 (70.2 to 123.7)	0.0 (0.0 to 0.0)	2406.9 (2294.8 to 2523.7)	1401.1 (1335.9 to 1478.8)	840.7 (765.6 to 919.8)	165.1 (124.0 to 218.7)	0.0 (0.0 to 0.0)
Chile	2023	1388.9 (1325.6 to 1459.4)	810.7 (773.4 to 857.0)	483.0 (440.5 to 528.2)	95.1 (71.5 to 126.1)	0.0 (0.0 to 0.0)	2456.0 (2344.0 to 2580.5)	1433.6 (1367.6 to 1515.4)	854.1 (778.9 to 934.0)	168.2 (126.3 to 223.0)	0.0 (0.0 to 0.0)
Chile	2024	1413.5 (1346.6 to 1479.2)	829.0 (788.3 to 877.9)	487.6 (444.2 to 535.2)	96.9 (72.7 to 128.5)	0.0 (0.0 to 0.0)	2499.5 (2381.2 to 2615.6)	1465.9 (1394.0 to 1552.4)	862.2 (785.5 to 946.3)	171.3 (128.6 to 227.2)	0.0 (0.0 to 0.0)
Chile	2025	1438.4 (1371.7 to 1510.1)	847.7 (805.3 to 897.0)	492.0 (447.6 to 539.1)	98.7 (74.0 to 130.7)	0.0 (0.0 to 0.0)	2543.4 (2425.6 to 2670.2)	1498.9 (1424.0 to 1586.1)	870.1 (791.4 to 953.3)	174.5 (130.9 to 231.2)	0.0 (0.0 to 0.0)
Chile	2026	1463.5 (1392.5 to 1535.6)	866.4 (821.4 to 918.7)	496.6 (451.4 to 544.1)	100.5 (75.4 to 133.0)	0.0 (0.0 to 0.0)	2587.8 (2462.3 to 2715.3)	1532.0 (1452.5 to 1624.5)	878.1 (798.2 to 962.2)	177.6 (133.3 to 235.2)	0.0 (0.0 to 0.0)
Chile	2027	1489.0 (1418.2 to 1572.2)	885.5 (838.7 to 942.1)	501.2 (456.0 to 550.5)	102.3 (76.7 to 135.3)	0.0 (0.0 to 0.0)	2632.9 (2507.7 to 2780.0)	1565.8 (1483.1 to 1665.9)	886.2 (806.3 to 973.5)	180.9 (135.7 to 239.3)	0.0 (0.0 to 0.0)
Chile	2028	1513.9 (1436.8 to 1588.9)	904.4 (854.3 to 961.7)	505.5 (458.8 to 556.1)	104.1 (78.0 to 137.6)	0.0 (0.0 to 0.0)	2677.0 (2540.6 to 2809.6)	1599.1 (1510.7 to 1700.6)	893.8 (811.2 to 983.3)	184.0 (137.8 to 243.4)	0.0 (0.0 to 0.0)
Chile	2029	1539.7 (1458.8 to 1627.5)	923.9 (871.4 to 986.0)	509.9 (461.4 to 561.5)	105.9 (79.2 to 140.1)	0.0 (0.0 to 0.0)	2722.6 (2579.6 to 2877.8)	1633.6 (1540.9 to 1743.5)	901.7 (815.9 to 992.9)	187.3 (140.1 to 247.7)	0.0 (0.0 to 0.0)

Country	Year	2018 US Dollars per capita					2018 PPP per capita				
		Health spending	Government health spending	Out-of-pocket health spending	Prepaid private health spending	Development assistance for health	Health spending	Government health spending	Out-of-pocket health spending	Prepaid private health spending	Development assistance for health
Chile	2030	1565.1 (1483.5 to 1646.6)	943.2 (887.1 to 1007.6)	514.1 (463.6 to 566.4)	107.8 (80.5 to 142.5)	0.0 (0.0 to 0.0)	2767.6 (2623.2 to 2911.6)	1667.9 (1568.7 to 1781.7)	909.1 (819.7 to 1001.5)	190.5 (142.4 to 252.0)	0.0 (0.0 to 0.0)
Chile	2031	1590.1 (1503.9 to 1682.0)	962.3 (901.6 to 1030.9)	518.2 (465.6 to 573.4)	109.6 (81.8 to 145.0)	0.0 (0.0 to 0.0)	2811.8 (2659.3 to 2974.3)	1701.7 (1594.3 to 1822.8)	916.4 (823.3 to 1014.0)	193.8 (144.6 to 256.3)	0.0 (0.0 to 0.0)
Chile	2032	1614.8 (1524.5 to 1702.7)	981.3 (916.6 to 1052.0)	522.1 (468.2 to 580.2)	111.4 (83.0 to 147.4)	0.0 (0.0 to 0.0)	2855.4 (2695.7 to 3010.7)	1735.2 (1620.7 to 1860.3)	923.3 (827.9 to 1025.9)	196.9 (146.8 to 260.6)	0.0 (0.0 to 0.0)
Chile	2033	1639.7 (1541.9 to 1743.5)	1000.5 (930.8 to 1075.2)	526.0 (469.9 to 586.4)	113.2 (84.3 to 149.6)	0.0 (0.0 to 0.0)	2899.4 (2726.5 to 3083.0)	1769.1 (1645.8 to 1901.3)	930.1 (830.8 to 1036.8)	200.2 (149.0 to 264.6)	0.0 (0.0 to 0.0)
Chile	2034	1664.3 (1569.1 to 1765.0)	1019.5 (945.3 to 1098.2)	529.8 (471.6 to 591.0)	115.0 (85.5 to 152.2)	0.0 (0.0 to 0.0)	2942.8 (2774.5 to 3121.0)	1802.7 (1671.6 to 1941.9)	936.8 (833.8 to 1045.0)	203.4 (151.2 to 269.1)	0.0 (0.0 to 0.0)
Chile	2035	1690.2 (1586.0 to 1798.7)	1039.3 (957.7 to 1122.0)	534.0 (473.7 to 596.8)	116.9 (86.9 to 155.0)	0.0 (0.0 to 0.0)	2988.7 (2804.5 to 3180.5)	1837.7 (1693.5 to 1984.0)	944.2 (837.6 to 1055.4)	206.8 (153.7 to 274.1)	0.0 (0.0 to 0.0)
Chile	2036	1715.1 (1614.8 to 1825.5)	1058.3 (972.5 to 1146.9)	538.0 (475.8 to 603.4)	118.8 (88.2 to 157.8)	0.0 (0.0 to 0.0)	3032.7 (2855.3 to 3228.0)	1871.3 (1719.6 to 2028.0)	951.3 (841.4 to 1066.9)	210.1 (156.0 to 279.0)	0.0 (0.0 to 0.0)
Chile	2037	1740.7 (1623.7 to 1860.6)	1077.6 (988.2 to 1171.2)	542.2 (477.7 to 609.2)	120.8 (89.5 to 160.7)	0.0 (0.0 to 0.0)	3078.0 (2871.1 to 3290.0)	1905.5 (1747.4 to 2071.0)	958.8 (844.8 to 1077.3)	213.6 (158.3 to 284.1)	0.0 (0.0 to 0.0)
Chile	2038	1766.3 (1652.8 to 1887.2)	1097.0 (1001.8 to 1195.7)	546.5 (480.1 to 613.8)	122.8 (90.8 to 163.6)	0.0 (0.0 to 0.0)	3123.4 (2922.5 to 3337.0)	1939.8 (1771.5 to 2114.3)	966.4 (849.0 to 1085.4)	217.2 (160.5 to 289.3)	0.0 (0.0 to 0.0)
Chile	2039	1793.2 (1670.5 to 1927.3)	1117.2 (1017.3 to 1221.5)	551.1 (482.7 to 619.6)	124.9 (92.2 to 166.7)	0.0 (0.0 to 0.0)	3170.8 (2953.9 to 3408.0)	1975.5 (1798.8 to 2160.0)	974.4 (853.6 to 1095.6)	220.9 (163.0 to 294.7)	0.0 (0.0 to 0.0)
Chile	2040	1820.1 (1700.8 to 1951.7)	1137.4 (1033.9 to 1246.8)	555.6 (485.5 to 625.3)	127.1 (93.5 to 169.8)	0.0 (0.0 to 0.0)	3218.4 (3007.5 to 3451.1)	2011.3 (1828.2 to 2204.6)	982.5 (858.4 to 1105.6)	224.7 (165.3 to 300.2)	0.0 (0.0 to 0.0)
Chile	2041	1847.4 (1716.0 to 1985.4)	1157.7 (1050.5 to 1272.3)	560.4 (489.2 to 631.1)	129.3 (94.9 to 173.0)	0.0 (0.0 to 0.0)	3266.7 (3034.4 to 3510.7)	2047.1 (1857.6 to 2249.8)	991.0 (865.0 to 1115.9)	228.6 (167.9 to 305.8)	0.0 (0.0 to 0.0)
Chile	2042	1874.0 (1745.8 to 2017.0)	1177.5 (1065.5 to 1299.5)	565.0 (493.4 to 636.7)	131.4 (96.3 to 176.2)	0.0 (0.0 to 0.0)	3313.8 (3087.0 to 3566.7)	2082.2 (1884.1 to 2297.8)	999.2 (872.4 to 1125.9)	232.4 (170.4 to 311.5)	0.0 (0.0 to 0.0)
Chile	2043	1902.1 (1769.1 to 2058.8)	1198.4 (1082.3 to 1326.7)	570.0 (497.1 to 642.8)	133.7 (97.8 to 179.2)	0.0 (0.0 to 0.0)	3363.4 (3128.1 to 3640.4)	2119.0 (1913.9 to 2346.0)	1007.9 (879.0 to 1136.7)	236.5 (173.0 to 316.9)	0.0 (0.0 to 0.0)
Chile	2044	1931.1 (1783.7 to 2089.1)	1220.0 (1098.5 to 1353.4)	575.1 (501.0 to 649.1)	136.1 (99.3 to 182.1)	0.0 (0.0 to 0.0)	3414.7 (3154.1 to 3694.1)	2157.2 (1942.4 to 2393.2)	1016.9 (885.9 to 1147.8)	240.7 (175.6 to 322.0)	0.0 (0.0 to 0.0)
Chile	2045	1959.7 (1815.6 to 2126.8)	1241.4 (1115.5 to 1383.8)	579.8 (505.4 to 655.5)	138.4 (100.8 to 185.0)	0.0 (0.0 to 0.0)	3465.2 (3210.5 to 3760.8)	2195.1 (1972.5 to 2446.9)	1025.3 (893.7 to 1159.1)	244.8 (178.3 to 327.1)	0.0 (0.0 to 0.0)
Chile	2046	1987.6 (1828.8 to 2171.5)	1262.3 (1129.6 to 1408.2)	584.6 (508.7 to 660.9)	140.7 (102.4 to 188.1)	0.0 (0.0 to 0.0)	3514.5 (3233.8 to 3839.7)	2232.0 (1997.4 to 2490.1)	1033.7 (899.6 to 1168.7)	248.9 (181.0 to 332.6)	0.0 (0.0 to 0.0)
Chile	2047	2015.8 (1866.2 to 2187.9)	1283.5 (1146.5 to 1434.7)	589.2 (512.7 to 666.1)	143.1 (104.0 to 191.7)	0.0 (0.0 to 0.0)	3564.5 (3300.0 to 3868.9)	2269.5 (2027.4 to 2536.9)	1041.9 (906.6 to 1177.9)	253.0 (184.0 to 338.9)	0.0 (0.0 to 0.0)
Chile	2048	2044.3 (1875.5 to 2241.0)	1304.9 (1163.0 to 1460.3)	593.9 (516.6 to 671.7)	145.5 (105.7 to 194.8)	0.0 (0.0 to 0.0)	3614.8 (3316.3 to 3962.7)	2307.5 (2056.4 to 2582.1)	1050.1 (913.5 to 1187.8)	257.2 (186.9 to 344.5)	0.0 (0.0 to 0.0)
Chile	2049	2072.3 (1909.6 to 2263.9)	1326.3 (1178.3 to 1490.6)	598.2 (520.2 to 677.1)	147.8 (107.4 to 198.4)	0.0 (0.0 to 0.0)	3664.4 (3376.6 to 4003.1)	2345.3 (2083.6 to 2635.8)	1057.8 (919.9 to 1197.3)	261.4 (189.8 to 350.9)	0.0 (0.0 to 0.0)
Chile	2050	2099.6 (1919.5 to 2303.4)	1347.5 (1192.4 to 1517.5)	602.1 (523.3 to 681.4)	150.1 (108.7 to 202.3)	0.0 (0.0 to 0.0)	3712.7 (3394.1 to 4073.0)	2382.7 (2108.4 to 2683.4)	1064.6 (925.3 to 1205.0)	265.3 (192.2 to 357.7)	0.0 (0.0 to 0.0)

Country	Year	2018 US Dollars per capita					2018 PPP per capita				
		Health spending	Government health spending	Out-of-pocket health spending	Prepaid private health spending	Development assistance for health	Health spending	Government health spending	Out-of-pocket health spending	Prepaid private health spending	Development assistance for health
China	1995	56.4 (46.4 to 68.2)	15.9 (12.8 to 19.4)	28.5 (21.5 to 36.1)	12.0 (6.3 to 20.7)	0.1 (0.1 to 0.1)	104.5 (85.9 to 126.2)	29.4 (23.6 to 36.0)	52.7 (39.9 to 66.9)	22.2 (11.7 to 38.4)	0.2 (0.2 to 0.2)
China	1996	63.0 (52.7 to 75.9)	17.3 (13.9 to 21.0)	33.0 (25.8 to 41.5)	12.6 (6.7 to 21.9)	0.1 (0.1 to 0.1)	116.7 (97.6 to 140.6)	32.0 (25.7 to 38.9)	61.0 (47.7 to 76.8)	23.4 (12.5 to 40.6)	0.2 (0.2 to 0.2)
China	1997	71.3 (59.8 to 85.5)	19.1 (15.4 to 23.0)	38.6 (30.8 to 48.3)	13.5 (7.2 to 23.1)	0.1 (0.1 to 0.1)	132.1 (110.8 to 158.3)	35.3 (28.6 to 42.6)	71.5 (57.0 to 89.4)	24.9 (13.3 to 42.8)	0.3 (0.3 to 0.3)
China	1998	79.1 (67.1 to 94.3)	20.5 (16.8 to 24.6)	44.6 (35.7 to 54.8)	13.9 (7.5 to 23.8)	0.2 (0.2 to 0.2)	146.6 (124.2 to 174.7)	37.9 (31.2 to 45.6)	82.5 (66.2 to 101.5)	25.8 (13.9 to 44.1)	0.3 (0.3 to 0.3)
China	1999	87.4 (74.4 to 104.4)	22.1 (18.4 to 26.3)	50.9 (41.4 to 62.9)	14.4 (8.0 to 24.9)	0.1 (0.1 to 0.1)	161.9 (137.9 to 193.3)	40.9 (34.0 to 48.7)	94.2 (76.6 to 116.5)	26.6 (14.7 to 46.2)	0.3 (0.3 to 0.3)
China	2000	95.3 (82.0 to 112.0)	23.2 (19.2 to 27.5)	57.2 (47.3 to 69.6)	14.6 (8.0 to 25.8)	0.2 (0.2 to 0.2)	176.4 (151.9 to 207.3)	43.0 (35.6 to 50.9)	106.0 (87.7 to 129.0)	27.1 (14.9 to 47.7)	0.3 (0.3 to 0.3)
China	2001	101.7 (87.9 to 119.3)	25.3 (21.0 to 30.0)	62.7 (52.4 to 76.2)	13.6 (7.3 to 24.4)	0.1 (0.1 to 0.1)	188.4 (162.8 to 220.9)	46.8 (39.0 to 55.5)	116.2 (97.0 to 141.1)	25.2 (13.4 to 45.3)	0.2 (0.2 to 0.2)
China	2002	111.7 (97.4 to 130.1)	29.2 (24.5 to 34.8)	68.7 (57.8 to 82.2)	13.6 (7.2 to 23.6)	0.1 (0.1 to 0.1)	206.8 (180.4 to 240.9)	54.1 (45.4 to 64.4)	127.3 (107.0 to 152.2)	25.2 (13.4 to 43.7)	0.2 (0.2 to 0.2)
China	2003	121.9 (106.7 to 140.5)	34.1 (28.9 to 40.4)	73.7 (61.9 to 86.6)	13.8 (7.3 to 23.5)	0.1 (0.1 to 0.1)	225.6 (197.6 to 260.1)	63.2 (53.5 to 74.8)	136.5 (114.6 to 160.4)	25.6 (13.6 to 43.5)	0.2 (0.2 to 0.2)
China	2004	131.6 (116.0 to 149.4)	39.8 (33.7 to 46.8)	77.5 (65.6 to 90.4)	14.1 (7.5 to 23.6)	0.2 (0.2 to 0.2)	243.7 (214.7 to 276.7)	73.7 (62.3 to 86.6)	143.6 (121.5 to 167.4)	26.1 (13.9 to 43.8)	0.4 (0.4 to 0.4)
China	2005	141.9 (126.7 to 160.3)	46.8 (39.7 to 54.5)	81.0 (68.2 to 95.2)	14.0 (7.8 to 23.3)	0.2 (0.2 to 0.2)	262.8 (234.6 to 296.9)	86.6 (73.5 to 101.0)	149.9 (126.2 to 176.2)	26.0 (14.4 to 43.2)	0.3 (0.3 to 0.3)
China	2006	153.3 (136.9 to 172.4)	55.4 (47.6 to 63.9)	83.5 (69.9 to 97.7)	14.2 (7.8 to 23.3)	0.2 (0.2 to 0.2)	283.9 (253.6 to 319.2)	102.6 (88.2 to 118.4)	154.6 (129.4 to 181.0)	26.3 (14.4 to 43.1)	0.4 (0.4 to 0.4)
China	2007	168.9 (150.7 to 189.2)	67.5 (58.9 to 77.5)	86.0 (72.3 to 101.5)	15.0 (8.2 to 24.6)	0.3 (0.3 to 0.3)	312.7 (279.1 to 350.3)	125.0 (109.1 to 143.5)	159.3 (133.9 to 188.0)	27.8 (15.2 to 45.6)	0.6 (0.6 to 0.6)
China	2008	187.7 (168.3 to 208.2)	82.4 (72.6 to 94.1)	89.6 (75.9 to 105.0)	15.5 (8.3 to 25.6)	0.2 (0.2 to 0.2)	347.6 (311.6 to 385.6)	152.6 (134.5 to 174.2)	165.9 (140.6 to 194.5)	28.6 (15.4 to 47.5)	0.4 (0.4 to 0.4)
China	2009	214.4 (193.5 to 236.4)	102.2 (90.6 to 115.7)	95.6 (81.7 to 111.9)	16.3 (8.8 to 27.2)	0.3 (0.3 to 0.3)	397.0 (358.3 to 437.7)	189.2 (167.7 to 214.2)	177.0 (151.3 to 207.2)	30.3 (16.2 to 50.4)	0.5 (0.5 to 0.5)
China	2010	240.6 (219.5 to 265.5)	122.5 (109.3 to 138.0)	100.9 (86.8 to 117.6)	16.9 (9.3 to 28.4)	0.2 (0.2 to 0.2)	445.5 (406.5 to 491.7)	226.8 (202.4 to 255.5)	186.8 (160.7 to 217.8)	31.4 (17.2 to 52.7)	0.4 (0.4 to 0.4)
China	2011	269.6 (246.8 to 297.7)	144.0 (129.5 to 161.5)	108.4 (93.6 to 125.8)	17.0 (9.1 to 28.7)	0.2 (0.2 to 0.2)	499.3 (457.0 to 551.4)	266.7 (239.8 to 299.1)	200.8 (173.4 to 233.0)	31.4 (16.9 to 53.2)	0.4 (0.4 to 0.4)
China	2012	301.2 (274.8 to 331.7)	166.7 (150.9 to 186.5)	117.1 (100.9 to 136.5)	17.1 (9.2 to 28.4)	0.3 (0.3 to 0.3)	557.7 (508.8 to 614.2)	308.7 (279.4 to 345.4)	216.9 (186.9 to 252.8)	31.7 (17.0 to 52.6)	0.5 (0.5 to 0.5)
China	2013	333.7 (304.8 to 366.6)	189.7 (172.0 to 211.5)	125.9 (107.8 to 147.0)	17.9 (9.6 to 29.4)	0.1 (0.1 to 0.1)	618.0 (564.5 to 678.9)	351.4 (318.5 to 391.7)	233.1 (199.6 to 272.3)	33.2 (17.8 to 54.4)	0.3 (0.3 to 0.3)
China	2014	366.5 (333.5 to 402.6)	212.8 (192.7 to 236.3)	134.0 (113.3 to 155.4)	19.6 (10.4 to 32.6)	0.1 (0.1 to 0.1)	678.7 (617.6 to 745.6)	394.1 (356.8 to 437.6)	248.1 (209.9 to 287.7)	36.3 (19.3 to 60.3)	0.1 (0.1 to 0.1)
China	2015	401.5 (364.5 to 440.3)	236.5 (212.6 to 263.5)	143.1 (119.7 to 168.2)	21.9 (11.5 to 37.7)	0.1 (0.1 to 0.1)	743.5 (674.9 to 815.3)	438.0 (393.7 to 488.0)	264.9 (221.6 to 311.4)	40.5 (21.4 to 69.9)	0.2 (0.2 to 0.2)

		2018 US Dollars per capita					2018 PPP per capita				
Country	Year	Health spending	Government health spending	Out-of-pocket health spending	Prepaid private health spending	Development assistance for health	Health spending	Government health spending	Out-of-pocket health spending	Prepaid private health spending	Development assistance for health
China	2016	436.1 (390.5 to 487.0)	256.5 (226.8 to 291.8)	153.8 (127.0 to 183.2)	25.7 (13.4 to 45.2)	0.2 (0.2 to 0.2)	807.6 (723.2 to 901.9)	475.0 (420.0 to 540.3)	284.8 (235.2 to 339.3)	47.5 (24.8 to 83.7)	0.3 (0.3 to 0.3)
China	2017	464.9 (418.3 to 517.8)	274.7 (242.9 to 312.2)	162.9 (134.2 to 194.8)	27.1 (14.2 to 47.8)	0.1 (0.1 to 0.1)	860.8 (774.5 to 958.9)	508.7 (449.8 to 578.0)	301.7 (248.5 to 360.7)	50.2 (26.2 to 88.4)	0.3 (0.3 to 0.3)
China	2018	482.3 (431.5 to 533.2)	282.9 (250.1 to 322.0)	170.9 (140.6 to 203.5)	28.4 (14.8 to 50.2)	0.1 (0.1 to 0.1)	893.2 (799.1 to 987.4)	523.8 (463.1 to 596.2)	316.6 (260.4 to 376.8)	52.6 (27.4 to 92.9)	0.3 (0.3 to 0.3)
China	2019	504.3 (452.6 to 562.4)	295.5 (261.1 to 336.6)	179.1 (148.1 to 213.4)	29.6 (15.4 to 52.5)	0.1 (0.1 to 0.1)	933.9 (838.2 to 1041.5)	547.1 (483.5 to 623.3)	331.6 (274.3 to 395.2)	54.9 (28.6 to 97.1)	0.3 (0.2 to 0.3)
China	2020	528.8 (477.3 to 587.9)	310.1 (273.3 to 353.3)	187.6 (154.1 to 223.8)	31.0 (16.1 to 54.9)	0.1 (0.1 to 0.2)	979.2 (883.9 to 1088.7)	574.2 (506.0 to 654.2)	347.4 (285.4 to 414.4)	57.4 (29.9 to 101.6)	0.3 (0.2 to 0.3)
China	2021	554.7 (494.0 to 615.3)	325.6 (287.2 to 370.7)	196.5 (162.0 to 235.3)	32.4 (16.9 to 57.4)	0.1 (0.1 to 0.2)	1027.1 (914.7 to 1139.4)	602.9 (531.8 to 686.4)	364.0 (300.0 to 435.7)	60.0 (31.3 to 106.3)	0.3 (0.2 to 0.3)
China	2022	582.6 (522.4 to 647.1)	342.7 (301.8 to 389.9)	205.9 (169.2 to 246.9)	33.9 (17.7 to 59.9)	0.1 (0.1 to 0.2)	1078.8 (967.4 to 1198.3)	634.5 (558.9 to 722.0)	381.2 (313.4 to 457.3)	62.7 (32.8 to 111.0)	0.3 (0.2 to 0.3)
China	2023	611.8 (548.4 to 682.3)	360.6 (317.5 to 410.5)	215.6 (176.7 to 258.9)	35.4 (18.5 to 62.5)	0.2 (0.1 to 0.2)	1132.9 (1015.5 to 1263.5)	667.8 (587.8 to 760.2)	399.2 (327.2 to 479.3)	65.6 (34.2 to 115.8)	0.3 (0.2 to 0.3)
China	2024	643.1 (577.6 to 717.4)	380.1 (333.5 to 431.9)	225.7 (185.6 to 271.8)	37.0 (19.3 to 65.2)	0.2 (0.1 to 0.2)	1190.8 (1069.6 to 1328.4)	703.9 (617.5 to 799.8)	418.0 (343.7 to 503.3)	68.6 (35.8 to 120.8)	0.3 (0.2 to 0.3)
China	2025	675.4 (605.5 to 750.9)	400.2 (351.1 to 454.3)	236.3 (193.8 to 284.4)	38.7 (20.2 to 68.0)	0.2 (0.1 to 0.2)	1250.7 (1121.3 to 1390.5)	741.1 (650.1 to 841.2)	437.6 (358.8 to 526.6)	71.6 (37.4 to 125.9)	0.3 (0.3 to 0.3)
China	2026	709.7 (635.5 to 789.3)	421.8 (369.7 to 479.6)	247.4 (202.8 to 299.0)	40.4 (21.1 to 71.0)	0.2 (0.1 to 0.2)	1314.2 (1176.7 to 1461.6)	781.0 (684.6 to 888.1)	458.1 (375.5 to 553.8)	74.8 (39.0 to 131.5)	0.3 (0.3 to 0.4)
China	2027	746.2 (665.1 to 834.0)	444.9 (389.0 to 507.1)	259.0 (211.2 to 313.6)	42.2 (22.0 to 74.0)	0.2 (0.1 to 0.2)	1381.9 (1231.7 to 1544.4)	823.9 (720.4 to 939.0)	479.6 (391.1 to 580.7)	78.1 (40.7 to 137.1)	0.3 (0.3 to 0.4)
China	2028	784.3 (693.1 to 886.0)	469.3 (408.2 to 536.8)	270.8 (219.6 to 330.5)	44.0 (22.9 to 77.0)	0.2 (0.1 to 0.2)	1452.4 (1283.6 to 1640.7)	869.1 (755.9 to 994.0)	501.5 (406.7 to 612.0)	81.5 (42.3 to 142.6)	0.3 (0.3 to 0.4)
China	2029	824.2 (730.7 to 927.3)	495.3 (429.8 to 568.0)	282.9 (227.9 to 346.7)	45.9 (23.8 to 80.1)	0.2 (0.1 to 0.2)	1526.3 (1353.1 to 1717.1)	917.1 (795.8 to 1051.8)	523.9 (422.0 to 642.0)	85.0 (44.0 to 148.3)	0.3 (0.3 to 0.4)
China	2030	865.4 (764.2 to 979.7)	522.1 (450.8 to 600.3)	295.2 (236.7 to 364.0)	47.9 (24.7 to 83.5)	0.2 (0.0 to 0.2)	1602.5 (1415.2 to 1814.3)	966.9 (834.8 to 1111.7)	546.7 (438.3 to 674.0)	88.6 (45.8 to 154.6)	0.3 (0.0 to 0.4)
China	2031	908.3 (802.7 to 1031.7)	550.6 (471.3 to 635.7)	307.7 (245.9 to 383.2)	49.9 (25.7 to 87.1)	0.2 (0.0 to 0.2)	1682.0 (1486.5 to 1910.5)	1019.5 (872.8 to 1177.1)	569.9 (455.4 to 709.7)	92.3 (47.6 to 161.4)	0.3 (0.0 to 0.4)
China	2032	953.3 (834.8 to 1081.3)	580.7 (494.2 to 673.3)	320.5 (253.9 to 400.7)	51.9 (26.7 to 90.9)	0.2 (0.0 to 0.2)	1765.3 (1545.9 to 2002.3)	1075.4 (915.1 to 1246.9)	593.4 (470.2 to 742.0)	96.2 (49.5 to 168.4)	0.3 (0.0 to 0.4)
China	2033	1000.7 (875.9 to 1142.1)	613.0 (517.8 to 714.3)	333.4 (262.5 to 417.7)	54.1 (27.8 to 94.9)	0.2 (0.0 to 0.2)	1853.0 (1621.9 to 2114.9)	1135.1 (958.8 to 1322.7)	617.5 (486.0 to 773.6)	100.2 (51.4 to 175.7)	0.3 (0.0 to 0.5)
China	2034	1049.3 (906.1 to 1200.0)	646.1 (545.3 to 758.2)	346.6 (271.6 to 436.3)	56.4 (28.9 to 98.8)	0.1 (0.0 to 0.3)	1943.0 (1677.8 to 2222.1)	1196.5 (1009.8 to 1404.0)	641.9 (502.9 to 807.9)	104.4 (53.6 to 183.0)	0.3 (0.0 to 0.5)
China	2035	1099.8 (948.4 to 1258.4)	681.0 (571.9 to 802.9)	359.9 (279.9 to 455.3)	58.7 (30.1 to 103.0)	0.1 (0.0 to 0.3)	2036.5 (1756.1 to 2330.2)	1261.0 (1059.0 to 1486.8)	666.5 (518.4 to 843.2)	108.8 (55.7 to 190.7)	0.2 (0.0 to 0.5)
China	2036	1151.5 (997.5 to 1318.8)	716.7 (599.5 to 848.4)	373.4 (289.1 to 474.6)	61.2 (31.3 to 107.3)	0.1 (0.0 to 0.3)	2132.3 (1847.1 to 2442.1)	1327.2 (1110.2 to 1571.1)	691.5 (535.3 to 878.8)	113.4 (57.9 to 198.7)	0.2 (0.0 to 0.5)

		2018 US Dollars per capita					2018 PPP per capita				
Country	Year	Health spending	Government health spending	Out-of-pocket health spending	Prepaid private health spending	Development assistance for health	Health spending	Government health spending	Out-of-pocket health spending	Prepaid private health spending	Development assistance for health
China	2037	1204.6 (1033.1 to 1397.1)	753.6 (627.3 to 896.6)	387.1 (296.5 to 493.9)	63.8 (32.5 to 111.8)	0.1 (0.0 to 0.3)	2230.7 (1913.1 to 2587.1)	1395.5 (1161.5 to 1660.3)	716.8 (549.1 to 914.5)	118.2 (60.2 to 207.1)	0.2 (0.0 to 0.5)
China	2038	1260.0 (1082.5 to 1453.7)	792.5 (657.3 to 947.5)	400.9 (306.1 to 513.6)	66.6 (33.8 to 116.6)	0.1 (0.0 to 0.3)	2333.3 (2004.6 to 2691.9)	1467.4 (1217.1 to 1754.5)	742.4 (566.8 to 951.1)	123.3 (62.6 to 215.9)	0.1 (0.0 to 0.5)
China	2039	1316.4 (1130.6 to 1519.5)	831.8 (688.2 to 999.1)	415.0 (316.1 to 533.6)	69.5 (35.2 to 121.7)	0.1 (0.0 to 0.3)	2437.6 (2093.7 to 2813.8)	1540.3 (1274.4 to 1850.1)	768.5 (585.4 to 988.0)	128.7 (65.3 to 225.3)	0.1 (0.0 to 0.5)
China	2040	1375.9 (1193.3 to 1595.2)	873.9 (720.4 to 1051.5)	429.3 (326.4 to 553.4)	72.6 (36.8 to 127.2)	0.1 (0.0 to 0.3)	2547.9 (2209.8 to 2953.9)	1618.2 (1334.0 to 1947.2)	795.0 (604.4 to 1024.8)	134.5 (68.1 to 235.6)	0.1 (0.0 to 0.5)
China	2041	1436.9 (1225.2 to 1656.8)	917.0 (754.2 to 1106.8)	443.9 (335.7 to 572.4)	76.0 (38.4 to 133.1)	0.0 (0.0 to 0.3)	2660.9 (2268.9 to 3068.1)	1698.1 (1396.7 to 2049.5)	821.9 (621.7 to 1060.0)	140.8 (71.1 to 246.5)	0.1 (0.0 to 0.5)
China	2042	1500.9 (1282.4 to 1745.0)	962.4 (788.7 to 1162.6)	458.8 (346.0 to 592.6)	79.7 (40.2 to 139.4)	0.0 (0.0 to 0.3)	2779.3 (2374.7 to 3231.3)	1782.1 (1460.5 to 2152.9)	849.5 (640.7 to 1097.4)	147.5 (74.4 to 258.1)	0.1 (0.0 to 0.5)
China	2043	1566.6 (1329.9 to 1820.9)	1009.3 (825.9 to 1216.9)	473.8 (355.9 to 612.6)	83.5 (42.0 to 146.1)	0.0 (0.0 to 0.3)	2901.0 (2462.6 to 3371.9)	1868.9 (1529.4 to 2253.4)	877.3 (659.1 to 1134.4)	154.7 (77.8 to 270.6)	0.1 (0.0 to 0.5)
China	2044	1634.1 (1404.6 to 1894.3)	1057.6 (865.2 to 1271.3)	488.8 (366.9 to 634.5)	87.6 (44.0 to 153.4)	0.0 (0.0 to 0.3)	3025.9 (2601.0 to 3507.8)	1958.5 (1602.1 to 2354.1)	905.2 (679.5 to 1175.0)	162.2 (81.4 to 284.1)	0.0 (0.0 to 0.5)
China	2045	1703.2 (1432.5 to 1995.6)	1107.7 (907.3 to 1330.9)	503.7 (378.4 to 655.9)	91.8 (46.0 to 161.2)	0.0 (0.0 to 0.3)	3153.9 (2652.6 to 3695.3)	2051.2 (1680.1 to 2464.5)	932.7 (700.7 to 1214.5)	170.0 (85.1 to 298.6)	0.0 (0.0 to 0.5)
China	2046	1772.4 (1516.2 to 2069.5)	1158.2 (949.4 to 1392.8)	518.1 (388.9 to 673.8)	96.0 (48.0 to 169.2)	0.0 (0.0 to 0.3)	3282.1 (2807.6 to 3832.3)	2144.8 (1758.0 to 2579.1)	959.5 (720.2 to 1247.8)	177.8 (88.8 to 313.3)	0.0 (0.0 to 0.5)
China	2047	1841.7 (1565.6 to 2132.1)	1209.2 (991.8 to 1456.4)	532.2 (399.4 to 691.7)	100.3 (50.0 to 177.4)	0.0 (0.0 to 0.3)	3410.4 (2899.2 to 3948.2)	2239.1 (1836.6 to 2697.0)	985.5 (739.6 to 1280.9)	185.8 (92.6 to 328.5)	0.0 (0.0 to 0.5)
China	2048	1911.6 (1640.2 to 2240.3)	1260.5 (1034.5 to 1518.9)	546.4 (410.2 to 710.0)	104.7 (52.1 to 185.6)	0.0 (0.0 to 0.3)	3539.9 (3037.3 to 4148.6)	2334.2 (1915.7 to 2812.6)	1011.8 (759.5 to 1314.8)	193.9 (96.5 to 343.7)	0.0 (0.0 to 0.5)
China	2049	1982.3 (1668.6 to 2331.0)	1313.0 (1074.5 to 1587.4)	560.3 (421.4 to 729.4)	109.0 (54.1 to 193.2)	0.0 (0.0 to 0.3)	3670.8 (3090.0 to 4316.5)	2431.4 (1989.7 to 2939.5)	1037.6 (780.4 to 1350.6)	201.8 (100.2 to 357.8)	0.0 (0.0 to 0.5)
China	2050	2053.9 (1728.1 to 2407.5)	1366.8 (1116.4 to 1652.5)	574.0 (432.3 to 748.7)	113.1 (56.1 to 200.4)	0.0 (0.0 to 0.0)	3803.3 (3200.0 to 4458.1)	2530.9 (2067.4 to 3060.1)	1063.0 (800.5 to 1386.5)	209.4 (103.8 to 371.2)	0.0 (0.0 to 0.0)
Colombia	1995	301.9 (267.7 to 337.1)	168.0 (145.3 to 192.3)	115.7 (92.7 to 143.7)	17.6 (10.2 to 28.4)	0.7 (0.7 to 0.7)	719.7 (638.1 to 803.5)	400.5 (346.3 to 458.4)	275.7 (221.0 to 342.4)	41.9 (24.2 to 67.8)	1.6 (1.6 to 1.6)
Colombia	1996	319.4 (286.6 to 351.8)	183.5 (161.8 to 207.0)	115.4 (94.6 to 141.3)	18.6 (10.7 to 29.5)	1.9 (1.9 to 1.9)	761.4 (683.2 to 838.5)	437.5 (385.6 to 493.5)	275.1 (225.5 to 336.8)	44.4 (25.5 to 70.3)	4.4 (4.4 to 4.4)
Colombia	1997	327.9 (298.7 to 358.7)	197.3 (175.2 to 220.4)	109.0 (90.1 to 130.7)	20.1 (11.5 to 31.9)	1.5 (1.5 to 1.5)	781.6 (711.9 to 855.1)	470.3 (417.6 to 525.4)	259.9 (214.8 to 311.6)	47.8 (27.4 to 76.0)	3.6 (3.6 to 3.6)
Colombia	1998	311.1 (284.9 to 339.3)	201.7 (179.0 to 224.7)	87.3 (72.0 to 105.3)	21.2 (12.5 to 33.2)	0.9 (0.9 to 0.9)	741.6 (679.2 to 808.9)	480.7 (426.6 to 535.7)	208.1 (171.6 to 251.0)	50.5 (29.8 to 79.0)	2.2 (2.2 to 2.2)
Colombia	1999	278.2 (254.0 to 304.7)	192.3 (171.2 to 212.9)	63.0 (51.5 to 76.5)	21.2 (12.8 to 33.5)	1.6 (1.6 to 1.6)	663.0 (605.4 to 726.2)	458.5 (408.1 to 507.4)	150.2 (122.9 to 182.3)	50.5 (30.5 to 79.8)	3.9 (3.9 to 3.9)
Colombia	2000	248.9 (225.9 to 272.6)	184.5 (164.4 to 205.1)	43.5 (35.0 to 53.0)	20.4 (12.3 to 31.9)	0.6 (0.6 to 0.6)	593.3 (538.4 to 649.8)	439.7 (391.8 to 488.8)	103.6 (83.5 to 126.3)	48.6 (29.2 to 76.1)	1.4 (1.4 to 1.4)
Colombia	2001	235.7 (213.9 to 259.2)	177.9 (158.7 to 198.4)	35.7 (28.7 to 43.6)	20.1 (12.0 to 31.4)	2.1 (2.1 to 2.1)	561.8 (509.8 to 617.9)	424.1 (378.2 to 473.0)	85.0 (68.3 to 103.9)	47.9 (28.7 to 74.8)	4.9 (4.9 to 4.9)

		2018 US Dollars per capita					2018 PPP per capita				
Country	Year	Health spending	Government health spending	Out-of-pocket health spending	Prepaid private health spending	Development assistance for health	Health spending	Government health spending	Out-of-pocket health spending	Prepaid private health spending	Development assistance for health
Colombia	2002	229.9 (208.6 to 253.5)	175.1 (156.8 to 194.4)	31.3 (24.9 to 38.9)	20.0 (12.0 to 30.9)	3.5 (3.5 to 3.5)	548.1 (497.2 to 604.2)	417.4 (373.7 to 463.4)	74.6 (59.3 to 92.8)	47.7 (28.7 to 73.6)	8.4 (8.4 to 8.4)
Colombia	2003	232.4 (212.5 to 255.7)	177.0 (158.5 to 196.8)	30.3 (23.9 to 38.2)	20.8 (12.8 to 31.8)	4.3 (4.3 to 4.3)	554.0 (506.5 to 609.4)	422.0 (377.8 to 469.1)	72.3 (57.0 to 91.1)	49.6 (30.4 to 75.9)	10.2 (10.2 to 10.2)
Colombia	2004	242.3 (221.0 to 265.9)	175.7 (157.4 to 194.9)	35.6 (28.0 to 44.5)	20.9 (12.7 to 31.6)	10.1 (10.1 to 10.1)	577.5 (526.8 to 633.8)	418.8 (375.2 to 464.6)	84.9 (66.6 to 106.0)	49.8 (30.3 to 75.3)	24.1 (24.1 to 24.1)
Colombia	2005	249.9 (227.4 to 274.8)	180.0 (161.0 to 199.7)	42.8 (34.0 to 53.2)	21.3 (13.2 to 31.9)	5.8 (5.8 to 5.8)	595.7 (541.9 to 655.1)	429.1 (383.7 to 475.9)	102.0 (81.0 to 126.8)	50.7 (31.5 to 76.0)	13.9 (13.9 to 13.9)
Colombia	2006	266.3 (241.3 to 292.3)	190.7 (170.6 to 212.1)	50.8 (41.1 to 62.9)	21.9 (13.6 to 32.6)	2.9 (2.9 to 2.9)	634.6 (575.1 to 696.8)	454.5 (406.6 to 505.4)	121.1 (98.1 to 149.8)	52.1 (32.3 to 77.8)	6.9 (6.9 to 6.9)
Colombia	2007	285.8 (259.2 to 313.4)	200.9 (181.0 to 222.7)	58.5 (47.8 to 71.4)	23.3 (14.3 to 35.4)	3.0 (3.0 to 3.0)	681.2 (617.8 to 747.0)	478.9 (431.4 to 530.9)	139.5 (113.9 to 170.3)	55.5 (34.1 to 84.3)	7.3 (7.3 to 7.3)
Colombia	2008	300.0 (272.3 to 329.0)	209.0 (189.5 to 231.5)	64.5 (52.4 to 78.7)	25.0 (15.4 to 37.1)	1.5 (1.5 to 1.5)	715.1 (649.0 to 784.3)	498.3 (451.8 to 551.8)	153.6 (124.9 to 187.5)	59.7 (36.8 to 88.4)	3.6 (3.6 to 3.6)
Colombia	2009	311.5 (282.7 to 341.6)	214.1 (193.2 to 237.0)	66.0 (54.1 to 80.7)	26.7 (16.9 to 39.2)	4.7 (4.7 to 4.7)	742.6 (673.9 to 814.1)	510.3 (460.6 to 564.9)	157.3 (128.9 to 192.4)	63.7 (40.4 to 93.4)	11.3 (11.3 to 11.3)
Colombia	2010	319.9 (291.9 to 349.3)	217.5 (198.0 to 240.9)	67.2 (55.1 to 82.4)	28.3 (17.8 to 40.9)	6.9 (6.9 to 6.9)	762.5 (695.7 to 832.7)	518.4 (472.1 to 574.3)	160.3 (131.3 to 196.5)	67.5 (42.5 to 97.6)	16.4 (16.4 to 16.4)
Colombia	2011	321.5 (294.0 to 350.6)	220.8 (200.5 to 243.9)	68.3 (55.9 to 83.0)	30.2 (19.4 to 44.7)	2.3 (2.3 to 2.3)	766.3 (700.9 to 835.6)	526.2 (478.0 to 581.4)	162.8 (133.3 to 197.8)	71.9 (46.3 to 106.6)	5.4 (5.4 to 5.4)
Colombia	2012	323.0 (296.4 to 351.6)	219.6 (198.6 to 241.2)	69.0 (55.9 to 83.0)	32.6 (21.5 to 47.7)	1.9 (1.9 to 1.9)	770.0 (706.4 to 838.1)	523.4 (473.3 to 574.9)	164.4 (133.2 to 197.9)	77.7 (51.4 to 113.6)	4.4 (4.4 to 4.4)
Colombia	2013	338.1 (310.7 to 367.9)	221.6 (199.8 to 244.1)	70.3 (56.8 to 85.5)	37.7 (25.4 to 55.3)	8.6 (8.6 to 8.6)	805.9 (740.6 to 876.8)	528.1 (476.2 to 581.9)	167.6 (135.4 to 203.9)	89.7 (60.5 to 131.8)	20.5 (20.5 to 20.5)
Colombia	2014	355.0 (323.8 to 387.1)	224.8 (200.7 to 248.3)	71.3 (57.3 to 87.9)	42.3 (27.6 to 63.9)	16.6 (16.6 to 16.6)	846.2 (771.7 to 922.8)	535.8 (478.3 to 591.9)	170.1 (136.6 to 209.5)	100.7 (65.9 to 152.3)	39.6 (39.6 to 39.6)
Colombia	2015	349.8 (313.9 to 386.3)	229.7 (203.1 to 255.9)	72.6 (57.4 to 89.7)	47.1 (29.8 to 70.2)	0.4 (0.4 to 0.4)	833.8 (748.1 to 920.9)	547.6 (484.0 to 609.9)	173.0 (136.7 to 213.8)	112.2 (71.0 to 167.4)	0.9 (0.9 to 0.9)
Colombia	2016	357.8 (315.0 to 398.8)	232.8 (203.1 to 263.2)	73.7 (57.0 to 92.5)	51.0 (30.8 to 77.8)	0.3 (0.3 to 0.3)	852.8 (750.7 to 950.5)	554.9 (484.1 to 627.2)	175.6 (135.9 to 220.6)	121.6 (73.4 to 185.4)	0.7 (0.7 to 0.7)
Colombia	2017	369.1 (326.3 to 414.5)	242.4 (211.0 to 274.9)	74.6 (57.6 to 93.6)	51.8 (31.2 to 79.1)	0.3 (0.3 to 0.3)	879.8 (777.6 to 988.1)	577.8 (502.9 to 655.2)	177.7 (137.2 to 223.0)	123.4 (74.4 to 188.5)	0.8 (0.8 to 0.8)
Colombia	2018	374.9 (329.8 to 421.4)	246.5 (214.4 to 279.3)	75.3 (58.2 to 95.1)	52.7 (31.8 to 80.4)	0.3 (0.3 to 0.3)	893.5 (786.1 to 1004.4)	587.6 (511.1 to 665.7)	179.5 (138.6 to 226.8)	125.6 (75.8 to 191.7)	0.7 (0.7 to 0.7)
Colombia	2019	380.6 (335.5 to 426.8)	250.6 (218.5 to 284.5)	76.1 (58.9 to 95.9)	53.6 (32.4 to 81.8)	0.3 (0.3 to 0.3)	907.2 (799.8 to 1017.4)	597.2 (520.8 to 678.1)	181.4 (140.4 to 228.6)	127.8 (77.3 to 195.0)	0.8 (0.7 to 0.8)
Colombia	2020	386.4 (339.5 to 432.9)	254.6 (221.8 to 288.6)	76.9 (59.5 to 96.9)	54.6 (32.9 to 83.3)	0.3 (0.3 to 0.3)	920.9 (809.1 to 1031.7)	606.7 (528.7 to 687.8)	183.3 (141.9 to 231.0)	130.1 (78.5 to 198.7)	0.8 (0.7 to 0.8)
Colombia	2021	392.4 (344.8 to 440.3)	258.7 (225.3 to 293.3)	77.8 (60.2 to 97.9)	55.6 (33.6 to 84.5)	0.3 (0.3 to 0.4)	935.2 (821.9 to 1049.5)	616.5 (537.0 to 699.0)	185.4 (143.5 to 233.4)	132.6 (80.0 to 201.4)	0.8 (0.7 to 0.9)
Colombia	2022	398.2 (351.1 to 447.0)	262.7 (228.8 to 297.8)	78.6 (60.8 to 99.1)	56.6 (34.2 to 85.9)	0.3 (0.3 to 0.4)	949.1 (836.9 to 1065.5)	626.1 (545.3 to 709.7)	187.3 (144.8 to 236.2)	134.9 (81.6 to 204.8)	0.8 (0.7 to 0.9)

Country	Year	2018 US Dollars per capita					2018 PPP per capita				
		Health spending	Government health spending	Out-of-pocket health spending	Prepaid private health spending	Development assistance for health	Health spending	Government health spending	Out-of-pocket health spending	Prepaid private health spending	Development assistance for health
Colombia	2023	404.6 (357.3 to 454.4)	267.2 (233.1 to 303.0)	79.5 (61.4 to 100.4)	57.6 (34.8 to 87.6)	0.3 (0.3 to 0.4)	964.4 (851.6 to 1083.1)	636.8 (555.5 to 722.2)	189.4 (146.3 to 239.2)	137.4 (83.0 to 208.9)	0.8 (0.7 to 0.9)
Colombia	2024	411.2 (361.8 to 462.0)	271.8 (236.9 to 308.6)	80.3 (62.0 to 101.4)	58.7 (35.5 to 89.2)	0.3 (0.3 to 0.4)	980.0 (862.4 to 1101.3)	647.9 (564.8 to 735.6)	191.4 (147.8 to 241.6)	139.9 (84.7 to 212.6)	0.8 (0.7 to 1.0)
Colombia	2025	417.5 (368.6 to 468.1)	276.3 (240.4 to 313.8)	81.1 (62.7 to 102.2)	59.7 (36.2 to 90.8)	0.4 (0.3 to 0.4)	995.1 (878.5 to 1115.7)	658.6 (573.1 to 747.9)	193.3 (149.3 to 243.6)	142.3 (86.3 to 216.5)	0.8 (0.7 to 1.0)
Colombia	2026	423.3 (373.0 to 477.3)	280.3 (244.0 to 318.6)	81.9 (63.3 to 103.1)	60.8 (36.9 to 92.4)	0.4 (0.3 to 0.4)	1008.9 (889.0 to 1137.7)	668.0 (581.5 to 759.3)	195.2 (150.8 to 245.7)	144.8 (87.9 to 220.3)	0.9 (0.7 to 1.0)
Colombia	2027	428.9 (378.4 to 480.5)	284.1 (247.4 to 323.1)	82.7 (63.9 to 104.1)	61.8 (37.5 to 94.0)	0.4 (0.3 to 0.4)	1022.3 (902.0 to 1145.2)	677.1 (589.7 to 770.2)	197.0 (152.2 to 248.0)	147.3 (89.4 to 224.1)	0.9 (0.7 to 1.1)
Colombia	2028	435.0 (385.1 to 488.2)	288.5 (251.6 to 328.4)	83.4 (64.3 to 105.6)	62.8 (38.1 to 95.7)	0.4 (0.3 to 0.5)	1036.9 (918.0 to 1163.7)	687.5 (599.7 to 782.7)	198.7 (153.3 to 251.8)	149.7 (90.9 to 228.0)	0.9 (0.8 to 1.1)
Colombia	2029	441.0 (388.2 to 495.9)	292.7 (255.1 to 333.8)	84.1 (64.7 to 106.4)	63.8 (38.7 to 97.5)	0.4 (0.3 to 0.5)	1051.1 (925.2 to 1181.9)	697.8 (608.1 to 795.6)	200.3 (154.2 to 253.7)	152.0 (92.3 to 232.5)	0.9 (0.8 to 1.2)
Colombia	2030	447.0 (393.3 to 502.8)	297.1 (258.3 to 339.2)	84.7 (65.1 to 107.2)	64.8 (39.3 to 99.5)	0.4 (0.3 to 0.5)	1065.5 (937.5 to 1198.5)	708.2 (615.7 to 808.6)	202.0 (155.2 to 255.6)	154.4 (93.7 to 237.1)	1.0 (0.8 to 1.2)
Colombia	2031	453.0 (398.7 to 509.7)	301.4 (261.8 to 344.6)	85.4 (65.5 to 108.0)	65.8 (39.8 to 101.4)	0.4 (0.3 to 0.5)	1079.9 (950.3 to 1214.9)	718.5 (624.0 to 821.4)	203.6 (156.1 to 257.4)	156.8 (94.8 to 241.8)	1.0 (0.8 to 1.2)
Colombia	2032	459.1 (405.6 to 518.9)	305.8 (265.1 to 350.1)	86.1 (66.0 to 108.7)	66.8 (40.2 to 103.4)	0.4 (0.3 to 0.5)	1094.2 (966.7 to 1236.8)	728.9 (631.9 to 834.4)	205.1 (157.3 to 259.2)	159.2 (95.8 to 246.4)	1.0 (0.8 to 1.3)
Colombia	2033	465.2 (409.2 to 527.4)	310.2 (268.5 to 356.3)	86.7 (66.5 to 109.7)	67.8 (40.7 to 105.1)	0.4 (0.3 to 0.6)	1108.9 (975.4 to 1257.1)	739.5 (640.0 to 849.1)	206.7 (158.4 to 261.4)	161.6 (97.1 to 250.6)	1.0 (0.8 to 1.4)
Colombia	2034	471.5 (417.0 to 533.1)	314.8 (272.0 to 361.6)	87.4 (66.9 to 110.4)	68.9 (41.4 to 106.9)	0.5 (0.4 to 0.6)	1123.9 (993.9 to 1270.7)	750.3 (648.2 to 862.0)	208.4 (159.6 to 263.2)	164.2 (98.7 to 254.7)	1.1 (0.8 to 1.4)
Colombia	2035	477.8 (420.3 to 543.0)	319.3 (275.4 to 368.3)	88.1 (67.4 to 111.1)	69.9 (42.0 to 108.6)	0.5 (0.4 to 0.6)	1138.8 (1001.8 to 1294.3)	761.0 (656.3 to 877.9)	210.0 (160.7 to 264.9)	166.7 (100.2 to 258.9)	1.1 (0.9 to 1.5)
Colombia	2036	484.7 (424.5 to 549.5)	324.3 (279.6 to 374.8)	88.9 (68.0 to 112.4)	71.1 (42.8 to 110.5)	0.5 (0.4 to 0.6)	1155.3 (1011.8 to 1309.9)	772.9 (666.3 to 893.4)	211.9 (162.2 to 268.0)	169.4 (101.9 to 263.4)	1.1 (0.9 to 1.5)
Colombia	2037	491.8 (429.4 to 559.4)	329.4 (283.6 to 381.0)	89.7 (68.6 to 113.2)	72.3 (43.5 to 112.5)	0.5 (0.4 to 0.7)	1172.2 (1023.4 to 1333.4)	785.1 (676.0 to 908.0)	213.8 (163.4 to 269.9)	172.2 (103.7 to 268.1)	1.2 (0.9 to 1.6)
Colombia	2038	499.2 (436.1 to 570.9)	334.7 (287.6 to 387.7)	90.5 (69.4 to 114.1)	73.5 (44.3 to 114.5)	0.5 (0.4 to 0.7)	1189.9 (1039.6 to 1360.6)	797.8 (685.4 to 924.2)	215.8 (165.3 to 272.0)	175.2 (105.5 to 272.9)	1.2 (0.9 to 1.7)
Colombia	2039	506.6 (441.7 to 581.6)	340.0 (291.4 to 393.9)	91.4 (70.0 to 115.3)	74.7 (45.0 to 116.8)	0.5 (0.4 to 0.7)	1207.6 (1052.9 to 1386.2)	810.4 (694.5 to 938.8)	217.8 (166.9 to 274.8)	178.1 (107.3 to 278.4)	1.2 (0.9 to 1.7)
Colombia	2040	514.0 (448.2 to 588.6)	345.3 (295.3 to 400.7)	92.2 (70.7 to 116.5)	75.9 (45.7 to 119.0)	0.5 (0.4 to 0.7)	1225.0 (1068.4 to 1403.0)	823.0 (703.8 to 955.1)	219.7 (168.4 to 277.7)	181.0 (108.9 to 283.7)	1.3 (1.0 to 1.8)
Colombia	2041	521.5 (453.8 to 598.4)	350.7 (299.3 to 407.3)	93.0 (71.2 to 117.7)	77.2 (46.4 to 120.7)	0.6 (0.4 to 0.8)	1242.9 (1081.6 to 1426.3)	835.9 (713.5 to 970.8)	221.7 (169.8 to 280.6)	184.0 (110.6 to 287.7)	1.3 (1.0 to 1.8)
Colombia	2042	528.7 (459.4 to 604.8)	355.9 (303.3 to 414.0)	93.8 (71.7 to 118.7)	78.4 (47.1 to 122.9)	0.6 (0.4 to 0.8)	1260.2 (1095.0 to 1441.5)	848.4 (722.8 to 986.8)	223.5 (171.0 to 283.0)	186.9 (112.4 to 293.0)	1.4 (1.0 to 2.0)
Colombia	2043	535.8 (465.6 to 615.9)	361.1 (307.1 to 421.2)	94.5 (72.2 to 119.5)	79.6 (47.9 to 124.9)	0.6 (0.4 to 0.9)	1277.1 (1109.9 to 1468.1)	860.6 (732.0 to 1004.0)	225.3 (172.1 to 284.7)	189.8 (114.1 to 297.7)	1.4 (1.0 to 2.1)

		2018 US Dollars per capita					2018 PPP per capita				
Country	Year	Health spending	Government health spending	Out-of-pocket health spending	Prepaid private health spending	Development assistance for health	Health spending	Government health spending	Out-of-pocket health spending	Prepaid private health spending	Development assistance for health
Colombia	2044	542.8 (468.0 to 624.9)	366.1 (310.9 to 427.9)	95.2 (72.7 to 120.3)	80.8 (48.6 to 126.7)	0.6 (0.5 to 0.9)	1293.8 (1115.5 to 1489.4)	872.7 (741.0 to 1019.9)	226.9 (173.2 to 286.7)	192.7 (115.8 to 301.9)	1.4 (1.1 to 2.1)
Colombia	2045	549.8 (475.5 to 631.2)	371.2 (314.6 to 435.0)	95.9 (73.2 to 121.7)	82.0 (49.3 to 128.8)	0.6 (0.5 to 0.9)	1310.5 (1133.5 to 1504.4)	884.8 (749.9 to 1036.9)	228.6 (174.4 to 290.1)	195.5 (117.6 to 307.1)	1.5 (1.1 to 2.2)
Colombia	2046	556.6 (481.0 to 640.3)	376.1 (318.2 to 442.0)	96.6 (73.6 to 122.8)	83.2 (50.1 to 131.0)	0.6 (0.5 to 1.0)	1326.6 (1146.6 to 1526.1)	896.5 (758.5 to 1053.5)	230.2 (175.5 to 292.8)	198.3 (119.5 to 312.2)	1.5 (1.1 to 2.4)
Colombia	2047	563.6 (484.5 to 649.3)	381.2 (321.5 to 449.8)	97.3 (74.2 to 123.7)	84.4 (50.8 to 132.7)	0.7 (0.5 to 1.0)	1343.3 (1154.8 to 1547.5)	908.6 (766.2 to 1072.1)	231.8 (176.8 to 294.8)	201.3 (121.0 to 316.3)	1.6 (1.2 to 2.5)
Colombia	2048	570.6 (488.6 to 658.7)	386.3 (324.7 to 458.0)	97.9 (74.7 to 124.5)	85.7 (51.4 to 134.6)	0.7 (0.5 to 1.1)	1360.0 (1164.7 to 1570.1)	920.7 (773.9 to 1091.6)	233.5 (177.9 to 296.8)	204.2 (122.5 to 320.9)	1.7 (1.2 to 2.6)
Colombia	2049	577.3 (493.5 to 667.9)	391.1 (328.0 to 465.2)	98.6 (75.0 to 125.6)	86.9 (52.0 to 136.4)	0.7 (0.5 to 1.2)	1376.0 (1176.3 to 1592.0)	932.3 (781.9 to 1108.9)	235.0 (178.8 to 299.3)	207.0 (124.0 to 325.1)	1.7 (1.2 to 2.8)
Colombia	2050	584.2 (499.9 to 675.2)	396.2 (331.6 to 472.4)	99.2 (75.5 to 126.4)	88.1 (52.7 to 138.2)	0.7 (0.5 to 1.2)	1392.5 (1191.5 to 1609.4)	944.3 (790.4 to 1125.9)	236.5 (180.0 to 301.3)	209.9 (125.6 to 329.5)	1.8 (1.2 to 2.9)
Comoros	1995	112.0 (94.4 to 132.3)	11.6 (8.9 to 14.8)	75.7 (58.2 to 95.8)	1.6 (0.7 to 3.3)	23.1 (23.1 to 23.1)	219.4 (185.0 to 259.1)	22.7 (17.5 to 28.9)	148.4 (114.0 to 187.7)	3.1 (1.4 to 6.4)	45.2 (45.2 to 45.2)
Comoros	1996	97.2 (80.9 to 116.0)	11.1 (8.6 to 14.0)	73.9 (58.1 to 92.0)	1.5 (0.7 to 3.1)	10.7 (10.7 to 10.7)	190.5 (158.4 to 227.3)	21.8 (16.8 to 27.5)	144.8 (113.8 to 180.3)	3.0 (1.4 to 6.0)	21.0 (21.0 to 21.0)
Comoros	1997	102.8 (87.3 to 120.6)	11.2 (8.7 to 14.2)	73.5 (57.7 to 90.6)	1.5 (0.7 to 2.9)	16.6 (16.6 to 16.6)	201.4 (171.0 to 236.2)	22.0 (17.1 to 27.9)	144.0 (113.1 to 177.5)	2.9 (1.4 to 5.7)	32.5 (32.5 to 32.5)
Comoros	1998	106.6 (91.5 to 123.5)	11.0 (8.6 to 13.8)	72.8 (57.7 to 89.8)	1.4 (0.7 to 2.7)	21.3 (21.3 to 21.3)	208.8 (179.2 to 242.0)	21.6 (16.8 to 27.0)	142.7 (113.0 to 175.9)	2.8 (1.3 to 5.3)	41.7 (41.7 to 41.7)
Comoros	1999	90.0 (75.3 to 107.6)	11.3 (8.8 to 14.1)	74.1 (59.9 to 91.0)	1.4 (0.7 to 2.7)	3.2 (3.2 to 3.2)	176.4 (147.6 to 210.9)	22.1 (17.3 to 27.7)	145.2 (117.4 to 178.3)	2.8 (1.3 to 5.2)	6.3 (6.3 to 6.3)
Comoros	2000	95.5 (80.1 to 113.4)	12.0 (9.4 to 15.0)	77.5 (62.5 to 94.6)	1.4 (0.7 to 2.7)	4.5 (4.5 to 4.5)	187.0 (157.0 to 222.1)	23.6 (18.4 to 29.4)	151.9 (122.4 to 185.2)	2.8 (1.3 to 5.3)	8.7 (8.7 to 8.7)
Comoros	2001	95.7 (80.7 to 113.1)	12.3 (9.6 to 15.4)	77.1 (62.0 to 93.5)	1.4 (0.7 to 2.7)	4.8 (4.8 to 4.8)	187.5 (158.0 to 221.7)	24.1 (18.7 to 30.2)	151.1 (121.4 to 183.3)	2.8 (1.3 to 5.2)	9.5 (9.5 to 9.5)
Comoros	2002	100.7 (85.8 to 118.1)	13.6 (10.4 to 17.1)	77.5 (62.7 to 94.2)	1.5 (0.7 to 2.9)	8.0 (8.0 to 8.0)	197.2 (168.1 to 231.4)	26.6 (20.4 to 33.5)	151.9 (122.9 to 184.6)	3.0 (1.4 to 5.7)	15.7 (15.7 to 15.7)
Comoros	2003	100.5 (86.3 to 118.2)	13.2 (10.1 to 16.5)	74.6 (60.5 to 91.0)	1.5 (0.7 to 2.8)	11.2 (11.2 to 11.2)	196.8 (169.2 to 231.5)	25.9 (19.8 to 32.3)	146.1 (118.5 to 178.4)	2.9 (1.4 to 5.4)	22.0 (22.0 to 22.0)
Comoros	2004	93.3 (79.9 to 109.0)	12.4 (9.5 to 15.6)	71.8 (58.3 to 87.9)	1.4 (0.7 to 2.6)	7.7 (7.7 to 7.7)	182.8 (156.5 to 213.5)	24.3 (18.6 to 30.5)	140.8 (114.3 to 172.2)	2.7 (1.3 to 5.1)	15.1 (15.1 to 15.1)
Comoros	2005	88.3 (74.7 to 104.0)	11.7 (8.9 to 14.8)	70.5 (57.5 to 85.7)	1.3 (0.6 to 2.4)	4.8 (4.8 to 4.8)	172.9 (146.4 to 203.8)	22.9 (17.5 to 29.0)	138.1 (112.6 to 168.0)	2.5 (1.2 to 4.8)	9.4 (9.4 to 9.4)
Comoros	2006	85.3 (72.1 to 100.1)	11.0 (8.4 to 14.0)	69.2 (56.5 to 84.1)	1.2 (0.6 to 2.3)	3.9 (3.9 to 3.9)	167.2 (141.3 to 196.1)	21.6 (16.5 to 27.4)	135.6 (110.6 to 164.7)	2.4 (1.1 to 4.5)	7.6 (7.6 to 7.6)
Comoros	2007	81.2 (68.5 to 96.3)	9.9 (7.6 to 12.6)	66.6 (54.4 to 81.1)	1.1 (0.5 to 2.1)	3.6 (3.6 to 3.6)	159.2 (134.2 to 188.7)	19.5 (14.9 to 24.8)	130.4 (106.5 to 158.9)	2.2 (1.1 to 4.1)	7.1 (7.1 to 7.1)
Comoros	2008	76.8 (64.6 to 91.2)	9.0 (6.8 to 11.4)	63.7 (51.7 to 77.6)	1.0 (0.5 to 1.8)	3.1 (3.1 to 3.1)	150.4 (126.6 to 178.7)	17.6 (13.4 to 22.3)	124.8 (101.3 to 152.1)	2.0 (1.0 to 3.6)	6.0 (6.0 to 6.0)

Country	Year	2018 US Dollars per capita					2018 PPP per capita				
		Health spending	Government health spending	Out-of-pocket health spending	Prepaid private health spending	Development assistance for health	Health spending	Government health spending	Out-of-pocket health spending	Prepaid private health spending	Development assistance for health
Comoros	2009	77.0 (65.4 to 91.0)	8.5 (6.4 to 10.8)	61.4 (49.8 to 75.6)	0.9 (0.5 to 1.7)	6.2 (6.2 to 6.2)	150.9 (128.2 to 178.2)	16.6 (12.6 to 21.1)	120.3 (97.5 to 148.0)	1.8 (0.9 to 3.4)	12.2 (12.2 to 12.2)
Comoros	2010	86.8 (74.9 to 100.6)	8.7 (6.6 to 11.0)	60.0 (48.3 to 74.2)	0.9 (0.4 to 1.6)	17.2 (17.2 to 17.2)	170.0 (146.8 to 197.2)	17.0 (13.0 to 21.6)	117.6 (94.6 to 145.4)	1.7 (0.9 to 3.2)	33.7 (33.7 to 33.7)
Comoros	2011	81.9 (69.5 to 96.5)	10.0 (7.6 to 12.8)	60.6 (48.3 to 75.2)	1.0 (0.5 to 1.8)	10.4 (10.4 to 10.4)	160.5 (136.1 to 189.1)	19.6 (15.0 to 25.0)	118.6 (94.6 to 147.3)	1.9 (0.9 to 3.5)	20.4 (20.4 to 20.4)
Comoros	2012	87.5 (75.0 to 102.3)	9.4 (7.1 to 12.1)	58.3 (45.9 to 72.8)	0.9 (0.4 to 1.7)	19.0 (19.0 to 19.0)	171.4 (147.0 to 200.3)	18.3 (13.9 to 23.7)	114.2 (90.0 to 142.7)	1.7 (0.9 to 3.2)	37.2 (37.2 to 37.2)
Comoros	2013	84.4 (71.7 to 99.4)	9.4 (7.2 to 12.2)	57.6 (44.9 to 72.7)	0.9 (0.4 to 1.6)	16.5 (16.5 to 16.5)	165.3 (140.5 to 194.8)	18.5 (14.1 to 24.0)	112.8 (88.0 to 142.4)	1.7 (0.8 to 3.1)	32.4 (32.4 to 32.4)
Comoros	2014	77.2 (64.4 to 92.4)	9.5 (7.2 to 12.4)	56.6 (43.9 to 72.1)	0.8 (0.4 to 1.5)	10.3 (10.3 to 10.3)	151.3 (126.2 to 181.1)	18.6 (14.2 to 24.2)	110.9 (85.9 to 141.3)	1.6 (0.8 to 3.0)	20.1 (20.1 to 20.1)
Comoros	2015	72.5 (59.1 to 87.8)	9.8 (7.4 to 12.9)	55.7 (42.9 to 71.0)	0.8 (0.4 to 1.5)	6.1 (6.1 to 6.1)	142.1 (115.7 to 172.0)	19.3 (14.5 to 25.3)	109.2 (84.0 to 139.1)	1.6 (0.8 to 3.0)	12.0 (12.0 to 12.0)
Comoros	2016	80.3 (66.3 to 96.2)	10.2 (7.7 to 13.4)	55.1 (41.6 to 71.0)	0.8 (0.4 to 1.6)	14.1 (14.1 to 14.1)	157.3 (130.0 to 188.5)	20.0 (15.0 to 26.2)	108.0 (81.6 to 139.2)	1.7 (0.8 to 3.1)	27.6 (27.6 to 27.6)
Comoros	2017	74.6 (60.7 to 91.0)	10.5 (7.9 to 13.8)	55.3 (41.7 to 71.0)	0.9 (0.4 to 1.6)	8.0 (8.0 to 8.0)	146.3 (118.9 to 178.3)	20.6 (15.5 to 27.0)	108.2 (81.6 to 139.2)	1.7 (0.8 to 3.1)	15.7 (15.7 to 15.7)
Comoros	2018	74.5 (60.3 to 90.5)	10.7 (8.0 to 14.0)	55.4 (41.9 to 71.4)	0.9 (0.4 to 1.6)	7.5 (7.5 to 7.5)	145.9 (118.1 to 177.3)	20.9 (15.8 to 27.4)	108.6 (82.0 to 139.8)	1.7 (0.8 to 3.2)	14.7 (14.7 to 14.8)
Comoros	2019	74.8 (60.8 to 91.1)	10.8 (8.1 to 14.2)	55.5 (41.8 to 71.4)	0.9 (0.4 to 1.6)	7.6 (7.1 to 8.0)	146.6 (119.1 to 178.4)	21.2 (15.9 to 27.8)	108.8 (81.9 to 140.0)	1.7 (0.8 to 3.2)	14.9 (13.8 to 15.7)
Comoros	2020	75.2 (60.9 to 91.1)	10.9 (8.2 to 14.4)	55.6 (41.8 to 71.5)	0.9 (0.4 to 1.7)	7.8 (7.1 to 8.4)	147.4 (119.3 to 178.5)	21.4 (16.1 to 28.1)	109.0 (82.0 to 140.1)	1.8 (0.8 to 3.2)	15.2 (13.9 to 16.5)
Comoros	2021	75.8 (62.0 to 92.2)	11.1 (8.4 to 14.6)	55.8 (41.9 to 71.8)	0.9 (0.4 to 1.7)	8.0 (7.2 to 8.8)	148.4 (121.4 to 180.6)	21.8 (16.4 to 28.5)	109.3 (82.0 to 140.6)	1.8 (0.8 to 3.3)	15.6 (14.0 to 17.3)
Comoros	2022	76.3 (62.2 to 92.8)	11.3 (8.5 to 14.8)	55.9 (42.0 to 71.9)	0.9 (0.4 to 1.7)	8.3 (7.3 to 9.3)	149.6 (121.9 to 181.9)	22.1 (16.6 to 29.0)	109.5 (82.2 to 140.8)	1.8 (0.8 to 3.3)	16.2 (14.3 to 18.2)
Comoros	2023	76.9 (63.2 to 93.3)	11.4 (8.5 to 15.0)	56.0 (42.1 to 72.0)	0.9 (0.4 to 1.7)	8.5 (7.4 to 9.7)	150.7 (123.9 to 182.9)	22.4 (16.7 to 29.4)	109.7 (82.4 to 141.0)	1.8 (0.8 to 3.4)	16.7 (14.5 to 19.0)
Comoros	2024	77.5 (63.2 to 93.9)	11.6 (8.6 to 15.2)	56.1 (42.2 to 72.2)	0.9 (0.4 to 1.7)	8.9 (7.6 to 10.2)	151.8 (123.8 to 183.9)	22.7 (16.9 to 29.8)	109.9 (82.6 to 141.5)	1.8 (0.9 to 3.4)	17.4 (14.9 to 20.0)
Comoros	2025	78.0 (63.5 to 94.7)	11.8 (8.8 to 15.5)	56.2 (42.2 to 72.5)	1.0 (0.4 to 1.8)	9.1 (7.8 to 10.7)	152.9 (124.4 to 185.6)	23.0 (17.2 to 30.4)	110.1 (82.7 to 142.0)	1.9 (0.9 to 3.4)	17.9 (15.2 to 20.9)
Comoros	2026	78.6 (64.4 to 95.4)	11.9 (9.0 to 15.8)	56.3 (42.3 to 72.5)	1.0 (0.4 to 1.8)	9.4 (7.9 to 11.0)	154.1 (126.1 to 187.0)	23.4 (17.6 to 30.9)	110.3 (82.9 to 142.0)	1.9 (0.9 to 3.5)	18.4 (15.4 to 21.6)
Comoros	2027	79.3 (65.0 to 96.0)	12.1 (9.1 to 16.0)	56.5 (42.5 to 73.0)	1.0 (0.5 to 1.8)	9.7 (8.0 to 11.6)	155.3 (127.3 to 188.2)	23.8 (17.8 to 31.4)	110.7 (83.2 to 143.0)	1.9 (0.9 to 3.5)	18.9 (15.7 to 22.7)
Comoros	2028	80.0 (65.1 to 97.0)	12.3 (9.2 to 16.3)	56.7 (42.6 to 73.3)	1.0 (0.5 to 1.8)	9.9 (8.3 to 12.1)	156.7 (127.6 to 190.0)	24.2 (18.1 to 32.0)	111.1 (83.5 to 143.6)	1.9 (0.9 to 3.6)	19.5 (16.2 to 23.8)
Comoros	2029	80.7 (66.0 to 97.9)	12.5 (9.4 to 16.6)	56.9 (42.5 to 73.9)	1.0 (0.5 to 1.9)	10.2 (8.3 to 12.6)	158.1 (129.3 to 191.8)	24.6 (18.4 to 32.6)	111.5 (83.4 to 144.9)	2.0 (0.9 to 3.6)	20.1 (16.3 to 24.6)

		2018 US Dollars per capita					2018 PPP per capita				
Country	Year	Health spending	Government health spending	Out-of-pocket health spending	Prepaid private health spending	Development assistance for health	Health spending	Government health spending	Out-of-pocket health spending	Prepaid private health spending	Development assistance for health
Comoros	2030	81.4 (66.6 to 98.8)	12.7 (9.5 to 16.9)	57.1 (42.7 to 74.2)	1.0 (0.5 to 1.9)	10.5 (8.5 to 13.0)	159.4 (130.4 to 193.6)	25.0 (18.7 to 33.1)	111.9 (83.6 to 145.4)	2.0 (0.9 to 3.7)	20.6 (16.7 to 25.5)
Comoros	2031	82.1 (67.6 to 99.8)	13.0 (9.7 to 17.1)	57.3 (42.7 to 74.5)	1.0 (0.5 to 1.9)	10.8 (8.6 to 13.6)	160.9 (132.4 to 195.6)	25.4 (19.0 to 33.6)	112.3 (83.7 to 146.1)	2.0 (0.9 to 3.7)	21.1 (16.9 to 26.6)
Comoros	2032	82.9 (67.4 to 100.8)	13.2 (9.9 to 17.5)	57.6 (42.9 to 75.0)	1.0 (0.5 to 1.9)	11.1 (8.7 to 14.2)	162.4 (132.0 to 197.5)	25.8 (19.4 to 34.2)	112.8 (84.0 to 146.9)	2.1 (0.9 to 3.8)	21.7 (17.0 to 27.9)
Comoros	2033	83.7 (68.8 to 101.4)	13.4 (10.1 to 17.6)	57.8 (43.0 to 75.2)	1.1 (0.5 to 2.0)	11.4 (8.8 to 14.6)	163.9 (134.8 to 198.7)	26.2 (19.7 to 34.6)	113.3 (84.3 to 147.4)	2.1 (1.0 to 3.8)	22.3 (17.2 to 28.5)
Comoros	2034	84.5 (68.2 to 102.6)	13.6 (10.2 to 18.0)	58.1 (43.2 to 75.9)	1.1 (0.5 to 2.0)	11.7 (8.9 to 15.4)	165.5 (133.7 to 201.0)	26.7 (20.0 to 35.3)	113.8 (84.6 to 148.6)	2.1 (1.0 to 3.9)	22.9 (17.4 to 30.1)
Comoros	2035	85.3 (70.0 to 103.4)	13.8 (10.4 to 18.3)	58.3 (43.3 to 76.3)	1.1 (0.5 to 2.0)	12.0 (9.0 to 16.1)	167.1 (137.2 to 202.5)	27.1 (20.4 to 35.9)	114.3 (84.9 to 149.6)	2.1 (1.0 to 3.9)	23.6 (17.7 to 31.5)
Comoros	2036	86.1 (70.1 to 104.2)	14.1 (10.6 to 18.6)	58.6 (43.5 to 76.8)	1.1 (0.5 to 2.0)	12.4 (9.3 to 17.1)	168.8 (137.4 to 204.2)	27.6 (20.7 to 36.5)	114.8 (85.2 to 150.4)	2.2 (1.0 to 4.0)	24.2 (18.1 to 33.6)
Comoros	2037	87.0 (70.9 to 105.1)	14.3 (10.7 to 18.9)	58.8 (43.7 to 77.0)	1.1 (0.5 to 2.1)	12.7 (9.4 to 17.9)	170.4 (139.0 to 206.0)	28.0 (21.0 to 37.0)	115.3 (85.5 to 150.9)	2.2 (1.0 to 4.1)	24.8 (18.4 to 35.0)
Comoros	2038	87.8 (71.4 to 107.0)	14.5 (10.9 to 19.2)	59.1 (43.8 to 77.5)	1.1 (0.5 to 2.1)	13.0 (9.5 to 18.5)	172.0 (139.8 to 209.6)	28.5 (21.3 to 37.6)	115.7 (85.9 to 151.9)	2.2 (1.0 to 4.1)	25.6 (18.6 to 36.3)
Comoros	2039	88.6 (72.0 to 107.7)	14.8 (11.0 to 19.5)	59.3 (43.9 to 78.0)	1.2 (0.5 to 2.1)	13.4 (9.7 to 19.4)	173.6 (141.1 to 211.0)	28.9 (21.6 to 38.3)	116.2 (86.0 to 152.8)	2.3 (1.0 to 4.2)	26.2 (19.0 to 38.0)
Comoros	2040	89.5 (73.0 to 108.5)	15.0 (11.2 to 19.9)	59.5 (43.9 to 78.4)	1.2 (0.5 to 2.2)	13.8 (10.0 to 19.9)	175.3 (143.0 to 212.6)	29.4 (22.0 to 38.9)	116.6 (86.0 to 153.6)	2.3 (1.0 to 4.2)	27.0 (19.6 to 39.0)
Comoros	2041	90.4 (73.4 to 110.4)	15.3 (11.4 to 20.2)	59.8 (43.9 to 78.7)	1.2 (0.5 to 2.2)	14.2 (10.2 to 20.7)	177.1 (143.8 to 216.3)	29.9 (22.3 to 39.6)	117.1 (86.1 to 154.1)	2.3 (1.1 to 4.3)	27.8 (19.9 to 40.6)
Comoros	2042	91.3 (74.0 to 110.6)	15.5 (11.6 to 20.6)	60.0 (44.0 to 79.0)	1.2 (0.5 to 2.2)	14.6 (10.2 to 21.6)	178.9 (144.9 to 216.7)	30.4 (22.7 to 40.4)	117.5 (86.3 to 154.8)	2.4 (1.1 to 4.3)	28.7 (20.1 to 42.4)
Comoros	2043	92.3 (75.2 to 111.8)	15.8 (11.7 to 21.1)	60.2 (44.3 to 79.3)	1.2 (0.6 to 2.2)	15.1 (10.5 to 22.3)	180.7 (147.4 to 219.1)	30.9 (23.0 to 41.2)	118.0 (86.8 to 155.4)	2.4 (1.1 to 4.4)	29.5 (20.6 to 43.7)
Comoros	2044	93.2 (75.1 to 112.7)	16.0 (11.9 to 21.4)	60.4 (44.4 to 79.6)	1.2 (0.6 to 2.3)	15.6 (10.7 to 23.7)	182.7 (147.1 to 220.7)	31.4 (23.4 to 42.0)	118.4 (87.0 to 155.9)	2.4 (1.1 to 4.4)	30.5 (21.0 to 46.5)
Comoros	2045	94.3 (76.0 to 115.6)	16.3 (12.1 to 21.9)	60.6 (44.6 to 79.9)	1.3 (0.6 to 2.3)	16.1 (11.0 to 24.8)	184.7 (148.9 to 226.4)	31.9 (23.7 to 42.9)	118.8 (87.3 to 156.6)	2.5 (1.1 to 4.5)	31.5 (21.5 to 48.7)
Comoros	2046	95.4 (76.9 to 116.3)	16.6 (12.3 to 22.3)	60.9 (44.8 to 80.3)	1.3 (0.6 to 2.3)	16.6 (11.2 to 26.0)	186.8 (150.6 to 227.8)	32.5 (24.1 to 43.8)	119.3 (87.8 to 157.2)	2.5 (1.1 to 4.6)	32.5 (21.9 to 51.0)
Comoros	2047	96.5 (78.0 to 118.0)	16.9 (12.5 to 22.6)	61.1 (45.1 to 80.6)	1.3 (0.6 to 2.4)	17.2 (11.6 to 27.4)	189.0 (152.8 to 231.3)	33.1 (24.6 to 44.3)	119.7 (88.3 to 157.9)	2.5 (1.1 to 4.6)	33.7 (22.7 to 53.6)
Comoros	2048	97.7 (78.4 to 118.9)	17.2 (12.7 to 23.0)	61.3 (45.2 to 81.0)	1.3 (0.6 to 2.4)	17.8 (11.8 to 28.5)	191.4 (153.5 to 232.9)	33.7 (25.0 to 45.1)	120.2 (88.5 to 158.7)	2.6 (1.2 to 4.7)	34.9 (23.1 to 55.8)
Comoros	2049	98.9 (79.9 to 121.1)	17.5 (12.9 to 23.5)	61.6 (45.4 to 81.3)	1.3 (0.6 to 2.4)	18.4 (12.1 to 30.1)	193.7 (156.5 to 237.3)	34.3 (25.3 to 46.1)	120.7 (88.9 to 159.3)	2.6 (1.2 to 4.8)	36.1 (23.8 to 58.9)
Comoros	2050	100.2 (80.0 to 123.9)	17.8 (13.1 to 24.0)	61.8 (45.6 to 81.6)	1.4 (0.6 to 2.5)	19.2 (12.4 to 31.6)	196.3 (156.7 to 242.8)	34.9 (25.7 to 47.0)	121.2 (89.3 to 159.9)	2.7 (1.2 to 4.8)	37.5 (24.4 to 61.8)

Country	Year	2018 US Dollars per capita					2018 PPP per capita				
		Health spending	Government health spending	Out-of-pocket health spending	Prepaid private health spending	Development assistance for health	Health spending	Government health spending	Out-of-pocket health spending	Prepaid private health spending	Development assistance for health
Congo	1995	41.9 (34.7 to 50.9)	15.5 (11.8 to 20.1)	21.5 (15.7 to 29.3)	0.6 (0.3 to 1.2)	4.3 (4.3 to 4.3)	124.7 (103.3 to 151.3)	46.0 (35.1 to 59.6)	64.0 (46.6 to 87.0)	1.8 (0.9 to 3.4)	12.9 (12.9 to 12.9)
Congo	1996	37.4 (30.3 to 46.4)	15.4 (11.7 to 19.8)	21.1 (15.4 to 28.7)	0.6 (0.3 to 1.1)	0.2 (0.2 to 0.2)	111.1 (90.1 to 137.9)	45.8 (34.8 to 58.7)	62.8 (45.9 to 85.4)	1.8 (0.9 to 3.3)	0.6 (0.6 to 0.6)
Congo	1997	38.3 (31.5 to 47.2)	15.0 (11.4 to 19.2)	21.1 (15.5 to 28.4)	0.6 (0.3 to 1.1)	1.6 (1.6 to 1.6)	113.8 (93.7 to 140.3)	44.6 (33.9 to 57.0)	62.7 (46.2 to 84.4)	1.8 (0.9 to 3.3)	4.8 (4.8 to 4.8)
Congo	1998	39.0 (32.2 to 47.4)	14.7 (11.2 to 18.7)	21.7 (16.0 to 29.3)	0.6 (0.3 to 1.1)	2.1 (2.1 to 2.1)	116.0 (95.9 to 141.0)	43.6 (33.3 to 55.7)	64.4 (47.6 to 87.2)	1.8 (0.8 to 3.3)	6.2 (6.2 to 6.2)
Congo	1999	34.7 (28.6 to 42.7)	13.3 (10.2 to 17.0)	20.3 (14.9 to 27.1)	0.6 (0.3 to 1.1)	0.6 (0.6 to 0.6)	103.3 (84.9 to 126.8)	39.6 (30.4 to 50.7)	60.2 (44.2 to 80.5)	1.7 (0.8 to 3.2)	1.7 (1.7 to 1.7)
Congo	2000	33.5 (27.6 to 41.1)	13.1 (10.1 to 16.7)	19.5 (14.5 to 26.1)	0.6 (0.3 to 1.1)	0.4 (0.4 to 0.4)	99.7 (82.0 to 122.1)	38.9 (30.0 to 49.8)	58.1 (43.3 to 77.5)	1.7 (0.8 to 3.2)	1.1 (1.1 to 1.1)
Congo	2001	34.5 (28.1 to 42.6)	13.1 (10.1 to 16.8)	20.4 (15.2 to 27.4)	0.6 (0.3 to 1.1)	0.4 (0.4 to 0.4)	102.5 (83.6 to 126.7)	39.0 (30.1 to 50.0)	60.6 (45.3 to 81.4)	1.8 (0.8 to 3.4)	1.2 (1.2 to 1.2)
Congo	2002	33.2 (26.9 to 40.7)	11.1 (8.6 to 14.2)	20.6 (15.1 to 27.5)	0.6 (0.3 to 1.1)	0.9 (0.9 to 0.9)	98.6 (80.1 to 121.0)	33.0 (25.7 to 42.2)	61.2 (45.0 to 81.9)	1.7 (0.8 to 3.1)	2.8 (2.8 to 2.8)
Congo	2003	32.3 (26.0 to 40.0)	10.2 (7.9 to 13.0)	20.6 (15.2 to 27.2)	0.5 (0.3 to 1.0)	0.9 (0.9 to 0.9)	95.9 (77.4 to 118.8)	30.2 (23.4 to 38.6)	61.3 (45.1 to 80.7)	1.6 (0.8 to 2.9)	2.8 (2.8 to 2.8)
Congo	2004	32.6 (26.5 to 40.0)	9.8 (7.6 to 12.4)	19.8 (14.5 to 26.5)	0.6 (0.3 to 1.0)	2.5 (2.5 to 2.5)	97.0 (78.9 to 118.9)	29.1 (22.5 to 36.9)	58.8 (43.2 to 78.8)	1.6 (0.8 to 3.0)	7.5 (7.5 to 7.5)
Congo	2005	32.2 (26.1 to 39.2)	10.4 (7.9 to 13.3)	19.0 (13.8 to 25.1)	0.6 (0.3 to 1.1)	2.3 (2.3 to 2.3)	95.8 (77.7 to 116.6)	31.0 (23.6 to 39.4)	56.4 (41.0 to 74.7)	1.7 (0.8 to 3.2)	6.7 (6.7 to 6.7)
Congo	2006	33.5 (27.3 to 40.7)	11.7 (8.9 to 14.9)	18.5 (13.4 to 24.7)	0.6 (0.3 to 1.1)	2.7 (2.7 to 2.7)	99.8 (81.1 to 121.0)	34.8 (26.6 to 44.2)	55.1 (39.7 to 73.4)	1.8 (0.9 to 3.3)	8.0 (8.0 to 8.0)
Congo	2007	33.3 (27.2 to 40.2)	12.2 (9.3 to 15.7)	18.0 (12.8 to 23.9)	0.6 (0.3 to 1.1)	2.5 (2.5 to 2.5)	99.0 (80.9 to 119.6)	36.4 (27.7 to 46.8)	53.4 (38.2 to 70.9)	1.8 (0.9 to 3.4)	7.4 (7.4 to 7.4)
Congo	2008	38.0 (31.6 to 45.4)	14.6 (11.2 to 18.8)	18.2 (13.1 to 24.3)	0.7 (0.3 to 1.3)	4.6 (4.6 to 4.6)	113.0 (94.1 to 135.0)	43.4 (33.3 to 56.0)	54.1 (38.9 to 72.2)	2.0 (0.9 to 3.8)	13.6 (13.6 to 13.6)
Congo	2009	36.7 (30.3 to 44.0)	14.6 (11.2 to 18.9)	18.5 (13.5 to 24.7)	0.6 (0.3 to 1.2)	3.0 (3.0 to 3.0)	109.2 (90.0 to 130.9)	43.4 (33.5 to 56.1)	55.1 (40.1 to 73.3)	1.9 (0.9 to 3.5)	8.8 (8.8 to 8.8)
Congo	2010	44.7 (37.6 to 52.5)	17.7 (13.8 to 22.9)	18.7 (13.8 to 25.1)	0.6 (0.3 to 1.1)	7.6 (7.6 to 7.6)	132.9 (111.8 to 156.2)	52.7 (40.9 to 68.0)	55.6 (40.9 to 74.6)	1.9 (0.9 to 3.4)	22.7 (22.7 to 22.7)
Congo	2011	44.3 (37.2 to 52.1)	18.9 (14.5 to 24.4)	17.9 (13.0 to 24.0)	0.6 (0.3 to 1.0)	7.0 (7.0 to 7.0)	131.7 (110.6 to 154.8)	56.2 (43.1 to 72.5)	53.1 (38.7 to 71.5)	1.7 (0.8 to 3.0)	20.7 (20.7 to 20.7)
Congo	2012	48.7 (40.8 to 57.2)	22.3 (17.0 to 28.7)	18.1 (13.0 to 24.7)	0.7 (0.3 to 1.3)	7.6 (7.6 to 7.6)	144.7 (121.4 to 170.2)	66.2 (50.4 to 85.4)	53.7 (38.6 to 73.4)	2.1 (1.0 to 3.8)	22.6 (22.6 to 22.6)
Congo	2013	52.2 (43.6 to 61.8)	25.8 (19.8 to 33.3)	19.2 (14.0 to 26.2)	1.0 (0.4 to 1.8)	6.2 (6.2 to 6.2)	155.1 (129.8 to 183.9)	76.8 (58.8 to 98.9)	57.1 (41.5 to 77.9)	2.8 (1.3 to 5.3)	18.3 (18.3 to 18.3)
Congo	2014	54.8 (45.0 to 65.2)	29.3 (22.4 to 37.8)	21.6 (15.5 to 29.5)	1.5 (0.7 to 2.8)	2.5 (2.5 to 2.5)	163.0 (134.0 to 193.9)	87.0 (66.7 to 112.3)	64.3 (46.2 to 87.7)	4.3 (2.0 to 8.3)	7.5 (7.5 to 7.5)
Congo	2015	66.3 (54.4 to 79.7)	34.1 (25.9 to 44.1)	27.1 (19.7 to 36.4)	2.2 (1.1 to 4.3)	2.9 (2.9 to 2.9)	197.2 (161.8 to 236.9)	101.4 (76.9 to 131.1)	80.6 (58.5 to 108.4)	6.7 (3.2 to 12.7)	8.6 (8.6 to 8.6)

		2018 US Dollars per capita					2018 PPP per capita				
Country	Year	Health spending	Government health spending	Out-of-pocket health spending	Prepaid private health spending	Development assistance for health	Health spending	Government health spending	Out-of-pocket health spending	Prepaid private health spending	Development assistance for health
Congo	2016	79.0 (65.4 to 94.4)	37.1 (28.4 to 48.2)	35.3 (25.1 to 47.5)	3.2 (1.6 to 6.0)	3.4 (3.4 to 3.4)	235.0 (194.5 to 280.6)	110.2 (84.5 to 143.2)	105.1 (74.6 to 141.3)	9.5 (4.6 to 17.8)	10.2 (10.2 to 10.2)
Congo	2017	63.4 (52.1 to 77.0)	24.3 (18.6 to 31.6)	32.6 (22.7 to 44.3)	2.5 (1.2 to 4.6)	4.1 (4.1 to 4.1)	188.7 (154.8 to 229.0)	72.2 (55.4 to 93.9)	96.8 (67.5 to 131.9)	7.4 (3.6 to 13.8)	12.3 (12.3 to 12.3)
Congo	2018	62.9 (51.6 to 76.0)	23.8 (18.2 to 31.0)	32.8 (23.0 to 45.0)	2.5 (1.2 to 4.8)	3.8 (3.8 to 3.8)	187.1 (153.3 to 225.9)	70.7 (54.2 to 92.0)	97.6 (68.3 to 133.7)	7.5 (3.6 to 14.1)	11.3 (11.3 to 11.4)
Congo	2019	63.2 (51.7 to 77.0)	23.3 (17.8 to 30.3)	33.5 (23.6 to 45.7)	2.5 (1.2 to 4.8)	3.9 (3.6 to 4.1)	188.0 (153.7 to 229.0)	69.3 (53.1 to 90.2)	99.6 (70.1 to 136.0)	7.6 (3.6 to 14.2)	11.5 (10.8 to 12.2)
Congo	2020	63.8 (52.2 to 77.3)	23.0 (17.6 to 29.9)	34.3 (24.3 to 46.4)	2.6 (1.2 to 4.8)	4.0 (3.6 to 4.3)	189.7 (155.1 to 229.8)	68.3 (52.3 to 88.9)	101.9 (72.2 to 138.0)	7.6 (3.7 to 14.3)	11.8 (10.8 to 12.8)
Congo	2021	64.5 (52.6 to 78.0)	22.7 (17.4 to 29.6)	35.1 (24.8 to 47.8)	2.6 (1.3 to 4.8)	4.1 (3.7 to 4.5)	191.8 (156.4 to 232.0)	67.6 (51.7 to 88.0)	104.3 (73.7 to 142.2)	7.7 (3.7 to 14.4)	12.2 (11.0 to 13.5)
Congo	2022	65.2 (53.3 to 79.3)	22.5 (17.2 to 29.3)	35.9 (25.3 to 48.8)	2.6 (1.3 to 4.9)	4.3 (3.8 to 4.8)	194.0 (158.4 to 235.8)	66.9 (51.2 to 87.2)	106.7 (75.3 to 145.2)	7.8 (3.8 to 14.4)	12.7 (11.3 to 14.3)
Congo	2023	65.9 (53.7 to 80.2)	22.3 (17.1 to 29.1)	36.6 (25.9 to 50.0)	2.6 (1.3 to 4.9)	4.4 (3.8 to 5.1)	196.1 (159.7 to 238.5)	66.3 (50.8 to 86.5)	108.8 (77.0 to 148.6)	7.8 (3.8 to 14.5)	13.2 (11.4 to 15.0)
Congo	2024	66.3 (53.8 to 80.3)	22.2 (17.0 to 29.0)	36.9 (26.0 to 50.4)	2.6 (1.3 to 4.9)	4.5 (3.9 to 5.2)	197.1 (159.9 to 238.9)	66.1 (50.6 to 86.3)	109.7 (77.4 to 150.0)	7.8 (3.8 to 14.6)	13.5 (11.6 to 15.6)
Congo	2025	66.5 (54.3 to 81.0)	22.2 (17.0 to 29.1)	37.0 (26.2 to 50.7)	2.7 (1.3 to 5.0)	4.6 (3.9 to 5.4)	197.7 (161.4 to 240.8)	66.1 (50.4 to 86.5)	110.0 (77.8 to 150.7)	7.9 (3.8 to 14.8)	13.7 (11.7 to 16.1)
Congo	2026	66.7 (54.2 to 81.1)	22.2 (17.0 to 29.1)	37.1 (26.2 to 51.0)	2.7 (1.3 to 5.0)	4.7 (3.9 to 5.5)	198.4 (161.0 to 241.2)	66.1 (50.5 to 86.6)	110.4 (77.8 to 151.5)	8.0 (3.9 to 14.9)	13.9 (11.7 to 16.4)
Congo	2027	67.5 (54.8 to 82.1)	22.6 (17.2 to 29.5)	37.5 (26.4 to 51.3)	2.7 (1.3 to 5.1)	4.8 (4.0 to 5.7)	200.7 (163.1 to 244.1)	67.1 (51.1 to 87.7)	111.4 (78.4 to 152.6)	8.1 (3.9 to 15.2)	14.2 (11.8 to 17.0)
Congo	2028	67.9 (54.9 to 82.5)	22.6 (17.2 to 29.6)	37.7 (26.5 to 51.5)	2.8 (1.3 to 5.2)	4.9 (4.1 to 5.9)	201.9 (163.2 to 245.5)	67.3 (51.3 to 88.0)	112.0 (78.8 to 153.2)	8.2 (4.0 to 15.4)	14.4 (12.1 to 17.5)
Congo	2029	68.4 (55.9 to 83.2)	22.8 (17.4 to 29.7)	37.9 (26.6 to 51.9)	2.8 (1.3 to 5.3)	4.9 (4.1 to 6.0)	203.4 (166.2 to 247.5)	67.7 (51.6 to 88.3)	112.7 (79.1 to 154.5)	8.3 (4.0 to 15.6)	14.7 (12.1 to 17.9)
Congo	2030	68.9 (55.7 to 84.1)	22.9 (17.4 to 29.8)	38.2 (26.8 to 52.7)	2.8 (1.4 to 5.3)	5.0 (4.1 to 6.2)	204.8 (165.6 to 250.2)	68.0 (51.8 to 88.7)	113.5 (79.6 to 156.8)	8.4 (4.1 to 15.9)	15.0 (12.2 to 18.4)
Congo	2031	69.4 (56.5 to 84.8)	23.0 (17.5 to 30.1)	38.4 (27.0 to 53.1)	2.9 (1.4 to 5.4)	5.1 (4.1 to 6.4)	206.5 (168.0 to 252.1)	68.5 (52.0 to 89.6)	114.3 (80.2 to 157.9)	8.5 (4.1 to 16.1)	15.2 (12.3 to 19.0)
Congo	2032	70.0 (56.6 to 85.9)	23.2 (17.6 to 30.5)	38.7 (27.1 to 53.6)	2.9 (1.4 to 5.5)	5.2 (4.2 to 6.6)	208.2 (168.2 to 255.4)	69.0 (52.3 to 90.7)	115.1 (80.6 to 159.3)	8.6 (4.1 to 16.3)	15.5 (12.4 to 19.5)
Congo	2033	70.6 (56.8 to 86.7)	23.4 (17.7 to 30.8)	39.0 (27.2 to 54.0)	2.9 (1.4 to 5.5)	5.3 (4.2 to 6.8)	209.9 (169.0 to 257.7)	69.5 (52.7 to 91.6)	115.9 (80.8 to 160.5)	8.7 (4.2 to 16.5)	15.8 (12.5 to 20.3)
Congo	2034	71.1 (57.5 to 87.1)	23.5 (17.8 to 31.1)	39.2 (27.2 to 54.4)	3.0 (1.4 to 5.6)	5.4 (4.3 to 7.0)	211.5 (171.0 to 259.0)	70.0 (53.0 to 92.6)	116.6 (81.0 to 161.6)	8.8 (4.3 to 16.6)	16.1 (12.7 to 20.7)
Congo	2035	71.6 (57.5 to 88.8)	23.7 (17.9 to 31.4)	39.4 (27.3 to 54.7)	3.0 (1.4 to 5.7)	5.5 (4.3 to 7.2)	213.0 (170.9 to 264.1)	70.4 (53.3 to 93.4)	117.3 (81.3 to 162.6)	8.9 (4.3 to 16.8)	16.4 (12.8 to 21.4)
Congo	2036	72.2 (58.2 to 89.3)	23.8 (18.0 to 31.6)	39.7 (27.5 to 55.0)	3.0 (1.5 to 5.7)	5.6 (4.3 to 7.5)	214.6 (173.2 to 265.5)	70.9 (53.7 to 93.9)	117.9 (81.6 to 163.5)	9.0 (4.4 to 17.0)	16.7 (12.9 to 22.4)

Country	Year	2018 US Dollars per capita					2018 PPP per capita				
		Health spending	Government health spending	Out-of-pocket health spending	Prepaid private health spending	Development assistance for health	Health spending	Government health spending	Out-of-pocket health spending	Prepaid private health spending	Development assistance for health
Congo	2037	72.7 (58.5 to 89.4)	24.0 (18.2 to 31.8)	39.9 (27.5 to 55.6)	3.1 (1.5 to 5.8)	5.7 (4.4 to 7.7)	216.1 (173.9 to 266.0)	71.4 (54.0 to 94.6)	118.6 (81.9 to 165.3)	9.2 (4.4 to 17.2)	17.0 (13.1 to 22.8)
Congo	2038	73.2 (59.1 to 90.2)	24.2 (18.3 to 32.0)	40.1 (27.6 to 55.9)	3.1 (1.5 to 5.8)	5.8 (4.4 to 8.0)	217.7 (175.7 to 268.1)	71.8 (54.4 to 95.3)	119.2 (82.2 to 166.3)	9.3 (4.5 to 17.4)	17.3 (13.2 to 23.7)
Congo	2039	73.7 (59.2 to 90.6)	24.3 (18.3 to 32.3)	40.3 (27.7 to 56.3)	3.2 (1.5 to 5.9)	5.9 (4.5 to 8.1)	219.1 (176.0 to 269.5)	72.3 (54.5 to 96.1)	119.8 (82.4 to 167.5)	9.4 (4.5 to 17.5)	17.6 (13.4 to 24.2)
Congo	2040	74.2 (59.9 to 91.4)	24.5 (18.5 to 32.6)	40.5 (27.9 to 56.8)	3.2 (1.6 to 5.9)	6.0 (4.6 to 8.3)	220.6 (178.1 to 271.7)	72.7 (54.9 to 97.1)	120.5 (82.9 to 168.9)	9.5 (4.6 to 17.7)	18.0 (13.7 to 24.7)
Congo	2041	74.6 (59.8 to 91.9)	24.6 (18.6 to 32.9)	40.7 (27.9 to 57.0)	3.2 (1.6 to 6.0)	6.2 (4.6 to 8.6)	222.0 (178.0 to 273.4)	73.1 (55.2 to 97.9)	120.9 (83.0 to 169.6)	9.6 (4.7 to 17.8)	18.3 (13.8 to 25.5)
Congo	2042	75.0 (60.1 to 92.7)	24.7 (18.6 to 33.2)	40.8 (28.0 to 57.5)	3.3 (1.6 to 6.0)	6.3 (4.7 to 9.1)	223.2 (178.8 to 275.8)	73.5 (55.3 to 98.7)	121.3 (83.2 to 171.1)	9.7 (4.7 to 18.0)	18.7 (14.0 to 26.9)
Congo	2043	75.5 (60.4 to 93.3)	24.8 (18.7 to 33.5)	41.0 (28.1 to 57.9)	3.3 (1.6 to 6.1)	6.4 (4.8 to 9.4)	224.5 (179.5 to 277.5)	73.8 (55.5 to 99.6)	121.8 (83.5 to 172.2)	9.8 (4.8 to 18.1)	19.0 (14.1 to 27.8)
Congo	2044	76.0 (60.9 to 94.9)	25.0 (18.7 to 33.8)	41.1 (28.2 to 58.3)	3.3 (1.6 to 6.1)	6.5 (4.8 to 9.5)	225.9 (181.2 to 282.3)	74.2 (55.8 to 100.5)	122.3 (83.9 to 173.2)	9.9 (4.8 to 18.3)	19.5 (14.3 to 28.3)
Congo	2045	76.5 (60.9 to 94.5)	25.1 (18.8 to 34.1)	41.3 (28.3 to 58.6)	3.4 (1.6 to 6.2)	6.7 (4.8 to 10.0)	227.4 (181.2 to 281.0)	74.7 (55.9 to 101.4)	122.9 (84.2 to 174.3)	10.0 (4.9 to 18.5)	19.9 (14.3 to 29.8)
Congo	2046	77.0 (61.8 to 95.4)	25.3 (18.9 to 34.4)	41.5 (28.5 to 59.0)	3.4 (1.7 to 6.3)	6.8 (4.9 to 10.4)	229.1 (183.7 to 283.6)	75.3 (56.1 to 102.2)	123.4 (84.6 to 175.4)	10.1 (4.9 to 18.7)	20.3 (14.7 to 30.8)
Congo	2047	77.7 (62.3 to 96.5)	25.5 (18.9 to 34.6)	41.7 (28.6 to 59.3)	3.5 (1.7 to 6.3)	7.0 (5.0 to 10.9)	231.0 (185.3 to 287.0)	75.9 (56.3 to 103.0)	124.1 (85.1 to 176.2)	10.3 (5.0 to 18.9)	20.7 (14.9 to 32.3)
Congo	2048	78.3 (62.6 to 97.2)	25.7 (19.0 to 35.1)	42.0 (28.8 to 59.5)	3.5 (1.7 to 6.5)	7.1 (5.1 to 11.2)	233.0 (186.1 to 289.1)	76.6 (56.6 to 104.4)	124.8 (85.7 to 177.0)	10.4 (5.1 to 19.2)	21.2 (15.2 to 33.4)
Congo	2049	79.0 (63.5 to 98.2)	26.0 (19.2 to 35.5)	42.2 (29.0 to 60.1)	3.5 (1.7 to 6.6)	7.3 (5.2 to 11.7)	235.1 (188.8 to 292.1)	77.3 (57.0 to 105.5)	125.6 (86.2 to 178.7)	10.5 (5.1 to 19.6)	21.7 (15.5 to 34.9)
Congo	2050	79.8 (63.8 to 98.9)	26.2 (19.3 to 35.9)	42.5 (29.2 to 60.5)	3.6 (1.7 to 6.7)	7.5 (5.3 to 12.2)	237.3 (189.7 to 294.0)	78.0 (57.5 to 106.8)	126.4 (86.8 to 180.0)	10.7 (5.2 to 19.9)	22.2 (15.8 to 36.4)
Costa Rica	1995	407.2 (371.6 to 445.6)	254.7 (228.4 to 281.0)	144.1 (121.9 to 169.5)	6.1 (2.9 to 11.3)	2.4 (2.4 to 2.4)	608.5 (555.2 to 665.9)	380.6 (341.2 to 419.9)	215.3 (182.2 to 253.2)	9.2 (4.3 to 17.0)	3.5 (3.5 to 3.5)
Costa Rica	1996	406.3 (375.8 to 438.2)	254.6 (233.9 to 276.1)	142.9 (123.2 to 165.0)	6.1 (3.0 to 11.2)	2.6 (2.6 to 2.6)	607.1 (561.6 to 654.7)	380.5 (349.5 to 412.5)	213.6 (184.0 to 246.5)	9.2 (4.4 to 16.8)	3.8 (3.8 to 3.8)
Costa Rica	1997	419.5 (392.1 to 448.6)	266.2 (247.6 to 286.2)	143.7 (126.0 to 164.4)	6.2 (3.0 to 11.3)	3.3 (3.3 to 3.3)	626.8 (586.0 to 670.3)	397.8 (370.1 to 427.6)	214.7 (188.2 to 245.7)	9.3 (4.5 to 16.9)	5.0 (5.0 to 5.0)
Costa Rica	1998	452.7 (426.5 to 482.2)	294.3 (275.6 to 313.9)	147.7 (130.1 to 166.8)	6.4 (3.3 to 11.7)	4.3 (4.3 to 4.3)	676.4 (637.4 to 720.6)	439.8 (411.8 to 469.0)	220.7 (194.4 to 249.2)	9.5 (4.9 to 17.6)	6.4 (6.4 to 6.4)
Costa Rica	1999	473.2 (444.8 to 503.9)	310.7 (291.3 to 331.4)	150.8 (132.9 to 169.9)	6.5 (3.3 to 11.5)	5.2 (5.2 to 5.2)	707.1 (664.7 to 753.0)	464.2 (435.2 to 495.2)	225.4 (198.5 to 253.9)	9.8 (5.0 to 17.2)	7.7 (7.7 to 7.7)
Costa Rica	2000	493.8 (464.0 to 523.0)	324.7 (304.2 to 346.9)	155.2 (137.2 to 175.6)	6.8 (3.4 to 11.9)	7.1 (7.1 to 7.1)	737.9 (693.4 to 781.6)	485.2 (454.6 to 518.4)	231.9 (205.0 to 262.4)	10.2 (5.0 to 17.7)	10.6 (10.6 to 10.6)
Costa Rica	2001	502.0 (473.7 to 530.9)	335.3 (314.4 to 356.2)	157.8 (140.1 to 176.8)	6.2 (3.2 to 11.0)	2.7 (2.7 to 2.7)	750.1 (707.8 to 793.4)	501.0 (469.7 to 532.3)	235.7 (209.3 to 264.2)	9.3 (4.8 to 16.4)	4.1 (4.1 to 4.1)

Country	Year	2018 US Dollars per capita					2018 PPP per capita				
		Health spending	Government health spending	Out-of-pocket health spending	Prepaid private health spending	Development assistance for health	Health spending	Government health spending	Out-of-pocket health spending	Prepaid private health spending	Development assistance for health
Costa Rica	2002	526.6 (499.5 to 556.2)	351.6 (330.9 to 371.9)	164.6 (146.4 to 183.8)	7.2 (3.7 to 12.7)	3.2 (3.2 to 3.2)	786.9 (746.4 to 831.2)	525.4 (494.4 to 555.7)	246.0 (218.7 to 274.6)	10.7 (5.5 to 19.0)	4.8 (4.8 to 4.8)
Costa Rica	2003	549.0 (521.3 to 581.0)	365.5 (344.6 to 386.5)	172.1 (153.7 to 192.0)	8.2 (4.2 to 14.2)	3.3 (3.3 to 3.3)	820.4 (779.0 to 868.1)	546.2 (514.9 to 577.5)	257.1 (229.7 to 286.9)	12.2 (6.3 to 21.2)	4.9 (4.9 to 4.9)
Costa Rica	2004	558.9 (529.1 to 589.2)	371.7 (349.2 to 393.4)	177.0 (158.3 to 198.7)	8.2 (4.3 to 14.1)	2.0 (2.0 to 2.0)	835.1 (790.7 to 880.5)	555.5 (521.8 to 587.8)	264.4 (236.5 to 297.0)	12.2 (6.4 to 21.1)	3.0 (3.0 to 3.0)
Costa Rica	2005	578.3 (547.7 to 611.3)	382.5 (360.4 to 405.2)	184.5 (165.0 to 206.8)	10.3 (5.4 to 18.1)	1.0 (1.0 to 1.0)	864.2 (818.5 to 913.4)	571.5 (538.6 to 605.5)	275.7 (246.5 to 309.0)	15.5 (8.1 to 27.0)	1.5 (1.5 to 1.5)
Costa Rica	2006	625.4 (592.5 to 660.2)	417.2 (394.5 to 442.8)	194.7 (173.7 to 218.5)	11.8 (6.4 to 20.3)	1.7 (1.7 to 1.7)	934.5 (885.3 to 986.5)	623.4 (589.5 to 661.7)	290.9 (259.5 to 326.6)	17.6 (9.5 to 30.4)	2.6 (2.6 to 2.6)
Costa Rica	2007	683.6 (647.8 to 722.4)	462.9 (437.6 to 488.7)	206.1 (183.8 to 230.6)	12.9 (7.0 to 22.2)	1.7 (1.7 to 1.7)	1021.5 (968.0 to 1079.4)	691.7 (653.9 to 730.2)	307.9 (274.6 to 344.6)	19.3 (10.5 to 33.2)	2.6 (2.6 to 2.6)
Costa Rica	2008	737.8 (700.7 to 774.5)	509.2 (480.7 to 534.5)	212.7 (190.3 to 238.4)	13.3 (7.3 to 22.8)	2.5 (2.5 to 2.5)	1102.5 (1047.1 to 1157.4)	761.0 (718.3 to 798.7)	317.9 (284.3 to 356.2)	19.9 (10.9 to 34.0)	3.7 (3.7 to 3.7)
Costa Rica	2009	767.7 (731.4 to 802.7)	542.8 (513.6 to 568.8)	210.1 (187.6 to 233.3)	12.9 (7.1 to 22.5)	1.8 (1.8 to 1.8)	1147.2 (1093.0 to 1199.4)	811.1 (767.5 to 849.9)	314.0 (280.3 to 348.6)	19.3 (10.6 to 33.6)	2.7 (2.7 to 2.7)
Costa Rica	2010	813.8 (776.2 to 849.4)	587.0 (557.1 to 615.4)	212.9 (191.7 to 236.6)	12.8 (7.1 to 21.7)	1.0 (1.0 to 1.0)	1216.0 (1159.8 to 1269.3)	877.2 (832.4 to 919.5)	318.1 (286.4 to 353.6)	19.2 (10.6 to 32.4)	1.5 (1.5 to 1.5)
Costa Rica	2011	839.2 (804.0 to 878.2)	610.7 (582.1 to 641.5)	214.9 (192.7 to 238.4)	13.2 (7.2 to 22.7)	0.4 (0.4 to 0.4)	1254.0 (1201.4 to 1312.3)	912.6 (869.9 to 958.5)	321.1 (288.0 to 356.3)	19.8 (10.8 to 33.9)	0.6 (0.6 to 0.6)
Costa Rica	2012	855.4 (821.1 to 894.2)	627.5 (598.7 to 657.0)	212.8 (192.0 to 236.9)	14.9 (8.1 to 26.6)	0.3 (0.3 to 0.3)	1278.3 (1227.0 to 1336.2)	937.6 (894.7 to 981.8)	317.9 (286.9 to 354.1)	22.3 (12.1 to 39.7)	0.4 (0.4 to 0.4)
Costa Rica	2013	861.9 (825.9 to 902.4)	634.2 (605.4 to 664.9)	209.6 (188.9 to 233.2)	17.8 (9.7 to 30.6)	0.4 (0.4 to 0.4)	1287.9 (1234.2 to 1348.5)	947.6 (904.7 to 993.6)	313.2 (282.2 to 348.5)	26.6 (14.5 to 45.7)	0.5 (0.5 to 0.5)
Costa Rica	2014	880.2 (839.6 to 920.3)	646.9 (616.0 to 679.5)	211.4 (188.9 to 236.4)	21.4 (11.9 to 37.2)	0.4 (0.4 to 0.4)	1315.2 (1254.7 to 1375.2)	966.7 (920.4 to 1015.4)	315.9 (282.3 to 353.2)	32.0 (17.8 to 55.7)	0.6 (0.6 to 0.6)
Costa Rica	2015	904.5 (859.9 to 951.6)	670.0 (634.3 to 707.1)	210.1 (184.6 to 239.2)	23.9 (13.1 to 41.0)	0.5 (0.5 to 0.5)	1351.6 (1285.0 to 1421.9)	1001.2 (947.8 to 1056.6)	313.9 (275.9 to 357.4)	35.7 (19.6 to 61.3)	0.8 (0.8 to 0.8)
Costa Rica	2016	947.8 (890.5 to 1002.2)	689.4 (644.4 to 736.3)	209.4 (180.4 to 241.8)	25.7 (13.8 to 45.2)	23.3 (23.3 to 23.3)	1416.2 (1330.7 to 1497.6)	1030.1 (962.9 to 1100.2)	312.9 (269.6 to 361.4)	38.4 (20.6 to 67.6)	34.8 (34.8 to 34.8)
Costa Rica	2017	968.6 (909.7 to 1030.9)	729.8 (681.6 to 779.2)	212.0 (181.8 to 244.7)	26.3 (14.3 to 46.5)	0.4 (0.4 to 0.4)	1447.4 (1359.4 to 1540.5)	1090.6 (1018.5 to 1164.4)	316.8 (271.7 to 365.7)	39.3 (21.3 to 69.5)	0.6 (0.6 to 0.6)
Costa Rica	2018	989.1 (927.3 to 1049.2)	746.8 (696.8 to 798.1)	215.0 (184.7 to 248.4)	27.0 (14.6 to 47.5)	0.4 (0.4 to 0.4)	1478.1 (1385.7 to 1567.8)	1115.9 (1041.2 to 1192.6)	321.3 (275.9 to 371.2)	40.3 (21.8 to 71.0)	0.6 (0.6 to 0.6)
Costa Rica	2019	1010.4 (950.2 to 1077.5)	764.5 (712.5 to 818.9)	217.9 (187.1 to 251.5)	27.6 (14.9 to 48.5)	0.4 (0.4 to 0.4)	1509.8 (1419.8 to 1610.1)	1142.4 (1064.7 to 1223.7)	325.6 (279.7 to 375.8)	41.2 (22.2 to 72.5)	0.6 (0.5 to 0.6)
Costa Rica	2020	1032.0 (966.9 to 1098.2)	782.8 (730.4 to 839.2)	220.6 (189.4 to 254.4)	28.1 (15.1 to 49.6)	0.4 (0.4 to 0.4)	1542.1 (1444.8 to 1641.1)	1169.8 (1091.4 to 1253.9)	329.7 (283.0 to 380.2)	42.1 (22.6 to 74.1)	0.6 (0.5 to 0.6)
Costa Rica	2021	1053.0 (988.6 to 1125.2)	800.7 (744.0 to 860.1)	223.2 (191.6 to 257.5)	28.7 (15.4 to 50.6)	0.4 (0.4 to 0.4)	1573.5 (1477.3 to 1681.3)	1196.4 (1111.8 to 1285.3)	333.5 (286.3 to 384.8)	42.9 (23.1 to 75.7)	0.6 (0.5 to 0.7)
Costa Rica	2022	1075.5 (1004.9 to 1146.6)	820.0 (761.5 to 881.4)	225.8 (193.8 to 260.4)	29.3 (15.7 to 51.7)	0.4 (0.4 to 0.5)	1607.0 (1501.7 to 1713.3)	1225.3 (1137.9 to 1317.1)	337.4 (289.6 to 389.1)	43.8 (23.5 to 77.3)	0.6 (0.5 to 0.7)

		2018 US Dollars per capita					2018 PPP per capita				
Country	Year	Health spending	Government health spending	Out-of-pocket health spending	Prepaid private health spending	Development assistance for health	Health spending	Government health spending	Out-of-pocket health spending	Prepaid private health spending	Development assistance for health
Costa Rica	2023	1092.2 (1023.2 to 1168.3)	833.8 (775.5 to 897.1)	228.1 (195.7 to 263.1)	29.8 (16.0 to 52.7)	0.4 (0.4 to 0.5)	1632.0 (1528.9 to 1745.8)	1245.9 (1158.8 to 1340.6)	340.9 (292.5 to 393.2)	44.6 (24.0 to 78.8)	0.6 (0.5 to 0.7)
Costa Rica	2024	1109.5 (1037.2 to 1182.4)	848.1 (788.8 to 913.0)	230.5 (198.1 to 265.9)	30.4 (16.3 to 53.6)	0.4 (0.4 to 0.5)	1657.9 (1549.9 to 1766.9)	1267.4 (1178.8 to 1364.3)	344.5 (296.0 to 397.4)	45.4 (24.4 to 80.1)	0.6 (0.6 to 0.7)
Costa Rica	2025	1127.0 (1055.8 to 1204.7)	862.7 (802.6 to 928.4)	232.9 (199.9 to 269.0)	30.9 (16.6 to 54.5)	0.4 (0.4 to 0.5)	1684.1 (1577.7 to 1800.2)	1289.2 (1199.3 to 1387.4)	348.0 (298.8 to 401.9)	46.2 (24.9 to 81.4)	0.7 (0.6 to 0.8)
Costa Rica	2026	1144.5 (1069.5 to 1221.0)	877.4 (816.4 to 943.5)	235.2 (201.7 to 271.3)	31.5 (16.9 to 55.4)	0.5 (0.4 to 0.5)	1710.3 (1598.2 to 1824.5)	1311.1 (1219.9 to 1409.9)	351.5 (301.4 to 405.4)	47.0 (25.3 to 82.7)	0.7 (0.6 to 0.8)
Costa Rica	2027	1162.3 (1088.6 to 1244.1)	892.2 (829.9 to 959.7)	237.5 (203.6 to 274.2)	32.0 (17.2 to 56.3)	0.5 (0.4 to 0.6)	1736.8 (1626.7 to 1859.0)	1333.2 (1240.0 to 1434.1)	355.0 (304.2 to 409.7)	47.9 (25.7 to 84.2)	0.7 (0.6 to 0.8)
Costa Rica	2028	1179.8 (1103.4 to 1259.7)	906.9 (843.2 to 975.8)	239.8 (205.2 to 276.6)	32.6 (17.4 to 57.2)	0.5 (0.4 to 0.6)	1762.9 (1648.9 to 1882.4)	1355.2 (1260.0 to 1458.2)	358.3 (306.7 to 413.4)	48.7 (26.1 to 85.5)	0.7 (0.6 to 0.9)
Costa Rica	2029	1197.9 (1121.8 to 1282.5)	922.2 (856.9 to 995.0)	242.0 (207.2 to 279.0)	33.2 (17.8 to 58.2)	0.5 (0.4 to 0.6)	1790.0 (1676.3 to 1916.4)	1378.1 (1280.4 to 1486.9)	361.7 (309.7 to 417.0)	49.5 (26.5 to 87.0)	0.7 (0.6 to 0.9)
Costa Rica	2030	1216.3 (1136.5 to 1299.1)	937.7 (871.1 to 1013.9)	244.3 (209.4 to 281.3)	33.7 (18.0 to 59.2)	0.5 (0.4 to 0.6)	1817.5 (1698.3 to 1941.2)	1401.3 (1301.7 to 1515.1)	365.1 (312.9 to 420.4)	50.4 (27.0 to 88.5)	0.7 (0.6 to 0.9)
Costa Rica	2031	1235.1 (1155.1 to 1323.1)	953.7 (885.4 to 1031.8)	246.6 (211.3 to 283.9)	34.3 (18.3 to 60.2)	0.5 (0.4 to 0.6)	1845.6 (1726.0 to 1977.2)	1425.0 (1323.1 to 1541.8)	368.5 (315.8 to 424.3)	51.3 (27.4 to 89.9)	0.8 (0.6 to 1.0)
Costa Rica	2032	1254.6 (1171.0 to 1339.7)	970.1 (899.2 to 1051.0)	249.0 (213.1 to 286.8)	34.9 (18.7 to 61.2)	0.5 (0.4 to 0.7)	1874.7 (1749.9 to 2001.9)	1449.6 (1343.7 to 1570.5)	372.1 (318.5 to 428.6)	52.2 (27.9 to 91.4)	0.8 (0.6 to 1.0)
Costa Rica	2033	1274.0 (1190.1 to 1368.5)	986.5 (913.0 to 1071.7)	251.4 (215.4 to 290.0)	35.5 (19.0 to 62.1)	0.5 (0.4 to 0.7)	1903.7 (1778.4 to 2044.9)	1474.1 (1364.2 to 1601.5)	375.6 (321.8 to 433.3)	53.1 (28.5 to 92.8)	0.8 (0.6 to 1.0)
Costa Rica	2034	1294.5 (1206.5 to 1386.1)	1003.8 (927.5 to 1093.2)	253.9 (217.7 to 293.2)	36.2 (19.4 to 63.2)	0.5 (0.4 to 0.7)	1934.3 (1802.9 to 2071.2)	1500.0 (1385.9 to 1633.6)	379.4 (325.2 to 438.1)	54.1 (29.0 to 94.4)	0.8 (0.6 to 1.1)
Costa Rica	2035	1315.9 (1226.9 to 1416.0)	1021.9 (943.4 to 1113.2)	256.5 (220.0 to 296.4)	36.8 (19.8 to 64.3)	0.6 (0.0 to 0.7)	1966.3 (1833.3 to 2115.9)	1527.0 (1409.8 to 1663.4)	383.4 (328.7 to 443.0)	55.1 (29.6 to 96.0)	0.8 (0.0 to 1.1)
Costa Rica	2036	1338.5 (1246.1 to 1435.2)	1041.0 (958.6 to 1134.2)	259.4 (222.1 to 300.0)	37.5 (20.2 to 65.4)	0.6 (0.0 to 0.8)	2000.1 (1862.0 to 2144.6)	1555.5 (1432.5 to 1694.8)	387.6 (331.8 to 448.2)	56.1 (30.2 to 97.7)	0.8 (0.0 to 1.2)
Costa Rica	2037	1361.8 (1268.4 to 1469.0)	1060.6 (975.5 to 1157.2)	262.3 (224.3 to 303.6)	38.3 (20.6 to 66.6)	0.6 (0.0 to 0.8)	2034.9 (1895.4 to 2195.1)	1584.8 (1457.7 to 1729.2)	392.0 (335.2 to 453.7)	57.2 (30.8 to 99.5)	0.9 (0.0 to 1.2)
Costa Rica	2038	1385.9 (1287.6 to 1489.7)	1081.0 (994.0 to 1181.6)	265.4 (227.0 to 307.5)	39.0 (21.0 to 67.9)	0.6 (0.0 to 0.9)	2071.0 (1924.0 to 2226.1)	1615.3 (1485.3 to 1765.6)	396.5 (339.2 to 459.5)	58.3 (31.4 to 101.5)	0.9 (0.0 to 1.3)
Costa Rica	2039	1409.9 (1311.0 to 1524.3)	1101.2 (1009.4 to 1206.8)	268.3 (229.6 to 311.1)	39.8 (21.4 to 69.3)	0.6 (0.0 to 0.9)	2106.8 (1959.0 to 2277.8)	1645.5 (1508.4 to 1803.3)	401.0 (343.1 to 464.9)	59.4 (32.0 to 103.5)	0.9 (0.0 to 1.3)
Costa Rica	2040	1433.6 (1328.8 to 1545.3)	1121.4 (1026.7 to 1230.1)	271.2 (232.1 to 314.5)	40.5 (21.8 to 70.5)	0.6 (0.0 to 0.9)	2142.2 (1985.7 to 2309.1)	1675.6 (1534.1 to 1838.2)	405.2 (346.9 to 469.9)	60.5 (32.6 to 105.4)	0.9 (0.0 to 1.4)
Costa Rica	2041	1458.4 (1352.2 to 1579.7)	1142.5 (1044.1 to 1254.9)	274.1 (234.7 to 318.1)	41.3 (22.2 to 71.8)	0.6 (0.0 to 0.9)	2179.3 (2020.5 to 2360.5)	1707.2 (1560.2 to 1875.2)	409.6 (350.8 to 475.4)	61.7 (33.2 to 107.3)	0.8 (0.0 to 1.4)
Costa Rica	2042	1482.4 (1370.5 to 1600.7)	1162.8 (1060.4 to 1277.0)	276.9 (237.0 to 321.7)	42.0 (22.6 to 73.1)	0.6 (0.0 to 1.0)	2215.1 (2047.9 to 2391.9)	1737.6 (1584.5 to 1908.2)	413.8 (354.1 to 480.6)	62.8 (33.8 to 109.2)	0.8 (0.0 to 1.5)
Costa Rica	2043	1506.0 (1391.3 to 1637.7)	1183.1 (1076.7 to 1303.2)	279.6 (239.4 to 325.2)	42.8 (23.0 to 74.3)	0.6 (0.0 to 1.0)	2250.5 (2079.0 to 2447.1)	1767.8 (1608.9 to 1947.4)	417.9 (357.7 to 485.9)	63.9 (34.4 to 111.1)	0.8 (0.0 to 1.5)

Country	Year	2018 US Dollars per capita					2018 PPP per capita				
		Health spending	Government health spending	Out-of-pocket health spending	Prepaid private health spending	Development assistance for health	Health spending	Government health spending	Out-of-pocket health spending	Prepaid private health spending	Development assistance for health
Costa Rica	2044	1528.7 (1407.6 to 1657.2)	1202.5 (1092.2 to 1326.8)	282.1 (241.4 to 328.7)	43.5 (23.4 to 75.5)	0.6 (0.0 to 1.1)	2284.3 (2103.4 to 2476.3)	1796.9 (1632.0 to 1982.6)	421.6 (360.7 to 491.2)	65.0 (35.0 to 112.8)	0.8 (0.0 to 1.6)
Costa Rica	2045	1551.1 (1430.8 to 1692.3)	1221.9 (1107.4 to 1351.4)	284.5 (243.2 to 331.4)	44.2 (23.8 to 76.8)	0.6 (0.0 to 1.1)	2317.9 (2138.0 to 2528.7)	1825.8 (1654.8 to 2019.4)	425.1 (363.5 to 495.2)	66.1 (35.6 to 114.8)	0.8 (0.0 to 1.7)
Costa Rica	2046	1573.8 (1446.1 to 1712.7)	1241.3 (1122.7 to 1374.2)	287.0 (245.0 to 334.3)	45.0 (24.2 to 78.2)	0.6 (0.0 to 1.2)	2351.7 (2160.9 to 2559.3)	1854.9 (1677.7 to 2053.5)	428.8 (366.2 to 499.6)	67.2 (36.2 to 116.8)	0.8 (0.0 to 1.8)
Costa Rica	2047	1596.2 (1466.3 to 1747.5)	1260.6 (1136.5 to 1396.8)	289.4 (246.8 to 337.3)	45.7 (24.6 to 79.4)	0.6 (0.0 to 1.2)	2385.2 (2191.0 to 2611.2)	1883.7 (1698.2 to 2087.3)	432.4 (368.8 to 504.1)	68.3 (36.7 to 118.7)	0.8 (0.0 to 1.8)
Costa Rica	2048	1617.7 (1479.3 to 1768.0)	1279.2 (1150.5 to 1420.4)	291.6 (248.5 to 340.1)	46.4 (25.0 to 80.6)	0.6 (0.0 to 1.3)	2417.3 (2210.4 to 2641.9)	1911.5 (1719.1 to 2122.5)	435.7 (371.3 to 508.3)	69.3 (37.3 to 120.4)	0.8 (0.0 to 2.0)
Costa Rica	2049	1638.9 (1499.4 to 1797.9)	1297.5 (1165.4 to 1443.9)	293.7 (250.4 to 343.0)	47.1 (25.3 to 81.7)	0.6 (0.0 to 1.4)	2448.9 (2240.6 to 2686.6)	1938.8 (1741.4 to 2157.7)	438.9 (374.2 to 512.5)	70.4 (37.9 to 122.1)	0.9 (0.0 to 2.0)
Costa Rica	2050	1658.8 (1511.0 to 1817.9)	1314.8 (1179.0 to 1466.0)	295.6 (252.1 to 345.8)	47.8 (25.7 to 82.8)	0.6 (0.0 to 1.4)	2478.7 (2257.9 to 2716.5)	1964.7 (1761.7 to 2190.6)	441.7 (376.7 to 516.7)	71.4 (38.4 to 123.7)	0.9 (0.0 to 2.1)
Cote d'Ivoire	1995	80.0 (65.4 to 96.9)	11.1 (8.3 to 14.5)	51.7 (39.1 to 66.8)	9.6 (4.9 to 17.4)	7.6 (7.6 to 7.6)	185.1 (151.4 to 224.2)	25.6 (19.2 to 33.5)	119.6 (90.5 to 154.4)	22.2 (11.3 to 40.2)	17.6 (17.6 to 17.6)
Cote d'Ivoire	1996	82.0 (66.3 to 100.4)	12.6 (9.5 to 16.3)	54.6 (41.5 to 69.5)	10.3 (5.2 to 18.3)	4.5 (4.5 to 4.5)	189.6 (153.4 to 232.2)	29.2 (21.9 to 37.7)	126.2 (96.0 to 160.8)	23.8 (12.1 to 42.3)	10.5 (10.5 to 10.5)
Cote d'Ivoire	1997	82.6 (66.6 to 100.9)	13.4 (10.1 to 17.6)	56.2 (42.8 to 72.3)	10.5 (5.4 to 18.6)	2.4 (2.4 to 2.4)	191.1 (154.2 to 233.4)	31.0 (23.3 to 40.7)	130.0 (98.9 to 167.4)	24.4 (12.5 to 43.0)	5.7 (5.7 to 5.7)
Cote d'Ivoire	1998	82.5 (67.1 to 100.2)	13.4 (10.1 to 17.5)	55.6 (42.6 to 71.4)	10.6 (5.5 to 19.3)	2.9 (2.9 to 2.9)	190.9 (155.2 to 231.8)	30.9 (23.3 to 40.6)	128.7 (98.5 to 165.2)	24.6 (12.8 to 44.7)	6.7 (6.7 to 6.7)
Cote d'Ivoire	1999	75.8 (61.6 to 93.9)	12.5 (9.5 to 16.5)	50.0 (38.1 to 64.0)	10.6 (5.5 to 19.5)	2.7 (2.7 to 2.7)	175.2 (142.5 to 217.3)	28.9 (21.9 to 38.1)	115.6 (88.1 to 148.1)	24.6 (12.6 to 45.0)	6.2 (6.2 to 6.2)
Cote d'Ivoire	2000	63.5 (51.1 to 78.6)	10.5 (8.1 to 13.9)	41.7 (31.7 to 53.6)	10.3 (5.3 to 18.7)	1.0 (1.0 to 1.0)	147.0 (118.3 to 181.8)	24.4 (18.8 to 32.1)	96.6 (73.4 to 124.0)	23.8 (12.3 to 43.4)	2.3 (2.3 to 2.3)
Cote d'Ivoire	2001	56.8 (46.1 to 69.9)	9.7 (7.4 to 12.8)	35.9 (27.5 to 45.4)	10.1 (5.2 to 18.4)	1.1 (1.1 to 1.1)	131.5 (106.6 to 161.7)	22.4 (17.2 to 29.7)	83.1 (63.6 to 105.1)	23.5 (12.1 to 42.6)	2.6 (2.6 to 2.6)
Cote d'Ivoire	2002	54.0 (44.5 to 65.9)	9.2 (7.0 to 11.9)	32.1 (24.6 to 40.9)	10.0 (5.1 to 18.4)	2.8 (2.8 to 2.8)	125.0 (102.8 to 152.4)	21.2 (16.2 to 27.6)	74.4 (57.0 to 94.6)	23.1 (11.8 to 42.7)	6.4 (6.4 to 6.4)
Cote d'Ivoire	2003	52.3 (42.9 to 64.0)	8.7 (6.6 to 11.4)	31.6 (24.1 to 39.7)	9.7 (5.0 to 17.9)	2.4 (2.4 to 2.4)	120.9 (99.2 to 148.1)	20.0 (15.4 to 26.3)	73.0 (55.8 to 91.9)	22.3 (11.5 to 41.4)	5.5 (5.5 to 5.5)
Cote d'Ivoire	2004	54.9 (45.2 to 66.8)	8.6 (6.6 to 11.3)	33.5 (25.8 to 42.5)	9.7 (5.0 to 18.3)	3.0 (3.0 to 3.0)	127.0 (104.6 to 154.5)	20.0 (15.3 to 26.1)	77.5 (59.6 to 98.4)	22.5 (11.5 to 42.3)	7.0 (7.0 to 7.0)
Cote d'Ivoire	2005	57.4 (46.7 to 70.2)	8.6 (6.6 to 11.2)	36.1 (27.6 to 46.2)	9.7 (4.9 to 17.8)	3.0 (3.0 to 3.0)	132.8 (108.0 to 162.4)	20.0 (15.3 to 25.8)	83.5 (63.9 to 106.8)	22.5 (11.2 to 41.2)	6.9 (6.9 to 6.9)
Cote d'Ivoire	2006	62.6 (51.2 to 76.6)	8.9 (6.8 to 11.5)	39.3 (29.9 to 50.8)	10.0 (4.9 to 18.1)	4.4 (4.4 to 4.4)	144.7 (118.4 to 177.3)	20.6 (15.8 to 26.5)	90.8 (69.2 to 117.5)	23.2 (11.3 to 41.9)	10.1 (10.1 to 10.1)
Cote d'Ivoire	2007	64.9 (52.9 to 80.1)	9.2 (7.0 to 11.9)	41.4 (31.4 to 53.5)	10.2 (5.0 to 18.1)	4.1 (4.1 to 4.1)	150.2 (122.4 to 185.2)	21.3 (16.3 to 27.5)	95.8 (72.6 to 123.7)	23.6 (11.6 to 42.0)	9.6 (9.6 to 9.6)
Cote d'Ivoire	2008	70.1 (58.0 to 85.8)	9.7 (7.5 to 12.5)	42.2 (32.4 to 54.5)	10.4 (5.3 to 18.7)	7.8 (7.8 to 7.8)	162.3 (134.3 to 198.6)	22.4 (17.2 to 29.0)	97.6 (74.9 to 126.0)	24.1 (12.2 to 43.3)	18.1 (18.1 to 18.1)

		2018 US Dollars per capita					2018 PPP per capita				
Country	Year	Health spending	Government health spending	Out-of-pocket health spending	Prepaid private health spending	Development assistance for health	Health spending	Government health spending	Out-of-pocket health spending	Prepaid private health spending	Development assistance for health
Cote d'Ivoire	2009	69.2 (57.1 to 85.3)	10.2 (7.8 to 13.2)	42.5 (32.5 to 54.9)	10.6 (5.2 to 19.5)	5.9 (5.9 to 5.9)	160.1 (132.0 to 197.4)	23.7 (18.0 to 30.6)	98.3 (75.2 to 126.9)	24.5 (12.1 to 45.2)	13.6 (13.6 to 13.6)
Cote d'Ivoire	2010	72.3 (60.4 to 87.7)	10.5 (8.0 to 13.5)	41.8 (31.6 to 54.0)	10.4 (5.1 to 18.7)	9.6 (9.6 to 9.6)	167.2 (139.7 to 203.0)	24.2 (18.5 to 31.1)	96.7 (73.2 to 125.0)	24.1 (11.9 to 43.4)	22.3 (22.3 to 22.3)
Cote d'Ivoire	2011	64.2 (53.1 to 78.3)	10.3 (7.8 to 13.2)	38.9 (29.2 to 50.5)	9.4 (4.7 to 16.6)	5.6 (5.6 to 5.6)	148.5 (122.8 to 181.1)	23.7 (18.0 to 30.5)	90.1 (67.6 to 116.8)	21.8 (11.0 to 38.3)	12.9 (12.9 to 12.9)
Cote d'Ivoire	2012	67.6 (56.3 to 81.9)	12.1 (9.1 to 15.5)	39.0 (29.4 to 50.7)	10.0 (5.1 to 17.5)	6.5 (6.5 to 6.5)	156.4 (130.2 to 189.4)	28.0 (21.1 to 35.9)	90.3 (67.9 to 117.2)	23.1 (11.8 to 40.5)	15.0 (15.0 to 15.0)
Cote d'Ivoire	2013	69.4 (58.1 to 83.9)	13.5 (10.1 to 17.3)	38.0 (28.8 to 49.9)	10.1 (5.2 to 17.5)	7.8 (7.8 to 7.8)	160.5 (134.4 to 194.1)	31.2 (23.4 to 39.9)	88.0 (66.6 to 115.5)	23.4 (12.1 to 40.6)	18.0 (18.0 to 18.0)
Cote d'Ivoire	2014	71.7 (60.5 to 86.0)	14.4 (11.0 to 18.5)	36.5 (27.1 to 47.6)	10.6 (5.4 to 17.9)	10.2 (10.2 to 10.2)	165.9 (140.0 to 198.9)	33.3 (25.4 to 42.7)	84.3 (62.7 to 110.2)	24.6 (12.5 to 41.5)	23.7 (23.7 to 23.7)
Cote d'Ivoire	2015	70.4 (58.3 to 85.2)	16.5 (12.6 to 21.5)	34.2 (25.7 to 44.4)	12.9 (6.5 to 22.0)	6.8 (6.8 to 6.8)	162.9 (134.8 to 197.2)	38.3 (29.1 to 49.7)	79.2 (59.4 to 102.8)	29.8 (15.0 to 51.0)	15.7 (15.7 to 15.7)
Cote d'Ivoire	2016	76.7 (63.4 to 92.4)	18.1 (13.8 to 23.4)	33.3 (24.9 to 43.6)	14.4 (7.2 to 25.2)	11.0 (11.0 to 11.0)	177.5 (146.6 to 213.9)	41.8 (31.8 to 54.1)	77.0 (57.5 to 100.9)	33.3 (16.7 to 58.4)	25.5 (25.5 to 25.5)
Cote d'Ivoire	2017	79.5 (66.8 to 95.5)	19.2 (14.6 to 24.8)	33.9 (25.3 to 44.7)	15.1 (7.6 to 26.6)	11.3 (11.3 to 11.3)	183.9 (154.5 to 220.9)	44.4 (33.9 to 57.4)	78.4 (58.5 to 103.4)	35.0 (17.5 to 61.5)	26.1 (26.1 to 26.1)
Cote d'Ivoire	2018	79.5 (65.7 to 95.3)	19.8 (15.1 to 25.5)	34.1 (25.4 to 44.8)	15.6 (7.9 to 27.4)	10.0 (9.9 to 10.0)	183.8 (152.1 to 220.6)	45.8 (34.9 to 59.1)	78.8 (58.8 to 103.7)	36.1 (18.2 to 63.3)	23.1 (23.0 to 23.3)
Cote d'Ivoire	2019	80.5 (66.8 to 97.6)	20.3 (15.5 to 26.3)	34.2 (25.5 to 45.5)	16.0 (8.1 to 28.1)	9.9 (9.2 to 10.4)	186.2 (154.6 to 225.7)	47.0 (35.9 to 60.9)	79.2 (58.9 to 105.3)	37.1 (18.6 to 64.9)	22.8 (21.3 to 24.1)
Cote d'Ivoire	2020	81.6 (67.5 to 99.5)	20.9 (15.9 to 27.1)	34.4 (25.5 to 45.4)	16.5 (8.3 to 28.9)	9.8 (9.0 to 10.6)	188.8 (156.1 to 230.3)	48.3 (36.9 to 62.7)	79.6 (59.0 to 105.1)	38.1 (19.1 to 66.9)	22.7 (20.7 to 24.5)
Cote d'Ivoire	2021	82.7 (68.8 to 100.6)	21.4 (16.4 to 27.8)	34.6 (25.6 to 45.5)	16.9 (8.5 to 29.7)	9.8 (8.8 to 10.8)	191.4 (159.1 to 232.8)	49.6 (37.9 to 64.4)	80.0 (59.3 to 105.3)	39.2 (19.6 to 68.8)	22.6 (20.3 to 25.0)
Cote d'Ivoire	2022	83.9 (68.7 to 101.1)	22.0 (16.8 to 28.6)	34.7 (25.6 to 45.6)	17.4 (8.7 to 30.5)	9.8 (8.7 to 10.9)	194.0 (158.9 to 233.9)	50.9 (38.9 to 66.1)	80.2 (59.3 to 105.4)	40.3 (20.2 to 70.6)	22.7 (20.1 to 25.3)
Cote d'Ivoire	2023	85.1 (69.9 to 104.7)	22.6 (17.3 to 29.3)	34.7 (25.7 to 46.0)	17.9 (9.0 to 31.3)	9.8 (8.5 to 11.2)	196.8 (161.6 to 242.2)	52.3 (39.9 to 67.8)	80.4 (59.5 to 106.4)	41.4 (20.8 to 72.5)	22.8 (19.8 to 25.9)
Cote d'Ivoire	2024	86.3 (71.2 to 104.3)	23.2 (17.7 to 30.1)	34.8 (25.8 to 46.2)	18.4 (9.2 to 32.1)	9.9 (8.6 to 11.4)	199.6 (164.7 to 241.4)	53.6 (41.0 to 69.5)	80.6 (59.6 to 106.9)	42.5 (21.3 to 74.3)	23.0 (19.8 to 26.4)
Cote d'Ivoire	2025	87.6 (72.4 to 107.1)	23.8 (18.2 to 30.9)	34.9 (25.8 to 46.5)	18.9 (9.5 to 33.0)	10.0 (8.5 to 11.7)	202.6 (167.4 to 247.9)	55.0 (42.0 to 71.4)	80.7 (59.7 to 107.5)	43.6 (22.0 to 76.4)	23.2 (19.8 to 27.1)
Cote d'Ivoire	2026	88.9 (73.0 to 108.1)	24.4 (18.7 to 31.7)	35.0 (26.0 to 47.1)	19.4 (9.8 to 33.7)	10.1 (8.6 to 11.9)	205.7 (169.0 to 250.1)	56.5 (43.2 to 73.3)	81.0 (60.1 to 109.0)	44.8 (22.6 to 78.1)	23.5 (19.8 to 27.6)
Cote d'Ivoire	2027	90.1 (74.5 to 111.8)	25.0 (19.1 to 32.5)	35.1 (25.8 to 47.1)	19.8 (10.0 to 34.4)	10.3 (8.6 to 12.3)	208.5 (172.4 to 258.6)	57.8 (44.1 to 75.1)	81.1 (59.7 to 109.0)	45.9 (23.2 to 79.5)	23.8 (19.9 to 28.5)
Cote d'Ivoire	2028	91.1 (74.3 to 111.9)	25.4 (19.3 to 33.0)	35.0 (25.8 to 47.4)	20.2 (10.2 to 34.8)	10.5 (8.8 to 12.8)	210.8 (171.9 to 259.0)	58.7 (44.7 to 76.4)	81.0 (59.7 to 109.6)	46.7 (23.6 to 80.6)	24.4 (20.3 to 29.5)
Cote d'Ivoire	2029	92.1 (75.5 to 113.6)	25.8 (19.6 to 33.6)	35.0 (25.5 to 47.5)	20.5 (10.4 to 35.3)	10.8 (8.9 to 13.3)	213.1 (174.7 to 262.7)	59.7 (45.3 to 77.6)	81.0 (59.1 to 109.8)	47.4 (24.0 to 81.7)	24.9 (20.5 to 30.9)

		2018 US Dollars per capita					2018 PPP per capita				
Country	Year	Health spending	Government health spending	Out-of-pocket health spending	Prepaid private health spending	Development assistance for health	Health spending	Government health spending	Out-of-pocket health spending	Prepaid private health spending	Development assistance for health
Cote d'Ivoire	2030	93.2 (76.4 to 112.6)	26.2 (19.9 to 34.2)	35.1 (25.5 to 47.5)	20.9 (10.6 to 35.9)	11.0 (9.0 to 13.5)	215.5 (176.7 to 260.4)	60.7 (46.0 to 79.0)	81.1 (59.0 to 110.0)	48.3 (24.4 to 83.1)	25.4 (20.8 to 31.3)
Cote d'Ivoire	2031	94.2 (77.0 to 114.5)	26.6 (20.2 to 34.8)	35.1 (25.3 to 47.6)	21.2 (10.7 to 36.6)	11.2 (9.1 to 14.1)	218.0 (178.2 to 265.0)	61.6 (46.7 to 80.5)	81.3 (58.6 to 110.2)	49.1 (24.8 to 84.7)	26.0 (21.1 to 32.7)
Cote d'Ivoire	2032	95.4 (78.3 to 118.3)	27.1 (20.5 to 35.4)	35.2 (25.3 to 47.9)	21.6 (10.9 to 37.3)	11.5 (9.3 to 14.5)	220.7 (181.1 to 273.8)	62.6 (47.5 to 82.0)	81.5 (58.5 to 110.8)	49.9 (25.3 to 86.3)	26.6 (21.5 to 33.5)
Cote d'Ivoire	2033	96.6 (78.5 to 118.2)	27.5 (20.9 to 36.0)	35.4 (25.1 to 48.4)	22.0 (11.1 to 38.1)	11.8 (9.3 to 15.1)	223.5 (181.6 to 273.4)	63.7 (48.3 to 83.3)	81.8 (58.2 to 111.9)	50.8 (25.7 to 88.1)	27.2 (21.5 to 35.0)
Cote d'Ivoire	2034	97.9 (79.6 to 120.9)	28.0 (21.2 to 36.7)	35.5 (25.1 to 48.5)	22.4 (11.3 to 38.7)	12.1 (9.5 to 15.5)	226.5 (184.2 to 279.6)	64.7 (49.1 to 84.9)	82.2 (58.1 to 112.3)	51.7 (26.1 to 89.6)	27.9 (22.0 to 36.0)
Cote d'Ivoire	2035	99.3 (80.3 to 122.7)	28.5 (21.6 to 37.3)	35.7 (25.1 to 48.9)	22.8 (11.5 to 39.4)	12.4 (9.5 to 16.3)	229.8 (185.7 to 283.9)	65.9 (50.0 to 86.4)	82.6 (58.2 to 113.2)	52.7 (26.5 to 91.2)	28.6 (22.1 to 37.7)
Cote d'Ivoire	2036	100.8 (82.3 to 125.6)	29.0 (22.0 to 38.0)	36.0 (25.2 to 49.4)	23.2 (11.7 to 40.3)	12.7 (9.7 to 17.0)	233.1 (190.3 to 290.5)	67.0 (50.9 to 88.0)	83.2 (58.3 to 114.3)	53.6 (27.0 to 93.2)	29.3 (22.5 to 39.3)
Cote d'Ivoire	2037	102.2 (81.7 to 126.6)	29.5 (22.4 to 38.7)	36.2 (25.4 to 49.9)	23.6 (11.9 to 41.0)	12.9 (9.9 to 17.3)	236.5 (189.1 to 292.9)	68.1 (51.8 to 89.5)	83.8 (58.7 to 115.5)	54.6 (27.5 to 94.9)	29.9 (22.8 to 40.1)
Cote d'Ivoire	2038	103.8 (84.4 to 129.9)	30.0 (22.8 to 39.4)	36.5 (25.5 to 50.6)	24.0 (12.1 to 41.8)	13.3 (10.1 to 18.4)	240.1 (195.3 to 300.6)	69.3 (52.6 to 91.2)	84.5 (58.9 to 117.0)	55.6 (27.9 to 96.6)	30.7 (23.3 to 42.5)
Cote d'Ivoire	2039	105.4 (86.4 to 131.2)	30.5 (23.1 to 40.1)	36.9 (25.4 to 51.2)	24.5 (12.3 to 42.4)	13.5 (10.1 to 18.6)	243.8 (199.9 to 303.4)	70.5 (53.5 to 92.9)	85.3 (58.8 to 118.5)	56.7 (28.4 to 98.2)	31.3 (23.4 to 43.1)
Cote d'Ivoire	2040	107.1 (86.3 to 132.7)	31.0 (23.5 to 40.9)	37.2 (25.5 to 51.9)	24.9 (12.5 to 43.2)	13.9 (10.5 to 19.3)	247.7 (199.6 to 307.1)	71.8 (54.3 to 94.5)	86.1 (59.1 to 120.1)	57.7 (28.8 to 99.9)	32.1 (24.4 to 44.7)
Cote d'Ivoire	2041	108.7 (88.0 to 135.7)	31.5 (23.8 to 41.5)	37.6 (25.7 to 52.6)	25.3 (12.7 to 44.0)	14.3 (10.7 to 20.3)	251.6 (203.7 to 314.0)	72.9 (55.1 to 96.1)	87.0 (59.6 to 121.6)	58.6 (29.3 to 101.7)	33.0 (24.7 to 47.1)
Cote d'Ivoire	2042	110.6 (89.2 to 136.0)	32.0 (24.2 to 42.3)	38.0 (26.0 to 53.2)	25.8 (12.9 to 44.8)	14.7 (10.9 to 21.2)	255.8 (206.3 to 314.6)	74.1 (55.9 to 97.9)	88.0 (60.2 to 123.2)	59.7 (29.8 to 103.6)	34.0 (25.2 to 49.0)
Cote d'Ivoire	2043	112.4 (89.3 to 140.9)	32.6 (24.6 to 43.1)	38.5 (26.3 to 54.0)	26.3 (13.1 to 45.7)	15.1 (11.1 to 21.9)	260.1 (206.5 to 326.0)	75.4 (56.8 to 99.7)	89.1 (60.9 to 124.8)	60.8 (30.3 to 105.7)	34.9 (25.6 to 50.7)
Cote d'Ivoire	2044	114.5 (92.4 to 140.7)	33.1 (25.0 to 43.9)	39.0 (26.6 to 54.7)	26.8 (13.4 to 46.6)	15.5 (11.4 to 22.7)	264.8 (213.9 to 325.5)	76.7 (57.7 to 101.5)	90.3 (61.6 to 126.5)	61.9 (30.9 to 107.8)	35.9 (26.3 to 52.6)
Cote d'Ivoire	2045	116.5 (93.3 to 145.4)	33.7 (25.4 to 44.7)	39.6 (27.0 to 55.4)	27.2 (13.6 to 47.4)	16.0 (11.5 to 23.8)	269.5 (215.9 to 336.4)	78.0 (58.7 to 103.4)	91.6 (62.4 to 128.2)	63.0 (31.5 to 109.6)	37.0 (26.7 to 55.0)
Cote d'Ivoire	2046	118.6 (96.3 to 147.1)	34.2 (25.8 to 45.5)	40.1 (27.3 to 56.1)	27.7 (13.9 to 48.1)	16.5 (11.7 to 25.2)	274.3 (222.7 to 340.2)	79.2 (59.6 to 105.2)	92.9 (63.2 to 129.9)	64.1 (32.1 to 111.4)	38.1 (27.2 to 58.4)
Cote d'Ivoire	2047	120.8 (96.5 to 151.7)	34.8 (26.1 to 46.3)	40.8 (27.7 to 56.9)	28.2 (14.1 to 48.9)	17.0 (12.1 to 27.0)	279.5 (223.3 to 350.9)	80.6 (60.5 to 107.1)	94.3 (64.2 to 131.7)	65.3 (32.7 to 113.2)	39.4 (28.1 to 62.5)
Cote d'Ivoire	2048	123.2 (99.4 to 153.2)	35.4 (26.6 to 47.2)	41.4 (28.2 to 57.7)	28.7 (14.4 to 49.8)	17.6 (12.4 to 28.5)	285.0 (229.9 to 354.4)	82.0 (61.4 to 109.1)	95.8 (65.2 to 133.5)	66.5 (33.3 to 115.2)	40.7 (28.7 to 65.9)
Cote d'Ivoire	2049	125.5 (99.8 to 157.3)	36.0 (27.0 to 48.0)	42.1 (28.6 to 58.6)	29.3 (14.7 to 50.6)	18.2 (12.8 to 29.6)	290.4 (230.9 to 363.9)	83.3 (62.4 to 111.0)	97.3 (66.1 to 135.5)	67.7 (33.9 to 117.1)	42.1 (29.7 to 68.4)
Cote d'Ivoire	2050	128.0 (101.5 to 161.3)	36.7 (27.4 to 48.9)	42.7 (29.1 to 59.6)	29.8 (14.9 to 51.5)	18.8 (13.1 to 30.9)	296.2 (234.7 to 373.1)	84.8 (63.4 to 113.2)	98.8 (67.2 to 138.0)	69.0 (34.6 to 119.2)	43.6 (30.4 to 71.6)

Country	Year	2018 US Dollars per capita					2018 PPP per capita				
		Health spending	Government health spending	Out-of-pocket health spending	Prepaid private health spending	Development assistance for health	Health spending	Government health spending	Out-of-pocket health spending	Prepaid private health spending	Development assistance for health
Croatia	1995	528.5 (483.6 to 578.8)	440.1 (402.1 to 483.9)	74.2 (58.7 to 92.4)	9.8 (4.9 to 18.1)	4.4 (4.4 to 4.4)	961.2 (879.5 to 1052.7)	800.4 (731.3 to 880.2)	134.9 (106.7 to 168.0)	17.8 (9.0 to 32.9)	8.1 (8.1 to 8.1)
Croatia	1996	564.5 (523.7 to 605.9)	471.4 (435.7 to 508.4)	77.5 (63.4 to 93.4)	10.4 (5.3 to 18.5)	5.2 (5.2 to 5.2)	1026.7 (952.6 to 1102.0)	857.3 (792.5 to 924.7)	140.9 (115.2 to 169.9)	18.9 (9.6 to 33.7)	9.5 (9.5 to 9.5)
Croatia	1997	579.9 (541.4 to 617.0)	484.1 (448.9 to 515.2)	81.1 (67.7 to 97.0)	10.8 (5.4 to 18.9)	4.0 (4.0 to 4.0)	1054.7 (984.7 to 1122.2)	880.4 (816.5 to 937.0)	147.5 (123.1 to 176.4)	19.6 (9.8 to 34.3)	7.2 (7.2 to 7.2)
Croatia	1998	624.8 (586.5 to 665.3)	525.6 (489.7 to 558.5)	85.4 (72.0 to 100.8)	11.7 (5.9 to 20.2)	2.0 (2.0 to 2.0)	1136.3 (1066.7 to 1210.0)	956.0 (890.7 to 1015.8)	155.3 (131.0 to 183.3)	21.3 (10.8 to 36.8)	3.7 (3.7 to 3.7)
Croatia	1999	647.7 (611.2 to 689.4)	544.6 (511.4 to 579.2)	89.9 (75.9 to 106.1)	12.3 (6.3 to 20.8)	0.8 (0.8 to 0.8)	1178.0 (1111.7 to 1254.0)	990.6 (930.1 to 1053.4)	163.6 (138.0 to 192.9)	22.4 (11.4 to 37.9)	1.5 (1.5 to 1.5)
Croatia	2000	689.1 (650.9 to 728.1)	575.1 (541.9 to 609.6)	98.8 (84.2 to 116.2)	13.2 (7.1 to 22.0)	1.9 (1.9 to 1.9)	1253.4 (1183.8 to 1324.4)	1046.1 (985.6 to 1108.8)	179.7 (153.2 to 211.3)	24.1 (13.0 to 40.1)	3.5 (3.5 to 3.5)
Croatia	2001	690.9 (654.8 to 729.0)	566.3 (532.9 to 601.5)	105.6 (90.6 to 124.3)	17.4 (9.6 to 28.6)	1.6 (1.6 to 1.6)	1256.6 (1191.0 to 1326.0)	1030.0 (969.3 to 1094.0)	192.1 (164.7 to 226.0)	31.6 (17.4 to 52.0)	2.9 (2.9 to 2.9)
Croatia	2002	688.4 (652.2 to 726.7)	557.0 (525.0 to 592.3)	113.0 (97.3 to 131.5)	16.6 (9.4 to 26.3)	1.8 (1.8 to 1.8)	1252.1 (1186.2 to 1321.8)	1013.1 (954.9 to 1077.2)	205.6 (177.0 to 239.2)	30.2 (17.1 to 47.8)	3.3 (3.3 to 3.3)
Croatia	2003	724.0 (684.8 to 761.3)	586.6 (552.3 to 620.5)	118.8 (102.9 to 137.1)	16.2 (9.5 to 25.5)	2.4 (2.4 to 2.4)	1316.8 (1245.5 to 1384.7)	1067.0 (1004.6 to 1128.5)	216.0 (187.1 to 249.5)	29.4 (17.2 to 46.4)	4.4 (4.4 to 4.4)
Croatia	2004	768.2 (727.4 to 807.4)	626.0 (590.3 to 661.7)	124.3 (107.0 to 143.5)	16.3 (9.9 to 25.1)	1.5 (1.5 to 1.5)	1397.2 (1323.0 to 1468.5)	1138.7 (1073.6 to 1203.6)	226.1 (194.7 to 261.1)	29.7 (17.9 to 45.7)	2.8 (2.8 to 2.8)
Croatia	2005	831.9 (790.3 to 872.7)	688.6 (652.7 to 728.5)	122.9 (105.9 to 140.3)	16.6 (10.4 to 25.7)	3.9 (3.9 to 3.9)	1513.1 (1437.5 to 1587.3)	1252.4 (1187.2 to 1325.0)	223.4 (192.6 to 255.2)	30.2 (18.9 to 46.8)	7.1 (7.1 to 7.1)
Croatia	2006	909.4 (867.3 to 951.7)	758.9 (721.0 to 797.8)	126.6 (109.7 to 144.3)	18.0 (11.5 to 27.0)	5.8 (5.8 to 5.8)	1654.0 (1577.4 to 1730.9)	1380.4 (1311.3 to 1451.0)	230.3 (199.5 to 262.4)	32.8 (20.8 to 49.1)	10.5 (10.5 to 10.5)
Croatia	2007	1000.0 (956.5 to 1043.4)	846.5 (807.2 to 885.6)	134.1 (116.4 to 152.4)	19.2 (12.3 to 28.0)	0.2 (0.2 to 0.2)	1818.9 (1739.7 to 1897.7)	1539.7 (1468.2 to 1610.7)	244.0 (211.7 to 277.2)	34.8 (22.4 to 51.0)	0.4 (0.4 to 0.4)
Croatia	2008	1060.8 (1016.7 to 1102.7)	893.3 (856.2 to 931.8)	143.6 (125.0 to 162.9)	23.9 (15.8 to 33.9)	0.0 (0.0 to 0.0)	1929.5 (1849.3 to 2005.7)	1624.8 (1557.3 to 1694.9)	261.1 (227.4 to 296.3)	43.5 (28.8 to 61.6)	0.0 (0.0 to 0.0)
Croatia	2009	1031.4 (991.3 to 1073.5)	860.1 (824.8 to 897.4)	140.3 (123.0 to 158.2)	31.1 (21.3 to 43.7)	0.0 (0.0 to 0.0)	1876.0 (1803.0 to 1952.6)	1564.4 (1500.2 to 1632.3)	255.2 (223.8 to 287.8)	56.5 (38.7 to 79.5)	0.0 (0.0 to 0.0)
Croatia	2010	1006.1 (966.8 to 1046.9)	835.0 (799.0 to 871.4)	134.8 (119.4 to 151.2)	36.3 (26.2 to 50.2)	0.0 (0.0 to 0.0)	1830.0 (1758.4 to 1904.2)	1518.7 (1453.2 to 1584.9)	245.2 (217.1 to 275.1)	66.1 (47.7 to 91.3)	0.0 (0.0 to 0.0)
Croatia	2011	991.0 (949.8 to 1033.5)	816.4 (781.8 to 851.6)	127.1 (112.8 to 142.6)	47.5 (35.1 to 63.8)	0.0 (0.0 to 0.0)	1802.5 (1727.6 to 1879.8)	1484.9 (1422.0 to 1549.0)	231.2 (205.1 to 259.4)	86.5 (63.8 to 116.0)	0.0 (0.0 to 0.0)
Croatia	2012	943.8 (901.9 to 987.7)	771.4 (735.0 to 808.6)	120.9 (107.2 to 136.1)	51.5 (38.4 to 67.6)	0.0 (0.0 to 0.0)	1716.6 (1640.4 to 1796.4)	1403.0 (1336.8 to 1470.8)	219.9 (195.0 to 247.6)	93.6 (69.8 to 123.0)	0.0 (0.0 to 0.0)
Croatia	2013	887.9 (848.5 to 932.0)	718.1 (686.1 to 755.6)	119.9 (105.7 to 134.9)	49.9 (36.7 to 65.9)	0.0 (0.0 to 0.0)	1615.0 (1543.3 to 1695.1)	1306.1 (1247.9 to 1374.3)	218.0 (192.2 to 245.4)	90.8 (66.7 to 119.8)	0.0 (0.0 to 0.0)
Croatia	2014	873.2 (830.9 to 917.3)	697.1 (662.0 to 731.4)	127.9 (112.5 to 144.9)	48.2 (34.3 to 64.5)	0.0 (0.0 to 0.0)	1588.2 (1511.4 to 1668.5)	1267.8 (1204.1 to 1330.3)	232.7 (204.7 to 263.6)	87.7 (62.4 to 117.3)	0.0 (0.0 to 0.0)
Croatia	2015	893.5 (846.5 to 945.6)	705.6 (665.1 to 746.2)	135.5 (115.9 to 155.1)	52.5 (36.1 to 71.4)	0.0 (0.0 to 0.0)	1625.2 (1539.7 to 1719.8)	1283.3 (1209.7 to 1357.2)	246.4 (210.8 to 282.1)	95.5 (65.6 to 129.9)	0.0 (0.0 to 0.0)

		2018 US Dollars per capita					2018 PPP per capita				
Country	Year	Health spending	Government health spending	Out-of-pocket health spending	Prepaid private health spending	Development assistance for health	Health spending	Government health spending	Out-of-pocket health spending	Prepaid private health spending	Development assistance for health
Croatia	2016	938.6 (884.9 to 1005.0)	729.0 (679.9 to 786.4)	142.2 (118.9 to 167.1)	57.9 (37.9 to 81.0)	9.5 (9.5 to 9.5)	1707.2 (1609.5 to 1828.0)	1326.0 (1236.6 to 1430.4)	258.7 (216.3 to 303.9)	105.3 (69.0 to 147.2)	17.2 (17.2 to 17.2)
Croatia	2017	960.4 (899.3 to 1030.8)	754.7 (704.1 to 813.4)	146.5 (122.2 to 172.7)	59.2 (39.1 to 83.6)	0.0 (0.0 to 0.0)	1746.9 (1635.6 to 1874.9)	1372.8 (1280.6 to 1479.5)	266.5 (222.3 to 314.1)	107.6 (71.1 to 152.1)	0.0 (0.0 to 0.0)
Croatia	2018	978.7 (919.2 to 1050.9)	768.8 (716.9 to 828.5)	149.4 (124.7 to 175.9)	60.5 (39.8 to 85.4)	0.0 (0.0 to 0.0)	1780.0 (1671.9 to 1911.4)	1398.3 (1303.9 to 1506.9)	271.7 (226.8 to 319.9)	110.1 (72.5 to 155.4)	0.0 (0.0 to 0.0)
Croatia	2019	992.0 (928.4 to 1065.6)	778.6 (725.6 to 839.6)	152.0 (127.0 to 180.2)	61.4 (40.3 to 86.5)	0.0 (0.0 to 0.0)	1804.3 (1688.5 to 1938.1)	1416.2 (1319.7 to 1527.2)	276.4 (231.1 to 327.8)	111.7 (73.4 to 157.3)	0.0 (0.0 to 0.0)
Croatia	2020	1004.7 (943.4 to 1081.0)	788.7 (734.2 to 850.8)	153.6 (127.7 to 182.3)	62.4 (41.0 to 87.8)	0.0 (0.0 to 0.0)	1827.4 (1716.0 to 1966.2)	1434.6 (1335.4 to 1547.4)	279.3 (232.3 to 331.5)	113.5 (74.6 to 159.8)	0.0 (0.0 to 0.0)
Croatia	2021	1015.2 (947.8 to 1090.8)	797.2 (740.1 to 860.9)	154.7 (128.6 to 183.3)	63.3 (41.5 to 89.1)	0.0 (0.0 to 0.0)	1846.5 (1723.8 to 1984.1)	1450.0 (1346.1 to 1565.8)	281.3 (233.9 to 333.4)	115.1 (75.5 to 162.1)	0.0 (0.0 to 0.0)
Croatia	2022	1029.3 (965.6 to 1110.1)	809.0 (751.5 to 872.6)	156.0 (129.5 to 185.0)	64.3 (42.1 to 90.6)	0.0 (0.0 to 0.0)	1872.2 (1756.3 to 2019.2)	1471.5 (1366.9 to 1587.1)	283.7 (235.6 to 336.5)	117.0 (76.6 to 164.8)	0.0 (0.0 to 0.0)
Croatia	2023	1045.5 (974.8 to 1125.9)	822.8 (762.2 to 889.5)	157.3 (130.7 to 186.6)	65.4 (42.9 to 91.9)	0.0 (0.0 to 0.0)	1901.6 (1773.0 to 2047.9)	1496.5 (1386.3 to 1617.9)	286.1 (237.7 to 339.4)	118.9 (78.0 to 167.2)	0.0 (0.0 to 0.0)
Croatia	2024	1062.0 (994.5 to 1145.8)	836.9 (774.1 to 905.3)	158.7 (131.7 to 188.2)	66.4 (43.7 to 93.3)	0.0 (0.0 to 0.0)	1931.6 (1808.8 to 2084.1)	1522.1 (1408.0 to 1646.5)	288.6 (239.6 to 342.3)	120.9 (79.5 to 169.8)	0.0 (0.0 to 0.0)
Croatia	2025	1078.2 (1004.2 to 1161.2)	850.5 (784.7 to 920.3)	160.1 (133.0 to 190.2)	67.6 (44.6 to 95.2)	0.0 (0.0 to 0.0)	1961.0 (1826.5 to 2112.0)	1546.8 (1427.2 to 1673.9)	291.2 (241.9 to 345.9)	122.9 (81.1 to 173.1)	0.0 (0.0 to 0.0)
Croatia	2026	1094.5 (1023.3 to 1181.0)	864.2 (796.9 to 937.1)	161.5 (134.1 to 192.1)	68.7 (45.3 to 96.9)	0.0 (0.0 to 0.0)	1990.7 (1861.2 to 2148.0)	1571.9 (1449.5 to 1704.4)	293.8 (243.9 to 349.4)	125.0 (82.5 to 176.2)	0.0 (0.0 to 0.0)
Croatia	2027	1110.7 (1034.0 to 1200.6)	877.9 (807.8 to 953.6)	162.9 (135.2 to 193.9)	69.9 (46.1 to 98.2)	0.0 (0.0 to 0.0)	2020.2 (1880.6 to 2183.7)	1596.8 (1469.2 to 1734.4)	296.3 (246.0 to 352.7)	127.1 (83.9 to 178.6)	0.0 (0.0 to 0.0)
Croatia	2028	1126.9 (1051.4 to 1211.5)	891.6 (819.4 to 969.0)	164.3 (136.5 to 195.4)	71.1 (46.8 to 99.4)	0.0 (0.0 to 0.0)	2049.7 (1912.3 to 2203.5)	1621.6 (1490.3 to 1762.4)	298.8 (248.2 to 355.3)	129.3 (85.1 to 180.8)	0.0 (0.0 to 0.0)
Croatia	2029	1143.9 (1062.7 to 1237.2)	905.8 (832.3 to 987.4)	165.8 (137.8 to 197.3)	72.3 (47.5 to 101.1)	0.0 (0.0 to 0.0)	2080.6 (1932.8 to 2250.2)	1647.4 (1513.7 to 1795.9)	301.7 (250.7 to 358.8)	131.5 (86.5 to 183.9)	0.0 (0.0 to 0.0)
Croatia	2030	1161.7 (1080.7 to 1254.0)	920.5 (847.5 to 1005.0)	167.6 (139.2 to 199.9)	73.7 (48.3 to 103.2)	0.0 (0.0 to 0.0)	2113.0 (1965.7 to 2280.8)	1674.2 (1541.5 to 1827.9)	304.8 (253.2 to 363.6)	134.0 (87.9 to 187.6)	0.0 (0.0 to 0.0)
Croatia	2031	1181.0 (1095.3 to 1283.4)	936.3 (859.6 to 1026.2)	169.5 (140.9 to 202.7)	75.1 (49.3 to 105.3)	0.0 (0.0 to 0.0)	2148.0 (1992.1 to 2334.2)	1703.0 (1563.6 to 1866.5)	308.3 (256.3 to 368.7)	136.6 (89.6 to 191.5)	0.0 (0.0 to 0.0)
Croatia	2032	1201.0 (1114.4 to 1298.4)	952.6 (874.7 to 1044.9)	171.7 (142.8 to 205.2)	76.7 (50.4 to 107.5)	0.0 (0.0 to 0.0)	2184.3 (2026.8 to 2361.6)	1732.6 (1590.9 to 1900.4)	312.2 (259.7 to 373.2)	139.5 (91.6 to 195.5)	0.0 (0.0 to 0.0)
Croatia	2033	1222.2 (1131.2 to 1328.3)	969.9 (888.1 to 1065.6)	174.0 (144.9 to 207.9)	78.4 (51.5 to 109.5)	0.0 (0.0 to 0.0)	2223.0 (2057.4 to 2416.0)	1764.1 (1615.3 to 1938.2)	316.4 (263.6 to 378.1)	142.5 (93.6 to 199.2)	0.0 (0.0 to 0.0)
Croatia	2034	1243.9 (1151.2 to 1349.0)	987.5 (902.0 to 1085.5)	176.3 (146.8 to 210.6)	80.0 (52.6 to 112.0)	0.0 (0.0 to 0.0)	2262.4 (2093.8 to 2453.6)	1796.2 (1640.6 to 1974.3)	320.6 (266.9 to 383.0)	145.6 (95.7 to 203.8)	0.0 (0.0 to 0.0)
Croatia	2035	1264.9 (1167.0 to 1378.9)	1004.7 (914.6 to 1104.0)	178.5 (148.7 to 213.2)	81.7 (53.6 to 114.4)	0.0 (0.0 to 0.0)	2300.7 (2122.6 to 2508.0)	1827.5 (1663.6 to 2007.9)	324.6 (270.5 to 387.7)	148.6 (97.6 to 208.1)	0.0 (0.0 to 0.0)
Croatia	2036	1285.5 (1184.9 to 1398.3)	1021.6 (928.7 to 1125.2)	180.6 (150.6 to 215.6)	83.3 (54.5 to 117.1)	0.0 (0.0 to 0.0)	2338.2 (2155.1 to 2543.4)	1858.2 (1689.1 to 2046.5)	328.4 (273.9 to 392.2)	151.6 (99.1 to 213.0)	0.0 (0.0 to 0.0)

		2018 US Dollars per capita					2018 PPP per capita				
Country	Year	Health spending	Government health spending	Out-of-pocket health spending	Prepaid private health spending	Development assistance for health	Health spending	Government health spending	Out-of-pocket health spending	Prepaid private health spending	Development assistance for health
Croatia	2037	1305.9 (1201.2 to 1425.1)	1038.4 (942.0 to 1146.2)	182.6 (152.2 to 217.9)	84.9 (55.4 to 119.4)	0.0 (0.0 to 0.0)	2375.2 (2184.8 to 2592.0)	1888.7 (1713.3 to 2084.7)	332.1 (276.8 to 396.3)	154.5 (100.7 to 217.2)	0.0 (0.0 to 0.0)
Croatia	2038	1325.8 (1219.2 to 1446.8)	1054.9 (954.4 to 1166.8)	184.4 (153.7 to 220.2)	86.5 (56.3 to 121.5)	0.0 (0.0 to 0.0)	2411.4 (2217.5 to 2631.4)	1918.6 (1736.0 to 2122.2)	335.5 (279.6 to 400.5)	157.4 (102.4 to 221.0)	0.0 (0.0 to 0.0)
Croatia	2039	1346.7 (1232.4 to 1474.2)	1072.2 (966.7 to 1185.8)	186.4 (155.5 to 222.8)	88.1 (57.3 to 123.9)	0.0 (0.0 to 0.0)	2449.4 (2241.6 to 2681.3)	1950.1 (1758.3 to 2156.7)	339.0 (282.9 to 405.3)	160.3 (104.1 to 225.4)	0.0 (0.0 to 0.0)
Croatia	2040	1366.6 (1250.4 to 1497.6)	1088.7 (979.3 to 1207.5)	188.2 (157.2 to 225.1)	89.7 (58.2 to 126.4)	0.0 (0.0 to 0.0)	2485.6 (2274.2 to 2723.9)	1980.1 (1781.2 to 2196.3)	342.3 (286.0 to 409.4)	163.2 (105.8 to 229.9)	0.0 (0.0 to 0.0)
Croatia	2041	1386.9 (1264.7 to 1524.0)	1105.5 (993.1 to 1230.5)	190.0 (159.0 to 227.6)	91.3 (59.2 to 128.9)	0.0 (0.0 to 0.0)	2522.5 (2300.3 to 2771.8)	2010.7 (1806.3 to 2238.1)	345.7 (289.2 to 413.9)	166.1 (107.8 to 234.5)	0.0 (0.0 to 0.0)
Croatia	2042	1408.0 (1284.3 to 1543.6)	1122.9 (1007.6 to 1252.1)	192.0 (160.9 to 230.2)	93.0 (60.4 to 131.6)	0.0 (0.0 to 0.0)	2560.9 (2335.9 to 2807.5)	2042.4 (1832.7 to 2277.3)	349.3 (292.6 to 418.6)	169.2 (109.8 to 239.3)	0.0 (0.0 to 0.0)
Croatia	2043	1428.6 (1297.4 to 1579.3)	1139.9 (1019.3 to 1275.1)	193.9 (162.3 to 232.5)	94.7 (61.6 to 134.2)	0.0 (0.0 to 0.0)	2598.4 (2359.7 to 2872.4)	2073.4 (1853.9 to 2319.1)	352.7 (295.2 to 422.9)	172.3 (112.0 to 244.1)	0.0 (0.0 to 0.0)
Croatia	2044	1449.1 (1317.7 to 1594.8)	1156.9 (1032.3 to 1294.1)	195.8 (163.7 to 234.6)	96.4 (62.9 to 136.4)	0.0 (0.0 to 0.0)	2635.6 (2396.6 to 2900.7)	2104.2 (1877.6 to 2353.8)	356.1 (297.8 to 426.6)	175.4 (114.4 to 248.1)	0.0 (0.0 to 0.0)
Croatia	2045	1469.7 (1331.0 to 1628.6)	1173.9 (1044.8 to 1318.4)	197.6 (165.2 to 236.8)	98.1 (64.3 to 138.7)	0.0 (0.0 to 0.0)	2673.1 (2420.9 to 2962.2)	2135.2 (1900.4 to 2398.0)	359.4 (300.5 to 430.7)	178.4 (116.9 to 252.3)	0.0 (0.0 to 0.0)
Croatia	2046	1489.7 (1350.0 to 1645.2)	1190.6 (1056.9 to 1342.3)	199.3 (166.5 to 239.3)	99.8 (65.6 to 141.7)	0.0 (0.0 to 0.0)	2709.4 (2455.4 to 2992.4)	2165.5 (1922.3 to 2441.4)	362.5 (302.8 to 435.3)	181.5 (119.4 to 257.7)	0.0 (0.0 to 0.0)
Croatia	2047	1509.2 (1361.7 to 1677.5)	1206.9 (1068.9 to 1363.0)	201.0 (167.7 to 241.7)	101.4 (66.6 to 144.6)	0.0 (0.0 to 0.0)	2745.0 (2476.7 to 3051.1)	2195.1 (1944.2 to 2479.0)	365.5 (305.0 to 439.7)	184.4 (121.1 to 263.0)	0.0 (0.0 to 0.0)
Croatia	2048	1528.4 (1375.0 to 1695.8)	1222.8 (1079.8 to 1386.1)	202.6 (168.9 to 243.8)	103.0 (67.5 to 146.8)	0.0 (0.0 to 0.0)	2780.0 (2500.9 to 3084.4)	2224.1 (1963.9 to 2521.1)	368.4 (307.2 to 443.4)	187.4 (122.7 to 267.1)	0.0 (0.0 to 0.0)
Croatia	2049	1548.7 (1391.9 to 1729.5)	1239.6 (1089.0 to 1408.1)	204.3 (170.4 to 245.9)	104.8 (68.4 to 149.7)	0.0 (0.0 to 0.0)	2816.9 (2531.6 to 3145.7)	2254.7 (1980.7 to 2561.2)	371.6 (310.0 to 447.3)	190.6 (124.4 to 272.2)	0.0 (0.0 to 0.0)
Croatia	2050	1569.7 (1407.0 to 1748.1)	1257.0 (1103.3 to 1430.1)	206.1 (172.0 to 248.0)	106.6 (69.4 to 152.8)	0.0 (0.0 to 0.0)	2855.0 (2559.0 to 3179.5)	2286.3 (2006.8 to 2601.1)	374.8 (312.8 to 451.0)	193.8 (126.2 to 277.9)	0.0 (0.0 to 0.0)
Cuba	1995	218.3 (191.1 to 248.5)	172.5 (148.6 to 200.6)	32.8 (24.3 to 42.4)	13.0 (6.1 to 24.5)	0.0 (0.0 to 0.0)	478.0 (418.5 to 544.2)	377.7 (325.4 to 439.2)	71.8 (53.1 to 92.8)	28.5 (13.3 to 53.6)	0.0 (0.0 to 0.0)
Cuba	1996	229.5 (202.7 to 257.6)	181.8 (159.0 to 207.6)	34.4 (26.0 to 44.3)	13.3 (6.2 to 25.5)	0.1 (0.1 to 0.1)	502.6 (443.9 to 564.1)	398.0 (348.2 to 454.6)	75.4 (56.9 to 97.0)	29.0 (13.7 to 55.9)	0.2 (0.2 to 0.2)
Cuba	1997	238.9 (212.7 to 265.9)	189.3 (167.4 to 213.6)	36.3 (27.6 to 46.3)	13.2 (6.3 to 25.8)	0.2 (0.2 to 0.2)	523.2 (465.7 to 582.3)	414.4 (366.6 to 467.6)	79.4 (60.4 to 101.4)	28.9 (13.8 to 56.4)	0.5 (0.5 to 0.5)
Cuba	1998	246.9 (222.4 to 272.7)	195.5 (174.4 to 218.1)	38.4 (30.0 to 48.4)	13.0 (6.0 to 25.1)	0.1 (0.1 to 0.1)	540.7 (486.9 to 597.2)	428.0 (381.9 to 477.5)	84.0 (65.8 to 106.0)	28.4 (13.2 to 54.9)	0.3 (0.3 to 0.3)
Cuba	1999	268.8 (243.6 to 296.2)	212.8 (191.0 to 235.6)	42.0 (33.3 to 51.8)	13.5 (6.2 to 26.2)	0.5 (0.5 to 0.5)	588.7 (533.3 to 648.6)	466.0 (418.2 to 515.9)	92.0 (72.8 to 113.4)	29.5 (13.6 to 57.4)	1.2 (1.2 to 1.2)
Cuba	2000	292.2 (267.1 to 319.3)	230.3 (207.7 to 254.8)	47.5 (38.3 to 58.2)	14.1 (6.7 to 27.4)	0.4 (0.4 to 0.4)	639.9 (584.8 to 699.1)	504.2 (454.8 to 558.0)	104.0 (83.9 to 127.4)	30.9 (14.6 to 60.0)	0.9 (0.9 to 0.9)
Cuba	2001	312.0 (285.9 to 339.7)	243.3 (221.0 to 268.4)	53.8 (43.6 to 65.6)	14.3 (6.8 to 27.3)	0.5 (0.5 to 0.5)	683.1 (626.0 to 743.9)	532.8 (484.0 to 587.7)	117.9 (95.4 to 143.6)	31.4 (15.0 to 59.8)	1.0 (1.0 to 1.0)

		2018 US Dollars per capita					2018 PPP per capita				
**Country**	**Year**	**Health spending**	**Government health spending**	**Out-of-pocket health spending**	**Prepaid private health spending**	**Development assistance for health**	**Health spending**	**Government health spending**	**Out-of-pocket health spending**	**Prepaid private health spending**	**Development assistance for health**
Cuba	2002	327.3 (300.6 to 356.5)	253.3 (230.4 to 278.4)	58.3 (47.3 to 71.0)	15.1 (7.1 to 28.9)	0.5 (0.5 to 0.5)	716.6 (658.2 to 780.7)	554.8 (504.6 to 609.5)	127.6 (103.6 to 155.4)	33.1 (15.6 to 63.4)	1.2 (1.2 to 1.2)
Cuba	2003	351.8 (321.7 to 382.9)	271.5 (247.2 to 296.1)	61.9 (49.9 to 75.3)	17.0 (7.9 to 32.9)	1.4 (1.4 to 1.4)	770.3 (704.5 to 838.5)	594.5 (541.3 to 648.3)	135.5 (109.3 to 165.0)	37.3 (17.4 to 72.1)	3.0 (3.0 to 3.0)
Cuba	2004	392.4 (360.2 to 426.9)	306.1 (281.5 to 330.7)	64.2 (52.2 to 77.6)	20.8 (9.6 to 39.8)	1.4 (1.4 to 1.4)	859.3 (788.8 to 934.9)	670.2 (616.5 to 724.0)	140.5 (114.3 to 170.0)	45.5 (20.9 to 87.1)	3.0 (3.0 to 3.0)
Cuba	2005	486.0 (448.9 to 530.3)	388.8 (360.4 to 419.1)	68.6 (56.1 to 83.1)	27.6 (13.0 to 51.6)	0.9 (0.9 to 0.9)	1064.1 (983.0 to 1161.2)	851.4 (789.1 to 917.6)	150.2 (122.9 to 181.9)	60.5 (28.5 to 113.1)	2.0 (2.0 to 2.0)
Cuba	2006	594.8 (551.5 to 643.3)	486.3 (453.8 to 518.8)	71.7 (58.6 to 86.6)	35.8 (16.7 to 67.2)	1.0 (1.0 to 1.0)	1302.4 (1207.6 to 1408.7)	1064.8 (993.7 to 1135.9)	157.0 (128.2 to 189.6)	78.4 (36.6 to 147.1)	2.2 (2.2 to 2.2)
Cuba	2007	733.7 (684.8 to 790.9)	612.1 (574.8 to 648.6)	73.9 (60.5 to 88.6)	46.1 (21.8 to 86.3)	1.6 (1.6 to 1.6)	1606.6 (1499.4 to 1731.8)	1340.2 (1258.6 to 1420.2)	161.8 (132.4 to 194.1)	100.9 (47.8 to 189.1)	3.6 (3.6 to 3.6)
Cuba	2008	831.4 (777.2 to 895.3)	701.1 (663.6 to 740.5)	74.8 (61.1 to 89.1)	54.4 (25.1 to 100.9)	1.1 (1.1 to 1.1)	1820.6 (1701.8 to 1960.4)	1535.2 (1453.1 to 1621.4)	163.8 (133.8 to 195.0)	119.1 (55.1 to 221.0)	2.5 (2.5 to 2.5)
Cuba	2009	877.7 (821.7 to 948.7)	740.6 (705.8 to 780.2)	75.9 (62.2 to 90.3)	59.3 (27.2 to 110.8)	2.0 (2.0 to 2.0)	1922.0 (1799.4 to 2077.3)	1621.6 (1545.4 to 1708.4)	166.1 (136.2 to 197.8)	129.9 (59.5 to 242.7)	4.4 (4.4 to 4.4)
Cuba	2010	869.1 (815.5 to 937.5)	728.2 (694.1 to 768.0)	78.3 (63.9 to 93.6)	60.7 (27.8 to 114.2)	1.9 (1.9 to 1.9)	1903.0 (1785.6 to 2052.8)	1594.5 (1519.9 to 1681.7)	171.4 (140.0 to 205.0)	133.0 (60.8 to 250.0)	4.1 (4.1 to 4.1)
Cuba	2011	866.7 (813.2 to 934.6)	718.5 (684.5 to 758.8)	85.0 (69.4 to 101.7)	61.7 (28.6 to 117.6)	1.5 (1.5 to 1.5)	1897.8 (1780.7 to 2046.5)	1573.2 (1498.8 to 1661.4)	186.2 (151.9 to 222.6)	135.2 (62.6 to 257.5)	3.2 (3.2 to 3.2)
Cuba	2012	854.8 (801.6 to 919.3)	702.0 (666.1 to 739.3)	89.4 (72.9 to 106.4)	62.2 (29.3 to 118.1)	1.2 (1.2 to 1.2)	1871.8 (1755.3 to 2013.0)	1537.2 (1458.6 to 1618.8)	195.7 (159.6 to 233.1)	136.3 (64.1 to 258.6)	2.7 (2.7 to 2.7)
Cuba	2013	911.5 (854.2 to 977.3)	749.1 (711.6 to 786.0)	94.3 (77.2 to 113.0)	66.8 (32.1 to 125.9)	1.3 (1.3 to 1.3)	1996.0 (1870.3 to 2140.1)	1640.4 (1558.1 to 1721.0)	206.4 (169.2 to 247.5)	146.3 (70.3 to 275.8)	2.9 (2.9 to 2.9)
Cuba	2014	995.9 (936.7 to 1065.8)	825.4 (784.9 to 865.2)	96.8 (78.8 to 117.3)	73.0 (34.0 to 138.1)	0.8 (0.8 to 0.8)	2180.7 (2051.0 to 2333.7)	1807.3 (1718.8 to 1894.6)	211.9 (172.6 to 256.8)	159.8 (74.4 to 302.4)	1.7 (1.7 to 1.7)
Cuba	2015	1093.1 (1027.5 to 1170.0)	910.1 (864.9 to 955.8)	102.2 (82.5 to 125.9)	79.6 (36.9 to 151.3)	1.1 (1.1 to 1.1)	2393.5 (2250.0 to 2561.9)	1992.9 (1893.8 to 2092.9)	223.9 (180.6 to 275.6)	174.2 (80.8 to 331.3)	2.5 (2.5 to 2.5)
Cuba	2016	1128.1 (1046.7 to 1228.1)	939.5 (881.6 to 999.4)	105.2 (82.9 to 131.4)	82.4 (38.4 to 158.7)	1.0 (1.0 to 1.0)	2470.1 (2291.9 to 2689.1)	2057.1 (1930.4 to 2188.4)	230.3 (181.5 to 287.7)	180.5 (84.1 to 347.5)	2.2 (2.2 to 2.2)
Cuba	2017	1145.8 (1063.9 to 1250.5)	953.4 (894.5 to 1016.1)	107.8 (84.9 to 135.1)	83.5 (38.8 to 161.0)	1.0 (1.0 to 1.0)	2508.8 (2329.6 to 2738.3)	2087.6 (1958.7 to 2224.8)	236.1 (185.8 to 295.9)	182.9 (85.0 to 352.6)	2.3 (2.3 to 2.3)
Cuba	2018	1171.3 (1090.2 to 1273.9)	974.9 (912.8 to 1038.8)	110.1 (86.7 to 138.0)	85.3 (39.6 to 164.3)	1.0 (1.0 to 1.0)	2564.8 (2387.2 to 2789.4)	2134.7 (1998.8 to 2274.6)	241.0 (189.8 to 302.1)	186.9 (86.7 to 359.8)	2.2 (2.2 to 2.2)
Cuba	2019	1197.2 (1110.3 to 1302.5)	996.8 (933.8 to 1063.0)	112.2 (88.7 to 139.9)	87.1 (40.4 to 167.5)	1.0 (0.9 to 1.1)	2621.4 (2431.3 to 2852.0)	2182.8 (2044.8 to 2327.7)	245.7 (194.2 to 306.3)	190.8 (88.5 to 366.7)	2.2 (2.1 to 2.3)
Cuba	2020	1221.0 (1135.2 to 1326.5)	1017.4 (952.4 to 1085.3)	113.8 (89.9 to 141.7)	88.8 (41.1 to 170.6)	1.0 (0.9 to 1.1)	2673.6 (2485.8 to 2904.5)	2227.7 (2085.4 to 2376.5)	249.1 (196.9 to 310.2)	194.5 (90.1 to 373.6)	2.3 (2.1 to 2.4)
Cuba	2021	1244.5 (1155.0 to 1349.7)	1037.6 (969.0 to 1106.8)	115.2 (90.9 to 143.9)	90.6 (42.0 to 174.3)	1.1 (1.0 to 1.2)	2725.1 (2529.1 to 2955.5)	2272.1 (2121.8 to 2423.5)	252.3 (199.1 to 315.1)	198.4 (91.9 to 381.6)	2.3 (2.1 to 2.6)
Cuba	2022	1267.8 (1178.2 to 1375.9)	1056.0 (985.8 to 1129.5)	118.3 (93.4 to 147.7)	92.3 (42.8 to 177.6)	1.1 (1.0 to 1.2)	2776.0 (2579.8 to 3012.8)	2312.4 (2158.5 to 2473.3)	259.1 (204.6 to 323.4)	202.1 (93.6 to 388.9)	2.4 (2.1 to 2.7)

		2018 US Dollars per capita					2018 PPP per capita				
Country	Year	Health spending	Government health spending	Out-of-pocket health spending	Prepaid private health spending	Development assistance for health	Health spending	Government health spending	Out-of-pocket health spending	Prepaid private health spending	Development assistance for health
Cuba	2023	1288.0 (1197.1 to 1398.6)	1072.8 (1001.5 to 1148.5)	120.2 (94.3 to 150.0)	93.8 (43.5 to 180.6)	1.1 (1.0 to 1.3)	2820.4 (2621.3 to 3062.6)	2349.2 (2192.9 to 2514.8)	263.2 (206.5 to 328.4)	205.5 (95.3 to 395.5)	2.5 (2.2 to 2.8)
Cuba	2024	1308.3 (1212.5 to 1418.7)	1089.9 (1015.4 to 1166.7)	122.0 (95.6 to 153.0)	95.3 (44.3 to 183.4)	1.2 (1.0 to 1.4)	2864.8 (2655.0 to 3106.6)	2386.5 (2223.5 to 2554.7)	267.1 (209.4 to 335.1)	208.7 (96.9 to 401.7)	2.6 (2.2 to 3.0)
Cuba	2025	1330.4 (1235.8 to 1441.4)	1108.1 (1031.6 to 1188.6)	124.5 (97.7 to 155.6)	96.7 (44.9 to 186.1)	1.2 (1.1 to 1.4)	2913.2 (2706.0 to 3156.3)	2426.3 (2258.8 to 2602.7)	272.6 (214.0 to 340.7)	211.7 (98.4 to 407.5)	2.7 (2.3 to 3.2)
Cuba	2026	1352.6 (1247.2 to 1471.7)	1126.4 (1044.8 to 1210.1)	127.0 (99.3 to 160.0)	97.9 (45.6 to 188.5)	1.3 (1.1 to 1.5)	2961.8 (2731.0 to 3222.6)	2466.5 (2287.7 to 2649.7)	278.1 (217.5 to 350.3)	214.4 (99.8 to 412.9)	2.8 (2.4 to 3.3)
Cuba	2027	1374.9 (1266.6 to 1500.9)	1144.9 (1057.5 to 1233.7)	129.6 (100.7 to 163.9)	99.1 (46.2 to 190.8)	1.3 (1.1 to 1.6)	3010.5 (2773.5 to 3286.6)	2507.0 (2315.6 to 2701.3)	283.7 (220.5 to 358.9)	216.9 (101.2 to 417.7)	2.9 (2.4 to 3.4)
Cuba	2028	1396.4 (1283.1 to 1527.8)	1162.9 (1069.1 to 1255.7)	132.1 (102.3 to 168.5)	100.1 (46.7 to 192.7)	1.4 (1.1 to 1.7)	3057.8 (2809.5 to 3345.3)	2546.4 (2341.0 to 2749.5)	289.2 (224.0 to 368.9)	219.1 (102.3 to 422.0)	3.0 (2.5 to 3.6)
Cuba	2029	1419.0 (1298.7 to 1549.9)	1181.8 (1079.4 to 1278.2)	134.6 (104.5 to 170.8)	101.2 (47.3 to 194.7)	1.4 (1.2 to 1.7)	3107.2 (2843.8 to 3393.9)	2587.7 (2363.6 to 2798.8)	294.8 (228.7 to 374.0)	221.5 (103.6 to 426.4)	3.1 (2.6 to 3.8)
Cuba	2030	1440.2 (1313.3 to 1579.3)	1199.4 (1090.5 to 1304.1)	137.1 (105.8 to 175.6)	102.2 (47.9 to 196.6)	1.5 (1.2 to 1.8)	3153.6 (2875.7 to 3458.1)	2626.3 (2387.9 to 2855.6)	300.3 (231.6 to 384.6)	223.8 (104.8 to 430.5)	3.2 (2.7 to 4.0)
Cuba	2031	1460.9 (1325.9 to 1606.5)	1216.5 (1101.2 to 1329.7)	139.6 (106.3 to 180.0)	103.2 (48.4 to 198.6)	1.5 (1.2 to 1.9)	3198.8 (2903.2 to 3517.7)	2663.7 (2411.3 to 2911.7)	305.7 (232.9 to 394.2)	226.1 (105.9 to 434.8)	3.4 (2.7 to 4.2)
Cuba	2032	1481.3 (1337.1 to 1634.3)	1233.4 (1113.8 to 1353.7)	142.1 (106.8 to 184.6)	104.3 (48.9 to 200.6)	1.6 (1.3 to 2.0)	3243.6 (2927.7 to 3578.5)	2700.7 (2438.9 to 2964.3)	311.1 (233.8 to 404.3)	228.4 (107.2 to 439.3)	3.5 (2.8 to 4.4)
Cuba	2033	1503.3 (1363.2 to 1669.8)	1251.5 (1127.4 to 1379.1)	144.6 (107.0 to 189.3)	105.6 (49.5 to 203.0)	1.7 (1.3 to 2.1)	3291.7 (2985.0 to 3656.4)	2740.3 (2468.7 to 3019.8)	316.6 (234.4 to 414.4)	231.1 (108.4 to 444.4)	3.6 (2.9 to 4.7)
Cuba	2034	1524.3 (1377.5 to 1694.1)	1268.8 (1139.0 to 1402.3)	147.1 (107.2 to 193.7)	106.7 (50.1 to 205.2)	1.7 (1.4 to 2.2)	3337.8 (3016.3 to 3709.6)	2778.3 (2494.0 to 3070.5)	322.0 (234.7 to 424.1)	233.7 (109.6 to 449.4)	3.8 (3.0 to 4.9)
Cuba	2035	1544.9 (1385.9 to 1724.3)	1285.8 (1148.9 to 1425.6)	149.5 (107.3 to 198.4)	107.9 (50.6 to 207.4)	1.8 (1.4 to 2.4)	3382.9 (3034.7 to 3775.7)	2815.4 (2515.8 to 3121.7)	327.3 (235.0 to 434.4)	236.2 (110.8 to 454.1)	3.9 (3.1 to 5.1)
Cuba	2036	1565.0 (1397.8 to 1751.0)	1302.2 (1154.2 to 1450.0)	151.9 (107.6 to 202.5)	109.0 (51.2 to 209.0)	1.9 (1.4 to 2.5)	3426.9 (3060.7 to 3834.1)	2851.5 (2527.3 to 3175.1)	332.5 (235.7 to 443.3)	238.8 (112.0 to 457.7)	4.1 (3.2 to 5.5)
Cuba	2037	1584.5 (1413.2 to 1770.3)	1318.2 (1163.8 to 1472.9)	154.2 (108.1 to 207.2)	110.2 (51.7 to 211.5)	1.9 (1.5 to 2.6)	3469.5 (3094.4 to 3876.4)	2886.3 (2548.4 to 3225.2)	337.6 (236.7 to 453.7)	241.3 (113.2 to 463.2)	4.2 (3.3 to 5.7)
Cuba	2038	1605.2 (1426.6 to 1804.8)	1335.2 (1175.1 to 1497.6)	156.6 (108.8 to 211.9)	111.5 (52.3 to 214.2)	2.0 (1.5 to 2.8)	3515.0 (3123.7 to 3952.0)	2923.6 (2573.2 to 3279.3)	342.9 (238.3 to 464.0)	244.1 (114.5 to 469.1)	4.4 (3.4 to 6.1)
Cuba	2039	1626.7 (1436.8 to 1821.2)	1352.7 (1187.1 to 1519.6)	159.0 (109.3 to 216.4)	112.9 (53.0 to 216.7)	2.1 (1.6 to 2.9)	3561.9 (3146.1 to 3987.8)	2962.0 (2599.3 to 3327.5)	348.1 (239.4 to 473.9)	247.2 (116.0 to 474.6)	4.6 (3.5 to 6.4)
Cuba	2040	1648.6 (1453.6 to 1859.9)	1370.6 (1200.3 to 1546.6)	161.4 (110.2 to 220.6)	114.5 (53.8 to 219.3)	2.2 (1.7 to 3.0)	3610.0 (3182.9 to 4072.7)	3001.1 (2628.3 to 3386.6)	353.5 (241.2 to 482.9)	250.6 (117.7 to 480.3)	4.8 (3.6 to 6.6)
Cuba	2041	1672.7 (1481.3 to 1889.6)	1390.1 (1216.3 to 1570.6)	164.0 (111.1 to 224.8)	116.4 (54.6 to 223.1)	2.3 (1.7 to 3.1)	3662.6 (3243.5 to 4137.5)	3043.8 (2663.3 to 3439.1)	359.0 (243.2 to 492.3)	254.8 (119.6 to 488.6)	5.0 (3.7 to 6.9)
Cuba	2042	1698.8 (1504.0 to 1923.6)	1411.3 (1234.9 to 1597.9)	166.6 (112.2 to 229.2)	118.6 (55.6 to 227.6)	2.4 (1.8 to 3.4)	3719.9 (3293.2 to 4212.2)	3090.3 (2704.1 to 3498.9)	364.8 (245.7 to 501.9)	259.6 (121.8 to 498.3)	5.2 (3.9 to 7.4)
Cuba	2043	1727.1 (1530.7 to 1951.2)	1434.3 (1254.8 to 1628.2)	169.3 (113.5 to 233.8)	121.0 (56.7 to 232.5)	2.4 (1.8 to 3.5)	3781.9 (3351.8 to 4272.5)	3140.8 (2747.7 to 3565.2)	370.8 (248.6 to 512.0)	265.0 (124.2 to 509.1)	5.4 (4.0 to 7.8)

		2018 US Dollars per capita					2018 PPP per capita				
Country	Year	Health spending	Government health spending	Out-of-pocket health spending	Prepaid private health spending	Development assistance for health	Health spending	Government health spending	Out-of-pocket health spending	Prepaid private health spending	Development assistance for health
Cuba	2044	1754.5 (1547.7 to 1981.5)	1456.5 (1273.1 to 1651.7)	172.0 (114.8 to 237.9)	123.4 (57.8 to 237.4)	2.5 (1.9 to 3.7)	3841.7 (3389.0 to 4338.9)	3189.3 (2787.6 to 3616.7)	376.6 (251.5 to 520.9)	270.3 (126.6 to 519.8)	5.6 (4.1 to 8.1)
Cuba	2045	1783.3 (1570.9 to 2006.8)	1480.0 (1295.8 to 1680.8)	174.7 (116.2 to 241.6)	126.0 (59.0 to 241.0)	2.6 (1.9 to 4.0)	3904.8 (3439.9 to 4394.3)	3240.8 (2837.3 to 3680.3)	382.4 (254.5 to 529.1)	275.8 (129.2 to 527.7)	5.8 (4.2 to 8.7)
Cuba	2046	1810.8 (1603.9 to 2039.9)	1502.4 (1316.6 to 1708.7)	177.2 (117.8 to 245.3)	128.5 (60.2 to 245.7)	2.7 (2.0 to 4.2)	3965.1 (3512.1 to 4466.6)	3289.7 (2883.0 to 3741.4)	388.0 (257.9 to 537.2)	281.3 (131.7 to 537.9)	6.0 (4.4 to 9.2)
Cuba	2047	1839.5 (1627.0 to 2076.0)	1525.8 (1337.4 to 1734.0)	179.8 (119.4 to 248.6)	131.1 (61.4 to 249.7)	2.9 (2.1 to 4.5)	4028.0 (3562.5 to 4545.7)	3341.0 (2928.5 to 3796.9)	393.7 (261.5 to 544.3)	287.0 (134.4 to 546.7)	6.3 (4.5 to 9.9)
Cuba	2048	1868.3 (1642.4 to 2112.6)	1549.2 (1359.9 to 1762.3)	182.4 (120.6 to 252.2)	133.7 (62.6 to 253.7)	3.0 (2.1 to 4.8)	4090.9 (3596.2 to 4626.0)	3392.4 (2977.7 to 3858.8)	399.3 (264.0 to 552.2)	292.7 (137.0 to 555.4)	6.5 (4.7 to 10.5)
Cuba	2049	1897.2 (1669.1 to 2144.0)	1572.9 (1379.4 to 1791.6)	185.0 (122.1 to 255.7)	136.2 (63.8 to 257.5)	3.1 (2.2 to 5.0)	4154.3 (3654.8 to 4694.7)	3444.2 (3020.5 to 3923.0)	405.0 (267.3 to 559.8)	298.3 (139.6 to 563.9)	6.8 (4.9 to 11.0)
Cuba	2050	1925.1 (1686.7 to 2180.1)	1595.7 (1395.1 to 1819.4)	187.5 (123.9 to 259.3)	138.7 (64.9 to 261.1)	3.3 (2.3 to 5.3)	4215.4 (3693.4 to 4773.6)	3494.0 (3054.8 to 3983.8)	410.5 (271.4 to 567.9)	303.7 (142.1 to 571.7)	7.1 (5.0 to 11.7)
Cyprus	1995	773.2 (722.8 to 833.6)	268.5 (240.1 to 298.7)	490.4 (443.4 to 541.1)	14.3 (8.5 to 22.2)	0.0 (0.0 to 0.0)	1079.8 (1009.5 to 1164.2)	375.0 (335.3 to 417.1)	684.8 (619.3 to 755.7)	20.0 (11.9 to 31.0)	0.0 (0.0 to 0.0)
Cyprus	1996	811.3 (768.4 to 858.3)	284.2 (259.5 to 310.5)	510.7 (471.6 to 549.9)	16.4 (10.2 to 25.7)	0.0 (0.0 to 0.0)	1133.1 (1073.1 to 1198.7)	396.9 (362.5 to 433.6)	713.3 (658.6 to 768.0)	22.9 (14.2 to 35.9)	0.0 (0.0 to 0.0)
Cyprus	1997	855.7 (815.1 to 898.6)	311.5 (288.5 to 336.4)	525.0 (491.2 to 558.5)	19.2 (12.3 to 29.4)	0.0 (0.0 to 0.0)	1195.1 (1138.3 to 1255.0)	435.0 (402.9 to 469.8)	733.2 (686.0 to 780.0)	26.8 (17.2 to 41.1)	0.0 (0.0 to 0.0)
Cyprus	1998	905.4 (865.5 to 947.0)	351.6 (329.3 to 376.5)	530.7 (498.5 to 564.3)	23.1 (15.1 to 34.2)	0.0 (0.0 to 0.0)	1264.4 (1208.7 to 1322.5)	491.1 (459.9 to 525.8)	741.1 (696.2 to 788.1)	32.2 (21.1 to 47.7)	0.0 (0.0 to 0.0)
Cyprus	1999	957.2 (917.1 to 996.9)	386.5 (364.5 to 410.2)	540.4 (509.2 to 573.1)	30.2 (20.8 to 42.8)	0.0 (0.0 to 0.0)	1336.7 (1280.8 to 1392.2)	539.8 (509.0 to 572.9)	754.7 (711.1 to 800.4)	42.2 (29.1 to 59.8)	0.0 (0.0 to 0.0)
Cyprus	2000	1019.7 (980.1 to 1061.3)	424.9 (402.8 to 449.1)	561.9 (530.1 to 594.2)	32.9 (23.6 to 45.0)	0.0 (0.0 to 0.0)	1424.1 (1368.9 to 1482.2)	593.4 (562.6 to 627.2)	784.8 (740.4 to 829.9)	45.9 (33.0 to 62.8)	0.0 (0.0 to 0.0)
Cyprus	2001	1069.9 (1029.5 to 1110.7)	456.6 (432.7 to 481.9)	575.3 (544.7 to 606.9)	38.0 (28.5 to 50.2)	0.0 (0.0 to 0.0)	1494.2 (1437.8 to 1551.1)	637.7 (604.4 to 673.1)	803.4 (760.8 to 847.6)	53.0 (39.8 to 70.2)	0.0 (0.0 to 0.0)
Cyprus	2002	1098.8 (1056.6 to 1138.5)	475.8 (449.7 to 500.4)	575.3 (544.1 to 607.9)	47.7 (37.3 to 60.7)	0.0 (0.0 to 0.0)	1534.6 (1475.6 to 1590.0)	664.5 (628.1 to 698.8)	803.5 (759.9 to 849.0)	66.7 (52.1 to 84.8)	0.0 (0.0 to 0.0)
Cyprus	2003	1097.9 (1056.4 to 1139.7)	465.9 (441.5 to 491.0)	560.1 (530.3 to 590.3)	71.9 (57.8 to 88.3)	0.0 (0.0 to 0.0)	1533.3 (1475.3 to 1591.7)	650.6 (616.6 to 685.7)	782.2 (740.6 to 824.3)	100.5 (80.7 to 123.3)	0.0 (0.0 to 0.0)
Cyprus	2004	1103.4 (1061.1 to 1145.6)	457.0 (432.2 to 481.7)	558.9 (529.5 to 589.1)	87.4 (72.1 to 104.8)	0.0 (0.0 to 0.0)	1541.0 (1481.8 to 1600.0)	638.3 (603.6 to 672.8)	780.6 (739.5 to 822.7)	122.1 (100.7 to 146.4)	0.0 (0.0 to 0.0)
Cyprus	2005	1112.0 (1070.5 to 1153.1)	455.0 (431.5 to 479.4)	563.2 (532.0 to 594.7)	93.9 (78.2 to 112.0)	0.0 (0.0 to 0.0)	1553.1 (1495.0 to 1610.4)	635.4 (602.6 to 669.5)	786.5 (743.0 to 830.6)	131.2 (109.2 to 156.4)	0.0 (0.0 to 0.0)
Cyprus	2006	1145.1 (1103.2 to 1187.3)	471.5 (447.5 to 496.8)	577.2 (546.0 to 608.2)	96.4 (80.8 to 114.2)	0.0 (0.0 to 0.0)	1599.2 (1540.7 to 1658.1)	658.5 (625.0 to 693.8)	806.1 (762.5 to 849.5)	134.6 (112.8 to 159.5)	0.0 (0.0 to 0.0)
Cyprus	2007	1202.1 (1160.6 to 1244.2)	501.8 (477.6 to 527.9)	604.4 (574.6 to 636.6)	95.9 (80.3 to 114.0)	0.0 (0.0 to 0.0)	1678.9 (1620.9 to 1737.6)	700.9 (667.0 to 737.3)	844.1 (802.4 to 889.1)	133.9 (112.1 to 159.3)	0.0 (0.0 to 0.0)
Cyprus	2008	1313.9 (1268.3 to 1359.5)	560.1 (533.6 to 587.4)	652.4 (620.1 to 687.2)	101.4 (84.3 to 120.6)	0.0 (0.0 to 0.0)	1835.0 (1771.3 to 1898.7)	782.2 (745.2 to 820.3)	911.2 (866.0 to 959.7)	141.6 (117.7 to 168.4)	0.0 (0.0 to 0.0)

Country	Year	2018 US Dollars per capita					2018 PPP per capita				
		Health spending	Government health spending	Out-of-pocket health spending	Prepaid private health spending	Development assistance for health	Health spending	Government health spending	Out-of-pocket health spending	Prepaid private health spending	Development assistance for health
Cyprus	2009	1351.2 (1304.1 to 1394.5)	594.0 (566.7 to 622.1)	648.1 (616.0 to 681.3)	109.1 (91.3 to 128.3)	0.0 (0.0 to 0.0)	1887.0 (1821.2 to 1947.5)	829.6 (791.5 to 868.8)	905.1 (860.3 to 951.5)	152.4 (127.5 to 179.1)	0.0 (0.0 to 0.0)
Cyprus	2010	1371.0 (1324.4 to 1413.2)	633.0 (605.6 to 662.8)	613.1 (581.3 to 644.3)	124.9 (105.2 to 145.1)	0.0 (0.0 to 0.0)	1914.8 (1849.6 to 1973.6)	884.1 (845.7 to 925.7)	856.2 (811.8 to 899.8)	174.5 (146.9 to 202.6)	0.0 (0.0 to 0.0)
Cyprus	2011	1369.5 (1324.4 to 1413.3)	640.0 (612.8 to 668.2)	597.5 (566.5 to 629.7)	131.9 (112.2 to 152.4)	0.0 (0.0 to 0.0)	1912.6 (1849.7 to 1973.8)	893.8 (855.8 to 933.1)	834.5 (791.2 to 879.5)	184.2 (156.7 to 212.8)	0.0 (0.0 to 0.0)
Cyprus	2012	1326.5 (1282.8 to 1369.2)	615.1 (588.9 to 641.0)	578.1 (546.1 to 608.7)	133.3 (114.6 to 153.3)	0.0 (0.0 to 0.0)	1852.6 (1791.6 to 1912.2)	859.1 (822.5 to 895.2)	807.3 (762.7 to 850.1)	186.2 (160.1 to 214.1)	0.0 (0.0 to 0.0)
Cyprus	2013	1253.9 (1209.8 to 1297.4)	571.5 (545.9 to 597.6)	551.2 (522.1 to 579.8)	131.2 (113.4 to 151.8)	0.0 (0.0 to 0.0)	1751.2 (1689.6 to 1812.0)	798.2 (762.4 to 834.6)	769.7 (729.1 to 809.8)	183.3 (158.4 to 212.0)	0.0 (0.0 to 0.0)
Cyprus	2014	1211.1 (1168.0 to 1254.5)	538.2 (511.5 to 564.8)	540.0 (511.3 to 571.2)	132.9 (115.0 to 153.0)	0.0 (0.0 to 0.0)	1691.3 (1631.2 to 1752.0)	751.6 (714.4 to 788.8)	754.2 (714.1 to 797.7)	185.5 (160.6 to 213.7)	0.0 (0.0 to 0.0)
Cyprus	2015	1212.8 (1162.6 to 1261.5)	527.2 (498.3 to 556.6)	544.4 (512.6 to 579.1)	141.2 (119.0 to 165.6)	0.0 (0.0 to 0.0)	1693.8 (1623.6 to 1761.8)	736.3 (696.0 to 777.4)	760.2 (715.9 to 808.7)	197.2 (166.2 to 231.2)	0.0 (0.0 to 0.0)
Cyprus	2016	1226.1 (1161.3 to 1292.8)	524.8 (489.1 to 562.5)	555.0 (513.3 to 601.8)	146.3 (119.1 to 176.1)	0.0 (0.0 to 0.0)	1712.4 (1621.9 to 1805.4)	732.9 (683.1 to 785.6)	775.1 (716.9 to 840.4)	204.3 (166.3 to 246.0)	0.0 (0.0 to 0.0)
Cyprus	2017	1270.1 (1203.0 to 1346.5)	546.3 (509.0 to 584.9)	571.9 (529.6 to 621.7)	152.0 (123.4 to 183.1)	0.0 (0.0 to 0.0)	1773.9 (1680.0 to 1880.5)	763.0 (710.8 to 816.8)	798.6 (739.7 to 868.3)	212.3 (172.4 to 255.7)	0.0 (0.0 to 0.0)
Cyprus	2018	1288.8 (1219.3 to 1361.2)	556.8 (518.5 to 597.0)	577.2 (533.5 to 625.5)	154.8 (125.6 to 186.3)	0.0 (0.0 to 0.0)	1800.0 (1702.9 to 1901.0)	777.7 (724.1 to 833.7)	806.1 (745.1 to 873.5)	216.1 (175.4 to 260.2)	0.0 (0.0 to 0.0)
Cyprus	2019	1307.0 (1237.2 to 1385.5)	567.5 (528.8 to 609.2)	582.1 (537.9 to 630.6)	157.4 (127.6 to 189.1)	0.0 (0.0 to 0.0)	1825.3 (1727.9 to 1934.9)	792.6 (738.5 to 850.8)	812.9 (751.2 to 880.6)	219.9 (178.2 to 264.1)	0.0 (0.0 to 0.0)
Cyprus	2020	1327.2 (1253.1 to 1402.7)	579.3 (538.7 to 621.4)	587.5 (541.4 to 638.4)	160.3 (129.9 to 193.1)	0.0 (0.0 to 0.0)	1853.5 (1750.1 to 1959.0)	809.1 (752.3 to 867.8)	820.6 (756.1 to 891.6)	223.9 (181.5 to 269.7)	0.0 (0.0 to 0.0)
Cyprus	2021	1345.2 (1274.2 to 1424.8)	590.1 (546.3 to 633.7)	592.1 (544.7 to 643.5)	163.0 (132.1 to 196.5)	0.0 (0.0 to 0.0)	1878.7 (1779.5 to 1989.8)	824.1 (763.0 to 885.1)	827.0 (760.7 to 898.8)	227.7 (184.4 to 274.5)	0.0 (0.0 to 0.0)
Cyprus	2022	1363.1 (1286.3 to 1441.6)	601.4 (556.3 to 647.6)	596.1 (548.6 to 647.5)	165.7 (134.1 to 199.6)	0.0 (0.0 to 0.0)	1903.7 (1796.4 to 2013.3)	839.8 (776.9 to 904.4)	832.4 (766.1 to 904.3)	231.4 (187.3 to 278.8)	0.0 (0.0 to 0.0)
Cyprus	2023	1379.9 (1302.2 to 1465.1)	611.9 (564.1 to 660.4)	599.8 (551.9 to 653.1)	168.3 (136.1 to 203.1)	0.0 (0.0 to 0.0)	1927.1 (1818.6 to 2046.1)	854.5 (787.7 to 922.3)	837.6 (770.8 to 912.1)	235.0 (190.1 to 283.6)	0.0 (0.0 to 0.0)
Cyprus	2024	1396.7 (1316.3 to 1477.3)	622.4 (573.9 to 673.5)	603.5 (554.0 to 657.6)	170.8 (138.3 to 206.5)	0.0 (0.0 to 0.0)	1950.6 (1838.3 to 2063.1)	869.3 (801.5 to 940.5)	842.8 (773.7 to 918.4)	238.6 (193.1 to 288.4)	0.0 (0.0 to 0.0)
Cyprus	2025	1411.2 (1332.3 to 1499.0)	632.1 (582.6 to 686.0)	606.0 (555.4 to 662.2)	173.1 (140.0 to 209.6)	0.0 (0.0 to 0.0)	1970.9 (1860.7 to 2093.5)	882.8 (813.6 to 958.1)	846.3 (775.7 to 924.8)	241.8 (195.5 to 292.7)	0.0 (0.0 to 0.0)
Cyprus	2026	1424.5 (1340.0 to 1509.1)	641.1 (590.8 to 696.5)	608.1 (557.3 to 665.0)	175.3 (141.8 to 212.6)	0.0 (0.0 to 0.0)	1989.4 (1871.5 to 2107.5)	895.3 (825.1 to 972.8)	849.3 (778.3 to 928.8)	244.8 (198.1 to 296.9)	0.0 (0.0 to 0.0)
Cyprus	2027	1436.3 (1352.4 to 1528.3)	649.4 (596.3 to 707.5)	609.7 (558.2 to 667.1)	177.3 (143.4 to 215.4)	0.0 (0.0 to 0.0)	2005.9 (1888.7 to 2134.3)	906.9 (832.7 to 988.0)	851.5 (779.5 to 931.7)	247.6 (200.3 to 300.8)	0.0 (0.0 to 0.0)
Cyprus	2028	1449.1 (1358.0 to 1539.9)	658.1 (602.4 to 718.9)	611.6 (558.9 to 672.0)	179.4 (145.1 to 218.3)	0.0 (0.0 to 0.0)	2023.7 (1896.5 to 2150.6)	919.1 (841.3 to 1004.0)	854.1 (780.5 to 938.5)	250.5 (202.6 to 304.9)	0.0 (0.0 to 0.0)
Cyprus	2029	1461.9 (1372.3 to 1559.2)	666.9 (608.1 to 731.5)	613.5 (558.2 to 674.1)	181.5 (146.8 to 220.9)	0.0 (0.0 to 0.0)	2041.6 (1916.5 to 2177.5)	931.3 (849.2 to 1021.6)	856.8 (779.6 to 941.4)	253.5 (205.0 to 308.5)	0.0 (0.0 to 0.0)

Country	Year	2018 US Dollars per capita					2018 PPP per capita				
		Health spending	Government health spending	Out-of-pocket health spending	Prepaid private health spending	Development assistance for health	Health spending	Government health spending	Out-of-pocket health spending	Prepaid private health spending	Development assistance for health
Cyprus	2030	1476.2 (1378.9 to 1575.5)	676.6 (614.6 to 744.6)	615.7 (559.6 to 681.1)	183.8 (148.6 to 223.0)	0.0 (0.0 to 0.0)	2061.6 (1925.7 to 2200.3)	945.0 (858.3 to 1039.8)	859.9 (781.6 to 951.2)	256.7 (207.5 to 311.5)	0.0 (0.0 to 0.0)
Cyprus	2031	1490.1 (1396.3 to 1597.0)	686.1 (622.2 to 758.4)	617.9 (560.5 to 685.0)	186.1 (150.3 to 226.0)	0.0 (0.0 to 0.0)	2081.0 (1950.0 to 2230.4)	958.2 (868.9 to 1059.1)	862.9 (782.8 to 956.6)	259.9 (210.0 to 315.6)	0.0 (0.0 to 0.0)
Cyprus	2032	1505.6 (1403.1 to 1611.4)	696.4 (627.6 to 772.7)	620.6 (561.4 to 687.9)	188.5 (152.3 to 229.2)	0.0 (0.0 to 0.0)	2102.7 (1959.5 to 2250.4)	972.6 (876.5 to 1079.1)	866.8 (784.1 to 960.8)	263.3 (212.7 to 320.1)	0.0 (0.0 to 0.0)
Cyprus	2033	1520.1 (1419.3 to 1632.9)	706.2 (636.1 to 784.2)	623.0 (562.1 to 693.4)	190.9 (154.2 to 232.1)	0.0 (0.0 to 0.0)	2122.9 (1982.1 to 2280.5)	986.3 (888.3 to 1095.2)	870.0 (785.1 to 968.4)	266.6 (215.4 to 324.1)	0.0 (0.0 to 0.0)
Cyprus	2034	1535.8 (1424.0 to 1644.4)	716.7 (642.1 to 796.7)	625.7 (562.1 to 698.4)	193.4 (156.1 to 234.6)	0.0 (0.0 to 0.0)	2144.9 (1988.7 to 2296.5)	1001.0 (896.8 to 1112.6)	873.8 (785.0 to 975.4)	270.1 (218.1 to 327.7)	0.0 (0.0 to 0.0)
Cyprus	2035	1552.7 (1444.1 to 1671.7)	727.9 (649.7 to 812.2)	628.8 (564.4 to 703.4)	196.0 (157.9 to 238.2)	0.0 (0.0 to 0.0)	2168.4 (2016.8 to 2334.6)	1016.5 (907.3 to 1134.3)	878.1 (788.3 to 982.3)	273.8 (220.5 to 332.7)	0.0 (0.0 to 0.0)
Cyprus	2036	1567.4 (1447.5 to 1684.9)	737.7 (657.6 to 825.2)	631.2 (566.3 to 707.4)	198.5 (159.7 to 241.5)	0.0 (0.0 to 0.0)	2189.1 (2021.5 to 2353.1)	1030.3 (918.4 to 1152.5)	881.6 (790.9 to 988.0)	277.2 (223.0 to 337.3)	0.0 (0.0 to 0.0)
Cyprus	2037	1581.7 (1464.6 to 1713.0)	747.3 (662.1 to 837.4)	633.5 (568.0 to 710.6)	200.9 (161.9 to 244.7)	0.0 (0.0 to 0.0)	2209.0 (2045.4 to 2392.4)	1043.7 (924.6 to 1169.5)	884.8 (793.3 to 992.4)	280.5 (226.1 to 341.7)	0.0 (0.0 to 0.0)
Cyprus	2038	1596.3 (1470.1 to 1723.6)	757.1 (671.5 to 852.5)	635.9 (568.7 to 713.8)	203.3 (163.9 to 248.1)	0.0 (0.0 to 0.0)	2229.3 (2053.1 to 2407.1)	1057.4 (937.9 to 1190.6)	888.0 (794.2 to 996.8)	284.0 (229.0 to 346.5)	0.0 (0.0 to 0.0)
Cyprus	2039	1610.6 (1480.9 to 1748.4)	766.9 (677.1 to 866.7)	637.9 (568.9 to 718.6)	205.7 (165.6 to 251.5)	0.0 (0.0 to 0.0)	2249.3 (2068.2 to 2441.8)	1071.1 (945.6 to 1210.5)	890.9 (794.5 to 1003.6)	287.3 (231.3 to 351.2)	0.0 (0.0 to 0.0)
Cyprus	2040	1624.9 (1491.0 to 1762.2)	777.0 (683.9 to 881.4)	639.9 (569.4 to 722.3)	208.1 (167.2 to 255.0)	0.0 (0.0 to 0.0)	2269.3 (2082.2 to 2461.1)	1085.1 (955.1 to 1231.0)	893.6 (795.2 to 1008.8)	290.6 (233.5 to 356.1)	0.0 (0.0 to 0.0)
Cyprus	2041	1637.5 (1509.0 to 1788.2)	786.0 (688.0 to 891.6)	641.2 (569.6 to 724.5)	210.3 (168.9 to 258.2)	0.0 (0.0 to 0.0)	2286.9 (2107.5 to 2497.4)	1097.7 (960.9 to 1245.2)	895.5 (795.5 to 1011.8)	293.7 (235.9 to 360.7)	0.0 (0.0 to 0.0)
Cyprus	2042	1649.1 (1508.2 to 1795.1)	794.7 (693.9 to 907.5)	642.1 (569.4 to 725.7)	212.3 (170.4 to 261.4)	0.0 (0.0 to 0.0)	2303.2 (2106.4 to 2507.1)	1109.8 (969.1 to 1267.4)	896.8 (795.2 to 1013.4)	296.5 (238.0 to 365.0)	0.0 (0.0 to 0.0)
Cyprus	2043	1660.8 (1524.7 to 1821.4)	803.5 (698.7 to 920.4)	642.9 (568.8 to 727.6)	214.4 (171.8 to 264.4)	0.0 (0.0 to 0.0)	2319.4 (2129.4 to 2543.7)	1122.2 (975.7 to 1285.4)	897.8 (794.4 to 1016.2)	299.4 (240.0 to 369.2)	0.0 (0.0 to 0.0)
Cyprus	2044	1673.6 (1523.5 to 1829.4)	813.1 (703.8 to 934.0)	644.0 (568.7 to 730.0)	216.5 (173.3 to 267.2)	0.0 (0.0 to 0.0)	2337.4 (2127.7 to 2555.0)	1135.5 (983.0 to 1304.4)	899.4 (794.3 to 1019.5)	302.4 (242.0 to 373.2)	0.0 (0.0 to 0.0)
Cyprus	2045	1686.7 (1544.0 to 1856.9)	822.8 (711.3 to 951.0)	645.2 (568.9 to 732.6)	218.7 (174.8 to 270.1)	0.0 (0.0 to 0.0)	2355.7 (2156.3 to 2593.4)	1149.1 (993.3 to 1328.2)	901.1 (794.5 to 1023.1)	305.5 (244.2 to 377.2)	0.0 (0.0 to 0.0)
Cyprus	2046	1698.2 (1540.0 to 1862.2)	831.4 (714.5 to 961.1)	646.0 (569.2 to 736.4)	220.8 (175.9 to 273.5)	0.0 (0.0 to 0.0)	2371.7 (2150.7 to 2600.8)	1161.1 (997.9 to 1342.2)	902.3 (794.9 to 1028.5)	308.4 (245.7 to 382.0)	0.0 (0.0 to 0.0)
Cyprus	2047	1709.5 (1557.5 to 1884.3)	839.9 (721.3 to 972.4)	646.7 (569.0 to 739.2)	222.8 (177.2 to 276.3)	0.0 (0.0 to 0.0)	2387.4 (2175.1 to 2631.5)	1173.0 (1007.3 to 1358.0)	903.2 (794.6 to 1032.4)	311.2 (247.4 to 385.9)	0.0 (0.0 to 0.0)
Cyprus	2048	1721.1 (1560.5 to 1894.9)	848.7 (728.0 to 988.6)	647.5 (568.9 to 741.9)	224.9 (178.5 to 279.7)	0.0 (0.0 to 0.0)	2403.7 (2179.4 to 2646.4)	1185.3 (1016.7 to 1380.7)	904.2 (794.6 to 1036.1)	314.1 (249.2 to 390.7)	0.0 (0.0 to 0.0)
Cyprus	2049	1733.2 (1575.0 to 1921.4)	857.9 (731.9 to 1000.6)	648.3 (569.0 to 742.7)	227.1 (179.8 to 283.6)	0.0 (0.0 to 0.0)	2420.6 (2199.7 to 2683.4)	1198.2 (1022.2 to 1397.5)	905.3 (794.6 to 1037.3)	317.1 (251.1 to 396.1)	0.0 (0.0 to 0.0)
Cyprus	2050	1744.1 (1572.7 to 1934.7)	866.6 (737.5 to 1015.0)	648.6 (568.7 to 747.0)	229.0 (180.8 to 287.1)	0.0 (0.0 to 0.0)	2435.8 (2196.4 to 2702.0)	1210.2 (1030.0 to 1417.6)	905.8 (794.3 to 1043.3)	319.9 (252.5 to 401.0)	0.0 (0.0 to 0.0)

Country	Year	2018 US Dollars per capita					2018 PPP per capita				
		Health spending	Government health spending	Out-of-pocket health spending	Prepaid private health spending	Development assistance for health	Health spending	Government health spending	Out-of-pocket health spending	Prepaid private health spending	Development assistance for health
Czech Republic	1995	779.7 (730.1 to 834.1)	688.7 (643.2 to 741.2)	77.0 (62.8 to 93.6)	14.0 (7.3 to 23.6)	0.0 (0.0 to 0.0)	1292.0 (1209.8 to 1382.2)	1141.2 (1065.8 to 1228.2)	127.6 (104.1 to 155.1)	23.2 (12.2 to 39.1)	0.0 (0.0 to 0.0)
Czech Republic	1996	784.2 (744.2 to 823.8)	691.8 (656.8 to 728.9)	78.3 (64.3 to 94.3)	14.1 (7.4 to 23.1)	0.0 (0.0 to 0.0)	1299.3 (1233.1 to 1365.0)	1146.2 (1088.3 to 1207.8)	129.7 (106.6 to 156.3)	23.4 (12.3 to 38.2)	0.0 (0.0 to 0.0)
Czech Republic	1997	768.3 (729.7 to 803.5)	675.8 (644.7 to 708.3)	78.5 (65.2 to 92.6)	13.9 (7.5 to 22.3)	0.0 (0.0 to 0.0)	1273.0 (1209.1 to 1331.4)	1119.8 (1068.2 to 1173.6)	130.1 (108.1 to 153.5)	23.1 (12.5 to 36.9)	0.0 (0.0 to 0.0)
Czech Republic	1998	766.8 (732.4 to 801.9)	674.1 (641.5 to 706.6)	78.8 (66.1 to 91.7)	13.8 (7.7 to 22.4)	0.0 (0.0 to 0.0)	1270.5 (1213.5 to 1328.7)	1117.0 (1062.9 to 1170.9)	130.6 (109.6 to 151.9)	22.9 (12.8 to 37.1)	0.0 (0.0 to 0.0)
Czech Republic	1999	780.2 (744.8 to 815.5)	686.3 (653.2 to 718.6)	80.2 (68.1 to 92.8)	13.7 (7.8 to 21.7)	0.0 (0.0 to 0.0)	1292.8 (1234.2 to 1351.3)	1137.3 (1082.3 to 1190.7)	132.8 (112.8 to 153.8)	22.7 (12.9 to 35.9)	0.0 (0.0 to 0.0)
Czech Republic	2000	825.7 (788.2 to 864.0)	727.6 (694.3 to 762.6)	84.4 (72.1 to 98.0)	13.8 (7.9 to 21.5)	0.0 (0.0 to 0.0)	1368.2 (1306.0 to 1431.6)	1205.6 (1150.5 to 1263.6)	139.8 (119.4 to 162.4)	22.8 (13.1 to 35.6)	0.0 (0.0 to 0.0)
Czech Republic	2001	887.9 (851.0 to 926.6)	780.2 (747.1 to 816.1)	90.9 (78.2 to 105.2)	16.8 (9.7 to 26.6)	0.0 (0.0 to 0.0)	1471.3 (1410.0 to 1535.4)	1292.8 (1237.9 to 1352.2)	150.7 (129.7 to 174.3)	27.8 (16.0 to 44.0)	0.0 (0.0 to 0.0)
Czech Republic	2002	943.0 (906.7 to 982.1)	827.1 (795.2 to 863.0)	96.5 (83.4 to 111.0)	19.4 (11.7 to 30.1)	0.0 (0.0 to 0.0)	1562.5 (1502.3 to 1627.3)	1370.4 (1317.7 to 1429.9)	159.9 (138.2 to 183.9)	32.1 (19.3 to 49.9)	0.0 (0.0 to 0.0)
Czech Republic	2003	1003.5 (966.9 to 1041.6)	876.2 (843.3 to 911.9)	105.0 (91.1 to 120.3)	22.3 (14.0 to 33.5)	0.0 (0.0 to 0.0)	1662.8 (1602.1 to 1725.9)	1451.8 (1397.3 to 1511.1)	174.0 (150.9 to 199.3)	37.0 (23.2 to 55.6)	0.0 (0.0 to 0.0)
Czech Republic	2004	1050.2 (1012.2 to 1090.8)	911.4 (877.7 to 948.1)	114.2 (100.1 to 129.7)	24.6 (16.0 to 36.2)	0.0 (0.0 to 0.0)	1740.2 (1677.2 to 1807.5)	1510.2 (1454.3 to 1570.9)	189.2 (165.8 to 214.9)	40.8 (26.5 to 59.9)	0.0 (0.0 to 0.0)
Czech Republic	2005	1093.4 (1052.5 to 1134.9)	936.2 (900.3 to 971.3)	126.5 (112.1 to 142.4)	30.6 (20.8 to 44.0)	0.0 (0.0 to 0.0)	1811.7 (1744.1 to 1880.5)	1551.3 (1491.8 to 1609.5)	209.6 (185.7 to 236.0)	50.8 (34.4 to 72.9)	0.0 (0.0 to 0.0)
Czech Republic	2006	1142.8 (1100.5 to 1180.5)	968.5 (933.9 to 1002.5)	142.1 (127.2 to 157.8)	32.1 (21.6 to 46.8)	0.0 (0.0 to 0.0)	1893.6 (1823.4 to 1956.1)	1604.8 (1547.5 to 1661.2)	235.5 (210.7 to 261.5)	53.2 (35.8 to 77.5)	0.0 (0.0 to 0.0)
Czech Republic	2007	1208.6 (1169.1 to 1247.5)	1011.4 (978.5 to 1044.8)	164.7 (149.0 to 181.0)	32.5 (21.3 to 46.2)	0.0 (0.0 to 0.0)	2002.6 (1937.2 to 2067.1)	1675.9 (1621.4 to 1731.3)	272.9 (246.8 to 300.0)	53.8 (35.4 to 76.6)	0.0 (0.0 to 0.0)
Czech Republic	2008	1275.2 (1235.0 to 1314.4)	1053.3 (1019.5 to 1086.7)	189.3 (172.3 to 206.2)	32.6 (21.5 to 45.7)	0.0 (0.0 to 0.0)	2113.0 (2046.5 to 2177.9)	1745.3 (1689.3 to 1800.6)	313.6 (285.6 to 341.7)	54.0 (35.7 to 75.7)	0.0 (0.0 to 0.0)
Czech Republic	2009	1310.7 (1270.2 to 1351.3)	1081.1 (1047.3 to 1116.7)	200.2 (183.1 to 217.3)	29.4 (19.7 to 41.4)	0.0 (0.0 to 0.0)	2171.9 (2104.7 to 2239.1)	1791.3 (1735.3 to 1850.4)	331.7 (303.5 to 360.1)	48.8 (32.6 to 68.6)	0.0 (0.0 to 0.0)
Czech Republic	2010	1341.4 (1301.4 to 1383.1)	1110.9 (1078.0 to 1146.0)	204.4 (188.5 to 221.6)	26.1 (17.8 to 36.8)	0.0 (0.0 to 0.0)	2222.7 (2156.3 to 2291.7)	1840.7 (1786.3 to 1898.9)	338.7 (312.3 to 367.2)	43.2 (29.6 to 60.9)	0.0 (0.0 to 0.0)
Czech Republic	2011	1386.4 (1345.1 to 1426.9)	1154.0 (1119.2 to 1188.4)	207.5 (191.5 to 225.0)	24.9 (17.1 to 35.3)	0.0 (0.0 to 0.0)	2297.2 (2228.8 to 2364.3)	1912.1 (1854.5 to 1969.2)	343.8 (317.4 to 372.8)	41.3 (28.3 to 58.5)	0.0 (0.0 to 0.0)
Czech Republic	2012	1404.6 (1361.9 to 1445.8)	1170.5 (1134.3 to 1206.6)	207.5 (190.6 to 225.0)	26.7 (18.4 to 37.1)	0.0 (0.0 to 0.0)	2327.5 (2256.6 to 2395.7)	1939.4 (1879.6 to 1999.4)	343.8 (315.9 to 372.8)	44.3 (30.5 to 61.4)	0.0 (0.0 to 0.0)
Czech Republic	2013	1455.4 (1414.8 to 1499.3)	1208.7 (1173.0 to 1248.3)	209.8 (192.7 to 227.1)	36.9 (25.2 to 51.2)	0.0 (0.0 to 0.0)	2411.5 (2344.3 to 2484.4)	2002.9 (1943.6 to 2068.5)	347.6 (319.4 to 376.4)	61.1 (41.8 to 84.9)	0.0 (0.0 to 0.0)
Czech Republic	2014	1488.8 (1447.0 to 1532.4)	1230.7 (1194.2 to 1266.9)	214.3 (196.9 to 233.9)	43.8 (30.0 to 60.8)	0.0 (0.0 to 0.0)	2467.0 (2397.6 to 2539.2)	2039.3 (1978.7 to 2099.2)	355.1 (326.3 to 387.6)	72.6 (49.7 to 100.8)	0.0 (0.0 to 0.0)
Czech Republic	2015	1515.1 (1471.0 to 1565.0)	1247.8 (1209.3 to 1285.8)	220.8 (201.6 to 243.6)	46.5 (31.7 to 65.0)	0.0 (0.0 to 0.0)	2510.4 (2437.5 to 2593.2)	2067.6 (2003.9 to 2130.6)	365.9 (334.0 to 403.6)	77.0 (52.6 to 107.7)	0.0 (0.0 to 0.0)

		2018 US Dollars per capita					2018 PPP per capita				
Country	Year	Health spending	Government health spending	Out-of-pocket health spending	Prepaid private health spending	Development assistance for health	Health spending	Government health spending	Out-of-pocket health spending	Prepaid private health spending	Development assistance for health
Czech Republic	2016	1515.1 (1456.6 to 1578.5)	1242.3 (1192.9 to 1298.0)	224.9 (201.0 to 252.1)	47.9 (31.6 to 69.5)	0.0 (0.0 to 0.0)	2510.6 (2413.5 to 2615.5)	2058.5 (1976.7 to 2150.8)	372.7 (333.0 to 417.8)	79.4 (52.4 to 115.1)	0.0 (0.0 to 0.0)
Czech Republic	2017	1586.5 (1524.8 to 1654.2)	1302.1 (1248.9 to 1360.0)	234.2 (208.9 to 262.3)	50.2 (33.1 to 72.7)	0.0 (0.0 to 0.0)	2628.7 (2526.6 to 2741.0)	2157.6 (2069.4 to 2253.5)	388.0 (346.2 to 434.7)	83.1 (54.9 to 120.5)	0.0 (0.0 to 0.0)
Czech Republic	2018	1616.8 (1551.8 to 1687.5)	1325.6 (1267.9 to 1385.2)	240.2 (214.3 to 268.7)	51.0 (33.7 to 74.0)	0.0 (0.0 to 0.0)	2679.1 (2571.3 to 2796.2)	2196.5 (2100.9 to 2295.3)	398.0 (355.0 to 445.3)	84.6 (55.9 to 122.6)	0.0 (0.0 to 0.0)
Czech Republic	2019	1640.6 (1574.0 to 1716.5)	1342.9 (1281.7 to 1407.7)	245.9 (219.4 to 276.7)	51.8 (34.2 to 75.1)	0.0 (0.0 to 0.0)	2718.4 (2608.1 to 2844.2)	2225.1 (2123.8 to 2332.6)	407.5 (363.5 to 458.5)	85.8 (56.6 to 124.4)	0.0 (0.0 to 0.0)
Czech Republic	2020	1668.2 (1596.2 to 1748.8)	1363.5 (1300.5 to 1432.1)	252.1 (223.2 to 283.7)	52.7 (34.7 to 76.3)	0.0 (0.0 to 0.0)	2764.2 (2644.9 to 2897.7)	2259.3 (2154.9 to 2372.9)	417.6 (369.9 to 470.1)	87.2 (57.5 to 126.4)	0.0 (0.0 to 0.0)
Czech Republic	2021	1695.2 (1619.3 to 1778.9)	1383.4 (1316.7 to 1456.8)	258.3 (228.7 to 290.7)	53.6 (35.2 to 77.6)	0.0 (0.0 to 0.0)	2808.9 (2683.1 to 2947.7)	2292.3 (2181.8 to 2414.0)	427.9 (378.9 to 481.7)	88.7 (58.4 to 128.6)	0.0 (0.0 to 0.0)
Czech Republic	2022	1724.6 (1642.6 to 1814.7)	1405.1 (1333.3 to 1483.8)	264.8 (233.3 to 299.1)	54.6 (35.9 to 79.1)	0.0 (0.0 to 0.0)	2857.6 (2721.7 to 3006.9)	2328.3 (2209.3 to 2458.6)	438.8 (386.5 to 495.6)	90.5 (59.5 to 131.0)	0.0 (0.0 to 0.0)
Czech Republic	2023	1755.3 (1669.8 to 1848.9)	1427.8 (1353.6 to 1510.0)	271.7 (240.0 to 308.1)	55.8 (36.6 to 80.7)	0.0 (0.0 to 0.0)	2908.5 (2766.8 to 3063.7)	2365.8 (2242.9 to 2502.0)	450.3 (397.7 to 510.5)	92.4 (60.7 to 133.8)	0.0 (0.0 to 0.0)
Czech Republic	2024	1788.6 (1698.7 to 1887.5)	1452.8 (1371.3 to 1543.4)	278.9 (247.4 to 315.5)	56.9 (37.4 to 82.4)	0.0 (0.0 to 0.0)	2963.7 (2814.7 to 3127.5)	2407.2 (2272.2 to 2557.4)	462.1 (410.0 to 522.7)	94.4 (62.0 to 136.5)	0.0 (0.0 to 0.0)
Czech Republic	2025	1824.1 (1726.3 to 1930.5)	1479.6 (1394.0 to 1574.4)	286.3 (254.0 to 324.5)	58.2 (38.3 to 84.1)	0.0 (0.0 to 0.0)	3022.5 (2860.5 to 3198.8)	2451.8 (2309.8 to 2608.8)	474.4 (420.8 to 537.7)	96.4 (63.5 to 139.4)	0.0 (0.0 to 0.0)
Czech Republic	2026	1861.4 (1759.0 to 1975.0)	1508.0 (1413.7 to 1610.5)	294.0 (260.8 to 335.4)	59.4 (39.1 to 85.9)	0.0 (0.0 to 0.0)	3084.2 (2914.7 to 3272.6)	2498.7 (2342.6 to 2668.7)	487.1 (432.1 to 555.8)	98.5 (64.9 to 142.3)	0.0 (0.0 to 0.0)
Czech Republic	2027	1902.0 (1791.5 to 2021.6)	1539.2 (1439.2 to 1646.9)	302.0 (265.7 to 344.9)	60.8 (40.1 to 87.7)	0.0 (0.0 to 0.0)	3151.7 (2968.5 to 3349.8)	2550.5 (2384.8 to 2728.8)	500.5 (440.3 to 571.5)	100.7 (66.4 to 145.3)	0.0 (0.0 to 0.0)
Czech Republic	2028	1942.2 (1827.7 to 2068.1)	1569.8 (1462.3 to 1684.7)	310.2 (270.8 to 355.7)	62.2 (41.1 to 89.7)	0.0 (0.0 to 0.0)	3218.2 (3028.6 to 3426.9)	2601.2 (2423.0 to 2791.5)	514.0 (448.7 to 589.4)	103.0 (68.1 to 148.6)	0.0 (0.0 to 0.0)
Czech Republic	2029	1981.9 (1857.3 to 2116.5)	1599.9 (1488.5 to 1720.7)	318.4 (276.2 to 367.9)	63.6 (42.2 to 91.8)	0.0 (0.0 to 0.0)	3284.1 (3077.6 to 3507.1)	2651.1 (2466.5 to 2851.3)	527.6 (457.7 to 609.6)	105.4 (69.9 to 152.1)	0.0 (0.0 to 0.0)
Czech Republic	2030	2021.6 (1891.1 to 2161.7)	1629.9 (1506.5 to 1757.0)	326.6 (282.4 to 380.1)	65.1 (43.2 to 93.9)	0.0 (0.0 to 0.0)	3349.8 (3133.5 to 3582.0)	2700.8 (2496.3 to 2911.3)	541.2 (467.9 to 629.8)	107.8 (71.6 to 155.6)	0.0 (0.0 to 0.0)
Czech Republic	2031	2062.1 (1923.4 to 2212.0)	1660.6 (1530.4 to 1794.0)	334.9 (287.3 to 391.8)	66.5 (44.1 to 96.1)	0.0 (0.0 to 0.0)	3416.9 (3187.1 to 3665.2)	2751.6 (2535.8 to 2972.7)	555.0 (476.0 to 649.1)	110.3 (73.1 to 159.2)	0.0 (0.0 to 0.0)
Czech Republic	2032	2103.0 (1958.1 to 2261.0)	1691.6 (1558.5 to 1831.9)	343.3 (292.2 to 403.0)	68.1 (45.2 to 98.3)	0.0 (0.0 to 0.0)	3484.6 (3244.5 to 3746.5)	2803.0 (2582.5 to 3035.4)	568.8 (484.2 to 667.8)	112.8 (74.8 to 163.0)	0.0 (0.0 to 0.0)
Czech Republic	2033	2143.2 (1988.9 to 2312.7)	1722.1 (1576.1 to 1876.6)	351.6 (297.6 to 416.1)	69.6 (46.1 to 100.6)	0.0 (0.0 to 0.0)	3551.3 (3295.6 to 3832.1)	2853.4 (2611.6 to 3109.4)	582.6 (493.2 to 689.4)	115.3 (76.3 to 166.7)	0.0 (0.0 to 0.0)
Czech Republic	2034	2183.2 (2018.6 to 2359.5)	1752.3 (1599.7 to 1912.2)	359.8 (303.1 to 428.6)	71.1 (47.1 to 102.8)	0.0 (0.0 to 0.0)	3617.5 (3344.8 to 3909.6)	2903.5 (2650.6 to 3168.4)	596.2 (502.2 to 710.1)	117.8 (78.0 to 170.3)	0.0 (0.0 to 0.0)
Czech Republic	2035	2225.2 (2050.8 to 2407.9)	1784.5 (1619.4 to 1953.0)	368.2 (307.5 to 441.9)	72.6 (48.2 to 105.0)	0.0 (0.0 to 0.0)	3687.1 (3398.2 to 3989.8)	2956.8 (2683.4 to 3236.1)	610.1 (509.5 to 732.2)	120.3 (79.8 to 173.9)	0.0 (0.0 to 0.0)
Czech Republic	2036	2266.9 (2083.0 to 2458.5)	1816.5 (1647.9 to 1991.9)	376.4 (312.0 to 453.1)	74.0 (49.2 to 107.1)	0.0 (0.0 to 0.0)	3756.3 (3451.4 to 4073.6)	3009.9 (2730.6 to 3300.6)	623.7 (517.0 to 750.8)	122.6 (81.5 to 177.4)	0.0 (0.0 to 0.0)

Country	Year	2018 US Dollars per capita					2018 PPP per capita				
		Health spending	Government health spending	Out-of-pocket health spending	Prepaid private health spending	Development assistance for health	Health spending	Government health spending	Out-of-pocket health spending	Prepaid private health spending	Development assistance for health
Czech Republic	2037	2309.1 (2120.6 to 2515.3)	1849.0 (1671.9 to 2031.2)	384.6 (316.8 to 466.3)	75.4 (50.2 to 109.4)	0.0 (0.0 to 0.0)	3826.1 (3513.9 to 4167.8)	3063.8 (2770.3 to 3365.6)	637.3 (524.9 to 772.6)	125.0 (83.2 to 181.2)	0.0 (0.0 to 0.0)
Czech Republic	2038	2351.3 (2151.3 to 2560.5)	1881.7 (1694.8 to 2068.6)	392.7 (321.5 to 477.6)	76.9 (51.1 to 111.4)	0.0 (0.0 to 0.0)	3896.1 (3564.8 to 4242.7)	3118.0 (2808.2 to 3427.6)	650.7 (532.7 to 791.4)	127.3 (84.7 to 184.6)	0.0 (0.0 to 0.0)
Czech Republic	2039	2393.3 (2177.6 to 2621.3)	1914.4 (1716.1 to 2115.0)	400.8 (326.6 to 489.5)	78.2 (52.0 to 113.2)	0.0 (0.0 to 0.0)	3965.7 (3608.2 to 4343.4)	3172.1 (2843.6 to 3504.5)	664.0 (541.2 to 811.0)	129.6 (86.2 to 187.6)	0.0 (0.0 to 0.0)
Czech Republic	2040	2434.7 (2208.9 to 2678.4)	1946.5 (1744.2 to 2162.5)	408.7 (330.5 to 501.3)	79.6 (52.9 to 115.2)	0.0 (0.0 to 0.0)	4034.3 (3660.1 to 4438.0)	3225.3 (2890.1 to 3583.2)	677.1 (547.6 to 830.6)	131.9 (87.6 to 190.9)	0.0 (0.0 to 0.0)
Czech Republic	2041	2476.5 (2243.9 to 2744.1)	1979.2 (1766.7 to 2200.1)	416.4 (335.3 to 512.8)	80.9 (53.9 to 117.1)	0.0 (0.0 to 0.0)	4103.5 (3718.0 to 4547.0)	3279.4 (2927.4 to 3645.5)	690.0 (555.6 to 849.7)	134.1 (89.2 to 194.1)	0.0 (0.0 to 0.0)
Czech Republic	2042	2518.0 (2284.2 to 2785.5)	2011.7 (1783.9 to 2245.5)	424.1 (340.5 to 524.0)	82.2 (54.8 to 119.1)	0.0 (0.0 to 0.0)	4172.3 (3784.9 to 4615.6)	3333.4 (2955.8 to 3720.8)	702.7 (564.2 to 868.3)	136.2 (90.8 to 197.3)	0.0 (0.0 to 0.0)
Czech Republic	2043	2558.9 (2308.4 to 2846.6)	2043.8 (1802.5 to 2294.8)	431.6 (346.3 to 534.8)	83.5 (55.7 to 121.0)	0.0 (0.0 to 0.0)	4240.0 (3825.0 to 4716.8)	3386.5 (2986.7 to 3802.4)	715.1 (573.8 to 886.1)	138.4 (92.3 to 200.5)	0.0 (0.0 to 0.0)
Czech Republic	2044	2600.6 (2334.3 to 2876.4)	2076.6 (1822.1 to 2332.1)	439.0 (352.5 to 545.5)	84.9 (56.7 to 123.0)	0.0 (0.0 to 0.0)	4309.1 (3867.9 to 4766.2)	3441.0 (3019.1 to 3864.2)	727.5 (584.1 to 903.9)	140.7 (93.9 to 203.8)	0.0 (0.0 to 0.0)
Czech Republic	2045	2642.8 (2371.0 to 2928.5)	2110.0 (1852.4 to 2379.0)	446.4 (358.2 to 556.4)	86.4 (57.8 to 125.2)	0.0 (0.0 to 0.0)	4379.1 (3928.7 to 4852.5)	3496.2 (3069.4 to 3941.9)	739.7 (593.5 to 922.0)	143.2 (95.7 to 207.4)	0.0 (0.0 to 0.0)
Czech Republic	2046	2683.7 (2408.3 to 2990.1)	2142.2 (1868.3 to 2421.2)	453.6 (364.0 to 566.5)	87.9 (58.9 to 127.5)	0.0 (0.0 to 0.0)	4446.9 (3990.5 to 4954.5)	3549.6 (3095.8 to 4011.8)	751.6 (603.1 to 938.7)	145.7 (97.6 to 211.3)	0.0 (0.0 to 0.0)
Czech Republic	2047	2724.6 (2440.2 to 3053.7)	2174.4 (1886.9 to 2463.1)	460.6 (369.8 to 575.3)	89.5 (60.0 to 129.7)	0.0 (0.0 to 0.0)	4514.6 (4043.4 to 5059.9)	3603.0 (3126.6 to 4081.4)	763.3 (612.7 to 953.3)	148.3 (99.4 to 215.0)	0.0 (0.0 to 0.0)
Czech Republic	2048	2765.5 (2464.3 to 3086.3)	2206.6 (1916.2 to 2510.4)	467.7 (375.8 to 584.0)	91.2 (61.0 to 132.4)	0.0 (0.0 to 0.0)	4582.4 (4083.4 to 5113.9)	3656.3 (3175.1 to 4159.6)	775.0 (622.8 to 967.6)	151.0 (101.1 to 219.3)	0.0 (0.0 to 0.0)
Czech Republic	2049	2807.4 (2480.7 to 3135.7)	2239.7 (1930.2 to 2558.7)	474.9 (381.6 to 592.7)	92.8 (62.1 to 134.7)	0.0 (0.0 to 0.0)	4651.9 (4110.6 to 5195.8)	3711.2 (3198.4 to 4239.8)	786.9 (632.3 to 982.1)	153.8 (102.9 to 223.1)	0.0 (0.0 to 0.0)
Czech Republic	2050	2847.8 (2506.4 to 3188.0)	2271.3 (1952.5 to 2604.2)	482.0 (387.5 to 601.9)	94.5 (63.2 to 137.1)	0.0 (0.0 to 0.0)	4718.7 (4153.1 to 5282.4)	3763.6 (3235.3 to 4315.0)	798.6 (642.1 to 997.3)	156.5 (104.8 to 227.2)	0.0 (0.0 to 0.0)
Democratic Republic of the Congo	1995	12.9 (9.6 to 17.0)	0.9 (0.7 to 1.1)	10.0 (6.8 to 14.0)	1.6 (0.8 to 3.0)	0.4 (0.4 to 0.4)	20.1 (15.0 to 26.6)	1.4 (1.0 to 1.8)	15.7 (10.6 to 21.8)	2.5 (1.2 to 4.6)	0.6 (0.6 to 0.6)
Democratic Republic of the Congo	1996	12.9 (9.6 to 16.9)	0.8 (0.6 to 1.1)	9.7 (6.6 to 13.6)	1.5 (0.7 to 2.9)	0.8 (0.8 to 0.8)	20.1 (15.0 to 26.4)	1.3 (1.0 to 1.6)	15.1 (10.4 to 21.2)	2.4 (1.2 to 4.5)	1.3 (1.3 to 1.3)
Democratic Republic of the Congo	1997	12.4 (9.2 to 16.4)	0.8 (0.6 to 1.0)	9.5 (6.5 to 13.3)	1.6 (0.8 to 2.9)	0.5 (0.5 to 0.5)	19.3 (14.4 to 25.6)	1.2 (0.9 to 1.6)	14.9 (10.1 to 20.7)	2.4 (1.2 to 4.5)	0.8 (0.8 to 0.8)
Democratic Republic of the Congo	1998	12.1 (8.9 to 16.0)	0.7 (0.6 to 1.0)	9.3 (6.4 to 13.0)	1.6 (0.8 to 3.0)	0.5 (0.5 to 0.5)	18.8 (13.9 to 25.0)	1.2 (0.9 to 1.5)	14.4 (9.9 to 20.2)	2.5 (1.2 to 4.6)	0.8 (0.8 to 0.8)
Democratic Republic of the Congo	1999	11.7 (8.7 to 15.4)	0.7 (0.5 to 0.9)	8.8 (6.2 to 12.2)	1.7 (0.8 to 3.1)	0.5 (0.5 to 0.5)	18.3 (13.6 to 24.1)	1.1 (0.8 to 1.4)	13.8 (9.6 to 19.1)	2.6 (1.2 to 4.8)	0.8 (0.8 to 0.8)
Democratic Republic of the Congo	2000	10.5 (8.0 to 13.9)	0.6 (0.4 to 0.7)	7.8 (5.4 to 10.5)	1.6 (0.8 to 3.0)	0.6 (0.6 to 0.6)	16.4 (12.5 to 21.8)	0.9 (0.7 to 1.1)	12.1 (8.4 to 16.5)	2.6 (1.2 to 4.7)	0.9 (0.9 to 0.9)
Democratic Republic of the Congo	2001	9.4 (7.3 to 12.4)	0.5 (0.4 to 0.6)	7.0 (5.0 to 9.5)	1.2 (0.6 to 2.2)	0.8 (0.8 to 0.8)	14.7 (11.3 to 19.3)	0.7 (0.6 to 1.0)	10.9 (7.8 to 14.9)	1.9 (0.9 to 3.4)	1.2 (1.2 to 1.2)

Country	Year	2018 US Dollars per capita					2018 PPP per capita				
		Health spending	Government health spending	Out-of-pocket health spending	Prepaid private health spending	Development assistance for health	Health spending	Government health spending	Out-of-pocket health spending	Prepaid private health spending	Development assistance for health
Democratic Republic of the Congo	2002	9.5 (7.3 to 12.3)	0.5 (0.4 to 0.6)	7.1 (5.0 to 9.6)	1.1 (0.5 to 2.0)	0.8 (0.8 to 0.8)	14.8 (11.4 to 19.2)	0.8 (0.6 to 1.0)	11.1 (7.8 to 15.0)	1.7 (0.8 to 3.2)	1.3 (1.3 to 1.3)
Democratic Republic of the Congo	2003	11.5 (8.9 to 14.9)	0.6 (0.5 to 0.8)	8.4 (5.9 to 11.5)	1.2 (0.5 to 2.2)	1.4 (1.4 to 1.4)	18.0 (13.9 to 23.3)	1.0 (0.7 to 1.3)	13.1 (9.2 to 17.9)	1.8 (0.9 to 3.4)	2.1 (2.1 to 2.1)
Democratic Republic of the Congo	2004	13.1 (10.1 to 16.8)	0.7 (0.6 to 0.9)	9.6 (6.8 to 13.1)	1.2 (0.6 to 2.2)	1.6 (1.6 to 1.6)	20.4 (15.8 to 26.2)	1.1 (0.9 to 1.5)	14.9 (10.6 to 20.5)	1.9 (0.9 to 3.5)	2.4 (2.4 to 2.4)
Democratic Republic of the Congo	2005	15.1 (11.9 to 19.0)	0.8 (0.6 to 1.1)	10.2 (7.2 to 14.0)	1.2 (0.6 to 2.4)	2.8 (2.8 to 2.8)	23.5 (18.5 to 29.6)	1.3 (1.0 to 1.7)	15.9 (11.2 to 21.8)	1.9 (0.9 to 3.7)	4.4 (4.4 to 4.4)
Democratic Republic of the Congo	2006	14.7 (11.4 to 18.5)	0.9 (0.7 to 1.1)	10.1 (7.0 to 13.9)	1.2 (0.6 to 2.3)	2.6 (2.6 to 2.6)	22.9 (17.9 to 28.8)	1.3 (1.0 to 1.7)	15.7 (11.0 to 21.7)	1.9 (0.9 to 3.6)	4.0 (4.0 to 4.0)
Democratic Republic of the Congo	2007	14.6 (11.6 to 18.6)	1.0 (0.7 to 1.3)	9.8 (6.9 to 13.8)	1.2 (0.6 to 2.3)	2.6 (2.6 to 2.6)	22.8 (18.1 to 29.1)	1.5 (1.1 to 2.0)	15.3 (10.7 to 21.5)	1.9 (0.9 to 3.6)	4.1 (4.1 to 4.1)
Democratic Republic of the Congo	2008	17.9 (15.0 to 21.7)	1.1 (0.9 to 1.5)	9.2 (6.4 to 13.1)	1.3 (0.6 to 2.5)	6.3 (6.3 to 6.3)	27.9 (23.4 to 33.8)	1.8 (1.3 to 2.3)	14.4 (10.0 to 20.4)	2.0 (0.9 to 3.8)	9.8 (9.8 to 9.8)
Democratic Republic of the Congo	2009	16.7 (14.2 to 20.0)	1.3 (1.0 to 1.6)	7.9 (5.5 to 11.2)	1.2 (0.6 to 2.3)	6.4 (6.4 to 6.4)	26.1 (22.1 to 31.2)	2.0 (1.5 to 2.5)	12.3 (8.5 to 17.4)	1.9 (0.9 to 3.6)	9.9 (9.9 to 9.9)
Democratic Republic of the Congo	2010	16.1 (13.8 to 19.0)	1.6 (1.2 to 2.0)	7.0 (4.8 to 9.8)	1.2 (0.6 to 2.3)	6.2 (6.2 to 6.2)	25.1 (21.5 to 29.7)	2.5 (1.9 to 3.2)	11.0 (7.6 to 15.3)	1.9 (0.9 to 3.7)	9.7 (9.7 to 9.7)
Democratic Republic of the Congo	2011	16.0 (14.0 to 18.8)	1.8 (1.4 to 2.3)	6.5 (4.5 to 9.0)	1.1 (0.5 to 2.0)	6.7 (6.7 to 6.7)	25.0 (21.8 to 29.3)	2.8 (2.1 to 3.6)	10.1 (7.0 to 14.1)	1.7 (0.8 to 3.1)	10.5 (10.5 to 10.5)
Democratic Republic of the Congo	2012	17.0 (15.0 to 19.9)	2.1 (1.6 to 2.7)	6.6 (4.6 to 9.1)	1.1 (0.5 to 2.1)	7.2 (7.2 to 7.2)	26.6 (23.4 to 31.0)	3.3 (2.5 to 4.3)	10.3 (7.2 to 14.2)	1.7 (0.8 to 3.2)	11.3 (11.3 to 11.3)
Democratic Republic of the Congo	2013	18.3 (16.0 to 21.3)	2.6 (1.9 to 3.3)	7.2 (5.1 to 10.0)	1.3 (0.6 to 2.5)	7.2 (7.2 to 7.2)	28.5 (24.9 to 33.2)	4.0 (3.0 to 5.2)	11.3 (7.9 to 15.5)	2.0 (1.0 to 3.8)	11.2 (11.2 to 11.2)
Democratic Republic of the Congo	2014	18.9 (16.4 to 22.1)	2.8 (2.1 to 3.6)	7.7 (5.4 to 10.8)	1.5 (0.7 to 2.8)	6.9 (6.9 to 6.9)	29.5 (25.6 to 34.5)	4.4 (3.3 to 5.6)	12.0 (8.5 to 16.8)	2.3 (1.1 to 4.3)	10.8 (10.8 to 10.8)
Democratic Republic of the Congo	2015	19.7 (17.1 to 23.0)	3.1 (2.3 to 3.9)	8.1 (5.7 to 11.4)	1.6 (0.8 to 3.0)	7.0 (7.0 to 7.0)	30.7 (26.7 to 35.9)	4.8 (3.6 to 6.1)	12.6 (8.8 to 17.7)	2.5 (1.2 to 4.7)	10.9 (10.9 to 10.9)
Democratic Republic of the Congo	2016	19.4 (16.7 to 22.8)	2.8 (2.1 to 3.7)	8.0 (5.6 to 11.2)	1.6 (0.7 to 2.8)	6.9 (6.9 to 6.9)	30.2 (26.1 to 35.6)	4.4 (3.3 to 5.7)	12.5 (8.8 to 17.4)	2.4 (1.2 to 4.4)	10.8 (10.8 to 10.8)
Democratic Republic of the Congo	2017	20.2 (17.4 to 23.5)	2.8 (2.1 to 3.6)	8.0 (5.6 to 11.2)	1.6 (0.8 to 2.9)	7.7 (7.7 to 7.7)	31.5 (27.2 to 36.7)	4.4 (3.3 to 5.6)	12.6 (8.8 to 17.4)	2.5 (1.2 to 4.5)	12.0 (12.0 to 12.0)
Democratic Republic of the Congo	2018	19.5 (16.7 to 23.0)	2.9 (2.1 to 3.7)	8.1 (5.6 to 11.3)	1.6 (0.8 to 2.9)	6.9 (6.9 to 6.9)	30.3 (26.0 to 35.8)	4.5 (3.3 to 5.8)	12.6 (8.8 to 17.6)	2.5 (1.2 to 4.6)	10.8 (10.7 to 10.8)
Democratic Republic of the Congo	2019	19.6 (16.8 to 23.0)	2.9 (2.2 to 3.8)	8.1 (5.6 to 11.3)	1.6 (0.8 to 3.0)	6.9 (6.4 to 7.3)	30.5 (26.2 to 35.9)	4.6 (3.4 to 5.9)	12.6 (8.8 to 17.6)	2.6 (1.2 to 4.6)	10.8 (10.1 to 11.4)
Democratic Republic of the Congo	2020	19.8 (16.9 to 23.5)	3.0 (2.2 to 3.8)	8.1 (5.7 to 11.5)	1.7 (0.8 to 3.0)	7.0 (6.4 to 7.5)	30.8 (26.4 to 36.7)	4.6 (3.5 to 6.0)	12.7 (8.8 to 17.9)	2.6 (1.2 to 4.7)	10.9 (10.0 to 11.7)
Democratic Republic of the Congo	2021	20.0 (17.1 to 23.5)	3.0 (2.3 to 3.9)	8.2 (5.7 to 11.5)	1.7 (0.8 to 3.1)	7.1 (6.4 to 7.8)	31.2 (26.6 to 36.6)	4.7 (3.6 to 6.1)	12.7 (8.9 to 17.9)	2.6 (1.3 to 4.8)	11.1 (10.0 to 12.2)
Democratic Republic of the Congo	2022	20.3 (17.3 to 24.0)	3.1 (2.4 to 4.0)	8.2 (5.7 to 11.6)	1.7 (0.8 to 3.1)	7.3 (6.4 to 8.1)	31.6 (27.0 to 37.4)	4.8 (3.7 to 6.2)	12.8 (8.9 to 18.0)	2.7 (1.3 to 4.8)	11.3 (10.1 to 12.6)

Country	Year	2018 US Dollars per capita					2018 PPP per capita				
		Health spending	Government health spending	Out-of-pocket health spending	Prepaid private health spending	Development assistance for health	Health spending	Government health spending	Out-of-pocket health spending	Prepaid private health spending	Development assistance for health
Democratic Republic of the Congo	2023	20.5 (17.5 to 24.4)	3.2 (2.4 to 4.1)	8.2 (5.7 to 11.6)	1.7 (0.8 to 3.1)	7.4 (6.4 to 8.4)	32.0 (27.3 to 38.1)	4.9 (3.8 to 6.4)	12.8 (8.9 to 18.1)	2.7 (1.3 to 4.9)	11.6 (10.0 to 13.1)
Democratic Republic of the Congo	2024	20.8 (17.5 to 24.7)	3.2 (2.5 to 4.2)	8.3 (5.7 to 11.7)	1.8 (0.8 to 3.2)	7.6 (6.5 to 8.7)	32.5 (27.3 to 38.5)	5.1 (3.8 to 6.5)	12.9 (9.0 to 18.2)	2.7 (1.3 to 5.0)	11.8 (10.2 to 13.6)
Democratic Republic of the Congo	2025	21.1 (17.8 to 25.0)	3.3 (2.5 to 4.3)	8.3 (5.8 to 11.7)	1.8 (0.8 to 3.2)	7.7 (6.6 to 9.1)	33.0 (27.8 to 39.0)	5.2 (3.9 to 6.7)	12.9 (9.0 to 18.3)	2.8 (1.3 to 5.0)	12.1 (10.3 to 14.1)
Democratic Republic of the Congo	2026	21.4 (18.1 to 25.5)	3.4 (2.6 to 4.4)	8.3 (5.8 to 11.7)	1.8 (0.9 to 3.3)	7.9 (6.6 to 9.3)	33.4 (28.2 to 39.8)	5.3 (4.0 to 6.8)	13.0 (9.0 to 18.3)	2.8 (1.3 to 5.1)	12.3 (10.4 to 14.5)
Democratic Republic of the Congo	2027	21.7 (18.1 to 25.7)	3.4 (2.6 to 4.5)	8.4 (5.8 to 11.8)	1.8 (0.9 to 3.3)	8.0 (6.8 to 9.6)	33.8 (28.2 to 40.1)	5.4 (4.1 to 7.0)	13.1 (9.0 to 18.5)	2.9 (1.4 to 5.2)	12.5 (10.5 to 14.9)
Democratic Republic of the Congo	2028	22.0 (18.2 to 26.2)	3.5 (2.7 to 4.6)	8.4 (5.8 to 11.9)	1.9 (0.9 to 3.4)	8.2 (6.9 to 9.9)	34.3 (28.4 to 40.8)	5.5 (4.2 to 7.1)	13.1 (9.1 to 18.5)	2.9 (1.4 to 5.3)	12.8 (10.7 to 15.5)
Democratic Republic of the Congo	2029	22.3 (18.9 to 26.6)	3.6 (2.7 to 4.7)	8.5 (5.8 to 12.0)	1.9 (0.9 to 3.5)	8.4 (6.9 to 10.3)	34.8 (29.4 to 41.4)	5.6 (4.2 to 7.3)	13.2 (9.1 to 18.7)	3.0 (1.4 to 5.4)	13.1 (10.8 to 16.0)
Democratic Republic of the Congo	2030	22.7 (18.9 to 27.0)	3.7 (2.8 to 4.8)	8.5 (5.8 to 12.1)	1.9 (0.9 to 3.5)	8.5 (7.0 to 10.5)	35.3 (29.6 to 42.1)	5.7 (4.3 to 7.5)	13.3 (9.1 to 18.8)	3.0 (1.4 to 5.5)	13.3 (10.9 to 16.3)
Democratic Republic of the Congo	2031	23.0 (19.2 to 27.8)	3.7 (2.8 to 4.9)	8.6 (5.8 to 12.3)	2.0 (0.9 to 3.6)	8.7 (6.9 to 11.0)	35.9 (29.9 to 43.4)	5.8 (4.4 to 7.6)	13.4 (9.0 to 19.1)	3.1 (1.5 to 5.6)	13.5 (10.8 to 17.1)
Democratic Republic of the Congo	2032	23.3 (19.2 to 28.2)	3.8 (2.9 to 5.0)	8.7 (5.8 to 12.4)	2.0 (0.9 to 3.7)	8.8 (7.0 to 11.2)	36.4 (30.0 to 44.0)	6.0 (4.5 to 7.8)	13.5 (9.1 to 19.3)	3.1 (1.5 to 5.7)	13.8 (11.0 to 17.5)
Democratic Republic of the Congo	2033	23.7 (19.8 to 28.7)	3.9 (3.0 to 5.1)	8.8 (5.9 to 12.6)	2.0 (1.0 to 3.8)	9.0 (7.0 to 11.7)	37.0 (30.8 to 44.8)	6.1 (4.6 to 7.9)	13.7 (9.2 to 19.6)	3.2 (1.5 to 5.9)	14.0 (11.0 to 18.2)
Democratic Republic of the Congo	2034	24.1 (19.8 to 29.2)	4.0 (3.1 to 5.2)	8.8 (5.9 to 12.8)	2.1 (1.0 to 3.8)	9.2 (7.1 to 12.0)	37.6 (30.8 to 45.5)	6.2 (4.8 to 8.1)	13.8 (9.2 to 19.9)	3.3 (1.5 to 6.0)	14.3 (11.1 to 18.7)
Democratic Republic of the Congo	2035	24.5 (20.3 to 29.7)	4.1 (3.1 to 5.3)	8.9 (5.9 to 12.9)	2.1 (1.0 to 3.9)	9.4 (7.2 to 12.4)	38.2 (31.6 to 46.3)	6.4 (4.9 to 8.3)	13.9 (9.3 to 20.2)	3.3 (1.6 to 6.1)	14.6 (11.2 to 19.3)
Democratic Republic of the Congo	2036	24.9 (20.6 to 30.3)	4.2 (3.2 to 5.4)	9.0 (6.0 to 13.1)	2.2 (1.0 to 4.0)	9.6 (7.3 to 13.1)	38.9 (32.1 to 47.2)	6.5 (5.0 to 8.5)	14.1 (9.3 to 20.4)	3.4 (1.6 to 6.2)	14.9 (11.4 to 20.5)
Democratic Republic of the Congo	2037	25.3 (20.6 to 30.7)	4.3 (3.2 to 5.6)	9.1 (6.0 to 13.3)	2.2 (1.0 to 4.1)	9.8 (7.3 to 13.4)	39.5 (32.2 to 47.9)	6.6 (5.1 to 8.7)	14.2 (9.4 to 20.7)	3.4 (1.6 to 6.4)	15.2 (11.4 to 20.9)
Democratic Republic of the Congo	2038	25.8 (21.3 to 31.5)	4.3 (3.3 to 5.7)	9.2 (6.1 to 13.5)	2.2 (1.1 to 4.2)	10.0 (7.5 to 14.1)	40.2 (33.2 to 49.2)	6.8 (5.2 to 8.9)	14.4 (9.5 to 21.0)	3.5 (1.7 to 6.5)	15.5 (11.6 to 22.0)
Democratic Republic of the Congo	2039	26.2 (21.5 to 32.1)	4.4 (3.4 to 5.8)	9.3 (6.1 to 13.7)	2.3 (1.1 to 4.2)	10.2 (7.5 to 14.5)	40.9 (33.5 to 50.1)	6.9 (5.3 to 9.1)	14.6 (9.6 to 21.3)	3.6 (1.7 to 6.6)	15.8 (11.7 to 22.5)
Democratic Republic of the Congo	2040	26.7 (22.0 to 32.9)	4.5 (3.4 to 5.9)	9.4 (6.2 to 13.9)	2.3 (1.1 to 4.3)	10.4 (7.7 to 14.9)	41.6 (34.3 to 51.3)	7.0 (5.3 to 9.3)	14.7 (9.6 to 21.6)	3.6 (1.7 to 6.7)	16.2 (12.0 to 23.3)
Democratic Republic of the Congo	2041	27.2 (21.9 to 33.5)	4.6 (3.5 to 6.1)	9.6 (6.2 to 14.0)	2.4 (1.1 to 4.4)	10.6 (7.7 to 15.1)	42.4 (34.2 to 52.3)	7.2 (5.4 to 9.5)	14.9 (9.7 to 21.9)	3.7 (1.8 to 6.9)	16.6 (12.0 to 23.6)
Democratic Republic of the Congo	2042	27.7 (22.0 to 34.3)	4.7 (3.6 to 6.2)	9.7 (6.3 to 14.2)	2.4 (1.1 to 4.5)	10.9 (7.8 to 15.8)	43.2 (34.4 to 53.4)	7.3 (5.6 to 9.7)	15.1 (9.8 to 22.2)	3.8 (1.8 to 7.0)	17.0 (12.2 to 24.7)
Democratic Republic of the Congo	2043	28.2 (22.5 to 35.6)	4.8 (3.6 to 6.3)	9.8 (6.4 to 14.5)	2.4 (1.2 to 4.6)	11.1 (7.9 to 16.3)	44.0 (35.1 to 55.6)	7.5 (5.7 to 9.8)	15.3 (9.9 to 22.6)	3.8 (1.8 to 7.2)	17.4 (12.3 to 25.5)

		2018 US Dollars per capita					2018 PPP per capita				
Country	Year	Health spending	Government health spending	Out-of-pocket health spending	Prepaid private health spending	Development assistance for health	Health spending	Government health spending	Out-of-pocket health spending	Prepaid private health spending	Development assistance for health
Democratic Republic of the Congo	2044	28.8 (23.2 to 36.1)	4.9 (3.7 to 6.5)	10.0 (6.5 to 14.7)	2.5 (1.2 to 4.7)	11.4 (8.0 to 17.1)	44.9 (36.2 to 56.3)	7.6 (5.8 to 10.1)	15.5 (10.1 to 23.0)	3.9 (1.8 to 7.3)	17.8 (12.5 to 26.7)
Democratic Republic of the Congo	2045	29.4 (23.4 to 36.9)	5.0 (3.8 to 6.6)	10.1 (6.5 to 15.0)	2.5 (1.2 to 4.8)	11.7 (8.1 to 18.1)	45.8 (36.5 to 57.5)	7.8 (5.9 to 10.3)	15.8 (10.2 to 23.4)	4.0 (1.9 to 7.5)	18.3 (12.6 to 28.2)
Democratic Republic of the Congo	2046	30.0 (23.9 to 38.2)	5.1 (3.9 to 6.8)	10.3 (6.6 to 15.2)	2.6 (1.2 to 4.9)	12.1 (8.2 to 19.1)	46.8 (37.3 to 59.6)	7.9 (6.0 to 10.5)	16.0 (10.3 to 23.7)	4.0 (1.9 to 7.6)	18.8 (12.7 to 29.8)
Democratic Republic of the Congo	2047	30.7 (24.2 to 39.8)	5.2 (3.9 to 6.9)	10.4 (6.7 to 15.5)	2.6 (1.2 to 5.0)	12.4 (8.4 to 19.5)	47.9 (37.7 to 62.0)	8.2 (6.2 to 10.8)	16.2 (10.5 to 24.1)	4.1 (1.9 to 7.8)	19.4 (13.1 to 30.5)
Democratic Republic of the Congo	2048	31.4 (24.8 to 40.9)	5.4 (4.0 to 7.1)	10.6 (6.8 to 15.7)	2.7 (1.3 to 5.1)	12.8 (8.6 to 20.3)	49.0 (38.7 to 63.8)	8.4 (6.3 to 11.1)	16.5 (10.7 to 24.5)	4.2 (2.0 to 7.9)	20.0 (13.4 to 31.7)
Democratic Republic of the Congo	2049	32.2 (25.3 to 41.8)	5.5 (4.1 to 7.4)	10.7 (7.0 to 16.0)	2.7 (1.3 to 5.2)	13.2 (8.8 to 21.2)	50.2 (39.5 to 65.3)	8.6 (6.5 to 11.5)	16.8 (10.8 to 25.0)	4.3 (2.0 to 8.0)	20.5 (13.7 to 33.1)
Democratic Republic of the Congo	2050	33.0 (25.7 to 43.7)	5.7 (4.3 to 7.6)	10.9 (7.1 to 16.3)	2.8 (1.3 to 5.3)	13.6 (9.0 to 22.9)	51.5 (40.1 to 68.1)	8.9 (6.7 to 11.9)	17.0 (11.0 to 25.4)	4.3 (2.1 to 8.2)	21.2 (14.0 to 35.7)
Denmark	1995	3730.8 (3588.3 to 3884.3)	3044.1 (2904.4 to 3189.5)	636.6 (596.0 to 679.4)	50.1 (34.6 to 69.7)	0.0 (0.0 to 0.0)	3155.7 (3035.1 to 3285.5)	2574.8 (2456.6 to 2697.8)	538.5 (504.1 to 574.7)	42.4 (29.2 to 59.0)	0.0 (0.0 to 0.0)
Denmark	1996	3807.7 (3700.5 to 3925.2)	3103.3 (3003.5 to 3212.7)	650.8 (619.7 to 684.1)	53.6 (39.5 to 71.7)	0.0 (0.0 to 0.0)	3220.8 (3130.0 to 3320.1)	2624.9 (2540.5 to 2717.5)	550.5 (524.1 to 578.7)	45.4 (33.4 to 60.6)	0.0 (0.0 to 0.0)
Denmark	1997	3937.4 (3832.9 to 4047.1)	3213.7 (3109.9 to 3317.5)	666.3 (636.1 to 697.2)	57.4 (44.0 to 73.6)	0.0 (0.0 to 0.0)	3330.4 (3242.0 to 3423.3)	2718.3 (2630.5 to 2806.1)	563.6 (538.1 to 589.7)	48.5 (37.2 to 62.3)	0.0 (0.0 to 0.0)
Denmark	1998	4089.4 (3989.0 to 4191.8)	3351.5 (3257.5 to 3453.0)	676.3 (646.3 to 705.4)	61.7 (48.5 to 77.3)	0.0 (0.0 to 0.0)	3459.0 (3374.1 to 3545.6)	2834.8 (2755.4 to 2920.7)	572.1 (546.7 to 596.6)	52.2 (41.0 to 65.4)	0.0 (0.0 to 0.0)
Denmark	1999	4328.5 (4229.0 to 4431.7)	3578.0 (3482.3 to 3680.3)	684.8 (657.6 to 712.7)	65.7 (52.8 to 80.8)	0.0 (0.0 to 0.0)	3661.3 (3577.1 to 3748.6)	3026.5 (2945.5 to 3113.0)	579.2 (556.3 to 602.8)	55.5 (44.7 to 68.3)	0.0 (0.0 to 0.0)
Denmark	2000	4543.9 (4443.3 to 4647.9)	3780.8 (3682.7 to 3878.1)	694.6 (670.7 to 720.3)	68.5 (55.8 to 82.7)	0.0 (0.0 to 0.0)	3843.5 (3758.3 to 3931.4)	3197.9 (3115.0 to 3280.3)	587.6 (567.3 to 609.2)	58.0 (47.2 to 69.9)	0.0 (0.0 to 0.0)
Denmark	2001	4687.6 (4583.1 to 4788.9)	3914.1 (3810.5 to 4012.9)	703.3 (679.3 to 728.3)	70.2 (57.9 to 84.7)	0.0 (0.0 to 0.0)	3965.0 (3876.6 to 4050.6)	3310.7 (3223.1 to 3394.3)	594.9 (574.6 to 616.0)	59.4 (49.0 to 71.7)	0.0 (0.0 to 0.0)
Denmark	2002	4847.2 (4742.6 to 4961.0)	4059.5 (3957.3 to 4166.0)	715.0 (691.2 to 739.0)	72.8 (61.0 to 87.0)	0.0 (0.0 to 0.0)	4100.0 (4011.5 to 4196.2)	3433.7 (3347.3 to 3523.8)	604.8 (584.6 to 625.1)	61.5 (51.6 to 73.6)	0.0 (0.0 to 0.0)
Denmark	2003	4933.0 (4826.2 to 5037.6)	4130.4 (4030.8 to 4233.3)	726.9 (704.7 to 750.0)	75.7 (63.8 to 89.6)	0.0 (0.0 to 0.0)	4172.5 (4082.2 to 4261.0)	3493.7 (3409.5 to 3580.8)	614.8 (596.0 to 634.4)	64.0 (54.0 to 75.8)	0.0 (0.0 to 0.0)
Denmark	2004	5135.7 (5038.2 to 5238.9)	4299.1 (4201.7 to 4398.6)	756.3 (731.6 to 781.0)	80.3 (67.6 to 94.0)	0.0 (0.0 to 0.0)	4344.0 (4261.5 to 4431.3)	3636.4 (3553.9 to 3720.5)	639.7 (618.8 to 660.6)	68.0 (57.1 to 79.5)	0.0 (0.0 to 0.0)
Denmark	2005	5261.1 (5152.2 to 5373.3)	4402.5 (4300.5 to 4501.9)	774.8 (750.4 to 799.6)	83.9 (71.0 to 97.8)	0.0 (0.0 to 0.0)	4450.1 (4357.9 to 4545.0)	3723.8 (3637.6 to 3807.9)	655.3 (634.7 to 676.4)	71.0 (60.1 to 82.7)	0.0 (0.0 to 0.0)
Denmark	2006	5500.5 (5388.8 to 5607.2)	4610.2 (4499.7 to 4713.2)	799.7 (776.1 to 825.1)	90.6 (76.9 to 105.4)	0.0 (0.0 to 0.0)	4652.6 (4558.1 to 4742.8)	3899.5 (3806.1 to 3986.6)	676.4 (656.4 to 697.9)	76.6 (65.0 to 89.2)	0.0 (0.0 to 0.0)
Denmark	2007	5659.3 (5556.3 to 5765.1)	4749.3 (4648.0 to 4855.5)	812.5 (787.8 to 838.4)	97.4 (83.2 to 112.6)	0.0 (0.0 to 0.0)	4786.9 (4699.8 to 4876.4)	4017.2 (3931.5 to 4107.0)	687.3 (666.3 to 709.2)	82.4 (70.4 to 95.2)	0.0 (0.0 to 0.0)
Denmark	2008	5837.8 (5722.3 to 5955.1)	4911.4 (4803.1 to 5022.9)	821.9 (796.4 to 847.9)	104.4 (89.9 to 120.0)	0.0 (0.0 to 0.0)	4937.8 (4840.1 to 5037.1)	4154.3 (4062.7 to 4248.6)	695.2 (673.6 to 717.2)	88.3 (76.0 to 101.5)	0.0 (0.0 to 0.0)

		2018 US Dollars per capita					2018 PPP per capita				
Country	Year	Health spending	Government health spending	Out-of-pocket health spending	Prepaid private health spending	Development assistance for health	Health spending	Government health spending	Out-of-pocket health spending	Prepaid private health spending	Development assistance for health
Denmark	2009	5854.3 (5748.4 to 5971.2)	4920.2 (4814.8 to 5027.6)	827.3 (802.0 to 853.7)	106.8 (93.2 to 123.9)	0.0 (0.0 to 0.0)	4951.8 (4862.3 to 5050.7)	4161.7 (4072.5 to 4252.6)	699.8 (678.4 to 722.1)	90.3 (78.8 to 104.8)	0.0 (0.0 to 0.0)
Denmark	2010	5960.6 (5848.1 to 6070.6)	5005.0 (4904.5 to 5111.5)	847.7 (821.8 to 873.9)	107.9 (93.8 to 125.5)	0.0 (0.0 to 0.0)	5041.7 (4946.6 to 5134.8)	4233.4 (4148.5 to 4323.6)	717.0 (695.1 to 739.2)	91.3 (79.3 to 106.2)	0.0 (0.0 to 0.0)
Denmark	2011	5948.7 (5839.7 to 6058.6)	4988.8 (4883.5 to 5098.3)	850.4 (824.3 to 876.5)	109.6 (94.0 to 126.4)	0.0 (0.0 to 0.0)	5031.7 (4939.5 to 5124.7)	4219.7 (4130.7 to 4312.4)	719.3 (697.3 to 741.4)	92.7 (79.5 to 106.9)	0.0 (0.0 to 0.0)
Denmark	2012	5914.7 (5801.3 to 6028.1)	4965.4 (4854.6 to 5073.3)	837.8 (812.3 to 863.6)	111.4 (95.7 to 128.8)	0.0 (0.0 to 0.0)	5002.9 (4907.0 to 5098.8)	4200.0 (4106.2 to 4291.2)	708.7 (687.1 to 730.4)	94.3 (81.0 to 109.0)	0.0 (0.0 to 0.0)
Denmark	2013	5918.5 (5808.8 to 6024.7)	4977.9 (4873.7 to 5078.9)	825.2 (799.6 to 850.4)	115.5 (100.1 to 133.0)	0.0 (0.0 to 0.0)	5006.2 (4913.4 to 5096.0)	4210.5 (4122.4 to 4296.0)	698.0 (676.3 to 719.3)	97.7 (84.7 to 112.5)	0.0 (0.0 to 0.0)
Denmark	2014	5981.3 (5874.4 to 6094.9)	5034.3 (4923.6 to 5146.3)	825.3 (799.7 to 850.1)	121.7 (104.0 to 141.0)	0.0 (0.0 to 0.0)	5059.3 (4968.8 to 5155.4)	4258.2 (4164.6 to 4352.9)	698.1 (676.4 to 719.0)	103.0 (87.9 to 119.2)	0.0 (0.0 to 0.0)
Denmark	2015	6090.7 (5979.4 to 6211.8)	5126.9 (5016.4 to 5244.2)	835.0 (808.1 to 861.8)	128.9 (109.5 to 153.5)	0.0 (0.0 to 0.0)	5151.8 (5057.7 to 5254.3)	4336.5 (4243.1 to 4435.8)	706.2 (683.5 to 729.0)	109.0 (92.6 to 129.8)	0.0 (0.0 to 0.0)
Denmark	2016	6195.3 (6033.2 to 6363.2)	5212.6 (5063.0 to 5375.9)	848.4 (813.9 to 884.6)	134.3 (109.7 to 168.3)	0.0 (0.0 to 0.0)	5240.3 (5103.2 to 5382.3)	4409.1 (4282.5 to 4547.2)	717.6 (688.4 to 748.2)	113.6 (92.8 to 142.4)	0.0 (0.0 to 0.0)
Denmark	2017	6207.7 (6034.9 to 6385.9)	5214.5 (5047.9 to 5388.8)	857.5 (820.3 to 896.7)	135.7 (110.7 to 170.3)	0.0 (0.0 to 0.0)	5250.8 (5104.6 to 5401.5)	4410.7 (4269.7 to 4558.1)	725.3 (693.8 to 758.4)	114.8 (93.7 to 144.0)	0.0 (0.0 to 0.0)
Denmark	2018	6274.2 (6094.4 to 6470.7)	5271.8 (5088.5 to 5458.0)	864.7 (824.3 to 908.3)	137.8 (112.4 to 172.8)	0.0 (0.0 to 0.0)	5307.0 (5154.9 to 5473.2)	4459.1 (4304.0 to 4616.6)	731.4 (697.3 to 768.3)	116.5 (95.0 to 146.2)	0.0 (0.0 to 0.0)
Denmark	2019	6323.6 (6122.0 to 6532.3)	5312.7 (5122.8 to 5518.3)	871.5 (829.3 to 919.4)	139.4 (113.6 to 174.4)	0.0 (0.0 to 0.0)	5348.8 (5178.2 to 5525.3)	4493.7 (4333.1 to 4667.6)	737.2 (701.4 to 777.7)	117.9 (96.1 to 147.5)	0.0 (0.0 to 0.0)
Denmark	2020	6381.1 (6175.3 to 6587.3)	5360.7 (5169.6 to 5568.1)	879.3 (834.7 to 928.2)	141.1 (114.8 to 176.5)	0.0 (0.0 to 0.0)	5397.4 (5223.4 to 5571.9)	4534.3 (4372.7 to 4709.7)	743.7 (706.0 to 785.1)	119.4 (97.1 to 149.3)	0.0 (0.0 to 0.0)
Denmark	2021	6433.5 (6222.6 to 6646.0)	5404.4 (5210.1 to 5613.0)	886.3 (837.2 to 940.6)	142.8 (116.0 to 178.6)	0.0 (0.0 to 0.0)	5441.7 (5263.3 to 5621.5)	4571.3 (4406.9 to 4747.8)	749.6 (708.1 to 795.6)	120.8 (98.2 to 151.1)	0.0 (0.0 to 0.0)
Denmark	2022	6493.9 (6291.8 to 6713.2)	5455.3 (5256.1 to 5663.6)	893.9 (842.7 to 948.6)	144.6 (117.4 to 180.6)	0.0 (0.0 to 0.0)	5492.8 (5321.9 to 5678.4)	4614.4 (4445.9 to 4790.5)	756.1 (712.8 to 802.4)	122.3 (99.3 to 152.7)	0.0 (0.0 to 0.0)
Denmark	2023	6566.9 (6347.4 to 6788.6)	5517.7 (5305.2 to 5732.5)	902.5 (849.3 to 959.5)	146.7 (119.1 to 182.8)	0.0 (0.0 to 0.0)	5554.6 (5368.9 to 5742.1)	4667.1 (4487.4 to 4848.8)	763.4 (718.3 to 811.6)	124.1 (100.7 to 154.6)	0.0 (0.0 to 0.0)
Denmark	2024	6648.6 (6415.3 to 6875.2)	5588.1 (5368.3 to 5811.5)	911.5 (856.1 to 969.8)	148.9 (120.8 to 185.8)	0.0 (0.0 to 0.0)	5623.7 (5426.4 to 5815.4)	4726.7 (4540.7 to 4915.6)	771.0 (724.1 to 820.3)	126.0 (102.2 to 157.2)	0.0 (0.0 to 0.0)
Denmark	2025	6729.7 (6473.1 to 6967.8)	5658.8 (5426.0 to 5904.5)	919.8 (861.6 to 980.8)	151.1 (122.5 to 188.9)	0.0 (0.0 to 0.0)	5692.3 (5475.3 to 5893.7)	4786.5 (4589.5 to 4994.3)	778.0 (728.8 to 829.6)	127.8 (103.7 to 159.8)	0.0 (0.0 to 0.0)
Denmark	2026	6805.7 (6538.6 to 7079.9)	5724.7 (5478.9 to 5986.4)	927.8 (865.5 to 994.7)	153.3 (124.5 to 191.8)	0.0 (0.0 to 0.0)	5756.6 (5530.7 to 5988.5)	4842.2 (4634.3 to 5063.6)	784.8 (732.0 to 841.3)	129.6 (105.3 to 162.3)	0.0 (0.0 to 0.0)
Denmark	2027	6887.2 (6590.9 to 7170.2)	5795.6 (5527.4 to 6074.7)	936.1 (867.1 to 1011.4)	155.5 (126.3 to 194.9)	0.0 (0.0 to 0.0)	5825.5 (5574.9 to 6064.9)	4902.2 (4675.3 to 5138.3)	791.8 (733.5 to 855.5)	131.5 (106.8 to 164.9)	0.0 (0.0 to 0.0)
Denmark	2028	6965.5 (6677.6 to 7257.9)	5863.5 (5578.6 to 6159.0)	944.4 (865.6 to 1026.7)	157.7 (128.1 to 197.9)	0.0 (0.0 to 0.0)	5891.8 (5648.3 to 6139.0)	4959.6 (4718.6 to 5209.6)	798.8 (732.2 to 868.5)	133.3 (108.3 to 167.4)	0.0 (0.0 to 0.0)
Denmark	2029	7041.9 (6716.9 to 7379.4)	5929.5 (5612.9 to 6255.8)	952.6 (864.6 to 1048.2)	159.8 (129.8 to 200.9)	0.0 (0.0 to 0.0)	5956.3 (5681.4 to 6241.8)	5015.5 (4747.7 to 5291.4)	805.8 (731.3 to 886.6)	135.1 (109.8 to 169.9)	0.0 (0.0 to 0.0)

Country	Year	2018 US Dollars per capita					2018 PPP per capita				
		Health spending	Government health spending	Out-of-pocket health spending	Prepaid private health spending	Development assistance for health	Health spending	Government health spending	Out-of-pocket health spending	Prepaid private health spending	Development assistance for health
Denmark	2030	7114.2 (6766.1 to 7483.5)	5992.0 (5657.3 to 6340.5)	960.5 (861.9 to 1068.1)	161.8 (131.4 to 203.3)	0.0 (0.0 to 0.0)	6017.5 (5723.1 to 6329.9)	5068.3 (4785.2 to 5363.0)	812.4 (729.0 to 903.5)	136.8 (111.2 to 172.0)	0.0 (0.0 to 0.0)
Denmark	2031	7180.5 (6818.0 to 7555.7)	6048.9 (5692.5 to 6420.6)	968.0 (859.5 to 1086.2)	163.6 (132.8 to 205.5)	0.0 (0.0 to 0.0)	6073.6 (5766.9 to 6390.9)	5116.4 (4815.0 to 5430.8)	818.8 (727.0 to 918.8)	138.4 (112.3 to 173.8)	0.0 (0.0 to 0.0)
Denmark	2032	7241.9 (6858.8 to 7643.3)	6101.3 (5741.9 to 6479.0)	975.2 (858.4 to 1104.3)	165.4 (134.4 to 207.6)	0.0 (0.0 to 0.0)	6125.5 (5801.5 to 6465.1)	5160.8 (4856.8 to 5480.2)	824.9 (726.0 to 934.1)	139.9 (113.7 to 175.6)	0.0 (0.0 to 0.0)
Denmark	2033	7300.6 (6885.6 to 7708.5)	6151.1 (5768.0 to 6555.3)	982.5 (856.9 to 1122.8)	167.1 (135.7 to 209.6)	0.0 (0.0 to 0.0)	6175.2 (5824.2 to 6520.2)	5202.8 (4878.9 to 5544.8)	831.0 (724.8 to 949.7)	141.4 (114.7 to 177.3)	0.0 (0.0 to 0.0)
Denmark	2034	7360.4 (6924.3 to 7791.6)	6201.7 (5789.6 to 6614.4)	989.9 (857.2 to 1141.4)	168.9 (136.8 to 211.7)	0.0 (0.0 to 0.0)	6225.8 (5856.9 to 6590.5)	5245.7 (4897.1 to 5594.8)	837.3 (725.1 to 965.5)	142.8 (115.7 to 179.0)	0.0 (0.0 to 0.0)
Denmark	2035	7424.7 (6997.6 to 7875.7)	6255.9 (5829.7 to 6681.4)	998.1 (858.9 to 1160.7)	170.8 (138.1 to 213.9)	0.0 (0.0 to 0.0)	6280.1 (5918.9 to 6661.6)	5291.5 (4931.0 to 5651.5)	844.2 (726.5 to 981.8)	144.5 (116.8 to 180.9)	0.0 (0.0 to 0.0)
Denmark	2036	7490.4 (7009.9 to 7972.3)	6310.7 (5865.9 to 6759.4)	1006.8 (862.7 to 1180.5)	172.8 (139.4 to 216.2)	0.0 (0.0 to 0.0)	6335.7 (5929.3 to 6743.3)	5337.9 (4961.6 to 5717.4)	851.6 (729.7 to 998.5)	146.2 (117.9 to 182.9)	0.0 (0.0 to 0.0)
Denmark	2037	7561.5 (7047.7 to 8068.2)	6369.9 (5884.5 to 6823.7)	1016.5 (864.3 to 1203.7)	175.1 (140.9 to 218.9)	0.0 (0.0 to 0.0)	6395.9 (5961.2 to 6824.5)	5387.9 (4977.3 to 5771.8)	859.8 (731.1 to 1018.1)	148.1 (119.1 to 185.2)	0.0 (0.0 to 0.0)
Denmark	2038	7635.7 (7113.9 to 8154.5)	6431.3 (5895.3 to 6909.6)	1026.9 (868.3 to 1223.4)	177.5 (142.5 to 221.9)	0.0 (0.0 to 0.0)	6458.6 (6017.3 to 6897.5)	5439.8 (4986.5 to 5844.5)	868.6 (734.4 to 1034.8)	150.2 (120.6 to 187.7)	0.0 (0.0 to 0.0)
Denmark	2039	7718.4 (7146.0 to 8256.5)	6500.3 (5944.3 to 7009.7)	1038.0 (873.9 to 1242.1)	180.1 (144.2 to 224.8)	0.0 (0.0 to 0.0)	6528.6 (6044.4 to 6983.7)	5498.3 (5027.9 to 5929.1)	878.0 (739.2 to 1050.6)	152.4 (122.0 to 190.2)	0.0 (0.0 to 0.0)
Denmark	2040	7804.1 (7189.6 to 8407.9)	6571.8 (5982.7 to 7116.4)	1049.4 (879.1 to 1265.6)	182.8 (146.1 to 228.0)	0.0 (0.0 to 0.0)	6601.1 (6081.3 to 7111.8)	5558.8 (5060.4 to 6019.4)	887.7 (743.6 to 1070.5)	154.6 (123.5 to 192.8)	0.0 (0.0 to 0.0)
Denmark	2041	7889.1 (7261.7 to 8536.5)	6642.4 (5998.9 to 7230.6)	1061.2 (887.0 to 1285.3)	185.5 (148.1 to 231.2)	0.0 (0.0 to 0.0)	6672.9 (6142.3 to 7220.6)	5618.4 (5074.2 to 6115.9)	897.6 (750.3 to 1087.2)	156.9 (125.3 to 195.5)	0.0 (0.0 to 0.0)
Denmark	2042	7980.8 (7270.7 to 8604.7)	6718.9 (6036.6 to 7337.8)	1073.5 (896.0 to 1304.8)	188.4 (149.9 to 234.8)	0.0 (0.0 to 0.0)	6750.5 (6149.9 to 7278.3)	5683.1 (5106.0 to 6206.7)	908.0 (757.9 to 1103.7)	159.4 (126.8 to 198.6)	0.0 (0.0 to 0.0)
Denmark	2043	8071.3 (7339.2 to 8762.1)	6793.9 (6099.2 to 7458.0)	1086.1 (904.4 to 1323.7)	191.3 (151.9 to 238.6)	0.0 (0.0 to 0.0)	6827.1 (6207.9 to 7411.4)	5746.6 (5159.0 to 6308.3)	918.7 (765.0 to 1119.7)	161.8 (128.5 to 201.8)	0.0 (0.0 to 0.0)
Denmark	2044	8170.3 (7417.6 to 8874.1)	6876.1 (6125.7 to 7594.3)	1099.7 (914.1 to 1342.8)	194.5 (153.9 to 242.4)	0.0 (0.0 to 0.0)	6910.9 (6274.1 to 7506.1)	5816.1 (5181.4 to 6423.6)	930.2 (773.2 to 1135.8)	164.5 (130.2 to 205.0)	0.0 (0.0 to 0.0)
Denmark	2045	8277.5 (7461.0 to 9078.5)	6965.3 (6148.9 to 7725.2)	1114.2 (922.8 to 1361.8)	197.9 (156.2 to 246.4)	0.0 (0.0 to 0.0)	7001.5 (6310.9 to 7679.0)	5891.6 (5201.1 to 6534.3)	942.5 (780.5 to 1151.9)	167.4 (132.1 to 208.4)	0.0 (0.0 to 0.0)
Denmark	2046	8383.1 (7532.6 to 9258.1)	7052.4 (6212.2 to 7874.6)	1129.3 (935.6 to 1380.3)	201.4 (158.7 to 251.4)	0.0 (0.0 to 0.0)	7090.8 (6371.4 to 7831.0)	5965.3 (5254.5 to 6660.7)	955.2 (791.3 to 1167.6)	170.4 (134.3 to 212.6)	0.0 (0.0 to 0.0)
Denmark	2047	8496.6 (7580.0 to 9433.0)	7146.1 (6229.1 to 8019.7)	1145.3 (947.6 to 1398.9)	205.2 (161.6 to 257.0)	0.0 (0.0 to 0.0)	7186.8 (6411.5 to 7978.9)	6044.5 (5268.8 to 6783.4)	968.7 (801.5 to 1183.3)	173.6 (136.7 to 217.4)	0.0 (0.0 to 0.0)
Denmark	2048	8612.2 (7576.6 to 9578.7)	7241.5 (6261.9 to 8182.3)	1161.7 (960.4 to 1418.6)	209.1 (164.4 to 262.6)	0.0 (0.0 to 0.0)	7284.6 (6408.6 to 8102.1)	6125.2 (5296.6 to 6920.9)	982.6 (812.3 to 1199.9)	176.8 (139.1 to 222.1)	0.0 (0.0 to 0.0)
Denmark	2049	8727.4 (7672.4 to 9736.7)	7336.4 (6297.8 to 8344.3)	1178.0 (974.4 to 1437.7)	213.0 (167.3 to 267.8)	0.0 (0.0 to 0.0)	7382.0 (6489.6 to 8235.8)	6205.4 (5326.9 to 7058.0)	996.4 (824.2 to 1216.1)	180.1 (141.5 to 226.6)	0.0 (0.0 to 0.0)
Denmark	2050	8845.5 (7697.2 to 9960.1)	7434.4 (6337.1 to 8500.8)	1194.3 (987.2 to 1459.9)	216.8 (170.1 to 272.5)	0.0 (0.0 to 0.0)	7482.0 (6510.6 to 8424.7)	6288.4 (5360.2 to 7190.4)	1010.2 (835.0 to 1234.9)	183.4 (143.9 to 230.5)	0.0 (0.0 to 0.0)

		2018 US Dollars per capita					2018 PPP per capita				
Country	Year	Health spending	Government health spending	Out-of-pocket health spending	Prepaid private health spending	Development assistance for health	Health spending	Government health spending	Out-of-pocket health spending	Prepaid private health spending	Development assistance for health
Djibouti	1995	69.0 (57.6 to 82.5)	25.8 (19.7 to 32.2)	31.6 (22.7 to 43.5)	0.4 (0.2 to 0.7)	11.1 (11.1 to 11.1)	129.1 (107.9 to 154.4)	48.3 (36.9 to 60.2)	59.2 (42.5 to 81.4)	0.7 (0.3 to 1.3)	20.8 (20.8 to 20.8)
Djibouti	1996	58.8 (47.7 to 71.2)	24.6 (19.0 to 30.6)	30.5 (22.1 to 41.6)	0.3 (0.2 to 0.6)	3.3 (3.3 to 3.3)	110.1 (89.3 to 133.2)	46.1 (35.6 to 57.3)	57.2 (41.5 to 77.8)	0.6 (0.3 to 1.2)	6.2 (6.2 to 6.2)
Djibouti	1997	75.6 (64.9 to 88.5)	26.1 (20.2 to 32.6)	30.5 (22.1 to 41.0)	0.4 (0.2 to 0.6)	18.7 (18.7 to 18.7)	141.6 (121.5 to 165.6)	48.9 (37.8 to 61.0)	57.0 (41.3 to 76.7)	0.7 (0.3 to 1.2)	34.9 (34.9 to 34.9)
Djibouti	1998	78.6 (67.8 to 91.7)	27.3 (21.0 to 34.1)	30.2 (22.0 to 40.9)	0.4 (0.2 to 0.7)	20.7 (20.7 to 20.7)	147.1 (126.9 to 171.6)	51.2 (39.4 to 63.9)	56.6 (41.1 to 76.5)	0.7 (0.3 to 1.3)	38.7 (38.7 to 38.7)
Djibouti	1999	67.5 (56.3 to 80.7)	29.2 (22.4 to 36.9)	30.8 (22.3 to 41.5)	0.4 (0.2 to 0.7)	7.2 (7.2 to 7.2)	126.4 (105.3 to 151.1)	54.6 (42.0 to 69.1)	57.7 (41.7 to 77.7)	0.7 (0.4 to 1.4)	13.4 (13.4 to 13.4)
Djibouti	2000	69.8 (58.7 to 82.9)	27.9 (21.5 to 35.3)	30.3 (21.7 to 41.2)	0.4 (0.2 to 0.7)	11.2 (11.2 to 11.2)	130.6 (109.9 to 155.2)	52.2 (40.2 to 66.0)	56.7 (40.6 to 77.1)	0.7 (0.3 to 1.4)	20.9 (20.9 to 20.9)
Djibouti	2001	61.5 (50.9 to 73.8)	26.7 (20.5 to 33.6)	29.6 (21.2 to 39.7)	0.4 (0.2 to 0.7)	4.8 (4.8 to 4.8)	115.0 (95.2 to 138.1)	50.0 (38.4 to 62.8)	55.4 (39.7 to 74.2)	0.7 (0.3 to 1.3)	9.0 (9.0 to 9.0)
Djibouti	2002	59.5 (49.1 to 71.5)	26.5 (20.6 to 33.2)	28.7 (20.7 to 38.7)	0.4 (0.2 to 0.7)	4.0 (4.0 to 4.0)	111.4 (91.9 to 133.8)	49.6 (38.5 to 62.1)	53.7 (38.8 to 72.3)	0.7 (0.3 to 1.2)	7.4 (7.4 to 7.4)
Djibouti	2003	59.8 (49.1 to 71.8)	27.8 (21.4 to 35.0)	28.0 (20.1 to 37.2)	0.4 (0.2 to 0.7)	3.6 (3.6 to 3.6)	111.9 (91.9 to 134.3)	52.1 (40.1 to 65.5)	52.4 (37.7 to 69.7)	0.7 (0.3 to 1.3)	6.7 (6.7 to 6.7)
Djibouti	2004	66.2 (55.8 to 77.8)	28.6 (22.3 to 35.8)	26.8 (19.3 to 36.2)	0.4 (0.2 to 0.7)	10.4 (10.4 to 10.4)	124.0 (104.4 to 145.6)	53.6 (41.8 to 67.0)	50.2 (36.0 to 67.8)	0.7 (0.3 to 1.3)	19.5 (19.5 to 19.5)
Djibouti	2005	72.5 (62.3 to 83.6)	29.8 (23.2 to 37.4)	25.9 (18.7 to 34.7)	0.4 (0.2 to 0.7)	16.4 (16.4 to 16.4)	135.8 (116.6 to 156.5)	55.8 (43.4 to 70.1)	48.5 (35.0 to 65.0)	0.7 (0.3 to 1.3)	30.8 (30.8 to 30.8)
Djibouti	2006	75.6 (65.3 to 87.6)	32.8 (25.3 to 40.9)	25.2 (18.1 to 33.7)	0.3 (0.2 to 0.6)	17.2 (17.2 to 17.2)	141.5 (122.2 to 163.9)	61.4 (47.3 to 76.6)	47.2 (33.9 to 63.0)	0.6 (0.3 to 1.2)	32.3 (32.3 to 32.3)
Djibouti	2007	79.4 (69.2 to 91.3)	34.4 (26.5 to 42.8)	23.6 (17.2 to 31.6)	0.4 (0.2 to 0.6)	21.1 (21.1 to 21.1)	148.6 (129.6 to 170.8)	64.3 (49.6 to 80.2)	44.2 (32.2 to 59.2)	0.7 (0.3 to 1.2)	39.5 (39.5 to 39.5)
Djibouti	2008	78.6 (68.3 to 89.9)	36.7 (28.6 to 46.2)	21.8 (15.8 to 29.0)	0.4 (0.2 to 0.7)	19.7 (19.7 to 19.7)	147.0 (127.8 to 168.3)	68.7 (53.5 to 86.4)	40.7 (29.5 to 54.2)	0.7 (0.3 to 1.3)	37.0 (37.0 to 37.0)
Djibouti	2009	74.3 (64.0 to 85.0)	37.7 (29.1 to 47.0)	20.1 (14.6 to 26.9)	0.4 (0.2 to 0.7)	16.0 (16.0 to 16.0)	139.0 (119.9 to 159.2)	70.6 (54.5 to 87.9)	37.7 (27.3 to 50.3)	0.7 (0.4 to 1.4)	30.0 (30.0 to 30.0)
Djibouti	2010	66.3 (56.3 to 77.3)	38.5 (29.7 to 47.9)	18.7 (13.5 to 25.1)	0.4 (0.2 to 0.8)	8.7 (8.7 to 8.7)	124.2 (105.3 to 144.7)	72.0 (55.5 to 89.7)	35.1 (25.3 to 47.1)	0.8 (0.4 to 1.6)	16.2 (16.2 to 16.2)
Djibouti	2011	69.5 (59.0 to 80.5)	40.0 (30.8 to 50.0)	17.9 (12.9 to 24.1)	0.6 (0.3 to 1.1)	11.0 (11.0 to 11.0)	130.0 (110.4 to 150.6)	74.9 (57.7 to 93.6)	33.4 (24.2 to 45.1)	1.1 (0.5 to 2.1)	20.5 (20.5 to 20.5)
Djibouti	2012	75.9 (66.0 to 87.3)	39.8 (30.3 to 49.7)	16.9 (12.1 to 23.0)	0.7 (0.3 to 1.3)	18.4 (18.4 to 18.4)	142.0 (123.6 to 163.4)	74.5 (56.7 to 93.0)	31.7 (22.6 to 43.1)	1.3 (0.6 to 2.5)	34.5 (34.5 to 34.5)
Djibouti	2013	68.2 (58.1 to 79.2)	39.2 (29.8 to 49.6)	16.2 (11.4 to 21.9)	0.7 (0.3 to 1.3)	12.1 (12.1 to 12.1)	127.7 (108.7 to 148.2)	73.4 (55.8 to 92.9)	30.3 (21.4 to 41.0)	1.3 (0.6 to 2.4)	22.6 (22.6 to 22.6)
Djibouti	2014	66.5 (56.0 to 77.7)	39.5 (30.2 to 50.0)	16.0 (11.3 to 21.6)	0.8 (0.4 to 1.4)	10.3 (10.3 to 10.3)	124.4 (104.8 to 145.4)	73.8 (56.4 to 93.5)	29.9 (21.1 to 40.5)	1.4 (0.7 to 2.7)	19.2 (19.2 to 19.2)
Djibouti	2015	69.1 (59.0 to 80.8)	38.5 (29.3 to 49.1)	15.8 (11.2 to 21.4)	0.7 (0.3 to 1.4)	14.0 (14.0 to 14.0)	129.3 (110.4 to 151.3)	72.1 (54.9 to 91.9)	29.6 (20.9 to 40.1)	1.4 (0.7 to 2.6)	26.3 (26.3 to 26.3)

Country	Year	2018 US Dollars per capita					2018 PPP per capita				
		Health spending	Government health spending	Out-of-pocket health spending	Prepaid private health spending	Development assistance for health	Health spending	Government health spending	Out-of-pocket health spending	Prepaid private health spending	Development assistance for health
Djibouti	2016	66.4 (57.0 to 77.1)	35.1 (27.1 to 44.9)	15.6 (11.2 to 21.5)	0.6 (0.3 to 1.1)	15.1 (15.1 to 15.1)	124.3 (106.8 to 144.3)	65.7 (50.7 to 84.0)	29.3 (20.9 to 40.3)	1.1 (0.5 to 2.0)	28.3 (28.3 to 28.3)
Djibouti	2017	57.9 (49.5 to 67.1)	29.4 (22.7 to 37.7)	15.8 (11.2 to 21.7)	0.6 (0.3 to 1.2)	12.1 (12.1 to 12.1)	108.3 (92.7 to 125.5)	55.1 (42.6 to 70.6)	29.5 (21.0 to 40.6)	1.2 (0.6 to 2.2)	22.6 (22.6 to 22.6)
Djibouti	2018	57.6 (49.1 to 67.5)	30.0 (23.2 to 38.4)	15.8 (11.2 to 21.8)	0.6 (0.3 to 1.2)	11.2 (11.2 to 11.3)	107.9 (91.9 to 126.4)	56.2 (43.4 to 71.9)	29.5 (21.0 to 40.8)	1.2 (0.6 to 2.2)	21.0 (21.0 to 21.1)
Djibouti	2019	58.2 (49.5 to 68.2)	30.6 (23.6 to 39.1)	15.8 (11.2 to 21.8)	0.6 (0.3 to 1.2)	11.2 (10.4 to 11.8)	108.9 (92.6 to 127.6)	57.2 (44.2 to 73.2)	29.5 (20.9 to 40.8)	1.2 (0.6 to 2.2)	21.0 (19.5 to 22.2)
Djibouti	2020	58.8 (49.7 to 69.5)	31.2 (24.0 to 39.8)	15.8 (11.1 to 21.8)	0.7 (0.3 to 1.2)	11.2 (10.3 to 12.1)	110.1 (93.1 to 130.1)	58.3 (45.0 to 74.5)	29.5 (20.8 to 40.8)	1.2 (0.6 to 2.3)	21.0 (19.3 to 22.7)
Djibouti	2021	59.6 (50.6 to 70.0)	31.8 (24.5 to 40.6)	15.8 (11.2 to 22.0)	0.7 (0.3 to 1.3)	11.3 (10.2 to 12.5)	111.5 (94.7 to 131.0)	59.5 (45.9 to 76.1)	29.5 (20.9 to 41.2)	1.2 (0.6 to 2.3)	21.2 (19.1 to 23.4)
Djibouti	2022	60.2 (51.2 to 71.3)	32.3 (24.9 to 41.3)	15.8 (11.1 to 21.9)	0.7 (0.3 to 1.3)	11.5 (10.2 to 12.8)	112.8 (95.9 to 133.5)	60.5 (46.6 to 77.3)	29.5 (20.8 to 40.9)	1.3 (0.6 to 2.4)	21.5 (19.1 to 24.0)
Djibouti	2023	60.9 (51.7 to 71.4)	32.8 (25.3 to 42.0)	15.8 (11.2 to 22.1)	0.7 (0.3 to 1.3)	11.6 (10.1 to 13.2)	114.0 (96.8 to 133.6)	61.4 (47.3 to 78.6)	29.5 (20.9 to 41.3)	1.3 (0.6 to 2.4)	21.8 (19.0 to 24.7)
Djibouti	2024	61.6 (52.9 to 73.4)	33.3 (25.7 to 42.7)	15.8 (11.1 to 22.0)	0.7 (0.3 to 1.3)	11.8 (10.2 to 13.6)	115.3 (99.0 to 137.3)	62.4 (48.1 to 79.9)	29.5 (20.9 to 41.2)	1.3 (0.6 to 2.5)	22.1 (19.1 to 25.4)
Djibouti	2025	62.3 (52.3 to 73.7)	33.9 (26.1 to 43.3)	15.7 (11.1 to 21.9)	0.7 (0.3 to 1.3)	12.0 (10.2 to 14.0)	116.6 (98.0 to 138.0)	63.4 (48.8 to 81.1)	29.5 (20.7 to 40.9)	1.3 (0.6 to 2.5)	22.4 (19.2 to 26.3)
Djibouti	2026	62.9 (53.0 to 74.2)	34.3 (26.4 to 44.0)	15.7 (11.1 to 21.7)	0.7 (0.4 to 1.4)	12.2 (10.3 to 14.2)	117.8 (99.2 to 138.8)	64.3 (49.5 to 82.3)	29.4 (20.7 to 40.5)	1.4 (0.7 to 2.6)	22.7 (19.2 to 26.7)
Djibouti	2027	63.6 (53.2 to 75.3)	34.9 (26.8 to 44.6)	15.7 (11.0 to 21.5)	0.7 (0.4 to 1.4)	12.3 (10.4 to 14.7)	119.1 (99.5 to 140.8)	65.2 (50.1 to 83.5)	29.4 (20.6 to 40.2)	1.4 (0.7 to 2.6)	23.1 (19.4 to 27.4)
Djibouti	2028	64.4 (54.6 to 75.6)	35.3 (27.2 to 45.2)	15.7 (10.9 to 21.4)	0.7 (0.4 to 1.4)	12.6 (10.5 to 15.2)	120.5 (102.3 to 141.6)	66.2 (50.8 to 84.5)	29.4 (20.5 to 40.1)	1.4 (0.7 to 2.6)	23.5 (19.7 to 28.4)
Djibouti	2029	65.1 (54.4 to 76.5)	35.9 (27.6 to 45.8)	15.7 (10.9 to 21.5)	0.8 (0.4 to 1.4)	12.8 (10.6 to 15.7)	121.9 (101.9 to 143.2)	67.1 (51.7 to 85.8)	29.4 (20.4 to 40.2)	1.4 (0.7 to 2.7)	24.0 (19.8 to 29.4)
Djibouti	2030	65.9 (55.9 to 78.1)	36.4 (27.9 to 46.6)	15.7 (10.9 to 21.6)	0.8 (0.4 to 1.5)	13.0 (10.7 to 16.0)	123.4 (104.6 to 146.2)	68.1 (52.3 to 87.2)	29.4 (20.4 to 40.4)	1.4 (0.7 to 2.7)	24.4 (20.0 to 29.9)
Djibouti	2031	66.7 (55.4 to 79.6)	36.9 (28.4 to 47.4)	15.8 (10.9 to 21.7)	0.8 (0.4 to 1.5)	13.3 (10.8 to 16.6)	124.9 (103.7 to 149.0)	69.1 (53.2 to 88.7)	29.5 (20.4 to 40.7)	1.5 (0.7 to 2.8)	24.8 (20.1 to 31.1)
Djibouti	2032	67.5 (56.2 to 79.7)	37.4 (28.8 to 48.3)	15.8 (10.8 to 21.8)	0.8 (0.4 to 1.5)	13.5 (10.9 to 17.0)	126.4 (105.2 to 149.2)	70.0 (53.9 to 90.4)	29.6 (20.2 to 40.8)	1.5 (0.7 to 2.8)	25.3 (20.3 to 31.9)
Djibouti	2033	68.4 (57.3 to 81.2)	37.9 (29.2 to 49.1)	15.9 (10.8 to 21.9)	0.8 (0.4 to 1.5)	13.8 (11.0 to 17.7)	128.0 (107.2 to 152.0)	71.0 (54.6 to 91.8)	29.7 (20.3 to 41.1)	1.5 (0.7 to 2.9)	25.8 (20.5 to 33.1)
Djibouti	2034	69.3 (57.6 to 82.2)	38.5 (29.6 to 49.9)	15.9 (10.8 to 22.1)	0.8 (0.4 to 1.5)	14.1 (11.1 to 18.2)	129.7 (107.7 to 153.9)	72.0 (55.4 to 93.4)	29.8 (20.3 to 41.4)	1.5 (0.7 to 2.9)	26.4 (20.7 to 34.0)
Djibouti	2035	70.2 (59.0 to 83.9)	39.0 (29.9 to 50.5)	16.0 (10.8 to 22.3)	0.8 (0.4 to 1.6)	14.4 (11.2 to 18.8)	131.4 (110.5 to 157.1)	73.0 (56.0 to 94.5)	30.0 (20.2 to 41.7)	1.6 (0.8 to 2.9)	26.9 (20.9 to 35.1)
Djibouti	2036	71.2 (58.9 to 86.3)	39.5 (30.3 to 51.4)	16.1 (10.8 to 22.4)	0.9 (0.4 to 1.6)	14.6 (11.3 to 19.7)	133.2 (110.2 to 161.5)	74.0 (56.6 to 96.1)	30.2 (20.3 to 41.9)	1.6 (0.8 to 3.0)	27.4 (21.2 to 36.8)

		2018 US Dollars per capita					2018 PPP per capita				
Country	Year	Health spending	Government health spending	Out-of-pocket health spending	Prepaid private health spending	Development assistance for health	Health spending	Government health spending	Out-of-pocket health spending	Prepaid private health spending	Development assistance for health
Djibouti	2037	72.1 (59.8 to 87.0)	40.1 (30.6 to 52.3)	16.2 (10.8 to 22.7)	0.9 (0.4 to 1.6)	14.9 (11.4 to 20.0)	134.9 (112.0 to 162.8)	75.0 (57.3 to 97.9)	30.4 (20.3 to 42.4)	1.6 (0.8 to 3.0)	27.9 (21.4 to 37.4)
Djibouti	2038	73.0 (60.9 to 87.5)	40.6 (31.0 to 53.2)	16.4 (10.9 to 22.9)	0.9 (0.4 to 1.6)	15.2 (11.6 to 21.1)	136.7 (113.9 to 163.7)	76.0 (58.0 to 99.6)	30.6 (20.3 to 42.8)	1.6 (0.8 to 3.1)	28.5 (21.7 to 39.5)
Djibouti	2039	74.1 (61.5 to 90.0)	41.2 (31.3 to 54.1)	16.5 (10.9 to 23.1)	0.9 (0.4 to 1.7)	15.5 (11.8 to 21.5)	138.6 (115.1 to 168.4)	77.1 (58.6 to 101.3)	30.9 (20.4 to 43.3)	1.7 (0.8 to 3.1)	28.9 (22.1 to 40.2)
Djibouti	2040	75.0 (62.2 to 89.7)	41.7 (31.6 to 55.0)	16.6 (10.9 to 23.4)	0.9 (0.4 to 1.7)	15.8 (12.0 to 21.8)	140.5 (116.5 to 167.8)	78.1 (59.1 to 102.9)	31.2 (20.5 to 43.9)	1.7 (0.8 to 3.2)	29.5 (22.4 to 40.9)
Djibouti	2041	76.1 (62.9 to 92.0)	42.3 (31.9 to 55.7)	16.8 (11.0 to 23.8)	0.9 (0.4 to 1.7)	16.1 (12.1 to 22.3)	142.4 (117.7 to 172.3)	79.1 (59.7 to 104.2)	31.5 (20.6 to 44.5)	1.7 (0.8 to 3.2)	30.1 (22.7 to 41.8)
Djibouti	2042	77.1 (62.9 to 94.0)	42.8 (32.2 to 56.4)	17.0 (11.1 to 24.1)	0.9 (0.5 to 1.7)	16.4 (12.3 to 23.4)	144.4 (117.7 to 175.9)	80.1 (60.3 to 105.5)	31.8 (20.8 to 45.1)	1.7 (0.8 to 3.3)	30.7 (23.0 to 43.7)
Djibouti	2043	78.2 (64.7 to 94.6)	43.4 (32.6 to 57.1)	17.2 (11.2 to 24.5)	0.9 (0.5 to 1.8)	16.7 (12.4 to 24.2)	146.4 (121.1 to 177.0)	81.2 (61.0 to 106.9)	32.2 (21.0 to 45.8)	1.8 (0.9 to 3.3)	31.3 (23.2 to 45.2)
Djibouti	2044	79.4 (65.5 to 97.4)	44.0 (32.9 to 58.2)	17.4 (11.3 to 24.8)	1.0 (0.5 to 1.8)	17.0 (12.6 to 24.6)	148.5 (122.6 to 182.3)	82.3 (61.5 to 109.0)	32.6 (21.2 to 46.4)	1.8 (0.9 to 3.3)	31.9 (23.5 to 46.1)
Djibouti	2045	80.5 (65.4 to 99.6)	44.5 (33.2 to 59.4)	17.6 (11.5 to 25.2)	1.0 (0.5 to 1.8)	17.4 (12.5 to 26.2)	150.7 (122.3 to 186.5)	83.3 (62.1 to 111.2)	33.0 (21.5 to 47.1)	1.8 (0.9 to 3.4)	32.5 (23.4 to 49.1)
Djibouti	2046	81.7 (66.7 to 100.3)	45.1 (33.5 to 60.2)	17.9 (11.7 to 25.5)	1.0 (0.5 to 1.8)	17.7 (12.8 to 27.2)	152.9 (124.9 to 187.7)	84.4 (62.8 to 112.7)	33.5 (21.9 to 47.8)	1.9 (0.9 to 3.5)	33.1 (23.9 to 50.9)
Djibouti	2047	82.9 (67.1 to 101.8)	45.7 (33.9 to 61.0)	18.1 (11.9 to 26.0)	1.0 (0.5 to 1.9)	18.0 (12.9 to 28.4)	155.1 (125.5 to 190.6)	85.5 (63.5 to 114.2)	34.0 (22.3 to 48.6)	1.9 (0.9 to 3.5)	33.8 (24.2 to 53.2)
Djibouti	2048	84.2 (68.7 to 103.0)	46.3 (34.3 to 61.8)	18.4 (12.1 to 26.4)	1.0 (0.5 to 1.9)	18.4 (13.2 to 29.5)	157.5 (128.7 to 192.8)	86.7 (64.2 to 115.7)	34.5 (22.6 to 49.3)	1.9 (0.9 to 3.6)	34.5 (24.7 to 55.2)
Djibouti	2049	85.4 (69.7 to 105.3)	46.9 (34.6 to 62.9)	18.7 (12.3 to 26.7)	1.0 (0.5 to 1.9)	18.8 (13.4 to 30.2)	159.8 (130.4 to 197.1)	87.8 (64.8 to 117.7)	35.0 (23.0 to 50.0)	1.9 (0.9 to 3.6)	35.2 (25.1 to 56.6)
Djibouti	2050	86.8 (69.9 to 107.5)	47.5 (35.0 to 64.1)	19.0 (12.5 to 27.1)	1.1 (0.5 to 2.0)	19.2 (13.5 to 31.4)	162.4 (130.8 to 201.3)	89.0 (65.5 to 120.0)	35.5 (23.3 to 50.7)	2.0 (1.0 to 3.7)	36.0 (25.3 to 58.8)
Dominica	1995	346.1 (313.4 to 382.7)	218.0 (192.7 to 246.1)	126.4 (104.3 to 151.7)	1.6 (0.8 to 3.1)	0.0 (0.0 to 0.0)	504.7 (457.0 to 558.1)	318.0 (281.0 to 359.0)	184.4 (152.1 to 221.3)	2.4 (1.1 to 4.5)	0.0 (0.0 to 0.0)
Dominica	1996	355.2 (325.8 to 386.8)	224.8 (201.6 to 251.0)	128.5 (108.4 to 151.0)	1.5 (0.7 to 2.8)	0.3 (0.3 to 0.3)	518.0 (475.2 to 564.1)	327.9 (294.1 to 366.0)	187.4 (158.1 to 220.2)	2.2 (1.0 to 4.2)	0.5 (0.5 to 0.5)
Dominica	1997	373.2 (345.6 to 404.0)	227.4 (206.6 to 251.4)	129.5 (110.5 to 150.7)	1.3 (0.6 to 2.4)	15.0 (15.0 to 15.0)	544.3 (504.0 to 589.2)	331.6 (301.3 to 366.6)	188.9 (161.1 to 219.7)	1.8 (0.9 to 3.5)	21.9 (21.9 to 21.9)
Dominica	1998	380.5 (355.0 to 409.8)	235.1 (213.5 to 257.8)	133.0 (114.1 to 154.1)	0.9 (0.4 to 1.7)	11.5 (11.5 to 11.5)	554.9 (517.7 to 597.7)	342.9 (311.4 to 376.0)	194.0 (166.5 to 224.8)	1.3 (0.6 to 2.5)	16.7 (16.7 to 16.7)
Dominica	1999	360.8 (335.6 to 389.3)	228.6 (208.8 to 250.7)	131.1 (112.7 to 150.9)	0.4 (0.2 to 0.8)	0.6 (0.6 to 0.6)	526.2 (489.5 to 567.8)	333.4 (304.5 to 365.6)	191.2 (164.4 to 220.0)	0.6 (0.3 to 1.2)	0.8 (0.8 to 0.8)
Dominica	2000	333.7 (309.2 to 358.6)	212.9 (193.2 to 233.5)	120.5 (103.6 to 137.8)	0.1 (0.0 to 0.2)	0.2 (0.2 to 0.2)	486.6 (451.0 to 523.0)	310.4 (281.7 to 340.5)	175.8 (151.1 to 200.9)	0.2 (0.1 to 0.3)	0.2 (0.2 to 0.2)
Dominica	2001	313.2 (288.7 to 337.9)	201.4 (181.9 to 221.1)	111.1 (96.3 to 127.5)	0.0 (0.0 to 0.0)	0.7 (0.7 to 0.7)	456.8 (421.0 to 492.8)	293.7 (265.2 to 322.4)	162.1 (140.5 to 186.0)	0.0 (0.0 to 0.0)	1.1 (1.1 to 1.1)

		2018 US Dollars per capita					2018 PPP per capita				
Country	Year	Health spending	Government health spending	Out-of-pocket health spending	Prepaid private health spending	Development assistance for health	Health spending	Government health spending	Out-of-pocket health spending	Prepaid private health spending	Development assistance for health
Dominica	2002	304.8 (279.9 to 329.3)	194.4 (176.2 to 212.5)	110.0 (95.6 to 126.7)	0.0 (0.0 to 0.0)	0.3 (0.3 to 0.3)	444.5 (408.3 to 480.3)	283.5 (256.9 to 310.0)	160.5 (139.5 to 184.9)	0.0 (0.0 to 0.0)	0.5 (0.5 to 0.5)
Dominica	2003	317.1 (291.1 to 343.5)	196.4 (177.6 to 216.2)	116.6 (101.0 to 133.7)	0.0 (0.0 to 0.0)	4.1 (4.1 to 4.1)	462.4 (424.6 to 501.0)	286.5 (259.1 to 315.4)	170.0 (147.3 to 195.0)	0.0 (0.0 to 0.0)	6.0 (6.0 to 6.0)
Dominica	2004	337.4 (310.5 to 365.2)	197.9 (179.1 to 217.7)	133.6 (115.3 to 154.5)	0.3 (0.1 to 0.5)	5.6 (5.6 to 5.6)	492.1 (452.8 to 532.6)	288.6 (261.3 to 317.5)	194.9 (168.2 to 225.3)	0.4 (0.2 to 0.7)	8.2 (8.2 to 8.2)
Dominica	2005	347.0 (321.0 to 377.1)	197.7 (179.6 to 217.0)	141.8 (122.2 to 162.6)	1.4 (0.6 to 2.5)	6.2 (6.2 to 6.2)	506.1 (468.2 to 549.9)	288.4 (262.0 to 316.5)	206.8 (178.3 to 237.1)	2.0 (0.9 to 3.6)	9.0 (9.0 to 9.0)
Dominica	2006	370.9 (342.4 to 401.1)	207.7 (188.8 to 228.0)	150.0 (129.7 to 172.2)	3.0 (1.5 to 5.6)	10.1 (10.1 to 10.1)	541.0 (499.4 to 584.9)	303.0 (275.4 to 332.6)	218.8 (189.1 to 251.1)	4.4 (2.1 to 8.2)	14.7 (14.7 to 14.7)
Dominica	2007	392.2 (364.5 to 421.6)	220.7 (202.1 to 241.9)	161.0 (140.1 to 183.8)	4.5 (2.2 to 8.2)	5.9 (5.9 to 5.9)	572.0 (531.5 to 614.9)	321.9 (294.7 to 352.7)	234.8 (204.3 to 268.0)	6.6 (3.3 to 12.0)	8.7 (8.7 to 8.7)
Dominica	2008	426.0 (396.0 to 459.0)	237.0 (217.0 to 257.7)	172.5 (150.5 to 195.5)	5.5 (2.7 to 10.0)	11.0 (11.0 to 11.0)	621.3 (577.6 to 669.5)	345.7 (316.4 to 375.8)	251.6 (219.4 to 285.1)	8.0 (4.0 to 14.6)	16.0 (16.0 to 16.0)
Dominica	2009	431.0 (400.2 to 464.2)	242.9 (223.2 to 263.9)	169.1 (147.5 to 191.6)	5.8 (2.9 to 10.7)	13.1 (13.1 to 13.1)	628.5 (583.7 to 677.0)	354.3 (325.5 to 384.9)	246.6 (215.2 to 279.5)	8.5 (4.2 to 15.6)	19.1 (19.1 to 19.1)
Dominica	2010	431.1 (399.9 to 461.1)	251.8 (230.5 to 274.2)	162.3 (141.1 to 184.1)	6.0 (3.0 to 11.1)	11.0 (11.0 to 11.0)	628.8 (583.2 to 672.4)	367.3 (336.2 to 399.9)	236.8 (205.8 to 268.5)	8.8 (4.3 to 16.2)	16.0 (16.0 to 16.0)
Dominica	2011	430.6 (401.3 to 460.4)	256.9 (235.6 to 280.5)	156.5 (136.3 to 176.5)	6.0 (3.0 to 11.3)	11.2 (11.2 to 11.2)	628.1 (585.2 to 671.5)	374.7 (343.7 to 409.1)	228.2 (198.8 to 257.4)	8.8 (4.3 to 16.4)	16.3 (16.3 to 16.3)
Dominica	2012	422.9 (395.6 to 453.4)	257.2 (236.1 to 280.6)	153.7 (133.7 to 173.9)	6.0 (2.9 to 11.0)	6.0 (6.0 to 6.0)	616.7 (576.9 to 661.3)	375.1 (344.4 to 409.3)	224.1 (194.9 to 253.6)	8.7 (4.2 to 16.1)	8.8 (8.8 to 8.8)
Dominica	2013	424.6 (396.3 to 456.4)	258.3 (236.3 to 281.2)	149.7 (130.0 to 170.8)	5.9 (2.9 to 10.8)	10.8 (10.8 to 10.8)	619.3 (577.9 to 665.7)	376.7 (344.6 to 410.1)	218.3 (189.5 to 249.2)	8.6 (4.2 to 15.7)	15.7 (15.7 to 15.7)
Dominica	2014	430.1 (396.7 to 462.5)	272.6 (248.2 to 297.4)	148.2 (128.9 to 171.0)	6.0 (2.9 to 10.6)	3.2 (3.2 to 3.2)	627.3 (578.6 to 674.6)	397.6 (362.0 to 433.7)	216.2 (187.9 to 249.4)	8.8 (4.3 to 15.5)	4.7 (4.7 to 4.7)
Dominica	2015	438.9 (401.9 to 475.1)	282.7 (255.8 to 309.4)	142.7 (120.9 to 166.7)	6.1 (2.8 to 10.9)	7.4 (7.4 to 7.4)	640.1 (586.1 to 693.0)	412.2 (373.1 to 451.2)	208.2 (176.4 to 243.1)	8.8 (4.2 to 15.9)	10.8 (10.8 to 10.8)
Dominica	2016	437.6 (397.5 to 478.7)	290.7 (260.6 to 323.6)	137.5 (114.2 to 163.0)	6.0 (2.8 to 11.1)	3.5 (3.5 to 3.5)	638.2 (579.7 to 698.1)	424.0 (380.1 to 471.9)	200.5 (166.5 to 237.7)	8.7 (4.1 to 16.2)	5.0 (5.0 to 5.0)
Dominica	2017	420.9 (384.0 to 460.6)	277.0 (248.2 to 308.2)	131.3 (108.8 to 154.7)	5.8 (2.8 to 10.8)	6.8 (6.8 to 6.8)	613.9 (560.1 to 671.7)	404.0 (362.0 to 449.5)	191.5 (158.7 to 225.6)	8.5 (4.0 to 15.8)	10.0 (10.0 to 10.0)
Dominica	2018	425.7 (387.6 to 465.3)	281.6 (252.3 to 313.9)	131.9 (109.5 to 155.5)	5.9 (2.8 to 11.0)	6.2 (6.1 to 6.3)	620.9 (565.3 to 678.6)	410.8 (368.0 to 457.8)	192.4 (159.7 to 226.8)	8.6 (4.1 to 16.0)	9.1 (9.0 to 9.2)
Dominica	2019	433.0 (394.0 to 473.1)	287.5 (257.3 to 320.3)	133.1 (110.6 to 157.1)	6.0 (2.8 to 11.2)	6.3 (5.9 to 6.7)	631.4 (574.6 to 690.0)	419.3 (375.3 to 467.2)	194.2 (161.2 to 229.0)	8.7 (4.1 to 16.3)	9.2 (8.6 to 9.8)
Dominica	2020	440.2 (400.8 to 482.5)	293.4 (261.1 to 327.3)	134.3 (110.8 to 158.6)	6.1 (2.9 to 11.4)	6.4 (5.9 to 7.0)	642.0 (584.5 to 703.7)	428.0 (380.8 to 477.3)	195.8 (161.6 to 231.3)	8.9 (4.2 to 16.6)	9.4 (8.5 to 10.2)
Dominica	2021	447.6 (408.1 to 490.1)	299.5 (267.6 to 333.4)	135.3 (112.7 to 159.8)	6.2 (3.0 to 11.6)	6.6 (5.9 to 7.3)	652.8 (595.2 to 714.8)	436.8 (390.3 to 486.3)	197.4 (164.4 to 233.1)	9.1 (4.3 to 16.9)	9.6 (8.6 to 10.6)
Dominica	2022	455.4 (414.2 to 498.0)	305.7 (273.2 to 341.5)	136.6 (113.8 to 161.3)	6.3 (3.0 to 11.7)	6.8 (5.9 to 7.6)	664.1 (604.0 to 726.3)	445.9 (398.4 to 498.1)	199.2 (165.9 to 235.2)	9.2 (4.4 to 17.1)	9.9 (8.7 to 11.1)

Country	Year	2018 US Dollars per capita					2018 PPP per capita				
		Health spending	Government health spending	Out-of-pocket health spending	Prepaid private health spending	Development assistance for health	Health spending	Government health spending	Out-of-pocket health spending	Prepaid private health spending	Development assistance for health
Dominica	2023	463.2 (422.0 to 506.3)	312.0 (278.2 to 348.7)	137.8 (114.8 to 162.9)	6.4 (3.1 to 12.0)	7.0 (6.0 to 8.0)	675.6 (615.5 to 738.4)	455.0 (405.8 to 508.6)	201.0 (167.5 to 237.6)	9.4 (4.5 to 17.5)	10.2 (8.8 to 11.7)
Dominica	2024	469.7 (425.9 to 514.3)	316.9 (282.9 to 353.8)	139.1 (115.8 to 164.4)	6.5 (3.1 to 12.1)	7.2 (6.1 to 8.3)	685.0 (621.2 to 750.1)	462.2 (412.6 to 516.0)	202.8 (168.9 to 239.8)	9.5 (4.5 to 17.7)	10.5 (8.9 to 12.2)
Dominica	2025	477.3 (434.3 to 521.9)	322.9 (287.9 to 361.7)	140.3 (116.9 to 166.1)	6.7 (3.2 to 12.4)	7.4 (6.2 to 8.7)	696.1 (633.4 to 761.2)	470.9 (419.9 to 527.5)	204.7 (170.4 to 242.2)	9.7 (4.6 to 18.0)	10.8 (9.1 to 12.7)
Dominica	2026	484.7 (439.7 to 530.9)	328.9 (293.1 to 368.6)	141.4 (117.8 to 167.6)	6.8 (3.2 to 12.6)	7.6 (6.4 to 8.9)	706.9 (641.3 to 774.3)	479.7 (427.5 to 537.6)	206.3 (171.8 to 244.4)	9.9 (4.7 to 18.3)	11.1 (9.3 to 13.0)
Dominica	2027	492.3 (446.8 to 539.4)	335.1 (297.3 to 375.6)	142.5 (118.6 to 169.0)	6.9 (3.3 to 12.8)	7.8 (6.5 to 9.3)	718.0 (651.7 to 786.7)	488.7 (433.6 to 547.8)	207.8 (172.9 to 246.5)	10.0 (4.8 to 18.6)	11.4 (9.5 to 13.6)
Dominica	2028	499.9 (453.8 to 548.7)	341.3 (302.6 to 383.1)	143.6 (119.2 to 170.4)	7.0 (3.3 to 13.0)	8.1 (6.7 to 9.8)	729.0 (661.9 to 800.2)	497.7 (441.4 to 558.7)	209.4 (173.9 to 248.5)	10.2 (4.8 to 18.9)	11.7 (9.8 to 14.2)
Dominica	2029	507.6 (458.8 to 559.4)	347.7 (308.4 to 390.9)	144.5 (119.8 to 171.5)	7.1 (3.4 to 13.2)	8.3 (6.8 to 10.2)	740.3 (669.2 to 815.8)	507.1 (449.7 to 570.1)	210.8 (174.8 to 250.2)	10.4 (4.9 to 19.2)	12.1 (9.9 to 14.9)
Dominica	2030	515.4 (466.8 to 567.1)	354.4 (313.5 to 399.4)	145.3 (120.5 to 172.4)	7.2 (3.4 to 13.3)	8.5 (7.0 to 10.5)	751.7 (680.7 to 827.1)	516.8 (457.2 to 582.5)	211.8 (175.7 to 251.4)	10.5 (5.0 to 19.4)	12.5 (10.2 to 15.3)
Dominica	2031	522.9 (470.2 to 577.7)	361.0 (316.5 to 408.7)	145.8 (121.0 to 172.9)	7.3 (3.5 to 13.5)	8.8 (7.1 to 11.0)	762.7 (685.7 to 842.5)	526.5 (461.7 to 596.1)	212.7 (176.5 to 252.2)	10.7 (5.1 to 19.6)	12.9 (10.3 to 16.1)
Dominica	2032	530.5 (476.7 to 583.9)	367.8 (320.0 to 418.8)	146.2 (121.4 to 173.3)	7.4 (3.5 to 13.6)	9.1 (7.3 to 11.5)	773.7 (695.2 to 851.5)	536.4 (466.7 to 610.7)	213.2 (177.0 to 252.7)	10.8 (5.1 to 19.8)	13.3 (10.6 to 16.8)
Dominica	2033	537.8 (478.6 to 599.0)	374.6 (322.7 to 427.6)	146.3 (121.5 to 173.4)	7.5 (3.6 to 13.7)	9.4 (7.5 to 12.0)	784.4 (698.0 to 873.6)	546.4 (470.6 to 623.6)	213.4 (177.2 to 252.8)	10.9 (5.2 to 20.0)	13.7 (10.9 to 17.5)
Dominica	2034	545.1 (487.2 to 606.8)	381.4 (325.3 to 438.0)	146.5 (121.6 to 173.6)	7.6 (3.6 to 13.9)	9.7 (7.6 to 12.6)	795.1 (710.5 to 885.0)	556.2 (474.4 to 638.7)	213.6 (177.4 to 253.2)	11.1 (5.2 to 20.2)	14.2 (11.1 to 18.3)
Dominica	2035	552.9 (489.8 to 618.3)	388.2 (328.6 to 447.6)	146.9 (122.1 to 174.3)	7.7 (3.6 to 14.0)	10.0 (7.8 to 13.2)	806.3 (714.4 to 901.8)	566.2 (479.2 to 652.8)	214.3 (178.1 to 254.2)	11.2 (5.3 to 20.4)	14.6 (11.3 to 19.2)
Dominica	2036	561.0 (496.1 to 629.6)	395.1 (332.2 to 457.1)	147.7 (122.9 to 175.3)	7.8 (3.7 to 14.2)	10.4 (8.0 to 14.1)	818.2 (723.5 to 918.2)	576.2 (484.6 to 666.7)	215.5 (179.2 to 255.7)	11.4 (5.4 to 20.7)	15.1 (11.7 to 20.5)
Dominica	2037	569.5 (497.7 to 640.5)	402.0 (336.5 to 465.9)	149.0 (123.9 to 176.9)	7.9 (3.7 to 14.4)	10.7 (8.2 to 14.5)	830.6 (725.8 to 934.1)	586.2 (490.8 to 679.5)	217.3 (180.7 to 257.9)	11.5 (5.5 to 21.1)	15.6 (11.9 to 21.1)
Dominica	2038	578.6 (511.0 to 652.2)	408.9 (341.7 to 475.6)	150.6 (125.3 to 178.7)	8.1 (3.8 to 14.7)	11.0 (8.4 to 15.3)	843.9 (745.3 to 951.2)	596.4 (498.3 to 693.6)	219.6 (182.7 to 260.6)	11.8 (5.5 to 21.5)	16.1 (12.2 to 22.3)
Dominica	2039	588.6 (515.0 to 664.1)	416.3 (347.9 to 484.1)	152.7 (126.8 to 181.1)	8.2 (3.9 to 15.0)	11.3 (8.6 to 15.6)	858.4 (751.1 to 968.5)	607.2 (507.5 to 706.0)	222.7 (185.0 to 264.2)	12.0 (5.6 to 21.9)	16.5 (12.5 to 22.8)
Dominica	2040	598.7 (529.7 to 680.0)	423.9 (354.4 to 493.4)	154.7 (128.4 to 183.4)	8.4 (3.9 to 15.3)	11.7 (8.9 to 16.2)	873.2 (772.6 to 991.8)	618.2 (516.8 to 719.7)	225.6 (187.3 to 267.5)	12.3 (5.7 to 22.4)	17.1 (13.0 to 23.6)
Dominica	2041	608.9 (534.1 to 687.6)	431.4 (361.0 to 504.0)	156.8 (130.0 to 185.8)	8.6 (4.0 to 15.7)	12.1 (9.1 to 17.0)	888.1 (778.9 to 1002.8)	629.2 (526.5 to 735.0)	228.8 (189.6 to 271.0)	12.5 (5.8 to 22.9)	17.6 (13.3 to 24.8)
Dominica	2042	619.1 (546.1 to 699.7)	439.0 (367.3 to 513.8)	158.9 (131.6 to 188.1)	8.7 (4.1 to 16.0)	12.5 (9.3 to 18.0)	903.0 (796.5 to 1020.5)	640.3 (535.7 to 749.3)	231.7 (191.9 to 274.4)	12.8 (5.9 to 23.4)	18.2 (13.6 to 26.2)
Dominica	2043	629.0 (551.6 to 712.8)	446.6 (372.4 to 524.7)	160.6 (132.9 to 190.1)	8.9 (4.1 to 16.4)	12.9 (9.5 to 18.8)	917.3 (804.5 to 1039.6)	651.4 (543.2 to 765.2)	234.2 (193.8 to 277.2)	13.0 (6.0 to 23.9)	18.8 (13.9 to 27.4)

Country	Year	2018 US Dollars per capita					2018 PPP per capita				
		Health spending	Government health spending	Out-of-pocket health spending	Prepaid private health spending	Development assistance for health	Health spending	Government health spending	Out-of-pocket health spending	Prepaid private health spending	Development assistance for health
Dominica	2044	638.9 (556.2 to 722.9)	454.5 (377.7 to 534.0)	162.0 (134.1 to 191.9)	9.1 (4.2 to 16.7)	13.3 (9.8 to 19.4)	931.8 (811.2 to 1054.4)	662.9 (550.9 to 778.8)	236.3 (195.6 to 279.9)	13.2 (6.1 to 24.3)	19.4 (14.3 to 28.4)
Dominica	2045	648.4 (566.2 to 737.8)	462.2 (382.3 to 545.6)	163.2 (135.1 to 193.4)	9.2 (4.2 to 16.9)	13.8 (10.0 to 20.7)	945.7 (825.7 to 1076.1)	674.1 (557.5 to 795.7)	238.0 (197.0 to 282.1)	13.4 (6.2 to 24.6)	20.1 (14.6 to 30.2)
Dominica	2046	657.8 (570.5 to 747.8)	469.9 (387.1 to 557.1)	164.3 (135.8 to 194.9)	9.3 (4.3 to 17.1)	14.3 (10.3 to 22.0)	959.4 (832.1 to 1090.5)	685.3 (564.6 to 812.6)	239.6 (198.0 to 284.3)	13.6 (6.3 to 25.0)	20.9 (15.0 to 32.1)
Dominica	2047	667.7 (579.2 to 765.1)	477.8 (392.7 to 567.7)	165.6 (136.6 to 196.4)	9.5 (4.4 to 17.4)	14.8 (10.7 to 23.1)	973.8 (844.7 to 1115.9)	696.8 (572.7 to 827.9)	241.4 (199.2 to 286.4)	13.8 (6.4 to 25.4)	21.6 (15.6 to 33.7)
Dominica	2048	677.9 (585.3 to 775.4)	485.8 (397.9 to 579.0)	167.0 (137.6 to 198.2)	9.7 (4.4 to 17.8)	15.4 (11.0 to 24.5)	988.6 (853.6 to 1130.9)	708.5 (580.3 to 844.4)	243.6 (200.7 to 289.1)	14.1 (6.5 to 25.9)	22.4 (16.0 to 35.7)
Dominica	2049	687.4 (593.6 to 788.4)	493.5 (402.5 to 587.9)	168.1 (138.6 to 199.7)	9.8 (4.5 to 18.1)	16.0 (11.4 to 25.7)	1002.6 (865.7 to 1149.8)	719.8 (587.1 to 857.4)	245.2 (202.1 to 291.2)	14.3 (6.6 to 26.4)	23.3 (16.6 to 37.4)
Dominica	2050	697.1 (599.6 to 800.6)	501.2 (407.5 to 600.3)	169.4 (139.4 to 201.3)	10.0 (4.6 to 18.4)	16.6 (11.7 to 27.2)	1016.7 (874.5 to 1167.6)	731.0 (594.3 to 875.4)	247.0 (203.3 to 293.6)	14.5 (6.7 to 26.8)	24.2 (17.1 to 39.7)
Dominican Republic	1995	151.7 (129.9 to 176.1)	29.5 (23.9 to 35.9)	104.0 (85.1 to 126.9)	16.9 (9.3 to 29.1)	1.2 (1.2 to 1.2)	359.1 (307.5 to 417.0)	69.8 (56.6 to 85.0)	246.3 (201.5 to 300.5)	40.0 (21.9 to 68.9)	2.9 (2.9 to 2.9)
Dominican Republic	1996	162.2 (142.2 to 185.4)	33.9 (27.9 to 40.9)	104.8 (88.2 to 126.0)	18.0 (10.0 to 30.0)	5.6 (5.6 to 5.6)	384.0 (336.6 to 438.9)	80.2 (66.2 to 96.9)	248.0 (208.8 to 298.3)	42.6 (23.6 to 71.1)	13.2 (13.2 to 13.2)
Dominican Republic	1997	170.0 (149.7 to 192.4)	42.1 (35.2 to 50.0)	107.4 (91.4 to 127.1)	18.0 (10.2 to 29.4)	2.6 (2.6 to 2.6)	402.5 (354.4 to 455.6)	99.6 (83.2 to 118.3)	254.2 (216.4 to 301.0)	42.5 (24.2 to 69.7)	6.2 (6.2 to 6.2)
Dominican Republic	1998	184.2 (163.1 to 206.1)	51.2 (43.0 to 60.7)	108.7 (93.4 to 126.6)	18.1 (10.3 to 29.3)	6.2 (6.2 to 6.2)	436.0 (386.1 to 488.0)	121.2 (101.8 to 143.6)	257.4 (221.2 to 299.7)	42.8 (24.4 to 69.3)	14.7 (14.7 to 14.7)
Dominican Republic	1999	192.6 (170.7 to 215.4)	58.8 (49.1 to 69.5)	108.3 (92.9 to 126.0)	17.8 (10.1 to 28.8)	7.7 (7.7 to 7.7)	456.1 (404.0 to 510.0)	139.1 (116.3 to 164.5)	256.4 (219.9 to 298.2)	42.3 (23.8 to 68.3)	18.3 (18.3 to 18.3)
Dominican Republic	2000	191.1 (171.3 to 213.2)	62.5 (52.2 to 73.3)	107.4 (92.2 to 125.4)	17.0 (9.8 to 27.5)	4.3 (4.3 to 4.3)	452.5 (405.5 to 504.8)	147.9 (123.6 to 173.5)	254.2 (218.3 to 296.8)	40.3 (23.2 to 65.1)	10.2 (10.2 to 10.2)
Dominican Republic	2001	190.2 (170.4 to 211.5)	63.0 (53.1 to 73.4)	106.8 (91.4 to 123.6)	16.4 (9.9 to 26.7)	4.0 (4.0 to 4.0)	450.3 (403.4 to 500.7)	149.1 (125.7 to 173.7)	252.8 (216.4 to 292.7)	38.9 (23.3 to 63.3)	9.5 (9.5 to 9.5)
Dominican Republic	2002	190.0 (170.6 to 210.6)	62.2 (52.7 to 72.5)	107.7 (92.9 to 124.4)	16.4 (9.9 to 27.2)	3.7 (3.7 to 3.7)	449.9 (403.9 to 498.6)	147.2 (124.7 to 171.5)	255.0 (219.8 to 294.5)	38.8 (23.5 to 64.3)	8.8 (8.8 to 8.8)
Dominican Republic	2003	178.2 (160.1 to 196.9)	55.2 (47.1 to 64.9)	103.4 (89.8 to 118.6)	15.4 (9.2 to 25.2)	4.2 (4.2 to 4.2)	421.8 (379.1 to 466.1)	130.6 (111.6 to 153.8)	244.8 (212.5 to 280.9)	36.5 (21.7 to 59.5)	9.9 (9.9 to 9.9)
Dominican Republic	2004	179.0 (161.7 to 198.7)	55.2 (47.2 to 64.3)	101.6 (88.8 to 116.6)	15.9 (9.3 to 26.0)	6.3 (6.3 to 6.3)	423.8 (382.8 to 470.4)	130.6 (111.7 to 152.3)	240.6 (210.3 to 276.0)	37.7 (22.0 to 61.6)	14.9 (14.9 to 14.9)
Dominican Republic	2005	193.3 (175.3 to 213.8)	63.3 (54.6 to 73.9)	103.0 (89.6 to 117.8)	18.8 (11.0 to 30.0)	8.2 (8.2 to 8.2)	457.7 (415.0 to 506.2)	149.9 (129.2 to 175.1)	243.7 (212.2 to 278.8)	44.5 (26.0 to 71.0)	19.5 (19.5 to 19.5)
Dominican Republic	2006	203.3 (184.0 to 223.4)	74.0 (63.7 to 86.1)	104.2 (90.4 to 119.5)	19.7 (11.7 to 31.1)	5.4 (5.4 to 5.4)	481.4 (435.6 to 528.9)	175.2 (150.7 to 203.8)	246.7 (214.0 to 282.9)	46.6 (27.7 to 73.7)	12.8 (12.8 to 12.8)
Dominican Republic	2007	221.7 (200.7 to 243.3)	88.6 (76.3 to 102.7)	105.7 (91.9 to 120.6)	21.9 (12.8 to 34.6)	5.5 (5.5 to 5.5)	524.9 (475.2 to 575.9)	209.7 (180.7 to 243.1)	250.3 (217.5 to 285.6)	51.8 (30.4 to 82.0)	13.1 (13.1 to 13.1)
Dominican Republic	2008	241.6 (218.1 to 264.9)	103.4 (89.6 to 118.4)	109.2 (94.6 to 125.2)	23.4 (13.8 to 36.8)	5.7 (5.7 to 5.7)	572.0 (516.3 to 627.2)	244.7 (212.1 to 280.3)	258.5 (223.9 to 296.3)	55.4 (32.7 to 87.1)	13.4 (13.4 to 13.4)

		2018 US Dollars per capita					2018 PPP per capita				
**Country**	**Year**	**Health spending**	**Government health spending**	**Out-of-pocket health spending**	**Prepaid private health spending**	**Development assistance for health**	**Health spending**	**Government health spending**	**Out-of-pocket health spending**	**Prepaid private health spending**	**Development assistance for health**
Dominican Republic	2009	268.4 (243.4 to 295.5)	116.4 (101.5 to 132.8)	115.0 (99.8 to 131.9)	24.5 (14.8 to 38.5)	12.4 (12.4 to 12.4)	635.3 (576.2 to 699.5)	275.6 (240.3 to 314.4)	272.2 (236.3 to 312.3)	58.1 (35.1 to 91.2)	29.4 (29.4 to 29.4)
Dominican Republic	2010	299.0 (271.4 to 329.5)	133.9 (117.1 to 151.8)	126.8 (110.8 to 145.5)	26.5 (15.9 to 40.3)	11.9 (11.9 to 11.9)	707.8 (642.6 to 780.1)	317.0 (277.2 to 359.5)	300.1 (262.4 to 344.5)	62.6 (37.7 to 95.4)	28.1 (28.1 to 28.1)
Dominican Republic	2011	311.0 (282.4 to 340.8)	144.6 (127.1 to 164.2)	133.4 (116.7 to 152.3)	27.3 (16.6 to 41.7)	5.7 (5.7 to 5.7)	736.2 (668.5 to 806.8)	342.3 (300.9 to 388.7)	315.8 (276.2 to 360.6)	64.7 (39.4 to 98.8)	13.5 (13.5 to 13.5)
Dominican Republic	2012	329.2 (299.9 to 359.6)	152.1 (134.5 to 172.5)	140.4 (122.8 to 160.1)	28.8 (17.7 to 44.8)	7.9 (7.9 to 7.9)	779.3 (709.9 to 851.4)	360.0 (318.4 to 408.4)	332.4 (290.6 to 378.9)	68.3 (41.9 to 105.9)	18.6 (18.6 to 18.6)
Dominican Republic	2013	343.5 (313.6 to 375.0)	158.3 (139.5 to 178.7)	147.1 (128.5 to 166.8)	30.2 (18.5 to 46.8)	7.9 (7.9 to 7.9)	813.2 (742.5 to 887.7)	374.8 (330.2 to 423.1)	348.3 (304.1 to 394.9)	71.4 (43.9 to 110.7)	18.7 (18.7 to 18.7)
Dominican Republic	2014	364.8 (333.0 to 399.5)	164.4 (145.0 to 186.0)	157.4 (136.0 to 179.1)	31.9 (19.4 to 50.2)	11.0 (11.0 to 11.0)	863.6 (788.3 to 945.7)	389.3 (343.2 to 440.3)	372.7 (322.0 to 424.0)	75.6 (45.9 to 118.7)	26.1 (26.1 to 26.1)
Dominican Republic	2015	405.9 (370.6 to 447.1)	173.9 (152.2 to 199.2)	170.1 (145.8 to 196.1)	34.8 (20.8 to 54.6)	27.1 (27.1 to 27.1)	960.9 (877.4 to 1058.5)	411.8 (360.2 to 471.5)	402.6 (345.1 to 464.2)	82.3 (49.1 to 129.3)	64.2 (64.2 to 64.2)
Dominican Republic	2016	420.3 (377.5 to 467.5)	190.3 (165.0 to 220.5)	185.2 (156.1 to 218.8)	38.7 (22.6 to 63.7)	6.2 (6.2 to 6.2)	995.0 (893.6 to 1106.7)	450.4 (390.7 to 522.1)	438.4 (369.4 to 518.0)	91.6 (53.6 to 150.7)	14.6 (14.6 to 14.6)
Dominican Republic	2017	441.5 (397.5 to 489.3)	204.6 (177.5 to 237.8)	191.4 (161.7 to 226.8)	40.5 (23.6 to 66.7)	5.0 (5.0 to 5.0)	1045.1 (941.1 to 1158.4)	484.4 (420.2 to 562.9)	453.1 (382.8 to 537.0)	95.8 (55.8 to 158.0)	11.8 (11.8 to 11.8)
Dominican Republic	2018	452.4 (407.5 to 502.0)	210.6 (182.8 to 244.4)	195.9 (165.4 to 232.1)	41.4 (24.1 to 68.1)	4.5 (4.5 to 4.5)	1071.0 (964.7 to 1188.3)	498.6 (432.8 to 578.6)	463.8 (391.5 to 549.4)	98.0 (57.2 to 161.3)	10.7 (10.6 to 10.8)
Dominican Republic	2019	462.7 (416.2 to 512.7)	216.3 (187.6 to 251.5)	199.6 (167.2 to 235.8)	42.2 (24.6 to 69.5)	4.5 (4.2 to 4.8)	1095.4 (985.3 to 1213.7)	512.1 (444.1 to 595.4)	472.5 (395.9 to 558.3)	100.0 (58.2 to 164.4)	10.7 (10.0 to 11.4)
Dominican Republic	2020	477.5 (430.5 to 529.3)	224.9 (195.0 to 261.2)	204.4 (170.9 to 241.3)	43.6 (25.4 to 71.5)	4.6 (4.2 to 4.9)	1130.4 (1019.1 to 1253.2)	532.4 (461.7 to 618.3)	484.0 (404.5 to 571.4)	103.3 (60.1 to 169.4)	10.8 (9.9 to 11.7)
Dominican Republic	2021	492.6 (443.4 to 546.9)	233.7 (202.2 to 270.8)	209.2 (175.1 to 246.7)	45.0 (26.2 to 74.0)	4.6 (4.1 to 5.1)	1166.1 (1049.6 to 1294.7)	553.3 (478.6 to 641.0)	495.2 (414.5 to 584.1)	106.7 (62.1 to 175.2)	10.9 (9.8 to 12.0)
Dominican Republic	2022	508.4 (457.2 to 565.7)	243.1 (210.4 to 281.1)	214.1 (179.3 to 252.5)	46.5 (27.1 to 76.4)	4.7 (4.2 to 5.3)	1203.7 (1082.4 to 1339.2)	575.5 (498.1 to 665.6)	506.9 (424.5 to 597.8)	110.2 (64.1 to 180.9)	11.1 (9.9 to 12.4)
Dominican Republic	2023	524.7 (470.5 to 584.9)	252.7 (218.7 to 292.2)	219.1 (183.9 to 258.9)	48.1 (28.1 to 79.2)	4.8 (4.1 to 5.4)	1242.1 (1113.8 to 1384.8)	598.3 (517.7 to 691.9)	518.7 (435.3 to 612.8)	113.8 (66.4 to 187.4)	11.3 (9.8 to 12.7)
Dominican Republic	2024	540.9 (485.3 to 602.4)	262.4 (227.0 to 304.3)	224.1 (188.1 to 264.5)	49.6 (29.0 to 81.6)	4.8 (4.2 to 5.6)	1280.6 (1148.9 to 1426.2)	621.2 (537.5 to 720.4)	530.4 (445.3 to 626.2)	117.5 (68.7 to 193.2)	11.5 (9.9 to 13.2)
Dominican Republic	2025	557.9 (499.2 to 623.7)	272.6 (235.8 to 316.6)	229.2 (192.4 to 270.3)	51.2 (29.9 to 84.1)	4.9 (4.2 to 5.8)	1320.8 (1181.8 to 1476.6)	645.3 (558.3 to 749.5)	542.5 (455.5 to 639.9)	121.3 (70.8 to 199.1)	11.7 (10.0 to 13.7)
Dominican Republic	2026	575.7 (513.5 to 642.2)	283.2 (244.3 to 329.3)	234.6 (196.8 to 277.0)	52.9 (30.9 to 87.0)	5.0 (4.2 to 5.9)	1362.9 (1215.6 to 1520.4)	670.5 (578.4 to 779.7)	555.3 (466.0 to 655.8)	125.3 (73.2 to 206.0)	11.8 (10.0 to 13.9)
Dominican Republic	2027	593.8 (530.2 to 661.1)	294.2 (252.6 to 343.2)	240.0 (201.3 to 284.1)	54.7 (32.0 to 89.5)	5.0 (4.2 to 6.0)	1405.9 (1255.3 to 1565.0)	696.4 (598.0 to 812.4)	568.2 (476.6 to 672.6)	129.4 (75.7 to 212.0)	11.9 (10.0 to 14.2)
Dominican Republic	2028	613.0 (546.8 to 684.2)	305.7 (261.2 to 356.4)	245.7 (206.2 to 291.3)	56.5 (33.0 to 92.3)	5.1 (4.3 to 6.2)	1451.2 (1294.5 to 1619.8)	723.8 (618.4 to 843.7)	581.6 (488.1 to 689.6)	133.7 (78.1 to 218.4)	12.1 (10.1 to 14.7)
Dominican Republic	2029	632.7 (564.1 to 707.8)	317.8 (271.5 to 371.8)	251.4 (210.9 to 298.0)	58.3 (34.0 to 95.0)	5.2 (4.2 to 6.4)	1497.9 (1335.4 to 1675.6)	752.3 (642.8 to 880.3)	595.2 (499.3 to 705.6)	138.1 (80.6 to 224.9)	12.3 (10.0 to 15.1)

		2018 US Dollars per capita					2018 PPP per capita				
Country	Year	Health spending	Government health spending	Out-of-pocket health spending	Prepaid private health spending	Development assistance for health	Health spending	Government health spending	Out-of-pocket health spending	Prepaid private health spending	Development assistance for health
Dominican Republic	2030	652.9 (582.7 to 730.4)	330.3 (281.8 to 387.3)	257.2 (215.4 to 305.3)	60.2 (35.1 to 97.8)	5.2 (4.2 to 6.5)	1545.7 (1379.4 to 1729.0)	781.9 (667.2 to 916.9)	608.8 (509.9 to 722.7)	142.6 (83.1 to 231.6)	12.4 (10.0 to 15.4)
Dominican Republic	2031	673.7 (598.5 to 753.8)	343.2 (291.8 to 402.4)	263.1 (219.5 to 312.4)	62.2 (36.2 to 101.3)	5.3 (4.2 to 6.7)	1595.0 (1416.8 to 1784.7)	812.4 (690.7 to 952.6)	622.9 (519.6 to 739.6)	147.2 (85.6 to 239.9)	12.6 (10.0 to 15.9)
Dominican Republic	2032	695.7 (617.3 to 780.7)	356.8 (303.1 to 419.7)	269.3 (224.6 to 320.4)	64.3 (37.3 to 104.6)	5.4 (4.2 to 6.9)	1647.0 (1461.4 to 1848.3)	844.6 (717.5 to 993.6)	637.6 (531.7 to 758.6)	152.1 (88.3 to 247.7)	12.7 (10.0 to 16.3)
Dominican Republic	2033	717.6 (636.5 to 806.6)	370.4 (314.9 to 437.1)	275.4 (229.5 to 327.8)	66.3 (38.5 to 107.9)	5.5 (4.2 to 7.0)	1698.8 (1506.9 to 1909.5)	876.8 (745.6 to 1034.8)	652.1 (543.4 to 776.0)	157.0 (91.0 to 255.4)	12.9 (9.9 to 16.7)
Dominican Republic	2034	741.1 (656.6 to 835.9)	385.1 (326.4 to 455.5)	282.0 (235.3 to 336.2)	68.5 (39.8 to 111.7)	5.5 (4.2 to 7.3)	1754.6 (1554.4 to 1979.0)	911.7 (772.7 to 1078.2)	667.5 (557.1 to 796.0)	162.3 (94.3 to 264.4)	13.1 (9.9 to 17.4)
Dominican Republic	2035	764.9 (675.7 to 860.5)	400.0 (336.6 to 473.3)	288.5 (240.8 to 344.2)	70.8 (41.2 to 115.3)	5.6 (4.2 to 7.5)	1810.9 (1599.7 to 2037.3)	946.9 (796.8 to 1120.5)	683.1 (570.0 to 814.7)	167.6 (97.5 to 272.9)	13.3 (9.8 to 17.7)
Dominican Republic	2036	789.1 (698.6 to 887.2)	415.2 (348.3 to 492.3)	295.1 (245.6 to 352.9)	73.1 (42.4 to 118.8)	5.7 (4.1 to 7.8)	1868.1 (1653.9 to 2100.3)	983.0 (824.6 to 1165.6)	698.7 (581.4 to 835.6)	173.0 (100.4 to 281.2)	13.4 (9.8 to 18.4)
Dominican Republic	2037	813.7 (718.1 to 916.4)	430.8 (360.6 to 509.2)	301.8 (250.4 to 362.0)	75.4 (43.7 to 122.8)	5.7 (3.9 to 8.0)	1926.3 (1700.1 to 2169.4)	1019.8 (853.6 to 1205.5)	714.5 (592.7 to 857.0)	178.5 (103.4 to 290.6)	13.4 (9.3 to 18.9)
Dominican Republic	2038	839.3 (740.2 to 945.3)	447.1 (374.3 to 530.8)	308.7 (255.5 to 370.8)	77.8 (44.9 to 127.1)	5.8 (3.9 to 8.3)	1987.1 (1752.3 to 2237.9)	1058.4 (886.1 to 1256.7)	730.8 (605.0 to 877.7)	184.3 (106.3 to 300.9)	13.7 (9.2 to 19.6)
Dominican Republic	2039	865.7 (760.7 to 975.1)	464.0 (387.9 to 552.2)	315.7 (261.4 to 378.8)	80.3 (46.2 to 131.6)	5.7 (0.0 to 8.7)	2049.6 (1800.8 to 2308.4)	1098.4 (918.4 to 1307.3)	747.4 (618.8 to 896.9)	190.2 (109.5 to 311.5)	13.5 (0.0 to 20.6)
Dominican Republic	2040	892.6 (785.8 to 1010.6)	481.3 (401.1 to 573.9)	322.8 (267.2 to 387.0)	82.9 (47.7 to 136.2)	5.6 (0.0 to 8.8)	2113.3 (1860.2 to 2392.5)	1139.5 (949.7 to 1358.6)	764.3 (632.6 to 916.2)	196.3 (112.9 to 322.4)	13.2 (0.0 to 20.8)
Dominican Republic	2041	920.5 (810.0 to 1045.7)	499.1 (414.9 to 596.0)	330.1 (272.9 to 395.6)	85.6 (49.3 to 141.0)	5.6 (0.0 to 9.1)	2179.1 (1917.7 to 2475.7)	1181.7 (982.3 to 1411.0)	781.5 (646.2 to 936.7)	202.5 (116.7 to 333.7)	13.3 (0.0 to 21.5)
Dominican Republic	2042	948.9 (838.3 to 1077.8)	517.6 (429.4 to 620.3)	337.6 (278.9 to 404.9)	88.3 (50.9 to 145.8)	5.5 (0.0 to 9.4)	2246.5 (1984.7 to 2551.7)	1225.3 (1016.5 to 1468.6)	799.2 (660.2 to 958.5)	209.0 (120.4 to 345.2)	13.1 (0.0 to 22.4)
Dominican Republic	2043	978.3 (862.4 to 1113.0)	536.6 (444.3 to 643.5)	345.1 (284.9 to 414.6)	91.1 (52.5 to 150.2)	5.5 (0.0 to 9.9)	2316.0 (2041.7 to 2634.9)	1270.4 (1051.8 to 1523.4)	817.0 (674.5 to 981.4)	215.6 (124.3 to 355.6)	13.0 (0.0 to 23.4)
Dominican Republic	2044	1008.4 (885.7 to 1149.9)	556.3 (458.6 to 669.0)	352.7 (290.4 to 424.2)	94.0 (54.2 to 154.7)	5.4 (0.0 to 10.0)	2387.3 (2096.9 to 2722.2)	1317.0 (1085.8 to 1583.9)	835.1 (687.5 to 1004.2)	222.4 (128.3 to 366.2)	12.8 (0.0 to 23.7)
Dominican Republic	2045	1038.3 (908.3 to 1185.2)	576.0 (472.8 to 694.5)	360.2 (296.6 to 433.4)	96.8 (55.8 to 159.1)	5.4 (0.0 to 10.4)	2458.2 (2150.3 to 2805.8)	1363.5 (1119.2 to 1644.1)	852.7 (702.1 to 1026.1)	229.2 (132.2 to 376.7)	12.8 (0.0 to 24.7)
Dominican Republic	2046	1069.0 (936.9 to 1219.3)	596.3 (488.7 to 718.9)	367.7 (302.9 to 442.6)	99.7 (57.5 to 163.7)	5.3 (0.0 to 10.8)	2530.8 (2218.1 to 2886.6)	1411.6 (1157.0 to 1702.0)	870.5 (717.1 to 1047.8)	236.1 (136.2 to 387.5)	12.6 (0.0 to 25.6)
Dominican Republic	2047	1099.8 (964.6 to 1260.8)	616.7 (503.6 to 746.8)	375.2 (309.3 to 451.6)	102.7 (59.3 to 168.2)	5.2 (0.0 to 11.1)	2603.7 (2283.7 to 2984.9)	1459.9 (1192.1 to 1768.1)	888.2 (732.1 to 1069.2)	243.1 (140.3 to 398.1)	12.4 (0.0 to 26.2)
Dominican Republic	2048	1131.1 (986.8 to 1299.1)	637.6 (518.7 to 772.4)	382.6 (315.4 to 460.9)	105.7 (61.0 to 172.9)	5.2 (0.0 to 11.4)	2677.7 (2336.2 to 3075.4)	1509.4 (1228.0 to 1828.7)	905.8 (746.7 to 1091.1)	250.2 (144.5 to 409.3)	12.4 (0.0 to 27.1)
Dominican Republic	2049	1162.0 (1012.2 to 1340.6)	658.3 (533.7 to 798.7)	390.0 (321.6 to 470.0)	108.7 (62.8 to 177.5)	5.1 (0.0 to 11.7)	2751.0 (2396.4 to 3173.6)	1558.5 (1263.5 to 1890.9)	923.2 (761.3 to 1112.8)	257.3 (148.6 to 420.3)	12.0 (0.0 to 27.8)
Dominican Republic	2050	1193.2 (1033.2 to 1375.7)	679.3 (550.2 to 826.8)	397.3 (327.6 to 479.0)	111.7 (64.3 to 182.8)	4.9 (0.0 to 12.8)	2824.8 (2446.1 to 3256.8)	1608.1 (1302.6 to 1957.4)	940.6 (775.6 to 1133.9)	264.5 (152.3 to 432.7)	11.6 (0.0 to 30.4)

Country	Year	2018 US Dollars per capita					2018 PPP per capita				
		Health spending	Government health spending	Out-of-pocket health spending	Prepaid private health spending	Development assistance for health	Health spending	Government health spending	Out-of-pocket health spending	Prepaid private health spending	Development assistance for health
Ecuador	1995	204.7 (179.6 to 231.2)	113.9 (96.3 to 133.0)	66.8 (53.9 to 81.1)	20.7 (11.3 to 35.6)	3.4 (3.4 to 3.4)	387.7 (340.2 to 437.8)	215.7 (182.3 to 251.8)	126.5 (102.1 to 153.6)	39.2 (21.4 to 67.5)	6.4 (6.4 to 6.4)
Ecuador	1996	207.0 (182.6 to 232.8)	112.7 (96.8 to 129.4)	70.9 (58.5 to 84.6)	20.9 (11.6 to 35.1)	2.5 (2.5 to 2.5)	392.0 (345.8 to 440.9)	213.5 (183.4 to 245.1)	134.3 (110.8 to 160.2)	39.6 (21.9 to 66.6)	4.7 (4.7 to 4.7)
Ecuador	1997	205.0 (182.5 to 229.9)	103.4 (89.9 to 117.8)	79.3 (66.4 to 93.9)	20.1 (11.3 to 32.6)	2.1 (2.1 to 2.1)	388.2 (345.7 to 435.4)	195.9 (170.2 to 223.2)	150.2 (125.8 to 177.8)	38.1 (21.3 to 61.7)	4.0 (4.0 to 4.0)
Ecuador	1998	198.8 (178.1 to 221.3)	86.8 (75.1 to 99.7)	90.0 (77.0 to 105.0)	17.7 (10.0 to 28.6)	4.3 (4.3 to 4.3)	376.5 (337.2 to 419.1)	164.4 (142.3 to 188.8)	170.4 (145.8 to 198.8)	33.4 (18.9 to 54.1)	8.2 (8.2 to 8.2)
Ecuador	1999	184.7 (166.6 to 204.1)	71.7 (62.5 to 83.2)	96.4 (83.2 to 111.5)	14.0 (8.1 to 22.9)	2.5 (2.5 to 2.5)	349.8 (315.5 to 386.6)	135.9 (118.3 to 157.5)	182.7 (157.6 to 211.2)	26.4 (15.3 to 43.3)	4.8 (4.8 to 4.8)
Ecuador	2000	172.9 (155.9 to 190.5)	61.0 (53.1 to 71.0)	100.0 (86.7 to 114.9)	9.5 (5.5 to 15.4)	2.3 (2.3 to 2.3)	327.4 (295.2 to 360.8)	115.5 (100.6 to 134.5)	189.5 (164.2 to 217.7)	18.0 (10.3 to 29.2)	4.4 (4.4 to 4.4)
Ecuador	2001	186.4 (169.3 to 205.4)	62.3 (54.3 to 72.6)	111.9 (97.8 to 127.6)	8.9 (5.1 to 14.4)	3.2 (3.2 to 3.2)	353.0 (320.6 to 389.0)	118.0 (102.8 to 137.6)	212.0 (185.2 to 241.6)	16.9 (9.6 to 27.2)	6.1 (6.1 to 6.1)
Ecuador	2002	211.1 (190.9 to 231.5)	67.7 (58.8 to 78.2)	131.3 (114.6 to 149.2)	10.9 (6.0 to 17.3)	1.1 (1.1 to 1.1)	399.7 (361.6 to 438.5)	128.2 (111.4 to 148.0)	248.8 (217.0 to 282.6)	20.6 (11.4 to 32.7)	2.1 (2.1 to 2.1)
Ecuador	2003	242.1 (218.2 to 265.1)	74.5 (64.5 to 85.6)	151.3 (133.1 to 171.0)	14.1 (7.8 to 22.1)	2.2 (2.2 to 2.2)	458.5 (413.2 to 502.1)	141.1 (122.2 to 162.1)	286.5 (252.0 to 323.8)	26.8 (14.8 to 41.9)	4.1 (4.1 to 4.1)
Ecuador	2004	270.4 (244.7 to 295.9)	83.7 (72.7 to 95.6)	167.4 (148.3 to 189.0)	16.8 (9.5 to 25.8)	2.5 (2.5 to 2.5)	512.2 (463.4 to 560.5)	158.5 (137.8 to 181.1)	317.0 (281.0 to 357.9)	31.9 (17.9 to 48.9)	4.7 (4.7 to 4.7)
Ecuador	2005	290.1 (263.1 to 315.4)	92.0 (80.1 to 105.5)	176.8 (157.9 to 199.0)	18.4 (10.4 to 28.4)	2.8 (2.8 to 2.8)	549.4 (498.3 to 597.4)	174.3 (151.7 to 199.7)	334.9 (299.0 to 376.9)	34.8 (19.7 to 53.7)	5.4 (5.4 to 5.4)
Ecuador	2006	306.1 (278.1 to 333.7)	103.0 (90.2 to 118.2)	179.8 (160.3 to 201.3)	19.9 (11.6 to 31.1)	3.4 (3.4 to 3.4)	579.8 (526.7 to 631.9)	195.0 (170.9 to 223.8)	340.6 (303.6 to 381.2)	37.7 (21.9 to 58.9)	6.4 (6.4 to 6.4)
Ecuador	2007	321.0 (294.1 to 350.5)	117.0 (102.9 to 131.7)	177.2 (158.3 to 198.6)	22.5 (13.3 to 34.6)	4.3 (4.3 to 4.3)	607.9 (556.9 to 663.9)	221.5 (194.8 to 249.4)	335.6 (299.8 to 376.2)	42.6 (25.2 to 65.6)	8.2 (8.2 to 8.2)
Ecuador	2008	346.4 (318.8 to 376.9)	136.3 (120.2 to 153.3)	180.9 (161.8 to 201.5)	25.2 (15.3 to 39.5)	4.1 (4.1 to 4.1)	656.1 (603.8 to 713.9)	258.2 (227.7 to 290.3)	342.5 (306.4 to 381.6)	47.7 (29.1 to 74.9)	7.7 (7.7 to 7.7)
Ecuador	2009	368.2 (339.2 to 397.6)	153.5 (137.1 to 169.9)	184.8 (165.6 to 205.6)	26.7 (16.2 to 42.7)	3.1 (3.1 to 3.1)	697.3 (642.5 to 753.0)	290.7 (259.6 to 321.8)	350.1 (313.6 to 389.4)	50.6 (30.7 to 80.9)	5.9 (5.9 to 5.9)
Ecuador	2010	408.4 (378.0 to 440.7)	181.1 (163.6 to 199.7)	195.1 (174.5 to 217.1)	29.4 (18.0 to 46.5)	2.9 (2.9 to 2.9)	773.5 (716.0 to 834.6)	343.0 (309.8 to 378.3)	369.5 (330.4 to 411.2)	55.6 (34.2 to 88.1)	5.4 (5.4 to 5.4)
Ecuador	2011	466.5 (431.5 to 501.9)	218.2 (198.3 to 239.1)	212.0 (189.4 to 236.6)	33.2 (20.7 to 51.8)	3.2 (3.2 to 3.2)	883.6 (817.2 to 950.6)	413.3 (375.6 to 452.8)	401.5 (358.7 to 448.1)	62.8 (39.2 to 98.1)	6.0 (6.0 to 6.0)
Ecuador	2012	512.3 (474.8 to 547.8)	249.6 (229.2 to 273.0)	224.7 (202.0 to 249.3)	35.8 (22.1 to 56.3)	2.3 (2.3 to 2.3)	970.3 (899.3 to 1037.5)	472.8 (434.1 to 517.0)	425.5 (382.5 to 472.1)	67.8 (41.9 to 106.6)	4.3 (4.3 to 4.3)
Ecuador	2013	543.3 (507.4 to 580.5)	272.6 (249.5 to 295.9)	231.8 (207.3 to 258.1)	36.7 (23.1 to 57.4)	2.2 (2.2 to 2.2)	1028.9 (961.0 to 1099.4)	516.2 (472.6 to 560.5)	439.0 (392.7 to 488.8)	69.6 (43.7 to 108.8)	4.1 (4.1 to 4.1)
Ecuador	2014	561.1 (520.6 to 603.0)	285.1 (258.7 to 311.2)	235.3 (209.4 to 262.6)	37.4 (23.4 to 57.8)	3.3 (3.3 to 3.3)	1062.7 (985.9 to 1142.0)	539.9 (490.0 to 589.5)	445.6 (396.6 to 497.3)	70.9 (44.4 to 109.5)	6.2 (6.2 to 6.2)
Ecuador	2015	551.8 (507.6 to 595.6)	280.8 (252.2 to 310.7)	232.3 (204.0 to 261.7)	37.7 (23.1 to 59.4)	0.9 (0.9 to 0.9)	1045.0 (961.3 to 1128.1)	531.8 (477.7 to 588.4)	439.9 (386.3 to 495.7)	71.5 (43.8 to 112.5)	1.7 (1.7 to 1.7)

Country	Year	2018 US Dollars per capita					2018 PPP per capita				
		Health spending	Government health spending	Out-of-pocket health spending	Prepaid private health spending	Development assistance for health	Health spending	Government health spending	Out-of-pocket health spending	Prepaid private health spending	Development assistance for health
Ecuador	2016	535.7 (488.6 to 586.2)	273.7 (240.4 to 309.1)	221.6 (191.5 to 254.6)	39.5 (23.5 to 63.3)	1.0 (1.0 to 1.0)	1014.6 (925.4 to 1110.2)	518.3 (455.4 to 585.4)	419.7 (362.7 to 482.2)	74.8 (44.6 to 119.9)	1.8 (1.8 to 1.8)
Ecuador	2017	534.9 (487.4 to 586.3)	270.4 (237.8 to 305.0)	224.2 (193.7 to 257.9)	39.4 (23.5 to 63.3)	0.8 (0.8 to 0.8)	1013.0 (923.1 to 1110.4)	512.2 (450.3 to 577.7)	424.6 (366.9 to 488.5)	74.7 (44.5 to 119.9)	1.5 (1.5 to 1.5)
Ecuador	2018	543.1 (494.9 to 594.6)	275.9 (243.6 to 312.2)	226.2 (195.3 to 260.3)	40.3 (24.0 to 64.7)	0.8 (0.8 to 0.8)	1028.6 (937.3 to 1126.1)	522.6 (461.3 to 591.3)	428.3 (370.0 to 492.9)	76.3 (45.5 to 122.5)	1.4 (1.4 to 1.4)
Ecuador	2019	551.8 (503.8 to 604.1)	282.1 (248.9 to 319.4)	227.9 (196.9 to 262.0)	41.0 (24.4 to 65.7)	0.8 (0.7 to 0.8)	1045.1 (954.1 to 1144.2)	534.4 (471.3 to 604.9)	431.6 (373.0 to 496.1)	77.7 (46.2 to 124.4)	1.5 (1.4 to 1.5)
Ecuador	2020	560.7 (510.8 to 614.5)	288.4 (254.0 to 326.9)	229.7 (198.9 to 264.1)	41.7 (24.8 to 66.7)	0.8 (0.7 to 0.8)	1061.9 (967.3 to 1163.8)	546.3 (481.1 to 619.1)	435.1 (376.8 to 500.2)	79.1 (47.0 to 126.4)	1.5 (1.4 to 1.6)
Ecuador	2021	572.0 (521.4 to 628.6)	294.7 (259.7 to 334.0)	234.0 (202.4 to 269.7)	42.5 (25.3 to 67.8)	0.8 (0.7 to 0.9)	1083.2 (987.5 to 1190.5)	558.1 (491.9 to 632.6)	443.2 (383.3 to 510.7)	80.5 (47.9 to 128.5)	1.5 (1.4 to 1.7)
Ecuador	2022	583.3 (531.4 to 640.5)	300.8 (265.2 to 340.5)	238.5 (205.3 to 275.3)	43.2 (25.8 to 69.1)	0.8 (0.7 to 0.9)	1104.7 (1006.4 to 1213.1)	569.7 (502.3 to 644.8)	451.6 (388.8 to 521.4)	81.9 (48.9 to 130.9)	1.5 (1.4 to 1.7)
Ecuador	2023	594.8 (542.0 to 653.0)	307.0 (271.2 to 347.3)	243.0 (209.7 to 279.4)	44.0 (26.3 to 70.5)	0.8 (0.7 to 0.9)	1126.5 (1026.6 to 1236.8)	581.4 (513.7 to 657.7)	460.2 (397.1 to 529.1)	83.3 (49.8 to 133.5)	1.6 (1.4 to 1.8)
Ecuador	2024	606.4 (552.9 to 666.2)	313.3 (277.0 to 355.1)	247.5 (212.0 to 284.5)	44.7 (26.6 to 71.9)	0.8 (0.7 to 1.0)	1148.5 (1047.2 to 1261.7)	593.4 (524.6 to 672.5)	468.7 (401.6 to 538.9)	84.7 (50.4 to 136.2)	1.6 (1.4 to 1.8)
Ecuador	2025	616.9 (562.0 to 679.4)	319.7 (282.4 to 361.7)	250.9 (216.7 to 290.2)	45.5 (26.9 to 73.1)	0.9 (0.7 to 1.0)	1168.4 (1064.3 to 1286.7)	605.5 (534.8 to 685.1)	475.2 (410.5 to 549.5)	86.1 (51.0 to 138.4)	1.6 (1.4 to 1.9)
Ecuador	2026	627.7 (570.6 to 690.9)	326.1 (287.5 to 370.0)	254.5 (218.8 to 295.2)	46.2 (27.4 to 74.2)	0.9 (0.7 to 1.0)	1188.8 (1080.6 to 1308.5)	617.6 (544.5 to 700.7)	481.9 (414.4 to 559.0)	87.6 (51.8 to 140.6)	1.7 (1.4 to 2.0)
Ecuador	2027	639.0 (580.2 to 701.8)	332.8 (293.3 to 378.2)	258.2 (221.3 to 301.8)	47.1 (27.9 to 75.6)	0.9 (0.8 to 1.1)	1210.2 (1098.8 to 1329.1)	630.4 (555.4 to 716.2)	489.0 (419.2 to 571.6)	89.1 (52.9 to 143.1)	1.7 (1.4 to 2.0)
Ecuador	2028	650.4 (590.8 to 716.9)	339.7 (298.5 to 385.4)	261.9 (223.3 to 307.4)	47.9 (28.5 to 77.0)	0.9 (0.8 to 1.1)	1231.9 (1119.0 to 1357.7)	643.4 (565.4 to 730.0)	496.1 (422.9 to 582.3)	90.7 (53.9 to 145.9)	1.7 (1.5 to 2.1)
Ecuador	2029	662.0 (599.6 to 728.2)	346.7 (304.2 to 393.1)	265.6 (225.1 to 312.9)	48.7 (29.0 to 78.5)	0.9 (0.8 to 1.2)	1253.7 (1135.7 to 1379.2)	656.5 (576.0 to 744.4)	503.1 (426.4 to 592.5)	92.3 (55.0 to 148.6)	1.8 (1.5 to 2.2)
Ecuador	2030	673.6 (609.4 to 744.3)	353.7 (309.8 to 401.9)	269.3 (227.1 to 318.3)	49.6 (29.6 to 79.7)	1.0 (0.8 to 1.2)	1275.8 (1154.2 to 1409.6)	669.9 (586.7 to 761.2)	510.1 (430.1 to 602.9)	93.9 (56.1 to 150.9)	1.8 (1.5 to 2.2)
Ecuador	2031	685.0 (618.5 to 759.5)	360.6 (314.8 to 411.2)	273.0 (228.8 to 326.8)	50.4 (30.2 to 80.8)	1.0 (0.8 to 1.2)	1297.3 (1171.5 to 1438.4)	683.0 (596.2 to 778.9)	517.0 (433.3 to 619.0)	95.5 (57.1 to 153.1)	1.9 (1.5 to 2.3)
Ecuador	2032	696.8 (626.0 to 776.5)	367.8 (320.3 to 421.1)	276.7 (230.5 to 333.7)	51.3 (30.7 to 82.2)	1.0 (0.8 to 1.3)	1319.7 (1185.7 to 1470.7)	696.6 (606.7 to 797.6)	524.1 (436.6 to 632.0)	97.1 (58.1 to 155.7)	1.9 (1.5 to 2.4)
Ecuador	2033	708.6 (637.9 to 794.3)	375.1 (325.9 to 429.8)	280.4 (232.3 to 341.7)	52.1 (31.1 to 83.7)	1.0 (0.8 to 1.3)	1342.1 (1208.2 to 1504.3)	710.4 (617.3 to 814.0)	531.0 (440.0 to 647.2)	98.8 (58.9 to 158.5)	2.0 (1.6 to 2.5)
Ecuador	2034	720.6 (647.1 to 808.2)	382.5 (332.0 to 439.9)	284.0 (233.8 to 349.5)	53.0 (31.5 to 85.1)	1.1 (0.8 to 1.4)	1364.8 (1225.6 to 1530.7)	724.4 (628.8 to 833.2)	537.9 (442.7 to 662.0)	100.4 (59.7 to 161.2)	2.0 (1.6 to 2.6)
Ecuador	2035	732.6 (655.6 to 823.0)	390.0 (338.1 to 450.0)	287.6 (235.5 to 356.7)	53.9 (32.0 to 86.6)	1.1 (0.8 to 1.4)	1387.5 (1241.7 to 1558.8)	738.6 (640.3 to 852.2)	544.8 (446.0 to 675.6)	102.1 (60.6 to 164.0)	2.1 (1.6 to 2.7)
Ecuador	2036	744.5 (665.2 to 841.2)	397.3 (343.5 to 459.6)	291.3 (237.4 to 364.0)	54.8 (32.5 to 88.2)	1.1 (0.9 to 1.5)	1410.1 (1259.9 to 1593.3)	752.5 (650.6 to 870.5)	551.6 (449.6 to 689.3)	103.8 (61.6 to 167.1)	2.1 (1.6 to 2.8)

		2018 US Dollars per capita					2018 PPP per capita				
Country	Year	Health spending	Government health spending	Out-of-pocket health spending	Prepaid private health spending	Development assistance for health	Health spending	Government health spending	Out-of-pocket health spending	Prepaid private health spending	Development assistance for health
Ecuador	2037	756.4 (674.0 to 852.8)	404.7 (349.9 to 468.9)	294.8 (239.1 to 371.6)	55.7 (33.1 to 89.9)	1.1 (0.9 to 1.5)	1432.5 (1276.4 to 1615.1)	766.5 (662.6 to 888.1)	558.3 (452.9 to 703.8)	105.5 (62.6 to 170.2)	2.2 (1.7 to 2.9)
Ecuador	2038	768.5 (683.2 to 870.1)	412.3 (355.1 to 478.1)	298.4 (241.0 to 380.2)	56.6 (33.6 to 91.4)	1.2 (0.9 to 1.6)	1455.5 (1294.0 to 1648.0)	780.9 (672.5 to 905.4)	565.1 (456.4 to 720.1)	107.2 (63.6 to 173.2)	2.2 (1.7 to 3.1)
Ecuador	2039	780.9 (692.3 to 880.9)	420.2 (361.1 to 488.2)	302.0 (242.8 to 387.2)	57.6 (34.1 to 93.3)	1.2 (0.9 to 1.7)	1479.0 (1311.2 to 1668.4)	795.8 (683.8 to 924.5)	571.9 (459.8 to 733.4)	109.0 (64.5 to 176.8)	2.3 (1.7 to 3.2)
Ecuador	2040	793.1 (700.2 to 901.9)	427.9 (367.5 to 498.8)	305.4 (244.4 to 393.7)	58.5 (34.6 to 95.1)	1.2 (0.9 to 1.7)	1502.1 (1326.2 to 1708.2)	810.5 (696.1 to 944.6)	578.4 (462.9 to 745.6)	110.8 (65.4 to 180.0)	2.3 (1.8 to 3.2)
Ecuador	2041	805.3 (708.2 to 919.4)	435.6 (373.1 to 508.2)	308.9 (246.6 to 399.8)	59.5 (35.0 to 96.8)	1.3 (1.0 to 1.8)	1525.1 (1341.4 to 1741.3)	825.0 (706.7 to 962.4)	585.1 (467.0 to 757.2)	112.6 (66.4 to 183.4)	2.4 (1.8 to 3.3)
Ecuador	2042	817.5 (717.7 to 930.2)	443.4 (378.8 to 517.9)	312.4 (248.5 to 405.8)	60.4 (35.5 to 98.6)	1.3 (1.0 to 1.9)	1548.3 (1359.4 to 1761.7)	839.8 (717.5 to 980.8)	591.7 (470.6 to 768.6)	114.4 (67.3 to 186.8)	2.5 (1.8 to 3.5)
Ecuador	2043	829.8 (728.9 to 952.4)	451.3 (384.6 to 527.3)	315.8 (250.1 to 411.9)	61.4 (36.0 to 100.3)	1.3 (1.0 to 1.9)	1571.7 (1380.5 to 1803.9)	854.7 (728.5 to 998.6)	598.1 (473.7 to 780.1)	116.3 (68.2 to 190.0)	2.5 (1.9 to 3.7)
Ecuador	2044	842.1 (736.8 to 963.4)	459.2 (390.6 to 537.4)	319.1 (251.5 to 419.2)	62.4 (36.5 to 101.9)	1.4 (1.0 to 2.0)	1594.9 (1395.4 to 1824.5)	869.7 (739.8 to 1017.8)	604.4 (476.4 to 793.9)	118.1 (69.0 to 193.1)	2.6 (1.9 to 3.8)
Ecuador	2045	854.6 (746.6 to 985.3)	467.3 (395.7 to 550.3)	322.4 (252.9 to 425.8)	63.4 (36.9 to 103.6)	1.4 (1.0 to 2.1)	1618.5 (1414.0 to 1866.2)	885.1 (749.4 to 1042.2)	610.7 (479.0 to 806.4)	120.0 (69.9 to 196.1)	2.7 (1.9 to 4.0)
Ecuador	2046	867.1 (758.3 to 1002.9)	475.5 (402.1 to 560.8)	325.8 (254.4 to 428.8)	64.4 (37.4 to 105.3)	1.5 (1.0 to 2.2)	1642.2 (1436.2 to 1899.4)	900.5 (761.6 to 1062.2)	617.0 (481.9 to 812.1)	122.0 (70.8 to 199.4)	2.8 (2.0 to 4.2)
Ecuador	2047	879.4 (766.6 to 1014.6)	483.5 (407.6 to 572.1)	329.0 (255.9 to 434.6)	65.4 (37.8 to 107.2)	1.5 (1.1 to 2.4)	1665.6 (1452.0 to 1921.5)	915.8 (771.9 to 1083.5)	623.1 (484.6 to 823.1)	123.9 (71.7 to 203.0)	2.8 (2.0 to 4.5)
Ecuador	2048	891.9 (776.1 to 1034.2)	491.7 (413.2 to 584.2)	332.3 (257.3 to 440.2)	66.4 (38.4 to 109.1)	1.5 (1.1 to 2.5)	1689.2 (1469.8 to 1958.8)	931.2 (782.7 to 1106.5)	629.3 (487.3 to 833.7)	125.8 (72.7 to 206.7)	2.9 (2.1 to 4.7)
Ecuador	2049	904.6 (787.8 to 1047.9)	500.0 (418.7 to 595.9)	335.5 (259.5 to 447.2)	67.5 (38.9 to 111.0)	1.6 (1.1 to 2.6)	1713.2 (1492.1 to 1984.6)	947.0 (792.9 to 1128.7)	635.5 (491.6 to 847.1)	127.8 (73.7 to 210.3)	3.0 (2.1 to 4.8)
Ecuador	2050	917.3 (798.0 to 1066.5)	508.4 (424.4 to 607.5)	338.7 (261.3 to 454.4)	68.5 (39.5 to 112.7)	1.6 (1.2 to 2.7)	1737.3 (1511.3 to 2019.8)	962.9 (803.8 to 1150.5)	641.5 (494.8 to 860.6)	129.7 (74.8 to 213.4)	3.1 (2.2 to 5.1)
Egypt	1995	90.5 (74.5 to 108.6)	30.3 (23.4 to 38.4)	56.1 (42.6 to 73.0)	1.7 (0.8 to 3.1)	2.3 (2.3 to 2.3)	418.7 (344.6 to 502.5)	140.4 (108.4 to 177.7)	259.8 (197.2 to 337.7)	7.8 (3.6 to 14.6)	10.6 (10.6 to 10.6)
Egypt	1996	94.0 (78.3 to 112.9)	31.3 (24.2 to 39.9)	58.8 (45.1 to 75.5)	1.8 (0.9 to 3.4)	2.1 (2.1 to 2.1)	434.9 (362.5 to 522.2)	144.8 (112.0 to 184.5)	271.9 (208.9 to 349.2)	8.4 (4.0 to 15.7)	9.7 (9.7 to 9.7)
Egypt	1997	97.2 (81.8 to 116.5)	32.2 (24.9 to 40.7)	61.2 (47.4 to 78.0)	1.8 (0.8 to 3.4)	2.0 (2.0 to 2.0)	450.0 (378.5 to 539.3)	149.1 (115.3 to 188.2)	283.3 (219.4 to 360.7)	8.4 (3.9 to 15.9)	9.2 (9.2 to 9.2)
Egypt	1998	100.7 (85.3 to 118.7)	33.2 (25.8 to 42.1)	63.3 (50.0 to 79.0)	2.0 (0.9 to 3.7)	2.2 (2.2 to 2.2)	465.8 (394.9 to 549.2)	153.9 (119.5 to 194.8)	292.8 (231.5 to 365.4)	9.2 (4.3 to 16.9)	10.0 (10.0 to 10.0)
Egypt	1999	103.6 (88.0 to 121.4)	34.7 (26.8 to 44.0)	65.4 (52.1 to 80.7)	2.1 (1.0 to 4.0)	1.4 (1.4 to 1.4)	479.6 (407.4 to 561.9)	160.8 (124.2 to 203.5)	302.4 (241.2 to 373.5)	9.9 (4.6 to 18.7)	6.5 (6.5 to 6.5)
Egypt	2000	106.8 (91.4 to 124.7)	36.0 (28.1 to 45.3)	66.9 (53.4 to 82.1)	2.3 (1.0 to 4.1)	1.5 (1.5 to 1.5)	494.4 (422.9 to 577.1)	166.8 (129.8 to 209.8)	309.8 (246.9 to 379.9)	10.6 (4.8 to 19.1)	7.2 (7.2 to 7.2)
Egypt	2001	110.7 (94.4 to 128.5)	37.2 (28.9 to 46.5)	69.9 (55.6 to 85.7)	2.3 (1.0 to 4.3)	1.3 (1.3 to 1.3)	512.5 (436.9 to 594.4)	172.3 (133.5 to 215.3)	323.6 (257.5 to 396.7)	10.8 (4.8 to 20.0)	5.9 (5.9 to 5.9)

Country	Year	2018 US Dollars per capita					2018 PPP per capita				
		Health spending	Government health spending	Out-of-pocket health spending	Prepaid private health spending	Development assistance for health	Health spending	Government health spending	Out-of-pocket health spending	Prepaid private health spending	Development assistance for health
Egypt	2002	111.7 (94.8 to 129.3)	37.4 (28.8 to 47.0)	71.1 (56.9 to 87.4)	2.3 (1.0 to 4.1)	1.0 (1.0 to 1.0)	517.0 (438.8 to 598.5)	172.9 (133.3 to 217.5)	328.8 (263.5 to 404.3)	10.5 (4.8 to 19.0)	4.8 (4.8 to 4.8)
Egypt	2003	109.6 (94.2 to 127.4)	36.4 (28.0 to 45.3)	70.2 (56.1 to 86.8)	2.2 (1.0 to 4.0)	0.8 (0.8 to 0.8)	507.1 (435.7 to 589.7)	168.3 (129.6 to 209.6)	324.9 (259.4 to 401.4)	10.3 (4.7 to 18.7)	3.7 (3.7 to 3.7)
Egypt	2004	108.1 (91.8 to 126.5)	35.9 (27.8 to 44.4)	68.7 (55.1 to 85.1)	2.2 (1.0 to 4.0)	1.3 (1.3 to 1.3)	500.1 (424.9 to 585.2)	166.1 (128.8 to 205.4)	317.9 (254.8 to 393.7)	10.2 (4.7 to 18.7)	5.9 (5.9 to 5.9)
Egypt	2005	108.1 (92.1 to 127.7)	36.1 (28.1 to 45.2)	68.1 (54.6 to 85.6)	2.3 (1.0 to 4.3)	1.5 (1.5 to 1.5)	500.1 (426.2 to 590.8)	167.2 (129.9 to 209.2)	315.2 (252.6 to 396.2)	10.7 (4.8 to 19.9)	7.0 (7.0 to 7.0)
Egypt	2006	110.2 (93.8 to 128.8)	37.7 (29.4 to 47.3)	67.8 (53.7 to 84.2)	2.5 (1.1 to 4.7)	2.1 (2.1 to 2.1)	510.1 (434.2 to 595.8)	174.7 (136.0 to 219.1)	314.0 (248.6 to 389.5)	11.7 (5.0 to 21.8)	9.8 (9.8 to 9.8)
Egypt	2007	109.9 (93.6 to 127.3)	38.2 (30.1 to 48.1)	67.5 (53.2 to 83.3)	2.8 (1.2 to 5.2)	1.4 (1.4 to 1.4)	508.6 (433.3 to 589.2)	176.9 (139.3 to 222.7)	312.2 (246.2 to 385.3)	12.9 (5.6 to 23.9)	6.6 (6.6 to 6.6)
Egypt	2008	112.9 (96.6 to 130.7)	38.8 (30.5 to 49.0)	68.8 (54.7 to 84.0)	3.2 (1.4 to 6.1)	2.1 (2.1 to 2.1)	522.2 (446.8 to 604.7)	179.7 (141.3 to 226.9)	318.1 (252.9 to 388.8)	14.8 (6.6 to 28.2)	9.6 (9.6 to 9.6)
Egypt	2009	112.3 (95.3 to 130.4)	38.7 (30.3 to 48.5)	69.0 (54.3 to 84.7)	3.8 (1.7 to 7.2)	0.7 (0.7 to 0.7)	519.5 (441.1 to 603.2)	179.2 (140.3 to 224.2)	319.5 (251.4 to 391.9)	17.5 (7.9 to 33.3)	3.3 (3.3 to 3.3)
Egypt	2010	114.0 (96.9 to 133.3)	38.5 (30.1 to 48.0)	69.9 (54.7 to 86.1)	4.7 (2.1 to 8.9)	1.0 (1.0 to 1.0)	527.6 (448.5 to 616.9)	178.1 (139.4 to 222.0)	323.4 (253.3 to 398.2)	21.7 (9.8 to 41.0)	4.4 (4.4 to 4.4)
Egypt	2011	116.2 (98.8 to 135.5)	38.6 (29.9 to 48.3)	71.0 (55.7 to 87.1)	5.9 (2.8 to 11.3)	0.7 (0.7 to 0.7)	537.9 (457.2 to 627.0)	178.5 (138.5 to 223.7)	328.5 (257.8 to 402.9)	27.5 (12.9 to 52.1)	3.4 (3.4 to 3.4)
Egypt	2012	116.4 (98.0 to 136.0)	37.3 (28.4 to 46.7)	71.5 (56.5 to 88.4)	7.2 (3.4 to 13.9)	0.4 (0.4 to 0.4)	538.8 (453.7 to 629.1)	172.8 (131.5 to 216.2)	330.9 (261.3 to 408.9)	33.3 (15.5 to 64.3)	1.8 (1.8 to 1.8)
Egypt	2013	118.6 (99.5 to 139.0)	37.8 (28.8 to 47.0)	72.1 (56.8 to 90.4)	8.3 (4.0 to 15.4)	0.4 (0.4 to 0.4)	548.7 (460.4 to 643.0)	174.7 (133.3 to 217.4)	333.6 (262.7 to 418.4)	38.6 (18.3 to 71.4)	1.9 (1.9 to 1.9)
Egypt	2014	120.6 (101.5 to 143.1)	38.5 (29.7 to 48.1)	72.5 (56.6 to 91.7)	9.1 (4.4 to 17.1)	0.5 (0.5 to 0.5)	558.2 (469.5 to 662.2)	178.4 (137.3 to 222.4)	335.3 (262.1 to 424.4)	42.1 (20.4 to 79.0)	2.4 (2.4 to 2.4)
Egypt	2015	124.2 (103.6 to 147.5)	39.9 (31.2 to 49.7)	74.2 (57.0 to 94.7)	9.6 (4.7 to 18.0)	0.5 (0.5 to 0.5)	575.0 (479.4 to 682.7)	184.5 (144.4 to 229.9)	343.6 (264.0 to 438.2)	44.5 (21.6 to 83.2)	2.4 (2.4 to 2.4)
Egypt	2016	124.6 (103.0 to 150.3)	39.1 (30.5 to 49.1)	75.2 (57.4 to 97.0)	9.7 (4.7 to 17.9)	0.6 (0.6 to 0.6)	576.6 (476.9 to 695.5)	180.9 (141.0 to 227.3)	348.0 (265.7 to 448.8)	44.9 (21.9 to 82.6)	2.8 (2.8 to 2.8)
Egypt	2017	126.4 (105.4 to 151.1)	39.5 (30.8 to 49.6)	76.0 (58.2 to 98.3)	9.9 (4.8 to 18.2)	0.9 (0.9 to 0.9)	584.8 (487.9 to 699.0)	182.9 (142.6 to 229.4)	351.8 (269.3 to 454.9)	45.8 (22.3 to 84.4)	4.2 (4.2 to 4.2)
Egypt	2018	128.6 (105.8 to 154.3)	40.3 (31.3 to 50.7)	77.3 (59.1 to 99.9)	10.1 (4.9 to 18.7)	0.9 (0.9 to 0.9)	595.2 (489.4 to 713.9)	186.6 (144.9 to 234.8)	357.7 (273.3 to 462.2)	47.0 (22.8 to 86.4)	4.0 (4.0 to 4.0)
Egypt	2019	131.0 (108.5 to 156.6)	41.1 (32.0 to 51.7)	78.6 (60.1 to 102.1)	10.4 (5.1 to 19.2)	0.9 (0.8 to 0.9)	606.4 (502.0 to 724.8)	190.4 (148.1 to 239.3)	363.8 (278.0 to 472.5)	48.2 (23.5 to 88.7)	4.0 (3.7 to 4.2)
Egypt	2020	133.4 (110.0 to 158.2)	42.0 (32.7 to 52.8)	79.9 (61.2 to 103.7)	10.7 (5.2 to 19.6)	0.9 (0.8 to 0.9)	617.2 (509.2 to 732.3)	194.3 (151.2 to 244.5)	369.6 (283.4 to 480.0)	49.3 (24.0 to 90.7)	4.0 (3.7 to 4.3)
Egypt	2021	136.0 (112.7 to 163.2)	43.0 (33.4 to 54.3)	81.2 (62.3 to 105.6)	10.9 (5.3 to 20.0)	0.9 (0.8 to 1.0)	629.3 (521.6 to 755.4)	199.0 (154.7 to 251.2)	375.7 (288.1 to 488.8)	50.5 (24.5 to 92.7)	4.1 (3.7 to 4.5)
Egypt	2022	138.0 (114.1 to 164.7)	43.8 (34.1 to 55.2)	82.2 (62.9 to 106.9)	11.1 (5.4 to 20.4)	0.9 (0.8 to 1.0)	638.5 (527.9 to 762.0)	202.6 (158.0 to 255.3)	380.3 (291.1 to 494.6)	51.4 (25.0 to 94.4)	4.1 (3.7 to 4.6)

		2018 US Dollars per capita					2018 PPP per capita				
Country	Year	Health spending	Government health spending	Out-of-pocket health spending	Prepaid private health spending	Development assistance for health	Health spending	Government health spending	Out-of-pocket health spending	Prepaid private health spending	Development assistance for health
Egypt	2023	139.4 (115.6 to 167.5)	44.4 (34.6 to 55.9)	82.8 (63.5 to 108.2)	11.3 (5.5 to 20.7)	0.9 (0.8 to 1.0)	645.1 (535.1 to 775.1)	205.5 (160.2 to 258.8)	383.2 (293.8 to 500.7)	52.2 (25.3 to 95.7)	4.2 (3.7 to 4.8)
Egypt	2024	140.7 (117.2 to 167.6)	45.0 (35.0 to 56.7)	83.3 (64.0 to 108.9)	11.4 (5.5 to 20.9)	0.9 (0.8 to 1.1)	651.0 (542.1 to 775.5)	208.3 (162.1 to 262.5)	385.5 (296.1 to 503.7)	52.9 (25.6 to 96.9)	4.3 (3.7 to 5.0)
Egypt	2025	142.2 (118.6 to 169.9)	45.7 (35.6 to 57.5)	83.9 (64.5 to 109.3)	11.6 (5.6 to 21.2)	1.0 (0.8 to 1.1)	658.0 (548.9 to 786.1)	211.4 (164.9 to 266.3)	388.4 (298.4 to 505.7)	53.7 (26.0 to 98.3)	4.4 (3.8 to 5.2)
Egypt	2026	143.9 (119.9 to 172.0)	46.5 (36.1 to 58.6)	84.6 (65.0 to 110.3)	11.8 (5.7 to 21.6)	1.0 (0.8 to 1.1)	665.8 (554.8 to 795.7)	215.0 (167.3 to 271.0)	391.7 (300.9 to 510.5)	54.6 (26.4 to 99.8)	4.5 (3.8 to 5.3)
Egypt	2027	145.8 (121.5 to 174.7)	47.3 (36.8 to 59.6)	85.5 (65.5 to 111.2)	12.0 (5.8 to 21.9)	1.0 (0.8 to 1.2)	674.7 (562.0 to 808.6)	219.0 (170.2 to 275.7)	395.6 (303.3 to 514.8)	55.5 (26.8 to 101.4)	4.6 (3.9 to 5.5)
Egypt	2028	147.9 (122.5 to 176.0)	48.3 (37.5 to 60.8)	86.4 (66.1 to 112.3)	12.2 (5.9 to 22.3)	1.0 (0.9 to 1.2)	684.5 (567.1 to 814.3)	223.3 (173.7 to 281.1)	400.0 (306.1 to 519.7)	56.6 (27.3 to 103.2)	4.7 (3.9 to 5.7)
Egypt	2029	150.3 (125.4 to 179.1)	49.3 (38.0 to 62.2)	87.5 (66.9 to 113.5)	12.5 (6.0 to 22.8)	1.0 (0.9 to 1.3)	695.7 (580.5 to 828.6)	228.1 (175.8 to 288.0)	405.0 (309.7 to 525.3)	57.7 (27.9 to 105.3)	4.8 (4.0 to 5.9)
Egypt	2030	153.0 (126.7 to 183.8)	50.4 (38.9 to 63.4)	88.7 (67.8 to 115.0)	12.8 (6.2 to 23.2)	1.1 (0.9 to 1.3)	707.8 (586.2 to 850.4)	233.2 (179.9 to 293.4)	410.7 (314.0 to 532.3)	59.0 (28.5 to 107.5)	4.9 (4.0 to 6.0)
Egypt	2031	155.8 (129.2 to 185.3)	51.6 (39.8 to 65.1)	90.0 (68.9 to 116.6)	13.0 (6.3 to 23.7)	1.1 (0.9 to 1.4)	720.8 (597.7 to 857.3)	238.8 (184.3 to 301.3)	416.6 (318.8 to 539.8)	60.4 (29.2 to 109.9)	5.0 (4.1 to 6.3)
Egypt	2032	158.8 (131.5 to 190.9)	52.9 (40.6 to 66.8)	91.5 (69.9 to 118.3)	13.4 (6.5 to 24.3)	1.1 (0.9 to 1.4)	735.0 (608.3 to 883.3)	244.7 (188.1 to 309.0)	423.3 (323.6 to 547.6)	61.8 (30.0 to 112.7)	5.1 (4.1 to 6.4)
Egypt	2033	162.1 (135.2 to 194.3)	54.3 (41.7 to 68.5)	93.0 (71.2 to 120.4)	13.7 (6.7 to 25.0)	1.1 (0.9 to 1.4)	750.3 (625.5 to 899.3)	251.1 (193.0 to 316.9)	430.5 (329.4 to 557.3)	63.4 (30.8 to 115.7)	5.2 (4.1 to 6.7)
Egypt	2034	165.5 (136.3 to 198.8)	55.7 (43.0 to 70.4)	94.6 (72.3 to 122.6)	14.1 (6.8 to 25.7)	1.2 (0.9 to 1.5)	765.8 (630.9 to 920.1)	257.6 (199.1 to 325.8)	437.8 (334.4 to 567.5)	65.0 (31.6 to 118.8)	5.3 (4.2 to 6.9)
Egypt	2035	168.8 (138.9 to 202.1)	57.1 (44.0 to 71.9)	96.1 (73.3 to 124.7)	14.4 (7.0 to 26.3)	1.2 (0.9 to 1.5)	780.9 (642.6 to 935.3)	264.0 (203.7 to 332.7)	444.8 (339.2 to 577.0)	66.7 (32.3 to 121.9)	5.5 (4.2 to 7.1)
Egypt	2036	172.0 (141.4 to 208.4)	58.4 (45.1 to 73.7)	97.6 (74.2 to 126.6)	14.7 (7.1 to 27.0)	1.2 (0.9 to 1.6)	795.9 (654.2 to 964.2)	270.4 (208.8 to 341.3)	451.6 (343.3 to 586.0)	68.3 (33.0 to 124.9)	5.6 (4.3 to 7.5)
Egypt	2037	175.2 (144.9 to 209.8)	59.8 (46.1 to 75.6)	99.1 (75.2 to 128.7)	15.1 (7.3 to 27.7)	1.2 (0.9 to 1.7)	810.9 (670.7 to 970.7)	276.9 (213.3 to 349.9)	458.5 (348.1 to 595.3)	69.9 (33.8 to 128.0)	5.7 (4.4 to 7.7)
Egypt	2038	178.5 (147.4 to 215.3)	61.2 (47.0 to 77.5)	100.5 (76.2 to 130.7)	15.4 (7.5 to 28.3)	1.3 (1.0 to 1.8)	825.9 (681.9 to 996.1)	283.4 (217.5 to 358.8)	465.2 (352.6 to 604.9)	71.5 (34.7 to 131.1)	5.8 (4.5 to 8.1)
Egypt	2039	181.8 (149.9 to 217.6)	62.7 (48.1 to 79.7)	102.0 (77.0 to 132.8)	15.8 (7.7 to 29.0)	1.3 (1.0 to 1.8)	841.2 (693.8 to 1007.1)	290.1 (222.5 to 368.8)	472.0 (356.5 to 614.3)	73.1 (35.6 to 134.3)	6.0 (4.6 to 8.3)
Egypt	2040	185.1 (151.8 to 221.9)	64.2 (49.4 to 81.8)	103.4 (78.0 to 134.7)	16.2 (7.9 to 29.7)	1.3 (1.0 to 1.8)	856.4 (702.4 to 1026.8)	297.0 (228.4 to 378.6)	478.6 (360.7 to 623.5)	74.8 (36.4 to 137.4)	6.1 (4.6 to 8.5)
Egypt	2041	188.4 (155.4 to 226.7)	65.7 (50.9 to 83.6)	104.8 (78.9 to 136.7)	16.5 (8.1 to 30.4)	1.4 (1.0 to 1.9)	871.7 (719.1 to 1049.1)	304.0 (235.7 to 386.8)	485.1 (365.1 to 632.5)	76.4 (37.3 to 140.5)	6.3 (4.7 to 8.7)
Egypt	2042	191.7 (157.2 to 229.9)	67.2 (52.1 to 85.7)	106.2 (79.8 to 138.9)	16.9 (8.3 to 31.0)	1.4 (1.0 to 2.0)	887.0 (727.5 to 1064.1)	311.1 (240.9 to 396.7)	491.5 (369.4 to 642.9)	78.0 (38.2 to 143.7)	6.4 (4.8 to 9.2)
Egypt	2043	195.0 (162.6 to 234.3)	68.8 (53.1 to 88.0)	107.6 (80.8 to 140.8)	17.2 (8.4 to 31.7)	1.4 (1.1 to 2.1)	902.5 (752.3 to 1084.4)	318.4 (245.8 to 407.3)	497.8 (373.7 to 651.6)	79.6 (39.1 to 146.8)	6.6 (4.9 to 9.5)

		2018 US Dollars per capita					2018 PPP per capita				
Country	Year	Health spending	Government health spending	Out-of-pocket health spending	Prepaid private health spending	Development assistance for health	Health spending	Government health spending	Out-of-pocket health spending	Prepaid private health spending	Development assistance for health
Egypt	2044	198.4 (163.4 to 237.1)	70.4 (54.2 to 90.2)	108.9 (81.6 to 142.6)	17.5 (8.6 to 32.4)	1.5 (1.1 to 2.1)	917.9 (756.2 to 1097.3)	325.9 (250.9 to 417.3)	504.0 (377.8 to 659.7)	81.2 (39.9 to 150.0)	6.8 (5.0 to 9.8)
Egypt	2045	201.6 (167.0 to 243.6)	72.0 (55.1 to 92.4)	110.2 (82.6 to 144.5)	17.9 (8.8 to 33.1)	1.5 (1.1 to 2.3)	933.0 (773.0 to 1127.4)	333.2 (255.2 to 427.5)	510.0 (382.4 to 668.9)	82.8 (40.8 to 153.4)	7.0 (5.0 to 10.5)
Egypt	2046	205.0 (168.8 to 245.8)	73.7 (56.4 to 94.7)	111.5 (83.7 to 146.1)	18.2 (9.0 to 33.8)	1.5 (1.1 to 2.4)	948.5 (781.0 to 1137.6)	340.9 (261.2 to 438.2)	516.1 (387.3 to 676.3)	84.4 (41.7 to 156.6)	7.2 (5.2 to 11.0)
Egypt	2047	208.2 (170.8 to 249.7)	75.3 (57.4 to 96.5)	112.8 (84.7 to 147.8)	18.6 (9.2 to 34.4)	1.6 (1.1 to 2.5)	963.4 (790.4 to 1155.6)	348.3 (265.7 to 446.7)	521.8 (392.1 to 684.1)	86.0 (42.4 to 159.3)	7.4 (5.3 to 11.6)
Egypt	2048	211.5 (176.7 to 253.6)	76.9 (58.4 to 99.2)	114.0 (85.8 to 149.5)	18.9 (9.3 to 35.1)	1.6 (1.2 to 2.6)	978.9 (817.8 to 1173.6)	356.1 (270.5 to 459.1)	527.6 (397.2 to 691.6)	87.6 (43.2 to 162.4)	7.6 (5.4 to 12.2)
Egypt	2049	214.8 (176.8 to 262.3)	78.6 (59.7 to 101.6)	115.2 (86.9 to 151.1)	19.3 (9.5 to 35.8)	1.7 (1.2 to 2.7)	993.8 (818.0 to 1213.7)	363.7 (276.5 to 470.0)	533.2 (402.0 to 699.2)	89.1 (44.0 to 165.5)	7.8 (5.6 to 12.6)
Egypt	2050	218.2 (180.6 to 262.8)	80.3 (61.0 to 104.1)	116.5 (87.7 to 152.8)	19.6 (9.7 to 36.5)	1.7 (1.2 to 2.8)	1009.5 (835.6 to 1216.2)	371.7 (282.2 to 481.6)	539.0 (405.7 to 707.3)	90.8 (44.7 to 168.7)	8.1 (5.7 to 13.1)
El Salvador	1995	214.5 (189.4 to 243.5)	72.9 (60.7 to 85.7)	129.7 (107.1 to 157.6)	3.9 (1.9 to 7.3)	7.9 (7.9 to 7.9)	449.3 (396.7 to 510.2)	152.8 (127.1 to 179.6)	271.8 (224.3 to 330.2)	8.1 (4.0 to 15.4)	16.6 (16.6 to 16.6)
El Salvador	1996	236.8 (212.1 to 265.8)	82.7 (70.7 to 96.4)	139.8 (117.3 to 166.4)	5.2 (2.6 to 9.7)	9.1 (9.1 to 9.1)	496.0 (444.4 to 556.7)	173.2 (148.0 to 201.9)	292.9 (245.7 to 348.6)	10.9 (5.5 to 20.4)	19.1 (19.1 to 19.1)
El Salvador	1997	254.6 (229.7 to 282.8)	94.5 (81.7 to 109.2)	149.3 (126.6 to 174.8)	6.4 (3.3 to 11.5)	4.5 (4.5 to 4.5)	533.4 (481.1 to 592.5)	198.0 (171.2 to 228.8)	312.7 (265.2 to 366.1)	13.3 (6.9 to 24.2)	9.4 (9.4 to 9.4)
El Salvador	1998	271.7 (245.8 to 299.0)	106.7 (93.2 to 121.9)	151.5 (129.5 to 175.0)	7.5 (3.9 to 13.2)	6.0 (6.0 to 6.0)	569.2 (514.8 to 626.3)	223.5 (195.3 to 255.5)	317.4 (271.3 to 366.6)	15.7 (8.2 to 27.6)	12.6 (12.6 to 12.6)
El Salvador	1999	278.4 (252.6 to 306.0)	114.7 (99.9 to 130.7)	149.4 (128.0 to 172.8)	8.9 (4.7 to 15.7)	5.3 (5.3 to 5.3)	583.2 (529.2 to 640.9)	240.3 (209.3 to 273.8)	313.0 (268.1 to 362.0)	18.7 (9.8 to 32.9)	11.2 (11.2 to 11.2)
El Salvador	2000	279.1 (254.2 to 305.6)	119.9 (105.5 to 135.4)	145.1 (124.6 to 167.9)	9.4 (4.9 to 16.2)	4.7 (4.7 to 4.7)	584.7 (532.4 to 640.2)	251.2 (221.1 to 283.7)	304.0 (261.0 to 351.6)	19.6 (10.4 to 33.9)	9.9 (9.9 to 9.9)
El Salvador	2001	280.5 (255.6 to 307.1)	122.6 (108.5 to 138.7)	140.2 (121.2 to 163.1)	10.0 (5.4 to 17.3)	7.7 (7.7 to 7.7)	587.6 (535.4 to 643.4)	256.9 (227.2 to 290.6)	293.7 (253.9 to 341.6)	20.9 (11.2 to 36.2)	16.1 (16.1 to 16.1)
El Salvador	2002	276.4 (251.6 to 302.2)	126.2 (111.9 to 142.6)	135.2 (116.6 to 157.0)	10.2 (5.4 to 17.8)	4.9 (4.9 to 4.9)	579.1 (527.1 to 633.1)	264.3 (234.5 to 298.6)	283.2 (244.2 to 328.9)	21.4 (11.4 to 37.3)	10.2 (10.2 to 10.2)
El Salvador	2003	271.2 (247.5 to 295.4)	128.2 (113.8 to 144.2)	128.4 (110.4 to 148.9)	10.3 (5.4 to 17.6)	4.2 (4.2 to 4.2)	568.1 (518.4 to 618.9)	268.6 (238.5 to 302.1)	269.0 (231.2 to 311.9)	21.6 (11.3 to 36.9)	8.9 (8.9 to 8.9)
El Salvador	2004	267.6 (244.1 to 290.7)	131.2 (116.1 to 147.7)	120.5 (102.7 to 139.6)	10.6 (5.7 to 18.1)	5.2 (5.2 to 5.2)	560.6 (511.4 to 609.0)	274.9 (243.1 to 309.4)	252.5 (215.1 to 292.4)	22.3 (11.8 to 38.0)	11.0 (11.0 to 11.0)
El Salvador	2005	267.1 (242.3 to 291.3)	137.3 (121.9 to 154.8)	111.9 (95.1 to 129.8)	11.2 (5.9 to 19.2)	6.6 (6.6 to 6.6)	559.5 (507.6 to 610.3)	287.7 (255.4 to 324.3)	234.4 (199.1 to 271.9)	23.5 (12.3 to 40.2)	13.9 (13.9 to 13.9)
El Salvador	2006	262.6 (238.4 to 288.0)	143.8 (127.8 to 161.3)	101.2 (85.9 to 118.3)	11.8 (6.0 to 20.5)	5.8 (5.8 to 5.8)	550.1 (499.3 to 603.2)	301.2 (267.7 to 338.0)	212.0 (180.0 to 247.7)	24.7 (12.7 to 42.9)	12.1 (12.1 to 12.1)
El Salvador	2007	261.4 (237.4 to 286.0)	146.2 (129.4 to 163.6)	97.3 (82.5 to 114.1)	12.2 (6.2 to 21.1)	5.8 (5.8 to 5.8)	547.7 (497.3 to 599.1)	306.2 (271.0 to 342.7)	203.9 (172.8 to 239.1)	25.5 (13.0 to 44.2)	12.1 (12.1 to 12.1)
El Salvador	2008	261.4 (237.8 to 288.4)	148.2 (131.4 to 165.6)	95.0 (80.1 to 111.8)	12.5 (6.5 to 21.6)	5.7 (5.7 to 5.7)	547.7 (498.1 to 604.2)	310.4 (275.2 to 346.8)	199.1 (167.9 to 234.1)	26.2 (13.6 to 45.3)	12.0 (12.0 to 12.0)

		2018 US Dollars per capita					2018 PPP per capita				
Country	Year	Health spending	Government health spending	Out-of-pocket health spending	Prepaid private health spending	Development assistance for health	Health spending	Government health spending	Out-of-pocket health spending	Prepaid private health spending	Development assistance for health
El Salvador	2009	260.1 (236.4 to 287.1)	148.1 (132.1 to 166.7)	93.0 (78.9 to 109.1)	12.7 (6.6 to 22.6)	6.3 (6.3 to 6.3)	545.0 (495.2 to 601.4)	310.2 (276.8 to 349.2)	194.9 (165.3 to 228.6)	26.6 (13.8 to 47.3)	13.2 (13.2 to 13.2)
El Salvador	2010	274.8 (252.0 to 302.1)	157.0 (140.7 to 176.3)	92.3 (78.8 to 109.4)	13.1 (6.9 to 22.8)	12.4 (12.4 to 12.4)	575.7 (527.8 to 632.9)	328.9 (294.7 to 369.4)	193.4 (165.1 to 229.1)	27.4 (14.4 to 47.8)	26.0 (26.0 to 26.0)
El Salvador	2011	281.1 (256.5 to 307.8)	169.6 (152.1 to 190.2)	91.5 (77.9 to 108.3)	13.8 (7.3 to 24.0)	6.1 (6.1 to 6.1)	588.9 (537.3 to 644.8)	355.3 (318.6 to 398.4)	191.7 (163.3 to 226.8)	29.0 (15.2 to 50.3)	12.9 (12.9 to 12.9)
El Salvador	2012	283.3 (258.5 to 309.9)	174.1 (155.8 to 192.8)	89.2 (75.7 to 105.5)	14.4 (7.5 to 24.4)	5.5 (5.5 to 5.5)	593.4 (541.6 to 649.1)	364.8 (326.5 to 404.0)	186.8 (158.5 to 221.1)	30.2 (15.7 to 51.0)	11.6 (11.6 to 11.6)
El Salvador	2013	290.1 (264.8 to 316.9)	180.9 (160.8 to 200.6)	86.5 (73.0 to 102.5)	15.3 (7.8 to 26.0)	7.3 (7.3 to 7.3)	607.7 (554.7 to 663.8)	379.0 (336.8 to 420.3)	181.2 (153.0 to 214.8)	32.1 (16.4 to 54.5)	15.4 (15.4 to 15.4)
El Salvador	2014	292.0 (265.0 to 320.9)	186.2 (165.4 to 207.7)	85.4 (71.6 to 102.3)	16.4 (8.4 to 27.6)	4.0 (4.0 to 4.0)	611.7 (555.1 to 672.2)	390.2 (346.5 to 435.1)	178.9 (150.0 to 214.2)	34.4 (17.6 to 57.8)	8.3 (8.3 to 8.3)
El Salvador	2015	302.6 (272.5 to 334.4)	194.0 (169.5 to 219.2)	85.5 (71.0 to 103.2)	17.9 (9.3 to 31.8)	5.2 (5.2 to 5.2)	633.8 (570.8 to 700.4)	406.3 (355.1 to 459.3)	179.1 (148.8 to 216.3)	37.4 (19.4 to 66.6)	10.9 (10.9 to 10.9)
El Salvador	2016	313.2 (279.3 to 349.4)	201.6 (173.3 to 231.5)	86.4 (70.5 to 106.5)	19.3 (10.0 to 35.2)	5.9 (5.9 to 5.9)	656.1 (585.0 to 732.0)	422.4 (363.1 to 484.9)	180.9 (147.7 to 223.2)	40.4 (20.9 to 73.7)	12.4 (12.4 to 12.4)
El Salvador	2017	321.0 (285.9 to 359.5)	207.0 (178.7 to 237.9)	86.5 (70.3 to 106.7)	19.7 (10.2 to 35.9)	7.8 (7.8 to 7.8)	672.3 (598.9 to 753.2)	433.7 (374.3 to 498.4)	181.1 (147.4 to 223.5)	41.2 (21.4 to 75.2)	16.4 (16.4 to 16.4)
El Salvador	2018	326.2 (290.5 to 363.8)	212.2 (183.1 to 244.1)	86.4 (70.3 to 106.4)	20.1 (10.5 to 36.7)	7.4 (7.4 to 7.4)	683.3 (608.5 to 762.1)	444.5 (383.5 to 511.4)	181.1 (147.2 to 222.9)	42.2 (21.9 to 76.9)	15.5 (15.5 to 15.5)
El Salvador	2019	331.7 (296.4 to 375.0)	217.3 (187.5 to 249.9)	86.4 (70.2 to 106.4)	20.6 (10.7 to 37.4)	7.5 (7.0 to 7.9)	694.9 (620.8 to 785.5)	455.1 (392.7 to 523.4)	180.9 (147.2 to 222.8)	43.1 (22.4 to 78.4)	15.7 (14.7 to 16.6)
El Salvador	2020	336.4 (300.7 to 375.7)	221.5 (191.3 to 254.9)	86.2 (70.0 to 106.9)	21.0 (10.9 to 38.1)	7.7 (7.0 to 8.3)	704.8 (629.9 to 787.1)	464.0 (400.8 to 534.1)	180.6 (146.6 to 223.9)	44.0 (22.9 to 79.8)	16.1 (14.8 to 17.4)
El Salvador	2021	341.2 (306.1 to 382.3)	225.8 (195.5 to 259.5)	86.1 (69.7 to 106.8)	21.4 (11.1 to 38.8)	7.9 (7.1 to 8.7)	714.7 (641.3 to 800.8)	473.0 (409.4 to 543.6)	180.4 (146.1 to 223.8)	44.9 (23.3 to 81.2)	16.5 (14.8 to 18.2)
El Salvador	2022	346.2 (309.5 to 384.3)	230.2 (199.5 to 264.7)	86.0 (69.4 to 106.8)	21.9 (11.4 to 39.5)	8.1 (7.2 to 9.0)	725.2 (648.3 to 805.1)	482.2 (417.9 to 554.6)	180.2 (145.4 to 223.6)	45.8 (23.8 to 82.7)	17.0 (15.1 to 19.0)
El Salvador	2023	351.6 (313.0 to 392.8)	235.1 (203.3 to 270.7)	85.9 (69.0 to 106.6)	22.3 (11.6 to 40.3)	8.3 (7.3 to 9.5)	736.7 (655.7 to 822.8)	492.6 (425.9 to 567.1)	179.9 (144.6 to 223.4)	46.7 (24.2 to 84.3)	17.5 (15.2 to 19.8)
El Salvador	2024	357.1 (319.4 to 398.6)	240.0 (207.6 to 276.8)	85.7 (68.7 to 106.4)	22.7 (11.8 to 41.2)	8.6 (7.4 to 9.9)	748.0 (669.1 to 835.1)	502.8 (434.9 to 579.9)	179.6 (143.9 to 222.9)	47.6 (24.7 to 86.2)	18.0 (15.5 to 20.7)
El Salvador	2025	362.5 (324.4 to 402.7)	244.9 (212.1 to 282.8)	85.6 (68.5 to 106.8)	23.2 (12.0 to 42.1)	8.8 (7.6 to 10.4)	759.4 (679.6 to 843.5)	513.1 (444.4 to 592.4)	179.3 (143.5 to 223.6)	48.5 (25.1 to 88.1)	18.5 (15.8 to 21.7)
El Salvador	2026	368.2 (329.2 to 411.8)	250.0 (216.5 to 288.6)	85.4 (68.4 to 106.7)	23.6 (12.2 to 43.0)	9.1 (7.7 to 10.6)	771.2 (689.7 to 862.7)	523.8 (453.6 to 604.5)	179.0 (143.4 to 223.5)	49.4 (25.6 to 90.1)	19.0 (16.1 to 22.3)
El Salvador	2027	373.8 (334.4 to 421.4)	255.1 (220.7 to 294.9)	85.3 (68.6 to 107.0)	24.0 (12.4 to 43.9)	9.4 (7.8 to 11.1)	783.0 (700.5 to 882.7)	534.4 (462.3 to 617.7)	178.7 (143.8 to 224.2)	50.3 (26.0 to 92.0)	19.6 (16.4 to 23.3)
El Salvador	2028	379.4 (339.3 to 426.4)	260.1 (224.9 to 300.6)	85.2 (68.2 to 107.3)	24.5 (12.7 to 44.9)	9.6 (8.1 to 11.6)	794.8 (710.7 to 893.3)	545.0 (471.1 to 629.7)	178.5 (142.8 to 224.8)	51.2 (26.5 to 94.0)	20.2 (16.9 to 24.4)
El Salvador	2029	385.2 (342.8 to 431.8)	265.2 (229.1 to 306.3)	85.1 (68.0 to 107.5)	24.9 (12.9 to 45.8)	9.9 (8.2 to 12.2)	806.8 (718.0 to 904.5)	555.5 (480.0 to 641.6)	178.4 (142.3 to 225.3)	52.1 (27.0 to 95.9)	20.8 (17.2 to 25.5)

Country	Year	2018 US Dollars per capita					2018 PPP per capita				
		Health spending	Government health spending	Out-of-pocket health spending	Prepaid private health spending	Development assistance for health	Health spending	Government health spending	Out-of-pocket health spending	Prepaid private health spending	Development assistance for health
El Salvador	2030	391.0 (348.8 to 437.6)	270.3 (233.8 to 311.9)	85.2 (67.7 to 108.3)	25.3 (13.1 to 46.5)	10.2 (8.4 to 12.5)	819.1 (730.6 to 916.6)	566.2 (489.8 to 653.3)	178.4 (141.9 to 226.8)	53.0 (27.4 to 97.3)	21.4 (17.5 to 26.3)
El Salvador	2031	397.3 (353.4 to 448.7)	275.6 (238.5 to 318.0)	85.3 (67.6 to 109.0)	25.8 (13.3 to 47.1)	10.6 (8.6 to 13.2)	832.2 (740.4 to 940.0)	577.4 (499.7 to 666.2)	178.7 (141.5 to 228.3)	54.0 (27.9 to 98.7)	22.1 (17.9 to 27.7)
El Salvador	2032	403.6 (359.1 to 451.2)	281.0 (243.2 to 324.2)	85.5 (67.3 to 109.7)	26.2 (13.6 to 47.7)	10.9 (8.7 to 13.7)	845.5 (752.4 to 945.3)	588.7 (509.4 to 679.1)	179.1 (140.9 to 229.7)	54.9 (28.4 to 100.0)	22.8 (18.3 to 28.7)
El Salvador	2033	410.3 (362.8 to 462.4)	286.6 (247.3 to 330.3)	85.8 (67.2 to 110.3)	26.7 (13.8 to 48.4)	11.2 (8.9 to 14.4)	859.4 (760.0 to 968.6)	600.3 (518.0 to 692.0)	179.7 (140.8 to 231.1)	55.9 (28.9 to 101.4)	23.5 (18.7 to 30.2)
El Salvador	2034	417.0 (372.7 to 467.8)	292.2 (252.1 to 336.8)	86.1 (67.2 to 111.1)	27.1 (14.0 to 49.0)	11.6 (9.1 to 15.0)	873.6 (780.8 to 980.0)	612.0 (528.1 to 705.5)	180.4 (140.7 to 232.8)	56.9 (29.4 to 102.7)	24.3 (19.1 to 31.4)
El Salvador	2035	424.1 (375.2 to 479.5)	297.9 (256.6 to 343.2)	86.6 (67.1 to 112.0)	27.6 (14.3 to 49.8)	12.0 (9.3 to 15.7)	888.4 (786.0 to 1004.6)	624.1 (537.5 to 718.9)	181.3 (140.6 to 234.6)	57.9 (29.9 to 104.4)	25.1 (19.5 to 32.9)
El Salvador	2036	431.5 (382.3 to 486.3)	303.9 (261.2 to 350.4)	87.1 (67.1 to 113.2)	28.1 (14.5 to 50.8)	12.4 (9.6 to 16.6)	903.9 (800.8 to 1018.8)	636.6 (547.2 to 734.0)	182.4 (140.5 to 237.1)	58.9 (30.4 to 106.4)	26.0 (20.0 to 34.8)
El Salvador	2037	439.2 (388.9 to 498.5)	310.1 (266.5 to 357.6)	87.7 (67.4 to 114.4)	28.6 (14.8 to 51.8)	12.8 (9.8 to 17.1)	920.1 (814.6 to 1044.4)	649.6 (558.2 to 749.1)	183.7 (141.1 to 239.7)	60.0 (31.0 to 108.5)	26.8 (20.6 to 35.9)
El Salvador	2038	447.2 (395.2 to 508.7)	316.4 (271.3 to 364.9)	88.4 (67.7 to 116.0)	29.2 (15.0 to 52.8)	13.2 (10.1 to 18.3)	936.8 (827.8 to 1065.7)	662.9 (568.4 to 764.4)	185.1 (141.8 to 243.0)	61.1 (31.5 to 110.7)	27.7 (21.1 to 38.4)
El Salvador	2039	455.3 (402.7 to 518.8)	322.9 (276.8 to 373.1)	89.1 (68.1 to 117.7)	29.7 (15.3 to 53.7)	13.6 (10.4 to 18.9)	953.8 (843.5 to 1086.9)	676.4 (579.8 to 781.6)	186.7 (142.6 to 246.5)	62.2 (32.0 to 112.6)	28.5 (21.8 to 39.6)
El Salvador	2040	463.6 (406.7 to 533.3)	329.3 (282.0 to 381.1)	90.0 (68.3 to 119.4)	30.2 (15.5 to 54.6)	14.1 (10.7 to 19.5)	971.2 (852.0 to 1117.1)	689.9 (590.8 to 798.4)	188.4 (143.1 to 250.1)	63.3 (32.5 to 114.3)	29.5 (22.4 to 40.8)
El Salvador	2041	472.2 (417.0 to 535.6)	336.0 (288.2 to 389.5)	90.9 (68.7 to 120.9)	30.8 (15.8 to 55.5)	14.5 (11.0 to 20.2)	989.2 (873.6 to 1122.1)	703.9 (603.8 to 815.9)	190.4 (143.9 to 253.3)	64.5 (33.0 to 116.2)	30.5 (22.9 to 42.3)
El Salvador	2042	481.0 (420.7 to 547.0)	342.8 (294.3 to 397.6)	91.9 (69.2 to 122.5)	31.3 (16.0 to 56.4)	15.1 (11.3 to 21.5)	1007.7 (881.4 to 1145.9)	718.1 (616.5 to 832.9)	192.5 (145.0 to 256.5)	65.6 (33.6 to 118.1)	31.5 (23.6 to 44.9)
El Salvador	2043	490.1 (429.6 to 556.2)	349.6 (299.5 to 406.4)	93.0 (69.8 to 124.1)	31.9 (16.3 to 57.5)	15.6 (11.6 to 22.5)	1026.6 (900.1 to 1165.1)	732.4 (627.4 to 851.3)	194.7 (146.3 to 259.9)	66.8 (34.1 to 120.4)	32.6 (24.2 to 47.2)
El Salvador	2044	499.2 (439.5 to 564.7)	356.5 (304.8 to 414.1)	94.1 (70.6 to 125.7)	32.5 (16.6 to 58.5)	16.1 (11.9 to 23.3)	1045.8 (920.7 to 1182.9)	746.9 (638.5 to 867.5)	197.2 (147.9 to 263.4)	68.0 (34.7 to 122.5)	33.8 (24.9 to 48.9)
El Salvador	2045	508.6 (447.0 to 575.7)	363.5 (310.6 to 423.2)	95.4 (71.5 to 127.3)	33.0 (16.9 to 59.3)	16.7 (12.0 to 25.2)	1065.5 (936.3 to 1206.0)	761.5 (650.6 to 886.6)	199.8 (149.8 to 266.7)	69.2 (35.4 to 124.3)	35.0 (25.2 to 52.8)
El Salvador	2046	518.1 (455.4 to 589.2)	370.5 (316.3 to 432.0)	96.7 (72.5 to 129.0)	33.6 (17.2 to 60.2)	17.3 (12.5 to 26.6)	1085.4 (954.0 to 1234.2)	776.2 (662.5 to 905.0)	202.6 (151.9 to 270.2)	70.4 (36.0 to 126.2)	36.2 (26.2 to 55.6)
El Salvador	2047	528.0 (466.0 to 601.7)	377.6 (322.6 to 441.0)	98.1 (73.7 to 130.8)	34.2 (17.5 to 61.2)	17.9 (12.9 to 28.2)	1106.0 (976.3 to 1260.6)	791.1 (675.8 to 923.8)	205.6 (154.4 to 274.0)	71.7 (36.7 to 128.1)	37.5 (27.0 to 59.2)
El Salvador	2048	537.9 (471.7 to 609.1)	384.9 (328.5 to 449.3)	99.6 (74.9 to 132.6)	34.8 (17.8 to 62.1)	18.6 (13.3 to 29.8)	1126.9 (988.2 to 1276.0)	806.3 (688.2 to 941.3)	208.7 (156.9 to 277.7)	73.0 (37.3 to 130.1)	39.0 (27.9 to 62.4)
El Salvador	2049	547.7 (479.3 to 622.3)	391.9 (334.3 to 457.8)	101.0 (76.1 to 134.8)	35.4 (18.1 to 63.0)	19.3 (13.8 to 31.0)	1147.4 (1004.2 to 1303.7)	821.1 (700.3 to 959.1)	211.7 (159.4 to 282.3)	74.2 (38.0 to 131.9)	40.5 (28.9 to 65.0)
El Salvador	2050	557.8 (485.9 to 633.4)	399.2 (339.9 to 467.6)	102.5 (77.2 to 136.8)	36.0 (18.4 to 63.9)	20.1 (14.1 to 32.9)	1168.6 (1017.9 to 1326.9)	836.3 (712.1 to 979.5)	214.7 (161.7 to 286.6)	75.5 (38.5 to 133.8)	42.1 (29.6 to 68.8)

Country	Year	2018 US Dollars per capita					2018 PPP per capita				
		Health spending	Government health spending	Out-of-pocket health spending	Prepaid private health spending	Development assistance for health	Health spending	Government health spending	Out-of-pocket health spending	Prepaid private health spending	Development assistance for health
Equatorial Guinea	1995	96.3 (81.0 to 114.8)	15.6 (12.1 to 19.7)	76.7 (61.5 to 95.1)	1.8 (0.8 to 3.4)	2.2 (2.2 to 2.2)	247.7 (208.3 to 295.3)	40.0 (31.3 to 50.6)	197.5 (158.2 to 244.6)	4.7 (2.1 to 8.8)	5.6 (5.6 to 5.6)
Equatorial Guinea	1996	116.5 (100.9 to 134.5)	17.6 (13.7 to 22.3)	82.5 (67.4 to 100.0)	2.1 (0.9 to 4.0)	14.4 (14.4 to 14.4)	299.8 (259.5 to 346.2)	45.2 (35.3 to 57.3)	212.2 (173.5 to 257.3)	5.3 (2.4 to 10.2)	37.1 (37.1 to 37.1)
Equatorial Guinea	1997	139.4 (121.0 to 159.7)	27.8 (21.9 to 35.0)	104.8 (87.5 to 125.1)	3.4 (1.5 to 6.5)	3.3 (3.3 to 3.3)	358.5 (311.4 to 410.9)	71.6 (56.3 to 90.0)	269.6 (225.2 to 321.9)	8.8 (3.9 to 16.7)	8.6 (8.6 to 8.6)
Equatorial Guinea	1998	137.0 (120.0 to 155.6)	26.7 (21.0 to 33.3)	103.6 (87.3 to 121.8)	3.5 (1.6 to 6.4)	3.2 (3.2 to 3.2)	352.4 (308.6 to 400.3)	68.7 (53.9 to 85.7)	266.6 (224.7 to 313.5)	9.0 (4.0 to 16.5)	8.2 (8.2 to 8.2)
Equatorial Guinea	1999	134.2 (118.6 to 151.5)	25.5 (20.0 to 32.0)	100.0 (85.5 to 117.1)	3.5 (1.6 to 6.5)	5.2 (5.2 to 5.2)	345.4 (305.2 to 389.8)	65.6 (51.5 to 82.3)	257.3 (220.0 to 301.3)	9.1 (4.1 to 16.7)	13.4 (13.4 to 13.4)
Equatorial Guinea	2000	146.7 (130.7 to 164.0)	28.1 (22.0 to 35.4)	104.9 (90.3 to 121.6)	4.1 (1.9 to 7.7)	9.5 (9.5 to 9.5)	377.5 (336.4 to 422.0)	72.4 (56.6 to 91.1)	269.9 (232.2 to 312.7)	10.6 (4.8 to 19.9)	24.6 (24.6 to 24.6)
Equatorial Guinea	2001	172.7 (152.9 to 193.8)	31.5 (24.7 to 39.1)	128.5 (110.3 to 148.6)	4.9 (2.3 to 9.5)	7.8 (7.8 to 7.8)	444.2 (393.5 to 498.7)	80.9 (63.5 to 100.6)	330.5 (283.8 to 382.2)	12.6 (5.9 to 24.5)	20.1 (20.1 to 20.1)
Equatorial Guinea	2002	190.9 (169.5 to 213.9)	32.7 (26.0 to 40.2)	147.3 (127.5 to 169.8)	5.4 (2.5 to 10.3)	5.5 (5.5 to 5.5)	491.3 (436.2 to 550.5)	84.1 (66.8 to 103.4)	379.0 (328.1 to 436.9)	13.9 (6.4 to 26.4)	14.3 (14.3 to 14.3)
Equatorial Guinea	2003	202.6 (180.1 to 227.2)	35.1 (28.2 to 42.9)	154.7 (134.3 to 178.3)	5.9 (2.7 to 11.2)	6.9 (6.9 to 6.9)	521.2 (463.3 to 584.5)	90.3 (72.7 to 110.5)	398.0 (345.5 to 458.7)	15.2 (6.9 to 28.8)	17.7 (17.7 to 17.7)
Equatorial Guinea	2004	222.1 (198.3 to 247.7)	44.5 (36.4 to 54.4)	160.8 (139.3 to 184.2)	7.6 (3.5 to 14.6)	9.2 (9.2 to 9.2)	571.4 (510.3 to 637.4)	114.6 (93.7 to 139.9)	413.7 (358.4 to 473.9)	19.5 (9.0 to 37.6)	23.6 (23.6 to 23.6)
Equatorial Guinea	2005	214.7 (193.8 to 237.4)	46.5 (38.4 to 56.6)	141.7 (123.1 to 162.5)	8.2 (3.6 to 15.8)	18.4 (18.4 to 18.4)	552.4 (498.5 to 610.9)	119.7 (98.7 to 145.5)	364.5 (316.7 to 418.1)	21.0 (9.3 to 40.7)	47.2 (47.2 to 47.2)
Equatorial Guinea	2006	212.2 (191.6 to 233.6)	50.0 (41.5 to 60.3)	130.7 (114.1 to 150.7)	8.8 (3.9 to 16.7)	22.7 (22.7 to 22.7)	546.0 (492.9 to 601.0)	128.6 (106.8 to 155.2)	336.4 (293.6 to 387.7)	22.7 (10.1 to 43.1)	58.3 (58.3 to 58.3)
Equatorial Guinea	2007	200.5 (180.8 to 222.1)	57.6 (48.0 to 68.2)	132.3 (116.9 to 152.2)	10.1 (4.6 to 19.5)	0.5 (0.5 to 0.5)	515.9 (465.3 to 571.5)	148.1 (123.4 to 175.5)	340.5 (300.9 to 391.5)	26.1 (11.9 to 50.1)	1.3 (1.3 to 1.3)
Equatorial Guinea	2008	223.5 (201.5 to 246.7)	68.4 (57.5 to 80.4)	142.0 (125.4 to 161.5)	12.0 (5.6 to 23.6)	1.0 (1.0 to 1.0)	574.9 (518.4 to 634.8)	175.9 (147.9 to 207.0)	365.4 (322.6 to 415.5)	31.0 (14.5 to 60.7)	2.6 (2.6 to 2.6)
Equatorial Guinea	2009	216.5 (195.9 to 239.2)	61.0 (51.5 to 71.8)	144.0 (128.1 to 161.7)	11.0 (5.1 to 21.0)	0.5 (0.5 to 0.5)	556.9 (504.1 to 615.3)	157.0 (132.4 to 184.9)	370.4 (329.5 to 416.1)	28.2 (13.1 to 54.1)	1.3 (1.3 to 1.3)
Equatorial Guinea	2010	200.0 (180.7 to 219.6)	52.6 (44.4 to 61.7)	137.5 (122.0 to 153.3)	9.6 (4.4 to 18.5)	0.3 (0.3 to 0.3)	514.6 (464.8 to 564.9)	135.2 (114.1 to 158.6)	353.9 (313.8 to 394.4)	24.7 (11.3 to 47.7)	0.8 (0.8 to 0.8)
Equatorial Guinea	2011	203.7 (184.9 to 223.8)	54.3 (45.8 to 63.9)	139.3 (123.8 to 156.3)	9.8 (4.5 to 18.9)	0.3 (0.3 to 0.3)	524.1 (475.8 to 575.8)	139.7 (117.8 to 164.4)	358.4 (318.6 to 402.2)	25.2 (11.6 to 48.7)	0.8 (0.8 to 0.8)
Equatorial Guinea	2012	218.0 (198.2 to 239.8)	59.5 (49.7 to 70.9)	147.5 (131.8 to 164.2)	10.6 (4.7 to 20.0)	0.3 (0.3 to 0.3)	560.8 (510.1 to 617.1)	153.1 (127.9 to 182.3)	379.5 (339.2 to 422.5)	27.3 (12.2 to 51.5)	0.9 (0.9 to 0.9)
Equatorial Guinea	2013	234.5 (215.0 to 258.4)	61.6 (50.9 to 73.5)	161.7 (145.7 to 179.9)	11.0 (4.9 to 20.7)	0.3 (0.3 to 0.3)	603.4 (553.2 to 664.9)	158.4 (130.9 to 189.0)	416.0 (374.8 to 462.9)	28.4 (12.5 to 53.2)	0.7 (0.7 to 0.7)
Equatorial Guinea	2014	254.3 (232.0 to 279.5)	62.9 (51.7 to 75.7)	179.3 (159.4 to 200.6)	11.6 (5.1 to 21.7)	0.5 (0.5 to 0.5)	654.2 (597.0 to 719.2)	161.7 (133.0 to 194.8)	461.3 (410.1 to 516.2)	29.9 (13.2 to 55.8)	1.3 (1.3 to 1.3)
Equatorial Guinea	2015	288.6 (260.3 to 320.0)	65.8 (53.4 to 79.8)	206.1 (179.0 to 232.4)	12.5 (5.6 to 22.6)	4.1 (4.1 to 4.1)	742.5 (669.7 to 823.2)	169.4 (137.3 to 205.3)	530.4 (460.7 to 597.9)	32.0 (14.4 to 58.2)	10.7 (10.7 to 10.7)

Country	Year	2018 US Dollars per capita					2018 PPP per capita				
		Health spending	Government health spending	Out-of-pocket health spending	Prepaid private health spending	Development assistance for health	Health spending	Government health spending	Out-of-pocket health spending	Prepaid private health spending	Development assistance for health
Equatorial Guinea	2016	309.9 (275.1 to 351.0)	66.6 (53.8 to 81.1)	222.1 (190.3 to 257.8)	12.8 (5.8 to 22.8)	8.5 (8.5 to 8.5)	797.4 (707.9 to 903.1)	171.3 (138.5 to 208.8)	571.3 (489.5 to 663.3)	32.9 (15.0 to 58.6)	21.9 (21.9 to 21.9)
Equatorial Guinea	2017	290.4 (255.0 to 328.5)	51.4 (41.5 to 62.4)	218.0 (185.4 to 252.9)	11.0 (5.0 to 19.6)	10.0 (10.0 to 10.0)	747.1 (656.0 to 845.3)	132.3 (106.8 to 160.6)	560.8 (477.0 to 650.8)	28.3 (12.9 to 50.4)	25.8 (25.8 to 25.8)
Equatorial Guinea	2018	291.9 (256.0 to 331.1)	51.6 (41.7 to 62.5)	223.2 (189.5 to 259.9)	11.2 (5.1 to 20.0)	5.8 (0.0 to 9.0)	751.0 (658.6 to 851.8)	132.9 (107.2 to 160.7)	574.4 (487.6 to 668.7)	28.7 (13.1 to 51.4)	15.0 (0.0 to 23.3)
Equatorial Guinea	2019	298.0 (262.3 to 338.9)	51.9 (41.9 to 62.8)	229.2 (195.0 to 266.3)	11.2 (5.1 to 20.1)	5.6 (0.0 to 9.3)	766.7 (674.8 to 871.9)	133.6 (107.8 to 161.6)	589.8 (501.6 to 685.1)	28.9 (13.2 to 51.7)	14.4 (0.0 to 23.9)
Equatorial Guinea	2020	299.0 (262.5 to 339.6)	52.2 (42.1 to 63.1)	230.0 (195.6 to 267.9)	11.4 (5.2 to 20.4)	5.4 (0.0 to 9.3)	769.2 (675.4 to 873.7)	134.3 (108.4 to 162.4)	591.7 (503.2 to 689.2)	29.3 (13.4 to 52.4)	14.0 (0.0 to 24.0)
Equatorial Guinea	2021	297.0 (261.0 to 336.3)	52.1 (42.1 to 63.1)	228.2 (194.2 to 265.2)	11.4 (5.2 to 20.3)	5.3 (0.0 to 9.5)	764.1 (671.4 to 865.2)	134.0 (108.2 to 162.3)	587.1 (499.6 to 682.3)	29.3 (13.3 to 52.3)	13.7 (0.0 to 24.5)
Equatorial Guinea	2022	298.5 (258.6 to 338.5)	51.9 (42.0 to 63.0)	229.9 (194.0 to 266.6)	11.4 (5.2 to 20.3)	5.3 (0.0 to 9.7)	767.9 (665.3 to 870.8)	133.6 (108.0 to 162.0)	591.4 (499.3 to 686.0)	29.3 (13.4 to 52.3)	13.6 (0.0 to 24.8)
Equatorial Guinea	2023	305.3 (264.0 to 348.4)	52.3 (42.1 to 63.5)	236.3 (198.7 to 274.7)	11.5 (5.2 to 20.5)	5.3 (0.0 to 9.7)	785.4 (679.3 to 896.4)	134.5 (108.4 to 163.5)	607.9 (511.2 to 706.8)	29.5 (13.5 to 52.7)	13.6 (0.0 to 25.1)
Equatorial Guinea	2024	314.6 (271.3 to 358.1)	52.7 (42.3 to 64.1)	245.2 (207.4 to 286.4)	11.6 (5.3 to 20.7)	5.2 (0.0 to 10.0)	809.4 (698.1 to 921.5)	135.6 (108.8 to 164.9)	630.8 (533.6 to 736.8)	29.8 (13.6 to 53.3)	13.3 (0.0 to 25.6)
Equatorial Guinea	2025	325.9 (280.3 to 371.9)	53.6 (42.9 to 65.1)	255.3 (216.0 to 298.6)	11.8 (5.4 to 21.1)	5.2 (0.0 to 10.1)	838.4 (721.3 to 957.0)	138.0 (110.3 to 167.4)	656.8 (555.7 to 768.4)	30.3 (13.9 to 54.4)	13.3 (0.0 to 26.1)
Equatorial Guinea	2026	337.8 (291.9 to 389.1)	54.7 (43.6 to 66.3)	265.9 (224.6 to 312.2)	12.0 (5.5 to 21.5)	5.1 (0.0 to 10.3)	869.0 (751.0 to 1001.1)	140.8 (112.1 to 170.6)	684.2 (577.8 to 803.3)	30.9 (14.1 to 55.4)	13.2 (0.0 to 26.5)
Equatorial Guinea	2027	350.0 (301.8 to 403.0)	55.7 (44.2 to 67.4)	277.1 (233.8 to 326.6)	12.2 (5.6 to 22.0)	5.0 (0.0 to 10.6)	900.5 (776.4 to 1036.9)	143.2 (113.6 to 173.5)	713.0 (601.5 to 840.2)	31.4 (14.4 to 56.6)	12.9 (0.0 to 27.2)
Equatorial Guinea	2028	363.9 (310.6 to 423.2)	57.1 (45.2 to 69.3)	289.4 (242.0 to 344.7)	12.5 (5.7 to 22.6)	5.0 (0.0 to 10.9)	936.4 (799.2 to 1088.9)	146.8 (116.2 to 178.4)	744.5 (622.6 to 886.9)	32.2 (14.7 to 58.2)	12.8 (0.0 to 28.0)
Equatorial Guinea	2029	378.0 (323.2 to 440.6)	58.4 (46.2 to 71.2)	301.9 (251.3 to 361.1)	12.9 (5.8 to 23.3)	4.8 (0.0 to 11.1)	972.6 (831.7 to 1133.5)	150.3 (118.8 to 183.2)	776.7 (646.6 to 929.2)	33.1 (15.0 to 59.9)	12.4 (0.0 to 28.7)
Equatorial Guinea	2030	392.3 (333.8 to 459.3)	59.9 (47.3 to 73.3)	314.5 (259.0 to 379.1)	13.2 (6.0 to 23.9)	4.7 (0.0 to 11.2)	1009.2 (858.7 to 1181.7)	154.1 (121.8 to 188.5)	809.1 (666.4 to 975.4)	33.9 (15.4 to 61.6)	12.2 (0.0 to 28.9)
Equatorial Guinea	2031	406.3 (344.2 to 475.1)	61.3 (48.5 to 75.2)	326.9 (266.5 to 395.4)	13.5 (6.1 to 24.4)	4.7 (0.0 to 11.5)	1045.3 (885.5 to 1222.5)	157.6 (124.8 to 193.5)	841.0 (685.6 to 1017.3)	34.7 (15.7 to 62.9)	12.0 (0.0 to 29.7)
Equatorial Guinea	2032	420.3 (352.6 to 496.2)	62.6 (49.6 to 76.7)	339.3 (274.0 to 412.6)	13.8 (6.2 to 25.0)	4.6 (0.0 to 11.6)	1081.5 (907.3 to 1276.6)	161.1 (127.6 to 197.4)	873.1 (705.0 to 1061.6)	35.5 (16.0 to 64.3)	11.7 (0.0 to 29.8)
Equatorial Guinea	2033	435.2 (361.2 to 520.1)	64.4 (50.9 to 78.9)	352.1 (283.9 to 431.3)	14.1 (6.4 to 25.7)	4.5 (0.0 to 12.2)	1119.6 (929.3 to 1338.1)	165.6 (131.1 to 203.0)	906.0 (730.3 to 1109.7)	36.4 (16.3 to 66.1)	11.7 (0.0 to 31.3)
Equatorial Guinea	2034	448.6 (371.3 to 534.9)	65.5 (51.8 to 80.3)	364.2 (290.6 to 449.8)	14.4 (6.5 to 26.2)	4.5 (0.0 to 12.2)	1154.1 (955.3 to 1376.3)	168.5 (133.2 to 206.7)	936.9 (747.8 to 1157.3)	37.1 (16.6 to 67.5)	11.6 (0.0 to 31.3)
Equatorial Guinea	2035	463.0 (381.9 to 554.2)	67.1 (52.9 to 82.5)	376.7 (299.1 to 468.7)	14.8 (6.6 to 26.9)	4.5 (0.0 to 12.4)	1191.3 (982.5 to 1425.8)	172.7 (136.2 to 212.2)	969.1 (769.4 to 1205.9)	38.0 (17.1 to 69.2)	11.5 (0.0 to 32.0)
Equatorial Guinea	2036	476.6 (392.7 to 574.3)	68.6 (54.1 to 84.5)	388.4 (305.4 to 486.6)	15.1 (6.8 to 27.4)	4.4 (0.0 to 13.0)	1226.2 (1010.3 to 1477.5)	176.6 (139.3 to 217.4)	999.3 (785.7 to 1251.9)	38.8 (17.5 to 70.5)	11.4 (0.0 to 33.5)

		2018 US Dollars per capita					2018 PPP per capita				
Country	Year	Health spending	Government health spending	Out-of-pocket health spending	Prepaid private health spending	Development assistance for health	Health spending	Government health spending	Out-of-pocket health spending	Prepaid private health spending	Development assistance for health
Equatorial Guinea	2037	490.8 (400.7 to 593.6)	70.3 (55.2 to 86.5)	400.6 (311.9 to 503.0)	15.5 (7.0 to 28.1)	4.4 (0.0 to 13.1)	1262.9 (1031.0 to 1527.3)	181.0 (142.1 to 222.6)	1030.8 (802.6 to 1294.3)	39.8 (18.0 to 72.3)	11.3 (0.0 to 33.7)
Equatorial Guinea	2038	504.1 (406.0 to 610.7)	71.7 (56.4 to 88.3)	412.1 (319.1 to 520.2)	15.8 (7.2 to 28.7)	4.4 (0.0 to 13.4)	1296.9 (1044.7 to 1571.2)	184.5 (145.0 to 227.3)	1060.3 (820.9 to 1338.5)	40.7 (18.4 to 73.7)	11.3 (0.0 to 34.5)
Equatorial Guinea	2039	516.7 (416.3 to 628.5)	73.1 (57.0 to 90.1)	423.1 (326.3 to 537.6)	16.2 (7.3 to 29.3)	4.4 (0.0 to 13.8)	1329.4 (1071.2 to 1617.0)	188.0 (146.7 to 231.9)	1088.6 (839.4 to 1383.1)	41.6 (18.9 to 75.5)	11.3 (0.0 to 35.5)
Equatorial Guinea	2040	529.4 (426.6 to 653.0)	74.4 (57.9 to 91.9)	434.1 (333.3 to 554.1)	16.5 (7.5 to 30.1)	4.4 (0.0 to 14.1)	1362.1 (1097.7 to 1680.2)	191.5 (149.0 to 236.4)	1116.8 (857.6 to 1425.6)	42.5 (19.3 to 77.6)	11.3 (0.0 to 36.4)
Equatorial Guinea	2041	542.1 (438.1 to 668.8)	76.0 (59.0 to 94.0)	444.9 (341.0 to 569.0)	16.9 (7.7 to 31.0)	4.3 (0.0 to 14.5)	1394.8 (1127.3 to 1720.8)	195.6 (151.8 to 242.0)	1144.7 (877.4 to 1464.0)	43.5 (19.8 to 79.7)	11.0 (0.0 to 37.4)
Equatorial Guinea	2042	554.7 (444.5 to 689.1)	77.7 (60.1 to 96.2)	455.4 (348.5 to 584.7)	17.3 (7.9 to 31.8)	4.3 (0.0 to 15.3)	1427.3 (1143.8 to 1772.9)	200.0 (154.6 to 247.5)	1171.7 (896.7 to 1504.5)	44.6 (20.4 to 81.9)	11.0 (0.0 to 39.4)
Equatorial Guinea	2043	567.3 (456.3 to 704.5)	79.5 (61.3 to 98.6)	465.8 (356.4 to 599.1)	17.8 (8.1 to 32.8)	4.2 (0.0 to 15.5)	1459.5 (1174.0 to 1812.5)	204.6 (157.8 to 253.6)	1198.3 (916.9 to 1541.5)	45.7 (20.9 to 84.3)	10.9 (0.0 to 39.9)
Equatorial Guinea	2044	578.9 (464.6 to 723.2)	81.1 (62.3 to 100.8)	475.5 (363.2 to 612.5)	18.2 (8.3 to 33.6)	4.2 (0.0 to 15.8)	1489.5 (1195.3 to 1860.8)	208.6 (160.2 to 259.4)	1223.3 (934.4 to 1575.9)	46.7 (21.4 to 86.5)	10.8 (0.0 to 40.5)
Equatorial Guinea	2045	591.1 (475.9 to 734.1)	83.0 (63.8 to 103.4)	485.2 (370.6 to 625.6)	18.6 (8.6 to 34.6)	4.2 (0.0 to 15.7)	1520.7 (1224.5 to 1888.7)	213.6 (164.0 to 265.9)	1248.4 (953.4 to 1609.5)	47.9 (22.0 to 89.0)	10.8 (0.0 to 40.4)
Equatorial Guinea	2046	603.6 (487.0 to 752.1)	85.5 (65.5 to 106.7)	494.8 (377.9 to 638.1)	19.1 (8.8 to 35.6)	4.2 (0.0 to 16.4)	1552.9 (1252.9 to 1935.1)	219.9 (168.5 to 274.6)	1273.0 (972.3 to 1641.6)	49.2 (22.7 to 91.5)	10.8 (0.0 to 42.1)
Equatorial Guinea	2047	614.3 (493.2 to 757.6)	87.3 (66.6 to 109.1)	503.3 (384.2 to 650.7)	19.5 (9.0 to 36.3)	4.1 (0.0 to 17.4)	1580.6 (1269.0 to 1949.1)	224.7 (171.2 to 280.7)	1294.9 (988.6 to 1674.2)	50.3 (23.1 to 93.5)	10.6 (0.0 to 44.7)
Equatorial Guinea	2048	627.1 (503.0 to 781.4)	90.1 (69.1 to 112.6)	512.8 (390.7 to 662.6)	20.1 (9.2 to 37.2)	4.2 (0.0 to 16.7)	1613.5 (1294.3 to 2010.4)	231.7 (177.8 to 289.8)	1319.3 (1005.1 to 1704.7)	51.6 (23.7 to 95.8)	10.9 (0.0 to 42.9)
Equatorial Guinea	2049	640.9 (513.9 to 802.5)	93.2 (71.3 to 117.3)	522.9 (398.4 to 672.7)	20.6 (9.4 to 38.2)	4.3 (0.0 to 17.6)	1649.0 (1322.2 to 2064.8)	239.7 (183.6 to 301.7)	1345.3 (1025.0 to 1730.9)	53.0 (24.3 to 98.2)	11.0 (0.0 to 45.3)
Equatorial Guinea	2050	655.3 (523.4 to 823.8)	96.6 (73.6 to 121.8)	533.3 (405.9 to 685.4)	21.1 (9.7 to 39.2)	4.3 (0.0 to 18.3)	1686.1 (1346.7 to 2119.6)	248.5 (189.3 to 313.3)	1372.0 (1044.3 to 1763.3)	54.4 (24.9 to 100.9)	11.2 (0.0 to 47.2)
Eritrea	1995	47.9 (39.0 to 59.2)	14.2 (10.9 to 18.2)	28.9 (20.6 to 38.6)	1.2 (0.5 to 2.1)	3.6 (3.6 to 3.6)	72.8 (59.2 to 89.9)	21.6 (16.5 to 27.6)	43.9 (31.3 to 58.6)	1.7 (0.8 to 3.2)	5.5 (5.5 to 5.5)
Eritrea	1996	50.4 (41.3 to 61.9)	14.2 (10.8 to 18.3)	30.2 (22.1 to 40.5)	1.2 (0.5 to 2.2)	4.8 (4.8 to 4.8)	76.5 (62.8 to 94.0)	21.6 (16.5 to 27.8)	45.9 (33.6 to 61.4)	1.8 (0.8 to 3.3)	7.3 (7.3 to 7.3)
Eritrea	1997	51.9 (41.8 to 64.1)	15.3 (11.7 to 19.8)	33.4 (24.3 to 44.7)	1.3 (0.6 to 2.4)	1.8 (1.8 to 1.8)	78.8 (63.5 to 97.3)	23.3 (17.8 to 30.1)	50.8 (36.8 to 67.8)	2.0 (0.9 to 3.6)	2.8 (2.8 to 2.8)
Eritrea	1998	58.7 (47.2 to 71.7)	16.2 (12.4 to 20.8)	36.5 (26.1 to 49.2)	1.4 (0.6 to 2.6)	4.6 (4.6 to 4.6)	89.1 (71.7 to 108.8)	24.6 (18.8 to 31.5)	55.4 (39.6 to 74.7)	2.1 (1.0 to 3.9)	7.0 (7.0 to 7.0)
Eritrea	1999	63.0 (51.1 to 76.7)	17.1 (13.0 to 21.8)	38.3 (27.8 to 51.0)	1.5 (0.7 to 2.7)	6.1 (6.1 to 6.1)	95.6 (77.6 to 116.4)	25.9 (19.7 to 33.1)	58.2 (42.2 to 77.4)	2.3 (1.0 to 4.2)	9.3 (9.3 to 9.3)
Eritrea	2000	65.2 (53.8 to 78.5)	18.1 (13.6 to 23.1)	37.1 (26.8 to 49.4)	1.5 (0.7 to 2.9)	8.4 (8.4 to 8.4)	99.0 (81.7 to 119.3)	27.4 (20.7 to 35.0)	56.4 (40.7 to 75.0)	2.4 (1.1 to 4.4)	12.8 (12.8 to 12.8)
Eritrea	2001	55.0 (45.0 to 66.7)	16.7 (12.6 to 21.3)	32.4 (23.4 to 42.8)	1.5 (0.7 to 2.7)	4.5 (4.5 to 4.5)	83.5 (68.3 to 101.4)	25.3 (19.1 to 32.4)	49.1 (35.5 to 65.0)	2.2 (1.0 to 4.1)	6.9 (6.9 to 6.9)

		2018 US Dollars per capita					2018 PPP per capita				
Country	Year	Health spending	Government health spending	Out-of-pocket health spending	Prepaid private health spending	Development assistance for health	Health spending	Government health spending	Out-of-pocket health spending	Prepaid private health spending	Development assistance for health
Eritrea	2002	54.4 (45.3 to 65.7)	16.3 (12.2 to 21.2)	29.7 (21.5 to 39.2)	1.4 (0.7 to 2.8)	7.0 (7.0 to 7.0)	82.7 (68.8 to 99.8)	24.8 (18.6 to 32.2)	45.1 (32.7 to 59.5)	2.2 (1.0 to 4.2)	10.6 (10.6 to 10.6)
Eritrea	2003	52.4 (44.3 to 61.9)	13.9 (10.4 to 18.1)	26.1 (18.9 to 34.5)	1.3 (0.6 to 2.4)	11.1 (11.1 to 11.1)	79.6 (67.2 to 94.0)	21.1 (15.8 to 27.4)	39.7 (28.6 to 52.4)	1.9 (0.9 to 3.7)	16.9 (16.9 to 16.9)
Eritrea	2004	45.6 (38.5 to 54.1)	11.4 (8.6 to 15.0)	22.9 (16.6 to 30.3)	1.1 (0.5 to 2.1)	10.3 (10.3 to 10.3)	69.3 (58.5 to 82.2)	17.3 (13.0 to 22.8)	34.7 (25.3 to 46.1)	1.6 (0.8 to 3.2)	15.6 (15.6 to 15.6)
Eritrea	2005	40.0 (33.5 to 47.6)	8.9 (6.7 to 11.6)	20.7 (15.0 to 27.5)	0.9 (0.4 to 1.7)	9.6 (9.6 to 9.6)	60.7 (50.9 to 72.2)	13.5 (10.2 to 17.6)	31.4 (22.7 to 41.8)	1.3 (0.6 to 2.6)	14.5 (14.5 to 14.5)
Eritrea	2006	32.4 (26.8 to 39.4)	7.6 (5.7 to 9.9)	19.3 (14.0 to 26.0)	0.8 (0.4 to 1.5)	4.8 (4.8 to 4.8)	49.3 (40.6 to 59.8)	11.5 (8.6 to 15.0)	29.3 (21.2 to 39.4)	1.2 (0.5 to 2.3)	7.3 (7.3 to 7.3)
Eritrea	2007	32.3 (26.6 to 38.9)	7.1 (5.3 to 9.2)	18.7 (13.5 to 25.1)	0.7 (0.3 to 1.4)	5.8 (5.8 to 5.8)	49.0 (40.4 to 59.1)	10.8 (8.0 to 13.9)	28.4 (20.4 to 38.1)	1.1 (0.5 to 2.1)	8.8 (8.8 to 8.8)
Eritrea	2008	28.7 (23.9 to 34.5)	5.1 (3.8 to 6.6)	16.4 (11.9 to 22.0)	0.5 (0.3 to 1.0)	6.7 (6.7 to 6.7)	43.6 (36.3 to 52.4)	7.8 (5.8 to 10.1)	24.9 (18.1 to 33.5)	0.8 (0.4 to 1.6)	10.1 (10.1 to 10.1)
Eritrea	2009	28.6 (23.5 to 34.9)	6.1 (4.6 to 7.8)	17.3 (12.6 to 23.2)	0.6 (0.3 to 1.1)	4.7 (4.7 to 4.7)	43.5 (35.8 to 53.0)	9.2 (6.9 to 11.9)	26.2 (19.1 to 35.3)	0.9 (0.4 to 1.7)	7.1 (7.1 to 7.1)
Eritrea	2010	34.1 (29.4 to 40.1)	5.4 (4.0 to 6.9)	16.6 (12.1 to 22.5)	0.5 (0.3 to 1.0)	11.6 (11.6 to 11.6)	51.8 (44.7 to 60.8)	8.2 (6.1 to 10.5)	25.2 (18.4 to 34.2)	0.8 (0.4 to 1.5)	17.6 (17.6 to 17.6)
Eritrea	2011	27.2 (22.7 to 33.3)	5.3 (4.0 to 6.8)	16.8 (12.2 to 22.5)	0.5 (0.2 to 1.0)	4.7 (4.7 to 4.7)	41.4 (34.4 to 50.6)	8.1 (6.1 to 10.4)	25.4 (18.5 to 34.1)	0.8 (0.4 to 1.4)	7.1 (7.1 to 7.1)
Eritrea	2012	29.5 (24.9 to 35.6)	5.1 (3.9 to 6.5)	17.0 (12.2 to 23.0)	0.5 (0.2 to 0.9)	6.9 (6.9 to 6.9)	44.9 (37.8 to 54.1)	7.8 (5.9 to 9.9)	25.8 (18.6 to 34.9)	0.7 (0.4 to 1.4)	10.5 (10.5 to 10.5)
Eritrea	2013	28.4 (23.8 to 34.4)	4.3 (3.2 to 5.4)	16.7 (11.9 to 22.8)	0.4 (0.2 to 0.8)	7.0 (7.0 to 7.0)	43.1 (36.2 to 52.3)	6.5 (4.9 to 8.3)	25.4 (18.1 to 34.6)	0.6 (0.3 to 1.2)	10.6 (10.6 to 10.6)
Eritrea	2014	29.4 (24.5 to 35.5)	4.2 (3.2 to 5.4)	17.2 (12.3 to 23.4)	0.4 (0.2 to 0.8)	7.5 (7.5 to 7.5)	44.6 (37.3 to 53.9)	6.4 (4.9 to 8.3)	26.1 (18.6 to 35.5)	0.6 (0.3 to 1.2)	11.4 (11.4 to 11.4)
Eritrea	2015	26.2 (20.9 to 32.8)	4.6 (3.5 to 6.0)	17.9 (12.7 to 24.4)	0.4 (0.2 to 0.8)	3.2 (3.2 to 3.2)	39.9 (31.8 to 49.8)	7.1 (5.4 to 9.1)	27.2 (19.3 to 37.0)	0.7 (0.3 to 1.3)	4.9 (4.9 to 4.9)
Eritrea	2016	30.0 (24.3 to 37.3)	6.1 (4.6 to 7.8)	19.0 (13.4 to 26.3)	0.5 (0.3 to 1.0)	4.4 (4.4 to 4.4)	45.6 (36.8 to 56.6)	9.2 (6.9 to 11.9)	28.9 (20.3 to 39.9)	0.8 (0.4 to 1.5)	6.7 (6.7 to 6.7)
Eritrea	2017	32.9 (26.9 to 40.5)	6.1 (4.6 to 7.9)	19.3 (13.5 to 26.5)	0.5 (0.3 to 1.0)	7.0 (7.0 to 7.0)	49.9 (40.9 to 61.5)	9.3 (7.0 to 12.0)	29.3 (20.5 to 40.3)	0.8 (0.4 to 1.5)	10.6 (10.6 to 10.6)
Eritrea	2018	32.6 (26.4 to 39.9)	6.2 (4.7 to 8.0)	19.4 (13.6 to 26.7)	0.6 (0.3 to 1.0)	6.5 (6.5 to 6.5)	49.5 (40.1 to 60.6)	9.4 (7.1 to 12.2)	29.4 (20.6 to 40.6)	0.8 (0.4 to 1.6)	9.9 (9.9 to 9.9)
Eritrea	2019	32.8 (26.8 to 40.4)	6.3 (4.7 to 8.1)	19.4 (13.6 to 26.8)	0.6 (0.3 to 1.1)	6.5 (6.1 to 6.8)	49.7 (40.7 to 61.3)	9.6 (7.2 to 12.3)	29.5 (20.7 to 40.7)	0.8 (0.4 to 1.6)	9.8 (9.2 to 10.4)
Eritrea	2020	32.9 (26.6 to 40.6)	6.4 (4.8 to 8.2)	19.5 (13.6 to 26.9)	0.6 (0.3 to 1.1)	6.5 (6.0 to 7.0)	50.0 (40.4 to 61.7)	9.7 (7.3 to 12.5)	29.6 (20.7 to 40.9)	0.9 (0.4 to 1.6)	9.9 (9.1 to 10.7)
Eritrea	2021	33.2 (27.2 to 40.8)	6.5 (4.8 to 8.3)	19.6 (13.6 to 27.1)	0.6 (0.3 to 1.1)	6.6 (5.9 to 7.2)	50.4 (41.3 to 61.9)	9.8 (7.3 to 12.6)	29.8 (20.7 to 41.1)	0.9 (0.4 to 1.7)	10.0 (9.0 to 11.0)
Eritrea	2022	33.6 (27.1 to 41.2)	6.6 (4.9 to 8.5)	19.8 (13.8 to 27.3)	0.6 (0.3 to 1.1)	6.6 (5.9 to 7.4)	51.0 (41.2 to 62.6)	10.0 (7.5 to 12.9)	30.1 (20.9 to 41.5)	0.9 (0.4 to 1.7)	10.1 (8.9 to 11.3)

Country	Year	2018 US Dollars per capita					2018 PPP per capita				
		Health spending	Government health spending	Out-of-pocket health spending	Prepaid private health spending	Development assistance for health	Health spending	Government health spending	Out-of-pocket health spending	Prepaid private health spending	Development assistance for health
Eritrea	2023	34.0 (27.5 to 41.7)	6.7 (5.0 to 8.6)	20.0 (13.9 to 27.6)	0.6 (0.3 to 1.1)	6.7 (5.8 to 7.6)	51.6 (41.7 to 63.4)	10.2 (7.6 to 13.1)	30.4 (21.0 to 41.9)	0.9 (0.4 to 1.7)	10.2 (8.9 to 11.6)
Eritrea	2024	34.4 (27.9 to 42.4)	6.8 (5.1 to 8.8)	20.2 (13.9 to 27.8)	0.6 (0.3 to 1.2)	6.8 (5.9 to 7.9)	52.2 (42.4 to 64.4)	10.3 (7.7 to 13.3)	30.6 (21.2 to 42.3)	0.9 (0.4 to 1.8)	10.3 (8.9 to 11.9)
Eritrea	2025	34.9 (28.4 to 42.7)	6.9 (5.1 to 8.9)	20.4 (14.2 to 28.2)	0.6 (0.3 to 1.2)	6.9 (5.9 to 8.1)	52.9 (43.1 to 64.9)	10.5 (7.8 to 13.6)	31.0 (21.5 to 42.8)	1.0 (0.4 to 1.8)	10.5 (8.9 to 12.3)
Eritrea	2026	35.4 (28.7 to 43.6)	7.0 (5.2 to 9.1)	20.7 (14.4 to 28.6)	0.6 (0.3 to 1.2)	7.0 (5.9 to 8.2)	53.7 (43.7 to 66.3)	10.7 (7.9 to 13.8)	31.5 (21.9 to 43.4)	1.0 (0.5 to 1.8)	10.6 (8.9 to 12.4)
Eritrea	2027	35.9 (29.0 to 44.3)	7.1 (5.3 to 9.2)	21.0 (14.6 to 29.0)	0.7 (0.3 to 1.2)	7.1 (5.9 to 8.4)	54.5 (44.0 to 67.2)	10.9 (8.1 to 14.0)	31.9 (22.2 to 44.0)	1.0 (0.5 to 1.9)	10.7 (9.0 to 12.8)
Eritrea	2028	36.5 (29.3 to 45.2)	7.3 (5.4 to 9.4)	21.3 (14.9 to 29.4)	0.7 (0.3 to 1.3)	7.2 (6.0 to 8.7)	55.4 (44.5 to 68.6)	11.0 (8.2 to 14.3)	32.4 (22.6 to 44.6)	1.0 (0.5 to 1.9)	10.9 (9.1 to 13.2)
Eritrea	2029	37.0 (29.9 to 45.5)	7.4 (5.5 to 9.6)	21.6 (15.1 to 29.8)	0.7 (0.3 to 1.3)	7.3 (6.0 to 8.9)	56.2 (45.5 to 69.1)	11.2 (8.4 to 14.6)	32.9 (22.9 to 45.3)	1.0 (0.5 to 2.0)	11.1 (9.1 to 13.5)
Eritrea	2030	37.5 (30.1 to 46.3)	7.5 (5.6 to 9.7)	21.9 (15.3 to 30.3)	0.7 (0.3 to 1.3)	7.4 (6.1 to 9.1)	57.0 (45.8 to 70.3)	11.4 (8.5 to 14.8)	33.3 (23.2 to 46.1)	1.1 (0.5 to 2.0)	11.3 (9.2 to 13.8)
Eritrea	2031	38.1 (30.3 to 47.0)	7.6 (5.7 to 9.9)	22.2 (15.4 to 30.7)	0.7 (0.3 to 1.3)	7.5 (6.1 to 9.4)	57.9 (46.0 to 71.4)	11.6 (8.6 to 15.0)	33.8 (23.5 to 46.7)	1.1 (0.5 to 2.0)	11.4 (9.3 to 14.3)
Eritrea	2032	38.6 (31.3 to 47.2)	7.7 (5.7 to 10.0)	22.5 (15.6 to 31.1)	0.7 (0.3 to 1.4)	7.7 (6.1 to 9.6)	58.7 (47.5 to 71.7)	11.7 (8.7 to 15.2)	34.2 (23.7 to 47.3)	1.1 (0.5 to 2.1)	11.6 (9.3 to 14.6)
Eritrea	2033	39.2 (31.4 to 48.2)	7.9 (5.8 to 10.2)	22.8 (15.8 to 31.5)	0.7 (0.3 to 1.4)	7.8 (6.2 to 10.0)	59.5 (47.7 to 73.2)	11.9 (8.8 to 15.5)	34.6 (24.1 to 47.9)	1.1 (0.5 to 2.2)	11.8 (9.4 to 15.2)
Eritrea	2034	39.8 (32.2 to 49.0)	8.0 (5.8 to 10.4)	23.1 (16.1 to 32.0)	0.8 (0.4 to 1.5)	8.0 (6.3 to 10.2)	60.4 (48.9 to 74.4)	12.1 (8.9 to 15.7)	35.1 (24.4 to 48.6)	1.2 (0.5 to 2.2)	12.1 (9.5 to 15.6)
Eritrea	2035	40.4 (32.4 to 49.7)	8.1 (5.9 to 10.5)	23.4 (16.3 to 32.4)	0.8 (0.4 to 1.5)	8.1 (6.3 to 10.6)	61.3 (49.2 to 75.4)	12.3 (9.0 to 16.0)	35.5 (24.7 to 49.2)	1.2 (0.5 to 2.2)	12.3 (9.6 to 16.1)
Eritrea	2036	41.0 (32.9 to 50.9)	8.2 (6.0 to 10.7)	23.7 (16.5 to 32.8)	0.8 (0.4 to 1.5)	8.3 (6.4 to 11.1)	62.2 (49.9 to 77.3)	12.5 (9.2 to 16.3)	36.0 (25.0 to 49.8)	1.2 (0.6 to 2.3)	12.5 (9.7 to 16.8)
Eritrea	2037	41.6 (33.1 to 50.8)	8.4 (6.1 to 10.9)	24.0 (16.6 to 33.2)	0.8 (0.4 to 1.5)	8.4 (6.5 to 11.2)	63.2 (50.3 to 77.1)	12.7 (9.3 to 16.6)	36.5 (25.3 to 50.5)	1.2 (0.6 to 2.3)	12.8 (9.8 to 17.1)
Eritrea	2038	42.2 (34.0 to 52.1)	8.5 (6.2 to 11.1)	24.3 (16.9 to 33.8)	0.8 (0.4 to 1.6)	8.6 (6.5 to 11.9)	64.1 (51.6 to 79.1)	12.9 (9.5 to 16.9)	36.9 (25.6 to 51.3)	1.2 (0.6 to 2.4)	13.0 (9.9 to 18.0)
Eritrea	2039	42.8 (34.4 to 52.5)	8.7 (6.3 to 11.3)	24.6 (17.1 to 34.2)	0.8 (0.4 to 1.6)	8.7 (6.7 to 12.1)	65.0 (52.3 to 79.7)	13.2 (9.6 to 17.2)	37.3 (25.9 to 52.0)	1.3 (0.6 to 2.4)	13.2 (10.1 to 18.4)
Eritrea	2040	43.5 (34.9 to 53.7)	8.9 (6.5 to 11.6)	24.9 (17.3 to 34.6)	0.9 (0.4 to 1.6)	8.9 (6.8 to 12.3)	66.0 (53.0 to 81.6)	13.5 (9.8 to 17.6)	37.8 (26.2 to 52.6)	1.3 (0.6 to 2.4)	13.5 (10.3 to 18.6)
Eritrea	2041	44.2 (35.2 to 54.4)	9.1 (6.6 to 11.8)	25.2 (17.5 to 35.0)	0.9 (0.4 to 1.6)	9.1 (6.8 to 12.6)	67.1 (53.5 to 82.6)	13.8 (10.1 to 17.9)	38.2 (26.6 to 53.2)	1.3 (0.6 to 2.5)	13.7 (10.3 to 19.1)
Eritrea	2042	44.9 (35.9 to 55.5)	9.3 (6.8 to 12.0)	25.5 (17.7 to 35.4)	0.9 (0.4 to 1.7)	9.2 (6.9 to 13.2)	68.1 (54.6 to 84.3)	14.1 (10.3 to 18.3)	38.7 (26.9 to 53.8)	1.4 (0.6 to 2.5)	14.0 (10.5 to 20.0)
Eritrea	2043	45.6 (36.8 to 56.4)	9.5 (6.9 to 12.3)	25.8 (17.9 to 35.8)	0.9 (0.4 to 1.7)	9.4 (7.0 to 13.6)	69.2 (55.9 to 85.7)	14.4 (10.5 to 18.7)	39.1 (27.2 to 54.4)	1.4 (0.6 to 2.6)	14.3 (10.6 to 20.7)

		2018 US Dollars per capita					2018 PPP per capita				
Country	Year	Health spending	Government health spending	Out-of-pocket health spending	Prepaid private health spending	Development assistance for health	Health spending	Government health spending	Out-of-pocket health spending	Prepaid private health spending	Development assistance for health
Eritrea	2044	46.3 (37.3 to 58.0)	9.7 (7.0 to 12.6)	26.0 (18.1 to 36.1)	0.9 (0.4 to 1.7)	9.6 (7.1 to 13.9)	70.3 (56.7 to 88.1)	14.8 (10.7 to 19.2)	39.5 (27.5 to 54.8)	1.4 (0.7 to 2.6)	14.6 (10.8 to 21.1)
Eritrea	2045	47.0 (38.1 to 58.5)	10.0 (7.3 to 13.0)	26.3 (18.3 to 36.5)	0.9 (0.4 to 1.8)	9.8 (7.1 to 14.8)	71.4 (57.8 to 88.8)	15.1 (11.0 to 19.7)	40.0 (27.7 to 55.5)	1.4 (0.7 to 2.7)	14.9 (10.7 to 22.5)
Eritrea	2046	47.8 (38.1 to 59.4)	10.2 (7.5 to 13.3)	26.6 (18.4 to 36.9)	1.0 (0.4 to 1.8)	10.0 (7.2 to 15.4)	72.6 (57.8 to 90.2)	15.5 (11.4 to 20.2)	40.4 (28.0 to 56.0)	1.5 (0.7 to 2.8)	15.2 (11.0 to 23.4)
Eritrea	2047	48.6 (38.5 to 60.3)	10.5 (7.7 to 13.7)	26.9 (18.6 to 37.3)	1.0 (0.5 to 1.9)	10.2 (7.4 to 16.1)	73.7 (58.5 to 91.5)	15.9 (11.7 to 20.8)	40.8 (28.3 to 56.6)	1.5 (0.7 to 2.8)	15.6 (11.2 to 24.5)
Eritrea	2048	49.4 (39.3 to 61.9)	10.7 (7.9 to 14.0)	27.1 (18.8 to 37.6)	1.0 (0.5 to 1.9)	10.5 (7.5 to 16.8)	75.0 (59.7 to 94.0)	16.3 (11.9 to 21.3)	41.2 (28.5 to 57.1)	1.5 (0.7 to 2.9)	15.9 (11.4 to 25.5)
Eritrea	2049	50.1 (39.6 to 62.4)	11.0 (8.0 to 14.4)	27.4 (18.9 to 38.0)	1.0 (0.5 to 1.9)	10.7 (7.7 to 17.2)	76.1 (60.1 to 94.7)	16.7 (12.2 to 21.8)	41.6 (28.8 to 57.7)	1.6 (0.7 to 2.9)	16.3 (11.6 to 26.2)
Eritrea	2050	50.9 (41.4 to 62.9)	11.2 (8.2 to 14.7)	27.7 (19.1 to 38.3)	1.0 (0.5 to 2.0)	11.0 (7.7 to 18.0)	77.3 (62.8 to 95.6)	17.0 (12.5 to 22.3)	42.0 (29.0 to 58.2)	1.6 (0.7 to 3.0)	16.7 (11.7 to 27.3)
Estonia	1995	577.1 (535.1 to 624.5)	495.2 (455.4 to 538.4)	61.4 (49.7 to 74.5)	20.5 (11.1 to 34.5)	0.0 (0.0 to 0.0)	850.4 (788.4 to 920.2)	729.7 (671.0 to 793.3)	90.5 (73.3 to 109.8)	30.2 (16.4 to 50.8)	0.0 (0.0 to 0.0)
Estonia	1996	594.3 (556.2 to 632.4)	506.3 (473.4 to 541.5)	67.2 (55.9 to 80.3)	20.7 (11.4 to 34.8)	0.0 (0.0 to 0.0)	875.6 (819.5 to 931.9)	746.1 (697.5 to 797.8)	99.0 (82.3 to 118.4)	30.5 (16.9 to 51.3)	0.1 (0.1 to 0.1)
Estonia	1997	628.2 (591.5 to 664.7)	532.6 (500.7 to 565.0)	74.0 (63.1 to 86.6)	21.5 (12.1 to 35.3)	0.1 (0.1 to 0.1)	925.7 (871.6 to 979.4)	784.7 (737.7 to 832.5)	109.1 (93.0 to 127.6)	31.7 (17.8 to 52.0)	0.1 (0.1 to 0.1)
Estonia	1998	621.8 (587.7 to 658.1)	517.0 (486.6 to 550.5)	83.1 (71.9 to 95.6)	21.3 (12.1 to 34.7)	0.4 (0.4 to 0.4)	916.3 (865.9 to 969.8)	761.8 (717.0 to 811.2)	122.4 (105.9 to 140.9)	31.4 (17.8 to 51.1)	0.6 (0.6 to 0.6)
Estonia	1999	595.4 (562.1 to 629.3)	480.6 (452.2 to 511.6)	92.9 (80.9 to 106.8)	20.5 (11.6 to 32.4)	1.5 (1.5 to 1.5)	877.4 (828.3 to 927.3)	708.2 (666.4 to 753.8)	136.9 (119.3 to 157.3)	30.1 (17.1 to 47.7)	2.1 (2.1 to 2.1)
Estonia	2000	608.2 (575.3 to 641.6)	477.8 (450.3 to 507.4)	109.6 (95.9 to 124.4)	20.7 (11.7 to 32.7)	0.1 (0.1 to 0.1)	896.2 (847.7 to 945.4)	704.0 (663.5 to 747.6)	161.5 (141.4 to 183.4)	30.5 (17.2 to 48.1)	0.2 (0.2 to 0.2)
Estonia	2001	618.5 (585.9 to 652.4)	479.2 (452.1 to 509.2)	119.7 (104.9 to 135.5)	19.5 (11.3 to 31.0)	0.0 (0.0 to 0.0)	911.3 (863.4 to 961.3)	706.1 (666.1 to 750.2)	176.4 (154.5 to 199.7)	28.8 (16.6 to 45.7)	0.0 (0.0 to 0.0)
Estonia	2002	650.2 (616.1 to 686.0)	496.0 (468.4 to 526.0)	133.7 (118.4 to 151.5)	20.4 (11.7 to 32.0)	0.0 (0.0 to 0.0)	958.1 (907.8 to 1010.8)	730.9 (690.2 to 775.1)	197.1 (174.4 to 223.3)	30.1 (17.3 to 47.1)	0.0 (0.0 to 0.0)
Estonia	2003	705.3 (669.5 to 739.8)	532.5 (504.2 to 560.5)	150.5 (134.2 to 168.6)	21.3 (12.2 to 33.9)	1.0 (1.0 to 1.0)	1039.2 (986.4 to 1090.0)	784.6 (743.0 to 825.9)	221.7 (197.8 to 248.5)	31.5 (17.9 to 49.9)	1.4 (1.4 to 1.4)
Estonia	2004	767.8 (731.7 to 802.6)	574.0 (543.9 to 603.5)	169.5 (152.7 to 187.2)	22.6 (13.1 to 35.8)	1.7 (1.7 to 1.7)	1131.3 (1078.2 to 1182.6)	845.7 (801.5 to 889.3)	249.7 (225.0 to 275.8)	33.3 (19.3 to 52.8)	2.5 (2.5 to 2.5)
Estonia	2005	843.1 (807.4 to 879.4)	629.3 (599.1 to 660.8)	188.9 (172.4 to 206.8)	22.5 (13.0 to 34.9)	2.3 (2.3 to 2.3)	1242.2 (1189.7 to 1295.8)	927.3 (882.7 to 973.6)	278.4 (254.0 to 304.7)	33.2 (19.2 to 51.5)	3.4 (3.4 to 3.4)
Estonia	2006	939.3 (903.9 to 976.6)	703.0 (672.8 to 735.3)	215.9 (198.8 to 234.3)	20.3 (11.9 to 31.2)	0.0 (0.0 to 0.0)	1384.0 (1331.8 to 1439.0)	1035.9 (991.4 to 1083.5)	318.1 (292.9 to 345.2)	30.0 (17.5 to 45.9)	0.0 (0.0 to 0.0)
Estonia	2007	1061.6 (1023.7 to 1100.5)	805.8 (773.0 to 838.8)	233.4 (216.9 to 251.2)	22.4 (13.3 to 33.9)	0.0 (0.0 to 0.0)	1564.3 (1508.5 to 1621.5)	1187.3 (1139.0 to 1236.0)	344.0 (319.6 to 370.2)	33.0 (19.7 to 50.0)	0.0 (0.0 to 0.0)
Estonia	2008	1107.6 (1067.8 to 1146.2)	845.3 (811.5 to 877.9)	237.2 (220.9 to 255.4)	25.0 (15.3 to 37.2)	0.0 (0.0 to 0.0)	1632.0 (1573.4 to 1688.9)	1245.6 (1195.7 to 1293.5)	349.6 (325.5 to 376.3)	36.9 (22.6 to 54.8)	0.0 (0.0 to 0.0)

Country	Year	2018 US Dollars per capita					2018 PPP per capita				
		Health spending	Government health spending	Out-of-pocket health spending	Prepaid private health spending	Development assistance for health	Health spending	Government health spending	Out-of-pocket health spending	Prepaid private health spending	Development assistance for health
Estonia	2009	1041.0 (1006.2 to 1077.6)	792.1 (762.8 to 822.5)	225.9 (210.3 to 243.7)	23.0 (14.4 to 33.7)	0.0 (0.0 to 0.0)	1533.9 (1482.5 to 1587.8)	1167.1 (1123.9 to 1212.0)	332.9 (309.8 to 359.1)	33.9 (21.2 to 49.7)	0.0 (0.0 to 0.0)
Estonia	2010	1049.1 (1016.0 to 1085.0)	797.7 (768.6 to 829.1)	229.2 (213.5 to 246.1)	22.3 (14.1 to 32.8)	0.0 (0.0 to 0.0)	1545.9 (1497.0 to 1598.8)	1175.3 (1132.5 to 1221.7)	337.8 (314.6 to 362.7)	32.8 (20.7 to 48.3)	0.0 (0.0 to 0.0)
Estonia	2011	1090.1 (1054.7 to 1126.5)	831.5 (801.2 to 861.2)	236.3 (220.6 to 253.1)	22.3 (14.2 to 33.3)	0.0 (0.0 to 0.0)	1606.3 (1554.1 to 1659.9)	1225.2 (1180.6 to 1269.0)	348.2 (325.1 to 372.9)	32.9 (21.0 to 49.1)	0.0 (0.0 to 0.0)
Estonia	2012	1134.8 (1096.0 to 1172.0)	864.9 (832.5 to 896.9)	247.6 (232.0 to 264.3)	22.3 (14.1 to 34.6)	0.0 (0.0 to 0.0)	1672.1 (1615.0 to 1727.0)	1274.4 (1226.7 to 1321.6)	364.8 (341.9 to 389.4)	32.9 (20.8 to 51.0)	0.0 (0.0 to 0.0)
Estonia	2013	1180.6 (1143.0 to 1222.8)	894.8 (863.8 to 928.1)	263.4 (246.5 to 281.7)	22.4 (14.1 to 34.6)	0.0 (0.0 to 0.0)	1739.5 (1684.2 to 1801.8)	1318.4 (1272.8 to 1367.5)	388.1 (363.2 to 415.1)	33.0 (20.7 to 50.9)	0.0 (0.0 to 0.0)
Estonia	2014	1253.3 (1215.9 to 1294.6)	947.6 (915.5 to 980.3)	282.6 (263.6 to 301.6)	23.1 (14.3 to 36.4)	0.0 (0.0 to 0.0)	1846.7 (1791.6 to 1907.6)	1396.3 (1349.0 to 1444.4)	416.4 (388.4 to 444.4)	34.0 (21.1 to 53.6)	0.0 (0.0 to 0.0)
Estonia	2015	1328.0 (1285.7 to 1368.8)	1002.8 (967.9 to 1037.5)	301.4 (279.5 to 323.8)	23.8 (14.1 to 38.0)	0.0 (0.0 to 0.0)	1956.8 (1894.5 to 2016.9)	1477.6 (1426.1 to 1528.7)	444.2 (411.9 to 477.1)	35.0 (20.7 to 56.0)	0.0 (0.0 to 0.0)
Estonia	2016	1392.0 (1338.1 to 1450.6)	1050.8 (1006.9 to 1097.9)	316.5 (288.4 to 346.9)	24.7 (14.0 to 40.0)	0.0 (0.0 to 0.0)	2051.0 (1971.6 to 2137.4)	1548.3 (1483.7 to 1617.7)	466.3 (425.0 to 511.1)	36.4 (20.7 to 58.9)	0.0 (0.0 to 0.0)
Estonia	2017	1448.7 (1391.1 to 1510.7)	1091.4 (1045.2 to 1140.8)	331.5 (301.4 to 363.3)	25.8 (14.6 to 41.6)	0.0 (0.0 to 0.0)	2134.6 (2049.8 to 2226.0)	1608.1 (1540.1 to 1681.0)	488.5 (444.1 to 535.4)	38.0 (21.6 to 61.3)	0.0 (0.0 to 0.0)
Estonia	2018	1470.2 (1408.0 to 1535.8)	1101.6 (1055.5 to 1152.2)	342.5 (311.3 to 376.6)	26.2 (14.9 to 42.1)	0.0 (0.0 to 0.0)	2166.4 (2074.6 to 2263.0)	1623.2 (1555.2 to 1697.7)	504.6 (458.6 to 554.9)	38.6 (21.9 to 62.0)	0.0 (0.0 to 0.0)
Estonia	2019	1490.5 (1429.9 to 1556.8)	1110.9 (1064.2 to 1161.9)	353.1 (319.2 to 388.8)	26.5 (15.1 to 42.4)	0.0 (0.0 to 0.0)	2196.2 (2106.9 to 2293.9)	1636.9 (1568.1 to 1712.0)	520.2 (470.3 to 573.0)	39.1 (22.3 to 62.5)	0.0 (0.0 to 0.0)
Estonia	2020	1514.1 (1450.2 to 1581.8)	1123.0 (1075.4 to 1174.7)	364.2 (328.3 to 401.6)	26.9 (15.4 to 42.9)	0.0 (0.0 to 0.0)	2231.0 (2136.8 to 2330.7)	1654.7 (1584.5 to 1730.8)	536.6 (483.7 to 591.7)	39.7 (22.6 to 63.1)	0.0 (0.0 to 0.0)
Estonia	2021	1539.1 (1471.7 to 1607.9)	1136.9 (1087.5 to 1189.8)	374.9 (338.1 to 415.5)	27.3 (15.6 to 43.4)	0.0 (0.0 to 0.0)	2267.9 (2168.5 to 2369.2)	1675.1 (1602.5 to 1753.1)	552.5 (498.1 to 612.3)	40.3 (23.0 to 64.0)	0.0 (0.0 to 0.0)
Estonia	2022	1570.1 (1501.9 to 1643.2)	1157.4 (1107.7 to 1212.6)	384.9 (346.9 to 426.8)	27.8 (15.9 to 44.4)	0.0 (0.0 to 0.0)	2313.4 (2213.1 to 2421.2)	1705.4 (1632.1 to 1786.7)	567.1 (511.1 to 628.8)	41.0 (23.4 to 65.4)	0.0 (0.0 to 0.0)
Estonia	2023	1609.2 (1539.2 to 1682.8)	1183.0 (1131.3 to 1240.3)	397.8 (358.1 to 441.2)	28.4 (16.2 to 45.4)	0.0 (0.0 to 0.0)	2371.2 (2268.0 to 2479.6)	1743.1 (1667.0 to 1827.5)	586.2 (527.7 to 650.0)	41.9 (23.9 to 66.9)	0.0 (0.0 to 0.0)
Estonia	2024	1650.4 (1576.4 to 1728.1)	1210.0 (1157.7 to 1269.9)	411.3 (370.2 to 458.0)	29.1 (16.6 to 46.4)	0.0 (0.0 to 0.0)	2431.9 (2322.7 to 2546.3)	1782.9 (1705.9 to 1871.1)	606.1 (545.5 to 674.8)	42.8 (24.5 to 68.4)	0.0 (0.0 to 0.0)
Estonia	2025	1693.2 (1616.0 to 1774.7)	1238.0 (1183.1 to 1301.1)	425.4 (381.6 to 474.1)	29.7 (16.9 to 47.4)	0.0 (0.0 to 0.0)	2494.9 (2381.2 to 2614.9)	1824.2 (1743.2 to 1917.2)	626.9 (562.3 to 698.6)	43.8 (24.9 to 69.9)	0.0 (0.0 to 0.0)
Estonia	2026	1738.3 (1661.0 to 1827.9)	1267.7 (1211.2 to 1332.9)	440.1 (394.4 to 490.7)	30.4 (17.3 to 48.5)	0.0 (0.0 to 0.0)	2561.3 (2447.4 to 2693.3)	1867.9 (1784.7 to 1964.0)	648.5 (581.2 to 723.0)	44.8 (25.5 to 71.4)	0.0 (0.0 to 0.0)
Estonia	2027	1785.4 (1697.9 to 1875.0)	1298.7 (1239.4 to 1366.5)	455.5 (406.8 to 512.3)	31.2 (17.8 to 49.7)	0.0 (0.0 to 0.0)	2630.7 (2501.7 to 2762.8)	1913.6 (1826.2 to 2013.5)	671.2 (599.5 to 754.9)	45.9 (26.2 to 73.2)	0.0 (0.0 to 0.0)
Estonia	2028	1834.4 (1742.5 to 1932.6)	1331.2 (1267.2 to 1402.4)	471.2 (418.2 to 532.1)	32.0 (18.2 to 50.8)	0.0 (0.0 to 0.0)	2703.0 (2567.5 to 2847.7)	1961.6 (1867.1 to 2066.4)	694.4 (616.3 to 784.1)	47.1 (26.9 to 74.9)	0.0 (0.0 to 0.0)
Estonia	2029	1883.0 (1783.8 to 1986.2)	1363.3 (1293.5 to 1439.8)	487.0 (428.2 to 554.5)	32.8 (18.7 to 52.0)	0.0 (0.0 to 0.0)	2774.5 (2628.4 to 2926.7)	2008.7 (1905.9 to 2121.4)	717.5 (630.9 to 817.1)	48.3 (27.6 to 76.6)	0.0 (0.0 to 0.0)

Country	Year	2018 US Dollars per capita					2018 PPP per capita				
		Health spending	Government health spending	Out-of-pocket health spending	Prepaid private health spending	Development assistance for health	Health spending	Government health spending	Out-of-pocket health spending	Prepaid private health spending	Development assistance for health
Estonia	2030	1932.0 (1824.6 to 2038.1)	1395.7 (1322.5 to 1475.3)	502.8 (438.0 to 576.8)	33.6 (19.2 to 53.3)	0.0 (0.0 to 0.0)	2846.8 (2688.5 to 3003.1)	2056.5 (1948.7 to 2173.8)	740.9 (645.3 to 849.9)	49.5 (28.4 to 78.5)	0.0 (0.0 to 0.0)
Estonia	2031	1981.9 (1868.6 to 2101.5)	1428.7 (1349.9 to 1515.2)	518.8 (449.7 to 598.2)	34.4 (19.8 to 54.5)	0.0 (0.0 to 0.0)	2920.3 (2753.4 to 3096.6)	2105.1 (1989.0 to 2232.6)	764.4 (662.6 to 881.4)	50.8 (29.1 to 80.4)	0.0 (0.0 to 0.0)
Estonia	2032	2031.2 (1908.4 to 2152.3)	1461.1 (1377.2 to 1553.2)	534.7 (459.7 to 621.8)	35.3 (20.2 to 56.0)	0.0 (0.0 to 0.0)	2992.9 (2811.9 to 3171.3)	2152.9 (2029.3 to 2288.6)	787.9 (677.4 to 916.2)	52.0 (29.8 to 82.5)	0.0 (0.0 to 0.0)
Estonia	2033	2080.5 (1950.7 to 2216.5)	1493.7 (1405.5 to 1589.2)	550.6 (468.7 to 644.3)	36.2 (20.7 to 57.5)	0.0 (0.0 to 0.0)	3065.6 (2874.3 to 3266.0)	2200.9 (2071.0 to 2341.7)	811.3 (690.6 to 949.3)	53.4 (30.6 to 84.8)	0.0 (0.0 to 0.0)
Estonia	2034	2130.4 (1993.6 to 2275.0)	1526.8 (1433.4 to 1624.5)	566.5 (477.6 to 668.4)	37.1 (21.3 to 59.1)	0.0 (0.0 to 0.0)	3139.1 (2937.6 to 3352.1)	2249.7 (2112.1 to 2393.7)	834.7 (703.8 to 984.8)	54.7 (31.4 to 87.1)	0.0 (0.0 to 0.0)
Estonia	2035	2180.9 (2033.5 to 2333.9)	1560.7 (1463.2 to 1664.8)	582.2 (487.3 to 692.1)	38.0 (21.8 to 60.6)	0.0 (0.0 to 0.0)	3213.5 (2996.3 to 3438.9)	2299.7 (2156.0 to 2453.0)	857.8 (718.1 to 1019.8)	56.0 (32.2 to 89.3)	0.0 (0.0 to 0.0)
Estonia	2036	2232.3 (2075.6 to 2393.5)	1595.7 (1491.7 to 1705.2)	597.7 (497.0 to 715.6)	38.9 (22.4 to 62.1)	0.0 (0.0 to 0.0)	3289.3 (3058.4 to 3526.7)	2351.2 (2198.0 to 2512.6)	880.7 (732.3 to 1054.4)	57.3 (33.0 to 91.4)	0.0 (0.0 to 0.0)
Estonia	2037	2284.3 (2120.1 to 2455.2)	1631.5 (1517.1 to 1752.2)	613.1 (506.5 to 738.7)	39.8 (22.9 to 63.4)	0.0 (0.0 to 0.0)	3365.9 (3123.9 to 3617.6)	2404.0 (2235.4 to 2581.8)	903.3 (746.4 to 1088.4)	58.7 (33.8 to 93.4)	0.0 (0.0 to 0.0)
Estonia	2038	2337.9 (2166.0 to 2525.4)	1668.8 (1548.2 to 1795.1)	628.3 (515.9 to 761.7)	40.7 (23.5 to 65.0)	0.0 (0.0 to 0.0)	3444.8 (3191.5 to 3721.1)	2458.9 (2281.3 to 2645.1)	925.9 (760.2 to 1122.4)	60.0 (34.6 to 95.8)	0.0 (0.0 to 0.0)
Estonia	2039	2392.2 (2207.5 to 2580.6)	1707.0 (1579.0 to 1846.6)	643.5 (525.2 to 783.6)	41.7 (24.1 to 66.5)	0.0 (0.0 to 0.0)	3524.9 (3252.7 to 3802.5)	2515.3 (2326.7 to 2721.0)	948.2 (773.8 to 1154.6)	61.4 (35.4 to 98.0)	0.0 (0.0 to 0.0)
Estonia	2040	2446.6 (2259.6 to 2644.8)	1745.5 (1606.9 to 1892.6)	658.4 (533.6 to 805.5)	42.7 (24.6 to 68.1)	0.0 (0.0 to 0.0)	3605.0 (3329.4 to 3897.0)	2572.0 (2367.7 to 2788.7)	970.1 (786.2 to 1186.8)	62.9 (36.2 to 100.3)	0.0 (0.0 to 0.0)
Estonia	2041	2501.2 (2298.0 to 2712.6)	1784.3 (1635.8 to 1941.0)	673.1 (543.0 to 827.5)	43.7 (25.1 to 69.7)	0.0 (0.0 to 0.0)	3685.4 (3386.1 to 3996.9)	2629.2 (2410.4 to 2860.1)	991.8 (800.1 to 1219.3)	64.4 (37.0 to 102.8)	0.0 (0.0 to 0.0)
Estonia	2042	2556.1 (2342.9 to 2784.9)	1823.7 (1667.1 to 1989.5)	687.6 (552.8 to 846.6)	44.8 (25.7 to 71.5)	0.0 (0.0 to 0.0)	3766.3 (3452.2 to 4103.4)	2687.1 (2456.5 to 2931.4)	1013.2 (814.5 to 1247.4)	66.0 (37.9 to 105.4)	0.0 (0.0 to 0.0)
Estonia	2043	2611.7 (2391.4 to 2858.8)	1863.9 (1695.8 to 2037.6)	701.9 (562.5 to 866.1)	45.9 (26.3 to 73.4)	0.0 (0.0 to 0.0)	3848.2 (3523.6 to 4212.4)	2746.4 (2498.7 to 3002.3)	1034.2 (828.9 to 1276.2)	67.6 (38.7 to 108.1)	0.0 (0.0 to 0.0)
Estonia	2044	2667.0 (2437.0 to 2920.8)	1904.3 (1726.2 to 2086.4)	715.7 (572.1 to 884.7)	47.0 (26.8 to 75.3)	0.0 (0.0 to 0.0)	3929.7 (3590.9 to 4303.7)	2806.0 (2543.5 to 3074.3)	1054.6 (843.0 to 1303.5)	69.2 (39.5 to 111.0)	0.0 (0.0 to 0.0)
Estonia	2045	2721.2 (2478.9 to 2965.7)	1944.2 (1759.4 to 2138.2)	728.9 (581.4 to 901.9)	48.0 (27.3 to 77.2)	0.0 (0.0 to 0.0)	4009.6 (3652.6 to 4369.9)	2864.8 (2592.4 to 3150.6)	1074.0 (856.6 to 1328.9)	70.8 (40.3 to 113.7)	0.0 (0.0 to 0.0)
Estonia	2046	2773.1 (2533.5 to 3039.5)	1982.8 (1786.9 to 2187.8)	741.3 (590.3 to 918.0)	49.1 (27.8 to 78.8)	0.0 (0.0 to 0.0)	4086.1 (3733.0 to 4478.6)	2921.6 (2632.9 to 3223.6)	1092.2 (869.7 to 1352.7)	72.3 (41.0 to 116.1)	0.0 (0.0 to 0.0)
Estonia	2047	2826.0 (2571.8 to 3107.1)	2022.5 (1818.3 to 2236.6)	753.3 (599.1 to 934.1)	50.2 (28.4 to 80.6)	0.0 (0.0 to 0.0)	4164.0 (3789.5 to 4578.2)	2980.2 (2679.2 to 3295.5)	1109.9 (882.8 to 1376.3)	73.9 (41.8 to 118.8)	0.0 (0.0 to 0.0)
Estonia	2048	2876.7 (2614.1 to 3153.2)	2060.5 (1844.1 to 2285.0)	765.1 (607.8 to 949.9)	51.2 (28.9 to 82.4)	0.0 (0.0 to 0.0)	4238.8 (3851.9 to 4646.2)	3036.1 (2717.3 to 3367.0)	1127.3 (895.6 to 1399.6)	75.4 (42.6 to 121.4)	0.0 (0.0 to 0.0)
Estonia	2049	2926.2 (2658.5 to 3219.9)	2097.5 (1872.3 to 2333.8)	776.5 (616.6 to 965.2)	52.2 (29.5 to 84.1)	0.0 (0.0 to 0.0)	4311.7 (3917.2 to 4744.5)	3090.6 (2758.8 to 3438.8)	1144.2 (908.5 to 1422.3)	76.9 (43.4 to 123.9)	0.0 (0.0 to 0.0)
Estonia	2050	2975.4 (2693.5 to 3280.3)	2134.4 (1902.6 to 2379.0)	787.9 (624.8 to 980.3)	53.1 (30.1 to 85.7)	0.0 (0.0 to 0.0)	4384.2 (3968.8 to 4833.5)	3145.0 (2803.4 to 3505.3)	1160.9 (920.7 to 1444.4)	78.3 (44.3 to 126.3)	0.0 (0.0 to 0.0)

		2018 US Dollars per capita					2018 PPP per capita				
Country	Year	Health spending	Government health spending	Out-of-pocket health spending	Prepaid private health spending	Development assistance for health	Health spending	Government health spending	Out-of-pocket health spending	Prepaid private health spending	Development assistance for health
Ethiopia	1995	9.4 (7.8 to 11.2)	4.0 (3.0 to 5.2)	3.4 (2.4 to 4.7)	0.7 (0.3 to 1.2)	1.2 (1.2 to 1.2)	25.1 (20.9 to 30.0)	10.8 (8.1 to 14.1)	9.2 (6.4 to 12.7)	1.8 (0.8 to 3.3)	3.3 (3.3 to 3.3)
Ethiopia	1996	10.1 (8.5 to 11.9)	4.2 (3.1 to 5.4)	3.6 (2.5 to 5.0)	0.7 (0.3 to 1.2)	1.6 (1.6 to 1.6)	27.0 (22.8 to 31.9)	11.2 (8.4 to 14.5)	9.8 (6.7 to 13.3)	1.8 (0.8 to 3.3)	4.3 (4.3 to 4.3)
Ethiopia	1997	10.0 (8.4 to 11.8)	4.3 (3.2 to 5.6)	3.8 (2.6 to 5.2)	0.7 (0.3 to 1.3)	1.3 (1.3 to 1.3)	26.8 (22.5 to 31.6)	11.4 (8.5 to 14.9)	10.2 (7.0 to 14.1)	1.8 (0.8 to 3.4)	3.4 (3.4 to 3.4)
Ethiopia	1998	9.7 (8.1 to 11.5)	4.1 (3.0 to 5.3)	3.8 (2.6 to 5.2)	0.7 (0.3 to 1.3)	1.1 (1.1 to 1.1)	26.1 (21.7 to 31.0)	11.0 (8.1 to 14.3)	10.2 (7.1 to 14.1)	1.9 (0.8 to 3.5)	3.0 (3.0 to 3.0)
Ethiopia	1999	10.7 (9.0 to 12.6)	4.2 (3.2 to 5.5)	3.9 (2.7 to 5.4)	0.8 (0.3 to 1.5)	1.8 (1.8 to 1.8)	28.8 (24.1 to 33.9)	11.4 (8.5 to 14.8)	10.6 (7.3 to 14.6)	2.0 (0.9 to 3.9)	4.7 (4.7 to 4.7)
Ethiopia	2000	10.9 (9.1 to 12.8)	4.5 (3.4 to 5.8)	4.0 (2.8 to 5.6)	0.8 (0.4 to 1.4)	1.6 (1.6 to 1.6)	29.2 (24.4 to 34.4)	12.1 (9.0 to 15.6)	10.8 (7.5 to 15.0)	2.0 (0.9 to 3.9)	4.3 (4.3 to 4.3)
Ethiopia	2001	12.2 (10.2 to 14.3)	4.9 (3.7 to 6.4)	4.3 (3.0 to 5.9)	0.8 (0.4 to 1.5)	2.2 (2.2 to 2.2)	32.7 (27.5 to 38.5)	13.3 (9.9 to 17.1)	11.4 (8.0 to 15.8)	2.1 (0.9 to 4.0)	5.9 (5.9 to 5.9)
Ethiopia	2002	11.7 (9.8 to 13.9)	4.8 (3.6 to 6.2)	4.3 (3.0 to 6.0)	0.7 (0.3 to 1.4)	1.9 (1.9 to 1.9)	31.5 (26.4 to 37.3)	13.0 (9.7 to 16.7)	11.5 (8.1 to 16.0)	2.0 (0.9 to 3.7)	5.0 (5.0 to 5.0)
Ethiopia	2003	12.4 (10.6 to 14.4)	4.5 (3.4 to 5.7)	4.1 (2.9 to 5.6)	0.6 (0.3 to 1.2)	3.2 (3.2 to 3.2)	33.3 (28.4 to 38.8)	12.0 (9.1 to 15.3)	11.1 (7.9 to 15.0)	1.7 (0.8 to 3.3)	8.5 (8.5 to 8.5)
Ethiopia	2004	12.1 (10.3 to 14.1)	4.2 (3.1 to 5.4)	4.2 (2.9 to 5.7)	0.6 (0.3 to 1.1)	3.1 (3.1 to 3.1)	32.6 (27.7 to 37.9)	11.2 (8.5 to 14.4)	11.3 (7.9 to 15.4)	1.6 (0.7 to 3.0)	8.4 (8.4 to 8.4)
Ethiopia	2005	13.0 (11.1 to 15.1)	4.2 (3.2 to 5.5)	4.4 (3.0 to 6.1)	0.6 (0.3 to 1.1)	3.7 (3.7 to 3.7)	35.0 (29.8 to 40.5)	11.4 (8.6 to 14.6)	11.9 (8.1 to 16.3)	1.6 (0.8 to 3.0)	10.0 (10.0 to 10.0)
Ethiopia	2006	15.1 (13.2 to 17.3)	3.8 (2.9 to 4.9)	4.9 (3.3 to 6.8)	0.6 (0.3 to 1.1)	5.8 (5.8 to 5.8)	40.6 (35.4 to 46.5)	10.3 (7.7 to 13.2)	13.2 (8.9 to 18.2)	1.6 (0.7 to 3.0)	15.5 (15.5 to 15.5)
Ethiopia	2007	17.3 (15.0 to 19.7)	3.7 (2.8 to 4.8)	5.6 (3.8 to 7.8)	0.6 (0.3 to 1.2)	7.3 (7.3 to 7.3)	46.4 (40.4 to 53.0)	10.0 (7.6 to 12.9)	15.1 (10.2 to 21.1)	1.7 (0.8 to 3.1)	19.7 (19.7 to 19.7)
Ethiopia	2008	17.3 (15.1 to 20.1)	3.2 (2.4 to 4.1)	6.1 (4.2 to 8.5)	0.6 (0.3 to 1.2)	7.4 (7.4 to 7.4)	46.6 (40.5 to 54.0)	8.5 (6.4 to 11.0)	16.5 (11.3 to 22.8)	1.7 (0.8 to 3.2)	19.9 (19.9 to 19.9)
Ethiopia	2009	18.5 (15.9 to 21.5)	3.3 (2.5 to 4.3)	7.1 (4.8 to 9.8)	0.9 (0.4 to 1.7)	7.2 (7.2 to 7.2)	49.7 (42.8 to 57.7)	9.0 (6.7 to 11.6)	19.0 (12.9 to 26.2)	2.5 (1.2 to 4.7)	19.3 (19.3 to 19.3)
Ethiopia	2010	22.2 (19.3 to 25.7)	3.5 (2.6 to 4.5)	8.4 (5.7 to 11.6)	1.2 (0.6 to 2.3)	9.1 (9.1 to 9.1)	59.6 (51.7 to 69.1)	9.4 (7.0 to 12.2)	22.4 (15.4 to 31.1)	3.3 (1.6 to 6.2)	24.6 (24.6 to 24.6)
Ethiopia	2011	24.0 (20.9 to 27.6)	3.4 (2.5 to 4.4)	8.8 (6.1 to 12.1)	1.4 (0.7 to 2.6)	10.5 (10.5 to 10.5)	64.4 (56.1 to 74.3)	9.0 (6.7 to 11.8)	23.5 (16.4 to 32.5)	3.7 (1.8 to 7.1)	28.1 (28.1 to 28.1)
Ethiopia	2012	24.7 (21.2 to 28.6)	4.3 (3.3 to 5.7)	9.3 (6.5 to 12.9)	1.8 (0.9 to 3.4)	9.2 (9.2 to 9.2)	66.3 (57.1 to 76.9)	11.6 (8.8 to 15.2)	25.0 (17.5 to 34.8)	4.8 (2.4 to 9.2)	24.8 (24.8 to 24.8)
Ethiopia	2013	28.8 (25.1 to 33.0)	5.0 (3.8 to 6.5)	9.5 (6.6 to 13.3)	2.3 (1.1 to 4.4)	12.0 (12.0 to 12.0)	77.3 (67.5 to 88.6)	13.4 (10.1 to 17.5)	25.5 (17.7 to 35.7)	6.3 (3.0 to 11.7)	32.2 (32.2 to 32.2)
Ethiopia	2014	27.1 (23.2 to 32.0)	5.2 (3.9 to 6.7)	9.5 (6.6 to 13.2)	3.4 (1.6 to 6.3)	9.0 (9.0 to 9.0)	72.9 (62.4 to 86.0)	14.0 (10.6 to 18.1)	25.6 (17.8 to 35.5)	9.1 (4.3 to 17.0)	24.3 (24.3 to 24.3)
Ethiopia	2015	29.0 (24.4 to 34.4)	6.2 (4.7 to 8.0)	10.1 (7.0 to 14.1)	4.5 (2.1 to 8.3)	8.2 (8.2 to 8.2)	77.8 (65.5 to 92.3)	16.6 (12.6 to 21.6)	27.2 (18.9 to 37.9)	12.1 (5.6 to 22.3)	22.0 (22.0 to 22.0)

Country	Year	2018 US Dollars per capita					2018 PPP per capita				
		Health spending	Government health spending	Out-of-pocket health spending	Prepaid private health spending	Development assistance for health	Health spending	Government health spending	Out-of-pocket health spending	Prepaid private health spending	Development assistance for health
Ethiopia	2016	30.7 (26.1 to 36.9)	6.9 (5.2 to 9.0)	10.5 (7.4 to 14.8)	5.2 (2.4 to 9.7)	8.0 (8.0 to 8.0)	82.5 (70.0 to 99.1)	18.6 (14.1 to 24.2)	28.3 (19.8 to 39.7)	14.1 (6.6 to 26.1)	21.5 (21.5 to 21.5)
Ethiopia	2017	33.0 (27.8 to 39.7)	7.6 (5.7 to 9.9)	11.4 (8.0 to 16.0)	5.6 (2.6 to 10.4)	8.4 (8.4 to 8.4)	88.7 (74.8 to 106.6)	20.4 (15.4 to 26.5)	30.6 (21.4 to 42.9)	15.1 (7.1 to 28.1)	22.6 (22.6 to 22.6)
Ethiopia	2018	33.5 (28.3 to 40.5)	8.0 (6.0 to 10.4)	11.8 (8.3 to 16.6)	5.9 (2.8 to 11.0)	7.7 (7.7 to 7.7)	90.0 (76.0 to 108.8)	21.5 (16.2 to 28.1)	31.8 (22.2 to 44.7)	15.9 (7.4 to 29.4)	20.7 (20.7 to 20.8)
Ethiopia	2019	34.2 (28.5 to 41.1)	8.3 (6.3 to 10.9)	12.1 (8.5 to 17.0)	6.1 (2.9 to 11.3)	7.6 (7.1 to 8.1)	91.8 (76.6 to 110.4)	22.4 (16.8 to 29.2)	32.6 (22.8 to 45.7)	16.4 (7.7 to 30.4)	20.4 (19.1 to 21.6)
Ethiopia	2020	34.9 (29.2 to 42.5)	8.6 (6.5 to 11.3)	12.4 (8.7 to 17.4)	6.3 (3.0 to 11.7)	7.5 (6.9 to 8.1)	93.8 (78.4 to 114.2)	23.2 (17.5 to 30.2)	33.4 (23.3 to 46.8)	17.0 (7.9 to 31.5)	20.2 (18.6 to 21.8)
Ethiopia	2021	35.7 (29.4 to 43.2)	9.0 (6.7 to 11.7)	12.7 (8.9 to 17.8)	6.5 (3.0 to 12.1)	7.5 (6.7 to 8.3)	95.8 (79.1 to 115.9)	24.1 (18.1 to 31.3)	34.1 (23.8 to 47.9)	17.5 (8.2 to 32.4)	20.1 (18.1 to 22.2)
Ethiopia	2022	36.4 (30.3 to 44.4)	9.3 (7.0 to 12.0)	13.0 (9.1 to 18.2)	6.7 (3.1 to 12.4)	7.4 (6.6 to 8.3)	97.8 (81.4 to 119.3)	24.9 (18.7 to 32.4)	34.9 (24.4 to 48.9)	18.0 (8.4 to 33.4)	19.9 (17.6 to 22.3)
Ethiopia	2023	37.1 (30.9 to 45.2)	9.6 (7.2 to 12.4)	13.3 (9.3 to 18.6)	6.9 (3.2 to 12.8)	7.4 (6.4 to 8.4)	99.7 (82.9 to 121.5)	25.7 (19.3 to 33.4)	35.7 (24.9 to 50.0)	18.6 (8.7 to 34.4)	19.8 (17.1 to 22.5)
Ethiopia	2024	37.9 (31.3 to 46.4)	9.9 (7.4 to 12.8)	13.6 (9.5 to 19.0)	7.1 (3.3 to 13.2)	7.3 (6.3 to 8.5)	101.8 (84.1 to 124.6)	26.5 (20.0 to 34.4)	36.5 (25.4 to 51.0)	19.2 (8.9 to 35.5)	19.6 (16.9 to 22.9)
Ethiopia	2025	38.7 (32.2 to 47.1)	10.2 (7.7 to 13.2)	13.9 (9.7 to 19.4)	7.4 (3.4 to 13.6)	7.3 (6.2 to 8.6)	103.9 (86.5 to 126.6)	27.3 (20.6 to 35.5)	37.3 (26.0 to 52.1)	19.8 (9.2 to 36.5)	19.6 (16.6 to 23.0)
Ethiopia	2026	39.4 (32.6 to 48.3)	10.5 (7.9 to 13.6)	14.1 (9.8 to 19.8)	7.6 (3.5 to 14.0)	7.2 (6.1 to 8.6)	106.0 (87.7 to 129.9)	28.1 (21.2 to 36.5)	38.0 (26.5 to 53.1)	20.3 (9.4 to 37.5)	19.5 (16.3 to 23.0)
Ethiopia	2027	40.2 (33.2 to 49.1)	10.8 (8.1 to 14.0)	14.4 (10.1 to 20.1)	7.8 (3.6 to 14.4)	7.2 (6.0 to 8.7)	108.1 (89.2 to 131.9)	28.9 (21.8 to 37.6)	38.8 (27.0 to 54.1)	20.9 (9.7 to 38.6)	19.4 (16.1 to 23.5)
Ethiopia	2028	41.1 (33.7 to 50.7)	11.1 (8.3 to 14.4)	14.7 (10.3 to 20.5)	8.0 (3.7 to 14.8)	7.2 (6.0 to 8.8)	110.3 (90.6 to 136.3)	29.7 (22.4 to 38.6)	39.6 (27.6 to 55.2)	21.6 (10.0 to 39.6)	19.4 (16.2 to 23.5)
Ethiopia	2029	41.9 (34.5 to 51.1)	11.4 (8.6 to 14.8)	15.1 (10.5 to 20.9)	8.3 (3.8 to 15.2)	7.2 (5.9 to 8.9)	112.6 (92.6 to 137.2)	30.6 (23.1 to 39.7)	40.4 (28.2 to 56.3)	22.2 (10.3 to 40.8)	19.4 (15.9 to 23.9)
Ethiopia	2030	42.8 (35.0 to 52.7)	11.7 (8.8 to 15.2)	15.4 (10.7 to 21.3)	8.5 (4.0 to 15.6)	7.2 (5.9 to 8.9)	115.0 (94.0 to 141.5)	31.4 (23.7 to 40.9)	41.3 (28.8 to 57.3)	22.8 (10.6 to 41.9)	19.4 (15.8 to 23.8)
Ethiopia	2031	43.7 (35.7 to 53.6)	12.0 (9.1 to 15.7)	15.7 (10.9 to 21.8)	8.8 (4.1 to 16.1)	7.2 (5.8 to 9.1)	117.4 (95.9 to 144.0)	32.3 (24.3 to 42.1)	42.1 (29.4 to 58.5)	23.5 (10.9 to 43.1)	19.4 (15.6 to 24.4)
Ethiopia	2032	44.6 (36.4 to 55.1)	12.3 (9.3 to 16.1)	16.0 (11.2 to 22.2)	9.0 (4.2 to 16.5)	7.2 (5.8 to 9.1)	119.8 (97.7 to 148.0)	33.2 (24.9 to 43.3)	43.0 (30.0 to 59.7)	24.2 (11.2 to 44.3)	19.5 (15.5 to 24.4)
Ethiopia	2033	45.5 (37.1 to 56.0)	12.7 (9.5 to 16.5)	16.3 (11.4 to 22.7)	9.3 (4.3 to 16.9)	7.3 (5.7 to 9.3)	122.2 (99.7 to 150.4)	34.0 (25.6 to 44.4)	43.8 (30.5 to 60.9)	24.9 (11.5 to 45.4)	19.5 (15.4 to 25.0)
Ethiopia	2034	46.5 (37.9 to 57.2)	13.0 (9.8 to 16.9)	16.6 (11.6 to 23.1)	9.5 (4.4 to 17.3)	7.3 (5.7 to 9.5)	124.9 (101.9 to 153.7)	34.9 (26.2 to 45.5)	44.7 (31.2 to 62.2)	25.6 (11.8 to 46.6)	19.6 (15.3 to 25.7)
Ethiopia	2035	47.5 (38.7 to 58.4)	13.3 (10.0 to 17.4)	17.0 (11.8 to 23.6)	9.8 (4.5 to 17.8)	7.3 (5.7 to 9.7)	127.6 (103.9 to 156.9)	35.9 (26.9 to 46.8)	45.6 (31.8 to 63.5)	26.4 (12.1 to 47.8)	19.7 (15.3 to 26.0)
Ethiopia	2036	48.5 (39.2 to 60.3)	13.7 (10.3 to 17.9)	17.3 (12.0 to 24.1)	10.1 (4.6 to 18.2)	7.4 (5.7 to 10.0)	130.2 (105.2 to 162.0)	36.8 (27.6 to 48.0)	46.5 (32.4 to 64.7)	27.1 (12.4 to 49.0)	19.8 (15.2 to 26.8)

Country	Year	2018 US Dollars per capita					2018 PPP per capita				
		Health spending	Government health spending	Out-of-pocket health spending	Prepaid private health spending	Development assistance for health	Health spending	Government health spending	Out-of-pocket health spending	Prepaid private health spending	Development assistance for health
Ethiopia	2037	49.5 (40.3 to 61.2)	14.1 (10.5 to 18.3)	17.6 (12.3 to 24.5)	10.4 (4.8 to 18.7)	7.4 (5.6 to 10.1)	132.9 (108.3 to 164.4)	37.8 (28.2 to 49.2)	47.4 (33.0 to 65.9)	27.9 (12.8 to 50.3)	19.9 (15.0 to 27.1)
Ethiopia	2038	50.6 (40.9 to 63.0)	14.4 (10.8 to 18.8)	18.0 (12.5 to 25.0)	10.7 (4.9 to 19.2)	7.5 (5.6 to 10.4)	135.8 (109.8 to 169.2)	38.8 (29.0 to 50.4)	48.3 (33.7 to 67.3)	28.7 (13.1 to 51.6)	20.0 (14.9 to 27.8)
Ethiopia	2039	51.7 (42.0 to 63.9)	14.8 (11.1 to 19.3)	18.4 (12.8 to 25.6)	11.0 (5.0 to 19.7)	7.5 (5.5 to 10.4)	138.8 (113.0 to 171.6)	39.8 (29.8 to 51.8)	49.3 (34.4 to 68.7)	29.5 (13.5 to 53.0)	20.1 (14.8 to 27.9)
Ethiopia	2040	52.7 (42.6 to 65.6)	15.2 (11.3 to 19.7)	18.7 (13.1 to 26.1)	11.3 (5.2 to 20.2)	7.5 (5.5 to 10.8)	141.7 (114.3 to 176.2)	40.8 (30.5 to 53.0)	50.2 (35.1 to 70.0)	30.3 (13.9 to 54.4)	20.3 (14.9 to 29.1)
Ethiopia	2041	53.9 (43.7 to 67.0)	15.6 (11.6 to 20.2)	19.1 (13.3 to 26.6)	11.6 (5.3 to 20.8)	7.6 (5.5 to 11.0)	144.8 (117.3 to 179.9)	41.9 (31.2 to 54.4)	51.3 (35.8 to 71.4)	31.2 (14.4 to 56.0)	20.4 (14.7 to 29.6)
Ethiopia	2042	55.1 (44.3 to 69.1)	16.0 (12.0 to 20.8)	19.5 (13.6 to 27.2)	12.0 (5.5 to 21.5)	7.7 (5.4 to 11.3)	148.0 (119.1 to 185.5)	43.0 (32.1 to 55.8)	52.3 (36.6 to 73.0)	32.1 (14.8 to 57.7)	20.6 (14.6 to 30.4)
Ethiopia	2043	56.3 (45.6 to 70.3)	16.4 (12.3 to 21.3)	19.8 (13.9 to 27.7)	12.3 (5.6 to 22.2)	7.7 (5.5 to 11.6)	151.3 (122.5 to 188.7)	44.1 (32.9 to 57.2)	53.3 (37.3 to 74.4)	33.1 (15.2 to 59.5)	20.8 (14.7 to 31.1)
Ethiopia	2044	57.5 (45.9 to 72.0)	16.8 (12.6 to 21.9)	20.2 (14.1 to 28.3)	12.6 (5.8 to 22.8)	7.8 (5.4 to 11.9)	154.6 (123.3 to 193.3)	45.3 (33.7 to 59.0)	54.4 (38.0 to 76.0)	34.0 (15.6 to 61.4)	21.0 (14.4 to 32.0)
Ethiopia	2045	58.9 (47.5 to 73.5)	17.3 (12.9 to 22.7)	20.6 (14.4 to 28.9)	13.0 (5.9 to 23.5)	7.9 (5.3 to 12.2)	158.2 (127.6 to 197.4)	46.6 (34.8 to 61.0)	55.4 (38.7 to 77.6)	34.9 (16.0 to 63.1)	21.2 (14.2 to 32.9)
Ethiopia	2046	60.2 (48.0 to 75.2)	17.8 (13.3 to 23.3)	21.0 (14.7 to 29.4)	13.4 (6.1 to 24.2)	8.0 (5.2 to 12.4)	161.7 (128.9 to 201.9)	47.9 (35.7 to 62.7)	56.4 (39.4 to 79.0)	35.9 (16.4 to 64.9)	21.5 (14.1 to 33.4)
Ethiopia	2047	61.6 (49.8 to 76.8)	18.4 (13.6 to 24.1)	21.4 (14.9 to 29.9)	13.7 (6.2 to 24.9)	8.1 (5.3 to 13.0)	165.5 (133.7 to 206.3)	49.4 (36.6 to 64.8)	57.4 (40.1 to 80.4)	36.8 (16.8 to 66.8)	21.7 (14.3 to 35.0)
Ethiopia	2048	63.1 (49.9 to 78.7)	19.0 (14.1 to 24.9)	21.8 (15.2 to 30.5)	14.1 (6.4 to 25.6)	8.2 (5.3 to 13.3)	169.5 (134.2 to 211.5)	51.1 (37.9 to 66.9)	58.5 (40.8 to 81.9)	37.8 (17.2 to 68.7)	22.0 (14.3 to 35.7)
Ethiopia	2049	64.6 (51.9 to 81.2)	19.7 (14.5 to 25.9)	22.2 (15.4 to 31.1)	14.5 (6.6 to 26.3)	8.3 (5.4 to 13.8)	173.6 (139.5 to 218.2)	52.9 (39.1 to 69.5)	59.6 (41.5 to 83.4)	38.9 (17.6 to 70.6)	22.3 (14.4 to 37.0)
Ethiopia	2050	66.2 (53.0 to 83.6)	20.4 (15.0 to 26.8)	22.6 (15.7 to 31.6)	14.8 (6.7 to 27.0)	8.4 (5.3 to 14.1)	177.9 (142.4 to 224.6)	54.7 (40.3 to 71.9)	60.6 (42.2 to 85.0)	39.9 (18.0 to 72.6)	22.7 (14.3 to 37.9)
Federated States of Micronesia	1995	89.7 (75.1 to 106.3)	73.9 (60.3 to 90.0)	13.9 (9.9 to 19.2)	0.0 (0.0 to 0.0)	1.9 (1.9 to 1.9)	99.5 (83.3 to 118.0)	82.0 (66.9 to 99.8)	15.4 (11.0 to 21.4)	0.0 (0.0 to 0.0)	2.1 (2.1 to 2.1)
Federated States of Micronesia	1996	84.9 (71.4 to 100.6)	71.2 (58.9 to 86.3)	13.7 (9.8 to 19.1)	0.0 (0.0 to 0.0)	0.0 (0.0 to 0.0)	94.2 (79.2 to 111.6)	79.0 (65.3 to 95.8)	15.2 (10.8 to 21.2)	0.0 (0.0 to 0.0)	0.0 (0.0 to 0.0)
Federated States of Micronesia	1997	81.8 (69.1 to 95.9)	67.3 (56.0 to 81.1)	13.4 (9.6 to 18.8)	0.0 (0.0 to 0.0)	1.1 (1.1 to 1.1)	90.7 (76.7 to 106.3)	74.6 (62.1 to 89.9)	14.8 (10.7 to 20.9)	0.0 (0.0 to 0.0)	1.3 (1.3 to 1.3)
Federated States of Micronesia	1998	82.3 (69.7 to 95.8)	67.9 (55.8 to 81.3)	13.5 (9.5 to 18.8)	0.0 (0.0 to 0.0)	0.8 (0.8 to 0.8)	91.3 (77.3 to 106.3)	75.4 (61.9 to 90.2)	15.0 (10.5 to 20.9)	0.0 (0.0 to 0.0)	0.9 (0.9 to 0.9)
Federated States of Micronesia	1999	173.2 (161.1 to 186.6)	65.3 (53.6 to 78.0)	13.2 (9.3 to 18.3)	0.0 (0.0 to 0.0)	94.6 (94.6 to 94.6)	192.1 (178.7 to 207.0)	72.4 (59.5 to 86.6)	14.7 (10.3 to 20.3)	0.0 (0.0 to 0.0)	105.0 (105.0 to 105.0)
Federated States of Micronesia	2000	74.4 (63.4 to 86.1)	60.0 (49.3 to 71.4)	12.7 (8.9 to 17.5)	0.0 (0.0 to 0.0)	1.8 (1.8 to 1.8)	82.6 (70.3 to 95.5)	66.5 (54.7 to 79.2)	14.1 (9.8 to 19.4)	0.0 (0.0 to 0.0)	2.0 (2.0 to 2.0)
Federated States of Micronesia	2001	75.7 (65.0 to 87.3)	59.7 (49.1 to 71.2)	12.5 (8.7 to 17.2)	0.0 (0.0 to 0.0)	3.5 (3.5 to 3.5)	84.0 (72.1 to 96.8)	66.3 (54.5 to 79.0)	13.9 (9.7 to 19.1)	0.0 (0.0 to 0.0)	3.8 (3.8 to 3.8)

		2018 US Dollars per capita					2018 PPP per capita				
Country	Year	Health spending	Government health spending	Out-of-pocket health spending	Prepaid private health spending	Development assistance for health	Health spending	Government health spending	Out-of-pocket health spending	Prepaid private health spending	Development assistance for health
Federated States of Micronesia	2002	74.0 (63.5 to 85.8)	58.8 (48.5 to 69.8)	12.3 (8.6 to 17.0)	0.0 (0.0 to 0.0)	2.9 (2.9 to 2.9)	82.1 (70.4 to 95.2)	65.2 (53.8 to 77.4)	13.6 (9.6 to 18.9)	0.0 (0.0 to 0.0)	3.2 (3.2 to 3.2)
Federated States of Micronesia	2003	131.1 (119.8 to 143.5)	64.8 (53.8 to 77.5)	12.5 (8.7 to 17.3)	0.0 (0.0 to 0.0)	53.8 (53.8 to 53.8)	145.4 (132.9 to 159.2)	71.9 (59.7 to 86.0)	13.8 (9.7 to 19.2)	0.0 (0.0 to 0.0)	59.7 (59.7 to 59.7)
Federated States of Micronesia	2004	310.8 (299.5 to 324.7)	69.3 (57.7 to 82.7)	12.4 (8.6 to 17.2)	0.0 (0.0 to 0.0)	229.2 (229.2 to 229.2)	344.8 (332.2 to 360.2)	76.8 (64.0 to 91.7)	13.7 (9.6 to 19.1)	0.0 (0.0 to 0.0)	254.2 (254.2 to 254.2)
Federated States of Micronesia	2005	335.5 (323.6 to 350.5)	73.0 (61.3 to 87.4)	12.2 (8.6 to 17.2)	0.0 (0.0 to 0.0)	250.2 (250.2 to 250.2)	372.1 (358.9 to 388.8)	81.0 (68.0 to 97.0)	13.6 (9.5 to 19.1)	0.0 (0.0 to 0.0)	277.5 (277.5 to 277.5)
Federated States of Micronesia	2006	298.4 (285.9 to 312.8)	75.5 (63.7 to 90.0)	12.0 (8.4 to 16.9)	0.0 (0.0 to 0.0)	210.8 (210.8 to 210.8)	331.0 (317.2 to 347.0)	83.8 (70.6 to 99.9)	13.3 (9.3 to 18.8)	0.0 (0.0 to 0.0)	233.8 (233.8 to 233.8)
Federated States of Micronesia	2007	298.9 (286.4 to 313.1)	74.4 (62.4 to 87.8)	11.6 (7.9 to 16.3)	0.0 (0.0 to 0.0)	212.9 (212.9 to 212.9)	331.6 (317.7 to 347.3)	82.6 (69.3 to 97.4)	12.8 (8.8 to 18.1)	0.0 (0.0 to 0.0)	236.2 (236.2 to 236.2)
Federated States of Micronesia	2008	319.6 (306.7 to 334.0)	76.9 (64.6 to 90.8)	11.2 (7.8 to 15.8)	0.0 (0.0 to 0.0)	231.5 (231.5 to 231.5)	354.6 (340.3 to 370.6)	85.3 (71.6 to 100.7)	12.4 (8.7 to 17.5)	0.0 (0.0 to 0.0)	256.9 (256.9 to 256.9)
Federated States of Micronesia	2009	376.3 (363.8 to 391.2)	76.5 (64.8 to 90.4)	10.8 (7.5 to 15.1)	0.0 (0.0 to 0.0)	289.0 (289.0 to 289.0)	417.4 (403.6 to 434.0)	84.8 (71.9 to 100.3)	12.0 (8.3 to 16.7)	0.0 (0.0 to 0.0)	320.6 (320.6 to 320.6)
Federated States of Micronesia	2010	117.7 (103.6 to 133.1)	82.8 (69.7 to 97.5)	10.7 (7.5 to 14.9)	0.0 (0.0 to 0.0)	24.2 (24.2 to 24.2)	130.6 (115.0 to 147.7)	91.9 (77.3 to 108.2)	11.9 (8.4 to 16.6)	0.0 (0.0 to 0.0)	26.9 (26.9 to 26.9)
Federated States of Micronesia	2011	128.4 (113.6 to 145.0)	90.4 (76.0 to 105.5)	10.5 (7.4 to 14.6)	0.0 (0.0 to 0.0)	27.5 (27.5 to 27.5)	142.5 (126.1 to 160.8)	100.2 (84.3 to 117.0)	11.7 (8.2 to 16.2)	0.0 (0.0 to 0.0)	30.5 (30.5 to 30.5)
Federated States of Micronesia	2012	354.0 (339.6 to 369.6)	88.0 (73.4 to 103.0)	10.1 (7.0 to 13.9)	0.0 (0.0 to 0.0)	255.9 (255.9 to 255.9)	392.7 (376.7 to 410.0)	97.6 (81.5 to 114.2)	11.2 (7.8 to 15.5)	0.0 (0.0 to 0.0)	283.9 (283.9 to 283.9)
Federated States of Micronesia	2013	114.3 (99.2 to 130.4)	87.8 (72.5 to 103.9)	9.8 (6.7 to 13.5)	0.0 (0.0 to 0.0)	16.7 (16.7 to 16.7)	126.8 (110.0 to 144.6)	97.4 (80.4 to 115.2)	10.8 (7.5 to 14.9)	0.0 (0.0 to 0.0)	18.6 (18.6 to 18.6)
Federated States of Micronesia	2014	397.8 (381.7 to 414.8)	89.8 (73.8 to 106.5)	9.5 (6.6 to 13.1)	0.0 (0.0 to 0.0)	298.5 (298.5 to 298.5)	441.3 (423.4 to 460.2)	99.6 (81.9 to 118.2)	10.6 (7.3 to 14.5)	0.0 (0.0 to 0.0)	331.1 (331.1 to 331.1)
Federated States of Micronesia	2015	203.3 (185.3 to 223.5)	98.2 (80.4 to 118.3)	9.8 (6.7 to 13.6)	0.0 (0.0 to 0.0)	95.3 (95.3 to 95.3)	225.5 (205.6 to 247.9)	108.9 (89.2 to 131.3)	10.8 (7.5 to 15.0)	0.0 (0.0 to 0.0)	105.7 (105.7 to 105.7)
Federated States of Micronesia	2016	130.1 (109.1 to 153.8)	109.6 (88.7 to 133.0)	10.0 (6.9 to 13.8)	0.0 (0.0 to 0.0)	10.5 (10.5 to 10.5)	144.3 (121.1 to 170.6)	121.6 (98.4 to 147.5)	11.1 (7.6 to 15.3)	0.0 (0.0 to 0.0)	11.6 (11.6 to 11.6)
Federated States of Micronesia	2017	131.0 (109.4 to 155.0)	110.6 (89.5 to 134.2)	10.2 (7.0 to 14.1)	0.0 (0.0 to 0.0)	10.2 (10.2 to 10.2)	145.3 (121.3 to 172.0)	122.7 (99.3 to 148.9)	11.3 (7.8 to 15.6)	0.0 (0.0 to 0.0)	11.3 (11.3 to 11.3)
Federated States of Micronesia	2018	132.4 (110.4 to 157.5)	112.6 (91.3 to 136.7)	10.3 (7.1 to 14.3)	0.0 (0.0 to 0.0)	9.5 (9.5 to 9.5)	146.9 (122.5 to 174.7)	124.9 (101.3 to 151.7)	11.4 (7.9 to 15.8)	0.0 (0.0 to 0.0)	10.6 (10.6 to 10.6)
Federated States of Micronesia	2019	135.8 (113.9 to 160.7)	115.8 (93.4 to 140.7)	10.4 (7.2 to 14.4)	0.0 (0.0 to 0.0)	9.6 (8.9 to 10.1)	150.6 (126.3 to 178.3)	128.5 (103.6 to 156.1)	11.5 (7.9 to 16.0)	0.0 (0.0 to 0.0)	10.6 (9.9 to 11.2)
Federated States of Micronesia	2020	139.0 (115.7 to 164.5)	118.8 (96.4 to 143.9)	10.5 (7.2 to 14.6)	0.0 (0.0 to 0.0)	9.7 (8.9 to 10.4)	154.2 (128.4 to 182.5)	131.8 (106.9 to 159.7)	11.6 (8.0 to 16.1)	0.0 (0.0 to 0.0)	10.7 (9.8 to 11.6)
Federated States of Micronesia	2021	141.3 (117.8 to 167.7)	120.9 (98.2 to 146.8)	10.6 (7.3 to 14.7)	0.0 (0.0 to 0.0)	9.8 (8.8 to 10.8)	156.7 (130.6 to 186.0)	134.1 (108.9 to 162.9)	11.7 (8.1 to 16.3)	0.0 (0.0 to 0.0)	10.9 (9.8 to 12.0)
Federated States of Micronesia	2022	143.7 (120.3 to 171.7)	123.0 (99.9 to 149.8)	10.7 (7.4 to 14.9)	0.0 (0.0 to 0.0)	10.0 (8.9 to 11.1)	159.4 (133.4 to 190.4)	136.4 (110.8 to 166.1)	11.9 (8.2 to 16.5)	0.0 (0.0 to 0.0)	11.1 (9.8 to 12.4)

Country	Year	2018 US Dollars per capita					2018 PPP per capita				
		Health spending	Government health spending	Out-of-pocket health spending	Prepaid private health spending	Development assistance for health	Health spending	Government health spending	Out-of-pocket health spending	Prepaid private health spending	Development assistance for health
Federated States of Micronesia	2023	146.2 (121.7 to 173.5)	125.2 (101.6 to 152.5)	10.8 (7.5 to 15.0)	0.0 (0.0 to 0.0)	10.1 (8.8 to 11.5)	162.2 (135.0 to 192.4)	138.9 (112.7 to 169.2)	12.0 (8.3 to 16.7)	0.0 (0.0 to 0.0)	11.2 (9.8 to 12.8)
Federated States of Micronesia	2024	148.7 (123.8 to 177.0)	127.4 (103.3 to 155.2)	11.0 (7.5 to 15.2)	0.0 (0.0 to 0.0)	10.3 (8.9 to 11.9)	165.0 (137.4 to 196.4)	141.3 (114.6 to 172.2)	12.2 (8.4 to 16.9)	0.0 (0.0 to 0.0)	11.5 (9.9 to 13.2)
Federated States of Micronesia	2025	151.3 (126.4 to 180.2)	129.7 (104.9 to 158.2)	11.1 (7.6 to 15.4)	0.0 (0.0 to 0.0)	10.5 (9.0 to 12.3)	167.8 (140.2 to 199.9)	143.9 (116.4 to 175.4)	12.3 (8.5 to 17.1)	0.0 (0.0 to 0.0)	11.6 (9.9 to 13.6)
Federated States of Micronesia	2026	153.9 (128.2 to 182.9)	132.0 (106.7 to 160.7)	11.2 (7.7 to 15.6)	0.0 (0.0 to 0.0)	10.7 (9.0 to 12.5)	170.7 (142.2 to 202.9)	146.5 (118.3 to 178.2)	12.4 (8.6 to 17.3)	0.0 (0.0 to 0.0)	11.8 (10.0 to 13.9)
Federated States of Micronesia	2027	156.4 (129.6 to 185.7)	134.2 (108.3 to 163.2)	11.3 (7.8 to 15.8)	0.0 (0.0 to 0.0)	10.9 (9.1 to 12.9)	173.5 (143.8 to 206.0)	148.9 (120.2 to 181.0)	12.5 (8.6 to 17.5)	0.0 (0.0 to 0.1)	12.0 (10.1 to 14.3)
Federated States of Micronesia	2028	158.7 (132.0 to 188.9)	136.2 (109.8 to 165.7)	11.4 (7.9 to 15.9)	0.0 (0.0 to 0.0)	11.1 (9.3 to 13.3)	176.1 (146.4 to 209.6)	151.1 (121.8 to 183.8)	12.7 (8.7 to 17.6)	0.0 (0.0 to 0.1)	12.3 (10.3 to 14.8)
Federated States of Micronesia	2029	160.9 (133.6 to 191.1)	138.0 (111.4 to 168.2)	11.5 (7.9 to 16.0)	0.0 (0.0 to 0.0)	11.3 (9.3 to 13.8)	178.4 (148.3 to 212.0)	153.1 (123.6 to 186.6)	12.8 (8.8 to 17.8)	0.0 (0.0 to 0.1)	12.5 (10.3 to 15.3)
Federated States of Micronesia	2030	163.0 (135.6 to 194.2)	139.9 (112.7 to 170.5)	11.6 (8.0 to 16.2)	0.0 (0.0 to 0.0)	11.5 (9.4 to 14.1)	180.8 (150.4 to 215.4)	155.2 (125.1 to 189.1)	12.9 (8.9 to 17.9)	0.0 (0.0 to 0.1)	12.8 (10.4 to 15.6)
Federated States of Micronesia	2031	165.3 (138.3 to 196.2)	141.8 (113.9 to 172.3)	11.7 (8.1 to 16.3)	0.0 (0.0 to 0.1)	11.7 (9.5 to 14.7)	183.4 (153.5 to 217.6)	157.3 (126.4 to 191.1)	13.0 (9.0 to 18.1)	0.0 (0.0 to 0.1)	13.0 (10.6 to 16.3)
Federated States of Micronesia	2032	167.5 (138.4 to 198.2)	143.6 (115.2 to 174.5)	11.8 (8.2 to 16.5)	0.0 (0.0 to 0.1)	12.0 (9.6 to 15.1)	185.8 (153.6 to 219.9)	159.3 (127.8 to 193.6)	13.1 (9.1 to 18.3)	0.0 (0.0 to 0.1)	13.3 (10.7 to 16.7)
Federated States of Micronesia	2033	169.7 (140.6 to 201.5)	145.5 (116.8 to 176.6)	12.0 (8.3 to 16.6)	0.0 (0.0 to 0.1)	12.2 (9.7 to 15.7)	188.3 (156.0 to 223.6)	161.4 (129.6 to 195.9)	13.3 (9.2 to 18.5)	0.0 (0.0 to 0.1)	13.6 (10.8 to 17.4)
Federated States of Micronesia	2034	171.8 (143.3 to 204.4)	147.2 (118.3 to 178.9)	12.1 (8.3 to 16.8)	0.0 (0.0 to 0.1)	12.5 (9.9 to 16.2)	190.6 (158.9 to 226.7)	163.3 (131.3 to 198.4)	13.4 (9.3 to 18.7)	0.0 (0.0 to 0.1)	13.9 (10.9 to 17.9)
Federated States of Micronesia	2035	174.0 (143.7 to 208.3)	148.9 (119.9 to 181.6)	12.2 (8.4 to 17.0)	0.0 (0.0 to 0.1)	12.8 (10.0 to 16.8)	193.0 (159.4 to 231.1)	165.2 (133.1 to 201.4)	13.5 (9.4 to 18.9)	0.0 (0.0 to 0.1)	14.2 (11.1 to 18.6)
Federated States of Micronesia	2036	176.3 (145.9 to 209.7)	150.8 (121.5 to 184.2)	12.4 (8.5 to 17.2)	0.0 (0.0 to 0.1)	13.1 (10.1 to 17.6)	195.6 (161.8 to 232.7)	167.3 (134.8 to 204.3)	13.7 (9.5 to 19.1)	0.0 (0.0 to 0.1)	14.6 (11.2 to 19.6)
Federated States of Micronesia	2037	178.6 (147.2 to 214.0)	152.6 (122.9 to 187.5)	12.5 (8.6 to 17.5)	0.0 (0.0 to 0.1)	13.4 (10.3 to 18.0)	198.1 (163.3 to 237.4)	169.3 (136.4 to 208.0)	13.9 (9.6 to 19.4)	0.0 (0.0 to 0.1)	14.9 (11.4 to 20.0)
Federated States of Micronesia	2038	180.9 (148.6 to 216.6)	154.4 (123.8 to 191.0)	12.7 (8.7 to 17.7)	0.0 (0.0 to 0.1)	13.7 (10.5 to 19.1)	200.6 (164.8 to 240.2)	171.3 (137.3 to 211.8)	14.1 (9.7 to 19.7)	0.0 (0.0 to 0.1)	15.2 (11.6 to 21.2)
Federated States of Micronesia	2039	183.2 (151.1 to 218.5)	156.2 (124.6 to 192.9)	12.9 (8.9 to 18.0)	0.0 (0.0 to 0.1)	14.1 (10.7 to 19.5)	203.2 (167.6 to 242.4)	173.3 (138.2 to 214.0)	14.3 (9.8 to 20.0)	0.0 (0.0 to 0.1)	15.6 (11.9 to 21.6)
Federated States of Micronesia	2040	185.5 (151.3 to 224.0)	158.1 (126.1 to 196.6)	13.0 (9.0 to 18.3)	0.0 (0.0 to 0.1)	14.4 (10.9 to 19.9)	205.8 (167.8 to 248.5)	175.3 (139.9 to 218.1)	14.5 (10.0 to 20.2)	0.0 (0.0 to 0.1)	16.0 (12.1 to 22.1)
Federated States of Micronesia	2041	188.2 (153.5 to 229.5)	160.2 (127.2 to 199.7)	13.2 (9.1 to 18.5)	0.0 (0.0 to 0.1)	14.8 (11.1 to 20.5)	208.8 (170.3 to 254.6)	177.8 (141.2 to 221.5)	14.7 (10.1 to 20.5)	0.0 (0.0 to 0.1)	16.4 (12.3 to 22.7)
Federated States of Micronesia	2042	191.1 (154.7 to 233.9)	162.5 (127.9 to 203.5)	13.4 (9.2 to 18.7)	0.0 (0.0 to 0.1)	15.1 (11.4 to 21.5)	211.9 (171.6 to 259.5)	180.3 (141.8 to 225.8)	14.9 (10.2 to 20.8)	0.0 (0.0 to 0.1)	16.8 (12.6 to 23.9)
Federated States of Micronesia	2043	193.9 (156.6 to 235.2)	164.8 (129.0 to 207.8)	13.6 (9.3 to 19.0)	0.0 (0.0 to 0.1)	15.5 (11.5 to 22.4)	215.1 (173.8 to 260.9)	182.8 (143.1 to 230.5)	15.0 (10.3 to 21.1)	0.0 (0.0 to 0.1)	17.2 (12.8 to 24.9)

Country	Year	2018 US Dollars per capita					2018 PPP per capita				
		Health spending	Government health spending	Out-of-pocket health spending	Prepaid private health spending	Development assistance for health	Health spending	Government health spending	Out-of-pocket health spending	Prepaid private health spending	Development assistance for health
Federated States of Micronesia	2044	197.1 (159.1 to 242.4)	167.4 (130.4 to 211.9)	13.7 (9.4 to 19.2)	0.0 (0.0 to 0.1)	15.9 (11.7 to 23.1)	218.6 (176.4 to 268.9)	185.7 (144.7 to 235.1)	15.2 (10.4 to 21.3)	0.0 (0.0 to 0.1)	17.7 (13.0 to 25.6)
Federated States of Micronesia	2045	200.5 (159.9 to 245.6)	170.3 (132.9 to 215.7)	13.9 (9.5 to 19.4)	0.0 (0.0 to 0.1)	16.4 (11.8 to 24.7)	222.5 (177.4 to 272.4)	188.9 (147.4 to 239.3)	15.4 (10.5 to 21.5)	0.0 (0.0 to 0.1)	18.2 (13.1 to 27.4)
Federated States of Micronesia	2046	204.6 (165.8 to 249.7)	173.7 (136.4 to 219.6)	14.0 (9.6 to 19.5)	0.0 (0.0 to 0.1)	16.8 (12.2 to 25.9)	226.9 (183.9 to 277.0)	192.7 (151.3 to 243.6)	15.5 (10.6 to 21.6)	0.0 (0.0 to 0.1)	18.6 (13.5 to 28.7)
Federated States of Micronesia	2047	209.0 (169.1 to 256.0)	177.5 (139.3 to 223.5)	14.1 (9.7 to 19.7)	0.0 (0.0 to 0.1)	17.3 (12.4 to 27.2)	231.8 (187.6 to 283.9)	196.9 (154.5 to 247.9)	15.7 (10.7 to 21.8)	0.0 (0.0 to 0.1)	19.2 (13.8 to 30.2)
Federated States of Micronesia	2048	213.6 (173.3 to 260.9)	181.6 (143.7 to 228.0)	14.2 (9.8 to 19.9)	0.0 (0.0 to 0.1)	17.8 (12.7 to 28.5)	237.0 (192.2 to 289.4)	201.4 (159.4 to 253.0)	15.8 (10.8 to 22.1)	0.0 (0.0 to 0.1)	19.8 (14.1 to 31.7)
Federated States of Micronesia	2049	218.1 (177.4 to 265.6)	185.4 (146.8 to 231.9)	14.3 (9.8 to 20.1)	0.0 (0.0 to 0.1)	18.3 (13.1 to 29.5)	242.0 (196.8 to 294.6)	205.7 (162.9 to 257.3)	15.9 (10.9 to 22.3)	0.0 (0.0 to 0.1)	20.3 (14.5 to 32.7)
Federated States of Micronesia	2050	222.7 (179.8 to 270.7)	189.3 (149.2 to 236.6)	14.4 (9.9 to 20.2)	0.0 (0.0 to 0.1)	18.9 (13.3 to 30.9)	247.1 (199.4 to 300.3)	210.0 (165.5 to 262.5)	16.0 (11.0 to 22.4)	0.0 (0.0 to 0.1)	21.0 (14.7 to 34.2)
Fiji	1995	119.9 (101.5 to 141.5)	91.5 (75.0 to 110.7)	16.6 (11.4 to 22.7)	10.9 (5.6 to 19.6)	0.9 (0.9 to 0.9)	209.4 (177.4 to 247.2)	159.8 (131.1 to 193.4)	29.0 (20.0 to 39.6)	19.1 (9.7 to 34.3)	1.6 (1.6 to 1.6)
Fiji	1996	126.7 (107.9 to 147.9)	98.4 (81.5 to 118.2)	17.1 (11.9 to 23.2)	11.1 (5.7 to 19.7)	0.1 (0.1 to 0.1)	221.5 (188.6 to 258.4)	172.0 (142.3 to 206.6)	29.8 (20.7 to 40.6)	19.5 (10.0 to 34.3)	0.1 (0.1 to 0.1)
Fiji	1997	126.9 (109.1 to 146.6)	99.0 (82.8 to 118.3)	16.9 (11.8 to 23.0)	10.3 (5.4 to 18.5)	0.6 (0.6 to 0.6)	221.7 (190.6 to 256.1)	173.1 (144.7 to 206.7)	29.5 (20.7 to 40.2)	18.1 (9.4 to 32.4)	1.1 (1.1 to 1.1)
Fiji	1998	134.4 (117.9 to 153.5)	97.4 (82.0 to 114.9)	16.6 (11.6 to 22.8)	8.7 (4.5 to 15.7)	11.7 (11.7 to 11.7)	234.9 (205.9 to 268.3)	170.1 (143.3 to 200.8)	29.0 (20.3 to 39.8)	15.3 (7.9 to 27.4)	20.4 (20.4 to 20.4)
Fiji	1999	143.5 (126.4 to 162.8)	102.3 (86.3 to 120.5)	17.3 (12.2 to 23.9)	6.8 (3.5 to 11.6)	17.3 (17.3 to 17.3)	250.8 (220.9 to 284.5)	178.7 (150.8 to 210.5)	30.2 (21.3 to 41.8)	11.8 (6.1 to 20.3)	30.2 (30.2 to 30.2)
Fiji	2000	141.0 (124.0 to 159.8)	103.7 (88.0 to 120.4)	17.3 (12.2 to 24.0)	7.2 (3.7 to 12.5)	12.9 (12.9 to 12.9)	246.4 (216.7 to 279.3)	181.2 (153.8 to 210.4)	30.3 (21.3 to 41.9)	12.5 (6.5 to 21.9)	22.5 (22.5 to 22.5)
Fiji	2001	133.9 (117.8 to 152.3)	102.5 (87.3 to 118.5)	17.8 (12.7 to 24.8)	7.3 (3.8 to 12.8)	6.3 (6.3 to 6.3)	233.9 (205.8 to 266.1)	179.1 (152.5 to 207.0)	31.1 (22.2 to 43.3)	12.7 (6.6 to 22.4)	11.0 (11.0 to 11.0)
Fiji	2002	139.9 (124.1 to 158.1)	105.8 (91.1 to 121.5)	18.4 (13.2 to 25.3)	7.8 (4.0 to 14.1)	7.9 (7.9 to 7.9)	244.4 (216.9 to 276.2)	184.9 (159.2 to 212.4)	32.1 (23.1 to 44.2)	13.6 (7.0 to 24.6)	13.8 (13.8 to 13.8)
Fiji	2003	154.7 (138.8 to 173.2)	107.1 (92.5 to 123.2)	19.1 (13.7 to 25.9)	8.0 (4.1 to 14.4)	20.5 (20.5 to 20.5)	270.4 (242.5 to 302.7)	187.2 (161.6 to 215.2)	33.4 (24.0 to 45.3)	14.0 (7.2 to 25.2)	35.8 (35.8 to 35.8)
Fiji	2004	154.2 (138.1 to 173.4)	112.8 (97.7 to 129.3)	20.9 (14.9 to 27.9)	9.1 (4.6 to 16.1)	11.5 (11.5 to 11.5)	269.5 (241.3 to 302.9)	197.0 (170.7 to 225.9)	36.4 (26.1 to 48.7)	15.8 (8.0 to 28.1)	20.1 (20.1 to 20.1)
Fiji	2005	155.2 (137.9 to 175.0)	116.4 (101.5 to 134.2)	22.1 (15.9 to 29.2)	10.0 (5.0 to 17.9)	6.7 (6.7 to 6.7)	271.2 (240.9 to 305.8)	203.5 (177.4 to 234.5)	38.6 (27.7 to 51.0)	17.5 (8.7 to 31.2)	11.7 (11.7 to 11.7)
Fiji	2006	166.7 (148.5 to 188.5)	120.0 (104.7 to 139.0)	24.7 (17.8 to 32.6)	11.9 (6.0 to 21.1)	10.2 (10.2 to 10.2)	291.3 (259.5 to 329.4)	209.6 (183.0 to 242.9)	43.1 (31.1 to 56.9)	20.8 (10.4 to 36.9)	17.8 (17.8 to 17.8)
Fiji	2007	169.4 (150.7 to 191.5)	116.4 (100.8 to 135.2)	27.0 (19.7 to 34.9)	13.0 (6.7 to 22.8)	13.0 (13.0 to 13.0)	296.0 (263.3 to 334.7)	203.4 (176.2 to 236.2)	47.2 (34.5 to 61.0)	22.6 (11.7 to 39.9)	22.8 (22.8 to 22.8)
Fiji	2008	163.6 (145.1 to 184.5)	110.9 (95.7 to 128.7)	29.6 (21.6 to 37.8)	13.1 (6.9 to 23.1)	10.1 (10.1 to 10.1)	285.8 (253.6 to 322.5)	193.8 (167.2 to 224.9)	51.7 (37.8 to 66.1)	22.8 (12.1 to 40.3)	17.6 (17.6 to 17.6)

		2018 US Dollars per capita					2018 PPP per capita				
Country	Year	Health spending	Government health spending	Out-of-pocket health spending	Prepaid private health spending	Development assistance for health	Health spending	Government health spending	Out-of-pocket health spending	Prepaid private health spending	Development assistance for health
Fiji	2009	162.2 (142.1 to 182.9)	104.9 (90.3 to 121.7)	34.2 (25.1 to 44.3)	13.1 (7.0 to 22.7)	10.0 (10.0 to 10.0)	283.5 (248.3 to 319.7)	183.4 (157.9 to 212.7)	59.8 (43.8 to 77.5)	22.9 (12.2 to 39.8)	17.4 (17.4 to 17.4)
Fiji	2010	170.1 (149.5 to 190.7)	102.5 (87.7 to 118.4)	36.3 (27.2 to 47.3)	14.3 (7.7 to 24.1)	17.0 (17.0 to 17.0)	297.2 (261.2 to 333.3)	179.0 (153.2 to 206.8)	63.5 (47.6 to 82.6)	25.0 (13.4 to 42.2)	29.7 (29.7 to 29.7)
Fiji	2011	169.7 (149.1 to 191.3)	99.6 (84.9 to 116.2)	37.8 (27.7 to 49.6)	15.9 (8.6 to 26.5)	16.3 (16.3 to 16.3)	296.5 (260.6 to 334.3)	174.1 (148.4 to 203.1)	66.1 (48.4 to 86.6)	27.9 (15.0 to 46.3)	28.5 (28.5 to 28.5)
Fiji	2012	165.7 (144.4 to 188.1)	99.7 (84.9 to 116.3)	38.6 (28.3 to 51.2)	18.1 (9.8 to 30.1)	9.4 (9.4 to 9.4)	289.6 (252.3 to 328.6)	174.1 (148.3 to 203.2)	67.4 (49.5 to 89.5)	31.6 (17.2 to 52.6)	16.5 (16.5 to 16.5)
Fiji	2013	178.1 (155.1 to 202.8)	105.5 (90.1 to 123.7)	39.1 (28.6 to 52.2)	22.0 (11.8 to 36.2)	11.4 (11.4 to 11.4)	311.2 (271.0 to 354.3)	184.4 (157.4 to 216.1)	68.3 (49.9 to 91.2)	38.5 (20.7 to 63.2)	20.0 (20.0 to 20.0)
Fiji	2014	191.7 (167.1 to 221.2)	114.6 (97.6 to 135.7)	39.8 (29.1 to 53.1)	25.6 (13.6 to 43.2)	11.7 (11.7 to 11.7)	335.0 (291.9 to 386.6)	200.3 (170.6 to 237.1)	69.5 (50.8 to 92.9)	44.8 (23.8 to 75.5)	20.4 (20.4 to 20.4)
Fiji	2015	199.4 (173.7 to 231.2)	121.6 (102.7 to 143.1)	40.9 (29.3 to 54.7)	27.0 (14.4 to 46.5)	9.8 (9.8 to 9.8)	348.4 (303.5 to 404.0)	212.5 (179.5 to 250.0)	71.5 (51.2 to 95.6)	47.2 (25.2 to 81.2)	17.1 (17.1 to 17.1)
Fiji	2016	200.4 (173.1 to 233.6)	123.7 (103.1 to 146.3)	40.7 (29.1 to 55.4)	27.0 (14.1 to 46.6)	9.0 (9.0 to 9.0)	350.2 (302.5 to 408.2)	216.2 (180.2 to 255.7)	71.1 (50.8 to 96.8)	47.1 (24.7 to 81.4)	15.7 (15.7 to 15.7)
Fiji	2017	213.9 (184.7 to 248.9)	137.5 (114.6 to 163.1)	42.0 (29.9 to 56.9)	28.0 (14.6 to 48.2)	6.4 (6.4 to 6.4)	373.7 (322.8 to 435.0)	240.3 (200.3 to 285.0)	73.4 (52.2 to 99.5)	48.8 (25.6 to 84.2)	11.2 (11.2 to 11.2)
Fiji	2018	217.1 (187.0 to 252.5)	139.6 (116.5 to 165.8)	43.0 (30.7 to 58.1)	28.5 (15.0 to 49.3)	6.1 (6.1 to 6.1)	379.4 (326.8 to 441.2)	244.0 (203.6 to 289.7)	75.1 (53.6 to 101.5)	49.7 (26.1 to 86.1)	10.6 (10.6 to 10.6)
Fiji	2019	220.6 (189.9 to 256.7)	141.6 (117.9 to 168.3)	43.9 (31.3 to 59.4)	28.9 (15.2 to 50.2)	6.1 (5.7 to 6.5)	385.5 (331.8 to 448.6)	247.4 (206.0 to 294.0)	76.8 (54.7 to 103.7)	50.6 (26.5 to 87.7)	10.7 (10.0 to 11.3)
Fiji	2020	224.1 (192.5 to 260.2)	143.5 (119.5 to 170.5)	44.9 (32.2 to 60.5)	29.4 (15.4 to 50.7)	6.3 (5.7 to 6.7)	391.6 (336.3 to 454.7)	250.8 (208.8 to 297.9)	78.5 (56.3 to 105.8)	51.4 (27.0 to 88.6)	10.9 (10.0 to 11.8)
Fiji	2021	227.0 (195.3 to 263.7)	145.0 (121.0 to 172.2)	45.9 (32.9 to 62.2)	29.8 (15.6 to 51.2)	6.4 (5.8 to 7.1)	396.7 (341.3 to 460.8)	253.4 (211.4 to 300.9)	80.1 (57.4 to 108.7)	52.0 (27.3 to 89.5)	11.2 (10.1 to 12.4)
Fiji	2022	230.0 (197.8 to 266.6)	146.4 (122.3 to 173.8)	46.8 (33.4 to 63.3)	30.1 (15.8 to 51.9)	6.6 (5.9 to 7.4)	401.8 (345.7 to 465.8)	255.8 (213.8 to 303.6)	81.8 (58.4 to 110.6)	52.7 (27.6 to 90.7)	11.5 (10.2 to 12.9)
Fiji	2023	233.0 (200.5 to 270.7)	147.9 (123.7 to 175.7)	47.8 (34.2 to 64.6)	30.5 (16.1 to 52.6)	6.8 (5.9 to 7.7)	407.1 (350.4 to 473.1)	258.4 (216.1 to 307.0)	83.5 (59.8 to 113.0)	53.3 (28.0 to 91.8)	11.9 (10.3 to 13.5)
Fiji	2024	236.0 (202.9 to 273.9)	149.3 (124.9 to 177.6)	48.8 (35.0 to 66.0)	30.9 (16.3 to 53.2)	7.0 (6.1 to 8.1)	412.4 (354.5 to 478.6)	260.9 (218.2 to 310.4)	85.2 (61.1 to 115.3)	54.0 (28.4 to 93.0)	12.3 (10.6 to 14.2)
Fiji	2025	239.1 (206.2 to 277.3)	150.8 (126.3 to 179.7)	49.8 (35.8 to 67.6)	31.3 (16.5 to 54.0)	7.2 (6.2 to 8.5)	417.8 (360.3 to 484.5)	263.4 (220.6 to 314.0)	87.0 (62.6 to 118.1)	54.8 (28.8 to 94.3)	12.6 (10.8 to 14.8)
Fiji	2026	242.4 (209.0 to 281.4)	152.3 (127.6 to 182.1)	50.8 (36.5 to 69.0)	31.8 (16.8 to 54.8)	7.4 (6.3 to 8.7)	423.5 (365.2 to 491.7)	266.2 (222.9 to 318.1)	88.8 (63.7 to 120.6)	55.5 (29.3 to 95.7)	13.0 (11.0 to 15.2)
Fiji	2027	245.9 (211.9 to 285.9)	154.1 (128.9 to 184.2)	51.9 (37.1 to 70.9)	32.2 (17.0 to 55.6)	7.7 (6.4 to 9.1)	429.7 (370.2 to 499.6)	269.2 (225.3 to 321.9)	90.7 (64.8 to 123.9)	56.3 (29.7 to 97.2)	13.4 (11.2 to 15.9)
Fiji	2028	250.2 (215.1 to 292.3)	156.5 (130.9 to 187.4)	53.1 (37.7 to 72.9)	32.8 (17.3 to 56.6)	7.9 (6.6 to 9.5)	437.2 (375.9 to 510.7)	273.4 (228.8 to 327.4)	92.7 (65.9 to 127.4)	57.3 (30.2 to 98.9)	13.8 (11.5 to 16.7)
Fiji	2029	255.3 (218.9 to 297.6)	159.5 (133.4 to 191.2)	54.3 (38.8 to 74.4)	33.4 (17.7 to 57.8)	8.1 (6.7 to 9.9)	446.2 (382.5 to 520.0)	278.7 (233.1 to 334.1)	94.9 (67.7 to 130.0)	58.4 (30.9 to 101.0)	14.2 (11.7 to 17.3)

Country	Year	2018 US Dollars per capita					2018 PPP per capita				
		Health spending	Government health spending	Out-of-pocket health spending	Prepaid private health spending	Development assistance for health	Health spending	Government health spending	Out-of-pocket health spending	Prepaid private health spending	Development assistance for health
Fiji	2030	260.6 (223.3 to 303.8)	162.6 (135.6 to 195.0)	55.5 (39.7 to 76.3)	34.1 (18.1 to 59.0)	8.4 (6.8 to 10.2)	455.4 (390.2 to 530.8)	284.2 (237.0 to 340.8)	97.0 (69.3 to 133.3)	59.6 (31.6 to 103.1)	14.6 (11.9 to 17.9)
Fiji	2031	266.0 (227.3 to 310.9)	165.8 (138.2 to 199.1)	56.8 (40.4 to 78.5)	34.8 (18.5 to 60.3)	8.6 (7.0 to 10.8)	464.8 (397.2 to 543.3)	289.8 (241.5 to 348.0)	99.2 (70.7 to 137.2)	60.8 (32.2 to 105.3)	15.0 (12.2 to 18.9)
Fiji	2032	271.8 (233.4 to 319.1)	169.3 (141.0 to 203.5)	58.0 (41.2 to 80.6)	35.6 (18.9 to 61.6)	8.8 (7.1 to 11.1)	474.9 (407.9 to 557.6)	295.8 (246.4 to 355.6)	101.4 (71.9 to 140.8)	62.1 (33.0 to 107.7)	15.5 (12.4 to 19.4)
Fiji	2033	277.5 (238.7 to 325.1)	172.8 (143.9 to 208.3)	59.3 (42.1 to 82.7)	36.3 (19.2 to 63.0)	9.1 (7.2 to 11.7)	485.0 (417.0 to 568.0)	301.9 (251.5 to 363.9)	103.7 (73.5 to 144.5)	63.5 (33.6 to 110.1)	15.9 (12.6 to 20.4)
Fiji	2034	283.5 (243.0 to 331.8)	176.4 (146.7 to 213.0)	60.6 (42.8 to 84.9)	37.1 (19.6 to 64.4)	9.4 (7.4 to 12.1)	495.5 (424.7 to 579.7)	308.3 (256.3 to 372.1)	105.9 (74.8 to 148.4)	64.8 (34.3 to 112.5)	16.4 (13.0 to 21.2)
Fiji	2035	289.6 (247.3 to 340.3)	180.1 (149.6 to 217.9)	61.9 (43.4 to 86.7)	37.9 (20.0 to 65.9)	9.7 (7.6 to 12.7)	506.1 (432.2 to 594.7)	314.7 (261.3 to 380.8)	108.1 (75.8 to 151.6)	66.3 (35.0 to 115.1)	17.0 (13.2 to 22.2)
Fiji	2036	295.8 (252.3 to 348.3)	183.9 (152.4 to 223.0)	63.2 (44.0 to 88.8)	38.7 (20.5 to 67.3)	10.0 (7.7 to 13.5)	517.0 (440.8 to 608.7)	321.3 (266.3 to 389.6)	110.4 (76.8 to 155.1)	67.7 (35.7 to 117.7)	17.5 (13.5 to 23.5)
Fiji	2037	302.1 (257.7 to 354.9)	187.7 (155.4 to 227.6)	64.5 (44.9 to 91.1)	39.6 (20.9 to 68.8)	10.3 (8.0 to 13.8)	527.9 (450.3 to 620.1)	328.0 (271.5 to 397.8)	112.7 (78.4 to 159.3)	69.2 (36.5 to 120.3)	18.1 (13.9 to 24.2)
Fiji	2038	308.5 (263.2 to 363.6)	191.6 (158.3 to 233.1)	65.8 (45.5 to 93.5)	40.4 (21.3 to 70.4)	10.7 (8.1 to 14.8)	539.1 (459.9 to 635.3)	334.9 (276.6 to 407.3)	115.0 (79.6 to 163.5)	70.7 (37.2 to 123.0)	18.6 (14.2 to 25.8)
Fiji	2039	314.9 (267.7 to 371.9)	195.5 (161.5 to 238.2)	67.1 (46.3 to 95.5)	41.3 (21.7 to 71.9)	11.0 (8.4 to 15.2)	550.3 (467.8 to 649.9)	341.7 (282.2 to 416.2)	117.2 (80.9 to 167.0)	72.2 (37.9 to 125.7)	19.2 (14.7 to 26.6)
Fiji	2040	321.3 (272.5 to 379.2)	199.4 (164.5 to 243.3)	68.4 (47.2 to 97.5)	42.1 (22.0 to 73.2)	11.4 (8.7 to 15.7)	561.4 (476.1 to 662.7)	348.5 (287.4 to 425.2)	119.5 (82.5 to 170.4)	73.6 (38.5 to 127.9)	19.9 (15.1 to 27.4)
Fiji	2041	327.8 (279.9 to 387.7)	203.4 (167.5 to 248.1)	69.7 (48.0 to 99.5)	43.0 (22.4 to 74.4)	11.7 (8.9 to 16.3)	572.8 (489.0 to 677.4)	355.4 (292.8 to 433.5)	121.8 (83.9 to 173.9)	75.2 (39.2 to 130.1)	20.5 (15.5 to 28.5)
Fiji	2042	334.3 (284.2 to 396.0)	207.3 (170.6 to 253.1)	71.0 (48.8 to 101.5)	43.9 (22.8 to 75.6)	12.1 (9.1 to 17.3)	584.1 (496.6 to 692.0)	362.2 (298.1 to 442.2)	124.0 (85.2 to 177.3)	76.7 (39.8 to 132.2)	21.2 (15.9 to 30.3)
Fiji	2043	340.7 (289.6 to 403.8)	211.1 (173.5 to 258.4)	72.3 (49.6 to 103.4)	44.7 (23.2 to 77.0)	12.6 (9.3 to 18.2)	595.3 (506.1 to 705.6)	368.9 (303.1 to 451.6)	126.3 (86.6 to 180.7)	78.2 (40.6 to 134.6)	21.9 (16.3 to 31.8)
Fiji	2044	347.1 (294.5 to 411.7)	215.0 (176.1 to 263.6)	73.6 (50.4 to 105.3)	45.6 (23.6 to 78.5)	13.0 (9.6 to 18.8)	606.5 (514.6 to 719.3)	375.6 (307.7 to 460.6)	128.5 (88.0 to 184.0)	79.6 (41.3 to 137.2)	22.7 (16.8 to 32.9)
Fiji	2045	353.5 (299.0 to 419.9)	218.8 (178.6 to 269.0)	74.8 (51.2 to 107.2)	46.4 (24.1 to 80.0)	13.5 (9.7 to 20.3)	617.7 (522.4 to 733.8)	382.3 (312.0 to 470.0)	130.7 (89.5 to 187.3)	81.1 (42.1 to 139.8)	23.5 (17.0 to 35.5)
Fiji	2046	359.9 (304.3 to 427.9)	222.6 (181.3 to 274.3)	76.1 (52.1 to 109.2)	47.3 (24.5 to 81.5)	13.9 (10.1 to 21.4)	628.9 (531.8 to 747.7)	388.9 (316.8 to 479.4)	133.0 (91.0 to 190.9)	82.6 (42.8 to 142.4)	24.4 (17.6 to 37.4)
Fiji	2047	366.4 (310.1 to 435.8)	226.4 (183.9 to 279.6)	77.4 (53.0 to 111.3)	48.1 (24.9 to 82.8)	14.4 (10.4 to 22.8)	640.3 (541.9 to 761.5)	395.7 (321.4 to 488.5)	135.2 (92.7 to 194.5)	84.1 (43.6 to 144.6)	25.2 (18.1 to 39.8)
Fiji	2048	372.9 (315.1 to 443.4)	230.3 (186.5 to 284.2)	78.6 (54.0 to 113.3)	49.0 (25.4 to 84.1)	15.0 (10.8 to 24.0)	651.6 (550.6 to 774.8)	402.4 (325.8 to 496.5)	137.4 (94.4 to 198.1)	85.6 (44.3 to 146.9)	26.2 (18.8 to 41.9)
Fiji	2049	379.6 (320.3 to 453.1)	234.2 (189.1 to 289.4)	79.9 (54.9 to 115.1)	49.9 (25.8 to 85.4)	15.5 (11.1 to 25.0)	663.3 (559.7 to 791.8)	409.3 (330.5 to 505.8)	139.7 (96.0 to 201.1)	87.2 (45.1 to 149.2)	27.2 (19.4 to 43.6)
Fiji	2050	386.4 (324.4 to 462.2)	238.3 (191.8 to 295.1)	81.2 (55.8 to 116.8)	50.8 (26.3 to 86.9)	16.1 (11.4 to 26.4)	675.3 (566.8 to 807.7)	416.3 (335.2 to 515.6)	142.0 (97.5 to 204.1)	88.8 (46.0 to 151.9)	28.2 (19.9 to 46.2)

Country	Year	2018 US Dollars per capita					2018 PPP per capita				
		Health spending	Government health spending	Out-of-pocket health spending	Prepaid private health spending	Development assistance for health	Health spending	Government health spending	Out-of-pocket health spending	Prepaid private health spending	Development assistance for health
Finland	1995	2501.5 (2404.6 to 2600.8)	1881.9 (1789.5 to 1968.7)	559.1 (520.7 to 603.8)	60.5 (43.8 to 83.6)	0.0 (0.0 to 0.0)	2275.4 (2187.2 to 2365.7)	1711.8 (1627.7 to 1790.8)	508.6 (473.6 to 549.2)	55.0 (39.9 to 76.0)	0.0 (0.0 to 0.0)
Finland	1996	2563.0 (2488.9 to 2640.8)	1925.6 (1862.7 to 1993.9)	573.3 (541.7 to 607.1)	64.1 (47.7 to 83.7)	0.0 (0.0 to 0.0)	2331.3 (2264.0 to 2402.1)	1751.5 (1694.3 to 1813.7)	521.5 (492.8 to 552.2)	58.3 (43.4 to 76.1)	0.0 (0.0 to 0.0)
Finland	1997	2650.4 (2578.3 to 2721.1)	1997.6 (1936.4 to 2064.6)	585.4 (556.9 to 615.7)	67.3 (52.8 to 84.8)	0.0 (0.0 to 0.0)	2410.8 (2345.2 to 2475.1)	1817.1 (1761.4 to 1878.0)	532.5 (506.6 to 560.1)	61.2 (48.1 to 77.1)	0.0 (0.0 to 0.0)
Finland	1998	2713.7 (2645.6 to 2783.4)	2045.7 (1982.8 to 2110.9)	598.5 (570.3 to 628.9)	69.5 (55.8 to 86.2)	0.0 (0.0 to 0.0)	2468.4 (2406.4 to 2531.8)	1860.8 (1803.6 to 1920.1)	544.4 (518.8 to 572.1)	63.2 (50.8 to 78.4)	0.0 (0.0 to 0.0)
Finland	1999	2794.5 (2728.9 to 2860.9)	2090.5 (2032.1 to 2151.8)	630.9 (603.7 to 659.5)	73.1 (59.8 to 89.5)	0.0 (0.0 to 0.0)	2541.9 (2482.3 to 2602.3)	1901.5 (1848.4 to 1957.3)	573.9 (549.1 to 599.9)	66.5 (54.4 to 81.4)	0.0 (0.0 to 0.0)
Finland	2000	2946.2 (2878.9 to 3015.7)	2196.2 (2138.0 to 2259.4)	672.0 (644.9 to 700.6)	78.0 (65.0 to 93.7)	0.0 (0.0 to 0.0)	2679.9 (2618.7 to 2743.2)	1997.7 (1944.8 to 2055.2)	611.3 (586.6 to 637.3)	70.9 (59.1 to 85.2)	0.0 (0.0 to 0.0)
Finland	2001	3095.8 (3025.3 to 3165.7)	2320.5 (2261.3 to 2383.7)	694.7 (667.8 to 721.6)	80.5 (68.0 to 95.5)	0.0 (0.0 to 0.0)	2816.0 (2751.8 to 2879.6)	2110.8 (2056.9 to 2168.3)	631.9 (607.4 to 656.4)	73.2 (61.9 to 86.8)	0.0 (0.0 to 0.0)
Finland	2002	3275.0 (3209.0 to 3349.8)	2487.6 (2430.1 to 2553.8)	703.9 (677.4 to 728.8)	83.4 (70.9 to 98.6)	0.0 (0.0 to 0.0)	2978.9 (2918.9 to 3047.0)	2262.8 (2210.4 to 2322.9)	640.3 (616.2 to 663.0)	75.9 (64.5 to 89.7)	0.0 (0.0 to 0.0)
Finland	2003	3458.3 (3392.1 to 3526.8)	2673.3 (2611.6 to 2732.8)	698.1 (670.7 to 724.9)	86.9 (74.1 to 102.4)	0.0 (0.0 to 0.0)	3145.7 (3085.5 to 3208.0)	2431.6 (2375.5 to 2485.8)	635.0 (610.0 to 659.4)	79.0 (67.4 to 93.2)	0.0 (0.0 to 0.0)
Finland	2004	3690.9 (3619.9 to 3756.9)	2884.5 (2820.3 to 2947.3)	714.3 (686.1 to 742.8)	92.1 (78.8 to 108.1)	0.0 (0.0 to 0.0)	3357.3 (3292.7 to 3417.4)	2623.8 (2565.4 to 2680.9)	649.7 (624.1 to 675.7)	83.7 (71.7 to 98.3)	0.0 (0.0 to 0.0)
Finland	2005	3826.8 (3757.4 to 3901.1)	2993.6 (2928.4 to 3064.2)	736.5 (709.3 to 763.8)	96.7 (82.4 to 112.6)	0.0 (0.0 to 0.0)	3480.9 (3417.8 to 3548.5)	2723.0 (2663.7 to 2787.2)	670.0 (645.2 to 694.8)	87.9 (75.0 to 102.4)	0.0 (0.0 to 0.0)
Finland	2006	3962.5 (3887.6 to 4041.1)	3088.1 (3022.7 to 3161.0)	772.7 (744.7 to 802.9)	101.7 (86.8 to 117.8)	0.0 (0.0 to 0.0)	3604.3 (3536.2 to 3675.8)	2809.0 (2749.5 to 2875.2)	702.8 (677.4 to 730.3)	92.5 (79.0 to 107.2)	0.0 (0.0 to 0.0)
Finland	2007	4142.4 (4065.1 to 4218.1)	3224.3 (3159.5 to 3291.7)	810.1 (783.2 to 839.0)	107.9 (92.5 to 125.8)	0.0 (0.0 to 0.0)	3768.0 (3697.7 to 3836.8)	2932.9 (2873.9 to 2994.1)	736.9 (712.4 to 763.1)	98.2 (84.1 to 114.4)	0.0 (0.0 to 0.0)
Finland	2008	4274.0 (4198.6 to 4348.3)	3322.9 (3256.6 to 3391.6)	835.7 (807.7 to 865.0)	115.4 (99.7 to 133.2)	0.0 (0.0 to 0.0)	3887.7 (3819.1 to 3955.3)	3022.5 (2962.3 to 3085.0)	760.1 (734.7 to 786.8)	105.0 (90.7 to 121.2)	0.0 (0.0 to 0.0)
Finland	2009	4187.6 (4115.7 to 4260.7)	3240.1 (3175.2 to 3305.9)	830.7 (801.9 to 857.3)	116.8 (101.2 to 134.7)	0.0 (0.0 to 0.0)	3809.1 (3743.7 to 3875.6)	2947.3 (2888.2 to 3007.1)	755.6 (729.4 to 779.8)	106.2 (92.1 to 122.5)	0.0 (0.0 to 0.0)
Finland	2010	4345.0 (4267.3 to 4424.1)	3366.8 (3300.1 to 3437.4)	855.2 (826.9 to 884.0)	123.1 (106.4 to 141.8)	0.0 (0.0 to 0.0)	3952.2 (3881.6 to 4024.2)	3062.4 (3001.8 to 3126.7)	777.9 (752.2 to 804.1)	111.9 (96.8 to 128.9)	0.0 (0.0 to 0.0)
Finland	2011	4509.0 (4429.1 to 4583.1)	3511.8 (3445.1 to 3587.0)	867.5 (838.7 to 898.6)	129.6 (111.5 to 147.6)	0.0 (0.0 to 0.0)	4101.4 (4028.8 to 4168.9)	3194.4 (3133.7 to 3262.8)	789.1 (762.9 to 817.4)	117.9 (101.5 to 134.3)	0.0 (0.0 to 0.0)
Finland	2012	4561.0 (4479.7 to 4637.3)	3564.6 (3495.3 to 3633.9)	863.7 (835.2 to 893.1)	132.7 (114.5 to 152.3)	0.0 (0.0 to 0.0)	4148.8 (4074.8 to 4218.1)	3242.4 (3179.4 to 3305.5)	785.7 (759.7 to 812.3)	120.7 (104.1 to 138.5)	0.0 (0.0 to 0.0)
Finland	2013	4591.5 (4512.8 to 4673.7)	3588.9 (3519.3 to 3660.8)	869.1 (840.9 to 899.6)	133.4 (116.3 to 151.9)	0.0 (0.0 to 0.0)	4176.5 (4104.9 to 4251.3)	3264.6 (3201.2 to 3329.9)	790.6 (764.9 to 818.3)	121.4 (105.7 to 138.2)	0.0 (0.0 to 0.0)
Finland	2014	4593.6 (4515.8 to 4673.3)	3581.6 (3507.4 to 3648.5)	880.4 (851.6 to 912.3)	131.6 (113.8 to 151.2)	0.0 (0.0 to 0.0)	4178.4 (4107.6 to 4250.9)	3257.9 (3190.3 to 3318.7)	800.8 (774.6 to 829.9)	119.7 (103.5 to 137.5)	0.0 (0.0 to 0.0)
Finland	2015	4632.8 (4553.5 to 4708.9)	3598.4 (3528.2 to 3670.0)	912.5 (882.1 to 944.3)	121.9 (103.2 to 143.8)	0.0 (0.0 to 0.0)	4214.0 (4142.0 to 4283.3)	3273.1 (3209.3 to 3338.3)	830.0 (802.4 to 858.9)	110.9 (93.9 to 130.8)	0.0 (0.0 to 0.0)

Country	Year	2018 US Dollars per capita					2018 PPP per capita				
		Health spending	Government health spending	Out-of-pocket health spending	Prepaid private health spending	Development assistance for health	Health spending	Government health spending	Out-of-pocket health spending	Prepaid private health spending	Development assistance for health
Finland	2016	4656.2 (4550.3 to 4763.9)	3601.6 (3506.8 to 3701.7)	940.8 (897.4 to 984.9)	113.7 (91.5 to 137.9)	0.0 (0.0 to 0.0)	4235.4 (4139.1 to 4333.3)	3276.1 (3189.8 to 3367.1)	855.8 (816.3 to 895.9)	103.5 (83.3 to 125.4)	0.0 (0.0 to 0.0)
Finland	2017	4713.7 (4602.0 to 4830.1)	3633.8 (3540.7 to 3733.5)	961.9 (915.0 to 1009.0)	118.1 (94.8 to 143.3)	0.0 (0.0 to 0.0)	4287.7 (4186.1 to 4393.6)	3305.3 (3220.7 to 3396.0)	874.9 (832.3 to 917.8)	107.4 (86.2 to 130.3)	0.0 (0.0 to 0.0)
Finland	2018	4769.5 (4653.5 to 4886.3)	3674.9 (3579.0 to 3778.7)	975.1 (926.1 to 1023.0)	119.5 (96.1 to 144.9)	0.0 (0.0 to 0.0)	4338.4 (4232.9 to 4444.7)	3342.7 (3255.5 to 3437.2)	886.9 (842.4 to 930.5)	108.7 (87.5 to 131.8)	0.0 (0.0 to 0.0)
Finland	2019	4823.2 (4705.2 to 4945.3)	3714.0 (3617.3 to 3822.6)	988.1 (935.3 to 1039.3)	121.0 (97.1 to 146.6)	0.0 (0.0 to 0.0)	4387.2 (4279.9 to 4498.4)	3378.3 (3290.4 to 3477.1)	898.8 (850.8 to 945.3)	110.0 (88.3 to 133.3)	0.0 (0.0 to 0.0)
Finland	2020	4878.9 (4756.5 to 5006.1)	3754.8 (3653.9 to 3866.0)	1001.6 (947.9 to 1057.4)	122.5 (98.4 to 148.4)	0.0 (0.0 to 0.0)	4437.9 (4326.6 to 4553.6)	3415.4 (3323.6 to 3516.5)	911.0 (862.2 to 961.8)	111.4 (89.5 to 135.0)	0.0 (0.0 to 0.0)
Finland	2021	4935.3 (4808.3 to 5064.2)	3796.0 (3690.0 to 3904.8)	1015.3 (959.9 to 1074.0)	124.1 (99.8 to 150.2)	0.0 (0.0 to 0.0)	4489.3 (4373.7 to 4606.4)	3452.9 (3356.5 to 3551.9)	923.5 (873.1 to 977.0)	112.9 (90.8 to 136.6)	0.0 (0.0 to 0.0)
Finland	2022	4994.9 (4862.4 to 5133.1)	3839.6 (3730.6 to 3954.8)	1029.5 (974.5 to 1091.1)	125.7 (101.2 to 152.2)	0.0 (0.0 to 0.0)	4543.4 (4422.9 to 4669.2)	3492.6 (3393.4 to 3597.4)	936.5 (886.4 to 992.5)	114.4 (92.0 to 138.5)	0.0 (0.0 to 0.0)
Finland	2023	5059.4 (4921.1 to 5200.3)	3887.5 (3771.5 to 4008.0)	1044.4 (984.5 to 1108.8)	127.5 (102.2 to 154.8)	0.0 (0.0 to 0.0)	4602.1 (4476.3 to 4730.3)	3536.1 (3430.6 to 3645.7)	950.0 (895.5 to 1008.6)	116.0 (93.0 to 140.8)	0.0 (0.0 to 0.0)
Finland	2024	5127.6 (4984.9 to 5278.3)	3938.2 (3816.4 to 4069.4)	1059.9 (999.0 to 1129.0)	129.4 (103.3 to 157.4)	0.0 (0.0 to 0.0)	4664.1 (4534.3 to 4801.2)	3582.3 (3471.5 to 3701.5)	964.1 (908.7 to 1026.9)	117.7 (94.0 to 143.2)	0.0 (0.0 to 0.0)
Finland	2025	5195.3 (5046.5 to 5353.0)	3988.1 (3866.4 to 4124.1)	1075.9 (1007.1 to 1149.5)	131.3 (104.6 to 159.6)	0.0 (0.0 to 0.0)	4725.7 (4590.4 to 4869.2)	3627.7 (3516.9 to 3751.4)	978.6 (916.0 to 1045.6)	119.4 (95.2 to 145.2)	0.0 (0.0 to 0.0)
Finland	2026	5265.1 (5106.8 to 5431.4)	4039.9 (3917.1 to 4177.3)	1092.0 (1016.2 to 1174.6)	133.3 (106.2 to 162.3)	0.0 (0.0 to 0.0)	4789.2 (4645.2 to 4940.5)	3674.7 (3563.0 to 3799.8)	993.3 (924.3 to 1068.5)	121.2 (96.6 to 147.6)	0.0 (0.0 to 0.0)
Finland	2027	5337.6 (5171.7 to 5512.0)	4093.7 (3961.1 to 4235.2)	1108.7 (1025.0 to 1204.1)	135.3 (107.9 to 164.7)	0.0 (0.0 to 0.0)	4855.2 (4704.3 to 5013.8)	3723.7 (3603.1 to 3852.4)	1008.5 (932.4 to 1095.2)	123.1 (98.1 to 149.8)	0.0 (0.0 to 0.0)
Finland	2028	5404.4 (5225.6 to 5594.2)	4142.5 (3999.0 to 4291.8)	1124.7 (1029.2 to 1233.1)	137.2 (109.6 to 166.8)	0.0 (0.0 to 0.0)	4915.9 (4753.3 to 5088.6)	3768.1 (3637.6 to 3903.8)	1023.1 (936.1 to 1121.7)	124.8 (99.7 to 151.7)	0.0 (0.0 to 0.0)
Finland	2029	5467.6 (5267.8 to 5671.4)	4188.2 (4034.2 to 4347.6)	1140.5 (1031.2 to 1261.9)	138.9 (111.0 to 169.6)	0.0 (0.0 to 0.0)	4973.4 (4791.7 to 5158.8)	3809.7 (3669.5 to 3954.7)	1037.4 (938.0 to 1147.8)	126.4 (101.0 to 154.3)	0.0 (0.0 to 0.0)
Finland	2030	5533.9 (5323.8 to 5758.6)	4236.5 (4075.1 to 4404.4)	1156.6 (1033.2 to 1295.2)	140.8 (112.6 to 172.0)	0.0 (0.0 to 0.0)	5033.7 (4842.6 to 5238.1)	3853.6 (3706.8 to 4006.3)	1052.0 (939.8 to 1178.1)	128.1 (102.5 to 156.5)	0.0 (0.0 to 0.0)
Finland	2031	5605.7 (5381.6 to 5845.5)	4289.9 (4115.8 to 4470.1)	1173.0 (1037.6 to 1332.0)	142.8 (114.5 to 174.7)	0.0 (0.0 to 0.0)	5099.0 (4895.2 to 5317.2)	3902.1 (3743.8 to 4066.1)	1067.0 (943.8 to 1211.6)	129.9 (104.1 to 158.9)	0.0 (0.0 to 0.0)
Finland	2032	5680.8 (5435.9 to 5944.7)	4346.0 (4159.2 to 4538.7)	1189.7 (1041.2 to 1359.6)	145.0 (116.3 to 177.8)	0.0 (0.0 to 0.0)	5167.3 (4944.5 to 5407.4)	3953.2 (3783.3 to 4128.4)	1082.2 (947.0 to 1236.7)	131.9 (105.8 to 161.7)	0.0 (0.0 to 0.0)
Finland	2033	5759.8 (5497.6 to 6046.2)	4405.7 (4206.3 to 4613.8)	1206.9 (1045.5 to 1392.8)	147.2 (118.3 to 181.2)	0.0 (0.0 to 0.0)	5239.2 (5000.7 to 5499.7)	4007.5 (3826.1 to 4196.7)	1097.8 (951.0 to 1266.9)	133.9 (107.6 to 164.8)	0.0 (0.0 to 0.0)
Finland	2034	5841.2 (5569.6 to 6136.4)	4467.5 (4254.3 to 4693.8)	1224.1 (1050.5 to 1426.3)	149.6 (120.0 to 184.0)	0.0 (0.0 to 0.0)	5313.3 (5066.2 to 5581.8)	4063.7 (3869.8 to 4269.5)	1113.5 (955.5 to 1297.4)	136.1 (109.2 to 167.4)	0.0 (0.0 to 0.0)
Finland	2035	5928.2 (5625.6 to 6258.6)	4534.2 (4307.2 to 4779.2)	1241.9 (1055.1 to 1458.8)	152.1 (121.7 to 187.1)	0.0 (0.0 to 0.0)	5392.4 (5117.1 to 5692.9)	4124.3 (3917.9 to 4347.2)	1129.6 (959.8 to 1326.9)	138.4 (110.7 to 170.1)	0.0 (0.0 to 0.0)
Finland	2036	6022.2 (5699.7 to 6370.4)	4607.4 (4362.1 to 4868.4)	1259.9 (1061.9 to 1490.8)	154.9 (123.6 to 190.6)	0.0 (0.0 to 0.0)	5477.9 (5184.5 to 5794.6)	4191.0 (3967.9 to 4428.4)	1146.0 (965.9 to 1356.0)	140.9 (112.5 to 173.4)	0.0 (0.0 to 0.0)

		2018 US Dollars per capita					2018 PPP per capita				
Country	Year	Health spending	Government health spending	Out-of-pocket health spending	Prepaid private health spending	Development assistance for health	Health spending	Government health spending	Out-of-pocket health spending	Prepaid private health spending	Development assistance for health
Finland	2037	6121.0 (5772.9 to 6477.4)	4685.0 (4427.5 to 4963.6)	1278.2 (1067.4 to 1519.6)	157.8 (125.9 to 194.0)	0.0 (0.0 to 0.0)	5567.7 (5251.1 to 5891.9)	4261.5 (4027.3 to 4515.0)	1162.7 (970.9 to 1382.3)	143.5 (114.5 to 176.4)	0.0 (0.0 to 0.0)
Finland	2038	6225.9 (5859.9 to 6627.6)	4767.9 (4489.1 to 5068.3)	1297.1 (1077.0 to 1554.9)	160.9 (128.1 to 198.0)	0.0 (0.0 to 0.0)	5663.2 (5330.2 to 6028.6)	4336.9 (4083.3 to 4610.1)	1179.9 (979.6 to 1414.3)	146.3 (116.5 to 180.1)	0.0 (0.0 to 0.0)
Finland	2039	6329.7 (5945.3 to 6748.5)	4849.8 (4555.1 to 5168.1)	1316.0 (1085.6 to 1584.9)	164.0 (130.5 to 202.1)	0.0 (0.0 to 0.0)	5757.6 (5407.9 to 6138.6)	4411.4 (4143.3 to 4701.0)	1197.1 (987.5 to 1441.6)	149.1 (118.7 to 183.8)	0.0 (0.0 to 0.0)
Finland	2040	6433.2 (6023.1 to 6874.7)	4931.1 (4618.0 to 5262.2)	1335.1 (1095.1 to 1614.0)	167.0 (133.0 to 206.4)	0.0 (0.0 to 0.0)	5851.7 (5478.7 to 6253.3)	4485.4 (4200.6 to 4786.6)	1214.4 (996.1 to 1468.1)	151.9 (121.0 to 187.7)	0.0 (0.0 to 0.0)
Finland	2041	6534.6 (6095.8 to 6989.9)	5010.7 (4675.3 to 5358.1)	1353.9 (1105.7 to 1641.7)	170.1 (135.0 to 210.3)	0.0 (0.0 to 0.0)	5944.0 (5544.9 to 6358.1)	4557.8 (4252.7 to 4873.8)	1231.5 (1005.7 to 1493.3)	154.7 (122.8 to 191.3)	0.0 (0.0 to 0.0)
Finland	2042	6633.3 (6179.7 to 7102.7)	5087.8 (4732.0 to 5459.8)	1372.5 (1117.8 to 1668.0)	173.0 (137.4 to 214.1)	0.0 (0.0 to 0.0)	6033.8 (5621.2 to 6460.7)	4627.9 (4304.3 to 4966.3)	1248.5 (1016.7 to 1517.3)	157.4 (125.0 to 194.8)	0.0 (0.0 to 0.0)
Finland	2043	6729.9 (6242.1 to 7239.9)	5162.9 (4788.4 to 5562.8)	1391.1 (1129.7 to 1695.7)	175.9 (139.2 to 217.8)	0.0 (0.0 to 0.0)	6121.6 (5677.9 to 6585.5)	4696.2 (4355.6 to 5060.0)	1265.3 (1027.6 to 1542.5)	160.0 (126.6 to 198.2)	0.0 (0.0 to 0.0)
Finland	2044	6832.0 (6322.3 to 7355.7)	5242.7 (4848.4 to 5666.4)	1410.3 (1142.7 to 1717.7)	179.0 (141.7 to 222.4)	0.0 (0.0 to 0.0)	6214.5 (5750.9 to 6690.8)	4768.8 (4410.2 to 5154.3)	1282.8 (1039.5 to 1562.4)	162.8 (128.9 to 202.3)	0.0 (0.0 to 0.0)
Finland	2045	6928.1 (6400.6 to 7477.7)	5317.2 (4902.9 to 5771.2)	1429.0 (1156.4 to 1747.2)	181.9 (144.1 to 226.2)	0.0 (0.0 to 0.0)	6301.9 (5822.0 to 6801.8)	4836.6 (4459.8 to 5249.5)	1299.8 (1051.9 to 1589.3)	165.5 (131.1 to 205.7)	0.0 (0.0 to 0.0)
Finland	2046	7025.1 (6475.8 to 7588.7)	5392.6 (4957.0 to 5870.1)	1447.7 (1170.2 to 1776.4)	184.9 (146.5 to 229.5)	0.0 (0.0 to 0.0)	6390.1 (5890.5 to 6902.8)	4905.2 (4508.9 to 5339.5)	1316.8 (1064.4 to 1615.8)	168.1 (133.3 to 208.7)	0.0 (0.0 to 0.0)
Finland	2047	7124.6 (6568.9 to 7710.9)	5470.1 (5011.2 to 5970.7)	1466.7 (1186.0 to 1803.2)	187.9 (148.6 to 234.2)	0.0 (0.0 to 0.0)	6480.7 (5975.2 to 7014.0)	4975.7 (4558.3 to 5431.0)	1334.1 (1078.8 to 1640.2)	170.9 (135.2 to 213.0)	0.0 (0.0 to 0.0)
Finland	2048	7220.7 (6625.9 to 7835.1)	5544.4 (5067.9 to 6071.8)	1485.5 (1199.9 to 1829.7)	190.8 (150.9 to 239.2)	0.0 (0.0 to 0.0)	6568.0 (6027.0 to 7126.9)	5043.2 (4609.8 to 5523.0)	1351.2 (1091.4 to 1664.3)	173.6 (137.3 to 217.6)	0.0 (0.0 to 0.0)
Finland	2049	7315.7 (6699.8 to 7955.4)	5617.6 (5125.9 to 6175.4)	1504.4 (1213.9 to 1858.9)	193.7 (153.3 to 244.0)	0.0 (0.0 to 0.0)	6654.5 (6094.2 to 7236.4)	5109.8 (4662.6 to 5617.2)	1368.4 (1104.2 to 1690.9)	176.2 (139.5 to 221.9)	0.0 (0.0 to 0.0)
Finland	2050	7409.7 (6781.3 to 8067.1)	5689.8 (5175.3 to 6279.2)	1523.4 (1229.3 to 1886.1)	196.6 (155.2 to 247.8)	0.0 (0.0 to 0.0)	6740.0 (6168.3 to 7337.9)	5175.5 (4707.5 to 5711.7)	1385.7 (1118.2 to 1715.6)	178.8 (141.2 to 225.4)	0.0 (0.0 to 0.0)
France	1995	3341.6 (3232.0 to 3456.4)	2650.2 (2564.5 to 2732.9)	246.3 (218.2 to 276.9)	445.1 (380.5 to 524.7)	0.0 (0.0 to 0.0)	3478.1 (3364.1 to 3597.6)	2758.5 (2669.2 to 2844.6)	256.3 (227.1 to 288.2)	463.3 (396.1 to 546.2)	0.0 (0.0 to 0.0)
France	1996	3355.9 (3274.1 to 3438.6)	2661.6 (2597.9 to 2720.6)	240.1 (216.9 to 265.0)	454.2 (401.8 to 513.1)	0.0 (0.0 to 0.0)	3493.0 (3407.9 to 3579.1)	2770.3 (2704.0 to 2831.8)	249.9 (225.8 to 275.8)	472.8 (418.2 to 534.1)	0.0 (0.0 to 0.0)
France	1997	3394.2 (3310.6 to 3479.5)	2692.3 (2628.2 to 2753.8)	238.4 (218.8 to 259.9)	463.5 (414.1 to 516.0)	0.0 (0.0 to 0.0)	3532.9 (3445.9 to 3621.7)	2802.3 (2735.6 to 2866.3)	248.1 (227.8 to 270.5)	482.4 (431.0 to 537.1)	0.0 (0.0 to 0.0)
France	1998	3473.0 (3383.9 to 3559.7)	2751.8 (2686.3 to 2820.8)	243.7 (225.0 to 265.2)	477.5 (424.1 to 531.3)	0.0 (0.0 to 0.0)	3614.9 (3522.2 to 3705.1)	2864.2 (2796.0 to 2936.0)	253.7 (234.1 to 276.0)	497.0 (441.4 to 553.0)	0.0 (0.0 to 0.0)
France	1999	3551.5 (3466.1 to 3637.4)	2807.9 (2747.1 to 2871.8)	253.6 (235.8 to 274.9)	490.0 (437.1 to 545.1)	0.0 (0.0 to 0.0)	3696.7 (3607.8 to 3786.0)	2922.6 (2859.3 to 2989.1)	264.0 (245.4 to 286.1)	510.0 (454.9 to 567.4)	0.0 (0.0 to 0.0)
France	2000	3668.4 (3590.0 to 3747.6)	2898.6 (2837.1 to 2958.4)	264.0 (246.8 to 285.1)	505.8 (455.1 to 559.9)	0.0 (0.0 to 0.0)	3818.3 (3736.7 to 3900.7)	3017.0 (2953.0 to 3079.3)	274.8 (256.9 to 296.7)	526.5 (473.7 to 582.7)	0.0 (0.0 to 0.0)
France	2001	3777.3 (3697.8 to 3854.3)	2984.6 (2924.4 to 3045.1)	272.5 (254.6 to 291.9)	520.2 (473.0 to 572.3)	0.0 (0.0 to 0.0)	3931.7 (3848.9 to 4011.8)	3106.6 (3043.9 to 3169.5)	283.6 (265.1 to 303.8)	541.5 (492.3 to 595.7)	0.0 (0.0 to 0.0)

Country	Year	2018 US Dollars per capita					2018 PPP per capita				
		Health spending	Government health spending	Out-of-pocket health spending	Prepaid private health spending	Development assistance for health	Health spending	Government health spending	Out-of-pocket health spending	Prepaid private health spending	Development assistance for health
France	2002	3891.0 (3813.4 to 3973.9)	3074.0 (3010.9 to 3138.6)	279.7 (261.6 to 298.5)	537.3 (488.8 to 591.8)	0.0 (0.0 to 0.0)	4050.0 (3969.2 to 4136.3)	3199.6 (3133.9 to 3266.8)	291.2 (272.2 to 310.7)	559.3 (508.8 to 616.0)	0.0 (0.0 to 0.0)
France	2003	3928.5 (3848.5 to 4008.8)	3095.9 (3038.9 to 3162.2)	287.1 (269.4 to 306.0)	545.5 (496.9 to 600.4)	0.0 (0.0 to 0.0)	4089.0 (4005.7 to 4172.6)	3222.4 (3163.0 to 3291.4)	298.8 (280.4 to 318.5)	567.8 (517.2 to 625.0)	0.0 (0.0 to 0.0)
France	2004	4053.5 (3972.8 to 4133.5)	3188.0 (3132.6 to 3245.0)	301.9 (283.4 to 321.4)	563.6 (515.1 to 618.3)	0.0 (0.0 to 0.0)	4219.1 (4135.1 to 4302.4)	3318.2 (3260.6 to 3377.6)	314.2 (295.0 to 334.5)	586.7 (536.2 to 643.6)	0.0 (0.0 to 0.0)
France	2005	4120.9 (4039.7 to 4208.1)	3225.9 (3163.4 to 3286.7)	324.9 (306.1 to 344.7)	570.1 (522.1 to 629.2)	0.0 (0.0 to 0.0)	4289.3 (4204.8 to 4380.0)	3357.7 (3292.6 to 3421.0)	338.2 (318.6 to 358.8)	593.4 (543.4 to 654.9)	0.0 (0.0 to 0.0)
France	2006	4201.2 (4113.5 to 4286.3)	3262.6 (3202.3 to 3325.5)	366.2 (345.6 to 387.8)	572.4 (521.5 to 628.6)	0.0 (0.0 to 0.0)	4372.9 (4281.6 to 4461.5)	3395.9 (3333.1 to 3461.4)	381.1 (359.7 to 403.6)	595.8 (542.8 to 654.3)	0.0 (0.0 to 0.0)
France	2007	4306.6 (4222.6 to 4387.5)	3325.7 (3262.0 to 3389.0)	400.6 (377.3 to 423.7)	580.4 (531.6 to 636.5)	0.0 (0.0 to 0.0)	4482.6 (4395.1 to 4566.8)	3461.6 (3395.3 to 3527.5)	416.9 (392.8 to 441.0)	604.1 (553.3 to 662.5)	0.0 (0.0 to 0.0)
France	2008	4394.2 (4309.7 to 4479.3)	3374.4 (3313.2 to 3437.9)	430.2 (406.6 to 454.3)	589.6 (539.6 to 644.0)	0.0 (0.0 to 0.0)	4573.7 (4485.8 to 4662.3)	3512.3 (3448.6 to 3578.4)	447.8 (423.2 to 472.8)	613.7 (561.7 to 670.3)	0.0 (0.0 to 0.0)
France	2009	4442.6 (4358.0 to 4525.3)	3395.7 (3335.1 to 3457.3)	449.0 (427.4 to 471.0)	597.9 (547.9 to 648.7)	0.0 (0.0 to 0.0)	4624.1 (4536.1 to 4710.2)	3534.4 (3471.4 to 3598.5)	467.4 (444.9 to 490.3)	622.3 (570.3 to 675.2)	0.0 (0.0 to 0.0)
France	2010	4559.7 (4475.8 to 4643.9)	3477.4 (3414.0 to 3541.3)	463.6 (441.5 to 487.3)	618.7 (568.1 to 670.2)	0.0 (0.0 to 0.0)	4746.0 (4658.7 to 4833.6)	3619.5 (3553.5 to 3686.0)	482.5 (459.6 to 507.3)	644.0 (591.3 to 697.5)	0.0 (0.0 to 0.0)
France	2011	4649.7 (4566.6 to 4735.1)	3537.0 (3470.6 to 3604.6)	472.1 (448.2 to 495.8)	640.6 (586.8 to 695.3)	0.0 (0.0 to 0.0)	4839.7 (4753.2 to 4928.6)	3681.5 (3612.4 to 3751.9)	491.4 (466.5 to 516.0)	666.7 (610.8 to 723.7)	0.0 (0.0 to 0.0)
France	2012	4670.5 (4579.2 to 4755.3)	3546.5 (3486.2 to 3605.9)	472.5 (449.4 to 497.5)	651.4 (595.6 to 705.2)	0.0 (0.0 to 0.0)	4861.3 (4766.3 to 4949.6)	3691.5 (3628.6 to 3753.2)	491.8 (467.8 to 517.8)	678.1 (620.0 to 734.0)	0.0 (0.0 to 0.0)
France	2013	4732.9 (4643.5 to 4822.9)	3600.0 (3538.3 to 3665.0)	473.4 (450.6 to 497.8)	659.5 (602.1 to 717.2)	0.0 (0.0 to 0.0)	4926.3 (4833.3 to 5019.9)	3747.1 (3682.9 to 3814.8)	492.7 (469.0 to 518.1)	686.5 (626.7 to 746.5)	0.0 (0.0 to 0.0)
France	2014	4773.9 (4687.7 to 4862.2)	3656.0 (3596.2 to 3719.9)	473.1 (451.3 to 497.7)	644.8 (589.3 to 703.9)	0.0 (0.0 to 0.0)	4969.0 (4879.2 to 5060.9)	3805.4 (3743.1 to 3871.9)	492.5 (469.7 to 518.0)	671.2 (613.3 to 732.7)	0.0 (0.0 to 0.0)
France	2015	4831.8 (4739.4 to 4929.0)	3764.4 (3695.2 to 3831.6)	472.4 (449.0 to 499.3)	595.0 (538.5 to 656.1)	0.0 (0.0 to 0.0)	5029.2 (4933.0 to 5130.4)	3918.2 (3846.2 to 3988.2)	491.7 (467.3 to 519.7)	619.3 (560.5 to 682.9)	0.0 (0.0 to 0.0)
France	2016	4945.5 (4825.5 to 5063.2)	3984.6 (3891.9 to 4069.7)	474.0 (443.5 to 507.4)	486.9 (419.6 to 560.3)	0.0 (0.0 to 0.0)	5147.6 (5022.7 to 5270.1)	4147.4 (4051.0 to 4236.0)	493.4 (461.7 to 528.1)	506.8 (436.7 to 583.2)	0.0 (0.0 to 0.0)
France	2017	5050.7 (4931.3 to 5169.7)	4053.5 (3960.9 to 4143.0)	483.8 (450.5 to 519.1)	513.4 (442.7 to 591.7)	0.0 (0.0 to 0.0)	5257.1 (5132.8 to 5380.9)	4219.2 (4122.8 to 4312.3)	503.5 (468.9 to 540.3)	534.4 (460.8 to 615.9)	0.0 (0.0 to 0.0)
France	2018	5114.5 (4992.4 to 5234.5)	4105.4 (4007.8 to 4199.1)	491.6 (457.6 to 529.0)	517.5 (446.1 to 596.6)	0.0 (0.0 to 0.0)	5323.5 (5196.4 to 5448.4)	4273.2 (4171.6 to 4370.7)	511.7 (476.3 to 550.6)	538.6 (464.4 to 621.0)	0.0 (0.0 to 0.0)
France	2019	5180.7 (5049.5 to 5306.7)	4156.5 (4049.9 to 4261.7)	499.7 (465.7 to 537.3)	524.5 (451.8 to 605.8)	0.0 (0.0 to 0.0)	5392.4 (5255.8 to 5523.5)	4326.3 (4215.4 to 4435.8)	520.2 (484.8 to 559.2)	545.9 (470.2 to 630.6)	0.0 (0.0 to 0.0)
France	2020	5249.3 (5110.1 to 5390.6)	4210.0 (4094.2 to 4331.0)	508.0 (473.0 to 547.0)	531.3 (457.3 to 613.9)	0.0 (0.0 to 0.0)	5463.8 (5319.0 to 5610.9)	4382.0 (4261.5 to 4508.0)	528.8 (492.3 to 569.3)	553.0 (475.9 to 639.0)	0.0 (0.0 to 0.0)
France	2021	5310.8 (5165.1 to 5457.4)	4256.7 (4134.5 to 4379.9)	516.2 (480.1 to 555.6)	537.9 (462.0 to 621.7)	0.0 (0.0 to 0.0)	5527.8 (5376.2 to 5680.4)	4430.6 (4303.4 to 4558.8)	537.3 (499.7 to 578.3)	559.9 (480.9 to 647.1)	0.0 (0.0 to 0.0)
France	2022	5374.0 (5223.3 to 5528.2)	4304.3 (4179.0 to 4432.9)	524.7 (486.4 to 567.2)	545.0 (467.8 to 628.3)	0.0 (0.0 to 0.0)	5593.6 (5436.8 to 5754.1)	4480.2 (4349.7 to 4614.1)	546.1 (506.2 to 590.4)	567.2 (486.9 to 654.0)	0.0 (0.0 to 0.0)

Country	Year	2018 US Dollars per capita					2018 PPP per capita				
		Health spending	Government health spending	Out-of-pocket health spending	Prepaid private health spending	Development assistance for health	Health spending	Government health spending	Out-of-pocket health spending	Prepaid private health spending	Development assistance for health
France	2023	5425.7 (5258.9 to 5581.0)	4342.2 (4218.8 to 4476.0)	532.8 (493.3 to 578.9)	550.8 (472.8 to 635.6)	0.0 (0.0 to 0.0)	5647.4 (5473.8 to 5809.0)	4519.6 (4391.2 to 4658.9)	554.5 (513.4 to 602.6)	573.3 (492.1 to 661.5)	0.0 (0.0 to 0.0)
France	2024	5475.6 (5312.9 to 5642.4)	4379.2 (4247.4 to 4514.3)	540.6 (499.6 to 587.8)	555.8 (477.1 to 641.3)	0.0 (0.0 to 0.0)	5699.4 (5530.0 to 5873.0)	4558.1 (4421.0 to 4698.8)	562.7 (520.0 to 611.8)	578.5 (496.6 to 667.5)	0.0 (0.0 to 0.0)
France	2025	5525.5 (5359.9 to 5694.0)	4416.0 (4271.2 to 4562.2)	548.5 (506.1 to 595.6)	560.9 (480.2 to 647.6)	0.0 (0.0 to 0.0)	5751.2 (5578.9 to 5926.7)	4596.4 (4445.7 to 4748.6)	571.0 (526.8 to 619.9)	583.9 (499.8 to 674.0)	0.0 (0.0 to 0.0)
France	2026	5579.1 (5404.8 to 5755.5)	4455.6 (4305.5 to 4611.1)	556.8 (511.2 to 606.3)	566.7 (485.3 to 655.8)	0.0 (0.0 to 0.0)	5807.1 (5625.6 to 5990.7)	4637.7 (4481.4 to 4799.5)	579.6 (532.1 to 631.1)	589.8 (505.1 to 682.6)	0.0 (0.0 to 0.0)
France	2027	5635.0 (5447.7 to 5821.3)	4497.1 (4329.7 to 4658.2)	565.3 (516.4 to 620.3)	572.6 (491.2 to 663.1)	0.0 (0.0 to 0.0)	5865.3 (5670.3 to 6059.2)	4680.9 (4506.6 to 4848.5)	588.4 (537.5 to 645.7)	596.0 (511.3 to 690.1)	0.0 (0.0 to 0.0)
France	2028	5690.8 (5486.0 to 5884.3)	4538.5 (4369.4 to 4707.9)	573.8 (520.2 to 635.9)	578.6 (495.2 to 671.4)	0.0 (0.0 to 0.0)	5923.4 (5710.2 to 6124.8)	4723.9 (4548.0 to 4900.2)	597.3 (541.4 to 661.9)	602.2 (515.4 to 698.8)	0.0 (0.0 to 0.0)
France	2029	5748.8 (5521.1 to 5963.9)	4581.6 (4396.2 to 4759.6)	582.5 (522.6 to 650.0)	584.8 (501.5 to 678.9)	0.0 (0.0 to 0.0)	5983.7 (5746.7 to 6207.6)	4768.8 (4575.8 to 4954.1)	606.3 (544.0 to 676.6)	608.7 (522.0 to 706.6)	0.0 (0.0 to 0.0)
France	2030	5809.9 (5568.3 to 6038.0)	4627.1 (4435.3 to 4821.1)	591.3 (525.6 to 666.8)	591.4 (507.6 to 688.5)	0.0 (0.0 to 0.0)	6047.3 (5795.8 to 6284.8)	4816.2 (4616.5 to 5018.0)	615.5 (547.1 to 694.1)	615.6 (528.3 to 716.6)	0.0 (0.0 to 0.0)
France	2031	5873.3 (5623.8 to 6122.1)	4674.1 (4464.1 to 4879.6)	600.4 (528.8 to 684.2)	598.9 (514.3 to 699.1)	0.0 (0.0 to 0.0)	6113.3 (5853.6 to 6372.2)	4865.1 (4646.5 to 5079.0)	624.9 (550.4 to 712.2)	623.3 (535.3 to 727.7)	0.0 (0.0 to 0.0)
France	2032	5937.8 (5678.3 to 6199.3)	4721.8 (4491.8 to 4941.2)	609.5 (530.3 to 699.1)	606.5 (521.2 to 708.1)	0.0 (0.0 to 0.0)	6180.5 (5910.3 to 6452.6)	4914.7 (4675.3 to 5143.1)	634.4 (552.0 to 727.7)	631.3 (542.5 to 737.1)	0.0 (0.0 to 0.0)
France	2033	6002.2 (5728.8 to 6280.1)	4769.4 (4533.4 to 5010.3)	618.6 (532.9 to 716.6)	614.3 (527.8 to 717.9)	0.0 (0.0 to 0.0)	6247.5 (5962.8 to 6536.7)	4964.2 (4718.6 to 5215.0)	643.9 (554.7 to 745.9)	639.4 (549.4 to 747.2)	0.0 (0.0 to 0.0)
France	2034	6068.1 (5776.1 to 6364.0)	4818.3 (4566.3 to 5075.0)	627.8 (537.3 to 733.0)	622.1 (534.1 to 728.2)	0.0 (0.0 to 0.0)	6316.1 (6012.1 to 6624.1)	5015.2 (4752.9 to 5282.3)	653.4 (559.2 to 763.0)	647.5 (556.0 to 758.0)	0.0 (0.0 to 0.0)
France	2035	6134.1 (5830.4 to 6467.0)	4867.3 (4604.0 to 5141.0)	636.9 (540.9 to 749.5)	629.9 (540.3 to 738.1)	0.0 (0.0 to 0.0)	6384.8 (6068.7 to 6731.3)	5066.2 (4792.2 to 5351.1)	662.9 (563.0 to 780.1)	655.6 (562.4 to 768.3)	0.0 (0.0 to 0.0)
France	2036	6198.1 (5878.3 to 6545.6)	4913.9 (4634.3 to 5222.0)	646.0 (542.7 to 767.9)	638.2 (546.0 to 748.3)	0.0 (0.0 to 0.0)	6451.3 (6118.5 to 6813.1)	5114.7 (4823.7 to 5435.3)	672.4 (564.9 to 799.3)	664.3 (568.3 to 778.9)	0.0 (0.0 to 0.0)
France	2037	6261.7 (5934.2 to 6633.6)	4960.2 (4662.1 to 5275.3)	655.0 (545.9 to 784.4)	646.4 (551.6 to 758.7)	0.0 (0.0 to 0.0)	6517.6 (6176.7 to 6904.6)	5162.9 (4852.6 to 5490.9)	681.8 (568.3 to 816.5)	672.8 (574.1 to 789.7)	0.0 (0.0 to 0.0)
France	2038	6325.6 (5977.8 to 6721.4)	5006.8 (4700.1 to 5350.8)	664.0 (549.6 to 799.9)	654.7 (558.8 to 769.2)	0.0 (0.0 to 0.0)	6584.0 (6222.0 to 6996.1)	5211.4 (4892.1 to 5569.4)	691.2 (572.1 to 832.6)	681.5 (581.6 to 800.6)	0.0 (0.0 to 0.0)
France	2039	6392.8 (6030.0 to 6792.7)	5056.1 (4730.0 to 5413.9)	673.2 (552.5 to 815.8)	663.6 (565.2 to 780.4)	0.0 (0.0 to 0.0)	6654.1 (6276.4 to 7070.3)	5262.7 (4923.3 to 5635.1)	700.7 (575.1 to 849.1)	690.7 (588.3 to 812.3)	0.0 (0.0 to 0.0)
France	2040	6459.0 (6076.0 to 6857.9)	5104.0 (4767.5 to 5483.7)	682.3 (557.8 to 830.5)	672.7 (572.3 to 792.6)	0.0 (0.0 to 0.0)	6722.9 (6324.3 to 7138.1)	5312.5 (4962.3 to 5707.8)	710.2 (580.6 to 864.4)	700.2 (595.6 to 825.0)	0.0 (0.0 to 0.0)
France	2041	6526.4 (6137.0 to 6927.5)	5152.1 (4799.1 to 5543.5)	691.5 (564.1 to 845.1)	682.8 (578.9 to 806.2)	0.0 (0.0 to 0.0)	6793.1 (6387.7 to 7210.6)	5362.7 (4995.2 to 5770.1)	719.8 (587.2 to 879.7)	710.7 (602.6 to 839.2)	0.0 (0.0 to 0.0)
France	2042	6595.2 (6193.6 to 7031.0)	5201.1 (4825.2 to 5615.1)	700.7 (570.8 to 858.2)	693.4 (586.0 to 820.5)	0.0 (0.0 to 0.0)	6864.7 (6446.7 to 7318.3)	5413.6 (5022.3 to 5844.6)	729.3 (594.2 to 893.3)	721.8 (610.0 to 854.0)	0.0 (0.0 to 0.0)
France	2043	6664.3 (6219.3 to 7131.5)	5249.9 (4862.4 to 5680.2)	709.9 (576.1 to 870.3)	704.5 (593.8 to 835.1)	0.0 (0.0 to 0.0)	6936.7 (6473.5 to 7423.0)	5464.4 (5061.1 to 5912.3)	738.9 (599.6 to 905.9)	733.3 (618.1 to 869.2)	0.0 (0.0 to 0.0)

Country	Year	2018 US Dollars per capita					2018 PPP per capita				
		Health spending	Government health spending	Out-of-pocket health spending	Prepaid private health spending	Development assistance for health	Health spending	Government health spending	Out-of-pocket health spending	Prepaid private health spending	Development assistance for health
France	2044	6734.4 (6258.8 to 7210.0)	5299.6 (4881.7 to 5761.2)	719.1 (582.6 to 882.3)	715.7 (601.8 to 847.9)	0.0 (0.0 to 0.0)	7009.6 (6514.6 to 7504.6)	5516.2 (5081.2 to 5996.6)	748.5 (606.4 to 918.4)	745.0 (626.4 to 882.5)	0.0 (0.0 to 0.0)
France	2045	6804.1 (6335.6 to 7287.8)	5349.2 (4908.5 to 5823.3)	728.2 (588.5 to 895.5)	726.7 (610.1 to 861.8)	0.0 (0.0 to 0.0)	7082.1 (6594.5 to 7585.6)	5567.8 (5109.1 to 6061.3)	757.9 (612.6 to 932.1)	756.4 (635.0 to 897.0)	0.0 (0.0 to 0.0)
France	2046	6874.2 (6375.4 to 7400.8)	5398.8 (4944.7 to 5917.6)	737.2 (594.8 to 908.0)	738.2 (620.0 to 877.2)	0.0 (0.0 to 0.0)	7155.1 (6635.9 to 7703.2)	5619.4 (5146.8 to 6159.4)	767.4 (619.1 to 945.1)	768.3 (645.4 to 913.0)	0.0 (0.0 to 0.0)
France	2047	6943.2 (6415.8 to 7501.0)	5447.5 (4983.5 to 5992.1)	746.2 (601.4 to 917.1)	749.5 (626.6 to 893.5)	0.0 (0.0 to 0.0)	7226.9 (6678.0 to 7807.6)	5670.1 (5187.1 to 6237.0)	776.6 (626.0 to 954.6)	780.1 (652.2 to 930.0)	0.0 (0.0 to 0.0)
France	2048	7011.7 (6458.7 to 7578.2)	5495.7 (4989.5 to 6065.6)	755.1 (609.8 to 925.5)	760.8 (632.7 to 909.8)	0.0 (0.0 to 0.0)	7298.2 (6722.6 to 7887.9)	5720.3 (5193.4 to 6313.4)	786.0 (634.8 to 963.3)	791.9 (658.5 to 947.0)	0.0 (0.0 to 0.0)
France	2049	7081.8 (6512.6 to 7685.3)	5545.4 (5025.5 to 6121.2)	764.3 (616.0 to 935.0)	772.2 (641.0 to 925.8)	0.0 (0.0 to 0.0)	7371.2 (6778.7 to 7999.3)	5772.0 (5230.8 to 6371.3)	795.5 (641.1 to 973.2)	803.7 (667.2 to 963.7)	0.0 (0.0 to 0.0)
France	2050	7153.6 (6557.9 to 7776.3)	5596.5 (5061.2 to 6190.5)	773.5 (623.3 to 945.9)	783.6 (649.6 to 941.3)	0.0 (0.0 to 0.0)	7445.9 (6825.8 to 8094.0)	5825.1 (5268.0 to 6443.4)	805.1 (648.7 to 984.6)	815.6 (676.2 to 979.8)	0.0 (0.0 to 0.0)
Gabon	1995	334.4 (297.2 to 373.7)	110.0 (92.9 to 129.4)	198.2 (167.2 to 232.0)	26.0 (14.3 to 43.1)	0.2 (0.2 to 0.2)	772.1 (686.2 to 862.7)	254.0 (214.5 to 298.7)	457.7 (386.0 to 535.7)	59.9 (33.0 to 99.6)	0.4 (0.4 to 0.4)
Gabon	1996	342.7 (308.3 to 379.5)	115.7 (98.9 to 134.3)	198.0 (170.2 to 226.7)	26.4 (15.0 to 43.2)	2.6 (2.6 to 2.6)	791.2 (711.8 to 876.2)	267.1 (228.2 to 310.1)	457.1 (393.0 to 523.4)	61.0 (34.6 to 99.8)	6.0 (6.0 to 6.0)
Gabon	1997	350.1 (318.1 to 383.7)	120.2 (103.4 to 137.4)	197.1 (172.6 to 223.0)	26.7 (15.4 to 43.9)	6.1 (6.1 to 6.1)	808.4 (734.5 to 886.0)	277.5 (238.7 to 317.2)	455.1 (398.5 to 514.8)	61.7 (35.5 to 101.3)	14.1 (14.1 to 14.1)
Gabon	1998	369.6 (338.5 to 406.6)	125.8 (109.8 to 142.6)	207.3 (183.6 to 233.4)	28.0 (16.5 to 44.7)	8.5 (8.5 to 8.5)	853.4 (781.6 to 938.8)	290.5 (253.6 to 329.2)	478.6 (424.0 to 538.9)	64.6 (38.2 to 103.1)	19.7 (19.7 to 19.7)
Gabon	1999	323.9 (295.7 to 356.6)	112.6 (99.0 to 127.6)	184.2 (162.6 to 207.2)	25.1 (14.9 to 40.4)	2.0 (2.0 to 2.0)	747.8 (682.7 to 823.3)	260.0 (228.5 to 294.6)	425.2 (375.4 to 478.3)	58.0 (34.4 to 93.3)	4.6 (4.6 to 4.6)
Gabon	2000	296.2 (269.3 to 325.0)	108.5 (94.6 to 123.9)	162.1 (143.3 to 184.0)	23.4 (13.8 to 37.6)	2.2 (2.2 to 2.2)	683.9 (621.7 to 750.5)	250.6 (218.5 to 286.0)	374.2 (330.8 to 424.9)	54.1 (31.8 to 86.9)	5.0 (5.0 to 5.0)
Gabon	2001	284.9 (258.1 to 315.3)	107.5 (93.4 to 123.3)	152.2 (133.9 to 174.7)	22.8 (13.5 to 36.6)	2.4 (2.4 to 2.4)	657.8 (596.0 to 727.9)	248.2 (215.6 to 284.6)	351.5 (309.1 to 403.3)	52.7 (31.2 to 84.5)	5.5 (5.5 to 5.5)
Gabon	2002	281.0 (254.2 to 313.1)	104.8 (91.0 to 120.4)	148.9 (129.7 to 171.5)	22.5 (13.3 to 35.4)	4.8 (4.8 to 4.8)	648.9 (586.9 to 722.8)	242.0 (210.1 to 278.0)	343.8 (299.6 to 396.0)	51.9 (30.8 to 81.7)	11.1 (11.1 to 11.1)
Gabon	2003	280.5 (253.8 to 314.0)	107.3 (93.1 to 123.9)	146.9 (127.8 to 170.0)	22.6 (13.2 to 35.0)	3.7 (3.7 to 3.7)	647.6 (586.1 to 725.0)	247.8 (214.9 to 286.0)	339.1 (295.0 to 392.4)	52.1 (30.5 to 80.9)	8.5 (8.5 to 8.5)
Gabon	2004	273.8 (248.2 to 304.1)	104.8 (90.3 to 121.3)	141.1 (121.3 to 163.2)	21.5 (12.5 to 32.9)	6.3 (6.3 to 6.3)	632.1 (573.0 to 702.1)	241.9 (208.5 to 280.2)	325.8 (280.2 to 376.8)	49.7 (28.8 to 76.1)	14.6 (14.6 to 14.6)
Gabon	2005	269.8 (244.3 to 301.1)	104.1 (88.6 to 121.0)	138.5 (119.1 to 161.2)	21.0 (12.4 to 32.3)	6.2 (6.2 to 6.2)	623.0 (564.0 to 695.2)	240.4 (204.7 to 279.3)	319.7 (275.0 to 372.2)	48.5 (28.6 to 74.6)	14.4 (14.4 to 14.4)
Gabon	2006	261.1 (236.4 to 289.9)	99.1 (84.8 to 115.7)	131.8 (114.0 to 152.0)	19.9 (11.7 to 30.4)	10.3 (10.3 to 10.3)	602.9 (545.9 to 669.4)	228.9 (195.8 to 267.0)	304.4 (263.3 to 350.8)	45.8 (27.1 to 70.3)	23.8 (23.8 to 23.8)
Gabon	2007	257.8 (232.4 to 287.1)	101.9 (87.5 to 118.8)	128.7 (110.9 to 149.4)	20.0 (11.7 to 30.4)	7.3 (7.3 to 7.3)	595.3 (536.5 to 662.8)	235.3 (202.0 to 274.3)	297.1 (256.0 to 345.0)	46.1 (27.0 to 70.3)	16.8 (16.8 to 16.8)
Gabon	2008	246.5 (220.9 to 272.8)	103.0 (88.8 to 120.4)	118.6 (101.5 to 138.6)	19.5 (11.4 to 30.5)	5.3 (5.3 to 5.3)	569.0 (510.0 to 629.7)	237.8 (205.1 to 277.9)	273.9 (234.4 to 320.1)	45.1 (26.4 to 70.4)	12.3 (12.3 to 12.3)

Country	Year	2018 US Dollars per capita					2018 PPP per capita				
		Health spending	Government health spending	Out-of-pocket health spending	Prepaid private health spending	Development assistance for health	Health spending	Government health spending	Out-of-pocket health spending	Prepaid private health spending	Development assistance for health
Gabon	2009	240.9 (215.6 to 266.0)	107.4 (92.5 to 124.8)	105.7 (90.0 to 124.6)	20.2 (12.0 to 31.2)	7.6 (7.6 to 7.6)	556.2 (497.7 to 614.2)	248.0 (213.5 to 288.1)	244.0 (207.7 to 287.6)	46.7 (27.7 to 71.9)	17.5 (17.5 to 17.5)
Gabon	2010	224.5 (201.6 to 248.2)	120.3 (104.9 to 138.6)	79.5 (67.1 to 94.8)	20.8 (12.5 to 32.4)	3.8 (3.8 to 3.8)	518.2 (465.4 to 573.0)	277.7 (242.2 to 320.0)	183.5 (154.9 to 218.8)	48.1 (28.8 to 74.8)	8.8 (8.8 to 8.8)
Gabon	2011	233.7 (209.5 to 258.7)	134.4 (118.0 to 153.1)	73.0 (61.0 to 87.6)	21.8 (13.3 to 34.3)	4.4 (4.4 to 4.4)	539.5 (483.6 to 597.3)	310.4 (272.5 to 353.5)	168.5 (140.9 to 202.2)	50.4 (30.8 to 79.2)	10.3 (10.3 to 10.3)
Gabon	2012	238.4 (214.5 to 264.7)	142.2 (125.5 to 161.4)	69.9 (57.7 to 84.3)	22.5 (13.6 to 35.3)	3.7 (3.7 to 3.7)	550.4 (495.3 to 611.1)	328.4 (289.7 to 372.6)	161.4 (133.3 to 194.6)	52.0 (31.4 to 81.4)	8.6 (8.6 to 8.6)
Gabon	2013	252.7 (227.9 to 280.7)	152.6 (133.8 to 172.5)	71.2 (57.8 to 85.8)	23.4 (14.1 to 36.7)	5.5 (5.5 to 5.5)	583.4 (526.2 to 648.2)	352.3 (308.9 to 398.4)	164.3 (133.4 to 198.1)	54.1 (32.5 to 84.8)	12.7 (12.7 to 12.7)
Gabon	2014	254.8 (227.7 to 284.6)	153.8 (133.5 to 176.0)	68.9 (55.4 to 84.8)	26.5 (15.6 to 41.9)	5.4 (5.4 to 5.4)	588.2 (525.7 to 657.0)	355.2 (308.3 to 406.2)	159.2 (127.8 to 195.8)	61.3 (36.0 to 96.7)	12.6 (12.6 to 12.6)
Gabon	2015	264.7 (233.7 to 298.1)	161.9 (139.3 to 187.3)	68.5 (54.7 to 85.4)	31.5 (17.9 to 50.1)	2.8 (2.8 to 2.8)	611.2 (539.6 to 688.4)	373.7 (321.6 to 432.5)	158.1 (126.4 to 197.2)	72.8 (41.3 to 115.7)	6.5 (6.5 to 6.5)
Gabon	2016	281.1 (245.0 to 321.4)	174.6 (145.8 to 205.5)	68.6 (54.2 to 86.4)	34.8 (19.4 to 54.8)	3.2 (3.2 to 3.2)	649.1 (565.7 to 742.0)	403.0 (336.6 to 474.4)	158.3 (125.1 to 199.5)	80.3 (44.8 to 126.5)	7.4 (7.4 to 7.4)
Gabon	2017	290.5 (258.3 to 325.7)	158.7 (133.0 to 187.0)	67.2 (53.0 to 84.4)	34.0 (19.0 to 53.6)	30.5 (30.5 to 30.5)	670.7 (596.4 to 751.9)	366.5 (307.1 to 431.6)	155.2 (122.5 to 194.8)	78.6 (44.0 to 123.7)	70.4 (70.4 to 70.4)
Gabon	2018	289.8 (256.4 to 326.3)	159.3 (133.4 to 187.4)	67.3 (53.0 to 84.7)	34.5 (19.3 to 54.4)	28.7 (28.7 to 28.7)	669.1 (592.1 to 753.3)	367.8 (307.9 to 432.7)	155.3 (122.4 to 195.5)	79.8 (44.6 to 125.7)	66.2 (66.2 to 66.3)
Gabon	2019	290.3 (257.9 to 328.6)	159.8 (134.0 to 188.0)	66.8 (52.5 to 83.7)	34.8 (19.5 to 54.7)	28.8 (27.0 to 30.5)	670.2 (595.4 to 758.7)	369.0 (309.3 to 434.1)	154.2 (121.3 to 193.3)	80.4 (45.0 to 126.3)	66.6 (62.2 to 70.4)
Gabon	2020	290.5 (256.3 to 330.5)	160.2 (134.5 to 188.3)	66.0 (51.8 to 82.7)	35.1 (19.7 to 55.3)	29.2 (26.8 to 31.5)	670.8 (591.8 to 763.2)	370.0 (310.5 to 434.8)	152.3 (119.5 to 191.0)	81.0 (45.4 to 127.8)	67.5 (61.9 to 72.8)
Gabon	2021	290.6 (257.2 to 330.0)	160.3 (134.7 to 188.5)	65.2 (51.1 to 82.1)	35.3 (19.8 to 55.7)	29.7 (26.7 to 32.8)	670.9 (593.8 to 761.9)	370.2 (311.1 to 435.3)	150.5 (117.9 to 189.6)	81.6 (45.8 to 128.6)	68.6 (61.7 to 75.8)
Gabon	2022	290.5 (256.1 to 327.8)	160.3 (134.8 to 188.3)	64.3 (50.1 to 81.4)	35.5 (20.0 to 56.0)	30.4 (26.9 to 33.9)	670.8 (591.2 to 756.7)	370.1 (311.3 to 434.8)	148.6 (115.6 to 188.1)	82.0 (46.1 to 129.2)	70.1 (62.2 to 78.2)
Gabon	2023	290.7 (256.9 to 327.9)	160.5 (135.3 to 188.4)	63.5 (49.3 to 80.6)	35.7 (20.1 to 56.3)	31.0 (27.0 to 35.2)	671.2 (593.1 to 757.0)	370.5 (312.3 to 434.9)	146.6 (113.7 to 186.2)	82.5 (46.4 to 129.9)	71.6 (62.2 to 81.3)
Gabon	2024	291.6 (256.4 to 328.8)	161.2 (135.8 to 189.2)	62.7 (48.6 to 79.4)	36.0 (20.2 to 56.5)	31.7 (27.3 to 36.6)	673.2 (592.1 to 759.2)	372.3 (313.4 to 436.8)	144.7 (112.3 to 183.2)	83.0 (46.7 to 130.4)	73.2 (63.1 to 84.4)
Gabon	2025	293.1 (259.0 to 330.4)	162.6 (137.0 to 191.1)	61.9 (48.0 to 78.3)	36.2 (20.4 to 56.8)	32.4 (27.6 to 37.9)	676.8 (598.0 to 762.8)	375.4 (316.3 to 441.1)	142.9 (110.8 to 180.9)	83.7 (47.2 to 131.2)	74.8 (63.7 to 87.5)
Gabon	2026	294.6 (261.2 to 334.0)	163.8 (137.8 to 192.4)	61.2 (47.1 to 77.3)	36.5 (20.6 to 57.3)	33.1 (27.9 to 38.8)	680.1 (603.1 to 771.2)	378.3 (318.3 to 444.3)	141.2 (108.8 to 178.4)	84.3 (47.6 to 132.3)	76.3 (64.5 to 89.5)
Gabon	2027	295.9 (262.5 to 335.5)	164.9 (138.6 to 193.7)	60.5 (46.2 to 76.6)	36.8 (20.8 to 57.7)	33.8 (28.3 to 40.3)	683.2 (606.0 to 774.5)	380.7 (319.9 to 447.2)	139.6 (106.6 to 176.9)	84.9 (47.9 to 133.2)	78.0 (65.3 to 93.0)
Gabon	2028	297.5 (261.5 to 336.9)	166.0 (139.3 to 195.2)	59.9 (45.5 to 76.0)	37.1 (20.9 to 58.0)	34.6 (28.9 to 41.8)	687.0 (603.9 to 777.9)	383.3 (321.7 to 450.7)	138.3 (105.1 to 175.5)	85.7 (48.3 to 134.0)	79.8 (66.6 to 96.4)
Gabon	2029	299.0 (263.3 to 340.6)	166.8 (139.6 to 196.5)	59.4 (44.9 to 75.8)	37.4 (21.1 to 58.4)	35.4 (29.1 to 43.2)	690.3 (608.0 to 786.4)	385.1 (322.3 to 453.7)	137.1 (103.8 to 174.9)	86.3 (48.7 to 134.9)	81.7 (67.2 to 99.7)

		2018 US Dollars per capita					2018 PPP per capita				
Country	Year	Health spending	Government health spending	Out-of-pocket health spending	Prepaid private health spending	Development assistance for health	Health spending	Government health spending	Out-of-pocket health spending	Prepaid private health spending	Development assistance for health
Gabon	2030	301.1 (265.5 to 343.3)	168.2 (140.5 to 198.5)	59.0 (44.4 to 75.7)	37.8 (21.3 to 59.3)	36.1 (29.6 to 44.3)	695.3 (613.0 to 792.7)	388.4 (324.4 to 458.4)	136.3 (102.6 to 174.7)	87.2 (49.1 to 136.9)	83.4 (68.2 to 102.3)
Gabon	2031	303.6 (265.3 to 347.4)	169.8 (141.5 to 200.8)	58.8 (43.6 to 75.5)	38.1 (21.5 to 60.0)	36.9 (29.9 to 46.4)	700.9 (612.5 to 802.1)	392.0 (326.7 to 463.6)	135.7 (100.8 to 174.4)	88.0 (49.5 to 138.6)	85.3 (69.1 to 107.1)
Gabon	2032	306.2 (270.3 to 346.5)	171.4 (142.5 to 202.8)	58.6 (43.2 to 75.8)	38.5 (21.7 to 60.7)	37.7 (30.2 to 47.5)	707.0 (624.2 to 800.1)	395.8 (329.1 to 468.3)	135.3 (99.6 to 175.0)	88.9 (50.0 to 140.1)	87.0 (69.8 to 109.8)
Gabon	2033	309.2 (269.2 to 351.0)	173.2 (143.9 to 204.7)	58.5 (42.9 to 76.1)	39.0 (21.9 to 61.3)	38.5 (30.6 to 49.5)	713.9 (621.6 to 810.5)	399.9 (332.2 to 472.6)	135.1 (99.1 to 175.7)	90.0 (50.5 to 141.6)	88.9 (70.6 to 114.3)
Gabon	2034	312.1 (274.8 to 355.9)	174.8 (145.1 to 206.5)	58.5 (42.8 to 76.5)	39.4 (22.1 to 61.9)	39.4 (31.0 to 50.8)	720.7 (634.4 to 821.8)	403.6 (334.9 to 476.8)	135.1 (98.9 to 176.5)	90.9 (51.0 to 142.9)	91.0 (71.7 to 117.2)
Gabon	2035	314.7 (273.6 to 360.2)	176.2 (145.9 to 208.3)	58.6 (42.6 to 77.0)	39.8 (22.3 to 62.4)	40.1 (31.0 to 52.9)	726.7 (631.7 to 831.6)	406.9 (336.8 to 481.0)	135.3 (98.5 to 177.8)	91.8 (51.5 to 144.0)	92.6 (71.6 to 122.2)
Gabon	2036	317.2 (275.0 to 363.6)	177.6 (146.6 to 210.1)	58.7 (42.7 to 77.4)	40.2 (22.5 to 62.9)	40.7 (31.3 to 55.6)	732.5 (634.9 to 839.5)	410.1 (338.4 to 485.2)	135.6 (98.5 to 178.8)	92.7 (52.0 to 145.2)	94.0 (72.3 to 128.5)
Gabon	2037	320.2 (278.0 to 368.3)	179.2 (147.6 to 212.2)	58.9 (42.8 to 77.9)	40.6 (22.7 to 63.4)	41.5 (31.1 to 56.6)	739.2 (641.9 to 850.4)	413.7 (340.7 to 489.8)	136.0 (98.8 to 180.0)	93.7 (52.5 to 146.4)	95.8 (71.8 to 130.6)
Gabon	2038	322.7 (275.4 to 370.2)	180.7 (148.4 to 214.1)	59.2 (42.9 to 78.6)	41.0 (22.9 to 64.1)	41.9 (0.0 to 59.1)	745.2 (635.9 to 854.7)	417.2 (342.6 to 494.3)	136.7 (99.2 to 181.5)	94.6 (53.0 to 148.0)	96.6 (0.0 to 136.4)
Gabon	2039	325.2 (276.8 to 375.5)	182.0 (149.2 to 215.9)	59.5 (43.0 to 79.1)	41.3 (23.2 to 64.9)	42.4 (0.0 to 60.9)	750.9 (639.1 to 867.0)	420.1 (344.5 to 498.5)	137.4 (99.3 to 182.6)	95.5 (53.5 to 149.8)	97.9 (0.0 to 140.7)
Gabon	2040	327.6 (272.8 to 377.4)	183.2 (149.8 to 217.8)	59.9 (43.1 to 79.8)	41.7 (23.4 to 65.6)	42.8 (0.0 to 62.2)	756.4 (629.8 to 871.5)	423.0 (345.8 to 502.9)	138.4 (99.5 to 184.2)	96.3 (54.1 to 151.5)	98.7 (0.0 to 143.6)
Gabon	2041	330.4 (276.2 to 384.3)	184.3 (150.4 to 219.6)	60.4 (43.2 to 80.3)	42.1 (23.7 to 66.3)	43.6 (0.0 to 64.1)	762.8 (637.7 to 887.2)	425.6 (347.3 to 507.1)	139.4 (99.8 to 185.5)	97.1 (54.6 to 153.0)	100.7 (0.0 to 148.1)
Gabon	2042	333.1 (274.8 to 389.9)	185.4 (151.1 to 221.6)	60.9 (43.5 to 81.2)	42.4 (23.9 to 66.8)	44.5 (0.0 to 67.7)	769.1 (634.4 to 900.3)	428.0 (348.8 to 511.6)	140.6 (100.3 to 187.4)	97.9 (55.2 to 154.3)	102.6 (0.0 to 156.4)
Gabon	2043	335.8 (275.6 to 394.2)	186.6 (151.8 to 223.9)	61.5 (43.7 to 82.3)	42.8 (24.1 to 67.5)	45.0 (0.0 to 70.5)	775.3 (636.3 to 910.1)	430.7 (350.5 to 517.0)	142.0 (101.0 to 189.9)	98.7 (55.8 to 155.9)	103.8 (0.0 to 162.8)
Gabon	2044	338.5 (274.1 to 399.2)	187.5 (152.3 to 225.9)	62.2 (44.1 to 83.6)	43.1 (24.4 to 68.2)	45.8 (0.0 to 72.1)	781.6 (633.0 to 921.6)	432.9 (351.6 to 521.6)	143.5 (101.8 to 193.1)	99.5 (56.3 to 157.4)	105.7 (0.0 to 166.4)
Gabon	2045	341.4 (277.4 to 399.7)	188.6 (152.6 to 228.1)	62.9 (44.4 to 84.6)	43.4 (24.6 to 68.7)	46.5 (0.0 to 76.4)	788.3 (640.5 to 922.9)	435.5 (352.4 to 526.8)	145.3 (102.6 to 195.4)	100.3 (56.8 to 158.7)	107.3 (0.0 to 176.4)
Gabon	2046	344.4 (278.3 to 405.7)	189.9 (153.4 to 230.6)	63.7 (44.9 to 85.9)	43.8 (24.8 to 69.2)	47.0 (0.0 to 79.1)	795.3 (642.5 to 936.8)	438.5 (354.1 to 532.4)	147.2 (103.6 to 198.4)	101.1 (57.3 to 159.7)	108.5 (0.0 to 182.7)
Gabon	2047	348.1 (278.3 to 413.8)	191.5 (154.5 to 233.3)	64.7 (45.4 to 87.2)	44.2 (25.0 to 69.6)	47.8 (0.0 to 82.7)	803.8 (642.5 to 955.5)	442.1 (356.6 to 538.6)	149.3 (104.9 to 201.3)	102.0 (57.7 to 160.8)	110.4 (0.0 to 190.9)
Gabon	2048	351.7 (277.2 to 416.6)	193.2 (155.6 to 236.3)	65.6 (45.9 to 88.6)	44.6 (25.2 to 70.3)	48.4 (0.0 to 87.4)	812.1 (640.0 to 961.8)	446.0 (359.2 to 545.6)	151.5 (106.1 to 204.5)	102.9 (58.1 to 162.2)	111.7 (0.0 to 201.7)
Gabon	2049	355.4 (283.0 to 422.9)	194.9 (156.4 to 238.4)	66.5 (46.3 to 89.9)	45.0 (25.3 to 71.0)	49.0 (0.0 to 89.8)	820.6 (653.4 to 976.5)	450.0 (361.0 to 550.5)	153.6 (107.0 to 207.5)	103.8 (58.5 to 163.9)	113.2 (0.0 to 207.3)
Gabon	2050	358.8 (283.2 to 431.2)	196.9 (157.4 to 241.6)	67.5 (46.7 to 91.1)	45.4 (25.5 to 71.8)	49.0 (0.0 to 91.7)	828.5 (653.9 to 995.5)	454.7 (363.5 to 557.8)	155.8 (107.9 to 210.3)	104.8 (59.0 to 165.8)	113.1 (0.0 to 211.7)

		2018 US Dollars per capita					2018 PPP per capita				
Country	Year	Health spending	Government health spending	Out-of-pocket health spending	Prepaid private health spending	Development assistance for health	Health spending	Government health spending	Out-of-pocket health spending	Prepaid private health spending	Development assistance for health
Georgia	1995	49.6 (40.1 to 60.7)	2.0 (1.5 to 2.5)	47.5 (37.9 to 58.8)	0.0 (0.0 to 0.0)	0.1 (0.1 to 0.1)	132.3 (106.9 to 161.9)	5.2 (4.0 to 6.6)	126.8 (101.2 to 156.7)	0.0 (0.0 to 0.0)	0.3 (0.3 to 0.3)
Georgia	1996	69.7 (57.3 to 83.4)	4.8 (3.7 to 6.1)	64.3 (51.6 to 77.7)	0.0 (0.0 to 0.0)	0.6 (0.6 to 0.6)	185.9 (152.6 to 222.4)	12.8 (9.9 to 16.2)	171.4 (137.4 to 207.1)	0.0 (0.0 to 0.0)	1.7 (1.7 to 1.7)
Georgia	1997	87.1 (73.7 to 102.4)	8.3 (6.4 to 10.5)	75.9 (61.8 to 90.9)	0.0 (0.0 to 0.0)	3.0 (3.0 to 3.0)	232.3 (196.4 to 273.0)	22.2 (17.1 to 27.9)	202.2 (164.9 to 242.2)	0.0 (0.0 to 0.0)	7.9 (7.9 to 7.9)
Georgia	1998	81.4 (69.3 to 95.2)	9.4 (7.3 to 11.8)	69.9 (57.7 to 83.0)	0.0 (0.0 to 0.0)	2.1 (2.1 to 2.1)	217.0 (184.7 to 253.8)	25.1 (19.4 to 31.6)	186.2 (153.8 to 221.3)	0.0 (0.0 to 0.0)	5.7 (5.7 to 5.7)
Georgia	1999	85.6 (74.3 to 98.1)	9.8 (7.6 to 12.4)	69.3 (57.9 to 81.8)	0.0 (0.0 to 0.0)	6.5 (6.5 to 6.5)	228.3 (198.1 to 261.4)	26.2 (20.3 to 33.0)	184.8 (154.5 to 218.2)	0.0 (0.0 to 0.0)	17.3 (17.3 to 17.3)
Georgia	2000	93.8 (81.4 to 107.7)	10.8 (8.4 to 13.5)	78.2 (65.8 to 92.3)	0.1 (0.0 to 0.1)	4.8 (4.8 to 4.8)	250.1 (217.1 to 287.0)	28.8 (22.4 to 36.1)	208.3 (175.4 to 246.1)	0.1 (0.1 to 0.3)	12.8 (12.8 to 12.8)
Georgia	2001	103.4 (89.9 to 118.0)	12.2 (9.6 to 15.3)	86.1 (72.8 to 100.6)	0.2 (0.1 to 0.5)	4.8 (4.8 to 4.8)	275.7 (239.8 to 314.5)	32.6 (25.5 to 40.8)	229.6 (194.0 to 268.1)	0.7 (0.3 to 1.2)	12.9 (12.9 to 12.9)
Georgia	2002	115.2 (100.1 to 130.9)	14.4 (11.3 to 17.7)	95.6 (81.4 to 111.1)	0.5 (0.2 to 1.0)	4.7 (4.7 to 4.7)	307.0 (266.8 to 349.1)	38.3 (30.2 to 47.2)	254.9 (217.0 to 296.2)	1.4 (0.6 to 2.6)	12.5 (12.5 to 12.5)
Georgia	2003	130.9 (114.6 to 147.1)	18.3 (14.6 to 22.6)	109.1 (93.9 to 125.6)	0.9 (0.4 to 1.6)	2.7 (2.7 to 2.7)	349.1 (305.4 to 392.1)	48.8 (38.9 to 60.2)	290.8 (250.4 to 335.0)	2.3 (1.1 to 4.3)	7.1 (7.1 to 7.1)
Georgia	2004	145.7 (129.6 to 161.7)	21.5 (17.3 to 26.2)	119.4 (104.0 to 135.5)	1.4 (0.7 to 2.7)	3.4 (3.4 to 3.4)	388.3 (345.5 to 431.2)	57.2 (46.2 to 69.9)	318.3 (277.1 to 361.2)	3.8 (1.8 to 7.1)	9.0 (9.0 to 9.0)
Georgia	2005	164.9 (147.5 to 182.8)	25.1 (20.2 to 30.5)	129.9 (114.3 to 146.8)	2.1 (1.0 to 4.0)	7.7 (7.7 to 7.7)	439.5 (393.2 to 487.3)	66.9 (53.9 to 81.2)	346.4 (304.8 to 391.3)	5.7 (2.8 to 10.6)	20.5 (20.5 to 20.5)
Georgia	2006	180.6 (161.9 to 201.5)	29.3 (23.6 to 35.6)	138.9 (121.9 to 158.0)	3.1 (1.6 to 5.7)	9.2 (9.2 to 9.2)	481.4 (431.6 to 537.3)	78.0 (62.9 to 94.8)	370.4 (325.1 to 421.3)	8.4 (4.2 to 15.2)	24.6 (24.6 to 24.6)
Georgia	2007	202.9 (183.5 to 224.9)	35.3 (28.6 to 42.6)	152.6 (134.8 to 172.3)	5.2 (2.6 to 9.7)	9.8 (9.8 to 9.8)	540.9 (489.1 to 599.5)	94.0 (76.4 to 113.5)	406.9 (359.4 to 459.5)	13.9 (7.0 to 25.8)	26.0 (26.0 to 26.0)
Georgia	2008	223.1 (201.3 to 246.3)	42.8 (35.1 to 51.5)	165.8 (145.3 to 187.8)	7.9 (3.9 to 14.7)	6.7 (6.7 to 6.7)	594.8 (536.7 to 656.5)	114.1 (93.5 to 137.3)	441.9 (387.4 to 500.8)	21.0 (10.4 to 39.2)	17.8 (17.8 to 17.8)
Georgia	2009	244.3 (221.4 to 268.3)	48.4 (39.2 to 58.0)	176.9 (156.5 to 200.1)	8.8 (4.5 to 16.2)	10.3 (10.3 to 10.3)	651.4 (590.1 to 715.3)	129.0 (104.4 to 154.6)	471.6 (417.3 to 533.5)	23.4 (12.0 to 43.2)	27.3 (27.3 to 27.3)
Georgia	2010	258.0 (234.2 to 285.0)	52.8 (42.9 to 63.2)	186.8 (166.0 to 210.8)	8.4 (4.4 to 15.6)	10.0 (10.0 to 10.0)	687.9 (624.4 to 759.7)	140.6 (114.4 to 168.6)	498.1 (442.7 to 562.0)	22.5 (11.6 to 41.5)	26.7 (26.7 to 26.7)
Georgia	2011	264.8 (240.8 to 290.5)	54.7 (45.1 to 66.0)	190.4 (169.3 to 213.1)	8.8 (4.5 to 15.8)	11.0 (11.0 to 11.0)	706.0 (642.1 to 774.4)	145.8 (120.2 to 176.0)	507.5 (451.3 to 568.2)	23.5 (12.0 to 42.2)	29.2 (29.2 to 29.2)
Georgia	2012	276.3 (250.5 to 302.0)	61.2 (50.0 to 73.3)	194.7 (173.7 to 217.9)	9.3 (4.8 to 17.1)	11.1 (11.1 to 11.1)	736.6 (667.9 to 805.1)	163.1 (133.4 to 195.4)	518.9 (463.1 to 581.0)	24.9 (12.8 to 45.5)	29.6 (29.6 to 29.6)
Georgia	2013	284.4 (257.1 to 310.9)	71.2 (58.6 to 84.8)	195.8 (174.6 to 219.7)	11.0 (5.6 to 20.1)	6.4 (6.4 to 6.4)	758.2 (685.3 to 828.9)	189.9 (156.2 to 226.0)	522.1 (465.4 to 585.7)	29.3 (14.8 to 53.5)	17.0 (17.0 to 17.0)
Georgia	2014	298.3 (269.0 to 328.8)	84.3 (69.4 to 100.4)	195.1 (172.6 to 222.0)	12.9 (6.4 to 23.1)	5.9 (5.9 to 5.9)	795.2 (717.2 to 876.6)	224.6 (185.0 to 267.6)	520.2 (460.2 to 591.8)	34.5 (17.1 to 61.5)	15.8 (15.8 to 15.8)
Georgia	2015	308.5 (276.8 to 341.9)	97.5 (80.4 to 116.5)	187.7 (165.1 to 216.8)	15.0 (7.4 to 26.6)	8.2 (8.2 to 8.2)	822.3 (738.0 to 911.5)	260.0 (214.4 to 310.6)	500.4 (440.1 to 578.1)	40.1 (19.8 to 70.9)	21.9 (21.9 to 21.9)

		2018 US Dollars per capita					2018 PPP per capita				
Country	Year	Health spending	Government health spending	Out-of-pocket health spending	Prepaid private health spending	Development assistance for health	Health spending	Government health spending	Out-of-pocket health spending	Prepaid private health spending	Development assistance for health
Georgia	2016	319.1 (281.6 to 359.8)	108.5 (88.1 to 129.6)	188.8 (162.1 to 221.6)	17.6 (8.7 to 30.8)	4.2 (4.2 to 4.2)	850.8 (750.8 to 959.3)	289.3 (234.8 to 345.5)	503.4 (432.1 to 590.7)	47.0 (23.2 to 82.1)	11.2 (11.2 to 11.2)
Georgia	2017	329.9 (290.1 to 371.4)	111.6 (90.7 to 132.9)	194.0 (166.1 to 228.0)	18.0 (8.8 to 31.4)	6.3 (6.3 to 6.3)	879.5 (773.5 to 990.1)	297.4 (241.7 to 354.4)	517.2 (442.8 to 607.8)	48.0 (23.6 to 83.8)	16.9 (16.9 to 16.9)
Georgia	2018	337.6 (297.8 to 386.1)	114.8 (93.3 to 137.7)	198.1 (169.8 to 232.6)	18.6 (9.1 to 32.4)	6.0 (6.0 to 6.0)	900.0 (793.9 to 1029.2)	306.2 (248.6 to 367.1)	528.2 (452.6 to 620.0)	49.6 (24.4 to 86.4)	16.1 (16.1 to 16.1)
Georgia	2019	345.0 (304.1 to 390.3)	118.0 (95.9 to 141.3)	201.8 (172.7 to 237.5)	19.1 (9.4 to 33.3)	6.1 (5.7 to 6.5)	919.7 (810.7 to 1040.5)	314.5 (255.8 to 376.7)	537.9 (460.4 to 633.2)	50.9 (25.1 to 88.8)	16.4 (15.3 to 17.3)
Georgia	2020	351.6 (312.4 to 400.3)	121.0 (98.2 to 144.9)	204.8 (174.4 to 242.0)	19.5 (9.7 to 34.2)	6.3 (5.8 to 6.8)	937.4 (832.8 to 1067.1)	322.6 (261.7 to 386.2)	545.9 (464.8 to 645.0)	52.1 (25.8 to 91.1)	16.8 (15.4 to 18.1)
Georgia	2021	358.9 (315.6 to 407.3)	124.0 (100.6 to 148.4)	208.4 (177.3 to 244.9)	20.0 (9.9 to 35.0)	6.5 (5.8 to 7.1)	956.9 (841.5 to 1085.7)	330.7 (268.2 to 395.7)	555.6 (472.8 to 652.8)	53.4 (26.4 to 93.2)	17.2 (15.5 to 19.0)
Georgia	2022	366.3 (323.7 to 416.6)	126.8 (102.9 to 151.7)	212.4 (180.9 to 250.9)	20.4 (10.1 to 35.7)	6.7 (5.9 to 7.5)	976.6 (863.0 to 1110.6)	338.1 (274.3 to 404.5)	566.2 (482.1 to 668.9)	54.5 (27.0 to 95.2)	17.8 (15.8 to 19.9)
Georgia	2023	374.1 (328.6 to 423.1)	129.5 (105.1 to 155.2)	216.8 (183.8 to 256.3)	20.8 (10.3 to 36.4)	6.9 (6.0 to 7.8)	997.3 (876.1 to 1128.1)	345.3 (280.3 to 413.8)	578.0 (490.0 to 683.4)	55.6 (27.4 to 97.1)	18.4 (16.0 to 20.9)
Georgia	2024	381.8 (335.6 to 429.9)	132.2 (107.3 to 158.8)	221.1 (187.4 to 261.3)	21.3 (10.5 to 37.2)	7.2 (6.2 to 8.2)	1017.8 (894.6 to 1146.0)	352.5 (286.2 to 423.3)	589.5 (499.5 to 696.6)	56.7 (28.0 to 99.1)	19.1 (16.5 to 21.9)
Georgia	2025	389.9 (343.6 to 443.1)	135.2 (109.8 to 162.6)	225.7 (191.0 to 267.9)	21.7 (10.7 to 38.0)	7.4 (6.3 to 8.7)	1039.5 (916.1 to 1181.2)	360.3 (292.8 to 433.4)	601.6 (509.3 to 714.3)	57.8 (28.6 to 101.2)	19.7 (16.9 to 23.1)
Georgia	2026	398.4 (350.1 to 453.8)	138.2 (112.2 to 166.1)	230.4 (194.4 to 273.3)	22.1 (10.9 to 38.8)	7.7 (6.5 to 9.0)	1062.1 (933.3 to 1209.7)	368.3 (299.2 to 442.8)	614.3 (518.2 to 728.7)	59.0 (29.2 to 103.4)	20.4 (17.2 to 23.9)
Georgia	2027	407.4 (357.4 to 465.6)	141.4 (114.8 to 170.4)	235.4 (198.2 to 282.1)	22.6 (11.2 to 39.7)	7.9 (6.6 to 9.4)	1086.1 (952.9 to 1241.1)	376.9 (306.1 to 454.2)	627.7 (528.4 to 752.0)	60.3 (29.9 to 105.8)	21.1 (17.7 to 25.1)
Georgia	2028	417.0 (363.9 to 479.8)	144.9 (117.7 to 175.3)	240.7 (200.8 to 289.3)	23.2 (11.5 to 40.6)	8.2 (6.9 to 9.9)	1111.8 (970.2 to 1279.2)	386.4 (313.8 to 467.4)	641.7 (535.4 to 771.3)	61.8 (30.6 to 108.2)	21.9 (18.4 to 26.5)
Georgia	2029	427.5 (372.8 to 489.7)	149.0 (121.0 to 180.7)	246.2 (204.0 to 298.7)	23.8 (11.8 to 41.7)	8.5 (7.0 to 10.4)	1139.7 (994.0 to 1305.4)	397.2 (322.5 to 481.7)	656.4 (543.8 to 796.3)	63.4 (31.5 to 111.1)	22.7 (18.7 to 27.7)
Georgia	2030	438.1 (380.1 to 504.2)	153.2 (124.4 to 186.1)	251.8 (206.5 to 307.4)	24.4 (12.1 to 42.8)	8.8 (7.2 to 10.8)	1168.1 (1013.3 to 1344.1)	408.3 (331.6 to 496.1)	671.2 (550.5 to 819.5)	65.1 (32.3 to 114.0)	23.4 (19.2 to 28.8)
Georgia	2031	449.6 (392.7 to 523.2)	157.8 (128.0 to 191.4)	257.6 (209.2 to 315.4)	25.1 (12.5 to 44.0)	9.1 (7.4 to 11.4)	1198.5 (1046.8 to 1394.8)	420.6 (341.3 to 510.3)	686.8 (557.8 to 840.9)	67.0 (33.2 to 117.2)	24.2 (19.6 to 30.4)
Georgia	2032	461.8 (394.7 to 529.7)	162.8 (132.0 to 197.4)	263.7 (212.4 to 325.7)	25.9 (12.8 to 45.3)	9.4 (7.5 to 11.8)	1231.0 (1052.2 to 1412.0)	434.0 (352.0 to 526.3)	702.9 (566.2 to 868.2)	69.1 (34.2 to 120.8)	25.0 (20.1 to 31.6)
Georgia	2033	474.3 (404.0 to 554.2)	168.1 (136.3 to 203.5)	269.8 (215.6 to 335.9)	26.7 (13.2 to 46.7)	9.7 (7.7 to 12.4)	1264.5 (1077.1 to 1477.4)	448.2 (363.5 to 542.5)	719.2 (574.8 to 895.4)	71.2 (35.3 to 124.5)	25.9 (20.5 to 33.2)
Georgia	2034	487.2 (419.6 to 569.6)	173.6 (140.7 to 210.5)	276.0 (218.5 to 346.0)	27.6 (13.6 to 48.2)	10.0 (7.9 to 12.9)	1298.9 (1118.6 to 1518.4)	462.9 (375.0 to 561.1)	735.8 (582.5 to 922.4)	73.5 (36.3 to 128.4)	26.8 (21.1 to 34.5)
Georgia	2035	500.1 (424.1 to 588.8)	179.2 (145.3 to 217.4)	282.1 (221.4 to 356.0)	28.4 (14.0 to 49.6)	10.4 (8.1 to 13.5)	1333.2 (1130.6 to 1569.6)	477.6 (387.4 to 579.7)	752.1 (590.3 to 949.0)	75.7 (37.4 to 132.3)	27.7 (21.5 to 36.1)
Georgia	2036	513.1 (436.7 to 604.5)	184.8 (150.0 to 224.5)	288.3 (224.2 to 365.7)	29.3 (14.4 to 51.1)	10.7 (8.3 to 14.4)	1367.8 (1164.2 to 1611.7)	492.6 (399.9 to 598.6)	768.6 (597.8 to 975.0)	78.0 (38.5 to 136.2)	28.6 (22.2 to 38.5)

Country	Year	2018 US Dollars per capita					2018 PPP per capita				
		Health spending	Government health spending	Out-of-pocket health spending	Prepaid private health spending	Development assistance for health	Health spending	Government health spending	Out-of-pocket health spending	Prepaid private health spending	Development assistance for health
Georgia	2037	526.2 (444.9 to 621.9)	190.5 (154.6 to 232.4)	294.5 (226.7 to 376.1)	30.1 (14.9 to 52.6)	11.1 (8.5 to 14.9)	1402.9 (1186.1 to 1657.9)	507.9 (412.2 to 619.6)	785.1 (604.4 to 1002.7)	80.3 (39.6 to 140.2)	29.5 (22.6 to 39.7)
Georgia	2038	539.6 (458.7 to 641.4)	196.4 (159.1 to 240.5)	300.7 (229.6 to 386.7)	31.0 (15.3 to 54.1)	11.4 (8.7 to 15.9)	1438.5 (1223.0 to 1709.8)	523.6 (424.2 to 641.1)	801.7 (612.2 to 1030.9)	82.7 (40.8 to 144.3)	30.5 (23.1 to 42.4)
Georgia	2039	552.9 (461.6 to 659.4)	202.4 (163.9 to 248.0)	306.8 (232.8 to 396.2)	31.9 (15.7 to 55.7)	11.8 (9.0 to 16.4)	1474.1 (1230.6 to 1758.0)	539.5 (437.0 to 661.1)	818.0 (620.6 to 1056.2)	85.1 (42.0 to 148.5)	31.5 (24.1 to 43.6)
Georgia	2040	566.5 (477.3 to 673.9)	208.5 (169.2 to 255.7)	313.0 (235.9 to 405.0)	32.9 (16.2 to 57.3)	12.2 (9.2 to 17.0)	1510.2 (1272.4 to 1796.7)	555.7 (451.1 to 681.7)	834.4 (628.9 to 1079.6)	87.6 (43.1 to 152.7)	32.6 (24.6 to 45.3)
Georgia	2041	580.1 (485.0 to 688.8)	214.6 (173.6 to 263.5)	319.1 (238.7 to 413.4)	33.8 (16.6 to 58.9)	12.6 (9.5 to 17.6)	1546.6 (1293.0 to 1836.2)	572.1 (462.7 to 702.4)	850.7 (636.4 to 1102.0)	90.1 (44.2 to 157.1)	33.7 (25.3 to 46.8)
Georgia	2042	594.3 (494.0 to 711.5)	221.1 (178.2 to 271.7)	325.3 (242.6 to 422.8)	34.8 (17.0 to 60.7)	13.1 (9.7 to 18.7)	1584.2 (1316.8 to 1896.9)	589.4 (475.0 to 724.3)	867.3 (646.9 to 1127.1)	92.7 (45.4 to 161.9)	34.8 (25.9 to 49.8)
Georgia	2043	608.2 (508.7 to 722.8)	227.6 (183.0 to 279.7)	331.4 (246.5 to 432.5)	35.8 (17.5 to 62.5)	13.5 (10.0 to 19.5)	1621.5 (1356.3 to 1926.9)	606.7 (487.9 to 745.6)	883.5 (657.2 to 1153.1)	95.3 (46.6 to 166.6)	36.0 (26.6 to 52.0)
Georgia	2044	622.2 (522.5 to 753.0)	234.1 (188.3 to 288.0)	337.3 (250.7 to 441.3)	36.7 (17.9 to 64.2)	14.0 (10.3 to 20.2)	1658.7 (1392.9 to 2007.6)	624.1 (502.0 to 767.7)	899.3 (668.4 to 1176.5)	97.9 (47.8 to 171.1)	37.4 (27.4 to 54.0)
Georgia	2045	636.3 (536.2 to 764.9)	240.8 (193.8 to 296.6)	343.3 (254.8 to 450.2)	37.7 (18.4 to 66.0)	14.5 (10.4 to 21.9)	1696.3 (1429.5 to 2039.3)	641.9 (516.5 to 790.8)	915.2 (679.3 to 1200.3)	100.6 (49.0 to 175.9)	38.6 (27.7 to 58.3)
Georgia	2046	650.3 (538.6 to 775.6)	247.4 (199.1 to 305.2)	349.1 (259.0 to 459.1)	38.8 (18.8 to 67.8)	15.0 (10.8 to 23.2)	1733.7 (1436.0 to 2067.8)	659.6 (530.9 to 813.7)	930.8 (690.5 to 1224.0)	103.3 (50.2 to 180.6)	40.1 (28.9 to 61.9)
Georgia	2047	664.5 (552.6 to 800.1)	254.2 (204.4 to 313.8)	355.0 (264.0 to 467.7)	39.8 (19.3 to 69.6)	15.6 (11.1 to 24.6)	1771.6 (1473.1 to 2133.1)	677.7 (545.0 to 836.5)	946.3 (703.8 to 1246.8)	106.1 (51.4 to 185.6)	41.5 (29.5 to 65.6)
Georgia	2048	678.8 (561.4 to 807.6)	261.0 (209.6 to 322.4)	360.7 (268.0 to 476.4)	40.8 (19.8 to 71.5)	16.2 (11.4 to 26.1)	1809.5 (1496.8 to 2152.9)	695.8 (558.7 to 859.6)	961.7 (714.5 to 1270.0)	108.8 (52.7 to 190.6)	43.1 (30.5 to 69.6)
Georgia	2049	693.0 (574.8 to 837.5)	267.8 (214.2 to 331.4)	366.6 (271.4 to 484.9)	41.9 (20.2 to 73.3)	16.7 (11.9 to 27.4)	1847.5 (1532.5 to 2232.8)	714.0 (571.0 to 883.5)	977.2 (723.6 to 1292.7)	111.6 (54.0 to 195.5)	44.6 (31.8 to 72.9)
Georgia	2050	707.2 (589.2 to 849.8)	274.6 (219.5 to 340.6)	372.4 (275.9 to 493.5)	42.9 (20.8 to 75.2)	17.3 (11.9 to 28.6)	1885.4 (1570.8 to 2265.5)	732.1 (585.0 to 908.0)	992.8 (735.4 to 1315.7)	114.4 (55.3 to 200.4)	46.1 (31.8 to 76.2)
Germany	1995	4097.3 (3921.8 to 4276.1)	2529.7 (2397.6 to 2676.1)	349.4 (319.3 to 380.9)	1218.2 (1122.4 to 1323.7)	0.0 (0.0 to 0.0)	4374.5 (4187.1 to 4565.4)	2700.8 (2559.8 to 2857.2)	373.0 (340.9 to 406.7)	1300.6 (1198.3 to 1413.3)	0.0 (0.0 to 0.0)
Germany	1996	4102.6 (3969.3 to 4239.0)	2639.2 (2537.6 to 2757.3)	367.0 (344.1 to 394.4)	1096.4 (1025.8 to 1170.2)	0.0 (0.0 to 0.0)	4380.1 (4237.9 to 4525.8)	2817.8 (2709.3 to 2943.8)	391.8 (367.4 to 421.1)	1170.5 (1095.2 to 1249.3)	0.0 (0.0 to 0.0)
Germany	1997	4020.2 (3890.9 to 4148.9)	2736.4 (2638.7 to 2835.4)	392.7 (370.2 to 416.2)	891.1 (824.3 to 962.7)	0.0 (0.0 to 0.0)	4292.1 (4154.1 to 4429.6)	2921.6 (2817.2 to 3027.3)	419.2 (395.2 to 444.4)	951.3 (880.0 to 1027.8)	0.0 (0.0 to 0.0)
Germany	1998	3967.2 (3844.9 to 4086.0)	2821.0 (2725.4 to 2918.7)	418.2 (395.3 to 442.5)	728.0 (667.3 to 794.6)	0.0 (0.0 to 0.0)	4235.6 (4105.0 to 4362.4)	3011.9 (2909.8 to 3116.1)	446.5 (422.0 to 472.5)	777.2 (712.4 to 848.4)	0.0 (0.0 to 0.0)
Germany	1999	3936.4 (3821.7 to 4045.0)	2913.5 (2816.2 to 3011.5)	440.5 (417.2 to 465.0)	582.4 (529.6 to 642.6)	0.0 (0.0 to 0.0)	4202.7 (4080.3 to 4318.7)	3110.6 (3006.8 to 3215.2)	470.3 (445.4 to 496.4)	621.8 (565.4 to 686.1)	0.0 (0.0 to 0.0)
Germany	2000	3948.1 (3837.7 to 4066.3)	3031.7 (2939.4 to 3135.1)	463.8 (441.7 to 486.7)	452.6 (402.0 to 507.4)	0.0 (0.0 to 0.0)	4215.2 (4097.3 to 4341.4)	3236.8 (3138.3 to 3347.2)	495.2 (471.6 to 519.6)	483.2 (429.2 to 541.7)	0.0 (0.0 to 0.0)
Germany	2001	4011.3 (3904.7 to 4128.0)	3122.8 (3029.6 to 3219.6)	484.0 (462.0 to 505.9)	404.4 (356.1 to 456.5)	0.0 (0.0 to 0.0)	4282.6 (4168.8 to 4407.3)	3334.0 (3234.6 to 3437.5)	516.8 (493.3 to 540.1)	431.8 (380.2 to 487.4)	0.0 (0.0 to 0.0)

Country	Year	2018 US Dollars per capita					2018 PPP per capita				
		Health spending	Government health spending	Out-of-pocket health spending	Prepaid private health spending	Development assistance for health	Health spending	Government health spending	Out-of-pocket health spending	Prepaid private health spending	Development assistance for health
Germany	2002	4052.6 (3939.9 to 4165.5)	3155.7 (3057.3 to 3255.8)	504.8 (481.7 to 525.8)	392.1 (346.5 to 444.8)	0.0 (0.0 to 0.0)	4326.7 (4206.5 to 4447.3)	3369.2 (3264.1 to 3476.1)	538.9 (514.3 to 561.4)	418.6 (369.9 to 474.9)	0.0 (0.0 to 0.0)
Germany	2003	4067.8 (3953.5 to 4182.7)	3142.0 (3039.4 to 3242.6)	526.8 (504.9 to 548.4)	399.0 (352.6 to 452.6)	0.0 (0.0 to 0.0)	4343.0 (4220.9 to 4465.6)	3354.6 (3245.0 to 3461.9)	562.4 (539.1 to 585.5)	426.0 (376.4 to 483.3)	0.0 (0.0 to 0.0)
Germany	2004	4096.0 (3977.3 to 4210.4)	3124.0 (3026.3 to 3219.8)	555.2 (533.5 to 578.2)	416.9 (365.6 to 472.2)	0.0 (0.0 to 0.0)	4373.1 (4246.3 to 4495.2)	3335.3 (3231.0 to 3437.7)	592.7 (569.6 to 617.3)	445.1 (390.3 to 504.2)	0.0 (0.0 to 0.0)
Germany	2005	4131.9 (4018.7 to 4250.9)	3115.9 (3020.6 to 3216.0)	578.5 (555.2 to 601.5)	437.4 (387.2 to 491.1)	0.0 (0.0 to 0.0)	4411.4 (4290.6 to 4538.5)	3326.7 (3225.0 to 3433.5)	617.7 (592.8 to 642.2)	467.0 (413.4 to 524.3)	0.0 (0.0 to 0.0)
Germany	2006	4263.6 (4148.7 to 4381.6)	3201.7 (3094.8 to 3302.0)	604.9 (583.5 to 629.0)	457.0 (401.8 to 514.9)	0.0 (0.0 to 0.0)	4552.1 (4429.4 to 4678.0)	3418.3 (3304.1 to 3525.4)	645.8 (623.0 to 671.5)	487.9 (429.0 to 549.7)	0.0 (0.0 to 0.0)
Germany	2007	4423.0 (4306.9 to 4546.2)	3349.2 (3238.6 to 3457.2)	624.5 (601.1 to 647.1)	449.3 (395.4 to 508.9)	0.0 (0.0 to 0.0)	4722.2 (4598.3 to 4853.7)	3575.8 (3457.7 to 3691.1)	666.8 (641.8 to 690.9)	479.7 (422.2 to 543.3)	0.0 (0.0 to 0.0)
Germany	2008	4579.8 (4459.8 to 4695.1)	3554.4 (3456.2 to 3655.1)	639.7 (614.9 to 663.1)	385.7 (332.0 to 444.6)	0.0 (0.0 to 0.0)	4889.6 (4761.5 to 5012.7)	3794.8 (3690.0 to 3902.3)	683.0 (656.5 to 707.9)	411.8 (354.5 to 474.6)	0.0 (0.0 to 0.0)
Germany	2009	4585.6 (4477.8 to 4696.2)	3695.5 (3594.8 to 3793.8)	640.6 (616.2 to 665.7)	249.5 (207.4 to 298.0)	0.0 (0.0 to 0.0)	4895.9 (4780.8 to 5013.9)	3945.5 (3838.0 to 4050.5)	684.0 (657.9 to 710.8)	266.4 (221.4 to 318.2)	0.0 (0.0 to 0.0)
Germany	2010	4802.4 (4691.4 to 4926.2)	3941.2 (3840.8 to 4040.6)	661.5 (636.3 to 686.7)	199.7 (160.2 to 243.4)	0.0 (0.0 to 0.0)	5127.3 (5008.8 to 5259.5)	4207.9 (4100.6 to 4314.0)	706.3 (679.3 to 733.2)	213.2 (171.0 to 259.9)	0.0 (0.0 to 0.0)
Germany	2011	4945.8 (4837.0 to 5070.4)	4093.8 (3997.0 to 4195.6)	674.2 (651.0 to 698.6)	177.8 (138.3 to 223.0)	0.0 (0.0 to 0.0)	5280.4 (5164.3 to 5413.4)	4370.8 (4267.4 to 4479.5)	719.8 (695.0 to 745.8)	189.8 (147.7 to 238.1)	0.0 (0.0 to 0.0)
Germany	2012	4947.2 (4838.4 to 5061.1)	4114.2 (4011.4 to 4224.9)	669.1 (644.5 to 693.6)	163.8 (127.1 to 207.2)	0.0 (0.0 to 0.0)	5281.9 (5165.7 to 5403.5)	4392.6 (4282.8 to 4510.7)	714.4 (688.1 to 740.5)	174.9 (135.7 to 221.3)	0.0 (0.0 to 0.0)
Germany	2013	4982.6 (4865.3 to 5100.5)	4170.2 (4062.3 to 4276.1)	655.1 (632.2 to 681.3)	157.4 (122.1 to 200.9)	0.0 (0.0 to 0.0)	5319.7 (5194.5 to 5445.6)	4452.3 (4337.1 to 4565.4)	699.4 (674.9 to 727.4)	168.0 (130.4 to 214.5)	0.0 (0.0 to 0.0)
Germany	2014	5085.6 (4972.6 to 5206.0)	4281.2 (4180.8 to 4390.0)	649.1 (626.3 to 672.2)	155.3 (119.7 to 199.5)	0.0 (0.0 to 0.0)	5429.6 (5309.0 to 5558.2)	4570.8 (4463.6 to 4687.0)	693.0 (668.7 to 717.7)	165.8 (127.8 to 213.0)	0.0 (0.0 to 0.0)
Germany	2015	5176.6 (5059.5 to 5298.4)	4370.2 (4264.5 to 4482.6)	651.3 (626.8 to 675.3)	155.0 (115.0 to 203.1)	0.0 (0.0 to 0.0)	5526.8 (5401.8 to 5656.8)	4665.9 (4553.0 to 4785.8)	695.4 (669.2 to 721.0)	165.5 (122.8 to 216.8)	0.0 (0.0 to 0.0)
Germany	2016	5262.7 (5094.8 to 5434.8)	4454.0 (4299.4 to 4609.3)	652.9 (618.9 to 687.0)	155.8 (108.6 to 210.8)	0.0 (0.0 to 0.0)	5618.8 (5439.5 to 5802.5)	4755.3 (4590.3 to 4921.2)	697.1 (660.8 to 733.5)	166.3 (115.9 to 225.1)	0.0 (0.0 to 0.0)
Germany	2017	5374.5 (5203.6 to 5545.8)	4549.5 (4386.6 to 4704.3)	664.7 (630.8 to 701.7)	160.3 (112.0 to 216.6)	0.0 (0.0 to 0.0)	5738.1 (5555.7 to 5921.0)	4857.3 (4683.4 to 5022.5)	709.7 (673.5 to 749.2)	171.1 (119.6 to 231.3)	0.0 (0.0 to 0.0)
Germany	2018	5419.4 (5239.1 to 5618.8)	4584.3 (4421.9 to 4747.0)	673.0 (636.8 to 711.0)	162.1 (113.0 to 219.0)	0.0 (0.0 to 0.0)	5786.0 (5593.6 to 5999.0)	4894.4 (4721.0 to 5068.2)	718.5 (679.9 to 759.1)	173.1 (120.6 to 233.8)	0.0 (0.0 to 0.0)
Germany	2019	5469.9 (5289.3 to 5647.9)	4623.9 (4455.4 to 4805.2)	681.8 (642.9 to 723.8)	164.2 (114.8 to 221.5)	0.0 (0.0 to 0.0)	5840.0 (5647.2 to 6030.0)	4936.7 (4756.8 to 5130.2)	727.9 (686.4 to 772.7)	175.4 (122.5 to 236.4)	0.0 (0.0 to 0.0)
Germany	2020	5541.1 (5344.3 to 5739.8)	4682.2 (4507.2 to 4874.1)	691.9 (650.6 to 735.8)	166.9 (117.1 to 225.2)	0.0 (0.0 to 0.0)	5916.0 (5705.9 to 6128.1)	4999.0 (4812.1 to 5203.9)	738.7 (694.6 to 785.6)	178.2 (125.0 to 240.4)	0.0 (0.0 to 0.0)
Germany	2021	5601.7 (5408.6 to 5802.9)	4736.3 (4552.9 to 4935.0)	696.0 (654.0 to 739.7)	169.4 (119.0 to 228.4)	0.0 (0.0 to 0.0)	5980.6 (5774.5 to 6195.5)	5056.7 (4860.9 to 5268.9)	743.1 (698.2 to 789.7)	180.8 (127.1 to 243.9)	0.0 (0.0 to 0.0)
Germany	2022	5660.1 (5441.6 to 5898.4)	4788.9 (4593.7 to 5000.3)	699.6 (658.2 to 745.0)	171.6 (120.7 to 231.7)	0.0 (0.0 to 0.0)	6043.1 (5809.7 to 6297.4)	5112.9 (4904.5 to 5338.6)	746.9 (702.7 to 795.4)	183.2 (128.9 to 247.3)	0.0 (0.0 to 0.0)

		2018 US Dollars per capita					2018 PPP per capita				
Country	Year	Health spending	Government health spending	Out-of-pocket health spending	Prepaid private health spending	Development assistance for health	Health spending	Government health spending	Out-of-pocket health spending	Prepaid private health spending	Development assistance for health
Germany	2023	5718.1 (5483.5 to 5949.8)	4841.3 (4631.4 to 5065.1)	703.1 (661.4 to 747.7)	173.8 (122.1 to 235.1)	0.0 (0.0 to 0.0)	6105.0 (5854.4 to 6352.4)	5168.8 (4944.7 to 5407.8)	750.6 (706.2 to 798.3)	185.6 (130.4 to 251.0)	0.0 (0.0 to 0.0)
Germany	2024	5775.5 (5529.3 to 6030.2)	4893.7 (4671.5 to 5121.6)	706.0 (663.1 to 751.4)	175.9 (123.4 to 237.7)	0.0 (0.0 to 0.0)	6166.2 (5903.3 to 6438.2)	5224.8 (4987.5 to 5468.1)	753.7 (707.9 to 802.2)	187.8 (131.7 to 253.8)	0.0 (0.0 to 0.0)
Germany	2025	5830.5 (5578.9 to 6081.0)	4944.4 (4713.8 to 5182.9)	708.3 (666.4 to 754.1)	177.8 (124.4 to 240.3)	0.0 (0.0 to 0.0)	6224.9 (5956.3 to 6492.4)	5278.9 (5032.7 to 5533.5)	756.2 (711.5 to 805.1)	189.8 (132.9 to 256.5)	0.0 (0.0 to 0.0)
Germany	2026	5882.5 (5617.9 to 6145.3)	4992.9 (4749.7 to 5246.3)	710.1 (667.3 to 755.4)	179.6 (125.4 to 242.4)	0.0 (0.0 to 0.0)	6280.5 (5997.9 to 6561.0)	5330.6 (5071.1 to 5601.2)	758.1 (712.5 to 806.6)	191.7 (133.9 to 258.8)	0.0 (0.0 to 0.0)
Germany	2027	5930.7 (5653.3 to 6208.8)	5038.4 (4778.9 to 5299.9)	711.1 (667.6 to 758.6)	181.2 (126.7 to 244.5)	0.0 (0.0 to 0.0)	6331.9 (6035.7 to 6628.9)	5379.3 (5102.2 to 5658.4)	759.2 (712.8 to 809.9)	193.4 (135.3 to 261.1)	0.0 (0.0 to 0.0)
Germany	2028	5980.2 (5689.5 to 6272.5)	5085.2 (4812.6 to 5362.8)	712.2 (667.2 to 759.4)	182.8 (128.2 to 246.7)	0.0 (0.0 to 0.0)	6384.8 (6074.4 to 6696.8)	5429.2 (5138.2 to 5725.6)	760.4 (712.3 to 810.8)	195.2 (136.9 to 263.4)	0.0 (0.0 to 0.0)
Germany	2029	6029.8 (5736.8 to 6340.0)	5132.3 (4844.6 to 5427.2)	713.0 (667.1 to 761.4)	184.4 (129.3 to 248.7)	0.0 (0.0 to 0.0)	6437.7 (6125.0 to 6768.9)	5479.5 (5172.3 to 5794.3)	761.3 (712.2 to 812.9)	196.9 (138.0 to 265.6)	0.0 (0.0 to 0.0)
Germany	2030	6076.9 (5762.7 to 6398.3)	5177.2 (4875.2 to 5496.7)	713.7 (667.1 to 763.8)	186.0 (130.2 to 251.1)	0.0 (0.0 to 0.0)	6488.0 (6152.5 to 6831.2)	5527.4 (5205.0 to 5868.6)	762.0 (712.2 to 815.5)	198.6 (139.0 to 268.1)	0.0 (0.0 to 0.0)
Germany	2031	6122.5 (5802.8 to 6482.1)	5220.7 (4903.1 to 5561.2)	714.2 (667.1 to 765.3)	187.5 (131.1 to 253.6)	0.0 (0.0 to 0.0)	6536.7 (6195.4 to 6920.6)	5573.9 (5234.8 to 5937.4)	762.5 (712.3 to 817.1)	200.2 (140.0 to 270.8)	0.0 (0.0 to 0.0)
Germany	2032	6169.3 (5821.7 to 6531.0)	5265.4 (4930.8 to 5624.7)	714.8 (666.6 to 766.7)	189.1 (132.3 to 256.8)	0.0 (0.0 to 0.0)	6586.6 (6215.5 to 6972.8)	5621.6 (5264.4 to 6005.2)	763.2 (711.7 to 818.6)	201.9 (141.2 to 274.1)	0.0 (0.0 to 0.0)
Germany	2033	6218.1 (5844.1 to 6608.7)	5311.4 (4952.2 to 5693.0)	715.9 (665.5 to 769.1)	190.8 (133.0 to 259.8)	0.0 (0.0 to 0.0)	6638.7 (6239.5 to 7055.8)	5670.7 (5287.2 to 6078.1)	764.3 (710.5 to 821.1)	203.7 (142.0 to 277.4)	0.0 (0.0 to 0.0)
Germany	2034	6269.3 (5883.0 to 6681.0)	5358.7 (4986.5 to 5757.8)	717.8 (665.1 to 771.6)	192.8 (134.3 to 263.4)	0.0 (0.0 to 0.0)	6693.5 (6281.0 to 7133.0)	5721.2 (5323.9 to 6147.3)	766.4 (710.1 to 823.8)	205.9 (143.4 to 281.2)	0.0 (0.0 to 0.0)
Germany	2035	6327.5 (5924.8 to 6775.4)	5411.4 (5022.3 to 5826.1)	721.0 (666.4 to 775.7)	195.2 (136.1 to 266.6)	0.0 (0.0 to 0.0)	6755.6 (6325.7 to 7233.8)	5777.5 (5362.1 to 6220.2)	769.8 (711.5 to 828.2)	208.4 (145.3 to 284.6)	0.0 (0.0 to 0.0)
Germany	2036	6389.2 (5985.7 to 6839.7)	5466.1 (5065.5 to 5901.4)	725.3 (668.8 to 781.3)	197.8 (138.0 to 270.1)	0.0 (0.0 to 0.0)	6821.4 (6390.7 to 7302.5)	5835.9 (5408.2 to 6300.7)	774.3 (714.0 to 834.1)	211.2 (147.4 to 288.4)	0.0 (0.0 to 0.0)
Germany	2037	6460.2 (6043.7 to 6943.0)	5527.9 (5122.1 to 5981.9)	731.2 (672.7 to 788.8)	201.0 (140.2 to 274.7)	0.0 (0.0 to 0.0)	6897.2 (6452.6 to 7412.7)	5901.9 (5468.6 to 6386.5)	780.7 (718.2 to 842.2)	214.6 (149.7 to 293.3)	0.0 (0.0 to 0.0)
Germany	2038	6535.8 (6101.8 to 7003.1)	5593.0 (5178.4 to 6058.3)	738.2 (677.6 to 797.0)	204.6 (142.9 to 280.3)	0.0 (0.0 to 0.0)	6977.9 (6514.6 to 7476.9)	5971.4 (5528.8 to 6468.2)	788.2 (723.4 to 851.0)	218.4 (152.5 to 299.3)	0.0 (0.0 to 0.0)
Germany	2039	6616.8 (6169.9 to 7118.4)	5662.7 (5232.2 to 6159.4)	745.8 (683.1 to 805.2)	208.3 (145.6 to 286.0)	0.0 (0.0 to 0.0)	7064.4 (6587.3 to 7600.0)	6045.8 (5586.2 to 6576.1)	796.2 (729.3 to 859.7)	222.4 (155.4 to 305.3)	0.0 (0.0 to 0.0)
Germany	2040	6700.0 (6231.8 to 7204.2)	5734.4 (5266.7 to 6233.3)	753.5 (688.8 to 814.1)	212.1 (148.0 to 291.1)	0.0 (0.0 to 0.0)	7153.3 (6653.4 to 7691.6)	6122.3 (5623.0 to 6655.0)	804.5 (735.4 to 869.2)	226.5 (158.0 to 310.8)	0.0 (0.0 to 0.0)
Germany	2041	6784.5 (6307.3 to 7313.2)	5806.4 (5324.9 to 6348.8)	761.9 (695.0 to 823.3)	216.2 (150.6 to 296.2)	0.0 (0.0 to 0.0)	7243.5 (6734.0 to 7808.0)	6199.2 (5685.2 to 6778.3)	813.4 (742.0 to 879.0)	230.8 (160.8 to 316.2)	0.0 (0.0 to 0.0)
Germany	2042	6871.6 (6386.1 to 7415.3)	5880.6 (5380.6 to 6427.1)	770.6 (700.8 to 834.6)	220.4 (153.1 to 302.1)	0.0 (0.0 to 0.0)	7336.4 (6818.1 to 7916.9)	6278.4 (5744.6 to 6861.9)	822.7 (748.3 to 891.1)	235.3 (163.5 to 322.5)	0.0 (0.0 to 0.0)
Germany	2043	6962.7 (6434.1 to 7526.3)	5958.4 (5432.8 to 6515.9)	779.5 (707.5 to 846.2)	224.8 (156.0 to 308.7)	0.0 (0.0 to 0.0)	7433.7 (6869.4 to 8035.4)	6361.5 (5800.4 to 6956.7)	832.2 (755.3 to 903.5)	240.0 (166.6 to 329.5)	0.0 (0.0 to 0.0)

		2018 US Dollars per capita					2018 PPP per capita				
Country	Year	Health spending	Government health spending	Out-of-pocket health spending	Prepaid private health spending	Development assistance for health	Health spending	Government health spending	Out-of-pocket health spending	Prepaid private health spending	Development assistance for health
Germany	2044	7055.0 (6539.9 to 7633.9)	6037.5 (5493.4 to 6601.8)	788.3 (713.5 to 857.1)	229.1 (158.9 to 314.5)	0.0 (0.0 to 0.0)	7532.3 (6982.3 to 8150.4)	6446.0 (5865.1 to 7048.4)	841.7 (761.8 to 915.1)	244.6 (169.7 to 335.8)	0.0 (0.0 to 0.0)
Germany	2045	7147.9 (6591.6 to 7722.8)	6117.4 (5558.2 to 6689.9)	797.0 (719.6 to 866.8)	233.5 (161.7 to 321.1)	0.0 (0.0 to 0.0)	7631.5 (7037.5 to 8245.3)	6531.3 (5934.2 to 7142.5)	850.9 (768.2 to 925.4)	249.3 (172.6 to 342.8)	0.0 (0.0 to 0.0)
Germany	2046	7240.7 (6645.7 to 7843.1)	6196.8 (5611.6 to 6796.0)	805.9 (725.9 to 878.7)	238.0 (164.6 to 328.4)	0.0 (0.0 to 0.0)	7730.5 (7095.3 to 8373.7)	6616.1 (5991.2 to 7255.8)	860.4 (775.0 to 938.2)	254.1 (175.7 to 350.6)	0.0 (0.0 to 0.0)
Germany	2047	7333.6 (6718.0 to 7955.9)	6276.8 (5672.1 to 6897.7)	814.4 (731.4 to 888.4)	242.4 (167.8 to 334.3)	0.0 (0.0 to 0.0)	7829.8 (7172.5 to 8494.1)	6701.4 (6055.8 to 7364.3)	869.5 (780.9 to 948.5)	258.8 (179.1 to 356.9)	0.0 (0.0 to 0.0)
Germany	2048	7425.7 (6775.1 to 8073.9)	6356.1 (5730.2 to 6997.6)	822.7 (736.8 to 897.3)	246.9 (170.7 to 340.0)	0.0 (0.0 to 0.0)	7928.1 (7233.4 to 8620.1)	6786.1 (6117.9 to 7471.0)	878.4 (786.7 to 958.0)	263.6 (182.3 to 363.0)	0.0 (0.0 to 0.0)
Germany	2049	7515.4 (6829.8 to 8191.8)	6433.8 (5779.0 to 7113.6)	830.4 (742.7 to 907.2)	251.2 (173.2 to 346.8)	0.0 (0.0 to 0.0)	8023.8 (7291.8 to 8745.9)	6869.1 (6170.0 to 7594.9)	886.6 (792.9 to 968.5)	268.2 (185.0 to 370.3)	0.0 (0.0 to 0.0)
Germany	2050	7605.6 (6915.9 to 8317.2)	6512.3 (5818.6 to 7214.1)	837.9 (748.3 to 916.0)	255.5 (175.6 to 352.9)	0.0 (0.0 to 0.0)	8120.2 (7383.8 to 8879.9)	6952.9 (6212.3 to 7702.2)	894.6 (798.9 to 978.0)	272.7 (187.5 to 376.7)	0.0 (0.0 to 0.0)
Ghana	1995	34.6 (28.4 to 42.6)	10.8 (8.2 to 13.8)	18.4 (13.2 to 25.2)	3.2 (1.5 to 6.2)	2.2 (2.2 to 2.2)	97.2 (79.8 to 119.8)	30.3 (23.1 to 38.7)	51.6 (37.1 to 70.9)	9.0 (4.1 to 17.3)	6.3 (6.3 to 6.3)
Ghana	1996	35.8 (29.3 to 43.5)	11.4 (8.6 to 14.7)	18.7 (13.4 to 25.2)	3.3 (1.5 to 6.4)	2.3 (2.3 to 2.3)	100.6 (82.3 to 122.2)	32.0 (24.3 to 41.2)	52.7 (37.8 to 71.0)	9.3 (4.3 to 18.0)	6.6 (6.6 to 6.6)
Ghana	1997	39.0 (32.4 to 46.3)	11.0 (8.4 to 14.1)	19.1 (13.7 to 25.3)	3.2 (1.5 to 6.2)	5.7 (5.7 to 5.7)	109.6 (91.2 to 130.2)	30.9 (23.5 to 39.6)	53.6 (38.6 to 71.0)	9.0 (4.1 to 17.4)	16.1 (16.1 to 16.1)
Ghana	1998	36.2 (29.9 to 43.4)	11.3 (8.7 to 14.6)	19.2 (13.9 to 25.6)	3.2 (1.5 to 6.1)	2.5 (2.5 to 2.5)	101.7 (84.0 to 122.0)	31.9 (24.5 to 40.9)	53.9 (39.2 to 71.8)	8.9 (4.1 to 17.0)	7.0 (7.0 to 7.0)
Ghana	1999	37.7 (31.6 to 45.4)	11.4 (8.8 to 14.6)	19.5 (14.3 to 26.0)	3.0 (1.4 to 5.8)	3.7 (3.7 to 3.7)	106.0 (88.7 to 127.5)	32.1 (24.8 to 41.0)	54.9 (40.3 to 73.2)	8.5 (4.0 to 16.2)	10.5 (10.5 to 10.5)
Ghana	2000	37.1 (31.1 to 45.1)	11.3 (8.8 to 14.4)	19.7 (14.5 to 26.5)	2.7 (1.3 to 5.1)	3.3 (3.3 to 3.3)	104.4 (87.4 to 126.7)	31.9 (24.7 to 40.4)	55.4 (40.8 to 74.5)	7.7 (3.7 to 14.3)	9.4 (9.4 to 9.4)
Ghana	2001	39.9 (33.8 to 47.5)	11.8 (9.1 to 15.0)	20.1 (14.7 to 26.9)	2.7 (1.3 to 5.1)	5.4 (5.4 to 5.4)	112.2 (94.9 to 133.6)	33.1 (25.7 to 42.0)	56.4 (41.3 to 75.5)	7.6 (3.6 to 14.2)	15.1 (15.1 to 15.1)
Ghana	2002	38.4 (32.2 to 46.0)	11.4 (8.9 to 14.6)	20.0 (14.6 to 26.6)	2.6 (1.2 to 4.9)	4.4 (4.4 to 4.4)	108.1 (90.5 to 129.4)	32.2 (25.1 to 41.0)	56.3 (41.0 to 74.9)	7.3 (3.4 to 13.7)	12.3 (12.3 to 12.3)
Ghana	2003	42.9 (36.7 to 50.8)	12.3 (9.6 to 15.7)	20.4 (14.7 to 27.4)	2.7 (1.2 to 5.2)	7.5 (7.5 to 7.5)	120.6 (103.1 to 142.7)	34.6 (27.1 to 44.1)	57.3 (41.4 to 77.0)	7.6 (3.4 to 14.6)	21.1 (21.1 to 21.1)
Ghana	2004	48.7 (42.2 to 57.1)	13.8 (10.8 to 17.5)	21.0 (15.5 to 28.2)	2.9 (1.3 to 5.5)	11.0 (11.0 to 11.0)	136.9 (118.5 to 160.6)	38.7 (30.4 to 49.2)	59.1 (43.7 to 79.1)	8.2 (3.8 to 15.6)	30.9 (30.9 to 30.9)
Ghana	2005	54.6 (47.2 to 64.1)	17.6 (13.7 to 22.6)	22.4 (16.3 to 29.7)	3.6 (1.7 to 6.8)	10.9 (10.9 to 10.9)	153.4 (132.8 to 180.1)	49.6 (38.6 to 63.4)	63.0 (45.9 to 83.5)	10.2 (4.7 to 19.0)	30.6 (30.6 to 30.6)
Ghana	2006	56.8 (48.7 to 66.6)	20.5 (15.7 to 26.1)	23.1 (16.9 to 30.5)	3.8 (1.8 to 7.3)	9.5 (9.5 to 9.5)	159.6 (136.9 to 187.3)	57.5 (44.1 to 73.3)	64.8 (47.6 to 85.6)	10.7 (5.0 to 20.5)	26.6 (26.6 to 26.6)
Ghana	2007	60.5 (51.8 to 71.2)	23.2 (17.9 to 29.3)	23.7 (17.7 to 31.2)	4.0 (1.8 to 7.6)	9.6 (9.6 to 9.6)	170.0 (145.6 to 200.1)	65.3 (50.3 to 82.3)	66.5 (49.7 to 87.6)	11.2 (5.1 to 21.4)	27.0 (27.0 to 27.0)
Ghana	2008	67.2 (57.8 to 78.5)	26.9 (20.9 to 33.8)	24.9 (18.1 to 32.7)	4.3 (2.0 to 8.2)	11.1 (11.1 to 11.1)	188.9 (162.5 to 220.6)	75.7 (58.6 to 95.1)	69.9 (50.9 to 92.0)	12.2 (5.5 to 23.1)	31.1 (31.1 to 31.1)

		2018 US Dollars per capita					2018 PPP per capita				
Country	Year	Health spending	Government health spending	Out-of-pocket health spending	Prepaid private health spending	Development assistance for health	Health spending	Government health spending	Out-of-pocket health spending	Prepaid private health spending	Development assistance for health
Ghana	2009	71.7 (62.1 to 83.9)	30.5 (23.7 to 38.7)	25.5 (18.7 to 33.9)	4.7 (2.1 to 8.8)	11.1 (11.1 to 11.1)	201.7 (174.4 to 235.7)	85.9 (66.7 to 108.9)	71.6 (52.7 to 95.4)	13.2 (6.0 to 24.7)	31.1 (31.1 to 31.1)
Ghana	2010	76.2 (65.5 to 88.8)	34.1 (26.7 to 43.3)	26.8 (19.5 to 35.3)	4.6 (2.1 to 8.6)	10.7 (10.7 to 10.7)	214.2 (184.2 to 249.7)	95.9 (74.9 to 121.6)	75.4 (54.9 to 99.3)	12.9 (5.8 to 24.1)	30.0 (30.0 to 30.0)
Ghana	2011	84.0 (72.4 to 97.7)	38.6 (30.0 to 48.6)	30.1 (21.6 to 39.6)	4.4 (2.0 to 8.1)	10.9 (10.9 to 10.9)	236.1 (203.4 to 274.6)	108.5 (84.3 to 136.5)	84.6 (60.8 to 111.2)	12.3 (5.6 to 22.8)	30.6 (30.6 to 30.6)
Ghana	2012	88.4 (75.9 to 102.6)	38.8 (30.3 to 49.3)	32.2 (23.2 to 43.0)	3.9 (1.8 to 7.5)	13.4 (13.4 to 13.4)	248.5 (213.4 to 288.4)	109.2 (85.1 to 138.6)	90.4 (65.2 to 120.7)	11.1 (5.2 to 21.1)	37.8 (37.8 to 37.8)
Ghana	2013	87.0 (74.1 to 101.4)	37.0 (28.7 to 46.6)	33.8 (24.3 to 45.4)	3.6 (1.7 to 6.9)	12.6 (12.6 to 12.6)	244.5 (208.2 to 284.9)	103.9 (80.6 to 130.9)	95.0 (68.3 to 127.5)	10.2 (4.9 to 19.3)	35.4 (35.4 to 35.4)
Ghana	2014	80.4 (67.8 to 94.5)	33.6 (26.2 to 42.7)	34.0 (24.9 to 45.6)	3.8 (1.8 to 7.4)	9.0 (9.0 to 9.0)	226.0 (190.5 to 265.5)	94.4 (73.7 to 120.0)	95.5 (69.9 to 128.1)	10.7 (5.1 to 20.7)	25.3 (25.3 to 25.3)
Ghana	2015	80.0 (67.8 to 93.4)	32.1 (25.2 to 40.5)	32.3 (23.2 to 43.3)	4.3 (2.1 to 8.4)	11.2 (11.2 to 11.2)	224.8 (190.7 to 262.6)	90.4 (70.7 to 113.7)	90.8 (65.1 to 121.6)	12.1 (5.9 to 23.6)	31.4 (31.4 to 31.4)
Ghana	2016	74.7 (62.6 to 87.9)	29.8 (23.4 to 37.5)	29.5 (20.8 to 39.6)	5.3 (2.6 to 10.2)	10.1 (10.1 to 10.1)	210.1 (175.9 to 246.9)	83.7 (65.7 to 105.5)	83.0 (58.5 to 111.3)	14.8 (7.3 to 28.8)	28.5 (28.5 to 28.5)
Ghana	2017	74.3 (62.8 to 86.7)	29.0 (22.7 to 36.5)	31.0 (21.8 to 41.9)	5.3 (2.6 to 10.3)	9.0 (9.0 to 9.0)	208.8 (176.5 to 243.7)	81.5 (63.9 to 102.7)	87.0 (61.3 to 117.6)	14.9 (7.3 to 28.9)	25.3 (25.3 to 25.3)
Ghana	2018	75.9 (63.9 to 90.6)	30.2 (23.7 to 38.0)	31.8 (22.4 to 43.0)	5.6 (2.7 to 10.8)	8.4 (8.3 to 8.4)	213.3 (179.6 to 254.6)	84.9 (66.6 to 106.7)	89.4 (63.0 to 120.8)	15.6 (7.6 to 30.3)	23.5 (23.4 to 23.5)
Ghana	2019	77.8 (64.9 to 91.9)	31.2 (24.4 to 39.3)	32.5 (23.0 to 44.0)	5.7 (2.8 to 11.1)	8.3 (7.8 to 8.8)	218.6 (182.4 to 258.4)	87.6 (68.6 to 110.4)	91.5 (64.5 to 123.8)	16.1 (7.9 to 31.3)	23.4 (21.8 to 24.7)
Ghana	2020	79.8 (67.2 to 94.6)	32.2 (25.2 to 40.7)	33.3 (23.5 to 45.0)	5.9 (2.9 to 11.5)	8.3 (7.7 to 9.0)	224.2 (188.8 to 266.0)	90.6 (70.8 to 114.3)	93.6 (66.0 to 126.4)	16.6 (8.1 to 32.2)	23.5 (21.5 to 25.3)
Ghana	2021	81.8 (68.6 to 96.9)	33.3 (26.0 to 42.1)	34.0 (24.0 to 46.0)	6.1 (3.0 to 11.8)	8.4 (7.5 to 9.3)	229.9 (192.8 to 272.3)	93.6 (73.1 to 118.4)	95.7 (67.5 to 129.2)	17.1 (8.4 to 33.2)	23.5 (21.2 to 26.0)
Ghana	2022	83.9 (70.3 to 99.9)	34.4 (26.9 to 43.3)	34.8 (24.5 to 46.9)	6.3 (3.1 to 12.2)	8.5 (7.5 to 9.5)	235.8 (197.6 to 280.7)	96.7 (75.7 to 121.6)	97.7 (68.9 to 131.9)	17.6 (8.6 to 34.3)	23.8 (21.0 to 26.6)
Ghana	2023	86.0 (72.6 to 102.0)	35.5 (27.8 to 44.9)	35.5 (25.0 to 47.9)	6.4 (3.2 to 12.6)	8.5 (7.4 to 9.7)	241.6 (204.1 to 286.8)	99.9 (78.1 to 126.1)	99.7 (70.3 to 134.5)	18.1 (8.9 to 35.4)	24.0 (20.8 to 27.3)
Ghana	2024	88.1 (73.8 to 105.7)	36.7 (28.7 to 46.3)	36.2 (25.4 to 48.7)	6.6 (3.2 to 13.0)	8.6 (7.4 to 10.0)	247.7 (207.4 to 297.2)	103.2 (80.6 to 130.3)	101.6 (71.5 to 137.0)	18.6 (9.1 to 36.4)	24.2 (20.8 to 28.0)
Ghana	2025	90.4 (76.3 to 106.9)	38.0 (29.6 to 47.9)	36.9 (26.0 to 49.8)	6.8 (3.3 to 13.4)	8.7 (7.4 to 10.2)	254.1 (214.5 to 300.6)	106.7 (83.3 to 134.6)	103.7 (73.1 to 139.9)	19.2 (9.4 to 37.5)	24.5 (20.9 to 28.8)
Ghana	2026	92.8 (77.7 to 110.7)	39.3 (30.7 to 49.6)	37.6 (26.6 to 50.8)	7.0 (3.4 to 13.8)	8.8 (7.5 to 10.3)	260.7 (218.5 to 311.1)	110.4 (86.2 to 139.5)	105.8 (74.7 to 142.9)	19.7 (9.6 to 38.7)	24.8 (21.0 to 29.0)
Ghana	2027	95.3 (79.4 to 115.3)	40.7 (31.7 to 51.7)	38.4 (27.1 to 51.8)	7.2 (3.5 to 14.2)	8.9 (7.5 to 10.6)	267.9 (223.3 to 324.2)	114.4 (89.1 to 145.2)	108.0 (76.3 to 145.6)	20.3 (9.9 to 39.9)	25.1 (21.0 to 29.9)
Ghana	2028	98.0 (82.3 to 116.3)	42.2 (32.9 to 53.5)	39.3 (27.8 to 52.9)	7.5 (3.6 to 14.6)	9.1 (7.6 to 11.0)	275.4 (231.3 to 326.9)	118.5 (92.4 to 150.4)	110.4 (78.0 to 148.8)	21.0 (10.2 to 41.1)	25.5 (21.3 to 30.8)
Ghana	2029	100.8 (83.7 to 120.2)	43.7 (34.1 to 55.4)	40.2 (28.4 to 54.5)	7.7 (3.7 to 15.1)	9.2 (7.6 to 11.2)	283.3 (235.3 to 337.9)	122.9 (95.7 to 155.8)	112.9 (79.8 to 153.2)	21.6 (10.5 to 42.4)	25.8 (21.2 to 31.5)

Country	Year	2018 US Dollars per capita					2018 PPP per capita				
		Health spending	Government health spending	Out-of-pocket health spending	Prepaid private health spending	Development assistance for health	Health spending	Government health spending	Out-of-pocket health spending	Prepaid private health spending	Development assistance for health
Ghana	2030	103.7 (86.2 to 122.2)	45.4 (35.3 to 57.8)	41.1 (29.0 to 56.0)	7.9 (3.8 to 15.5)	9.3 (7.6 to 11.4)	291.5 (242.2 to 343.4)	127.5 (99.3 to 162.4)	115.5 (81.5 to 157.4)	22.3 (10.8 to 43.7)	26.2 (21.4 to 32.1)
Ghana	2031	106.8 (88.1 to 127.8)	47.1 (36.6 to 60.1)	42.0 (29.6 to 57.3)	8.2 (4.0 to 16.0)	9.4 (7.7 to 11.9)	300.2 (247.6 to 359.2)	132.5 (102.8 to 168.9)	118.2 (83.1 to 161.1)	23.0 (11.1 to 45.1)	26.5 (21.6 to 33.3)
Ghana	2032	110.0 (92.0 to 130.6)	49.0 (38.1 to 62.5)	43.0 (30.2 to 58.6)	8.4 (4.1 to 16.6)	9.6 (7.7 to 12.0)	309.2 (258.7 to 367.0)	137.6 (107.0 to 175.6)	120.9 (84.9 to 164.6)	23.7 (11.5 to 46.6)	26.9 (21.7 to 33.9)
Ghana	2033	113.4 (93.3 to 135.4)	50.9 (39.5 to 64.8)	44.1 (31.0 to 59.9)	8.7 (4.2 to 17.1)	9.7 (7.7 to 12.5)	318.7 (262.4 to 380.5)	143.0 (110.9 to 182.2)	123.8 (87.1 to 168.4)	24.5 (11.8 to 48.2)	27.3 (21.7 to 35.0)
Ghana	2034	116.9 (97.0 to 139.2)	52.9 (40.9 to 67.6)	45.1 (31.8 to 61.6)	9.0 (4.3 to 17.6)	9.9 (7.8 to 12.7)	328.4 (272.7 to 391.3)	148.6 (115.0 to 189.9)	126.8 (89.3 to 173.0)	25.3 (12.2 to 49.6)	27.8 (21.8 to 35.8)
Ghana	2035	120.4 (100.2 to 143.1)	54.9 (42.6 to 70.2)	46.2 (32.5 to 63.0)	9.3 (4.5 to 18.2)	10.0 (7.8 to 13.1)	338.5 (281.8 to 402.3)	154.4 (119.6 to 197.4)	129.8 (91.5 to 177.0)	26.1 (12.6 to 51.1)	28.2 (22.0 to 36.9)
Ghana	2036	124.1 (103.2 to 149.8)	57.1 (44.0 to 73.0)	47.3 (33.3 to 64.5)	9.6 (4.6 to 18.8)	10.2 (7.9 to 13.6)	348.8 (290.1 to 420.9)	160.4 (123.7 to 205.1)	132.9 (93.6 to 181.3)	26.9 (13.0 to 52.9)	28.6 (22.2 to 38.3)
Ghana	2037	127.9 (106.2 to 152.6)	59.3 (45.7 to 76.0)	48.4 (34.1 to 66.0)	9.9 (4.8 to 19.4)	10.3 (8.0 to 13.9)	359.4 (298.5 to 428.8)	166.6 (128.5 to 213.5)	136.0 (95.9 to 185.6)	27.8 (13.4 to 54.6)	29.0 (22.4 to 39.1)
Ghana	2038	131.7 (107.9 to 159.2)	61.5 (47.3 to 79.1)	49.5 (34.9 to 67.7)	10.2 (4.9 to 20.0)	10.5 (8.0 to 14.4)	370.2 (303.1 to 447.6)	172.9 (133.1 to 222.2)	139.2 (98.1 to 190.3)	28.7 (13.8 to 56.3)	29.5 (22.6 to 40.5)
Ghana	2039	135.6 (113.1 to 161.4)	63.8 (48.9 to 82.5)	50.7 (35.7 to 69.3)	10.5 (5.1 to 20.7)	10.6 (8.1 to 14.6)	381.3 (317.8 to 453.7)	179.4 (137.5 to 232.0)	142.5 (100.3 to 194.8)	29.6 (14.3 to 58.1)	29.9 (22.8 to 41.1)
Ghana	2040	139.7 (115.4 to 167.2)	66.2 (50.8 to 85.9)	51.9 (36.5 to 71.0)	10.8 (5.2 to 21.2)	10.8 (8.2 to 14.9)	392.8 (324.2 to 470.1)	186.2 (142.8 to 241.4)	145.8 (102.5 to 199.5)	30.5 (14.7 to 59.6)	30.3 (23.2 to 41.8)
Ghana	2041	143.9 (119.7 to 172.8)	68.7 (52.8 to 89.1)	53.1 (37.3 to 72.6)	11.2 (5.4 to 21.8)	11.0 (8.2 to 15.6)	404.5 (336.4 to 485.6)	193.2 (148.4 to 250.4)	149.1 (104.9 to 204.0)	31.4 (15.1 to 61.2)	30.8 (23.1 to 43.7)
Ghana	2042	148.2 (122.1 to 177.9)	71.3 (54.8 to 92.5)	54.3 (38.1 to 74.2)	11.5 (5.5 to 22.5)	11.1 (8.3 to 16.2)	416.6 (343.1 to 500.1)	200.4 (154.0 to 260.0)	152.5 (107.2 to 208.6)	32.4 (15.6 to 63.2)	31.3 (23.3 to 45.7)
Ghana	2043	152.6 (127.2 to 182.6)	74.0 (57.0 to 96.2)	55.5 (39.0 to 75.8)	11.9 (5.7 to 23.3)	11.3 (8.4 to 16.6)	429.0 (357.4 to 513.3)	207.9 (160.2 to 270.3)	156.0 (109.6 to 213.2)	33.3 (16.0 to 65.4)	31.8 (23.5 to 46.5)
Ghana	2044	157.2 (130.7 to 190.0)	76.7 (58.9 to 99.9)	56.8 (39.9 to 77.6)	12.2 (5.9 to 24.0)	11.5 (8.5 to 16.8)	441.9 (367.4 to 534.1)	215.6 (165.5 to 280.8)	159.5 (112.1 to 218.2)	34.3 (16.4 to 67.5)	32.4 (23.9 to 47.2)
Ghana	2045	161.9 (133.1 to 194.4)	79.5 (60.9 to 104.0)	58.0 (40.8 to 79.5)	12.6 (6.0 to 24.8)	11.7 (8.5 to 17.7)	455.0 (374.1 to 546.3)	223.5 (171.2 to 292.4)	163.1 (114.6 to 223.3)	35.4 (16.9 to 69.6)	32.9 (23.8 to 49.7)
Ghana	2046	166.6 (137.4 to 201.8)	82.4 (63.0 to 107.7)	59.3 (41.8 to 81.3)	13.0 (6.2 to 25.5)	11.9 (8.6 to 18.2)	468.3 (386.1 to 567.2)	231.6 (176.9 to 302.8)	166.7 (117.4 to 228.5)	36.4 (17.3 to 71.6)	33.5 (24.2 to 51.1)
Ghana	2047	171.4 (140.6 to 210.2)	85.3 (65.0 to 111.5)	60.6 (42.8 to 83.1)	13.3 (6.3 to 26.2)	12.1 (8.8 to 19.0)	481.8 (395.3 to 590.8)	239.8 (182.6 to 313.4)	170.3 (120.2 to 233.6)	37.5 (17.8 to 73.7)	34.1 (24.7 to 53.3)
Ghana	2048	176.3 (144.7 to 213.2)	88.3 (66.9 to 115.5)	61.9 (43.7 to 84.9)	13.7 (6.5 to 27.0)	12.4 (8.9 to 19.7)	495.5 (406.7 to 599.2)	248.1 (188.1 to 324.6)	174.0 (122.9 to 238.6)	38.6 (18.2 to 75.9)	34.8 (24.9 to 55.3)
Ghana	2049	181.2 (147.2 to 221.7)	91.3 (69.5 to 119.8)	63.2 (44.6 to 86.7)	14.1 (6.7 to 27.8)	12.6 (8.9 to 20.4)	509.4 (413.8 to 623.2)	256.6 (195.2 to 336.8)	177.7 (125.4 to 243.6)	39.7 (18.7 to 78.1)	35.4 (25.0 to 57.3)
Ghana	2050	186.3 (153.9 to 227.5)	94.3 (71.5 to 124.5)	64.5 (45.5 to 88.5)	14.5 (6.8 to 28.6)	12.9 (9.1 to 21.0)	523.6 (432.6 to 639.5)	265.1 (201.0 to 350.0)	181.4 (128.0 to 248.6)	40.8 (19.2 to 80.3)	36.2 (25.5 to 59.0)

Country	Year	2018 US Dollars per capita					2018 PPP per capita				
		Health spending	Government health spending	Out-of-pocket health spending	Prepaid private health spending	Development assistance for health	Health spending	Government health spending	Out-of-pocket health spending	Prepaid private health spending	Development assistance for health
Greece	1995	1355.6 (1270.7 to 1447.1)	689.7 (628.7 to 753.5)	633.2 (572.5 to 699.0)	32.7 (21.0 to 49.6)	0.0 (0.0 to 0.0)	1915.8 (1795.9 to 2045.0)	974.6 (888.5 to 1064.9)	894.9 (809.0 to 987.8)	46.2 (29.6 to 70.0)	0.0 (0.0 to 0.0)
Greece	1996	1376.4 (1305.7 to 1452.6)	708.8 (657.4 to 761.4)	634.2 (584.3 to 686.4)	33.5 (22.3 to 48.8)	0.0 (0.0 to 0.0)	1945.2 (1845.3 to 2052.9)	1001.6 (929.0 to 1076.0)	896.3 (825.8 to 970.1)	47.3 (31.4 to 68.9)	0.0 (0.0 to 0.0)
Greece	1997	1412.1 (1343.6 to 1482.9)	738.9 (690.3 to 790.0)	638.8 (594.2 to 684.7)	34.4 (23.8 to 48.7)	0.0 (0.0 to 0.0)	1995.6 (1898.8 to 2095.7)	1044.2 (975.5 to 1116.5)	902.8 (839.8 to 967.7)	48.7 (33.6 to 68.8)	0.0 (0.0 to 0.0)
Greece	1998	1441.6 (1374.1 to 1509.8)	770.8 (723.5 to 820.0)	635.4 (589.6 to 685.6)	35.4 (24.9 to 48.4)	0.0 (0.0 to 0.0)	2037.3 (1942.0 to 2133.6)	1089.4 (1022.5 to 1158.8)	897.9 (833.2 to 968.9)	50.0 (35.1 to 68.4)	0.0 (0.0 to 0.0)
Greece	1999	1477.0 (1410.3 to 1546.1)	820.7 (771.4 to 874.3)	619.1 (575.6 to 664.5)	37.2 (26.9 to 49.4)	0.0 (0.0 to 0.0)	2087.3 (1993.1 to 2185.0)	1159.9 (1090.1 to 1235.6)	874.9 (813.4 to 939.1)	52.6 (38.0 to 69.8)	0.0 (0.0 to 0.0)
Greece	2000	1524.2 (1459.7 to 1592.1)	895.0 (846.9 to 947.5)	588.3 (545.0 to 630.4)	40.9 (30.3 to 53.5)	0.0 (0.0 to 0.0)	2154.1 (2062.8 to 2250.0)	1264.8 (1196.9 to 1339.1)	831.5 (770.3 to 890.9)	57.8 (42.8 to 75.6)	0.0 (0.0 to 0.0)
Greece	2001	1635.1 (1567.2 to 1702.8)	987.0 (937.9 to 1039.2)	602.8 (561.5 to 645.0)	45.3 (34.3 to 58.3)	0.0 (0.0 to 0.0)	2310.8 (2214.8 to 2406.4)	1394.8 (1325.5 to 1468.7)	851.9 (793.5 to 911.6)	64.1 (48.4 to 82.4)	0.0 (0.0 to 0.0)
Greece	2002	1747.7 (1681.8 to 1824.9)	1059.6 (1004.8 to 1115.8)	639.0 (598.8 to 681.1)	49.1 (37.9 to 61.6)	0.0 (0.0 to 0.0)	2469.9 (2376.8 to 2578.9)	1497.5 (1420.0 to 1576.9)	903.1 (846.3 to 962.5)	69.4 (53.6 to 87.0)	0.0 (0.0 to 0.0)
Greece	2003	1863.0 (1794.5 to 1937.3)	1135.0 (1080.6 to 1189.2)	675.6 (632.6 to 720.7)	52.4 (41.2 to 65.2)	0.0 (0.0 to 0.0)	2632.8 (2536.0 to 2737.9)	1604.0 (1527.1 to 1680.6)	954.8 (894.0 to 1018.5)	74.1 (58.2 to 92.2)	0.0 (0.0 to 0.0)
Greece	2004	1998.5 (1929.1 to 2070.2)	1219.2 (1166.1 to 1272.5)	723.5 (676.4 to 769.9)	55.8 (44.0 to 69.1)	0.0 (0.0 to 0.0)	2824.3 (2726.2 to 2925.7)	1723.0 (1647.9 to 1798.3)	1022.4 (956.0 to 1088.0)	78.9 (62.1 to 97.6)	0.0 (0.0 to 0.0)
Greece	2005	2116.3 (2046.9 to 2189.9)	1285.7 (1234.3 to 1338.4)	771.7 (725.8 to 819.6)	58.9 (46.5 to 72.2)	0.0 (0.0 to 0.0)	2990.8 (2892.8 to 3094.8)	1817.0 (1744.4 to 1891.5)	1090.6 (1025.8 to 1158.3)	83.2 (65.8 to 102.1)	0.0 (0.0 to 0.0)
Greece	2006	2295.6 (2222.7 to 2368.1)	1406.8 (1351.8 to 1462.8)	825.3 (780.0 to 875.9)	63.5 (50.4 to 77.6)	0.0 (0.0 to 0.0)	3244.2 (3141.2 to 3346.6)	1988.1 (1910.5 to 2067.3)	1166.3 (1102.4 to 1237.9)	89.8 (71.3 to 109.7)	0.0 (0.0 to 0.0)
Greece	2007	2414.4 (2335.9 to 2487.3)	1480.3 (1426.9 to 1535.8)	866.5 (820.1 to 918.1)	67.6 (54.7 to 82.2)	0.0 (0.0 to 0.0)	3412.1 (3301.2 to 3515.1)	2092.0 (2016.6 to 2170.5)	1224.6 (1159.0 to 1297.4)	95.5 (77.3 to 116.2)	0.0 (0.0 to 0.0)
Greece	2008	2469.0 (2393.2 to 2546.1)	1531.2 (1472.6 to 1586.9)	865.8 (818.9 to 913.3)	72.1 (58.7 to 87.9)	0.0 (0.0 to 0.0)	3489.3 (3382.1 to 3598.2)	2163.9 (2081.1 to 2242.7)	1223.5 (1157.3 to 1290.7)	101.9 (82.9 to 124.3)	0.0 (0.0 to 0.0)
Greece	2009	2412.0 (2335.7 to 2485.8)	1565.9 (1504.6 to 1624.5)	770.0 (726.5 to 817.6)	76.1 (61.8 to 92.7)	0.0 (0.0 to 0.0)	3408.7 (3300.9 to 3513.0)	2212.9 (2126.3 to 2295.7)	1088.2 (1026.8 to 1155.5)	107.6 (87.4 to 131.0)	0.0 (0.0 to 0.0)
Greece	2010	2264.5 (2188.9 to 2338.4)	1498.2 (1440.2 to 1558.6)	687.3 (646.2 to 731.9)	79.0 (64.7 to 96.9)	0.0 (0.0 to 0.0)	3200.3 (3093.4 to 3304.7)	2117.2 (2035.3 to 2202.7)	971.4 (913.3 to 1034.3)	111.7 (91.4 to 136.9)	0.0 (0.0 to 0.0)
Greece	2011	2009.5 (1939.0 to 2080.6)	1317.0 (1265.1 to 1376.1)	617.2 (578.2 to 662.8)	75.3 (61.5 to 91.2)	0.0 (0.0 to 0.0)	2839.9 (2740.2 to 2940.3)	1861.3 (1787.9 to 1944.8)	872.2 (817.1 to 936.7)	106.4 (87.0 to 128.8)	0.0 (0.0 to 0.0)
Greece	2012	1819.7 (1749.1 to 1890.2)	1173.8 (1121.1 to 1230.5)	573.5 (535.3 to 615.5)	72.4 (59.2 to 88.1)	0.0 (0.0 to 0.0)	2571.7 (2471.9 to 2671.4)	1658.9 (1584.4 to 1738.9)	810.5 (756.5 to 869.8)	102.3 (83.6 to 124.5)	0.0 (0.0 to 0.0)
Greece	2013	1700.9 (1633.3 to 1771.7)	1064.0 (1014.3 to 1119.3)	566.4 (529.6 to 606.0)	70.5 (57.3 to 85.9)	0.0 (0.0 to 0.0)	2403.8 (2308.3 to 2503.8)	1503.7 (1433.5 to 1581.9)	800.5 (748.5 to 856.4)	99.7 (80.9 to 121.4)	0.0 (0.0 to 0.0)
Greece	2014	1647.6 (1583.8 to 1710.8)	998.1 (945.8 to 1051.6)	577.4 (538.3 to 619.6)	72.1 (58.0 to 87.7)	0.0 (0.0 to 0.0)	2328.4 (2238.3 to 2417.7)	1410.5 (1336.6 to 1486.1)	816.0 (760.8 to 875.6)	101.9 (81.9 to 123.9)	0.0 (0.0 to 0.0)
Greece	2015	1658.8 (1581.0 to 1729.7)	989.4 (931.7 to 1048.5)	593.2 (548.4 to 639.9)	76.2 (58.7 to 94.7)	0.0 (0.0 to 0.0)	2344.2 (2234.3 to 2444.5)	1398.2 (1316.8 to 1481.7)	838.3 (775.0 to 904.4)	107.7 (82.9 to 133.9)	0.0 (0.0 to 0.0)

		2018 US Dollars per capita					2018 PPP per capita				
Country	Year	Health spending	Government health spending	Out-of-pocket health spending	Prepaid private health spending	Development assistance for health	Health spending	Government health spending	Out-of-pocket health spending	Prepaid private health spending	Development assistance for health
Greece	2016	1692.7 (1601.3 to 1789.7)	1010.0 (945.5 to 1087.3)	602.2 (542.8 to 664.7)	80.5 (58.9 to 105.0)	0.0 (0.0 to 0.0)	2392.2 (2263.0 to 2529.3)	1427.4 (1336.2 to 1536.5)	851.0 (767.0 to 939.4)	113.8 (83.2 to 148.4)	0.0 (0.0 to 0.0)
Greece	2017	1704.2 (1606.2 to 1809.5)	1016.3 (950.5 to 1095.0)	606.6 (547.2 to 670.6)	81.3 (59.5 to 105.8)	0.0 (0.0 to 0.0)	2408.4 (2270.0 to 2557.3)	1436.2 (1343.3 to 1547.4)	857.3 (773.4 to 947.7)	114.9 (84.2 to 149.5)	0.0 (0.0 to 0.0)
Greece	2018	1714.0 (1620.6 to 1814.6)	1024.5 (957.2 to 1105.0)	607.3 (547.8 to 671.0)	82.2 (60.0 to 107.1)	0.0 (0.0 to 0.0)	2422.3 (2290.3 to 2564.4)	1447.9 (1352.7 to 1561.6)	858.3 (774.2 to 948.3)	116.1 (84.7 to 151.4)	0.0 (0.0 to 0.0)
Greece	2019	1720.8 (1623.1 to 1829.1)	1030.8 (961.4 to 1110.9)	607.2 (547.4 to 671.0)	82.7 (60.4 to 107.9)	0.0 (0.0 to 0.0)	2431.8 (2293.8 to 2584.9)	1456.8 (1358.7 to 1570.0)	858.1 (773.7 to 948.3)	116.9 (85.3 to 152.5)	0.0 (0.0 to 0.0)
Greece	2020	1728.3 (1632.5 to 1830.5)	1037.8 (967.3 to 1117.6)	607.0 (547.9 to 670.1)	83.4 (60.8 to 109.1)	0.0 (0.0 to 0.0)	2442.4 (2307.2 to 2586.9)	1466.7 (1367.0 to 1579.4)	857.9 (774.3 to 947.0)	117.8 (86.0 to 154.2)	0.0 (0.0 to 0.0)
Greece	2021	1735.2 (1635.3 to 1846.7)	1044.9 (974.2 to 1128.2)	606.3 (547.5 to 669.5)	84.0 (61.3 to 109.9)	0.0 (0.0 to 0.0)	2452.3 (2311.1 to 2609.8)	1476.7 (1376.8 to 1594.3)	856.9 (773.8 to 946.1)	118.7 (86.6 to 155.3)	0.0 (0.0 to 0.0)
Greece	2022	1744.4 (1646.6 to 1846.2)	1054.0 (981.0 to 1139.0)	605.8 (546.8 to 668.9)	84.6 (61.7 to 110.7)	0.0 (0.0 to 0.0)	2465.3 (2327.1 to 2609.1)	1489.5 (1386.4 to 1609.7)	856.1 (772.8 to 945.3)	119.6 (87.2 to 156.4)	0.0 (0.0 to 0.0)
Greece	2023	1752.3 (1650.5 to 1863.8)	1062.0 (987.9 to 1145.5)	605.1 (542.9 to 667.1)	85.3 (62.1 to 111.5)	0.0 (0.0 to 0.0)	2476.4 (2332.6 to 2633.9)	1500.8 (1396.2 to 1618.8)	855.1 (767.3 to 942.7)	120.5 (87.8 to 157.5)	0.0 (0.0 to 0.0)
Greece	2024	1760.5 (1661.1 to 1862.4)	1070.1 (995.4 to 1154.2)	604.5 (544.3 to 666.2)	85.9 (62.5 to 112.3)	0.0 (0.0 to 0.0)	2488.0 (2347.5 to 2632.0)	1512.4 (1406.8 to 1631.1)	854.2 (769.3 to 941.5)	121.5 (88.3 to 158.7)	0.0 (0.0 to 0.0)
Greece	2025	1772.6 (1668.1 to 1886.7)	1080.7 (1003.7 to 1167.6)	605.2 (545.0 to 668.0)	86.8 (63.1 to 113.5)	0.0 (0.0 to 0.0)	2505.1 (2357.4 to 2666.3)	1527.2 (1418.5 to 1650.1)	855.2 (770.3 to 944.0)	122.7 (89.2 to 160.4)	0.0 (0.0 to 0.0)
Greece	2026	1783.2 (1682.3 to 1894.8)	1090.3 (1008.8 to 1181.0)	605.3 (544.2 to 667.7)	87.6 (63.6 to 114.4)	0.0 (0.0 to 0.0)	2520.1 (2377.5 to 2677.8)	1540.8 (1425.7 to 1669.1)	855.4 (769.1 to 943.6)	123.8 (89.9 to 161.7)	0.0 (0.0 to 0.0)
Greece	2027	1796.3 (1687.9 to 1914.8)	1101.5 (1019.4 to 1195.2)	606.2 (545.0 to 668.2)	88.5 (64.3 to 115.6)	0.0 (0.0 to 0.0)	2538.5 (2385.4 to 2706.0)	1556.7 (1440.7 to 1689.0)	856.7 (770.2 to 944.3)	125.1 (90.9 to 163.4)	0.0 (0.0 to 0.0)
Greece	2028	1808.6 (1704.0 to 1929.6)	1112.4 (1027.8 to 1210.8)	606.8 (544.9 to 669.5)	89.4 (64.9 to 116.5)	0.0 (0.0 to 0.0)	2555.9 (2408.1 to 2727.0)	1572.1 (1452.5 to 1711.2)	857.5 (770.1 to 946.2)	126.3 (91.7 to 164.6)	0.0 (0.0 to 0.0)
Greece	2029	1821.4 (1709.5 to 1943.4)	1123.7 (1035.5 to 1226.6)	607.5 (544.6 to 672.3)	90.3 (65.5 to 117.5)	0.0 (0.0 to 0.0)	2574.0 (2415.9 to 2746.5)	1588.0 (1463.4 to 1733.5)	858.5 (769.6 to 950.1)	127.6 (92.6 to 166.1)	0.0 (0.0 to 0.0)
Greece	2030	1832.7 (1723.8 to 1954.1)	1134.2 (1043.5 to 1240.6)	607.5 (542.4 to 675.0)	91.1 (66.1 to 118.5)	0.0 (0.0 to 0.0)	2590.1 (2436.1 to 2761.7)	1602.8 (1474.6 to 1753.3)	858.5 (766.6 to 953.9)	128.7 (93.4 to 167.5)	0.0 (0.0 to 0.0)
Greece	2031	1841.7 (1722.3 to 1975.8)	1143.3 (1049.1 to 1257.1)	606.7 (539.5 to 675.8)	91.7 (66.5 to 119.4)	0.0 (0.0 to 0.0)	2602.8 (2434.0 to 2792.2)	1615.8 (1482.6 to 1776.6)	857.4 (762.5 to 955.0)	129.6 (94.0 to 168.7)	0.0 (0.0 to 0.0)
Greece	2032	1850.6 (1732.6 to 1982.0)	1152.5 (1054.0 to 1270.5)	605.7 (536.4 to 675.3)	92.4 (67.0 to 120.3)	0.0 (0.0 to 0.0)	2615.3 (2448.6 to 2801.0)	1628.7 (1489.5 to 1795.5)	856.0 (758.0 to 954.4)	130.5 (94.7 to 170.0)	0.0 (0.0 to 0.0)
Greece	2033	1856.7 (1728.2 to 2001.7)	1160.0 (1058.7 to 1285.8)	603.8 (530.7 to 675.0)	92.9 (67.4 to 121.3)	0.0 (0.0 to 0.0)	2624.0 (2442.3 to 2828.9)	1639.4 (1496.2 to 1817.1)	853.4 (750.0 to 954.0)	131.2 (95.2 to 171.4)	0.0 (0.0 to 0.0)
Greece	2034	1863.4 (1738.0 to 2013.6)	1167.9 (1060.3 to 1297.8)	602.1 (527.8 to 674.8)	93.4 (67.7 to 122.3)	0.0 (0.0 to 0.0)	2633.4 (2456.2 to 2845.7)	1650.6 (1498.5 to 1834.2)	850.9 (745.9 to 953.6)	132.0 (95.7 to 172.9)	0.0 (0.0 to 0.0)
Greece	2035	1868.1 (1732.8 to 2016.6)	1174.6 (1064.6 to 1310.0)	599.7 (525.1 to 674.0)	93.8 (67.9 to 122.6)	0.0 (0.0 to 0.0)	2640.1 (2448.8 to 2849.9)	1659.9 (1504.6 to 1851.4)	847.6 (742.1 to 952.5)	132.6 (96.0 to 173.3)	0.0 (0.0 to 0.0)
Greece	2036	1872.3 (1742.5 to 2032.4)	1180.6 (1066.4 to 1321.2)	597.4 (521.6 to 672.0)	94.3 (68.3 to 123.0)	0.0 (0.0 to 0.0)	2646.0 (2462.6 to 2872.3)	1668.5 (1507.1 to 1867.1)	844.3 (737.1 to 949.7)	133.3 (96.5 to 173.8)	0.0 (0.0 to 0.0)

		2018 US Dollars per capita					2018 PPP per capita				
Country	Year	Health spending	Government health spending	Out-of-pocket health spending	Prepaid private health spending	Development assistance for health	Health spending	Government health spending	Out-of-pocket health spending	Prepaid private health spending	Development assistance for health
Greece	2037	1874.9 (1730.5 to 2036.4)	1185.7 (1066.9 to 1331.0)	594.5 (519.2 to 669.7)	94.7 (68.6 to 123.5)	0.0 (0.0 to 0.0)	2649.7 (2445.6 to 2877.9)	1675.6 (1507.8 to 1880.9)	840.2 (733.8 to 946.4)	133.8 (96.9 to 174.6)	0.0 (0.0 to 0.0)
Greece	2038	1878.4 (1739.0 to 2045.5)	1191.3 (1068.6 to 1344.8)	592.0 (516.8 to 667.8)	95.1 (68.9 to 124.1)	0.0 (0.0 to 0.0)	2654.7 (2457.7 to 2890.7)	1683.6 (1510.1 to 1900.5)	836.7 (730.3 to 943.7)	134.4 (97.3 to 175.4)	0.0 (0.0 to 0.0)
Greece	2039	1880.8 (1726.3 to 2055.3)	1196.3 (1068.9 to 1357.0)	589.0 (513.5 to 665.3)	95.5 (69.2 to 124.6)	0.0 (0.0 to 0.0)	2658.0 (2439.6 to 2904.7)	1690.6 (1510.6 to 1917.8)	832.4 (725.7 to 940.2)	134.9 (97.9 to 176.2)	0.0 (0.0 to 0.0)
Greece	2040	1881.7 (1732.6 to 2068.4)	1200.3 (1068.5 to 1367.2)	585.6 (509.0 to 662.2)	95.7 (69.6 to 125.1)	0.0 (0.0 to 0.0)	2659.3 (2448.6 to 2923.1)	1696.4 (1510.1 to 1932.2)	827.7 (719.3 to 935.8)	135.3 (98.3 to 176.7)	0.0 (0.0 to 0.0)
Greece	2041	1883.6 (1721.5 to 2069.0)	1204.8 (1068.9 to 1378.4)	582.7 (505.6 to 659.8)	96.1 (70.0 to 125.6)	0.0 (0.0 to 0.0)	2661.9 (2432.9 to 2924.0)	1702.7 (1510.6 to 1948.0)	823.4 (714.5 to 932.4)	135.8 (98.9 to 177.5)	0.0 (0.0 to 0.0)
Greece	2042	1883.4 (1727.8 to 2083.3)	1208.0 (1067.4 to 1385.6)	579.0 (502.2 to 656.1)	96.4 (70.2 to 126.1)	0.0 (0.0 to 0.0)	2661.7 (2441.7 to 2944.2)	1707.2 (1508.4 to 1958.2)	818.3 (709.7 to 927.3)	136.2 (99.1 to 178.2)	0.0 (0.0 to 0.0)
Greece	2043	1883.5 (1712.4 to 2084.2)	1211.3 (1067.3 to 1394.9)	575.5 (498.9 to 652.4)	96.6 (70.4 to 126.5)	0.0 (0.0 to 0.0)	2661.8 (2420.0 to 2945.5)	1711.9 (1508.3 to 1971.4)	813.4 (705.1 to 922.0)	136.6 (99.5 to 178.8)	0.0 (0.0 to 0.0)
Greece	2044	1883.5 (1720.5 to 2091.8)	1214.6 (1066.7 to 1401.2)	572.1 (495.1 to 648.9)	96.9 (70.6 to 127.1)	0.0 (0.0 to 0.0)	2661.9 (2431.5 to 2956.1)	1716.4 (1507.5 to 1980.3)	808.4 (699.7 to 917.0)	137.0 (99.7 to 179.6)	0.0 (0.0 to 0.0)
Greece	2045	1884.4 (1709.8 to 2100.5)	1218.3 (1066.6 to 1410.6)	568.8 (491.7 to 645.5)	97.2 (70.5 to 127.6)	0.0 (0.0 to 0.0)	2663.0 (2416.3 to 2968.5)	1721.7 (1507.4 to 1993.5)	803.9 (694.8 to 912.3)	137.4 (99.7 to 180.4)	0.0 (0.0 to 0.0)
Greece	2046	1885.0 (1709.8 to 2096.0)	1221.6 (1066.8 to 1423.4)	565.8 (488.5 to 642.5)	97.6 (70.8 to 128.0)	0.0 (0.0 to 0.0)	2664.0 (2416.3 to 2962.1)	1726.4 (1507.6 to 2011.6)	799.6 (690.4 to 908.0)	138.0 (100.1 to 181.0)	0.0 (0.0 to 0.0)
Greece	2047	1887.7 (1707.2 to 2115.7)	1226.1 (1068.1 to 1435.0)	563.5 (486.1 to 639.8)	98.1 (71.3 to 128.6)	0.0 (0.0 to 0.0)	2667.8 (2412.7 to 2989.9)	1732.8 (1509.4 to 2027.9)	796.3 (687.0 to 904.1)	138.7 (100.7 to 181.8)	0.0 (0.0 to 0.0)
Greece	2048	1891.6 (1708.8 to 2106.9)	1231.4 (1070.2 to 1444.1)	561.5 (484.1 to 637.4)	98.7 (71.4 to 129.3)	0.0 (0.0 to 0.0)	2673.2 (2414.9 to 2977.6)	1740.2 (1512.4 to 2040.8)	793.5 (684.1 to 900.8)	139.5 (100.9 to 182.8)	0.0 (0.0 to 0.0)
Greece	2049	1896.6 (1712.4 to 2127.7)	1237.2 (1071.3 to 1452.6)	560.0 (482.8 to 635.2)	99.4 (71.7 to 130.4)	0.0 (0.0 to 0.0)	2680.4 (2420.0 to 3006.9)	1748.5 (1514.0 to 2052.8)	791.5 (682.3 to 897.7)	140.4 (101.3 to 184.2)	0.0 (0.0 to 0.0)
Greece	2050	1902.0 (1713.1 to 2133.9)	1243.2 (1073.7 to 1459.5)	558.8 (481.3 to 634.0)	100.1 (72.2 to 131.4)	0.0 (0.0 to 0.0)	2688.0 (2421.1 to 3015.7)	1756.9 (1517.4 to 2062.6)	789.7 (680.2 to 896.0)	141.5 (102.0 to 185.8)	0.0 (0.0 to 0.0)
Greenland	1995	2663.0 (2099.6 to 3341.1)	2662.4 (2098.9 to 3340.4)	0.3 (0.0 to 0.3)	0.3 (0.0 to 0.3)	0.0 (0.0 to 0.0)	2101.0 (1656.5 to 2635.9)	2100.5 (1655.9 to 2635.4)	0.2 (0.0 to 0.3)	0.2 (0.0 to 0.3)	0.0 (0.0 to 0.0)
Greenland	1996	2760.6 (2198.5 to 3474.5)	2760.0 (2198.2 to 3473.8)	0.3 (0.0 to 0.3)	0.3 (0.0 to 0.3)	0.0 (0.0 to 0.0)	2178.0 (1734.5 to 2741.2)	2177.5 (1734.3 to 2740.7)	0.2 (0.0 to 0.3)	0.2 (0.0 to 0.3)	0.0 (0.0 to 0.0)
Greenland	1997	2863.2 (2329.2 to 3554.6)	2862.6 (2328.5 to 3554.2)	0.3 (0.0 to 0.3)	0.3 (0.0 to 0.3)	0.0 (0.0 to 0.0)	2258.9 (1837.6 to 2804.4)	2258.4 (1837.1 to 2804.1)	0.2 (0.0 to 0.3)	0.2 (0.0 to 0.3)	0.0 (0.0 to 0.0)
Greenland	1998	3095.7 (2585.5 to 3763.9)	3095.1 (2584.8 to 3763.2)	0.3 (0.0 to 0.4)	0.3 (0.0 to 0.4)	0.0 (0.0 to 0.0)	2442.3 (2039.8 to 2969.5)	2441.9 (2039.2 to 2968.9)	0.2 (0.0 to 0.3)	0.2 (0.0 to 0.3)	0.0 (0.0 to 0.0)
Greenland	1999	3219.3 (2747.1 to 3813.7)	3218.7 (2746.4 to 3813.0)	0.3 (0.0 to 0.4)	0.3 (0.0 to 0.4)	0.0 (0.0 to 0.0)	2539.8 (2167.3 to 3008.8)	2539.4 (2166.7 to 3008.3)	0.2 (0.0 to 0.3)	0.2 (0.0 to 0.3)	0.0 (0.0 to 0.0)
Greenland	2000	3453.4 (3039.2 to 3952.3)	3452.9 (3038.4 to 3951.5)	0.3 (0.0 to 0.4)	0.3 (0.0 to 0.4)	0.0 (0.0 to 0.0)	2724.6 (2397.7 to 3118.2)	2724.2 (2397.1 to 3117.6)	0.2 (0.0 to 0.3)	0.2 (0.0 to 0.3)	0.0 (0.0 to 0.0)
Greenland	2001	3542.7 (3238.0 to 3901.7)	3542.3 (3237.3 to 3900.9)	0.2 (0.0 to 0.4)	0.2 (0.0 to 0.4)	0.0 (0.0 to 0.0)	2795.0 (2554.6 to 3078.2)	2794.6 (2554.0 to 3077.6)	0.2 (0.0 to 0.3)	0.2 (0.0 to 0.3)	0.0 (0.0 to 0.0)

		2018 US Dollars per capita					2018 PPP per capita				
Country	Year	Health spending	Government health spending	Out-of-pocket health spending	Prepaid private health spending	Development assistance for health	Health spending	Government health spending	Out-of-pocket health spending	Prepaid private health spending	Development assistance for health
Greenland	2002	3556.5 (3360.0 to 3791.8)	3556.1 (3359.6 to 3791.6)	0.2 (0.0 to 0.4)	0.2 (0.0 to 0.4)	0.0 (0.0 to 0.0)	2805.9 (2650.8 to 2991.5)	2805.6 (2650.5 to 2991.3)	0.2 (0.0 to 0.3)	0.2 (0.0 to 0.3)	0.0 (0.0 to 0.0)
Greenland	2003	3581.6 (3455.8 to 3708.3)	3581.2 (3455.4 to 3707.7)	0.2 (0.0 to 0.4)	0.2 (0.0 to 0.4)	0.0 (0.0 to 0.0)	2825.7 (2726.4 to 2925.6)	2825.4 (2726.1 to 2925.2)	0.2 (0.0 to 0.3)	0.2 (0.0 to 0.3)	0.0 (0.0 to 0.0)
Greenland	2004	3696.8 (3604.2 to 3795.3)	3696.3 (3603.7 to 3794.5)	0.2 (0.0 to 0.4)	0.2 (0.0 to 0.4)	0.0 (0.0 to 0.0)	2916.5 (2843.5 to 2994.3)	2916.2 (2843.2 to 2993.6)	0.2 (0.0 to 0.3)	0.2 (0.0 to 0.3)	0.0 (0.0 to 0.0)
Greenland	2005	3751.9 (3662.4 to 3846.1)	3751.5 (3662.4 to 3845.7)	0.2 (0.0 to 0.4)	0.2 (0.0 to 0.4)	0.0 (0.0 to 0.0)	2960.1 (2889.5 to 3034.4)	2959.7 (2889.5 to 3034.0)	0.2 (0.0 to 0.3)	0.2 (0.0 to 0.3)	0.0 (0.0 to 0.0)
Greenland	2006	3978.7 (3883.1 to 4072.2)	3978.2 (3882.6 to 4072.2)	0.2 (0.0 to 0.5)	0.2 (0.0 to 0.5)	0.0 (0.0 to 0.0)	3138.9 (3063.5 to 3212.7)	3138.6 (3063.2 to 3212.7)	0.2 (0.0 to 0.4)	0.2 (0.0 to 0.4)	0.0 (0.0 to 0.0)
Greenland	2007	4178.5 (4086.8 to 4273.5)	4178.1 (4086.2 to 4273.1)	0.2 (0.0 to 0.5)	0.2 (0.0 to 0.5)	0.0 (0.0 to 0.0)	3296.6 (3224.3 to 3371.5)	3296.3 (3223.8 to 3371.2)	0.2 (0.0 to 0.4)	0.2 (0.0 to 0.4)	0.0 (0.0 to 0.0)
Greenland	2008	4523.9 (4427.3 to 4625.0)	4523.4 (4427.3 to 4624.9)	0.2 (0.0 to 0.5)	0.2 (0.0 to 0.5)	0.0 (0.0 to 0.0)	3569.1 (3492.9 to 3648.8)	3568.7 (3492.9 to 3648.8)	0.2 (0.0 to 0.4)	0.2 (0.0 to 0.4)	0.0 (0.0 to 0.0)
Greenland	2009	4648.7 (4555.9 to 4743.4)	4648.2 (4555.4 to 4742.9)	0.2 (0.0 to 0.5)	0.2 (0.0 to 0.5)	0.0 (0.0 to 0.0)	3667.6 (3594.3 to 3742.3)	3667.2 (3594.0 to 3741.9)	0.2 (0.0 to 0.4)	0.2 (0.0 to 0.4)	0.0 (0.0 to 0.0)
Greenland	2010	4737.4 (4640.9 to 4832.2)	4736.9 (4640.3 to 4832.2)	0.2 (0.0 to 0.5)	0.2 (0.0 to 0.5)	0.0 (0.0 to 0.0)	3737.5 (3661.4 to 3812.4)	3737.2 (3661.0 to 3812.3)	0.2 (0.0 to 0.4)	0.2 (0.0 to 0.4)	0.0 (0.0 to 0.0)
Greenland	2011	4766.3 (4668.7 to 4868.2)	4765.8 (4667.8 to 4867.7)	0.2 (0.0 to 0.5)	0.2 (0.0 to 0.5)	0.0 (0.0 to 0.0)	3760.4 (3683.3 to 3840.7)	3760.0 (3682.6 to 3840.3)	0.2 (0.0 to 0.4)	0.2 (0.0 to 0.4)	0.0 (0.0 to 0.0)
Greenland	2012	4827.4 (4723.5 to 4931.5)	4826.9 (4722.5 to 4931.3)	0.2 (0.0 to 0.5)	0.2 (0.0 to 0.5)	0.0 (0.0 to 0.0)	3808.5 (3726.6 to 3890.6)	3808.2 (3725.8 to 3890.5)	0.2 (0.0 to 0.4)	0.2 (0.0 to 0.4)	0.0 (0.0 to 0.0)
Greenland	2013	4709.4 (4613.6 to 4808.0)	4708.9 (4613.5 to 4808.0)	0.2 (0.0 to 0.5)	0.2 (0.0 to 0.5)	0.0 (0.0 to 0.0)	3715.5 (3639.9 to 3793.3)	3715.1 (3639.8 to 3793.2)	0.2 (0.0 to 0.4)	0.2 (0.0 to 0.4)	0.0 (0.0 to 0.0)
Greenland	2014	4569.4 (4474.0 to 4670.2)	4568.9 (4474.0 to 4669.9)	0.3 (0.0 to 0.5)	0.3 (0.0 to 0.5)	0.0 (0.0 to 0.0)	3605.0 (3529.7 to 3684.5)	3604.6 (3529.7 to 3684.3)	0.2 (0.0 to 0.4)	0.2 (0.0 to 0.4)	0.0 (0.0 to 0.0)
Greenland	2015	4437.8 (4298.4 to 4579.1)	4437.3 (4297.9 to 4578.1)	0.3 (0.0 to 0.5)	0.3 (0.0 to 0.5)	0.0 (0.0 to 0.0)	3501.2 (3391.2 to 3612.7)	3500.8 (3390.8 to 3611.9)	0.2 (0.0 to 0.4)	0.2 (0.0 to 0.4)	0.0 (0.0 to 0.0)
Greenland	2016	4456.8 (4203.5 to 4731.0)	4456.2 (4202.9 to 4730.4)	0.3 (0.0 to 0.6)	0.3 (0.0 to 0.6)	0.0 (0.0 to 0.0)	3516.1 (3316.3 to 3732.5)	3515.7 (3315.9 to 3732.0)	0.2 (0.0 to 0.4)	0.2 (0.0 to 0.4)	0.0 (0.0 to 0.0)
Greenland	2017	4575.2 (4324.1 to 4867.3)	4574.6 (4323.4 to 4866.1)	0.3 (0.0 to 0.6)	0.3 (0.0 to 0.6)	0.0 (0.0 to 0.0)	3609.6 (3411.5 to 3840.0)	3609.1 (3411.0 to 3839.1)	0.2 (0.0 to 0.5)	0.2 (0.0 to 0.5)	0.0 (0.0 to 0.0)
Greenland	2018	4680.8 (4411.3 to 4982.3)	4680.3 (4410.8 to 4981.7)	0.3 (0.0 to 0.6)	0.3 (0.0 to 0.6)	0.0 (0.0 to 0.0)	3692.9 (3480.3 to 3930.8)	3692.5 (3479.8 to 3930.3)	0.2 (0.0 to 0.5)	0.2 (0.0 to 0.5)	0.0 (0.0 to 0.0)
Greenland	2019	4776.9 (4492.1 to 5100.4)	4776.3 (4491.1 to 5099.2)	0.3 (0.0 to 0.6)	0.3 (0.0 to 0.6)	0.0 (0.0 to 0.0)	3768.7 (3544.0 to 4024.0)	3768.2 (3543.2 to 4023.0)	0.2 (0.0 to 0.5)	0.2 (0.0 to 0.5)	0.0 (0.0 to 0.0)
Greenland	2020	4877.1 (4583.1 to 5214.9)	4876.5 (4582.5 to 5214.3)	0.3 (0.0 to 0.6)	0.3 (0.0 to 0.6)	0.0 (0.0 to 0.0)	3847.8 (3615.8 to 4114.3)	3847.3 (3615.4 to 4113.8)	0.2 (0.0 to 0.5)	0.2 (0.0 to 0.5)	0.0 (0.0 to 0.0)
Greenland	2021	4978.4 (4661.1 to 5329.0)	4977.8 (4659.9 to 5327.7)	0.3 (0.0 to 0.6)	0.3 (0.0 to 0.7)	0.0 (0.0 to 0.0)	3927.7 (3677.4 to 4204.3)	3927.2 (3676.4 to 4203.3)	0.2 (0.0 to 0.5)	0.3 (0.0 to 0.5)	0.0 (0.0 to 0.0)
Greenland	2022	5087.3 (4758.7 to 5444.5)	5086.7 (4758.1 to 5443.8)	0.3 (0.0 to 0.6)	0.3 (0.0 to 0.7)	0.0 (0.0 to 0.0)	4013.6 (3754.4 to 4295.4)	4013.1 (3753.8 to 4294.8)	0.2 (0.0 to 0.5)	0.3 (0.0 to 0.5)	0.0 (0.0 to 0.0)

		2018 US Dollars per capita					2018 PPP per capita				
Country	Year	Health spending	Government health spending	Out-of-pocket health spending	Prepaid private health spending	Development assistance for health	Health spending	Government health spending	Out-of-pocket health spending	Prepaid private health spending	Development assistance for health
Greenland	2023	5196.0 (4864.3 to 5559.4)	5195.4 (4863.0 to 5558.0)	0.3 (0.0 to 0.6)	0.3 (0.0 to 0.7)	0.0 (0.0 to 0.0)	4099.4 (3837.6 to 4386.0)	4098.9 (3836.6 to 4385.0)	0.2 (0.0 to 0.5)	0.3 (0.0 to 0.5)	0.0 (0.0 to 0.0)
Greenland	2024	5244.6 (4907.6 to 5609.1)	5244.0 (4906.9 to 5608.3)	0.3 (0.0 to 0.6)	0.3 (0.0 to 0.7)	0.0 (0.0 to 0.0)	4137.7 (3871.8 to 4425.2)	4137.2 (3871.3 to 4424.7)	0.2 (0.0 to 0.5)	0.3 (0.0 to 0.6)	0.0 (0.0 to 0.0)
Greenland	2025	5288.3 (4951.4 to 5656.4)	5287.7 (4951.3 to 5655.1)	0.3 (0.0 to 0.6)	0.4 (0.0 to 0.7)	0.0 (0.0 to 0.0)	4172.2 (3906.3 to 4462.6)	4171.7 (3906.3 to 4461.5)	0.2 (0.0 to 0.5)	0.3 (0.0 to 0.6)	0.0 (0.0 to 0.0)
Greenland	2026	5325.5 (4983.6 to 5697.0)	5324.9 (4982.9 to 5695.8)	0.3 (0.0 to 0.6)	0.4 (0.0 to 0.7)	0.0 (0.0 to 0.0)	4201.5 (3931.8 to 4494.6)	4201.0 (3931.2 to 4493.7)	0.2 (0.0 to 0.5)	0.3 (0.0 to 0.6)	0.0 (0.0 to 0.0)
Greenland	2027	5368.7 (5021.1 to 5750.4)	5368.0 (5019.9 to 5749.0)	0.3 (0.0 to 0.6)	0.4 (0.0 to 0.7)	0.0 (0.0 to 0.0)	4235.6 (3961.4 to 4536.7)	4235.0 (3960.4 to 4535.6)	0.2 (0.0 to 0.5)	0.3 (0.0 to 0.6)	0.0 (0.0 to 0.0)
Greenland	2028	5412.6 (5058.2 to 5797.6)	5411.9 (5057.5 to 5796.9)	0.3 (0.0 to 0.6)	0.4 (0.0 to 0.8)	0.0 (0.0 to 0.0)	4270.3 (3990.6 to 4574.0)	4269.7 (3990.1 to 4573.4)	0.2 (0.0 to 0.5)	0.3 (0.0 to 0.6)	0.0 (0.0 to 0.0)
Greenland	2029	5448.5 (5086.2 to 5844.6)	5447.8 (5084.9 to 5843.2)	0.3 (0.0 to 0.6)	0.4 (0.0 to 0.8)	0.0 (0.0 to 0.0)	4298.5 (4012.7 to 4611.0)	4298.0 (4011.7 to 4609.9)	0.2 (0.0 to 0.5)	0.3 (0.0 to 0.6)	0.0 (0.0 to 0.0)
Greenland	2030	5485.3 (5112.6 to 5885.4)	5484.7 (5111.9 to 5885.0)	0.3 (0.0 to 0.6)	0.4 (0.0 to 0.8)	0.0 (0.0 to 0.0)	4327.6 (4033.6 to 4643.3)	4327.1 (4033.0 to 4642.9)	0.2 (0.0 to 0.5)	0.3 (0.0 to 0.6)	0.0 (0.0 to 0.0)
Greenland	2031	5527.2 (5140.6 to 5937.9)	5526.5 (5139.3 to 5936.6)	0.3 (0.0 to 0.6)	0.4 (0.0 to 0.8)	0.0 (0.0 to 0.0)	4360.6 (4055.7 to 4684.6)	4360.1 (4054.6 to 4683.6)	0.2 (0.0 to 0.5)	0.3 (0.0 to 0.6)	0.0 (0.0 to 0.0)
Greenland	2032	5573.7 (5178.5 to 5996.4)	5573.0 (5177.2 to 5996.3)	0.3 (0.0 to 0.6)	0.4 (0.0 to 0.8)	0.0 (0.0 to 0.0)	4397.4 (4085.5 to 4730.8)	4396.8 (4084.5 to 4730.7)	0.2 (0.0 to 0.5)	0.3 (0.0 to 0.6)	0.0 (0.0 to 0.0)
Greenland	2033	5629.3 (5220.8 to 6070.5)	5628.6 (5220.0 to 6069.0)	0.3 (0.0 to 0.7)	0.4 (0.1 to 0.8)	0.0 (0.0 to 0.0)	4441.2 (4118.9 to 4789.3)	4440.7 (4118.3 to 4788.1)	0.2 (0.0 to 0.5)	0.3 (0.0 to 0.7)	0.0 (0.0 to 0.0)
Greenland	2034	5702.0 (5279.9 to 6153.2)	5701.2 (5278.6 to 6152.3)	0.3 (0.0 to 0.7)	0.4 (0.1 to 0.9)	0.0 (0.0 to 0.0)	4498.5 (4165.6 to 4854.5)	4497.9 (4164.5 to 4853.8)	0.2 (0.0 to 0.5)	0.3 (0.0 to 0.7)	0.0 (0.0 to 0.0)
Greenland	2035	5797.1 (5361.6 to 6260.0)	5796.3 (5360.8 to 6258.4)	0.3 (0.0 to 0.7)	0.4 (0.1 to 0.9)	0.0 (0.0 to 0.0)	4573.6 (4230.0 to 4938.8)	4573.0 (4229.4 to 4937.6)	0.2 (0.0 to 0.5)	0.3 (0.0 to 0.7)	0.0 (0.0 to 0.0)
Greenland	2036	5914.3 (5463.1 to 6397.6)	5913.5 (5461.7 to 6396.6)	0.3 (0.0 to 0.7)	0.5 (0.1 to 0.9)	0.0 (0.0 to 0.0)	4666.1 (4310.1 to 5047.3)	4665.5 (4309.0 to 5046.6)	0.3 (0.0 to 0.5)	0.4 (0.0 to 0.7)	0.0 (0.0 to 0.0)
Greenland	2037	6042.9 (5572.2 to 6540.8)	6042.1 (5570.8 to 6539.7)	0.3 (0.0 to 0.7)	0.5 (0.1 to 0.9)	0.0 (0.0 to 0.0)	4767.5 (4396.2 to 5160.3)	4766.9 (4395.0 to 5159.5)	0.3 (0.0 to 0.5)	0.4 (0.1 to 0.7)	0.0 (0.0 to 0.0)
Greenland	2038	6182.5 (5690.6 to 6695.9)	6181.7 (5689.7 to 6694.9)	0.3 (0.0 to 0.7)	0.5 (0.1 to 1.0)	0.0 (0.0 to 0.0)	4877.7 (4489.6 to 5282.7)	4877.0 (4488.9 to 5281.9)	0.3 (0.0 to 0.6)	0.4 (0.1 to 0.8)	0.0 (0.0 to 0.0)
Greenland	2039	6337.3 (5825.5 to 6871.2)	6336.5 (5824.8 to 6869.4)	0.3 (0.0 to 0.7)	0.5 (0.1 to 1.0)	0.0 (0.0 to 0.0)	4999.8 (4596.0 to 5421.0)	4999.1 (4595.4 to 5419.6)	0.3 (0.0 to 0.6)	0.4 (0.1 to 0.8)	0.0 (0.0 to 0.0)
Greenland	2040	6487.2 (5949.8 to 7043.7)	6486.3 (5948.9 to 7043.5)	0.3 (0.0 to 0.7)	0.5 (0.1 to 1.0)	0.0 (0.0 to 0.0)	5118.0 (4694.1 to 5557.1)	5117.3 (4693.4 to 5556.9)	0.3 (0.0 to 0.6)	0.4 (0.1 to 0.8)	0.0 (0.0 to 0.0)
Greenland	2041	6629.4 (6068.0 to 7211.0)	6628.5 (6066.5 to 7209.1)	0.3 (0.0 to 0.7)	0.5 (0.1 to 1.1)	0.0 (0.0 to 0.0)	5230.2 (4787.3 to 5689.1)	5229.5 (4786.1 to 5687.6)	0.3 (0.0 to 0.6)	0.4 (0.1 to 0.9)	0.0 (0.0 to 0.0)
Greenland	2042	6778.7 (6196.2 to 7387.7)	6777.8 (6195.3 to 7387.5)	0.4 (0.0 to 0.8)	0.6 (0.1 to 1.1)	0.0 (0.0 to 0.0)	5348.0 (4888.5 to 5828.5)	5347.3 (4887.7 to 5828.3)	0.3 (0.0 to 0.6)	0.4 (0.1 to 0.9)	0.0 (0.0 to 0.0)
Greenland	2043	6936.6 (6329.7 to 7588.6)	6935.7 (6328.9 to 7587.6)	0.4 (0.0 to 0.8)	0.6 (0.1 to 1.2)	0.0 (0.0 to 0.0)	5472.6 (4993.8 to 5986.9)	5471.9 (4993.1 to 5986.2)	0.3 (0.0 to 0.6)	0.5 (0.1 to 0.9)	0.0 (0.0 to 0.0)

Country	Year	2018 US Dollars per capita					2018 PPP per capita				
		Health spending	Government health spending	Out-of-pocket health spending	Prepaid private health spending	Development assistance for health	Health spending	Government health spending	Out-of-pocket health spending	Prepaid private health spending	Development assistance for health
Greenland	2044	7104.2 (6477.4 to 7782.1)	7103.2 (6475.7 to 7781.1)	0.4 (0.0 to 0.8)	0.6 (0.1 to 1.2)	0.0 (0.0 to 0.0)	5604.8 (5110.3 to 6139.7)	5604.1 (5108.9 to 6138.9)	0.3 (0.0 to 0.6)	0.5 (0.1 to 0.9)	0.0 (0.0 to 0.0)
Greenland	2045	7262.3 (6606.8 to 7978.5)	7261.3 (6605.7 to 7976.4)	0.4 (0.0 to 0.8)	0.6 (0.1 to 1.2)	0.0 (0.0 to 0.0)	5729.5 (5212.4 to 6294.6)	5728.7 (5211.6 to 6292.9)	0.3 (0.0 to 0.6)	0.5 (0.1 to 1.0)	0.0 (0.0 to 0.0)
Greenland	2046	7417.4 (6733.1 to 8170.9)	7416.4 (6731.3 to 8170.6)	0.4 (0.0 to 0.8)	0.6 (0.1 to 1.3)	0.0 (0.0 to 0.0)	5851.9 (5312.0 to 6446.4)	5851.1 (5310.6 to 6446.2)	0.3 (0.0 to 0.6)	0.5 (0.1 to 1.0)	0.0 (0.0 to 0.0)
Greenland	2047	7571.2 (6854.9 to 8364.8)	7570.2 (6853.8 to 8363.4)	0.4 (0.0 to 0.8)	0.7 (0.1 to 1.3)	0.0 (0.0 to 0.0)	5973.3 (5408.2 to 6599.3)	5972.4 (5407.3 to 6598.3)	0.3 (0.0 to 0.7)	0.5 (0.1 to 1.0)	0.0 (0.0 to 0.0)
Greenland	2048	7734.9 (6991.1 to 8550.9)	7733.9 (6990.9 to 8548.9)	0.4 (0.0 to 0.8)	0.7 (0.1 to 1.4)	0.0 (0.0 to 0.0)	6102.4 (5515.6 to 6746.2)	6101.6 (5515.4 to 6744.6)	0.3 (0.0 to 0.7)	0.5 (0.1 to 1.1)	0.0 (0.0 to 0.0)
Greenland	2049	7894.6 (7121.3 to 8740.3)	7893.5 (7120.1 to 8738.9)	0.4 (0.0 to 0.9)	0.7 (0.1 to 1.4)	0.0 (0.0 to 0.0)	6228.4 (5618.3 to 6895.6)	6227.5 (5617.3 to 6894.5)	0.3 (0.0 to 0.7)	0.5 (0.1 to 1.1)	0.0 (0.0 to 0.0)
Greenland	2050	8046.6 (7244.4 to 8937.9)	8045.5 (7244.2 to 8935.6)	0.4 (0.0 to 0.9)	0.7 (0.1 to 1.5)	0.0 (0.0 to 0.0)	6348.3 (5715.4 to 7051.5)	6347.4 (5715.3 to 7049.7)	0.3 (0.0 to 0.7)	0.6 (0.1 to 1.1)	0.0 (0.0 to 0.0)
Grenada	1995	431.7 (392.4 to 474.0)	225.0 (196.3 to 257.9)	206.7 (178.9 to 239.4)	0.0 (0.0 to 0.1)	0.0 (0.0 to 0.0)	642.7 (584.2 to 705.8)	334.9 (292.3 to 384.0)	307.7 (266.3 to 356.4)	0.0 (0.0 to 0.1)	0.0 (0.0 to 0.0)
Grenada	1996	443.0 (408.9 to 480.7)	224.5 (198.9 to 253.8)	218.1 (193.1 to 246.8)	0.0 (0.0 to 0.1)	0.4 (0.4 to 0.4)	659.6 (608.7 to 715.7)	334.3 (296.1 to 377.8)	324.8 (287.6 to 367.5)	0.0 (0.0 to 0.1)	0.5 (0.5 to 0.5)
Grenada	1997	448.1 (417.8 to 482.6)	213.1 (190.4 to 238.4)	230.5 (206.0 to 257.6)	0.0 (0.0 to 0.1)	4.5 (4.5 to 4.5)	667.2 (622.0 to 718.6)	317.3 (283.4 to 354.9)	343.2 (306.7 to 383.5)	0.0 (0.0 to 0.1)	6.7 (6.7 to 6.7)
Grenada	1998	454.9 (423.6 to 491.6)	204.2 (182.9 to 227.9)	247.1 (222.2 to 272.6)	0.0 (0.0 to 0.1)	3.6 (3.6 to 3.6)	677.3 (630.7 to 731.9)	304.1 (272.4 to 339.4)	367.9 (330.8 to 405.9)	0.0 (0.0 to 0.1)	5.3 (5.3 to 5.3)
Grenada	1999	432.0 (400.1 to 466.0)	183.9 (163.4 to 206.0)	247.9 (224.8 to 273.6)	0.0 (0.0 to 0.1)	0.2 (0.2 to 0.2)	643.3 (595.7 to 693.9)	273.8 (243.3 to 306.6)	369.1 (334.6 to 407.3)	0.0 (0.0 to 0.1)	0.3 (0.3 to 0.3)
Grenada	2000	394.7 (366.1 to 424.8)	161.8 (144.4 to 181.1)	232.8 (211.3 to 257.3)	0.0 (0.0 to 0.1)	0.2 (0.2 to 0.2)	587.7 (545.1 to 632.4)	240.8 (215.0 to 269.6)	346.6 (314.6 to 383.1)	0.1 (0.0 to 0.1)	0.2 (0.2 to 0.2)
Grenada	2001	375.9 (348.9 to 404.0)	155.9 (139.6 to 174.4)	219.7 (198.6 to 242.6)	0.0 (0.0 to 0.1)	0.3 (0.3 to 0.3)	559.7 (519.5 to 601.5)	232.1 (207.8 to 259.6)	327.1 (295.7 to 361.2)	0.1 (0.0 to 0.1)	0.4 (0.4 to 0.4)
Grenada	2002	382.6 (355.3 to 410.2)	166.2 (148.7 to 184.9)	216.0 (194.5 to 238.6)	0.0 (0.0 to 0.1)	0.3 (0.3 to 0.3)	569.7 (529.1 to 610.8)	247.5 (221.3 to 275.2)	321.6 (289.6 to 355.2)	0.1 (0.0 to 0.1)	0.5 (0.5 to 0.5)
Grenada	2003	398.4 (369.4 to 427.3)	181.4 (161.8 to 202.2)	213.1 (191.5 to 235.5)	0.0 (0.0 to 0.1)	3.8 (3.8 to 3.8)	593.1 (550.0 to 636.3)	270.0 (240.9 to 301.1)	317.3 (285.1 to 350.7)	0.1 (0.0 to 0.1)	5.7 (5.7 to 5.7)
Grenada	2004	399.4 (369.5 to 430.4)	189.9 (169.2 to 211.9)	204.2 (183.2 to 226.4)	0.0 (0.0 to 0.1)	5.2 (5.2 to 5.2)	594.6 (550.1 to 640.8)	282.8 (251.9 to 315.4)	304.0 (272.8 to 337.0)	0.1 (0.0 to 0.1)	7.8 (7.8 to 7.8)
Grenada	2005	441.9 (408.9 to 476.0)	220.0 (197.3 to 244.4)	217.7 (196.7 to 241.7)	0.0 (0.0 to 0.1)	4.1 (4.1 to 4.1)	657.9 (608.8 to 708.8)	327.5 (293.7 to 363.9)	324.2 (292.9 to 359.9)	0.1 (0.0 to 0.1)	6.1 (6.1 to 6.1)
Grenada	2006	450.8 (417.4 to 484.3)	220.3 (196.9 to 244.1)	222.1 (201.5 to 243.7)	0.0 (0.0 to 0.1)	8.3 (8.3 to 8.3)	671.2 (621.5 to 721.1)	328.0 (293.1 to 363.5)	330.8 (300.0 to 362.9)	0.1 (0.0 to 0.1)	12.4 (12.4 to 12.4)
Grenada	2007	476.5 (442.1 to 512.5)	228.1 (204.5 to 253.2)	239.0 (216.6 to 264.1)	0.0 (0.0 to 0.1)	9.3 (9.3 to 9.3)	709.4 (658.3 to 763.1)	339.7 (304.4 to 376.9)	355.8 (322.6 to 393.3)	0.1 (0.0 to 0.1)	13.8 (13.8 to 13.8)
Grenada	2008	487.3 (453.4 to 522.8)	227.4 (204.5 to 251.8)	250.0 (228.1 to 276.3)	0.0 (0.0 to 0.1)	9.8 (9.8 to 9.8)	725.5 (675.0 to 778.3)	338.5 (304.5 to 374.9)	372.2 (339.6 to 411.3)	0.1 (0.0 to 0.1)	14.6 (14.6 to 14.6)

		2018 US Dollars per capita					2018 PPP per capita				
Country	Year	Health spending	Government health spending	Out-of-pocket health spending	Prepaid private health spending	Development assistance for health	Health spending	Government health spending	Out-of-pocket health spending	Prepaid private health spending	Development assistance for health
Grenada	2009	479.0 (447.0 to 514.5)	218.8 (197.0 to 243.3)	250.3 (228.6 to 276.2)	0.0 (0.0 to 0.1)	9.9 (9.9 to 9.9)	713.2 (665.5 to 766.1)	325.7 (293.3 to 362.2)	372.7 (340.4 to 411.3)	0.1 (0.0 to 0.1)	14.8 (14.8 to 14.8)
Grenada	2010	474.9 (442.0 to 510.4)	215.1 (194.0 to 239.6)	254.6 (231.2 to 279.8)	0.0 (0.0 to 0.1)	5.1 (5.1 to 5.1)	707.1 (658.1 to 760.0)	320.2 (288.9 to 356.8)	379.1 (344.3 to 416.7)	0.1 (0.0 to 0.1)	7.6 (7.6 to 7.6)
Grenada	2011	472.7 (439.3 to 509.2)	208.6 (186.3 to 232.3)	256.9 (233.6 to 283.1)	0.0 (0.0 to 0.1)	7.2 (7.2 to 7.2)	703.9 (654.1 to 758.2)	310.5 (277.4 to 345.9)	382.5 (347.9 to 421.5)	0.1 (0.0 to 0.1)	10.7 (10.7 to 10.7)
Grenada	2012	457.8 (425.1 to 490.8)	193.8 (172.9 to 216.4)	255.0 (231.0 to 281.4)	0.0 (0.0 to 0.1)	8.9 (8.9 to 8.9)	681.6 (632.9 to 730.8)	288.6 (257.5 to 322.2)	379.6 (344.0 to 419.0)	0.1 (0.0 to 0.1)	13.3 (13.3 to 13.3)
Grenada	2013	452.0 (418.7 to 484.7)	186.1 (165.0 to 207.8)	257.8 (232.2 to 284.1)	0.0 (0.0 to 0.1)	8.1 (8.1 to 8.1)	673.0 (623.4 to 721.7)	277.1 (245.6 to 309.4)	383.8 (345.8 to 423.0)	0.1 (0.0 to 0.1)	12.0 (12.0 to 12.0)
Grenada	2014	451.8 (415.8 to 488.4)	185.4 (163.9 to 207.8)	263.5 (237.7 to 293.0)	0.0 (0.0 to 0.1)	2.8 (2.8 to 2.8)	672.6 (619.1 to 727.2)	276.0 (244.0 to 309.5)	392.3 (353.8 to 436.2)	0.1 (0.0 to 0.1)	4.2 (4.2 to 4.2)
Grenada	2015	466.7 (427.8 to 506.1)	188.9 (165.6 to 214.5)	270.8 (243.8 to 303.1)	0.0 (0.0 to 0.1)	7.0 (7.0 to 7.0)	694.9 (636.9 to 753.6)	281.3 (246.6 to 319.4)	403.1 (363.0 to 451.3)	0.1 (0.0 to 0.1)	10.4 (10.4 to 10.4)
Grenada	2016	485.8 (437.8 to 535.5)	198.8 (170.6 to 229.6)	284.6 (249.9 to 323.1)	0.0 (0.0 to 0.1)	2.4 (2.4 to 2.4)	723.3 (651.8 to 797.4)	296.0 (254.0 to 341.9)	423.7 (372.1 to 481.0)	0.1 (0.0 to 0.1)	3.5 (3.5 to 3.5)
Grenada	2017	507.9 (459.6 to 559.0)	194.9 (167.2 to 225.7)	294.2 (258.2 to 335.0)	0.0 (0.0 to 0.1)	18.7 (18.7 to 18.7)	756.2 (684.3 to 832.4)	290.2 (248.9 to 336.0)	438.1 (384.4 to 498.8)	0.1 (0.0 to 0.1)	27.8 (27.8 to 27.8)
Grenada	2018	517.8 (470.9 to 569.3)	199.8 (171.4 to 231.6)	300.2 (263.8 to 343.0)	0.0 (0.0 to 0.1)	17.7 (17.7 to 17.7)	771.0 (701.1 to 847.6)	297.6 (255.2 to 344.8)	447.0 (392.8 to 510.7)	0.1 (0.0 to 0.2)	26.3 (26.3 to 26.4)
Grenada	2019	527.5 (479.9 to 580.0)	203.8 (174.7 to 236.1)	305.8 (267.9 to 348.0)	0.0 (0.0 to 0.1)	17.9 (16.7 to 19.0)	785.5 (714.5 to 863.5)	303.4 (260.1 to 351.6)	455.3 (398.8 to 518.2)	0.1 (0.0 to 0.2)	26.7 (24.9 to 28.2)
Grenada	2020	537.1 (487.6 to 590.5)	207.6 (177.9 to 240.4)	311.2 (271.9 to 354.1)	0.1 (0.0 to 0.1)	18.3 (16.8 to 19.8)	799.8 (725.9 to 879.2)	309.1 (264.9 to 357.9)	463.3 (404.8 to 527.2)	0.1 (0.0 to 0.2)	27.3 (25.0 to 29.5)
Grenada	2021	547.1 (496.6 to 602.3)	211.7 (181.4 to 245.4)	316.6 (276.6 to 361.2)	0.1 (0.0 to 0.1)	18.8 (16.9 to 20.7)	814.6 (739.4 to 896.7)	315.2 (270.1 to 365.3)	471.4 (411.8 to 537.7)	0.1 (0.0 to 0.2)	27.9 (25.1 to 30.9)
Grenada	2022	556.9 (505.0 to 611.6)	215.7 (185.4 to 250.4)	321.9 (280.2 to 366.8)	0.1 (0.0 to 0.1)	19.3 (17.1 to 21.7)	829.2 (751.9 to 910.6)	321.1 (276.0 to 372.9)	479.3 (417.1 to 546.2)	0.1 (0.0 to 0.2)	28.7 (25.4 to 32.2)
Grenada	2023	567.3 (514.2 to 621.9)	220.4 (189.5 to 256.8)	327.0 (284.4 to 374.8)	0.1 (0.0 to 0.1)	19.9 (17.2 to 22.7)	844.7 (765.6 to 926.0)	328.1 (282.1 to 382.3)	486.9 (423.5 to 558.1)	0.1 (0.0 to 0.2)	29.6 (25.6 to 33.7)
Grenada	2024	577.3 (522.6 to 635.1)	225.0 (192.5 to 262.0)	331.8 (288.3 to 381.2)	0.1 (0.0 to 0.1)	20.5 (17.6 to 23.7)	859.6 (778.1 to 945.6)	335.0 (286.7 to 390.1)	494.0 (429.3 to 567.5)	0.1 (0.0 to 0.2)	30.5 (26.3 to 35.3)
Grenada	2025	586.9 (527.5 to 644.9)	229.3 (196.8 to 266.1)	336.3 (290.9 to 385.8)	0.1 (0.0 to 0.1)	21.2 (18.0 to 24.8)	873.8 (785.3 to 960.2)	341.4 (293.0 to 396.2)	500.8 (433.1 to 574.4)	0.1 (0.0 to 0.2)	31.5 (26.8 to 37.0)
Grenada	2026	597.4 (538.2 to 658.5)	234.3 (200.9 to 271.4)	341.1 (293.5 to 390.4)	0.1 (0.0 to 0.1)	21.8 (18.4 to 25.6)	889.4 (801.4 to 980.4)	348.9 (299.1 to 404.1)	507.9 (437.0 to 581.3)	0.1 (0.0 to 0.2)	32.5 (27.4 to 38.2)
Grenada	2027	608.0 (545.5 to 673.2)	239.5 (204.0 to 278.8)	345.9 (295.4 to 398.0)	0.1 (0.0 to 0.1)	22.5 (18.8 to 26.8)	905.2 (812.3 to 1002.3)	356.6 (303.7 to 415.1)	515.0 (439.8 to 592.6)	0.1 (0.0 to 0.2)	33.6 (28.0 to 39.9)
Grenada	2028	619.3 (551.2 to 686.0)	245.2 (208.8 to 285.1)	350.8 (297.1 to 406.3)	0.1 (0.0 to 0.1)	23.3 (19.5 to 28.2)	922.1 (820.7 to 1021.5)	365.1 (310.8 to 424.4)	522.3 (442.4 to 604.9)	0.1 (0.0 to 0.2)	34.7 (29.0 to 42.0)
Grenada	2029	630.3 (560.9 to 702.1)	250.4 (212.7 to 291.4)	355.8 (298.1 to 413.3)	0.1 (0.0 to 0.1)	24.1 (19.8 to 29.5)	938.5 (835.1 to 1045.4)	372.8 (316.6 to 433.8)	529.7 (443.9 to 615.4)	0.1 (0.0 to 0.2)	35.9 (29.5 to 43.9)

		2018 US Dollars per capita					2018 PPP per capita				
**Country**	**Year**	**Health spending**	**Government health spending**	**Out-of-pocket health spending**	**Prepaid private health spending**	**Development assistance for health**	**Health spending**	**Government health spending**	**Out-of-pocket health spending**	**Prepaid private health spending**	**Development assistance for health**
Grenada	2030	641.4 (568.0 to 715.2)	255.3 (217.4 to 298.3)	361.2 (300.9 to 421.9)	0.1 (0.0 to 0.1)	24.9 (20.3 to 30.6)	955.0 (845.8 to 1064.9)	380.1 (323.8 to 444.2)	537.7 (448.0 to 628.1)	0.1 (0.0 to 0.2)	37.1 (30.3 to 45.5)
Grenada	2031	653.1 (575.4 to 730.9)	260.3 (221.5 to 304.5)	367.1 (302.6 to 430.7)	0.1 (0.0 to 0.1)	25.7 (20.8 to 32.3)	972.4 (856.7 to 1088.2)	387.5 (329.7 to 453.4)	546.5 (450.6 to 641.2)	0.1 (0.0 to 0.2)	38.2 (31.0 to 48.0)
Grenada	2032	664.7 (582.2 to 742.5)	265.0 (225.1 to 309.8)	373.2 (304.8 to 439.5)	0.1 (0.0 to 0.2)	26.5 (21.3 to 33.5)	989.8 (866.9 to 1105.6)	394.5 (335.2 to 461.2)	555.7 (453.8 to 654.3)	0.1 (0.0 to 0.2)	39.5 (31.8 to 49.9)
Grenada	2033	677.1 (589.5 to 761.1)	269.9 (229.2 to 315.4)	379.7 (307.6 to 448.2)	0.1 (0.0 to 0.2)	27.4 (21.7 to 35.2)	1008.1 (877.7 to 1133.2)	401.9 (341.3 to 469.6)	565.4 (458.0 to 667.3)	0.1 (0.0 to 0.2)	40.7 (32.3 to 52.3)
Grenada	2034	689.3 (597.1 to 775.9)	274.7 (232.2 to 320.2)	386.2 (309.5 to 457.6)	0.1 (0.0 to 0.2)	28.3 (22.3 to 36.2)	1026.3 (889.1 to 1155.3)	409.0 (345.7 to 476.7)	575.1 (460.9 to 681.3)	0.1 (0.0 to 0.2)	42.1 (33.2 to 53.9)
Grenada	2035	701.8 (606.2 to 794.0)	279.7 (237.0 to 327.4)	392.8 (312.8 to 467.8)	0.1 (0.0 to 0.2)	29.2 (22.8 to 38.2)	1044.9 (902.6 to 1182.2)	416.4 (352.8 to 487.5)	584.9 (465.7 to 696.5)	0.1 (0.0 to 0.2)	43.5 (33.9 to 56.9)
Grenada	2036	714.9 (616.6 to 811.6)	285.1 (241.0 to 334.2)	399.5 (316.5 to 478.4)	0.1 (0.0 to 0.2)	30.2 (23.4 to 40.5)	1064.4 (918.0 to 1208.4)	424.4 (358.8 to 497.5)	594.8 (471.2 to 712.3)	0.1 (0.0 to 0.3)	45.0 (34.8 to 60.2)
Grenada	2037	728.0 (626.7 to 830.1)	290.5 (245.4 to 340.7)	406.2 (319.7 to 488.8)	0.1 (0.0 to 0.2)	31.2 (24.0 to 41.9)	1083.9 (933.0 to 1235.9)	432.5 (365.4 to 507.2)	604.8 (476.0 to 727.7)	0.1 (0.0 to 0.3)	46.4 (35.7 to 62.4)
Grenada	2038	741.8 (640.4 to 845.9)	296.3 (250.0 to 347.0)	413.1 (323.3 to 499.2)	0.1 (0.0 to 0.2)	32.3 (24.5 to 44.5)	1104.4 (953.5 to 1259.5)	441.2 (372.3 to 516.6)	615.0 (481.3 to 743.2)	0.1 (0.0 to 0.3)	48.0 (36.5 to 66.2)
Grenada	2039	755.5 (644.6 to 858.3)	302.1 (254.0 to 355.3)	419.9 (326.4 to 509.4)	0.1 (0.0 to 0.2)	33.3 (25.4 to 45.8)	1124.9 (959.8 to 1278.0)	449.9 (378.1 to 529.0)	625.2 (486.0 to 758.4)	0.1 (0.0 to 0.3)	49.6 (37.8 to 68.3)
Grenada	2040	769.5 (656.7 to 883.8)	308.2 (258.0 to 362.6)	426.8 (330.0 to 520.7)	0.1 (0.0 to 0.2)	34.4 (26.2 to 47.5)	1145.7 (977.8 to 1315.8)	458.9 (384.2 to 540.0)	635.5 (491.4 to 775.3)	0.1 (0.0 to 0.3)	51.2 (39.0 to 70.7)
Grenada	2041	783.6 (664.9 to 903.3)	314.2 (262.4 to 369.5)	433.7 (334.2 to 532.2)	0.1 (0.0 to 0.2)	35.5 (26.7 to 49.6)	1166.7 (990.0 to 1344.9)	467.9 (390.7 to 550.1)	645.8 (497.5 to 792.4)	0.1 (0.0 to 0.3)	52.9 (39.7 to 73.8)
Grenada	2042	798.2 (675.9 to 917.5)	320.5 (267.0 to 378.8)	440.8 (338.4 to 542.7)	0.1 (0.0 to 0.2)	36.8 (27.4 to 53.0)	1188.5 (1006.4 to 1366.0)	477.2 (397.5 to 564.0)	656.3 (503.9 to 808.1)	0.1 (0.0 to 0.3)	54.8 (40.8 to 78.9)
Grenada	2043	812.3 (686.7 to 935.9)	326.6 (271.2 to 386.6)	447.6 (342.4 to 552.5)	0.1 (0.0 to 0.2)	38.0 (28.0 to 55.8)	1209.5 (1022.4 to 1393.5)	486.3 (403.9 to 575.6)	666.4 (509.9 to 822.7)	0.2 (0.0 to 0.3)	56.6 (41.6 to 83.1)
Grenada	2044	826.1 (697.8 to 959.0)	332.9 (275.2 to 394.9)	454.1 (346.5 to 562.0)	0.1 (0.0 to 0.2)	39.0 (27.7 to 57.8)	1230.0 (1038.9 to 1427.9)	495.6 (409.7 to 587.9)	676.1 (515.9 to 836.8)	0.2 (0.0 to 0.3)	58.1 (41.3 to 86.0)
Grenada	2045	840.8 (710.4 to 981.3)	339.8 (280.1 to 403.4)	460.6 (351.4 to 573.0)	0.1 (0.0 to 0.2)	40.3 (0.0 to 62.2)	1251.8 (1057.7 to 1461.0)	505.9 (417.1 to 600.6)	685.8 (523.3 to 853.2)	0.2 (0.0 to 0.3)	59.9 (0.0 to 92.6)
Grenada	2046	856.2 (725.9 to 994.1)	347.5 (286.0 to 413.6)	467.1 (355.5 to 581.6)	0.1 (0.0 to 0.2)	41.4 (0.0 to 65.5)	1274.8 (1080.8 to 1480.1)	517.4 (425.8 to 615.9)	695.5 (529.4 to 866.0)	0.2 (0.0 to 0.3)	61.7 (0.0 to 97.5)
Grenada	2047	871.8 (742.7 to 1015.1)	355.6 (291.2 to 424.0)	473.4 (359.7 to 590.3)	0.1 (0.0 to 0.2)	42.6 (0.0 to 69.0)	1298.0 (1105.8 to 1511.4)	529.5 (433.5 to 631.3)	704.9 (535.6 to 879.0)	0.2 (0.0 to 0.4)	63.5 (0.0 to 102.8)
Grenada	2048	887.8 (753.0 to 1035.8)	364.3 (298.2 to 435.4)	479.6 (363.6 to 599.0)	0.1 (0.0 to 0.2)	43.8 (0.0 to 73.7)	1321.9 (1121.1 to 1542.2)	542.4 (443.9 to 648.2)	714.0 (541.4 to 891.9)	0.2 (0.0 to 0.4)	65.2 (0.0 to 109.7)
Grenada	2049	903.8 (766.3 to 1059.0)	373.3 (304.9 to 447.6)	485.5 (367.3 to 607.6)	0.1 (0.0 to 0.2)	44.9 (0.0 to 77.8)	1345.7 (1141.0 to 1576.7)	555.8 (454.0 to 666.5)	722.9 (546.9 to 904.6)	0.2 (0.0 to 0.4)	66.9 (0.0 to 115.8)
Grenada	2050	920.6 (778.0 to 1076.4)	382.7 (312.3 to 461.1)	491.1 (370.4 to 614.6)	0.1 (0.0 to 0.3)	46.7 (0.0 to 82.4)	1370.7 (1158.4 to 1602.6)	569.8 (465.0 to 686.6)	731.2 (551.5 to 915.2)	0.2 (0.0 to 0.4)	69.5 (0.0 to 122.7)

Country	Year	2018 US Dollars per capita					2018 PPP per capita				
		Health spending	Government health spending	Out-of-pocket health spending	Prepaid private health spending	Development assistance for health	Health spending	Government health spending	Out-of-pocket health spending	Prepaid private health spending	Development assistance for health
Guam	1995	1312.7 (1016.6 to 1659.0)	1189.2 (896.8 to 1534.1)	108.4 (76.6 to 150.1)	15.1 (7.1 to 27.7)	0.0 (0.0 to 0.0)	1312.7 (1016.6 to 1659.0)	1189.2 (896.8 to 1534.1)	108.4 (76.6 to 150.1)	15.1 (7.1 to 27.7)	0.0 (0.0 to 0.0)
Guam	1996	1325.3 (1031.3 to 1667.5)	1201.2 (914.7 to 1548.2)	108.9 (75.9 to 151.8)	15.2 (7.2 to 28.3)	0.0 (0.0 to 0.0)	1325.3 (1031.3 to 1667.5)	1201.2 (914.7 to 1548.2)	108.9 (75.9 to 151.8)	15.2 (7.2 to 28.3)	0.0 (0.0 to 0.0)
Guam	1997	1340.0 (1044.4 to 1694.0)	1215.6 (923.6 to 1559.0)	109.6 (75.7 to 153.8)	14.8 (6.9 to 28.0)	0.0 (0.0 to 0.0)	1340.0 (1044.4 to 1694.0)	1215.6 (923.6 to 1559.0)	109.6 (75.7 to 153.8)	14.8 (6.9 to 28.0)	0.0 (0.0 to 0.0)
Guam	1998	1344.9 (1046.8 to 1697.1)	1221.1 (924.2 to 1563.0)	110.1 (76.0 to 154.1)	13.8 (6.5 to 27.0)	0.0 (0.0 to 0.0)	1344.9 (1046.8 to 1697.1)	1221.1 (924.2 to 1563.0)	110.1 (76.0 to 154.1)	13.8 (6.5 to 27.0)	0.0 (0.0 to 0.0)
Guam	1999	1351.2 (1051.6 to 1713.4)	1228.2 (932.0 to 1571.5)	110.6 (76.4 to 155.2)	12.3 (5.8 to 23.8)	0.0 (0.0 to 0.0)	1351.2 (1051.6 to 1713.4)	1228.2 (932.0 to 1571.5)	110.6 (76.4 to 155.2)	12.3 (5.8 to 23.8)	0.0 (0.0 to 0.0)
Guam	2000	1371.9 (1065.7 to 1727.0)	1249.3 (942.3 to 1586.1)	111.9 (76.5 to 158.0)	10.7 (5.1 to 20.6)	0.0 (0.0 to 0.0)	1371.9 (1065.7 to 1727.0)	1249.3 (942.3 to 1586.1)	111.9 (76.5 to 158.0)	10.7 (5.1 to 20.6)	0.0 (0.0 to 0.0)
Guam	2001	1403.6 (1090.1 to 1778.5)	1280.4 (963.6 to 1661.8)	114.4 (79.2 to 160.5)	8.9 (4.2 to 17.7)	0.0 (0.0 to 0.0)	1403.6 (1090.1 to 1778.5)	1280.4 (963.6 to 1661.8)	114.4 (79.2 to 160.5)	8.9 (4.2 to 17.7)	0.0 (0.0 to 0.0)
Guam	2002	1443.7 (1124.4 to 1825.8)	1316.2 (995.0 to 1679.4)	118.6 (82.0 to 166.9)	9.0 (4.2 to 17.2)	0.0 (0.0 to 0.0)	1443.7 (1124.4 to 1825.8)	1316.2 (995.0 to 1679.4)	118.6 (82.0 to 166.9)	9.0 (4.2 to 17.2)	0.0 (0.0 to 0.0)
Guam	2003	1478.6 (1158.7 to 1859.0)	1346.3 (1017.5 to 1724.7)	123.0 (85.7 to 172.6)	9.3 (4.4 to 17.7)	0.0 (0.0 to 0.0)	1478.6 (1158.7 to 1859.0)	1346.3 (1017.5 to 1724.7)	123.0 (85.7 to 172.6)	9.3 (4.4 to 17.7)	0.0 (0.0 to 0.0)
Guam	2004	1579.5 (1229.7 to 1985.4)	1438.2 (1097.8 to 1843.2)	130.7 (91.5 to 181.6)	10.6 (5.0 to 20.2)	0.0 (0.0 to 0.0)	1579.5 (1229.7 to 1985.4)	1438.2 (1097.8 to 1843.2)	130.7 (91.5 to 181.6)	10.6 (5.0 to 20.2)	0.0 (0.0 to 0.0)
Guam	2005	1647.7 (1297.0 to 2087.9)	1498.8 (1144.5 to 1921.5)	136.6 (95.4 to 188.1)	12.3 (5.9 to 23.8)	0.0 (0.0 to 0.0)	1647.7 (1297.0 to 2087.9)	1498.8 (1144.5 to 1921.5)	136.6 (95.4 to 188.1)	12.3 (5.9 to 23.8)	0.0 (0.0 to 0.0)
Guam	2006	1617.6 (1258.1 to 2038.5)	1465.6 (1115.6 to 1869.4)	137.6 (96.4 to 189.0)	14.5 (7.0 to 28.1)	0.0 (0.0 to 0.0)	1617.6 (1258.1 to 2038.5)	1465.6 (1115.6 to 1869.4)	137.6 (96.4 to 189.0)	14.5 (7.0 to 28.1)	0.0 (0.0 to 0.0)
Guam	2007	1638.9 (1274.5 to 2052.0)	1478.6 (1110.1 to 1885.0)	141.5 (99.5 to 196.7)	18.7 (9.1 to 36.5)	0.0 (0.0 to 0.0)	1638.9 (1274.5 to 2052.0)	1478.6 (1110.1 to 1885.0)	141.5 (99.5 to 196.7)	18.7 (9.1 to 36.5)	0.0 (0.0 to 0.0)
Guam	2008	1675.6 (1302.6 to 2122.4)	1501.6 (1116.8 to 1931.1)	147.0 (102.5 to 205.3)	27.1 (13.0 to 52.3)	0.0 (0.0 to 0.0)	1675.6 (1302.6 to 2122.4)	1501.6 (1116.8 to 1931.1)	147.0 (102.5 to 205.3)	27.1 (13.0 to 52.3)	0.0 (0.0 to 0.0)
Guam	2009	1690.1 (1312.4 to 2136.5)	1503.4 (1125.4 to 1941.1)	151.3 (103.8 to 209.0)	35.4 (16.9 to 67.1)	0.0 (0.0 to 0.0)	1690.1 (1312.4 to 2136.5)	1503.4 (1125.4 to 1941.1)	151.3 (103.8 to 209.0)	35.4 (16.9 to 67.1)	0.0 (0.0 to 0.0)
Guam	2010	1727.9 (1346.0 to 2166.8)	1527.9 (1154.2 to 1963.2)	156.0 (107.3 to 218.4)	44.1 (20.7 to 83.5)	0.0 (0.0 to 0.0)	1727.9 (1346.0 to 2166.8)	1527.9 (1154.2 to 1963.2)	156.0 (107.3 to 218.4)	44.1 (20.7 to 83.5)	0.0 (0.0 to 0.0)
Guam	2011	1742.0 (1356.1 to 2173.3)	1532.7 (1155.9 to 1943.7)	158.2 (109.8 to 224.2)	51.1 (24.5 to 98.6)	0.0 (0.0 to 0.0)	1742.0 (1356.1 to 2173.3)	1532.7 (1155.9 to 1943.7)	158.2 (109.8 to 224.2)	51.1 (24.5 to 98.6)	0.0 (0.0 to 0.0)
Guam	2012	1789.0 (1394.2 to 2246.7)	1569.1 (1186.7 to 2006.6)	161.4 (110.6 to 226.5)	58.5 (28.2 to 109.2)	0.0 (0.0 to 0.0)	1789.0 (1394.2 to 2246.7)	1569.1 (1186.7 to 2006.6)	161.4 (110.6 to 226.5)	58.5 (28.2 to 109.2)	0.0 (0.0 to 0.0)
Guam	2013	1845.5 (1429.2 to 2303.0)	1615.0 (1211.5 to 2068.4)	165.0 (113.0 to 230.6)	65.5 (31.2 to 121.3)	0.0 (0.0 to 0.0)	1845.5 (1429.2 to 2303.0)	1615.0 (1211.5 to 2068.4)	165.0 (113.0 to 230.6)	65.5 (31.2 to 121.3)	0.0 (0.0 to 0.0)
Guam	2014	1892.6 (1460.0 to 2389.1)	1653.5 (1230.0 to 2133.7)	167.7 (114.7 to 234.9)	71.4 (33.3 to 132.2)	0.0 (0.0 to 0.0)	1892.6 (1460.0 to 2389.1)	1653.5 (1230.0 to 2133.7)	167.7 (114.7 to 234.9)	71.4 (33.3 to 132.2)	0.0 (0.0 to 0.0)
Guam	2015	1944.2 (1499.9 to 2450.9)	1698.9 (1263.1 to 2186.6)	170.2 (116.5 to 238.2)	75.0 (34.4 to 139.0)	0.0 (0.0 to 0.0)	1944.2 (1499.9 to 2450.9)	1698.9 (1263.1 to 2186.6)	170.2 (116.5 to 238.2)	75.0 (34.4 to 139.0)	0.0 (0.0 to 0.0)

Country	Year	2018 US Dollars per capita					2018 PPP per capita				
		Health spending	Government health spending	Out-of-pocket health spending	Prepaid private health spending	Development assistance for health	Health spending	Government health spending	Out-of-pocket health spending	Prepaid private health spending	Development assistance for health
Guam	2016	1990.0 (1548.2 to 2479.8)	1742.1 (1299.0 to 2226.7)	172.4 (118.4 to 238.8)	75.6 (35.4 to 138.3)	0.0 (0.0 to 0.0)	1990.0 (1548.2 to 2479.8)	1742.1 (1299.0 to 2226.7)	172.4 (118.4 to 238.8)	75.6 (35.4 to 138.3)	0.0 (0.0 to 0.0)
Guam	2017	2017.4 (1565.7 to 2515.4)	1766.3 (1318.8 to 2256.8)	174.5 (120.1 to 241.1)	76.6 (35.9 to 140.1)	0.0 (0.0 to 0.0)	2017.4 (1565.7 to 2515.4)	1766.3 (1318.8 to 2256.8)	174.5 (120.1 to 241.1)	76.6 (35.9 to 140.1)	0.0 (0.0 to 0.0)
Guam	2018	2043.8 (1586.5 to 2555.8)	1789.3 (1337.6 to 2285.1)	176.9 (121.3 to 246.0)	77.7 (36.5 to 142.0)	0.0 (0.0 to 0.0)	2043.8 (1586.5 to 2555.8)	1789.3 (1337.6 to 2285.1)	176.9 (121.3 to 246.0)	77.7 (36.5 to 142.0)	0.0 (0.0 to 0.0)
Guam	2019	2071.3 (1606.8 to 2587.2)	1813.1 (1356.6 to 2314.6)	179.3 (123.3 to 248.9)	78.8 (37.0 to 144.1)	0.0 (0.0 to 0.0)	2071.3 (1606.8 to 2587.2)	1813.1 (1356.6 to 2314.6)	179.3 (123.3 to 248.9)	78.8 (37.0 to 144.1)	0.0 (0.0 to 0.0)
Guam	2020	2097.6 (1623.9 to 2624.5)	1835.9 (1372.7 to 2344.0)	181.6 (124.4 to 251.5)	80.0 (37.5 to 146.1)	0.0 (0.0 to 0.0)	2097.6 (1623.9 to 2624.5)	1835.9 (1372.7 to 2344.0)	181.6 (124.4 to 251.5)	80.0 (37.5 to 146.1)	0.0 (0.0 to 0.0)
Guam	2021	2124.5 (1645.8 to 2656.1)	1859.3 (1390.6 to 2375.1)	184.0 (126.4 to 254.6)	81.1 (38.0 to 148.2)	0.0 (0.0 to 0.0)	2124.5 (1645.8 to 2656.1)	1859.3 (1390.6 to 2375.1)	184.0 (126.4 to 254.6)	81.1 (38.0 to 148.2)	0.0 (0.0 to 0.0)
Guam	2022	2151.7 (1666.4 to 2687.9)	1883.0 (1409.8 to 2410.8)	186.4 (127.3 to 258.8)	82.3 (38.4 to 150.2)	0.0 (0.0 to 0.0)	2151.7 (1666.4 to 2687.9)	1883.0 (1409.8 to 2410.8)	186.4 (127.3 to 258.8)	82.3 (38.4 to 150.2)	0.0 (0.0 to 0.0)
Guam	2023	2177.6 (1687.0 to 2725.1)	1905.5 (1425.2 to 2446.8)	188.8 (129.6 to 263.6)	83.3 (38.9 to 152.3)	0.0 (0.0 to 0.0)	2177.6 (1687.0 to 2725.1)	1905.5 (1425.2 to 2446.8)	188.8 (129.6 to 263.6)	83.3 (38.9 to 152.3)	0.0 (0.0 to 0.0)
Guam	2024	2202.8 (1703.5 to 2752.9)	1927.2 (1438.2 to 2481.6)	191.2 (131.3 to 265.6)	84.4 (39.3 to 154.6)	0.0 (0.0 to 0.0)	2202.8 (1703.5 to 2752.9)	1927.2 (1438.2 to 2481.6)	191.2 (131.3 to 265.6)	84.4 (39.3 to 154.6)	0.0 (0.0 to 0.0)
Guam	2025	2225.9 (1722.4 to 2783.3)	1946.9 (1453.5 to 2504.6)	193.7 (133.2 to 267.4)	85.3 (39.8 to 156.4)	0.0 (0.0 to 0.0)	2225.9 (1722.4 to 2783.3)	1946.9 (1453.5 to 2504.6)	193.7 (133.2 to 267.4)	85.3 (39.8 to 156.4)	0.0 (0.0 to 0.0)
Guam	2026	2248.5 (1738.7 to 2810.6)	1966.1 (1467.3 to 2526.8)	196.1 (135.5 to 269.5)	86.3 (40.3 to 158.4)	0.0 (0.0 to 0.0)	2248.5 (1738.7 to 2810.6)	1966.1 (1467.3 to 2526.8)	196.1 (135.5 to 269.5)	86.3 (40.3 to 158.4)	0.0 (0.0 to 0.0)
Guam	2027	2269.5 (1756.2 to 2839.3)	1983.8 (1478.7 to 2553.4)	198.5 (137.5 to 274.2)	87.1 (40.8 to 160.2)	0.0 (0.0 to 0.0)	2269.5 (1756.2 to 2839.3)	1983.8 (1478.7 to 2553.4)	198.5 (137.5 to 274.2)	87.1 (40.8 to 160.2)	0.0 (0.0 to 0.0)
Guam	2028	2290.0 (1766.7 to 2871.9)	2001.1 (1491.2 to 2581.4)	200.9 (139.0 to 278.3)	88.0 (41.2 to 161.4)	0.0 (0.0 to 0.0)	2290.0 (1766.7 to 2871.9)	2001.1 (1491.2 to 2581.4)	200.9 (139.0 to 278.3)	88.0 (41.2 to 161.4)	0.0 (0.0 to 0.0)
Guam	2029	2310.5 (1783.5 to 2899.6)	2018.3 (1501.7 to 2609.4)	203.4 (140.9 to 280.7)	88.9 (41.6 to 163.1)	0.0 (0.0 to 0.0)	2310.5 (1783.5 to 2899.6)	2018.3 (1501.7 to 2609.4)	203.4 (140.9 to 280.7)	88.9 (41.6 to 163.1)	0.0 (0.0 to 0.0)
Guam	2030	2331.2 (1798.8 to 2928.2)	2035.6 (1511.8 to 2632.6)	205.9 (142.6 to 286.1)	89.7 (42.0 to 164.9)	0.0 (0.0 to 0.0)	2331.2 (1798.8 to 2928.2)	2035.6 (1511.8 to 2632.6)	205.9 (142.6 to 286.1)	89.7 (42.0 to 164.9)	0.0 (0.0 to 0.0)
Guam	2031	2353.9 (1821.2 to 2961.7)	2054.7 (1523.9 to 2656.2)	208.5 (143.6 to 291.6)	90.7 (42.4 to 166.6)	0.0 (0.0 to 0.0)	2353.9 (1821.2 to 2961.7)	2054.7 (1523.9 to 2656.2)	208.5 (143.6 to 291.6)	90.7 (42.4 to 166.6)	0.0 (0.0 to 0.0)
Guam	2032	2377.4 (1840.8 to 2993.7)	2074.6 (1537.3 to 2680.6)	211.1 (144.6 to 295.8)	91.7 (42.8 to 168.6)	0.0 (0.0 to 0.0)	2377.4 (1840.8 to 2993.7)	2074.6 (1537.3 to 2680.6)	211.1 (144.6 to 295.8)	91.7 (42.8 to 168.6)	0.0 (0.0 to 0.0)
Guam	2033	2401.5 (1858.3 to 3024.7)	2095.0 (1551.1 to 2706.0)	213.8 (145.8 to 299.0)	92.7 (43.2 to 171.0)	0.0 (0.0 to 0.0)	2401.5 (1858.3 to 3024.7)	2095.0 (1551.1 to 2706.0)	213.8 (145.8 to 299.0)	92.7 (43.2 to 171.0)	0.0 (0.0 to 0.0)
Guam	2034	2426.1 (1877.7 to 3057.1)	2115.8 (1565.2 to 2740.2)	216.6 (147.8 to 303.8)	93.7 (43.7 to 173.1)	0.0 (0.0 to 0.0)	2426.1 (1877.7 to 3057.1)	2115.8 (1565.2 to 2740.2)	216.6 (147.8 to 303.8)	93.7 (43.7 to 173.1)	0.0 (0.0 to 0.0)
Guam	2035	2450.5 (1892.6 to 3093.9)	2136.5 (1579.9 to 2767.4)	219.4 (149.6 to 308.7)	94.7 (44.1 to 175.2)	0.0 (0.0 to 0.0)	2450.5 (1892.6 to 3093.9)	2136.5 (1579.9 to 2767.4)	219.4 (149.6 to 308.7)	94.7 (44.1 to 175.2)	0.0 (0.0 to 0.0)
Guam	2036	2477.2 (1911.6 to 3133.0)	2159.1 (1594.5 to 2798.7)	222.2 (150.8 to 312.9)	95.8 (44.6 to 177.5)	0.0 (0.0 to 0.0)	2477.2 (1911.6 to 3133.0)	2159.1 (1594.5 to 2798.7)	222.2 (150.8 to 312.9)	95.8 (44.6 to 177.5)	0.0 (0.0 to 0.0)

		2018 US Dollars per capita					2018 PPP per capita				
**Country**	**Year**	**Health spending**	**Government health spending**	**Out-of-pocket health spending**	**Prepaid private health spending**	**Development assistance for health**	**Health spending**	**Government health spending**	**Out-of-pocket health spending**	**Prepaid private health spending**	**Development assistance for health**
Guam	2037	2504.0 (1932.1 to 3166.7)	2181.9 (1609.9 to 2831.6)	225.2 (152.8 to 318.8)	97.0 (45.3 to 179.8)	0.0 (0.0 to 0.0)	2504.0 (1932.1 to 3166.7)	2181.9 (1609.9 to 2831.6)	225.2 (152.8 to 318.8)	97.0 (45.3 to 179.8)	0.0 (0.0 to 0.0)
Guam	2038	2532.7 (1954.3 to 3206.3)	2206.3 (1626.5 to 2866.8)	228.2 (154.6 to 324.7)	98.2 (46.2 to 182.2)	0.0 (0.0 to 0.0)	2532.7 (1954.3 to 3206.3)	2206.3 (1626.5 to 2866.8)	228.2 (154.6 to 324.7)	98.2 (46.2 to 182.2)	0.0 (0.0 to 0.0)
Guam	2039	2562.3 (1978.5 to 3249.5)	2231.6 (1643.9 to 2903.3)	231.3 (156.4 to 330.6)	99.4 (47.0 to 184.8)	0.0 (0.0 to 0.0)	2562.3 (1978.5 to 3249.5)	2231.6 (1643.9 to 2903.3)	231.3 (156.4 to 330.6)	99.4 (47.0 to 184.8)	0.0 (0.0 to 0.0)
Guam	2040	2593.6 (2006.8 to 3287.1)	2258.3 (1661.9 to 2941.6)	234.5 (157.3 to 336.6)	100.7 (47.5 to 187.4)	0.0 (0.0 to 0.0)	2593.6 (2006.8 to 3287.1)	2258.3 (1661.9 to 2941.6)	234.5 (157.3 to 336.6)	100.7 (47.5 to 187.4)	0.0 (0.0 to 0.0)
Guam	2041	2627.8 (2025.0 to 3332.1)	2287.8 (1682.2 to 2983.6)	237.9 (159.3 to 342.4)	102.1 (48.1 to 190.3)	0.0 (0.0 to 0.0)	2627.8 (2025.0 to 3332.1)	2287.8 (1682.2 to 2983.6)	237.9 (159.3 to 342.4)	102.1 (48.1 to 190.3)	0.0 (0.0 to 0.0)
Guam	2042	2664.2 (2047.6 to 3383.3)	2319.2 (1703.8 to 3027.4)	241.3 (161.5 to 347.9)	103.7 (48.7 to 193.6)	0.0 (0.0 to 0.0)	2664.2 (2047.6 to 3383.3)	2319.2 (1703.8 to 3027.4)	241.3 (161.5 to 347.9)	103.7 (48.7 to 193.6)	0.0 (0.0 to 0.0)
Guam	2043	2702.7 (2073.5 to 3438.8)	2352.5 (1726.9 to 3075.3)	244.9 (163.9 to 353.1)	105.3 (49.5 to 196.7)	0.0 (0.0 to 0.0)	2702.7 (2073.5 to 3438.8)	2352.5 (1726.9 to 3075.3)	244.9 (163.9 to 353.1)	105.3 (49.5 to 196.7)	0.0 (0.0 to 0.0)
Guam	2044	2742.3 (2101.3 to 3485.6)	2386.8 (1751.1 to 3123.8)	248.6 (166.0 to 358.2)	107.0 (50.4 to 200.0)	0.0 (0.0 to 0.0)	2742.3 (2101.3 to 3485.6)	2386.8 (1751.1 to 3123.8)	248.6 (166.0 to 358.2)	107.0 (50.4 to 200.0)	0.0 (0.0 to 0.0)
Guam	2045	2781.9 (2125.8 to 3547.3)	2421.0 (1774.7 to 3172.4)	252.3 (168.1 to 363.1)	108.6 (51.3 to 203.4)	0.0 (0.0 to 0.0)	2781.9 (2125.8 to 3547.3)	2421.0 (1774.7 to 3172.4)	252.3 (168.1 to 363.1)	108.6 (51.3 to 203.4)	0.0 (0.0 to 0.0)
Guam	2046	2823.1 (2161.6 to 3602.8)	2456.6 (1798.4 to 3226.6)	256.1 (170.4 to 369.5)	110.4 (52.2 to 207.0)	0.0 (0.0 to 0.0)	2823.1 (2161.6 to 3602.8)	2456.6 (1798.4 to 3226.6)	256.1 (170.4 to 369.5)	110.4 (52.2 to 207.0)	0.0 (0.0 to 0.0)
Guam	2047	2865.9 (2182.0 to 3666.5)	2493.6 (1823.8 to 3282.7)	260.1 (172.8 to 376.1)	112.2 (53.2 to 210.5)	0.0 (0.0 to 0.0)	2865.9 (2182.0 to 3666.5)	2493.6 (1823.8 to 3282.7)	260.1 (172.8 to 376.1)	112.2 (53.2 to 210.5)	0.0 (0.0 to 0.0)
Guam	2048	2907.8 (2212.5 to 3720.5)	2529.8 (1848.6 to 3338.4)	264.0 (175.1 to 381.8)	114.0 (54.0 to 214.2)	0.0 (0.0 to 0.0)	2907.8 (2212.5 to 3720.5)	2529.8 (1848.6 to 3338.4)	264.0 (175.1 to 381.8)	114.0 (54.0 to 214.2)	0.0 (0.0 to 0.0)
Guam	2049	2949.5 (2244.5 to 3774.2)	2565.7 (1873.2 to 3390.6)	268.0 (177.5 to 386.9)	115.8 (54.8 to 217.8)	0.0 (0.0 to 0.0)	2949.5 (2244.5 to 3774.2)	2565.7 (1873.2 to 3390.6)	268.0 (177.5 to 386.9)	115.8 (54.8 to 217.8)	0.0 (0.0 to 0.0)
Guam	2050	2989.6 (2271.1 to 3838.1)	2600.1 (1896.7 to 3441.6)	272.0 (180.0 to 393.6)	117.5 (55.6 to 221.3)	0.0 (0.0 to 0.0)	2989.6 (2271.1 to 3838.1)	2600.1 (1896.7 to 3441.6)	272.0 (180.0 to 393.6)	117.5 (55.6 to 221.3)	0.0 (0.0 to 0.0)
Guatemala	1995	147.8 (127.5 to 170.4)	59.1 (48.0 to 72.8)	82.6 (65.6 to 101.0)	2.9 (1.4 to 5.3)	3.1 (3.1 to 3.1)	270.3 (233.2 to 311.7)	108.2 (87.7 to 133.2)	151.1 (120.1 to 184.8)	5.4 (2.6 to 9.8)	5.7 (5.7 to 5.7)
Guatemala	1996	148.8 (129.3 to 169.4)	59.3 (48.5 to 71.9)	83.4 (67.2 to 100.1)	3.0 (1.5 to 5.6)	3.1 (3.1 to 3.1)	272.3 (236.5 to 309.8)	108.5 (88.7 to 131.6)	152.5 (122.8 to 183.0)	5.6 (2.7 to 10.2)	5.8 (5.8 to 5.8)
Guatemala	1997	163.9 (144.3 to 185.8)	62.3 (51.6 to 74.5)	85.7 (70.6 to 101.5)	3.3 (1.6 to 5.9)	12.6 (12.6 to 12.6)	299.7 (264.0 to 339.8)	114.0 (94.3 to 136.3)	156.7 (129.2 to 185.7)	6.0 (3.0 to 10.9)	23.0 (23.0 to 23.0)
Guatemala	1998	162.8 (143.7 to 184.8)	66.0 (55.0 to 79.3)	89.3 (75.4 to 104.9)	3.7 (1.9 to 6.6)	3.7 (3.7 to 3.7)	297.8 (262.8 to 338.0)	120.8 (100.6 to 145.0)	163.4 (137.9 to 191.9)	6.9 (3.5 to 12.1)	6.8 (6.8 to 6.8)
Guatemala	1999	180.5 (160.4 to 202.6)	69.7 (58.8 to 83.5)	99.2 (84.4 to 116.1)	5.1 (2.5 to 9.0)	6.5 (6.5 to 6.5)	330.1 (293.5 to 370.5)	127.4 (107.5 to 152.7)	181.5 (154.3 to 212.4)	9.3 (4.6 to 16.5)	11.9 (11.9 to 11.9)
Guatemala	2000	202.3 (180.0 to 226.8)	73.9 (61.8 to 88.6)	118.5 (102.4 to 137.4)	6.1 (3.1 to 11.0)	3.7 (3.7 to 3.7)	370.0 (329.2 to 414.8)	135.2 (113.1 to 162.0)	216.7 (187.4 to 251.3)	11.2 (5.6 to 20.1)	6.8 (6.8 to 6.8)
Guatemala	2001	230.6 (204.9 to 256.7)	78.9 (66.4 to 94.8)	139.6 (120.7 to 160.6)	7.4 (3.7 to 13.3)	4.7 (4.7 to 4.7)	421.8 (374.8 to 469.6)	144.3 (121.5 to 173.3)	255.3 (220.8 to 293.7)	13.6 (6.8 to 24.4)	8.6 (8.6 to 8.6)

Country	Year	2018 US Dollars per capita					2018 PPP per capita				
		Health spending	Government health spending	Out-of-pocket health spending	Prepaid private health spending	Development assistance for health	Health spending	Government health spending	Out-of-pocket health spending	Prepaid private health spending	Development assistance for health
Guatemala	2002	245.0 (219.3 to 272.0)	82.1 (69.1 to 98.0)	151.4 (132.0 to 173.0)	8.3 (4.1 to 14.6)	3.2 (3.2 to 3.2)	448.1 (401.2 to 497.5)	150.1 (126.4 to 179.3)	277.0 (241.4 to 316.4)	15.1 (7.5 to 26.7)	5.8 (5.8 to 5.8)
Guatemala	2003	253.5 (227.1 to 281.0)	83.4 (70.4 to 100.1)	157.0 (136.2 to 176.7)	8.6 (4.3 to 15.4)	4.5 (4.5 to 4.5)	463.7 (415.4 to 514.0)	152.5 (128.7 to 183.0)	287.1 (249.1 to 323.2)	15.8 (8.0 to 28.1)	8.3 (8.3 to 8.3)
Guatemala	2004	255.5 (229.4 to 282.8)	83.3 (70.4 to 100.4)	159.3 (138.4 to 179.7)	9.9 (5.1 to 17.9)	3.0 (3.0 to 3.0)	467.3 (419.7 to 517.2)	152.3 (128.7 to 183.6)	291.3 (253.2 to 328.6)	18.1 (9.4 to 32.7)	5.5 (5.5 to 5.5)
Guatemala	2005	255.5 (229.1 to 283.6)	82.3 (69.2 to 98.6)	159.8 (138.9 to 180.1)	10.0 (5.3 to 18.0)	3.3 (3.3 to 3.3)	467.4 (419.1 to 518.7)	150.6 (126.7 to 180.4)	292.4 (254.0 to 329.3)	18.3 (9.6 to 32.9)	6.1 (6.1 to 6.1)
Guatemala	2006	260.5 (234.2 to 289.0)	85.2 (71.6 to 101.8)	161.9 (140.6 to 184.0)	9.6 (5.0 to 16.8)	3.8 (3.8 to 3.8)	476.5 (428.4 to 528.6)	155.9 (131.0 to 186.2)	296.2 (257.1 to 336.6)	17.5 (9.1 to 30.8)	7.0 (7.0 to 7.0)
Guatemala	2007	265.5 (237.6 to 293.9)	88.2 (74.0 to 104.6)	162.5 (141.3 to 184.5)	10.3 (5.3 to 18.2)	4.5 (4.5 to 4.5)	485.7 (434.6 to 537.7)	161.3 (135.3 to 191.2)	297.3 (258.5 to 337.4)	18.9 (9.8 to 33.4)	8.2 (8.2 to 8.2)
Guatemala	2008	267.1 (239.3 to 295.4)	89.9 (75.5 to 105.3)	159.2 (139.2 to 181.2)	11.4 (6.0 to 20.1)	6.7 (6.7 to 6.7)	488.6 (437.6 to 540.4)	164.3 (138.1 to 192.7)	291.2 (254.7 to 331.5)	20.9 (11.0 to 36.8)	12.2 (12.2 to 12.2)
Guatemala	2009	260.6 (234.0 to 287.5)	89.8 (75.3 to 105.5)	154.3 (135.0 to 176.6)	11.4 (6.0 to 19.7)	5.1 (5.1 to 5.1)	476.6 (428.0 to 526.0)	164.2 (137.7 to 193.0)	282.3 (246.9 to 323.0)	20.8 (10.9 to 36.1)	9.4 (9.4 to 9.4)
Guatemala	2010	256.8 (231.4 to 283.4)	88.2 (73.7 to 103.4)	151.2 (131.4 to 173.0)	11.8 (6.1 to 20.5)	5.6 (5.6 to 5.6)	469.8 (423.4 to 518.3)	161.4 (134.9 to 189.1)	276.6 (240.3 to 316.4)	21.6 (11.1 to 37.5)	10.2 (10.2 to 10.2)
Guatemala	2011	256.5 (231.6 to 283.4)	88.4 (73.6 to 104.8)	149.1 (129.7 to 168.5)	12.0 (6.1 to 20.5)	7.0 (7.0 to 7.0)	469.1 (423.7 to 518.4)	161.7 (134.7 to 191.7)	272.7 (237.2 to 308.3)	22.0 (11.2 to 37.5)	12.8 (12.8 to 12.8)
Guatemala	2012	253.1 (227.8 to 280.6)	88.9 (73.7 to 106.0)	147.1 (127.1 to 167.3)	12.5 (6.4 to 21.4)	4.7 (4.7 to 4.7)	463.0 (416.7 to 513.2)	162.6 (134.8 to 193.9)	269.0 (232.4 to 306.1)	22.8 (11.8 to 39.2)	8.6 (8.6 to 8.6)
Guatemala	2013	259.6 (232.9 to 287.6)	92.0 (76.8 to 110.1)	145.5 (125.3 to 165.3)	13.7 (7.1 to 24.2)	8.4 (8.4 to 8.4)	474.8 (426.0 to 526.1)	168.3 (140.5 to 201.4)	266.1 (229.1 to 302.4)	25.1 (12.9 to 44.2)	15.3 (15.3 to 15.3)
Guatemala	2014	258.8 (230.6 to 288.4)	94.0 (78.2 to 112.8)	145.0 (123.8 to 165.8)	14.8 (7.5 to 26.5)	4.9 (4.9 to 4.9)	473.4 (421.8 to 527.5)	172.0 (143.0 to 206.3)	265.2 (226.4 to 303.3)	27.1 (13.7 to 48.5)	9.0 (9.0 to 9.0)
Guatemala	2015	275.2 (245.8 to 306.6)	93.3 (76.3 to 112.3)	143.4 (120.5 to 167.0)	16.5 (8.3 to 29.5)	21.9 (21.9 to 21.9)	503.3 (449.5 to 560.8)	170.7 (139.6 to 205.5)	262.3 (220.3 to 305.5)	30.2 (15.2 to 54.0)	40.1 (40.1 to 40.1)
Guatemala	2016	262.0 (226.8 to 300.6)	95.8 (77.4 to 115.6)	143.5 (118.2 to 171.9)	19.5 (9.8 to 35.2)	3.2 (3.2 to 3.2)	479.3 (414.9 to 549.8)	175.3 (141.6 to 211.4)	262.5 (216.2 to 314.4)	35.6 (17.9 to 64.3)	5.9 (5.9 to 5.9)
Guatemala	2017	263.4 (230.2 to 302.0)	96.1 (77.7 to 116.0)	144.1 (118.4 to 172.7)	19.2 (9.7 to 34.9)	3.9 (3.9 to 3.9)	481.8 (421.0 to 552.4)	175.8 (142.1 to 212.1)	263.6 (216.6 to 316.0)	35.2 (17.7 to 63.9)	7.2 (7.2 to 7.2)
Guatemala	2018	268.0 (232.1 to 307.4)	98.6 (79.8 to 119.1)	145.9 (119.8 to 174.7)	19.8 (9.9 to 35.8)	3.7 (3.7 to 3.7)	490.2 (424.5 to 562.3)	180.4 (146.0 to 217.8)	266.9 (219.1 to 319.6)	36.2 (18.1 to 65.6)	6.7 (6.7 to 6.7)
Guatemala	2019	272.7 (237.4 to 311.8)	101.0 (81.8 to 121.9)	147.9 (122.0 to 177.0)	20.2 (10.2 to 36.6)	3.7 (3.4 to 3.9)	498.8 (434.3 to 570.4)	184.7 (149.6 to 223.0)	270.5 (223.2 to 323.8)	37.0 (18.6 to 67.0)	6.7 (6.3 to 7.1)
Guatemala	2020	277.8 (242.1 to 318.7)	103.6 (83.9 to 124.9)	149.8 (123.8 to 179.5)	20.7 (10.4 to 37.5)	3.7 (3.4 to 4.0)	508.1 (442.8 to 582.9)	189.4 (153.5 to 228.5)	274.1 (226.4 to 328.4)	37.8 (19.0 to 68.6)	6.8 (6.2 to 7.3)
Guatemala	2021	282.4 (246.2 to 322.5)	105.7 (85.7 to 127.5)	151.8 (125.4 to 181.7)	21.1 (10.6 to 38.4)	3.7 (3.4 to 4.1)	516.5 (450.3 to 589.9)	193.4 (156.7 to 233.3)	277.6 (229.4 to 332.3)	38.7 (19.4 to 70.3)	6.8 (6.1 to 7.5)
Guatemala	2022	288.0 (251.4 to 330.4)	108.6 (88.1 to 131.1)	153.9 (127.0 to 184.0)	21.6 (10.8 to 39.3)	3.8 (3.3 to 4.2)	526.7 (459.8 to 604.4)	198.7 (161.1 to 239.8)	281.6 (232.3 to 336.6)	39.6 (19.8 to 71.9)	6.9 (6.1 to 7.7)

		2018 US Dollars per capita					2018 PPP per capita				
Country	Year	Health spending	Government health spending	Out-of-pocket health spending	Prepaid private health spending	Development assistance for health	Health spending	Government health spending	Out-of-pocket health spending	Prepaid private health spending	Development assistance for health
Guatemala	2023	293.2 (255.7 to 334.0)	111.2 (90.3 to 134.4)	156.0 (128.6 to 186.6)	22.1 (11.1 to 40.1)	3.8 (3.3 to 4.4)	536.3 (467.7 to 610.9)	203.5 (165.2 to 245.8)	285.4 (235.3 to 341.3)	40.5 (20.2 to 73.4)	7.0 (6.1 to 8.0)
Guatemala	2024	298.6 (260.3 to 342.3)	114.1 (92.6 to 137.9)	158.1 (130.0 to 188.8)	22.6 (11.3 to 40.9)	3.9 (3.3 to 4.5)	546.2 (476.1 to 626.2)	208.7 (169.4 to 252.2)	289.1 (237.7 to 345.3)	41.4 (20.6 to 74.8)	7.1 (6.1 to 8.2)
Guatemala	2025	304.5 (264.9 to 347.3)	117.5 (95.4 to 142.1)	160.1 (132.3 to 191.0)	23.1 (11.5 to 41.8)	3.9 (3.3 to 4.6)	557.0 (484.5 to 635.3)	214.9 (174.5 to 259.9)	292.8 (242.1 to 349.4)	42.2 (21.0 to 76.4)	7.2 (6.1 to 8.4)
Guatemala	2026	309.9 (270.7 to 355.0)	120.5 (97.8 to 145.8)	161.9 (133.8 to 193.1)	23.6 (11.7 to 42.6)	4.0 (3.3 to 4.7)	566.8 (495.1 to 649.4)	220.3 (179.0 to 266.7)	296.1 (244.8 to 353.2)	43.1 (21.4 to 77.9)	7.2 (6.1 to 8.5)
Guatemala	2027	314.3 (275.0 to 358.9)	122.7 (99.7 to 148.6)	163.5 (135.2 to 195.0)	24.0 (11.9 to 43.4)	4.0 (3.3 to 4.8)	574.8 (503.1 to 656.6)	224.5 (182.5 to 271.9)	299.1 (247.2 to 356.8)	43.9 (21.9 to 79.4)	7.3 (6.1 to 8.7)
Guatemala	2028	318.3 (276.3 to 365.0)	124.8 (101.4 to 151.2)	165.0 (136.3 to 196.8)	24.5 (12.2 to 44.2)	4.1 (3.4 to 4.9)	582.2 (505.4 to 667.7)	228.2 (185.5 to 276.6)	301.8 (249.3 to 360.0)	44.8 (22.2 to 80.9)	7.5 (6.2 to 9.0)
Guatemala	2029	322.3 (280.0 to 367.2)	126.7 (102.7 to 153.7)	166.5 (137.5 to 198.8)	24.9 (12.4 to 45.1)	4.1 (3.4 to 5.1)	589.6 (512.2 to 671.7)	231.8 (187.9 to 281.1)	304.6 (251.5 to 363.7)	45.6 (22.6 to 82.4)	7.6 (6.2 to 9.3)
Guatemala	2030	326.4 (283.2 to 372.7)	128.7 (104.0 to 155.7)	168.0 (138.3 to 201.2)	25.4 (12.6 to 45.8)	4.2 (3.4 to 5.2)	597.0 (518.0 to 681.8)	235.4 (190.2 to 284.7)	307.4 (253.0 to 368.0)	46.5 (23.0 to 83.8)	7.7 (6.3 to 9.5)
Guatemala	2031	330.5 (286.8 to 376.6)	130.7 (105.7 to 158.2)	169.6 (139.4 to 203.0)	25.9 (12.9 to 46.6)	4.3 (3.4 to 5.4)	604.5 (524.5 to 688.8)	239.1 (193.3 to 289.3)	310.2 (255.0 to 371.4)	47.4 (23.5 to 85.3)	7.8 (6.3 to 9.8)
Guatemala	2032	334.7 (290.8 to 384.0)	132.8 (107.3 to 160.9)	171.1 (140.6 to 204.9)	26.4 (13.1 to 47.4)	4.3 (3.5 to 5.5)	612.2 (531.9 to 702.4)	242.9 (196.3 to 294.4)	313.0 (257.1 to 374.8)	48.3 (24.0 to 86.8)	7.9 (6.4 to 10.1)
Guatemala	2033	338.8 (294.1 to 386.5)	134.8 (108.7 to 163.4)	172.7 (142.1 to 206.7)	26.9 (13.4 to 48.4)	4.4 (3.5 to 5.6)	619.7 (537.9 to 707.0)	246.6 (198.8 to 299.0)	315.8 (259.8 to 378.2)	49.2 (24.4 to 88.6)	8.1 (6.4 to 10.3)
Guatemala	2034	342.8 (297.1 to 393.3)	136.8 (110.0 to 165.9)	174.1 (143.2 to 208.4)	27.4 (13.6 to 49.5)	4.5 (3.5 to 5.8)	627.1 (543.4 to 719.4)	250.1 (201.3 to 303.5)	318.5 (262.0 to 381.3)	50.2 (24.9 to 90.5)	8.2 (6.5 to 10.6)
Guatemala	2035	346.9 (302.5 to 396.7)	138.8 (111.4 to 168.7)	175.6 (144.2 to 210.2)	27.9 (13.9 to 50.4)	4.6 (3.6 to 6.0)	634.6 (553.4 to 725.6)	253.8 (203.8 to 308.5)	321.3 (263.8 to 384.4)	51.1 (25.4 to 92.3)	8.4 (6.6 to 11.0)
Guatemala	2036	351.0 (303.0 to 404.0)	140.8 (113.0 to 171.2)	177.1 (145.1 to 212.0)	28.5 (14.1 to 51.4)	4.7 (3.6 to 6.2)	642.1 (554.3 to 739.0)	257.5 (206.8 to 313.1)	324.0 (265.4 to 387.8)	52.1 (25.9 to 94.0)	8.6 (6.7 to 11.4)
Guatemala	2037	355.1 (308.4 to 407.4)	142.7 (114.4 to 173.7)	178.6 (146.2 to 213.7)	29.0 (14.4 to 52.3)	4.8 (3.7 to 6.4)	649.5 (564.2 to 745.2)	261.1 (209.3 to 317.6)	326.7 (267.5 to 390.9)	53.0 (26.4 to 95.6)	8.7 (6.7 to 11.8)
Guatemala	2038	359.2 (308.5 to 412.6)	144.7 (115.7 to 176.4)	180.0 (147.2 to 215.8)	29.5 (14.7 to 53.1)	4.9 (3.7 to 6.7)	656.9 (564.3 to 754.8)	264.8 (211.7 to 322.7)	329.3 (269.2 to 394.6)	54.0 (26.9 to 97.2)	8.9 (6.7 to 12.2)
Guatemala	2039	363.2 (315.0 to 416.0)	146.8 (117.3 to 178.9)	181.4 (148.3 to 217.8)	30.1 (15.0 to 54.1)	4.9 (3.8 to 6.8)	664.4 (576.2 to 760.9)	268.4 (214.5 to 327.2)	331.9 (271.3 to 398.4)	55.0 (27.4 to 99.0)	9.1 (6.9 to 12.4)
Guatemala	2040	367.0 (318.1 to 421.1)	148.6 (118.6 to 181.0)	182.8 (149.1 to 219.8)	30.6 (15.3 to 55.2)	5.1 (3.8 to 7.0)	671.4 (581.8 to 770.3)	271.9 (216.9 to 331.2)	334.3 (272.8 to 402.0)	56.0 (27.9 to 100.9)	9.2 (7.0 to 12.7)
Guatemala	2041	370.9 (323.2 to 424.7)	150.5 (120.0 to 183.4)	184.1 (150.1 to 221.6)	31.1 (15.6 to 56.1)	5.2 (3.9 to 7.3)	678.5 (591.2 to 776.8)	275.4 (219.5 to 335.6)	336.7 (274.6 to 405.4)	57.0 (28.5 to 102.7)	9.4 (7.1 to 13.3)
Guatemala	2042	374.8 (322.4 to 430.0)	152.5 (121.5 to 185.9)	185.4 (151.1 to 223.3)	31.7 (15.8 to 57.1)	5.3 (3.9 to 7.6)	685.6 (589.8 to 786.5)	278.9 (222.2 to 340.1)	339.1 (276.4 to 408.4)	58.0 (29.0 to 104.5)	9.7 (7.1 to 13.9)
Guatemala	2043	378.6 (329.7 to 435.1)	154.3 (122.7 to 188.4)	186.7 (152.1 to 225.3)	32.2 (16.1 to 58.1)	5.4 (4.0 to 7.9)	692.5 (603.0 to 795.8)	282.3 (224.5 to 344.7)	341.4 (278.1 to 412.1)	59.0 (29.5 to 106.2)	9.9 (7.3 to 14.5)

Country	Year	2018 US Dollars per capita					2018 PPP per capita				
		Health spending	Government health spending	Out-of-pocket health spending	Prepaid private health spending	Development assistance for health	Health spending	Government health spending	Out-of-pocket health spending	Prepaid private health spending	Development assistance for health
Guatemala	2044	382.3 (330.0 to 437.9)	156.2 (123.9 to 190.9)	187.9 (152.9 to 226.7)	32.8 (16.4 to 59.1)	5.5 (4.1 to 8.1)	699.4 (603.6 to 800.9)	285.7 (226.7 to 349.3)	343.6 (279.8 to 414.7)	60.0 (30.0 to 108.0)	10.1 (7.4 to 14.8)
Guatemala	2045	386.9 (335.5 to 443.7)	158.7 (125.7 to 194.3)	189.2 (153.9 to 228.5)	33.3 (16.7 to 60.0)	5.7 (4.1 to 8.6)	707.6 (613.7 to 811.6)	290.3 (230.0 to 355.4)	346.0 (281.5 to 417.9)	61.0 (30.5 to 109.8)	10.3 (7.5 to 15.8)
Guatemala	2046	392.2 (335.8 to 450.2)	162.0 (128.3 to 198.6)	190.6 (154.8 to 230.3)	33.9 (17.0 to 61.0)	5.8 (4.2 to 8.8)	717.5 (614.2 to 823.5)	296.3 (234.7 to 363.2)	348.6 (283.2 to 421.3)	62.0 (31.1 to 111.6)	10.6 (7.6 to 16.1)
Guatemala	2047	398.3 (343.6 to 460.1)	165.8 (131.0 to 203.5)	192.1 (156.0 to 232.2)	34.4 (17.3 to 62.0)	5.9 (4.2 to 9.3)	728.5 (628.5 to 841.7)	303.4 (239.7 to 372.2)	351.3 (285.4 to 424.6)	63.0 (31.6 to 113.4)	10.9 (7.8 to 17.0)
Guatemala	2048	405.0 (346.7 to 466.8)	170.3 (134.3 to 209.3)	193.6 (157.4 to 234.3)	35.0 (17.5 to 63.0)	6.1 (4.4 to 9.8)	740.8 (634.2 to 853.8)	311.6 (245.7 to 382.9)	354.1 (288.0 to 428.5)	64.0 (32.0 to 115.2)	11.1 (8.0 to 17.9)
Guatemala	2049	412.4 (355.2 to 477.7)	175.4 (138.2 to 215.8)	195.2 (159.0 to 236.3)	35.5 (17.7 to 64.0)	6.2 (4.4 to 10.0)	754.3 (649.6 to 873.8)	320.8 (252.8 to 394.8)	357.0 (290.8 to 432.2)	65.0 (32.3 to 117.1)	11.4 (8.0 to 18.3)
Guatemala	2050	420.7 (360.6 to 483.0)	181.3 (142.8 to 223.3)	196.9 (160.7 to 238.4)	36.1 (18.0 to 65.1)	6.4 (4.5 to 10.3)	769.6 (659.7 to 883.4)	331.6 (261.2 to 408.5)	360.2 (293.9 to 436.1)	66.1 (32.9 to 119.1)	11.7 (8.2 to 18.9)
Guinea	1995	21.5 (17.4 to 26.5)	2.4 (1.8 to 3.1)	12.9 (8.9 to 17.7)	0.1 (0.0 to 0.1)	6.2 (6.2 to 6.2)	57.8 (46.8 to 71.0)	6.5 (4.8 to 8.4)	34.6 (23.8 to 47.5)	0.2 (0.1 to 0.3)	16.5 (16.5 to 16.5)
Guinea	1996	18.4 (14.1 to 23.3)	2.7 (2.0 to 3.5)	13.4 (9.3 to 18.4)	0.1 (0.0 to 0.1)	2.1 (2.1 to 2.1)	49.2 (37.8 to 62.5)	7.3 (5.4 to 9.5)	36.0 (25.1 to 49.3)	0.2 (0.1 to 0.3)	5.8 (5.8 to 5.8)
Guinea	1997	20.7 (16.6 to 25.5)	2.8 (2.1 to 3.6)	13.4 (9.5 to 18.2)	0.1 (0.0 to 0.1)	4.5 (4.5 to 4.5)	55.6 (44.5 to 68.5)	7.4 (5.5 to 9.7)	36.0 (25.3 to 48.8)	0.2 (0.1 to 0.3)	12.0 (12.0 to 12.0)
Guinea	1998	20.4 (16.0 to 25.5)	2.9 (2.2 to 3.8)	13.8 (9.4 to 18.7)	0.1 (0.0 to 0.1)	3.7 (3.7 to 3.7)	54.7 (43.0 to 68.3)	7.8 (5.9 to 10.3)	36.9 (25.3 to 50.1)	0.2 (0.1 to 0.3)	9.8 (9.8 to 9.8)
Guinea	1999	19.3 (14.9 to 24.5)	3.0 (2.3 to 3.9)	13.9 (9.5 to 18.9)	0.1 (0.0 to 0.1)	2.4 (2.4 to 2.4)	51.7 (39.8 to 65.6)	8.0 (6.1 to 10.5)	37.2 (25.4 to 50.7)	0.2 (0.1 to 0.3)	6.3 (6.3 to 6.3)
Guinea	2000	20.1 (15.8 to 25.3)	3.0 (2.3 to 4.0)	13.9 (9.6 to 18.9)	0.1 (0.0 to 0.1)	3.1 (3.1 to 3.1)	53.9 (42.4 to 67.8)	8.1 (6.2 to 10.7)	37.3 (25.7 to 50.8)	0.2 (0.1 to 0.3)	8.4 (8.4 to 8.4)
Guinea	2001	22.2 (17.8 to 27.7)	3.3 (2.6 to 4.4)	14.3 (9.9 to 19.6)	0.1 (0.0 to 0.1)	4.5 (4.5 to 4.5)	59.5 (47.8 to 74.3)	8.9 (6.9 to 11.9)	38.4 (26.4 to 52.5)	0.2 (0.1 to 0.3)	12.0 (12.0 to 12.0)
Guinea	2002	22.0 (17.3 to 27.9)	3.8 (2.9 to 5.0)	15.1 (10.4 to 20.9)	0.1 (0.0 to 0.1)	3.0 (3.0 to 3.0)	59.0 (46.4 to 74.9)	10.2 (7.9 to 13.5)	40.5 (27.8 to 56.0)	0.2 (0.1 to 0.4)	8.1 (8.1 to 8.1)
Guinea	2003	20.6 (16.1 to 26.3)	3.4 (2.6 to 4.5)	14.4 (10.0 to 20.1)	0.1 (0.0 to 0.1)	2.8 (2.8 to 2.8)	55.3 (43.1 to 70.6)	9.0 (7.0 to 12.0)	38.5 (26.7 to 53.8)	0.2 (0.1 to 0.4)	7.6 (7.6 to 7.6)
Guinea	2004	20.2 (15.9 to 25.8)	2.8 (2.1 to 3.7)	14.0 (9.7 to 19.3)	0.1 (0.0 to 0.1)	3.4 (3.4 to 3.4)	54.2 (42.5 to 69.1)	7.5 (5.7 to 9.9)	37.5 (26.0 to 51.9)	0.2 (0.1 to 0.3)	9.0 (9.0 to 9.0)
Guinea	2005	20.5 (16.2 to 26.3)	2.6 (2.0 to 3.4)	14.1 (9.8 to 19.5)	0.1 (0.0 to 0.1)	3.7 (3.7 to 3.7)	54.9 (43.5 to 70.6)	7.1 (5.4 to 9.2)	37.8 (26.3 to 52.2)	0.2 (0.1 to 0.4)	9.8 (9.8 to 9.8)
Guinea	2006	20.8 (16.6 to 26.4)	2.5 (1.9 to 3.2)	14.1 (9.9 to 19.3)	0.1 (0.0 to 0.1)	4.2 (4.2 to 4.2)	55.9 (44.4 to 70.8)	6.6 (5.1 to 8.7)	37.9 (26.7 to 51.7)	0.2 (0.1 to 0.4)	11.2 (11.2 to 11.2)
Guinea	2007	20.1 (15.9 to 25.5)	2.5 (1.9 to 3.2)	14.6 (10.4 to 19.9)	0.1 (0.0 to 0.1)	2.9 (2.9 to 2.9)	53.8 (42.6 to 68.5)	6.6 (5.0 to 8.7)	39.2 (27.9 to 53.3)	0.2 (0.1 to 0.4)	7.8 (7.8 to 7.8)
Guinea	2008	21.4 (17.2 to 26.6)	2.6 (2.0 to 3.4)	14.9 (10.8 to 20.1)	0.1 (0.0 to 0.2)	3.8 (3.8 to 3.8)	57.4 (46.2 to 71.3)	6.9 (5.2 to 9.1)	40.0 (28.8 to 53.9)	0.3 (0.1 to 0.5)	10.2 (10.2 to 10.2)

		2018 US Dollars per capita					2018 PPP per capita				
Country	Year	Health spending	Government health spending	Out-of-pocket health spending	Prepaid private health spending	Development assistance for health	Health spending	Government health spending	Out-of-pocket health spending	Prepaid private health spending	Development assistance for health
Guinea	2009	20.6 (16.5 to 25.9)	2.6 (2.0 to 3.4)	15.1 (11.0 to 20.3)	0.1 (0.1 to 0.2)	2.8 (2.8 to 2.8)	55.3 (44.3 to 69.4)	7.0 (5.3 to 9.1)	40.5 (29.4 to 54.5)	0.3 (0.1 to 0.6)	7.6 (7.6 to 7.6)
Guinea	2010	23.7 (19.3 to 29.4)	3.1 (2.3 to 4.0)	16.3 (11.8 to 22.1)	0.2 (0.1 to 0.3)	4.1 (4.1 to 4.1)	63.6 (51.7 to 79.0)	8.4 (6.2 to 10.8)	43.7 (31.7 to 59.3)	0.4 (0.2 to 0.9)	11.1 (11.1 to 11.1)
Guinea	2011	24.7 (19.5 to 31.2)	3.6 (2.7 to 4.8)	17.7 (12.8 to 23.9)	0.3 (0.1 to 0.5)	3.1 (3.1 to 3.1)	66.2 (52.4 to 83.6)	9.8 (7.3 to 12.8)	47.5 (34.2 to 64.1)	0.7 (0.3 to 1.3)	8.3 (8.3 to 8.3)
Guinea	2012	26.4 (20.8 to 33.0)	4.0 (3.0 to 5.2)	18.9 (13.5 to 25.4)	0.4 (0.2 to 0.8)	3.1 (3.1 to 3.1)	70.8 (55.8 to 88.6)	10.8 (8.1 to 14.1)	50.6 (36.1 to 68.1)	1.1 (0.5 to 2.1)	8.3 (8.3 to 8.3)
Guinea	2013	28.0 (22.1 to 35.3)	4.3 (3.2 to 5.5)	20.0 (14.2 to 27.1)	0.8 (0.4 to 1.5)	2.9 (2.9 to 2.9)	75.0 (59.3 to 94.6)	11.4 (8.5 to 14.8)	53.8 (38.1 to 72.6)	2.1 (1.0 to 4.0)	7.8 (7.8 to 7.8)
Guinea	2014	43.9 (37.2 to 51.9)	4.5 (3.3 to 5.8)	22.3 (16.0 to 30.3)	2.0 (1.0 to 4.0)	15.1 (15.1 to 15.1)	117.8 (99.7 to 139.2)	12.1 (9.0 to 15.6)	59.8 (43.0 to 81.4)	5.4 (2.6 to 10.7)	40.5 (40.5 to 40.5)
Guinea	2015	46.1 (38.9 to 54.9)	4.3 (3.2 to 5.6)	22.9 (16.3 to 31.0)	3.6 (1.7 to 7.2)	15.3 (15.3 to 15.3)	123.6 (104.3 to 147.3)	11.6 (8.7 to 15.0)	61.4 (43.6 to 83.2)	9.7 (4.5 to 19.3)	40.9 (40.9 to 40.9)
Guinea	2016	44.2 (37.0 to 53.5)	4.9 (3.6 to 6.3)	23.7 (16.9 to 32.0)	4.4 (2.1 to 8.9)	11.2 (11.2 to 11.2)	118.6 (99.1 to 143.4)	13.0 (9.8 to 16.9)	63.7 (45.2 to 85.7)	11.8 (5.5 to 24.0)	30.2 (30.2 to 30.2)
Guinea	2017	42.3 (34.0 to 51.8)	5.2 (3.9 to 6.7)	24.7 (17.6 to 33.4)	4.5 (2.1 to 9.2)	7.8 (7.8 to 7.8)	113.4 (91.2 to 139.0)	13.9 (10.5 to 18.0)	66.3 (47.1 to 89.6)	12.2 (5.7 to 24.8)	20.9 (20.9 to 20.9)
Guinea	2018	42.2 (34.5 to 51.7)	5.3 (4.0 to 6.8)	25.1 (17.8 to 33.9)	4.7 (2.2 to 9.4)	7.2 (7.2 to 7.3)	113.2 (92.5 to 138.5)	14.1 (10.6 to 18.3)	67.2 (47.7 to 90.9)	12.5 (5.8 to 25.2)	19.4 (19.4 to 19.4)
Guinea	2019	42.6 (34.2 to 52.6)	5.3 (4.0 to 6.9)	25.4 (18.1 to 34.4)	4.7 (2.2 to 9.6)	7.2 (6.7 to 7.6)	114.2 (91.7 to 141.1)	14.3 (10.8 to 18.5)	68.0 (48.5 to 92.1)	12.7 (5.9 to 25.7)	19.2 (18.0 to 20.4)
Guinea	2020	43.0 (35.0 to 52.5)	5.4 (4.1 to 7.0)	25.6 (18.2 to 34.7)	4.8 (2.2 to 9.7)	7.2 (6.6 to 7.8)	115.4 (94.0 to 140.9)	14.5 (10.9 to 18.8)	68.8 (48.8 to 93.1)	12.9 (6.0 to 26.1)	19.3 (17.7 to 20.8)
Guinea	2021	43.5 (34.8 to 53.5)	5.5 (4.1 to 7.1)	25.9 (18.4 to 35.1)	4.9 (2.3 to 9.9)	7.2 (6.5 to 8.0)	116.7 (93.3 to 143.4)	14.7 (11.0 to 19.1)	69.5 (49.4 to 94.2)	13.1 (6.1 to 26.5)	19.3 (17.4 to 21.4)
Guinea	2022	44.0 (35.6 to 54.0)	5.5 (4.2 to 7.2)	26.2 (18.7 to 35.4)	5.0 (2.3 to 10.0)	7.3 (6.5 to 8.1)	118.0 (95.5 to 144.7)	14.9 (11.2 to 19.3)	70.3 (50.1 to 95.0)	13.3 (6.2 to 26.9)	19.5 (17.3 to 21.8)
Guinea	2023	44.5 (35.5 to 54.7)	5.6 (4.2 to 7.3)	26.5 (18.8 to 36.0)	5.1 (2.4 to 10.2)	7.4 (6.4 to 8.4)	119.4 (95.1 to 146.6)	15.1 (11.4 to 19.6)	71.0 (50.5 to 96.6)	13.6 (6.3 to 27.5)	19.7 (17.1 to 22.4)
Guinea	2024	45.2 (36.5 to 55.4)	5.7 (4.3 to 7.4)	26.8 (19.0 to 36.4)	5.2 (2.4 to 10.4)	7.5 (6.5 to 8.7)	121.3 (97.9 to 148.6)	15.4 (11.6 to 19.9)	71.9 (50.8 to 97.5)	13.8 (6.5 to 28.0)	20.2 (17.4 to 23.3)
Guinea	2025	45.9 (36.6 to 56.6)	5.8 (4.4 to 7.6)	27.1 (19.2 to 36.7)	5.3 (2.5 to 10.6)	7.7 (6.6 to 9.0)	123.1 (98.0 to 151.7)	15.7 (11.8 to 20.3)	72.8 (51.4 to 98.4)	14.1 (6.6 to 28.5)	20.6 (17.6 to 24.0)
Guinea	2026	46.6 (37.8 to 57.5)	5.9 (4.5 to 7.7)	27.5 (19.4 to 37.3)	5.4 (2.5 to 10.9)	7.8 (6.6 to 9.2)	125.0 (101.3 to 154.1)	16.0 (12.0 to 20.7)	73.7 (52.1 to 99.9)	14.4 (6.7 to 29.1)	21.0 (17.7 to 24.6)
Guinea	2027	47.4 (37.8 to 58.2)	6.1 (4.6 to 7.9)	27.9 (19.7 to 37.9)	5.5 (2.6 to 11.1)	8.0 (6.7 to 9.4)	127.2 (101.3 to 156.1)	16.3 (12.2 to 21.1)	74.8 (52.9 to 101.7)	14.7 (6.9 to 29.8)	21.3 (17.9 to 25.3)
Guinea	2028	48.3 (39.2 to 59.8)	6.2 (4.7 to 8.1)	28.3 (20.0 to 38.7)	5.6 (2.6 to 11.4)	8.1 (6.8 to 9.8)	129.5 (105.1 to 160.4)	16.7 (12.5 to 21.6)	76.0 (53.7 to 103.8)	15.1 (7.0 to 30.6)	21.7 (18.2 to 26.3)
Guinea	2029	49.2 (39.3 to 60.9)	6.4 (4.8 to 8.3)	28.8 (20.4 to 39.5)	5.8 (2.7 to 11.7)	8.2 (6.8 to 10.2)	131.9 (105.3 to 163.2)	17.1 (12.8 to 22.2)	77.2 (54.6 to 105.9)	15.4 (7.2 to 31.4)	22.1 (18.3 to 27.2)

Country	Year	2018 US Dollars per capita					2018 PPP per capita				
		Health spending	Government health spending	Out-of-pocket health spending	Prepaid private health spending	Development assistance for health	Health spending	Government health spending	Out-of-pocket health spending	Prepaid private health spending	Development assistance for health
Guinea	2030	50.1 (40.5 to 62.1)	6.5 (4.9 to 8.5)	29.3 (20.7 to 40.2)	5.9 (2.8 to 12.0)	8.4 (6.9 to 10.3)	134.5 (108.6 to 166.5)	17.6 (13.2 to 22.7)	78.6 (55.4 to 107.7)	15.9 (7.4 to 32.3)	22.4 (18.4 to 27.7)
Guinea	2031	51.2 (40.9 to 63.6)	6.7 (5.0 to 8.7)	29.9 (21.0 to 41.1)	6.1 (2.8 to 12.4)	8.5 (6.8 to 10.7)	137.2 (109.7 to 170.6)	18.0 (13.5 to 23.4)	80.1 (56.3 to 110.1)	16.3 (7.6 to 33.2)	22.8 (18.3 to 28.8)
Guinea	2032	52.2 (41.9 to 64.9)	6.9 (5.2 to 9.0)	30.4 (21.4 to 42.0)	6.2 (2.9 to 12.7)	8.6 (6.9 to 10.8)	140.0 (112.4 to 174.1)	18.5 (13.9 to 24.0)	81.6 (57.3 to 112.6)	16.8 (7.8 to 34.1)	23.1 (18.6 to 29.1)
Guinea	2033	53.3 (42.2 to 66.7)	7.1 (5.3 to 9.2)	31.0 (21.7 to 42.8)	6.4 (3.0 to 13.1)	8.8 (7.0 to 11.2)	143.0 (113.1 to 178.9)	19.0 (14.2 to 24.8)	83.2 (58.2 to 114.9)	17.2 (8.0 to 35.1)	23.5 (18.7 to 30.2)
Guinea	2034	54.4 (43.4 to 68.5)	7.3 (5.4 to 9.5)	31.6 (22.0 to 43.9)	6.6 (3.0 to 13.5)	8.9 (7.0 to 11.7)	146.0 (116.4 to 183.8)	19.6 (14.6 to 25.5)	84.7 (59.1 to 117.6)	17.7 (8.2 to 36.1)	24.0 (18.8 to 31.3)
Guinea	2035	55.6 (44.0 to 69.4)	7.5 (5.6 to 9.8)	32.2 (22.4 to 44.9)	6.8 (3.1 to 13.9)	9.1 (7.1 to 11.9)	149.2 (118.0 to 186.1)	20.1 (15.0 to 26.4)	86.4 (60.1 to 120.5)	18.2 (8.4 to 37.2)	24.4 (19.0 to 31.9)
Guinea	2036	56.8 (45.3 to 71.5)	7.7 (5.8 to 10.1)	32.8 (22.7 to 45.9)	7.0 (3.2 to 14.3)	9.3 (7.2 to 12.4)	152.3 (121.4 to 191.7)	20.7 (15.4 to 27.2)	88.0 (60.9 to 123.2)	18.7 (8.6 to 38.2)	24.9 (19.3 to 33.4)
Guinea	2037	58.0 (45.6 to 72.7)	7.9 (5.9 to 10.4)	33.4 (23.1 to 47.0)	7.2 (3.3 to 14.7)	9.4 (7.3 to 12.7)	155.6 (122.3 to 194.8)	21.3 (15.8 to 28.0)	89.7 (61.9 to 125.9)	19.3 (8.8 to 39.3)	25.3 (19.5 to 34.1)
Guinea	2038	59.3 (47.3 to 75.2)	8.2 (6.1 to 10.7)	34.1 (23.5 to 48.0)	7.4 (3.4 to 15.1)	9.6 (7.3 to 13.5)	158.9 (126.7 to 201.8)	21.9 (16.3 to 28.7)	91.4 (63.0 to 128.7)	19.8 (9.0 to 40.4)	25.8 (19.5 to 36.2)
Guinea	2039	60.5 (47.2 to 75.8)	8.4 (6.2 to 11.0)	34.8 (23.9 to 49.0)	7.6 (3.4 to 15.5)	9.8 (7.4 to 13.8)	162.4 (126.5 to 203.4)	22.5 (16.7 to 29.5)	93.2 (64.2 to 131.5)	20.4 (9.2 to 41.6)	26.3 (19.9 to 37.1)
Guinea	2040	61.9 (49.0 to 79.3)	8.6 (6.4 to 11.3)	35.4 (24.4 to 50.1)	7.8 (3.5 to 16.0)	10.0 (7.5 to 14.1)	165.9 (131.4 to 212.5)	23.1 (17.2 to 30.4)	95.0 (65.4 to 134.3)	21.0 (9.4 to 42.8)	26.8 (20.0 to 37.7)
Guinea	2041	63.2 (49.3 to 79.2)	8.9 (6.6 to 11.6)	36.1 (24.9 to 51.1)	8.0 (3.6 to 16.4)	10.2 (7.6 to 14.5)	169.6 (132.2 to 212.3)	23.8 (17.6 to 31.2)	96.8 (66.7 to 137.0)	21.6 (9.7 to 44.0)	27.5 (20.3 to 38.9)
Guinea	2042	64.7 (51.1 to 82.0)	9.1 (6.8 to 12.0)	36.8 (25.3 to 52.2)	8.3 (3.7 to 16.9)	10.5 (7.6 to 15.2)	173.4 (137.1 to 219.9)	24.4 (18.1 to 32.1)	98.7 (67.8 to 139.9)	22.2 (9.9 to 45.3)	28.1 (20.3 to 40.8)
Guinea	2043	66.1 (52.0 to 83.5)	9.4 (6.9 to 12.4)	37.5 (25.7 to 53.3)	8.5 (3.8 to 17.4)	10.7 (7.8 to 15.6)	177.3 (139.3 to 224.0)	25.1 (18.6 to 33.1)	100.6 (68.9 to 142.8)	22.8 (10.2 to 46.6)	28.7 (20.9 to 41.9)
Guinea	2044	67.6 (53.1 to 85.7)	9.6 (7.1 to 12.7)	38.3 (26.1 to 54.4)	8.8 (3.9 to 17.9)	11.0 (7.9 to 16.1)	181.3 (142.3 to 229.8)	25.8 (19.2 to 34.2)	102.6 (69.9 to 145.9)	23.5 (10.4 to 47.9)	29.5 (21.1 to 43.3)
Guinea	2045	69.2 (54.2 to 88.0)	9.9 (7.3 to 13.1)	39.0 (26.5 to 55.6)	9.0 (4.0 to 18.4)	11.3 (7.9 to 17.3)	185.5 (145.4 to 236.0)	26.6 (19.7 to 35.2)	104.6 (71.0 to 149.0)	24.2 (10.7 to 49.4)	30.2 (21.3 to 46.4)
Guinea	2046	70.7 (55.3 to 89.6)	10.2 (7.5 to 13.5)	39.7 (26.9 to 56.7)	9.3 (4.1 to 18.9)	11.6 (8.1 to 18.2)	189.7 (148.3 to 240.4)	27.3 (20.2 to 36.3)	106.6 (72.2 to 152.0)	24.8 (11.0 to 50.7)	31.0 (21.8 to 48.8)
Guinea	2047	72.4 (57.0 to 92.3)	10.5 (7.7 to 13.9)	40.5 (27.5 to 57.9)	9.5 (4.2 to 19.4)	11.9 (8.4 to 19.1)	194.1 (153.0 to 247.4)	28.0 (20.8 to 37.4)	108.6 (73.7 to 155.2)	25.5 (11.3 to 52.1)	31.9 (22.4 to 51.2)
Guinea	2048	74.1 (57.8 to 94.4)	10.7 (7.9 to 14.4)	41.3 (28.0 to 59.1)	9.8 (4.3 to 20.0)	12.3 (8.6 to 20.1)	198.6 (154.9 to 253.3)	28.8 (21.3 to 38.5)	110.7 (75.0 to 158.4)	26.2 (11.6 to 53.6)	32.9 (23.0 to 53.8)
Guinea	2049	75.7 (59.5 to 97.8)	11.0 (8.1 to 14.7)	42.0 (28.5 to 60.3)	10.0 (4.5 to 20.5)	12.6 (8.8 to 20.7)	203.1 (159.4 to 262.4)	29.6 (21.8 to 39.5)	112.8 (76.3 to 161.6)	26.9 (12.0 to 55.0)	33.8 (23.5 to 55.5)
Guinea	2050	77.5 (60.1 to 99.9)	11.3 (8.3 to 15.1)	42.8 (29.0 to 61.5)	10.3 (4.6 to 21.0)	13.0 (8.8 to 21.6)	207.9 (161.3 to 267.9)	30.4 (22.4 to 40.5)	114.9 (77.8 to 164.9)	27.7 (12.3 to 56.4)	34.9 (23.6 to 58.0)

Country	Year	2018 US Dollars per capita					2018 PPP per capita				
		Health spending	Government health spending	Out-of-pocket health spending	Prepaid private health spending	Development assistance for health	Health spending	Government health spending	Out-of-pocket health spending	Prepaid private health spending	Development assistance for health
Guinea-Bissau	1995	56.5 (49.9 to 64.4)	14.0 (10.8 to 18.4)	17.9 (12.5 to 25.1)	0.0 (0.0 to 0.0)	24.5 (24.5 to 24.5)	126.5 (111.8 to 144.2)	31.4 (24.2 to 41.1)	40.1 (28.0 to 56.1)	0.0 (0.0 to 0.0)	54.9 (54.9 to 54.9)
Guinea-Bissau	1996	38.7 (32.1 to 46.7)	14.8 (11.4 to 19.3)	18.2 (12.6 to 25.6)	0.0 (0.0 to 0.0)	5.8 (5.8 to 5.8)	86.7 (71.9 to 104.5)	33.0 (25.4 to 43.3)	40.7 (28.3 to 57.4)	0.0 (0.0 to 0.0)	13.0 (13.0 to 13.0)
Guinea-Bissau	1997	41.8 (35.0 to 50.0)	16.1 (12.2 to 21.0)	18.5 (12.9 to 26.1)	0.0 (0.0 to 0.0)	7.2 (7.2 to 7.2)	93.7 (78.4 to 112.0)	36.0 (27.3 to 47.1)	41.4 (28.8 to 58.4)	0.0 (0.0 to 0.0)	16.2 (16.2 to 16.2)
Guinea-Bissau	1998	38.1 (32.0 to 45.2)	14.3 (10.8 to 18.6)	16.8 (11.7 to 23.5)	0.0 (0.0 to 0.0)	7.0 (7.0 to 7.0)	85.3 (71.6 to 101.3)	32.0 (24.2 to 41.6)	37.6 (26.2 to 52.5)	0.0 (0.0 to 0.0)	15.7 (15.7 to 15.7)
Guinea-Bissau	1999	34.7 (28.3 to 42.1)	16.3 (12.3 to 21.0)	17.3 (12.1 to 23.8)	0.0 (0.0 to 0.0)	1.1 (1.1 to 1.1)	77.6 (63.3 to 94.3)	36.5 (27.6 to 47.0)	38.7 (27.2 to 53.2)	0.0 (0.0 to 0.0)	2.4 (2.4 to 2.4)
Guinea-Bissau	2000	43.1 (36.2 to 51.2)	18.2 (13.7 to 23.2)	17.8 (12.6 to 24.3)	0.0 (0.0 to 0.0)	7.0 (7.0 to 7.0)	96.4 (81.1 to 114.7)	40.8 (30.7 to 51.9)	39.9 (28.2 to 54.3)	0.0 (0.0 to 0.0)	15.7 (15.7 to 15.7)
Guinea-Bissau	2001	42.2 (35.6 to 50.5)	18.2 (13.9 to 23.0)	17.4 (12.4 to 24.0)	0.0 (0.0 to 0.0)	6.7 (6.7 to 6.7)	94.6 (79.7 to 113.1)	40.7 (31.1 to 51.5)	38.9 (27.8 to 53.6)	0.0 (0.0 to 0.0)	15.0 (15.0 to 15.0)
Guinea-Bissau	2002	38.2 (32.1 to 45.5)	16.4 (12.7 to 20.9)	16.3 (11.7 to 22.6)	0.0 (0.0 to 0.0)	5.5 (5.5 to 5.5)	85.5 (71.9 to 102.0)	36.8 (28.4 to 46.8)	36.4 (26.2 to 50.7)	0.0 (0.0 to 0.0)	12.2 (12.2 to 12.2)
Guinea-Bissau	2003	39.0 (32.9 to 46.6)	17.1 (13.0 to 21.7)	16.1 (11.6 to 22.2)	0.0 (0.0 to 0.0)	5.8 (5.8 to 5.8)	87.2 (73.6 to 104.2)	38.3 (29.0 to 48.7)	36.0 (25.9 to 49.7)	0.0 (0.0 to 0.0)	13.0 (13.0 to 13.0)
Guinea-Bissau	2004	43.3 (37.1 to 51.1)	18.5 (14.0 to 23.6)	16.8 (12.1 to 23.2)	0.0 (0.0 to 0.0)	8.0 (8.0 to 8.0)	96.9 (83.0 to 114.4)	41.5 (31.4 to 52.9)	37.6 (27.1 to 52.0)	0.0 (0.0 to 0.0)	17.9 (17.9 to 17.9)
Guinea-Bissau	2005	44.4 (38.0 to 52.4)	18.2 (14.1 to 23.2)	17.3 (12.5 to 23.9)	0.0 (0.0 to 0.0)	8.9 (8.9 to 8.9)	99.4 (85.1 to 117.4)	40.8 (31.5 to 52.0)	38.7 (27.9 to 53.5)	0.0 (0.0 to 0.0)	19.9 (19.9 to 19.9)
Guinea-Bissau	2006	45.1 (38.5 to 53.3)	17.2 (13.3 to 21.9)	17.7 (12.8 to 24.5)	0.0 (0.0 to 0.0)	10.2 (10.2 to 10.2)	101.0 (86.2 to 119.3)	38.5 (29.8 to 49.0)	39.6 (28.7 to 54.8)	0.0 (0.0 to 0.0)	22.9 (22.9 to 22.9)
Guinea-Bissau	2007	48.6 (41.8 to 56.7)	16.5 (12.7 to 21.0)	18.1 (13.1 to 25.0)	0.0 (0.0 to 0.0)	14.0 (14.0 to 14.0)	108.9 (93.6 to 127.1)	36.9 (28.3 to 46.9)	40.5 (29.3 to 56.0)	0.0 (0.0 to 0.0)	31.4 (31.4 to 31.4)
Guinea-Bissau	2008	43.1 (36.2 to 51.1)	15.1 (11.6 to 19.3)	18.5 (13.1 to 25.7)	0.0 (0.0 to 0.0)	9.5 (9.5 to 9.5)	96.4 (81.0 to 114.4)	33.7 (26.0 to 43.3)	41.3 (29.3 to 57.5)	0.0 (0.0 to 0.0)	21.3 (21.3 to 21.3)
Guinea-Bissau	2009	42.2 (35.6 to 49.9)	13.2 (10.2 to 16.8)	18.4 (12.8 to 25.4)	0.0 (0.0 to 0.0)	10.6 (10.6 to 10.6)	94.4 (79.6 to 111.8)	29.5 (22.9 to 37.7)	41.2 (28.6 to 56.8)	0.0 (0.0 to 0.0)	23.8 (23.8 to 23.8)
Guinea-Bissau	2010	48.2 (42.3 to 55.6)	9.7 (7.6 to 12.4)	17.3 (11.9 to 24.0)	0.0 (0.0 to 0.0)	21.2 (21.2 to 21.2)	107.9 (94.7 to 124.4)	21.8 (17.0 to 27.7)	38.7 (26.6 to 53.8)	0.0 (0.0 to 0.0)	47.4 (47.4 to 47.4)
Guinea-Bissau	2011	34.7 (29.2 to 41.6)	8.1 (6.3 to 10.3)	16.3 (11.3 to 22.6)	0.0 (0.0 to 0.0)	10.4 (10.4 to 10.4)	77.8 (65.4 to 93.2)	18.2 (14.2 to 23.1)	36.4 (25.4 to 50.7)	0.0 (0.0 to 0.0)	23.2 (23.2 to 23.2)
Guinea-Bissau	2012	37.1 (31.8 to 44.2)	9.0 (7.0 to 11.6)	16.0 (11.0 to 22.3)	0.0 (0.0 to 0.0)	12.0 (12.0 to 12.0)	83.0 (71.2 to 99.0)	20.2 (15.7 to 25.9)	35.9 (24.7 to 49.9)	0.0 (0.0 to 0.0)	26.9 (26.9 to 26.9)
Guinea-Bissau	2013	50.5 (44.9 to 57.6)	11.0 (8.5 to 14.2)	16.2 (11.2 to 22.5)	0.0 (0.0 to 0.0)	23.3 (23.3 to 23.3)	113.1 (100.5 to 129.1)	24.7 (19.1 to 31.9)	36.3 (25.1 to 50.5)	0.0 (0.0 to 0.0)	52.1 (52.1 to 52.1)
Guinea-Bissau	2014	40.1 (34.2 to 47.8)	13.0 (10.1 to 16.9)	16.2 (11.5 to 22.6)	0.0 (0.0 to 0.0)	10.9 (10.9 to 10.9)	89.8 (76.7 to 107.0)	29.0 (22.6 to 37.9)	36.3 (25.8 to 50.7)	0.0 (0.0 to 0.0)	24.4 (24.4 to 24.4)
Guinea-Bissau	2015	46.8 (40.9 to 54.8)	14.8 (11.4 to 19.3)	16.4 (11.7 to 22.7)	0.0 (0.0 to 0.0)	15.7 (15.7 to 15.7)	104.8 (91.6 to 122.7)	33.1 (25.6 to 43.2)	36.6 (26.2 to 50.9)	0.0 (0.0 to 0.0)	35.1 (35.1 to 35.1)

		2018 US Dollars per capita					2018 PPP per capita				
Country	Year	Health spending	Government health spending	Out-of-pocket health spending	Prepaid private health spending	Development assistance for health	Health spending	Government health spending	Out-of-pocket health spending	Prepaid private health spending	Development assistance for health
Guinea-Bissau	2016	49.1 (42.5 to 57.0)	16.9 (13.0 to 21.9)	16.7 (12.0 to 23.1)	0.0 (0.0 to 0.0)	15.5 (15.5 to 15.5)	110.0 (95.3 to 127.6)	37.8 (29.0 to 49.0)	37.5 (26.9 to 51.8)	0.0 (0.0 to 0.0)	34.7 (34.7 to 34.7)
Guinea-Bissau	2017	46.7 (40.4 to 54.9)	16.3 (12.4 to 21.0)	17.1 (12.2 to 23.6)	0.0 (0.0 to 0.0)	13.4 (13.4 to 13.4)	104.6 (90.5 to 123.0)	36.4 (27.9 to 47.1)	38.2 (27.4 to 52.7)	0.0 (0.0 to 0.0)	29.9 (29.9 to 29.9)
Guinea-Bissau	2018	46.4 (39.8 to 54.5)	16.8 (12.8 to 21.6)	17.2 (12.4 to 23.7)	0.0 (0.0 to 0.0)	12.4 (12.4 to 12.5)	104.0 (89.2 to 122.1)	37.5 (28.7 to 48.5)	38.6 (27.7 to 53.1)	0.0 (0.0 to 0.0)	27.9 (27.8 to 27.9)
Guinea-Bissau	2019	46.9 (40.5 to 55.1)	17.1 (13.1 to 22.0)	17.4 (12.4 to 24.0)	0.0 (0.0 to 0.0)	12.4 (11.5 to 13.1)	104.9 (90.7 to 123.5)	38.3 (29.3 to 49.4)	38.9 (27.7 to 53.6)	0.0 (0.0 to 0.0)	27.7 (25.8 to 29.3)
Guinea-Bissau	2020	47.3 (40.0 to 56.0)	17.4 (13.3 to 22.5)	17.5 (12.5 to 24.1)	0.0 (0.0 to 0.0)	12.4 (11.4 to 13.4)	106.0 (89.5 to 125.3)	39.0 (29.7 to 50.3)	39.2 (27.9 to 53.9)	0.0 (0.0 to 0.0)	27.7 (25.4 to 30.0)
Guinea-Bissau	2021	47.9 (41.0 to 55.7)	17.8 (13.6 to 22.8)	17.6 (12.5 to 24.4)	0.0 (0.0 to 0.0)	12.5 (11.2 to 13.8)	107.1 (91.8 to 124.6)	39.8 (30.4 to 51.1)	39.5 (27.9 to 54.6)	0.0 (0.0 to 0.0)	27.9 (25.1 to 30.8)
Guinea-Bissau	2022	48.4 (41.4 to 57.1)	18.1 (13.8 to 23.3)	17.8 (12.6 to 24.5)	0.0 (0.0 to 0.0)	12.6 (11.2 to 14.1)	108.4 (92.6 to 127.7)	40.5 (30.9 to 52.1)	39.8 (28.2 to 54.9)	0.0 (0.0 to 0.0)	28.2 (25.0 to 31.5)
Guinea-Bissau	2023	49.0 (41.8 to 57.9)	18.3 (14.0 to 23.7)	17.9 (12.7 to 24.5)	0.0 (0.0 to 0.0)	12.7 (11.1 to 14.4)	109.6 (93.7 to 129.6)	41.1 (31.4 to 53.0)	40.1 (28.3 to 55.0)	0.0 (0.0 to 0.0)	28.5 (24.8 to 32.3)
Guinea-Bissau	2024	49.5 (42.1 to 58.5)	18.6 (14.2 to 24.1)	18.0 (12.7 to 24.7)	0.0 (0.0 to 0.0)	12.9 (11.1 to 14.8)	111.0 (94.2 to 131.0)	41.7 (31.8 to 54.0)	40.4 (28.5 to 55.3)	0.0 (0.0 to 0.0)	28.8 (24.9 to 33.2)
Guinea-Bissau	2025	50.1 (42.9 to 58.9)	18.9 (14.4 to 24.4)	18.2 (12.8 to 24.9)	0.0 (0.0 to 0.0)	13.0 (11.1 to 15.2)	112.2 (96.1 to 131.8)	42.4 (32.2 to 54.7)	40.7 (28.8 to 55.8)	0.0 (0.0 to 0.0)	29.1 (24.9 to 34.1)
Guinea-Bissau	2026	50.7 (42.9 to 59.7)	19.2 (14.6 to 24.9)	18.3 (13.0 to 25.0)	0.0 (0.0 to 0.0)	13.1 (11.1 to 15.4)	113.5 (96.0 to 133.8)	43.1 (32.8 to 55.7)	41.0 (29.1 to 56.1)	0.0 (0.0 to 0.0)	29.4 (24.8 to 34.5)
Guinea-Bissau	2027	51.3 (44.0 to 61.2)	19.5 (14.9 to 25.3)	18.5 (13.1 to 25.3)	0.0 (0.0 to 0.0)	13.3 (11.2 to 15.8)	114.9 (98.5 to 137.0)	43.8 (33.3 to 56.6)	41.4 (29.4 to 56.8)	0.0 (0.0 to 0.0)	29.8 (25.0 to 35.4)
Guinea-Bissau	2028	52.0 (43.9 to 62.1)	19.9 (15.1 to 25.7)	18.6 (13.2 to 25.5)	0.0 (0.0 to 0.0)	13.5 (11.3 to 16.3)	116.4 (98.3 to 139.1)	44.5 (33.8 to 57.5)	41.7 (29.6 to 57.1)	0.0 (0.0 to 0.0)	30.2 (25.3 to 36.4)
Guinea-Bissau	2029	52.7 (44.9 to 62.8)	20.2 (15.4 to 26.1)	18.8 (13.4 to 25.8)	0.0 (0.0 to 0.0)	13.7 (11.3 to 16.7)	118.0 (100.6 to 140.5)	45.3 (34.4 to 58.4)	42.1 (29.9 to 57.8)	0.0 (0.0 to 0.0)	30.6 (25.2 to 37.5)
Guinea-Bissau	2030	53.4 (45.0 to 63.4)	20.6 (15.6 to 26.6)	19.0 (13.5 to 26.2)	0.0 (0.0 to 0.0)	13.8 (11.3 to 17.0)	119.5 (100.8 to 142.1)	46.0 (34.9 to 59.5)	42.5 (30.2 to 58.6)	0.0 (0.0 to 0.0)	31.0 (25.4 to 38.0)
Guinea-Bissau	2031	54.1 (46.3 to 64.2)	20.9 (15.9 to 27.2)	19.2 (13.6 to 26.6)	0.0 (0.0 to 0.0)	14.0 (11.4 to 17.6)	121.2 (103.8 to 143.8)	46.9 (35.6 to 60.8)	43.0 (30.5 to 59.5)	0.0 (0.0 to 0.0)	31.4 (25.4 to 39.4)
Guinea-Bissau	2032	54.9 (45.8 to 65.7)	21.3 (16.2 to 27.5)	19.4 (13.7 to 27.0)	0.0 (0.0 to 0.0)	14.2 (11.4 to 17.9)	123.0 (102.6 to 147.0)	47.7 (36.3 to 61.7)	43.4 (30.6 to 60.4)	0.0 (0.0 to 0.0)	31.9 (25.6 to 40.1)
Guinea-Bissau	2033	55.7 (46.9 to 66.8)	21.7 (16.4 to 28.1)	19.6 (13.8 to 27.3)	0.0 (0.0 to 0.0)	14.4 (11.5 to 18.5)	124.7 (105.1 to 149.6)	48.5 (36.7 to 62.9)	43.9 (30.8 to 61.2)	0.0 (0.0 to 0.0)	32.3 (25.7 to 41.5)
Guinea-Bissau	2034	56.6 (47.0 to 67.7)	22.1 (16.7 to 28.7)	19.8 (13.9 to 27.7)	0.0 (0.0 to 0.0)	14.7 (11.6 to 19.0)	126.7 (105.3 to 151.5)	49.4 (37.5 to 64.2)	44.3 (31.2 to 61.9)	0.0 (0.0 to 0.0)	32.9 (25.9 to 42.4)
Guinea-Bissau	2035	57.5 (48.4 to 68.6)	22.5 (17.0 to 29.2)	20.0 (14.1 to 28.0)	0.0 (0.0 to 0.0)	14.9 (11.6 to 19.5)	128.7 (108.5 to 153.7)	50.4 (38.1 to 65.3)	44.8 (31.5 to 62.7)	0.0 (0.0 to 0.0)	33.5 (26.0 to 43.7)
Guinea-Bissau	2036	58.4 (48.7 to 70.1)	23.0 (17.3 to 29.8)	20.2 (14.2 to 28.3)	0.0 (0.0 to 0.0)	15.2 (11.8 to 20.4)	130.8 (109.0 to 156.9)	51.4 (38.6 to 66.8)	45.3 (31.8 to 63.5)	0.0 (0.0 to 0.0)	34.0 (26.3 to 45.7)

		2018 US Dollars per capita					2018 PPP per capita				
Country	Year	Health spending	Government health spending	Out-of-pocket health spending	Prepaid private health spending	Development assistance for health	Health spending	Government health spending	Out-of-pocket health spending	Prepaid private health spending	Development assistance for health
Guinea-Bissau	2037	59.4 (49.3 to 71.1)	23.5 (17.6 to 30.4)	20.5 (14.3 to 28.8)	0.0 (0.0 to 0.0)	15.4 (11.9 to 20.7)	132.9 (110.5 to 159.3)	52.5 (39.5 to 68.1)	45.8 (32.1 to 64.4)	0.0 (0.0 to 0.0)	34.6 (26.6 to 46.4)
Guinea-Bissau	2038	60.4 (50.1 to 71.8)	24.0 (17.9 to 31.2)	20.7 (14.5 to 29.2)	0.0 (0.0 to 0.0)	15.7 (12.0 to 21.9)	135.2 (112.1 to 160.7)	53.7 (40.2 to 69.9)	46.4 (32.4 to 65.3)	0.0 (0.0 to 0.0)	35.2 (26.8 to 49.0)
Guinea-Bissau	2039	61.4 (51.5 to 74.1)	24.5 (18.2 to 31.8)	20.9 (14.6 to 29.6)	0.0 (0.0 to 0.0)	16.0 (12.2 to 22.2)	137.5 (115.3 to 166.0)	54.8 (40.8 to 71.2)	46.9 (32.8 to 66.2)	0.0 (0.0 to 0.0)	35.8 (27.3 to 49.7)
Guinea-Bissau	2040	62.5 (51.8 to 75.3)	25.0 (18.6 to 32.5)	21.2 (14.8 to 30.0)	0.0 (0.0 to 0.0)	16.3 (12.4 to 22.6)	139.9 (116.1 to 168.7)	56.0 (41.7 to 72.9)	47.4 (33.1 to 67.1)	0.0 (0.0 to 0.0)	36.5 (27.7 to 50.5)
Guinea-Bissau	2041	63.6 (52.9 to 77.3)	25.5 (19.1 to 33.3)	21.4 (14.9 to 30.4)	0.0 (0.0 to 0.0)	16.6 (12.5 to 23.0)	142.3 (118.6 to 173.0)	57.2 (42.7 to 74.6)	48.0 (33.3 to 68.1)	0.0 (0.0 to 0.0)	37.2 (27.9 to 51.6)
Guinea-Bissau	2042	64.7 (53.6 to 78.1)	26.1 (19.5 to 34.1)	21.7 (15.0 to 30.8)	0.0 (0.0 to 0.0)	16.9 (12.7 to 24.1)	144.8 (120.1 to 175.0)	58.4 (43.6 to 76.3)	48.5 (33.6 to 69.0)	0.0 (0.0 to 0.0)	38.0 (28.4 to 54.0)
Guinea-Bissau	2043	65.8 (54.8 to 78.5)	26.6 (19.8 to 34.9)	21.9 (15.2 to 31.2)	0.0 (0.0 to 0.0)	17.3 (12.8 to 25.0)	147.4 (122.7 to 175.8)	59.6 (44.4 to 78.3)	49.1 (34.0 to 69.8)	0.0 (0.0 to 0.0)	38.7 (28.7 to 55.9)
Guinea-Bissau	2044	67.0 (55.5 to 81.3)	27.2 (20.2 to 35.8)	22.1 (15.4 to 31.6)	0.0 (0.0 to 0.0)	17.7 (13.0 to 25.5)	150.0 (124.3 to 182.1)	60.8 (45.2 to 80.1)	49.6 (34.5 to 70.7)	0.0 (0.0 to 0.0)	39.5 (29.1 to 57.2)
Guinea-Bissau	2045	68.2 (56.8 to 83.5)	27.8 (20.6 to 36.7)	22.4 (15.6 to 32.0)	0.0 (0.0 to 0.0)	18.0 (13.0 to 27.2)	152.8 (127.3 to 187.0)	62.2 (46.1 to 82.1)	50.2 (35.0 to 71.6)	0.0 (0.0 to 0.0)	40.4 (29.1 to 61.0)
Guinea-Bissau	2046	69.5 (57.1 to 84.6)	28.4 (20.9 to 37.5)	22.6 (15.8 to 32.3)	0.0 (0.0 to 0.0)	18.4 (13.3 to 28.4)	155.6 (127.9 to 189.5)	63.6 (46.8 to 83.9)	50.7 (35.4 to 72.4)	0.0 (0.0 to 0.0)	41.3 (29.8 to 63.6)
Guinea-Bissau	2047	70.8 (58.4 to 88.4)	29.1 (21.4 to 38.4)	22.9 (16.0 to 32.8)	0.0 (0.0 to 0.0)	18.9 (13.5 to 29.7)	158.6 (130.7 to 197.9)	65.1 (47.9 to 86.0)	51.3 (35.8 to 73.4)	0.0 (0.0 to 0.0)	42.2 (30.3 to 66.5)
Guinea-Bissau	2048	72.2 (59.7 to 89.3)	29.8 (21.9 to 39.2)	23.2 (16.1 to 33.1)	0.0 (0.0 to 0.0)	19.3 (13.8 to 30.9)	161.8 (133.6 to 200.0)	66.6 (49.1 to 87.9)	51.9 (36.1 to 74.2)	0.0 (0.0 to 0.0)	43.3 (30.8 to 69.3)
Guinea-Bissau	2049	73.7 (60.3 to 89.9)	30.5 (22.5 to 40.3)	23.4 (16.3 to 33.4)	0.0 (0.0 to 0.0)	19.8 (14.1 to 31.8)	165.0 (135.1 to 201.2)	68.3 (50.4 to 90.2)	52.4 (36.5 to 74.8)	0.0 (0.0 to 0.0)	44.2 (31.6 to 71.2)
Guinea-Bissau	2050	75.2 (61.4 to 92.9)	31.2 (23.0 to 41.3)	23.7 (16.5 to 33.7)	0.0 (0.0 to 0.0)	20.3 (14.2 to 33.0)	168.4 (137.5 to 208.1)	69.9 (51.6 to 92.6)	53.0 (36.9 to 75.5)	0.0 (0.0 to 0.0)	45.4 (31.8 to 74.0)
Guyana	1995	111.4 (94.4 to 129.9)	84.6 (69.2 to 102.1)	19.3 (14.1 to 25.7)	0.1 (0.1 to 0.2)	7.3 (7.3 to 7.3)	202.0 (171.3 to 235.7)	153.5 (125.5 to 185.3)	35.0 (25.5 to 46.7)	0.2 (0.1 to 0.4)	13.2 (13.2 to 13.2)
Guyana	1996	114.1 (97.9 to 132.4)	86.9 (72.3 to 104.1)	20.0 (14.7 to 26.7)	0.1 (0.1 to 0.2)	7.1 (7.1 to 7.1)	207.0 (177.5 to 240.2)	157.7 (131.2 to 188.8)	36.2 (26.6 to 48.5)	0.2 (0.1 to 0.4)	12.8 (12.8 to 12.8)
Guyana	1997	118.0 (102.2 to 134.9)	88.4 (74.5 to 104.4)	20.6 (15.2 to 27.2)	0.1 (0.1 to 0.2)	8.9 (8.9 to 8.9)	214.0 (185.3 to 244.7)	160.3 (135.1 to 189.3)	37.3 (27.7 to 49.4)	0.2 (0.1 to 0.4)	16.1 (16.1 to 16.1)
Guyana	1998	114.3 (99.7 to 130.4)	87.0 (73.6 to 102.3)	20.8 (15.5 to 27.5)	0.1 (0.1 to 0.2)	6.3 (6.3 to 6.3)	207.3 (180.8 to 236.5)	157.9 (133.6 to 185.6)	37.7 (28.1 to 49.9)	0.2 (0.1 to 0.4)	11.4 (11.4 to 11.4)
Guyana	1999	117.7 (102.3 to 134.0)	90.7 (76.6 to 105.7)	21.1 (15.8 to 27.4)	0.1 (0.1 to 0.2)	5.8 (5.8 to 5.8)	213.5 (185.6 to 243.1)	164.5 (139.0 to 191.7)	38.2 (28.7 to 49.7)	0.2 (0.1 to 0.4)	10.6 (10.6 to 10.6)
Guyana	2000	117.0 (101.6 to 133.8)	93.4 (79.1 to 109.0)	21.6 (16.2 to 28.0)	0.1 (0.0 to 0.2)	1.9 (1.9 to 1.9)	212.3 (184.3 to 242.6)	169.4 (143.5 to 197.6)	39.2 (29.5 to 50.8)	0.2 (0.1 to 0.3)	3.5 (3.5 to 3.5)
Guyana	2001	128.2 (112.3 to 145.7)	99.8 (84.5 to 116.9)	23.2 (17.4 to 29.8)	0.1 (0.0 to 0.2)	5.2 (5.2 to 5.2)	232.6 (203.8 to 264.2)	181.0 (153.3 to 212.1)	42.1 (31.5 to 54.1)	0.2 (0.1 to 0.3)	9.4 (9.4 to 9.4)

		2018 US Dollars per capita					2018 PPP per capita				
Country	Year	Health spending	Government health spending	Out-of-pocket health spending	Prepaid private health spending	Development assistance for health	Health spending	Government health spending	Out-of-pocket health spending	Prepaid private health spending	Development assistance for health
Guyana	2002	134.8 (118.1 to 152.6)	104.9 (89.2 to 121.6)	25.4 (18.9 to 32.4)	0.1 (0.0 to 0.2)	4.5 (4.5 to 4.5)	244.6 (214.2 to 276.7)	190.3 (161.9 to 220.5)	46.0 (34.4 to 58.7)	0.2 (0.1 to 0.3)	8.1 (8.1 to 8.1)
Guyana	2003	152.6 (134.3 to 170.8)	103.9 (87.7 to 120.6)	29.5 (22.2 to 37.7)	0.1 (0.0 to 0.2)	19.2 (19.2 to 19.2)	276.9 (243.6 to 309.8)	188.4 (159.1 to 218.8)	53.5 (40.3 to 68.4)	0.2 (0.1 to 0.3)	34.9 (34.9 to 34.9)
Guyana	2004	174.7 (156.2 to 192.8)	100.8 (85.5 to 117.9)	33.5 (25.8 to 42.7)	0.1 (0.0 to 0.2)	40.2 (40.2 to 40.2)	316.8 (283.2 to 349.7)	182.9 (155.0 to 213.8)	60.8 (46.7 to 77.4)	0.2 (0.1 to 0.3)	73.0 (73.0 to 73.0)
Guyana	2005	160.7 (143.4 to 178.0)	87.7 (74.1 to 103.1)	38.1 (29.5 to 47.8)	0.1 (0.1 to 0.2)	34.9 (34.9 to 34.9)	291.6 (260.2 to 322.9)	159.0 (134.3 to 187.1)	69.0 (53.6 to 86.7)	0.2 (0.1 to 0.4)	63.3 (63.3 to 63.3)
Guyana	2006	160.5 (145.1 to 176.7)	70.8 (59.3 to 83.4)	42.7 (33.5 to 53.6)	0.1 (0.1 to 0.2)	46.8 (46.8 to 46.8)	291.1 (263.2 to 320.4)	128.4 (107.6 to 151.2)	77.5 (60.7 to 97.2)	0.2 (0.1 to 0.4)	85.0 (85.0 to 85.0)
Guyana	2007	154.8 (139.5 to 171.0)	64.4 (53.7 to 76.3)	48.9 (39.0 to 60.9)	0.1 (0.1 to 0.2)	41.4 (41.4 to 41.4)	280.9 (253.0 to 310.2)	116.7 (97.3 to 138.4)	88.8 (70.8 to 110.5)	0.2 (0.1 to 0.4)	75.2 (75.2 to 75.2)
Guyana	2008	166.8 (151.5 to 183.2)	59.8 (49.5 to 71.1)	54.2 (43.6 to 67.2)	0.1 (0.1 to 0.2)	52.7 (52.7 to 52.7)	302.6 (274.9 to 332.3)	108.4 (89.8 to 129.0)	98.3 (79.0 to 121.9)	0.2 (0.1 to 0.4)	95.7 (95.7 to 95.7)
Guyana	2009	165.7 (150.3 to 183.6)	60.3 (50.2 to 71.5)	60.6 (49.0 to 74.8)	0.1 (0.1 to 0.2)	44.7 (44.7 to 44.7)	300.6 (272.6 to 333.1)	109.4 (91.1 to 129.6)	110.0 (88.9 to 135.7)	0.2 (0.1 to 0.4)	81.0 (81.0 to 81.0)
Guyana	2010	174.1 (157.1 to 194.2)	65.0 (53.9 to 77.1)	67.7 (55.0 to 83.4)	0.1 (0.1 to 0.3)	41.3 (41.3 to 41.3)	315.7 (285.0 to 352.2)	117.8 (97.8 to 139.8)	122.7 (99.7 to 151.2)	0.3 (0.1 to 0.5)	74.9 (74.9 to 74.9)
Guyana	2011	180.7 (161.9 to 202.4)	73.1 (60.7 to 86.3)	74.5 (60.2 to 92.0)	0.2 (0.1 to 0.3)	33.0 (33.0 to 33.0)	327.8 (293.6 to 367.1)	132.6 (110.2 to 156.5)	135.0 (109.2 to 166.9)	0.3 (0.1 to 0.5)	59.9 (59.9 to 59.9)
Guyana	2012	188.4 (167.2 to 211.2)	83.4 (69.9 to 98.0)	80.0 (65.3 to 98.5)	0.2 (0.1 to 0.3)	24.8 (24.8 to 24.8)	341.7 (303.2 to 383.0)	151.3 (126.7 to 177.7)	145.1 (118.5 to 178.7)	0.3 (0.1 to 0.6)	45.0 (45.0 to 45.0)
Guyana	2013	199.8 (177.3 to 226.2)	96.1 (80.4 to 112.7)	84.7 (69.3 to 104.8)	0.2 (0.1 to 0.3)	18.8 (18.8 to 18.8)	362.4 (321.6 to 410.3)	174.3 (145.8 to 204.3)	153.7 (125.6 to 190.2)	0.3 (0.1 to 0.6)	34.1 (34.1 to 34.1)
Guyana	2014	207.4 (182.9 to 235.0)	106.4 (89.1 to 125.6)	85.7 (69.3 to 107.2)	0.2 (0.1 to 0.3)	15.2 (15.2 to 15.2)	376.2 (331.8 to 426.3)	192.9 (161.7 to 227.8)	155.4 (125.8 to 194.4)	0.3 (0.2 to 0.6)	27.6 (27.6 to 27.6)
Guyana	2015	203.9 (178.9 to 233.3)	110.2 (91.1 to 131.2)	82.9 (66.1 to 103.5)	0.2 (0.1 to 0.3)	10.7 (10.7 to 10.7)	369.9 (324.6 to 423.1)	199.8 (165.2 to 237.9)	150.3 (119.9 to 187.7)	0.3 (0.1 to 0.6)	19.5 (19.5 to 19.5)
Guyana	2016	208.0 (180.3 to 239.3)	117.8 (97.1 to 142.1)	80.1 (62.4 to 102.3)	0.2 (0.1 to 0.4)	10.0 (10.0 to 10.0)	377.3 (327.0 to 434.1)	213.7 (176.1 to 257.7)	145.2 (113.2 to 185.5)	0.4 (0.2 to 0.7)	18.1 (18.1 to 18.1)
Guyana	2017	219.4 (189.8 to 255.3)	126.2 (104.0 to 151.9)	82.8 (64.6 to 105.0)	0.2 (0.1 to 0.4)	10.2 (10.2 to 10.2)	397.9 (344.3 to 463.0)	228.8 (188.7 to 275.5)	150.2 (117.2 to 190.5)	0.4 (0.2 to 0.7)	18.5 (18.5 to 18.5)
Guyana	2018	225.2 (194.1 to 259.0)	129.5 (106.8 to 155.9)	85.8 (66.7 to 108.2)	0.2 (0.1 to 0.4)	9.6 (9.6 to 9.6)	408.4 (352.1 to 469.8)	235.0 (193.7 to 282.7)	155.6 (120.9 to 196.2)	0.4 (0.2 to 0.7)	17.5 (17.4 to 17.5)
Guyana	2019	232.0 (200.7 to 268.3)	133.2 (109.7 to 160.5)	88.8 (69.1 to 112.4)	0.2 (0.1 to 0.4)	9.7 (9.1 to 10.3)	420.8 (364.1 to 486.6)	241.7 (198.9 to 291.2)	161.1 (125.4 to 203.9)	0.4 (0.2 to 0.8)	17.7 (16.5 to 18.7)
Guyana	2020	238.7 (205.2 to 276.6)	136.7 (112.2 to 165.5)	91.9 (72.1 to 116.8)	0.2 (0.1 to 0.4)	9.9 (9.1 to 10.7)	433.0 (372.2 to 501.7)	247.9 (203.5 to 300.1)	166.8 (130.7 to 211.8)	0.4 (0.2 to 0.8)	18.0 (16.4 to 19.4)
Guyana	2021	247.0 (212.1 to 284.7)	141.3 (115.9 to 171.0)	95.4 (74.3 to 121.1)	0.2 (0.1 to 0.4)	10.1 (9.1 to 11.2)	448.0 (384.7 to 516.4)	256.3 (210.3 to 310.2)	173.0 (134.8 to 219.7)	0.4 (0.2 to 0.8)	18.3 (16.5 to 20.2)
Guyana	2022	255.1 (219.3 to 292.0)	145.7 (119.7 to 175.3)	98.8 (76.8 to 125.1)	0.2 (0.1 to 0.4)	10.3 (9.2 to 11.6)	462.7 (397.8 to 529.6)	264.3 (217.1 to 318.0)	179.2 (139.3 to 226.9)	0.4 (0.2 to 0.8)	18.7 (16.6 to 21.0)

		2018 US Dollars per capita					2018 PPP per capita				
Country	Year	Health spending	Government health spending	Out-of-pocket health spending	Prepaid private health spending	Development assistance for health	Health spending	Government health spending	Out-of-pocket health spending	Prepaid private health spending	Development assistance for health
Guyana	2023	263.3 (224.3 to 301.3)	150.2 (123.1 to 180.1)	102.3 (79.6 to 129.9)	0.2 (0.1 to 0.5)	10.6 (9.2 to 12.0)	477.6 (406.9 to 546.6)	272.5 (223.3 to 326.8)	185.5 (144.5 to 235.6)	0.4 (0.2 to 0.8)	19.2 (16.7 to 21.8)
Guyana	2024	271.5 (235.2 to 312.6)	154.5 (127.0 to 185.0)	105.8 (82.2 to 134.5)	0.3 (0.1 to 0.5)	10.8 (9.4 to 12.5)	492.4 (426.6 to 567.0)	280.3 (230.3 to 335.6)	192.0 (149.2 to 244.0)	0.5 (0.2 to 0.9)	19.7 (17.0 to 22.6)
Guyana	2025	279.4 (241.9 to 325.8)	158.6 (130.6 to 190.1)	109.4 (84.8 to 139.2)	0.3 (0.1 to 0.5)	11.1 (9.5 to 13.0)	506.8 (438.8 to 591.0)	287.8 (236.9 to 344.8)	198.5 (153.9 to 252.4)	0.5 (0.2 to 0.9)	20.1 (17.2 to 23.6)
Guyana	2026	287.8 (248.0 to 332.7)	163.0 (133.8 to 195.5)	113.2 (87.2 to 143.6)	0.3 (0.1 to 0.5)	11.4 (9.6 to 13.3)	522.0 (449.9 to 603.4)	295.6 (242.7 to 354.5)	205.3 (158.3 to 260.5)	0.5 (0.2 to 0.9)	20.6 (17.4 to 24.2)
Guyana	2027	295.8 (255.4 to 341.8)	167.0 (137.0 to 200.7)	116.9 (89.8 to 148.4)	0.3 (0.1 to 0.5)	11.7 (9.8 to 13.8)	536.6 (463.3 to 620.0)	302.9 (248.5 to 364.0)	212.1 (162.9 to 269.2)	0.5 (0.2 to 0.9)	21.1 (17.7 to 25.1)
Guyana	2028	304.1 (261.7 to 353.7)	171.1 (140.4 to 206.1)	120.7 (92.4 to 155.0)	0.3 (0.1 to 0.5)	12.0 (10.0 to 14.5)	551.6 (474.6 to 641.5)	310.4 (254.7 to 373.8)	219.0 (167.6 to 281.1)	0.5 (0.2 to 0.9)	21.7 (18.2 to 26.2)
Guyana	2029	312.2 (267.9 to 362.0)	175.2 (143.4 to 210.6)	124.5 (95.0 to 161.5)	0.3 (0.1 to 0.5)	12.3 (10.1 to 15.0)	566.4 (486.0 to 656.6)	317.8 (260.2 to 382.0)	225.8 (172.3 to 293.0)	0.5 (0.2 to 1.0)	22.3 (18.4 to 27.2)
Guyana	2030	320.3 (277.1 to 373.6)	179.1 (146.4 to 215.9)	128.3 (97.5 to 167.1)	0.3 (0.1 to 0.5)	12.6 (10.3 to 15.5)	581.0 (502.6 to 677.7)	324.9 (265.5 to 391.6)	232.6 (176.8 to 303.0)	0.5 (0.2 to 1.0)	22.9 (18.7 to 28.1)
Guyana	2031	329.0 (282.5 to 384.7)	183.6 (150.0 to 221.4)	132.1 (99.9 to 173.0)	0.3 (0.1 to 0.6)	13.0 (10.5 to 16.3)	596.8 (512.4 to 697.8)	333.1 (272.1 to 401.6)	239.7 (181.2 to 313.8)	0.5 (0.3 to 1.0)	23.5 (19.0 to 29.6)
Guyana	2032	337.6 (289.4 to 393.3)	187.9 (153.4 to 226.6)	136.0 (102.1 to 179.1)	0.3 (0.1 to 0.6)	13.3 (10.7 to 16.8)	612.3 (524.9 to 713.4)	340.9 (278.3 to 411.1)	246.7 (185.2 to 324.9)	0.5 (0.3 to 1.0)	24.2 (19.4 to 30.5)
Guyana	2033	346.1 (296.9 to 404.2)	192.3 (157.0 to 232.5)	139.8 (104.3 to 184.7)	0.3 (0.1 to 0.6)	13.7 (10.9 to 17.6)	627.8 (538.5 to 733.2)	348.7 (284.8 to 421.8)	253.6 (189.2 to 335.1)	0.6 (0.3 to 1.0)	24.9 (19.7 to 31.9)
Guyana	2034	354.7 (302.6 to 415.4)	196.6 (159.8 to 238.6)	143.7 (106.5 to 189.8)	0.3 (0.2 to 0.6)	14.1 (11.1 to 18.3)	643.4 (548.8 to 753.5)	356.6 (289.8 to 432.7)	260.6 (193.2 to 344.3)	0.6 (0.3 to 1.1)	25.7 (20.2 to 33.1)
Guyana	2035	363.2 (311.7 to 426.1)	200.9 (163.4 to 244.1)	147.4 (108.6 to 194.7)	0.3 (0.2 to 0.6)	14.6 (11.3 to 19.0)	658.8 (565.3 to 773.0)	364.4 (296.5 to 442.7)	267.4 (197.1 to 353.1)	0.6 (0.3 to 1.1)	26.4 (20.5 to 34.5)
Guyana	2036	372.2 (318.2 to 434.1)	205.5 (167.3 to 250.0)	151.3 (110.4 to 200.6)	0.3 (0.2 to 0.6)	15.0 (11.6 to 20.2)	675.0 (577.2 to 787.4)	372.8 (303.5 to 453.5)	274.4 (200.3 to 363.9)	0.6 (0.3 to 1.1)	27.2 (21.1 to 36.7)
Guyana	2037	381.2 (323.7 to 446.7)	210.3 (170.9 to 256.0)	155.1 (112.8 to 205.7)	0.3 (0.2 to 0.6)	15.5 (11.9 to 20.8)	691.5 (587.2 to 810.3)	381.5 (310.1 to 464.3)	281.3 (204.7 to 373.2)	0.6 (0.3 to 1.1)	28.0 (21.5 to 37.7)
Guyana	2038	390.3 (332.6 to 460.2)	215.1 (174.2 to 262.4)	158.9 (115.1 to 210.8)	0.3 (0.2 to 0.6)	15.9 (12.1 to 22.2)	707.9 (603.2 to 834.7)	390.2 (316.0 to 475.9)	288.2 (208.8 to 382.3)	0.6 (0.3 to 1.2)	28.9 (22.0 to 40.2)
Guyana	2039	399.1 (341.4 to 470.1)	219.8 (178.2 to 268.8)	162.6 (117.3 to 216.2)	0.4 (0.2 to 0.7)	16.4 (12.6 to 22.7)	724.0 (619.2 to 852.7)	398.7 (323.2 to 487.6)	294.9 (212.7 to 392.1)	0.6 (0.3 to 1.2)	29.8 (22.8 to 41.3)
Guyana	2040	407.9 (346.6 to 484.1)	224.5 (180.4 to 275.5)	166.2 (119.7 to 221.6)	0.4 (0.2 to 0.7)	16.9 (12.8 to 23.5)	739.9 (628.7 to 878.1)	407.1 (327.3 to 499.8)	301.4 (217.2 to 401.9)	0.7 (0.3 to 1.2)	30.7 (23.3 to 42.6)
Guyana	2041	417.0 (353.3 to 494.6)	229.4 (184.3 to 281.5)	169.8 (121.6 to 226.4)	0.4 (0.2 to 0.7)	17.5 (13.1 to 24.3)	756.4 (640.8 to 897.1)	416.1 (334.4 to 510.7)	307.9 (220.6 to 410.6)	0.7 (0.3 to 1.3)	31.7 (23.8 to 44.0)
Guyana	2042	426.0 (363.2 to 508.7)	234.3 (188.0 to 288.0)	173.3 (123.4 to 231.0)	0.4 (0.2 to 0.7)	18.0 (13.4 to 25.7)	772.7 (658.9 to 922.8)	425.0 (341.0 to 522.4)	314.3 (223.9 to 419.0)	0.7 (0.3 to 1.3)	32.7 (24.4 to 46.7)
Guyana	2043	434.9 (366.0 to 517.8)	239.2 (191.0 to 295.3)	176.7 (125.6 to 236.0)	0.4 (0.2 to 0.7)	18.6 (13.7 to 26.8)	788.8 (663.9 to 939.2)	433.8 (346.5 to 535.7)	320.5 (227.9 to 428.0)	0.7 (0.3 to 1.3)	33.8 (24.9 to 48.6)

Country	Year	2018 US Dollars per capita					2018 PPP per capita				
		Health spending	Government health spending	Out-of-pocket health spending	Prepaid private health spending	Development assistance for health	Health spending	Government health spending	Out-of-pocket health spending	Prepaid private health spending	Development assistance for health
Guyana	2044	443.7 (374.3 to 529.9)	244.0 (194.7 to 303.1)	180.0 (127.8 to 240.7)	0.4 (0.2 to 0.7)	19.2 (14.1 to 27.8)	804.8 (679.0 to 961.2)	442.7 (353.2 to 549.7)	326.5 (231.8 to 436.7)	0.7 (0.4 to 1.3)	34.9 (25.6 to 50.4)
Guyana	2045	452.0 (380.2 to 538.9)	248.6 (197.9 to 308.5)	183.2 (129.9 to 245.2)	0.4 (0.2 to 0.8)	19.9 (14.3 to 30.0)	819.9 (689.7 to 977.4)	450.9 (358.9 to 559.6)	332.3 (235.7 to 444.7)	0.7 (0.4 to 1.4)	36.0 (26.0 to 54.4)
Guyana	2046	460.9 (390.2 to 546.8)	253.6 (202.2 to 315.1)	186.4 (132.0 to 249.6)	0.4 (0.2 to 0.8)	20.5 (14.8 to 31.6)	836.0 (707.7 to 991.8)	460.1 (366.8 to 571.5)	338.0 (239.5 to 452.7)	0.8 (0.4 to 1.4)	37.2 (26.9 to 57.3)
Guyana	2047	469.6 (395.7 to 557.0)	258.6 (206.5 to 322.0)	189.4 (134.1 to 253.7)	0.4 (0.2 to 0.8)	21.2 (15.2 to 33.4)	851.8 (717.7 to 1010.3)	469.0 (374.6 to 584.1)	343.5 (243.2 to 460.1)	0.8 (0.4 to 1.5)	38.5 (27.5 to 60.6)
Guyana	2048	478.0 (401.0 to 565.3)	263.3 (209.8 to 329.6)	192.4 (136.1 to 257.6)	0.4 (0.2 to 0.8)	22.0 (15.6 to 35.3)	867.1 (727.4 to 1025.3)	477.5 (380.5 to 597.9)	348.9 (246.9 to 467.3)	0.8 (0.4 to 1.5)	39.8 (28.4 to 64.1)
Guyana	2049	486.1 (404.7 to 574.3)	267.6 (213.5 to 335.0)	195.4 (138.4 to 261.4)	0.5 (0.2 to 0.8)	22.7 (16.2 to 36.7)	881.8 (734.1 to 1041.7)	485.4 (387.3 to 607.7)	354.4 (251.1 to 474.1)	0.8 (0.4 to 1.5)	41.2 (29.3 to 66.5)
Guyana	2050	493.8 (412.8 to 588.3)	271.5 (215.9 to 340.4)	198.3 (140.4 to 264.8)	0.5 (0.2 to 0.9)	23.5 (16.5 to 38.2)	895.7 (748.8 to 1067.1)	492.5 (391.6 to 617.5)	359.6 (254.7 to 480.4)	0.8 (0.4 to 1.6)	42.7 (29.9 to 69.2)
Haiti	1995	59.8 (51.3 to 69.8)	9.4 (7.1 to 12.2)	25.6 (18.2 to 35.4)	3.4 (1.7 to 6.5)	21.4 (21.4 to 21.4)	143.9 (123.4 to 167.9)	22.5 (17.0 to 29.3)	61.7 (43.7 to 85.2)	8.3 (4.0 to 15.6)	51.4 (51.4 to 51.4)
Haiti	1996	50.1 (41.5 to 60.5)	9.4 (7.1 to 12.3)	26.6 (18.8 to 36.9)	3.4 (1.6 to 6.4)	10.7 (10.7 to 10.7)	120.4 (99.7 to 145.5)	22.6 (17.2 to 29.7)	63.9 (45.2 to 88.7)	8.3 (3.9 to 15.5)	25.6 (25.6 to 25.6)
Haiti	1997	43.6 (35.4 to 53.5)	9.2 (6.9 to 12.1)	26.2 (18.8 to 35.8)	3.3 (1.6 to 6.3)	4.9 (4.9 to 4.9)	104.8 (85.1 to 128.8)	22.0 (16.7 to 29.1)	62.9 (45.1 to 86.2)	8.0 (3.8 to 15.2)	11.9 (11.9 to 11.9)
Haiti	1998	46.1 (38.5 to 55.1)	8.7 (6.7 to 11.6)	24.9 (18.1 to 33.8)	3.2 (1.5 to 6.3)	9.2 (9.2 to 9.2)	110.8 (92.5 to 132.6)	21.0 (16.0 to 28.0)	59.9 (43.5 to 81.3)	7.7 (3.7 to 15.1)	22.2 (22.2 to 22.2)
Haiti	1999	44.2 (36.7 to 53.3)	9.1 (6.9 to 12.0)	24.8 (18.2 to 33.6)	3.3 (1.6 to 6.4)	6.9 (6.9 to 6.9)	106.3 (88.4 to 128.2)	21.9 (16.7 to 28.8)	59.8 (43.7 to 80.7)	8.0 (3.8 to 15.4)	16.7 (16.7 to 16.7)
Haiti	2000	44.4 (36.8 to 53.8)	9.9 (7.5 to 12.9)	25.5 (18.7 to 34.5)	3.6 (1.7 to 7.0)	5.4 (5.4 to 5.4)	106.8 (88.5 to 129.3)	23.8 (18.1 to 31.1)	61.4 (45.1 to 83.0)	8.7 (4.0 to 16.8)	13.0 (13.0 to 13.0)
Haiti	2001	41.7 (34.7 to 50.1)	9.0 (6.9 to 11.7)	24.2 (17.8 to 32.7)	3.4 (1.6 to 6.2)	5.1 (5.1 to 5.1)	100.3 (83.4 to 120.4)	21.6 (16.5 to 28.1)	58.2 (42.8 to 78.7)	8.2 (3.8 to 15.0)	12.3 (12.3 to 12.3)
Haiti	2002	37.1 (30.4 to 44.9)	7.7 (5.9 to 10.0)	22.5 (16.4 to 30.3)	3.1 (1.5 to 5.7)	3.8 (3.8 to 3.8)	89.2 (73.1 to 108.0)	18.5 (14.1 to 24.1)	54.2 (39.4 to 72.9)	7.5 (3.6 to 13.8)	9.1 (9.1 to 9.1)
Haiti	2003	38.6 (32.2 to 46.2)	7.1 (5.4 to 9.0)	21.1 (15.4 to 28.4)	3.0 (1.4 to 5.5)	7.5 (7.5 to 7.5)	92.7 (77.3 to 111.1)	17.0 (13.0 to 21.7)	50.7 (37.0 to 68.2)	7.1 (3.4 to 13.2)	17.9 (17.9 to 17.9)
Haiti	2004	37.3 (31.2 to 44.8)	6.7 (5.1 to 8.6)	19.7 (14.3 to 26.7)	2.8 (1.4 to 5.3)	8.1 (8.1 to 8.1)	89.8 (74.9 to 107.8)	16.1 (12.2 to 20.6)	47.3 (34.5 to 64.1)	6.8 (3.3 to 12.8)	19.5 (19.5 to 19.5)
Haiti	2005	37.1 (31.4 to 43.8)	6.4 (4.9 to 8.2)	18.5 (13.6 to 25.0)	2.7 (1.3 to 5.1)	9.5 (9.5 to 9.5)	89.2 (75.5 to 105.3)	15.4 (11.7 to 19.8)	44.5 (32.6 to 60.2)	6.5 (3.2 to 12.2)	22.9 (22.9 to 22.9)
Haiti	2006	41.2 (35.8 to 47.8)	5.9 (4.4 to 7.5)	17.3 (12.7 to 23.4)	2.4 (1.2 to 4.4)	15.6 (15.6 to 15.6)	99.0 (86.2 to 114.9)	14.1 (10.7 to 18.1)	41.5 (30.5 to 56.2)	5.8 (2.9 to 10.7)	37.5 (37.5 to 37.5)
Haiti	2007	44.4 (38.9 to 51.3)	7.4 (5.6 to 9.4)	17.9 (13.2 to 24.5)	2.6 (1.2 to 4.8)	16.5 (16.5 to 16.5)	106.7 (93.6 to 123.4)	17.9 (13.5 to 22.7)	43.0 (31.7 to 58.9)	6.2 (3.0 to 11.4)	39.7 (39.7 to 39.7)
Haiti	2008	47.5 (41.7 to 54.4)	8.2 (6.3 to 10.6)	18.0 (13.3 to 24.6)	2.7 (1.3 to 5.0)	18.4 (18.4 to 18.4)	114.2 (100.4 to 130.9)	19.8 (15.2 to 25.4)	43.4 (32.0 to 59.0)	6.6 (3.1 to 12.1)	44.4 (44.4 to 44.4)

Country	Year	2018 US Dollars per capita					2018 PPP per capita				
		Health spending	Government health spending	Out-of-pocket health spending	Prepaid private health spending	Development assistance for health	Health spending	Government health spending	Out-of-pocket health spending	Prepaid private health spending	Development assistance for health
Haiti	2009	45.5 (39.6 to 52.8)	8.7 (6.7 to 11.1)	18.8 (13.7 to 25.6)	2.9 (1.3 to 5.5)	15.2 (15.2 to 15.2)	109.5 (95.3 to 127.0)	20.8 (16.0 to 26.8)	45.1 (32.9 to 61.7)	7.0 (3.1 to 13.1)	36.6 (36.6 to 36.6)
Haiti	2010	48.8 (43.2 to 56.2)	7.9 (6.0 to 10.1)	18.8 (13.6 to 25.6)	2.8 (1.2 to 5.2)	19.4 (19.4 to 19.4)	117.4 (103.8 to 135.1)	18.9 (14.5 to 24.4)	45.1 (32.7 to 61.7)	6.7 (2.9 to 12.6)	46.7 (46.7 to 46.7)
Haiti	2011	50.3 (44.9 to 57.3)	6.9 (5.3 to 9.0)	18.8 (13.6 to 25.7)	2.2 (0.9 to 4.2)	22.5 (22.5 to 22.5)	121.1 (108.1 to 137.9)	16.6 (12.7 to 21.6)	45.2 (32.6 to 61.8)	5.3 (2.3 to 10.0)	54.0 (54.0 to 54.0)
Haiti	2012	50.4 (44.8 to 57.6)	6.5 (5.0 to 8.5)	18.9 (13.6 to 25.4)	2.1 (0.9 to 4.1)	22.9 (22.9 to 22.9)	121.3 (107.9 to 138.4)	15.6 (12.0 to 20.3)	45.5 (32.7 to 61.0)	5.1 (2.3 to 9.8)	55.1 (55.1 to 55.1)
Haiti	2013	50.3 (44.6 to 57.6)	6.5 (5.0 to 8.4)	18.8 (13.5 to 25.3)	2.2 (1.0 to 4.4)	22.8 (22.8 to 22.8)	121.1 (107.3 to 138.6)	15.6 (11.9 to 20.3)	45.2 (32.4 to 60.9)	5.4 (2.4 to 10.5)	54.9 (54.9 to 54.9)
Haiti	2014	47.7 (42.1 to 54.7)	6.4 (4.8 to 8.4)	18.1 (12.9 to 24.5)	2.2 (1.0 to 4.2)	21.1 (21.1 to 21.1)	114.8 (101.3 to 131.6)	15.3 (11.7 to 20.1)	43.5 (31.1 to 59.0)	5.3 (2.4 to 10.0)	50.8 (50.8 to 50.8)
Haiti	2015	47.6 (42.3 to 54.4)	6.3 (4.8 to 8.2)	17.3 (12.3 to 23.8)	2.1 (0.9 to 4.0)	21.9 (21.9 to 21.9)	114.4 (101.6 to 130.9)	15.1 (11.5 to 19.8)	41.6 (29.6 to 57.3)	5.1 (2.3 to 9.6)	52.6 (52.6 to 52.6)
Haiti	2016	47.0 (41.7 to 53.9)	6.1 (4.6 to 8.0)	16.8 (11.9 to 23.1)	2.0 (0.9 to 3.8)	22.0 (22.0 to 22.0)	113.1 (100.2 to 129.7)	14.8 (11.2 to 19.2)	40.5 (28.7 to 55.7)	4.9 (2.2 to 9.1)	53.0 (53.0 to 53.0)
Haiti	2017	42.2 (36.8 to 49.1)	6.2 (4.6 to 8.0)	16.8 (11.8 to 23.2)	2.0 (0.9 to 3.8)	17.2 (17.2 to 17.2)	101.4 (88.5 to 118.1)	14.8 (11.2 to 19.3)	40.4 (28.4 to 55.7)	4.9 (2.2 to 9.2)	41.3 (41.3 to 41.3)
Haiti	2018	41.3 (36.0 to 48.0)	6.2 (4.7 to 8.1)	16.8 (11.7 to 23.1)	2.1 (0.9 to 3.9)	16.2 (16.2 to 16.2)	99.3 (86.5 to 115.6)	15.0 (11.3 to 19.6)	40.5 (28.2 to 55.6)	5.0 (2.3 to 9.4)	38.9 (38.9 to 38.9)
Haiti	2019	41.5 (35.7 to 48.4)	6.3 (4.8 to 8.3)	16.9 (11.7 to 23.2)	2.1 (1.0 to 3.9)	16.3 (15.2 to 17.2)	99.9 (85.8 to 116.3)	15.2 (11.5 to 19.9)	40.5 (28.2 to 55.9)	5.0 (2.3 to 9.5)	39.1 (36.6 to 41.4)
Haiti	2020	41.9 (36.3 to 48.8)	6.4 (4.8 to 8.4)	16.9 (11.9 to 23.4)	2.1 (1.0 to 4.0)	16.5 (15.1 to 17.8)	100.8 (87.2 to 117.4)	15.4 (11.6 to 20.1)	40.7 (28.6 to 56.2)	5.1 (2.3 to 9.6)	39.7 (36.4 to 42.8)
Haiti	2021	42.4 (36.6 to 49.4)	6.5 (4.9 to 8.5)	17.0 (11.9 to 23.4)	2.1 (1.0 to 4.1)	16.8 (15.1 to 18.5)	101.9 (88.0 to 118.8)	15.6 (11.8 to 20.4)	40.8 (28.6 to 56.4)	5.2 (2.3 to 9.7)	40.3 (36.3 to 44.6)
Haiti	2022	42.9 (37.1 to 50.8)	6.6 (5.0 to 8.6)	17.0 (12.0 to 23.5)	2.2 (1.0 to 4.1)	17.1 (15.2 to 19.1)	103.2 (89.3 to 122.1)	15.8 (11.9 to 20.6)	41.0 (28.7 to 56.6)	5.2 (2.4 to 9.9)	41.2 (36.6 to 46.0)
Haiti	2023	43.5 (37.0 to 50.9)	6.7 (5.0 to 8.7)	17.1 (11.9 to 23.6)	2.2 (1.0 to 4.2)	17.5 (15.2 to 19.9)	104.6 (89.1 to 122.3)	16.0 (12.1 to 20.9)	41.2 (28.7 to 56.8)	5.3 (2.4 to 10.0)	42.1 (36.6 to 47.8)
Haiti	2024	44.1 (37.8 to 52.1)	6.8 (5.1 to 8.8)	17.2 (12.0 to 23.8)	2.2 (1.0 to 4.2)	17.9 (15.4 to 20.6)	106.0 (90.8 to 125.3)	16.3 (12.3 to 21.2)	41.4 (28.8 to 57.2)	5.4 (2.4 to 10.2)	43.0 (37.1 to 49.6)
Haiti	2025	44.6 (38.4 to 52.0)	6.9 (5.2 to 8.9)	17.3 (12.1 to 23.9)	2.3 (1.0 to 4.3)	18.2 (15.6 to 21.3)	107.4 (92.4 to 125.1)	16.5 (12.5 to 21.4)	41.5 (29.0 to 57.5)	5.4 (2.5 to 10.3)	43.9 (37.4 to 51.3)
Haiti	2026	45.2 (38.9 to 53.1)	7.0 (5.3 to 9.0)	17.3 (12.2 to 24.0)	2.3 (1.0 to 4.3)	18.6 (15.7 to 21.8)	108.7 (93.6 to 127.6)	16.8 (12.7 to 21.8)	41.7 (29.3 to 57.8)	5.5 (2.5 to 10.4)	44.8 (37.8 to 52.5)
Haiti	2027	45.8 (39.2 to 54.0)	7.1 (5.3 to 9.2)	17.4 (12.2 to 24.2)	2.3 (1.1 to 4.4)	19.0 (15.9 to 22.6)	110.2 (94.2 to 129.8)	17.0 (12.9 to 22.1)	41.9 (29.3 to 58.2)	5.6 (2.5 to 10.5)	45.7 (38.3 to 54.4)
Haiti	2028	46.5 (39.9 to 55.1)	7.2 (5.4 to 9.3)	17.5 (12.2 to 24.3)	2.3 (1.1 to 4.4)	19.5 (16.3 to 23.5)	111.7 (96.0 to 132.6)	17.3 (13.0 to 22.4)	42.0 (29.4 to 58.4)	5.6 (2.6 to 10.7)	46.8 (39.1 to 56.5)
Haiti	2029	47.1 (40.2 to 55.8)	7.3 (5.5 to 9.5)	17.5 (12.3 to 24.5)	2.4 (1.1 to 4.5)	19.9 (16.4 to 24.3)	113.3 (96.6 to 134.3)	17.5 (13.2 to 22.7)	42.1 (29.5 to 58.9)	5.7 (2.6 to 10.8)	47.9 (39.4 to 58.4)

Country	Year	2018 US Dollars per capita					2018 PPP per capita				
		Health spending	Government health spending	Out-of-pocket health spending	Prepaid private health spending	Development assistance for health	Health spending	Government health spending	Out-of-pocket health spending	Prepaid private health spending	Development assistance for health
Haiti	2030	47.8 (40.7 to 56.6)	7.4 (5.6 to 9.6)	17.6 (12.3 to 24.6)	2.4 (1.1 to 4.5)	20.4 (16.7 to 24.9)	114.8 (97.9 to 136.2)	17.8 (13.4 to 23.1)	42.3 (29.6 to 59.2)	5.8 (2.6 to 10.9)	49.0 (40.1 to 60.0)
Haiti	2031	48.4 (40.8 to 57.3)	7.5 (5.6 to 9.7)	17.6 (12.3 to 24.7)	2.4 (1.1 to 4.6)	20.8 (16.9 to 26.2)	116.4 (98.2 to 137.8)	18.0 (13.5 to 23.4)	42.4 (29.6 to 59.3)	5.9 (2.7 to 11.1)	50.1 (40.6 to 62.9)
Haiti	2032	49.1 (41.5 to 58.6)	7.6 (5.7 to 9.9)	17.7 (12.3 to 24.8)	2.5 (1.1 to 4.7)	21.3 (17.1 to 26.8)	118.1 (99.9 to 140.9)	18.3 (13.7 to 23.7)	42.6 (29.7 to 59.7)	5.9 (2.7 to 11.2)	51.3 (41.1 to 64.6)
Haiti	2033	49.8 (41.6 to 59.4)	7.7 (5.8 to 10.0)	17.8 (12.4 to 24.9)	2.5 (1.1 to 4.7)	21.9 (17.4 to 28.0)	119.7 (100.2 to 143.0)	18.5 (13.9 to 24.1)	42.7 (29.7 to 59.9)	6.0 (2.7 to 11.3)	52.6 (41.7 to 67.4)
Haiti	2034	50.5 (42.7 to 60.8)	7.8 (5.8 to 10.2)	17.8 (12.4 to 24.9)	2.5 (1.1 to 4.8)	22.4 (17.7 to 28.8)	121.5 (102.8 to 146.2)	18.7 (14.1 to 24.4)	42.8 (29.7 to 59.9)	6.1 (2.8 to 11.5)	53.9 (42.5 to 69.3)
Haiti	2035	51.3 (43.3 to 62.0)	7.9 (5.9 to 10.3)	17.9 (12.4 to 25.1)	2.6 (1.2 to 4.8)	23.0 (17.9 to 30.1)	123.4 (104.1 to 149.0)	18.9 (14.3 to 24.7)	43.0 (29.8 to 60.4)	6.1 (2.8 to 11.7)	55.3 (43.0 to 72.4)
Haiti	2036	52.1 (43.2 to 63.0)	8.0 (6.0 to 10.4)	17.9 (12.4 to 25.3)	2.6 (1.2 to 4.9)	23.6 (18.3 to 31.7)	125.2 (103.9 to 151.4)	19.2 (14.3 to 25.1)	43.2 (29.8 to 60.8)	6.2 (2.8 to 11.8)	56.7 (44.0 to 76.2)
Haiti	2037	52.8 (44.4 to 64.4)	8.1 (6.0 to 10.6)	18.0 (12.4 to 25.4)	2.6 (1.2 to 5.0)	24.1 (18.6 to 32.3)	127.0 (106.9 to 154.8)	19.4 (14.5 to 25.4)	43.3 (29.8 to 61.1)	6.3 (2.9 to 12.0)	58.1 (44.7 to 77.7)
Haiti	2038	53.6 (44.0 to 65.6)	8.2 (6.1 to 10.7)	18.1 (12.4 to 25.5)	2.7 (1.2 to 5.1)	24.8 (18.9 to 34.2)	129.0 (105.8 to 157.8)	19.6 (14.7 to 25.6)	43.4 (29.8 to 61.4)	6.4 (2.9 to 12.1)	59.5 (45.4 to 82.4)
Haiti	2039	54.4 (45.3 to 66.9)	8.3 (6.2 to 10.8)	18.1 (12.4 to 25.6)	2.7 (1.2 to 5.1)	25.4 (19.3 to 35.1)	130.9 (108.8 to 160.9)	19.8 (14.9 to 26.0)	43.6 (29.9 to 61.6)	6.5 (3.0 to 12.3)	61.0 (46.5 to 84.3)
Haiti	2040	55.3 (45.2 to 68.4)	8.3 (6.3 to 10.9)	18.2 (12.5 to 25.7)	2.7 (1.3 to 5.2)	26.0 (19.8 to 35.9)	132.9 (108.8 to 164.5)	20.1 (15.1 to 26.3)	43.7 (30.0 to 61.8)	6.5 (3.0 to 12.4)	62.6 (47.7 to 86.3)
Haiti	2041	56.2 (46.3 to 69.4)	8.5 (6.3 to 11.1)	18.2 (12.5 to 25.9)	2.7 (1.3 to 5.2)	26.7 (20.2 to 37.1)	135.1 (111.5 to 166.8)	20.3 (15.3 to 26.8)	43.9 (30.1 to 62.3)	6.6 (3.1 to 12.5)	64.2 (48.5 to 89.2)
Haiti	2042	57.1 (46.6 to 70.9)	8.6 (6.4 to 11.3)	18.3 (12.6 to 26.0)	2.8 (1.3 to 5.3)	27.4 (20.6 to 39.3)	137.3 (112.0 to 170.5)	20.6 (15.4 to 27.2)	44.0 (30.2 to 62.6)	6.7 (3.1 to 12.6)	66.0 (49.5 to 94.5)
Haiti	2043	58.0 (47.5 to 73.0)	8.7 (6.5 to 11.5)	18.4 (12.6 to 26.2)	2.8 (1.3 to 5.3)	28.2 (20.9 to 40.9)	139.6 (114.3 to 175.6)	20.9 (15.6 to 27.6)	44.2 (30.3 to 62.9)	6.8 (3.1 to 12.7)	67.7 (50.2 to 98.3)
Haiti	2044	59.0 (47.7 to 75.3)	8.8 (6.5 to 11.7)	18.4 (12.6 to 26.3)	2.8 (1.3 to 5.3)	28.9 (21.5 to 42.0)	142.0 (114.6 to 181.0)	21.2 (15.7 to 28.0)	44.3 (30.4 to 63.2)	6.8 (3.2 to 12.8)	69.6 (51.6 to 101.1)
Haiti	2045	60.1 (48.8 to 77.9)	9.0 (6.7 to 11.8)	18.5 (12.7 to 26.4)	2.9 (1.3 to 5.4)	29.8 (21.6 to 44.8)	144.5 (117.4 to 187.3)	21.5 (16.0 to 28.4)	44.5 (30.5 to 63.4)	6.9 (3.2 to 12.9)	71.6 (51.9 to 107.7)
Haiti	2046	61.2 (49.2 to 79.3)	9.1 (6.7 to 12.0)	18.5 (12.7 to 26.5)	2.9 (1.3 to 5.4)	30.6 (22.1 to 46.8)	147.1 (118.4 to 190.7)	21.9 (16.2 to 28.9)	44.6 (30.5 to 63.8)	7.0 (3.2 to 13.1)	73.6 (53.2 to 112.5)
Haiti	2047	62.3 (50.3 to 83.1)	9.3 (6.8 to 12.2)	18.6 (12.7 to 26.6)	2.9 (1.3 to 5.5)	31.5 (22.7 to 49.6)	149.8 (121.1 to 200.0)	22.3 (16.4 to 29.3)	44.7 (30.6 to 64.1)	7.1 (3.2 to 13.2)	75.8 (54.6 to 119.4)
Haiti	2048	63.5 (50.0 to 84.1)	9.4 (6.9 to 12.5)	18.7 (12.8 to 26.7)	3.0 (1.4 to 5.5)	32.5 (23.3 to 51.7)	152.8 (120.3 to 202.2)	22.7 (16.7 to 29.9)	44.9 (30.7 to 64.2)	7.1 (3.3 to 13.3)	78.1 (56.0 to 124.3)
Haiti	2049	64.7 (51.6 to 86.5)	9.6 (7.0 to 12.7)	18.7 (12.8 to 26.8)	3.0 (1.4 to 5.6)	33.4 (23.8 to 53.7)	155.7 (124.1 to 208.1)	23.1 (16.9 to 30.6)	45.0 (30.9 to 64.4)	7.2 (3.3 to 13.5)	80.4 (57.2 to 129.2)
Haiti	2050	66.1 (52.2 to 91.2)	9.8 (7.1 to 12.9)	18.8 (12.9 to 26.9)	3.0 (1.4 to 5.7)	34.5 (24.4 to 56.8)	158.9 (125.6 to 219.4)	23.5 (17.2 to 31.1)	45.2 (31.0 to 64.6)	7.3 (3.3 to 13.7)	83.0 (58.7 to 136.6)

		2018 US Dollars per capita					2018 PPP per capita				
Country	Year	Health spending	Government health spending	Out-of-pocket health spending	Prepaid private health spending	Development assistance for health	Health spending	Government health spending	Out-of-pocket health spending	Prepaid private health spending	Development assistance for health
Honduras	1995	100.8 (84.7 to 118.4)	39.4 (31.7 to 49.1)	44.4 (33.7 to 58.0)	10.3 (4.8 to 19.5)	6.7 (6.7 to 6.7)	209.7 (176.2 to 246.4)	82.1 (65.9 to 102.3)	92.3 (70.1 to 120.6)	21.4 (10.0 to 40.6)	13.9 (13.9 to 13.9)
Honduras	1996	101.9 (86.8 to 118.4)	37.6 (30.3 to 46.4)	44.5 (34.1 to 57.8)	9.8 (4.7 to 18.8)	9.9 (9.9 to 9.9)	212.0 (180.8 to 246.4)	78.3 (63.0 to 96.6)	92.7 (71.0 to 120.4)	20.4 (9.7 to 39.1)	20.6 (20.6 to 20.6)
Honduras	1997	105.7 (90.7 to 122.9)	39.5 (31.9 to 48.1)	46.2 (35.6 to 58.9)	9.8 (4.8 to 19.0)	10.1 (10.1 to 10.1)	220.0 (188.8 to 255.9)	82.3 (66.5 to 100.1)	96.2 (74.2 to 122.7)	20.4 (10.0 to 39.5)	21.1 (21.1 to 21.1)
Honduras	1998	107.7 (92.0 to 125.8)	42.8 (34.7 to 51.8)	48.9 (38.0 to 62.2)	9.8 (4.8 to 18.3)	6.1 (6.1 to 6.1)	224.1 (191.5 to 261.8)	89.2 (72.2 to 107.9)	101.8 (79.1 to 129.5)	20.4 (10.1 to 38.1)	12.7 (12.7 to 12.7)
Honduras	1999	118.7 (103.4 to 137.1)	43.7 (35.6 to 53.4)	51.6 (40.4 to 65.1)	9.1 (4.6 to 16.7)	14.3 (14.3 to 14.3)	247.1 (215.3 to 285.4)	90.9 (74.1 to 111.1)	107.4 (84.1 to 135.4)	18.9 (9.5 to 34.7)	29.8 (29.8 to 29.8)
Honduras	2000	122.1 (106.4 to 141.2)	48.8 (39.8 to 59.7)	55.4 (43.5 to 69.4)	8.7 (4.4 to 15.8)	9.3 (9.3 to 9.3)	254.2 (221.5 to 293.8)	101.5 (82.9 to 124.3)	115.2 (90.5 to 144.5)	18.1 (9.2 to 32.9)	19.3 (19.3 to 19.3)
Honduras	2001	121.7 (105.8 to 140.1)	48.7 (39.7 to 59.3)	58.0 (46.0 to 71.5)	8.5 (4.3 to 15.5)	6.5 (6.5 to 6.5)	253.4 (220.2 to 291.6)	101.4 (82.6 to 123.4)	120.8 (95.7 to 148.8)	17.7 (8.9 to 32.3)	13.5 (13.5 to 13.5)
Honduras	2002	124.5 (107.9 to 143.4)	49.7 (40.2 to 61.1)	61.2 (48.7 to 75.2)	9.0 (4.4 to 16.1)	4.5 (4.5 to 4.5)	259.1 (224.6 to 298.5)	103.5 (83.6 to 127.1)	127.5 (101.4 to 156.5)	18.8 (9.3 to 33.5)	9.3 (9.3 to 9.3)
Honduras	2003	133.6 (115.7 to 153.3)	51.5 (41.7 to 63.1)	65.0 (52.2 to 80.5)	10.1 (4.9 to 17.9)	7.0 (7.0 to 7.0)	278.1 (240.9 to 319.1)	107.2 (86.8 to 131.4)	135.4 (108.6 to 167.6)	20.9 (10.2 to 37.2)	14.6 (14.6 to 14.6)
Honduras	2004	146.1 (126.5 to 167.1)	54.3 (44.4 to 65.9)	70.0 (55.6 to 85.3)	11.5 (5.9 to 20.5)	10.4 (10.4 to 10.4)	304.2 (263.4 to 347.8)	113.0 (92.5 to 137.1)	145.6 (115.8 to 177.5)	24.0 (12.2 to 42.6)	21.6 (21.6 to 21.6)
Honduras	2005	161.6 (139.6 to 184.5)	60.2 (49.4 to 72.7)	76.6 (62.4 to 93.2)	13.1 (6.7 to 23.2)	11.7 (11.7 to 11.7)	336.3 (290.6 to 384.1)	125.4 (102.7 to 151.4)	159.4 (129.8 to 193.9)	27.2 (13.9 to 48.3)	24.3 (24.3 to 24.3)
Honduras	2006	171.5 (148.7 to 196.1)	67.0 (55.4 to 79.9)	83.4 (68.2 to 101.1)	13.4 (6.8 to 24.0)	7.6 (7.6 to 7.6)	356.9 (309.4 to 408.2)	139.6 (115.3 to 166.3)	173.6 (142.0 to 210.5)	27.9 (14.1 to 50.0)	15.8 (15.8 to 15.8)
Honduras	2007	182.2 (159.6 to 207.2)	72.8 (60.7 to 86.5)	88.9 (73.1 to 106.9)	12.1 (6.2 to 21.7)	8.4 (8.4 to 8.4)	379.3 (332.1 to 431.2)	151.6 (126.2 to 180.0)	185.0 (152.1 to 222.6)	25.2 (13.0 to 45.2)	17.6 (17.6 to 17.6)
Honduras	2008	188.0 (166.4 to 212.5)	76.8 (63.9 to 90.8)	92.5 (76.9 to 111.0)	10.9 (5.6 to 19.6)	7.8 (7.8 to 7.8)	391.2 (346.4 to 442.3)	159.8 (133.0 to 189.0)	192.5 (160.0 to 231.0)	22.7 (11.6 to 40.9)	16.2 (16.2 to 16.2)
Honduras	2009	186.4 (165.7 to 210.4)	78.6 (66.0 to 92.2)	90.4 (74.8 to 107.8)	10.5 (5.5 to 18.7)	7.0 (7.0 to 7.0)	388.0 (344.9 to 438.0)	163.6 (137.4 to 191.9)	188.2 (155.6 to 224.3)	21.8 (11.4 to 39.0)	14.5 (14.5 to 14.5)
Honduras	2010	188.5 (167.2 to 212.8)	79.8 (67.2 to 93.5)	90.7 (75.0 to 107.8)	10.9 (5.6 to 19.2)	7.1 (7.1 to 7.1)	392.3 (348.0 to 443.0)	166.2 (139.8 to 194.6)	188.7 (156.1 to 224.3)	22.6 (11.6 to 39.9)	14.7 (14.7 to 14.7)
Honduras	2011	191.1 (170.2 to 214.6)	80.4 (68.0 to 94.1)	91.8 (76.4 to 109.1)	11.1 (5.7 to 19.6)	7.8 (7.8 to 7.8)	397.8 (354.2 to 446.7)	167.4 (141.5 to 196.0)	191.0 (159.0 to 227.1)	23.2 (11.8 to 40.7)	16.3 (16.3 to 16.3)
Honduras	2012	192.3 (170.9 to 217.6)	77.8 (65.3 to 91.8)	93.0 (77.1 to 110.5)	11.3 (5.9 to 19.8)	10.2 (10.2 to 10.2)	400.3 (355.7 to 453.0)	161.9 (135.9 to 191.1)	193.6 (160.5 to 229.9)	23.5 (12.2 to 41.2)	21.2 (21.2 to 21.2)
Honduras	2013	185.9 (164.3 to 211.3)	75.1 (62.5 to 88.6)	91.5 (74.8 to 109.9)	11.0 (5.6 to 19.6)	8.2 (8.2 to 8.2)	386.9 (342.1 to 439.7)	156.4 (130.1 to 184.4)	190.5 (155.8 to 228.7)	22.9 (11.7 to 40.8)	17.1 (17.1 to 17.1)
Honduras	2014	182.6 (159.4 to 209.4)	74.9 (62.3 to 89.7)	89.8 (73.1 to 108.5)	11.0 (5.7 to 19.8)	6.9 (6.9 to 6.9)	380.1 (331.7 to 435.9)	156.0 (129.6 to 186.7)	186.9 (152.1 to 225.8)	22.9 (11.8 to 41.1)	14.3 (14.3 to 14.3)
Honduras	2015	183.7 (160.3 to 212.2)	76.2 (63.2 to 91.7)	89.5 (71.4 to 110.5)	11.4 (5.8 to 21.2)	6.6 (6.6 to 6.6)	382.4 (333.6 to 441.6)	158.6 (131.5 to 190.9)	186.2 (148.6 to 230.0)	23.8 (12.2 to 44.2)	13.8 (13.8 to 13.8)

		2018 US Dollars per capita					2018 PPP per capita				
Country	Year	Health spending	Government health spending	Out-of-pocket health spending	Prepaid private health spending	Development assistance for health	Health spending	Government health spending	Out-of-pocket health spending	Prepaid private health spending	Development assistance for health
Honduras	2016	192.9 (164.8 to 222.1)	83.1 (68.3 to 100.6)	91.3 (71.1 to 115.0)	12.5 (6.4 to 23.4)	6.0 (6.0 to 6.0)	401.4 (342.9 to 462.3)	172.9 (142.1 to 209.3)	190.0 (148.0 to 239.3)	26.0 (13.4 to 48.7)	12.6 (12.6 to 12.6)
Honduras	2017	196.2 (167.4 to 229.6)	86.2 (70.9 to 104.4)	93.9 (73.2 to 117.9)	12.9 (6.6 to 24.1)	3.2 (3.2 to 3.2)	408.4 (348.4 to 477.8)	179.5 (147.6 to 217.3)	195.4 (152.4 to 245.4)	26.7 (13.8 to 50.2)	6.7 (6.7 to 6.7)
Honduras	2018	200.7 (170.6 to 231.4)	88.7 (72.9 to 107.4)	95.8 (75.1 to 120.0)	13.2 (6.8 to 24.7)	3.0 (3.0 to 3.0)	417.8 (355.1 to 481.7)	184.6 (151.6 to 223.6)	199.5 (156.3 to 249.8)	27.4 (14.2 to 51.4)	6.3 (6.3 to 6.3)
Honduras	2019	205.2 (175.7 to 239.9)	91.0 (74.7 to 110.2)	97.8 (76.5 to 122.3)	13.5 (7.0 to 25.2)	3.0 (2.8 to 3.2)	427.2 (365.6 to 499.3)	189.3 (155.6 to 229.3)	203.6 (159.3 to 254.6)	28.1 (14.5 to 52.5)	6.3 (5.8 to 6.6)
Honduras	2020	209.9 (178.7 to 242.5)	93.3 (76.6 to 112.8)	99.8 (78.0 to 124.6)	13.8 (7.1 to 25.8)	3.0 (2.8 to 3.3)	436.9 (372.0 to 504.7)	194.1 (159.4 to 234.7)	207.7 (162.3 to 259.3)	28.7 (14.8 to 53.8)	6.3 (5.8 to 6.8)
Honduras	2021	214.1 (183.0 to 250.4)	95.3 (78.3 to 115.2)	101.6 (79.5 to 127.4)	14.1 (7.3 to 26.4)	3.1 (2.7 to 3.4)	445.6 (380.8 to 521.1)	198.3 (162.9 to 239.7)	211.5 (165.4 to 265.2)	29.4 (15.1 to 54.9)	6.4 (5.7 to 7.0)
Honduras	2022	218.5 (185.9 to 253.1)	97.7 (80.2 to 118.2)	103.3 (80.8 to 129.0)	14.5 (7.4 to 27.0)	3.1 (2.7 to 3.5)	454.7 (386.9 to 526.8)	203.3 (167.0 to 245.9)	214.9 (168.2 to 268.6)	30.1 (15.5 to 56.1)	6.4 (5.7 to 7.2)
Honduras	2023	223.0 (190.3 to 260.7)	100.1 (82.1 to 120.7)	104.9 (81.9 to 131.2)	14.8 (7.6 to 27.7)	3.1 (2.7 to 3.6)	464.1 (396.2 to 542.6)	208.3 (171.0 to 251.3)	218.4 (170.4 to 273.2)	30.8 (15.9 to 57.6)	6.5 (5.7 to 7.4)
Honduras	2024	227.4 (193.6 to 263.2)	102.5 (84.0 to 123.4)	106.5 (83.1 to 133.6)	15.2 (7.8 to 28.4)	3.2 (2.7 to 3.7)	473.3 (402.9 to 547.9)	213.4 (174.8 to 256.8)	221.7 (172.9 to 278.0)	31.6 (16.3 to 59.0)	6.6 (5.7 to 7.7)
Honduras	2025	231.7 (197.9 to 270.8)	104.9 (86.2 to 126.5)	108.0 (84.1 to 135.3)	15.5 (8.0 to 29.0)	3.2 (2.8 to 3.8)	482.2 (411.9 to 563.6)	218.4 (179.5 to 263.3)	224.8 (175.0 to 281.7)	32.3 (16.7 to 60.3)	6.7 (5.7 to 7.9)
Honduras	2026	236.0 (200.8 to 273.8)	107.4 (88.1 to 129.9)	109.4 (85.2 to 137.0)	15.9 (8.2 to 29.5)	3.3 (2.8 to 3.8)	491.1 (418.0 to 569.8)	223.5 (183.4 to 270.5)	227.8 (177.3 to 285.2)	33.1 (17.0 to 61.5)	6.8 (5.8 to 8.0)
Honduras	2027	240.1 (204.7 to 280.5)	109.7 (90.0 to 133.0)	110.8 (86.3 to 138.7)	16.2 (8.4 to 30.2)	3.3 (2.8 to 4.0)	499.8 (426.2 to 583.9)	228.3 (187.3 to 276.9)	230.7 (179.7 to 288.7)	33.8 (17.5 to 62.8)	6.9 (5.8 to 8.3)
Honduras	2028	244.1 (207.1 to 281.6)	112.0 (91.5 to 135.9)	112.1 (87.3 to 140.4)	16.6 (8.6 to 30.8)	3.4 (2.8 to 4.1)	508.0 (431.1 to 586.0)	233.1 (190.4 to 282.8)	233.3 (181.7 to 292.2)	34.5 (17.9 to 64.0)	7.0 (5.9 to 8.5)
Honduras	2029	247.9 (211.4 to 290.1)	114.3 (93.1 to 138.9)	113.3 (88.2 to 142.0)	16.9 (8.7 to 31.3)	3.4 (2.8 to 4.2)	516.1 (440.0 to 603.9)	237.8 (193.8 to 289.2)	235.8 (183.5 to 295.7)	35.2 (18.2 to 65.2)	7.2 (5.9 to 8.8)
Honduras	2030	251.9 (214.0 to 291.0)	116.7 (95.0 to 142.2)	114.5 (89.0 to 143.6)	17.3 (8.9 to 31.9)	3.5 (2.9 to 4.3)	524.4 (445.5 to 605.6)	242.8 (197.7 to 296.1)	238.3 (185.2 to 298.9)	35.9 (18.5 to 66.4)	7.3 (6.0 to 9.0)
Honduras	2031	255.8 (218.5 to 299.3)	119.0 (97.1 to 145.7)	115.6 (89.8 to 145.2)	17.6 (9.0 to 32.5)	3.6 (2.9 to 4.5)	532.5 (454.7 to 623.1)	247.8 (202.2 to 303.2)	240.7 (186.9 to 302.1)	36.6 (18.8 to 67.7)	7.4 (6.0 to 9.3)
Honduras	2032	259.8 (220.1 to 301.4)	121.5 (99.0 to 149.6)	116.8 (90.6 to 146.7)	17.9 (9.2 to 33.1)	3.6 (2.9 to 4.6)	540.8 (458.2 to 627.4)	252.8 (206.1 to 311.3)	243.1 (188.6 to 305.3)	37.3 (19.1 to 68.9)	7.6 (6.1 to 9.5)
Honduras	2033	263.8 (224.3 to 308.9)	123.9 (100.9 to 152.5)	117.9 (91.5 to 148.0)	18.3 (9.4 to 33.7)	3.7 (2.9 to 4.8)	549.1 (467.0 to 643.0)	257.9 (209.9 to 317.3)	245.4 (190.5 to 308.0)	38.0 (19.5 to 70.2)	7.7 (6.1 to 9.9)
Honduras	2034	267.6 (226.3 to 309.7)	126.3 (102.7 to 155.8)	118.9 (92.4 to 149.1)	18.6 (9.6 to 34.3)	3.8 (3.0 to 4.9)	557.1 (471.1 to 644.7)	263.0 (213.8 to 324.3)	247.5 (192.4 to 310.3)	38.7 (19.9 to 71.5)	7.9 (6.2 to 10.2)
Honduras	2035	271.7 (231.3 to 319.8)	128.9 (104.4 to 159.4)	120.0 (93.4 to 150.2)	18.9 (9.7 to 35.0)	3.9 (3.0 to 5.1)	565.4 (481.5 to 665.7)	268.2 (217.3 to 331.7)	249.7 (194.4 to 312.7)	39.4 (20.3 to 72.8)	8.1 (6.3 to 10.6)
Honduras	2036	275.7 (232.9 to 319.6)	131.4 (106.2 to 162.2)	121.1 (94.3 to 151.4)	19.3 (9.9 to 35.7)	4.0 (3.1 to 5.3)	573.9 (484.7 to 665.2)	273.5 (221.1 to 337.7)	252.0 (196.3 to 315.2)	40.2 (20.7 to 74.2)	8.2 (6.4 to 11.1)

Country	Year	2018 US Dollars per capita					2018 PPP per capita				
		Health spending	Government health spending	Out-of-pocket health spending	Prepaid private health spending	Development assistance for health	Health spending	Government health spending	Out-of-pocket health spending	Prepaid private health spending	Development assistance for health
Honduras	2037	279.8 (237.3 to 330.4)	134.0 (107.8 to 166.5)	122.2 (95.2 to 152.7)	19.7 (10.1 to 36.3)	4.0 (3.1 to 5.4)	582.5 (493.8 to 687.8)	278.9 (224.3 to 346.6)	254.3 (198.1 to 317.8)	40.9 (21.1 to 75.6)	8.4 (6.5 to 11.3)
Honduras	2038	284.0 (239.8 to 328.3)	136.6 (109.5 to 170.7)	123.2 (96.1 to 153.8)	20.0 (10.3 to 37.0)	4.1 (3.2 to 5.7)	591.1 (499.1 to 683.4)	284.4 (227.9 to 355.4)	256.5 (200.0 to 320.2)	41.7 (21.4 to 77.1)	8.6 (6.6 to 11.9)
Honduras	2039	288.3 (244.4 to 342.2)	139.3 (111.6 to 174.8)	124.4 (97.0 to 155.2)	20.4 (10.5 to 37.7)	4.2 (3.2 to 5.8)	600.1 (508.6 to 712.4)	290.0 (232.2 to 363.9)	258.8 (201.8 to 323.0)	42.4 (21.8 to 78.5)	8.8 (6.7 to 12.2)
Honduras	2040	292.5 (246.2 to 339.2)	142.1 (113.2 to 180.3)	125.4 (97.7 to 156.2)	20.7 (10.6 to 38.4)	4.3 (3.3 to 6.0)	608.9 (512.4 to 706.0)	295.7 (235.5 to 375.4)	261.0 (203.3 to 325.2)	43.2 (22.2 to 80.0)	9.0 (6.8 to 12.4)
Honduras	2041	296.8 (251.3 to 353.0)	144.8 (114.8 to 185.0)	126.5 (98.3 to 157.4)	21.1 (10.8 to 39.2)	4.4 (3.3 to 6.1)	617.7 (523.1 to 734.7)	301.4 (238.9 to 385.1)	263.2 (204.6 to 327.6)	44.0 (22.5 to 81.6)	9.2 (6.9 to 12.8)
Honduras	2042	301.0 (252.8 to 353.4)	147.6 (116.7 to 189.3)	127.5 (99.2 to 158.8)	21.5 (11.0 to 40.0)	4.5 (3.4 to 6.5)	626.6 (526.1 to 735.6)	307.1 (242.8 to 394.0)	265.3 (206.4 to 330.6)	44.7 (22.9 to 83.3)	9.4 (7.0 to 13.4)
Honduras	2043	305.4 (257.9 to 364.4)	150.4 (118.4 to 194.4)	128.5 (100.0 to 160.0)	21.9 (11.2 to 40.8)	4.6 (3.4 to 6.7)	635.6 (536.8 to 758.4)	313.1 (246.5 to 404.7)	267.4 (208.1 to 333.0)	45.5 (23.3 to 84.9)	9.6 (7.2 to 14.0)
Honduras	2044	309.7 (258.8 to 365.6)	153.2 (120.1 to 199.3)	129.5 (100.8 to 161.2)	22.2 (11.4 to 41.6)	4.7 (3.5 to 6.9)	644.5 (538.6 to 760.9)	318.9 (250.0 to 414.8)	269.5 (209.8 to 335.5)	46.3 (23.6 to 86.7)	9.9 (7.3 to 14.3)
Honduras	2045	314.0 (264.1 to 376.1)	156.1 (121.7 to 203.5)	130.5 (101.7 to 162.6)	22.6 (11.5 to 42.5)	4.9 (3.5 to 7.3)	653.6 (549.7 to 782.9)	324.9 (253.4 to 423.6)	271.5 (211.7 to 338.5)	47.1 (24.0 to 88.4)	10.1 (7.3 to 15.3)
Honduras	2046	318.4 (264.9 to 376.3)	159.0 (123.3 to 208.7)	131.4 (102.6 to 163.9)	23.0 (11.7 to 43.3)	5.0 (3.6 to 7.6)	662.6 (551.4 to 783.2)	330.9 (256.7 to 434.4)	273.5 (213.5 to 341.1)	47.9 (24.4 to 90.2)	10.4 (7.5 to 15.9)
Honduras	2047	322.8 (271.7 to 388.8)	161.9 (125.1 to 214.2)	132.4 (103.4 to 165.0)	23.4 (11.9 to 44.2)	5.1 (3.7 to 8.1)	671.9 (565.5 to 809.3)	337.0 (260.4 to 445.9)	275.6 (215.1 to 343.5)	48.7 (24.8 to 92.0)	10.7 (7.7 to 16.8)
Honduras	2048	327.2 (270.5 to 388.8)	164.8 (126.8 to 219.2)	133.3 (104.0 to 166.2)	23.8 (12.1 to 45.0)	5.3 (3.8 to 8.4)	681.0 (563.1 to 809.3)	343.1 (264.0 to 456.3)	277.5 (216.4 to 345.8)	49.5 (25.1 to 93.8)	11.0 (7.9 to 17.5)
Honduras	2049	331.5 (276.6 to 403.8)	167.8 (128.5 to 224.8)	134.2 (104.5 to 167.4)	24.1 (12.3 to 45.9)	5.4 (3.9 to 8.7)	690.0 (575.6 to 840.6)	349.2 (267.5 to 467.8)	279.3 (217.5 to 348.4)	50.2 (25.5 to 95.5)	11.3 (8.0 to 18.1)
Honduras	2050	335.9 (276.5 to 401.2)	170.7 (130.3 to 230.0)	135.1 (105.2 to 168.7)	24.5 (12.4 to 46.8)	5.6 (3.9 to 9.1)	699.2 (575.6 to 835.1)	355.4 (271.1 to 478.7)	281.2 (219.0 to 351.2)	51.1 (25.9 to 97.3)	11.6 (8.2 to 19.0)
Hungary	1995	607.7 (563.4 to 656.1)	476.0 (439.1 to 516.7)	103.2 (85.8 to 122.5)	28.2 (14.6 to 47.4)	0.2 (0.2 to 0.2)	1260.1 (1168.3 to 1360.3)	987.1 (910.5 to 1071.4)	214.1 (177.9 to 253.9)	58.4 (30.2 to 98.3)	0.5 (0.5 to 0.5)
Hungary	1996	590.4 (556.1 to 629.3)	452.9 (426.1 to 483.2)	109.0 (93.0 to 126.0)	27.0 (15.1 to 45.8)	1.4 (1.4 to 1.4)	1224.2 (1153.1 to 1304.8)	939.2 (883.5 to 1002.0)	226.0 (192.8 to 261.2)	56.1 (31.2 to 95.0)	3.0 (3.0 to 3.0)
Hungary	1997	594.1 (562.4 to 629.2)	446.8 (421.3 to 473.1)	119.1 (104.3 to 135.1)	26.8 (15.5 to 44.0)	1.4 (1.4 to 1.4)	1231.9 (1166.2 to 1304.6)	926.5 (873.6 to 980.9)	247.0 (216.2 to 280.1)	55.6 (32.0 to 91.2)	2.9 (2.9 to 2.9)
Hungary	1998	614.9 (583.4 to 649.1)	448.3 (423.1 to 475.1)	138.9 (122.8 to 156.3)	27.0 (15.8 to 43.4)	0.7 (0.7 to 0.7)	1275.0 (1209.6 to 1345.8)	929.6 (877.2 to 985.1)	287.9 (254.6 to 324.1)	56.0 (32.8 to 90.0)	1.5 (1.5 to 1.5)
Hungary	1999	638.5 (607.6 to 673.1)	451.9 (425.6 to 477.6)	159.2 (141.9 to 179.2)	26.9 (16.4 to 42.7)	0.3 (0.3 to 0.3)	1323.8 (1259.8 to 1395.6)	937.1 (882.5 to 990.3)	330.2 (294.3 to 371.5)	55.8 (34.0 to 88.5)	0.7 (0.7 to 0.7)
Hungary	2000	667.8 (634.5 to 702.8)	462.9 (435.6 to 489.3)	177.8 (160.2 to 199.6)	26.8 (16.3 to 41.2)	0.3 (0.3 to 0.3)	1384.7 (1315.7 to 1457.3)	959.8 (903.2 to 1014.6)	368.6 (332.2 to 413.8)	55.7 (33.7 to 85.3)	0.7 (0.7 to 0.7)
Hungary	2001	719.1 (684.9 to 755.6)	493.5 (468.5 to 520.3)	197.2 (178.0 to 218.6)	28.4 (18.1 to 42.4)	0.0 (0.0 to 0.0)	1491.0 (1420.2 to 1566.7)	1023.2 (971.5 to 1078.8)	409.0 (369.1 to 453.3)	58.9 (37.5 to 88.0)	0.0 (0.0 to 0.0)

Country	Year	2018 US Dollars per capita					2018 PPP per capita				
		Health spending	Government health spending	Out-of-pocket health spending	Prepaid private health spending	Development assistance for health	Health spending	Government health spending	Out-of-pocket health spending	Prepaid private health spending	Development assistance for health
Hungary	2002	790.5 (752.2 to 829.1)	545.7 (518.0 to 576.3)	214.9 (194.9 to 236.7)	29.9 (19.0 to 43.6)	0.0 (0.0 to 0.0)	1639.1 (1559.7 to 1719.2)	1131.4 (1074.1 to 1195.0)	445.6 (404.0 to 490.8)	62.0 (39.4 to 90.4)	0.1 (0.1 to 0.1)
Hungary	2003	868.5 (828.5 to 909.1)	601.6 (571.8 to 634.5)	232.4 (210.8 to 254.7)	34.5 (22.5 to 48.9)	0.0 (0.0 to 0.0)	1800.9 (1718.0 to 1885.0)	1247.5 (1185.6 to 1315.5)	481.8 (437.0 to 528.2)	71.5 (46.7 to 101.4)	0.1 (0.1 to 0.1)
Hungary	2004	923.8 (884.6 to 964.3)	642.2 (611.8 to 675.9)	242.2 (220.3 to 264.8)	39.4 (26.5 to 55.4)	0.0 (0.0 to 0.0)	1915.6 (1834.2 to 1999.6)	1331.6 (1268.6 to 1401.5)	502.3 (456.8 to 549.1)	81.6 (54.9 to 114.8)	0.1 (0.1 to 0.1)
Hungary	2005	962.9 (924.1 to 1005.4)	670.0 (640.8 to 701.3)	250.1 (229.3 to 273.0)	42.7 (29.3 to 59.0)	0.0 (0.0 to 0.0)	1996.5 (1916.1 to 2084.7)	1389.2 (1328.6 to 1454.1)	518.7 (475.4 to 566.1)	88.6 (60.8 to 122.3)	0.0 (0.0 to 0.0)
Hungary	2006	976.5 (940.3 to 1017.5)	677.3 (648.2 to 708.3)	252.6 (232.4 to 276.2)	46.6 (32.8 to 64.0)	0.0 (0.0 to 0.0)	2024.8 (1949.7 to 2109.7)	1404.4 (1344.0 to 1468.6)	523.8 (481.9 to 572.6)	96.6 (68.0 to 132.8)	0.0 (0.0 to 0.0)
Hungary	2007	948.9 (913.5 to 985.6)	650.3 (623.8 to 679.9)	250.2 (231.8 to 272.4)	48.4 (35.1 to 66.1)	0.0 (0.0 to 0.0)	1967.4 (1894.1 to 2043.7)	1348.4 (1293.4 to 1409.8)	518.7 (480.6 to 564.8)	100.3 (72.8 to 137.1)	0.0 (0.0 to 0.0)
Hungary	2008	938.9 (903.9 to 975.0)	639.0 (612.0 to 666.2)	249.4 (231.3 to 269.4)	50.5 (37.4 to 68.9)	0.0 (0.0 to 0.0)	1946.9 (1874.3 to 2021.7)	1325.1 (1268.9 to 1381.4)	517.1 (479.6 to 558.7)	104.7 (77.5 to 142.8)	0.0 (0.0 to 0.0)
Hungary	2009	903.4 (869.8 to 938.6)	608.6 (583.4 to 633.5)	243.9 (226.9 to 261.8)	50.9 (38.2 to 67.1)	0.0 (0.0 to 0.0)	1873.1 (1803.5 to 1946.1)	1261.9 (1209.8 to 1313.6)	505.8 (470.4 to 542.8)	105.5 (79.2 to 139.2)	0.0 (0.0 to 0.0)
Hungary	2010	917.7 (881.7 to 955.1)	612.4 (584.3 to 638.0)	252.7 (234.5 to 272.2)	52.6 (39.8 to 69.2)	0.0 (0.0 to 0.0)	1902.8 (1828.1 to 1980.4)	1269.8 (1211.6 to 1323.0)	523.9 (486.1 to 564.4)	109.1 (82.5 to 143.4)	0.0 (0.0 to 0.0)
Hungary	2011	934.2 (898.4 to 971.1)	619.3 (591.4 to 646.1)	262.2 (242.6 to 283.2)	52.7 (39.7 to 70.5)	0.0 (0.0 to 0.0)	1937.0 (1862.9 to 2013.5)	1284.0 (1226.3 to 1339.8)	543.7 (503.0 to 587.3)	109.3 (82.3 to 146.2)	0.0 (0.0 to 0.0)
Hungary	2012	922.2 (886.4 to 959.1)	606.4 (579.0 to 633.1)	264.6 (245.3 to 285.8)	51.0 (38.0 to 69.1)	0.2 (0.2 to 0.2)	1912.3 (1837.9 to 1988.8)	1257.4 (1200.6 to 1312.7)	548.7 (508.6 to 592.6)	105.7 (78.7 to 143.3)	0.5 (0.5 to 0.5)
Hungary	2013	931.4 (894.7 to 970.0)	615.1 (589.0 to 645.0)	267.0 (247.1 to 288.9)	49.3 (36.4 to 66.8)	0.0 (0.0 to 0.0)	1931.2 (1855.2 to 2011.3)	1275.4 (1221.2 to 1337.3)	553.6 (512.3 to 599.0)	102.2 (75.4 to 138.6)	0.1 (0.1 to 0.1)
Hungary	2014	956.4 (922.6 to 994.6)	634.2 (609.5 to 662.8)	274.3 (253.6 to 295.2)	47.9 (34.5 to 65.1)	0.0 (0.0 to 0.0)	1983.2 (1913.0 to 2062.2)	1315.1 (1263.8 to 1374.3)	568.7 (525.9 to 612.1)	99.4 (71.5 to 135.0)	0.0 (0.0 to 0.0)
Hungary	2015	991.3 (956.3 to 1031.0)	657.1 (630.2 to 685.2)	286.7 (265.5 to 309.7)	47.5 (33.2 to 65.9)	0.0 (0.0 to 0.0)	2055.4 (1982.9 to 2137.7)	1362.4 (1306.8 to 1420.8)	594.5 (550.4 to 642.2)	98.5 (68.7 to 136.6)	0.0 (0.0 to 0.0)
Hungary	2016	1028.6 (976.0 to 1081.2)	679.5 (642.0 to 716.6)	301.4 (273.3 to 333.0)	47.8 (31.7 to 68.8)	0.0 (0.0 to 0.0)	2132.9 (2023.6 to 2241.9)	1408.9 (1331.2 to 1485.9)	624.9 (566.6 to 690.5)	99.1 (65.7 to 142.7)	0.0 (0.0 to 0.0)
Hungary	2017	1113.4 (1056.8 to 1169.3)	745.8 (704.0 to 788.0)	317.2 (287.6 to 350.8)	50.4 (33.5 to 72.7)	0.0 (0.0 to 0.0)	2308.6 (2191.3 to 2424.6)	1546.4 (1459.8 to 1633.8)	657.8 (596.4 to 727.5)	104.5 (69.4 to 150.7)	0.0 (0.0 to 0.0)
Hungary	2018	1129.8 (1071.2 to 1190.9)	758.0 (714.4 to 804.2)	320.5 (290.7 to 355.3)	51.3 (34.1 to 73.9)	0.0 (0.0 to 0.0)	2342.6 (2221.2 to 2469.3)	1571.7 (1481.3 to 1667.4)	664.5 (602.8 to 736.7)	106.4 (70.8 to 153.3)	0.0 (0.0 to 0.0)
Hungary	2019	1148.3 (1088.7 to 1208.1)	773.0 (729.0 to 818.1)	323.1 (293.0 to 357.8)	52.2 (34.8 to 75.1)	0.0 (0.0 to 0.0)	2380.9 (2257.3 to 2504.9)	1602.9 (1511.7 to 1696.3)	669.9 (607.6 to 741.9)	108.2 (72.1 to 155.7)	0.0 (0.0 to 0.0)
Hungary	2020	1170.6 (1111.6 to 1230.3)	788.6 (744.2 to 835.6)	328.9 (297.3 to 365.4)	53.1 (35.5 to 76.5)	0.0 (0.0 to 0.0)	2427.3 (2304.9 to 2550.9)	1635.1 (1543.1 to 1732.6)	682.1 (616.4 to 757.7)	110.1 (73.5 to 158.7)	0.0 (0.0 to 0.0)
Hungary	2021	1194.7 (1133.4 to 1256.0)	804.4 (758.9 to 853.5)	336.2 (303.0 to 373.4)	54.1 (36.1 to 78.0)	0.0 (0.0 to 0.0)	2477.2 (2350.1 to 2604.4)	1668.0 (1573.5 to 1769.6)	697.2 (628.3 to 774.2)	112.1 (74.8 to 161.8)	0.0 (0.0 to 0.0)
Hungary	2022	1218.8 (1157.1 to 1285.4)	819.9 (773.7 to 870.2)	343.7 (309.5 to 381.6)	55.2 (36.8 to 79.6)	0.0 (0.0 to 0.0)	2527.2 (2399.2 to 2665.2)	1700.0 (1604.4 to 1804.3)	712.8 (641.8 to 791.3)	114.4 (76.3 to 165.0)	0.0 (0.0 to 0.0)

		2018 US Dollars per capita					2018 PPP per capita				
Country	Year	Health spending	Government health spending	Out-of-pocket health spending	Prepaid private health spending	Development assistance for health	Health spending	Government health spending	Out-of-pocket health spending	Prepaid private health spending	Development assistance for health
Hungary	2023	1243.7 (1178.6 to 1310.8)	835.7 (787.7 to 887.8)	351.6 (317.6 to 391.9)	56.4 (37.6 to 81.3)	0.0 (0.0 to 0.0)	2578.8 (2443.9 to 2717.8)	1732.8 (1633.3 to 1840.8)	729.0 (658.5 to 812.7)	116.9 (78.1 to 168.6)	0.0 (0.0 to 0.0)
Hungary	2024	1269.9 (1203.9 to 1336.7)	852.4 (802.5 to 906.2)	359.8 (323.9 to 399.6)	57.6 (38.4 to 83.1)	0.0 (0.0 to 0.0)	2633.0 (2496.2 to 2771.7)	1767.5 (1664.1 to 1878.9)	746.1 (671.5 to 828.5)	119.5 (79.7 to 172.3)	0.0 (0.0 to 0.0)
Hungary	2025	1297.4 (1228.9 to 1368.4)	870.0 (818.6 to 925.0)	368.4 (331.4 to 410.5)	59.0 (39.3 to 84.8)	0.0 (0.0 to 0.0)	2690.2 (2548.1 to 2837.3)	1804.0 (1697.4 to 1917.9)	763.9 (687.2 to 851.1)	122.3 (81.4 to 175.9)	0.0 (0.0 to 0.0)
Hungary	2026	1325.4 (1255.5 to 1398.7)	887.8 (834.5 to 944.7)	377.2 (337.6 to 422.0)	60.3 (40.2 to 86.7)	0.0 (0.0 to 0.0)	2748.1 (2603.2 to 2900.3)	1840.9 (1730.3 to 1958.8)	782.2 (699.9 to 875.0)	125.1 (83.3 to 179.9)	0.0 (0.0 to 0.0)
Hungary	2027	1354.8 (1280.8 to 1429.3)	906.6 (850.6 to 966.1)	386.4 (343.7 to 432.4)	61.8 (41.1 to 88.8)	0.0 (0.0 to 0.0)	2809.2 (2655.7 to 2963.7)	1879.8 (1763.6 to 2003.2)	801.2 (712.7 to 896.6)	128.1 (85.3 to 184.0)	0.0 (0.0 to 0.0)
Hungary	2028	1385.1 (1307.4 to 1466.2)	926.0 (864.3 to 987.0)	395.8 (349.5 to 445.9)	63.3 (42.2 to 90.9)	0.0 (0.0 to 0.0)	2872.0 (2710.8 to 3040.2)	1920.0 (1792.2 to 2046.5)	820.7 (724.7 to 924.6)	131.3 (87.4 to 188.4)	0.0 (0.0 to 0.0)
Hungary	2029	1415.5 (1328.7 to 1505.2)	945.4 (880.4 to 1012.4)	405.3 (354.9 to 460.0)	64.9 (43.2 to 93.0)	0.0 (0.0 to 0.0)	2935.1 (2755.1 to 3121.0)	1960.3 (1825.4 to 2099.3)	840.3 (736.0 to 953.9)	134.5 (89.5 to 192.9)	0.0 (0.0 to 0.0)
Hungary	2030	1446.3 (1358.8 to 1540.7)	965.1 (894.8 to 1036.7)	414.7 (361.0 to 475.4)	66.4 (44.2 to 95.4)	0.0 (0.0 to 0.0)	2998.8 (2817.5 to 3194.6)	2001.1 (1855.4 to 2149.6)	860.0 (748.6 to 985.7)	137.7 (91.7 to 197.9)	0.0 (0.0 to 0.0)
Hungary	2031	1476.6 (1380.9 to 1579.3)	984.5 (911.9 to 1059.7)	424.1 (366.7 to 490.4)	68.0 (45.3 to 97.6)	0.0 (0.0 to 0.0)	3061.7 (2863.3 to 3274.6)	2041.4 (1890.9 to 2197.3)	879.4 (760.4 to 1016.8)	140.9 (93.9 to 202.5)	0.0 (0.0 to 0.0)
Hungary	2032	1506.5 (1404.9 to 1612.9)	1003.7 (927.0 to 1082.9)	433.3 (371.8 to 504.4)	69.5 (46.3 to 99.8)	0.0 (0.0 to 0.0)	3123.8 (2913.0 to 3344.2)	2081.2 (1922.0 to 2245.4)	898.5 (771.0 to 1045.8)	144.1 (95.9 to 206.9)	0.0 (0.0 to 0.0)
Hungary	2033	1537.0 (1429.3 to 1649.7)	1023.4 (940.6 to 1107.4)	442.6 (377.1 to 521.7)	71.0 (47.2 to 102.0)	0.0 (0.0 to 0.0)	3186.9 (2963.6 to 3420.7)	2121.9 (1950.3 to 2296.2)	917.7 (782.0 to 1081.7)	147.2 (97.8 to 211.4)	0.0 (0.0 to 0.0)
Hungary	2034	1567.5 (1453.9 to 1687.0)	1043.1 (957.5 to 1131.2)	451.8 (381.6 to 535.4)	72.5 (48.0 to 104.0)	0.0 (0.0 to 0.0)	3250.2 (3014.7 to 3497.9)	2162.9 (1985.5 to 2345.5)	936.8 (791.3 to 1110.2)	150.4 (99.5 to 215.5)	0.0 (0.0 to 0.0)
Hungary	2035	1597.9 (1475.1 to 1723.2)	1062.9 (974.9 to 1154.5)	460.9 (386.7 to 549.3)	74.1 (48.8 to 105.9)	0.0 (0.0 to 0.0)	3313.1 (3058.5 to 3573.0)	2203.9 (2021.4 to 2393.9)	955.7 (801.8 to 1138.9)	153.6 (101.3 to 219.5)	0.0 (0.0 to 0.0)
Hungary	2036	1627.7 (1503.9 to 1760.3)	1082.4 (991.2 to 1179.1)	469.8 (392.2 to 564.5)	75.5 (49.8 to 108.2)	0.0 (0.0 to 0.0)	3374.9 (3118.4 to 3650.0)	2244.3 (2055.1 to 2444.8)	974.0 (813.3 to 1170.6)	156.6 (103.3 to 224.4)	0.0 (0.0 to 0.0)
Hungary	2037	1656.9 (1523.5 to 1795.6)	1101.5 (1006.8 to 1201.4)	478.4 (396.1 to 577.3)	77.0 (51.0 to 110.4)	0.0 (0.0 to 0.0)	3435.5 (3159.1 to 3723.2)	2283.9 (2087.6 to 2491.0)	992.1 (821.2 to 1197.0)	159.6 (105.6 to 228.9)	0.0 (0.0 to 0.0)
Hungary	2038	1685.9 (1548.0 to 1830.5)	1120.5 (1024.7 to 1223.5)	487.0 (401.0 to 588.7)	78.4 (52.1 to 112.4)	0.0 (0.0 to 0.0)	3495.7 (3209.7 to 3795.6)	2323.3 (2124.6 to 2537.0)	1009.8 (831.5 to 1220.7)	162.6 (108.0 to 233.1)	0.0 (0.0 to 0.0)
Hungary	2039	1714.8 (1572.0 to 1866.7)	1139.5 (1040.1 to 1245.1)	495.4 (405.7 to 602.2)	79.8 (53.1 to 114.5)	0.0 (0.0 to 0.0)	3555.6 (3259.4 to 3870.6)	2362.7 (2156.7 to 2581.7)	1027.3 (841.3 to 1248.7)	165.6 (110.0 to 237.5)	0.0 (0.0 to 0.0)
Hungary	2040	1743.6 (1594.6 to 1903.9)	1158.6 (1057.4 to 1267.3)	503.7 (410.7 to 613.5)	81.3 (53.9 to 116.8)	0.0 (0.0 to 0.0)	3615.3 (3306.4 to 3947.7)	2402.3 (2192.5 to 2627.8)	1044.5 (851.6 to 1272.1)	168.5 (111.7 to 242.2)	0.0 (0.0 to 0.0)
Hungary	2041	1770.5 (1618.3 to 1930.6)	1176.3 (1071.5 to 1287.0)	511.6 (414.9 to 625.7)	82.6 (54.6 to 119.0)	0.0 (0.0 to 0.0)	3671.0 (3355.5 to 4003.2)	2439.1 (2221.7 to 2668.7)	1060.7 (860.4 to 1297.4)	171.2 (113.2 to 246.7)	0.0 (0.0 to 0.0)
Hungary	2042	1798.5 (1639.4 to 1972.8)	1195.1 (1085.7 to 1309.3)	519.4 (420.5 to 636.7)	84.0 (55.2 to 121.2)	0.0 (0.0 to 0.0)	3729.1 (3399.3 to 4090.6)	2477.9 (2251.1 to 2714.8)	1077.1 (871.9 to 1320.2)	174.1 (114.4 to 251.4)	0.0 (0.0 to 0.0)
Hungary	2043	1826.4 (1661.3 to 2000.9)	1213.9 (1098.4 to 1332.3)	527.2 (426.6 to 646.8)	85.4 (55.8 to 123.5)	0.0 (0.0 to 0.0)	3787.1 (3444.7 to 4148.9)	2516.9 (2277.5 to 2762.5)	1093.2 (884.5 to 1341.2)	177.0 (115.7 to 256.1)	0.0 (0.0 to 0.0)

Country	Year	2018 US Dollars per capita					2018 PPP per capita				
		Health spending	Government health spending	Out-of-pocket health spending	Prepaid private health spending	Development assistance for health	Health spending	Government health spending	Out-of-pocket health spending	Prepaid private health spending	Development assistance for health
Hungary	2044	1855.5 (1683.5 to 2031.5)	1233.6 (1113.3 to 1357.9)	535.1 (432.3 to 657.7)	86.8 (56.6 to 125.5)	0.0 (0.0 to 0.0)	3847.4 (3490.6 to 4212.2)	2557.9 (2308.4 to 2815.6)	1109.5 (896.3 to 1363.8)	180.1 (117.3 to 260.2)	0.0 (0.0 to 0.0)
Hungary	2045	1886.2 (1718.5 to 2071.7)	1254.6 (1129.3 to 1384.1)	543.2 (437.7 to 668.7)	88.5 (57.7 to 127.5)	0.0 (0.0 to 0.0)	3911.1 (3563.4 to 4295.6)	2601.4 (2341.7 to 2870.0)	1126.3 (907.6 to 1386.5)	183.4 (119.5 to 264.4)	0.0 (0.0 to 0.0)
Hungary	2046	1917.0 (1737.3 to 2104.9)	1275.7 (1144.2 to 1411.9)	551.2 (443.9 to 678.7)	90.1 (58.8 to 129.8)	0.0 (0.0 to 0.0)	3974.9 (3602.3 to 4364.4)	2645.1 (2372.6 to 2927.5)	1142.9 (920.5 to 1407.2)	186.9 (121.9 to 269.1)	0.0 (0.0 to 0.0)
Hungary	2047	1949.7 (1765.7 to 2143.8)	1298.2 (1160.7 to 1441.7)	559.5 (451.0 to 687.1)	92.0 (60.0 to 132.6)	0.0 (0.0 to 0.0)	4042.6 (3661.1 to 4445.1)	2691.9 (2406.7 to 2989.3)	1160.1 (935.2 to 1424.8)	190.7 (124.4 to 274.9)	0.0 (0.0 to 0.0)
Hungary	2048	1984.1 (1794.7 to 2188.6)	1322.0 (1176.1 to 1473.7)	568.2 (457.2 to 697.9)	93.9 (61.3 to 135.6)	0.0 (0.0 to 0.0)	4114.1 (3721.2 to 4538.1)	2741.2 (2438.5 to 3055.7)	1178.1 (948.1 to 1447.1)	194.7 (127.1 to 281.1)	0.0 (0.0 to 0.0)
Hungary	2049	2019.6 (1822.9 to 2229.6)	1346.5 (1193.1 to 1507.9)	577.1 (463.1 to 709.5)	95.9 (62.7 to 138.7)	0.0 (0.0 to 0.0)	4187.6 (3779.7 to 4623.0)	2792.0 (2473.9 to 3126.6)	1196.7 (960.2 to 1471.1)	198.9 (129.9 to 287.6)	0.0 (0.0 to 0.0)
Hungary	2050	2054.1 (1853.6 to 2272.8)	1370.2 (1212.9 to 1538.6)	586.0 (470.7 to 720.9)	97.9 (63.9 to 141.7)	0.0 (0.0 to 0.0)	4259.2 (3843.4 to 4712.7)	2841.2 (2515.0 to 3190.2)	1215.0 (975.9 to 1494.9)	203.0 (132.5 to 293.9)	0.0 (0.0 to 0.0)
Iceland	1995	3778.4 (3607.9 to 3939.1)	2909.6 (2752.0 to 3069.5)	868.7 (806.7 to 933.6)	0.0 (0.0 to 0.0)	0.0 (0.0 to 0.0)	2604.0 (2486.5 to 2714.8)	2005.3 (1896.7 to 2115.5)	598.7 (556.0 to 643.5)	0.0 (0.0 to 0.0)	0.0 (0.0 to 0.0)
Iceland	1996	3924.7 (3793.7 to 4053.9)	3030.2 (2908.5 to 3155.5)	894.5 (846.5 to 946.6)	0.0 (0.0 to 0.0)	0.0 (0.0 to 0.0)	2704.9 (2614.6 to 2793.9)	2088.4 (2004.5 to 2174.7)	616.5 (583.4 to 652.4)	0.0 (0.0 to 0.0)	0.0 (0.0 to 0.0)
Iceland	1997	4141.4 (4027.1 to 4267.4)	3217.4 (3108.6 to 3337.7)	924.0 (879.1 to 971.0)	0.0 (0.0 to 0.0)	0.0 (0.0 to 0.0)	2854.3 (2775.5 to 2941.1)	2217.4 (2142.4 to 2300.3)	636.8 (605.9 to 669.2)	0.0 (0.0 to 0.0)	0.0 (0.0 to 0.0)
Iceland	1998	4566.5 (4436.1 to 4697.7)	3598.7 (3483.3 to 3720.1)	967.8 (919.9 to 1017.8)	0.0 (0.0 to 0.0)	0.0 (0.0 to 0.0)	3147.2 (3057.4 to 3237.7)	2480.2 (2400.7 to 2563.9)	667.0 (634.0 to 701.5)	0.0 (0.0 to 0.0)	0.0 (0.0 to 0.0)
Iceland	1999	4934.5 (4795.4 to 5070.1)	3944.5 (3814.5 to 4069.3)	989.9 (942.8 to 1040.6)	0.0 (0.0 to 0.0)	0.0 (0.0 to 0.0)	3400.8 (3305.0 to 3494.3)	2718.6 (2628.9 to 2804.6)	682.3 (649.7 to 717.1)	0.0 (0.0 to 0.0)	0.0 (0.0 to 0.0)
Iceland	2000	5153.9 (5017.0 to 5287.1)	4156.7 (4029.2 to 4278.2)	997.2 (951.3 to 1046.0)	0.0 (0.0 to 0.0)	0.0 (0.0 to 0.0)	3552.1 (3457.7 to 3643.9)	2864.8 (2776.9 to 2948.6)	687.3 (655.6 to 720.9)	0.0 (0.0 to 0.0)	0.0 (0.0 to 0.0)
Iceland	2001	5357.6 (5229.4 to 5486.5)	4362.3 (4245.5 to 4478.8)	995.3 (950.7 to 1042.8)	0.0 (0.0 to 0.0)	0.0 (0.0 to 0.0)	3692.5 (3604.1 to 3781.3)	3006.5 (2926.0 to 3086.8)	686.0 (655.2 to 718.7)	0.0 (0.0 to 0.0)	0.0 (0.0 to 0.0)
Iceland	2002	5535.8 (5407.3 to 5667.2)	4542.1 (4423.4 to 4665.0)	993.5 (950.4 to 1038.5)	0.1 (0.1 to 0.2)	0.0 (0.0 to 0.0)	3815.3 (3726.7 to 3905.8)	3130.4 (3048.6 to 3215.1)	684.7 (655.0 to 715.7)	0.1 (0.1 to 0.1)	0.0 (0.0 to 0.0)
Iceland	2003	5758.1 (5624.5 to 5888.1)	4690.4 (4575.7 to 4815.7)	1057.3 (1007.6 to 1103.5)	10.4 (8.0 to 13.2)	0.0 (0.0 to 0.0)	3968.5 (3876.4 to 4058.1)	3232.6 (3153.6 to 3318.9)	728.7 (694.5 to 760.5)	7.2 (5.5 to 9.1)	0.0 (0.0 to 0.0)
Iceland	2004	6068.1 (5934.0 to 6206.9)	4931.1 (4803.6 to 5064.6)	1068.4 (1022.7 to 1115.1)	68.5 (54.8 to 83.6)	0.0 (0.0 to 0.0)	4182.1 (4089.7 to 4277.8)	3398.5 (3310.6 to 3490.5)	736.4 (704.8 to 768.6)	47.2 (37.8 to 57.6)	0.0 (0.0 to 0.0)
Iceland	2005	6201.6 (6062.9 to 6340.9)	5024.1 (4897.4 to 5153.5)	1045.8 (1001.6 to 1092.9)	131.7 (107.9 to 157.8)	0.0 (0.0 to 0.0)	4274.1 (4178.5 to 4370.1)	3462.6 (3375.3 to 3551.8)	720.8 (690.3 to 753.2)	90.8 (74.4 to 108.7)	0.0 (0.0 to 0.0)
Iceland	2006	6207.8 (6079.4 to 6347.5)	5050.4 (4927.8 to 5180.5)	1014.2 (970.5 to 1059.4)	143.2 (119.6 to 170.1)	0.0 (0.0 to 0.0)	4278.4 (4189.9 to 4374.7)	3480.7 (3396.2 to 3570.4)	699.0 (668.9 to 730.1)	98.7 (82.4 to 117.2)	0.0 (0.0 to 0.0)
Iceland	2007	6411.6 (6273.6 to 6549.3)	5259.7 (5130.6 to 5387.3)	1021.6 (978.2 to 1068.0)	130.3 (109.0 to 156.9)	0.0 (0.0 to 0.0)	4418.8 (4323.8 to 4513.8)	3625.0 (3536.0 to 3712.9)	704.1 (674.2 to 736.1)	89.8 (75.1 to 108.1)	0.0 (0.0 to 0.0)
Iceland	2008	6378.3 (6243.1 to 6516.7)	5243.4 (5113.2 to 5380.5)	1025.0 (981.5 to 1071.2)	109.9 (91.7 to 132.4)	0.0 (0.0 to 0.0)	4395.9 (4302.8 to 4491.3)	3613.7 (3524.0 to 3708.3)	706.5 (676.5 to 738.3)	75.8 (63.2 to 91.2)	0.0 (0.0 to 0.0)

		2018 US Dollars per capita					2018 PPP per capita				
Country	Year	Health spending	Government health spending	Out-of-pocket health spending	Prepaid private health spending	Development assistance for health	Health spending	Government health spending	Out-of-pocket health spending	Prepaid private health spending	Development assistance for health
Iceland	2009	6033.6 (5894.8 to 6175.8)	4924.9 (4794.8 to 5058.0)	1017.3 (974.1 to 1063.8)	91.4 (75.9 to 109.7)	0.0 (0.0 to 0.0)	4158.3 (4062.7 to 4256.4)	3394.2 (3304.6 to 3486.0)	701.1 (671.3 to 733.2)	63.0 (52.3 to 75.6)	0.0 (0.0 to 0.0)
Iceland	2010	5703.5 (5570.2 to 5840.5)	4604.3 (4478.3 to 4725.4)	1018.7 (974.6 to 1067.0)	80.5 (65.8 to 96.3)	0.0 (0.0 to 0.0)	3930.9 (3838.9 to 4025.3)	3173.3 (3086.5 to 3256.8)	702.1 (671.7 to 735.4)	55.5 (45.4 to 66.4)	0.0 (0.0 to 0.0)
Iceland	2011	5638.8 (5515.6 to 5769.2)	4525.7 (4412.6 to 4641.7)	1034.5 (988.1 to 1082.5)	78.6 (64.7 to 93.9)	0.0 (0.0 to 0.0)	3886.2 (3801.3 to 3976.1)	3119.1 (3041.1 to 3199.1)	713.0 (681.0 to 746.0)	54.2 (44.6 to 64.7)	0.0 (0.0 to 0.0)
Iceland	2012	5634.5 (5508.9 to 5761.9)	4510.5 (4387.9 to 4627.7)	1044.3 (999.5 to 1087.7)	79.6 (65.9 to 95.4)	0.0 (0.0 to 0.0)	3883.3 (3796.8 to 3971.1)	3108.7 (3024.1 to 3189.4)	719.7 (688.9 to 749.6)	54.9 (45.4 to 65.8)	0.0 (0.0 to 0.0)
Iceland	2013	5804.1 (5671.2 to 5943.3)	4657.0 (4535.9 to 4794.5)	1062.8 (1018.2 to 1107.3)	84.3 (70.3 to 100.9)	0.0 (0.0 to 0.0)	4000.2 (3908.5 to 4096.1)	3209.6 (3126.1 to 3304.4)	732.5 (701.7 to 763.1)	58.1 (48.4 to 69.6)	0.0 (0.0 to 0.0)
Iceland	2014	5893.3 (5762.8 to 6031.9)	4743.7 (4612.7 to 4877.5)	1061.4 (1016.6 to 1106.2)	88.2 (73.1 to 105.6)	0.0 (0.0 to 0.0)	4061.7 (3971.7 to 4157.2)	3269.3 (3179.1 to 3361.6)	731.5 (700.6 to 762.4)	60.8 (50.4 to 72.8)	0.0 (0.0 to 0.0)
Iceland	2015	6038.6 (5908.2 to 6176.4)	4885.1 (4762.9 to 5015.7)	1060.4 (1015.2 to 1108.6)	93.1 (77.3 to 113.9)	0.0 (0.0 to 0.0)	4161.8 (4071.9 to 4256.7)	3366.8 (3282.6 to 3456.8)	730.8 (699.6 to 764.0)	64.2 (53.3 to 78.5)	0.0 (0.0 to 0.0)
Iceland	2016	6307.2 (6122.7 to 6494.0)	5134.3 (4960.1 to 5309.8)	1072.6 (1010.0 to 1135.4)	100.2 (79.6 to 126.9)	0.0 (0.0 to 0.0)	4346.9 (4219.7 to 4475.6)	3538.6 (3418.5 to 3659.5)	739.3 (696.1 to 782.5)	69.1 (54.8 to 87.5)	0.0 (0.0 to 0.0)
Iceland	2017	6196.7 (6017.7 to 6381.2)	5018.2 (4848.1 to 5188.4)	1077.5 (1013.2 to 1143.9)	101.0 (80.2 to 127.7)	0.0 (0.0 to 0.0)	4270.7 (4147.4 to 4397.9)	3458.5 (3341.3 to 3575.9)	742.6 (698.3 to 788.4)	69.6 (55.3 to 88.0)	0.0 (0.0 to 0.0)
Iceland	2018	6305.6 (6114.6 to 6497.2)	5113.7 (4929.7 to 5290.2)	1088.8 (1023.9 to 1162.8)	103.1 (81.8 to 130.3)	0.0 (0.0 to 0.0)	4345.8 (4214.2 to 4477.9)	3524.4 (3397.6 to 3646.0)	750.4 (705.7 to 801.4)	71.0 (56.4 to 89.8)	0.0 (0.0 to 0.0)
Iceland	2019	6408.6 (6211.7 to 6605.0)	5203.8 (5008.2 to 5394.6)	1100.2 (1031.4 to 1177.8)	104.7 (83.1 to 132.6)	0.0 (0.0 to 0.0)	4416.8 (4281.1 to 4552.2)	3586.4 (3451.7 to 3717.9)	758.2 (710.9 to 811.8)	72.1 (57.3 to 91.4)	0.0 (0.0 to 0.0)
Iceland	2020	6513.2 (6304.1 to 6717.8)	5295.2 (5089.9 to 5501.8)	1111.7 (1041.6 to 1194.5)	106.3 (84.4 to 134.8)	0.0 (0.0 to 0.0)	4488.9 (4344.7 to 4629.9)	3649.5 (3508.0 to 3791.9)	766.2 (717.9 to 823.2)	73.3 (58.2 to 92.9)	0.0 (0.0 to 0.0)
Iceland	2021	6614.0 (6385.2 to 6840.6)	5383.0 (5161.4 to 5601.2)	1123.0 (1045.9 to 1207.1)	107.9 (85.8 to 137.1)	0.0 (0.0 to 0.0)	4558.3 (4400.7 to 4714.6)	3709.9 (3557.2 to 3860.3)	774.0 (720.8 to 831.9)	74.4 (59.1 to 94.5)	0.0 (0.0 to 0.0)
Iceland	2022	6689.9 (6449.8 to 6916.9)	5446.1 (5221.9 to 5677.2)	1134.5 (1052.0 to 1221.3)	109.4 (86.9 to 138.8)	0.0 (0.0 to 0.0)	4610.7 (4445.2 to 4767.1)	3753.4 (3598.9 to 3912.7)	781.9 (725.0 to 841.7)	75.4 (59.9 to 95.6)	0.0 (0.0 to 0.0)
Iceland	2023	6769.2 (6526.0 to 7025.6)	5512.1 (5279.3 to 5755.8)	1146.2 (1058.2 to 1235.6)	110.9 (88.1 to 140.6)	0.0 (0.0 to 0.0)	4665.3 (4497.7 to 4842.0)	3798.9 (3638.5 to 3966.9)	790.0 (729.3 to 851.5)	76.4 (60.7 to 96.9)	0.0 (0.0 to 0.0)
Iceland	2024	6853.5 (6591.7 to 7124.8)	5583.2 (5329.9 to 5855.9)	1157.7 (1069.2 to 1250.2)	112.6 (89.4 to 142.9)	0.0 (0.0 to 0.0)	4723.4 (4543.0 to 4910.4)	3847.9 (3673.4 to 4035.9)	797.9 (736.9 to 861.7)	77.6 (61.6 to 98.5)	0.0 (0.0 to 0.0)
Iceland	2025	6934.2 (6663.8 to 7215.6)	5651.1 (5397.9 to 5921.2)	1168.8 (1074.0 to 1264.9)	114.3 (90.9 to 145.0)	0.0 (0.0 to 0.0)	4779.0 (4592.7 to 4973.0)	3894.7 (3720.3 to 4080.9)	805.5 (740.2 to 871.8)	78.7 (62.6 to 100.0)	0.0 (0.0 to 0.0)
Iceland	2026	7017.8 (6741.2 to 7301.4)	5721.3 (5456.3 to 5991.6)	1180.4 (1079.4 to 1286.2)	116.0 (92.4 to 147.2)	0.0 (0.0 to 0.0)	4836.6 (4646.0 to 5032.1)	3943.1 (3760.5 to 4129.4)	813.5 (744.0 to 886.4)	80.0 (63.7 to 101.5)	0.0 (0.0 to 0.0)
Iceland	2027	7123.3 (6832.6 to 7414.1)	5812.0 (5546.0 to 6111.2)	1193.2 (1086.5 to 1309.7)	118.0 (94.0 to 149.8)	0.0 (0.0 to 0.0)	4909.3 (4709.0 to 5109.8)	4005.6 (3822.3 to 4211.8)	822.4 (748.8 to 902.6)	81.3 (64.8 to 103.2)	0.0 (0.0 to 0.0)
Iceland	2028	7234.1 (6927.2 to 7533.1)	5907.2 (5627.0 to 6209.9)	1206.8 (1090.0 to 1336.8)	120.1 (95.6 to 152.2)	0.0 (0.0 to 0.0)	4985.7 (4774.2 to 5191.8)	4071.2 (3878.1 to 4279.9)	831.7 (751.2 to 921.3)	82.8 (65.9 to 104.9)	0.0 (0.0 to 0.0)
Iceland	2029	7349.6 (7018.7 to 7684.3)	6006.3 (5716.1 to 6320.3)	1221.0 (1097.5 to 1363.0)	122.3 (97.1 to 154.8)	0.0 (0.0 to 0.0)	5065.3 (4837.3 to 5296.0)	4139.5 (3939.5 to 4355.9)	841.5 (756.4 to 939.4)	84.3 (66.9 to 106.7)	0.0 (0.0 to 0.0)

Country	Year	2018 US Dollars per capita					2018 PPP per capita				
		Health spending	Government health spending	Out-of-pocket health spending	Prepaid private health spending	Development assistance for health	Health spending	Government health spending	Out-of-pocket health spending	Prepaid private health spending	Development assistance for health
Iceland	2030	7463.7 (7123.7 to 7830.2)	6104.0 (5797.1 to 6430.7)	1235.2 (1100.8 to 1389.3)	124.5 (98.6 to 157.4)	0.0 (0.0 to 0.0)	5144.0 (4909.6 to 5396.6)	4206.9 (3995.4 to 4432.0)	851.3 (758.6 to 957.5)	85.8 (67.9 to 108.5)	0.0 (0.0 to 0.0)
Iceland	2031	7583.7 (7232.7 to 7956.4)	6206.7 (5889.5 to 6548.4)	1250.2 (1106.9 to 1415.8)	126.8 (100.5 to 160.4)	0.0 (0.0 to 0.0)	5226.7 (4984.8 to 5483.5)	4277.7 (4059.0 to 4513.1)	861.7 (762.9 to 975.8)	87.4 (69.2 to 110.5)	0.0 (0.0 to 0.0)
Iceland	2032	7705.9 (7336.1 to 8100.0)	6311.2 (5975.3 to 6676.0)	1265.6 (1109.7 to 1448.7)	129.1 (102.4 to 163.6)	0.0 (0.0 to 0.0)	5310.9 (5056.0 to 5582.5)	4349.7 (4118.2 to 4601.1)	872.3 (764.8 to 998.5)	89.0 (70.6 to 112.7)	0.0 (0.0 to 0.0)
Iceland	2033	7836.5 (7451.1 to 8254.2)	6422.8 (6071.5 to 6799.0)	1282.0 (1114.3 to 1480.8)	131.6 (104.4 to 166.8)	0.0 (0.0 to 0.0)	5400.9 (5135.3 to 5688.8)	4426.6 (4184.4 to 4685.8)	883.6 (767.9 to 1020.6)	90.7 (71.9 to 114.9)	0.0 (0.0 to 0.0)
Iceland	2034	7961.6 (7567.2 to 8399.2)	6529.5 (6171.4 to 6919.0)	1298.0 (1118.0 to 1515.9)	134.1 (106.3 to 169.8)	0.0 (0.0 to 0.0)	5487.1 (5215.3 to 5788.7)	4500.2 (4253.3 to 4768.6)	894.6 (770.5 to 1044.7)	92.4 (73.2 to 117.0)	0.0 (0.0 to 0.0)
Iceland	2035	8092.8 (7677.2 to 8544.0)	6641.4 (6270.4 to 7055.6)	1314.8 (1125.2 to 1549.6)	136.6 (108.1 to 173.5)	0.0 (0.0 to 0.0)	5577.6 (5291.1 to 5888.5)	4577.2 (4321.6 to 4862.7)	906.2 (775.5 to 1068.0)	94.1 (74.5 to 119.6)	0.0 (0.0 to 0.0)
Iceland	2036	8226.8 (7796.0 to 8714.7)	6755.6 (6370.6 to 7190.6)	1332.0 (1132.3 to 1582.5)	139.2 (110.0 to 177.0)	0.0 (0.0 to 0.0)	5669.9 (5373.0 to 6006.1)	4655.9 (4390.6 to 4955.7)	918.0 (780.4 to 1090.6)	96.0 (75.8 to 122.0)	0.0 (0.0 to 0.0)
Iceland	2037	8367.3 (7913.3 to 8859.2)	6875.2 (6474.3 to 7326.9)	1350.0 (1141.5 to 1611.8)	142.0 (111.9 to 180.5)	0.0 (0.0 to 0.0)	5766.7 (5453.8 to 6105.7)	4738.4 (4462.1 to 5049.7)	930.5 (786.7 to 1110.9)	97.8 (77.2 to 124.4)	0.0 (0.0 to 0.0)
Iceland	2038	8509.8 (8017.0 to 9026.0)	6996.6 (6578.7 to 7465.5)	1368.4 (1152.8 to 1642.9)	144.8 (113.9 to 184.1)	0.0 (0.0 to 0.0)	5864.9 (5525.3 to 6220.7)	4822.0 (4534.0 to 5145.2)	943.1 (794.5 to 1132.3)	99.8 (78.5 to 126.9)	0.0 (0.0 to 0.0)
Iceland	2039	8653.7 (8138.4 to 9196.1)	7119.0 (6685.2 to 7602.9)	1387.1 (1164.6 to 1675.7)	147.6 (115.9 to 188.0)	0.0 (0.0 to 0.0)	5964.1 (5609.0 to 6338.0)	4906.4 (4607.4 to 5239.9)	956.0 (802.6 to 1154.9)	101.7 (79.9 to 129.5)	0.0 (0.0 to 0.0)
Iceland	2040	8799.9 (8248.9 to 9355.1)	7243.2 (6793.0 to 7750.6)	1406.2 (1177.0 to 1708.4)	150.5 (117.9 to 191.5)	0.0 (0.0 to 0.0)	6064.8 (5685.2 to 6447.5)	4992.0 (4681.8 to 5341.7)	969.1 (811.2 to 1177.5)	103.7 (81.3 to 132.0)	0.0 (0.0 to 0.0)
Iceland	2041	8952.9 (8391.1 to 9518.9)	7373.3 (6904.3 to 7894.7)	1426.1 (1190.4 to 1735.5)	153.5 (120.1 to 195.2)	0.0 (0.0 to 0.0)	6170.3 (5783.1 to 6560.4)	5081.7 (4758.5 to 5441.0)	982.9 (820.4 to 1196.1)	105.8 (82.8 to 134.6)	0.0 (0.0 to 0.0)
Iceland	2042	9109.7 (8509.5 to 9709.7)	7506.5 (7017.8 to 8053.7)	1446.6 (1203.8 to 1769.6)	156.6 (122.3 to 199.3)	0.0 (0.0 to 0.0)	6278.4 (5864.8 to 6691.9)	5173.4 (4836.7 to 5550.6)	997.0 (829.7 to 1219.6)	107.9 (84.3 to 137.4)	0.0 (0.0 to 0.0)
Iceland	2043	9268.2 (8645.7 to 9897.2)	7641.0 (7132.5 to 8208.4)	1467.4 (1217.6 to 1798.5)	159.7 (124.7 to 203.5)	0.0 (0.0 to 0.0)	6387.6 (5958.6 to 6821.1)	5266.2 (4915.7 to 5657.2)	1011.3 (839.2 to 1239.5)	110.1 (85.9 to 140.2)	0.0 (0.0 to 0.0)
Iceland	2044	9430.9 (8787.2 to 10094.4)	7779.2 (7246.9 to 8368.3)	1488.7 (1232.5 to 1826.2)	163.0 (127.1 to 207.8)	0.0 (0.0 to 0.0)	6499.7 (6056.1 to 6957.1)	5361.4 (4994.6 to 5767.4)	1026.0 (849.5 to 1258.6)	112.3 (87.6 to 143.2)	0.0 (0.0 to 0.0)
Iceland	2045	9589.0 (8916.7 to 10299.0)	7913.0 (7356.4 to 8539.4)	1509.8 (1248.1 to 1854.9)	166.1 (129.2 to 212.0)	0.0 (0.0 to 0.0)	6608.7 (6145.4 to 7098.0)	5453.6 (5070.0 to 5885.3)	1040.6 (860.2 to 1278.4)	114.5 (89.1 to 146.1)	0.0 (0.0 to 0.0)
Iceland	2046	9750.5 (9050.0 to 10508.2)	8049.7 (7454.2 to 8703.7)	1531.4 (1264.8 to 1881.0)	169.4 (131.5 to 216.1)	0.0 (0.0 to 0.0)	6720.0 (6237.3 to 7242.3)	5547.9 (5137.4 to 5998.5)	1055.5 (871.7 to 1296.4)	116.7 (90.6 to 148.9)	0.0 (0.0 to 0.0)
Iceland	2047	9906.2 (9189.1 to 10660.9)	8181.1 (7547.7 to 8871.4)	1552.6 (1281.7 to 1911.6)	172.5 (134.0 to 220.4)	0.0 (0.0 to 0.0)	6827.3 (6333.1 to 7347.5)	5638.4 (5201.9 to 6114.2)	1070.0 (883.4 to 1317.5)	118.9 (92.3 to 151.9)	0.0 (0.0 to 0.0)
Iceland	2048	10065.8 (9316.2 to 10872.6)	8315.9 (7652.5 to 9018.2)	1574.2 (1299.2 to 1944.1)	175.7 (135.9 to 225.1)	0.0 (0.0 to 0.0)	6937.3 (6420.7 to 7493.4)	5731.3 (5274.1 to 6215.3)	1085.0 (895.4 to 1339.9)	121.1 (93.7 to 155.1)	0.0 (0.0 to 0.0)
Iceland	2049	10227.2 (9440.2 to 11031.3)	8452.2 (7755.1 to 9192.0)	1596.1 (1316.9 to 1974.7)	178.9 (138.4 to 230.0)	0.0 (0.0 to 0.0)	7048.6 (6506.2 to 7602.8)	5825.2 (5344.8 to 6335.1)	1100.0 (907.6 to 1360.9)	123.3 (95.4 to 158.5)	0.0 (0.0 to 0.0)
Iceland	2050	10390.1 (9576.4 to 11232.9)	8589.8 (7860.7 to 9365.7)	1618.1 (1334.1 to 2005.1)	182.2 (141.4 to 234.7)	0.0 (0.0 to 0.0)	7160.8 (6600.0 to 7741.7)	5920.1 (5417.6 to 6454.8)	1115.2 (919.4 to 1381.9)	125.6 (97.4 to 161.7)	0.0 (0.0 to 0.0)

		2018 US Dollars per capita					2018 PPP per capita				
Country	Year	Health spending	Government health spending	Out-of-pocket health spending	Prepaid private health spending	Development assistance for health	Health spending	Government health spending	Out-of-pocket health spending	Prepaid private health spending	Development assistance for health
India	1995	25.9 (20.0 to 32.9)	4.8 (3.7 to 6.3)	19.2 (13.8 to 26.1)	1.4 (0.6 to 2.5)	0.5 (0.5 to 0.5)	98.9 (76.4 to 125.4)	18.5 (14.1 to 23.9)	73.4 (52.7 to 99.5)	5.2 (2.4 to 9.5)	1.7 (1.7 to 1.7)
India	1996	27.0 (20.8 to 34.1)	5.1 (3.9 to 6.5)	19.8 (14.1 to 26.8)	1.4 (0.7 to 2.6)	0.7 (0.7 to 0.7)	103.0 (79.3 to 130.1)	19.3 (14.8 to 24.7)	75.8 (53.9 to 102.3)	5.4 (2.5 to 10.0)	2.5 (2.5 to 2.5)
India	1997	27.9 (21.6 to 35.3)	5.3 (4.1 to 6.8)	20.6 (14.9 to 27.7)	1.5 (0.7 to 2.7)	0.4 (0.4 to 0.4)	106.4 (82.4 to 134.9)	20.3 (15.5 to 26.1)	78.7 (57.0 to 105.7)	5.7 (2.6 to 10.4)	1.7 (1.7 to 1.7)
India	1998	29.0 (22.8 to 36.7)	5.6 (4.3 to 7.3)	21.4 (15.4 to 28.6)	1.6 (0.7 to 2.9)	0.4 (0.4 to 0.4)	110.5 (86.9 to 140.2)	21.6 (16.3 to 27.7)	81.6 (58.9 to 109.1)	6.0 (2.7 to 11.0)	1.4 (1.4 to 1.4)
India	1999	30.2 (23.9 to 38.3)	6.0 (4.6 to 7.7)	22.0 (15.9 to 29.8)	1.7 (0.8 to 3.0)	0.5 (0.5 to 0.5)	115.4 (91.2 to 146.3)	23.0 (17.5 to 29.2)	84.1 (60.9 to 113.8)	6.3 (2.9 to 11.5)	2.0 (2.0 to 2.0)
India	2000	31.1 (24.8 to 39.2)	6.1 (4.6 to 7.9)	22.6 (16.6 to 30.4)	1.7 (0.8 to 3.1)	0.6 (0.6 to 0.6)	118.7 (94.7 to 149.6)	23.4 (17.6 to 30.0)	86.4 (63.3 to 115.9)	6.5 (3.0 to 11.9)	2.4 (2.4 to 2.4)
India	2001	32.2 (25.6 to 40.8)	6.2 (4.7 to 8.0)	23.6 (17.3 to 31.9)	1.8 (0.8 to 3.4)	0.6 (0.6 to 0.6)	123.1 (97.9 to 155.7)	23.7 (17.9 to 30.7)	90.3 (66.1 to 121.6)	6.8 (3.0 to 12.9)	2.3 (2.3 to 2.3)
India	2002	33.0 (26.2 to 41.5)	6.2 (4.7 to 8.1)	24.1 (17.6 to 32.6)	2.0 (0.9 to 3.7)	0.7 (0.7 to 0.7)	125.8 (100.2 to 158.6)	23.7 (17.8 to 30.9)	92.1 (67.2 to 124.4)	7.5 (3.3 to 14.2)	2.6 (2.6 to 2.6)
India	2003	33.7 (26.8 to 42.2)	6.4 (4.9 to 8.3)	24.7 (17.9 to 32.7)	2.1 (0.9 to 4.0)	0.5 (0.5 to 0.5)	128.8 (102.2 to 161.2)	24.5 (18.7 to 31.8)	94.4 (68.2 to 124.8)	8.1 (3.6 to 15.2)	1.8 (1.8 to 1.8)
India	2004	34.9 (27.6 to 43.5)	6.7 (5.1 to 8.6)	25.3 (18.4 to 33.5)	2.2 (1.0 to 4.2)	0.7 (0.7 to 0.7)	133.2 (105.5 to 166.0)	25.5 (19.4 to 32.8)	96.6 (70.4 to 128.0)	8.6 (3.7 to 16.2)	2.5 (2.5 to 2.5)
India	2005	36.3 (28.8 to 45.2)	7.2 (5.5 to 9.3)	26.1 (18.9 to 34.5)	2.2 (1.0 to 4.3)	0.7 (0.7 to 0.7)	138.5 (110.1 to 172.6)	27.6 (20.8 to 35.6)	99.7 (72.3 to 131.8)	8.6 (3.8 to 16.6)	2.6 (2.6 to 2.6)
India	2006	37.5 (29.6 to 46.5)	7.8 (6.0 to 10.1)	26.8 (19.3 to 35.3)	2.4 (1.1 to 4.6)	0.5 (0.5 to 0.5)	143.2 (113.1 to 177.6)	29.8 (22.7 to 38.5)	102.4 (73.8 to 134.6)	9.1 (4.1 to 17.4)	1.9 (1.9 to 1.9)
India	2007	39.6 (31.3 to 48.9)	8.6 (6.5 to 11.2)	27.7 (20.1 to 36.7)	2.6 (1.2 to 5.1)	0.7 (0.7 to 0.7)	151.3 (119.6 to 186.9)	32.7 (24.9 to 42.6)	105.9 (76.8 to 140.3)	10.1 (4.6 to 19.3)	2.6 (2.6 to 2.6)
India	2008	40.6 (32.3 to 50.1)	9.2 (7.0 to 12.0)	27.9 (20.3 to 37.1)	2.8 (1.3 to 5.3)	0.7 (0.7 to 0.7)	155.1 (123.2 to 191.1)	35.3 (26.7 to 45.7)	106.6 (77.5 to 141.5)	10.6 (4.9 to 20.1)	2.6 (2.6 to 2.6)
India	2009	42.8 (34.1 to 52.8)	10.3 (7.9 to 13.4)	28.6 (20.8 to 38.2)	3.1 (1.5 to 5.9)	0.7 (0.7 to 0.7)	163.3 (130.3 to 201.5)	39.4 (30.2 to 51.3)	109.3 (79.5 to 145.9)	11.8 (5.7 to 22.4)	2.9 (2.9 to 2.9)
India	2010	45.0 (36.0 to 54.7)	11.4 (8.7 to 14.9)	29.4 (21.4 to 38.9)	3.5 (1.7 to 6.5)	0.8 (0.8 to 0.8)	172.0 (137.4 to 208.9)	43.6 (33.0 to 56.7)	112.3 (81.8 to 148.4)	13.2 (6.5 to 24.6)	2.9 (2.9 to 2.9)
India	2011	47.0 (37.9 to 57.0)	12.3 (9.2 to 15.9)	30.2 (21.9 to 39.6)	3.8 (1.8 to 6.9)	0.8 (0.8 to 0.8)	179.5 (144.8 to 217.8)	46.8 (35.1 to 60.6)	115.2 (83.7 to 151.3)	14.4 (6.9 to 26.3)	3.1 (3.1 to 3.1)
India	2012	49.4 (39.5 to 60.1)	12.7 (9.6 to 16.4)	32.0 (23.1 to 41.8)	4.1 (2.0 to 7.6)	0.6 (0.6 to 0.6)	188.6 (150.7 to 229.3)	48.6 (36.6 to 62.7)	122.0 (88.0 to 159.7)	15.5 (7.4 to 29.0)	2.4 (2.4 to 2.4)
India	2013	53.4 (42.6 to 64.7)	13.1 (9.9 to 17.1)	35.1 (25.1 to 46.5)	4.4 (2.1 to 8.2)	0.7 (0.7 to 0.7)	204.0 (162.8 to 247.1)	50.1 (37.8 to 65.3)	134.2 (95.9 to 177.5)	16.9 (8.0 to 31.5)	2.9 (2.9 to 2.9)
India	2014	56.4 (44.9 to 68.3)	13.9 (10.5 to 18.0)	37.2 (26.7 to 49.4)	4.9 (2.3 to 9.3)	0.5 (0.5 to 0.5)	215.5 (171.3 to 260.7)	53.0 (40.0 to 68.8)	142.0 (102.0 to 188.5)	18.8 (9.0 to 35.6)	1.7 (1.7 to 1.7)
India	2015	60.7 (48.5 to 74.4)	15.1 (11.4 to 19.6)	39.3 (28.4 to 52.7)	5.6 (2.7 to 10.5)	0.7 (0.7 to 0.7)	231.6 (185.1 to 284.0)	57.7 (43.4 to 74.9)	150.2 (108.3 to 201.1)	21.2 (10.1 to 40.1)	2.5 (2.5 to 2.5)

Country	Year	2018 US Dollars per capita					2018 PPP per capita				
		Health spending	Government health spending	Out-of-pocket health spending	Prepaid private health spending	Development assistance for health	Health spending	Government health spending	Out-of-pocket health spending	Prepaid private health spending	Development assistance for health
India	2016	64.6 (52.0 to 79.8)	16.3 (12.3 to 21.0)	41.6 (29.9 to 55.8)	6.1 (2.9 to 11.1)	0.5 (0.5 to 0.5)	246.7 (198.7 to 304.5)	62.3 (47.0 to 80.3)	158.9 (114.0 to 213.0)	23.4 (11.2 to 42.5)	2.1 (2.1 to 2.1)
India	2017	68.2 (54.6 to 84.5)	17.6 (13.2 to 22.5)	43.5 (31.2 to 58.0)	6.5 (3.1 to 11.8)	0.6 (0.6 to 0.6)	260.2 (208.5 to 322.5)	67.0 (50.4 to 86.1)	166.0 (118.9 to 221.5)	24.8 (11.9 to 45.1)	2.4 (2.4 to 2.4)
India	2018	70.7 (57.0 to 87.1)	18.3 (13.8 to 23.6)	45.0 (32.3 to 60.5)	6.8 (3.2 to 12.3)	0.6 (0.6 to 0.6)	269.9 (217.6 to 332.6)	70.0 (52.7 to 90.1)	171.8 (123.2 to 230.8)	25.9 (12.4 to 46.9)	2.2 (2.2 to 2.2)
India	2019	73.3 (58.5 to 91.9)	19.1 (14.4 to 24.7)	46.5 (33.4 to 62.9)	7.0 (3.4 to 12.8)	0.6 (0.5 to 0.6)	279.7 (223.3 to 350.8)	73.1 (55.0 to 94.3)	177.6 (127.7 to 240.3)	26.9 (12.9 to 48.7)	2.2 (2.1 to 2.4)
India	2020	76.0 (61.9 to 93.4)	20.0 (15.1 to 25.8)	48.1 (34.6 to 64.8)	7.3 (3.5 to 13.3)	0.6 (0.5 to 0.6)	290.2 (236.4 to 356.5)	76.3 (57.5 to 98.6)	183.6 (132.1 to 247.5)	28.0 (13.3 to 50.7)	2.3 (2.1 to 2.4)
India	2021	78.8 (62.8 to 97.8)	20.9 (15.7 to 27.0)	49.7 (35.8 to 67.1)	7.6 (3.6 to 13.8)	0.6 (0.5 to 0.7)	300.9 (239.8 to 373.4)	79.6 (59.9 to 102.9)	189.9 (136.8 to 256.0)	29.1 (13.8 to 52.8)	2.3 (2.1 to 2.5)
India	2022	81.8 (66.6 to 100.4)	21.8 (16.4 to 28.0)	51.5 (37.1 to 69.0)	7.9 (3.7 to 14.4)	0.6 (0.5 to 0.7)	312.2 (254.1 to 383.3)	83.2 (62.8 to 106.8)	196.4 (141.5 to 263.6)	30.3 (14.3 to 55.0)	2.4 (2.1 to 2.6)
India	2023	84.9 (68.6 to 107.2)	22.8 (17.1 to 29.4)	53.3 (38.3 to 71.6)	8.3 (3.9 to 15.0)	0.6 (0.5 to 0.7)	324.2 (261.9 to 409.2)	87.0 (65.3 to 112.1)	203.3 (146.4 to 273.4)	31.5 (14.9 to 57.4)	2.4 (2.1 to 2.7)
India	2024	88.2 (72.0 to 108.2)	23.8 (17.9 to 30.6)	55.1 (39.6 to 74.2)	8.6 (4.1 to 15.7)	0.6 (0.6 to 0.7)	336.8 (274.9 to 413.2)	91.0 (68.4 to 117.0)	210.5 (151.2 to 283.2)	32.9 (15.5 to 59.8)	2.4 (2.1 to 2.8)
India	2025	91.6 (73.2 to 114.5)	24.9 (18.8 to 32.0)	57.1 (40.9 to 76.8)	9.0 (4.2 to 16.3)	0.7 (0.6 to 0.8)	349.8 (279.4 to 437.1)	95.2 (71.9 to 122.0)	217.8 (156.0 to 293.1)	34.3 (16.2 to 62.3)	2.5 (2.1 to 2.9)
India	2026	95.1 (77.1 to 117.4)	26.1 (19.7 to 33.5)	59.0 (42.5 to 79.3)	9.4 (4.4 to 17.0)	0.7 (0.6 to 0.8)	363.2 (294.1 to 448.3)	99.5 (75.1 to 127.7)	225.4 (162.2 to 302.8)	35.7 (17.0 to 64.9)	2.5 (2.1 to 3.0)
India	2027	98.8 (78.3 to 123.8)	27.2 (20.5 to 34.9)	61.1 (44.1 to 82.0)	9.7 (4.6 to 17.8)	0.7 (0.6 to 0.8)	377.0 (299.0 to 472.7)	104.0 (78.3 to 133.2)	233.2 (168.2 to 313.1)	37.2 (17.7 to 67.8)	2.6 (2.2 to 3.1)
India	2028	102.4 (82.3 to 126.7)	28.4 (21.4 to 36.3)	63.2 (45.4 to 85.0)	10.1 (4.9 to 18.5)	0.7 (0.6 to 0.8)	390.9 (314.2 to 483.6)	108.5 (81.6 to 138.7)	241.1 (173.2 to 324.4)	38.7 (18.5 to 70.6)	2.6 (2.2 to 3.2)
India	2029	106.2 (85.5 to 131.6)	29.7 (22.3 to 38.1)	65.3 (46.9 to 88.0)	10.5 (5.1 to 19.3)	0.7 (0.6 to 0.9)	405.3 (326.4 to 502.3)	113.2 (85.0 to 145.3)	249.1 (178.9 to 335.8)	40.3 (19.4 to 73.6)	2.7 (2.2 to 3.3)
India	2030	110.0 (88.0 to 136.1)	30.9 (23.2 to 39.7)	67.4 (48.3 to 90.7)	11.0 (5.3 to 20.1)	0.7 (0.6 to 0.9)	420.0 (336.0 to 519.7)	118.1 (88.7 to 151.7)	257.3 (184.3 to 346.4)	41.9 (20.1 to 76.7)	2.8 (2.3 to 3.4)
India	2031	113.9 (90.8 to 141.5)	32.2 (24.2 to 41.4)	69.6 (49.9 to 93.7)	11.4 (5.5 to 20.9)	0.7 (0.6 to 0.9)	435.0 (346.7 to 540.0)	123.1 (92.4 to 158.1)	265.5 (190.3 to 357.8)	43.5 (20.9 to 79.9)	2.8 (2.3 to 3.5)
India	2032	118.0 (94.5 to 146.5)	33.6 (25.2 to 43.3)	71.8 (51.2 to 97.1)	11.9 (5.7 to 21.8)	0.8 (0.6 to 1.0)	450.3 (360.8 to 559.1)	128.3 (96.2 to 165.3)	273.9 (195.4 to 370.8)	45.2 (21.6 to 83.2)	2.9 (2.3 to 3.7)
India	2033	122.1 (96.2 to 151.8)	35.0 (26.3 to 45.2)	74.0 (52.7 to 100.3)	12.3 (5.9 to 22.7)	0.8 (0.6 to 1.0)	466.0 (367.2 to 579.5)	133.7 (100.4 to 172.7)	282.4 (201.3 to 382.8)	47.0 (22.4 to 86.5)	2.9 (2.3 to 3.8)
India	2034	126.3 (100.6 to 158.0)	36.5 (27.4 to 47.1)	76.3 (54.2 to 104.1)	12.8 (6.1 to 23.5)	0.8 (0.6 to 1.0)	482.2 (384.2 to 603.0)	139.2 (104.7 to 179.6)	291.1 (207.0 to 397.5)	48.9 (23.3 to 89.8)	3.0 (2.4 to 3.9)
India	2035	130.7 (103.6 to 163.0)	38.0 (28.6 to 49.0)	78.6 (55.3 to 107.7)	13.3 (6.3 to 24.4)	0.8 (0.6 to 1.1)	498.8 (395.5 to 622.3)	145.0 (109.1 to 186.9)	299.9 (211.3 to 411.2)	50.8 (24.2 to 93.1)	3.1 (2.4 to 4.0)
India	2036	135.1 (106.8 to 169.2)	39.6 (29.8 to 50.9)	80.9 (56.8 to 111.3)	13.8 (6.6 to 25.3)	0.8 (0.6 to 1.1)	515.9 (407.6 to 645.8)	151.2 (113.6 to 194.5)	308.8 (216.9 to 424.9)	52.8 (25.1 to 96.6)	3.2 (2.5 to 4.3)

		2018 US Dollars per capita					2018 PPP per capita				
Country	Year	Health spending	Government health spending	Out-of-pocket health spending	Prepaid private health spending	Development assistance for health	Health spending	Government health spending	Out-of-pocket health spending	Prepaid private health spending	Development assistance for health
India	2037	139.8 (111.5 to 175.0)	41.3 (31.0 to 53.2)	83.3 (58.4 to 115.2)	14.4 (6.9 to 26.2)	0.9 (0.7 to 1.2)	533.5 (425.6 to 667.9)	157.6 (118.4 to 203.2)	317.9 (223.1 to 439.6)	54.8 (26.2 to 100.2)	3.3 (2.5 to 4.4)
India	2038	144.5 (113.1 to 182.4)	43.0 (32.3 to 55.6)	85.7 (59.9 to 119.1)	14.9 (7.1 to 27.2)	0.9 (0.7 to 1.2)	551.5 (431.9 to 696.3)	164.2 (123.4 to 212.3)	327.0 (228.7 to 454.8)	57.0 (27.2 to 103.9)	3.3 (2.5 to 4.6)
India	2039	149.3 (118.0 to 189.6)	44.8 (33.6 to 58.0)	88.1 (61.7 to 122.7)	15.5 (7.4 to 28.2)	0.9 (0.7 to 1.2)	569.9 (450.4 to 723.9)	171.0 (128.4 to 221.3)	336.3 (235.5 to 468.5)	59.2 (28.3 to 107.8)	3.4 (2.6 to 4.8)
India	2040	154.3 (121.2 to 194.0)	46.7 (35.0 to 60.4)	90.6 (63.3 to 126.4)	16.1 (7.7 to 29.3)	0.9 (0.7 to 1.3)	588.9 (462.6 to 740.7)	178.2 (133.5 to 230.5)	345.7 (241.7 to 482.4)	61.5 (29.3 to 111.9)	3.5 (2.7 to 4.9)
India	2041	159.3 (125.6 to 200.1)	48.6 (36.3 to 63.1)	93.0 (64.9 to 130.2)	16.7 (7.9 to 30.5)	0.9 (0.7 to 1.3)	608.2 (479.6 to 763.8)	185.6 (138.6 to 240.7)	355.1 (247.6 to 497.1)	63.9 (30.3 to 116.3)	3.6 (2.7 to 5.0)
India	2042	164.6 (128.8 to 207.5)	50.7 (37.7 to 65.9)	95.5 (66.6 to 134.1)	17.4 (8.2 to 31.7)	1.0 (0.7 to 1.4)	628.2 (491.6 to 792.3)	193.4 (143.9 to 251.5)	364.7 (254.3 to 511.9)	66.4 (31.4 to 121.2)	3.7 (2.7 to 5.3)
India	2043	169.9 (134.2 to 214.6)	52.8 (39.2 to 69.0)	98.1 (68.4 to 138.5)	18.1 (8.6 to 33.1)	1.0 (0.7 to 1.4)	648.7 (512.3 to 819.1)	201.5 (149.5 to 263.4)	374.4 (261.1 to 528.9)	69.0 (32.7 to 126.3)	3.8 (2.8 to 5.5)
India	2044	175.3 (138.3 to 221.4)	55.0 (40.7 to 72.0)	100.6 (70.0 to 142.3)	18.8 (8.9 to 34.3)	1.0 (0.7 to 1.5)	669.4 (527.8 to 845.1)	209.8 (155.3 to 274.8)	384.0 (267.3 to 543.2)	71.7 (33.9 to 131.0)	3.9 (2.8 to 5.7)
India	2045	181.0 (143.3 to 227.4)	57.3 (42.3 to 75.3)	103.1 (71.7 to 146.0)	19.5 (9.2 to 35.6)	1.1 (0.8 to 1.6)	690.8 (547.2 to 868.2)	218.6 (161.5 to 287.5)	393.8 (273.5 to 557.3)	74.4 (35.2 to 135.9)	4.0 (2.9 to 6.0)
India	2046	186.6 (147.2 to 234.7)	59.6 (43.9 to 78.4)	105.7 (73.2 to 149.8)	20.2 (9.6 to 36.9)	1.1 (0.8 to 1.7)	712.2 (561.8 to 895.9)	227.5 (167.6 to 299.4)	403.3 (279.5 to 571.8)	77.2 (36.5 to 141.0)	4.1 (3.0 to 6.4)
India	2047	192.2 (151.3 to 241.7)	62.0 (45.5 to 81.8)	108.2 (74.8 to 153.9)	21.0 (9.9 to 38.4)	1.1 (0.8 to 1.8)	733.8 (577.7 to 922.6)	236.7 (173.9 to 312.1)	412.9 (285.4 to 587.4)	80.0 (37.8 to 146.4)	4.3 (3.0 to 6.7)
India	2048	198.1 (154.8 to 247.0)	64.5 (47.2 to 85.2)	110.7 (76.3 to 157.6)	21.7 (10.2 to 39.8)	1.2 (0.8 to 1.9)	756.1 (591.1 to 943.0)	246.1 (180.3 to 325.2)	422.6 (291.4 to 601.4)	83.0 (39.1 to 152.0)	4.4 (3.1 to 7.1)
India	2049	204.0 (159.0 to 257.1)	67.0 (48.9 to 88.7)	113.3 (77.9 to 160.7)	22.5 (10.6 to 41.3)	1.2 (0.8 to 1.9)	778.7 (607.1 to 981.6)	255.8 (186.8 to 338.7)	432.4 (297.5 to 613.5)	85.9 (40.4 to 157.8)	4.6 (3.2 to 7.4)
India	2050	210.0 (163.4 to 262.2)	69.6 (50.7 to 92.3)	115.9 (79.5 to 164.2)	23.3 (10.9 to 42.9)	1.2 (0.9 to 2.0)	801.6 (623.7 to 1000.8)	265.6 (193.5 to 352.4)	442.3 (303.6 to 627.0)	89.0 (41.8 to 163.6)	4.7 (3.3 to 7.6)
Indonesia	1995	45.4 (35.6 to 58.0)	13.1 (9.9 to 16.9)	18.6 (13.4 to 25.5)	12.5 (6.2 to 22.0)	1.1 (1.1 to 1.1)	151.6 (119.0 to 193.9)	43.7 (33.1 to 56.5)	62.3 (44.8 to 85.2)	41.9 (20.7 to 73.7)	3.7 (3.7 to 3.7)
Indonesia	1996	47.7 (37.3 to 60.5)	14.0 (10.7 to 18.0)	19.7 (14.2 to 27.2)	13.4 (6.7 to 23.8)	0.6 (0.6 to 0.6)	159.5 (124.8 to 202.1)	46.8 (35.8 to 60.3)	66.0 (47.5 to 90.8)	44.7 (22.3 to 79.6)	2.1 (2.1 to 2.1)
Indonesia	1997	48.9 (38.6 to 62.4)	14.3 (11.1 to 18.6)	20.3 (14.7 to 27.8)	13.6 (6.9 to 24.4)	0.7 (0.7 to 0.7)	163.6 (128.9 to 208.6)	47.9 (37.0 to 62.2)	67.9 (49.1 to 92.9)	45.6 (22.9 to 81.5)	2.2 (2.2 to 2.2)
Indonesia	1998	45.6 (35.9 to 58.2)	13.1 (10.1 to 16.8)	19.4 (14.1 to 26.2)	12.5 (6.4 to 22.6)	0.6 (0.6 to 0.6)	152.4 (119.9 to 194.5)	43.7 (33.7 to 56.2)	64.8 (47.1 to 87.6)	41.7 (21.3 to 75.5)	2.1 (2.1 to 2.1)
Indonesia	1999	45.9 (36.5 to 57.9)	13.4 (10.3 to 17.1)	19.7 (14.3 to 26.8)	12.1 (6.4 to 21.8)	0.7 (0.7 to 0.7)	153.4 (122.1 to 193.5)	44.8 (34.3 to 57.2)	65.8 (47.9 to 89.8)	40.4 (21.5 to 72.8)	2.4 (2.4 to 2.4)
Indonesia	2000	47.3 (38.6 to 58.6)	14.0 (10.6 to 17.8)	19.5 (14.3 to 26.6)	11.1 (5.9 to 19.8)	2.7 (2.7 to 2.7)	158.3 (129.1 to 196.0)	46.8 (35.6 to 59.7)	65.3 (47.8 to 88.9)	37.0 (19.6 to 66.1)	9.2 (9.2 to 9.2)
Indonesia	2001	47.6 (38.7 to 59.1)	15.5 (11.6 to 19.6)	20.4 (14.9 to 27.2)	10.8 (5.6 to 19.0)	0.9 (0.9 to 0.9)	159.1 (129.4 to 197.4)	51.7 (38.8 to 65.5)	68.2 (49.9 to 91.0)	36.0 (18.7 to 63.5)	3.1 (3.1 to 3.1)

Country	Year	2018 US Dollars per capita					2018 PPP per capita				
		Health spending	Government health spending	Out-of-pocket health spending	Prepaid private health spending	Development assistance for health	Health spending	Government health spending	Out-of-pocket health spending	Prepaid private health spending	Development assistance for health
Indonesia	2002	48.9 (39.5 to 60.1)	16.2 (12.2 to 20.7)	21.8 (16.0 to 28.5)	10.2 (5.4 to 17.7)	0.6 (0.6 to 0.6)	163.4 (132.0 to 201.0)	54.2 (40.9 to 69.2)	72.9 (53.5 to 95.1)	34.2 (18.2 to 59.1)	2.0 (2.0 to 2.0)
Indonesia	2003	52.9 (42.8 to 64.0)	17.6 (13.4 to 22.1)	24.2 (18.0 to 31.5)	10.3 (5.4 to 18.0)	0.8 (0.8 to 0.8)	176.8 (142.9 to 213.9)	58.7 (44.8 to 74.0)	80.8 (60.1 to 105.4)	34.5 (18.1 to 60.1)	2.8 (2.8 to 2.8)
Indonesia	2004	56.3 (45.7 to 66.9)	18.5 (14.3 to 23.3)	26.6 (19.9 to 34.5)	10.3 (5.4 to 17.8)	0.9 (0.9 to 0.9)	188.1 (152.8 to 223.7)	61.7 (47.8 to 78.0)	88.8 (66.5 to 115.3)	34.5 (18.2 to 59.5)	3.0 (3.0 to 3.0)
Indonesia	2005	62.2 (50.8 to 74.3)	19.7 (15.2 to 24.8)	31.1 (23.4 to 41.0)	10.6 (5.6 to 18.4)	0.9 (0.9 to 0.9)	208.0 (169.7 to 248.4)	65.7 (50.7 to 83.0)	103.9 (78.1 to 137.1)	35.3 (18.7 to 61.7)	3.1 (3.1 to 3.1)
Indonesia	2006	68.9 (56.9 to 82.5)	21.8 (16.9 to 27.8)	34.5 (26.2 to 45.7)	11.2 (6.0 to 19.6)	1.3 (1.3 to 1.3)	230.3 (190.0 to 275.7)	72.9 (56.4 to 92.8)	115.5 (87.5 to 152.9)	37.5 (20.0 to 65.6)	4.4 (4.4 to 4.4)
Indonesia	2007	74.8 (61.3 to 88.9)	24.3 (18.8 to 30.8)	37.2 (28.2 to 49.8)	12.1 (6.5 to 20.8)	1.1 (1.1 to 1.1)	250.1 (205.0 to 297.3)	81.2 (63.0 to 103.0)	124.5 (94.2 to 166.5)	40.6 (21.7 to 69.7)	3.8 (3.8 to 3.8)
Indonesia	2008	78.3 (64.3 to 92.8)	25.3 (20.1 to 32.1)	38.7 (29.3 to 50.8)	12.8 (7.0 to 21.6)	1.5 (1.5 to 1.5)	261.9 (214.9 to 310.3)	84.6 (67.1 to 107.2)	129.5 (97.8 to 169.7)	42.8 (23.3 to 72.0)	5.0 (5.0 to 5.0)
Indonesia	2009	80.4 (66.6 to 96.3)	25.4 (20.1 to 32.0)	40.6 (30.8 to 52.7)	13.1 (7.2 to 21.5)	1.3 (1.3 to 1.3)	268.9 (222.5 to 321.9)	84.9 (67.1 to 107.0)	135.8 (103.0 to 176.2)	43.8 (24.1 to 71.8)	4.3 (4.3 to 4.3)
Indonesia	2010	83.8 (69.0 to 100.4)	25.1 (19.8 to 31.7)	44.1 (33.3 to 56.1)	13.4 (7.2 to 22.3)	1.2 (1.2 to 1.2)	280.0 (230.7 to 335.5)	83.9 (66.1 to 105.9)	147.3 (111.3 to 187.4)	44.8 (24.2 to 74.7)	3.9 (3.9 to 3.9)
Indonesia	2011	89.1 (73.9 to 106.7)	26.8 (21.0 to 33.6)	46.7 (35.1 to 59.5)	14.3 (7.5 to 24.0)	1.3 (1.3 to 1.3)	297.8 (247.0 to 356.5)	89.6 (70.3 to 112.3)	156.1 (117.5 to 198.9)	47.8 (25.1 to 80.3)	4.2 (4.2 to 4.2)
Indonesia	2012	93.2 (77.3 to 112.0)	29.1 (22.9 to 36.5)	47.2 (36.1 to 60.6)	15.7 (8.2 to 27.2)	1.3 (1.3 to 1.3)	311.7 (258.5 to 374.4)	97.1 (76.6 to 121.9)	157.9 (120.6 to 202.7)	52.4 (27.5 to 91.0)	4.3 (4.3 to 4.3)
Indonesia	2013	98.7 (82.4 to 118.9)	32.2 (25.2 to 40.4)	47.2 (35.9 to 60.5)	17.8 (9.2 to 30.6)	1.4 (1.4 to 1.4)	329.9 (275.4 to 397.6)	107.7 (84.2 to 135.0)	157.9 (120.0 to 202.1)	59.5 (30.7 to 102.4)	4.8 (4.8 to 4.8)
Indonesia	2014	104.2 (86.4 to 125.6)	36.5 (28.6 to 45.9)	46.7 (34.9 to 60.8)	20.2 (10.3 to 35.4)	0.8 (0.8 to 0.8)	348.4 (288.8 to 420.0)	122.0 (95.6 to 153.6)	156.0 (116.8 to 203.4)	67.5 (34.4 to 118.3)	2.8 (2.8 to 2.8)
Indonesia	2015	109.3 (90.3 to 131.2)	41.4 (32.5 to 52.0)	46.1 (34.2 to 60.6)	21.0 (10.7 to 36.9)	0.8 (0.8 to 0.8)	365.3 (301.9 to 438.7)	138.4 (108.7 to 173.7)	154.2 (114.3 to 202.7)	70.1 (35.7 to 123.5)	2.6 (2.6 to 2.6)
Indonesia	2016	116.0 (96.2 to 140.7)	46.6 (37.0 to 58.7)	46.5 (34.3 to 61.8)	22.1 (11.4 to 38.5)	0.8 (0.8 to 0.8)	387.6 (321.5 to 470.4)	155.7 (123.8 to 196.4)	155.4 (114.8 to 206.6)	73.8 (38.0 to 128.8)	2.7 (2.7 to 2.7)
Indonesia	2017	119.5 (98.8 to 144.6)	47.7 (37.9 to 60.2)	47.9 (35.5 to 63.7)	22.9 (11.8 to 40.0)	0.9 (0.9 to 0.9)	399.4 (330.2 to 483.5)	159.5 (126.7 to 201.1)	160.2 (118.7 to 212.8)	76.5 (39.4 to 133.8)	3.2 (3.2 to 3.2)
Indonesia	2018	123.2 (101.6 to 149.3)	49.5 (39.3 to 62.4)	49.2 (36.5 to 65.1)	23.7 (12.2 to 41.5)	0.9 (0.9 to 0.9)	411.9 (339.8 to 498.9)	165.4 (131.4 to 208.5)	164.3 (122.1 to 217.8)	79.3 (40.7 to 138.7)	3.0 (2.9 to 3.0)
Indonesia	2019	127.1 (104.5 to 153.6)	51.1 (40.6 to 64.4)	50.6 (37.7 to 66.9)	24.5 (12.5 to 42.9)	0.9 (0.8 to 0.9)	424.9 (349.4 to 513.3)	170.9 (135.7 to 215.3)	169.2 (126.0 to 223.7)	81.9 (41.9 to 143.3)	3.0 (2.8 to 3.1)
Indonesia	2020	130.9 (107.8 to 158.8)	52.6 (41.7 to 66.4)	52.1 (38.6 to 69.2)	25.3 (12.9 to 44.2)	0.9 (0.8 to 1.0)	437.5 (360.4 to 530.9)	175.9 (139.5 to 221.9)	174.2 (129.1 to 231.3)	84.4 (43.2 to 147.8)	3.0 (2.8 to 3.3)
Indonesia	2021	134.7 (111.0 to 163.0)	54.1 (42.9 to 68.2)	53.6 (39.6 to 71.3)	26.0 (13.3 to 45.7)	0.9 (0.8 to 1.0)	450.2 (370.9 to 544.8)	180.9 (143.3 to 227.9)	179.2 (132.5 to 238.5)	87.0 (44.5 to 152.7)	3.1 (2.8 to 3.4)
Indonesia	2022	138.5 (114.7 to 168.2)	55.6 (44.0 to 70.0)	55.1 (40.9 to 73.6)	26.8 (13.7 to 47.0)	0.9 (0.8 to 1.0)	462.9 (383.3 to 562.2)	185.9 (147.2 to 234.0)	184.3 (136.6 to 245.9)	89.6 (45.8 to 157.1)	3.1 (2.8 to 3.5)

		2018 US Dollars per capita					2018 PPP per capita				
Country	Year	Health spending	Government health spending	Out-of-pocket health spending	Prepaid private health spending	Development assistance for health	Health spending	Government health spending	Out-of-pocket health spending	Prepaid private health spending	Development assistance for health
Indonesia	2023	142.2 (116.7 to 171.6)	57.1 (45.1 to 71.9)	56.5 (41.8 to 75.3)	27.6 (14.1 to 48.4)	1.0 (0.8 to 1.1)	475.3 (389.9 to 573.5)	191.0 (150.9 to 240.3)	189.0 (139.6 to 251.7)	92.2 (47.1 to 161.9)	3.2 (2.8 to 3.6)
Indonesia	2024	146.1 (119.9 to 176.9)	58.9 (46.5 to 74.1)	57.8 (42.7 to 76.9)	28.4 (14.5 to 49.8)	1.0 (0.8 to 1.1)	488.4 (400.8 to 591.5)	197.0 (155.4 to 247.8)	193.1 (142.7 to 257.0)	95.0 (48.4 to 166.3)	3.3 (2.8 to 3.8)
Indonesia	2025	150.4 (124.1 to 181.8)	61.1 (48.1 to 76.8)	59.1 (43.6 to 78.5)	29.3 (14.9 to 51.3)	1.0 (0.9 to 1.2)	502.9 (414.7 to 607.6)	204.2 (160.9 to 256.6)	197.4 (145.8 to 262.5)	98.0 (49.8 to 171.4)	3.3 (2.9 to 3.9)
Indonesia	2026	154.7 (128.1 to 187.6)	63.2 (49.8 to 79.4)	60.3 (44.5 to 80.2)	30.2 (15.3 to 52.6)	1.0 (0.9 to 1.2)	517.3 (428.2 to 627.0)	211.2 (166.4 to 265.3)	201.7 (148.9 to 268.1)	101.0 (51.3 to 175.9)	3.4 (2.9 to 4.0)
Indonesia	2027	158.7 (130.8 to 192.2)	65.0 (51.1 to 81.6)	61.6 (45.5 to 81.9)	31.1 (15.7 to 54.0)	1.0 (0.9 to 1.2)	530.5 (437.3 to 642.7)	217.1 (170.7 to 272.6)	206.1 (152.0 to 273.8)	103.8 (52.6 to 180.6)	3.5 (2.9 to 4.1)
Indonesia	2028	162.8 (134.8 to 197.6)	66.8 (52.4 to 83.8)	63.0 (46.5 to 83.7)	32.0 (16.2 to 55.6)	1.1 (0.9 to 1.3)	544.3 (450.5 to 660.5)	223.3 (175.3 to 280.2)	210.6 (155.4 to 279.8)	106.9 (54.1 to 185.8)	3.6 (3.0 to 4.3)
Indonesia	2029	167.2 (137.3 to 202.3)	68.8 (54.0 to 86.3)	64.4 (47.6 to 85.5)	32.9 (16.7 to 57.3)	1.1 (0.9 to 1.3)	559.0 (459.1 to 676.4)	230.0 (180.4 to 288.5)	215.2 (159.0 to 285.9)	110.1 (55.8 to 191.5)	3.7 (3.0 to 4.5)
Indonesia	2030	172.0 (142.2 to 209.4)	71.2 (55.7 to 89.2)	65.8 (48.7 to 87.4)	34.0 (17.2 to 59.0)	1.1 (0.9 to 1.4)	575.1 (475.4 to 700.0)	237.9 (186.3 to 298.2)	220.0 (162.6 to 292.3)	113.5 (57.5 to 197.3)	3.7 (3.1 to 4.6)
Indonesia	2031	176.8 (145.7 to 214.0)	73.4 (57.1 to 92.0)	67.3 (49.7 to 89.4)	35.0 (17.7 to 60.7)	1.1 (0.9 to 1.4)	590.9 (487.1 to 715.3)	245.3 (191.0 to 307.7)	224.9 (166.2 to 298.8)	116.9 (59.2 to 203.1)	3.8 (3.1 to 4.8)
Indonesia	2032	181.7 (149.8 to 219.8)	75.7 (58.6 to 94.9)	68.8 (50.8 to 91.5)	36.0 (18.2 to 62.6)	1.2 (0.9 to 1.5)	607.3 (500.9 to 734.7)	253.1 (195.9 to 317.4)	229.9 (169.9 to 305.9)	120.4 (61.0 to 209.3)	3.9 (3.1 to 4.9)
Indonesia	2033	186.8 (154.4 to 225.5)	78.1 (60.5 to 98.1)	70.3 (52.0 to 93.7)	37.1 (18.8 to 64.6)	1.2 (0.9 to 1.5)	624.3 (516.1 to 753.9)	261.2 (202.1 to 327.8)	235.0 (173.8 to 313.1)	124.1 (62.8 to 215.8)	4.0 (3.2 to 5.1)
Indonesia	2034	191.9 (158.1 to 232.6)	80.6 (62.2 to 101.1)	71.9 (53.1 to 95.9)	38.3 (19.4 to 66.5)	1.2 (1.0 to 1.6)	641.6 (528.4 to 777.5)	269.3 (208.0 to 338.0)	240.3 (177.6 to 320.5)	127.9 (64.7 to 222.4)	4.1 (3.2 to 5.3)
Indonesia	2035	197.2 (162.2 to 238.5)	83.1 (64.0 to 104.2)	73.5 (54.3 to 98.1)	39.4 (19.9 to 68.5)	1.3 (1.0 to 1.6)	659.3 (542.3 to 797.3)	277.7 (213.8 to 348.3)	245.7 (181.4 to 328.0)	131.7 (66.7 to 229.1)	4.2 (3.3 to 5.5)
Indonesia	2036	202.5 (167.1 to 246.3)	85.6 (65.8 to 107.4)	75.1 (55.4 to 100.4)	40.6 (20.5 to 70.5)	1.3 (1.0 to 1.7)	677.0 (558.4 to 823.5)	286.0 (219.8 to 359.0)	251.0 (185.2 to 335.5)	135.6 (68.5 to 235.7)	4.3 (3.3 to 5.8)
Indonesia	2037	207.9 (171.3 to 251.7)	88.1 (67.6 to 110.8)	76.7 (56.6 to 102.7)	41.8 (21.1 to 72.7)	1.3 (1.0 to 1.8)	695.0 (572.8 to 841.3)	294.5 (225.9 to 370.4)	256.5 (189.2 to 343.2)	139.6 (70.4 to 243.0)	4.4 (3.4 to 5.9)
Indonesia	2038	213.4 (175.7 to 259.3)	90.7 (69.5 to 113.9)	78.4 (57.9 to 104.9)	43.0 (21.6 to 74.9)	1.3 (1.0 to 1.9)	713.4 (587.5 to 866.9)	303.1 (232.5 to 380.7)	262.0 (193.6 to 350.5)	143.7 (72.3 to 250.3)	4.5 (3.4 to 6.3)
Indonesia	2039	219.0 (180.7 to 266.4)	93.3 (71.5 to 117.1)	80.1 (59.2 to 107.1)	44.2 (22.2 to 77.1)	1.4 (1.1 to 1.9)	732.1 (604.2 to 890.5)	311.9 (239.1 to 391.5)	267.7 (197.9 to 357.9)	147.9 (74.2 to 257.8)	4.6 (3.5 to 6.4)
Indonesia	2040	224.8 (185.0 to 273.2)	96.0 (73.4 to 120.7)	81.8 (60.5 to 109.3)	45.5 (22.8 to 79.4)	1.4 (1.1 to 2.0)	751.5 (618.4 to 913.3)	321.0 (245.2 to 403.5)	273.5 (202.2 to 365.5)	152.2 (76.3 to 265.4)	4.7 (3.6 to 6.6)
Indonesia	2041	230.7 (189.3 to 279.9)	98.8 (75.2 to 124.4)	83.6 (61.8 to 111.9)	46.9 (23.4 to 81.7)	1.4 (1.1 to 2.0)	771.3 (632.7 to 935.8)	330.3 (251.5 to 416.0)	279.4 (206.5 to 374.0)	156.7 (78.3 to 273.2)	4.8 (3.6 to 6.7)
Indonesia	2042	236.7 (193.6 to 289.3)	101.6 (77.3 to 128.2)	85.4 (63.1 to 114.5)	48.2 (24.1 to 84.1)	1.5 (1.1 to 2.1)	791.3 (647.0 to 967.2)	339.7 (258.3 to 428.6)	285.4 (210.8 to 382.6)	161.2 (80.4 to 281.3)	5.0 (3.7 to 7.1)
Indonesia	2043	242.9 (198.9 to 294.6)	104.5 (79.4 to 132.3)	87.2 (64.4 to 117.0)	49.6 (24.7 to 86.6)	1.5 (1.1 to 2.2)	812.0 (665.0 to 984.6)	349.3 (265.6 to 442.2)	291.6 (215.1 to 391.0)	165.9 (82.6 to 289.6)	5.1 (3.8 to 7.3)

Country	Year	2018 US Dollars per capita					2018 PPP per capita				
		Health spending	Government health spending	Out-of-pocket health spending	Prepaid private health spending	Development assistance for health	Health spending	Government health spending	Out-of-pocket health spending	Prepaid private health spending	Development assistance for health
Indonesia	2044	249.2 (203.0 to 305.4)	107.4 (81.7 to 136.3)	89.1 (65.7 to 119.5)	51.1 (25.5 to 89.2)	1.6 (1.1 to 2.3)	833.0 (678.7 to 1021.0)	359.2 (273.2 to 455.6)	297.9 (219.6 to 399.4)	170.7 (85.1 to 298.1)	5.2 (3.8 to 7.6)
Indonesia	2045	255.8 (209.7 to 311.2)	110.6 (84.2 to 140.7)	91.0 (67.1 to 121.9)	52.6 (26.3 to 91.8)	1.6 (1.2 to 2.4)	855.0 (701.0 to 1040.4)	369.6 (281.5 to 470.5)	304.3 (224.4 to 407.7)	175.7 (88.0 to 306.9)	5.4 (3.9 to 8.1)
Indonesia	2046	262.5 (214.1 to 323.2)	113.9 (86.5 to 144.9)	92.9 (68.5 to 124.3)	54.1 (27.2 to 94.5)	1.7 (1.2 to 2.5)	877.5 (715.8 to 1080.5)	380.7 (289.1 to 484.3)	310.5 (229.0 to 415.6)	180.7 (90.9 to 315.8)	5.5 (4.0 to 8.5)
Indonesia	2047	269.4 (220.5 to 328.3)	117.3 (89.0 to 149.6)	94.8 (69.9 to 126.7)	55.6 (28.0 to 97.2)	1.7 (1.2 to 2.7)	900.6 (737.1 to 1097.5)	392.2 (297.5 to 500.1)	316.8 (233.7 to 423.6)	185.9 (93.6 to 324.9)	5.7 (4.1 to 8.9)
Indonesia	2048	276.6 (225.1 to 340.7)	121.0 (91.6 to 154.7)	96.7 (71.3 to 129.1)	57.2 (28.8 to 100.0)	1.8 (1.2 to 2.8)	924.7 (752.4 to 1138.8)	404.5 (306.2 to 517.1)	323.1 (238.4 to 431.6)	191.2 (96.2 to 334.2)	5.9 (4.2 to 9.4)
Indonesia	2049	284.1 (232.8 to 347.0)	124.9 (94.3 to 159.8)	98.6 (72.7 to 131.8)	58.8 (29.5 to 102.9)	1.8 (1.3 to 2.9)	949.7 (778.2 to 1160.1)	417.5 (315.2 to 534.0)	329.6 (243.1 to 440.5)	196.7 (98.6 to 343.9)	6.0 (4.3 to 9.8)
Indonesia	2050	292.1 (239.2 to 360.0)	129.1 (97.3 to 165.4)	100.6 (74.2 to 134.6)	60.6 (30.3 to 106.0)	1.8 (1.3 to 3.0)	976.6 (799.7 to 1203.4)	431.6 (325.1 to 552.8)	336.3 (248.1 to 449.9)	202.5 (101.2 to 354.2)	6.2 (4.3 to 10.1)
Iran	1995	116.4 (100.7 to 134.8)	46.2 (38.0 to 56.4)	66.6 (53.5 to 81.4)	3.5 (1.8 to 6.3)	0.1 (0.1 to 0.1)	472.9 (409.3 to 547.6)	187.7 (154.3 to 229.3)	270.6 (217.4 to 330.6)	14.2 (7.4 to 25.4)	0.3 (0.3 to 0.3)
Iran	1996	123.6 (108.0 to 141.2)	50.2 (42.2 to 60.2)	69.0 (56.5 to 83.1)	3.7 (2.0 to 6.6)	0.7 (0.7 to 0.7)	502.2 (438.8 to 573.5)	203.9 (171.3 to 244.4)	280.4 (229.4 to 337.4)	15.2 (8.0 to 26.7)	2.7 (2.7 to 2.7)
Iran	1997	130.1 (114.6 to 147.2)	52.8 (44.5 to 62.7)	72.4 (59.8 to 86.2)	4.3 (2.2 to 7.5)	0.6 (0.6 to 0.6)	528.6 (465.8 to 597.9)	214.5 (181.0 to 254.6)	294.3 (243.1 to 350.0)	17.3 (9.1 to 30.4)	2.6 (2.6 to 2.6)
Iran	1998	137.5 (122.3 to 153.7)	55.0 (46.7 to 64.7)	77.5 (65.2 to 91.5)	4.7 (2.5 to 8.1)	0.4 (0.4 to 0.4)	558.6 (496.7 to 624.6)	223.4 (189.6 to 262.7)	314.8 (265.0 to 371.7)	18.9 (10.0 to 32.9)	1.5 (1.5 to 1.5)
Iran	1999	143.2 (127.6 to 159.4)	55.1 (47.0 to 64.4)	82.8 (70.2 to 96.6)	5.2 (2.7 to 8.9)	0.2 (0.2 to 0.2)	581.9 (518.6 to 647.7)	223.9 (190.8 to 261.5)	336.4 (285.1 to 392.5)	20.9 (11.1 to 36.2)	0.8 (0.8 to 0.8)
Iran	2000	154.7 (137.9 to 171.5)	59.1 (50.6 to 68.9)	90.1 (76.2 to 104.1)	5.5 (2.9 to 9.4)	0.1 (0.1 to 0.1)	628.7 (560.2 to 696.6)	240.1 (205.4 to 279.8)	365.9 (309.6 to 423.1)	22.2 (11.7 to 38.1)	0.5 (0.5 to 0.5)
Iran	2001	163.5 (147.1 to 181.5)	62.2 (53.5 to 72.7)	94.8 (80.4 to 109.3)	6.3 (3.3 to 10.9)	0.2 (0.2 to 0.2)	664.4 (597.6 to 737.2)	252.8 (217.3 to 295.2)	385.2 (326.8 to 444.0)	25.6 (13.4 to 44.3)	0.9 (0.9 to 0.9)
Iran	2002	175.1 (157.7 to 194.4)	67.4 (57.9 to 78.7)	98.9 (84.8 to 113.7)	8.8 (4.6 to 15.0)	0.1 (0.1 to 0.1)	711.5 (640.5 to 789.7)	273.7 (235.4 to 319.6)	401.8 (344.7 to 461.9)	35.8 (18.7 to 61.0)	0.2 (0.2 to 0.2)
Iran	2003	192.1 (173.4 to 212.0)	73.8 (63.5 to 85.9)	106.9 (92.0 to 123.4)	11.4 (6.1 to 19.1)	0.0 (0.0 to 0.0)	780.4 (704.5 to 861.2)	299.7 (258.0 to 349.0)	434.4 (373.9 to 501.4)	46.3 (24.9 to 77.4)	0.0 (0.0 to 0.0)
Iran	2004	205.1 (185.0 to 226.3)	77.6 (66.5 to 90.3)	114.2 (98.4 to 131.0)	13.2 (7.2 to 21.0)	0.1 (0.1 to 0.1)	833.2 (751.4 to 919.4)	315.3 (270.3 to 366.8)	463.9 (399.9 to 532.3)	53.7 (29.4 to 85.5)	0.4 (0.4 to 0.4)
Iran	2005	222.7 (200.9 to 244.9)	84.7 (73.2 to 98.0)	122.0 (105.2 to 140.2)	15.0 (8.6 to 23.9)	1.0 (1.0 to 1.0)	904.6 (816.3 to 994.9)	344.0 (297.2 to 398.0)	495.5 (427.2 to 569.7)	61.0 (34.9 to 97.0)	4.1 (4.1 to 4.1)
Iran	2006	233.7 (210.1 to 256.7)	90.7 (79.1 to 104.3)	126.5 (109.8 to 145.2)	16.4 (9.7 to 24.9)	0.2 (0.2 to 0.2)	949.5 (853.7 to 1042.9)	368.3 (321.4 to 423.8)	513.9 (446.0 to 590.0)	66.5 (39.4 to 101.3)	0.8 (0.8 to 0.8)
Iran	2007	252.1 (227.6 to 277.3)	97.1 (84.6 to 110.3)	136.6 (118.1 to 156.4)	18.3 (11.1 to 28.0)	0.1 (0.1 to 0.1)	1024.4 (924.8 to 1126.6)	394.6 (343.6 to 448.3)	555.0 (479.9 to 635.2)	74.3 (45.2 to 113.6)	0.5 (0.5 to 0.5)
Iran	2008	265.7 (239.9 to 291.4)	98.4 (85.3 to 112.0)	147.1 (128.1 to 168.2)	20.0 (12.4 to 31.0)	0.2 (0.2 to 0.2)	1079.6 (974.7 to 1183.8)	399.8 (346.3 to 455.2)	597.5 (520.5 to 683.5)	81.4 (50.6 to 125.9)	0.9 (0.9 to 0.9)

Country	Year	2018 US Dollars per capita					2018 PPP per capita				
		Health spending	Government health spending	Out-of-pocket health spending	Prepaid private health spending	Development assistance for health	Health spending	Government health spending	Out-of-pocket health spending	Prepaid private health spending	Development assistance for health
Iran	2009	292.1 (265.1 to 322.1)	104.0 (90.0 to 118.0)	164.5 (144.5 to 186.7)	23.3 (14.5 to 35.9)	0.2 (0.2 to 0.2)	1186.5 (1077.0 to 1308.7)	422.4 (365.7 to 479.4)	668.4 (586.9 to 758.3)	94.7 (59.1 to 145.7)	1.0 (1.0 to 1.0)
Iran	2010	317.3 (288.3 to 348.9)	110.5 (96.2 to 125.3)	179.3 (159.4 to 203.4)	27.4 (17.1 to 41.7)	0.1 (0.1 to 0.1)	1289.2 (1171.3 to 1417.4)	448.8 (390.9 to 509.2)	728.5 (647.4 to 826.1)	111.3 (69.6 to 169.5)	0.6 (0.6 to 0.6)
Iran	2011	326.9 (298.0 to 360.0)	116.1 (102.2 to 131.1)	179.5 (158.3 to 204.4)	31.1 (19.5 to 46.8)	0.2 (0.2 to 0.2)	1328.0 (1210.6 to 1462.6)	471.8 (415.1 to 532.6)	729.4 (643.1 to 830.5)	126.2 (79.3 to 189.9)	0.7 (0.7 to 0.7)
Iran	2012	306.4 (279.2 to 335.7)	112.0 (98.6 to 126.6)	161.6 (141.0 to 184.7)	32.5 (20.7 to 48.2)	0.2 (0.2 to 0.2)	1244.7 (1134.3 to 1363.8)	455.0 (400.5 to 514.5)	656.7 (572.9 to 750.4)	132.0 (83.9 to 195.9)	1.0 (1.0 to 1.0)
Iran	2013	311.3 (283.0 to 340.8)	125.2 (109.5 to 141.3)	149.6 (130.3 to 172.0)	36.5 (23.3 to 53.8)	0.1 (0.1 to 0.1)	1264.9 (1149.7 to 1384.6)	508.5 (444.7 to 574.1)	607.7 (529.4 to 698.7)	148.1 (94.6 to 218.4)	0.5 (0.5 to 0.5)
Iran	2014	343.2 (311.0 to 377.2)	153.3 (134.4 to 173.2)	148.5 (128.2 to 171.6)	41.3 (26.3 to 61.7)	0.2 (0.2 to 0.2)	1394.4 (1263.4 to 1532.3)	622.8 (546.1 to 703.4)	603.4 (520.9 to 697.1)	167.6 (106.8 to 250.5)	0.6 (0.6 to 0.6)
Iran	2015	373.0 (336.5 to 414.5)	177.7 (154.2 to 202.3)	150.7 (128.5 to 176.0)	44.4 (27.6 to 67.6)	0.1 (0.1 to 0.1)	1515.2 (1367.2 to 1683.8)	721.9 (626.7 to 821.7)	612.3 (522.0 to 715.1)	180.5 (112.3 to 274.8)	0.5 (0.5 to 0.5)
Iran	2016	420.2 (375.1 to 471.4)	212.3 (181.0 to 243.7)	157.7 (131.3 to 188.0)	50.1 (29.7 to 79.3)	0.1 (0.1 to 0.1)	1707.1 (1524.0 to 1915.1)	862.4 (735.4 to 990.2)	640.7 (533.4 to 763.8)	203.6 (120.7 to 322.3)	0.4 (0.4 to 0.4)
Iran	2017	417.7 (372.4 to 467.7)	207.0 (176.5 to 238.2)	159.8 (133.2 to 190.4)	50.8 (30.1 to 80.3)	0.1 (0.1 to 0.1)	1696.9 (1513.0 to 1900.1)	841.2 (717.0 to 967.8)	649.0 (541.3 to 773.5)	206.4 (122.2 to 326.4)	0.3 (0.3 to 0.3)
Iran	2018	425.4 (377.8 to 476.4)	211.6 (180.4 to 243.2)	161.7 (135.1 to 193.5)	52.0 (30.8 to 82.4)	0.1 (0.1 to 0.1)	1728.1 (1534.9 to 1935.6)	859.7 (732.9 to 988.1)	656.9 (548.9 to 786.1)	211.2 (125.1 to 334.8)	0.3 (0.3 to 0.3)
Iran	2019	430.3 (382.3 to 481.1)	214.2 (182.6 to 246.4)	163.3 (135.9 to 195.4)	52.7 (31.3 to 83.5)	0.1 (0.1 to 0.1)	1748.0 (1553.0 to 1954.4)	870.2 (742.0 to 1001.2)	663.3 (551.9 to 793.8)	214.2 (127.0 to 339.2)	0.3 (0.3 to 0.3)
Iran	2020	435.1 (385.7 to 486.0)	216.7 (185.0 to 249.6)	164.8 (137.2 to 197.2)	53.5 (31.8 to 84.4)	0.1 (0.1 to 0.1)	1767.5 (1567.1 to 1974.4)	880.4 (751.7 to 1013.9)	669.6 (557.4 to 801.2)	217.2 (129.1 to 343.0)	0.3 (0.3 to 0.3)
Iran	2021	440.5 (391.2 to 494.0)	219.6 (187.6 to 252.9)	166.5 (138.2 to 199.1)	54.3 (32.2 to 85.5)	0.1 (0.1 to 0.1)	1789.4 (1589.2 to 2006.8)	892.0 (762.1 to 1027.5)	676.5 (561.6 to 809.0)	220.6 (130.7 to 347.2)	0.3 (0.3 to 0.3)
Iran	2022	445.4 (395.2 to 497.7)	222.1 (189.5 to 256.0)	168.1 (139.7 to 201.4)	55.1 (32.7 to 86.4)	0.1 (0.1 to 0.1)	1809.4 (1605.6 to 2022.1)	902.5 (769.8 to 1040.1)	682.8 (567.6 to 818.0)	223.8 (132.7 to 351.1)	0.3 (0.3 to 0.4)
Iran	2023	450.3 (399.6 to 502.0)	224.6 (191.3 to 258.9)	169.7 (141.2 to 202.8)	55.9 (33.1 to 87.5)	0.1 (0.1 to 0.1)	1829.3 (1623.3 to 2039.4)	912.6 (777.2 to 1052.0)	689.5 (573.7 to 824.0)	226.9 (134.5 to 355.5)	0.3 (0.3 to 0.4)
Iran	2024	456.3 (405.2 to 508.8)	227.3 (193.8 to 262.3)	172.2 (143.2 to 205.3)	56.6 (33.6 to 89.1)	0.1 (0.1 to 0.1)	1853.6 (1646.1 to 2067.0)	923.5 (787.2 to 1065.6)	699.6 (581.7 to 834.1)	230.1 (136.4 to 362.2)	0.3 (0.3 to 0.4)
Iran	2025	463.9 (412.0 to 518.6)	231.3 (197.3 to 267.1)	174.9 (145.5 to 208.0)	57.6 (34.1 to 91.0)	0.1 (0.1 to 0.1)	1884.7 (1673.6 to 2106.8)	939.8 (801.6 to 1085.1)	710.5 (591.0 to 844.9)	234.0 (138.5 to 369.6)	0.3 (0.3 to 0.4)
Iran	2026	470.5 (415.6 to 524.5)	234.5 (200.2 to 271.6)	177.5 (147.2 to 211.8)	58.4 (34.5 to 92.7)	0.1 (0.1 to 0.1)	1911.6 (1688.3 to 2130.8)	952.7 (813.2 to 1103.5)	721.2 (597.8 to 860.3)	237.3 (140.3 to 376.5)	0.3 (0.3 to 0.4)
Iran	2027	476.2 (419.4 to 532.1)	237.0 (202.4 to 274.2)	180.0 (148.6 to 215.3)	59.1 (34.9 to 93.9)	0.1 (0.1 to 0.1)	1934.5 (1703.8 to 2161.9)	962.9 (822.3 to 1114.0)	731.1 (603.6 to 874.6)	240.1 (141.9 to 381.5)	0.4 (0.3 to 0.4)
Iran	2028	482.1 (424.6 to 540.3)	239.7 (204.6 to 277.8)	182.5 (149.6 to 219.3)	59.8 (35.4 to 94.9)	0.1 (0.1 to 0.1)	1958.5 (1725.0 to 2195.1)	973.7 (831.3 to 1128.7)	741.3 (607.7 to 891.0)	243.0 (143.7 to 385.5)	0.4 (0.3 to 0.4)
Iran	2029	488.1 (430.7 to 545.4)	242.4 (206.5 to 280.6)	185.1 (150.8 to 225.0)	60.6 (35.8 to 95.9)	0.1 (0.1 to 0.1)	1983.1 (1749.9 to 2215.8)	984.8 (839.0 to 1139.8)	751.8 (612.5 to 913.9)	246.0 (145.5 to 389.8)	0.4 (0.3 to 0.5)

		2018 US Dollars per capita					2018 PPP per capita				
Country	Year	Health spending	Government health spending	Out-of-pocket health spending	Prepaid private health spending	Development assistance for health	Health spending	Government health spending	Out-of-pocket health spending	Prepaid private health spending	Development assistance for health
Iran	2030	494.8 (436.0 to 554.7)	245.7 (208.8 to 284.5)	187.7 (152.4 to 229.0)	61.4 (36.3 to 97.4)	0.1 (0.1 to 0.1)	2010.1 (1771.1 to 2253.6)	998.0 (848.4 to 1155.8)	762.4 (619.0 to 930.5)	249.3 (147.6 to 395.7)	0.4 (0.3 to 0.5)
Iran	2031	502.4 (440.6 to 565.4)	249.6 (212.1 to 289.1)	190.5 (153.3 to 234.5)	62.3 (36.9 to 99.1)	0.1 (0.1 to 0.1)	2041.1 (1789.9 to 2297.2)	1013.9 (861.6 to 1174.5)	773.8 (622.6 to 952.8)	253.1 (149.7 to 402.5)	0.4 (0.3 to 0.5)
Iran	2032	510.5 (448.4 to 573.8)	253.8 (215.5 to 293.9)	193.3 (155.0 to 238.7)	63.3 (37.4 to 100.8)	0.1 (0.1 to 0.1)	2073.9 (1821.9 to 2331.0)	1031.0 (875.5 to 1193.8)	785.5 (629.8 to 969.8)	257.0 (152.0 to 409.7)	0.4 (0.3 to 0.5)
Iran	2033	518.9 (453.0 to 584.7)	258.1 (219.7 to 299.5)	196.3 (155.9 to 243.6)	64.3 (38.0 to 102.7)	0.1 (0.1 to 0.1)	2107.9 (1840.3 to 2375.5)	1048.8 (892.5 to 1216.6)	797.5 (633.3 to 989.7)	261.2 (154.4 to 417.2)	0.4 (0.3 to 0.5)
Iran	2034	526.7 (460.7 to 595.5)	262.2 (223.2 to 304.4)	199.2 (157.1 to 248.1)	65.2 (38.6 to 104.3)	0.1 (0.1 to 0.1)	2139.6 (1871.8 to 2419.3)	1065.0 (906.9 to 1236.5)	809.1 (638.4 to 1007.8)	265.1 (156.8 to 423.5)	0.4 (0.3 to 0.6)
Iran	2035	534.6 (465.5 to 604.2)	266.2 (226.1 to 309.2)	202.1 (159.2 to 253.5)	66.2 (39.2 to 105.7)	0.1 (0.1 to 0.1)	2171.8 (1891.2 to 2454.7)	1081.4 (918.7 to 1256.3)	820.9 (646.8 to 1030.0)	269.0 (159.1 to 429.3)	0.4 (0.3 to 0.6)
Iran	2036	542.7 (472.9 to 616.1)	270.3 (229.1 to 314.6)	205.0 (160.9 to 259.3)	67.2 (39.8 to 107.6)	0.1 (0.1 to 0.2)	2204.7 (1921.4 to 2503.1)	1098.2 (930.6 to 1278.0)	832.9 (653.6 to 1053.5)	273.2 (161.6 to 437.0)	0.5 (0.4 to 0.6)
Iran	2037	550.4 (476.4 to 626.2)	274.2 (232.2 to 319.4)	207.9 (162.8 to 264.4)	68.2 (40.4 to 109.2)	0.1 (0.1 to 0.2)	2236.2 (1935.5 to 2543.9)	1113.9 (943.2 to 1297.8)	844.7 (661.6 to 1074.1)	277.1 (164.0 to 443.7)	0.5 (0.4 to 0.6)
Iran	2038	557.9 (483.5 to 636.7)	277.9 (235.1 to 323.7)	210.8 (164.6 to 269.3)	69.1 (40.9 to 110.7)	0.1 (0.1 to 0.2)	2266.5 (1964.2 to 2586.5)	1128.8 (955.3 to 1315.1)	856.3 (668.8 to 1094.1)	280.8 (166.1 to 449.9)	0.5 (0.4 to 0.7)
Iran	2039	565.0 (489.4 to 645.2)	281.3 (237.5 to 328.3)	213.6 (166.2 to 274.3)	70.0 (41.4 to 112.3)	0.1 (0.1 to 0.2)	2295.3 (1988.3 to 2621.1)	1142.6 (965.0 to 1333.6)	867.8 (675.1 to 1114.4)	284.4 (168.0 to 456.4)	0.5 (0.4 to 0.7)
Iran	2040	571.6 (495.4 to 653.9)	284.4 (239.5 to 332.5)	216.3 (167.7 to 279.0)	70.8 (41.8 to 113.8)	0.1 (0.1 to 0.2)	2322.3 (2012.6 to 2656.6)	1155.4 (973.1 to 1350.9)	878.7 (681.1 to 1133.4)	287.7 (169.9 to 462.2)	0.5 (0.4 to 0.7)
Iran	2041	577.5 (498.3 to 660.9)	287.0 (241.1 to 336.4)	218.9 (169.0 to 283.7)	71.5 (42.2 to 114.9)	0.1 (0.1 to 0.2)	2346.2 (2024.3 to 2684.9)	1165.9 (979.5 to 1366.6)	889.3 (686.5 to 1152.7)	290.5 (171.6 to 466.7)	0.5 (0.4 to 0.7)
Iran	2042	582.9 (504.0 to 668.5)	289.3 (242.4 to 339.7)	221.3 (170.3 to 287.6)	72.1 (42.6 to 115.9)	0.1 (0.1 to 0.2)	2367.9 (2047.5 to 2716.1)	1175.1 (984.9 to 1380.2)	899.2 (692.0 to 1168.4)	293.0 (173.0 to 470.9)	0.5 (0.4 to 0.8)
Iran	2043	587.4 (507.3 to 675.5)	290.9 (243.3 to 342.3)	223.7 (171.7 to 291.4)	72.6 (42.9 to 116.6)	0.1 (0.1 to 0.2)	2386.2 (2061.0 to 2744.2)	1181.8 (988.2 to 1390.8)	908.8 (697.7 to 1183.9)	295.0 (174.2 to 473.7)	0.6 (0.4 to 0.8)
Iran	2044	591.5 (508.6 to 677.9)	292.3 (244.1 to 344.2)	225.9 (173.2 to 294.4)	73.0 (43.1 to 117.2)	0.1 (0.1 to 0.2)	2402.8 (2066.3 to 2753.9)	1187.7 (991.6 to 1398.3)	917.9 (703.5 to 1196.0)	296.7 (175.2 to 476.3)	0.6 (0.4 to 0.8)
Iran	2045	594.7 (511.1 to 685.4)	293.3 (244.0 to 345.9)	227.9 (174.6 to 298.1)	73.3 (43.3 to 117.6)	0.1 (0.1 to 0.2)	2415.8 (2076.6 to 2784.5)	1191.5 (991.3 to 1405.4)	925.9 (709.5 to 1211.2)	297.8 (175.7 to 477.9)	0.6 (0.4 to 0.9)
Iran	2046	597.7 (513.2 to 688.0)	294.3 (244.0 to 347.9)	229.8 (175.9 to 300.9)	73.5 (43.3 to 117.9)	0.2 (0.1 to 0.2)	2428.1 (2085.0 to 2794.9)	1195.5 (991.3 to 1413.5)	933.4 (714.5 to 1222.3)	298.6 (175.9 to 479.0)	0.6 (0.5 to 1.0)
Iran	2047	599.3 (513.1 to 690.5)	294.5 (244.0 to 348.8)	231.2 (176.9 to 302.8)	73.5 (43.2 to 117.9)	0.2 (0.1 to 0.3)	2434.8 (2084.4 to 2805.2)	1196.3 (991.2 to 1416.9)	939.4 (718.6 to 1230.1)	298.4 (175.6 to 478.9)	0.6 (0.5 to 1.0)
Iran	2048	601.6 (513.6 to 695.2)	295.1 (244.3 to 350.3)	232.8 (178.0 to 305.9)	73.5 (43.2 to 117.9)	0.2 (0.1 to 0.3)	2444.0 (2086.6 to 2824.2)	1198.9 (992.5 to 1423.1)	945.9 (722.9 to 1242.6)	298.6 (175.5 to 478.9)	0.7 (0.5 to 1.1)
Iran	2049	603.2 (515.7 to 697.5)	295.4 (244.0 to 351.1)	234.2 (178.9 to 307.4)	73.4 (43.2 to 117.7)	0.2 (0.1 to 0.3)	2450.7 (2095.2 to 2833.8)	1200.2 (991.3 to 1426.2)	951.5 (726.8 to 1248.7)	298.3 (175.4 to 478.3)	0.7 (0.5 to 1.1)
Iran	2050	605.2 (516.1 to 700.6)	296.1 (243.9 to 353.0)	235.6 (179.8 to 311.3)	73.4 (43.2 to 117.6)	0.2 (0.1 to 0.3)	2458.7 (2096.9 to 2846.4)	1202.8 (990.9 to 1434.2)	957.1 (730.4 to 1264.9)	298.0 (175.5 to 477.9)	0.7 (0.5 to 1.2)

		2018 US Dollars per capita					2018 PPP per capita				
Country	Year	Health spending	Government health spending	Out-of-pocket health spending	Prepaid private health spending	Development assistance for health	Health spending	Government health spending	Out-of-pocket health spending	Prepaid private health spending	Development assistance for health
Iraq	1995	40.3 (32.3 to 50.0)	17.4 (13.0 to 22.5)	22.5 (15.8 to 31.1)	0.0 (0.0 to 0.0)	0.3 (0.3 to 0.3)	129.7 (103.9 to 160.7)	56.1 (41.8 to 72.3)	72.5 (50.8 to 100.1)	0.0 (0.0 to 0.1)	1.1 (1.1 to 1.1)
Iraq	1996	45.1 (36.0 to 55.4)	20.7 (15.6 to 27.0)	24.1 (17.0 to 33.5)	0.0 (0.0 to 0.0)	0.2 (0.2 to 0.2)	145.0 (115.9 to 178.4)	66.7 (50.3 to 87.0)	77.7 (54.6 to 107.8)	0.0 (0.0 to 0.1)	0.6 (0.6 to 0.6)
Iraq	1997	51.5 (41.8 to 62.9)	25.1 (18.9 to 32.9)	26.3 (18.7 to 36.3)	0.0 (0.0 to 0.0)	0.1 (0.1 to 0.1)	165.6 (134.5 to 202.2)	80.7 (60.9 to 105.7)	84.6 (60.2 to 116.7)	0.0 (0.0 to 0.1)	0.3 (0.3 to 0.3)
Iraq	1998	64.2 (52.3 to 78.4)	33.7 (25.9 to 43.6)	30.5 (22.0 to 41.1)	0.0 (0.0 to 0.0)	0.0 (0.0 to 0.0)	206.5 (168.3 to 252.2)	108.3 (83.4 to 140.2)	98.1 (70.9 to 132.4)	0.1 (0.0 to 0.1)	0.1 (0.1 to 0.1)
Iraq	1999	74.4 (61.5 to 91.2)	41.1 (31.9 to 52.2)	33.2 (24.3 to 44.8)	0.0 (0.0 to 0.0)	0.1 (0.1 to 0.1)	239.5 (197.8 to 293.4)	132.2 (102.7 to 168.0)	106.8 (78.2 to 144.2)	0.1 (0.0 to 0.1)	0.4 (0.4 to 0.4)
Iraq	2000	80.6 (66.4 to 97.7)	45.9 (35.7 to 58.3)	34.5 (25.6 to 46.4)	0.0 (0.0 to 0.0)	0.1 (0.1 to 0.1)	259.2 (213.5 to 314.4)	147.8 (114.8 to 187.5)	111.1 (82.3 to 149.3)	0.1 (0.0 to 0.1)	0.3 (0.3 to 0.3)
Iraq	2001	88.1 (72.8 to 106.2)	51.5 (40.3 to 64.7)	36.4 (27.1 to 48.7)	0.0 (0.0 to 0.0)	0.1 (0.1 to 0.1)	283.5 (234.1 to 341.6)	165.8 (129.6 to 208.0)	117.2 (87.3 to 156.8)	0.1 (0.0 to 0.1)	0.4 (0.4 to 0.4)
Iraq	2002	90.8 (75.9 to 108.4)	53.7 (42.4 to 66.3)	37.1 (27.9 to 49.0)	0.0 (0.0 to 0.0)	0.0 (0.0 to 0.0)	292.2 (244.2 to 348.9)	172.7 (136.4 to 213.5)	119.3 (89.7 to 157.7)	0.1 (0.0 to 0.1)	0.1 (0.1 to 0.1)
Iraq	2003	76.6 (64.8 to 90.9)	43.0 (34.5 to 52.6)	32.3 (24.4 to 42.2)	0.0 (0.0 to 0.0)	1.2 (1.2 to 1.2)	246.4 (208.3 to 292.4)	138.4 (110.9 to 169.2)	104.0 (78.4 to 135.7)	0.1 (0.0 to 0.1)	4.0 (4.0 to 4.0)
Iraq	2004	114.3 (97.8 to 133.4)	73.7 (60.0 to 89.4)	37.5 (28.7 to 47.9)	0.0 (0.0 to 0.1)	3.1 (3.1 to 3.1)	367.8 (314.6 to 429.3)	237.0 (193.0 to 287.6)	120.6 (92.2 to 154.0)	0.1 (0.0 to 0.2)	10.1 (10.1 to 10.1)
Iraq	2005	126.6 (111.1 to 144.2)	73.9 (60.8 to 88.6)	34.3 (26.5 to 43.7)	0.0 (0.0 to 0.1)	18.3 (18.3 to 18.3)	407.2 (357.3 to 463.9)	237.7 (195.5 to 285.0)	110.4 (85.3 to 140.7)	0.1 (0.0 to 0.2)	59.0 (59.0 to 59.0)
Iraq	2006	124.9 (109.4 to 142.1)	76.9 (63.5 to 90.7)	34.0 (26.4 to 42.8)	0.0 (0.0 to 0.1)	13.9 (13.9 to 13.9)	401.9 (351.9 to 457.1)	247.4 (204.3 to 291.8)	109.5 (85.1 to 137.7)	0.1 (0.1 to 0.2)	44.8 (44.8 to 44.8)
Iraq	2007	125.7 (109.9 to 142.9)	82.8 (69.6 to 97.8)	34.5 (26.8 to 42.9)	0.0 (0.0 to 0.1)	8.4 (8.4 to 8.4)	404.4 (353.6 to 459.7)	266.3 (223.9 to 314.8)	111.1 (86.2 to 138.1)	0.1 (0.1 to 0.2)	26.9 (26.9 to 26.9)
Iraq	2008	130.3 (113.9 to 148.6)	92.8 (78.1 to 109.0)	34.4 (26.8 to 42.6)	0.0 (0.0 to 0.1)	3.1 (3.1 to 3.1)	419.2 (366.5 to 478.1)	298.6 (251.3 to 350.6)	110.6 (86.3 to 137.0)	0.1 (0.1 to 0.2)	9.9 (9.9 to 9.9)
Iraq	2009	136.8 (119.8 to 155.9)	96.7 (81.4 to 113.4)	37.4 (29.8 to 46.0)	0.0 (0.0 to 0.1)	2.6 (2.6 to 2.6)	440.2 (385.6 to 501.6)	311.2 (261.8 to 365.0)	120.4 (96.0 to 148.1)	0.1 (0.1 to 0.2)	8.5 (8.5 to 8.5)
Iraq	2010	133.3 (116.3 to 152.1)	90.1 (75.6 to 106.4)	40.7 (32.5 to 50.1)	0.0 (0.0 to 0.1)	2.5 (2.5 to 2.5)	428.8 (374.1 to 489.2)	289.9 (243.1 to 342.3)	130.9 (104.6 to 161.1)	0.1 (0.0 to 0.2)	7.9 (7.9 to 7.9)
Iraq	2011	127.4 (110.4 to 145.4)	79.9 (66.5 to 95.1)	46.5 (37.5 to 56.8)	0.0 (0.0 to 0.1)	0.9 (0.9 to 0.9)	409.8 (355.3 to 467.8)	257.2 (214.0 to 306.0)	149.5 (120.6 to 182.8)	0.1 (0.0 to 0.2)	3.0 (3.0 to 3.0)
Iraq	2012	129.0 (112.1 to 147.9)	65.1 (53.9 to 77.9)	63.3 (51.3 to 77.3)	0.0 (0.0 to 0.1)	0.6 (0.6 to 0.6)	414.9 (360.7 to 475.8)	209.3 (173.3 to 250.6)	203.6 (165.1 to 248.6)	0.1 (0.0 to 0.2)	1.9 (1.9 to 1.9)
Iraq	2013	136.2 (118.6 to 156.9)	58.2 (47.5 to 70.7)	77.2 (63.0 to 93.1)	0.0 (0.0 to 0.1)	0.8 (0.8 to 0.8)	438.3 (381.6 to 504.7)	187.3 (152.7 to 227.5)	248.2 (202.6 to 299.4)	0.1 (0.0 to 0.2)	2.6 (2.6 to 2.6)
Iraq	2014	136.6 (118.1 to 157.4)	48.8 (39.1 to 59.6)	86.7 (71.0 to 104.5)	0.0 (0.0 to 0.1)	1.0 (1.0 to 1.0)	439.3 (380.1 to 506.4)	156.9 (125.9 to 191.9)	279.0 (228.3 to 336.2)	0.1 (0.0 to 0.2)	3.4 (3.4 to 3.4)
Iraq	2015	143.1 (122.8 to 166.9)	41.8 (33.0 to 51.4)	100.8 (82.3 to 123.4)	0.0 (0.0 to 0.1)	0.5 (0.5 to 0.5)	460.4 (395.1 to 537.0)	134.5 (106.3 to 165.4)	324.3 (264.8 to 397.1)	0.1 (0.0 to 0.2)	1.5 (1.5 to 1.5)

Country	Year	2018 US Dollars per capita					2018 PPP per capita				
		Health spending	Government health spending	Out-of-pocket health spending	Prepaid private health spending	Development assistance for health	Health spending	Government health spending	Out-of-pocket health spending	Prepaid private health spending	Development assistance for health
Iraq	2016	157.0 (132.8 to 186.7)	40.9 (31.8 to 51.0)	115.6 (93.7 to 143.2)	0.0 (0.0 to 0.1)	0.4 (0.4 to 0.4)	505.1 (427.2 to 600.7)	131.7 (102.4 to 164.1)	372.0 (301.6 to 460.9)	0.1 (0.0 to 0.2)	1.3 (1.3 to 1.3)
Iraq	2017	152.1 (126.6 to 181.8)	35.6 (27.7 to 44.5)	115.9 (92.5 to 145.4)	0.0 (0.0 to 0.1)	0.5 (0.5 to 0.5)	489.2 (407.4 to 584.8)	114.5 (89.2 to 143.2)	373.0 (297.5 to 467.8)	0.1 (0.0 to 0.2)	1.6 (1.6 to 1.6)
Iraq	2018	156.8 (131.1 to 188.3)	36.0 (28.0 to 45.1)	120.4 (96.0 to 151.1)	0.0 (0.0 to 0.1)	0.5 (0.5 to 0.5)	504.5 (421.7 to 605.8)	115.7 (90.2 to 145.2)	387.2 (308.9 to 486.1)	0.1 (0.0 to 0.2)	1.5 (1.5 to 1.5)
Iraq	2019	161.2 (134.1 to 194.2)	36.4 (28.4 to 45.7)	124.3 (98.9 to 155.2)	0.0 (0.0 to 0.1)	0.5 (0.4 to 0.5)	518.5 (431.4 to 624.7)	117.0 (91.2 to 147.2)	399.9 (318.2 to 499.2)	0.1 (0.0 to 0.2)	1.5 (1.4 to 1.5)
Iraq	2020	165.1 (137.3 to 198.2)	36.8 (28.8 to 46.4)	127.7 (101.7 to 159.8)	0.0 (0.0 to 0.1)	0.5 (0.4 to 0.5)	531.0 (441.6 to 637.5)	118.5 (92.5 to 149.4)	411.0 (327.3 to 514.2)	0.1 (0.0 to 0.2)	1.5 (1.3 to 1.6)
Iraq	2021	166.9 (139.3 to 200.3)	37.1 (29.0 to 46.8)	129.2 (103.1 to 161.7)	0.0 (0.0 to 0.1)	0.5 (0.4 to 0.5)	536.8 (448.0 to 644.2)	119.5 (93.2 to 150.6)	415.8 (331.7 to 520.3)	0.1 (0.1 to 0.2)	1.5 (1.3 to 1.6)
Iraq	2022	168.7 (140.6 to 202.1)	37.3 (29.2 to 47.1)	130.9 (104.3 to 163.9)	0.0 (0.0 to 0.1)	0.5 (0.4 to 0.5)	542.7 (452.5 to 650.3)	120.1 (93.8 to 151.5)	421.0 (335.5 to 527.2)	0.1 (0.1 to 0.2)	1.5 (1.3 to 1.7)
Iraq	2023	170.7 (141.9 to 204.7)	37.7 (29.4 to 47.4)	132.5 (105.5 to 165.8)	0.0 (0.0 to 0.1)	0.5 (0.4 to 0.5)	549.1 (456.5 to 658.5)	121.2 (94.5 to 152.6)	426.2 (339.5 to 533.3)	0.1 (0.1 to 0.2)	1.5 (1.3 to 1.7)
Iraq	2024	172.7 (143.5 to 206.6)	38.1 (29.7 to 48.0)	134.0 (106.7 to 167.8)	0.0 (0.0 to 0.1)	0.5 (0.4 to 0.5)	555.5 (461.6 to 664.8)	122.7 (95.4 to 154.3)	431.2 (343.4 to 539.9)	0.1 (0.1 to 0.2)	1.5 (1.3 to 1.8)
Iraq	2025	174.6 (145.7 to 210.4)	38.3 (29.8 to 48.4)	135.7 (108.0 to 170.0)	0.0 (0.0 to 0.1)	0.5 (0.4 to 0.6)	561.6 (468.8 to 676.8)	123.4 (95.9 to 155.7)	436.5 (347.5 to 546.9)	0.1 (0.1 to 0.2)	1.5 (1.3 to 1.8)
Iraq	2026	176.5 (146.9 to 211.0)	38.7 (30.0 to 48.8)	137.3 (109.2 to 172.3)	0.0 (0.0 to 0.1)	0.5 (0.4 to 0.6)	567.9 (472.5 to 678.7)	124.5 (96.7 to 157.1)	441.8 (351.4 to 554.3)	0.1 (0.1 to 0.2)	1.6 (1.3 to 1.8)
Iraq	2027	179.1 (149.2 to 216.7)	39.2 (30.5 to 49.6)	139.3 (110.8 to 175.0)	0.0 (0.0 to 0.1)	0.5 (0.4 to 0.6)	576.1 (480.0 to 697.1)	126.1 (98.0 to 159.5)	448.3 (356.3 to 562.9)	0.1 (0.1 to 0.2)	1.6 (1.3 to 1.9)
Iraq	2028	181.3 (151.2 to 217.3)	39.7 (30.8 to 50.2)	141.1 (112.1 to 177.4)	0.0 (0.0 to 0.1)	0.5 (0.4 to 0.6)	583.4 (486.4 to 699.1)	127.7 (99.0 to 161.4)	454.0 (360.6 to 570.8)	0.1 (0.1 to 0.2)	1.6 (1.3 to 1.9)
Iraq	2029	183.5 (152.3 to 221.7)	40.2 (31.3 to 50.9)	142.7 (113.3 to 179.8)	0.0 (0.0 to 0.1)	0.5 (0.4 to 0.6)	590.3 (490.1 to 713.4)	129.4 (100.8 to 163.7)	459.2 (364.5 to 578.3)	0.1 (0.1 to 0.2)	1.6 (1.3 to 2.0)
Iraq	2030	186.0 (154.7 to 223.6)	40.9 (31.8 to 51.4)	144.5 (114.7 to 182.3)	0.0 (0.0 to 0.1)	0.5 (0.4 to 0.6)	598.3 (497.7 to 719.4)	131.5 (102.3 to 165.3)	465.0 (368.9 to 586.6)	0.1 (0.1 to 0.2)	1.6 (1.3 to 2.0)
Iraq	2031	188.5 (156.1 to 228.5)	41.7 (32.3 to 52.6)	146.2 (116.0 to 184.6)	0.0 (0.0 to 0.1)	0.5 (0.4 to 0.6)	606.3 (502.1 to 735.0)	134.1 (104.1 to 169.1)	470.4 (373.1 to 593.7)	0.1 (0.1 to 0.2)	1.7 (1.3 to 2.1)
Iraq	2032	191.1 (158.6 to 230.7)	42.5 (33.1 to 53.9)	148.0 (117.5 to 186.8)	0.0 (0.0 to 0.1)	0.5 (0.4 to 0.7)	614.8 (510.3 to 742.2)	136.8 (106.4 to 173.3)	476.2 (377.9 to 601.1)	0.1 (0.1 to 0.3)	1.7 (1.4 to 2.1)
Iraq	2033	193.9 (160.4 to 235.1)	43.5 (33.8 to 54.8)	149.9 (118.7 to 189.3)	0.0 (0.0 to 0.1)	0.5 (0.4 to 0.7)	623.8 (516.2 to 756.4)	139.8 (108.9 to 176.4)	482.1 (381.9 to 609.1)	0.2 (0.1 to 0.3)	1.7 (1.4 to 2.2)
Iraq	2034	196.8 (163.7 to 239.1)	44.5 (34.5 to 56.5)	151.7 (120.0 to 192.8)	0.0 (0.0 to 0.1)	0.5 (0.4 to 0.7)	633.2 (526.8 to 769.2)	143.1 (111.0 to 181.8)	488.2 (386.1 to 620.2)	0.2 (0.1 to 0.3)	1.7 (1.4 to 2.2)
Iraq	2035	199.4 (164.6 to 241.5)	45.4 (35.2 to 57.8)	153.4 (121.2 to 194.9)	0.1 (0.0 to 0.1)	0.5 (0.4 to 0.7)	641.4 (529.7 to 776.9)	145.9 (113.1 to 186.1)	493.5 (390.0 to 626.9)	0.2 (0.1 to 0.3)	1.8 (1.4 to 2.3)
Iraq	2036	202.0 (168.1 to 245.0)	46.2 (35.9 to 59.0)	155.2 (122.4 to 197.7)	0.1 (0.0 to 0.1)	0.6 (0.4 to 0.7)	649.9 (540.9 to 788.2)	148.8 (115.4 to 189.8)	499.2 (393.9 to 636.1)	0.2 (0.1 to 0.3)	1.8 (1.4 to 2.4)

		2018 US Dollars per capita					2018 PPP per capita				
Country	Year	Health spending	Government health spending	Out-of-pocket health spending	Prepaid private health spending	Development assistance for health	Health spending	Government health spending	Out-of-pocket health spending	Prepaid private health spending	Development assistance for health
Iraq	2037	204.7 (169.3 to 248.3)	47.2 (36.6 to 60.2)	157.0 (123.6 to 199.8)	0.1 (0.0 to 0.1)	0.6 (0.4 to 0.8)	658.7 (544.7 to 799.0)	151.7 (117.8 to 193.6)	505.0 (397.7 to 642.9)	0.2 (0.1 to 0.3)	1.8 (1.4 to 2.4)
Iraq	2038	207.5 (172.9 to 252.0)	48.1 (37.4 to 61.4)	158.7 (124.8 to 201.9)	0.1 (0.0 to 0.1)	0.6 (0.4 to 0.8)	667.5 (556.3 to 810.8)	154.8 (120.2 to 197.5)	510.7 (401.5 to 649.5)	0.2 (0.1 to 0.3)	1.8 (1.4 to 2.5)
Iraq	2039	210.8 (174.4 to 257.1)	49.2 (38.4 to 62.9)	160.9 (126.3 to 204.5)	0.1 (0.0 to 0.1)	0.6 (0.4 to 0.8)	678.1 (560.9 to 827.2)	158.4 (123.6 to 202.3)	517.6 (406.2 to 657.8)	0.2 (0.1 to 0.3)	1.8 (1.4 to 2.6)
Iraq	2040	213.6 (177.3 to 258.7)	50.2 (38.9 to 64.2)	162.7 (127.4 to 207.1)	0.1 (0.0 to 0.1)	0.6 (0.4 to 0.8)	687.0 (570.5 to 832.1)	161.5 (125.3 to 206.7)	523.5 (410.0 to 666.1)	0.2 (0.1 to 0.3)	1.9 (1.4 to 2.6)
Iraq	2041	216.5 (178.3 to 263.4)	51.2 (39.6 to 65.8)	164.7 (128.7 to 210.7)	0.1 (0.0 to 0.1)	0.6 (0.4 to 0.8)	696.5 (573.6 to 847.5)	164.7 (127.4 to 211.5)	529.7 (414.2 to 677.9)	0.2 (0.1 to 0.3)	1.9 (1.4 to 2.6)
Iraq	2042	219.6 (182.2 to 266.6)	52.2 (40.5 to 67.2)	166.7 (130.2 to 212.8)	0.1 (0.0 to 0.1)	0.6 (0.4 to 0.9)	706.5 (586.2 to 857.9)	168.1 (130.2 to 216.3)	536.3 (419.0 to 684.7)	0.2 (0.1 to 0.3)	1.9 (1.4 to 2.8)
Iraq	2043	222.4 (184.1 to 269.3)	53.2 (41.2 to 68.5)	168.5 (131.8 to 214.9)	0.1 (0.0 to 0.1)	0.6 (0.4 to 0.9)	715.5 (592.1 to 866.5)	171.1 (132.6 to 220.5)	542.2 (423.9 to 691.4)	0.2 (0.1 to 0.4)	2.0 (1.4 to 2.8)
Iraq	2044	225.6 (187.0 to 274.0)	54.3 (41.9 to 70.0)	170.7 (133.2 to 217.8)	0.1 (0.0 to 0.1)	0.6 (0.5 to 0.9)	725.9 (601.6 to 881.6)	174.7 (134.7 to 225.2)	549.1 (428.5 to 700.8)	0.2 (0.1 to 0.4)	2.0 (1.5 to 2.9)
Iraq	2045	228.8 (188.4 to 278.4)	55.4 (42.5 to 71.5)	172.7 (134.5 to 220.7)	0.1 (0.0 to 0.1)	0.6 (0.5 to 1.0)	736.0 (606.0 to 895.7)	178.1 (136.7 to 230.1)	555.7 (432.6 to 709.9)	0.2 (0.1 to 0.4)	2.0 (1.5 to 3.1)
Iraq	2046	231.9 (191.9 to 283.4)	56.3 (42.9 to 72.9)	174.9 (136.1 to 223.6)	0.1 (0.0 to 0.1)	0.6 (0.5 to 1.0)	745.9 (617.5 to 911.6)	181.1 (138.0 to 234.6)	562.6 (437.9 to 719.4)	0.2 (0.1 to 0.4)	2.1 (1.5 to 3.2)
Iraq	2047	234.8 (195.5 to 286.2)	57.2 (43.5 to 74.1)	176.9 (137.7 to 226.3)	0.1 (0.0 to 0.1)	0.7 (0.5 to 1.0)	755.5 (628.9 to 920.8)	184.1 (139.9 to 238.5)	569.1 (443.0 to 728.2)	0.2 (0.1 to 0.4)	2.1 (1.5 to 3.3)
Iraq	2048	237.8 (196.4 to 289.0)	58.1 (44.0 to 75.1)	178.9 (139.3 to 229.0)	0.1 (0.0 to 0.1)	0.7 (0.5 to 1.1)	765.0 (631.9 to 929.6)	186.9 (141.6 to 241.6)	575.6 (448.1 to 736.8)	0.2 (0.1 to 0.4)	2.1 (1.5 to 3.5)
Iraq	2049	240.8 (199.1 to 293.2)	59.0 (44.6 to 76.3)	181.1 (140.9 to 231.7)	0.1 (0.0 to 0.1)	0.7 (0.5 to 1.1)	774.9 (640.5 to 943.2)	189.9 (143.6 to 245.3)	582.5 (453.4 to 745.4)	0.2 (0.1 to 0.4)	2.2 (1.6 to 3.5)
Iraq	2050	243.6 (201.5 to 293.8)	59.9 (45.2 to 78.0)	183.0 (142.4 to 234.1)	0.1 (0.0 to 0.1)	0.7 (0.5 to 1.1)	783.8 (648.2 to 945.1)	192.8 (145.4 to 250.8)	588.6 (458.1 to 753.1)	0.3 (0.1 to 0.4)	2.2 (1.5 to 3.7)
Ireland	1995	1715.2 (1576.6 to 1864.7)	1289.3 (1165.7 to 1424.4)	182.9 (153.6 to 214.8)	243.0 (199.4 to 295.3)	0.0 (0.0 to 0.0)	1748.0 (1606.8 to 1900.3)	1314.0 (1188.0 to 1451.6)	186.4 (156.5 to 218.9)	247.6 (203.3 to 301.0)	0.0 (0.0 to 0.0)
Ireland	1996	1823.7 (1699.8 to 1947.1)	1377.8 (1261.0 to 1496.9)	195.0 (169.2 to 223.4)	250.9 (213.9 to 292.3)	0.0 (0.0 to 0.0)	1858.6 (1732.3 to 1984.4)	1404.1 (1285.1 to 1525.6)	198.8 (172.4 to 227.6)	255.7 (218.0 to 297.9)	0.0 (0.0 to 0.0)
Ireland	1997	1994.7 (1877.5 to 2111.5)	1523.8 (1417.1 to 1631.6)	210.0 (185.8 to 235.4)	260.8 (227.3 to 298.6)	0.0 (0.0 to 0.0)	2032.8 (1913.4 to 2151.9)	1552.9 (1444.3 to 1662.8)	214.0 (189.4 to 239.9)	265.8 (231.7 to 304.3)	0.0 (0.0 to 0.0)
Ireland	1998	2155.6 (2044.2 to 2263.6)	1662.1 (1556.1 to 1762.9)	225.0 (201.2 to 248.8)	268.4 (237.2 to 304.4)	0.0 (0.0 to 0.0)	2196.8 (2083.3 to 2306.9)	1693.9 (1585.8 to 1796.6)	229.3 (205.0 to 253.6)	273.6 (241.7 to 310.3)	0.0 (0.0 to 0.0)
Ireland	1999	2374.2 (2263.2 to 2487.2)	1843.6 (1737.0 to 1952.0)	254.4 (230.4 to 280.2)	276.2 (243.5 to 311.7)	0.0 (0.0 to 0.0)	2419.6 (2306.5 to 2534.7)	1878.8 (1770.2 to 1989.3)	259.3 (234.8 to 285.5)	281.5 (248.2 to 317.7)	0.0 (0.0 to 0.0)
Ireland	2000	2653.6 (2539.3 to 2782.9)	2068.3 (1960.9 to 2189.9)	295.2 (269.3 to 321.2)	290.0 (256.4 to 324.1)	0.0 (0.0 to 0.0)	2704.3 (2587.9 to 2836.1)	2107.9 (1998.4 to 2231.8)	300.8 (274.5 to 327.4)	295.6 (261.3 to 330.3)	0.0 (0.0 to 0.0)
Ireland	2001	2923.7 (2805.0 to 3054.8)	2287.7 (2178.7 to 2409.4)	327.2 (299.7 to 355.2)	308.8 (273.4 to 346.3)	0.0 (0.0 to 0.0)	2979.7 (2858.7 to 3113.2)	2331.5 (2220.4 to 2455.5)	333.4 (305.4 to 362.0)	314.7 (278.7 to 352.9)	0.0 (0.0 to 0.0)

Country	Year	2018 US Dollars per capita					2018 PPP per capita				
		Health spending	Government health spending	Out-of-pocket health spending	Prepaid private health spending	Development assistance for health	Health spending	Government health spending	Out-of-pocket health spending	Prepaid private health spending	Development assistance for health
Ireland	2002	3200.1 (3079.7 to 3327.8)	2515.4 (2403.3 to 2635.4)	367.8 (340.1 to 394.9)	316.9 (280.2 to 355.9)	0.0 (0.0 to 0.0)	3261.3 (3138.6 to 3391.5)	2563.5 (2449.3 to 2685.8)	374.9 (346.6 to 402.4)	322.9 (285.6 to 362.7)	0.0 (0.0 to 0.0)
Ireland	2003	3406.1 (3283.1 to 3539.2)	2678.5 (2566.7 to 2804.6)	422.5 (392.9 to 451.6)	305.1 (271.4 to 343.4)	0.0 (0.0 to 0.0)	3471.2 (3345.9 to 3606.9)	2729.7 (2615.8 to 2858.2)	430.6 (400.4 to 460.3)	310.9 (276.6 to 350.0)	0.0 (0.0 to 0.0)
Ireland	2004	3675.8 (3553.9 to 3804.5)	2892.2 (2774.1 to 3010.3)	469.1 (440.7 to 495.9)	314.5 (281.3 to 352.0)	0.0 (0.0 to 0.0)	3746.1 (3621.9 to 3877.2)	2947.5 (2827.2 to 3067.8)	478.1 (449.2 to 505.4)	320.5 (286.6 to 358.8)	0.0 (0.0 to 0.0)
Ireland	2005	3946.4 (3826.5 to 4086.9)	3105.5 (2986.5 to 3238.6)	519.1 (489.8 to 549.8)	321.7 (286.9 to 359.3)	0.0 (0.0 to 0.0)	4021.9 (3899.7 to 4165.0)	3164.9 (3043.7 to 3300.6)	529.1 (499.2 to 560.3)	327.9 (292.4 to 366.2)	0.0 (0.0 to 0.0)
Ireland	2006	4203.6 (4073.7 to 4341.2)	3312.4 (3191.9 to 3455.2)	547.5 (515.5 to 579.8)	343.7 (310.9 to 381.3)	0.0 (0.0 to 0.0)	4284.0 (4151.6 to 4424.3)	3375.8 (3252.9 to 3521.3)	558.0 (525.4 to 590.9)	350.3 (316.8 to 388.6)	0.0 (0.0 to 0.0)
Ireland	2007	4575.7 (4444.0 to 4715.7)	3621.4 (3498.5 to 3759.7)	563.5 (531.3 to 594.1)	390.8 (355.0 to 428.5)	0.0 (0.0 to 0.0)	4663.3 (4529.0 to 4805.9)	3690.7 (3565.4 to 3831.6)	574.3 (541.5 to 605.4)	398.3 (361.8 to 436.7)	0.0 (0.0 to 0.0)
Ireland	2008	4798.0 (4663.5 to 4931.9)	3766.0 (3642.9 to 3893.3)	594.7 (561.1 to 628.2)	437.3 (400.2 to 475.0)	0.0 (0.0 to 0.0)	4889.8 (4752.7 to 5026.2)	3838.0 (3712.6 to 3967.7)	606.1 (571.8 to 640.3)	445.7 (407.8 to 484.1)	0.0 (0.0 to 0.0)
Ireland	2009	5027.0 (4897.3 to 5157.9)	3866.7 (3741.4 to 3986.6)	646.1 (613.5 to 676.6)	514.1 (473.9 to 554.8)	0.0 (0.0 to 0.0)	5123.1 (4990.9 to 5256.5)	3940.7 (3812.9 to 4062.8)	658.5 (625.2 to 689.5)	523.9 (482.9 to 565.4)	0.0 (0.0 to 0.0)
Ireland	2010	5247.7 (5105.9 to 5399.1)	3946.7 (3816.9 to 4081.0)	696.7 (663.4 to 732.1)	604.3 (563.1 to 647.9)	0.0 (0.0 to 0.0)	5348.1 (5203.6 to 5502.3)	4022.2 (3889.9 to 4159.1)	710.0 (676.1 to 746.1)	615.9 (573.8 to 660.3)	0.0 (0.0 to 0.0)
Ireland	2011	5306.7 (5164.4 to 5457.6)	3859.2 (3725.4 to 3993.2)	715.9 (681.1 to 752.6)	731.6 (686.8 to 779.0)	0.0 (0.0 to 0.0)	5408.2 (5263.2 to 5562.0)	3933.0 (3796.6 to 4069.6)	729.6 (694.1 to 767.0)	745.5 (699.9 to 793.9)	0.0 (0.0 to 0.0)
Ireland	2012	5183.3 (5048.6 to 5325.5)	3682.1 (3554.5 to 3815.2)	713.2 (681.4 to 747.5)	788.0 (743.8 to 834.4)	0.0 (0.0 to 0.0)	5282.5 (5145.1 to 5427.4)	3752.5 (3622.5 to 3888.1)	726.9 (694.5 to 761.8)	803.1 (758.0 to 850.3)	0.0 (0.0 to 0.0)
Ireland	2013	4967.3 (4836.7 to 5102.6)	3490.2 (3375.6 to 3611.9)	698.9 (664.8 to 733.3)	778.2 (733.1 to 823.0)	0.0 (0.0 to 0.0)	5062.3 (4929.3 to 5200.2)	3556.9 (3440.2 to 3681.0)	712.3 (677.5 to 747.3)	793.1 (747.2 to 838.7)	0.0 (0.0 to 0.0)
Ireland	2014	4866.2 (4738.8 to 4991.0)	3420.2 (3300.4 to 3536.5)	686.3 (652.3 to 718.3)	759.7 (717.8 to 802.7)	0.0 (0.0 to 0.0)	4959.3 (4829.4 to 5086.4)	3485.7 (3363.5 to 3604.1)	699.4 (664.8 to 732.0)	774.2 (731.5 to 818.1)	0.0 (0.0 to 0.0)
Ireland	2015	5195.4 (5056.9 to 5333.7)	3713.4 (3582.1 to 3842.5)	695.9 (659.6 to 732.2)	786.1 (741.3 to 834.2)	0.0 (0.0 to 0.0)	5294.8 (5153.6 to 5435.7)	3784.4 (3650.6 to 3916.0)	709.3 (672.2 to 746.2)	801.1 (755.4 to 850.2)	0.0 (0.0 to 0.0)
Ireland	2016	5097.0 (4900.8 to 5287.7)	3665.3 (3503.9 to 3838.1)	673.9 (628.7 to 724.4)	757.8 (693.9 to 826.3)	0.0 (0.0 to 0.0)	5194.5 (4994.5 to 5388.9)	3735.4 (3570.9 to 3911.5)	686.8 (640.7 to 738.3)	772.3 (707.2 to 842.1)	0.0 (0.0 to 0.0)
Ireland	2017	5326.7 (5113.9 to 5525.1)	3804.3 (3620.4 to 3988.6)	707.9 (658.5 to 760.6)	814.5 (746.0 to 887.2)	0.0 (0.0 to 0.0)	5428.6 (5211.7 to 5630.7)	3877.1 (3689.6 to 4064.9)	721.4 (671.1 to 775.1)	830.1 (760.3 to 904.2)	0.0 (0.0 to 0.0)
Ireland	2018	5474.1 (5246.6 to 5693.7)	3907.3 (3700.0 to 4102.1)	730.2 (675.7 to 784.5)	836.6 (764.4 to 911.1)	0.0 (0.0 to 0.0)	5578.8 (5347.0 to 5802.6)	3982.0 (3770.8 to 4180.6)	744.2 (688.6 to 799.6)	852.6 (779.0 to 928.5)	0.0 (0.0 to 0.0)
Ireland	2019	5630.7 (5389.7 to 5856.9)	4015.2 (3805.7 to 4223.1)	754.4 (696.9 to 811.9)	861.1 (787.1 to 939.0)	0.0 (0.0 to 0.0)	5738.4 (5492.8 to 5969.0)	4092.0 (3878.5 to 4303.9)	768.8 (710.2 to 827.4)	877.6 (802.2 to 957.0)	0.0 (0.0 to 0.0)
Ireland	2020	5787.6 (5536.5 to 6030.8)	4122.3 (3903.1 to 4356.6)	779.2 (716.5 to 841.8)	886.1 (810.1 to 965.3)	0.0 (0.0 to 0.0)	5898.3 (5642.4 to 6146.2)	4201.2 (3977.8 to 4439.9)	794.1 (730.3 to 857.9)	903.1 (825.6 to 983.7)	0.0 (0.0 to 0.0)
Ireland	2021	5953.5 (5697.6 to 6210.8)	4235.3 (4002.5 to 4477.9)	805.5 (738.2 to 874.9)	912.7 (834.2 to 995.0)	0.0 (0.0 to 0.0)	6067.4 (5806.6 to 6329.6)	4316.3 (4079.1 to 4563.6)	820.9 (752.4 to 891.7)	930.2 (850.1 to 1014.0)	0.0 (0.0 to 0.0)
Ireland	2022	6097.6 (5824.1 to 6370.4)	4331.0 (4095.6 to 4588.3)	830.7 (764.1 to 901.4)	935.9 (853.6 to 1019.4)	0.0 (0.0 to 0.0)	6214.2 (5935.5 to 6492.2)	4413.9 (4173.9 to 4676.1)	846.6 (778.7 to 918.7)	953.8 (869.9 to 1038.9)	0.0 (0.0 to 0.0)

Country	Year	2018 US Dollars per capita					2018 PPP per capita				
		Health spending	Government health spending	Out-of-pocket health spending	Prepaid private health spending	Development assistance for health	Health spending	Government health spending	Out-of-pocket health spending	Prepaid private health spending	Development assistance for health
Ireland	2023	6162.2 (5890.6 to 6430.4)	4365.8 (4123.1 to 4625.2)	849.9 (781.2 to 923.1)	946.4 (863.6 to 1031.8)	0.0 (0.0 to 0.0)	6280.1 (6003.2 to 6553.4)	4449.3 (4202.0 to 4713.7)	866.2 (796.1 to 940.7)	964.5 (880.1 to 1051.6)	0.0 (0.0 to 0.0)
Ireland	2024	6206.6 (5926.7 to 6474.4)	4384.3 (4132.9 to 4638.1)	867.5 (796.2 to 941.7)	954.8 (870.9 to 1043.0)	0.0 (0.0 to 0.0)	6325.3 (6040.0 to 6598.2)	4468.2 (4212.0 to 4726.9)	884.1 (811.5 to 959.7)	973.0 (887.6 to 1063.0)	0.0 (0.0 to 0.0)
Ireland	2025	6237.8 (5960.4 to 6516.3)	4403.0 (4150.5 to 4658.9)	871.1 (799.4 to 951.1)	963.7 (879.8 to 1052.8)	0.0 (0.0 to 0.0)	6357.1 (6074.4 to 6641.0)	4487.2 (4229.9 to 4748.0)	887.8 (814.7 to 969.3)	982.1 (896.6 to 1072.9)	0.0 (0.0 to 0.0)
Ireland	2026	6271.5 (5982.9 to 6550.1)	4423.3 (4166.8 to 4684.0)	875.1 (800.0 to 962.2)	973.0 (888.6 to 1062.7)	0.0 (0.0 to 0.0)	6391.4 (6097.4 to 6675.3)	4507.9 (4246.5 to 4773.6)	891.8 (815.3 to 980.6)	991.6 (905.6 to 1083.1)	0.0 (0.0 to 0.0)
Ireland	2027	6302.7 (6012.1 to 6589.0)	4441.9 (4177.4 to 4710.3)	878.8 (800.2 to 974.9)	982.0 (896.9 to 1071.3)	0.0 (0.0 to 0.0)	6423.3 (6127.1 to 6715.0)	4526.8 (4257.3 to 4800.4)	895.6 (815.5 to 993.6)	1000.8 (914.0 to 1091.8)	0.0 (0.0 to 0.0)
Ireland	2028	6355.5 (6050.5 to 6650.2)	4475.7 (4197.0 to 4749.4)	885.3 (802.7 to 998.5)	994.5 (907.8 to 1085.3)	0.0 (0.0 to 0.0)	6477.1 (6166.2 to 6777.4)	4561.3 (4277.3 to 4840.3)	902.2 (818.0 to 1017.6)	1013.5 (925.2 to 1106.0)	0.0 (0.0 to 0.0)
Ireland	2029	6501.7 (6200.4 to 6799.9)	4576.1 (4285.9 to 4854.3)	903.6 (816.9 to 1032.1)	1022.0 (932.5 to 1117.6)	0.0 (0.0 to 0.0)	6626.1 (6319.0 to 6930.0)	4663.7 (4367.9 to 4947.1)	920.9 (832.5 to 1051.9)	1041.5 (950.4 to 1139.0)	0.0 (0.0 to 0.0)
Ireland	2030	6688.9 (6341.9 to 7022.1)	4705.6 (4401.3 to 4997.3)	927.1 (837.1 to 1072.1)	1056.2 (964.0 to 1156.5)	0.0 (0.0 to 0.0)	6816.9 (6463.2 to 7156.4)	4795.6 (4485.5 to 5092.9)	944.8 (853.1 to 1092.6)	1076.4 (982.4 to 1178.6)	0.0 (0.0 to 0.0)
Ireland	2031	6845.4 (6511.7 to 7175.9)	4802.6 (4482.0 to 5104.5)	950.4 (855.4 to 1111.2)	1092.4 (994.7 to 1197.9)	0.0 (0.0 to 0.0)	6976.3 (6636.2 to 7313.1)	4894.5 (4567.7 to 5202.2)	968.5 (871.8 to 1132.5)	1113.3 (1013.7 to 1220.8)	0.0 (0.0 to 0.0)
Ireland	2032	7006.1 (6619.9 to 7351.6)	4901.8 (4577.5 to 5215.3)	974.3 (872.8 to 1147.4)	1130.1 (1027.5 to 1243.3)	0.0 (0.0 to 0.0)	7140.2 (6746.5 to 7492.2)	4995.5 (4665.0 to 5315.1)	992.9 (889.5 to 1169.3)	1151.7 (1047.2 to 1267.1)	0.0 (0.0 to 0.0)
Ireland	2033	7171.3 (6798.1 to 7525.2)	5003.6 (4662.8 to 5329.1)	998.7 (891.3 to 1182.9)	1169.1 (1061.7 to 1288.4)	0.0 (0.0 to 0.0)	7308.5 (6928.1 to 7669.2)	5099.3 (4752.0 to 5431.1)	1017.8 (908.4 to 1205.5)	1191.4 (1082.0 to 1313.1)	0.0 (0.0 to 0.0)
Ireland	2034	7336.3 (6919.3 to 7733.3)	5104.6 (4747.1 to 5442.3)	1023.0 (907.5 to 1216.9)	1208.6 (1097.5 to 1333.2)	0.0 (0.0 to 0.0)	7476.6 (7051.7 to 7881.3)	5202.3 (4837.9 to 5546.3)	1042.6 (924.9 to 1240.2)	1231.8 (1118.4 to 1358.7)	0.0 (0.0 to 0.0)
Ireland	2035	7492.1 (7074.4 to 7894.0)	5198.6 (4825.6 to 5547.9)	1046.2 (925.0 to 1253.5)	1247.3 (1129.2 to 1374.3)	0.0 (0.0 to 0.0)	7635.4 (7209.7 to 8045.0)	5298.0 (4917.9 to 5654.0)	1066.2 (942.7 to 1277.5)	1271.1 (1150.8 to 1400.6)	0.0 (0.0 to 0.0)
Ireland	2036	7643.3 (7187.3 to 8086.5)	5289.0 (4901.3 to 5657.4)	1068.6 (942.0 to 1290.9)	1285.7 (1163.0 to 1419.8)	0.0 (0.0 to 0.0)	7789.5 (7324.8 to 8241.2)	5390.1 (4995.1 to 5765.6)	1089.0 (960.0 to 1315.6)	1310.3 (1185.2 to 1447.0)	0.0 (0.0 to 0.0)
Ireland	2037	7794.3 (7338.5 to 8241.8)	5379.0 (4974.4 to 5762.0)	1090.8 (959.2 to 1326.7)	1324.5 (1197.3 to 1464.6)	0.0 (0.0 to 0.0)	7943.4 (7478.8 to 8399.4)	5481.9 (5069.6 to 5872.3)	1111.7 (977.5 to 1352.0)	1349.8 (1220.2 to 1492.6)	0.0 (0.0 to 0.0)
Ireland	2038	7938.9 (7444.2 to 8423.6)	5463.5 (5038.6 to 5867.9)	1112.4 (973.7 to 1359.8)	1363.1 (1232.7 to 1508.5)	0.0 (0.0 to 0.0)	8090.8 (7586.6 to 8584.7)	5568.0 (5134.9 to 5980.1)	1133.6 (992.3 to 1385.8)	1389.1 (1256.3 to 1537.4)	0.0 (0.0 to 0.0)
Ireland	2039	8089.1 (7579.1 to 8579.4)	5552.4 (5108.1 to 5979.9)	1134.1 (987.3 to 1393.0)	1402.5 (1267.2 to 1553.5)	0.0 (0.0 to 0.0)	8243.8 (7724.1 to 8743.5)	5658.6 (5205.8 to 6094.3)	1155.8 (1006.1 to 1419.6)	1429.4 (1291.4 to 1583.2)	0.0 (0.0 to 0.0)
Ireland	2040	8233.9 (7691.2 to 8770.1)	5636.6 (5170.6 to 6087.3)	1155.3 (1002.3 to 1426.4)	1442.0 (1299.9 to 1601.1)	0.0 (0.0 to 0.0)	8391.4 (7838.3 to 8937.8)	5744.4 (5269.5 to 6203.8)	1177.4 (1021.4 to 1453.7)	1469.6 (1324.7 to 1631.8)	0.0 (0.0 to 0.0)
Ireland	2041	8380.9 (7834.5 to 8944.5)	5722.0 (5237.7 to 6198.4)	1176.6 (1017.2 to 1459.3)	1482.3 (1332.0 to 1655.8)	0.0 (0.0 to 0.0)	8541.2 (7984.3 to 9115.6)	5831.5 (5337.9 to 6317.0)	1199.1 (1036.6 to 1487.2)	1510.6 (1357.4 to 1687.5)	0.0 (0.0 to 0.0)
Ireland	2042	8532.1 (7945.7 to 9135.7)	5810.4 (5303.1 to 6313.6)	1198.0 (1031.2 to 1491.8)	1523.6 (1364.6 to 1707.9)	0.0 (0.0 to 0.0)	8695.3 (8097.7 to 9310.5)	5921.6 (5404.5 to 6434.3)	1221.0 (1051.0 to 1520.3)	1552.7 (1390.7 to 1740.6)	0.0 (0.0 to 0.0)
Ireland	2043	8683.9 (8095.0 to 9279.3)	5898.3 (5361.6 to 6422.5)	1219.7 (1045.7 to 1523.5)	1566.0 (1398.0 to 1759.2)	0.0 (0.0 to 0.0)	8850.0 (8249.8 to 9456.8)	6011.1 (5464.2 to 6545.3)	1243.0 (1065.7 to 1552.6)	1595.9 (1424.7 to 1792.9)	0.0 (0.0 to 0.0)

		2018 US Dollars per capita					2018 PPP per capita				
Country	Year	Health spending	Government health spending	Out-of-pocket health spending	Prepaid private health spending	Development assistance for health	Health spending	Government health spending	Out-of-pocket health spending	Prepaid private health spending	Development assistance for health
Ireland	2044	8837.7 (8199.8 to 9506.5)	5987.5 (5421.2 to 6537.5)	1241.2 (1060.6 to 1554.4)	1609.0 (1437.3 to 1809.9)	0.0 (0.0 to 0.0)	9006.8 (8356.6 to 9688.3)	6102.0 (5524.9 to 6662.6)	1265.0 (1080.8 to 1584.1)	1639.8 (1464.8 to 1844.5)	0.0 (0.0 to 0.0)
Ireland	2045	8992.5 (8351.3 to 9628.8)	6078.0 (5491.3 to 6654.5)	1262.4 (1077.2 to 1584.2)	1652.1 (1471.4 to 1864.7)	0.0 (0.0 to 0.0)	9164.5 (8511.0 to 9812.9)	6194.2 (5596.4 to 6781.8)	1286.6 (1097.8 to 1614.5)	1683.7 (1499.5 to 1900.4)	0.0 (0.0 to 0.0)
Ireland	2046	9151.8 (8477.7 to 9876.8)	6171.7 (5564.4 to 6775.6)	1283.8 (1094.2 to 1613.3)	1696.3 (1506.1 to 1919.5)	0.0 (0.0 to 0.0)	9326.9 (8639.8 to 10065.7)	6289.8 (5670.8 to 6905.2)	1308.4 (1115.1 to 1644.1)	1728.7 (1534.9 to 1956.2)	0.0 (0.0 to 0.0)
Ireland	2047	9310.5 (8614.8 to 10021.4)	6265.7 (5633.3 to 6902.0)	1304.6 (1111.0 to 1641.0)	1740.2 (1542.0 to 1973.3)	0.0 (0.0 to 0.0)	9488.6 (8779.6 to 10213.1)	6385.5 (5741.1 to 7034.0)	1329.6 (1132.2 to 1672.4)	1773.5 (1571.5 to 2011.0)	0.0 (0.0 to 0.0)
Ireland	2048	9476.3 (8736.5 to 10251.4)	6365.0 (5702.8 to 7031.4)	1325.9 (1127.1 to 1669.4)	1785.4 (1577.1 to 2031.3)	0.0 (0.0 to 0.0)	9657.6 (8903.6 to 10447.4)	6486.8 (5811.9 to 7165.9)	1351.3 (1148.7 to 1701.3)	1819.5 (1607.3 to 2070.2)	0.0 (0.0 to 0.0)
Ireland	2049	9651.2 (8897.0 to 10442.3)	6471.5 (5784.7 to 7166.6)	1347.8 (1146.1 to 1698.6)	1831.9 (1613.2 to 2091.4)	0.0 (0.0 to 0.0)	9835.8 (9067.2 to 10642.1)	6595.3 (5895.3 to 7303.7)	1373.6 (1168.0 to 1731.0)	1866.9 (1644.0 to 2131.4)	0.0 (0.0 to 0.0)
Ireland	2050	9838.5 (9036.8 to 10659.2)	6586.6 (5866.3 to 7314.1)	1371.1 (1162.9 to 1729.0)	1880.9 (1649.4 to 2152.3)	0.0 (0.0 to 0.0)	10026.7 (9209.7 to 10863.1)	6712.6 (5978.5 to 7454.0)	1397.3 (1185.2 to 1762.0)	1916.8 (1680.9 to 2193.5)	0.0 (0.0 to 0.0)
Israel	1995	1953.4 (1882.0 to 2029.2)	1319.5 (1264.7 to 1376.1)	586.9 (541.0 to 638.7)	47.1 (34.3 to 63.1)	0.0 (0.0 to 0.0)	1839.9 (1772.6 to 1911.2)	1242.8 (1191.1 to 1296.0)	552.7 (509.5 to 601.5)	44.3 (32.3 to 59.4)	0.0 (0.0 to 0.0)
Israel	1996	2010.7 (1954.6 to 2066.4)	1363.4 (1322.7 to 1400.3)	593.5 (556.2 to 633.3)	53.9 (41.8 to 68.2)	0.0 (0.0 to 0.0)	1893.8 (1840.9 to 1946.3)	1284.1 (1245.8 to 1318.9)	559.0 (523.8 to 596.5)	50.7 (39.4 to 64.3)	0.0 (0.0 to 0.0)
Israel	1997	2029.5 (1978.5 to 2082.2)	1367.1 (1330.0 to 1404.5)	598.2 (564.1 to 632.0)	64.2 (51.7 to 78.0)	0.0 (0.0 to 0.0)	1911.5 (1863.4 to 1961.1)	1287.6 (1252.7 to 1322.8)	563.5 (531.3 to 595.2)	60.5 (48.7 to 73.5)	0.0 (0.0 to 0.0)
Israel	1998	2011.7 (1959.7 to 2061.9)	1335.9 (1301.1 to 1373.9)	601.3 (567.6 to 633.2)	74.6 (62.2 to 88.6)	0.0 (0.0 to 0.0)	1894.7 (1845.8 to 1942.0)	1258.2 (1225.4 to 1294.0)	566.3 (534.6 to 596.4)	70.2 (58.6 to 83.5)	0.0 (0.0 to 0.0)
Israel	1999	2003.9 (1958.3 to 2052.7)	1311.6 (1278.7 to 1346.9)	605.5 (572.8 to 638.1)	86.8 (74.0 to 100.3)	0.0 (0.0 to 0.0)	1887.4 (1844.4 to 1933.3)	1235.4 (1204.3 to 1268.6)	570.3 (539.5 to 601.0)	81.7 (69.7 to 94.4)	0.0 (0.0 to 0.0)
Israel	2000	2098.6 (2054.6 to 2143.1)	1364.6 (1332.4 to 1396.0)	630.9 (600.5 to 661.5)	103.1 (89.8 to 117.3)	0.0 (0.0 to 0.0)	1976.6 (1935.1 to 2018.4)	1285.3 (1254.9 to 1314.8)	594.2 (565.5 to 623.0)	97.1 (84.6 to 110.5)	0.0 (0.0 to 0.0)
Israel	2001	2113.7 (2070.7 to 2158.7)	1363.7 (1332.9 to 1394.2)	636.5 (608.1 to 665.2)	113.6 (100.4 to 127.8)	0.0 (0.0 to 0.0)	1990.8 (1950.3 to 2033.2)	1284.4 (1255.4 to 1313.1)	599.5 (572.7 to 626.5)	107.0 (94.6 to 120.4)	0.0 (0.0 to 0.0)
Israel	2002	2098.1 (2055.8 to 2140.4)	1351.9 (1322.5 to 1382.5)	619.7 (592.3 to 646.2)	126.5 (112.9 to 140.6)	0.0 (0.0 to 0.0)	1976.1 (1936.3 to 2015.9)	1273.3 (1245.6 to 1302.1)	583.7 (557.9 to 608.6)	119.1 (106.4 to 132.5)	0.0 (0.0 to 0.0)
Israel	2003	2066.7 (2026.5 to 2109.1)	1315.7 (1283.7 to 1348.8)	611.0 (582.5 to 639.3)	140.0 (125.0 to 154.7)	0.0 (0.0 to 0.0)	1946.5 (1908.6 to 1986.5)	1239.2 (1209.0 to 1270.3)	575.4 (548.6 to 602.1)	131.9 (117.8 to 145.7)	0.0 (0.0 to 0.0)
Israel	2004	2098.5 (2055.9 to 2140.4)	1321.7 (1290.7 to 1353.5)	618.0 (589.5 to 645.7)	158.8 (143.2 to 174.4)	0.0 (0.0 to 0.0)	1976.5 (1936.3 to 2015.9)	1244.9 (1215.6 to 1274.8)	582.1 (555.2 to 608.1)	149.5 (134.8 to 164.3)	0.0 (0.0 to 0.0)
Israel	2005	2136.6 (2091.6 to 2184.0)	1329.5 (1298.5 to 1361.2)	622.9 (595.1 to 652.4)	184.2 (167.7 to 202.3)	0.0 (0.0 to 0.0)	2012.4 (1970.0 to 2057.0)	1252.2 (1223.0 to 1282.0)	586.7 (560.5 to 614.4)	173.5 (157.9 to 190.6)	0.0 (0.0 to 0.0)
Israel	2006	2205.6 (2159.8 to 2250.9)	1373.2 (1343.9 to 1403.9)	607.5 (581.6 to 638.0)	224.9 (207.2 to 244.4)	0.0 (0.0 to 0.0)	2077.3 (2034.2 to 2120.0)	1293.3 (1265.7 to 1322.3)	572.2 (547.7 to 600.9)	211.8 (195.1 to 230.2)	0.0 (0.0 to 0.0)
Israel	2007	2286.1 (2241.6 to 2334.0)	1417.2 (1383.6 to 1449.3)	609.0 (582.7 to 637.6)	259.9 (240.7 to 279.3)	0.0 (0.0 to 0.0)	2153.2 (2111.3 to 2198.3)	1334.8 (1303.2 to 1365.0)	573.6 (548.8 to 600.5)	244.8 (226.7 to 263.1)	0.0 (0.0 to 0.0)
Israel	2008	2325.1 (2275.7 to 2373.8)	1444.3 (1411.2 to 1480.0)	600.4 (573.5 to 628.5)	280.4 (261.2 to 303.8)	0.0 (0.0 to 0.0)	2189.9 (2143.4 to 2235.8)	1360.4 (1329.1 to 1393.9)	565.5 (540.2 to 592.0)	264.1 (246.0 to 286.2)	0.0 (0.0 to 0.0)

Country	Year	2018 US Dollars per capita					2018 PPP per capita				
		Health spending	Government health spending	Out-of-pocket health spending	Prepaid private health spending	Development assistance for health	Health spending	Government health spending	Out-of-pocket health spending	Prepaid private health spending	Development assistance for health
Israel	2009	2342.8 (2292.6 to 2394.2)	1463.7 (1430.0 to 1496.6)	591.3 (564.6 to 620.6)	287.8 (267.7 to 309.7)	0.0 (0.0 to 0.0)	2206.6 (2159.3 to 2255.0)	1378.6 (1346.8 to 1409.6)	556.9 (531.8 to 584.5)	271.1 (252.1 to 291.7)	0.0 (0.0 to 0.0)
Israel	2010	2417.3 (2370.2 to 2464.3)	1528.9 (1495.0 to 1562.6)	588.8 (560.1 to 617.0)	299.6 (278.5 to 323.0)	0.0 (0.0 to 0.0)	2276.7 (2232.4 to 2321.0)	1440.0 (1408.1 to 1471.7)	554.5 (527.6 to 581.1)	282.2 (262.4 to 304.3)	0.0 (0.0 to 0.0)
Israel	2011	2475.0 (2426.4 to 2525.8)	1575.6 (1541.9 to 1609.8)	590.4 (563.8 to 617.5)	309.0 (287.4 to 332.2)	0.0 (0.0 to 0.0)	2331.1 (2285.3 to 2378.9)	1484.0 (1452.2 to 1516.1)	556.1 (531.0 to 581.6)	291.0 (270.7 to 312.9)	0.0 (0.0 to 0.0)
Israel	2012	2518.5 (2470.2 to 2568.7)	1609.0 (1575.6 to 1643.0)	591.3 (565.2 to 619.5)	318.2 (296.3 to 341.2)	0.0 (0.0 to 0.0)	2372.0 (2326.6 to 2419.4)	1515.4 (1483.9 to 1547.5)	557.0 (532.3 to 583.4)	299.7 (279.1 to 321.3)	0.0 (0.0 to 0.0)
Israel	2013	2585.3 (2536.9 to 2635.6)	1659.3 (1625.2 to 1695.0)	596.4 (568.0 to 623.7)	329.6 (306.9 to 354.4)	0.0 (0.0 to 0.0)	2435.0 (2389.4 to 2482.3)	1562.8 (1530.7 to 1596.4)	561.7 (534.9 to 587.4)	310.4 (289.0 to 333.8)	0.0 (0.0 to 0.0)
Israel	2014	2648.9 (2596.4 to 2701.3)	1696.8 (1661.6 to 1732.8)	609.4 (579.0 to 636.7)	342.8 (319.4 to 367.9)	0.0 (0.0 to 0.0)	2494.9 (2445.4 to 2544.2)	1598.1 (1565.0 to 1632.0)	573.9 (545.3 to 599.7)	322.8 (300.9 to 346.5)	0.0 (0.0 to 0.0)
Israel	2015	2700.0 (2647.9 to 2750.7)	1721.5 (1686.4 to 1754.7)	623.3 (593.1 to 651.9)	355.2 (330.7 to 379.2)	0.0 (0.0 to 0.0)	2542.9 (2493.9 to 2590.7)	1621.4 (1588.3 to 1652.7)	587.1 (558.6 to 614.0)	334.5 (311.5 to 357.1)	0.0 (0.0 to 0.0)
Israel	2016	2756.8 (2683.7 to 2827.5)	1752.1 (1706.6 to 1799.0)	639.7 (598.7 to 679.1)	365.1 (330.5 to 400.7)	0.0 (0.0 to 0.0)	2596.5 (2527.6 to 2663.1)	1650.2 (1607.3 to 1694.4)	602.5 (563.9 to 639.6)	343.8 (311.3 to 377.4)	0.0 (0.0 to 0.0)
Israel	2017	2858.6 (2785.6 to 2934.3)	1837.1 (1789.2 to 1886.0)	648.5 (603.8 to 691.2)	372.9 (337.2 to 409.0)	0.0 (0.0 to 0.0)	2692.3 (2623.6 to 2763.7)	1730.3 (1685.2 to 1776.3)	610.8 (568.7 to 651.0)	351.2 (317.6 to 385.2)	0.0 (0.0 to 0.0)
Israel	2018	2887.8 (2809.4 to 2965.3)	1855.9 (1806.8 to 1906.0)	654.6 (609.5 to 699.1)	377.4 (341.8 to 413.7)	0.0 (0.0 to 0.0)	2719.9 (2646.0 to 2792.9)	1748.0 (1701.7 to 1795.1)	616.5 (574.1 to 658.4)	355.4 (321.9 to 389.6)	0.0 (0.0 to 0.0)
Israel	2019	2922.4 (2841.7 to 3004.2)	1878.3 (1827.2 to 1930.9)	660.8 (614.6 to 706.0)	383.3 (347.4 to 420.2)	0.0 (0.0 to 0.0)	2752.5 (2676.5 to 2829.5)	1769.1 (1720.9 to 1818.6)	622.4 (578.8 to 665.0)	361.0 (327.2 to 395.8)	0.0 (0.0 to 0.0)
Israel	2020	2955.4 (2872.9 to 3036.6)	1899.6 (1844.3 to 1956.8)	666.8 (619.0 to 715.3)	389.0 (352.6 to 427.1)	0.0 (0.0 to 0.0)	2783.5 (2705.8 to 2860.0)	1789.1 (1737.1 to 1843.0)	628.1 (583.0 to 673.7)	366.3 (332.1 to 402.3)	0.0 (0.0 to 0.0)
Israel	2021	2992.5 (2907.5 to 3079.6)	1924.4 (1867.9 to 1982.0)	672.9 (624.5 to 721.9)	395.2 (358.1 to 433.9)	0.0 (0.0 to 0.0)	2818.4 (2738.4 to 2900.5)	1812.5 (1759.3 to 1866.7)	633.8 (588.1 to 679.9)	372.2 (337.3 to 408.7)	0.0 (0.0 to 0.0)
Israel	2022	3024.9 (2936.1 to 3112.0)	1945.7 (1886.8 to 2005.2)	678.4 (628.2 to 730.3)	400.7 (362.4 to 440.5)	0.0 (0.0 to 0.0)	2849.0 (2765.3 to 2931.1)	1832.6 (1777.1 to 1888.5)	639.0 (591.6 to 687.8)	377.4 (341.3 to 414.8)	0.0 (0.0 to 0.0)
Israel	2023	3054.6 (2959.7 to 3144.0)	1964.9 (1904.1 to 2025.1)	683.6 (630.9 to 737.9)	406.0 (367.6 to 446.6)	0.0 (0.0 to 0.0)	2877.0 (2787.6 to 2961.2)	1850.7 (1793.3 to 1907.4)	643.9 (594.2 to 694.9)	382.4 (346.2 to 420.6)	0.0 (0.0 to 0.0)
Israel	2024	3082.9 (2990.8 to 3179.5)	1982.8 (1920.4 to 2044.5)	688.9 (634.6 to 746.9)	411.3 (371.7 to 453.1)	0.0 (0.0 to 0.0)	2903.6 (2816.8 to 2994.6)	1867.5 (1808.8 to 1925.6)	648.8 (597.7 to 703.5)	387.3 (350.1 to 426.7)	0.0 (0.0 to 0.0)
Israel	2025	3115.3 (3014.6 to 3209.9)	2003.6 (1938.9 to 2067.9)	694.5 (636.2 to 751.2)	417.1 (376.7 to 459.4)	0.0 (0.0 to 0.0)	2934.1 (2839.3 to 3023.2)	1887.1 (1826.1 to 1947.6)	654.2 (599.2 to 707.5)	392.8 (354.8 to 432.7)	0.0 (0.0 to 0.0)
Israel	2026	3146.6 (3049.0 to 3242.6)	2023.4 (1957.2 to 2092.1)	700.3 (637.5 to 761.4)	422.9 (381.2 to 466.3)	0.0 (0.0 to 0.0)	2963.6 (2871.7 to 3054.0)	1905.7 (1843.4 to 1970.4)	659.6 (600.4 to 717.1)	398.3 (359.1 to 439.2)	0.0 (0.0 to 0.0)
Israel	2027	3181.8 (3072.7 to 3287.7)	2045.8 (1974.6 to 2119.3)	706.6 (637.9 to 773.3)	429.4 (386.6 to 474.0)	0.0 (0.0 to 0.0)	2996.8 (2894.0 to 3096.5)	1926.8 (1859.8 to 1996.1)	665.5 (600.8 to 728.3)	404.4 (364.2 to 446.4)	0.0 (0.0 to 0.0)
Israel	2028	3221.0 (3106.7 to 3332.5)	2071.0 (1995.6 to 2149.3)	713.6 (641.7 to 787.2)	436.5 (392.9 to 482.3)	0.0 (0.0 to 0.0)	3033.7 (2926.0 to 3138.7)	1950.6 (1879.6 to 2024.3)	672.1 (604.4 to 741.4)	411.1 (370.1 to 454.3)	0.0 (0.0 to 0.0)
Israel	2029	3264.0 (3133.6 to 3387.9)	2098.9 (2017.2 to 2184.4)	721.1 (641.2 to 805.6)	444.0 (399.3 to 490.3)	0.0 (0.0 to 0.0)	3074.2 (2951.4 to 3190.8)	1976.8 (1899.9 to 2057.4)	679.1 (603.9 to 758.7)	418.2 (376.0 to 461.8)	0.0 (0.0 to 0.0)

Country	Year	2018 US Dollars per capita					2018 PPP per capita				
		Health spending	Government health spending	Out-of-pocket health spending	Prepaid private health spending	Development assistance for health	Health spending	Government health spending	Out-of-pocket health spending	Prepaid private health spending	Development assistance for health
Israel	2030	3308.4 (3168.7 to 3442.2)	2127.6 (2039.6 to 2219.2)	729.0 (643.0 to 823.5)	451.8 (406.3 to 499.4)	0.0 (0.0 to 0.0)	3116.0 (2984.4 to 3242.0)	2003.9 (1921.0 to 2090.1)	686.6 (605.6 to 775.7)	425.5 (382.7 to 470.4)	0.0 (0.0 to 0.0)
Israel	2031	3353.6 (3209.6 to 3500.5)	2156.3 (2063.0 to 2257.6)	737.3 (643.1 to 842.9)	459.9 (413.7 to 509.2)	0.0 (0.0 to 0.0)	3158.5 (3022.9 to 3297.0)	2030.9 (1943.0 to 2126.3)	694.4 (605.7 to 793.8)	433.2 (389.6 to 479.6)	0.0 (0.0 to 0.0)
Israel	2032	3401.6 (3245.3 to 3554.6)	2186.8 (2086.9 to 2295.7)	746.2 (646.9 to 860.2)	468.5 (421.4 to 519.9)	0.0 (0.0 to 0.0)	3203.8 (3056.5 to 3347.9)	2059.6 (1965.5 to 2162.2)	702.8 (609.3 to 810.1)	441.3 (396.9 to 489.7)	0.0 (0.0 to 0.0)
Israel	2033	3449.8 (3281.8 to 3619.9)	2217.2 (2110.5 to 2333.2)	755.4 (648.5 to 876.2)	477.2 (429.2 to 529.4)	0.0 (0.0 to 0.0)	3249.2 (3091.0 to 3409.3)	2088.2 (1987.8 to 2197.5)	711.5 (610.8 to 825.2)	449.5 (404.3 to 498.6)	0.0 (0.0 to 0.0)
Israel	2034	3500.0 (3319.4 to 3693.3)	2249.2 (2133.3 to 2373.8)	764.8 (649.6 to 895.1)	486.0 (436.4 to 539.7)	0.0 (0.0 to 0.0)	3296.5 (3126.3 to 3478.5)	2118.4 (2009.2 to 2235.8)	720.4 (611.9 to 843.1)	457.7 (411.0 to 508.4)	0.0 (0.0 to 0.0)
Israel	2035	3551.8 (3358.2 to 3740.6)	2282.6 (2160.5 to 2416.0)	774.4 (653.8 to 913.0)	494.8 (442.8 to 550.1)	0.0 (0.0 to 0.0)	3345.2 (3162.9 to 3523.1)	2149.8 (2034.9 to 2275.5)	729.4 (615.8 to 859.9)	466.0 (417.0 to 518.1)	0.0 (0.0 to 0.0)
Israel	2036	3600.4 (3395.6 to 3809.5)	2313.0 (2180.9 to 2453.3)	784.1 (656.2 to 930.0)	503.3 (448.9 to 561.3)	0.0 (0.0 to 0.0)	3391.0 (3198.1 to 3588.0)	2178.5 (2054.1 to 2310.6)	738.5 (618.0 to 875.9)	474.0 (422.8 to 528.6)	0.0 (0.0 to 0.0)
Israel	2037	3650.4 (3440.7 to 3869.9)	2344.6 (2202.6 to 2496.0)	793.9 (660.5 to 947.3)	511.9 (454.9 to 571.9)	0.0 (0.0 to 0.0)	3438.1 (3240.6 to 3644.8)	2208.2 (2074.5 to 2350.9)	747.7 (622.1 to 892.2)	482.1 (428.5 to 538.6)	0.0 (0.0 to 0.0)
Israel	2038	3700.5 (3475.4 to 3923.6)	2376.2 (2220.5 to 2536.2)	804.0 (665.4 to 965.0)	520.4 (461.4 to 581.1)	0.0 (0.0 to 0.0)	3485.3 (3273.3 to 3695.5)	2238.0 (2091.4 to 2388.7)	757.2 (626.7 to 908.9)	490.1 (434.6 to 547.3)	0.0 (0.0 to 0.0)
Israel	2039	3750.5 (3507.7 to 3994.4)	2407.7 (2239.6 to 2580.6)	814.1 (670.9 to 981.2)	528.7 (467.8 to 592.2)	0.0 (0.0 to 0.0)	3532.4 (3303.8 to 3762.1)	2267.7 (2109.4 to 2430.6)	766.8 (631.9 to 924.2)	498.0 (440.6 to 557.7)	0.0 (0.0 to 0.0)
Israel	2040	3802.4 (3561.9 to 4061.5)	2440.5 (2264.3 to 2623.7)	824.6 (676.6 to 997.1)	537.3 (473.2 to 602.8)	0.0 (0.0 to 0.0)	3581.3 (3354.8 to 3825.4)	2298.6 (2132.7 to 2471.2)	776.6 (637.2 to 939.1)	506.0 (445.7 to 567.7)	0.0 (0.0 to 0.0)
Israel	2041	3852.9 (3599.4 to 4142.8)	2471.7 (2285.2 to 2667.5)	835.3 (681.2 to 1012.6)	545.8 (479.7 to 613.0)	0.0 (0.0 to 0.0)	3628.8 (3390.1 to 3901.9)	2328.0 (2152.3 to 2512.4)	786.7 (641.6 to 953.7)	514.1 (451.8 to 577.3)	0.0 (0.0 to 0.0)
Israel	2042	3902.5 (3640.0 to 4182.9)	2501.9 (2305.1 to 2708.2)	846.3 (686.5 to 1027.6)	554.3 (486.0 to 625.4)	0.0 (0.0 to 0.0)	3675.6 (3428.4 to 3939.6)	2356.4 (2171.1 to 2550.7)	797.1 (646.6 to 967.9)	522.1 (457.8 to 589.0)	0.0 (0.0 to 0.0)
Israel	2043	3953.5 (3693.3 to 4240.5)	2532.9 (2329.4 to 2749.0)	857.6 (693.7 to 1044.2)	563.0 (492.7 to 637.4)	0.0 (0.0 to 0.0)	3723.6 (3478.5 to 3993.9)	2385.6 (2193.9 to 2589.1)	807.7 (653.4 to 983.5)	530.3 (464.0 to 600.3)	0.0 (0.0 to 0.0)
Israel	2044	4006.6 (3725.7 to 4318.5)	2565.4 (2354.2 to 2794.5)	869.3 (702.3 to 1061.5)	571.9 (499.0 to 648.4)	0.0 (0.0 to 0.0)	3773.6 (3509.1 to 4067.4)	2416.2 (2217.3 to 2632.0)	818.7 (661.5 to 999.8)	538.7 (470.0 to 610.7)	0.0 (0.0 to 0.0)
Israel	2045	4057.3 (3769.6 to 4363.0)	2595.3 (2374.0 to 2841.5)	881.3 (711.3 to 1078.7)	580.7 (505.3 to 660.9)	0.0 (0.0 to 0.0)	3821.4 (3550.4 to 4109.3)	2444.4 (2235.9 to 2676.3)	830.0 (669.9 to 1016.0)	546.9 (475.9 to 622.5)	0.0 (0.0 to 0.0)
Israel	2046	4105.2 (3809.8 to 4435.6)	2622.2 (2390.4 to 2878.5)	893.6 (720.0 to 1095.0)	589.4 (511.3 to 673.4)	0.0 (0.0 to 0.0)	3866.5 (3588.2 to 4177.6)	2469.7 (2251.4 to 2711.1)	841.6 (678.2 to 1031.4)	555.2 (481.6 to 634.3)	0.0 (0.0 to 0.0)
Israel	2047	4153.9 (3843.9 to 4501.3)	2649.1 (2410.5 to 2912.1)	906.4 (730.0 to 1113.1)	598.4 (517.4 to 686.3)	0.0 (0.0 to 0.0)	3912.4 (3620.4 to 4239.5)	2495.1 (2270.3 to 2742.8)	853.7 (687.5 to 1048.3)	563.6 (487.3 to 646.4)	0.0 (0.0 to 0.0)
Israel	2048	4201.7 (3886.0 to 4551.9)	2674.8 (2428.5 to 2947.4)	919.3 (740.7 to 1129.5)	607.5 (523.5 to 699.3)	0.0 (0.0 to 0.0)	3957.4 (3660.0 to 4287.2)	2519.3 (2287.3 to 2776.0)	865.9 (697.6 to 1063.9)	572.2 (493.0 to 658.6)	0.0 (0.0 to 0.0)
Israel	2049	4248.8 (3908.7 to 4614.9)	2699.8 (2439.2 to 2984.2)	932.4 (751.5 to 1146.1)	616.6 (529.6 to 710.7)	0.0 (0.0 to 0.0)	4001.7 (3681.4 to 4346.5)	2542.8 (2297.3 to 2810.7)	878.2 (707.8 to 1079.4)	580.7 (498.8 to 669.4)	0.0 (0.0 to 0.0)
Israel	2050	4295.5 (3948.1 to 4670.6)	2724.3 (2454.8 to 3025.7)	945.6 (762.4 to 1162.8)	625.6 (536.4 to 723.1)	0.0 (0.0 to 0.0)	4045.7 (3718.5 to 4399.0)	2565.9 (2312.0 to 2849.7)	890.6 (718.0 to 1095.2)	589.2 (505.2 to 681.0)	0.0 (0.0 to 0.0)

		2018 US Dollars per capita					2018 PPP per capita				
Country	Year	Health spending	Government health spending	Out-of-pocket health spending	Prepaid private health spending	Development assistance for health	Health spending	Government health spending	Out-of-pocket health spending	Prepaid private health spending	Development assistance for health
Italy	1995	2282.6 (2205.4 to 2375.7)	1635.1 (1568.3 to 1708.1)	621.9 (579.0 to 667.7)	25.6 (15.9 to 40.1)	0.0 (0.0 to 0.0)	2583.3 (2495.9 to 2688.7)	1850.5 (1774.9 to 1933.1)	703.8 (655.3 to 755.7)	29.0 (18.0 to 45.4)	0.0 (0.0 to 0.0)
Italy	1996	2344.6 (2282.3 to 2411.6)	1675.0 (1623.3 to 1724.8)	643.2 (611.4 to 677.6)	26.4 (16.8 to 40.3)	0.0 (0.0 to 0.0)	2653.5 (2582.9 to 2729.3)	1895.7 (1837.2 to 1952.0)	727.9 (691.9 to 766.9)	29.9 (19.0 to 45.6)	0.0 (0.0 to 0.0)
Italy	1997	2437.4 (2378.7 to 2498.1)	1736.4 (1685.7 to 1784.3)	674.1 (641.3 to 707.7)	26.9 (17.8 to 39.8)	0.0 (0.0 to 0.0)	2758.5 (2692.1 to 2827.2)	1965.1 (1907.8 to 2019.4)	762.9 (725.7 to 801.0)	30.4 (20.1 to 45.0)	0.0 (0.0 to 0.0)
Italy	1998	2524.3 (2466.7 to 2590.1)	1797.1 (1747.3 to 1846.7)	700.7 (669.9 to 732.7)	26.6 (17.9 to 38.1)	0.0 (0.0 to 0.0)	2856.8 (2791.6 to 2931.3)	2033.8 (1977.5 to 2090.0)	793.0 (758.2 to 829.2)	30.1 (20.3 to 43.1)	0.0 (0.0 to 0.0)
Italy	1999	2605.1 (2549.3 to 2671.2)	1865.8 (1816.9 to 1916.2)	712.9 (683.4 to 743.0)	26.4 (18.4 to 36.9)	0.0 (0.0 to 0.0)	2948.3 (2885.2 to 3023.2)	2111.6 (2056.3 to 2168.6)	806.8 (773.5 to 840.9)	29.9 (20.8 to 41.7)	0.0 (0.0 to 0.0)
Italy	2000	2742.3 (2688.0 to 2794.7)	1995.8 (1949.7 to 2044.9)	720.2 (691.9 to 748.0)	26.3 (18.9 to 35.7)	0.0 (0.0 to 0.0)	3103.6 (3042.1 to 3162.9)	2258.7 (2206.6 to 2314.3)	815.1 (783.0 to 846.6)	29.8 (21.4 to 40.4)	0.0 (0.0 to 0.0)
Italy	2001	2852.5 (2800.0 to 2906.4)	2113.4 (2065.5 to 2160.4)	712.3 (685.5 to 739.3)	26.8 (19.3 to 36.5)	0.0 (0.0 to 0.0)	3228.3 (3168.9 to 3289.3)	2391.9 (2337.6 to 2445.1)	806.1 (775.8 to 836.7)	30.3 (21.9 to 41.3)	0.0 (0.0 to 0.0)
Italy	2002	2887.3 (2830.6 to 2943.9)	2162.3 (2114.3 to 2209.2)	698.0 (672.3 to 723.7)	27.0 (19.3 to 36.5)	0.0 (0.0 to 0.0)	3267.6 (3203.5 to 3331.7)	2447.2 (2392.9 to 2500.3)	790.0 (760.8 to 819.1)	30.5 (21.8 to 41.3)	0.0 (0.0 to 0.0)
Italy	2003	2919.2 (2863.2 to 2979.2)	2207.1 (2158.0 to 2257.6)	684.8 (660.5 to 711.6)	27.3 (19.7 to 37.1)	0.0 (0.0 to 0.0)	3303.7 (3240.4 to 3371.7)	2497.8 (2442.3 to 2555.0)	775.0 (747.5 to 805.4)	30.9 (22.2 to 41.9)	0.0 (0.0 to 0.0)
Italy	2004	3007.4 (2946.5 to 3059.8)	2298.3 (2247.4 to 2345.3)	681.0 (656.1 to 705.4)	28.0 (20.5 to 38.0)	0.0 (0.0 to 0.0)	3403.6 (3334.6 to 3463.0)	2601.1 (2543.5 to 2654.3)	770.8 (742.6 to 798.3)	31.7 (23.2 to 43.0)	0.0 (0.0 to 0.0)
Italy	2005	3065.3 (3010.7 to 3123.5)	2364.7 (2316.0 to 2415.2)	671.9 (645.9 to 697.5)	28.7 (21.1 to 38.4)	0.0 (0.0 to 0.0)	3469.1 (3407.3 to 3535.0)	2676.2 (2621.1 to 2733.4)	760.4 (731.0 to 789.4)	32.5 (23.8 to 43.5)	0.0 (0.0 to 0.0)
Italy	2006	3124.3 (3070.2 to 3180.8)	2423.9 (2372.5 to 2476.4)	670.5 (646.1 to 696.2)	29.9 (21.6 to 40.0)	0.0 (0.0 to 0.0)	3535.9 (3474.7 to 3599.8)	2743.2 (2685.0 to 2802.6)	758.8 (731.2 to 787.9)	33.8 (24.4 to 45.3)	0.0 (0.0 to 0.0)
Italy	2007	3145.9 (3090.5 to 3203.2)	2447.5 (2396.2 to 2496.1)	667.4 (643.9 to 694.5)	31.1 (22.7 to 41.4)	0.0 (0.0 to 0.0)	3560.4 (3497.7 to 3625.2)	2770.0 (2711.8 to 2825.0)	755.3 (728.8 to 786.0)	35.1 (25.7 to 46.9)	0.0 (0.0 to 0.0)
Italy	2008	3168.5 (3107.5 to 3228.3)	2469.9 (2419.3 to 2520.3)	666.2 (641.8 to 690.9)	32.4 (23.7 to 43.7)	0.0 (0.0 to 0.0)	3585.9 (3516.9 to 3653.6)	2795.2 (2738.0 to 2852.3)	754.0 (726.4 to 781.9)	36.7 (26.9 to 49.4)	0.0 (0.0 to 0.0)
Italy	2009	3102.4 (3044.2 to 3161.1)	2418.3 (2368.6 to 2466.2)	650.6 (626.1 to 675.1)	33.5 (24.7 to 44.4)	0.0 (0.0 to 0.0)	3511.2 (3445.2 to 3577.5)	2736.8 (2680.7 to 2791.1)	736.4 (708.6 to 764.0)	38.0 (28.0 to 50.3)	0.0 (0.0 to 0.0)
Italy	2010	3135.0 (3079.7 to 3191.0)	2441.8 (2391.9 to 2491.1)	656.8 (633.7 to 680.2)	36.4 (26.7 to 48.0)	0.0 (0.0 to 0.0)	3548.0 (3485.5 to 3611.4)	2763.5 (2707.0 to 2819.3)	743.3 (717.2 to 769.8)	41.2 (30.2 to 54.3)	0.0 (0.0 to 0.0)
Italy	2011	3123.6 (3069.8 to 3181.0)	2414.2 (2365.0 to 2466.4)	667.8 (644.5 to 693.0)	41.6 (30.8 to 54.0)	0.0 (0.0 to 0.0)	3535.1 (3474.2 to 3600.1)	2732.2 (2676.5 to 2791.3)	755.8 (729.4 to 784.3)	47.1 (34.9 to 61.1)	0.0 (0.0 to 0.0)
Italy	2012	3058.5 (3001.6 to 3119.1)	2338.9 (2288.8 to 2389.4)	665.5 (641.7 to 690.0)	54.1 (41.1 to 69.4)	0.0 (0.0 to 0.0)	3461.5 (3397.0 to 3530.1)	2647.0 (2590.3 to 2704.2)	753.1 (726.2 to 780.9)	61.3 (46.6 to 78.5)	0.0 (0.0 to 0.0)
Italy	2013	3011.3 (2952.3 to 3066.4)	2286.4 (2238.2 to 2333.4)	663.1 (637.4 to 688.7)	61.9 (47.3 to 79.1)	0.0 (0.0 to 0.0)	3408.0 (3341.2 to 3470.3)	2587.6 (2533.0 to 2640.9)	750.4 (721.4 to 779.4)	70.0 (53.5 to 89.5)	0.0 (0.0 to 0.0)
Italy	2014	3011.2 (2954.3 to 3066.8)	2270.1 (2222.2 to 2319.0)	673.8 (647.6 to 699.3)	67.2 (52.5 to 86.7)	0.0 (0.0 to 0.0)	3407.9 (3343.5 to 3470.9)	2569.2 (2514.9 to 2624.5)	762.6 (732.9 to 791.4)	76.1 (59.4 to 98.1)	0.0 (0.0 to 0.0)
Italy	2015	3042.3 (2978.8 to 3103.6)	2275.9 (2224.4 to 2326.2)	695.0 (669.0 to 721.8)	71.5 (53.8 to 93.0)	0.0 (0.0 to 0.0)	3443.1 (3371.3 to 3512.5)	2575.8 (2517.5 to 2632.7)	786.5 (757.1 to 816.9)	80.9 (60.9 to 105.3)	0.0 (0.0 to 0.0)

Country	Year	2018 US Dollars per capita					2018 PPP per capita				
		Health spending	Government health spending	Out-of-pocket health spending	Prepaid private health spending	Development assistance for health	Health spending	Government health spending	Out-of-pocket health spending	Prepaid private health spending	Development assistance for health
Italy	2016	3058.9 (2976.0 to 3140.8)	2276.9 (2208.3 to 2340.4)	707.3 (670.1 to 741.5)	74.6 (53.5 to 100.3)	0.0 (0.0 to 0.0)	3461.9 (3368.0 to 3554.6)	2576.9 (2499.3 to 2648.8)	800.5 (758.4 to 839.2)	84.4 (60.5 to 113.5)	0.0 (0.0 to 0.0)
Italy	2017	3089.1 (3004.9 to 3171.5)	2298.6 (2225.9 to 2366.6)	714.8 (677.7 to 750.2)	75.7 (54.1 to 101.9)	0.0 (0.0 to 0.0)	3496.1 (3400.8 to 3589.3)	2601.5 (2519.1 to 2678.4)	809.0 (767.0 to 849.1)	85.6 (61.2 to 115.3)	0.0 (0.0 to 0.0)
Italy	2018	3109.1 (3024.8 to 3198.1)	2315.6 (2242.8 to 2384.0)	717.1 (677.8 to 754.1)	76.5 (54.7 to 103.2)	0.0 (0.0 to 0.0)	3518.7 (3423.3 to 3619.4)	2620.6 (2538.2 to 2698.1)	811.5 (767.1 to 853.4)	86.5 (62.0 to 116.8)	0.0 (0.0 to 0.0)
Italy	2019	3128.9 (3045.7 to 3212.7)	2332.5 (2258.8 to 2402.6)	719.4 (677.9 to 758.4)	77.1 (55.4 to 103.9)	0.0 (0.0 to 0.0)	3541.1 (3447.0 to 3636.0)	2639.7 (2556.4 to 2719.1)	814.1 (767.2 to 858.3)	87.3 (62.7 to 117.6)	0.0 (0.0 to 0.0)
Italy	2020	3156.3 (3064.6 to 3244.8)	2355.4 (2281.1 to 2424.3)	722.9 (678.6 to 763.5)	77.9 (56.0 to 105.1)	0.0 (0.0 to 0.0)	3572.1 (3468.3 to 3672.3)	2665.7 (2581.6 to 2743.7)	818.2 (768.0 to 864.1)	88.2 (63.4 to 119.0)	0.0 (0.0 to 0.0)
Italy	2021	3180.6 (3088.8 to 3267.4)	2375.1 (2299.0 to 2448.4)	726.8 (682.9 to 770.0)	78.7 (56.6 to 105.7)	0.0 (0.0 to 0.0)	3599.6 (3495.7 to 3697.9)	2688.0 (2601.9 to 2771.0)	822.6 (772.8 to 871.5)	89.1 (64.0 to 119.7)	0.0 (0.0 to 0.0)
Italy	2022	3205.7 (3109.9 to 3293.9)	2395.5 (2315.3 to 2470.9)	730.7 (686.2 to 777.7)	79.5 (57.2 to 106.8)	0.0 (0.0 to 0.0)	3628.1 (3519.6 to 3727.8)	2711.1 (2620.4 to 2796.4)	827.0 (776.6 to 880.2)	90.0 (64.7 to 120.9)	0.0 (0.0 to 0.0)
Italy	2023	3222.1 (3116.6 to 3316.0)	2409.3 (2326.2 to 2488.2)	732.7 (689.1 to 781.5)	80.1 (57.5 to 107.8)	0.0 (0.0 to 0.0)	3646.6 (3527.1 to 3752.9)	2726.8 (2632.7 to 2816.0)	829.2 (779.9 to 884.4)	90.7 (65.1 to 122.0)	0.0 (0.0 to 0.0)
Italy	2024	3237.6 (3134.7 to 3335.3)	2422.6 (2336.8 to 2503.2)	734.4 (688.8 to 781.9)	80.7 (57.8 to 108.8)	0.0 (0.0 to 0.0)	3664.2 (3547.6 to 3774.7)	2741.8 (2644.6 to 2833.0)	831.1 (779.6 to 884.9)	91.3 (65.4 to 123.1)	0.0 (0.0 to 0.0)
Italy	2025	3252.7 (3147.6 to 3354.9)	2435.5 (2343.2 to 2523.2)	736.0 (688.6 to 785.1)	81.2 (58.4 to 109.6)	0.0 (0.0 to 0.0)	3681.2 (3562.3 to 3796.9)	2756.3 (2651.9 to 2855.6)	832.9 (779.3 to 888.6)	91.9 (66.1 to 124.1)	0.0 (0.0 to 0.0)
Italy	2026	3265.2 (3150.9 to 3375.2)	2446.3 (2345.2 to 2539.9)	737.3 (685.1 to 790.3)	81.7 (58.7 to 110.3)	0.0 (0.0 to 0.0)	3695.4 (3566.0 to 3819.8)	2768.5 (2654.2 to 2874.6)	834.5 (775.4 to 894.4)	92.4 (66.5 to 124.8)	0.0 (0.0 to 0.0)
Italy	2027	3278.1 (3152.0 to 3400.8)	2457.3 (2348.2 to 2564.2)	738.7 (680.9 to 799.4)	82.1 (59.0 to 110.7)	0.0 (0.0 to 0.0)	3710.0 (3567.3 to 3848.8)	2781.0 (2657.6 to 2902.0)	836.0 (770.6 to 904.8)	92.9 (66.8 to 125.3)	0.0 (0.0 to 0.0)
Italy	2028	3289.6 (3155.1 to 3426.6)	2466.8 (2349.3 to 2584.7)	740.2 (674.4 to 807.6)	82.5 (59.3 to 111.1)	0.0 (0.0 to 0.0)	3722.9 (3570.8 to 3878.0)	2791.8 (2658.8 to 2925.2)	837.7 (763.3 to 914.0)	93.4 (67.1 to 125.7)	0.0 (0.0 to 0.0)
Italy	2029	3300.6 (3161.0 to 3444.6)	2475.8 (2349.4 to 2606.0)	741.9 (669.7 to 816.8)	82.9 (59.6 to 111.4)	0.0 (0.0 to 0.0)	3735.4 (3577.5 to 3898.5)	2801.9 (2659.0 to 2949.3)	839.6 (757.9 to 924.4)	93.8 (67.4 to 126.1)	0.0 (0.0 to 0.0)
Italy	2030	3311.0 (3145.4 to 3478.3)	2484.0 (2348.7 to 2628.9)	743.8 (665.1 to 830.6)	83.2 (59.8 to 112.0)	0.0 (0.0 to 0.0)	3747.2 (3559.8 to 3936.5)	2811.3 (2658.1 to 2975.3)	841.8 (752.7 to 940.0)	94.2 (67.6 to 126.7)	0.0 (0.0 to 0.0)
Italy	2031	3320.1 (3152.3 to 3502.1)	2490.7 (2345.4 to 2650.5)	745.8 (659.5 to 843.5)	83.6 (59.9 to 112.2)	0.0 (0.0 to 0.0)	3757.5 (3567.6 to 3963.5)	2818.9 (2654.4 to 2999.7)	844.0 (746.4 to 954.6)	94.6 (67.8 to 127.0)	0.0 (0.0 to 0.0)
Italy	2032	3327.5 (3151.5 to 3526.2)	2495.9 (2343.1 to 2669.8)	747.8 (653.7 to 853.2)	83.8 (60.1 to 112.6)	0.0 (0.0 to 0.0)	3765.9 (3566.7 to 3990.8)	2824.7 (2651.7 to 3021.6)	846.3 (739.8 to 965.6)	94.9 (68.0 to 127.4)	0.0 (0.0 to 0.0)
Italy	2033	3335.0 (3144.2 to 3541.9)	2500.8 (2339.9 to 2684.3)	750.1 (650.1 to 862.4)	84.1 (60.2 to 112.6)	0.0 (0.0 to 0.0)	3774.4 (3558.4 to 4008.5)	2830.3 (2648.1 to 3037.9)	849.0 (735.7 to 976.0)	95.2 (68.1 to 127.5)	0.0 (0.0 to 0.0)
Italy	2034	3342.6 (3140.7 to 3581.2)	2505.6 (2333.9 to 2704.6)	752.7 (645.2 to 875.1)	84.3 (60.3 to 112.6)	0.0 (0.0 to 0.0)	3783.0 (3554.5 to 4053.0)	2835.7 (2641.4 to 3061.0)	851.9 (730.2 to 990.4)	95.4 (68.3 to 127.5)	0.0 (0.0 to 0.0)
Italy	2035	3348.9 (3135.5 to 3598.9)	2509.0 (2327.7 to 2722.8)	755.4 (641.1 to 889.6)	84.5 (60.4 to 112.5)	0.0 (0.0 to 0.0)	3790.1 (3548.6 to 4073.1)	2839.5 (2634.3 to 3081.5)	854.9 (725.5 to 1006.8)	95.7 (68.3 to 127.4)	0.0 (0.0 to 0.0)
Italy	2036	3355.2 (3131.7 to 3605.9)	2512.1 (2323.3 to 2738.1)	758.3 (640.1 to 900.9)	84.8 (60.4 to 112.6)	0.0 (0.0 to 0.0)	3797.3 (3544.3 to 4080.9)	2843.1 (2629.4 to 3098.8)	858.2 (724.4 to 1019.6)	95.9 (68.3 to 127.5)	0.0 (0.0 to 0.0)

Country	Year	2018 US Dollars per capita					2018 PPP per capita				
		Health spending	Government health spending	Out-of-pocket health spending	Prepaid private health spending	Development assistance for health	Health spending	Government health spending	Out-of-pocket health spending	Prepaid private health spending	Development assistance for health
Italy	2037	3362.2 (3132.1 to 3623.6)	2515.6 (2319.1 to 2754.4)	761.6 (638.3 to 910.6)	85.0 (60.4 to 112.9)	0.0 (0.0 to 0.0)	3805.2 (3544.8 to 4101.0)	2847.0 (2624.6 to 3117.2)	861.9 (722.4 to 1030.6)	96.2 (68.4 to 127.8)	0.0 (0.0 to 0.0)
Italy	2038	3370.6 (3118.5 to 3651.9)	2520.0 (2313.1 to 2771.0)	765.3 (637.7 to 921.0)	85.3 (60.5 to 113.4)	0.0 (0.0 to 0.0)	3814.6 (3529.3 to 4133.0)	2852.0 (2617.8 to 3136.0)	866.1 (721.7 to 1042.4)	96.5 (68.5 to 128.4)	0.0 (0.0 to 0.0)
Italy	2039	3379.3 (3110.5 to 3661.8)	2524.3 (2307.6 to 2780.2)	769.3 (638.1 to 931.6)	85.6 (60.8 to 114.5)	0.0 (0.0 to 0.0)	3824.5 (3520.3 to 4144.3)	2856.9 (2611.6 to 3146.4)	870.7 (722.1 to 1054.3)	96.9 (68.8 to 129.6)	0.0 (0.0 to 0.0)
Italy	2040	3390.4 (3113.0 to 3692.4)	2530.5 (2305.3 to 2798.3)	773.9 (639.3 to 941.1)	86.0 (61.1 to 115.0)	0.0 (0.0 to 0.0)	3837.1 (3523.1 to 4178.9)	2863.9 (2609.0 to 3167.0)	875.9 (723.6 to 1065.1)	97.3 (69.2 to 130.1)	0.0 (0.0 to 0.0)
Italy	2041	3402.4 (3116.6 to 3712.5)	2537.0 (2304.2 to 2812.4)	778.9 (641.5 to 951.1)	86.4 (61.5 to 115.6)	0.0 (0.0 to 0.0)	3850.6 (3527.2 to 4201.6)	2871.3 (2607.7 to 3182.9)	881.6 (726.0 to 1076.4)	97.8 (69.6 to 130.8)	0.0 (0.0 to 0.0)
Italy	2042	3417.2 (3127.0 to 3726.3)	2545.7 (2305.1 to 2824.9)	784.6 (644.6 to 961.5)	86.9 (61.9 to 116.6)	0.0 (0.0 to 0.0)	3867.4 (3538.9 to 4217.2)	2881.0 (2608.7 to 3197.1)	888.0 (729.6 to 1088.1)	98.4 (70.0 to 132.0)	0.0 (0.0 to 0.0)
Italy	2043	3433.5 (3143.2 to 3747.9)	2555.3 (2309.4 to 2835.7)	790.8 (647.8 to 970.4)	87.5 (62.3 to 117.6)	0.0 (0.0 to 0.0)	3885.9 (3557.3 to 4241.7)	2891.9 (2613.6 to 3209.3)	894.9 (733.2 to 1098.2)	99.0 (70.5 to 133.1)	0.0 (0.0 to 0.0)
Italy	2044	3453.1 (3159.4 to 3773.3)	2567.3 (2316.1 to 2843.2)	797.6 (651.7 to 979.3)	88.2 (62.7 to 118.4)	0.0 (0.0 to 0.0)	3908.0 (3575.6 to 4270.4)	2905.6 (2621.2 to 3217.8)	902.6 (737.6 to 1108.3)	99.8 (71.0 to 134.0)	0.0 (0.0 to 0.0)
Italy	2045	3472.8 (3177.8 to 3790.0)	2579.2 (2322.0 to 2858.5)	804.7 (656.3 to 987.0)	88.9 (63.2 to 119.1)	0.0 (0.0 to 0.0)	3930.3 (3596.5 to 4289.3)	2918.9 (2627.9 to 3235.0)	910.7 (742.8 to 1117.0)	100.6 (71.6 to 134.8)	0.0 (0.0 to 0.0)
Italy	2046	3494.9 (3179.4 to 3822.9)	2592.7 (2329.8 to 2871.7)	812.5 (663.3 to 996.4)	89.7 (63.9 to 120.4)	0.0 (0.0 to 0.0)	3955.3 (3598.2 to 4326.6)	2934.3 (2636.8 to 3250.0)	919.6 (750.7 to 1127.7)	101.5 (72.4 to 136.3)	0.0 (0.0 to 0.0)
Italy	2047	3519.5 (3205.1 to 3856.3)	2608.0 (2339.7 to 2889.0)	820.9 (671.0 to 1006.4)	90.6 (64.4 to 122.0)	0.0 (0.0 to 0.0)	3983.2 (3627.3 to 4364.3)	2951.6 (2647.9 to 3269.6)	929.1 (759.4 to 1139.0)	102.5 (72.8 to 138.0)	0.0 (0.0 to 0.0)
Italy	2048	3544.7 (3232.0 to 3879.3)	2623.8 (2354.5 to 2913.5)	829.5 (676.4 to 1017.5)	91.5 (65.0 to 123.5)	0.0 (0.0 to 0.0)	4011.7 (3657.8 to 4390.4)	2969.4 (2664.6 to 3297.3)	938.7 (765.5 to 1151.5)	103.5 (73.5 to 139.8)	0.0 (0.0 to 0.0)
Italy	2049	3571.0 (3239.5 to 3920.0)	2640.4 (2366.8 to 2936.7)	838.2 (682.2 to 1027.6)	92.4 (65.6 to 125.1)	0.0 (0.0 to 0.0)	4041.5 (3666.2 to 4436.5)	2988.3 (2678.6 to 3323.6)	948.6 (772.1 to 1163.0)	104.6 (74.3 to 141.6)	0.0 (0.0 to 0.0)
Italy	2050	3598.0 (3269.2 to 3964.3)	2657.6 (2382.3 to 2961.0)	847.1 (689.9 to 1036.7)	93.4 (66.4 to 126.7)	0.0 (0.0 to 0.0)	4072.0 (3699.9 to 4486.6)	3007.7 (2696.1 to 3351.1)	958.7 (780.8 to 1173.3)	105.7 (75.1 to 143.3)	0.0 (0.0 to 0.0)
Jamaica	1995	246.7 (218.0 to 280.4)	137.0 (116.7 to 160.5)	60.1 (46.0 to 76.4)	35.4 (20.7 to 56.0)	14.2 (14.2 to 14.2)	447.3 (395.3 to 508.5)	248.5 (211.5 to 291.0)	109.0 (83.4 to 138.5)	64.2 (37.6 to 101.6)	25.7 (25.7 to 25.7)
Jamaica	1996	262.6 (233.1 to 296.1)	152.0 (131.5 to 176.2)	63.8 (49.7 to 79.1)	37.2 (22.5 to 56.8)	9.6 (9.6 to 9.6)	476.1 (422.8 to 536.9)	275.6 (238.4 to 319.5)	115.7 (90.1 to 143.5)	67.5 (40.8 to 103.0)	17.4 (17.4 to 17.4)
Jamaica	1997	277.3 (245.8 to 309.1)	160.0 (140.7 to 183.4)	71.2 (56.3 to 88.0)	37.8 (23.9 to 56.6)	8.3 (8.3 to 8.3)	502.9 (445.8 to 560.4)	290.1 (255.1 to 332.5)	129.1 (102.1 to 159.6)	68.6 (43.3 to 102.6)	15.0 (15.0 to 15.0)
Jamaica	1998	276.0 (247.2 to 307.7)	158.7 (140.0 to 181.0)	71.6 (56.7 to 88.2)	36.4 (22.7 to 53.7)	9.4 (9.4 to 9.4)	500.5 (448.3 to 558.0)	287.8 (253.8 to 328.2)	129.8 (102.8 to 160.0)	65.9 (41.2 to 97.3)	17.0 (17.0 to 17.0)
Jamaica	1999	272.9 (244.7 to 303.6)	152.9 (135.2 to 174.4)	75.6 (60.5 to 93.2)	36.1 (23.0 to 53.0)	8.2 (8.2 to 8.2)	494.9 (443.8 to 550.5)	277.3 (245.2 to 316.2)	137.2 (109.8 to 168.9)	65.4 (41.7 to 96.2)	15.0 (15.0 to 15.0)
Jamaica	2000	277.8 (249.3 to 308.3)	150.1 (132.3 to 170.9)	81.5 (66.4 to 99.3)	37.7 (24.6 to 54.7)	8.4 (8.4 to 8.4)	503.8 (452.1 to 559.1)	272.2 (239.9 to 309.8)	147.8 (120.4 to 180.1)	68.4 (44.6 to 99.2)	15.3 (15.3 to 15.3)
Jamaica	2001	280.1 (252.6 to 310.1)	140.4 (123.9 to 158.3)	83.1 (67.8 to 101.7)	36.5 (23.6 to 52.5)	20.1 (20.1 to 20.1)	507.9 (458.1 to 562.3)	254.5 (224.7 to 287.0)	150.8 (123.0 to 184.4)	66.2 (42.8 to 95.2)	36.4 (36.4 to 36.4)

Country	Year	2018 US Dollars per capita					2018 PPP per capita				
		Health spending	Government health spending	Out-of-pocket health spending	Prepaid private health spending	Development assistance for health	Health spending	Government health spending	Out-of-pocket health spending	Prepaid private health spending	Development assistance for health
Jamaica	2002	255.8 (228.7 to 284.9)	139.5 (122.7 to 158.7)	76.9 (62.4 to 94.5)	35.8 (23.5 to 51.5)	3.7 (3.7 to 3.7)	463.9 (414.7 to 516.7)	252.9 (222.6 to 287.7)	139.4 (113.1 to 171.4)	64.9 (42.6 to 93.3)	6.7 (6.7 to 6.7)
Jamaica	2003	256.4 (228.5 to 285.8)	139.3 (122.2 to 158.3)	76.6 (62.9 to 94.1)	35.7 (23.4 to 51.9)	4.8 (4.8 to 4.8)	465.0 (414.3 to 518.3)	252.7 (221.7 to 287.0)	138.9 (114.0 to 170.7)	64.8 (42.4 to 94.1)	8.7 (8.7 to 8.7)
Jamaica	2004	263.7 (236.2 to 293.8)	146.0 (128.5 to 165.9)	75.5 (62.6 to 92.4)	36.3 (23.8 to 53.2)	5.8 (5.8 to 5.8)	478.1 (428.3 to 532.7)	264.7 (233.0 to 300.8)	137.0 (113.5 to 167.6)	65.8 (43.2 to 96.4)	10.6 (10.6 to 10.6)
Jamaica	2005	256.0 (231.7 to 285.4)	141.1 (124.3 to 159.9)	74.2 (60.8 to 90.5)	34.7 (22.9 to 51.5)	5.9 (5.9 to 5.9)	464.1 (420.1 to 517.5)	255.8 (225.4 to 289.9)	134.6 (110.3 to 164.1)	63.0 (41.6 to 93.4)	10.7 (10.7 to 10.7)
Jamaica	2006	263.8 (238.4 to 293.5)	146.3 (129.1 to 166.1)	75.2 (61.1 to 91.8)	34.4 (22.4 to 50.5)	7.8 (7.8 to 7.8)	478.3 (432.3 to 532.2)	265.3 (234.1 to 301.1)	136.4 (110.8 to 166.5)	62.4 (40.7 to 91.6)	14.2 (14.2 to 14.2)
Jamaica	2007	275.3 (247.3 to 307.1)	153.3 (135.1 to 172.4)	79.9 (65.1 to 97.3)	34.6 (22.5 to 50.5)	7.4 (7.4 to 7.4)	499.2 (448.4 to 556.8)	278.0 (245.0 to 312.5)	145.0 (118.0 to 176.5)	62.8 (40.8 to 91.6)	13.5 (13.5 to 13.5)
Jamaica	2008	275.8 (247.5 to 307.2)	156.5 (137.7 to 177.4)	77.0 (63.0 to 92.9)	34.7 (22.6 to 50.9)	7.6 (7.6 to 7.6)	500.1 (448.9 to 557.1)	283.8 (249.8 to 321.8)	139.6 (114.3 to 168.4)	62.9 (41.0 to 92.3)	13.7 (13.7 to 13.7)
Jamaica	2009	264.7 (237.0 to 295.3)	154.1 (135.4 to 175.0)	70.8 (57.2 to 85.4)	33.3 (21.6 to 49.2)	6.5 (6.5 to 6.5)	480.0 (429.7 to 535.5)	279.4 (245.5 to 317.4)	128.4 (103.7 to 154.9)	60.4 (39.2 to 89.2)	11.8 (11.8 to 11.8)
Jamaica	2010	269.4 (242.7 to 298.3)	154.7 (137.4 to 174.7)	65.3 (53.2 to 79.5)	34.0 (21.9 to 50.1)	15.4 (15.4 to 15.4)	488.5 (440.1 to 540.9)	280.6 (249.2 to 316.7)	118.4 (96.4 to 144.2)	61.7 (39.7 to 90.9)	27.9 (27.9 to 27.9)
Jamaica	2011	265.2 (239.8 to 295.7)	157.6 (139.7 to 178.5)	61.4 (49.8 to 75.2)	37.0 (23.6 to 54.7)	9.3 (9.3 to 9.3)	480.9 (434.9 to 536.2)	285.7 (253.4 to 323.8)	111.3 (90.4 to 136.3)	67.1 (42.8 to 99.1)	16.9 (16.9 to 16.9)
Jamaica	2012	262.9 (236.2 to 294.9)	157.3 (138.7 to 177.6)	57.9 (46.7 to 71.6)	42.0 (27.2 to 61.3)	5.7 (5.7 to 5.7)	476.7 (428.3 to 534.7)	285.3 (251.5 to 322.1)	105.0 (84.6 to 129.8)	76.2 (49.3 to 111.1)	10.3 (10.3 to 10.3)
Jamaica	2013	273.5 (246.4 to 307.7)	162.6 (143.2 to 183.7)	56.3 (44.9 to 69.8)	46.5 (30.0 to 67.4)	8.0 (8.0 to 8.0)	496.0 (446.7 to 557.9)	294.9 (259.6 to 333.1)	102.2 (81.4 to 126.5)	84.3 (54.5 to 122.2)	14.6 (14.6 to 14.6)
Jamaica	2014	277.9 (249.6 to 311.1)	166.5 (146.7 to 188.6)	57.8 (45.2 to 71.0)	48.5 (31.6 to 72.5)	5.1 (5.1 to 5.1)	503.9 (452.6 to 564.2)	301.9 (266.0 to 342.0)	104.9 (82.0 to 128.8)	87.9 (57.3 to 131.5)	9.3 (9.3 to 9.3)
Jamaica	2015	294.0 (262.4 to 333.3)	178.0 (156.0 to 202.2)	60.1 (46.8 to 75.5)	51.5 (32.7 to 77.0)	4.4 (4.4 to 4.4)	533.0 (475.9 to 604.4)	322.7 (282.8 to 366.7)	109.0 (84.8 to 136.9)	93.3 (59.2 to 139.7)	8.0 (8.0 to 8.0)
Jamaica	2016	314.1 (273.3 to 356.8)	188.2 (162.4 to 217.6)	66.0 (50.4 to 84.8)	54.4 (33.3 to 83.3)	5.5 (5.5 to 5.5)	569.5 (495.6 to 647.1)	341.3 (294.5 to 394.5)	119.6 (91.4 to 153.7)	98.7 (60.4 to 151.1)	9.9 (9.9 to 9.9)
Jamaica	2017	323.6 (282.8 to 367.9)	194.5 (167.8 to 225.7)	66.4 (50.7 to 85.3)	54.7 (33.4 to 83.6)	7.9 (7.9 to 7.9)	586.7 (512.7 to 667.2)	352.7 (304.3 to 409.3)	120.4 (92.0 to 154.8)	99.3 (60.6 to 151.5)	14.4 (14.4 to 14.4)
Jamaica	2018	327.4 (284.9 to 371.3)	197.3 (169.8 to 228.5)	66.9 (51.0 to 86.1)	55.6 (33.9 to 85.0)	7.6 (7.6 to 7.6)	593.7 (516.7 to 673.2)	357.8 (307.9 to 414.3)	121.2 (92.4 to 156.1)	100.9 (61.5 to 154.2)	13.8 (13.7 to 13.8)
Jamaica	2019	331.8 (289.1 to 377.1)	200.3 (172.6 to 231.9)	67.3 (51.3 to 86.6)	56.4 (34.5 to 86.2)	7.8 (7.2 to 8.2)	601.7 (524.2 to 683.9)	363.2 (312.9 to 420.5)	122.0 (93.1 to 157.1)	102.3 (62.5 to 156.2)	14.1 (13.1 to 14.9)
Jamaica	2020	336.2 (292.7 to 380.8)	203.3 (175.0 to 235.0)	67.7 (51.6 to 87.3)	57.2 (35.0 to 87.4)	8.0 (7.3 to 8.6)	609.7 (530.8 to 690.6)	368.6 (317.2 to 426.2)	122.8 (93.6 to 158.3)	103.8 (63.5 to 158.5)	14.5 (13.3 to 15.6)
Jamaica	2021	340.7 (296.7 to 387.7)	206.3 (177.6 to 237.6)	68.2 (51.9 to 87.9)	58.1 (35.5 to 88.4)	8.2 (7.4 to 9.1)	617.8 (538.0 to 703.0)	374.0 (322.0 to 430.8)	123.6 (94.2 to 159.4)	105.3 (64.3 to 160.4)	14.9 (13.5 to 16.5)
Jamaica	2022	346.5 (301.0 to 393.5)	210.2 (181.1 to 242.6)	68.8 (52.5 to 88.7)	59.1 (36.0 to 90.2)	8.5 (7.6 to 9.5)	628.4 (545.8 to 713.6)	381.1 (328.5 to 439.8)	124.7 (95.2 to 160.8)	107.1 (65.2 to 163.6)	15.5 (13.7 to 17.3)

		2018 US Dollars per capita					2018 PPP per capita				
Country	Year	Health spending	Government health spending	Out-of-pocket health spending	Prepaid private health spending	Development assistance for health	Health spending	Government health spending	Out-of-pocket health spending	Prepaid private health spending	Development assistance for health
Jamaica	2023	352.3 (307.1 to 400.5)	214.1 (184.4 to 247.1)	69.3 (52.9 to 89.4)	60.0 (36.7 to 92.0)	8.8 (7.7 to 10.0)	638.8 (556.8 to 726.2)	388.2 (334.4 to 448.1)	125.7 (95.9 to 162.1)	108.8 (66.5 to 166.7)	16.0 (13.9 to 18.2)
Jamaica	2024	357.1 (310.0 to 405.5)	217.5 (186.9 to 251.7)	69.7 (53.2 to 89.7)	60.8 (37.1 to 92.9)	9.2 (7.9 to 10.6)	647.6 (562.2 to 735.2)	394.4 (338.9 to 456.5)	126.3 (96.5 to 162.7)	110.3 (67.2 to 168.5)	16.6 (14.3 to 19.2)
Jamaica	2025	361.1 (314.1 to 410.9)	220.4 (188.8 to 255.0)	69.8 (53.2 to 89.9)	61.5 (37.4 to 93.9)	9.5 (8.1 to 11.1)	654.8 (569.5 to 745.2)	399.7 (342.3 to 462.3)	126.6 (96.5 to 163.0)	111.4 (67.8 to 170.2)	17.2 (14.6 to 20.2)
Jamaica	2026	365.7 (318.1 to 414.5)	223.7 (191.3 to 258.6)	70.0 (53.4 to 90.2)	62.2 (37.8 to 95.0)	9.8 (8.3 to 11.5)	663.2 (576.7 to 751.6)	405.7 (346.9 to 469.0)	127.0 (96.7 to 163.5)	112.8 (68.6 to 172.2)	17.8 (15.0 to 20.8)
Jamaica	2027	369.0 (320.1 to 421.6)	226.2 (193.0 to 260.9)	70.0 (53.3 to 90.2)	62.7 (38.1 to 95.9)	10.1 (8.5 to 12.1)	669.1 (580.4 to 764.4)	410.1 (349.9 to 473.1)	126.9 (96.7 to 163.5)	113.7 (69.1 to 174.0)	18.4 (15.4 to 21.9)
Jamaica	2028	371.1 (321.4 to 421.4)	227.8 (193.8 to 263.4)	69.8 (53.1 to 89.7)	63.0 (38.3 to 96.5)	10.5 (8.8 to 12.7)	672.9 (582.8 to 764.2)	413.0 (351.4 to 477.7)	126.5 (96.2 to 162.7)	114.2 (69.4 to 175.0)	19.1 (15.9 to 23.1)
Jamaica	2029	372.8 (323.0 to 425.1)	229.2 (194.8 to 266.0)	69.4 (52.8 to 89.1)	63.2 (38.4 to 97.0)	11.0 (9.0 to 13.4)	676.0 (585.6 to 770.8)	415.6 (353.1 to 482.3)	125.9 (95.7 to 161.5)	114.6 (69.6 to 175.9)	19.9 (16.4 to 24.3)
Jamaica	2030	374.4 (323.7 to 425.7)	230.5 (195.3 to 267.9)	69.1 (52.5 to 88.5)	63.4 (38.5 to 97.5)	11.4 (9.3 to 14.0)	678.9 (587.0 to 771.9)	418.0 (354.2 to 485.9)	125.3 (95.2 to 160.5)	115.0 (69.8 to 176.9)	20.7 (16.9 to 25.4)
Jamaica	2031	376.4 (325.5 to 428.7)	232.0 (196.0 to 270.5)	68.8 (52.3 to 88.2)	63.7 (38.6 to 98.2)	11.8 (9.6 to 14.9)	682.6 (590.2 to 777.3)	420.7 (355.5 to 490.6)	124.8 (94.8 to 160.0)	115.6 (70.1 to 178.1)	21.5 (17.4 to 27.0)
Jamaica	2032	378.4 (329.2 to 431.4)	233.5 (196.7 to 272.7)	68.6 (52.0 to 87.9)	64.0 (38.8 to 98.8)	12.3 (9.9 to 15.5)	686.2 (596.9 to 782.3)	423.3 (356.6 to 494.4)	124.4 (94.4 to 159.4)	116.1 (70.4 to 179.1)	22.4 (17.9 to 28.2)
Jamaica	2033	380.6 (329.2 to 435.5)	235.0 (197.3 to 273.6)	68.4 (51.9 to 87.7)	64.4 (39.0 to 99.1)	12.8 (10.2 to 16.5)	690.1 (597.0 to 789.8)	426.1 (357.8 to 496.1)	124.1 (94.0 to 159.0)	116.7 (70.7 to 179.8)	23.3 (18.5 to 29.9)
Jamaica	2034	383.1 (333.0 to 437.2)	236.7 (198.1 to 276.3)	68.3 (51.7 to 87.5)	64.7 (39.2 to 99.6)	13.4 (10.5 to 17.2)	694.6 (603.9 to 792.7)	429.2 (359.1 to 501.0)	123.8 (93.7 to 158.6)	117.4 (71.1 to 180.6)	24.2 (19.1 to 31.1)
Jamaica	2035	385.8 (332.4 to 441.0)	238.6 (199.2 to 278.5)	68.1 (51.6 to 87.3)	65.1 (39.4 to 100.4)	13.9 (10.8 to 18.2)	699.5 (602.8 to 799.6)	432.6 (361.3 to 504.9)	123.5 (93.5 to 158.3)	118.1 (71.5 to 182.1)	25.3 (19.6 to 33.0)
Jamaica	2036	389.0 (337.0 to 445.0)	240.7 (200.7 to 281.4)	68.1 (51.5 to 87.3)	65.6 (39.7 to 101.4)	14.5 (11.2 to 19.5)	705.3 (611.1 to 806.8)	436.5 (364.0 to 510.2)	123.5 (93.4 to 158.4)	119.0 (71.9 to 183.9)	26.3 (20.4 to 35.4)
Jamaica	2037	392.5 (337.2 to 450.2)	243.1 (202.5 to 284.9)	68.2 (51.6 to 87.4)	66.2 (40.0 to 102.4)	15.0 (11.6 to 20.2)	711.8 (611.4 to 816.3)	440.8 (367.2 to 516.6)	123.6 (93.5 to 158.6)	120.1 (72.6 to 185.6)	27.3 (21.0 to 36.6)
Jamaica	2038	396.5 (341.8 to 453.9)	245.7 (204.8 to 288.3)	68.3 (51.7 to 87.6)	66.8 (40.4 to 103.2)	15.6 (11.9 to 21.6)	719.0 (619.8 to 823.1)	445.6 (371.4 to 522.8)	123.8 (93.7 to 158.8)	121.2 (73.3 to 187.2)	28.4 (21.6 to 39.2)
Jamaica	2039	400.4 (342.7 to 461.4)	248.3 (206.6 to 292.1)	68.4 (51.7 to 87.7)	67.5 (40.8 to 104.4)	16.3 (12.4 to 22.4)	726.0 (621.5 to 836.7)	450.2 (374.7 to 529.7)	124.0 (93.8 to 159.1)	122.3 (74.0 to 189.4)	29.5 (22.5 to 40.6)
Jamaica	2040	404.0 (348.8 to 464.2)	250.5 (208.1 to 295.3)	68.5 (51.8 to 87.8)	68.0 (41.1 to 105.4)	16.9 (12.9 to 23.3)	732.5 (632.5 to 841.8)	454.3 (377.4 to 535.4)	124.1 (93.9 to 159.2)	123.4 (74.6 to 191.1)	30.7 (23.4 to 42.2)
Jamaica	2041	408.0 (348.6 to 470.3)	253.1 (209.6 to 298.8)	68.6 (51.9 to 88.0)	68.7 (41.4 to 106.4)	17.6 (13.3 to 24.5)	739.8 (632.2 to 852.9)	458.9 (380.0 to 541.8)	124.4 (94.0 to 159.6)	124.6 (75.2 to 192.9)	32.0 (24.2 to 44.4)
Jamaica	2042	411.8 (354.7 to 473.4)	255.3 (210.8 to 302.6)	68.8 (51.9 to 88.2)	69.3 (41.8 to 107.3)	18.4 (13.8 to 26.3)	746.7 (643.2 to 858.5)	463.0 (382.2 to 548.7)	124.7 (94.2 to 160.0)	125.7 (75.7 to 194.6)	33.3 (25.0 to 47.7)
Jamaica	2043	415.7 (354.9 to 480.4)	257.7 (212.4 to 306.3)	68.9 (51.9 to 88.4)	70.0 (42.1 to 108.3)	19.1 (14.3 to 27.9)	753.8 (643.6 to 871.1)	467.3 (385.1 to 555.4)	124.9 (94.2 to 160.3)	126.9 (76.4 to 196.3)	34.7 (25.9 to 50.6)

Country	Year	2018 US Dollars per capita					2018 PPP per capita				
		Health spending	Government health spending	Out-of-pocket health spending	Prepaid private health spending	Development assistance for health	Health spending	Government health spending	Out-of-pocket health spending	Prepaid private health spending	Development assistance for health
Jamaica	2044	419.2 (360.0 to 482.7)	259.8 (213.6 to 309.5)	68.9 (51.9 to 88.4)	70.5 (42.4 to 109.1)	20.0 (14.8 to 29.0)	760.1 (652.8 to 875.3)	471.0 (387.3 to 561.3)	125.0 (94.1 to 160.3)	127.9 (76.9 to 197.8)	36.2 (26.8 to 52.6)
Jamaica	2045	422.7 (357.8 to 489.4)	261.8 (215.0 to 312.7)	68.9 (51.8 to 88.4)	71.1 (42.7 to 109.8)	20.9 (15.1 to 31.4)	766.5 (648.8 to 887.5)	474.8 (389.9 to 567.1)	125.0 (94.0 to 160.4)	128.9 (77.5 to 199.2)	37.9 (27.4 to 56.9)
Jamaica	2046	426.5 (366.5 to 492.6)	264.1 (216.8 to 315.7)	69.0 (51.8 to 88.5)	71.7 (43.1 to 110.7)	21.8 (15.7 to 33.2)	773.4 (664.6 to 893.2)	478.8 (393.1 to 572.5)	125.1 (94.0 to 160.6)	130.0 (78.2 to 200.8)	39.5 (28.5 to 60.2)
Jamaica	2047	430.6 (365.9 to 499.1)	266.5 (218.4 to 319.8)	69.0 (51.8 to 88.6)	72.3 (43.6 to 111.6)	22.8 (16.5 to 35.9)	780.9 (663.5 to 905.0)	483.3 (395.9 to 579.8)	125.2 (93.9 to 160.7)	131.1 (79.0 to 202.4)	41.3 (29.9 to 65.1)
Jamaica	2048	434.7 (372.2 to 503.3)	268.9 (219.7 to 323.3)	69.0 (51.8 to 88.7)	72.9 (44.0 to 112.6)	23.9 (17.1 to 37.9)	788.2 (675.0 to 912.7)	487.6 (398.3 to 586.3)	125.2 (93.9 to 160.8)	132.1 (79.7 to 204.2)	43.3 (31.1 to 68.7)
Jamaica	2049	438.6 (370.5 to 508.5)	271.2 (220.7 to 326.3)	69.0 (51.8 to 88.6)	73.4 (44.2 to 113.7)	25.0 (17.9 to 40.3)	795.3 (671.8 to 922.1)	491.7 (400.2 to 591.7)	125.1 (93.9 to 160.7)	133.1 (80.2 to 206.2)	45.3 (32.4 to 73.1)
Jamaica	2050	442.4 (377.6 to 513.4)	273.4 (221.5 to 329.9)	68.9 (51.7 to 88.5)	73.9 (44.4 to 114.8)	26.3 (18.6 to 43.0)	802.2 (684.6 to 931.0)	495.7 (401.6 to 598.2)	124.9 (93.7 to 160.4)	134.0 (80.6 to 208.1)	47.6 (33.7 to 78.0)
Japan	1995	1873.8 (1782.1 to 1964.3)	1518.7 (1440.2 to 1601.0)	285.4 (256.7 to 313.9)	69.7 (49.4 to 95.5)	0.0 (0.0 to 0.0)	2094.5 (1991.9 to 2195.6)	1697.6 (1609.8 to 1789.5)	319.0 (286.9 to 350.9)	77.9 (55.2 to 106.8)	0.0 (0.0 to 0.0)
Japan	1996	1951.7 (1887.8 to 2025.0)	1579.4 (1518.8 to 1642.4)	300.4 (276.7 to 324.9)	72.0 (53.7 to 94.4)	0.0 (0.0 to 0.0)	2181.6 (2110.1 to 2263.5)	1765.4 (1697.7 to 1835.8)	335.8 (309.3 to 363.1)	80.5 (60.1 to 105.6)	0.0 (0.0 to 0.0)
Japan	1997	2038.2 (1977.5 to 2103.2)	1640.4 (1583.2 to 1697.8)	323.9 (302.9 to 346.0)	73.9 (57.5 to 95.3)	0.0 (0.0 to 0.0)	2278.3 (2210.4 to 2350.9)	1833.6 (1769.7 to 1897.7)	362.0 (338.6 to 386.7)	82.7 (64.3 to 106.5)	0.0 (0.0 to 0.0)
Japan	1998	2107.8 (2039.1 to 2172.2)	1688.8 (1628.3 to 1747.4)	343.4 (321.9 to 365.1)	75.7 (60.2 to 94.6)	0.0 (0.0 to 0.0)	2356.1 (2279.2 to 2428.1)	1887.7 (1820.0 to 1953.2)	383.8 (359.8 to 408.1)	84.6 (67.3 to 105.7)	0.0 (0.0 to 0.0)
Japan	1999	2215.5 (2146.2 to 2283.2)	1774.2 (1712.8 to 1836.2)	362.7 (342.3 to 385.1)	78.6 (64.0 to 95.7)	0.0 (0.0 to 0.0)	2476.5 (2398.9 to 2552.1)	1983.2 (1914.5 to 2052.5)	405.4 (382.6 to 430.4)	87.9 (71.5 to 107.0)	0.0 (0.0 to 0.0)
Japan	2000	2387.6 (2320.5 to 2457.5)	1919.6 (1855.5 to 1987.8)	384.2 (365.0 to 405.1)	83.7 (68.9 to 100.8)	0.0 (0.0 to 0.0)	2668.8 (2593.8 to 2746.9)	2145.7 (2074.0 to 2221.9)	429.5 (407.9 to 452.8)	93.6 (77.0 to 112.7)	0.0 (0.0 to 0.0)
Japan	2001	2490.9 (2425.5 to 2560.6)	2008.4 (1946.8 to 2072.4)	398.9 (380.0 to 418.6)	83.7 (70.0 to 99.7)	0.0 (0.0 to 0.0)	2784.3 (2711.1 to 2862.2)	2244.9 (2176.1 to 2316.5)	445.9 (424.8 to 468.0)	93.5 (78.3 to 111.4)	0.0 (0.0 to 0.0)
Japan	2002	2552.6 (2487.5 to 2621.2)	2056.3 (1997.6 to 2117.3)	413.1 (393.1 to 432.1)	83.2 (69.9 to 98.5)	0.0 (0.0 to 0.0)	2853.2 (2780.5 to 2929.9)	2298.4 (2232.8 to 2366.7)	461.7 (439.4 to 483.0)	93.0 (78.1 to 110.1)	0.0 (0.0 to 0.0)
Japan	2003	2620.8 (2554.1 to 2690.0)	2106.1 (2049.6 to 2167.7)	431.2 (410.5 to 450.2)	83.5 (70.4 to 98.7)	0.0 (0.0 to 0.0)	2929.5 (2854.9 to 3006.8)	2354.2 (2291.0 to 2423.0)	482.0 (458.8 to 503.2)	93.4 (78.7 to 110.3)	0.0 (0.0 to 0.0)
Japan	2004	2692.7 (2628.8 to 2758.5)	2165.7 (2101.6 to 2226.8)	441.7 (421.9 to 461.0)	85.3 (71.4 to 100.0)	0.0 (0.0 to 0.0)	3009.9 (2938.4 to 3083.3)	2420.8 (2349.1 to 2489.1)	493.7 (471.6 to 515.3)	95.4 (79.8 to 111.8)	0.0 (0.0 to 0.0)
Japan	2005	2754.0 (2682.9 to 2825.1)	2220.0 (2154.5 to 2281.7)	445.7 (425.7 to 465.4)	88.3 (74.2 to 103.7)	0.0 (0.0 to 0.0)	3078.3 (2998.9 to 3157.8)	2481.4 (2408.2 to 2550.4)	498.2 (475.9 to 520.2)	98.7 (82.9 to 115.9)	0.0 (0.0 to 0.0)
Japan	2006	2816.2 (2749.2 to 2887.6)	2270.7 (2206.8 to 2331.2)	453.2 (432.0 to 473.3)	92.3 (77.3 to 107.8)	0.0 (0.0 to 0.0)	3147.9 (3073.0 to 3227.7)	2538.2 (2466.8 to 2605.7)	506.5 (482.9 to 529.0)	103.2 (86.4 to 120.5)	0.0 (0.0 to 0.0)
Japan	2007	2911.9 (2844.0 to 2977.8)	2357.8 (2296.3 to 2421.8)	456.4 (436.9 to 475.6)	97.6 (82.6 to 113.5)	0.0 (0.0 to 0.0)	3254.8 (3179.0 to 3328.5)	2635.5 (2566.8 to 2707.0)	510.2 (488.4 to 531.6)	109.1 (92.4 to 126.8)	0.0 (0.0 to 0.0)
Japan	2008	2999.7 (2930.7 to 3063.2)	2435.6 (2372.4 to 2496.4)	460.0 (441.3 to 481.1)	104.1 (89.2 to 121.0)	0.0 (0.0 to 0.0)	3353.0 (3275.9 to 3424.0)	2722.5 (2651.8 to 2790.4)	514.2 (493.2 to 537.8)	116.3 (99.7 to 135.2)	0.0 (0.0 to 0.0)

		2018 US Dollars per capita					2018 PPP per capita				
Country	Year	Health spending	Government health spending	Out-of-pocket health spending	Prepaid private health spending	Development assistance for health	Health spending	Government health spending	Out-of-pocket health spending	Prepaid private health spending	Development assistance for health
Japan	2009	3076.3 (3010.7 to 3144.4)	2506.6 (2444.7 to 2568.3)	462.6 (443.5 to 482.7)	107.1 (92.1 to 123.2)	0.0 (0.0 to 0.0)	3438.6 (3365.3 to 3514.7)	2801.8 (2732.7 to 2870.8)	517.1 (495.7 to 539.6)	119.7 (102.9 to 137.7)	0.0 (0.0 to 0.0)
Japan	2010	3396.8 (3324.7 to 3468.4)	2800.8 (2735.2 to 2867.6)	481.1 (461.3 to 502.3)	114.8 (99.1 to 132.3)	0.0 (0.0 to 0.0)	3796.8 (3716.2 to 3876.9)	3130.7 (3057.4 to 3205.4)	537.8 (515.7 to 561.5)	128.3 (110.8 to 147.9)	0.0 (0.0 to 0.0)
Japan	2011	3694.8 (3620.4 to 3766.3)	3079.6 (3014.5 to 3148.9)	495.3 (474.3 to 517.1)	119.9 (103.2 to 138.6)	0.0 (0.0 to 0.0)	4129.9 (4046.8 to 4209.8)	3442.3 (3369.5 to 3519.7)	553.6 (530.2 to 578.0)	134.1 (115.4 to 155.0)	0.0 (0.0 to 0.0)
Japan	2012	3915.7 (3839.2 to 3989.0)	3286.7 (3217.9 to 3354.9)	506.5 (486.0 to 527.8)	122.5 (104.7 to 142.0)	0.0 (0.0 to 0.0)	4376.8 (4291.4 to 4458.8)	3673.8 (3596.9 to 3750.1)	566.2 (543.3 to 590.0)	136.9 (117.0 to 158.7)	0.0 (0.0 to 0.0)
Japan	2013	4033.3 (3956.1 to 4112.6)	3396.4 (3326.4 to 3472.9)	514.1 (491.8 to 535.7)	122.8 (104.4 to 141.8)	0.0 (0.0 to 0.0)	4508.3 (4422.0 to 4597.0)	3796.4 (3718.2 to 3881.9)	574.6 (549.8 to 598.8)	137.3 (116.7 to 158.5)	0.0 (0.0 to 0.0)
Japan	2014	4060.7 (3982.4 to 4133.7)	3416.9 (3345.5 to 3489.0)	522.2 (499.2 to 545.0)	121.6 (103.6 to 141.3)	0.0 (0.0 to 0.0)	4539.0 (4451.4 to 4620.6)	3819.3 (3739.6 to 3899.9)	583.7 (558.0 to 609.2)	136.0 (115.8 to 157.9)	0.0 (0.0 to 0.0)
Japan	2015	4119.9 (4039.5 to 4199.3)	3459.8 (3390.0 to 3530.4)	537.8 (513.7 to 563.2)	122.3 (102.6 to 143.7)	0.0 (0.0 to 0.0)	4605.1 (4515.2 to 4693.9)	3867.3 (3789.3 to 3946.2)	601.1 (574.2 to 629.5)	136.7 (114.7 to 160.6)	0.0 (0.0 to 0.0)
Japan	2016	4175.1 (4064.6 to 4277.9)	3494.0 (3387.6 to 3592.3)	556.9 (522.2 to 591.3)	124.2 (99.4 to 149.8)	0.0 (0.0 to 0.0)	4666.9 (4543.3 to 4781.7)	3905.5 (3786.6 to 4015.4)	622.5 (583.8 to 661.0)	138.9 (111.1 to 167.4)	0.0 (0.0 to 0.0)
Japan	2017	4253.1 (4143.0 to 4361.4)	3559.6 (3453.4 to 3661.4)	566.3 (532.3 to 601.2)	127.2 (101.5 to 153.4)	0.0 (0.0 to 0.0)	4754.0 (4631.0 to 4875.1)	3978.8 (3860.1 to 4092.6)	633.0 (595.0 to 672.0)	142.2 (113.4 to 171.5)	0.0 (0.0 to 0.0)
Japan	2018	4296.9 (4181.1 to 4411.0)	3593.0 (3487.0 to 3698.2)	574.9 (539.0 to 610.4)	128.9 (102.7 to 155.6)	0.0 (0.0 to 0.0)	4803.0 (4673.5 to 4930.5)	4016.2 (3897.7 to 4133.7)	642.6 (602.5 to 682.3)	144.1 (114.8 to 173.9)	0.0 (0.0 to 0.0)
Japan	2019	4341.0 (4220.5 to 4460.6)	3627.3 (3517.1 to 3737.5)	583.0 (544.2 to 620.2)	130.7 (103.9 to 157.9)	0.0 (0.0 to 0.0)	4852.2 (4717.5 to 4985.9)	4054.5 (3931.3 to 4177.7)	651.6 (608.3 to 693.3)	146.1 (116.2 to 176.5)	0.0 (0.0 to 0.0)
Japan	2020	4383.0 (4258.4 to 4507.7)	3660.1 (3547.5 to 3774.5)	590.4 (549.1 to 629.5)	132.4 (105.4 to 160.1)	0.0 (0.0 to 0.0)	4899.2 (4760.0 to 5038.6)	4091.2 (3965.3 to 4219.1)	659.9 (613.8 to 703.6)	148.0 (117.8 to 178.9)	0.0 (0.0 to 0.0)
Japan	2021	4424.0 (4292.9 to 4554.0)	3692.7 (3572.9 to 3812.8)	597.0 (555.8 to 636.5)	134.3 (106.7 to 162.5)	0.0 (0.0 to 0.0)	4945.0 (4798.6 to 5090.3)	4127.6 (3993.7 to 4261.8)	667.3 (621.2 to 711.4)	150.1 (119.2 to 181.6)	0.0 (0.0 to 0.0)
Japan	2022	4474.2 (4335.0 to 4611.0)	3734.6 (3610.4 to 3862.8)	603.2 (560.6 to 647.1)	136.4 (108.4 to 165.0)	0.0 (0.0 to 0.0)	5001.1 (4845.5 to 5154.0)	4174.4 (4035.6 to 4317.8)	674.2 (626.7 to 723.3)	152.5 (121.2 to 184.4)	0.0 (0.0 to 0.0)
Japan	2023	4537.3 (4394.2 to 4683.2)	3788.3 (3653.0 to 3924.0)	610.2 (565.2 to 655.0)	138.7 (110.4 to 167.8)	0.0 (0.0 to 0.0)	5071.6 (4911.8 to 5234.8)	4234.5 (4083.2 to 4386.1)	682.1 (631.8 to 732.1)	155.1 (123.4 to 187.5)	0.0 (0.0 to 0.0)
Japan	2024	4604.0 (4450.6 to 4762.3)	3844.3 (3701.9 to 3987.1)	618.6 (571.8 to 665.6)	141.1 (112.5 to 170.8)	0.0 (0.0 to 0.0)	5146.3 (4974.8 to 5323.2)	4297.1 (4137.9 to 4456.7)	691.5 (639.2 to 744.0)	157.7 (125.7 to 191.0)	0.0 (0.0 to 0.0)
Japan	2025	4668.6 (4505.6 to 4837.2)	3898.7 (3744.9 to 4050.9)	626.5 (577.0 to 675.9)	143.4 (114.3 to 173.8)	0.0 (0.0 to 0.0)	5218.4 (5036.2 to 5406.9)	4357.9 (4185.9 to 4528.0)	700.3 (644.9 to 755.5)	160.3 (127.7 to 194.3)	0.0 (0.0 to 0.0)
Japan	2026	4733.6 (4565.6 to 4910.1)	3953.2 (3790.4 to 4115.3)	634.6 (583.8 to 689.0)	145.8 (116.4 to 176.7)	0.0 (0.0 to 0.0)	5291.1 (5103.4 to 5488.3)	4418.8 (4236.8 to 4600.0)	709.3 (652.5 to 770.1)	163.0 (130.1 to 197.5)	0.0 (0.0 to 0.0)
Japan	2027	4799.3 (4623.1 to 4987.2)	4008.3 (3838.2 to 4183.4)	642.7 (586.3 to 704.4)	148.3 (118.2 to 179.8)	0.0 (0.0 to 0.0)	5364.6 (5167.6 to 5574.6)	4480.4 (4290.3 to 4676.1)	718.4 (655.3 to 787.4)	165.7 (132.2 to 200.9)	0.0 (0.0 to 0.0)
Japan	2028	4867.9 (4676.9 to 5072.8)	4066.0 (3885.4 to 4257.9)	651.2 (589.7 to 719.8)	150.8 (120.0 to 183.1)	0.0 (0.0 to 0.0)	5441.2 (5227.7 to 5670.3)	4544.9 (4343.0 to 4759.4)	727.8 (659.2 to 804.5)	168.5 (134.1 to 204.7)	0.0 (0.0 to 0.0)
Japan	2029	4937.0 (4730.5 to 5148.4)	4124.0 (3929.3 to 4329.8)	659.7 (593.5 to 734.4)	153.3 (121.5 to 186.4)	0.0 (0.0 to 0.0)	5518.4 (5287.6 to 5754.7)	4609.7 (4392.0 to 4839.8)	737.4 (663.5 to 820.8)	171.3 (135.8 to 208.4)	0.0 (0.0 to 0.0)

Country	Year	2018 US Dollars per capita					2018 PPP per capita				
		Health spending	Government health spending	Out-of-pocket health spending	Prepaid private health spending	Development assistance for health	Health spending	Government health spending	Out-of-pocket health spending	Prepaid private health spending	Development assistance for health
Japan	2030	5007.8 (4784.5 to 5231.4)	4183.8 (3974.0 to 4402.8)	668.3 (597.5 to 749.9)	155.8 (123.1 to 189.6)	0.0 (0.0 to 0.0)	5597.6 (5348.0 to 5847.5)	4676.5 (4442.1 to 4921.4)	747.0 (667.9 to 838.3)	174.1 (137.6 to 211.9)	0.0 (0.0 to 0.0)
Japan	2031	5079.3 (4842.2 to 5338.0)	4243.7 (4021.1 to 4480.7)	677.2 (599.9 to 765.1)	158.3 (125.1 to 192.9)	0.0 (0.0 to 0.0)	5677.5 (5412.5 to 5966.7)	4743.5 (4494.7 to 5008.4)	757.0 (670.5 to 855.2)	177.0 (139.9 to 215.6)	0.0 (0.0 to 0.0)
Japan	2032	5150.5 (4891.3 to 5408.4)	4303.4 (4068.7 to 4554.1)	686.2 (601.2 to 782.6)	160.9 (127.0 to 196.1)	0.0 (0.0 to 0.0)	5757.1 (5467.4 to 6045.4)	4810.3 (4547.9 to 5090.4)	767.0 (672.0 to 874.7)	179.9 (141.9 to 219.2)	0.0 (0.0 to 0.0)
Japan	2033	5224.0 (4946.6 to 5515.5)	4365.2 (4112.3 to 4630.1)	695.3 (603.4 to 800.5)	163.5 (128.9 to 199.4)	0.0 (0.0 to 0.0)	5839.3 (5529.2 to 6165.1)	4879.3 (4596.6 to 5175.4)	777.2 (674.4 to 894.8)	182.8 (144.1 to 222.8)	0.0 (0.0 to 0.0)
Japan	2034	5296.2 (5011.7 to 5598.1)	4426.0 (4155.8 to 4708.1)	704.2 (606.9 to 817.0)	166.0 (130.8 to 202.5)	0.0 (0.0 to 0.0)	5920.0 (5602.0 to 6257.4)	4947.3 (4645.2 to 5262.6)	787.2 (678.3 to 913.2)	185.5 (146.2 to 226.3)	0.0 (0.0 to 0.0)
Japan	2035	5367.3 (5062.6 to 5703.0)	4486.1 (4201.9 to 4781.7)	712.9 (609.6 to 833.7)	168.3 (132.4 to 205.4)	0.0 (0.0 to 0.0)	5999.5 (5658.8 to 6374.6)	5014.5 (4696.8 to 5344.8)	796.9 (681.4 to 931.9)	188.1 (148.0 to 229.6)	0.0 (0.0 to 0.0)
Japan	2036	5436.4 (5101.5 to 5810.4)	4544.3 (4239.3 to 4858.5)	721.6 (612.3 to 850.1)	170.5 (134.2 to 208.2)	0.0 (0.0 to 0.0)	6076.7 (5702.4 to 6494.7)	5079.5 (4738.6 to 5430.7)	806.6 (684.5 to 950.2)	190.6 (150.0 to 232.8)	0.0 (0.0 to 0.0)
Japan	2037	5504.7 (5154.0 to 5882.2)	4602.1 (4276.3 to 4931.8)	730.1 (615.3 to 865.5)	172.6 (135.8 to 210.9)	0.0 (0.0 to 0.0)	6153.1 (5761.0 to 6575.0)	5144.1 (4780.0 to 5512.6)	816.1 (687.8 to 967.5)	192.9 (151.8 to 235.8)	0.0 (0.0 to 0.0)
Japan	2038	5571.4 (5209.6 to 5962.8)	4658.4 (4314.3 to 5006.5)	738.4 (619.1 to 880.7)	174.6 (137.3 to 213.5)	0.0 (0.0 to 0.0)	6227.6 (5823.2 to 6665.0)	5207.0 (4822.4 to 5596.1)	825.4 (692.1 to 984.4)	195.1 (153.4 to 238.6)	0.0 (0.0 to 0.0)
Japan	2039	5638.5 (5256.7 to 6038.8)	4714.9 (4356.6 to 5078.4)	746.9 (623.1 to 896.0)	176.6 (138.8 to 216.1)	0.0 (0.0 to 0.0)	6302.6 (5875.8 to 6750.0)	5270.2 (4869.7 to 5676.5)	834.9 (696.5 to 1001.5)	197.4 (155.1 to 241.5)	0.0 (0.0 to 0.0)
Japan	2040	5708.4 (5302.7 to 6132.1)	4773.7 (4397.3 to 5158.7)	755.9 (628.0 to 910.2)	178.8 (140.3 to 219.1)	0.0 (0.0 to 0.0)	6380.7 (5927.3 to 6854.3)	5335.9 (4915.2 to 5766.2)	845.0 (701.9 to 1017.4)	199.9 (156.8 to 244.9)	0.0 (0.0 to 0.0)
Japan	2041	5781.9 (5338.2 to 6231.9)	4835.0 (4435.0 to 5243.5)	765.6 (633.3 to 925.2)	181.3 (142.2 to 223.1)	0.0 (0.0 to 0.0)	6462.8 (5966.9 to 6965.9)	5404.5 (4957.4 to 5861.0)	855.7 (707.9 to 1034.1)	202.6 (159.0 to 249.4)	0.0 (0.0 to 0.0)
Japan	2042	5858.6 (5431.8 to 6323.7)	4898.8 (4477.5 to 5322.5)	775.8 (640.8 to 940.3)	184.0 (144.2 to 226.7)	0.0 (0.0 to 0.0)	6548.6 (6071.6 to 7068.5)	5475.8 (5004.8 to 5949.3)	867.1 (716.2 to 1051.0)	205.6 (161.2 to 253.4)	0.0 (0.0 to 0.0)
Japan	2043	5936.9 (5467.0 to 6429.6)	4963.7 (4521.1 to 5416.5)	786.4 (647.8 to 955.1)	186.8 (146.5 to 230.3)	0.0 (0.0 to 0.0)	6636.1 (6110.9 to 7186.9)	5548.3 (5053.6 to 6054.4)	879.0 (724.1 to 1067.6)	208.8 (163.7 to 257.4)	0.0 (0.0 to 0.0)
Japan	2044	6017.5 (5536.7 to 6524.0)	5030.4 (4565.1 to 5502.0)	797.3 (654.7 to 970.0)	189.8 (148.7 to 234.0)	0.0 (0.0 to 0.0)	6726.2 (6188.8 to 7292.4)	5622.9 (5102.8 to 6150.0)	891.2 (731.8 to 1084.3)	212.2 (166.2 to 261.5)	0.0 (0.0 to 0.0)
Japan	2045	6098.0 (5592.6 to 6630.7)	5097.0 (4616.1 to 5590.2)	808.2 (663.3 to 984.6)	192.7 (151.0 to 237.8)	0.0 (0.0 to 0.0)	6816.2 (6251.3 to 7411.6)	5697.3 (5159.8 to 6248.6)	903.4 (741.4 to 1100.5)	215.4 (168.8 to 265.8)	0.0 (0.0 to 0.0)
Japan	2046	6179.7 (5630.7 to 6718.1)	5164.4 (4663.1 to 5672.8)	819.6 (671.7 to 999.6)	195.8 (153.2 to 242.2)	0.0 (0.0 to 0.0)	6907.6 (6293.9 to 7509.4)	5772.6 (5212.4 to 6341.0)	916.1 (750.8 to 1117.3)	218.9 (171.3 to 270.7)	0.0 (0.0 to 0.0)
Japan	2047	6264.3 (5706.5 to 6833.0)	5233.9 (4701.0 to 5767.5)	831.3 (680.6 to 1014.5)	199.0 (155.6 to 246.8)	0.0 (0.0 to 0.0)	7002.1 (6378.6 to 7637.8)	5850.4 (5254.7 to 6446.7)	929.2 (760.8 to 1133.9)	222.5 (174.0 to 275.8)	0.0 (0.0 to 0.0)
Japan	2048	6348.2 (5759.2 to 6945.4)	5302.9 (4741.5 to 5860.2)	843.1 (689.6 to 1029.4)	202.3 (157.9 to 251.5)	0.0 (0.0 to 0.0)	7095.9 (6437.5 to 7763.3)	5927.5 (5300.0 to 6550.4)	942.3 (770.9 to 1150.6)	226.1 (176.5 to 281.1)	0.0 (0.0 to 0.0)
Japan	2049	6434.9 (5824.3 to 7067.4)	5374.3 (4788.2 to 5955.9)	855.1 (698.8 to 1044.8)	205.6 (160.2 to 256.2)	0.0 (0.0 to 0.0)	7192.8 (6510.3 to 7899.8)	6007.2 (5352.1 to 6657.4)	955.8 (781.1 to 1167.8)	229.8 (179.0 to 286.4)	0.0 (0.0 to 0.0)
Japan	2050	6524.6 (5888.9 to 7183.2)	5448.3 (4830.7 to 6052.5)	867.3 (708.2 to 1060.2)	209.0 (162.5 to 261.2)	0.0 (0.0 to 0.0)	7293.1 (6582.5 to 8029.2)	6090.0 (5399.6 to 6765.3)	969.5 (791.6 to 1185.1)	233.6 (181.6 to 291.9)	0.0 (0.0 to 0.0)

		2018 US Dollars per capita					2018 PPP per capita				
Country	Year	Health spending	Government health spending	Out-of-pocket health spending	Prepaid private health spending	Development assistance for health	Health spending	Government health spending	Out-of-pocket health spending	Prepaid private health spending	Development assistance for health
Jordan	1995	272.9 (241.8 to 306.2)	172.0 (148.6 to 197.1)	69.5 (54.5 to 87.5)	25.5 (14.7 to 43.0)	5.8 (5.8 to 5.8)	619.5 (548.9 to 695.1)	390.6 (337.4 to 447.4)	157.8 (123.7 to 198.6)	58.0 (33.3 to 97.7)	13.2 (13.2 to 13.2)
Jordan	1996	274.4 (245.9 to 304.6)	169.9 (148.0 to 193.2)	75.7 (60.3 to 92.9)	25.9 (15.4 to 42.4)	2.8 (2.8 to 2.8)	622.9 (558.1 to 691.5)	385.8 (336.0 to 438.5)	171.9 (137.0 to 210.8)	58.8 (34.9 to 96.4)	6.3 (6.3 to 6.3)
Jordan	1997	285.2 (257.6 to 313.8)	169.0 (149.1 to 190.3)	85.0 (69.6 to 102.8)	26.7 (16.7 to 41.9)	4.4 (4.4 to 4.4)	647.5 (584.7 to 712.3)	383.7 (338.5 to 432.1)	193.0 (158.0 to 233.5)	60.7 (37.8 to 95.2)	10.1 (10.1 to 10.1)
Jordan	1998	299.0 (271.2 to 327.6)	168.7 (150.6 to 188.4)	95.6 (79.1 to 113.1)	28.4 (18.2 to 42.6)	6.3 (6.3 to 6.3)	678.8 (615.7 to 743.6)	383.0 (341.8 to 427.8)	217.1 (179.5 to 256.8)	64.4 (41.2 to 96.7)	14.2 (14.2 to 14.2)
Jordan	1999	320.8 (290.8 to 350.3)	167.0 (149.5 to 185.8)	110.5 (92.8 to 129.7)	31.3 (19.9 to 46.2)	12.0 (12.0 to 12.0)	728.3 (660.1 to 795.1)	379.0 (339.3 to 421.7)	250.9 (210.7 to 294.5)	71.0 (45.3 to 104.9)	27.3 (27.3 to 27.3)
Jordan	2000	346.8 (316.1 to 380.3)	167.8 (150.4 to 186.8)	130.3 (110.7 to 151.3)	38.7 (24.9 to 56.7)	10.0 (10.0 to 10.0)	787.2 (717.7 to 863.3)	380.9 (341.5 to 424.1)	295.7 (251.4 to 343.6)	87.9 (56.6 to 128.7)	22.6 (22.6 to 22.6)
Jordan	2001	367.5 (335.3 to 402.8)	172.6 (154.8 to 191.3)	144.5 (124.3 to 167.6)	41.6 (26.4 to 61.0)	8.8 (8.8 to 8.8)	834.2 (761.2 to 914.4)	391.8 (351.4 to 434.3)	328.0 (282.2 to 380.4)	94.5 (59.9 to 138.4)	20.0 (20.0 to 20.0)
Jordan	2002	377.5 (346.4 to 411.3)	176.3 (157.9 to 196.3)	153.2 (132.8 to 176.5)	38.3 (24.7 to 55.5)	9.7 (9.7 to 9.7)	856.9 (786.5 to 933.8)	400.2 (358.6 to 445.6)	347.9 (301.4 to 400.6)	86.9 (56.0 to 125.9)	21.9 (21.9 to 21.9)
Jordan	2003	368.5 (339.1 to 401.2)	172.8 (155.4 to 193.0)	154.8 (134.9 to 177.3)	30.9 (19.6 to 45.9)	10.0 (10.0 to 10.0)	836.5 (769.7 to 910.8)	392.3 (352.9 to 438.2)	351.4 (306.3 to 402.4)	70.2 (44.6 to 104.3)	22.6 (22.6 to 22.6)
Jordan	2004	361.4 (332.7 to 392.7)	174.3 (157.2 to 193.2)	153.6 (134.4 to 175.2)	25.5 (16.1 to 39.3)	8.1 (8.1 to 8.1)	820.5 (755.4 to 891.5)	395.6 (356.8 to 438.7)	348.6 (305.0 to 397.8)	57.8 (36.6 to 89.1)	18.4 (18.4 to 18.4)
Jordan	2005	364.2 (335.7 to 395.1)	187.6 (169.3 to 208.3)	149.0 (128.9 to 171.1)	22.7 (14.5 to 35.5)	4.9 (4.9 to 4.9)	826.7 (762.0 to 896.8)	425.9 (384.2 to 472.9)	338.2 (292.6 to 388.5)	51.5 (32.9 to 80.6)	11.0 (11.0 to 11.0)
Jordan	2006	358.6 (330.4 to 388.8)	196.6 (178.1 to 217.3)	138.2 (119.6 to 160.1)	20.2 (12.7 to 31.8)	3.6 (3.6 to 3.6)	814.1 (750.1 to 882.7)	446.3 (404.3 to 493.3)	313.7 (271.4 to 363.3)	45.8 (28.9 to 72.2)	8.2 (8.2 to 8.2)
Jordan	2007	369.0 (340.8 to 400.2)	216.0 (195.6 to 237.7)	129.5 (111.9 to 150.1)	20.5 (12.6 to 31.3)	3.0 (3.0 to 3.0)	837.7 (773.7 to 908.6)	490.4 (444.1 to 539.7)	294.0 (254.0 to 340.7)	46.6 (28.6 to 71.0)	6.7 (6.7 to 6.7)
Jordan	2008	394.7 (364.1 to 427.8)	243.2 (220.6 to 267.1)	120.3 (103.8 to 140.1)	24.7 (15.5 to 37.3)	6.4 (6.4 to 6.4)	896.0 (826.6 to 971.1)	552.1 (500.8 to 606.3)	273.2 (235.5 to 318.0)	56.1 (35.1 to 84.7)	14.6 (14.6 to 14.6)
Jordan	2009	405.2 (373.4 to 438.4)	264.3 (241.0 to 289.9)	105.8 (90.2 to 123.9)	28.3 (17.8 to 41.8)	6.8 (6.8 to 6.8)	919.9 (847.6 to 995.2)	600.1 (547.2 to 658.1)	240.1 (204.9 to 281.3)	64.2 (40.3 to 94.9)	15.5 (15.5 to 15.5)
Jordan	2010	380.2 (348.5 to 414.1)	254.1 (231.2 to 279.7)	90.7 (77.0 to 107.1)	28.8 (18.0 to 42.5)	6.7 (6.7 to 6.7)	863.2 (791.2 to 940.0)	576.7 (524.9 to 634.9)	205.9 (174.7 to 243.2)	65.3 (40.9 to 96.4)	15.2 (15.2 to 15.2)
Jordan	2011	359.4 (330.1 to 392.8)	241.4 (219.5 to 266.9)	81.6 (68.5 to 97.5)	29.6 (18.4 to 44.3)	6.8 (6.8 to 6.8)	815.9 (749.4 to 891.8)	548.0 (498.3 to 605.8)	185.2 (155.5 to 221.4)	67.2 (41.7 to 100.5)	15.5 (15.5 to 15.5)
Jordan	2012	337.9 (310.1 to 369.2)	220.5 (199.8 to 245.1)	74.7 (61.8 to 89.5)	28.9 (17.8 to 43.9)	13.8 (13.8 to 13.8)	767.0 (703.9 to 838.1)	500.5 (453.6 to 556.4)	169.5 (140.2 to 203.3)	65.6 (40.5 to 99.8)	31.4 (31.4 to 31.4)
Jordan	2013	310.5 (282.9 to 338.5)	197.6 (178.2 to 221.2)	68.7 (56.1 to 82.4)	28.1 (16.9 to 44.1)	16.1 (16.1 to 16.1)	704.9 (642.3 to 768.5)	448.6 (404.6 to 502.2)	156.0 (127.3 to 187.1)	63.7 (38.3 to 100.1)	36.6 (36.6 to 36.6)
Jordan	2014	285.6 (259.5 to 315.0)	181.9 (161.5 to 203.3)	64.9 (52.6 to 78.4)	25.8 (15.3 to 41.8)	13.0 (13.0 to 13.0)	648.3 (589.0 to 715.2)	413.0 (366.6 to 461.5)	147.3 (119.3 to 178.1)	58.6 (34.8 to 94.8)	29.5 (29.5 to 29.5)
Jordan	2015	246.2 (221.0 to 274.0)	159.5 (140.6 to 181.0)	61.8 (49.3 to 76.1)	21.3 (12.3 to 35.3)	3.6 (3.6 to 3.6)	559.0 (501.7 to 622.0)	362.1 (319.2 to 410.9)	140.3 (112.0 to 172.7)	48.4 (27.9 to 80.1)	8.3 (8.3 to 8.3)

		2018 US Dollars per capita					2018 PPP per capita				
Country	Year	Health spending	Government health spending	Out-of-pocket health spending	Prepaid private health spending	Development assistance for health	Health spending	Government health spending	Out-of-pocket health spending	Prepaid private health spending	Development assistance for health
Jordan	2016	224.2 (198.2 to 252.8)	147.2 (127.6 to 169.2)	58.8 (46.1 to 73.2)	13.6 (7.6 to 22.4)	4.6 (4.6 to 4.6)	508.9 (450.0 to 573.9)	334.1 (289.7 to 384.1)	133.5 (104.6 to 166.2)	30.8 (17.2 to 50.8)	10.4 (10.4 to 10.4)
Jordan	2017	234.2 (207.8 to 261.8)	152.3 (131.9 to 175.3)	58.5 (46.1 to 72.8)	14.8 (8.2 to 24.4)	8.7 (8.7 to 8.7)	531.6 (471.7 to 594.4)	345.6 (299.4 to 397.9)	132.7 (104.7 to 165.2)	33.6 (18.7 to 55.4)	19.7 (19.7 to 19.7)
Jordan	2018	234.6 (208.7 to 264.4)	153.8 (133.6 to 176.1)	58.3 (45.8 to 72.6)	14.8 (8.2 to 24.5)	7.8 (7.7 to 7.8)	532.7 (473.8 to 600.3)	349.1 (303.2 to 399.7)	132.3 (104.0 to 164.8)	33.6 (18.7 to 55.6)	17.6 (17.5 to 17.8)
Jordan	2019	236.9 (210.0 to 265.3)	155.6 (135.2 to 178.3)	58.4 (46.0 to 73.1)	15.0 (8.4 to 24.8)	7.8 (7.3 to 8.3)	537.7 (476.8 to 602.2)	353.2 (306.8 to 404.7)	132.6 (104.4 to 166.0)	34.1 (19.0 to 56.4)	17.7 (16.5 to 18.8)
Jordan	2020	239.5 (213.6 to 269.2)	157.5 (136.2 to 180.1)	58.8 (46.3 to 73.5)	15.2 (8.5 to 25.1)	8.0 (7.3 to 8.6)	543.8 (484.9 to 611.2)	357.5 (309.2 to 409.0)	133.5 (105.1 to 166.9)	34.6 (19.3 to 57.0)	18.1 (16.5 to 19.6)
Jordan	2021	242.6 (215.5 to 271.2)	159.7 (138.0 to 183.0)	59.2 (46.6 to 74.2)	15.5 (8.6 to 25.4)	8.2 (7.4 to 9.1)	550.7 (489.2 to 615.8)	362.5 (313.3 to 415.4)	134.5 (105.7 to 168.4)	35.1 (19.6 to 57.7)	18.6 (16.7 to 20.6)
Jordan	2022	245.6 (218.1 to 277.1)	161.7 (139.9 to 185.9)	59.6 (46.9 to 74.5)	15.7 (8.8 to 25.7)	8.6 (7.6 to 9.6)	557.4 (495.1 to 629.0)	367.2 (317.6 to 421.9)	135.2 (106.4 to 169.2)	35.6 (19.9 to 58.5)	19.4 (17.2 to 21.9)
Jordan	2023	248.5 (220.2 to 278.1)	163.8 (141.4 to 187.8)	59.8 (47.1 to 74.8)	15.9 (8.9 to 26.0)	8.9 (7.7 to 10.2)	564.0 (499.9 to 631.4)	371.9 (321.0 to 426.4)	135.8 (106.8 to 169.9)	36.1 (20.2 to 59.1)	20.3 (17.6 to 23.1)
Jordan	2024	251.1 (223.3 to 283.3)	165.7 (143.0 to 190.9)	60.0 (47.0 to 75.0)	16.1 (9.0 to 26.3)	9.4 (8.1 to 10.9)	570.1 (507.0 to 643.2)	376.2 (324.5 to 433.4)	136.1 (106.8 to 170.3)	36.5 (20.4 to 59.8)	21.3 (18.4 to 24.8)
Jordan	2025	254.2 (225.6 to 284.4)	168.0 (145.0 to 193.5)	60.2 (47.4 to 75.1)	16.3 (9.1 to 26.7)	9.7 (8.3 to 11.4)	577.0 (512.0 to 645.7)	381.3 (329.1 to 439.2)	136.6 (107.6 to 170.4)	37.0 (20.7 to 60.6)	22.1 (18.8 to 26.0)
Jordan	2026	257.0 (227.6 to 289.9)	170.1 (146.9 to 196.2)	60.4 (47.5 to 75.5)	16.5 (9.2 to 27.0)	10.0 (8.5 to 11.9)	583.4 (516.6 to 658.2)	386.1 (333.4 to 445.3)	137.1 (107.9 to 171.3)	37.4 (20.9 to 61.4)	22.8 (19.2 to 27.0)
Jordan	2027	260.6 (230.6 to 292.5)	172.9 (149.2 to 199.8)	60.7 (47.6 to 75.8)	16.7 (9.4 to 27.4)	10.4 (8.7 to 12.5)	591.7 (523.4 to 664.0)	392.5 (338.6 to 453.6)	137.7 (108.1 to 172.0)	38.0 (21.3 to 62.3)	23.5 (19.7 to 28.3)
Jordan	2028	264.5 (232.4 to 298.9)	175.9 (151.4 to 203.9)	61.0 (47.8 to 76.4)	17.0 (9.5 to 27.8)	10.7 (8.8 to 13.0)	600.3 (527.5 to 678.5)	399.2 (343.6 to 462.9)	138.4 (108.6 to 173.4)	38.5 (21.6 to 63.2)	24.2 (20.0 to 29.5)
Jordan	2029	268.6 (237.3 to 302.5)	179.1 (153.8 to 207.7)	61.3 (47.9 to 77.0)	17.2 (9.7 to 28.3)	11.0 (8.9 to 13.6)	609.8 (538.7 to 686.7)	406.6 (349.1 to 471.5)	139.2 (108.9 to 174.9)	39.1 (21.9 to 64.2)	24.9 (20.2 to 30.9)
Jordan	2030	272.5 (240.2 to 309.1)	182.2 (155.7 to 212.3)	61.6 (48.0 to 77.4)	17.5 (9.8 to 28.7)	11.2 (8.9 to 14.0)	618.7 (545.4 to 701.7)	413.6 (353.5 to 481.9)	139.9 (109.1 to 175.7)	39.7 (22.2 to 65.2)	25.5 (20.3 to 31.9)
Jordan	2031	276.0 (242.6 to 312.1)	184.9 (157.3 to 216.0)	61.9 (48.1 to 78.1)	17.7 (9.9 to 29.1)	11.5 (9.0 to 14.6)	626.6 (550.7 to 708.5)	419.7 (357.1 to 490.4)	140.6 (109.3 to 177.2)	40.2 (22.5 to 66.0)	26.1 (20.4 to 33.2)
Jordan	2032	279.6 (245.3 to 317.9)	187.7 (158.7 to 219.6)	62.2 (48.3 to 78.5)	18.0 (10.1 to 29.5)	11.8 (9.0 to 15.2)	634.8 (556.9 to 721.7)	426.1 (360.2 to 498.5)	141.3 (109.7 to 178.2)	40.8 (22.8 to 66.9)	26.7 (20.3 to 34.5)
Jordan	2033	283.2 (246.8 to 323.3)	190.5 (160.1 to 223.3)	62.5 (48.5 to 78.8)	18.2 (10.2 to 29.9)	12.0 (9.0 to 15.9)	643.0 (560.3 to 734.0)	432.4 (363.4 to 507.0)	142.0 (110.1 to 178.8)	41.3 (23.2 to 67.8)	27.3 (20.5 to 36.1)
Jordan	2034	286.8 (250.0 to 328.4)	193.2 (161.4 to 227.9)	62.8 (48.7 to 79.0)	18.4 (10.3 to 30.2)	12.3 (9.1 to 16.6)	651.0 (567.6 to 745.5)	438.6 (366.4 to 517.4)	142.6 (110.5 to 179.3)	41.9 (23.4 to 68.6)	28.0 (20.6 to 37.8)
Jordan	2035	290.3 (250.7 to 332.6)	196.0 (163.6 to 231.6)	63.1 (48.8 to 79.2)	18.7 (10.4 to 30.6)	12.6 (9.1 to 17.2)	659.1 (569.1 to 755.0)	444.9 (371.5 to 525.7)	143.2 (110.7 to 179.9)	42.4 (23.7 to 69.5)	28.7 (20.6 to 38.9)
Jordan	2036	293.9 (252.8 to 337.9)	198.7 (165.5 to 235.7)	63.3 (48.8 to 79.5)	18.9 (10.6 to 31.0)	12.9 (9.1 to 17.8)	667.3 (573.9 to 767.1)	451.1 (375.7 to 535.1)	143.8 (110.9 to 180.4)	43.0 (24.0 to 70.4)	29.3 (20.7 to 40.4)

		2018 US Dollars per capita					2018 PPP per capita				
Country	Year	Health spending	Government health spending	Out-of-pocket health spending	Prepaid private health spending	Development assistance for health	Health spending	Government health spending	Out-of-pocket health spending	Prepaid private health spending	Development assistance for health
Jordan	2037	297.0 (255.4 to 343.1)	201.1 (166.1 to 238.9)	63.5 (48.8 to 79.7)	19.2 (10.7 to 31.4)	13.2 (9.1 to 18.7)	674.3 (579.8 to 778.9)	456.6 (377.0 to 542.4)	144.2 (110.9 to 180.8)	43.5 (24.3 to 71.3)	30.0 (20.8 to 42.5)
Jordan	2038	300.4 (257.4 to 347.2)	203.7 (167.0 to 243.6)	63.8 (48.9 to 80.0)	19.4 (10.9 to 31.8)	13.5 (9.2 to 19.5)	681.9 (584.2 to 788.3)	462.4 (379.2 to 552.9)	144.8 (111.0 to 181.7)	44.0 (24.6 to 72.2)	30.7 (20.8 to 44.2)
Jordan	2039	303.5 (259.0 to 352.0)	206.1 (167.9 to 247.4)	64.0 (48.9 to 80.2)	19.6 (11.0 to 32.2)	13.8 (9.3 to 20.4)	689.1 (588.1 to 799.1)	467.8 (381.2 to 561.6)	145.3 (111.1 to 182.1)	44.6 (25.0 to 73.1)	31.4 (21.2 to 46.3)
Jordan	2040	306.7 (260.5 to 356.7)	208.4 (168.5 to 251.2)	64.2 (49.0 to 80.9)	19.9 (11.1 to 32.7)	14.2 (9.4 to 21.5)	696.2 (591.4 to 809.7)	473.2 (382.4 to 570.2)	145.7 (111.3 to 183.5)	45.1 (25.3 to 74.1)	32.2 (21.3 to 48.8)
Jordan	2041	309.6 (262.6 to 360.2)	210.5 (169.1 to 255.3)	64.4 (49.2 to 81.4)	20.1 (11.3 to 33.0)	14.6 (9.5 to 22.5)	702.8 (596.1 to 817.6)	477.9 (383.9 to 579.6)	146.2 (111.7 to 184.9)	45.6 (25.6 to 75.0)	33.1 (21.6 to 51.0)
Jordan	2042	312.6 (263.7 to 366.6)	212.7 (169.8 to 259.6)	64.6 (49.4 to 81.9)	20.3 (11.4 to 33.4)	15.0 (9.6 to 23.6)	709.5 (598.5 to 832.3)	482.8 (385.4 to 589.4)	146.7 (112.1 to 186.0)	46.1 (25.9 to 75.9)	34.0 (21.7 to 53.7)
Jordan	2043	315.4 (265.7 to 369.4)	214.7 (170.3 to 263.0)	64.8 (49.4 to 82.3)	20.5 (11.5 to 33.9)	15.4 (9.6 to 24.9)	715.9 (603.2 to 838.6)	487.3 (386.6 to 597.1)	147.1 (112.1 to 186.8)	46.6 (26.1 to 76.9)	34.9 (21.8 to 56.5)
Jordan	2044	318.3 (267.2 to 375.2)	216.7 (171.0 to 266.7)	65.0 (49.4 to 82.6)	20.8 (11.6 to 34.3)	15.8 (9.8 to 25.6)	722.5 (606.6 to 851.8)	491.9 (388.3 to 605.4)	147.5 (112.2 to 187.6)	47.2 (26.4 to 77.9)	35.9 (22.3 to 58.0)
Jordan	2045	321.2 (267.6 to 379.9)	218.8 (171.8 to 270.0)	65.1 (49.5 to 82.9)	21.0 (11.7 to 34.7)	16.3 (10.0 to 26.8)	729.1 (607.5 to 862.5)	496.6 (390.1 to 613.0)	147.8 (112.4 to 188.2)	47.7 (26.6 to 78.8)	37.0 (22.8 to 60.7)
Jordan	2046	324.4 (269.3 to 383.5)	221.1 (172.7 to 273.7)	65.4 (49.7 to 83.2)	21.2 (11.9 to 35.1)	16.8 (10.1 to 28.3)	736.5 (611.5 to 870.7)	501.9 (392.2 to 621.4)	148.4 (112.8 to 188.8)	48.2 (26.9 to 79.6)	38.0 (23.0 to 64.2)
Jordan	2047	327.6 (270.7 to 388.1)	223.3 (173.3 to 277.6)	65.5 (49.8 to 83.4)	21.5 (12.0 to 35.4)	17.3 (10.4 to 30.6)	743.7 (614.6 to 881.0)	506.8 (393.4 to 630.2)	148.8 (113.0 to 189.2)	48.8 (27.2 to 80.3)	39.3 (23.5 to 69.5)
Jordan	2048	330.9 (272.4 to 394.9)	225.6 (173.9 to 281.1)	65.7 (49.9 to 83.5)	21.7 (12.1 to 35.8)	17.9 (10.6 to 31.8)	751.2 (618.4 to 896.5)	512.0 (394.9 to 638.2)	149.2 (113.3 to 189.6)	49.3 (27.4 to 81.3)	40.6 (24.0 to 72.3)
Jordan	2049	334.2 (275.4 to 396.1)	227.8 (174.5 to 283.4)	65.9 (50.0 to 83.7)	22.0 (12.2 to 36.2)	18.5 (10.6 to 33.4)	758.6 (625.3 to 899.2)	517.1 (396.1 to 643.4)	149.6 (113.6 to 190.0)	49.9 (27.7 to 82.2)	42.0 (24.1 to 75.8)
Jordan	2050	337.6 (276.3 to 401.1)	230.1 (175.3 to 286.6)	66.1 (50.2 to 83.9)	22.2 (12.3 to 36.6)	19.1 (10.8 to 34.7)	766.4 (627.3 to 910.5)	522.4 (398.0 to 650.7)	150.1 (113.9 to 190.5)	50.4 (28.0 to 83.2)	43.5 (24.6 to 78.9)
Kazakhstan	1995	178.7 (154.2 to 205.9)	96.4 (79.6 to 115.1)	79.4 (61.9 to 100.0)	2.6 (1.2 to 4.7)	0.4 (0.4 to 0.4)	525.3 (453.3 to 605.1)	283.3 (233.8 to 338.3)	233.4 (181.8 to 293.8)	7.6 (3.6 to 14.0)	1.1 (1.1 to 1.1)
Kazakhstan	1996	176.0 (153.5 to 200.9)	94.7 (78.7 to 111.0)	78.3 (62.3 to 97.5)	2.5 (1.2 to 4.7)	0.5 (0.5 to 0.5)	517.1 (451.0 to 590.5)	278.2 (231.4 to 326.3)	230.0 (183.1 to 286.7)	7.4 (3.4 to 13.8)	1.5 (1.5 to 1.5)
Kazakhstan	1997	174.3 (153.4 to 198.9)	94.2 (78.5 to 110.3)	77.2 (62.9 to 95.5)	2.4 (1.1 to 4.5)	0.5 (0.5 to 0.5)	512.3 (450.9 to 584.4)	276.8 (230.6 to 324.1)	227.0 (184.8 to 280.7)	7.1 (3.3 to 13.3)	1.4 (1.4 to 1.4)
Kazakhstan	1998	166.2 (146.7 to 188.6)	88.5 (74.7 to 102.8)	74.6 (60.7 to 91.1)	2.2 (1.0 to 4.1)	0.9 (0.9 to 0.9)	488.3 (431.1 to 554.1)	260.1 (219.6 to 302.2)	219.2 (178.4 to 267.8)	6.3 (2.9 to 12.1)	2.7 (2.7 to 2.7)
Kazakhstan	1999	156.6 (138.7 to 178.0)	81.7 (69.6 to 95.1)	71.6 (58.7 to 86.8)	1.7 (0.8 to 3.2)	1.5 (1.5 to 1.5)	460.0 (407.4 to 523.1)	240.0 (204.7 to 279.6)	210.5 (172.4 to 254.9)	5.1 (2.3 to 9.5)	4.4 (4.4 to 4.4)
Kazakhstan	2000	155.9 (138.2 to 175.9)	82.4 (70.6 to 95.5)	70.9 (58.1 to 86.5)	1.3 (0.6 to 2.4)	1.5 (1.5 to 1.5)	458.2 (406.1 to 516.9)	242.0 (207.4 to 280.8)	208.2 (170.7 to 254.3)	3.7 (1.7 to 7.1)	4.3 (4.3 to 4.3)
Kazakhstan	2001	160.4 (142.9 to 181.5)	87.4 (74.8 to 101.2)	70.4 (57.8 to 85.9)	1.1 (0.5 to 2.2)	1.6 (1.6 to 1.6)	471.5 (419.9 to 533.3)	256.7 (219.9 to 297.3)	206.8 (169.8 to 252.3)	3.4 (1.6 to 6.4)	4.6 (4.6 to 4.6)

Country	Year	2018 US Dollars per capita					2018 PPP per capita				
		Health spending	Government health spending	Out-of-pocket health spending	Prepaid private health spending	Development assistance for health	Health spending	Government health spending	Out-of-pocket health spending	Prepaid private health spending	Development assistance for health
Kazakhstan	2002	170.9 (152.9 to 193.4)	94.4 (81.1 to 109.0)	74.6 (60.9 to 91.5)	1.2 (0.5 to 2.1)	0.7 (0.7 to 0.7)	502.1 (449.3 to 568.3)	277.4 (238.4 to 320.3)	219.2 (179.1 to 268.9)	3.4 (1.5 to 6.3)	2.1 (2.1 to 2.1)
Kazakhstan	2003	188.2 (169.1 to 212.5)	106.5 (92.5 to 121.9)	79.1 (64.9 to 97.7)	1.2 (0.6 to 2.4)	1.4 (1.4 to 1.4)	553.0 (496.9 to 624.4)	313.0 (271.8 to 358.4)	232.3 (190.7 to 287.0)	3.7 (1.7 to 7.0)	4.0 (4.0 to 4.0)
Kazakhstan	2004	206.1 (185.9 to 231.8)	122.6 (107.2 to 139.9)	81.2 (66.8 to 99.5)	1.4 (0.6 to 2.7)	1.0 (1.0 to 1.0)	605.8 (546.3 to 681.1)	360.2 (315.0 to 411.2)	238.5 (196.2 to 292.3)	4.1 (1.9 to 7.9)	3.0 (3.0 to 3.0)
Kazakhstan	2005	217.7 (195.4 to 243.6)	137.7 (120.5 to 156.7)	77.7 (64.5 to 95.0)	1.5 (0.7 to 3.0)	0.8 (0.8 to 0.8)	639.9 (574.1 to 715.8)	404.6 (354.0 to 460.3)	228.4 (189.6 to 279.3)	4.5 (2.0 to 8.8)	2.3 (2.3 to 2.3)
Kazakhstan	2006	222.6 (200.2 to 247.5)	147.8 (129.7 to 165.8)	71.8 (59.5 to 87.9)	1.7 (0.8 to 3.3)	1.2 (1.2 to 1.2)	654.1 (588.4 to 727.4)	434.4 (381.1 to 487.2)	211.1 (174.8 to 258.3)	5.1 (2.4 to 9.7)	3.6 (3.6 to 3.6)
Kazakhstan	2007	219.8 (198.3 to 243.3)	151.3 (133.2 to 169.8)	65.8 (54.6 to 80.1)	2.0 (1.0 to 3.8)	0.8 (0.8 to 0.8)	645.9 (582.6 to 715.1)	444.5 (391.3 to 498.9)	193.3 (160.5 to 235.3)	5.8 (2.8 to 11.1)	2.3 (2.3 to 2.3)
Kazakhstan	2008	227.1 (205.4 to 251.2)	161.4 (143.3 to 181.2)	61.6 (50.9 to 74.9)	2.6 (1.3 to 4.8)	1.5 (1.5 to 1.5)	667.4 (603.6 to 738.2)	474.3 (421.1 to 532.5)	181.1 (149.7 to 220.2)	7.6 (3.7 to 14.2)	4.4 (4.4 to 4.4)
Kazakhstan	2009	225.3 (203.8 to 248.8)	159.3 (141.5 to 178.5)	59.4 (48.4 to 72.9)	3.5 (1.7 to 6.7)	3.1 (3.1 to 3.1)	662.1 (598.9 to 731.2)	468.2 (415.7 to 524.4)	174.6 (142.3 to 214.1)	10.3 (5.1 to 19.7)	9.0 (9.0 to 9.0)
Kazakhstan	2010	225.0 (202.9 to 247.2)	155.7 (138.3 to 175.1)	59.9 (48.7 to 74.0)	5.9 (2.9 to 11.0)	3.4 (3.4 to 3.4)	661.2 (596.3 to 726.5)	457.6 (406.3 to 514.6)	176.2 (143.2 to 217.5)	17.4 (8.6 to 32.2)	10.1 (10.1 to 10.1)
Kazakhstan	2011	236.2 (214.4 to 260.0)	162.2 (143.9 to 181.1)	62.7 (50.4 to 76.4)	8.8 (4.4 to 16.4)	2.4 (2.4 to 2.4)	693.9 (630.2 to 764.1)	476.5 (422.9 to 532.2)	184.3 (148.1 to 224.4)	26.0 (12.8 to 48.1)	7.2 (7.2 to 7.2)
Kazakhstan	2012	247.4 (223.0 to 273.1)	166.3 (146.7 to 185.8)	66.9 (53.4 to 82.3)	11.5 (5.5 to 21.0)	2.7 (2.7 to 2.7)	727.1 (655.4 to 802.5)	488.7 (431.0 to 546.1)	196.7 (157.0 to 241.8)	33.9 (16.3 to 61.7)	7.9 (7.9 to 7.9)
Kazakhstan	2013	251.5 (226.2 to 279.5)	168.2 (147.3 to 188.0)	68.1 (54.5 to 84.7)	13.5 (6.5 to 24.9)	1.7 (1.7 to 1.7)	739.0 (664.7 to 821.3)	494.3 (432.9 to 552.6)	200.2 (160.1 to 249.0)	39.5 (19.1 to 73.1)	4.9 (4.9 to 4.9)
Kazakhstan	2014	261.0 (233.7 to 290.4)	171.6 (149.9 to 194.2)	73.2 (57.8 to 90.4)	15.0 (7.4 to 27.5)	1.2 (1.2 to 1.2)	766.9 (686.8 to 853.4)	504.2 (440.4 to 570.6)	215.0 (169.9 to 265.7)	44.1 (21.8 to 80.9)	3.6 (3.6 to 3.6)
Kazakhstan	2015	275.9 (244.8 to 309.2)	174.7 (150.4 to 200.2)	84.2 (66.6 to 105.5)	15.9 (7.5 to 29.4)	1.1 (1.1 to 1.1)	810.7 (719.4 to 908.6)	513.4 (442.0 to 588.4)	247.5 (195.8 to 310.1)	46.7 (22.1 to 86.3)	3.1 (3.1 to 3.1)
Kazakhstan	2016	295.4 (259.5 to 334.6)	181.0 (153.5 to 208.8)	96.3 (75.5 to 121.3)	15.9 (7.7 to 30.0)	2.2 (2.2 to 2.2)	868.1 (762.6 to 983.3)	531.8 (451.1 to 613.7)	283.0 (222.0 to 356.4)	46.7 (22.5 to 88.1)	6.6 (6.6 to 6.6)
Kazakhstan	2017	326.2 (286.1 to 368.7)	207.3 (176.1 to 239.3)	100.6 (78.9 to 126.5)	16.6 (8.0 to 31.3)	1.8 (1.8 to 1.8)	958.5 (840.8 to 1083.4)	609.1 (517.4 to 703.2)	295.6 (232.0 to 371.9)	48.6 (23.5 to 92.1)	5.2 (5.2 to 5.2)
Kazakhstan	2018	333.2 (293.1 to 377.7)	211.5 (179.0 to 245.1)	103.3 (80.9 to 129.3)	16.8 (8.1 to 31.8)	1.6 (1.6 to 1.6)	979.1 (861.2 to 1109.9)	621.5 (526.1 to 720.1)	303.4 (237.8 to 379.8)	49.5 (23.9 to 93.6)	4.7 (4.7 to 4.7)
Kazakhstan	2019	340.9 (298.8 to 388.0)	216.3 (182.7 to 251.0)	105.9 (83.2 to 133.5)	17.1 (8.3 to 32.4)	1.6 (1.5 to 1.7)	1001.9 (878.1 to 1140.2)	635.7 (536.8 to 737.4)	311.1 (244.4 to 392.3)	50.3 (24.3 to 95.3)	4.8 (4.5 to 5.0)
Kazakhstan	2020	348.1 (304.7 to 396.3)	220.6 (185.8 to 255.8)	108.5 (85.3 to 136.9)	17.4 (8.4 to 32.9)	1.7 (1.5 to 1.8)	1023.0 (895.5 to 1164.4)	648.2 (546.1 to 751.7)	318.7 (250.7 to 402.4)	51.1 (24.7 to 96.6)	4.9 (4.5 to 5.4)
Kazakhstan	2021	356.2 (311.0 to 406.9)	225.6 (189.2 to 261.7)	111.2 (87.0 to 140.7)	17.7 (8.5 to 33.4)	1.7 (1.6 to 1.9)	1046.8 (913.9 to 1195.6)	663.0 (556.0 to 768.9)	326.8 (255.6 to 413.4)	51.9 (25.1 to 98.1)	5.1 (4.6 to 5.7)
Kazakhstan	2022	362.9 (316.0 to 414.0)	229.4 (191.1 to 266.7)	113.7 (89.0 to 144.0)	17.9 (8.6 to 33.7)	1.8 (1.6 to 2.1)	1066.3 (928.5 to 1216.5)	674.2 (561.5 to 783.8)	334.2 (261.5 to 423.0)	52.5 (25.3 to 99.0)	5.4 (4.8 to 6.1)

Country	Year	2018 US Dollars per capita					2018 PPP per capita				
		Health spending	Government health spending	Out-of-pocket health spending	Prepaid private health spending	Development assistance for health	Health spending	Government health spending	Out-of-pocket health spending	Prepaid private health spending	Development assistance for health
Kazakhstan	2023	368.8 (320.7 to 419.6)	232.7 (193.5 to 271.1)	116.1 (90.1 to 147.0)	18.0 (8.7 to 33.9)	2.0 (1.7 to 2.3)	1083.8 (942.5 to 1233.1)	683.8 (568.7 to 796.8)	341.3 (264.9 to 432.1)	53.0 (25.6 to 99.7)	5.7 (4.9 to 6.7)
Kazakhstan	2024	375.4 (322.0 to 428.2)	236.4 (197.1 to 275.1)	118.7 (92.2 to 151.1)	18.2 (8.8 to 34.3)	2.0 (1.7 to 2.4)	1103.0 (946.3 to 1258.2)	694.7 (579.2 to 808.4)	348.8 (271.0 to 444.1)	53.5 (25.9 to 100.8)	6.0 (5.0 to 7.0)
Kazakhstan	2025	382.8 (329.4 to 435.5)	240.8 (200.2 to 280.7)	121.4 (94.3 to 153.3)	18.4 (8.9 to 34.7)	2.1 (1.8 to 2.6)	1124.8 (967.9 to 1279.9)	707.7 (588.3 to 824.8)	356.6 (277.0 to 450.5)	54.2 (26.2 to 101.9)	6.3 (5.2 to 7.5)
Kazakhstan	2026	391.0 (338.2 to 445.5)	245.9 (203.2 to 287.9)	124.2 (95.8 to 157.3)	18.7 (9.0 to 35.1)	2.2 (1.8 to 2.7)	1149.0 (993.9 to 1309.1)	722.7 (597.1 to 846.0)	364.9 (281.6 to 462.3)	54.9 (26.4 to 103.1)	6.5 (5.3 to 8.0)
Kazakhstan	2027	400.6 (348.1 to 457.1)	252.1 (207.8 to 295.1)	127.3 (98.1 to 161.4)	19.0 (9.1 to 35.6)	2.3 (1.8 to 2.9)	1177.2 (1022.8 to 1343.2)	740.8 (610.6 to 867.2)	374.0 (288.2 to 474.2)	55.7 (26.8 to 104.5)	6.7 (5.2 to 8.4)
Kazakhstan	2028	411.6 (357.4 to 472.4)	259.4 (213.9 to 304.3)	130.6 (99.7 to 165.5)	19.3 (9.3 to 36.2)	2.3 (0.0 to 3.0)	1209.6 (1050.2 to 1388.3)	762.2 (628.7 to 894.1)	383.9 (292.9 to 486.2)	56.8 (27.3 to 106.4)	6.7 (0.0 to 8.7)
Kazakhstan	2029	422.9 (366.0 to 484.3)	266.7 (219.5 to 313.0)	134.1 (101.4 to 170.8)	19.7 (9.5 to 37.0)	2.3 (0.0 to 3.1)	1242.7 (1075.4 to 1423.2)	783.8 (645.2 to 919.8)	394.2 (297.8 to 502.0)	58.0 (27.9 to 108.6)	6.7 (0.0 to 9.0)
Kazakhstan	2030	434.3 (372.3 to 501.4)	274.1 (224.3 to 322.5)	137.7 (103.2 to 176.4)	20.2 (9.7 to 37.8)	2.2 (0.0 to 3.1)	1276.2 (1093.9 to 1473.4)	805.6 (659.0 to 947.6)	404.7 (303.2 to 518.5)	59.4 (28.6 to 111.1)	6.4 (0.0 to 9.2)
Kazakhstan	2031	446.3 (381.9 to 515.8)	282.1 (231.5 to 332.6)	141.4 (105.1 to 181.5)	20.7 (10.0 to 38.8)	2.1 (0.0 to 3.2)	1311.6 (1122.2 to 1515.7)	828.9 (680.2 to 977.4)	415.6 (308.9 to 533.3)	60.9 (29.2 to 114.2)	6.1 (0.0 to 9.5)
Kazakhstan	2032	458.9 (392.5 to 529.7)	290.4 (238.2 to 343.4)	145.2 (107.2 to 186.6)	21.3 (10.2 to 39.9)	1.9 (0.0 to 3.3)	1348.4 (1153.3 to 1556.7)	853.3 (699.9 to 1009.0)	426.8 (315.0 to 548.4)	62.6 (30.1 to 117.3)	5.6 (0.0 to 9.6)
Kazakhstan	2033	471.5 (403.0 to 544.5)	298.7 (244.1 to 353.6)	149.1 (108.8 to 192.5)	21.9 (10.5 to 41.0)	1.7 (0.0 to 3.3)	1385.6 (1184.1 to 1600.0)	877.9 (717.4 to 1039.2)	438.2 (319.6 to 565.6)	64.4 (31.0 to 120.5)	5.1 (0.0 to 9.6)
Kazakhstan	2034	484.3 (412.9 to 559.8)	307.2 (250.7 to 365.2)	153.0 (110.8 to 197.9)	22.6 (10.8 to 42.1)	1.5 (0.0 to 3.3)	1423.1 (1213.3 to 1645.0)	902.7 (736.6 to 1073.3)	449.7 (325.7 to 581.6)	66.3 (31.8 to 123.8)	4.4 (0.0 to 9.8)
Kazakhstan	2035	497.1 (423.5 to 576.5)	315.6 (257.4 to 375.3)	156.9 (113.0 to 204.4)	23.2 (11.1 to 43.2)	1.4 (0.0 to 3.4)	1460.7 (1244.6 to 1694.0)	927.5 (756.4 to 1102.9)	461.1 (332.0 to 600.6)	68.1 (32.7 to 127.0)	4.0 (0.0 to 10.0)
Kazakhstan	2036	510.1 (432.5 to 589.9)	324.3 (264.2 to 386.3)	160.8 (115.1 to 210.2)	23.8 (11.4 to 44.3)	1.2 (0.0 to 3.5)	1499.1 (1270.8 to 1733.5)	953.1 (776.4 to 1135.3)	472.5 (338.3 to 617.8)	69.9 (33.6 to 130.1)	3.5 (0.0 to 10.4)
Kazakhstan	2037	523.1 (443.3 to 605.4)	333.0 (269.5 to 396.5)	164.6 (117.6 to 216.2)	24.4 (11.7 to 45.3)	1.1 (0.0 to 3.6)	1537.3 (1302.7 to 1779.2)	978.6 (792.0 to 1165.2)	483.8 (345.5 to 635.3)	71.7 (34.4 to 133.2)	3.2 (0.0 to 10.6)
Kazakhstan	2038	536.3 (453.7 to 623.6)	341.8 (276.0 to 408.4)	168.5 (119.9 to 221.2)	25.0 (12.0 to 46.4)	1.0 (0.0 to 3.7)	1576.0 (1333.3 to 1832.5)	1004.5 (810.9 to 1200.2)	495.0 (352.4 to 649.9)	73.5 (35.3 to 136.3)	3.0 (0.0 to 10.8)
Kazakhstan	2039	549.1 (467.0 to 642.5)	350.4 (281.4 to 418.3)	172.2 (121.9 to 226.4)	25.6 (12.3 to 47.4)	0.9 (0.0 to 3.7)	1613.7 (1372.3 to 1888.1)	1029.6 (827.0 to 1229.3)	506.1 (358.4 to 665.2)	75.3 (36.1 to 139.4)	2.7 (0.0 to 11.0)
Kazakhstan	2040	561.9 (477.4 to 657.6)	358.8 (287.9 to 429.0)	176.0 (124.1 to 231.7)	26.2 (12.6 to 48.5)	0.9 (0.0 to 3.9)	1651.2 (1402.9 to 1932.3)	1054.4 (846.1 to 1260.5)	517.2 (364.7 to 680.8)	77.1 (37.0 to 142.7)	2.5 (0.0 to 11.5)
Kazakhstan	2041	574.5 (484.8 to 676.0)	367.2 (295.0 to 439.6)	179.7 (126.3 to 237.0)	26.9 (12.9 to 49.6)	0.8 (0.0 to 4.0)	1688.3 (1424.6 to 1986.6)	1079.0 (866.8 to 1291.9)	528.0 (371.3 to 696.5)	78.9 (37.9 to 145.9)	2.4 (0.0 to 11.8)
Kazakhstan	2042	587.3 (494.7 to 687.5)	375.7 (302.4 to 449.5)	183.4 (128.7 to 242.7)	27.5 (13.2 to 50.9)	0.8 (0.0 to 4.1)	1726.0 (1453.7 to 2020.4)	1104.1 (888.8 to 1320.8)	538.8 (378.3 to 713.3)	80.8 (38.8 to 149.4)	2.3 (0.0 to 12.1)
Kazakhstan	2043	599.8 (506.5 to 704.3)	384.0 (308.3 to 461.3)	187.0 (131.2 to 248.1)	28.1 (13.5 to 52.2)	0.7 (0.0 to 4.1)	1762.7 (1488.5 to 2069.7)	1128.5 (905.8 to 1355.7)	549.4 (385.5 to 729.2)	82.6 (39.6 to 153.3)	2.1 (0.0 to 12.1)

		2018 US Dollars per capita					2018 PPP per capita				
Country	Year	Health spending	Government health spending	Out-of-pocket health spending	Prepaid private health spending	Development assistance for health	Health spending	Government health spending	Out-of-pocket health spending	Prepaid private health spending	Development assistance for health
Kazakhstan	2044	612.5 (514.1 to 717.7)	392.5 (312.8 to 471.8)	190.6 (133.6 to 252.7)	28.8 (13.8 to 53.5)	0.6 (0.0 to 4.2)	1799.8 (1510.6 to 2109.0)	1153.3 (919.2 to 1386.4)	560.0 (392.5 to 742.7)	84.5 (40.5 to 157.2)	1.9 (0.0 to 12.3)
Kazakhstan	2045	624.9 (525.9 to 734.6)	400.7 (319.8 to 482.4)	194.1 (136.0 to 257.5)	29.4 (14.1 to 54.8)	0.6 (0.0 to 4.3)	1836.3 (1545.5 to 2158.8)	1177.6 (939.7 to 1417.5)	570.4 (399.6 to 756.7)	86.4 (41.5 to 161.0)	1.8 (0.0 to 12.8)
Kazakhstan	2046	637.5 (535.4 to 747.6)	409.2 (324.8 to 492.8)	197.6 (138.2 to 262.5)	30.0 (14.4 to 55.9)	0.6 (0.0 to 4.4)	1873.4 (1573.4 to 2197.0)	1202.6 (954.5 to 1448.0)	580.7 (406.1 to 771.4)	88.3 (42.5 to 164.3)	1.8 (0.0 to 13.0)
Kazakhstan	2047	649.8 (544.8 to 760.1)	417.5 (329.6 to 503.7)	201.0 (140.3 to 267.3)	30.7 (14.7 to 57.1)	0.6 (0.0 to 4.4)	1909.6 (1601.1 to 2233.7)	1227.0 (968.7 to 1480.1)	590.7 (412.4 to 785.5)	90.1 (43.3 to 167.7)	1.7 (0.0 to 13.0)
Kazakhstan	2048	662.3 (554.2 to 781.6)	425.9 (332.9 to 515.0)	204.5 (143.1 to 271.7)	31.3 (15.0 to 58.2)	0.6 (0.0 to 4.7)	1946.1 (1628.6 to 2296.8)	1251.5 (978.2 to 1513.5)	600.8 (420.4 to 798.3)	92.0 (44.2 to 171.0)	1.8 (0.0 to 13.8)
Kazakhstan	2049	674.9 (559.2 to 789.3)	434.5 (338.7 to 527.4)	207.9 (145.3 to 276.0)	31.9 (15.3 to 59.3)	0.6 (0.0 to 4.9)	1983.2 (1643.4 to 2319.3)	1276.7 (995.2 to 1549.8)	610.9 (427.1 to 811.1)	93.8 (45.0 to 174.3)	1.8 (0.0 to 14.5)
Kazakhstan	2050	687.9 (569.8 to 806.2)	443.4 (344.7 to 541.0)	211.4 (148.3 to 280.5)	32.5 (15.6 to 60.6)	0.6 (0.0 to 5.1)	2021.3 (1674.3 to 2369.2)	1303.0 (1013.0 to 1589.9)	621.1 (435.8 to 824.1)	95.5 (45.8 to 178.1)	1.7 (0.0 to 14.8)
Kenya	1995	59.9 (49.1 to 72.7)	21.1 (16.1 to 27.5)	27.9 (20.4 to 37.6)	7.6 (3.6 to 13.9)	3.3 (3.3 to 3.3)	122.6 (100.7 to 149.0)	43.2 (33.0 to 56.3)	57.2 (41.8 to 77.1)	15.5 (7.4 to 28.4)	6.8 (6.8 to 6.8)
Kenya	1996	60.4 (50.0 to 73.2)	20.7 (15.7 to 26.8)	27.7 (19.9 to 37.6)	7.5 (3.5 to 13.6)	4.6 (4.6 to 4.6)	123.8 (102.4 to 149.8)	42.4 (32.1 to 54.8)	56.7 (40.9 to 77.0)	15.3 (7.3 to 27.8)	9.4 (9.4 to 9.4)
Kenya	1997	57.7 (47.8 to 70.2)	20.2 (15.4 to 26.1)	27.5 (19.8 to 37.4)	7.4 (3.5 to 13.5)	2.7 (2.7 to 2.7)	118.2 (97.8 to 143.8)	41.3 (31.5 to 53.4)	56.3 (40.5 to 76.6)	15.2 (7.2 to 27.8)	5.5 (5.5 to 5.5)
Kenya	1998	59.3 (49.1 to 71.6)	20.2 (15.5 to 26.0)	27.7 (19.9 to 37.9)	7.5 (3.5 to 13.5)	4.0 (4.0 to 4.0)	121.6 (100.5 to 146.6)	41.3 (31.7 to 53.3)	56.8 (40.8 to 77.7)	15.3 (7.1 to 27.7)	8.2 (8.2 to 8.2)
Kenya	1999	58.3 (48.1 to 70.4)	19.7 (15.0 to 25.2)	28.0 (19.9 to 38.2)	7.5 (3.5 to 13.9)	3.1 (3.1 to 3.1)	119.3 (98.5 to 144.1)	40.3 (30.7 to 51.6)	57.3 (40.7 to 78.2)	15.3 (7.3 to 28.4)	6.4 (6.4 to 6.4)
Kenya	2000	58.1 (48.2 to 69.9)	18.6 (14.3 to 23.9)	28.2 (19.9 to 38.5)	7.3 (3.6 to 13.1)	4.1 (4.1 to 4.1)	119.0 (98.7 to 143.3)	38.0 (29.3 to 49.0)	57.8 (40.8 to 79.0)	14.9 (7.4 to 26.9)	8.3 (8.3 to 8.3)
Kenya	2001	59.6 (49.3 to 71.8)	18.7 (14.4 to 24.1)	28.9 (20.6 to 39.6)	7.4 (3.6 to 13.2)	4.5 (4.5 to 4.5)	122.0 (101.0 to 147.1)	38.3 (29.4 to 49.3)	59.2 (42.1 to 81.1)	15.2 (7.3 to 27.1)	9.3 (9.3 to 9.3)
Kenya	2002	59.3 (48.9 to 71.9)	18.7 (14.5 to 24.1)	29.1 (21.0 to 40.1)	7.4 (3.5 to 13.2)	4.2 (4.2 to 4.2)	121.5 (100.1 to 147.2)	38.4 (29.8 to 49.4)	59.6 (43.0 to 82.1)	15.1 (7.2 to 27.0)	8.6 (8.6 to 8.6)
Kenya	2003	61.7 (51.2 to 74.5)	18.8 (14.5 to 24.0)	29.6 (21.5 to 40.8)	7.3 (3.4 to 13.2)	6.1 (6.1 to 6.1)	126.5 (104.8 to 152.6)	38.4 (29.6 to 49.2)	60.7 (44.0 to 83.6)	14.9 (7.1 to 27.0)	12.4 (12.4 to 12.4)
Kenya	2004	63.5 (53.0 to 76.7)	18.8 (14.5 to 24.0)	29.9 (21.9 to 40.5)	7.2 (3.5 to 13.0)	7.6 (7.6 to 7.6)	130.2 (108.6 to 157.0)	38.5 (29.6 to 49.1)	61.3 (44.9 to 83.0)	14.8 (7.2 to 26.6)	15.6 (15.6 to 15.6)
Kenya	2005	63.8 (53.4 to 76.7)	19.1 (14.7 to 24.3)	29.8 (21.8 to 40.3)	7.3 (3.5 to 12.9)	7.6 (7.6 to 7.6)	130.7 (109.3 to 157.1)	39.2 (30.1 to 49.8)	61.0 (44.6 to 82.5)	14.9 (7.1 to 26.4)	15.6 (15.6 to 15.6)
Kenya	2006	66.5 (56.3 to 78.5)	19.4 (14.8 to 24.7)	28.4 (21.0 to 38.2)	7.2 (3.5 to 12.8)	11.4 (11.4 to 11.4)	136.1 (115.4 to 160.8)	39.7 (30.4 to 50.6)	58.3 (42.9 to 78.2)	14.8 (7.1 to 26.2)	23.4 (23.4 to 23.4)
Kenya	2007	67.1 (56.9 to 79.5)	20.3 (15.6 to 26.2)	27.6 (20.4 to 36.8)	7.9 (3.8 to 14.0)	11.2 (11.2 to 11.2)	137.4 (116.6 to 162.9)	41.7 (31.9 to 53.7)	56.5 (41.7 to 75.4)	16.1 (7.8 to 28.8)	23.0 (23.0 to 23.0)
Kenya	2008	69.5 (59.6 to 81.9)	20.6 (15.9 to 26.6)	26.0 (19.2 to 35.2)	8.3 (4.1 to 14.5)	14.6 (14.6 to 14.6)	142.3 (122.0 to 167.7)	42.2 (32.6 to 54.5)	53.3 (39.3 to 72.2)	17.0 (8.3 to 29.6)	29.8 (29.8 to 29.8)

		2018 US Dollars per capita					2018 PPP per capita				
Country	Year	Health spending	Government health spending	Out-of-pocket health spending	Prepaid private health spending	Development assistance for health	Health spending	Government health spending	Out-of-pocket health spending	Prepaid private health spending	Development assistance for health
Kenya	2009	71.0 (61.1 to 83.5)	21.5 (16.6 to 27.9)	25.1 (18.5 to 34.3)	8.8 (4.5 to 15.8)	15.6 (15.6 to 15.6)	145.4 (125.2 to 171.0)	44.0 (34.0 to 57.1)	51.5 (37.9 to 70.2)	18.1 (9.3 to 32.3)	31.9 (31.9 to 31.9)
Kenya	2010	76.7 (65.7 to 88.9)	23.2 (17.8 to 30.4)	25.2 (18.6 to 34.1)	9.6 (4.9 to 16.9)	18.7 (18.7 to 18.7)	157.0 (134.6 to 182.0)	47.6 (36.5 to 62.2)	51.6 (38.0 to 69.8)	19.7 (10.0 to 34.6)	38.2 (38.2 to 38.2)
Kenya	2011	79.4 (68.4 to 91.7)	24.4 (18.7 to 31.5)	25.1 (18.4 to 33.6)	9.8 (5.1 to 17.5)	20.1 (20.1 to 20.1)	162.6 (140.1 to 187.9)	50.0 (38.3 to 64.5)	51.3 (37.8 to 68.9)	20.2 (10.4 to 35.9)	41.1 (41.1 to 41.1)
Kenya	2012	81.8 (70.5 to 94.8)	25.3 (19.2 to 32.9)	24.9 (18.4 to 33.5)	10.0 (5.1 to 17.9)	21.6 (21.6 to 21.6)	167.6 (144.4 to 194.2)	51.9 (39.2 to 67.3)	51.0 (37.7 to 68.5)	20.5 (10.4 to 36.7)	44.2 (44.2 to 44.2)
Kenya	2013	85.0 (73.4 to 98.9)	26.3 (20.1 to 33.9)	24.8 (18.2 to 33.3)	10.4 (5.2 to 18.9)	23.5 (23.5 to 23.5)	174.1 (150.3 to 202.5)	53.9 (41.2 to 69.5)	50.8 (37.3 to 68.1)	21.3 (10.7 to 38.7)	48.2 (48.2 to 48.2)
Kenya	2014	82.4 (70.6 to 97.3)	26.8 (20.5 to 34.8)	23.9 (17.4 to 32.1)	11.1 (5.5 to 20.9)	20.6 (20.6 to 20.6)	168.8 (144.6 to 199.2)	54.9 (42.1 to 71.3)	49.0 (35.6 to 65.7)	22.7 (11.3 to 42.8)	42.2 (42.2 to 42.2)
Kenya	2015	80.6 (68.5 to 96.1)	27.2 (21.0 to 35.4)	22.9 (16.5 to 30.8)	11.8 (5.7 to 22.5)	18.7 (18.7 to 18.7)	165.1 (140.4 to 196.8)	55.8 (43.0 to 72.5)	47.0 (33.7 to 63.0)	24.1 (11.7 to 46.0)	38.2 (38.2 to 38.2)
Kenya	2016	81.9 (70.0 to 95.9)	27.7 (21.1 to 35.8)	22.2 (15.8 to 30.0)	12.5 (6.1 to 24.5)	19.5 (19.5 to 19.5)	167.7 (143.4 to 196.4)	56.8 (43.3 to 73.3)	45.5 (32.4 to 61.5)	25.5 (12.5 to 50.1)	39.8 (39.8 to 39.8)
Kenya	2017	84.4 (71.6 to 99.7)	28.7 (21.9 to 37.1)	22.4 (16.0 to 30.2)	12.8 (6.3 to 25.2)	20.4 (20.4 to 20.4)	172.8 (146.7 to 204.2)	58.8 (44.8 to 76.0)	45.9 (32.9 to 61.8)	26.3 (12.9 to 51.6)	41.8 (41.8 to 41.8)
Kenya	2018	83.4 (70.9 to 99.4)	29.3 (22.2 to 37.9)	22.6 (16.1 to 30.5)	13.1 (6.4 to 25.6)	18.4 (18.3 to 18.5)	170.7 (145.1 to 203.6)	59.9 (45.5 to 77.6)	46.3 (33.0 to 62.5)	26.8 (13.1 to 52.5)	37.7 (37.4 to 38.0)
Kenya	2019	84.2 (70.5 to 99.6)	29.8 (22.6 to 38.6)	22.7 (16.2 to 30.9)	13.3 (6.5 to 26.1)	18.3 (17.1 to 19.4)	172.5 (144.4 to 203.9)	61.1 (46.4 to 79.1)	46.6 (33.2 to 63.3)	27.3 (13.4 to 53.5)	37.5 (35.0 to 39.6)
Kenya	2020	85.2 (71.3 to 102.1)	30.3 (23.1 to 39.2)	22.9 (16.3 to 31.1)	13.6 (6.6 to 26.6)	18.4 (16.8 to 19.9)	174.4 (146.0 to 209.1)	62.2 (47.3 to 80.3)	46.8 (33.4 to 63.6)	27.8 (13.6 to 54.4)	37.6 (34.5 to 40.7)
Kenya	2021	86.2 (72.6 to 102.5)	30.9 (23.5 to 39.9)	23.0 (16.5 to 31.4)	13.8 (6.7 to 27.0)	18.5 (16.7 to 20.5)	176.6 (148.6 to 210.0)	63.2 (48.0 to 81.8)	47.1 (33.7 to 64.3)	28.3 (13.8 to 55.4)	38.0 (34.1 to 42.0)
Kenya	2022	87.4 (73.2 to 103.1)	31.5 (23.9 to 40.6)	23.1 (16.5 to 31.5)	14.1 (6.9 to 27.6)	18.7 (16.6 to 21.0)	179.0 (149.9 to 211.2)	64.4 (49.0 to 83.2)	47.4 (33.7 to 64.5)	28.8 (14.1 to 56.5)	38.4 (34.1 to 43.1)
Kenya	2023	88.8 (75.5 to 105.2)	32.2 (24.5 to 41.5)	23.3 (16.6 to 31.6)	14.4 (7.1 to 28.3)	18.9 (16.4 to 21.5)	181.8 (154.6 to 215.5)	66.0 (50.2 to 85.1)	47.6 (34.0 to 64.7)	29.5 (14.5 to 57.9)	38.7 (33.6 to 44.1)
Kenya	2024	90.2 (75.8 to 107.2)	33.0 (25.1 to 42.6)	23.4 (16.7 to 31.9)	14.7 (7.2 to 28.9)	19.1 (16.4 to 22.0)	184.8 (155.3 to 219.6)	67.6 (51.5 to 87.3)	47.9 (34.1 to 65.3)	30.2 (14.8 to 59.3)	39.1 (33.7 to 45.1)
Kenya	2025	91.6 (77.1 to 109.5)	33.8 (25.8 to 43.7)	23.5 (16.8 to 32.0)	15.1 (7.4 to 29.6)	19.2 (16.3 to 22.5)	187.6 (158.0 to 224.3)	69.2 (52.9 to 89.5)	48.2 (34.5 to 65.5)	30.8 (15.2 to 60.7)	39.3 (33.5 to 46.1)
Kenya	2026	93.0 (78.4 to 110.9)	34.6 (26.4 to 44.8)	23.7 (17.0 to 32.1)	15.4 (7.6 to 30.3)	19.3 (16.3 to 22.9)	190.5 (160.5 to 227.1)	70.9 (54.1 to 91.8)	48.5 (34.7 to 65.7)	31.5 (15.6 to 62.1)	39.5 (33.3 to 46.9)
Kenya	2027	94.5 (78.7 to 112.7)	35.5 (27.1 to 45.9)	23.8 (16.9 to 32.2)	15.7 (7.8 to 31.0)	19.4 (16.1 to 23.6)	193.5 (161.2 to 230.8)	72.7 (55.5 to 94.1)	48.8 (34.6 to 65.9)	32.2 (15.9 to 63.6)	39.8 (32.9 to 48.3)
Kenya	2028	96.0 (79.4 to 114.4)	36.3 (27.6 to 46.9)	24.0 (16.9 to 32.4)	16.1 (8.0 to 31.8)	19.6 (16.0 to 23.9)	196.7 (162.7 to 234.4)	74.4 (56.6 to 96.1)	49.1 (34.6 to 66.4)	32.9 (16.3 to 65.0)	40.1 (32.9 to 49.0)
Kenya	2029	97.6 (81.4 to 116.5)	37.2 (28.2 to 48.1)	24.2 (17.0 to 32.8)	16.4 (8.1 to 32.5)	19.8 (15.9 to 24.5)	199.9 (166.6 to 238.5)	76.2 (57.8 to 98.4)	49.5 (34.8 to 67.1)	33.7 (16.6 to 66.5)	40.5 (32.5 to 50.3)

Country	Year	2018 US Dollars per capita					2018 PPP per capita				
		Health spending	Government health spending	Out-of-pocket health spending	Prepaid private health spending	Development assistance for health	Health spending	Government health spending	Out-of-pocket health spending	Prepaid private health spending	Development assistance for health
Kenya	2030	99.2 (82.1 to 119.1)	38.1 (28.9 to 49.3)	24.3 (17.2 to 33.2)	16.8 (8.3 to 33.2)	20.0 (15.9 to 25.0)	203.2 (168.1 to 243.9)	78.1 (59.2 to 100.9)	49.9 (35.2 to 67.9)	34.4 (16.9 to 68.0)	40.9 (32.6 to 51.1)
Kenya	2031	100.9 (83.2 to 121.7)	39.0 (29.5 to 50.5)	24.5 (17.3 to 33.5)	17.1 (8.4 to 33.9)	20.2 (15.8 to 25.5)	206.6 (170.4 to 249.4)	79.9 (60.4 to 103.4)	50.3 (35.3 to 68.7)	35.1 (17.2 to 69.5)	41.4 (32.3 to 52.3)
Kenya	2032	102.6 (84.5 to 123.0)	39.9 (30.1 to 51.8)	24.7 (17.3 to 34.0)	17.5 (8.6 to 34.8)	20.4 (15.7 to 26.2)	210.2 (173.1 to 251.8)	81.8 (61.7 to 106.2)	50.7 (35.4 to 69.6)	35.9 (17.5 to 71.2)	41.9 (32.2 to 53.6)
Kenya	2033	104.4 (87.0 to 126.2)	40.9 (30.8 to 53.1)	25.0 (17.3 to 34.4)	17.9 (8.7 to 35.6)	20.7 (15.7 to 27.0)	213.9 (178.1 to 258.5)	83.7 (63.1 to 108.8)	51.2 (35.4 to 70.4)	36.6 (17.9 to 73.0)	42.4 (32.3 to 55.3)
Kenya	2034	106.4 (88.0 to 128.9)	41.9 (31.5 to 54.5)	25.2 (17.4 to 35.0)	18.2 (8.9 to 36.5)	21.1 (15.8 to 28.2)	217.9 (180.2 to 263.9)	85.8 (64.6 to 111.6)	51.7 (35.6 to 71.8)	37.4 (18.2 to 74.8)	43.1 (32.4 to 57.7)
Kenya	2035	108.4 (89.3 to 131.1)	42.9 (32.3 to 56.0)	25.5 (17.4 to 35.8)	18.6 (9.1 to 37.3)	21.4 (15.8 to 28.6)	222.0 (182.9 to 268.6)	87.9 (66.1 to 114.6)	52.2 (35.7 to 73.2)	38.1 (18.6 to 76.4)	43.8 (32.3 to 58.6)
Kenya	2036	110.5 (91.0 to 133.3)	44.0 (33.0 to 57.1)	25.7 (17.6 to 36.2)	19.0 (9.3 to 38.0)	21.8 (16.0 to 29.5)	226.2 (186.3 to 273.0)	90.0 (67.6 to 117.0)	52.7 (36.0 to 74.1)	38.9 (19.0 to 77.9)	44.6 (32.7 to 60.5)
Kenya	2037	112.6 (92.8 to 137.3)	45.1 (33.8 to 58.6)	26.0 (17.7 to 36.8)	19.4 (9.5 to 38.8)	22.1 (15.9 to 30.4)	230.6 (190.0 to 281.2)	92.3 (69.3 to 120.0)	53.3 (36.3 to 75.4)	39.7 (19.5 to 79.4)	45.2 (32.6 to 62.2)
Kenya	2038	114.8 (93.2 to 140.1)	46.2 (34.7 to 59.9)	26.3 (17.9 to 37.5)	19.8 (9.7 to 39.5)	22.4 (15.9 to 31.5)	235.1 (190.9 to 286.9)	94.7 (71.1 to 122.7)	54.0 (36.6 to 76.8)	40.6 (19.9 to 81.0)	46.0 (32.6 to 64.5)
Kenya	2039	117.1 (95.4 to 144.0)	47.4 (35.6 to 61.4)	26.7 (18.0 to 38.1)	20.2 (9.9 to 40.3)	22.8 (16.0 to 32.8)	239.8 (195.3 to 294.9)	97.1 (72.8 to 125.7)	54.6 (36.9 to 78.0)	41.4 (20.3 to 82.6)	46.7 (32.8 to 67.3)
Kenya	2040	119.5 (98.8 to 147.2)	48.7 (36.4 to 62.9)	27.0 (18.2 to 38.6)	20.6 (10.1 to 41.2)	23.2 (16.0 to 34.5)	244.8 (202.4 to 301.6)	99.7 (74.6 to 128.9)	55.3 (37.2 to 79.2)	42.2 (20.8 to 84.4)	47.6 (32.8 to 70.7)
Kenya	2041	122.1 (100.3 to 149.5)	49.9 (37.4 to 64.5)	27.4 (18.3 to 39.3)	21.0 (10.3 to 42.1)	23.7 (16.2 to 35.5)	250.0 (205.4 to 306.2)	102.3 (76.6 to 132.1)	56.1 (37.5 to 80.4)	43.1 (21.2 to 86.2)	48.5 (33.2 to 72.7)
Kenya	2042	124.7 (102.2 to 153.4)	51.3 (38.2 to 66.5)	27.8 (18.5 to 39.9)	21.5 (10.5 to 43.0)	24.2 (16.3 to 36.9)	255.4 (209.3 to 314.2)	105.0 (78.2 to 136.2)	56.9 (38.0 to 81.8)	44.0 (21.6 to 88.1)	49.6 (33.4 to 75.5)
Kenya	2043	127.3 (103.8 to 157.6)	52.6 (39.3 to 68.4)	28.2 (18.8 to 40.6)	21.9 (10.7 to 43.7)	24.7 (16.4 to 38.8)	260.8 (212.7 to 322.8)	107.7 (80.4 to 140.2)	57.7 (38.4 to 83.2)	44.9 (22.0 to 89.6)	50.6 (33.5 to 79.4)
Kenya	2044	130.1 (106.7 to 162.6)	54.0 (40.3 to 70.6)	28.6 (19.0 to 41.2)	22.3 (10.9 to 44.5)	25.3 (16.5 to 39.6)	266.6 (218.4 to 333.1)	110.5 (82.5 to 144.5)	58.5 (38.9 to 84.4)	45.8 (22.4 to 91.2)	51.7 (33.8 to 81.1)
Kenya	2045	133.0 (108.4 to 164.5)	55.4 (41.2 to 72.6)	29.0 (19.3 to 41.8)	22.8 (11.1 to 45.4)	25.8 (16.8 to 41.0)	272.5 (222.0 to 337.0)	113.5 (84.4 to 148.7)	59.4 (39.5 to 85.6)	46.7 (22.8 to 93.1)	52.9 (34.5 to 84.1)
Kenya	2046	136.0 (110.5 to 170.9)	56.8 (41.8 to 74.5)	29.5 (19.5 to 42.3)	23.2 (11.3 to 46.4)	26.4 (17.1 to 43.2)	278.5 (226.3 to 350.1)	116.4 (85.7 to 152.6)	60.4 (40.0 to 86.7)	47.6 (23.2 to 95.1)	54.1 (34.9 to 88.4)
Kenya	2047	139.0 (111.6 to 174.1)	58.3 (42.8 to 76.8)	29.9 (19.8 to 43.1)	23.7 (11.6 to 47.4)	27.1 (17.0 to 46.5)	284.8 (228.5 to 356.6)	119.4 (87.8 to 157.2)	61.3 (40.6 to 88.2)	48.6 (23.7 to 97.2)	55.5 (34.8 to 95.3)
Kenya	2048	142.2 (113.0 to 181.2)	59.7 (44.1 to 78.9)	30.4 (20.1 to 43.8)	24.2 (11.8 to 48.5)	27.8 (17.3 to 48.5)	291.2 (231.4 to 371.1)	122.4 (90.2 to 161.6)	62.3 (41.2 to 89.7)	49.5 (24.1 to 99.3)	57.0 (35.4 to 99.4)
Kenya	2049	145.3 (115.0 to 182.3)	61.2 (45.3 to 80.9)	30.9 (20.4 to 44.4)	24.6 (12.0 to 49.5)	28.6 (17.4 to 49.9)	297.7 (235.5 to 373.4)	125.4 (92.8 to 165.8)	63.3 (41.9 to 91.0)	50.5 (24.6 to 101.4)	58.5 (35.7 to 102.1)
Kenya	2050	148.6 (118.0 to 186.5)	62.7 (46.3 to 83.4)	31.4 (20.8 to 45.2)	25.1 (12.2 to 50.6)	29.4 (17.7 to 51.8)	304.5 (241.7 to 381.9)	128.5 (94.8 to 170.9)	64.4 (42.6 to 92.5)	51.4 (25.0 to 103.5)	60.2 (36.2 to 106.2)

Country	Year	2018 US Dollars per capita					2018 PPP per capita				
		Health spending	Government health spending	Out-of-pocket health spending	Prepaid private health spending	Development assistance for health	Health spending	Government health spending	Out-of-pocket health spending	Prepaid private health spending	Development assistance for health
Kiribati	1995	168.7 (146.5 to 192.5)	145.0 (124.6 to 167.9)	20.1 (13.8 to 28.0)	0.5 (0.2 to 1.0)	3.1 (3.1 to 3.1)	198.2 (172.1 to 226.1)	170.3 (146.4 to 197.3)	23.6 (16.2 to 32.9)	0.6 (0.3 to 1.2)	3.6 (3.6 to 3.6)
Kiribati	1996	164.7 (144.4 to 186.7)	144.1 (125.3 to 164.8)	20.1 (13.8 to 27.9)	0.5 (0.2 to 0.9)	0.0 (0.0 to 0.0)	193.5 (169.6 to 219.3)	169.3 (147.2 to 193.6)	23.6 (16.3 to 32.7)	0.6 (0.3 to 1.1)	0.0 (0.0 to 0.0)
Kiribati	1997	163.9 (145.1 to 185.7)	142.3 (123.7 to 162.0)	20.0 (13.9 to 27.5)	0.4 (0.2 to 0.8)	1.2 (1.2 to 1.2)	192.6 (170.5 to 218.2)	167.1 (145.3 to 190.2)	23.5 (16.3 to 32.4)	0.5 (0.2 to 1.0)	1.5 (1.5 to 1.5)
Kiribati	1998	163.0 (144.1 to 184.4)	142.1 (124.4 to 161.2)	20.0 (13.8 to 27.5)	0.3 (0.1 to 0.6)	0.5 (0.5 to 0.5)	191.5 (169.3 to 216.6)	167.0 (146.2 to 189.4)	23.5 (16.2 to 32.3)	0.4 (0.2 to 0.7)	0.6 (0.6 to 0.6)
Kiribati	1999	164.9 (146.0 to 185.8)	143.3 (125.8 to 162.0)	19.5 (13.4 to 26.7)	0.2 (0.1 to 0.3)	1.9 (1.9 to 1.9)	193.8 (171.5 to 218.2)	168.4 (147.7 to 190.2)	23.0 (15.7 to 31.3)	0.2 (0.1 to 0.3)	2.3 (2.3 to 2.3)
Kiribati	2000	175.2 (155.8 to 197.2)	153.5 (135.4 to 173.8)	19.1 (13.2 to 26.3)	0.0 (0.0 to 0.1)	2.5 (2.5 to 2.5)	205.8 (183.0 to 231.7)	180.4 (159.0 to 204.2)	22.5 (15.5 to 30.9)	0.0 (0.0 to 0.1)	2.9 (2.9 to 2.9)
Kiribati	2001	186.4 (166.2 to 209.8)	165.1 (145.9 to 186.0)	17.5 (12.3 to 24.2)	0.0 (0.0 to 0.0)	3.8 (3.8 to 3.8)	219.0 (195.2 to 246.5)	193.9 (171.4 to 218.5)	20.6 (14.5 to 28.4)	0.0 (0.0 to 0.0)	4.4 (4.4 to 4.4)
Kiribati	2002	195.5 (174.3 to 219.5)	174.7 (153.7 to 196.8)	17.3 (12.1 to 24.1)	0.0 (0.0 to 0.0)	3.5 (3.5 to 3.5)	229.7 (204.7 to 257.8)	205.2 (180.5 to 231.2)	20.3 (14.3 to 28.3)	0.0 (0.0 to 0.0)	4.2 (4.2 to 4.2)
Kiribati	2003	199.8 (178.5 to 223.2)	175.9 (155.1 to 198.2)	17.3 (12.2 to 24.3)	0.0 (0.0 to 0.0)	6.6 (6.6 to 6.6)	234.7 (209.7 to 262.2)	206.6 (182.2 to 232.8)	20.4 (14.3 to 28.6)	0.0 (0.0 to 0.0)	7.7 (7.7 to 7.7)
Kiribati	2004	271.4 (250.0 to 294.1)	175.2 (155.0 to 195.8)	17.3 (12.2 to 24.6)	0.0 (0.0 to 0.0)	78.9 (78.9 to 78.9)	318.8 (293.7 to 345.5)	205.8 (182.0 to 230.0)	20.4 (14.3 to 28.8)	0.0 (0.0 to 0.0)	92.7 (92.7 to 92.7)
Kiribati	2005	225.2 (204.6 to 246.3)	171.9 (152.4 to 192.5)	17.6 (12.2 to 24.9)	0.0 (0.0 to 0.0)	35.7 (35.7 to 35.7)	264.6 (240.4 to 289.3)	202.0 (179.1 to 226.2)	20.7 (14.3 to 29.3)	0.0 (0.0 to 0.0)	41.9 (41.9 to 41.9)
Kiribati	2006	209.4 (190.5 to 229.0)	155.4 (137.3 to 174.8)	17.4 (12.2 to 24.6)	0.0 (0.0 to 0.0)	36.5 (36.5 to 36.5)	246.0 (223.8 to 269.1)	182.6 (161.2 to 205.3)	20.5 (14.3 to 28.9)	0.0 (0.0 to 0.0)	42.9 (42.9 to 42.9)
Kiribati	2007	225.1 (206.3 to 245.1)	148.2 (130.2 to 167.4)	18.4 (12.7 to 26.4)	0.0 (0.0 to 0.0)	58.4 (58.4 to 58.4)	264.4 (242.4 to 288.0)	174.1 (153.0 to 196.7)	21.7 (14.9 to 31.1)	0.0 (0.0 to 0.0)	68.6 (68.6 to 68.6)
Kiribati	2008	265.5 (247.0 to 285.5)	139.2 (122.7 to 157.5)	21.2 (14.6 to 30.8)	0.2 (0.1 to 0.4)	104.9 (104.9 to 104.9)	311.8 (290.2 to 335.3)	163.5 (144.2 to 185.0)	24.8 (17.2 to 36.1)	0.3 (0.1 to 0.5)	123.3 (123.3 to 123.3)
Kiribati	2009	218.0 (200.3 to 237.4)	126.6 (111.4 to 144.4)	22.3 (15.5 to 32.9)	1.5 (0.7 to 2.7)	67.6 (67.6 to 67.6)	256.1 (235.3 to 278.9)	148.8 (130.9 to 169.6)	26.2 (18.2 to 38.6)	1.7 (0.8 to 3.1)	79.4 (79.4 to 79.4)
Kiribati	2010	217.9 (201.0 to 235.8)	115.1 (100.3 to 131.2)	22.5 (16.0 to 32.5)	3.5 (1.7 to 6.5)	76.7 (76.7 to 76.7)	256.0 (236.1 to 277.0)	135.3 (117.8 to 154.2)	26.5 (18.8 to 38.2)	4.1 (2.0 to 7.6)	90.1 (90.1 to 90.1)
Kiribati	2011	203.9 (187.5 to 222.8)	109.9 (95.4 to 126.2)	22.8 (16.3 to 32.5)	5.4 (2.7 to 9.9)	65.8 (65.8 to 65.8)	239.5 (220.3 to 261.7)	129.1 (112.1 to 148.2)	26.8 (19.1 to 38.1)	6.3 (3.1 to 11.7)	77.3 (77.3 to 77.3)
Kiribati	2012	210.6 (192.8 to 230.3)	111.1 (96.4 to 127.6)	23.6 (16.6 to 33.8)	6.7 (3.2 to 12.3)	69.3 (69.3 to 69.3)	247.4 (226.4 to 270.6)	130.5 (113.2 to 149.9)	27.7 (19.5 to 39.7)	7.8 (3.8 to 14.4)	81.4 (81.4 to 81.4)
Kiribati	2013	198.2 (179.5 to 218.9)	115.7 (100.6 to 132.9)	24.6 (17.3 to 35.0)	7.5 (3.6 to 13.6)	50.5 (50.5 to 50.5)	232.9 (210.9 to 257.1)	135.9 (118.1 to 156.1)	28.9 (20.4 to 41.1)	8.8 (4.2 to 16.0)	59.3 (59.3 to 59.3)
Kiribati	2014	174.6 (155.4 to 197.3)	118.3 (102.4 to 137.5)	25.3 (17.5 to 35.7)	7.7 (3.7 to 14.1)	23.4 (23.4 to 23.4)	205.1 (182.6 to 231.8)	139.0 (120.2 to 161.5)	29.7 (20.5 to 41.9)	9.0 (4.3 to 16.6)	27.4 (27.4 to 27.4)
Kiribati	2015	184.5 (164.0 to 208.4)	123.1 (105.9 to 144.1)	26.2 (18.3 to 36.8)	7.8 (3.6 to 14.6)	27.3 (27.3 to 27.3)	216.7 (192.6 to 244.8)	144.7 (124.5 to 169.3)	30.8 (21.5 to 43.2)	9.2 (4.3 to 17.2)	32.1 (32.1 to 32.1)

Country	Year	2018 US Dollars per capita					2018 PPP per capita				
		Health spending	Government health spending	Out-of-pocket health spending	Prepaid private health spending	Development assistance for health	Health spending	Government health spending	Out-of-pocket health spending	Prepaid private health spending	Development assistance for health
Kiribati	2016	198.3 (175.9 to 223.6)	128.3 (108.4 to 151.8)	26.9 (18.8 to 37.5)	7.9 (3.7 to 14.6)	35.2 (35.2 to 35.2)	233.0 (206.7 to 262.7)	150.7 (127.4 to 178.4)	31.7 (22.1 to 44.1)	9.3 (4.3 to 17.2)	41.4 (41.4 to 41.4)
Kiribati	2017	189.6 (169.6 to 214.1)	114.2 (96.7 to 135.2)	27.1 (19.0 to 38.0)	8.1 (3.7 to 15.0)	40.3 (40.3 to 40.3)	222.7 (199.2 to 251.5)	134.1 (113.6 to 158.8)	31.8 (22.3 to 44.6)	9.5 (4.4 to 17.6)	47.3 (47.3 to 47.3)
Kiribati	2018	187.7 (166.8 to 212.2)	115.7 (97.5 to 136.8)	27.2 (19.1 to 38.1)	8.2 (3.8 to 15.2)	36.7 (36.5 to 36.9)	220.5 (196.0 to 249.3)	135.9 (114.6 to 160.7)	32.0 (22.4 to 44.7)	9.6 (4.4 to 17.8)	43.1 (42.8 to 43.4)
Kiribati	2019	187.3 (165.6 to 212.2)	115.0 (97.0 to 136.0)	27.3 (19.0 to 38.0)	8.2 (3.8 to 15.2)	36.8 (34.3 to 39.0)	220.1 (194.6 to 249.3)	135.1 (114.0 to 159.8)	32.1 (22.4 to 44.7)	9.6 (4.5 to 17.9)	43.2 (40.3 to 45.8)
Kiribati	2020	185.1 (164.2 to 210.5)	112.3 (94.3 to 133.4)	27.4 (19.0 to 38.1)	8.3 (3.8 to 15.3)	37.1 (34.0 to 40.2)	217.5 (192.9 to 247.2)	132.0 (110.7 to 156.7)	32.2 (22.4 to 44.8)	9.7 (4.5 to 18.0)	43.6 (39.9 to 47.2)
Kiribati	2021	183.4 (162.4 to 209.2)	110.0 (92.0 to 130.6)	27.5 (19.0 to 38.5)	8.3 (3.8 to 15.3)	37.6 (33.9 to 41.7)	215.4 (190.8 to 245.7)	129.2 (108.1 to 153.4)	32.3 (22.3 to 45.3)	9.7 (4.5 to 18.0)	44.2 (39.8 to 49.0)
Kiribati	2022	182.1 (161.5 to 208.2)	107.9 (90.6 to 127.9)	27.6 (19.0 to 38.7)	8.3 (3.8 to 15.4)	38.3 (34.0 to 42.8)	213.9 (189.7 to 244.6)	126.8 (106.5 to 150.2)	32.4 (22.3 to 45.5)	9.7 (4.5 to 18.1)	45.0 (39.9 to 50.3)
Kiribati	2023	181.2 (160.9 to 206.1)	106.3 (89.0 to 126.0)	27.6 (19.1 to 38.8)	8.3 (3.9 to 15.4)	39.0 (33.9 to 44.4)	212.9 (189.0 to 242.1)	124.9 (104.6 to 148.0)	32.4 (22.4 to 45.5)	9.8 (4.5 to 18.1)	45.8 (39.8 to 52.2)
Kiribati	2024	181.0 (159.8 to 205.5)	105.2 (87.6 to 125.0)	27.7 (19.1 to 38.7)	8.3 (3.8 to 15.5)	39.7 (34.2 to 45.7)	212.7 (187.7 to 241.4)	123.6 (102.9 to 146.9)	32.6 (22.4 to 45.5)	9.8 (4.5 to 18.2)	46.7 (40.2 to 53.7)
Kiribati	2025	181.3 (159.6 to 206.1)	104.6 (87.0 to 124.2)	27.8 (19.1 to 39.2)	8.4 (3.9 to 15.6)	40.4 (34.4 to 47.3)	212.9 (187.4 to 242.1)	122.9 (102.2 to 145.9)	32.7 (22.5 to 46.0)	9.9 (4.5 to 18.3)	47.5 (40.5 to 55.5)
Kiribati	2026	182.8 (161.9 to 208.7)	105.3 (87.4 to 125.1)	28.0 (19.1 to 39.4)	8.5 (3.9 to 15.7)	41.1 (34.6 to 47.9)	214.7 (190.2 to 245.2)	123.7 (102.7 to 146.9)	32.9 (22.5 to 46.2)	9.9 (4.6 to 18.4)	48.3 (40.7 to 56.3)
Kiribati	2027	185.0 (163.3 to 210.2)	106.5 (89.1 to 126.6)	28.2 (19.2 to 39.5)	8.5 (3.9 to 15.9)	41.8 (35.0 to 49.8)	217.3 (191.8 to 246.9)	125.1 (104.6 to 148.7)	33.1 (22.6 to 46.4)	10.0 (4.6 to 18.6)	49.1 (41.1 to 58.5)
Kiribati	2028	188.1 (166.3 to 214.8)	108.6 (90.2 to 129.3)	28.4 (19.2 to 40.0)	8.7 (4.0 to 16.1)	42.6 (35.5 to 51.3)	221.0 (195.4 to 252.3)	127.5 (106.0 to 151.9)	33.3 (22.6 to 47.0)	10.2 (4.7 to 18.9)	50.0 (41.7 to 60.3)
Kiribati	2029	191.4 (168.3 to 219.6)	110.7 (92.0 to 132.5)	28.6 (19.3 to 40.5)	8.8 (4.0 to 16.3)	43.3 (35.7 to 53.1)	224.9 (197.8 to 257.9)	130.1 (108.1 to 155.6)	33.6 (22.7 to 47.6)	10.3 (4.7 to 19.2)	50.9 (42.0 to 62.4)
Kiribati	2030	194.8 (170.1 to 223.1)	113.0 (93.8 to 135.3)	28.8 (19.4 to 40.9)	8.9 (4.1 to 16.6)	44.1 (36.0 to 54.0)	228.8 (199.8 to 262.1)	132.7 (110.2 to 158.9)	33.9 (22.8 to 48.1)	10.4 (4.8 to 19.4)	51.8 (42.3 to 63.4)
Kiribati	2031	198.1 (173.7 to 228.1)	115.2 (95.7 to 138.3)	29.1 (19.5 to 41.5)	9.0 (4.1 to 16.8)	44.9 (36.3 to 56.4)	232.8 (204.0 to 268.0)	135.3 (112.4 to 162.5)	34.2 (22.9 to 48.7)	10.6 (4.8 to 19.7)	52.7 (42.7 to 66.2)
Kiribati	2032	201.5 (178.3 to 231.1)	117.3 (97.5 to 141.6)	29.3 (19.6 to 41.9)	9.1 (4.2 to 17.0)	45.7 (36.7 to 57.8)	236.7 (209.4 to 271.5)	137.8 (114.6 to 166.4)	34.5 (23.0 to 49.2)	10.7 (4.9 to 19.9)	53.7 (43.2 to 67.9)
Kiribati	2033	204.8 (179.6 to 237.4)	119.4 (98.8 to 145.0)	29.6 (19.6 to 42.4)	9.2 (4.2 to 17.2)	46.6 (36.9 to 60.1)	240.6 (210.9 to 278.9)	140.3 (116.0 to 170.4)	34.8 (23.1 to 49.8)	10.8 (4.9 to 20.2)	54.7 (43.4 to 70.6)
Kiribati	2034	207.9 (182.0 to 241.4)	121.2 (100.1 to 147.2)	29.9 (19.7 to 43.0)	9.3 (4.3 to 17.4)	47.6 (37.4 to 61.4)	244.3 (213.8 to 283.6)	142.3 (117.6 to 172.9)	35.1 (23.1 to 50.5)	10.9 (5.0 to 20.5)	55.9 (43.9 to 72.1)
Kiribati	2035	211.0 (183.8 to 246.2)	122.8 (101.5 to 149.4)	30.1 (19.8 to 43.6)	9.4 (4.3 to 17.7)	48.6 (37.7 to 63.4)	247.9 (215.9 to 289.2)	144.3 (119.3 to 175.5)	35.4 (23.2 to 51.2)	11.1 (5.1 to 20.8)	57.1 (44.3 to 74.5)
Kiribati	2036	214.0 (185.1 to 248.8)	124.4 (102.8 to 151.4)	30.4 (19.9 to 44.2)	9.6 (4.4 to 17.9)	49.5 (38.4 to 66.3)	251.4 (217.5 to 292.3)	146.2 (120.7 to 177.8)	35.8 (23.4 to 51.9)	11.3 (5.1 to 21.0)	58.2 (45.1 to 77.9)

Country	Year	2018 US Dollars per capita					2018 PPP per capita				
		Health spending	Government health spending	Out-of-pocket health spending	Prepaid private health spending	Development assistance for health	Health spending	Government health spending	Out-of-pocket health spending	Prepaid private health spending	Development assistance for health
Kiribati	2037	216.7 (188.2 to 252.8)	125.8 (103.4 to 152.5)	30.8 (20.0 to 44.8)	9.7 (4.4 to 18.2)	50.5 (38.7 to 67.4)	254.6 (221.1 to 297.0)	147.7 (121.5 to 179.2)	36.1 (23.5 to 52.6)	11.4 (5.2 to 21.4)	59.3 (45.5 to 79.1)
Kiribati	2038	219.4 (189.3 to 255.7)	127.0 (104.2 to 154.2)	31.1 (20.0 to 45.4)	9.9 (4.5 to 18.5)	51.5 (39.1 to 71.6)	257.8 (222.4 to 300.3)	149.2 (122.4 to 181.1)	36.5 (23.6 to 53.3)	11.6 (5.3 to 21.7)	60.5 (46.0 to 84.2)
Kiribati	2039	222.1 (191.1 to 257.9)	128.2 (105.5 to 156.7)	31.4 (20.3 to 45.9)	10.0 (4.6 to 18.8)	52.5 (39.9 to 72.4)	260.9 (224.5 to 302.9)	150.6 (124.0 to 184.1)	36.9 (23.8 to 54.0)	11.8 (5.4 to 22.1)	61.6 (46.9 to 85.1)
Kiribati	2040	224.9 (194.2 to 263.5)	129.4 (106.0 to 158.6)	31.7 (20.5 to 46.5)	10.2 (4.6 to 19.0)	53.6 (40.6 to 73.9)	264.2 (228.2 to 309.6)	152.0 (124.5 to 186.4)	37.3 (24.0 to 54.6)	11.9 (5.4 to 22.4)	62.9 (47.7 to 86.8)
Kiribati	2041	227.8 (197.3 to 266.9)	130.8 (107.5 to 160.5)	32.1 (20.6 to 47.0)	10.3 (4.7 to 19.3)	54.7 (41.2 to 76.4)	267.6 (231.7 to 313.5)	153.6 (126.3 to 188.6)	37.7 (24.2 to 55.3)	12.1 (5.5 to 22.7)	64.2 (48.4 to 89.8)
Kiribati	2042	230.9 (198.2 to 271.1)	132.1 (108.6 to 162.6)	32.4 (20.8 to 47.6)	10.5 (4.7 to 19.7)	55.9 (41.8 to 79.8)	271.3 (232.8 to 318.5)	155.2 (127.5 to 191.0)	38.1 (24.4 to 55.9)	12.3 (5.6 to 23.1)	65.7 (49.1 to 93.7)
Kiribati	2043	234.1 (200.6 to 275.3)	133.6 (108.9 to 164.6)	32.8 (21.1 to 48.1)	10.6 (4.8 to 20.1)	57.1 (42.3 to 83.0)	275.0 (235.6 to 323.4)	157.0 (128.0 to 193.4)	38.5 (24.8 to 56.5)	12.5 (5.7 to 23.6)	67.0 (49.7 to 97.5)
Kiribati	2044	237.4 (203.0 to 280.0)	135.1 (109.8 to 166.1)	33.2 (21.4 to 48.7)	10.8 (4.9 to 20.4)	58.4 (43.2 to 84.6)	278.9 (238.4 to 328.9)	158.7 (128.9 to 195.2)	39.0 (25.1 to 57.2)	12.6 (5.7 to 23.9)	68.6 (50.7 to 99.3)
Kiribati	2045	240.8 (204.3 to 286.0)	136.6 (111.3 to 168.7)	33.6 (21.6 to 49.4)	10.9 (4.9 to 20.6)	59.7 (43.1 to 90.0)	282.8 (240.0 to 336.0)	160.5 (130.8 to 198.1)	39.4 (25.4 to 58.0)	12.8 (5.8 to 24.2)	70.1 (50.7 to 105.7)
Kiribati	2046	244.5 (206.9 to 292.3)	138.4 (111.4 to 170.9)	34.0 (21.9 to 50.0)	11.1 (5.0 to 20.9)	61.1 (44.0 to 93.0)	287.2 (243.0 to 343.4)	162.6 (130.9 to 200.8)	39.9 (25.7 to 58.8)	13.0 (5.9 to 24.5)	71.7 (51.7 to 109.3)
Kiribati	2047	248.2 (209.9 to 297.8)	140.1 (112.4 to 173.2)	34.4 (22.2 to 50.8)	11.2 (5.1 to 21.1)	62.5 (44.9 to 98.0)	291.6 (246.6 to 349.8)	164.5 (132.0 to 203.5)	40.4 (26.1 to 59.6)	13.2 (5.9 to 24.8)	73.5 (52.7 to 115.1)
Kiribati	2048	252.2 (214.4 to 304.0)	141.9 (113.8 to 175.8)	34.8 (22.5 to 51.5)	11.4 (5.1 to 21.4)	64.1 (46.0 to 102.1)	296.3 (251.8 to 357.1)	166.7 (133.7 to 206.5)	40.9 (26.4 to 60.5)	13.3 (6.0 to 25.2)	75.3 (54.0 to 119.9)
Kiribati	2049	256.2 (216.9 to 309.6)	143.7 (115.0 to 177.9)	35.3 (22.8 to 52.2)	11.5 (5.2 to 21.8)	65.7 (46.7 to 105.4)	301.0 (254.8 to 363.7)	168.8 (135.1 to 209.0)	41.4 (26.8 to 61.4)	13.5 (6.1 to 25.6)	77.2 (54.9 to 123.9)
Kiribati	2050	260.4 (218.9 to 314.7)	145.6 (115.6 to 180.4)	35.7 (23.1 to 53.0)	11.7 (5.2 to 22.1)	67.5 (47.6 to 110.9)	305.9 (257.1 to 369.6)	171.0 (135.7 to 212.0)	41.9 (27.1 to 62.2)	13.7 (6.2 to 26.0)	79.3 (56.0 to 130.2)
Kuwait	1995	1444.3 (1294.7 to 1613.1)	1179.1 (1031.2 to 1344.5)	247.9 (214.4 to 282.8)	17.4 (9.3 to 30.7)	0.0 (0.0 to 0.0)	3340.7 (2994.7 to 3731.0)	2727.2 (2385.2 to 3109.9)	573.3 (495.9 to 654.1)	40.2 (21.4 to 70.9)	0.0 (0.0 to 0.0)
Kuwait	1996	1400.6 (1273.5 to 1532.0)	1126.3 (1002.7 to 1255.6)	256.7 (228.6 to 286.3)	17.7 (9.6 to 30.6)	0.0 (0.0 to 0.0)	3239.7 (2945.6 to 3543.5)	2605.2 (2319.3 to 2904.3)	593.7 (528.7 to 662.2)	40.8 (22.1 to 70.7)	0.0 (0.0 to 0.0)
Kuwait	1997	1392.6 (1275.6 to 1514.6)	1110.4 (993.9 to 1226.0)	263.9 (239.4 to 290.8)	18.3 (10.2 to 31.3)	0.0 (0.0 to 0.0)	3221.1 (2950.6 to 3503.2)	2568.5 (2298.9 to 2835.7)	610.3 (553.7 to 672.6)	42.3 (23.7 to 72.3)	0.0 (0.0 to 0.0)
Kuwait	1998	1407.4 (1297.1 to 1526.2)	1115.0 (1006.6 to 1236.0)	272.8 (248.4 to 301.6)	19.7 (11.3 to 32.5)	0.0 (0.0 to 0.0)	3255.4 (3000.3 to 3530.0)	2579.0 (2328.3 to 2858.9)	630.9 (574.5 to 697.6)	45.5 (26.2 to 75.1)	0.0 (0.0 to 0.0)
Kuwait	1999	1247.1 (1152.0 to 1350.0)	986.7 (894.4 to 1091.7)	242.4 (221.2 to 267.0)	18.0 (10.4 to 28.8)	0.0 (0.0 to 0.0)	2884.5 (2664.5 to 3122.6)	2282.4 (2068.8 to 2525.0)	560.7 (511.7 to 617.5)	41.5 (24.2 to 66.6)	0.0 (0.0 to 0.0)
Kuwait	2000	1145.2 (1055.8 to 1243.7)	908.1 (820.1 to 1004.3)	220.4 (199.9 to 241.4)	16.8 (9.7 to 26.3)	0.0 (0.0 to 0.0)	2648.9 (2442.1 to 2876.8)	2100.3 (1897.0 to 2323.0)	509.7 (462.5 to 558.5)	38.8 (22.4 to 60.7)	0.0 (0.0 to 0.0)
Kuwait	2001	1142.0 (1048.1 to 1243.9)	909.6 (820.0 to 1006.8)	215.1 (196.3 to 235.5)	17.3 (9.8 to 26.9)	0.0 (0.0 to 0.0)	2641.5 (2424.2 to 2877.2)	2104.0 (1896.7 to 2328.8)	497.6 (454.0 to 544.7)	40.0 (22.6 to 62.2)	0.0 (0.0 to 0.0)

		2018 US Dollars per capita					2018 PPP per capita				
Country	Year	Health spending	Government health spending	Out-of-pocket health spending	Prepaid private health spending	Development assistance for health	Health spending	Government health spending	Out-of-pocket health spending	Prepaid private health spending	Development assistance for health
Kuwait	2002	1132.1 (1038.9 to 1239.2)	900.0 (810.7 to 1003.2)	213.7 (196.2 to 234.5)	18.3 (10.5 to 29.1)	0.0 (0.0 to 0.0)	2618.5 (2403.0 to 2866.4)	2081.8 (1875.2 to 2320.4)	494.3 (453.9 to 542.3)	42.4 (24.2 to 67.2)	0.0 (0.0 to 0.0)
Kuwait	2003	1187.7 (1091.8 to 1301.5)	950.5 (855.4 to 1060.7)	216.0 (197.5 to 236.0)	21.1 (11.9 to 33.8)	0.0 (0.0 to 0.0)	2747.1 (2525.4 to 3010.4)	2198.5 (1978.6 to 2453.4)	499.7 (456.9 to 545.8)	48.9 (27.6 to 78.2)	0.0 (0.0 to 0.0)
Kuwait	2004	1161.1 (1065.1 to 1267.3)	933.3 (839.0 to 1039.3)	206.6 (189.7 to 225.2)	21.1 (11.8 to 34.4)	0.0 (0.0 to 0.0)	2685.5 (2463.5 to 2931.2)	2158.7 (1940.6 to 2404.0)	478.0 (438.7 to 521.0)	48.9 (27.4 to 79.6)	0.0 (0.0 to 0.0)
Kuwait	2005	1143.1 (1046.4 to 1249.7)	926.5 (829.6 to 1034.5)	195.6 (178.9 to 212.9)	21.0 (12.0 to 33.3)	0.0 (0.0 to 0.0)	2644.0 (2420.2 to 2890.5)	2143.0 (1918.9 to 2392.7)	452.4 (413.9 to 492.4)	48.6 (27.9 to 77.0)	0.0 (0.0 to 0.0)
Kuwait	2006	1105.5 (1010.5 to 1210.0)	902.7 (808.0 to 1008.0)	183.2 (166.3 to 200.2)	19.6 (11.6 to 32.0)	0.0 (0.0 to 0.0)	2556.9 (2337.4 to 2798.8)	2087.9 (1868.9 to 2331.6)	423.8 (384.7 to 463.0)	45.3 (26.7 to 74.0)	0.0 (0.0 to 0.0)
Kuwait	2007	1073.7 (982.7 to 1173.1)	879.4 (786.1 to 978.8)	175.7 (159.5 to 193.7)	18.6 (11.0 to 30.0)	0.0 (0.0 to 0.0)	2483.5 (2273.0 to 2713.4)	2034.0 (1818.3 to 2264.0)	406.5 (369.0 to 448.0)	43.0 (25.5 to 69.3)	0.0 (0.0 to 0.0)
Kuwait	2008	1104.9 (1011.9 to 1205.0)	917.5 (824.7 to 1017.7)	169.2 (153.2 to 186.4)	18.2 (10.7 to 29.4)	0.0 (0.0 to 0.0)	2555.7 (2340.5 to 2787.3)	2122.3 (1907.6 to 2353.9)	391.4 (354.3 to 431.2)	42.0 (24.7 to 68.1)	0.0 (0.0 to 0.0)
Kuwait	2009	1110.3 (1015.9 to 1215.7)	936.5 (845.2 to 1040.1)	156.7 (141.0 to 173.2)	17.1 (10.1 to 28.1)	0.0 (0.0 to 0.0)	2568.2 (2349.7 to 2812.0)	2166.2 (1954.9 to 2405.9)	362.5 (326.2 to 400.5)	39.6 (23.3 to 64.9)	0.0 (0.0 to 0.0)
Kuwait	2010	1031.4 (939.3 to 1132.8)	877.7 (790.6 to 974.3)	138.9 (125.3 to 153.9)	14.8 (8.9 to 24.1)	0.0 (0.0 to 0.0)	2385.7 (2172.7 to 2620.3)	2030.1 (1828.7 to 2253.6)	321.2 (289.7 to 356.1)	34.3 (20.6 to 55.6)	0.0 (0.0 to 0.0)
Kuwait	2011	1057.4 (963.5 to 1162.3)	909.3 (819.3 to 1011.8)	133.6 (120.4 to 148.2)	14.5 (8.8 to 23.5)	0.0 (0.0 to 0.0)	2445.8 (2228.6 to 2688.4)	2103.1 (1894.9 to 2340.3)	309.1 (278.5 to 342.9)	33.6 (20.4 to 54.3)	0.0 (0.0 to 0.0)
Kuwait	2012	1068.8 (977.0 to 1166.7)	918.6 (828.1 to 1017.7)	135.4 (121.7 to 150.4)	14.7 (8.6 to 23.7)	0.0 (0.0 to 0.0)	2472.0 (2259.9 to 2698.7)	2124.8 (1915.4 to 2354.0)	313.3 (281.4 to 347.9)	33.9 (19.8 to 54.9)	0.0 (0.0 to 0.0)
Kuwait	2013	1061.3 (967.6 to 1160.5)	908.1 (812.1 to 1006.1)	138.5 (124.6 to 153.4)	14.7 (8.3 to 23.9)	0.0 (0.0 to 0.0)	2454.7 (2238.0 to 2684.2)	2100.4 (1878.3 to 2327.2)	320.3 (288.2 to 354.8)	34.1 (19.2 to 55.3)	0.0 (0.0 to 0.0)
Kuwait	2014	1109.5 (1007.2 to 1216.4)	943.7 (842.1 to 1053.3)	150.0 (134.7 to 167.0)	15.7 (8.7 to 25.9)	0.0 (0.0 to 0.0)	2566.2 (2329.6 to 2813.4)	2182.8 (1947.9 to 2436.3)	347.0 (311.5 to 386.3)	36.4 (20.1 to 59.9)	0.0 (0.0 to 0.0)
Kuwait	2015	1217.5 (1087.6 to 1340.3)	1023.2 (899.2 to 1148.6)	176.0 (157.2 to 195.7)	18.3 (9.6 to 30.2)	0.0 (0.0 to 0.0)	2816.2 (2515.6 to 3100.2)	2366.7 (2079.9 to 2656.6)	407.0 (363.7 to 452.7)	42.4 (22.1 to 69.9)	0.0 (0.0 to 0.0)
Kuwait	2016	1279.1 (1140.0 to 1432.8)	1065.4 (923.6 to 1215.8)	194.3 (170.4 to 220.8)	19.4 (10.0 to 32.7)	0.0 (0.0 to 0.0)	2958.6 (2636.9 to 3314.0)	2464.4 (2136.4 to 2812.2)	449.3 (394.1 to 510.8)	44.9 (23.2 to 75.6)	0.0 (0.0 to 0.0)
Kuwait	2017	1204.8 (1071.3 to 1346.1)	995.8 (860.7 to 1135.8)	190.5 (167.3 to 217.4)	18.5 (9.6 to 31.1)	0.0 (0.0 to 0.0)	2786.7 (2477.8 to 3113.6)	2303.2 (1990.7 to 2627.0)	440.7 (387.0 to 503.0)	42.8 (22.1 to 71.9)	0.0 (0.0 to 0.0)
Kuwait	2018	1225.2 (1087.4 to 1366.5)	1015.8 (874.5 to 1155.5)	190.8 (166.9 to 217.5)	18.6 (9.6 to 31.3)	0.0 (0.0 to 0.0)	2833.9 (2515.2 to 3160.8)	2349.5 (2022.8 to 2672.6)	441.3 (386.1 to 503.2)	43.1 (22.2 to 72.4)	0.0 (0.0 to 0.0)
Kuwait	2019	1248.1 (1103.3 to 1399.4)	1038.8 (890.8 to 1190.9)	190.5 (166.8 to 217.2)	18.8 (9.6 to 31.5)	0.0 (0.0 to 0.0)	2886.9 (2551.9 to 3236.9)	2402.8 (2060.3 to 2754.5)	440.7 (385.8 to 502.4)	43.4 (22.3 to 72.8)	0.0 (0.0 to 0.0)
Kuwait	2020	1279.1 (1129.8 to 1438.9)	1069.6 (919.8 to 1226.6)	190.6 (166.7 to 217.3)	18.9 (9.7 to 31.8)	0.0 (0.0 to 0.0)	2958.5 (2613.3 to 3328.2)	2473.9 (2127.6 to 2837.2)	440.8 (385.5 to 502.7)	43.8 (22.4 to 73.7)	0.0 (0.0 to 0.0)
Kuwait	2021	1305.4 (1148.6 to 1475.2)	1096.4 (941.9 to 1263.0)	189.9 (166.1 to 216.3)	19.0 (9.8 to 32.1)	0.0 (0.0 to 0.0)	3019.3 (2656.8 to 3412.2)	2536.1 (2178.6 to 2921.3)	439.3 (384.2 to 500.4)	44.0 (22.6 to 74.2)	0.0 (0.0 to 0.0)
Kuwait	2022	1338.8 (1181.4 to 1519.9)	1129.9 (969.8 to 1311.5)	189.7 (165.7 to 216.1)	19.1 (9.8 to 32.3)	0.0 (0.0 to 0.0)	3096.7 (2732.5 to 3515.6)	2613.6 (2243.2 to 3033.6)	438.8 (383.3 to 499.8)	44.3 (22.7 to 74.8)	0.0 (0.0 to 0.0)

Country	Year	2018 US Dollars per capita					2018 PPP per capita				
		Health spending	Government health spending	Out-of-pocket health spending	Prepaid private health spending	Development assistance for health	Health spending	Government health spending	Out-of-pocket health spending	Prepaid private health spending	Development assistance for health
Kuwait	2023	1374.3 (1206.9 to 1560.7)	1165.4 (997.4 to 1345.3)	189.7 (165.4 to 216.3)	19.3 (9.9 to 32.5)	0.0 (0.0 to 0.0)	3178.7 (2791.5 to 3610.0)	2695.5 (2307.0 to 3111.7)	438.7 (382.5 to 500.3)	44.5 (22.9 to 75.3)	0.0 (0.0 to 0.0)
Kuwait	2024	1412.9 (1235.8 to 1612.7)	1202.2 (1026.3 to 1406.7)	191.2 (166.3 to 218.2)	19.4 (10.0 to 32.7)	0.0 (0.0 to 0.0)	3268.0 (2858.4 to 3730.2)	2780.8 (2373.8 to 3253.7)	442.3 (384.7 to 504.7)	44.8 (23.1 to 75.6)	0.0 (0.0 to 0.0)
Kuwait	2025	1444.1 (1255.4 to 1662.5)	1232.3 (1045.0 to 1446.6)	192.2 (166.4 to 220.0)	19.6 (10.1 to 33.1)	0.0 (0.0 to 0.0)	3340.2 (2903.7 to 3845.4)	2850.4 (2417.0 to 3346.0)	444.5 (384.9 to 508.9)	45.4 (23.4 to 76.5)	0.0 (0.0 to 0.0)
Kuwait	2026	1471.8 (1266.5 to 1697.2)	1259.1 (1057.8 to 1494.4)	192.8 (166.3 to 221.0)	19.8 (10.3 to 33.4)	0.0 (0.0 to 0.0)	3404.2 (2929.4 to 3925.7)	2912.3 (2446.7 to 3456.6)	446.0 (384.6 to 511.3)	45.8 (23.7 to 77.3)	0.0 (0.0 to 0.0)
Kuwait	2027	1496.0 (1274.8 to 1744.9)	1282.6 (1065.0 to 1531.1)	193.4 (166.4 to 221.8)	20.0 (10.4 to 33.8)	0.0 (0.0 to 0.0)	3460.3 (2948.7 to 4035.9)	2966.6 (2463.3 to 3541.6)	447.4 (384.9 to 513.0)	46.3 (24.0 to 78.1)	0.0 (0.0 to 0.0)
Kuwait	2028	1515.7 (1282.3 to 1775.2)	1301.9 (1067.0 to 1566.7)	193.8 (166.2 to 223.1)	20.1 (10.4 to 33.9)	0.0 (0.0 to 0.0)	3505.9 (2966.0 to 4105.9)	3011.2 (2467.9 to 3623.9)	448.2 (384.5 to 516.0)	46.5 (24.2 to 78.5)	0.0 (0.0 to 0.0)
Kuwait	2029	1527.7 (1285.6 to 1811.6)	1314.0 (1075.5 to 1592.4)	193.6 (164.7 to 223.5)	20.1 (10.5 to 33.9)	0.0 (0.0 to 0.0)	3533.6 (2973.6 to 4190.3)	3039.3 (2487.6 to 3683.3)	447.7 (380.9 to 517.0)	46.5 (24.2 to 78.3)	0.0 (0.0 to 0.0)
Kuwait	2030	1543.7 (1283.2 to 1840.3)	1329.9 (1065.7 to 1624.9)	193.7 (163.4 to 225.1)	20.1 (10.5 to 33.9)	0.0 (0.0 to 0.0)	3570.5 (2968.0 to 4256.7)	3076.0 (2465.0 to 3758.5)	447.9 (378.0 to 520.6)	46.5 (24.2 to 78.5)	0.0 (0.0 to 0.0)
Kuwait	2031	1561.2 (1290.7 to 1890.7)	1347.3 (1076.8 to 1667.8)	193.8 (162.3 to 227.0)	20.1 (10.5 to 34.0)	0.0 (0.0 to 0.0)	3611.1 (2985.5 to 4373.2)	3116.4 (2490.6 to 3857.6)	448.2 (375.3 to 525.0)	46.5 (24.3 to 78.7)	0.0 (0.0 to 0.0)
Kuwait	2032	1578.3 (1298.9 to 1916.6)	1364.3 (1091.2 to 1698.2)	193.9 (162.1 to 228.0)	20.1 (10.5 to 34.0)	0.0 (0.0 to 0.0)	3650.7 (3004.4 to 4433.2)	3155.7 (2523.9 to 3928.0)	448.5 (374.9 to 527.5)	46.5 (24.2 to 78.6)	0.0 (0.0 to 0.0)
Kuwait	2033	1599.8 (1306.4 to 1952.2)	1385.3 (1093.1 to 1736.5)	194.4 (160.3 to 230.4)	20.1 (10.5 to 33.9)	0.0 (0.0 to 0.0)	3700.3 (3021.8 to 4515.3)	3204.2 (2528.4 to 4016.5)	449.5 (370.8 to 532.9)	46.5 (24.2 to 78.4)	0.0 (0.0 to 0.0)
Kuwait	2034	1622.0 (1315.2 to 1999.4)	1406.9 (1102.3 to 1781.9)	194.9 (158.7 to 232.2)	20.1 (10.5 to 33.9)	0.0 (0.0 to 0.0)	3751.6 (3042.0 to 4624.6)	3254.2 (2549.5 to 4121.5)	450.8 (367.0 to 537.0)	46.6 (24.3 to 78.5)	0.0 (0.0 to 0.0)
Kuwait	2035	1641.3 (1320.2 to 2022.2)	1425.6 (1101.4 to 1811.9)	195.5 (157.9 to 234.7)	20.2 (10.5 to 34.0)	0.0 (0.0 to 0.0)	3796.4 (3053.7 to 4677.4)	3297.5 (2547.5 to 4191.0)	452.2 (365.2 to 542.9)	46.6 (24.3 to 78.6)	0.0 (0.0 to 0.0)
Kuwait	2036	1660.1 (1325.9 to 2067.5)	1443.7 (1103.8 to 1847.4)	196.1 (158.1 to 237.0)	20.2 (10.6 to 34.0)	0.0 (0.0 to 0.0)	3839.7 (3066.8 to 4782.2)	3339.4 (2553.2 to 4273.0)	453.7 (365.6 to 548.1)	46.7 (24.4 to 78.6)	0.0 (0.0 to 0.0)
Kuwait	2037	1676.6 (1321.0 to 2082.6)	1459.6 (1105.8 to 1867.0)	196.8 (157.8 to 239.3)	20.2 (10.6 to 33.9)	0.0 (0.0 to 0.0)	3878.1 (3055.5 to 4817.0)	3376.1 (2557.7 to 4318.4)	455.3 (365.0 to 553.6)	46.7 (24.5 to 78.4)	0.0 (0.0 to 0.0)
Kuwait	2038	1692.5 (1336.7 to 2114.0)	1474.7 (1110.2 to 1898.2)	197.7 (157.3 to 241.7)	20.2 (10.6 to 33.8)	0.0 (0.0 to 0.0)	3914.8 (3091.9 to 4889.7)	3410.9 (2567.9 to 4390.5)	457.2 (363.9 to 558.9)	46.7 (24.5 to 78.2)	0.0 (0.0 to 0.0)
Kuwait	2039	1707.4 (1331.1 to 2139.2)	1488.6 (1119.0 to 1933.9)	198.5 (157.4 to 244.1)	20.2 (10.6 to 33.7)	0.0 (0.0 to 0.0)	3949.1 (3078.9 to 4948.0)	3443.3 (2588.1 to 4473.1)	459.2 (364.2 to 564.6)	46.7 (24.5 to 78.0)	0.0 (0.0 to 0.0)
Kuwait	2040	1719.6 (1338.9 to 2164.4)	1499.9 (1115.3 to 1953.2)	199.5 (157.5 to 246.4)	20.2 (10.6 to 33.8)	0.0 (0.0 to 0.0)	3977.4 (3096.9 to 5006.2)	3469.3 (2579.6 to 4517.7)	461.4 (364.3 to 569.8)	46.7 (24.5 to 78.2)	0.0 (0.0 to 0.0)
Kuwait	2041	1722.2 (1338.1 to 2193.5)	1501.8 (1112.9 to 1952.9)	200.3 (157.6 to 248.3)	20.1 (10.6 to 33.7)	0.0 (0.0 to 0.0)	3983.4 (3095.0 to 5073.7)	3473.6 (2574.1 to 4517.1)	463.2 (364.4 to 574.2)	46.6 (24.5 to 77.9)	0.0 (0.0 to 0.0)
Kuwait	2042	1723.5 (1333.3 to 2219.0)	1502.2 (1101.0 to 1982.0)	201.2 (157.7 to 250.2)	20.1 (10.5 to 33.7)	0.0 (0.0 to 0.0)	3986.5 (3084.0 to 5132.7)	3474.6 (2546.5 to 4584.5)	465.3 (364.7 to 578.8)	46.5 (24.4 to 77.9)	0.0 (0.0 to 0.0)
Kuwait	2043	1721.8 (1325.5 to 2216.5)	1499.7 (1088.1 to 1985.3)	202.1 (158.2 to 252.1)	20.1 (10.5 to 33.7)	0.0 (0.0 to 0.0)	3982.6 (3066.0 to 5126.8)	3468.7 (2516.8 to 4592.0)	467.5 (365.8 to 583.0)	46.4 (24.2 to 78.0)	0.0 (0.0 to 0.0)

		2018 US Dollars per capita					2018 PPP per capita				
Country	Year	Health spending	Government health spending	Out-of-pocket health spending	Prepaid private health spending	Development assistance for health	Health spending	Government health spending	Out-of-pocket health spending	Prepaid private health spending	Development assistance for health
Kuwait	2044	1722.6 (1318.3 to 2229.4)	1499.4 (1084.5 to 1995.0)	203.2 (158.5 to 254.0)	20.0 (10.4 to 33.8)	0.0 (0.0 to 0.0)	3984.5 (3049.3 to 5156.6)	3468.1 (2508.5 to 4614.5)	470.0 (366.6 to 587.4)	46.3 (24.1 to 78.1)	0.0 (0.0 to 0.0)
Kuwait	2045	1723.9 (1309.7 to 2219.5)	1499.4 (1083.3 to 2010.5)	204.5 (159.0 to 256.1)	20.0 (10.4 to 33.8)	0.0 (0.0 to 0.0)	3987.3 (3029.4 to 5133.8)	3468.1 (2505.8 to 4650.2)	472.9 (367.8 to 592.5)	46.3 (23.9 to 78.2)	0.0 (0.0 to 0.0)
Kuwait	2046	1708.0 (1282.3 to 2223.2)	1482.9 (1068.7 to 1989.8)	205.3 (159.5 to 257.7)	19.9 (10.2 to 33.7)	0.0 (0.0 to 0.0)	3950.7 (2966.0 to 5142.2)	3429.9 (2472.0 to 4602.4)	474.8 (369.0 to 596.0)	46.0 (23.7 to 78.0)	0.0 (0.0 to 0.0)
Kuwait	2047	1694.7 (1277.9 to 2195.7)	1468.4 (1057.0 to 1968.3)	206.4 (160.3 to 259.6)	19.8 (10.2 to 33.7)	0.0 (0.0 to 0.0)	3919.7 (2955.9 to 5078.7)	3396.5 (2444.8 to 4552.7)	477.4 (370.7 to 600.5)	45.8 (23.5 to 77.9)	0.0 (0.0 to 0.0)
Kuwait	2048	1683.9 (1275.9 to 2183.3)	1456.5 (1046.6 to 1972.0)	207.6 (160.9 to 261.4)	19.8 (10.1 to 33.7)	0.0 (0.0 to 0.0)	3894.8 (2951.2 to 5050.0)	3368.9 (2420.8 to 4561.2)	480.2 (372.2 to 604.6)	45.7 (23.4 to 77.9)	0.0 (0.0 to 0.0)
Kuwait	2049	1664.4 (1264.2 to 2165.7)	1436.0 (1021.2 to 1946.5)	208.7 (161.1 to 263.6)	19.7 (10.1 to 33.6)	0.0 (0.0 to 0.0)	3849.7 (2924.1 to 5009.3)	3321.5 (2361.9 to 4502.2)	482.7 (372.6 to 609.7)	45.5 (23.3 to 77.6)	0.0 (0.0 to 0.0)
Kuwait	2050	1653.2 (1246.5 to 2152.7)	1423.5 (1007.0 to 1920.7)	210.0 (161.8 to 265.4)	19.6 (10.1 to 33.5)	0.0 (0.0 to 0.0)	3823.8 (2883.2 to 4979.3)	3292.6 (2329.1 to 4442.7)	485.8 (374.2 to 614.0)	45.4 (23.3 to 77.6)	0.0 (0.0 to 0.0)
Kyrgyzstan	1995	41.4 (34.2 to 49.8)	20.5 (16.0 to 26.1)	16.9 (11.6 to 23.3)	0.0 (0.0 to 0.0)	3.9 (3.9 to 3.9)	137.0 (113.4 to 164.9)	68.1 (53.1 to 86.3)	55.9 (38.5 to 77.3)	0.0 (0.0 to 0.0)	13.0 (13.0 to 13.0)
Kyrgyzstan	1996	41.4 (34.4 to 49.6)	19.9 (15.4 to 25.3)	17.6 (12.4 to 24.3)	0.0 (0.0 to 0.0)	3.9 (3.9 to 3.9)	137.1 (113.9 to 164.3)	66.0 (51.1 to 83.7)	58.3 (41.2 to 80.6)	0.0 (0.0 to 0.0)	12.8 (12.8 to 12.8)
Kyrgyzstan	1997	42.0 (35.0 to 50.5)	19.6 (15.1 to 24.5)	17.8 (12.7 to 24.2)	0.0 (0.0 to 0.0)	4.7 (4.7 to 4.7)	139.2 (115.8 to 167.5)	64.8 (50.1 to 81.3)	58.9 (42.0 to 80.2)	0.0 (0.0 to 0.0)	15.4 (15.4 to 15.4)
Kyrgyzstan	1998	45.1 (38.2 to 53.1)	18.0 (13.9 to 22.8)	18.5 (13.3 to 25.0)	0.0 (0.0 to 0.0)	8.6 (8.6 to 8.6)	149.5 (126.7 to 176.1)	59.8 (46.2 to 75.7)	61.3 (44.1 to 82.7)	0.0 (0.0 to 0.0)	28.4 (28.4 to 28.4)
Kyrgyzstan	1999	37.0 (30.5 to 45.0)	17.2 (13.1 to 21.8)	17.9 (12.9 to 24.1)	0.0 (0.0 to 0.0)	1.9 (1.9 to 1.9)	122.7 (101.0 to 149.1)	57.1 (43.6 to 72.3)	59.3 (42.9 to 80.0)	0.0 (0.0 to 0.1)	6.2 (6.2 to 6.2)
Kyrgyzstan	2000	39.3 (33.3 to 47.4)	16.3 (12.5 to 20.7)	17.2 (12.7 to 23.5)	0.0 (0.0 to 0.0)	5.8 (5.8 to 5.8)	130.3 (110.5 to 157.1)	54.0 (41.3 to 68.6)	57.1 (42.0 to 77.7)	0.0 (0.0 to 0.1)	19.2 (19.2 to 19.2)
Kyrgyzstan	2001	35.3 (29.5 to 43.3)	16.2 (12.4 to 20.5)	17.5 (12.8 to 24.0)	0.0 (0.0 to 0.0)	1.5 (1.5 to 1.5)	117.0 (97.7 to 143.6)	53.7 (41.0 to 67.9)	58.1 (42.4 to 79.4)	0.0 (0.0 to 0.1)	5.1 (5.1 to 5.1)
Kyrgyzstan	2002	37.9 (31.4 to 45.9)	16.6 (12.7 to 20.8)	18.4 (13.4 to 25.0)	0.0 (0.0 to 0.0)	3.0 (3.0 to 3.0)	125.7 (104.1 to 152.2)	54.9 (42.2 to 69.0)	60.9 (44.5 to 82.7)	0.0 (0.0 to 0.1)	9.9 (9.9 to 9.9)
Kyrgyzstan	2003	45.4 (38.1 to 54.3)	18.6 (14.3 to 23.5)	21.0 (15.2 to 28.2)	0.0 (0.0 to 0.0)	5.8 (5.8 to 5.8)	150.4 (126.1 to 179.8)	61.6 (47.3 to 77.7)	69.6 (50.5 to 93.6)	0.0 (0.0 to 0.1)	19.1 (19.1 to 19.1)
Kyrgyzstan	2004	48.1 (40.0 to 57.7)	21.5 (16.5 to 27.3)	23.1 (17.1 to 30.6)	0.0 (0.0 to 0.0)	3.5 (3.5 to 3.5)	159.5 (132.7 to 191.2)	71.1 (54.8 to 90.5)	76.6 (56.7 to 101.5)	0.1 (0.0 to 0.1)	11.7 (11.7 to 11.7)
Kyrgyzstan	2005	55.9 (47.0 to 66.5)	24.9 (19.3 to 31.9)	24.8 (18.3 to 32.9)	0.0 (0.0 to 0.0)	6.2 (6.2 to 6.2)	185.1 (155.9 to 220.2)	82.6 (64.0 to 105.6)	82.0 (60.5 to 109.1)	0.1 (0.0 to 0.1)	20.4 (20.4 to 20.4)
Kyrgyzstan	2006	60.0 (50.6 to 70.9)	27.6 (21.4 to 35.6)	26.0 (19.4 to 34.0)	0.0 (0.0 to 0.0)	6.4 (6.4 to 6.4)	198.7 (167.7 to 235.1)	91.4 (70.9 to 117.9)	86.2 (64.2 to 112.5)	0.1 (0.0 to 0.1)	21.1 (21.1 to 21.1)
Kyrgyzstan	2007	62.2 (52.2 to 73.5)	28.6 (22.2 to 36.3)	26.8 (19.9 to 35.0)	0.0 (0.0 to 0.0)	6.8 (6.8 to 6.8)	206.0 (172.9 to 243.4)	94.7 (73.6 to 120.4)	88.7 (65.9 to 115.8)	0.1 (0.0 to 0.1)	22.7 (22.7 to 22.7)
Kyrgyzstan	2008	66.9 (56.4 to 78.6)	30.0 (23.2 to 38.5)	27.9 (20.8 to 36.7)	0.0 (0.0 to 0.0)	9.0 (9.0 to 9.0)	221.7 (186.9 to 260.6)	99.5 (76.9 to 127.5)	92.5 (68.8 to 121.7)	0.1 (0.0 to 0.1)	29.7 (29.7 to 29.7)

Country	Year	2018 US Dollars per capita					2018 PPP per capita				
		Health spending	Government health spending	Out-of-pocket health spending	Prepaid private health spending	Development assistance for health	Health spending	Government health spending	Out-of-pocket health spending	Prepaid private health spending	Development assistance for health
Kyrgyzstan	2009	66.9 (56.6 to 79.2)	32.1 (24.8 to 41.3)	28.0 (21.0 to 36.7)	0.0 (0.0 to 0.0)	6.8 (6.8 to 6.8)	221.7 (187.4 to 262.5)	106.3 (82.1 to 137.0)	92.9 (69.6 to 121.5)	0.1 (0.0 to 0.1)	22.4 (22.4 to 22.4)
Kyrgyzstan	2010	70.0 (59.4 to 83.3)	32.9 (25.3 to 42.2)	29.1 (21.5 to 38.1)	0.0 (0.0 to 0.0)	7.9 (7.9 to 7.9)	231.8 (196.9 to 276.0)	109.1 (83.7 to 139.9)	96.4 (71.2 to 126.3)	0.1 (0.0 to 0.1)	26.3 (26.3 to 26.3)
Kyrgyzstan	2011	78.6 (66.9 to 93.0)	37.0 (28.7 to 47.4)	32.4 (24.1 to 42.2)	0.0 (0.0 to 0.1)	9.3 (9.3 to 9.3)	260.5 (221.5 to 308.0)	122.5 (95.1 to 156.9)	107.2 (79.8 to 139.8)	0.1 (0.0 to 0.2)	30.7 (30.7 to 30.7)
Kyrgyzstan	2012	80.2 (68.3 to 94.9)	37.2 (28.7 to 47.2)	35.0 (26.3 to 45.8)	0.0 (0.0 to 0.1)	8.0 (8.0 to 8.0)	265.8 (226.2 to 314.3)	123.3 (95.1 to 156.4)	115.9 (87.1 to 151.6)	0.1 (0.0 to 0.2)	26.5 (26.5 to 26.5)
Kyrgyzstan	2013	85.7 (73.0 to 101.0)	38.7 (29.9 to 48.9)	38.9 (29.1 to 51.0)	0.0 (0.0 to 0.1)	8.1 (8.1 to 8.1)	283.9 (241.7 to 334.5)	128.1 (99.0 to 162.2)	128.9 (96.5 to 168.8)	0.1 (0.0 to 0.2)	26.9 (26.9 to 26.9)
Kyrgyzstan	2014	86.5 (73.3 to 102.3)	36.7 (28.7 to 46.0)	41.3 (30.6 to 54.6)	0.0 (0.0 to 0.0)	8.5 (8.5 to 8.5)	286.7 (242.8 to 339.1)	121.8 (94.9 to 152.5)	136.9 (101.3 to 181.0)	0.1 (0.0 to 0.2)	28.0 (28.0 to 28.0)
Kyrgyzstan	2015	82.1 (69.1 to 97.8)	33.1 (25.8 to 41.5)	41.7 (30.4 to 55.6)	0.0 (0.0 to 0.0)	7.2 (7.2 to 7.2)	272.0 (229.0 to 324.1)	109.8 (85.4 to 137.6)	138.2 (100.8 to 184.1)	0.1 (0.0 to 0.1)	24.0 (24.0 to 24.0)
Kyrgyzstan	2016	79.1 (65.4 to 96.1)	31.6 (24.5 to 39.6)	41.6 (30.2 to 55.9)	0.0 (0.0 to 0.0)	5.9 (5.9 to 5.9)	262.2 (216.7 to 318.5)	104.6 (81.3 to 131.1)	137.9 (99.9 to 185.3)	0.1 (0.0 to 0.1)	19.6 (19.6 to 19.6)
Kyrgyzstan	2017	83.9 (69.4 to 101.4)	33.6 (26.1 to 42.6)	43.1 (31.2 to 57.9)	0.0 (0.0 to 0.0)	7.2 (7.2 to 7.2)	278.0 (229.9 to 335.9)	111.2 (86.5 to 141.3)	142.7 (103.4 to 191.9)	0.1 (0.0 to 0.1)	23.9 (23.9 to 23.9)
Kyrgyzstan	2018	85.2 (70.8 to 102.8)	34.2 (26.7 to 43.1)	44.2 (32.2 to 59.4)	0.0 (0.0 to 0.0)	6.8 (6.8 to 6.8)	282.4 (234.4 to 340.5)	113.2 (88.3 to 142.9)	146.6 (106.6 to 196.8)	0.1 (0.0 to 0.1)	22.5 (22.5 to 22.5)
Kyrgyzstan	2019	87.4 (72.3 to 105.6)	35.1 (27.3 to 44.4)	45.4 (32.9 to 60.7)	0.0 (0.0 to 0.0)	6.8 (6.4 to 7.2)	289.4 (239.7 to 349.7)	116.2 (90.6 to 147.2)	150.6 (109.1 to 201.2)	0.1 (0.0 to 0.1)	22.6 (21.1 to 23.9)
Kyrgyzstan	2020	89.3 (73.8 to 107.4)	35.8 (27.7 to 45.5)	46.5 (33.8 to 62.1)	0.0 (0.0 to 0.0)	6.9 (6.3 to 7.5)	295.9 (244.5 to 355.8)	118.8 (91.9 to 150.7)	154.2 (111.9 to 205.9)	0.1 (0.0 to 0.1)	22.9 (21.0 to 24.7)
Kyrgyzstan	2021	91.3 (75.7 to 109.2)	36.8 (28.5 to 46.3)	47.4 (34.4 to 63.2)	0.0 (0.0 to 0.0)	7.0 (6.3 to 7.7)	302.4 (250.8 to 361.8)	121.9 (94.5 to 153.6)	157.2 (113.8 to 209.4)	0.1 (0.0 to 0.1)	23.2 (20.9 to 25.7)
Kyrgyzstan	2022	92.7 (77.5 to 111.7)	37.4 (29.1 to 47.3)	48.1 (35.0 to 64.3)	0.0 (0.0 to 0.0)	7.2 (6.4 to 8.0)	307.3 (256.8 to 370.2)	124.0 (96.3 to 156.8)	159.5 (116.0 to 213.0)	0.1 (0.0 to 0.1)	23.7 (21.1 to 26.5)
Kyrgyzstan	2023	94.2 (78.5 to 113.0)	38.0 (29.6 to 48.1)	48.8 (35.5 to 65.4)	0.0 (0.0 to 0.0)	7.3 (6.4 to 8.3)	312.0 (260.1 to 374.5)	125.9 (98.0 to 159.3)	161.8 (117.7 to 216.6)	0.1 (0.0 to 0.2)	24.3 (21.2 to 27.6)
Kyrgyzstan	2024	95.7 (79.6 to 114.7)	38.5 (30.0 to 48.9)	49.6 (36.1 to 66.3)	0.0 (0.0 to 0.0)	7.5 (6.5 to 8.7)	317.0 (263.7 to 380.0)	127.7 (99.3 to 162.1)	164.3 (119.6 to 219.7)	0.1 (0.0 to 0.2)	24.9 (21.5 to 28.7)
Kyrgyzstan	2025	97.4 (80.6 to 117.3)	39.2 (30.4 to 49.7)	50.4 (36.6 to 67.7)	0.0 (0.0 to 0.0)	7.7 (6.6 to 9.0)	322.6 (267.2 to 388.8)	129.9 (100.7 to 164.8)	167.0 (121.3 to 224.3)	0.1 (0.0 to 0.2)	25.5 (21.8 to 29.9)
Kyrgyzstan	2026	99.3 (81.9 to 120.6)	40.1 (31.1 to 50.8)	51.3 (37.0 to 69.0)	0.0 (0.0 to 0.0)	7.9 (6.7 to 9.3)	328.9 (271.4 to 399.6)	132.7 (102.9 to 168.4)	169.9 (122.6 to 228.7)	0.1 (0.0 to 0.2)	26.2 (22.1 to 30.7)
Kyrgyzstan	2027	101.4 (83.7 to 122.7)	41.0 (31.6 to 52.2)	52.2 (37.7 to 70.7)	0.0 (0.0 to 0.0)	8.1 (6.8 to 9.6)	335.9 (277.2 to 406.7)	135.9 (104.6 to 172.8)	173.1 (124.8 to 234.4)	0.1 (0.0 to 0.2)	26.8 (22.5 to 32.0)
Kyrgyzstan	2028	103.7 (84.8 to 124.9)	42.1 (32.3 to 53.9)	53.3 (38.0 to 72.5)	0.0 (0.0 to 0.0)	8.3 (7.0 to 10.0)	343.5 (280.9 to 413.9)	139.4 (106.9 to 178.5)	176.5 (126.0 to 240.1)	0.1 (0.0 to 0.2)	27.6 (23.1 to 33.3)
Kyrgyzstan	2029	106.1 (86.5 to 129.7)	43.2 (33.0 to 55.2)	54.3 (38.5 to 74.1)	0.0 (0.0 to 0.1)	8.5 (7.0 to 10.4)	351.5 (286.6 to 429.9)	143.1 (109.3 to 183.0)	180.0 (127.6 to 245.4)	0.1 (0.0 to 0.2)	28.3 (23.3 to 34.5)

		2018 US Dollars per capita					2018 PPP per capita				
Country	Year	Health spending	Government health spending	Out-of-pocket health spending	Prepaid private health spending	Development assistance for health	Health spending	Government health spending	Out-of-pocket health spending	Prepaid private health spending	Development assistance for health
Kyrgyzstan	2030	108.5 (88.6 to 132.4)	44.3 (33.7 to 56.7)	55.4 (39.1 to 75.7)	0.0 (0.0 to 0.1)	8.7 (7.1 to 10.7)	359.4 (293.4 to 438.9)	146.7 (111.6 to 187.8)	183.7 (129.6 to 250.9)	0.1 (0.0 to 0.2)	28.9 (23.7 to 35.5)
Kyrgyzstan	2031	110.9 (89.5 to 134.8)	45.4 (34.4 to 58.6)	56.6 (39.5 to 77.8)	0.0 (0.0 to 0.1)	8.9 (7.2 to 11.2)	367.6 (296.6 to 446.7)	150.4 (113.9 to 194.0)	187.5 (131.0 to 257.8)	0.1 (0.0 to 0.2)	29.6 (24.0 to 37.1)
Kyrgyzstan	2032	113.5 (92.0 to 139.0)	46.5 (35.3 to 59.8)	57.8 (40.2 to 80.0)	0.0 (0.0 to 0.1)	9.1 (7.3 to 11.5)	376.0 (304.9 to 460.5)	154.1 (117.0 to 198.1)	191.4 (133.1 to 265.0)	0.1 (0.0 to 0.2)	30.3 (24.3 to 38.1)
Kyrgyzstan	2033	116.1 (93.9 to 142.4)	47.7 (36.1 to 61.1)	59.0 (40.9 to 82.2)	0.0 (0.0 to 0.1)	9.4 (7.4 to 12.0)	384.6 (311.1 to 471.8)	157.9 (119.7 to 202.4)	195.5 (135.6 to 272.5)	0.1 (0.1 to 0.2)	31.0 (24.6 to 39.8)
Kyrgyzstan	2034	118.8 (95.8 to 145.7)	48.9 (36.9 to 62.9)	60.3 (41.4 to 84.0)	0.0 (0.0 to 0.1)	9.6 (7.6 to 12.4)	393.6 (317.5 to 482.8)	161.9 (122.4 to 208.3)	199.7 (137.2 to 278.3)	0.1 (0.1 to 0.2)	31.8 (25.0 to 40.9)
Kyrgyzstan	2035	121.4 (97.2 to 148.6)	50.0 (37.7 to 64.6)	61.5 (41.9 to 85.9)	0.0 (0.0 to 0.1)	9.8 (7.6 to 12.9)	402.1 (322.2 to 492.4)	165.6 (125.0 to 214.2)	203.8 (138.8 to 284.7)	0.1 (0.1 to 0.2)	32.6 (25.3 to 42.7)
Kyrgyzstan	2036	123.9 (98.8 to 152.5)	51.1 (38.4 to 66.3)	62.7 (42.5 to 88.1)	0.0 (0.0 to 0.1)	10.1 (7.8 to 13.5)	410.6 (327.3 to 505.2)	169.3 (127.1 to 219.8)	207.9 (141.0 to 291.8)	0.1 (0.1 to 0.2)	33.3 (25.8 to 44.8)
Kyrgyzstan	2037	126.4 (99.6 to 155.7)	52.1 (39.1 to 67.5)	63.9 (43.2 to 90.1)	0.0 (0.0 to 0.1)	10.3 (7.9 to 13.8)	418.8 (330.0 to 516.0)	172.8 (129.4 to 223.5)	211.8 (143.2 to 298.7)	0.1 (0.1 to 0.2)	34.1 (26.2 to 45.7)
Kyrgyzstan	2038	128.8 (102.3 to 159.5)	53.2 (39.7 to 69.3)	65.1 (43.9 to 91.8)	0.0 (0.0 to 0.1)	10.5 (8.0 to 14.6)	426.9 (339.0 to 528.6)	176.2 (131.6 to 229.6)	215.7 (145.3 to 304.0)	0.1 (0.1 to 0.2)	34.9 (26.6 to 48.5)
Kyrgyzstan	2039	131.3 (105.2 to 162.0)	54.2 (40.2 to 70.7)	66.3 (44.4 to 93.3)	0.0 (0.0 to 0.1)	10.8 (8.2 to 15.0)	435.0 (348.5 to 536.8)	179.6 (133.1 to 234.2)	219.5 (147.1 to 309.2)	0.1 (0.1 to 0.2)	35.7 (27.3 to 49.6)
Kyrgyzstan	2040	133.6 (105.8 to 163.2)	55.2 (41.2 to 71.7)	67.4 (44.9 to 95.2)	0.0 (0.0 to 0.1)	11.0 (8.4 to 15.3)	442.8 (350.7 to 540.8)	182.8 (136.4 to 237.5)	223.3 (148.8 to 315.5)	0.1 (0.1 to 0.2)	36.6 (27.9 to 50.6)
Kyrgyzstan	2041	136.0 (108.2 to 167.9)	56.1 (41.7 to 73.0)	68.5 (45.5 to 96.6)	0.0 (0.0 to 0.1)	11.3 (8.5 to 15.7)	450.6 (358.6 to 556.5)	185.9 (138.2 to 242.0)	227.0 (150.7 to 320.0)	0.1 (0.1 to 0.2)	37.5 (28.3 to 52.1)
Kyrgyzstan	2042	138.3 (109.7 to 170.8)	57.0 (42.3 to 74.3)	69.6 (46.1 to 97.9)	0.0 (0.0 to 0.1)	11.6 (8.7 to 16.6)	458.2 (363.6 to 565.8)	189.0 (140.3 to 246.0)	230.6 (152.6 to 324.4)	0.1 (0.1 to 0.2)	38.5 (28.8 to 54.9)
Kyrgyzstan	2043	140.5 (111.6 to 174.2)	57.9 (42.8 to 75.8)	70.6 (46.7 to 99.7)	0.0 (0.0 to 0.1)	11.9 (8.8 to 17.2)	465.6 (369.8 to 577.2)	192.0 (142.0 to 251.3)	234.1 (154.6 to 330.4)	0.1 (0.1 to 0.2)	39.4 (29.3 to 57.0)
Kyrgyzstan	2044	142.8 (113.1 to 175.5)	58.9 (43.4 to 77.7)	71.7 (47.3 to 101.5)	0.0 (0.0 to 0.1)	12.2 (9.0 to 17.7)	473.2 (374.7 to 581.7)	195.1 (143.8 to 257.3)	237.5 (156.8 to 336.3)	0.1 (0.1 to 0.2)	40.5 (29.9 to 58.6)
Kyrgyzstan	2045	145.1 (115.3 to 178.0)	59.9 (44.1 to 78.9)	72.7 (47.9 to 103.2)	0.0 (0.0 to 0.1)	12.5 (9.0 to 18.9)	480.8 (382.0 to 589.9)	198.4 (146.2 to 261.6)	240.8 (158.8 to 342.0)	0.1 (0.1 to 0.2)	41.5 (29.9 to 62.7)
Kyrgyzstan	2046	147.4 (116.9 to 182.0)	60.9 (44.6 to 80.1)	73.6 (48.5 to 104.6)	0.0 (0.0 to 0.1)	12.9 (9.3 to 19.8)	488.5 (387.3 to 603.1)	201.8 (147.8 to 265.4)	244.0 (160.6 to 346.8)	0.1 (0.1 to 0.2)	42.7 (30.8 to 65.5)
Kyrgyzstan	2047	149.7 (117.9 to 185.7)	61.9 (45.4 to 81.7)	74.5 (49.1 to 105.7)	0.0 (0.0 to 0.1)	13.2 (9.5 to 20.9)	496.0 (390.8 to 615.4)	205.0 (150.6 to 270.6)	247.0 (162.8 to 350.2)	0.1 (0.1 to 0.2)	43.9 (31.5 to 69.1)
Kyrgyzstan	2048	151.9 (119.0 to 188.6)	62.8 (46.1 to 82.5)	75.4 (49.7 to 107.2)	0.0 (0.0 to 0.1)	13.6 (9.8 to 21.8)	503.3 (394.2 to 624.8)	208.0 (152.8 to 273.2)	250.0 (164.7 to 355.3)	0.1 (0.1 to 0.2)	45.2 (32.4 to 72.3)
Kyrgyzstan	2049	154.2 (121.0 to 192.0)	63.8 (47.1 to 84.3)	76.3 (50.3 to 108.7)	0.0 (0.0 to 0.1)	14.1 (10.0 to 22.6)	511.0 (400.8 to 636.2)	211.4 (156.1 to 279.3)	252.9 (166.6 to 360.3)	0.1 (0.1 to 0.2)	46.6 (33.2 to 74.8)
Kyrgyzstan	2050	156.6 (122.0 to 194.9)	64.9 (47.5 to 85.6)	77.2 (50.8 to 110.2)	0.0 (0.0 to 0.1)	14.5 (10.2 to 23.8)	518.9 (404.2 to 645.9)	215.0 (157.5 to 283.6)	255.7 (168.3 to 365.1)	0.1 (0.1 to 0.3)	48.1 (33.8 to 78.7)

Country	Year	2018 US Dollars per capita					2018 PPP per capita				
		Health spending	Government health spending	Out-of-pocket health spending	Prepaid private health spending	Development assistance for health	Health spending	Government health spending	Out-of-pocket health spending	Prepaid private health spending	Development assistance for health
Laos	1995	31.9 (25.9 to 39.0)	11.7 (8.8 to 15.1)	16.2 (11.6 to 22.4)	1.7 (0.8 to 3.2)	2.2 (2.2 to 2.2)	96.9 (78.8 to 118.4)	35.7 (26.8 to 45.8)	49.2 (35.3 to 68.2)	5.3 (2.6 to 9.8)	6.7 (6.7 to 6.7)
Laos	1996	31.1 (25.0 to 38.3)	11.7 (8.8 to 15.0)	16.4 (11.7 to 22.6)	1.8 (0.9 to 3.3)	1.3 (1.3 to 1.3)	94.6 (76.0 to 116.4)	35.5 (26.7 to 45.5)	49.8 (35.7 to 68.6)	5.4 (2.6 to 9.9)	3.9 (3.9 to 3.9)
Laos	1997	31.8 (25.5 to 39.2)	11.7 (8.8 to 15.0)	16.7 (11.9 to 23.0)	1.9 (0.9 to 3.6)	1.5 (1.5 to 1.5)	96.6 (77.5 to 119.0)	35.5 (26.7 to 45.5)	50.9 (36.0 to 70.0)	5.8 (2.8 to 10.9)	4.4 (4.4 to 4.4)
Laos	1998	32.0 (25.6 to 39.1)	11.3 (8.5 to 14.5)	16.9 (11.8 to 23.0)	2.0 (1.0 to 3.8)	1.9 (1.9 to 1.9)	97.4 (77.9 to 118.8)	34.3 (25.9 to 44.1)	51.2 (35.8 to 69.9)	6.2 (3.0 to 11.4)	5.7 (5.7 to 5.7)
Laos	1999	32.7 (26.1 to 39.6)	10.9 (8.2 to 13.8)	16.9 (11.9 to 23.3)	2.3 (1.1 to 4.1)	2.6 (2.6 to 2.6)	99.5 (79.3 to 120.4)	33.0 (24.9 to 42.0)	51.5 (36.1 to 70.7)	7.0 (3.3 to 12.6)	8.0 (8.0 to 8.0)
Laos	2000	35.9 (29.1 to 43.4)	10.7 (8.1 to 13.7)	17.2 (11.9 to 23.9)	3.0 (1.4 to 5.4)	5.0 (5.0 to 5.0)	109.1 (88.5 to 131.7)	32.5 (24.7 to 41.6)	52.2 (36.3 to 72.6)	9.2 (4.3 to 16.5)	15.3 (15.3 to 15.3)
Laos	2001	33.8 (26.8 to 41.2)	10.8 (8.2 to 13.8)	17.7 (12.5 to 24.5)	2.7 (1.3 to 4.9)	2.6 (2.6 to 2.6)	102.6 (81.5 to 125.1)	32.7 (24.9 to 41.9)	53.8 (37.9 to 74.4)	8.3 (4.0 to 14.8)	7.8 (7.8 to 7.8)
Laos	2002	33.9 (27.1 to 41.8)	10.3 (7.9 to 13.3)	18.4 (13.0 to 25.4)	2.9 (1.4 to 5.2)	2.3 (2.3 to 2.3)	103.1 (82.4 to 127.0)	31.3 (23.9 to 40.3)	56.0 (39.4 to 77.1)	8.8 (4.2 to 15.7)	7.0 (7.0 to 7.0)
Laos	2003	38.8 (31.9 to 47.0)	10.0 (7.7 to 12.9)	19.4 (13.7 to 27.0)	3.2 (1.5 to 5.6)	6.2 (6.2 to 6.2)	117.9 (97.0 to 142.8)	30.4 (23.3 to 39.2)	59.0 (41.6 to 81.9)	9.6 (4.7 to 17.0)	18.8 (18.8 to 18.8)
Laos	2004	34.9 (28.7 to 42.7)	9.3 (7.1 to 12.1)	17.8 (12.7 to 24.7)	3.4 (1.7 to 6.0)	4.4 (4.4 to 4.4)	106.0 (87.1 to 129.7)	28.3 (21.7 to 36.7)	54.2 (38.5 to 75.0)	10.2 (5.1 to 18.1)	13.3 (13.3 to 13.3)
Laos	2005	36.0 (30.2 to 43.5)	9.0 (6.9 to 11.6)	16.4 (11.7 to 22.5)	3.7 (1.8 to 6.7)	6.9 (6.9 to 6.9)	109.4 (91.8 to 132.1)	27.3 (20.9 to 35.4)	49.9 (35.7 to 68.5)	11.3 (5.6 to 20.4)	21.0 (21.0 to 21.0)
Laos	2006	33.6 (28.0 to 40.3)	8.4 (6.4 to 10.9)	15.1 (10.9 to 20.6)	4.3 (2.1 to 8.0)	5.8 (5.8 to 5.8)	102.0 (85.0 to 122.5)	25.5 (19.5 to 33.1)	45.8 (33.0 to 62.6)	13.0 (6.5 to 24.2)	17.7 (17.7 to 17.7)
Laos	2007	35.4 (29.5 to 42.7)	8.2 (6.3 to 10.6)	14.3 (10.3 to 19.4)	5.9 (3.0 to 11.0)	6.9 (6.9 to 6.9)	107.5 (89.7 to 129.8)	25.0 (19.0 to 32.1)	43.6 (31.2 to 59.1)	17.9 (9.0 to 33.4)	21.0 (21.0 to 21.0)
Laos	2008	36.7 (30.8 to 44.0)	8.5 (6.4 to 10.9)	13.7 (9.8 to 18.7)	6.6 (3.3 to 12.3)	7.9 (7.9 to 7.9)	111.4 (93.6 to 133.6)	25.7 (19.6 to 33.1)	41.7 (29.9 to 56.7)	20.1 (10.0 to 37.3)	23.9 (23.9 to 23.9)
Laos	2009	39.1 (33.1 to 46.9)	9.9 (7.6 to 12.8)	14.8 (10.6 to 20.3)	6.4 (3.2 to 12.0)	7.9 (7.9 to 7.9)	118.8 (100.5 to 142.4)	30.2 (23.0 to 38.9)	44.9 (32.1 to 61.6)	19.5 (9.6 to 36.4)	24.1 (24.1 to 24.1)
Laos	2010	37.8 (32.4 to 44.6)	9.0 (6.9 to 11.7)	15.4 (11.1 to 21.0)	4.0 (1.9 to 7.2)	9.5 (9.5 to 9.5)	115.0 (98.3 to 135.5)	27.3 (20.8 to 35.4)	46.7 (33.8 to 63.8)	12.2 (5.8 to 22.0)	28.8 (28.8 to 28.8)
Laos	2011	36.2 (31.0 to 42.5)	8.4 (6.4 to 10.9)	15.8 (11.3 to 22.1)	1.5 (0.7 to 2.7)	10.5 (10.5 to 10.5)	110.0 (94.3 to 129.1)	25.5 (19.4 to 33.1)	48.2 (34.4 to 67.0)	4.4 (2.1 to 8.1)	32.0 (32.0 to 32.0)
Laos	2012	38.3 (32.6 to 45.4)	9.3 (7.1 to 12.2)	17.5 (12.6 to 24.2)	1.3 (0.7 to 2.4)	10.1 (10.1 to 10.1)	116.4 (99.1 to 137.9)	28.3 (21.7 to 37.1)	53.3 (38.2 to 73.5)	4.1 (2.0 to 7.3)	30.7 (30.7 to 30.7)
Laos	2013	44.7 (38.3 to 52.7)	11.5 (8.7 to 15.0)	20.1 (14.4 to 27.8)	1.4 (0.7 to 2.4)	11.7 (11.7 to 11.7)	135.8 (116.4 to 160.2)	35.0 (26.5 to 45.6)	61.2 (43.8 to 84.4)	4.1 (2.0 to 7.3)	35.4 (35.4 to 35.4)
Laos	2014	48.1 (40.6 to 58.0)	13.6 (10.3 to 17.7)	22.4 (15.9 to 31.3)	1.4 (0.7 to 2.6)	10.7 (10.7 to 10.7)	146.2 (123.5 to 176.3)	41.3 (31.3 to 53.7)	68.1 (48.2 to 95.1)	4.4 (2.2 to 7.8)	32.4 (32.4 to 32.4)
Laos	2015	53.0 (44.8 to 63.9)	16.0 (12.3 to 20.8)	24.2 (17.1 to 34.0)	1.6 (0.8 to 2.8)	11.1 (11.1 to 11.1)	160.9 (136.3 to 194.2)	48.7 (37.3 to 63.1)	73.7 (52.1 to 103.4)	4.8 (2.4 to 8.5)	33.7 (33.7 to 33.7)

Country	Year	2018 US Dollars per capita					2018 PPP per capita				
		Health spending	Government health spending	Out-of-pocket health spending	Prepaid private health spending	Development assistance for health	Health spending	Government health spending	Out-of-pocket health spending	Prepaid private health spending	Development assistance for health
Laos	2016	51.7 (42.6 to 62.2)	17.2 (13.1 to 22.5)	25.4 (18.1 to 35.7)	1.7 (0.9 to 3.1)	7.4 (7.4 to 7.4)	157.2 (129.6 to 189.1)	52.4 (39.8 to 68.5)	77.2 (54.9 to 108.4)	5.3 (2.6 to 9.5)	22.4 (22.4 to 22.4)
Laos	2017	55.5 (45.9 to 67.4)	18.7 (14.2 to 24.5)	26.6 (19.0 to 37.6)	1.8 (0.9 to 3.3)	8.4 (8.4 to 8.4)	168.7 (139.6 to 204.7)	56.8 (43.2 to 74.4)	80.8 (57.7 to 114.1)	5.6 (2.8 to 10.0)	25.4 (25.4 to 25.4)
Laos	2018	56.7 (47.0 to 68.0)	19.5 (14.8 to 25.5)	27.5 (19.6 to 38.6)	1.9 (1.0 to 3.4)	7.8 (7.7 to 7.8)	172.3 (143.0 to 206.5)	59.3 (45.1 to 77.6)	83.5 (59.4 to 117.2)	5.9 (2.9 to 10.5)	23.6 (23.5 to 23.7)
Laos	2019	58.4 (48.0 to 71.0)	20.3 (15.5 to 26.6)	28.4 (20.2 to 39.6)	2.0 (1.0 to 3.6)	7.7 (7.2 to 8.1)	177.5 (145.9 to 215.9)	61.8 (47.0 to 80.8)	86.3 (61.4 to 120.4)	6.1 (3.0 to 10.9)	23.4 (21.9 to 24.8)
Laos	2020	60.3 (49.9 to 72.5)	21.1 (16.1 to 27.6)	29.3 (20.9 to 41.1)	2.1 (1.0 to 3.7)	7.7 (7.0 to 8.3)	183.1 (151.6 to 220.2)	64.2 (49.0 to 84.0)	89.2 (63.5 to 125.0)	6.3 (3.1 to 11.3)	23.4 (21.4 to 25.3)
Laos	2021	62.3 (51.0 to 75.9)	22.1 (16.8 to 28.8)	30.4 (21.7 to 42.7)	2.2 (1.1 to 3.9)	7.7 (6.9 to 8.5)	189.4 (155.0 to 230.7)	67.0 (51.0 to 87.6)	92.3 (66.0 to 129.8)	6.6 (3.3 to 11.8)	23.5 (21.0 to 25.9)
Laos	2022	64.5 (53.0 to 77.8)	23.0 (17.5 to 30.1)	31.4 (22.4 to 44.2)	2.3 (1.1 to 4.0)	7.7 (6.8 to 8.7)	195.9 (161.1 to 236.3)	70.0 (53.0 to 91.6)	95.6 (68.2 to 134.4)	6.9 (3.4 to 12.3)	23.5 (20.8 to 26.5)
Laos	2023	66.6 (54.7 to 81.9)	24.0 (18.3 to 31.5)	32.5 (23.1 to 45.9)	2.3 (1.2 to 4.2)	7.7 (6.7 to 8.9)	202.5 (166.1 to 248.9)	73.0 (55.5 to 95.8)	98.8 (70.3 to 139.5)	7.1 (3.5 to 12.8)	23.5 (20.3 to 26.9)
Laos	2024	68.8 (56.6 to 83.5)	25.1 (19.0 to 32.9)	33.5 (23.8 to 47.4)	2.4 (1.2 to 4.4)	7.8 (6.6 to 9.0)	209.0 (172.1 to 253.6)	76.1 (57.8 to 99.9)	101.8 (72.3 to 143.9)	7.4 (3.7 to 13.3)	23.6 (20.1 to 27.3)
Laos	2025	71.0 (58.3 to 86.5)	26.1 (19.7 to 34.4)	34.6 (24.6 to 48.7)	2.5 (1.3 to 4.5)	7.8 (6.6 to 9.1)	215.8 (177.1 to 262.9)	79.4 (59.9 to 104.6)	105.0 (74.7 to 148.0)	7.7 (3.8 to 13.8)	23.6 (20.0 to 27.7)
Laos	2026	73.4 (60.5 to 89.2)	27.3 (20.6 to 35.9)	35.6 (25.4 to 50.2)	2.6 (1.3 to 4.7)	7.8 (6.5 to 9.2)	223.0 (183.8 to 271.1)	82.9 (62.5 to 109.0)	108.3 (77.1 to 152.5)	8.0 (3.9 to 14.4)	23.8 (19.9 to 28.1)
Laos	2027	75.9 (62.1 to 93.2)	28.5 (21.5 to 37.5)	36.8 (26.2 to 51.7)	2.7 (1.4 to 4.9)	7.9 (6.5 to 9.4)	230.7 (188.8 to 283.1)	86.6 (65.5 to 113.9)	111.7 (79.6 to 157.2)	8.3 (4.1 to 15.0)	24.0 (19.8 to 28.6)
Laos	2028	78.5 (64.2 to 94.6)	29.8 (22.5 to 39.1)	37.9 (27.0 to 53.4)	2.9 (1.4 to 5.1)	8.0 (6.6 to 9.7)	238.7 (195.1 to 287.6)	90.5 (68.5 to 118.7)	115.3 (82.2 to 162.3)	8.7 (4.3 to 15.6)	24.3 (20.1 to 29.6)
Laos	2029	81.2 (65.9 to 100.0)	31.1 (23.5 to 40.8)	39.1 (27.9 to 55.0)	3.0 (1.5 to 5.4)	8.1 (6.6 to 9.9)	246.9 (200.4 to 303.8)	94.5 (71.3 to 124.0)	118.9 (84.8 to 167.3)	9.0 (4.4 to 16.3)	24.5 (19.9 to 30.2)
Laos	2030	84.0 (68.6 to 101.5)	32.4 (24.5 to 42.4)	40.4 (28.8 to 56.8)	3.1 (1.5 to 5.6)	8.1 (6.6 to 10.1)	255.4 (208.3 to 308.4)	98.6 (74.4 to 128.9)	122.6 (87.4 to 172.8)	9.4 (4.6 to 17.0)	24.7 (20.0 to 30.6)
Laos	2031	86.9 (70.7 to 107.1)	33.9 (25.5 to 44.3)	41.6 (29.7 to 58.7)	3.2 (1.6 to 5.8)	8.2 (6.6 to 10.3)	264.2 (214.9 to 325.6)	103.0 (77.5 to 134.6)	126.5 (90.1 to 178.4)	9.8 (4.8 to 17.7)	25.0 (20.0 to 31.5)
Laos	2032	90.0 (73.5 to 108.5)	35.4 (26.6 to 46.3)	42.9 (30.6 to 60.7)	3.3 (1.7 to 6.1)	8.3 (6.6 to 10.7)	273.4 (223.2 to 329.6)	107.5 (80.7 to 140.7)	130.4 (92.9 to 184.4)	10.2 (5.0 to 18.4)	25.3 (20.0 to 32.4)
Laos	2033	93.1 (75.3 to 114.5)	36.9 (27.7 to 48.4)	44.3 (31.5 to 62.5)	3.5 (1.7 to 6.3)	8.4 (6.6 to 10.9)	282.8 (228.7 to 348.1)	112.2 (84.2 to 146.9)	134.5 (95.7 to 190.1)	10.6 (5.2 to 19.2)	25.6 (20.1 to 33.0)
Laos	2034	96.3 (79.1 to 115.9)	38.5 (28.9 to 50.5)	45.6 (32.4 to 64.4)	3.6 (1.8 to 6.6)	8.5 (6.6 to 11.2)	292.7 (240.4 to 352.3)	117.1 (87.8 to 153.4)	138.7 (98.5 to 195.8)	11.0 (5.4 to 19.9)	25.9 (20.2 to 34.1)
Laos	2035	99.7 (81.0 to 123.0)	40.2 (30.1 to 52.6)	47.0 (33.3 to 66.4)	3.8 (1.9 to 6.8)	8.6 (6.7 to 11.5)	302.8 (246.1 to 373.7)	122.3 (91.5 to 159.9)	142.9 (101.3 to 201.7)	11.4 (5.6 to 20.8)	26.2 (20.3 to 34.9)
Laos	2036	103.2 (84.9 to 124.2)	42.0 (31.4 to 55.1)	48.5 (34.3 to 68.5)	3.9 (1.9 to 7.1)	8.7 (6.6 to 11.8)	313.5 (257.9 to 377.3)	127.8 (95.5 to 167.3)	147.4 (104.3 to 208.0)	11.9 (5.8 to 21.6)	26.6 (20.1 to 35.9)

		2018 US Dollars per capita					2018 PPP per capita				
Country	Year	Health spending	Government health spending	Out-of-pocket health spending	Prepaid private health spending	Development assistance for health	Health spending	Government health spending	Out-of-pocket health spending	Prepaid private health spending	Development assistance for health
Laos	2037	106.8 (86.0 to 132.2)	43.9 (32.8 to 57.7)	50.0 (35.3 to 70.6)	4.1 (2.0 to 7.4)	8.8 (6.7 to 12.0)	324.5 (261.3 to 401.7)	133.4 (99.7 to 175.4)	151.9 (107.4 to 214.5)	12.3 (6.1 to 22.5)	26.9 (20.4 to 36.6)
Laos	2038	110.6 (90.4 to 133.4)	45.9 (34.2 to 60.4)	51.5 (36.4 to 72.8)	4.2 (2.1 to 7.7)	9.0 (6.8 to 12.3)	336.0 (274.7 to 405.4)	139.4 (103.9 to 183.6)	156.6 (110.5 to 221.2)	12.8 (6.3 to 23.5)	27.2 (20.6 to 37.3)
Laos	2039	114.4 (92.7 to 140.5)	47.9 (35.7 to 63.1)	53.1 (37.4 to 75.0)	4.4 (2.2 to 8.0)	9.1 (6.7 to 12.4)	347.7 (281.7 to 427.1)	145.5 (108.4 to 191.8)	161.4 (113.7 to 228.0)	13.3 (6.6 to 24.4)	27.5 (20.5 to 37.8)
Laos	2040	118.4 (96.9 to 143.1)	50.0 (37.2 to 66.0)	54.7 (38.5 to 77.3)	4.5 (2.2 to 8.3)	9.2 (6.9 to 12.8)	359.8 (294.3 to 435.0)	151.8 (113.0 to 200.6)	166.3 (117.1 to 234.9)	13.8 (6.8 to 25.3)	27.9 (20.8 to 39.0)
Laos	2041	122.6 (99.0 to 149.9)	52.2 (38.8 to 68.9)	56.4 (39.7 to 79.6)	4.7 (2.3 to 8.6)	9.3 (6.9 to 13.4)	372.5 (300.8 to 455.6)	158.5 (117.8 to 209.3)	171.3 (120.6 to 242.0)	14.4 (7.1 to 26.2)	28.3 (21.0 to 40.6)
Laos	2042	126.8 (103.6 to 154.1)	54.4 (40.3 to 72.1)	58.1 (40.9 to 82.0)	4.9 (2.4 to 8.9)	9.5 (6.9 to 13.7)	385.4 (315.0 to 468.2)	165.4 (122.6 to 219.1)	176.4 (124.2 to 249.3)	14.9 (7.3 to 27.2)	28.7 (21.0 to 41.6)
Laos	2043	131.3 (105.8 to 160.3)	56.8 (42.0 to 75.1)	59.8 (42.1 to 84.5)	5.1 (2.5 to 9.3)	9.6 (7.0 to 14.4)	398.9 (321.6 to 487.2)	172.5 (127.8 to 228.2)	181.7 (128.0 to 256.8)	15.5 (7.6 to 28.3)	29.2 (21.1 to 43.6)
Laos	2044	135.8 (110.1 to 164.2)	59.2 (43.5 to 78.7)	61.6 (43.3 to 87.0)	5.3 (2.6 to 9.7)	9.8 (7.0 to 14.7)	412.8 (334.7 to 498.9)	180.0 (132.1 to 239.2)	187.1 (131.7 to 264.4)	16.1 (7.9 to 29.5)	29.7 (21.3 to 44.5)
Laos	2045	140.5 (113.1 to 172.1)	61.8 (45.2 to 82.3)	63.3 (44.5 to 89.5)	5.5 (2.7 to 10.1)	9.9 (7.0 to 14.9)	427.0 (343.6 to 523.1)	187.7 (137.3 to 250.3)	192.4 (135.4 to 272.0)	16.7 (8.2 to 30.8)	30.2 (21.4 to 45.2)
Laos	2046	145.3 (118.6 to 176.1)	64.4 (46.8 to 85.8)	65.1 (45.8 to 92.0)	5.7 (2.8 to 10.5)	10.1 (7.2 to 15.5)	441.6 (360.4 to 535.2)	195.8 (142.3 to 260.9)	197.8 (139.2 to 279.7)	17.3 (8.4 to 31.8)	30.7 (21.8 to 47.0)
Laos	2047	150.2 (120.4 to 186.1)	67.2 (48.5 to 89.6)	66.9 (47.1 to 94.6)	5.9 (2.9 to 10.8)	10.3 (7.2 to 16.2)	456.6 (365.8 to 565.6)	204.1 (147.3 to 272.2)	203.2 (143.1 to 287.4)	17.9 (8.7 to 32.8)	31.3 (22.0 to 49.3)
Laos	2048	155.3 (126.5 to 190.0)	70.0 (50.5 to 93.1)	68.7 (48.4 to 97.1)	6.1 (3.0 to 11.1)	10.5 (7.3 to 16.9)	472.0 (384.4 to 577.4)	212.8 (153.5 to 282.8)	208.6 (147.0 to 295.0)	18.5 (9.0 to 33.8)	32.0 (22.3 to 51.2)
Laos	2049	160.4 (128.9 to 198.7)	72.9 (52.7 to 97.5)	70.4 (49.6 to 99.6)	6.3 (3.1 to 11.5)	10.7 (7.3 to 17.1)	487.5 (391.8 to 603.8)	221.7 (160.1 to 296.3)	214.0 (150.8 to 302.6)	19.1 (9.3 to 34.9)	32.7 (22.2 to 52.0)
Laos	2050	165.6 (133.6 to 202.6)	76.0 (54.7 to 101.7)	72.2 (50.9 to 102.0)	6.5 (3.2 to 11.8)	11.0 (7.5 to 17.9)	503.4 (406.1 to 615.6)	230.9 (166.2 to 309.1)	219.3 (154.7 to 310.1)	19.8 (9.6 to 35.9)	33.4 (22.8 to 54.4)
Latvia	1995	329.1 (298.0 to 362.3)	188.0 (167.4 to 211.0)	137.5 (116.4 to 161.3)	3.3 (1.6 to 5.9)	0.3 (0.3 to 0.3)	540.7 (489.5 to 595.2)	308.8 (275.1 to 346.6)	225.9 (191.2 to 264.9)	5.4 (2.7 to 9.7)	0.5 (0.5 to 0.5)
Latvia	1996	343.8 (315.8 to 373.4)	189.2 (171.3 to 209.0)	151.2 (131.9 to 173.0)	3.3 (1.6 to 6.0)	0.0 (0.0 to 0.0)	564.7 (518.8 to 613.4)	310.8 (281.3 to 343.4)	248.5 (216.7 to 284.2)	5.4 (2.7 to 9.8)	0.0 (0.0 to 0.0)
Latvia	1997	376.0 (347.9 to 403.5)	203.0 (185.8 to 222.1)	167.6 (149.3 to 189.6)	4.0 (2.0 to 7.1)	1.4 (1.4 to 1.4)	617.7 (571.5 to 662.8)	333.5 (305.2 to 364.9)	275.2 (245.2 to 311.4)	6.6 (3.3 to 11.7)	2.3 (2.3 to 2.3)
Latvia	1998	404.6 (376.9 to 432.0)	217.5 (200.0 to 236.8)	179.1 (160.8 to 199.9)	5.0 (2.5 to 8.7)	3.0 (3.0 to 3.0)	664.6 (619.2 to 709.7)	357.3 (328.6 to 389.0)	294.2 (264.2 to 328.3)	8.2 (4.1 to 14.4)	4.9 (4.9 to 4.9)
Latvia	1999	419.1 (391.6 to 447.1)	222.2 (204.2 to 241.4)	190.3 (171.0 to 209.6)	6.0 (3.1 to 10.5)	0.6 (0.6 to 0.6)	688.4 (643.3 to 734.5)	365.0 (335.4 to 396.6)	312.6 (280.9 to 344.3)	9.8 (5.0 to 17.3)	1.0 (1.0 to 1.0)
Latvia	2000	432.8 (405.7 to 461.4)	222.2 (203.6 to 240.9)	202.3 (182.3 to 222.3)	7.3 (3.7 to 13.0)	0.9 (0.9 to 0.9)	710.9 (666.4 to 757.9)	365.0 (334.4 to 395.7)	332.4 (299.5 to 365.2)	12.0 (6.1 to 21.4)	1.5 (1.5 to 1.5)
Latvia	2001	469.1 (441.2 to 498.4)	235.7 (217.5 to 254.7)	222.8 (201.5 to 244.1)	9.5 (5.0 to 17.0)	1.1 (1.1 to 1.1)	770.6 (724.7 to 818.7)	387.2 (357.3 to 418.4)	365.9 (330.9 to 401.0)	15.6 (8.2 to 27.9)	1.8 (1.8 to 1.8)

		2018 US Dollars per capita					2018 PPP per capita				
Country	Year	Health spending	Government health spending	Out-of-pocket health spending	Prepaid private health spending	Development assistance for health	Health spending	Government health spending	Out-of-pocket health spending	Prepaid private health spending	Development assistance for health
Latvia	2002	514.8 (483.9 to 544.9)	259.9 (240.7 to 281.1)	241.3 (219.8 to 264.0)	11.5 (6.1 to 20.7)	2.0 (2.0 to 2.0)	845.6 (794.9 to 895.1)	426.9 (395.5 to 461.7)	396.4 (361.1 to 433.7)	19.0 (10.0 to 34.0)	3.3 (3.3 to 3.3)
Latvia	2003	560.1 (529.4 to 591.9)	291.5 (272.4 to 312.8)	256.1 (234.4 to 278.3)	12.1 (6.6 to 21.3)	0.4 (0.4 to 0.4)	920.1 (869.7 to 972.3)	478.8 (447.5 to 513.8)	420.8 (385.1 to 457.2)	19.9 (10.8 to 34.9)	0.6 (0.6 to 0.6)
Latvia	2004	635.8 (602.3 to 672.0)	345.5 (324.0 to 367.9)	273.7 (249.7 to 296.8)	15.7 (8.5 to 26.5)	0.9 (0.9 to 0.9)	1044.4 (989.4 to 1103.8)	567.6 (532.3 to 604.4)	449.7 (410.2 to 487.5)	25.8 (14.0 to 43.5)	1.4 (1.4 to 1.4)
Latvia	2005	703.1 (668.5 to 740.6)	396.9 (373.9 to 422.5)	288.5 (264.5 to 313.6)	17.6 (9.7 to 29.3)	0.0 (0.0 to 0.0)	1155.0 (1098.2 to 1216.6)	652.1 (614.2 to 694.1)	474.0 (434.5 to 515.1)	28.9 (16.0 to 48.1)	0.0 (0.0 to 0.0)
Latvia	2006	786.0 (749.1 to 825.2)	462.3 (435.3 to 489.9)	303.4 (279.9 to 329.7)	20.3 (11.2 to 33.5)	0.0 (0.0 to 0.0)	1291.1 (1230.6 to 1355.6)	759.4 (715.1 to 804.8)	498.4 (459.8 to 541.6)	33.4 (18.5 to 55.0)	0.0 (0.0 to 0.0)
Latvia	2007	866.2 (824.6 to 909.7)	518.5 (489.6 to 548.1)	327.1 (302.8 to 354.3)	20.6 (11.3 to 33.7)	0.0 (0.0 to 0.0)	1422.9 (1354.5 to 1494.3)	851.8 (804.3 to 900.4)	537.3 (497.4 to 581.9)	33.9 (18.5 to 55.4)	0.0 (0.0 to 0.0)
Latvia	2008	859.5 (820.6 to 899.0)	519.7 (492.2 to 548.4)	320.2 (296.5 to 345.4)	18.7 (10.4 to 31.0)	0.8 (0.8 to 0.8)	1411.9 (1348.0 to 1476.8)	853.8 (808.5 to 900.8)	526.1 (487.1 to 567.5)	30.8 (17.2 to 51.0)	1.3 (1.3 to 1.3)
Latvia	2009	777.9 (744.0 to 811.0)	467.7 (443.7 to 493.7)	295.3 (273.3 to 319.4)	14.9 (8.3 to 24.5)	0.0 (0.0 to 0.0)	1277.9 (1222.2 to 1332.3)	768.3 (728.9 to 811.0)	485.1 (449.0 to 524.7)	24.4 (13.7 to 40.2)	0.0 (0.0 to 0.0)
Latvia	2010	777.9 (743.7 to 811.7)	456.6 (432.6 to 480.4)	283.3 (262.6 to 306.3)	15.9 (8.8 to 26.8)	22.1 (22.1 to 22.1)	1277.9 (1221.7 to 1333.5)	750.0 (710.7 to 789.1)	465.4 (431.4 to 503.1)	26.2 (14.5 to 44.0)	36.3 (36.3 to 36.3)
Latvia	2011	794.7 (759.7 to 828.9)	473.4 (448.8 to 497.6)	281.9 (261.2 to 304.9)	15.6 (8.8 to 26.3)	23.7 (23.7 to 23.7)	1305.4 (1248.0 to 1361.6)	777.6 (737.3 to 817.4)	463.1 (429.0 to 500.8)	25.7 (14.4 to 43.2)	39.0 (39.0 to 39.0)
Latvia	2012	785.8 (751.3 to 818.3)	477.8 (453.5 to 501.7)	293.9 (272.7 to 316.5)	14.1 (8.0 to 23.5)	0.0 (0.0 to 0.0)	1290.9 (1234.2 to 1344.2)	785.0 (745.0 to 824.1)	482.8 (448.0 to 519.9)	23.2 (13.1 to 38.6)	0.0 (0.0 to 0.0)
Latvia	2013	813.2 (781.3 to 847.0)	487.7 (464.3 to 510.3)	313.1 (292.1 to 336.2)	12.4 (6.9 to 21.0)	0.0 (0.0 to 0.0)	1335.9 (1283.4 to 1391.3)	801.2 (762.7 to 838.3)	514.4 (479.8 to 552.3)	20.3 (11.3 to 34.5)	0.0 (0.0 to 0.0)
Latvia	2014	857.3 (827.0 to 891.9)	504.0 (482.4 to 528.2)	342.4 (321.0 to 366.4)	10.9 (6.0 to 18.7)	0.0 (0.0 to 0.0)	1408.3 (1358.5 to 1465.1)	827.9 (792.5 to 867.6)	562.5 (527.4 to 601.8)	17.9 (9.8 to 30.7)	0.0 (0.0 to 0.0)
Latvia	2015	921.1 (886.8 to 959.1)	524.8 (500.2 to 551.0)	386.5 (361.0 to 412.3)	9.9 (5.3 to 17.4)	0.0 (0.0 to 0.0)	1513.1 (1456.8 to 1575.5)	862.0 (821.7 to 905.2)	634.9 (593.1 to 677.3)	16.2 (8.7 to 28.6)	0.0 (0.0 to 0.0)
Latvia	2016	995.0 (942.8 to 1045.1)	548.6 (512.7 to 581.4)	436.7 (401.0 to 474.0)	9.7 (5.1 to 17.2)	0.0 (0.0 to 0.0)	1634.5 (1548.8 to 1716.8)	901.3 (842.3 to 955.1)	717.3 (658.7 to 778.6)	16.0 (8.4 to 28.2)	0.0 (0.0 to 0.0)
Latvia	2017	1048.8 (992.8 to 1102.0)	582.0 (543.5 to 618.2)	456.4 (418.1 to 495.4)	10.3 (5.4 to 18.2)	0.0 (0.0 to 0.0)	1722.8 (1630.9 to 1810.2)	956.1 (892.9 to 1015.6)	749.8 (686.9 to 813.9)	16.9 (8.9 to 29.9)	0.0 (0.0 to 0.0)
Latvia	2018	1073.0 (1017.8 to 1127.8)	593.4 (553.9 to 631.7)	469.1 (429.2 to 508.6)	10.5 (5.5 to 18.6)	0.0 (0.0 to 0.0)	1762.6 (1671.9 to 1852.6)	974.7 (909.8 to 1037.7)	770.5 (705.0 to 835.5)	17.3 (9.1 to 30.5)	0.0 (0.0 to 0.0)
Latvia	2019	1087.6 (1029.2 to 1142.4)	602.9 (562.8 to 641.8)	473.9 (432.3 to 513.9)	10.7 (5.6 to 18.9)	0.0 (0.0 to 0.0)	1786.7 (1690.7 to 1876.6)	990.5 (924.5 to 1054.2)	778.6 (710.2 to 844.2)	17.6 (9.3 to 31.0)	0.0 (0.0 to 0.0)
Latvia	2020	1102.7 (1041.4 to 1156.7)	612.9 (571.7 to 651.4)	478.9 (438.0 to 519.7)	11.0 (5.7 to 19.3)	0.0 (0.0 to 0.0)	1811.4 (1710.7 to 1900.0)	1006.7 (939.2 to 1070.0)	786.7 (719.5 to 853.7)	18.0 (9.4 to 31.7)	0.0 (0.0 to 0.0)
Latvia	2021	1118.3 (1055.6 to 1175.2)	623.2 (581.3 to 664.5)	483.9 (441.7 to 525.5)	11.2 (5.8 to 19.7)	0.0 (0.0 to 0.0)	1837.0 (1734.0 to 1930.6)	1023.8 (955.0 to 1091.5)	794.8 (725.7 to 863.2)	18.3 (9.6 to 32.4)	0.0 (0.0 to 0.0)
Latvia	2022	1135.0 (1073.1 to 1192.8)	634.3 (592.9 to 677.6)	489.2 (445.8 to 531.7)	11.4 (5.9 to 20.1)	0.0 (0.0 to 0.0)	1864.4 (1762.7 to 1959.4)	1042.0 (974.0 to 1113.1)	803.7 (732.4 to 873.5)	18.7 (9.8 to 33.0)	0.0 (0.0 to 0.0)

		2018 US Dollars per capita					2018 PPP per capita				
Country	Year	Health spending	Government health spending	Out-of-pocket health spending	Prepaid private health spending	Development assistance for health	Health spending	Government health spending	Out-of-pocket health spending	Prepaid private health spending	Development assistance for health
Latvia	2023	1151.3 (1089.7 to 1208.6)	644.6 (602.5 to 690.4)	495.0 (451.6 to 538.5)	11.6 (6.1 to 20.5)	0.0 (0.0 to 0.0)	1891.2 (1790.0 to 1985.3)	1059.0 (989.8 to 1134.2)	813.2 (741.8 to 884.6)	19.1 (10.0 to 33.7)	0.0 (0.0 to 0.0)
Latvia	2024	1178.8 (1114.9 to 1241.7)	657.6 (614.0 to 703.7)	509.3 (463.3 to 556.5)	11.9 (6.2 to 21.0)	0.0 (0.0 to 0.0)	1936.4 (1831.4 to 2039.7)	1080.3 (1008.6 to 1155.9)	836.7 (761.0 to 914.1)	19.5 (10.2 to 34.4)	0.0 (0.0 to 0.0)
Latvia	2025	1208.1 (1139.1 to 1274.2)	670.7 (625.4 to 716.3)	525.2 (477.4 to 574.4)	12.1 (6.3 to 21.4)	0.0 (0.0 to 0.0)	1984.5 (1871.2 to 2093.2)	1101.8 (1027.4 to 1176.7)	862.8 (784.2 to 943.5)	19.9 (10.4 to 35.2)	0.0 (0.0 to 0.0)
Latvia	2026	1239.2 (1167.0 to 1306.9)	685.0 (638.7 to 731.9)	541.7 (491.9 to 595.2)	12.4 (6.5 to 21.9)	0.0 (0.0 to 0.0)	2035.6 (1917.1 to 2146.8)	1125.3 (1049.2 to 1202.4)	889.9 (808.0 to 977.7)	20.4 (10.7 to 36.0)	0.0 (0.0 to 0.0)
Latvia	2027	1273.5 (1194.8 to 1348.3)	701.7 (654.4 to 750.6)	559.1 (503.0 to 615.2)	12.7 (6.7 to 22.5)	0.0 (0.0 to 0.0)	2091.9 (1962.7 to 2214.8)	1152.6 (1074.9 to 1233.0)	918.5 (826.3 to 1010.6)	20.8 (11.0 to 36.9)	0.0 (0.0 to 0.0)
Latvia	2028	1309.1 (1223.2 to 1389.2)	719.1 (669.6 to 770.5)	577.0 (514.9 to 640.9)	13.0 (6.9 to 23.0)	0.0 (0.0 to 0.0)	2150.5 (2009.3 to 2282.1)	1181.3 (1099.9 to 1265.7)	947.8 (845.8 to 1052.8)	21.4 (11.3 to 37.9)	0.0 (0.0 to 0.0)
Latvia	2029	1345.6 (1256.8 to 1436.1)	737.1 (683.7 to 789.9)	595.2 (527.2 to 665.7)	13.3 (7.1 to 23.6)	0.0 (0.0 to 0.0)	2210.4 (2064.6 to 2359.0)	1210.8 (1123.1 to 1297.5)	977.7 (866.0 to 1093.6)	21.9 (11.6 to 38.8)	0.0 (0.0 to 0.0)
Latvia	2030	1382.9 (1287.7 to 1481.0)	755.6 (700.1 to 810.4)	613.6 (537.5 to 692.9)	13.7 (7.3 to 24.3)	0.0 (0.0 to 0.0)	2271.6 (2115.3 to 2432.9)	1241.2 (1150.1 to 1331.3)	1008.0 (882.9 to 1138.2)	22.5 (11.9 to 39.9)	0.0 (0.0 to 0.0)
Latvia	2031	1418.0 (1317.3 to 1522.9)	772.0 (713.7 to 828.6)	631.9 (548.1 to 718.5)	14.0 (7.4 to 24.9)	0.0 (0.0 to 0.0)	2329.3 (2163.9 to 2501.7)	1268.1 (1172.4 to 1361.2)	1038.1 (900.3 to 1180.3)	23.1 (12.2 to 40.9)	0.0 (0.0 to 0.0)
Latvia	2032	1452.9 (1339.9 to 1561.6)	788.2 (728.8 to 846.5)	650.3 (562.4 to 744.0)	14.4 (7.6 to 25.5)	0.0 (0.0 to 0.0)	2386.7 (2201.1 to 2565.3)	1294.8 (1197.2 to 1390.5)	1068.2 (923.9 to 1222.1)	23.6 (12.5 to 41.9)	0.0 (0.0 to 0.0)
Latvia	2033	1488.4 (1369.5 to 1602.1)	804.6 (742.8 to 865.1)	669.0 (573.3 to 771.8)	14.8 (7.8 to 26.2)	0.0 (0.0 to 0.0)	2445.0 (2249.7 to 2631.7)	1321.8 (1220.2 to 1421.1)	1099.0 (941.7 to 1267.8)	24.2 (12.9 to 43.0)	0.0 (0.0 to 0.0)
Latvia	2034	1523.1 (1401.6 to 1647.5)	820.6 (756.5 to 882.9)	687.3 (582.8 to 801.0)	15.1 (8.0 to 26.8)	0.0 (0.0 to 0.0)	2501.9 (2302.4 to 2706.3)	1348.0 (1242.6 to 1450.3)	1129.1 (957.4 to 1315.8)	24.8 (13.2 to 44.0)	0.0 (0.0 to 0.0)
Latvia	2035	1558.8 (1427.2 to 1696.6)	837.5 (771.0 to 902.2)	705.8 (595.1 to 828.8)	15.5 (8.2 to 27.4)	0.0 (0.0 to 0.0)	2560.7 (2344.5 to 2787.0)	1375.8 (1266.5 to 1482.1)	1159.4 (977.6 to 1361.5)	25.5 (13.5 to 45.1)	0.0 (0.0 to 0.0)
Latvia	2036	1594.5 (1459.6 to 1738.7)	854.6 (784.7 to 921.1)	724.0 (606.0 to 855.8)	15.9 (8.4 to 28.0)	0.0 (0.0 to 0.0)	2619.3 (2397.7 to 2856.2)	1403.9 (1289.0 to 1513.1)	1189.3 (995.5 to 1405.9)	26.1 (13.8 to 46.1)	0.0 (0.0 to 0.0)
Latvia	2037	1630.5 (1484.4 to 1784.7)	872.2 (799.6 to 943.7)	742.0 (615.3 to 883.6)	16.3 (8.6 to 28.7)	0.0 (0.0 to 0.0)	2678.4 (2438.4 to 2931.8)	1432.7 (1313.6 to 1550.3)	1219.0 (1010.7 to 1451.5)	26.7 (14.1 to 47.1)	0.0 (0.0 to 0.0)
Latvia	2038	1666.3 (1516.9 to 1838.8)	889.7 (814.1 to 965.0)	760.0 (626.0 to 912.4)	16.6 (8.8 to 29.3)	0.0 (0.0 to 0.0)	2737.3 (2491.8 to 3020.6)	1461.5 (1337.3 to 1585.1)	1248.4 (1028.3 to 1498.8)	27.3 (14.4 to 48.1)	0.0 (0.0 to 0.0)
Latvia	2039	1701.0 (1540.2 to 1876.3)	906.4 (827.7 to 984.3)	777.5 (636.2 to 937.5)	17.0 (9.0 to 29.9)	0.0 (0.0 to 0.0)	2794.2 (2530.1 to 3082.3)	1489.0 (1359.6 to 1616.9)	1277.2 (1045.1 to 1540.1)	28.0 (14.7 to 49.2)	0.0 (0.0 to 0.0)
Latvia	2040	1738.3 (1572.1 to 1933.7)	925.5 (843.0 to 1007.7)	795.4 (647.9 to 962.5)	17.5 (9.2 to 30.7)	0.0 (0.0 to 0.0)	2855.5 (2582.6 to 3176.4)	1520.3 (1384.8 to 1655.3)	1306.5 (1064.3 to 1581.2)	28.7 (15.1 to 50.4)	0.0 (0.0 to 0.0)
Latvia	2041	1773.7 (1600.2 to 1972.7)	943.2 (855.2 to 1030.0)	812.7 (658.0 to 986.1)	17.9 (9.4 to 31.4)	0.0 (0.0 to 0.0)	2913.7 (2628.7 to 3240.6)	1549.4 (1404.8 to 1692.0)	1335.0 (1080.9 to 1619.9)	29.4 (15.4 to 51.6)	0.0 (0.0 to 0.0)
Latvia	2042	1811.3 (1635.7 to 2022.3)	962.7 (871.2 to 1054.0)	830.3 (671.6 to 1009.7)	18.3 (9.6 to 32.2)	0.0 (0.0 to 0.0)	2975.4 (2686.9 to 3322.0)	1581.4 (1431.0 to 1731.5)	1364.0 (1103.2 to 1658.6)	30.1 (15.8 to 52.9)	0.0 (0.0 to 0.0)
Latvia	2043	1848.8 (1668.2 to 2068.2)	982.3 (887.3 to 1079.3)	847.7 (685.1 to 1032.2)	18.8 (9.8 to 33.0)	0.0 (0.0 to 0.0)	3037.0 (2740.4 to 3397.5)	1613.7 (1457.6 to 1772.9)	1392.5 (1125.4 to 1695.7)	30.9 (16.1 to 54.2)	0.0 (0.0 to 0.0)

		2018 US Dollars per capita					2018 PPP per capita				
Country	Year	Health spending	Government health spending	Out-of-pocket health spending	Prepaid private health spending	Development assistance for health	Health spending	Government health spending	Out-of-pocket health spending	Prepaid private health spending	Development assistance for health
Latvia	2044	1885.5 (1696.9 to 2110.4)	1001.4 (902.3 to 1104.8)	864.8 (697.4 to 1053.7)	19.3 (10.0 to 33.9)	0.0 (0.0 to 0.0)	3097.2 (2787.6 to 3466.8)	1645.1 (1482.3 to 1814.8)	1420.5 (1145.6 to 1730.9)	31.6 (16.5 to 55.6)	0.0 (0.0 to 0.0)
Latvia	2045	1920.6 (1709.9 to 2162.9)	1019.8 (916.2 to 1127.3)	881.0 (709.3 to 1075.6)	19.7 (10.3 to 34.7)	0.0 (0.0 to 0.0)	3154.9 (2808.8 to 3552.9)	1675.3 (1505.1 to 1851.9)	1447.3 (1165.1 to 1766.9)	32.4 (16.9 to 57.1)	0.0 (0.0 to 0.0)
Latvia	2046	1954.7 (1740.2 to 2203.4)	1038.0 (929.1 to 1149.8)	896.5 (721.9 to 1099.3)	20.2 (10.5 to 35.6)	0.0 (0.0 to 0.0)	3211.0 (2858.6 to 3619.5)	1705.2 (1526.2 to 1888.7)	1472.7 (1185.8 to 1805.8)	33.1 (17.3 to 58.5)	0.0 (0.0 to 0.0)
Latvia	2047	1986.4 (1774.2 to 2239.9)	1054.5 (941.7 to 1170.9)	911.2 (732.1 to 1119.1)	20.6 (10.7 to 36.5)	0.0 (0.0 to 0.0)	3263.0 (2914.5 to 3679.5)	1732.3 (1547.0 to 1923.4)	1496.9 (1202.7 to 1838.3)	33.8 (17.6 to 59.9)	0.0 (0.0 to 0.0)
Latvia	2048	2018.6 (1800.1 to 2283.1)	1071.6 (955.3 to 1192.2)	925.9 (743.0 to 1136.5)	21.0 (10.9 to 37.3)	0.0 (0.0 to 0.0)	3316.0 (2957.0 to 3750.4)	1760.4 (1569.3 to 1958.5)	1521.0 (1220.5 to 1866.9)	34.6 (18.0 to 61.3)	0.0 (0.0 to 0.0)
Latvia	2049	2048.3 (1826.4 to 2303.7)	1086.7 (963.1 to 1210.7)	940.1 (754.6 to 1154.1)	21.5 (11.2 to 38.1)	0.0 (0.0 to 0.0)	3364.7 (3000.2 to 3784.3)	1785.1 (1582.1 to 1988.7)	1544.4 (1239.6 to 1895.8)	35.2 (18.4 to 62.7)	0.0 (0.0 to 0.0)
Latvia	2050	2077.1 (1859.1 to 2351.4)	1101.0 (972.0 to 1230.0)	954.2 (766.3 to 1175.1)	21.8 (11.4 to 38.9)	0.0 (0.0 to 0.0)	3412.1 (3053.9 to 3862.7)	1808.6 (1596.8 to 2020.5)	1567.5 (1258.8 to 1930.3)	35.9 (18.8 to 64.0)	0.0 (0.0 to 0.0)
Lebanon	1995	629.6 (574.8 to 689.5)	237.4 (208.0 to 272.6)	311.4 (274.7 to 353.4)	73.2 (51.0 to 103.6)	7.6 (7.6 to 7.6)	1103.7 (1007.6 to 1208.6)	416.1 (364.6 to 477.9)	545.9 (481.6 to 619.4)	128.3 (89.5 to 181.7)	13.3 (13.3 to 13.3)
Lebanon	1996	649.6 (603.6 to 701.3)	248.7 (220.3 to 279.8)	322.3 (292.7 to 354.1)	77.8 (55.8 to 104.9)	0.8 (0.8 to 0.8)	1138.7 (1058.1 to 1229.3)	435.9 (386.2 to 490.5)	564.9 (513.1 to 620.8)	136.4 (97.9 to 183.9)	1.5 (1.5 to 1.5)
Lebanon	1997	635.8 (593.8 to 681.8)	234.2 (207.4 to 262.2)	323.1 (297.5 to 351.1)	76.7 (56.8 to 100.8)	1.7 (1.7 to 1.7)	1114.5 (1040.9 to 1195.2)	410.5 (363.5 to 459.7)	566.5 (521.6 to 615.4)	134.5 (99.6 to 176.7)	3.0 (3.0 to 3.0)
Lebanon	1998	592.2 (552.9 to 632.9)	197.7 (175.0 to 221.5)	321.0 (295.2 to 349.3)	71.2 (53.6 to 93.0)	2.4 (2.4 to 2.4)	1038.1 (969.2 to 1109.4)	346.5 (306.8 to 388.3)	562.7 (517.6 to 612.3)	124.8 (94.0 to 163.0)	4.2 (4.2 to 4.2)
Lebanon	1999	553.5 (517.3 to 590.8)	174.4 (153.8 to 196.1)	308.5 (283.9 to 336.3)	67.0 (49.8 to 88.5)	3.6 (3.6 to 3.6)	970.3 (906.8 to 1035.7)	305.7 (269.6 to 343.7)	540.8 (497.8 to 589.5)	117.5 (87.3 to 155.1)	6.2 (6.2 to 6.2)
Lebanon	2000	528.4 (492.0 to 566.4)	166.0 (146.3 to 187.4)	294.7 (270.2 to 322.8)	65.0 (48.8 to 87.0)	2.7 (2.7 to 2.7)	926.3 (862.5 to 992.9)	291.0 (256.4 to 328.5)	516.7 (473.7 to 565.8)	113.9 (85.5 to 152.4)	4.8 (4.8 to 4.8)
Lebanon	2001	503.3 (466.7 to 540.4)	162.3 (144.3 to 183.5)	275.3 (251.5 to 301.5)	62.8 (47.2 to 84.5)	2.9 (2.9 to 2.9)	882.3 (818.1 to 947.3)	284.5 (253.0 to 321.7)	482.6 (440.9 to 528.6)	110.2 (82.7 to 148.1)	5.0 (5.0 to 5.0)
Lebanon	2002	466.1 (430.6 to 502.0)	155.1 (136.8 to 176.5)	248.7 (224.9 to 273.7)	58.9 (43.5 to 79.1)	3.5 (3.5 to 3.5)	817.1 (754.9 to 880.0)	271.8 (239.9 to 309.4)	435.9 (394.3 to 479.8)	103.3 (76.3 to 138.6)	6.1 (6.1 to 6.1)
Lebanon	2003	435.9 (403.6 to 471.1)	150.4 (132.3 to 170.2)	224.6 (201.4 to 249.1)	56.0 (41.3 to 74.2)	4.9 (4.9 to 4.9)	764.1 (707.4 to 825.9)	263.6 (231.9 to 298.4)	393.7 (353.1 to 436.6)	98.1 (72.5 to 130.0)	8.7 (8.7 to 8.7)
Lebanon	2004	427.8 (395.9 to 463.7)	157.0 (138.2 to 177.9)	211.3 (190.1 to 234.6)	56.7 (41.9 to 74.6)	2.8 (2.8 to 2.8)	750.0 (693.9 to 812.9)	275.2 (242.3 to 311.8)	370.5 (333.2 to 411.3)	99.4 (73.4 to 130.7)	4.9 (4.9 to 4.9)
Lebanon	2005	425.3 (393.7 to 461.8)	163.4 (143.6 to 184.3)	202.7 (181.6 to 225.1)	58.2 (43.0 to 77.6)	1.0 (1.0 to 1.0)	745.6 (690.1 to 809.5)	286.5 (251.7 to 323.1)	355.4 (318.4 to 394.6)	102.0 (75.3 to 136.1)	1.7 (1.7 to 1.7)
Lebanon	2006	433.1 (399.7 to 469.8)	169.5 (148.9 to 192.0)	201.0 (180.8 to 223.0)	60.7 (44.7 to 81.2)	2.0 (2.0 to 2.0)	759.3 (700.6 to 823.5)	297.2 (261.0 to 336.6)	352.3 (316.9 to 390.8)	106.3 (78.3 to 142.4)	3.5 (3.5 to 3.5)
Lebanon	2007	456.9 (422.0 to 494.3)	181.9 (160.6 to 205.9)	206.1 (186.5 to 228.1)	65.3 (48.3 to 85.9)	3.7 (3.7 to 3.7)	801.0 (739.7 to 866.5)	318.8 (281.6 to 360.9)	361.3 (326.9 to 399.9)	114.4 (84.7 to 150.5)	6.5 (6.5 to 6.5)
Lebanon	2008	471.7 (435.0 to 509.6)	188.2 (166.5 to 212.6)	207.2 (187.6 to 230.0)	68.0 (51.2 to 88.1)	8.3 (8.3 to 8.3)	827.0 (762.5 to 893.3)	330.0 (291.9 to 372.7)	363.3 (328.8 to 403.1)	119.2 (89.7 to 154.5)	14.5 (14.5 to 14.5)

		2018 US Dollars per capita					2018 PPP per capita				
Country	Year	Health spending	Government health spending	Out-of-pocket health spending	Prepaid private health spending	Development assistance for health	Health spending	Government health spending	Out-of-pocket health spending	Prepaid private health spending	Development assistance for health
Lebanon	2009	489.4 (453.0 to 528.5)	203.4 (181.3 to 227.4)	210.9 (190.3 to 232.9)	73.1 (55.0 to 94.5)	2.0 (2.0 to 2.0)	857.9 (794.1 to 926.5)	356.6 (317.9 to 398.7)	369.8 (333.6 to 408.2)	128.1 (96.4 to 165.7)	3.5 (3.5 to 3.5)
Lebanon	2010	520.7 (483.0 to 562.1)	222.5 (198.4 to 247.6)	215.0 (193.3 to 238.4)	80.3 (61.4 to 103.7)	2.8 (2.8 to 2.8)	912.8 (846.6 to 985.4)	390.1 (347.8 to 434.1)	376.9 (338.9 to 418.0)	140.8 (107.6 to 181.7)	5.0 (5.0 to 5.0)
Lebanon	2011	521.7 (483.5 to 563.3)	232.8 (208.7 to 258.2)	204.1 (183.1 to 226.3)	82.9 (63.3 to 107.7)	2.0 (2.0 to 2.0)	914.5 (847.5 to 987.5)	408.1 (365.8 to 452.6)	357.7 (321.0 to 396.8)	145.3 (111.0 to 188.7)	3.4 (3.4 to 3.4)
Lebanon	2012	510.9 (473.4 to 552.7)	241.5 (216.5 to 266.9)	183.9 (165.1 to 203.8)	83.3 (63.5 to 107.4)	2.2 (2.2 to 2.2)	895.6 (829.8 to 968.9)	423.4 (379.4 to 467.9)	322.3 (289.4 to 357.2)	146.0 (111.3 to 188.2)	3.9 (3.9 to 3.9)
Lebanon	2013	492.3 (454.9 to 534.2)	240.8 (215.3 to 267.3)	169.9 (151.1 to 189.8)	79.9 (60.3 to 102.6)	1.6 (1.6 to 1.6)	863.0 (797.4 to 936.5)	422.2 (377.5 to 468.5)	297.9 (264.8 to 332.7)	140.1 (105.8 to 179.9)	2.8 (2.8 to 2.8)
Lebanon	2014	488.0 (447.9 to 531.4)	241.4 (216.0 to 269.0)	164.2 (143.8 to 185.5)	77.0 (57.7 to 100.8)	5.4 (5.4 to 5.4)	855.4 (785.1 to 931.5)	423.1 (378.7 to 471.6)	287.9 (252.1 to 325.1)	135.0 (101.2 to 176.7)	9.5 (9.5 to 9.5)
Lebanon	2015	481.4 (438.5 to 528.2)	242.8 (215.6 to 274.5)	160.1 (140.1 to 183.6)	75.7 (54.7 to 101.1)	2.9 (2.9 to 2.9)	843.9 (768.7 to 925.9)	425.6 (377.9 to 481.1)	280.6 (245.5 to 321.9)	132.6 (96.0 to 177.3)	5.0 (5.0 to 5.0)
Lebanon	2016	486.3 (436.9 to 539.9)	249.9 (218.1 to 286.1)	158.0 (135.7 to 184.2)	75.8 (52.3 to 105.1)	2.6 (2.6 to 2.6)	852.5 (766.0 to 946.5)	438.0 (382.3 to 501.5)	276.9 (237.9 to 322.9)	132.9 (91.7 to 184.2)	4.6 (4.6 to 4.6)
Lebanon	2017	490.0 (443.3 to 542.8)	252.9 (220.8 to 289.6)	157.2 (134.4 to 183.6)	76.8 (53.1 to 106.4)	3.1 (3.1 to 3.1)	858.9 (777.1 to 951.5)	443.3 (387.1 to 507.7)	275.6 (235.7 to 321.9)	134.6 (93.1 to 186.5)	5.4 (5.4 to 5.4)
Lebanon	2018	497.8 (450.3 to 551.8)	259.4 (226.8 to 296.5)	157.1 (134.0 to 183.2)	78.4 (54.3 to 108.6)	2.9 (2.9 to 2.9)	872.6 (789.4 to 967.3)	454.8 (397.6 to 519.8)	275.3 (234.9 to 321.1)	137.4 (95.1 to 190.3)	5.0 (5.0 to 5.1)
Lebanon	2019	505.5 (456.0 to 559.9)	265.7 (232.0 to 303.5)	156.8 (133.9 to 183.4)	80.0 (55.3 to 111.2)	2.9 (2.7 to 3.1)	886.1 (799.4 to 981.4)	465.8 (406.7 to 532.0)	274.9 (234.7 to 321.6)	140.2 (96.9 to 194.9)	5.1 (4.8 to 5.4)
Lebanon	2020	513.6 (463.1 to 567.8)	272.5 (238.2 to 310.9)	156.6 (133.9 to 183.6)	81.6 (56.2 to 113.4)	3.0 (2.7 to 3.2)	900.4 (811.9 to 995.4)	477.7 (417.6 to 545.0)	274.4 (234.7 to 321.9)	143.0 (98.6 to 198.7)	5.2 (4.8 to 5.6)
Lebanon	2021	522.8 (472.0 to 577.4)	279.7 (244.7 to 319.2)	156.6 (133.8 to 182.9)	83.4 (57.6 to 116.0)	3.1 (2.8 to 3.4)	916.4 (827.4 to 1012.2)	490.4 (428.9 to 559.6)	274.5 (234.5 to 320.6)	146.2 (100.9 to 203.3)	5.4 (4.9 to 6.0)
Lebanon	2022	531.2 (476.7 to 588.3)	286.3 (249.2 to 326.8)	156.6 (133.7 to 182.8)	85.1 (58.8 to 118.4)	3.2 (2.8 to 3.6)	931.1 (835.6 to 1031.2)	501.9 (436.8 to 573.0)	274.5 (234.4 to 320.4)	149.1 (103.0 to 207.6)	5.6 (5.0 to 6.3)
Lebanon	2023	536.5 (481.2 to 594.0)	290.6 (253.3 to 334.1)	156.2 (133.0 to 183.2)	86.3 (59.6 to 120.0)	3.3 (2.9 to 3.8)	940.4 (843.5 to 1041.2)	509.4 (444.1 to 585.6)	273.9 (233.2 to 321.1)	151.2 (104.4 to 210.4)	5.8 (5.1 to 6.6)
Lebanon	2024	541.0 (486.7 to 600.5)	294.3 (256.3 to 338.1)	155.9 (132.1 to 182.7)	87.4 (60.3 to 121.6)	3.5 (3.0 to 4.0)	948.4 (853.1 to 1052.6)	515.8 (449.4 to 592.8)	273.2 (231.5 to 320.2)	153.2 (105.7 to 213.2)	6.1 (5.3 to 7.0)
Lebanon	2025	545.6 (488.6 to 602.4)	297.9 (260.4 to 341.8)	155.5 (131.7 to 182.6)	88.6 (61.2 to 123.2)	3.6 (3.1 to 4.3)	956.4 (856.5 to 1056.0)	522.2 (456.5 to 599.2)	272.6 (230.8 to 320.1)	155.2 (107.3 to 216.0)	6.4 (5.5 to 7.5)
Lebanon	2026	549.0 (492.5 to 604.7)	300.8 (262.7 to 344.9)	154.9 (131.0 to 181.8)	89.5 (61.9 to 124.5)	3.8 (3.2 to 4.5)	962.4 (863.3 to 1060.1)	527.3 (460.5 to 604.6)	271.6 (229.6 to 318.7)	156.8 (108.5 to 218.2)	6.7 (5.6 to 7.8)
Lebanon	2027	552.4 (495.5 to 612.2)	303.6 (265.6 to 347.7)	154.4 (129.5 to 181.8)	90.4 (62.5 to 125.9)	4.0 (3.3 to 4.7)	968.3 (868.6 to 1073.2)	532.2 (465.7 to 609.4)	270.7 (227.1 to 318.7)	158.5 (109.6 to 220.7)	6.9 (5.8 to 8.3)
Lebanon	2028	555.4 (497.1 to 616.8)	306.0 (267.0 to 350.6)	154.0 (128.2 to 182.3)	91.3 (63.0 to 127.0)	4.1 (3.5 to 5.0)	973.6 (871.3 to 1081.2)	536.4 (468.1 to 614.6)	269.9 (224.7 to 319.7)	160.0 (110.4 to 222.6)	7.3 (6.1 to 8.7)
Lebanon	2029	558.4 (499.3 to 620.4)	308.3 (269.1 to 353.1)	153.7 (126.4 to 182.2)	92.2 (63.5 to 128.1)	4.3 (3.6 to 5.3)	978.9 (875.2 to 1087.5)	540.4 (471.8 to 618.9)	269.4 (221.6 to 319.4)	161.5 (111.3 to 224.6)	7.6 (6.2 to 9.2)

		2018 US Dollars per capita					2018 PPP per capita				
Country	Year	Health spending	Government health spending	Out-of-pocket health spending	Prepaid private health spending	Development assistance for health	Health spending	Government health spending	Out-of-pocket health spending	Prepaid private health spending	Development assistance for health
Lebanon	2030	561.3 (498.8 to 624.7)	310.4 (271.2 to 355.8)	153.5 (125.8 to 182.5)	93.0 (64.1 to 129.3)	4.5 (3.7 to 5.5)	984.0 (874.4 to 1095.1)	544.2 (475.4 to 623.6)	269.0 (220.5 to 319.9)	163.0 (112.3 to 226.6)	7.8 (6.4 to 9.6)
Lebanon	2031	564.4 (501.4 to 629.4)	312.6 (273.3 to 358.5)	153.3 (124.9 to 183.9)	93.9 (64.6 to 130.5)	4.6 (3.8 to 5.8)	989.5 (879.0 to 1103.4)	548.0 (479.1 to 628.5)	268.8 (218.9 to 322.4)	164.5 (113.2 to 228.8)	8.1 (6.6 to 10.2)
Lebanon	2032	568.1 (508.3 to 631.5)	315.1 (274.5 to 361.6)	153.4 (123.5 to 185.6)	94.8 (65.2 to 131.8)	4.8 (3.9 to 6.1)	995.9 (891.1 to 1107.0)	552.4 (481.3 to 633.9)	268.9 (216.5 to 325.4)	166.2 (114.3 to 231.0)	8.4 (6.8 to 10.6)
Lebanon	2033	571.5 (508.1 to 638.4)	317.2 (275.2 to 364.5)	153.5 (122.5 to 186.6)	95.8 (65.8 to 133.1)	5.0 (4.0 to 6.4)	1001.9 (890.6 to 1119.0)	556.1 (482.5 to 639.0)	269.1 (214.7 to 327.0)	167.9 (115.4 to 233.3)	8.8 (6.9 to 11.2)
Lebanon	2034	574.6 (511.9 to 643.1)	319.1 (275.8 to 366.7)	153.7 (121.6 to 188.4)	96.6 (66.4 to 134.2)	5.2 (4.1 to 6.7)	1007.2 (897.3 to 1127.4)	559.4 (483.5 to 642.8)	269.4 (213.1 to 330.3)	169.3 (116.3 to 235.2)	9.1 (7.1 to 11.7)
Lebanon	2035	577.7 (516.4 to 645.4)	320.9 (277.3 to 368.8)	154.0 (120.8 to 190.4)	97.4 (66.9 to 135.4)	5.4 (4.2 to 7.0)	1012.7 (905.3 to 1131.3)	562.5 (486.2 to 646.5)	270.0 (211.7 to 333.7)	170.8 (117.3 to 237.3)	9.4 (7.3 to 12.3)
Lebanon	2036	581.3 (514.5 to 646.9)	322.9 (279.1 to 370.9)	154.4 (120.3 to 191.8)	98.4 (67.6 to 136.7)	5.5 (4.3 to 7.4)	1019.0 (901.8 to 1134.1)	566.1 (489.2 to 650.2)	270.7 (210.9 to 336.2)	172.5 (118.5 to 239.6)	9.7 (7.5 to 13.0)
Lebanon	2037	585.1 (518.2 to 652.4)	325.0 (280.8 to 373.6)	155.0 (119.7 to 193.2)	99.4 (68.2 to 138.0)	5.7 (4.4 to 7.7)	1025.7 (908.3 to 1143.7)	569.8 (492.3 to 654.8)	271.7 (209.8 to 338.7)	174.2 (119.6 to 241.9)	10.0 (7.7 to 13.4)
Lebanon	2038	588.9 (521.5 to 662.7)	327.0 (282.5 to 376.2)	155.7 (119.3 to 195.0)	100.4 (68.8 to 139.4)	5.9 (4.5 to 8.2)	1032.4 (914.2 to 1161.8)	573.2 (495.3 to 659.5)	272.9 (209.2 to 341.8)	175.9 (120.5 to 244.3)	10.3 (7.9 to 14.3)
Lebanon	2039	592.5 (523.7 to 666.4)	328.7 (283.8 to 378.9)	156.4 (119.6 to 197.3)	101.3 (69.5 to 140.6)	6.1 (4.6 to 8.4)	1038.7 (918.1 to 1168.2)	576.3 (497.4 to 664.2)	274.2 (209.6 to 345.8)	177.5 (121.8 to 246.5)	10.7 (8.1 to 14.8)
Lebanon	2040	596.7 (527.1 to 670.5)	330.8 (285.8 to 381.5)	157.4 (120.3 to 199.9)	102.2 (70.1 to 141.9)	6.3 (4.8 to 8.7)	1046.0 (924.0 to 1175.3)	579.9 (501.0 to 668.7)	275.9 (210.8 to 350.4)	179.2 (123.0 to 248.8)	11.0 (8.4 to 15.2)
Lebanon	2041	601.6 (533.0 to 677.3)	333.2 (287.6 to 384.3)	158.6 (120.8 to 202.1)	103.3 (70.9 to 143.4)	6.5 (4.9 to 9.0)	1054.6 (934.4 to 1187.3)	584.1 (504.1 to 673.6)	278.0 (211.7 to 354.4)	181.2 (124.3 to 251.4)	11.4 (8.6 to 15.8)
Lebanon	2042	606.3 (536.0 to 684.6)	335.3 (288.9 to 386.7)	159.9 (121.4 to 204.6)	104.4 (71.6 to 144.5)	6.7 (5.0 to 9.6)	1062.8 (939.6 to 1200.0)	587.7 (506.4 to 677.8)	280.3 (212.7 to 358.6)	183.1 (125.5 to 253.3)	11.8 (8.8 to 16.8)
Lebanon	2043	610.9 (539.1 to 685.5)	337.2 (290.2 to 389.5)	161.3 (122.4 to 206.9)	105.5 (72.3 to 146.1)	6.9 (5.1 to 10.0)	1071.0 (945.0 to 1201.7)	591.1 (508.8 to 682.7)	282.8 (214.6 to 362.7)	184.9 (126.8 to 256.2)	12.1 (9.0 to 17.6)
Lebanon	2044	616.4 (542.4 to 693.8)	339.6 (291.7 to 392.6)	163.0 (123.8 to 208.7)	106.6 (73.1 to 147.9)	7.2 (5.3 to 10.4)	1080.5 (950.9 to 1216.2)	595.3 (511.4 to 688.2)	285.7 (217.0 to 365.9)	186.9 (128.2 to 259.3)	12.6 (9.3 to 18.2)
Lebanon	2045	621.5 (545.8 to 705.9)	341.7 (293.0 to 395.3)	164.8 (125.1 to 211.4)	107.7 (73.9 to 149.3)	7.4 (5.4 to 11.1)	1089.5 (956.7 to 1237.4)	599.0 (513.6 to 692.9)	288.8 (219.3 to 370.6)	188.8 (129.6 to 261.8)	13.0 (9.4 to 19.5)
Lebanon	2046	626.1 (542.9 to 713.0)	343.2 (293.5 to 397.4)	166.6 (126.4 to 214.4)	108.6 (74.7 to 150.7)	7.7 (5.5 to 11.7)	1097.5 (951.7 to 1249.9)	601.7 (514.6 to 696.7)	292.0 (221.5 to 375.8)	190.4 (130.9 to 264.2)	13.4 (9.7 to 20.5)
Lebanon	2047	630.9 (550.9 to 715.7)	344.8 (294.2 to 400.4)	168.6 (127.7 to 217.1)	109.6 (75.1 to 151.9)	7.9 (5.7 to 12.5)	1105.9 (965.8 to 1254.6)	604.5 (515.7 to 701.9)	295.5 (223.8 to 380.6)	192.0 (131.7 to 266.3)	13.9 (10.0 to 21.9)
Lebanon	2048	635.7 (559.5 to 722.4)	346.4 (294.7 to 403.0)	170.6 (129.0 to 220.7)	110.5 (75.5 to 153.2)	8.2 (5.9 to 13.1)	1114.3 (980.7 to 1266.4)	607.2 (516.7 to 706.5)	299.1 (226.1 to 386.9)	193.7 (132.4 to 268.6)	14.4 (10.3 to 22.9)
Lebanon	2049	639.8 (564.0 to 730.9)	347.4 (294.5 to 404.6)	172.6 (130.3 to 222.5)	111.3 (75.8 to 154.7)	8.5 (6.0 to 13.7)	1121.5 (988.7 to 1281.3)	609.0 (516.3 to 709.3)	302.5 (228.3 to 390.1)	195.1 (132.9 to 271.2)	14.9 (10.6 to 24.0)
Lebanon	2050	643.9 (567.0 to 737.5)	348.5 (294.5 to 406.2)	174.5 (131.5 to 225.4)	112.0 (76.1 to 155.7)	8.8 (6.2 to 14.5)	1128.8 (993.9 to 1292.8)	611.0 (516.3 to 712.1)	306.0 (230.5 to 395.0)	196.4 (133.3 to 272.9)	15.4 (10.9 to 25.4)

		2018 US Dollars per capita					2018 PPP per capita				
Country	Year	Health spending	Government health spending	Out-of-pocket health spending	Prepaid private health spending	Development assistance for health	Health spending	Government health spending	Out-of-pocket health spending	Prepaid private health spending	Development assistance for health
Lesotho	1995	36.9 (30.1 to 44.8)	15.7 (12.2 to 20.1)	16.0 (11.3 to 22.0)	3.4 (1.6 to 6.4)	1.9 (1.9 to 1.9)	97.8 (79.8 to 118.5)	41.6 (32.4 to 53.2)	42.2 (29.8 to 58.1)	8.9 (4.2 to 16.9)	5.0 (5.0 to 5.0)
Lesotho	1996	37.9 (31.1 to 45.9)	16.5 (12.8 to 21.2)	15.9 (11.2 to 21.9)	3.7 (1.8 to 6.8)	1.8 (1.8 to 1.8)	100.3 (82.3 to 121.4)	43.7 (33.9 to 56.0)	42.1 (29.8 to 57.9)	9.7 (4.7 to 18.1)	4.8 (4.8 to 4.8)
Lesotho	1997	37.6 (31.2 to 44.9)	16.3 (12.7 to 20.9)	15.2 (10.8 to 20.7)	3.8 (1.9 to 6.9)	2.2 (2.2 to 2.2)	99.5 (82.6 to 118.9)	43.2 (33.6 to 55.3)	40.3 (28.6 to 54.8)	10.0 (4.9 to 18.3)	5.9 (5.9 to 5.9)
Lesotho	1998	36.2 (30.2 to 43.5)	16.8 (13.1 to 21.3)	14.2 (10.2 to 19.4)	4.0 (2.0 to 7.4)	1.2 (1.2 to 1.2)	95.9 (79.9 to 115.1)	44.4 (34.7 to 56.2)	37.7 (27.1 to 51.4)	10.6 (5.2 to 19.7)	3.2 (3.2 to 3.2)
Lesotho	1999	35.8 (29.7 to 43.3)	17.5 (13.7 to 22.1)	13.6 (9.8 to 18.5)	4.2 (2.1 to 7.9)	0.5 (0.5 to 0.5)	94.7 (78.5 to 114.6)	46.2 (36.2 to 58.4)	36.1 (25.8 to 48.9)	11.2 (5.5 to 21.0)	1.2 (1.2 to 1.2)
Lesotho	2000	39.2 (32.9 to 46.6)	19.0 (14.9 to 24.0)	13.3 (9.3 to 18.2)	4.6 (2.2 to 8.6)	2.3 (2.3 to 2.3)	103.7 (87.2 to 123.2)	50.2 (39.6 to 63.4)	35.1 (24.7 to 48.2)	12.2 (5.8 to 22.6)	6.2 (6.2 to 6.2)
Lesotho	2001	42.1 (35.5 to 49.9)	20.9 (16.5 to 26.2)	12.9 (9.2 to 17.8)	4.6 (2.2 to 8.6)	3.7 (3.7 to 3.7)	111.3 (93.9 to 132.1)	55.2 (43.7 to 69.3)	34.2 (24.4 to 47.1)	12.1 (5.7 to 22.8)	9.8 (9.8 to 9.8)
Lesotho	2002	41.4 (35.1 to 48.6)	20.8 (16.4 to 26.3)	12.4 (9.0 to 17.2)	4.1 (2.0 to 7.7)	4.2 (4.2 to 4.2)	109.7 (92.8 to 128.6)	55.0 (43.5 to 69.7)	32.9 (23.7 to 45.6)	10.7 (5.2 to 20.3)	11.0 (11.0 to 11.0)
Lesotho	2003	43.9 (37.7 to 51.1)	21.0 (16.7 to 26.6)	12.3 (8.8 to 17.0)	3.1 (1.5 to 5.8)	7.5 (7.5 to 7.5)	116.3 (99.7 to 135.2)	55.7 (44.2 to 70.3)	32.6 (23.2 to 44.9)	8.3 (4.0 to 15.3)	19.8 (19.8 to 19.8)
Lesotho	2004	44.6 (38.4 to 51.6)	21.3 (16.9 to 26.9)	12.2 (8.6 to 16.8)	2.5 (1.2 to 4.7)	8.6 (8.6 to 8.6)	118.1 (101.6 to 136.6)	56.5 (44.8 to 71.3)	32.2 (22.7 to 44.4)	6.7 (3.3 to 12.5)	22.7 (22.7 to 22.7)
Lesotho	2005	44.8 (38.5 to 52.1)	21.8 (17.2 to 27.9)	12.2 (8.5 to 16.9)	2.3 (1.1 to 4.2)	8.6 (8.6 to 8.6)	118.5 (102.0 to 137.8)	57.6 (45.5 to 73.8)	32.3 (22.6 to 44.7)	6.0 (2.9 to 11.1)	22.6 (22.6 to 22.6)
Lesotho	2006	50.0 (43.2 to 57.9)	25.3 (19.9 to 31.9)	12.7 (8.9 to 17.4)	2.3 (1.1 to 4.3)	9.7 (9.7 to 9.7)	132.4 (114.3 to 153.1)	66.8 (52.8 to 84.5)	33.7 (23.7 to 46.1)	6.1 (3.0 to 11.3)	25.7 (25.7 to 25.7)
Lesotho	2007	62.7 (54.7 to 71.7)	30.9 (24.7 to 38.4)	13.8 (9.7 to 18.7)	2.5 (1.2 to 4.5)	15.4 (15.4 to 15.4)	165.9 (144.8 to 189.7)	81.8 (65.3 to 101.6)	36.5 (25.7 to 49.4)	6.6 (3.2 to 12.0)	40.8 (40.8 to 40.8)
Lesotho	2008	77.4 (69.0 to 86.8)	35.3 (28.3 to 43.4)	14.5 (10.2 to 19.7)	2.4 (1.2 to 4.4)	25.2 (25.2 to 25.2)	205.0 (182.6 to 229.6)	93.4 (74.8 to 114.9)	38.5 (27.1 to 52.1)	6.4 (3.1 to 11.6)	66.8 (66.8 to 66.8)
Lesotho	2009	78.0 (69.1 to 88.4)	39.2 (31.6 to 48.2)	15.2 (10.7 to 20.6)	2.0 (1.0 to 3.7)	21.5 (21.5 to 21.5)	206.4 (182.8 to 234.1)	103.7 (83.5 to 127.7)	40.3 (28.4 to 54.6)	5.4 (2.6 to 9.9)	57.0 (57.0 to 57.0)
Lesotho	2010	100.6 (91.2 to 112.2)	43.9 (35.5 to 53.8)	15.9 (11.3 to 21.7)	1.7 (0.8 to 3.1)	39.1 (39.1 to 39.1)	266.3 (241.4 to 296.9)	116.2 (93.9 to 142.3)	42.1 (29.9 to 57.4)	4.4 (2.1 to 8.1)	103.6 (103.6 to 103.6)
Lesotho	2011	118.4 (107.7 to 131.1)	49.7 (40.4 to 60.7)	16.2 (11.6 to 22.0)	1.1 (0.5 to 2.0)	51.5 (51.5 to 51.5)	313.4 (285.0 to 346.9)	131.4 (106.8 to 160.6)	42.8 (30.8 to 58.1)	2.8 (1.3 to 5.2)	136.4 (136.4 to 136.4)
Lesotho	2012	124.3 (112.9 to 137.6)	54.9 (44.8 to 67.1)	16.9 (12.2 to 22.9)	1.2 (0.6 to 2.2)	51.3 (51.3 to 51.3)	329.0 (298.8 to 364.1)	145.3 (118.5 to 177.5)	44.7 (32.2 to 60.7)	3.1 (1.5 to 5.8)	135.9 (135.9 to 135.9)
Lesotho	2013	143.2 (130.7 to 157.0)	59.1 (48.2 to 72.1)	17.1 (12.2 to 23.2)	1.3 (0.6 to 2.4)	65.7 (65.7 to 65.7)	378.9 (345.9 to 415.5)	156.5 (127.5 to 190.8)	45.1 (32.3 to 61.3)	3.3 (1.6 to 6.2)	174.0 (174.0 to 174.0)
Lesotho	2014	109.8 (95.9 to 124.1)	64.2 (52.2 to 78.8)	17.5 (12.5 to 23.5)	1.4 (0.7 to 2.6)	26.6 (26.6 to 26.6)	290.5 (253.9 to 328.4)	170.0 (138.1 to 208.4)	46.3 (33.1 to 62.1)	3.7 (1.8 to 6.9)	70.5 (70.5 to 70.5)
Lesotho	2015	109.3 (94.8 to 125.1)	67.5 (54.5 to 82.9)	18.0 (12.8 to 23.9)	1.6 (0.7 to 2.9)	22.3 (22.3 to 22.3)	289.4 (250.8 to 331.1)	178.6 (144.3 to 219.5)	47.6 (34.0 to 63.3)	4.1 (1.9 to 7.7)	59.0 (59.0 to 59.0)

		2018 US Dollars per capita					2018 PPP per capita				
Country	Year	Health spending	Government health spending	Out-of-pocket health spending	Prepaid private health spending	Development assistance for health	Health spending	Government health spending	Out-of-pocket health spending	Prepaid private health spending	Development assistance for health
Lesotho	2016	121.9 (106.6 to 138.6)	68.2 (54.7 to 84.3)	18.9 (13.3 to 25.6)	1.7 (0.8 to 3.2)	33.1 (33.1 to 33.1)	322.5 (282.1 to 366.9)	180.4 (144.9 to 223.2)	50.1 (35.1 to 67.7)	4.5 (2.1 to 8.3)	87.6 (87.6 to 87.6)
Lesotho	2017	138.7 (123.4 to 156.8)	69.5 (55.9 to 85.8)	19.5 (13.7 to 26.3)	1.7 (0.8 to 3.3)	48.0 (48.0 to 48.0)	367.1 (326.7 to 415.0)	183.9 (148.0 to 227.1)	51.5 (36.2 to 69.6)	4.6 (2.2 to 8.6)	127.1 (127.1 to 127.1)
Lesotho	2018	135.5 (119.6 to 153.1)	70.9 (57.3 to 87.5)	19.8 (14.0 to 26.9)	1.8 (0.8 to 3.4)	42.9 (42.3 to 43.5)	358.6 (316.5 to 405.2)	187.7 (151.6 to 231.5)	52.5 (37.2 to 71.1)	4.8 (2.2 to 8.9)	113.7 (111.9 to 115.2)
Lesotho	2019	137.5 (121.3 to 155.5)	72.6 (58.6 to 89.6)	20.3 (14.4 to 27.4)	1.9 (0.9 to 3.5)	42.8 (39.9 to 45.3)	363.9 (321.0 to 411.6)	192.2 (155.2 to 237.1)	53.6 (38.0 to 72.5)	4.9 (2.3 to 9.2)	113.2 (105.5 to 119.8)
Lesotho	2020	140.5 (123.2 to 158.0)	74.6 (60.2 to 92.0)	20.7 (14.7 to 27.9)	1.9 (0.9 to 3.6)	43.2 (39.6 to 47.0)	371.7 (326.0 to 418.2)	197.5 (159.3 to 243.5)	54.7 (38.8 to 73.9)	5.1 (2.4 to 9.4)	114.5 (104.7 to 124.3)
Lesotho	2021	143.7 (126.4 to 162.9)	76.6 (61.8 to 94.5)	21.1 (15.0 to 28.5)	2.0 (0.9 to 3.7)	44.0 (39.5 to 48.5)	380.3 (334.4 to 431.2)	202.9 (163.5 to 250.0)	55.8 (39.7 to 75.4)	5.2 (2.5 to 9.7)	116.5 (104.5 to 128.5)
Lesotho	2022	147.4 (128.9 to 166.8)	78.6 (63.4 to 96.8)	21.5 (15.3 to 29.0)	2.0 (1.0 to 3.8)	45.3 (40.2 to 50.9)	390.0 (341.1 to 441.4)	208.0 (167.7 to 256.2)	56.8 (40.5 to 76.9)	5.4 (2.5 to 10.0)	119.8 (106.3 to 134.8)
Lesotho	2023	151.0 (132.5 to 172.7)	80.5 (64.7 to 99.1)	21.8 (15.6 to 29.6)	2.1 (1.0 to 3.9)	46.6 (40.6 to 53.1)	399.7 (350.7 to 457.0)	213.0 (171.4 to 262.3)	57.8 (41.2 to 78.2)	5.5 (2.6 to 10.3)	123.4 (107.4 to 140.5)
Lesotho	2024	154.7 (134.4 to 176.5)	82.4 (66.2 to 101.5)	22.2 (15.8 to 30.2)	2.1 (1.0 to 4.0)	48.0 (41.4 to 55.8)	409.5 (355.8 to 467.1)	218.2 (175.1 to 268.6)	58.8 (41.8 to 79.9)	5.7 (2.7 to 10.5)	126.9 (109.6 to 147.6)
Lesotho	2025	158.3 (138.1 to 181.0)	84.5 (67.6 to 103.9)	22.6 (16.1 to 30.6)	2.2 (1.0 to 4.1)	49.1 (41.9 to 57.8)	419.0 (365.4 to 479.0)	223.5 (178.8 to 274.8)	59.8 (42.5 to 80.9)	5.8 (2.7 to 10.8)	129.9 (110.8 to 153.0)
Lesotho	2026	162.0 (140.5 to 185.0)	86.5 (69.0 to 106.5)	23.0 (16.3 to 31.1)	2.3 (1.1 to 4.2)	50.2 (42.5 to 59.2)	428.7 (372.0 to 489.6)	229.0 (182.7 to 281.7)	60.8 (43.2 to 82.4)	6.0 (2.8 to 11.0)	132.8 (112.5 to 156.8)
Lesotho	2027	165.8 (144.3 to 190.8)	88.7 (70.7 to 109.4)	23.4 (16.6 to 31.7)	2.3 (1.1 to 4.3)	51.4 (43.1 to 61.8)	438.9 (381.8 to 505.0)	234.7 (187.1 to 289.5)	61.9 (43.9 to 84.0)	6.1 (2.9 to 11.3)	136.2 (114.1 to 163.6)
Lesotho	2028	169.9 (146.6 to 195.0)	90.9 (72.6 to 112.4)	23.8 (16.9 to 32.4)	2.4 (1.1 to 4.4)	52.8 (44.1 to 64.2)	449.6 (388.0 to 516.0)	240.6 (192.1 to 297.6)	63.0 (44.7 to 85.8)	6.3 (3.0 to 11.7)	139.8 (116.8 to 169.9)
Lesotho	2029	174.1 (151.0 to 200.9)	93.2 (74.5 to 115.4)	24.3 (17.1 to 33.1)	2.4 (1.2 to 4.5)	54.2 (44.7 to 66.8)	460.7 (399.5 to 531.7)	246.7 (197.1 to 305.4)	64.2 (45.4 to 87.7)	6.5 (3.0 to 12.0)	143.4 (118.3 to 176.9)
Lesotho	2030	178.3 (153.7 to 205.4)	95.6 (76.1 to 118.2)	24.7 (17.4 to 33.8)	2.5 (1.2 to 4.6)	55.4 (45.6 to 68.4)	471.8 (406.7 to 543.6)	253.1 (201.5 to 312.9)	65.4 (46.1 to 89.4)	6.6 (3.1 to 12.3)	146.6 (120.7 to 181.0)
Lesotho	2031	182.5 (158.3 to 210.9)	98.2 (78.0 to 121.5)	25.2 (17.8 to 34.4)	2.6 (1.2 to 4.8)	56.6 (45.9 to 71.0)	483.1 (419.0 to 558.0)	259.8 (206.3 to 321.5)	66.7 (47.1 to 91.1)	6.8 (3.2 to 12.6)	149.7 (121.4 to 187.9)
Lesotho	2032	186.9 (161.0 to 216.3)	100.9 (80.0 to 124.7)	25.7 (18.2 to 35.1)	2.7 (1.3 to 4.9)	57.7 (46.6 to 72.6)	494.7 (426.2 to 572.4)	267.0 (211.6 to 330.0)	68.1 (48.2 to 92.8)	7.0 (3.3 to 13.0)	152.7 (123.4 to 192.3)
Lesotho	2033	191.5 (165.2 to 224.1)	103.7 (82.0 to 128.0)	26.2 (18.6 to 35.8)	2.7 (1.3 to 5.1)	58.8 (46.9 to 75.2)	506.8 (437.1 to 593.1)	274.4 (216.9 to 338.7)	69.5 (49.2 to 94.9)	7.3 (3.4 to 13.4)	155.7 (124.0 to 199.0)
Lesotho	2034	196.3 (169.3 to 228.1)	106.5 (84.0 to 131.4)	26.8 (18.9 to 36.6)	2.8 (1.3 to 5.2)	60.1 (47.3 to 77.8)	519.5 (448.1 to 603.6)	282.0 (222.3 to 347.6)	70.9 (50.1 to 96.9)	7.5 (3.5 to 13.8)	159.2 (125.2 to 205.8)
Lesotho	2035	201.2 (173.3 to 234.9)	109.5 (86.1 to 135.1)	27.3 (19.2 to 37.5)	2.9 (1.4 to 5.4)	61.4 (47.5 to 79.8)	532.4 (458.8 to 621.5)	289.8 (227.9 to 357.5)	72.4 (50.9 to 99.3)	7.7 (3.6 to 14.3)	162.5 (125.8 to 211.1)
Lesotho	2036	206.1 (174.5 to 240.5)	112.5 (88.6 to 138.8)	27.9 (19.5 to 38.3)	3.0 (1.4 to 5.6)	62.6 (48.6 to 83.9)	545.4 (461.7 to 636.5)	297.8 (234.5 to 367.4)	73.9 (51.6 to 101.4)	7.9 (3.7 to 14.7)	165.8 (128.5 to 222.1)

		2018 US Dollars per capita					2018 PPP per capita				
Country	Year	Health spending	Government health spending	Out-of-pocket health spending	Prepaid private health spending	Development assistance for health	Health spending	Government health spending	Out-of-pocket health spending	Prepaid private health spending	Development assistance for health
Lesotho	2037	211.0 (180.9 to 248.4)	115.7 (91.2 to 142.7)	28.5 (19.8 to 39.2)	3.1 (1.4 to 5.7)	63.7 (48.5 to 85.8)	558.4 (478.7 to 657.4)	306.2 (241.3 to 377.7)	75.4 (52.5 to 103.6)	8.2 (3.8 to 15.2)	168.6 (128.5 to 226.9)
Lesotho	2038	216.2 (182.7 to 254.3)	119.0 (93.9 to 147.1)	29.1 (20.3 to 40.1)	3.2 (1.5 to 5.9)	64.9 (49.6 to 90.8)	572.1 (483.6 to 673.1)	314.8 (248.4 to 389.2)	77.0 (53.6 to 106.2)	8.4 (3.9 to 15.7)	171.9 (131.3 to 240.2)
Lesotho	2039	221.3 (187.9 to 261.7)	122.3 (96.6 to 151.6)	29.7 (20.6 to 41.1)	3.3 (1.5 to 6.1)	66.1 (49.8 to 91.8)	585.7 (497.2 to 692.6)	323.6 (255.5 to 401.2)	78.7 (54.6 to 108.7)	8.7 (4.0 to 16.2)	174.8 (131.8 to 242.9)
Lesotho	2040	226.8 (191.5 to 268.3)	125.6 (99.1 to 156.2)	30.3 (21.1 to 42.1)	3.4 (1.6 to 6.3)	67.4 (51.3 to 95.2)	600.2 (506.7 to 710.0)	332.5 (262.1 to 413.5)	80.3 (55.7 to 111.3)	8.9 (4.2 to 16.7)	178.4 (135.7 to 251.9)
Lesotho	2041	232.3 (196.1 to 277.0)	129.1 (101.5 to 160.8)	31.0 (21.5 to 43.1)	3.5 (1.6 to 6.5)	68.8 (51.8 to 97.7)	614.9 (519.1 to 733.0)	341.6 (268.6 to 425.4)	82.0 (56.8 to 114.0)	9.2 (4.3 to 17.2)	182.2 (137.2 to 258.6)
Lesotho	2042	238.1 (199.0 to 283.9)	132.5 (104.1 to 165.3)	31.6 (21.8 to 43.9)	3.6 (1.7 to 6.7)	70.4 (51.8 to 102.7)	630.2 (526.8 to 751.4)	350.8 (275.5 to 437.6)	83.7 (57.7 to 116.3)	9.4 (4.4 to 17.7)	186.4 (137.1 to 271.8)
Lesotho	2043	243.9 (205.2 to 295.5)	136.0 (106.8 to 170.0)	32.3 (22.2 to 44.9)	3.7 (1.7 to 6.9)	72.0 (52.3 to 106.2)	645.6 (543.1 to 782.1)	360.0 (282.7 to 449.9)	85.4 (58.6 to 118.7)	9.7 (4.5 to 18.2)	190.5 (138.4 to 281.1)
Lesotho	2044	250.1 (207.8 to 300.6)	139.6 (109.6 to 174.7)	32.9 (22.6 to 45.8)	3.8 (1.8 to 7.1)	73.8 (53.1 to 110.7)	661.8 (550.0 to 795.6)	369.4 (290.1 to 462.3)	87.1 (59.8 to 121.1)	10.0 (4.6 to 18.8)	195.4 (140.5 to 293.0)
Lesotho	2045	256.3 (214.1 to 312.8)	143.1 (112.4 to 179.4)	33.5 (23.1 to 46.6)	3.9 (1.8 to 7.3)	75.8 (53.6 to 116.8)	678.3 (566.7 to 827.8)	378.7 (297.4 to 474.7)	88.8 (61.0 to 123.4)	10.3 (4.8 to 19.3)	200.6 (141.8 to 309.0)
Lesotho	2046	262.6 (217.8 to 319.1)	146.6 (115.1 to 184.0)	34.2 (23.5 to 47.5)	4.0 (1.9 to 7.5)	77.8 (54.5 to 122.6)	694.9 (576.4 to 844.5)	388.0 (304.7 to 486.9)	90.5 (62.3 to 125.6)	10.5 (4.9 to 19.8)	206.0 (144.1 to 324.5)
Lesotho	2047	269.1 (225.1 to 335.1)	150.0 (117.6 to 188.6)	34.8 (24.0 to 48.3)	4.1 (1.9 to 7.7)	80.2 (55.8 to 129.1)	712.1 (595.7 to 886.9)	397.0 (311.1 to 499.2)	92.1 (63.5 to 127.8)	10.8 (5.1 to 20.4)	212.2 (147.6 to 341.7)
Lesotho	2048	276.0 (225.5 to 341.9)	153.6 (120.3 to 193.5)	35.4 (24.4 to 49.1)	4.2 (2.0 to 7.9)	82.8 (57.0 to 133.4)	730.4 (596.7 to 904.8)	406.4 (318.4 to 512.0)	93.8 (64.6 to 129.9)	11.1 (5.2 to 20.9)	219.1 (151.0 to 353.1)
Lesotho	2049	282.9 (232.3 to 351.7)	157.1 (123.0 to 198.3)	36.1 (24.8 to 50.0)	4.3 (2.0 to 8.1)	85.4 (58.0 to 139.0)	748.7 (614.7 to 930.9)	415.9 (325.6 to 524.9)	95.5 (65.7 to 132.2)	11.4 (5.3 to 21.5)	226.0 (153.4 to 367.8)
Lesotho	2050	290.3 (237.9 to 361.0)	160.7 (125.6 to 203.1)	36.7 (25.3 to 50.8)	4.4 (2.1 to 8.3)	88.5 (59.9 to 147.9)	768.2 (629.5 to 955.4)	425.2 (332.5 to 537.5)	97.2 (66.9 to 134.5)	11.7 (5.5 to 22.0)	234.1 (158.5 to 391.5)
Liberia	1995	10.2 (7.6 to 13.3)	1.6 (1.2 to 2.1)	7.9 (5.4 to 11.0)	0.3 (0.1 to 0.5)	0.4 (0.4 to 0.4)	22.5 (16.7 to 29.4)	3.6 (2.7 to 4.7)	17.6 (12.0 to 24.2)	0.6 (0.3 to 1.0)	0.8 (0.8 to 0.8)
Liberia	1996	10.3 (7.8 to 13.2)	1.7 (1.3 to 2.2)	7.7 (5.3 to 10.5)	0.2 (0.1 to 0.4)	0.8 (0.8 to 0.8)	22.9 (17.3 to 29.2)	3.7 (2.8 to 4.8)	16.9 (11.8 to 23.2)	0.5 (0.2 to 0.9)	1.7 (1.7 to 1.7)
Liberia	1997	14.1 (11.1 to 17.6)	2.5 (1.9 to 3.3)	9.2 (6.4 to 12.7)	0.3 (0.1 to 0.6)	2.0 (2.0 to 2.0)	31.1 (24.5 to 38.9)	5.6 (4.2 to 7.3)	20.4 (14.2 to 28.2)	0.7 (0.3 to 1.3)	4.4 (4.4 to 4.4)
Liberia	1998	15.6 (12.4 to 19.4)	2.8 (2.1 to 3.7)	9.5 (6.6 to 13.3)	0.3 (0.2 to 0.6)	2.9 (2.9 to 2.9)	34.5 (27.5 to 42.8)	6.2 (4.7 to 8.1)	21.1 (14.6 to 29.4)	0.7 (0.3 to 1.3)	6.5 (6.5 to 6.5)
Liberia	1999	15.0 (12.0 to 18.7)	2.7 (2.0 to 3.5)	9.1 (6.3 to 12.7)	0.3 (0.1 to 0.6)	2.9 (2.9 to 2.9)	33.1 (26.6 to 41.4)	5.9 (4.4 to 7.8)	20.2 (14.0 to 28.1)	0.7 (0.3 to 1.2)	6.4 (6.4 to 6.4)
Liberia	2000	14.9 (12.4 to 18.3)	2.5 (1.8 to 3.3)	7.9 (5.5 to 11.2)	0.3 (0.1 to 0.5)	4.3 (4.3 to 4.3)	33.0 (27.4 to 40.4)	5.4 (4.0 to 7.2)	17.5 (12.2 to 24.7)	0.7 (0.3 to 1.2)	9.4 (9.4 to 9.4)
Liberia	2001	13.3 (10.6 to 16.6)	2.5 (1.9 to 3.3)	8.0 (5.6 to 11.1)	0.3 (0.2 to 0.6)	2.5 (2.5 to 2.5)	29.5 (23.5 to 36.8)	5.5 (4.2 to 7.3)	17.7 (12.4 to 24.6)	0.8 (0.3 to 1.4)	5.4 (5.4 to 5.4)

		2018 US Dollars per capita					2018 PPP per capita				
Country	Year	Health spending	Government health spending	Out-of-pocket health spending	Prepaid private health spending	Development assistance for health	Health spending	Government health spending	Out-of-pocket health spending	Prepaid private health spending	Development assistance for health
Liberia	2002	14.7 (11.7 to 18.7)	2.6 (2.0 to 3.4)	9.6 (6.7 to 13.3)	0.4 (0.2 to 0.7)	2.1 (2.1 to 2.1)	32.4 (25.8 to 41.3)	5.8 (4.4 to 7.6)	21.1 (14.9 to 29.3)	0.9 (0.4 to 1.6)	4.6 (4.6 to 4.6)
Liberia	2003	14.7 (11.8 to 18.5)	1.9 (1.4 to 2.5)	9.4 (6.5 to 13.2)	0.3 (0.1 to 0.5)	3.1 (3.1 to 3.1)	32.6 (26.1 to 40.9)	4.2 (3.2 to 5.6)	20.8 (14.4 to 29.1)	0.6 (0.3 to 1.2)	6.9 (6.9 to 6.9)
Liberia	2004	20.4 (16.3 to 25.4)	2.0 (1.5 to 2.6)	12.9 (9.0 to 17.7)	0.3 (0.1 to 0.6)	5.3 (5.3 to 5.3)	45.1 (36.2 to 56.1)	4.4 (3.3 to 5.8)	28.4 (20.0 to 39.1)	0.6 (0.3 to 1.2)	11.6 (11.6 to 11.6)
Liberia	2005	23.6 (18.9 to 29.4)	2.1 (1.5 to 2.8)	15.3 (10.6 to 20.9)	0.3 (0.1 to 0.6)	5.9 (5.9 to 5.9)	52.2 (41.8 to 65.1)	4.6 (3.4 to 6.1)	33.8 (23.5 to 46.3)	0.7 (0.3 to 1.3)	13.1 (13.1 to 13.1)
Liberia	2006	27.1 (21.7 to 33.5)	2.5 (1.9 to 3.4)	17.2 (11.9 to 23.6)	0.4 (0.2 to 0.7)	7.0 (7.0 to 7.0)	60.1 (48.1 to 74.2)	5.6 (4.2 to 7.4)	38.0 (26.3 to 52.3)	0.8 (0.4 to 1.6)	15.6 (15.6 to 15.6)
Liberia	2007	31.4 (25.2 to 39.1)	3.3 (2.5 to 4.4)	20.5 (14.3 to 27.7)	0.6 (0.3 to 1.1)	7.0 (7.0 to 7.0)	69.4 (55.7 to 86.6)	7.3 (5.5 to 9.6)	45.3 (31.5 to 61.4)	1.3 (0.6 to 2.4)	15.6 (15.6 to 15.6)
Liberia	2008	44.9 (38.0 to 53.4)	4.4 (3.3 to 5.8)	22.8 (16.0 to 31.1)	0.9 (0.5 to 1.8)	16.6 (16.6 to 16.6)	99.3 (84.2 to 118.1)	9.8 (7.3 to 12.9)	50.6 (35.4 to 68.7)	2.1 (1.0 to 3.9)	36.8 (36.8 to 36.8)
Liberia	2009	54.3 (47.1 to 63.4)	4.8 (3.6 to 6.4)	24.7 (17.5 to 33.6)	1.2 (0.6 to 2.3)	23.5 (23.5 to 23.5)	120.2 (104.3 to 140.2)	10.7 (8.0 to 14.1)	54.7 (38.7 to 74.3)	2.7 (1.3 to 5.1)	52.1 (52.1 to 52.1)
Liberia	2010	56.8 (49.2 to 65.8)	5.1 (3.8 to 6.7)	25.9 (18.7 to 35.0)	1.4 (0.7 to 2.8)	24.4 (24.4 to 24.4)	125.8 (109.0 to 145.6)	11.3 (8.4 to 14.8)	57.4 (41.3 to 77.5)	3.2 (1.6 to 6.2)	53.9 (53.9 to 53.9)
Liberia	2011	56.8 (49.3 to 65.7)	5.5 (4.1 to 7.2)	25.9 (18.8 to 34.6)	1.6 (0.8 to 3.1)	23.8 (23.8 to 23.8)	125.7 (109.0 to 145.5)	12.1 (9.1 to 15.8)	57.4 (41.6 to 76.5)	3.6 (1.7 to 6.9)	52.6 (52.6 to 52.6)
Liberia	2012	59.8 (52.3 to 68.8)	4.9 (3.6 to 6.3)	25.5 (18.4 to 34.0)	1.6 (0.7 to 3.1)	27.9 (27.9 to 27.9)	132.4 (115.8 to 152.2)	10.8 (8.0 to 14.0)	56.5 (40.8 to 75.2)	3.5 (1.6 to 6.8)	61.6 (61.6 to 61.6)
Liberia	2013	57.7 (50.3 to 67.1)	4.3 (3.2 to 5.6)	26.5 (19.2 to 35.5)	1.6 (0.7 to 3.0)	25.2 (25.2 to 25.2)	127.6 (111.4 to 148.5)	9.6 (7.1 to 12.4)	58.6 (42.5 to 78.6)	3.5 (1.7 to 6.7)	55.9 (55.9 to 55.9)
Liberia	2014	99.5 (90.9 to 110.4)	5.4 (4.0 to 7.1)	30.3 (21.6 to 40.8)	2.2 (1.0 to 4.2)	61.6 (61.6 to 61.6)	220.2 (201.2 to 244.3)	12.0 (8.9 to 15.7)	67.0 (47.9 to 90.2)	4.8 (2.2 to 9.2)	136.4 (136.4 to 136.4)
Liberia	2015	157.7 (148.0 to 170.1)	6.7 (5.0 to 8.7)	33.1 (23.8 to 44.8)	3.7 (1.7 to 7.2)	114.2 (114.2 to 114.2)	349.0 (327.5 to 376.3)	14.7 (11.0 to 19.2)	73.3 (52.7 to 99.1)	8.2 (3.8 to 16.0)	252.8 (252.8 to 252.8)
Liberia	2016	80.9 (70.8 to 94.0)	7.7 (5.7 to 10.0)	34.5 (24.8 to 46.8)	4.8 (2.1 to 9.4)	34.0 (34.0 to 34.0)	179.1 (156.6 to 208.0)	17.0 (12.6 to 22.1)	76.3 (54.9 to 103.6)	10.6 (4.7 to 20.7)	75.2 (75.2 to 75.2)
Liberia	2017	72.4 (62.2 to 85.5)	7.6 (5.7 to 9.9)	34.3 (24.7 to 46.7)	4.6 (2.0 to 9.0)	25.9 (25.9 to 25.9)	160.3 (137.7 to 189.2)	16.9 (12.6 to 21.9)	75.9 (54.6 to 103.4)	10.1 (4.5 to 19.9)	57.4 (57.4 to 57.4)
Liberia	2018	70.3 (60.1 to 83.1)	7.7 (5.7 to 10.0)	34.6 (24.8 to 47.2)	4.7 (2.1 to 9.1)	23.3 (23.2 to 23.4)	155.6 (133.1 to 183.8)	17.0 (12.7 to 22.1)	76.6 (55.0 to 104.4)	10.3 (4.6 to 20.2)	51.6 (51.4 to 51.8)
Liberia	2019	71.2 (60.4 to 84.3)	7.8 (5.8 to 10.1)	35.2 (25.2 to 48.3)	4.7 (2.1 to 9.2)	23.4 (21.9 to 24.7)	157.5 (133.7 to 186.6)	17.2 (12.9 to 22.3)	78.0 (55.7 to 107.0)	10.5 (4.6 to 20.4)	51.8 (48.4 to 54.8)
Liberia	2020	72.0 (61.0 to 85.8)	7.8 (5.8 to 10.1)	35.7 (25.3 to 48.7)	4.8 (2.1 to 9.3)	23.8 (21.8 to 25.7)	159.4 (135.1 to 189.8)	17.3 (12.9 to 22.4)	78.9 (56.1 to 107.7)	10.6 (4.7 to 20.6)	52.6 (48.2 to 56.9)
Liberia	2021	72.9 (61.7 to 87.6)	7.9 (5.9 to 10.2)	35.9 (25.6 to 48.9)	4.8 (2.1 to 9.4)	24.3 (21.9 to 26.8)	161.2 (136.5 to 193.8)	17.4 (13.0 to 22.6)	79.3 (56.6 to 108.3)	10.7 (4.7 to 20.7)	53.8 (48.5 to 59.3)
Liberia	2022	74.1 (63.1 to 88.5)	7.9 (5.9 to 10.3)	36.3 (25.8 to 49.7)	4.9 (2.2 to 9.5)	25.0 (22.2 to 27.8)	163.9 (139.5 to 195.7)	17.6 (13.1 to 22.9)	80.3 (57.1 to 110.0)	10.8 (4.8 to 21.0)	55.2 (49.1 to 61.6)

Country	Year	2018 US Dollars per capita					2018 PPP per capita				
		Health spending	Government health spending	Out-of-pocket health spending	Prepaid private health spending	Development assistance for health	Health spending	Government health spending	Out-of-pocket health spending	Prepaid private health spending	Development assistance for health
Liberia	2023	75.8 (64.3 to 89.6)	8.0 (6.0 to 10.4)	37.2 (26.4 to 50.9)	5.0 (2.2 to 9.6)	25.6 (22.2 to 29.0)	167.7 (142.3 to 198.3)	17.7 (13.2 to 23.0)	82.3 (58.5 to 112.6)	11.0 (4.9 to 21.2)	56.7 (49.1 to 64.2)
Liberia	2024	77.5 (65.3 to 91.9)	8.1 (6.0 to 10.5)	38.0 (27.1 to 51.6)	5.0 (2.2 to 9.7)	26.4 (22.7 to 30.4)	171.4 (144.4 to 203.3)	17.9 (13.3 to 23.2)	84.0 (60.0 to 114.1)	11.1 (4.9 to 21.4)	58.4 (50.3 to 67.2)
Liberia	2025	79.5 (67.0 to 94.7)	8.1 (6.1 to 10.5)	39.1 (27.8 to 53.3)	5.1 (2.3 to 9.8)	27.1 (23.0 to 31.7)	175.8 (148.2 to 209.7)	18.0 (13.5 to 23.3)	86.6 (61.5 to 117.8)	11.3 (5.0 to 21.7)	59.9 (51.0 to 70.2)
Liberia	2026	81.5 (68.5 to 97.4)	8.2 (6.1 to 10.7)	40.3 (28.9 to 55.0)	5.2 (2.3 to 10.0)	27.7 (23.4 to 32.8)	180.4 (151.6 to 215.5)	18.2 (13.6 to 23.7)	89.3 (63.9 to 121.6)	11.4 (5.1 to 22.1)	61.4 (51.7 to 72.6)
Liberia	2027	83.6 (70.2 to 100.3)	8.3 (6.2 to 10.8)	41.5 (29.7 to 56.4)	5.2 (2.3 to 10.1)	28.5 (23.7 to 34.3)	185.1 (155.3 to 222.0)	18.4 (13.7 to 24.0)	91.9 (65.8 to 124.9)	11.6 (5.1 to 22.4)	63.2 (52.5 to 75.9)
Liberia	2028	85.9 (72.0 to 103.3)	8.4 (6.3 to 10.9)	42.8 (30.4 to 58.2)	5.3 (2.3 to 10.3)	29.4 (24.4 to 35.8)	190.0 (159.4 to 228.5)	18.6 (13.8 to 24.1)	94.7 (67.3 to 128.8)	11.7 (5.2 to 22.7)	65.0 (54.1 to 79.2)
Liberia	2029	88.1 (73.4 to 105.5)	8.5 (6.3 to 11.0)	44.0 (31.0 to 59.7)	5.4 (2.4 to 10.4)	30.3 (24.7 to 37.7)	195.0 (162.4 to 233.5)	18.8 (14.0 to 24.4)	97.3 (68.5 to 132.0)	11.9 (5.2 to 23.1)	67.1 (54.6 to 83.3)
Liberia	2030	90.4 (74.8 to 108.5)	8.6 (6.4 to 11.2)	45.2 (31.8 to 61.4)	5.5 (2.4 to 10.6)	31.2 (25.3 to 38.6)	200.0 (165.5 to 240.0)	19.0 (14.1 to 24.7)	100.0 (70.4 to 135.8)	12.1 (5.3 to 23.4)	68.9 (56.0 to 85.5)
Liberia	2031	92.7 (76.1 to 112.0)	8.7 (6.5 to 11.3)	46.4 (32.8 to 63.2)	5.5 (2.4 to 10.7)	32.1 (25.8 to 40.7)	205.2 (168.4 to 247.7)	19.3 (14.3 to 25.1)	102.7 (72.5 to 139.8)	12.3 (5.4 to 23.7)	70.9 (57.0 to 90.0)
Liberia	2032	95.1 (78.0 to 115.3)	8.8 (6.6 to 11.6)	47.6 (33.5 to 65.1)	5.6 (2.5 to 10.8)	33.1 (26.1 to 42.2)	210.4 (172.5 to 255.2)	19.6 (14.5 to 25.6)	105.3 (74.0 to 144.0)	12.4 (5.5 to 24.0)	73.2 (57.7 to 93.3)
Liberia	2033	97.5 (79.6 to 117.9)	9.0 (6.6 to 11.7)	48.8 (33.8 to 67.1)	5.7 (2.5 to 11.0)	34.1 (26.5 to 44.4)	215.8 (176.2 to 260.9)	19.8 (14.7 to 25.8)	107.9 (74.8 to 148.5)	12.6 (5.5 to 24.3)	75.4 (58.6 to 98.2)
Liberia	2034	100.1 (81.4 to 122.2)	9.1 (6.7 to 12.0)	49.9 (34.2 to 69.0)	5.8 (2.5 to 11.1)	35.3 (27.1 to 46.5)	221.5 (180.2 to 270.3)	20.1 (14.9 to 26.5)	110.5 (75.6 to 152.7)	12.8 (5.6 to 24.6)	78.1 (60.0 to 102.9)
Liberia	2035	102.7 (82.9 to 125.9)	9.2 (6.8 to 12.1)	51.1 (34.7 to 70.6)	5.9 (2.6 to 11.3)	36.5 (27.7 to 48.2)	227.2 (183.4 to 278.6)	20.4 (15.1 to 26.8)	113.0 (76.8 to 156.3)	13.0 (5.7 to 24.9)	80.8 (61.2 to 106.7)
Liberia	2036	105.3 (84.9 to 130.4)	9.4 (6.9 to 12.3)	52.2 (35.2 to 72.6)	6.0 (2.6 to 11.5)	37.7 (28.4 to 51.5)	232.9 (187.8 to 288.6)	20.7 (15.2 to 27.3)	115.5 (77.9 to 160.6)	13.2 (5.8 to 25.3)	83.5 (62.9 to 114.0)
Liberia	2037	107.8 (85.6 to 133.7)	9.5 (7.0 to 12.5)	53.2 (35.9 to 74.4)	6.1 (2.7 to 11.6)	39.0 (29.1 to 53.8)	238.5 (189.4 to 295.8)	21.0 (15.5 to 27.7)	117.8 (79.5 to 164.7)	13.4 (5.9 to 25.7)	86.3 (64.5 to 119.0)
Liberia	2038	110.4 (87.5 to 137.0)	9.6 (7.1 to 12.7)	54.2 (36.4 to 76.2)	6.1 (2.7 to 11.8)	40.4 (29.9 to 57.6)	244.3 (193.7 to 303.1)	21.3 (15.7 to 28.2)	120.0 (80.6 to 168.7)	13.6 (6.0 to 26.0)	89.4 (66.1 to 127.4)
Liberia	2039	113.0 (90.0 to 140.3)	9.8 (7.2 to 13.0)	55.3 (36.9 to 78.1)	6.2 (2.8 to 11.9)	41.7 (30.6 to 59.4)	250.1 (199.0 to 310.5)	21.7 (15.9 to 28.7)	122.3 (81.7 to 172.7)	13.8 (6.1 to 26.3)	92.4 (67.7 to 131.4)
Liberia	2040	115.7 (92.3 to 143.3)	9.9 (7.3 to 13.2)	56.2 (37.4 to 79.8)	6.3 (2.8 to 12.0)	43.3 (31.9 to 63.0)	256.1 (204.3 to 317.0)	22.0 (16.1 to 29.1)	124.4 (82.7 to 176.5)	14.0 (6.2 to 26.6)	95.8 (70.6 to 139.3)
Liberia	2041	118.6 (93.9 to 149.1)	10.1 (7.4 to 13.4)	57.2 (37.9 to 81.6)	6.4 (2.8 to 12.2)	44.9 (32.2 to 65.4)	262.4 (207.7 to 330.0)	22.4 (16.4 to 29.6)	126.5 (83.9 to 180.5)	14.2 (6.3 to 27.1)	99.3 (71.3 to 144.8)
Liberia	2042	121.5 (95.7 to 153.0)	10.3 (7.5 to 13.6)	58.0 (38.4 to 83.2)	6.5 (2.9 to 12.4)	46.7 (33.2 to 67.4)	268.8 (211.9 to 338.5)	22.7 (16.6 to 30.1)	128.3 (84.9 to 184.1)	14.4 (6.4 to 27.5)	103.3 (73.4 to 149.2)
Liberia	2043	124.3 (97.3 to 159.3)	10.4 (7.6 to 13.8)	58.8 (38.8 to 84.8)	6.6 (2.9 to 12.6)	48.4 (34.3 to 70.9)	275.1 (215.3 to 352.4)	23.1 (16.9 to 30.6)	130.2 (85.8 to 187.6)	14.7 (6.5 to 27.9)	107.1 (75.9 to 157.0)

		2018 US Dollars per capita					2018 PPP per capita				
Country	Year	Health spending	Government health spending	Out-of-pocket health spending	Prepaid private health spending	Development assistance for health	Health spending	Government health spending	Out-of-pocket health spending	Prepaid private health spending	Development assistance for health
Liberia	2044	127.3 (98.6 to 161.4)	10.6 (7.8 to 14.1)	59.6 (39.2 to 86.3)	6.7 (3.0 to 12.8)	50.3 (35.1 to 75.8)	281.7 (218.1 to 357.2)	23.5 (17.2 to 31.1)	131.9 (86.8 to 191.1)	14.9 (6.6 to 28.4)	111.4 (77.6 to 167.7)
Liberia	2045	130.3 (100.5 to 167.3)	10.8 (7.9 to 14.3)	60.4 (39.8 to 87.7)	6.8 (3.0 to 13.0)	52.3 (36.3 to 80.2)	288.4 (222.3 to 370.2)	23.9 (17.4 to 31.8)	133.6 (88.1 to 194.2)	15.1 (6.7 to 28.9)	115.7 (80.2 to 177.5)
Liberia	2046	133.4 (101.5 to 171.9)	11.0 (8.0 to 14.7)	61.1 (40.0 to 88.8)	7.0 (3.1 to 13.3)	54.4 (36.8 to 86.1)	295.1 (224.6 to 380.4)	24.3 (17.7 to 32.4)	135.1 (88.5 to 196.5)	15.4 (6.8 to 29.3)	120.3 (81.4 to 190.4)
Liberia	2047	136.6 (104.7 to 177.1)	11.2 (8.2 to 14.9)	61.6 (40.1 to 89.9)	7.1 (3.1 to 13.5)	56.7 (39.1 to 89.4)	302.2 (231.7 to 392.0)	24.7 (18.1 to 32.9)	136.4 (88.8 to 199.0)	15.6 (6.9 to 29.8)	125.5 (86.5 to 197.7)
Liberia	2048	140.0 (106.4 to 185.0)	11.4 (8.3 to 15.2)	62.3 (40.5 to 91.4)	7.2 (3.2 to 13.7)	59.1 (39.7 to 93.5)	309.8 (235.5 to 409.4)	25.2 (18.3 to 33.6)	137.9 (89.6 to 202.3)	15.9 (7.0 to 30.3)	130.8 (87.8 to 206.8)
Liberia	2049	143.4 (109.8 to 194.2)	11.6 (8.4 to 15.5)	63.0 (40.8 to 92.3)	7.3 (3.2 to 13.9)	61.6 (41.4 to 100.4)	317.2 (243.0 to 429.8)	25.6 (18.6 to 34.2)	139.3 (90.3 to 204.2)	16.1 (7.1 to 30.8)	136.2 (91.5 to 222.1)
Liberia	2050	147.1 (112.8 to 198.9)	11.8 (8.5 to 15.7)	63.6 (41.2 to 93.4)	7.4 (3.3 to 14.1)	64.4 (42.7 to 109.4)	325.6 (249.5 to 440.2)	26.0 (18.9 to 34.8)	140.8 (91.1 to 206.6)	16.4 (7.2 to 31.3)	142.4 (94.6 to 242.2)
Libya	1995	415.3 (373.8 to 460.5)	193.7 (166.7 to 225.1)	207.0 (178.2 to 240.8)	14.5 (6.8 to 27.3)	0.0 (0.0 to 0.0)	756.4 (680.7 to 838.7)	352.9 (303.6 to 410.0)	377.0 (324.5 to 438.6)	26.5 (12.3 to 49.7)	0.0 (0.0 to 0.0)
Libya	1996	429.1 (390.2 to 472.8)	205.1 (177.6 to 235.9)	208.9 (182.8 to 237.6)	15.1 (7.1 to 27.9)	0.0 (0.0 to 0.0)	781.5 (710.7 to 861.0)	373.5 (323.4 to 429.7)	380.4 (333.0 to 432.7)	27.6 (12.9 to 50.8)	0.0 (0.0 to 0.0)
Libya	1997	444.5 (405.1 to 484.2)	214.9 (187.8 to 243.2)	213.6 (189.0 to 239.9)	16.0 (7.7 to 29.3)	0.0 (0.0 to 0.0)	809.6 (737.8 to 881.8)	391.4 (342.0 to 443.0)	388.9 (344.2 to 436.9)	29.2 (14.0 to 53.4)	0.0 (0.0 to 0.0)
Libya	1998	454.4 (416.9 to 495.1)	217.5 (192.0 to 243.6)	220.1 (196.0 to 246.4)	16.7 (8.0 to 31.4)	0.0 (0.0 to 0.0)	827.5 (759.3 to 901.6)	396.1 (349.6 to 443.6)	400.9 (357.0 to 448.8)	30.5 (14.6 to 57.2)	0.0 (0.0 to 0.0)
Libya	1999	453.8 (417.0 to 493.7)	219.1 (193.8 to 245.1)	217.2 (192.8 to 242.4)	17.5 (8.5 to 33.2)	0.0 (0.0 to 0.0)	826.5 (759.5 to 899.2)	399.1 (352.9 to 446.5)	395.6 (351.0 to 441.5)	31.8 (15.4 to 60.5)	0.0 (0.0 to 0.0)
Libya	2000	467.4 (427.9 to 505.8)	237.3 (211.1 to 264.6)	210.8 (186.8 to 234.5)	19.4 (9.1 to 37.1)	0.0 (0.0 to 0.0)	851.3 (779.3 to 921.1)	432.1 (384.5 to 482.0)	383.9 (340.2 to 427.0)	35.3 (16.5 to 67.5)	0.0 (0.0 to 0.0)
Libya	2001	484.9 (445.5 to 521.8)	263.2 (236.8 to 291.0)	200.4 (176.9 to 224.6)	21.4 (9.9 to 42.0)	0.0 (0.0 to 0.0)	883.1 (811.4 to 950.3)	479.3 (431.3 to 529.9)	364.9 (322.2 to 409.0)	38.9 (18.1 to 76.4)	0.0 (0.0 to 0.0)
Libya	2002	494.1 (455.5 to 532.7)	288.9 (260.3 to 318.9)	182.2 (160.7 to 203.9)	23.0 (10.5 to 44.3)	0.0 (0.0 to 0.0)	899.9 (829.5 to 970.2)	526.2 (474.1 to 580.8)	331.7 (292.6 to 371.3)	42.0 (19.1 to 80.8)	0.0 (0.0 to 0.0)
Libya	2003	497.2 (459.7 to 538.3)	304.2 (275.4 to 337.4)	168.8 (148.7 to 190.7)	24.2 (11.0 to 46.4)	0.0 (0.0 to 0.0)	905.5 (837.1 to 980.4)	554.0 (501.6 to 614.4)	307.5 (270.9 to 347.3)	44.0 (20.0 to 84.5)	0.0 (0.0 to 0.0)
Libya	2004	449.5 (415.4 to 488.4)	279.1 (253.6 to 309.1)	147.8 (129.3 to 167.0)	22.5 (10.3 to 42.7)	0.0 (0.0 to 0.0)	818.6 (756.5 to 889.5)	508.3 (461.8 to 562.9)	269.3 (235.4 to 304.1)	41.0 (18.8 to 77.7)	0.0 (0.0 to 0.0)
Libya	2005	417.8 (385.4 to 454.9)	262.8 (238.8 to 291.5)	133.5 (116.5 to 151.8)	21.5 (9.8 to 40.4)	0.1 (0.1 to 0.1)	761.0 (702.0 to 828.5)	478.5 (435.0 to 530.8)	243.2 (212.2 to 276.4)	39.2 (17.9 to 73.6)	0.1 (0.1 to 0.1)
Libya	2006	401.3 (368.2 to 437.6)	254.3 (229.2 to 283.1)	126.0 (109.8 to 142.9)	20.9 (9.5 to 40.2)	0.1 (0.1 to 0.1)	730.8 (670.7 to 796.9)	463.1 (417.5 to 515.5)	229.4 (199.9 to 260.2)	38.1 (17.4 to 73.3)	0.2 (0.2 to 0.2)
Libya	2007	405.4 (372.0 to 442.4)	259.8 (234.0 to 288.0)	123.8 (108.0 to 140.1)	21.4 (10.0 to 41.9)	0.3 (0.3 to 0.3)	738.3 (677.5 to 805.7)	473.1 (426.2 to 524.5)	225.5 (196.6 to 255.2)	39.1 (18.3 to 76.3)	0.6 (0.6 to 0.6)
Libya	2008	428.2 (392.2 to 466.9)	274.1 (247.5 to 301.5)	125.6 (108.8 to 143.7)	22.6 (10.7 to 44.1)	5.9 (5.9 to 5.9)	779.9 (714.2 to 850.3)	499.3 (450.7 to 549.1)	228.7 (198.2 to 261.7)	41.2 (19.4 to 80.3)	10.7 (10.7 to 10.7)

Country	Year	2018 US Dollars per capita					2018 PPP per capita				
		Health spending	Government health spending	Out-of-pocket health spending	Prepaid private health spending	Development assistance for health	Health spending	Government health spending	Out-of-pocket health spending	Prepaid private health spending	Development assistance for health
Libya	2009	484.1 (445.3 to 525.1)	313.9 (282.7 to 345.4)	141.4 (122.7 to 161.9)	25.3 (12.2 to 49.8)	3.5 (3.5 to 3.5)	881.7 (811.0 to 956.2)	571.6 (514.8 to 629.0)	257.6 (223.5 to 294.8)	46.1 (22.1 to 90.8)	6.4 (6.4 to 6.4)
Libya	2010	548.9 (507.5 to 595.6)	364.6 (329.6 to 402.9)	155.4 (135.2 to 177.7)	28.7 (13.9 to 56.9)	0.2 (0.2 to 0.2)	999.6 (924.3 to 1084.7)	664.1 (600.2 to 733.7)	283.0 (246.2 to 323.7)	52.2 (25.3 to 103.6)	0.3 (0.3 to 0.3)
Libya	2011	309.8 (285.3 to 335.7)	185.7 (167.9 to 206.2)	106.9 (92.4 to 122.8)	15.2 (7.2 to 28.5)	2.0 (2.0 to 2.0)	564.2 (519.6 to 611.4)	338.2 (305.9 to 375.6)	194.7 (168.2 to 223.7)	27.7 (13.2 to 51.9)	3.7 (3.7 to 3.7)
Libya	2012	547.9 (501.6 to 597.9)	368.2 (332.0 to 411.5)	151.7 (128.9 to 175.1)	27.6 (12.8 to 52.2)	0.3 (0.3 to 0.3)	997.8 (913.5 to 1088.9)	670.6 (604.6 to 749.4)	276.3 (234.8 to 318.9)	50.3 (23.4 to 95.1)	0.6 (0.6 to 0.6)
Libya	2013	414.4 (375.8 to 459.1)	275.3 (244.4 to 310.3)	117.4 (98.1 to 138.0)	21.0 (9.8 to 39.1)	0.7 (0.7 to 0.7)	754.8 (684.4 to 836.1)	501.5 (445.1 to 565.2)	213.8 (178.7 to 251.3)	38.2 (17.8 to 71.3)	1.3 (1.3 to 1.3)
Libya	2014	290.4 (259.7 to 326.0)	190.4 (165.2 to 219.4)	85.2 (70.0 to 102.6)	14.7 (6.9 to 28.1)	0.1 (0.1 to 0.1)	528.8 (472.9 to 593.7)	346.7 (300.9 to 399.6)	155.2 (127.5 to 186.9)	26.7 (12.5 to 51.1)	0.2 (0.2 to 0.2)
Libya	2015	268.5 (236.6 to 305.4)	175.9 (149.9 to 205.3)	79.0 (62.6 to 97.3)	13.3 (6.3 to 25.9)	0.3 (0.3 to 0.3)	489.0 (430.9 to 556.2)	320.3 (272.9 to 373.9)	143.8 (114.0 to 177.2)	24.3 (11.4 to 47.1)	0.6 (0.6 to 0.6)
Libya	2016	256.7 (221.6 to 293.8)	168.9 (139.8 to 202.9)	74.8 (57.6 to 93.5)	12.4 (5.9 to 24.4)	0.6 (0.6 to 0.6)	467.5 (403.7 to 535.0)	307.5 (254.7 to 369.6)	136.2 (104.9 to 170.3)	22.6 (10.7 to 44.4)	1.2 (1.2 to 1.2)
Libya	2017	247.3 (213.7 to 285.3)	151.2 (125.4 to 181.7)	80.3 (61.4 to 101.6)	14.6 (7.0 to 29.1)	1.2 (1.2 to 1.2)	450.3 (389.2 to 519.5)	275.4 (228.5 to 331.0)	146.2 (111.8 to 185.0)	26.7 (12.7 to 52.9)	2.1 (2.1 to 2.1)
Libya	2018	246.7 (212.6 to 284.6)	151.3 (125.8 to 182.3)	79.0 (60.1 to 99.4)	15.4 (7.3 to 30.6)	1.0 (1.0 to 1.0)	449.4 (387.1 to 518.2)	275.6 (229.1 to 332.1)	143.9 (109.4 to 181.1)	28.1 (13.3 to 55.7)	1.9 (1.8 to 1.9)
Libya	2019	241.1 (208.2 to 277.6)	146.1 (121.2 to 176.0)	78.6 (59.8 to 98.6)	15.4 (7.3 to 30.5)	1.0 (1.0 to 1.1)	439.0 (379.2 to 505.5)	266.1 (220.7 to 320.6)	143.1 (108.9 to 179.6)	28.0 (13.4 to 55.6)	1.9 (1.7 to 2.0)
Libya	2020	234.9 (203.5 to 271.8)	142.0 (117.8 to 170.5)	76.4 (58.4 to 96.0)	15.4 (7.3 to 30.7)	1.0 (0.9 to 1.1)	427.7 (370.6 to 495.0)	258.5 (214.5 to 310.6)	139.2 (106.3 to 174.8)	28.1 (13.4 to 55.9)	1.9 (1.7 to 2.1)
Libya	2021	229.9 (197.0 to 264.5)	138.8 (115.3 to 167.0)	74.5 (56.8 to 93.7)	15.5 (7.4 to 30.9)	1.1 (0.9 to 1.2)	418.6 (358.7 to 481.7)	252.8 (210.0 to 304.1)	135.7 (103.5 to 170.6)	28.2 (13.4 to 56.3)	1.9 (1.7 to 2.1)
Libya	2022	225.5 (193.9 to 259.9)	136.2 (112.4 to 163.2)	72.6 (55.5 to 91.1)	15.6 (7.4 to 31.1)	1.1 (1.0 to 1.2)	410.7 (353.1 to 473.3)	248.1 (204.8 to 297.2)	132.3 (101.1 to 166.0)	28.3 (13.5 to 56.7)	2.0 (1.7 to 2.2)
Libya	2023	225.5 (192.7 to 261.8)	137.5 (113.8 to 165.0)	71.1 (54.3 to 89.9)	15.8 (7.5 to 31.5)	1.1 (1.0 to 1.3)	410.7 (350.9 to 476.9)	250.4 (207.2 to 300.6)	129.6 (98.9 to 163.7)	28.7 (13.7 to 57.3)	2.0 (1.7 to 2.3)
Libya	2024	225.3 (193.8 to 258.5)	138.6 (114.6 to 166.7)	69.6 (52.7 to 88.3)	16.0 (7.6 to 31.7)	1.1 (1.0 to 1.3)	410.4 (353.0 to 470.7)	252.5 (208.6 to 303.6)	126.8 (96.0 to 160.8)	29.1 (13.9 to 57.7)	2.0 (1.8 to 2.4)
Libya	2025	229.0 (197.1 to 266.0)	142.6 (118.0 to 171.4)	68.8 (52.7 to 87.2)	16.4 (7.9 to 32.6)	1.1 (1.0 to 1.3)	417.0 (358.9 to 484.5)	259.6 (214.9 to 312.1)	125.3 (96.0 to 158.8)	29.9 (14.3 to 59.3)	2.1 (1.8 to 2.4)
Libya	2026	233.6 (200.2 to 270.8)	147.4 (122.3 to 177.2)	68.1 (52.1 to 86.7)	17.0 (8.1 to 33.6)	1.2 (1.0 to 1.4)	425.5 (364.5 to 493.2)	268.5 (222.7 to 322.8)	124.0 (94.9 to 157.9)	30.9 (14.8 to 61.2)	2.1 (1.8 to 2.5)
Libya	2027	239.0 (203.9 to 279.8)	152.8 (126.7 to 183.9)	67.5 (51.6 to 85.7)	17.6 (8.4 to 34.8)	1.2 (1.0 to 1.4)	435.2 (371.3 to 509.5)	278.2 (230.7 to 335.0)	122.8 (94.0 to 156.1)	32.0 (15.3 to 63.5)	2.1 (1.8 to 2.5)
Libya	2028	244.8 (207.8 to 284.7)	158.5 (131.1 to 191.2)	67.0 (51.0 to 85.6)	18.2 (8.7 to 36.1)	1.2 (1.0 to 1.4)	445.8 (378.5 to 518.5)	288.6 (238.7 to 348.2)	122.0 (92.8 to 155.8)	33.1 (15.9 to 65.8)	2.1 (1.8 to 2.6)
Libya	2029	251.5 (213.4 to 292.0)	164.8 (136.2 to 199.1)	66.7 (50.8 to 85.6)	18.8 (9.1 to 37.4)	1.2 (1.0 to 1.5)	458.1 (388.6 to 531.8)	300.2 (248.1 to 362.7)	121.4 (92.5 to 155.9)	34.3 (16.5 to 68.1)	2.2 (1.8 to 2.7)

		2018 US Dollars per capita					2018 PPP per capita				
Country	Year	Health spending	Government health spending	Out-of-pocket health spending	Prepaid private health spending	Development assistance for health	Health spending	Government health spending	Out-of-pocket health spending	Prepaid private health spending	Development assistance for health
Libya	2030	259.1 (221.0 to 301.4)	171.9 (141.7 to 208.0)	66.5 (50.2 to 85.9)	19.5 (9.4 to 38.7)	1.2 (1.0 to 1.5)	471.9 (402.6 to 548.9)	313.0 (258.1 to 378.8)	121.1 (91.5 to 156.4)	35.5 (17.1 to 70.4)	2.2 (1.8 to 2.7)
Libya	2031	267.1 (226.0 to 313.2)	179.2 (147.1 to 217.0)	66.5 (49.9 to 86.3)	20.2 (9.7 to 40.0)	1.2 (1.0 to 1.5)	486.5 (411.5 to 570.5)	326.4 (267.9 to 395.1)	121.1 (90.8 to 157.3)	36.8 (17.7 to 72.9)	2.2 (1.8 to 2.8)
Libya	2032	275.9 (234.7 to 323.2)	187.2 (152.3 to 226.7)	66.6 (49.5 to 86.7)	21.0 (10.1 to 41.4)	1.2 (1.0 to 1.6)	502.6 (427.4 to 588.6)	340.9 (277.4 to 413.0)	121.2 (90.2 to 157.9)	38.2 (18.4 to 75.4)	2.2 (1.8 to 2.9)
Libya	2033	285.5 (239.6 to 334.5)	195.8 (157.8 to 237.6)	66.8 (49.1 to 87.1)	21.7 (10.5 to 42.9)	1.2 (0.9 to 1.6)	520.0 (436.3 to 609.1)	356.6 (287.4 to 432.7)	121.7 (89.3 to 158.7)	39.6 (19.1 to 78.1)	2.2 (1.6 to 2.9)
Libya	2034	295.5 (248.4 to 346.9)	204.6 (163.5 to 249.0)	67.1 (48.9 to 87.9)	22.5 (10.9 to 44.4)	1.2 (0.0 to 1.6)	538.2 (452.3 to 631.8)	372.7 (297.7 to 453.4)	122.3 (89.0 to 160.1)	41.0 (19.8 to 80.8)	2.3 (0.0 to 3.0)
Libya	2035	305.8 (255.2 to 367.4)	213.7 (170.2 to 261.6)	67.6 (48.8 to 89.3)	23.3 (11.2 to 45.8)	1.2 (0.0 to 1.7)	556.9 (464.8 to 669.1)	389.2 (309.9 to 476.5)	123.0 (88.8 to 162.6)	42.4 (20.5 to 83.4)	2.3 (0.0 to 3.1)
Libya	2036	316.3 (266.3 to 375.0)	222.9 (176.3 to 273.7)	68.1 (48.8 to 90.2)	24.1 (11.6 to 47.3)	1.2 (0.0 to 1.8)	576.0 (485.0 to 682.9)	405.9 (321.0 to 498.5)	124.0 (88.9 to 164.3)	43.9 (21.2 to 86.2)	2.3 (0.0 to 3.2)
Libya	2037	327.2 (270.0 to 392.5)	232.4 (182.9 to 287.8)	68.7 (48.8 to 91.2)	24.9 (12.0 to 48.8)	1.2 (0.0 to 1.8)	595.9 (491.8 to 714.7)	423.2 (333.1 to 524.1)	125.1 (88.9 to 166.0)	45.4 (21.9 to 88.9)	2.2 (0.0 to 3.2)
Libya	2038	338.6 (282.6 to 402.7)	242.2 (189.0 to 301.4)	69.4 (49.2 to 92.2)	25.8 (12.4 to 50.4)	1.2 (0.0 to 1.8)	616.6 (514.6 to 733.4)	441.0 (344.3 to 548.9)	126.5 (89.6 to 167.9)	46.9 (22.6 to 91.8)	2.2 (0.0 to 3.3)
Libya	2039	350.1 (285.5 to 420.1)	252.0 (196.0 to 316.2)	70.3 (49.9 to 93.8)	26.6 (12.8 to 51.9)	1.2 (0.0 to 1.9)	637.5 (520.0 to 765.1)	458.9 (356.9 to 576.0)	128.0 (90.8 to 170.8)	48.4 (23.4 to 94.6)	2.2 (0.0 to 3.4)
Libya	2040	361.8 (298.9 to 434.3)	261.9 (202.6 to 331.5)	71.2 (50.6 to 95.8)	27.5 (13.3 to 53.5)	1.2 (0.0 to 1.9)	658.9 (544.3 to 790.9)	477.0 (368.9 to 603.6)	129.7 (92.1 to 174.4)	50.0 (24.2 to 97.5)	2.2 (0.0 to 3.5)
Libya	2041	373.7 (302.4 to 453.1)	272.0 (208.9 to 346.6)	72.3 (51.2 to 97.5)	28.3 (13.7 to 55.1)	1.2 (0.0 to 2.0)	680.6 (550.8 to 825.3)	495.3 (380.5 to 631.3)	131.6 (93.3 to 177.5)	51.6 (24.9 to 100.4)	2.2 (0.0 to 3.7)
Libya	2042	385.9 (316.7 to 466.3)	282.1 (215.5 to 362.5)	73.4 (52.0 to 99.8)	29.2 (14.1 to 56.7)	1.2 (0.0 to 2.1)	702.9 (576.8 to 849.1)	513.8 (392.5 to 660.3)	133.7 (94.6 to 181.8)	53.2 (25.7 to 103.3)	2.2 (0.0 to 3.8)
Libya	2043	398.7 (321.2 to 491.7)	292.7 (222.4 to 378.4)	74.7 (52.8 to 102.1)	30.1 (14.6 to 58.4)	1.2 (0.0 to 2.2)	726.1 (585.0 to 895.4)	533.0 (405.1 to 689.1)	136.1 (96.1 to 185.9)	54.8 (26.5 to 106.3)	2.2 (0.0 to 3.9)
Libya	2044	411.7 (333.6 to 503.5)	303.3 (229.0 to 393.4)	76.2 (53.6 to 104.2)	31.0 (15.0 to 60.1)	1.2 (0.0 to 2.2)	749.8 (607.5 to 917.0)	552.3 (417.0 to 716.4)	138.8 (97.6 to 189.7)	56.5 (27.4 to 109.4)	2.2 (0.0 to 4.0)
Libya	2045	424.2 (336.9 to 526.3)	313.3 (235.2 to 407.2)	77.7 (54.5 to 106.7)	31.9 (15.5 to 61.7)	1.2 (0.0 to 2.3)	772.6 (613.6 to 958.5)	570.6 (428.3 to 741.6)	141.6 (99.3 to 194.3)	58.2 (28.2 to 112.4)	2.2 (0.0 to 4.1)
Libya	2046	436.4 (352.0 to 535.9)	322.9 (241.7 to 422.2)	79.4 (55.5 to 109.0)	32.8 (15.9 to 63.4)	1.2 (0.0 to 2.3)	794.7 (641.1 to 975.9)	588.1 (440.2 to 768.9)	144.6 (101.1 to 198.6)	59.8 (29.0 to 115.4)	2.2 (0.0 to 4.2)
Libya	2047	449.0 (355.9 to 559.4)	332.7 (247.9 to 438.3)	81.3 (56.7 to 111.6)	33.8 (16.4 to 65.2)	1.2 (0.0 to 2.4)	817.7 (648.2 to 1018.7)	606.0 (451.4 to 798.2)	148.0 (103.3 to 203.3)	61.6 (29.8 to 118.8)	2.2 (0.0 to 4.4)
Libya	2048	460.8 (367.7 to 572.8)	341.8 (253.5 to 452.0)	83.1 (57.8 to 114.1)	34.7 (16.8 to 67.1)	1.2 (0.0 to 2.5)	839.3 (669.6 to 1043.2)	622.6 (461.7 to 823.1)	151.3 (105.4 to 207.8)	63.2 (30.6 to 122.2)	2.2 (0.0 to 4.5)
Libya	2049	472.8 (371.4 to 597.1)	351.0 (260.1 to 465.0)	84.9 (59.0 to 116.6)	35.7 (17.3 to 68.8)	1.2 (0.0 to 2.6)	861.0 (676.4 to 1087.4)	639.3 (473.7 to 846.9)	154.6 (107.5 to 212.3)	64.9 (31.5 to 125.2)	2.2 (0.0 to 4.7)
Libya	2050	485.0 (387.0 to 603.6)	360.5 (267.1 to 479.3)	86.8 (60.2 to 119.2)	36.6 (17.8 to 70.4)	1.2 (0.0 to 2.6)	883.4 (704.8 to 1099.2)	656.5 (486.4 to 872.9)	158.0 (109.7 to 217.1)	66.7 (32.3 to 128.3)	2.1 (0.0 to 4.7)

		2018 US Dollars per capita					2018 PPP per capita				
Country	Year	Health spending	Government health spending	Out-of-pocket health spending	Prepaid private health spending	Development assistance for health	Health spending	Government health spending	Out-of-pocket health spending	Prepaid private health spending	Development assistance for health
Lithuania	1995	291.7 (260.4 to 323.8)	200.8 (175.1 to 227.9)	75.7 (63.7 to 90.6)	13.8 (7.2 to 23.8)	1.3 (1.3 to 1.3)	531.8 (474.7 to 590.4)	366.2 (319.3 to 415.4)	138.1 (116.2 to 165.1)	25.2 (13.2 to 43.4)	2.3 (2.3 to 2.3)
Lithuania	1996	319.2 (289.3 to 348.3)	219.8 (195.2 to 245.3)	84.3 (71.9 to 98.3)	15.1 (8.3 to 25.4)	0.0 (0.0 to 0.0)	582.0 (527.5 to 635.0)	400.7 (355.9 to 447.2)	153.6 (131.1 to 179.3)	27.6 (15.1 to 46.2)	0.0 (0.0 to 0.0)
Lithuania	1997	360.7 (331.4 to 391.2)	251.7 (227.9 to 276.3)	92.6 (80.7 to 105.1)	16.4 (9.1 to 26.6)	0.0 (0.0 to 0.0)	657.7 (604.3 to 713.2)	458.9 (415.4 to 503.8)	168.9 (147.2 to 191.6)	29.9 (16.6 to 48.5)	0.0 (0.0 to 0.0)
Lithuania	1998	406.8 (378.0 to 435.1)	286.1 (262.5 to 311.0)	102.2 (90.0 to 115.1)	17.9 (10.2 to 28.5)	0.6 (0.6 to 0.6)	741.7 (689.1 to 793.4)	521.7 (478.7 to 567.1)	186.4 (164.0 to 209.9)	32.6 (18.5 to 51.9)	1.0 (1.0 to 1.0)
Lithuania	1999	424.1 (394.6 to 452.3)	296.2 (273.5 to 319.8)	109.6 (97.4 to 122.6)	18.2 (10.3 to 28.3)	0.1 (0.1 to 0.1)	773.3 (719.5 to 824.6)	540.1 (498.7 to 583.1)	199.8 (177.6 to 223.6)	33.2 (18.7 to 51.6)	0.1 (0.1 to 0.1)
Lithuania	2000	452.0 (422.6 to 482.8)	313.7 (290.3 to 339.0)	119.5 (106.2 to 133.8)	18.3 (10.5 to 28.5)	0.5 (0.5 to 0.5)	824.1 (770.6 to 880.3)	572.0 (529.3 to 618.2)	217.8 (193.6 to 243.9)	33.4 (19.2 to 52.0)	0.9 (0.9 to 0.9)
Lithuania	2001	483.5 (454.0 to 515.1)	339.4 (316.7 to 366.4)	128.8 (114.1 to 144.2)	15.0 (8.8 to 23.2)	0.2 (0.2 to 0.2)	881.6 (827.8 to 939.1)	618.8 (577.5 to 668.0)	234.9 (208.0 to 262.9)	27.4 (16.0 to 42.2)	0.4 (0.4 to 0.4)
Lithuania	2002	521.8 (491.6 to 555.8)	367.2 (341.7 to 395.4)	139.2 (123.9 to 155.8)	13.9 (8.4 to 21.3)	1.4 (1.4 to 1.4)	951.4 (896.2 to 1013.3)	669.5 (623.1 to 720.9)	253.9 (225.9 to 284.0)	25.4 (15.3 to 38.9)	2.6 (2.6 to 2.6)
Lithuania	2003	568.0 (536.5 to 602.9)	394.7 (368.2 to 423.2)	155.9 (140.4 to 173.3)	15.9 (9.9 to 24.2)	1.5 (1.5 to 1.5)	1035.5 (978.1 to 1099.3)	719.6 (671.2 to 771.6)	284.2 (256.0 to 316.0)	29.0 (18.0 to 44.0)	2.7 (2.7 to 2.7)
Lithuania	2004	593.9 (561.5 to 628.7)	390.3 (365.2 to 417.9)	178.4 (162.6 to 196.6)	22.9 (14.2 to 34.9)	2.4 (2.4 to 2.4)	1082.9 (1023.7 to 1146.2)	711.6 (665.8 to 761.9)	325.2 (296.4 to 358.5)	41.7 (26.0 to 63.6)	4.4 (4.4 to 4.4)
Lithuania	2005	644.4 (609.6 to 681.9)	413.0 (387.4 to 439.8)	200.5 (183.1 to 221.2)	30.2 (19.0 to 45.1)	0.7 (0.7 to 0.7)	1174.9 (1111.4 to 1243.2)	753.0 (706.3 to 801.8)	365.5 (333.9 to 403.4)	55.1 (34.7 to 82.3)	1.3 (1.3 to 1.3)
Lithuania	2006	717.3 (680.4 to 754.4)	463.8 (435.4 to 489.8)	220.2 (201.0 to 241.8)	32.7 (20.2 to 48.7)	0.7 (0.7 to 0.7)	1307.8 (1240.6 to 1375.4)	845.5 (793.8 to 893.0)	401.4 (366.5 to 440.9)	59.6 (36.9 to 88.7)	1.2 (1.2 to 1.2)
Lithuania	2007	822.9 (781.4 to 862.8)	553.3 (518.6 to 583.6)	239.1 (218.2 to 262.1)	30.5 (19.0 to 46.2)	0.0 (0.0 to 0.0)	1500.4 (1424.7 to 1573.2)	1008.7 (945.5 to 1064.0)	436.0 (397.9 to 477.9)	55.6 (34.6 to 84.3)	0.0 (0.0 to 0.0)
Lithuania	2008	909.4 (867.8 to 951.1)	634.0 (598.1 to 669.6)	253.7 (232.8 to 277.0)	20.3 (12.7 to 30.9)	1.4 (1.4 to 1.4)	1658.1 (1582.3 to 1734.0)	1155.9 (1090.5 to 1220.8)	462.6 (424.4 to 505.0)	37.1 (23.2 to 56.3)	2.5 (2.5 to 2.5)
Lithuania	2009	868.2 (829.5 to 907.6)	604.3 (573.6 to 636.4)	244.5 (225.2 to 266.2)	15.6 (9.5 to 24.2)	3.8 (3.8 to 3.8)	1582.9 (1512.3 to 1654.8)	1101.7 (1045.8 to 1160.2)	445.8 (410.5 to 485.3)	28.4 (17.3 to 44.1)	6.9 (6.9 to 6.9)
Lithuania	2010	885.7 (846.9 to 925.9)	618.3 (587.7 to 649.0)	250.5 (230.2 to 270.8)	14.1 (8.6 to 21.9)	2.8 (2.8 to 2.8)	1614.8 (1544.1 to 1688.2)	1127.2 (1071.5 to 1183.4)	456.8 (419.8 to 493.8)	25.7 (15.7 to 39.9)	5.1 (5.1 to 5.1)
Lithuania	2011	909.9 (872.9 to 950.1)	630.8 (601.8 to 662.1)	264.9 (246.2 to 286.2)	14.1 (8.7 to 21.9)	0.2 (0.2 to 0.2)	1658.9 (1591.4 to 1732.3)	1150.1 (1097.2 to 1207.2)	482.9 (448.9 to 521.7)	25.7 (15.8 to 39.9)	0.3 (0.3 to 0.3)
Lithuania	2012	926.8 (891.3 to 966.2)	627.8 (598.0 to 658.1)	284.6 (265.6 to 305.3)	14.4 (8.8 to 22.4)	0.0 (0.0 to 0.0)	1689.7 (1625.0 to 1761.5)	1144.5 (1090.2 to 1199.9)	519.0 (484.2 to 556.6)	26.2 (16.1 to 40.9)	0.0 (0.0 to 0.0)
Lithuania	2013	954.0 (919.3 to 990.6)	636.3 (607.5 to 666.6)	302.9 (284.7 to 323.5)	14.8 (9.0 to 23.4)	0.0 (0.0 to 0.0)	1739.4 (1676.0 to 1806.1)	1160.1 (1107.7 to 1215.4)	552.3 (519.2 to 589.8)	27.0 (16.5 to 42.6)	0.0 (0.0 to 0.0)
Lithuania	2014	1003.3 (968.5 to 1040.9)	665.9 (637.5 to 695.7)	321.3 (302.5 to 342.5)	16.1 (9.9 to 25.1)	0.0 (0.0 to 0.0)	1829.2 (1765.8 to 1897.8)	1214.0 (1162.3 to 1268.5)	585.8 (551.6 to 624.5)	29.4 (18.0 to 45.8)	0.0 (0.0 to 0.0)
Lithuania	2015	1062.4 (1023.6 to 1102.9)	701.5 (670.9 to 736.0)	343.7 (323.3 to 366.9)	17.2 (10.2 to 27.3)	0.0 (0.0 to 0.0)	1937.1 (1866.3 to 2010.8)	1279.1 (1223.2 to 1341.9)	626.7 (589.5 to 668.9)	31.3 (18.7 to 49.8)	0.0 (0.0 to 0.0)

		2018 US Dollars per capita					2018 PPP per capita				
Country	Year	Health spending	Government health spending	Out-of-pocket health spending	Prepaid private health spending	Development assistance for health	Health spending	Government health spending	Out-of-pocket health spending	Prepaid private health spending	Development assistance for health
Lithuania	2016	1120.9 (1069.0 to 1175.9)	741.0 (699.2 to 788.3)	364.2 (336.5 to 394.7)	15.8 (9.2 to 25.2)	0.0 (0.0 to 0.0)	2043.7 (1949.1 to 2143.9)	1350.9 (1274.8 to 1437.3)	664.0 (613.4 to 719.7)	28.8 (16.8 to 45.9)	0.0 (0.0 to 0.0)
Lithuania	2017	1167.0 (1113.5 to 1224.9)	768.5 (725.0 to 818.2)	381.6 (352.7 to 412.6)	16.9 (9.9 to 27.0)	0.0 (0.0 to 0.0)	2127.7 (2030.2 to 2233.2)	1401.2 (1321.8 to 1491.7)	695.7 (643.0 to 752.3)	30.9 (18.0 to 49.2)	0.0 (0.0 to 0.0)
Lithuania	2018	1201.6 (1145.1 to 1263.8)	787.9 (742.2 to 839.6)	396.4 (365.7 to 430.3)	17.4 (10.2 to 27.6)	0.0 (0.0 to 0.0)	2190.8 (2087.8 to 2304.3)	1436.5 (1353.2 to 1530.8)	722.7 (666.8 to 784.5)	31.7 (18.6 to 50.4)	0.0 (0.0 to 0.0)
Lithuania	2019	1233.9 (1177.3 to 1297.5)	805.2 (758.4 to 859.9)	410.8 (378.8 to 446.1)	17.9 (10.5 to 28.4)	0.0 (0.0 to 0.0)	2249.6 (2146.6 to 2365.7)	1468.0 (1382.8 to 1567.8)	749.0 (690.6 to 813.3)	32.6 (19.1 to 51.8)	0.0 (0.0 to 0.0)
Lithuania	2020	1266.4 (1208.6 to 1331.3)	822.5 (773.3 to 877.3)	425.6 (391.4 to 463.3)	18.3 (10.7 to 29.0)	0.0 (0.0 to 0.0)	2309.0 (2203.6 to 2427.2)	1499.7 (1409.8 to 1599.5)	775.9 (713.6 to 844.7)	33.4 (19.6 to 52.9)	0.0 (0.0 to 0.0)
Lithuania	2021	1300.8 (1235.8 to 1369.7)	841.4 (790.6 to 898.3)	440.7 (403.7 to 481.1)	18.7 (11.0 to 29.8)	0.0 (0.0 to 0.0)	2371.7 (2253.2 to 2497.2)	1534.1 (1441.5 to 1637.8)	803.5 (736.1 to 877.1)	34.2 (20.1 to 54.3)	0.0 (0.0 to 0.0)
Lithuania	2022	1334.2 (1265.6 to 1406.7)	859.1 (805.0 to 918.6)	455.9 (417.0 to 496.5)	19.1 (11.3 to 30.4)	0.0 (0.0 to 0.0)	2432.5 (2307.5 to 2564.8)	1566.4 (1467.7 to 1674.8)	831.2 (760.2 to 905.2)	34.9 (20.5 to 55.4)	0.0 (0.0 to 0.0)
Lithuania	2023	1367.2 (1298.7 to 1445.9)	876.1 (820.2 to 937.4)	471.5 (430.7 to 514.7)	19.5 (11.4 to 31.1)	0.0 (0.0 to 0.0)	2492.7 (2367.9 to 2636.2)	1597.4 (1495.5 to 1709.1)	859.7 (785.2 to 938.5)	35.6 (20.9 to 56.7)	0.0 (0.0 to 0.0)
Lithuania	2024	1400.3 (1328.0 to 1484.7)	893.2 (835.4 to 956.6)	487.1 (444.0 to 532.8)	19.9 (11.7 to 31.7)	0.0 (0.0 to 0.0)	2553.0 (2421.3 to 2707.0)	1628.6 (1523.2 to 1744.0)	888.1 (809.6 to 971.4)	36.3 (21.3 to 57.8)	0.0 (0.0 to 0.0)
Lithuania	2025	1434.5 (1359.8 to 1519.2)	911.0 (849.7 to 975.7)	503.2 (457.2 to 549.7)	20.3 (11.9 to 32.3)	0.0 (0.0 to 0.0)	2615.4 (2479.2 to 2769.8)	1660.9 (1549.1 to 1778.9)	917.5 (833.6 to 1002.2)	37.0 (21.7 to 58.9)	0.0 (0.0 to 0.0)
Lithuania	2026	1469.5 (1386.7 to 1559.0)	929.2 (865.2 to 997.6)	519.7 (472.4 to 569.7)	20.7 (12.1 to 33.0)	0.0 (0.0 to 0.0)	2679.3 (2528.3 to 2842.4)	1694.1 (1577.5 to 1818.8)	947.6 (861.3 to 1038.7)	37.7 (22.1 to 60.2)	0.0 (0.0 to 0.0)
Lithuania	2027	1505.9 (1417.0 to 1593.5)	948.0 (883.8 to 1019.7)	536.9 (484.8 to 591.4)	21.0 (12.3 to 33.6)	0.0 (0.0 to 0.0)	2745.6 (2583.5 to 2905.3)	1728.4 (1611.4 to 1859.2)	978.9 (884.0 to 1078.4)	38.4 (22.4 to 61.3)	0.0 (0.0 to 0.0)
Lithuania	2028	1544.3 (1447.2 to 1640.1)	968.4 (900.8 to 1043.4)	554.5 (494.8 to 617.4)	21.5 (12.5 to 34.3)	0.0 (0.0 to 0.0)	2815.6 (2638.7 to 2990.3)	1765.6 (1642.5 to 1902.4)	1010.9 (902.2 to 1125.6)	39.1 (22.9 to 62.5)	0.0 (0.0 to 0.0)
Lithuania	2029	1584.0 (1484.5 to 1683.9)	989.6 (918.0 to 1070.6)	572.4 (507.2 to 644.2)	21.9 (12.8 to 35.0)	0.0 (0.0 to 0.0)	2888.0 (2706.6 to 3070.2)	1804.3 (1673.8 to 1951.9)	1043.7 (924.8 to 1174.5)	40.0 (23.4 to 63.8)	0.0 (0.0 to 0.0)
Lithuania	2030	1623.7 (1516.0 to 1736.8)	1010.7 (935.5 to 1091.7)	590.6 (519.7 to 670.0)	22.4 (13.1 to 35.7)	0.0 (0.0 to 0.0)	2960.4 (2764.0 to 3166.5)	1842.8 (1705.6 to 1990.5)	1076.8 (947.5 to 1221.6)	40.9 (23.9 to 65.1)	0.0 (0.0 to 0.0)
Lithuania	2031	1664.5 (1554.9 to 1787.6)	1032.7 (952.9 to 1119.3)	608.9 (531.2 to 698.6)	22.9 (13.4 to 36.7)	0.0 (0.0 to 0.0)	3034.8 (2835.0 to 3259.3)	1882.9 (1737.3 to 2040.7)	1110.1 (968.4 to 1273.7)	41.8 (24.4 to 66.8)	0.0 (0.0 to 0.0)
Lithuania	2032	1706.0 (1587.0 to 1837.7)	1055.1 (969.3 to 1147.6)	627.4 (543.3 to 725.6)	23.5 (13.7 to 37.6)	0.0 (0.0 to 0.0)	3110.4 (2893.5 to 3350.6)	1923.7 (1767.2 to 2092.3)	1143.9 (990.5 to 1323.0)	42.8 (25.0 to 68.6)	0.0 (0.0 to 0.0)
Lithuania	2033	1747.2 (1618.0 to 1885.4)	1077.3 (987.3 to 1173.5)	645.9 (553.7 to 752.6)	24.1 (14.1 to 38.5)	0.0 (0.0 to 0.0)	3185.6 (2950.0 to 3437.5)	1964.2 (1800.2 to 2139.6)	1177.6 (1009.5 to 1372.1)	43.9 (25.6 to 70.2)	0.0 (0.0 to 0.0)
Lithuania	2034	1789.2 (1652.2 to 1943.4)	1100.2 (1005.4 to 1201.0)	664.4 (564.5 to 780.3)	24.6 (14.4 to 39.4)	0.0 (0.0 to 0.0)	3262.1 (3012.4 to 3543.4)	2005.9 (1833.1 to 2189.8)	1211.3 (1029.2 to 1422.7)	44.9 (26.3 to 71.9)	0.0 (0.0 to 0.0)
Lithuania	2035	1831.4 (1680.0 to 1989.2)	1123.5 (1024.0 to 1230.0)	682.7 (575.2 to 808.0)	25.3 (14.8 to 40.3)	0.0 (0.0 to 0.0)	3339.1 (3063.0 to 3626.8)	2048.3 (1867.0 to 2242.6)	1244.8 (1048.7 to 1473.3)	46.0 (26.9 to 73.5)	0.0 (0.0 to 0.0)
Lithuania	2036	1875.5 (1723.3 to 2056.4)	1148.4 (1043.3 to 1260.2)	701.2 (586.4 to 835.8)	25.9 (15.1 to 41.4)	0.0 (0.0 to 0.0)	3419.5 (3142.0 to 3749.2)	2093.8 (1902.3 to 2297.6)	1278.5 (1069.1 to 1523.9)	47.2 (27.5 to 75.5)	0.0 (0.0 to 0.0)

Country	Year	2018 US Dollars per capita					2018 PPP per capita				
		Health spending	Government health spending	Out-of-pocket health spending	Prepaid private health spending	Development assistance for health	Health spending	Government health spending	Out-of-pocket health spending	Prepaid private health spending	Development assistance for health
Lithuania	2037	1919.6 (1756.6 to 2119.0)	1173.5 (1064.6 to 1293.4)	719.5 (597.4 to 863.3)	26.5 (15.5 to 42.6)	0.0 (0.0 to 0.0)	3499.8 (3202.7 to 3863.5)	2139.5 (1941.0 to 2358.1)	1311.9 (1089.2 to 1574.1)	48.4 (28.2 to 77.6)	0.0 (0.0 to 0.0)
Lithuania	2038	1963.1 (1793.0 to 2167.4)	1198.4 (1082.3 to 1320.0)	737.5 (609.9 to 889.9)	27.2 (15.9 to 43.7)	0.0 (0.0 to 0.0)	3579.3 (3269.1 to 3951.8)	2185.0 (1973.4 to 2406.7)	1344.6 (1112.0 to 1622.6)	49.6 (28.9 to 79.7)	0.0 (0.0 to 0.0)
Lithuania	2039	2007.2 (1836.5 to 2223.1)	1223.9 (1103.8 to 1351.6)	755.4 (623.4 to 916.0)	27.9 (16.3 to 44.9)	0.0 (0.0 to 0.0)	3659.6 (3348.4 to 4053.2)	2231.5 (2012.5 to 2464.2)	1377.2 (1136.6 to 1670.0)	50.8 (29.7 to 81.9)	0.0 (0.0 to 0.0)
Lithuania	2040	2052.1 (1861.2 to 2255.5)	1250.3 (1124.9 to 1381.5)	773.2 (634.9 to 943.6)	28.6 (16.7 to 46.1)	0.0 (0.0 to 0.0)	3741.6 (3393.4 to 4112.4)	2279.6 (2051.0 to 2518.9)	1409.8 (1157.5 to 1720.5)	52.2 (30.5 to 84.1)	0.0 (0.0 to 0.0)
Lithuania	2041	2099.6 (1901.7 to 2328.0)	1278.8 (1145.6 to 1413.4)	791.4 (646.8 to 969.6)	29.4 (17.2 to 47.4)	0.0 (0.0 to 0.0)	3828.1 (3467.2 to 4244.4)	2331.6 (2088.7 to 2576.9)	1442.9 (1179.2 to 1767.8)	53.7 (31.3 to 86.5)	0.0 (0.0 to 0.0)
Lithuania	2042	2145.9 (1938.5 to 2362.6)	1306.7 (1168.7 to 1449.7)	809.0 (660.0 to 997.1)	30.3 (17.7 to 48.8)	0.0 (0.0 to 0.0)	3912.6 (3534.3 to 4307.6)	2382.4 (2130.8 to 2643.2)	1475.1 (1203.3 to 1818.0)	55.2 (32.2 to 88.9)	0.0 (0.0 to 0.0)
Lithuania	2043	2193.9 (1983.8 to 2442.4)	1336.1 (1190.9 to 1486.3)	826.7 (673.2 to 1021.8)	31.1 (18.2 to 50.2)	0.0 (0.0 to 0.0)	4000.0 (3616.9 to 4453.1)	2436.0 (2171.2 to 2709.8)	1507.2 (1227.3 to 1863.1)	56.8 (33.1 to 91.6)	0.0 (0.0 to 0.0)
Lithuania	2044	2242.5 (2022.2 to 2511.5)	1366.4 (1211.7 to 1529.0)	844.1 (684.1 to 1046.2)	32.0 (18.7 to 51.7)	0.0 (0.0 to 0.0)	4088.6 (3687.0 to 4579.0)	2491.2 (2209.2 to 2787.8)	1538.9 (1247.3 to 1907.5)	58.4 (34.1 to 94.3)	0.0 (0.0 to 0.0)
Lithuania	2045	2290.6 (2057.5 to 2558.1)	1396.7 (1237.0 to 1567.6)	860.9 (695.1 to 1071.3)	33.0 (19.2 to 53.3)	0.0 (0.0 to 0.0)	4176.3 (3751.3 to 4664.1)	2546.5 (2255.4 to 2858.2)	1569.7 (1267.4 to 1953.2)	60.1 (35.0 to 97.3)	0.0 (0.0 to 0.0)
Lithuania	2046	2338.5 (2099.1 to 2621.9)	1427.4 (1261.3 to 1604.6)	877.2 (707.5 to 1095.1)	33.9 (19.7 to 55.0)	0.0 (0.0 to 0.0)	4263.7 (3827.2 to 4780.4)	2602.5 (2299.7 to 2925.6)	1599.3 (1289.9 to 1996.6)	61.9 (35.9 to 100.3)	0.0 (0.0 to 0.0)
Lithuania	2047	2387.0 (2145.0 to 2684.9)	1459.0 (1286.0 to 1645.9)	893.0 (720.0 to 1117.8)	34.9 (20.2 to 56.8)	0.0 (0.0 to 0.0)	4352.0 (3910.8 to 4895.2)	2660.1 (2344.6 to 3000.9)	1628.2 (1312.8 to 2038.0)	63.7 (36.8 to 103.5)	0.0 (0.0 to 0.0)
Lithuania	2048	2434.9 (2179.5 to 2733.5)	1490.2 (1313.3 to 1686.1)	908.8 (732.6 to 1140.6)	35.9 (20.8 to 58.3)	0.0 (0.0 to 0.0)	4439.5 (3973.8 to 4983.8)	2716.9 (2394.4 to 3074.2)	1657.0 (1335.6 to 2079.5)	65.5 (37.8 to 106.4)	0.0 (0.0 to 0.0)
Lithuania	2049	2480.4 (2217.2 to 2780.3)	1519.4 (1337.9 to 1725.4)	924.1 (744.7 to 1161.8)	36.9 (21.2 to 59.8)	0.0 (0.0 to 0.0)	4522.3 (4042.4 to 5069.1)	2770.3 (2439.3 to 3145.8)	1684.8 (1357.7 to 2118.2)	67.2 (38.7 to 109.1)	0.0 (0.0 to 0.0)
Lithuania	2050	2527.2 (2246.4 to 2843.1)	1549.8 (1357.7 to 1764.9)	939.6 (755.0 to 1183.1)	37.8 (21.7 to 61.3)	0.0 (0.0 to 0.0)	4607.7 (4095.7 to 5183.6)	2825.6 (2475.4 to 3217.8)	1713.1 (1376.5 to 2157.1)	69.0 (39.6 to 111.8)	0.0 (0.0 to 0.0)
Luxembourg	1995	3782.2 (3500.1 to 4092.0)	3372.3 (3110.8 to 3656.2)	263.6 (216.3 to 325.8)	146.3 (88.5 to 227.8)	0.0 (0.0 to 0.0)	3594.0 (3325.9 to 3888.3)	3204.5 (2956.0 to 3474.2)	250.5 (205.6 to 309.6)	139.0 (84.1 to 216.5)	0.0 (0.0 to 0.0)
Luxembourg	1996	3873.0 (3655.3 to 4112.4)	3426.5 (3219.7 to 3647.3)	293.4 (245.1 to 355.9)	153.1 (98.9 to 225.8)	0.0 (0.0 to 0.0)	3680.3 (3473.4 to 3907.7)	3256.0 (3059.5 to 3465.8)	278.8 (232.9 to 338.2)	145.5 (94.0 to 214.5)	0.0 (0.0 to 0.0)
Luxembourg	1997	4066.1 (3862.9 to 4279.8)	3571.3 (3377.9 to 3768.9)	332.4 (283.6 to 388.1)	162.4 (113.2 to 224.5)	0.0 (0.0 to 0.0)	3863.8 (3670.6 to 4066.8)	3393.5 (3209.8 to 3581.4)	315.9 (269.5 to 368.8)	154.3 (107.6 to 213.4)	0.0 (0.0 to 0.0)
Luxembourg	1998	4370.7 (4155.9 to 4589.5)	3806.1 (3603.2 to 4005.6)	390.8 (340.7 to 444.5)	173.8 (130.0 to 227.1)	0.0 (0.0 to 0.0)	4153.1 (3949.1 to 4361.1)	3616.6 (3423.9 to 3806.3)	371.3 (323.8 to 422.4)	165.2 (123.6 to 215.8)	0.0 (0.0 to 0.0)
Luxembourg	1999	4918.4 (4696.0 to 5144.7)	4230.8 (4021.5 to 4436.7)	495.3 (442.5 to 549.4)	192.3 (154.7 to 234.2)	0.0 (0.0 to 0.0)	4673.7 (4462.3 to 4888.7)	4020.3 (3821.3 to 4215.9)	470.6 (420.5 to 522.1)	182.7 (147.0 to 222.5)	0.0 (0.0 to 0.0)
Luxembourg	2000	5707.9 (5480.5 to 5948.1)	4778.0 (4561.8 to 4990.7)	709.0 (650.6 to 772.4)	220.9 (187.9 to 257.5)	0.0 (0.0 to 0.0)	5423.8 (5207.8 to 5652.0)	4540.2 (4334.7 to 4742.4)	673.8 (618.2 to 734.0)	209.9 (178.6 to 244.7)	0.0 (0.0 to 0.0)
Luxembourg	2001	6261.1 (6027.3 to 6506.4)	5171.4 (4955.4 to 5396.2)	851.9 (790.8 to 916.0)	237.8 (208.4 to 270.0)	0.0 (0.0 to 0.0)	5949.5 (5727.4 to 6182.6)	4914.0 (4708.8 to 5127.7)	809.5 (751.5 to 870.4)	226.0 (198.0 to 256.6)	0.0 (0.0 to 0.0)

		2018 US Dollars per capita					2018 PPP per capita				
Country	Year	Health spending	Government health spending	Out-of-pocket health spending	Prepaid private health spending	Development assistance for health	Health spending	Government health spending	Out-of-pocket health spending	Prepaid private health spending	Development assistance for health
Luxembourg	2002	6866.2 (6629.8 to 7112.0)	5651.6 (5441.6 to 5896.7)	952.8 (890.2 to 1022.0)	261.8 (232.1 to 294.9)	0.0 (0.0 to 0.0)	6524.5 (6299.9 to 6758.1)	5370.3 (5170.8 to 5603.2)	905.4 (845.9 to 971.1)	248.8 (220.5 to 280.3)	0.0 (0.0 to 0.0)
Luxembourg	2003	7212.1 (6980.9 to 7444.6)	5933.8 (5718.4 to 6150.9)	987.6 (922.2 to 1058.7)	290.7 (260.2 to 326.4)	0.0 (0.0 to 0.0)	6853.2 (6633.5 to 7074.1)	5638.5 (5433.8 to 5844.8)	938.4 (876.3 to 1006.0)	276.3 (247.2 to 310.2)	0.0 (0.0 to 0.0)
Luxembourg	2004	7568.7 (7334.7 to 7806.0)	6246.7 (6024.7 to 6465.4)	1003.8 (940.9 to 1066.6)	318.2 (287.0 to 354.6)	0.0 (0.0 to 0.0)	7192.0 (6969.6 to 7417.5)	5935.8 (5724.8 to 6143.6)	953.8 (894.1 to 1013.5)	302.3 (272.7 to 337.0)	0.0 (0.0 to 0.0)
Luxembourg	2005	7531.2 (7299.6 to 7771.7)	6229.4 (6008.7 to 6458.3)	973.7 (912.1 to 1034.1)	328.1 (295.4 to 362.0)	0.0 (0.0 to 0.0)	7156.4 (6936.3 to 7384.9)	5919.3 (5709.7 to 6136.8)	925.2 (866.7 to 982.7)	311.8 (280.7 to 344.0)	0.0 (0.0 to 0.0)
Luxembourg	2006	7513.0 (7276.1 to 7751.0)	6257.3 (6032.7 to 6496.3)	921.2 (856.7 to 983.6)	334.5 (302.7 to 368.9)	0.0 (0.0 to 0.0)	7139.1 (6914.0 to 7365.2)	5945.9 (5732.5 to 6173.0)	875.3 (814.0 to 934.7)	317.9 (287.6 to 350.6)	0.0 (0.0 to 0.0)
Luxembourg	2007	7667.1 (7434.1 to 7919.6)	6470.1 (6243.3 to 6713.6)	842.2 (782.4 to 905.3)	354.9 (322.7 to 389.6)	0.0 (0.0 to 0.0)	7285.6 (7064.1 to 7525.4)	6148.1 (5932.6 to 6379.5)	800.3 (743.4 to 860.2)	337.2 (306.6 to 370.2)	0.0 (0.0 to 0.0)
Luxembourg	2008	7684.0 (7445.0 to 7933.0)	6548.7 (6311.0 to 6787.8)	796.4 (737.2 to 851.9)	339.0 (307.5 to 372.2)	0.0 (0.0 to 0.0)	7301.6 (7074.5 to 7538.1)	6222.7 (5996.9 to 6449.9)	756.7 (700.5 to 809.5)	322.1 (292.2 to 353.7)	0.0 (0.0 to 0.0)
Luxembourg	2009	7476.7 (7244.8 to 7714.9)	6355.6 (6139.5 to 6585.2)	768.4 (710.3 to 829.6)	352.7 (321.6 to 387.4)	0.0 (0.0 to 0.0)	7104.6 (6884.2 to 7330.9)	6039.3 (5834.0 to 6257.5)	730.2 (674.9 to 788.3)	335.1 (305.6 to 368.1)	0.0 (0.0 to 0.0)
Luxembourg	2010	7496.1 (7252.3 to 7748.7)	6360.0 (6127.4 to 6590.5)	767.2 (708.2 to 828.3)	368.9 (335.4 to 404.7)	0.0 (0.0 to 0.0)	7123.1 (6891.4 to 7363.1)	6043.5 (5822.5 to 6262.5)	729.1 (673.0 to 787.1)	350.5 (318.7 to 384.6)	0.0 (0.0 to 0.0)
Luxembourg	2011	7231.9 (7002.4 to 7461.8)	6121.2 (5893.5 to 6339.2)	750.7 (690.7 to 808.5)	360.0 (327.2 to 392.9)	0.0 (0.0 to 0.0)	6872.0 (6654.0 to 7090.5)	5816.6 (5600.2 to 6023.7)	713.4 (656.3 to 768.3)	342.1 (310.9 to 373.3)	0.0 (0.0 to 0.0)
Luxembourg	2012	7032.3 (6799.6 to 7263.2)	5921.4 (5705.9 to 6140.7)	742.5 (685.7 to 799.1)	368.3 (336.5 to 402.4)	0.0 (0.0 to 0.0)	6682.3 (6461.2 to 6901.8)	5626.7 (5422.0 to 5835.1)	705.5 (651.6 to 759.3)	350.0 (319.7 to 382.4)	0.0 (0.0 to 0.0)
Luxembourg	2013	7032.6 (6813.6 to 7277.7)	5890.8 (5688.7 to 6120.8)	750.3 (693.8 to 809.9)	391.5 (356.6 to 427.3)	0.0 (0.0 to 0.0)	6682.6 (6474.5 to 6915.5)	5597.6 (5405.6 to 5816.2)	712.9 (659.3 to 769.6)	372.0 (338.9 to 406.0)	0.0 (0.0 to 0.0)
Luxembourg	2014	7096.7 (6873.0 to 7331.7)	5918.5 (5720.4 to 6145.0)	772.4 (711.1 to 835.2)	405.9 (370.7 to 444.1)	0.0 (0.0 to 0.0)	6743.5 (6531.0 to 6966.9)	5623.9 (5435.7 to 5839.2)	734.0 (675.7 to 793.6)	385.7 (352.3 to 422.0)	0.0 (0.0 to 0.0)
Luxembourg	2015	7030.4 (6791.6 to 7283.6)	5829.4 (5608.3 to 6077.7)	781.9 (716.1 to 853.8)	419.2 (381.8 to 458.9)	0.0 (0.0 to 0.0)	6680.6 (6453.6 to 6921.1)	5539.3 (5329.2 to 5775.2)	743.0 (680.5 to 811.3)	398.3 (362.8 to 436.1)	0.0 (0.0 to 0.0)
Luxembourg	2016	7026.8 (6713.2 to 7360.0)	5790.0 (5487.3 to 6117.0)	794.3 (715.3 to 887.2)	442.5 (391.1 to 498.2)	0.0 (0.0 to 0.0)	6677.1 (6379.1 to 6993.7)	5501.9 (5214.2 to 5812.6)	754.7 (679.7 to 843.1)	420.5 (371.6 to 473.4)	0.0 (0.0 to 0.0)
Luxembourg	2017	7037.3 (6706.2 to 7384.2)	5798.5 (5492.6 to 6122.4)	794.6 (715.0 to 891.0)	444.2 (392.9 to 499.6)	0.0 (0.0 to 0.0)	6687.1 (6372.4 to 7016.7)	5510.0 (5219.3 to 5817.7)	755.0 (679.4 to 846.7)	422.1 (373.4 to 474.8)	0.0 (0.0 to 0.0)
Luxembourg	2018	7124.0 (6804.5 to 7482.5)	5872.2 (5566.4 to 6220.0)	800.5 (720.5 to 898.2)	451.3 (398.7 to 507.8)	0.0 (0.0 to 0.0)	6769.5 (6465.9 to 7110.1)	5580.0 (5289.4 to 5910.4)	760.6 (684.6 to 853.5)	428.8 (378.9 to 482.6)	0.0 (0.0 to 0.0)
Luxembourg	2019	7212.6 (6875.0 to 7582.4)	5948.3 (5637.3 to 6296.2)	806.5 (726.3 to 905.3)	457.8 (405.2 to 515.2)	0.0 (0.0 to 0.0)	6853.7 (6532.9 to 7205.1)	5652.3 (5356.7 to 5982.8)	766.4 (690.1 to 860.2)	435.0 (385.0 to 489.6)	0.0 (0.0 to 0.0)
Luxembourg	2020	7306.6 (6977.7 to 7664.6)	6029.1 (5715.1 to 6385.1)	812.5 (730.4 to 912.0)	464.9 (411.0 to 522.8)	0.0 (0.0 to 0.0)	6942.9 (6630.4 to 7283.2)	5729.1 (5430.7 to 6067.3)	772.1 (694.1 to 866.6)	441.8 (390.5 to 496.8)	0.0 (0.0 to 0.0)
Luxembourg	2021	7400.2 (7053.2 to 7769.3)	6111.1 (5785.4 to 6463.6)	817.6 (733.9 to 918.7)	471.5 (417.2 to 529.9)	0.0 (0.0 to 0.0)	7031.9 (6702.2 to 7382.7)	5807.0 (5497.5 to 6142.0)	776.9 (697.3 to 873.0)	448.0 (396.5 to 503.6)	0.0 (0.0 to 0.0)
Luxembourg	2022	7494.2 (7131.5 to 7885.4)	6193.6 (5860.8 to 6569.0)	822.7 (737.7 to 923.4)	478.0 (422.9 to 537.3)	0.0 (0.0 to 0.0)	7121.3 (6776.6 to 7493.0)	5885.3 (5569.2 to 6242.1)	781.7 (701.0 to 877.4)	454.2 (401.9 to 510.6)	0.0 (0.0 to 0.0)

Country	Year	2018 US Dollars per capita					2018 PPP per capita				
		Health spending	Government health spending	Out-of-pocket health spending	Prepaid private health spending	Development assistance for health	Health spending	Government health spending	Out-of-pocket health spending	Prepaid private health spending	Development assistance for health
Luxembourg	2023	7592.7 (7243.6 to 7992.5)	6280.0 (5933.1 to 6671.8)	828.0 (744.5 to 930.2)	484.7 (428.5 to 544.7)	0.0 (0.0 to 0.0)	7214.9 (6883.1 to 7594.8)	5967.5 (5637.9 to 6339.8)	786.8 (707.4 to 883.9)	460.6 (407.2 to 517.6)	0.0 (0.0 to 0.0)
Luxembourg	2024	7662.6 (7260.2 to 8065.7)	6342.0 (5977.9 to 6744.8)	830.6 (746.8 to 932.5)	489.9 (432.4 to 550.5)	0.0 (0.0 to 0.0)	7281.2 (6898.9 to 7664.3)	6026.4 (5680.4 to 6409.1)	789.3 (709.6 to 886.1)	465.5 (410.8 to 523.1)	0.0 (0.0 to 0.0)
Luxembourg	2025	7714.8 (7328.6 to 8129.1)	6390.0 (6006.5 to 6778.0)	831.0 (747.3 to 933.6)	493.7 (434.9 to 554.6)	0.0 (0.0 to 0.0)	7330.8 (6963.9 to 7724.5)	6072.0 (5707.6 to 6440.7)	789.7 (710.1 to 887.1)	469.2 (413.2 to 527.0)	0.0 (0.0 to 0.0)
Luxembourg	2026	7755.0 (7328.8 to 8159.7)	6429.1 (6033.2 to 6824.3)	829.5 (745.6 to 931.7)	496.4 (436.9 to 557.3)	0.0 (0.0 to 0.0)	7369.1 (6964.1 to 7753.6)	6109.2 (5733.0 to 6484.7)	788.2 (708.5 to 885.3)	471.7 (415.1 to 529.6)	0.0 (0.0 to 0.0)
Luxembourg	2027	7787.4 (7371.9 to 8228.7)	6461.7 (6042.2 to 6885.9)	827.2 (739.7 to 929.2)	498.5 (438.8 to 559.9)	0.0 (0.0 to 0.0)	7399.8 (7005.0 to 7819.2)	6140.1 (5741.5 to 6543.2)	786.0 (702.9 to 882.9)	473.7 (417.0 to 532.1)	0.0 (0.0 to 0.0)
Luxembourg	2028	7816.4 (7358.1 to 8271.8)	6491.5 (6049.4 to 6925.8)	824.5 (735.6 to 926.6)	500.4 (440.1 to 562.7)	0.0 (0.0 to 0.0)	7427.4 (6991.9 to 7860.1)	6168.5 (5748.4 to 6581.1)	783.4 (699.0 to 880.5)	475.5 (418.2 to 534.7)	0.0 (0.0 to 0.0)
Luxembourg	2029	7855.5 (7376.3 to 8335.4)	6531.1 (6068.5 to 6981.9)	822.1 (732.1 to 927.3)	502.4 (442.0 to 565.7)	0.0 (0.0 to 0.0)	7464.6 (7009.2 to 7920.6)	6206.0 (5766.5 to 6634.4)	781.2 (695.6 to 881.2)	477.4 (420.0 to 537.6)	0.0 (0.0 to 0.0)
Luxembourg	2030	7903.3 (7420.6 to 8399.0)	6578.4 (6096.3 to 7048.2)	820.2 (728.3 to 927.6)	504.6 (443.6 to 568.5)	0.0 (0.0 to 0.0)	7509.9 (7051.3 to 7981.0)	6251.0 (5792.9 to 6697.4)	779.4 (692.0 to 881.4)	479.5 (421.5 to 540.2)	0.0 (0.0 to 0.0)
Luxembourg	2031	7933.1 (7410.4 to 8448.8)	6610.4 (6102.8 to 7108.0)	816.7 (723.8 to 926.8)	506.0 (444.4 to 569.3)	0.0 (0.0 to 0.0)	7538.3 (7041.6 to 8028.3)	6281.4 (5799.1 to 6754.2)	776.1 (687.8 to 880.7)	480.8 (422.3 to 541.0)	0.0 (0.0 to 0.0)
Luxembourg	2032	7974.2 (7469.3 to 8506.9)	6652.1 (6136.2 to 7169.6)	814.2 (720.5 to 924.4)	507.9 (445.8 to 571.8)	0.0 (0.0 to 0.0)	7577.3 (7097.6 to 8083.6)	6321.0 (5830.8 to 6812.8)	773.7 (684.7 to 878.4)	482.6 (423.6 to 543.3)	0.0 (0.0 to 0.0)
Luxembourg	2033	8013.2 (7461.9 to 8572.1)	6691.8 (6151.4 to 7236.9)	811.6 (716.6 to 921.8)	509.8 (447.1 to 575.5)	0.0 (0.0 to 0.0)	7614.4 (7090.5 to 8145.5)	6358.7 (5845.3 to 6876.7)	771.2 (681.0 to 875.9)	484.4 (424.8 to 546.9)	0.0 (0.0 to 0.0)
Luxembourg	2034	8060.9 (7499.0 to 8655.0)	6738.3 (6167.1 to 7318.5)	810.1 (712.1 to 920.2)	512.5 (449.8 to 580.3)	0.0 (0.0 to 0.0)	7659.7 (7125.8 to 8224.3)	6402.9 (5860.2 to 6954.3)	769.8 (676.7 to 874.5)	487.0 (427.4 to 551.5)	0.0 (0.0 to 0.0)
Luxembourg	2035	8109.4 (7491.9 to 8738.4)	6784.9 (6190.6 to 7390.2)	808.9 (710.1 to 920.3)	515.5 (452.4 to 584.1)	0.0 (0.0 to 0.0)	7705.8 (7119.0 to 8303.5)	6447.3 (5882.5 to 7022.4)	768.7 (674.8 to 874.5)	489.9 (429.9 to 555.0)	0.0 (0.0 to 0.0)
Luxembourg	2036	8145.5 (7524.4 to 8790.1)	6819.6 (6209.1 to 7451.2)	807.4 (705.8 to 919.8)	518.5 (453.7 to 588.1)	0.0 (0.0 to 0.0)	7740.1 (7150.0 to 8352.6)	6480.2 (5900.1 to 7080.3)	767.2 (670.7 to 874.0)	492.7 (431.1 to 558.8)	0.0 (0.0 to 0.0)
Luxembourg	2037	8186.9 (7537.4 to 8837.9)	6858.2 (6220.5 to 7521.7)	806.6 (702.7 to 920.2)	522.1 (455.9 to 593.6)	0.0 (0.0 to 0.0)	7779.4 (7162.2 to 8398.1)	6516.9 (5911.0 to 7147.4)	766.4 (667.7 to 874.4)	496.1 (433.2 to 564.1)	0.0 (0.0 to 0.0)
Luxembourg	2038	8232.0 (7570.7 to 8955.0)	6899.2 (6229.5 to 7583.4)	806.5 (701.3 to 921.1)	526.3 (459.1 to 600.0)	0.0 (0.0 to 0.0)	7822.3 (7193.9 to 8509.3)	6555.9 (5919.5 to 7206.0)	766.3 (666.4 to 875.2)	500.1 (436.3 to 570.2)	0.0 (0.0 to 0.0)
Luxembourg	2039	8280.0 (7595.2 to 8988.3)	6942.6 (6260.6 to 7646.2)	806.7 (700.5 to 922.8)	530.7 (461.5 to 607.2)	0.0 (0.0 to 0.0)	7867.9 (7217.2 to 8541.0)	6597.1 (5949.0 to 7265.7)	766.5 (665.6 to 876.8)	504.3 (438.5 to 577.0)	0.0 (0.0 to 0.0)
Luxembourg	2040	8329.6 (7595.3 to 9031.0)	6986.9 (6260.9 to 7717.4)	807.2 (699.5 to 924.9)	535.5 (463.7 to 614.2)	0.0 (0.0 to 0.0)	7915.1 (7217.3 to 8581.6)	6639.2 (5949.3 to 7333.3)	767.0 (664.7 to 878.8)	508.8 (440.6 to 583.6)	0.0 (0.0 to 0.0)
Luxembourg	2041	8366.9 (7622.3 to 9109.3)	7019.7 (6269.2 to 7773.9)	807.1 (698.1 to 927.0)	540.1 (466.5 to 620.5)	0.0 (0.0 to 0.0)	7950.5 (7242.9 to 8655.9)	6670.3 (5957.2 to 7387.0)	767.0 (663.3 to 880.9)	513.2 (443.3 to 589.6)	0.0 (0.0 to 0.0)
Luxembourg	2042	8406.5 (7663.9 to 9180.1)	7054.1 (6289.8 to 7827.0)	807.4 (697.2 to 929.5)	545.0 (470.4 to 628.9)	0.0 (0.0 to 0.0)	7988.2 (7282.5 to 8723.2)	6703.0 (5976.8 to 7437.5)	767.2 (662.5 to 883.2)	517.9 (447.0 to 597.6)	0.0 (0.0 to 0.0)
Luxembourg	2043	8449.0 (7674.6 to 9258.5)	7090.9 (6325.3 to 7891.7)	807.9 (696.7 to 930.4)	550.2 (474.4 to 635.3)	0.0 (0.0 to 0.0)	8028.5 (7292.7 to 8797.8)	6738.0 (6010.5 to 7498.9)	767.7 (662.0 to 884.1)	522.8 (450.8 to 603.7)	0.0 (0.0 to 0.0)

Country	Year	2018 US Dollars per capita					2018 PPP per capita				
		Health spending	Government health spending	Out-of-pocket health spending	Prepaid private health spending	Development assistance for health	Health spending	Government health spending	Out-of-pocket health spending	Prepaid private health spending	Development assistance for health
Luxembourg	2044	8495.8 (7703.9 to 9309.7)	7131.7 (6336.4 to 7964.8)	808.6 (696.3 to 931.6)	555.5 (478.0 to 642.4)	0.0 (0.0 to 0.0)	8073.0 (7320.5 to 8846.4)	6776.8 (6021.1 to 7568.4)	768.4 (661.6 to 885.2)	527.8 (454.2 to 610.5)	0.0 (0.0 to 0.0)
Luxembourg	2045	8546.5 (7705.7 to 9415.3)	7176.4 (6366.6 to 8031.3)	809.3 (696.3 to 932.9)	560.7 (481.9 to 650.4)	0.0 (0.0 to 0.0)	8121.2 (7322.2 to 8946.7)	6819.3 (6049.7 to 7631.6)	769.0 (661.6 to 886.5)	532.8 (457.9 to 618.0)	0.0 (0.0 to 0.0)
Luxembourg	2046	8583.9 (7755.3 to 9467.7)	7209.5 (6379.1 to 8082.3)	809.0 (695.5 to 933.1)	565.4 (485.1 to 657.7)	0.0 (0.0 to 0.0)	8156.7 (7369.3 to 8996.5)	6850.7 (6061.6 to 7680.0)	768.7 (660.9 to 886.7)	537.3 (461.0 to 624.9)	0.0 (0.0 to 0.0)
Luxembourg	2047	8612.5 (7724.8 to 9497.3)	7235.7 (6372.1 to 8137.0)	807.5 (693.7 to 932.5)	569.3 (487.7 to 663.4)	0.0 (0.0 to 0.0)	8183.8 (7340.3 to 9024.7)	6875.6 (6055.0 to 7732.0)	767.3 (659.2 to 886.1)	540.9 (463.5 to 630.4)	0.0 (0.0 to 0.0)
Luxembourg	2048	8643.5 (7739.2 to 9555.0)	7264.2 (6372.9 to 8179.7)	806.0 (692.7 to 931.4)	573.2 (489.7 to 670.0)	0.0 (0.0 to 0.0)	8213.3 (7354.0 to 9079.5)	6902.7 (6055.7 to 7772.6)	765.9 (658.3 to 885.0)	544.6 (465.3 to 636.6)	0.0 (0.0 to 0.0)
Luxembourg	2049	8678.5 (7739.3 to 9637.0)	7296.0 (6357.7 to 8225.7)	805.1 (692.1 to 930.4)	577.4 (491.5 to 676.2)	0.0 (0.0 to 0.0)	8246.6 (7354.1 to 9157.4)	6932.9 (6041.3 to 7816.3)	765.0 (657.7 to 884.1)	548.7 (467.1 to 642.6)	0.0 (0.0 to 0.0)
Luxembourg	2050	8717.6 (7753.6 to 9667.4)	7330.9 (6369.2 to 8286.4)	804.6 (692.6 to 930.7)	582.1 (494.3 to 683.5)	0.0 (0.0 to 0.0)	8283.8 (7367.7 to 9186.3)	6966.1 (6052.3 to 7874.0)	764.6 (658.2 to 884.4)	553.2 (469.7 to 649.5)	0.0 (0.0 to 0.0)
Macedonia	1995	303.3 (270.8 to 339.7)	174.3 (150.8 to 202.0)	118.7 (97.2 to 142.3)	3.2 (1.5 to 5.9)	7.2 (7.2 to 7.2)	790.5 (705.7 to 885.3)	454.2 (393.1 to 526.4)	309.4 (253.3 to 370.9)	8.2 (3.9 to 15.5)	18.7 (18.7 to 18.7)
Macedonia	1996	305.5 (276.7 to 338.5)	176.9 (155.6 to 201.6)	120.1 (100.7 to 141.8)	3.2 (1.5 to 6.0)	5.3 (5.3 to 5.3)	796.3 (721.1 to 882.0)	461.1 (405.4 to 525.3)	313.1 (262.6 to 369.5)	8.4 (4.0 to 15.7)	13.7 (13.7 to 13.7)
Macedonia	1997	310.7 (284.3 to 341.0)	181.7 (161.5 to 204.0)	122.5 (104.5 to 142.7)	3.3 (1.6 to 6.0)	3.1 (3.1 to 3.1)	809.6 (741.0 to 888.7)	473.6 (420.9 to 531.6)	319.4 (272.3 to 371.8)	8.6 (4.1 to 15.8)	8.1 (8.1 to 8.1)
Macedonia	1998	327.1 (299.9 to 355.0)	188.7 (169.7 to 211.5)	126.6 (108.2 to 146.8)	3.4 (1.6 to 6.2)	8.5 (8.5 to 8.5)	852.3 (781.7 to 925.1)	491.7 (442.2 to 551.1)	329.8 (281.9 to 382.7)	8.7 (4.3 to 16.1)	22.1 (22.1 to 22.1)
Macedonia	1999	335.1 (307.3 to 362.3)	189.3 (171.0 to 210.9)	131.5 (113.3 to 152.3)	3.3 (1.6 to 6.1)	11.0 (11.0 to 11.0)	873.2 (800.9 to 944.2)	493.3 (445.8 to 549.5)	342.6 (295.2 to 397.0)	8.7 (4.3 to 15.9)	28.6 (28.6 to 28.6)
Macedonia	2000	342.4 (315.0 to 369.6)	189.9 (171.6 to 210.6)	139.0 (119.3 to 161.6)	3.3 (1.6 to 6.1)	10.1 (10.1 to 10.1)	892.2 (820.8 to 963.3)	495.0 (447.2 to 548.8)	362.1 (310.9 to 421.2)	8.7 (4.2 to 16.0)	26.4 (26.4 to 26.4)
Macedonia	2001	335.5 (308.8 to 362.1)	183.4 (165.8 to 202.9)	139.3 (119.6 to 161.9)	3.4 (1.6 to 6.1)	9.4 (9.4 to 9.4)	874.3 (804.7 to 943.6)	478.0 (432.0 to 528.8)	363.0 (311.8 to 422.0)	8.8 (4.2 to 16.0)	24.6 (24.6 to 24.6)
Macedonia	2002	329.4 (302.7 to 357.6)	181.7 (164.6 to 200.3)	141.6 (121.6 to 165.3)	3.5 (1.7 to 6.5)	2.6 (2.6 to 2.6)	858.4 (788.9 to 931.9)	473.4 (429.1 to 521.9)	369.0 (316.8 to 430.8)	9.2 (4.4 to 17.0)	6.7 (6.7 to 6.7)
Macedonia	2003	330.7 (304.0 to 358.1)	181.9 (165.0 to 200.9)	142.0 (123.3 to 165.2)	3.7 (1.8 to 7.0)	3.1 (3.1 to 3.1)	861.9 (792.4 to 933.3)	474.1 (430.0 to 523.7)	370.0 (321.3 to 430.4)	9.7 (4.6 to 18.1)	8.0 (8.0 to 8.0)
Macedonia	2004	327.9 (300.8 to 356.6)	181.3 (162.8 to 201.1)	137.3 (118.6 to 159.9)	3.9 (1.8 to 7.2)	5.4 (5.4 to 5.4)	854.5 (783.9 to 929.3)	472.6 (424.3 to 524.1)	357.7 (309.1 to 416.6)	10.2 (4.8 to 18.7)	14.0 (14.0 to 14.0)
Macedonia	2005	324.7 (296.8 to 353.7)	182.3 (163.5 to 202.1)	129.8 (111.8 to 150.2)	4.1 (1.8 to 7.5)	8.5 (8.5 to 8.5)	846.2 (773.6 to 921.9)	475.1 (426.1 to 526.8)	338.3 (291.5 to 391.4)	10.7 (4.8 to 19.5)	22.1 (22.1 to 22.1)
Macedonia	2006	313.6 (286.4 to 340.7)	183.7 (163.7 to 203.7)	121.1 (103.9 to 140.6)	4.3 (1.9 to 7.8)	4.5 (4.5 to 4.5)	817.3 (746.4 to 888.0)	478.8 (426.7 to 530.9)	315.6 (270.7 to 366.5)	11.1 (5.1 to 20.4)	11.8 (11.8 to 11.8)
Macedonia	2007	322.3 (296.0 to 349.5)	190.6 (170.8 to 210.7)	115.7 (98.9 to 134.2)	4.5 (2.1 to 8.3)	11.6 (11.6 to 11.6)	840.0 (771.5 to 910.8)	496.7 (445.1 to 549.1)	301.4 (257.8 to 349.9)	11.7 (5.4 to 21.7)	30.1 (30.1 to 30.1)
Macedonia	2008	322.8 (297.1 to 349.8)	200.3 (180.5 to 222.5)	112.5 (96.2 to 130.1)	4.8 (2.3 to 9.0)	5.2 (5.2 to 5.2)	841.1 (774.3 to 911.6)	521.9 (470.3 to 579.8)	293.2 (250.7 to 339.1)	12.5 (6.0 to 23.4)	13.6 (13.6 to 13.6)

		2018 US Dollars per capita					2018 PPP per capita				
Country	Year	Health spending	Government health spending	Out-of-pocket health spending	Prepaid private health spending	Development assistance for health	Health spending	Government health spending	Out-of-pocket health spending	Prepaid private health spending	Development assistance for health
Macedonia	2009	320.9 (295.5 to 348.7)	201.0 (181.4 to 222.9)	113.2 (96.4 to 131.5)	4.9 (2.3 to 9.5)	1.8 (1.8 to 1.8)	836.3 (770.2 to 908.8)	523.8 (472.6 to 580.9)	295.1 (251.2 to 342.6)	12.7 (6.0 to 24.6)	4.7 (4.7 to 4.7)
Macedonia	2010	334.8 (306.9 to 363.1)	205.4 (186.1 to 228.0)	117.6 (100.2 to 137.2)	5.0 (2.3 to 10.0)	6.8 (6.8 to 6.8)	872.5 (799.8 to 946.2)	535.2 (484.9 to 594.3)	306.4 (261.1 to 357.7)	13.1 (5.9 to 26.0)	17.7 (17.7 to 17.7)
Macedonia	2011	334.3 (307.9 to 363.0)	207.6 (188.0 to 230.6)	117.4 (100.2 to 137.0)	5.1 (2.3 to 10.1)	4.1 (4.1 to 4.1)	871.3 (802.5 to 946.0)	541.1 (490.0 to 601.1)	306.0 (261.0 to 357.1)	13.4 (5.9 to 26.4)	10.7 (10.7 to 10.7)
Macedonia	2012	331.9 (305.1 to 360.5)	209.0 (189.3 to 232.2)	116.6 (99.3 to 136.2)	5.2 (2.3 to 10.2)	1.1 (1.1 to 1.1)	864.9 (795.2 to 939.5)	544.6 (493.4 to 605.1)	303.8 (258.8 to 354.9)	13.6 (6.0 to 26.6)	2.7 (2.7 to 2.7)
Macedonia	2013	343.1 (314.1 to 373.3)	217.0 (195.4 to 240.2)	117.7 (99.8 to 138.7)	5.5 (2.5 to 10.7)	2.9 (2.9 to 2.9)	894.2 (818.5 to 972.9)	565.6 (509.2 to 625.9)	306.9 (260.2 to 361.6)	14.2 (6.5 to 27.8)	7.6 (7.6 to 7.6)
Macedonia	2014	345.4 (315.5 to 377.0)	219.8 (196.2 to 242.6)	118.9 (100.2 to 141.8)	5.6 (2.6 to 11.0)	1.1 (1.1 to 1.1)	900.2 (822.3 to 982.6)	572.9 (511.4 to 632.4)	309.9 (261.2 to 369.4)	14.6 (6.7 to 28.5)	2.8 (2.8 to 2.8)
Macedonia	2015	356.2 (323.3 to 392.8)	226.2 (200.0 to 253.0)	121.8 (102.2 to 146.9)	5.9 (2.6 to 11.1)	2.2 (2.2 to 2.2)	928.2 (842.6 to 1023.7)	589.6 (521.2 to 659.4)	317.5 (266.4 to 382.8)	15.3 (6.9 to 29.1)	5.8 (5.8 to 5.8)
Macedonia	2016	364.1 (325.9 to 403.9)	231.3 (201.6 to 263.8)	125.6 (103.4 to 153.8)	6.1 (2.8 to 11.4)	1.0 (1.0 to 1.0)	948.9 (849.5 to 1052.7)	602.8 (525.5 to 687.6)	327.3 (269.5 to 400.7)	16.0 (7.2 to 29.8)	2.7 (2.7 to 2.7)
Macedonia	2017	369.9 (330.5 to 410.0)	236.2 (205.9 to 269.9)	126.7 (103.7 to 155.4)	6.2 (2.8 to 11.6)	0.9 (0.9 to 0.9)	964.1 (861.3 to 1068.5)	615.5 (536.6 to 703.3)	330.1 (270.4 to 404.9)	16.2 (7.2 to 30.1)	2.3 (2.3 to 2.3)
Macedonia	2018	374.3 (333.6 to 415.4)	239.1 (208.6 to 273.2)	128.0 (104.6 to 156.8)	6.3 (2.8 to 11.7)	0.8 (0.8 to 0.8)	975.4 (869.5 to 1082.5)	623.1 (543.7 to 711.9)	333.7 (272.5 to 408.6)	16.4 (7.3 to 30.5)	2.2 (2.2 to 2.2)
Macedonia	2019	379.2 (338.6 to 420.8)	242.5 (211.6 to 276.5)	129.4 (105.2 to 158.3)	6.4 (2.9 to 11.9)	0.9 (0.8 to 0.9)	988.1 (882.5 to 1096.7)	631.9 (551.6 to 720.6)	337.4 (274.2 to 412.6)	16.6 (7.4 to 30.9)	2.2 (2.1 to 2.4)
Macedonia	2020	383.7 (342.7 to 425.9)	245.5 (214.2 to 280.0)	130.8 (106.4 to 159.6)	6.5 (2.9 to 12.0)	0.9 (0.8 to 1.0)	999.9 (893.1 to 1110.0)	639.9 (558.1 to 729.7)	340.9 (277.4 to 415.8)	16.8 (7.5 to 31.4)	2.3 (2.1 to 2.5)
Macedonia	2021	388.0 (345.5 to 430.8)	248.4 (216.1 to 282.6)	132.1 (107.7 to 161.6)	6.6 (2.9 to 12.2)	0.9 (0.8 to 1.0)	1011.1 (900.4 to 1122.9)	647.4 (563.1 to 736.4)	344.3 (280.8 to 421.0)	17.1 (7.6 to 31.8)	2.4 (2.1 to 2.6)
Macedonia	2022	392.1 (349.5 to 436.4)	251.1 (218.4 to 286.5)	133.4 (108.2 to 163.5)	6.6 (3.0 to 12.3)	0.9 (0.8 to 1.1)	1022.0 (910.9 to 1137.4)	654.5 (569.1 to 746.5)	347.7 (282.1 to 426.1)	17.3 (7.8 to 32.1)	2.5 (2.2 to 2.8)
Macedonia	2023	396.5 (353.3 to 440.9)	254.1 (220.7 to 289.7)	134.7 (109.9 to 165.2)	6.7 (3.0 to 12.4)	1.0 (0.9 to 1.1)	1033.4 (920.9 to 1149.1)	662.2 (575.2 to 755.0)	351.1 (286.4 to 430.5)	17.5 (7.9 to 32.4)	2.6 (2.2 to 2.9)
Macedonia	2024	400.5 (356.2 to 445.6)	256.7 (222.8 to 293.2)	136.0 (110.7 to 166.5)	6.8 (3.1 to 12.6)	1.0 (0.9 to 1.2)	1043.7 (928.4 to 1161.3)	669.0 (580.5 to 764.2)	354.3 (288.5 to 433.8)	17.8 (8.0 to 32.8)	2.6 (2.3 to 3.0)
Macedonia	2025	404.4 (360.5 to 450.9)	259.2 (224.7 to 296.7)	137.2 (111.5 to 167.8)	6.9 (3.1 to 12.7)	1.1 (0.9 to 1.2)	1053.9 (939.6 to 1175.0)	675.6 (585.7 to 773.2)	357.6 (290.7 to 437.3)	18.0 (8.1 to 33.2)	2.7 (2.3 to 3.2)
Macedonia	2026	408.2 (363.3 to 456.0)	261.7 (226.4 to 300.2)	138.4 (112.4 to 169.4)	7.0 (3.1 to 12.9)	1.1 (0.9 to 1.3)	1063.8 (946.9 to 1188.4)	682.0 (590.1 to 782.5)	360.8 (293.0 to 441.5)	18.2 (8.1 to 33.6)	2.8 (2.4 to 3.3)
Macedonia	2027	412.0 (366.1 to 460.3)	264.1 (228.8 to 303.6)	139.7 (113.3 to 172.0)	7.1 (3.2 to 13.1)	1.1 (0.9 to 1.3)	1073.8 (954.0 to 1199.7)	688.4 (596.4 to 791.2)	364.1 (295.3 to 448.2)	18.4 (8.2 to 34.0)	2.9 (2.5 to 3.5)
Macedonia	2028	415.9 (369.1 to 465.8)	266.6 (230.4 to 307.8)	141.0 (113.7 to 174.0)	7.2 (3.2 to 13.2)	1.2 (1.0 to 1.4)	1083.8 (961.8 to 1214.0)	694.7 (600.4 to 802.2)	367.4 (296.2 to 453.6)	18.6 (8.3 to 34.5)	3.0 (2.5 to 3.7)
Macedonia	2029	419.7 (373.6 to 470.9)	268.9 (232.3 to 310.6)	142.3 (114.6 to 176.9)	7.2 (3.3 to 13.4)	1.2 (1.0 to 1.5)	1093.8 (973.7 to 1227.1)	700.9 (605.4 to 809.5)	370.8 (298.7 to 460.9)	18.9 (8.5 to 34.9)	3.1 (2.6 to 3.9)

		2018 US Dollars per capita					2018 PPP per capita				
Country	Year	Health spending	Government health spending	Out-of-pocket health spending	Prepaid private health spending	Development assistance for health	Health spending	Government health spending	Out-of-pocket health spending	Prepaid private health spending	Development assistance for health
Macedonia	2030	423.6 (375.6 to 474.3)	271.4 (233.9 to 314.2)	143.6 (115.0 to 178.9)	7.3 (3.3 to 13.5)	1.3 (1.0 to 1.5)	1103.9 (978.9 to 1236.1)	707.2 (609.6 to 818.8)	374.3 (299.6 to 466.3)	19.1 (8.6 to 35.3)	3.3 (2.7 to 4.0)
Macedonia	2031	427.6 (379.1 to 481.1)	273.8 (236.4 to 318.3)	145.0 (115.4 to 181.2)	7.4 (3.4 to 13.7)	1.3 (1.1 to 1.6)	1114.4 (988.1 to 1253.9)	713.7 (616.0 to 829.5)	378.0 (300.8 to 472.4)	19.3 (8.7 to 35.8)	3.4 (2.7 to 4.2)
Macedonia	2032	431.6 (381.5 to 485.8)	276.3 (237.8 to 321.5)	146.5 (115.6 to 184.3)	7.5 (3.4 to 13.9)	1.3 (1.1 to 1.7)	1124.9 (994.2 to 1266.0)	720.0 (619.7 to 837.8)	381.8 (301.3 to 480.3)	19.6 (8.9 to 36.2)	3.5 (2.8 to 4.4)
Macedonia	2033	435.9 (385.2 to 492.9)	278.9 (239.3 to 325.3)	148.0 (115.9 to 187.1)	7.6 (3.5 to 14.1)	1.4 (1.1 to 1.8)	1136.1 (1004.0 to 1284.5)	727.0 (623.7 to 847.8)	385.7 (302.1 to 487.5)	19.8 (9.0 to 36.7)	3.6 (2.9 to 4.7)
Macedonia	2034	440.3 (388.9 to 499.0)	281.6 (240.8 to 329.3)	149.5 (116.5 to 190.5)	7.7 (3.5 to 14.3)	1.4 (1.1 to 1.9)	1147.5 (1013.6 to 1300.4)	733.9 (627.7 to 858.3)	389.7 (303.5 to 496.5)	20.1 (9.1 to 37.2)	3.8 (3.0 to 4.9)
Macedonia	2035	444.9 (390.9 to 506.2)	284.4 (243.7 to 333.5)	151.2 (116.9 to 193.4)	7.8 (3.5 to 14.5)	1.5 (1.2 to 2.0)	1159.5 (1018.7 to 1319.2)	741.2 (635.1 to 869.2)	394.0 (304.7 to 504.1)	20.4 (9.2 to 37.7)	3.9 (3.0 to 5.1)
Macedonia	2036	449.6 (394.1 to 510.9)	287.2 (245.6 to 337.9)	152.9 (117.9 to 196.9)	7.9 (3.6 to 14.7)	1.6 (1.2 to 2.1)	1171.7 (1027.0 to 1331.5)	748.6 (640.2 to 880.7)	398.4 (307.2 to 513.1)	20.7 (9.3 to 38.2)	4.1 (3.1 to 5.4)
Macedonia	2037	454.4 (396.8 to 516.3)	290.2 (247.5 to 342.0)	154.6 (118.7 to 199.2)	8.1 (3.6 to 14.8)	1.6 (1.2 to 2.2)	1184.3 (1034.1 to 1345.7)	756.2 (644.9 to 891.2)	402.9 (309.3 to 519.2)	21.0 (9.4 to 38.7)	4.2 (3.2 to 5.6)
Macedonia	2038	459.1 (400.5 to 524.4)	292.9 (249.8 to 345.5)	156.3 (119.6 to 201.6)	8.2 (3.7 to 15.0)	1.7 (1.3 to 2.3)	1196.4 (1043.8 to 1366.7)	763.4 (651.0 to 900.5)	407.3 (311.7 to 525.5)	21.3 (9.6 to 39.2)	4.4 (3.3 to 6.0)
Macedonia	2039	464.2 (405.1 to 529.8)	296.0 (252.4 to 349.7)	158.1 (120.5 to 205.3)	8.3 (3.7 to 15.3)	1.7 (1.3 to 2.4)	1209.7 (1055.8 to 1380.8)	771.5 (657.8 to 911.3)	412.1 (313.9 to 535.2)	21.6 (9.7 to 39.8)	4.5 (3.4 to 6.3)
Macedonia	2040	468.9 (408.6 to 536.8)	298.8 (254.4 to 351.8)	159.9 (121.4 to 208.3)	8.4 (3.8 to 15.5)	1.8 (1.4 to 2.5)	1222.0 (1064.9 to 1398.9)	778.6 (663.0 to 916.9)	416.8 (316.5 to 542.9)	21.9 (9.8 to 40.3)	4.7 (3.6 to 6.5)
Macedonia	2041	473.6 (412.8 to 542.9)	301.5 (256.3 to 357.2)	161.8 (122.4 to 212.0)	8.5 (3.8 to 15.6)	1.9 (1.4 to 2.6)	1234.3 (1075.9 to 1414.8)	785.7 (667.9 to 931.0)	421.5 (319.0 to 552.4)	22.2 (9.9 to 40.7)	4.9 (3.7 to 6.8)
Macedonia	2042	478.5 (415.1 to 549.5)	304.3 (258.2 to 361.8)	163.6 (123.3 to 215.2)	8.6 (3.9 to 15.8)	1.9 (1.5 to 2.8)	1247.1 (1081.9 to 1432.1)	793.1 (672.8 to 943.0)	426.5 (321.3 to 560.9)	22.5 (10.0 to 41.2)	5.1 (3.8 to 7.2)
Macedonia	2043	483.4 (419.5 to 555.1)	307.1 (260.7 to 365.0)	165.5 (124.3 to 217.7)	8.8 (3.9 to 16.0)	2.0 (1.5 to 2.9)	1259.8 (1093.3 to 1446.7)	800.3 (679.5 to 951.4)	431.4 (324.1 to 567.4)	22.9 (10.2 to 41.7)	5.2 (3.9 to 7.6)
Macedonia	2044	488.3 (423.7 to 561.7)	309.8 (263.0 to 368.4)	167.5 (125.3 to 220.6)	8.9 (3.9 to 16.2)	2.1 (1.6 to 3.0)	1272.5 (1104.2 to 1464.0)	807.4 (685.4 to 960.0)	436.4 (326.6 to 575.0)	23.2 (10.3 to 42.3)	5.5 (4.0 to 7.9)
Macedonia	2045	493.3 (425.9 to 568.2)	312.7 (264.6 to 372.5)	169.4 (126.5 to 223.2)	9.0 (4.0 to 16.5)	2.2 (1.6 to 3.3)	1285.6 (1109.8 to 1480.9)	814.9 (689.6 to 970.7)	441.5 (329.7 to 581.6)	23.5 (10.4 to 42.9)	5.7 (4.1 to 8.5)
Macedonia	2046	498.0 (431.3 to 575.2)	315.2 (266.4 to 376.8)	171.4 (127.9 to 225.1)	9.1 (4.0 to 16.7)	2.3 (1.6 to 3.5)	1297.8 (1124.0 to 1499.2)	821.5 (694.2 to 981.9)	446.6 (333.2 to 586.7)	23.8 (10.5 to 43.4)	5.9 (4.3 to 9.0)
Macedonia	2047	502.8 (434.5 to 580.8)	317.9 (268.8 to 381.1)	173.3 (129.5 to 227.2)	9.2 (4.1 to 16.9)	2.4 (1.7 to 3.7)	1310.3 (1132.4 to 1513.6)	828.4 (700.6 to 993.1)	451.7 (337.6 to 592.0)	24.1 (10.6 to 44.0)	6.1 (4.4 to 9.7)
Macedonia	2048	507.2 (437.2 to 583.7)	320.2 (270.8 to 384.4)	175.2 (130.9 to 229.9)	9.3 (4.1 to 17.1)	2.5 (1.8 to 3.9)	1321.9 (1139.5 to 1521.2)	834.4 (705.6 to 1001.7)	456.7 (341.3 to 599.1)	24.3 (10.7 to 44.6)	6.4 (4.6 to 10.2)
Macedonia	2049	511.7 (441.0 to 590.1)	322.5 (271.2 to 387.7)	177.2 (132.3 to 232.5)	9.4 (4.1 to 17.3)	2.6 (1.8 to 4.1)	1333.5 (1149.2 to 1537.8)	840.5 (706.7 to 1010.5)	461.7 (344.7 to 605.9)	24.6 (10.8 to 45.1)	6.7 (4.8 to 10.7)
Macedonia	2050	516.2 (444.4 to 596.7)	324.8 (271.8 to 390.9)	179.1 (133.5 to 235.8)	9.6 (4.2 to 17.5)	2.7 (1.9 to 4.4)	1345.2 (1158.1 to 1555.1)	846.5 (708.4 to 1018.7)	466.8 (348.0 to 614.5)	24.9 (10.9 to 45.7)	7.0 (4.9 to 11.4)

		2018 US Dollars per capita					2018 PPP per capita				
Country	Year	Health spending	Government health spending	Out-of-pocket health spending	Prepaid private health spending	Development assistance for health	Health spending	Government health spending	Out-of-pocket health spending	Prepaid private health spending	Development assistance for health
Madagascar	1995	20.9 (17.3 to 24.9)	7.2 (5.5 to 9.3)	8.8 (6.1 to 12.2)	1.9 (0.9 to 3.5)	3.0 (3.0 to 3.0)	73.3 (60.6 to 87.4)	25.2 (19.4 to 32.5)	30.9 (21.3 to 42.7)	6.6 (3.1 to 12.1)	10.6 (10.6 to 10.6)
Madagascar	1996	21.7 (18.2 to 25.6)	7.5 (5.8 to 9.7)	8.8 (6.0 to 12.0)	1.9 (0.9 to 3.6)	3.5 (3.5 to 3.5)	76.0 (63.7 to 89.9)	26.3 (20.2 to 34.0)	30.8 (21.1 to 42.1)	6.6 (3.1 to 12.5)	12.3 (12.3 to 12.3)
Madagascar	1997	21.0 (17.4 to 24.9)	7.9 (6.0 to 10.1)	8.8 (6.1 to 11.9)	1.9 (0.9 to 3.6)	2.4 (2.4 to 2.4)	73.6 (60.9 to 87.3)	27.6 (21.1 to 35.2)	30.7 (21.4 to 41.9)	6.7 (3.2 to 12.8)	8.6 (8.6 to 8.6)
Madagascar	1998	22.5 (19.1 to 26.8)	8.1 (6.2 to 10.5)	8.7 (6.0 to 12.2)	1.9 (0.9 to 3.7)	3.8 (3.8 to 3.8)	78.9 (66.9 to 93.9)	28.4 (21.9 to 36.8)	30.4 (21.1 to 42.6)	6.7 (3.3 to 13.1)	13.3 (13.3 to 13.3)
Madagascar	1999	21.4 (18.0 to 25.5)	8.6 (6.6 to 11.0)	8.7 (6.0 to 12.0)	1.9 (0.9 to 3.8)	2.2 (2.2 to 2.2)	74.9 (62.9 to 89.5)	30.0 (23.1 to 38.5)	30.4 (21.2 to 42.2)	6.8 (3.2 to 13.3)	7.7 (7.7 to 7.7)
Madagascar	2000	22.4 (18.6 to 26.7)	9.4 (7.2 to 12.1)	8.8 (6.0 to 12.3)	2.0 (1.0 to 3.9)	2.1 (2.1 to 2.1)	78.4 (65.2 to 93.5)	33.0 (25.3 to 42.3)	30.9 (21.2 to 43.1)	7.1 (3.4 to 13.8)	7.4 (7.4 to 7.4)
Madagascar	2001	23.2 (19.3 to 27.7)	9.9 (7.6 to 12.6)	8.8 (6.2 to 12.4)	2.1 (1.0 to 4.0)	2.3 (2.3 to 2.3)	81.2 (67.7 to 97.0)	34.8 (26.7 to 44.3)	30.9 (21.6 to 43.4)	7.2 (3.4 to 14.0)	8.2 (8.2 to 8.2)
Madagascar	2002	20.4 (16.9 to 24.6)	8.5 (6.5 to 10.8)	8.2 (5.7 to 11.5)	1.8 (0.8 to 3.4)	2.0 (2.0 to 2.0)	71.6 (59.3 to 86.1)	29.9 (22.8 to 37.8)	28.6 (20.0 to 40.3)	6.3 (2.9 to 12.1)	6.9 (6.9 to 6.9)
Madagascar	2003	21.9 (18.4 to 26.0)	8.4 (6.4 to 10.6)	8.3 (5.8 to 11.9)	1.8 (0.9 to 3.5)	3.4 (3.4 to 3.4)	76.9 (64.6 to 91.1)	29.3 (22.4 to 37.2)	29.2 (20.4 to 41.7)	6.4 (3.0 to 12.3)	12.0 (12.0 to 12.0)
Madagascar	2004	23.1 (19.5 to 27.3)	8.3 (6.4 to 10.7)	8.4 (5.9 to 12.0)	1.8 (0.9 to 3.6)	4.6 (4.6 to 4.6)	81.0 (68.3 to 95.8)	29.2 (22.3 to 37.4)	29.3 (20.6 to 42.1)	6.5 (3.0 to 12.6)	16.0 (16.0 to 16.0)
Madagascar	2005	23.7 (20.0 to 28.0)	8.4 (6.4 to 10.7)	8.5 (6.0 to 12.1)	1.9 (0.9 to 3.8)	4.9 (4.9 to 4.9)	83.0 (70.0 to 98.1)	29.3 (22.3 to 37.5)	29.9 (21.0 to 42.3)	6.6 (3.1 to 13.3)	17.2 (17.2 to 17.2)
Madagascar	2006	22.2 (18.6 to 26.3)	8.1 (6.2 to 10.2)	8.3 (5.8 to 11.7)	1.9 (0.9 to 3.8)	3.9 (3.9 to 3.9)	77.8 (65.1 to 92.1)	28.3 (21.6 to 35.8)	29.2 (20.2 to 41.1)	6.6 (3.1 to 13.3)	13.6 (13.6 to 13.6)
Madagascar	2007	22.9 (19.2 to 27.0)	8.1 (6.2 to 10.3)	8.3 (5.8 to 11.8)	2.0 (0.9 to 3.9)	4.5 (4.5 to 4.5)	80.3 (67.5 to 94.8)	28.4 (21.7 to 36.0)	29.3 (20.2 to 41.3)	6.9 (3.2 to 13.5)	15.7 (15.7 to 15.7)
Madagascar	2008	22.7 (19.2 to 26.8)	7.5 (5.7 to 9.6)	8.3 (5.7 to 11.7)	2.0 (0.9 to 3.7)	4.9 (4.9 to 4.9)	79.5 (67.3 to 93.9)	26.2 (19.9 to 33.5)	29.1 (20.0 to 41.1)	6.8 (3.3 to 12.8)	17.3 (17.3 to 17.3)
Madagascar	2009	19.7 (16.3 to 23.5)	6.7 (5.2 to 8.6)	7.9 (5.5 to 11.1)	1.8 (0.9 to 3.3)	3.3 (3.3 to 3.3)	68.9 (57.1 to 82.3)	23.5 (18.2 to 30.1)	27.6 (19.1 to 38.8)	6.3 (3.1 to 11.6)	11.4 (11.4 to 11.4)
Madagascar	2010	23.0 (19.8 to 26.9)	7.3 (5.6 to 9.3)	7.6 (5.3 to 10.7)	1.8 (0.9 to 3.3)	6.3 (6.3 to 6.3)	80.7 (69.3 to 94.2)	25.5 (19.5 to 32.7)	26.8 (18.6 to 37.4)	6.3 (3.0 to 11.7)	22.1 (22.1 to 22.1)
Madagascar	2011	20.5 (17.2 to 24.1)	7.7 (5.9 to 9.9)	7.1 (4.9 to 10.0)	1.8 (0.9 to 3.3)	3.8 (3.8 to 3.8)	71.8 (60.3 to 84.4)	27.0 (20.8 to 34.6)	25.1 (17.3 to 34.9)	6.3 (3.0 to 11.6)	13.5 (13.5 to 13.5)
Madagascar	2012	19.1 (16.1 to 22.5)	7.1 (5.4 to 9.1)	6.5 (4.4 to 9.1)	1.6 (0.8 to 3.0)	3.9 (3.9 to 3.9)	67.1 (56.4 to 78.8)	24.8 (18.9 to 31.8)	22.8 (15.5 to 32.0)	5.7 (2.7 to 10.5)	13.8 (13.8 to 13.8)
Madagascar	2013	20.1 (17.1 to 23.4)	7.3 (5.6 to 9.5)	6.2 (4.2 to 8.7)	1.6 (0.8 to 2.9)	4.9 (4.9 to 4.9)	70.4 (59.9 to 81.9)	25.7 (19.5 to 33.3)	21.8 (14.9 to 30.4)	5.6 (2.7 to 10.2)	17.3 (17.3 to 17.3)
Madagascar	2014	20.9 (17.9 to 24.6)	8.5 (6.5 to 11.1)	6.2 (4.2 to 8.5)	1.6 (0.8 to 3.1)	4.5 (4.5 to 4.5)	73.3 (62.8 to 86.2)	29.9 (22.7 to 38.8)	21.8 (14.7 to 29.9)	5.8 (2.7 to 10.9)	15.8 (15.8 to 15.8)
Madagascar	2015	21.6 (18.5 to 25.4)	9.6 (7.3 to 12.4)	6.2 (4.2 to 8.5)	1.7 (0.8 to 3.1)	4.2 (4.2 to 4.2)	75.7 (64.8 to 89.0)	33.5 (25.4 to 43.6)	21.7 (14.6 to 29.9)	5.8 (2.7 to 11.0)	14.6 (14.6 to 14.6)

		2018 US Dollars per capita					2018 PPP per capita				
Country	Year	Health spending	Government health spending	Out-of-pocket health spending	Prepaid private health spending	Development assistance for health	Health spending	Government health spending	Out-of-pocket health spending	Prepaid private health spending	Development assistance for health
Madagascar	2016	23.0 (19.5 to 26.9)	10.7 (8.1 to 14.0)	6.2 (4.2 to 8.7)	1.7 (0.8 to 3.0)	4.4 (4.4 to 4.4)	80.5 (68.4 to 94.2)	37.6 (28.4 to 49.0)	21.9 (14.6 to 30.4)	5.8 (2.7 to 10.6)	15.3 (15.3 to 15.3)
Madagascar	2017	23.4 (19.4 to 28.2)	12.1 (9.1 to 15.8)	6.4 (4.3 to 8.8)	1.8 (0.8 to 3.2)	3.1 (3.1 to 3.1)	81.9 (68.1 to 98.8)	42.4 (32.0 to 55.3)	22.3 (14.9 to 31.0)	6.1 (2.8 to 11.3)	11.0 (11.0 to 11.0)
Madagascar	2018	23.4 (19.5 to 27.8)	12.3 (9.3 to 16.1)	6.4 (4.3 to 8.9)	1.8 (0.8 to 3.2)	2.9 (2.9 to 2.9)	82.0 (68.4 to 97.3)	43.2 (32.6 to 56.3)	22.4 (14.9 to 31.2)	6.2 (2.9 to 11.4)	10.2 (10.1 to 10.2)
Madagascar	2019	23.7 (19.7 to 28.7)	12.6 (9.5 to 16.4)	6.4 (4.3 to 9.0)	1.8 (0.8 to 3.3)	2.9 (2.7 to 3.0)	83.1 (69.0 to 100.5)	44.2 (33.4 to 57.6)	22.6 (15.0 to 31.5)	6.3 (2.9 to 11.6)	10.0 (9.4 to 10.6)
Madagascar	2020	24.1 (20.1 to 28.6)	12.9 (9.7 to 16.8)	6.5 (4.3 to 9.0)	1.8 (0.8 to 3.4)	2.8 (2.6 to 3.1)	84.3 (70.5 to 100.1)	45.1 (34.1 to 58.8)	22.8 (15.1 to 31.7)	6.4 (3.0 to 11.8)	10.0 (9.2 to 10.8)
Madagascar	2021	24.4 (20.3 to 29.4)	13.1 (9.9 to 17.0)	6.6 (4.4 to 9.1)	1.9 (0.9 to 3.4)	2.8 (2.6 to 3.1)	85.5 (71.0 to 103.1)	46.0 (34.8 to 59.8)	23.0 (15.3 to 32.0)	6.6 (3.0 to 12.0)	10.0 (9.0 to 11.0)
Madagascar	2022	24.8 (20.7 to 29.5)	13.4 (10.1 to 17.4)	6.6 (4.4 to 9.2)	1.9 (0.9 to 3.5)	2.9 (2.5 to 3.2)	86.8 (72.5 to 103.3)	46.9 (35.5 to 60.9)	23.2 (15.5 to 32.2)	6.7 (3.1 to 12.3)	10.0 (8.9 to 11.2)
Madagascar	2023	25.1 (20.9 to 30.4)	13.6 (10.3 to 17.7)	6.7 (4.5 to 9.3)	1.9 (0.9 to 3.6)	2.9 (2.5 to 3.3)	88.1 (73.3 to 106.7)	47.8 (36.2 to 61.9)	23.4 (15.7 to 32.5)	6.8 (3.1 to 12.5)	10.0 (8.7 to 11.4)
Madagascar	2024	25.5 (21.3 to 30.4)	13.9 (10.5 to 18.0)	6.7 (4.5 to 9.4)	2.0 (0.9 to 3.6)	2.9 (2.5 to 3.3)	89.4 (74.6 to 106.4)	48.8 (36.9 to 63.0)	23.7 (15.8 to 32.9)	6.9 (3.2 to 12.7)	10.1 (8.7 to 11.6)
Madagascar	2025	25.9 (21.7 to 31.4)	14.2 (10.7 to 18.3)	6.8 (4.5 to 9.5)	2.0 (0.9 to 3.7)	2.9 (2.4 to 3.4)	90.6 (75.9 to 110.2)	49.6 (37.6 to 64.2)	23.9 (15.9 to 33.2)	7.0 (3.3 to 12.9)	10.1 (8.6 to 11.8)
Madagascar	2026	26.2 (21.8 to 31.2)	14.4 (10.9 to 18.6)	6.9 (4.6 to 9.6)	2.0 (0.9 to 3.8)	2.9 (2.4 to 3.4)	91.7 (76.3 to 109.3)	50.4 (38.2 to 65.3)	24.1 (16.1 to 33.6)	7.2 (3.3 to 13.2)	10.1 (8.5 to 11.9)
Madagascar	2027	26.5 (22.1 to 32.2)	14.6 (11.1 to 18.9)	6.9 (4.6 to 9.7)	2.1 (1.0 to 3.8)	2.9 (2.4 to 3.4)	92.9 (77.4 to 112.9)	51.2 (38.8 to 66.4)	24.3 (16.2 to 33.9)	7.3 (3.4 to 13.4)	10.1 (8.5 to 12.1)
Madagascar	2028	26.9 (22.4 to 32.1)	14.9 (11.3 to 19.3)	7.0 (4.7 to 9.8)	2.1 (1.0 to 3.9)	2.9 (2.4 to 3.5)	94.2 (78.4 to 112.5)	52.1 (39.5 to 67.7)	24.5 (16.3 to 34.2)	7.4 (3.4 to 13.6)	10.2 (8.5 to 12.4)
Madagascar	2029	27.2 (22.7 to 33.0)	15.1 (11.5 to 19.7)	7.1 (4.7 to 9.9)	2.1 (1.0 to 4.0)	2.9 (2.4 to 3.6)	95.5 (79.7 to 115.8)	53.0 (40.2 to 69.0)	24.7 (16.5 to 34.5)	7.5 (3.5 to 13.9)	10.3 (8.4 to 12.6)
Madagascar	2030	27.6 (23.0 to 33.1)	15.4 (11.7 to 20.0)	7.1 (4.7 to 9.9)	2.2 (1.0 to 4.0)	2.9 (2.4 to 3.6)	96.8 (80.6 to 116.0)	53.9 (40.9 to 70.3)	24.9 (16.6 to 34.8)	7.7 (3.6 to 14.2)	10.3 (8.4 to 12.7)
Madagascar	2031	28.0 (23.3 to 34.0)	15.6 (11.9 to 20.4)	7.2 (4.8 to 10.0)	2.2 (1.0 to 4.1)	3.0 (2.4 to 3.7)	98.1 (81.7 to 119.1)	54.8 (41.5 to 71.5)	25.1 (16.8 to 35.2)	7.8 (3.6 to 14.4)	10.4 (8.4 to 13.0)
Madagascar	2032	28.3 (23.6 to 34.0)	15.9 (12.1 to 20.7)	7.2 (4.8 to 10.1)	2.3 (1.1 to 4.2)	3.0 (2.4 to 3.8)	99.4 (82.7 to 119.1)	55.7 (42.3 to 72.6)	25.3 (16.9 to 35.4)	7.9 (3.7 to 14.7)	10.4 (8.4 to 13.2)
Madagascar	2033	28.7 (23.7 to 35.2)	16.2 (12.3 to 21.1)	7.3 (4.9 to 10.2)	2.3 (1.1 to 4.3)	3.0 (2.4 to 3.9)	100.7 (83.2 to 123.4)	56.6 (43.0 to 73.9)	25.5 (17.0 to 35.7)	8.0 (3.7 to 15.0)	10.5 (8.4 to 13.5)
Madagascar	2034	29.1 (24.0 to 34.8)	16.4 (12.5 to 21.5)	7.3 (4.9 to 10.3)	2.3 (1.1 to 4.4)	3.0 (2.4 to 3.9)	102.1 (84.1 to 122.2)	57.6 (43.7 to 75.2)	25.8 (17.1 to 36.0)	8.2 (3.8 to 15.3)	10.6 (8.3 to 13.6)
Madagascar	2035	29.5 (24.4 to 36.2)	16.7 (12.6 to 21.9)	7.4 (4.9 to 10.3)	2.4 (1.1 to 4.4)	3.0 (2.4 to 4.0)	103.5 (85.5 to 127.0)	58.5 (44.3 to 76.6)	26.0 (17.3 to 36.3)	8.3 (3.9 to 15.5)	10.7 (8.3 to 14.0)
Madagascar	2036	29.9 (24.6 to 35.7)	17.0 (12.8 to 22.2)	7.5 (5.0 to 10.4)	2.4 (1.1 to 4.5)	3.1 (2.4 to 4.1)	104.9 (86.2 to 125.3)	59.5 (44.9 to 77.7)	26.2 (17.4 to 36.5)	8.4 (3.9 to 15.8)	10.8 (8.3 to 14.5)

		2018 US Dollars per capita					2018 PPP per capita				
Country	Year	Health spending	Government health spending	Out-of-pocket health spending	Prepaid private health spending	Development assistance for health	Health spending	Government health spending	Out-of-pocket health spending	Prepaid private health spending	Development assistance for health
Madagascar	2037	30.3 (24.9 to 37.2)	17.2 (13.0 to 22.5)	7.5 (5.0 to 10.5)	2.4 (1.1 to 4.6)	3.1 (2.4 to 4.1)	106.2 (87.4 to 130.6)	60.4 (45.7 to 79.0)	26.4 (17.5 to 36.7)	8.6 (4.0 to 16.1)	10.8 (8.3 to 14.5)
Madagascar	2038	30.7 (25.3 to 36.9)	17.5 (13.2 to 22.9)	7.6 (5.0 to 10.6)	2.5 (1.1 to 4.7)	3.1 (2.4 to 4.3)	107.6 (88.5 to 129.2)	61.4 (46.3 to 80.3)	26.6 (17.7 to 37.1)	8.7 (4.0 to 16.4)	10.9 (8.3 to 14.9)
Madagascar	2039	31.1 (25.6 to 38.3)	17.8 (13.4 to 23.3)	7.7 (5.1 to 10.7)	2.5 (1.2 to 4.8)	3.1 (2.4 to 4.3)	109.1 (89.9 to 134.2)	62.4 (47.1 to 81.7)	26.8 (17.8 to 37.4)	8.9 (4.1 to 16.7)	11.0 (8.4 to 15.0)
Madagascar	2040	31.5 (26.0 to 37.8)	18.1 (13.7 to 23.7)	7.7 (5.1 to 10.7)	2.6 (1.2 to 4.9)	3.2 (2.4 to 4.3)	110.6 (91.0 to 132.6)	63.4 (47.9 to 83.0)	27.1 (18.0 to 37.7)	9.0 (4.1 to 17.0)	11.1 (8.5 to 15.2)
Madagascar	2041	32.0 (26.3 to 39.2)	18.4 (13.9 to 24.1)	7.8 (5.2 to 10.8)	2.6 (1.2 to 4.9)	3.2 (2.4 to 4.5)	112.0 (92.2 to 137.5)	64.4 (48.7 to 84.4)	27.3 (18.1 to 37.9)	9.2 (4.2 to 17.3)	11.2 (8.4 to 15.8)
Madagascar	2042	32.4 (26.6 to 39.1)	18.7 (14.1 to 24.5)	7.8 (5.2 to 10.9)	2.7 (1.2 to 5.0)	3.2 (2.4 to 4.7)	113.6 (93.2 to 136.9)	65.4 (49.5 to 85.9)	27.5 (18.3 to 38.2)	9.3 (4.3 to 17.7)	11.4 (8.4 to 16.5)
Madagascar	2043	32.9 (27.1 to 40.3)	19.0 (14.3 to 24.9)	7.9 (5.3 to 11.0)	2.7 (1.2 to 5.1)	3.3 (2.4 to 4.8)	115.2 (94.9 to 141.3)	66.5 (50.3 to 87.4)	27.7 (18.4 to 38.5)	9.5 (4.4 to 17.9)	11.5 (8.5 to 16.9)
Madagascar	2044	33.3 (27.3 to 40.2)	19.3 (14.5 to 25.3)	8.0 (5.3 to 11.1)	2.7 (1.3 to 5.2)	3.3 (2.4 to 4.8)	116.7 (95.8 to 141.1)	67.5 (51.0 to 88.7)	28.0 (18.6 to 38.9)	9.6 (4.4 to 18.2)	11.6 (8.6 to 17.0)
Madagascar	2045	33.8 (27.7 to 41.7)	19.6 (14.7 to 25.8)	8.0 (5.3 to 11.2)	2.8 (1.3 to 5.3)	3.4 (2.4 to 5.1)	118.4 (97.0 to 146.1)	68.6 (51.7 to 90.3)	28.2 (18.7 to 39.2)	9.8 (4.5 to 18.4)	11.7 (8.5 to 17.7)
Madagascar	2046	34.2 (28.1 to 41.5)	19.9 (14.9 to 26.2)	8.1 (5.4 to 11.3)	2.8 (1.3 to 5.4)	3.4 (2.4 to 5.1)	120.0 (98.5 to 145.6)	69.7 (52.3 to 91.9)	28.4 (18.8 to 39.6)	9.9 (4.5 to 18.8)	11.9 (8.6 to 18.0)
Madagascar	2047	34.7 (28.4 to 42.9)	20.2 (15.1 to 26.7)	8.2 (5.4 to 11.4)	2.9 (1.3 to 5.5)	3.4 (2.5 to 5.4)	121.7 (99.7 to 150.5)	70.9 (53.0 to 93.6)	28.7 (19.0 to 39.9)	10.1 (4.6 to 19.1)	12.1 (8.7 to 19.0)
Madagascar	2048	35.3 (28.9 to 43.0)	20.6 (15.4 to 27.3)	8.3 (5.5 to 11.5)	2.9 (1.3 to 5.6)	3.5 (2.5 to 5.6)	123.7 (101.4 to 150.6)	72.2 (53.8 to 95.5)	28.9 (19.2 to 40.3)	10.3 (4.7 to 19.5)	12.2 (8.8 to 19.7)
Madagascar	2049	35.8 (29.2 to 44.9)	21.0 (15.6 to 27.8)	8.3 (5.5 to 11.6)	3.0 (1.4 to 5.7)	3.5 (2.5 to 5.7)	125.6 (102.4 to 157.2)	73.5 (54.7 to 97.4)	29.2 (19.3 to 40.7)	10.5 (4.8 to 19.9)	12.4 (8.8 to 19.9)
Madagascar	2050	36.4 (29.7 to 44.5)	21.4 (15.9 to 28.3)	8.4 (5.6 to 11.7)	3.0 (1.4 to 5.8)	3.6 (2.5 to 5.8)	127.6 (104.0 to 155.8)	74.9 (55.7 to 99.3)	29.5 (19.5 to 41.1)	10.6 (4.8 to 20.3)	12.6 (8.9 to 20.4)
Malawi	1995	13.0 (11.5 to 14.9)	4.2 (3.2 to 5.5)	3.0 (2.1 to 4.2)	1.0 (0.5 to 1.9)	4.8 (4.8 to 4.8)	47.1 (41.5 to 54.0)	15.2 (11.5 to 20.0)	10.9 (7.5 to 15.3)	3.6 (1.7 to 6.8)	17.4 (17.4 to 17.4)
Malawi	1996	14.6 (13.0 to 16.5)	4.1 (3.2 to 5.5)	3.1 (2.1 to 4.4)	1.0 (0.5 to 1.8)	6.4 (6.4 to 6.4)	53.0 (47.2 to 59.6)	15.0 (11.5 to 20.0)	11.2 (7.7 to 15.9)	3.6 (1.7 to 6.7)	23.2 (23.2 to 23.2)
Malawi	1997	16.5 (14.9 to 18.4)	4.3 (3.2 to 5.6)	3.1 (2.2 to 4.5)	1.0 (0.5 to 1.8)	8.2 (8.2 to 8.2)	59.9 (54.1 to 66.5)	15.4 (11.7 to 20.2)	11.3 (7.9 to 16.2)	3.6 (1.7 to 6.6)	29.6 (29.6 to 29.6)
Malawi	1998	14.3 (12.8 to 16.0)	4.2 (3.2 to 5.4)	3.0 (2.1 to 4.3)	0.9 (0.4 to 1.7)	6.2 (6.2 to 6.2)	51.8 (46.4 to 57.8)	15.2 (11.5 to 19.7)	10.8 (7.6 to 15.6)	3.3 (1.6 to 6.2)	22.5 (22.5 to 22.5)
Malawi	1999	19.1 (17.6 to 20.8)	4.3 (3.3 to 5.6)	2.9 (2.0 to 4.2)	0.8 (0.4 to 1.6)	11.0 (11.0 to 11.0)	69.2 (63.7 to 75.4)	15.7 (11.9 to 20.2)	10.5 (7.4 to 15.2)	3.0 (1.4 to 5.7)	40.0 (40.0 to 40.0)
Malawi	2000	17.9 (16.5 to 19.7)	4.6 (3.5 to 6.0)	2.6 (1.8 to 3.8)	0.7 (0.3 to 1.3)	10.0 (10.0 to 10.0)	65.0 (59.6 to 71.2)	16.8 (12.6 to 21.9)	9.5 (6.6 to 13.8)	2.6 (1.2 to 4.8)	36.1 (36.1 to 36.1)
Malawi	2001	14.7 (13.5 to 16.2)	4.2 (3.1 to 5.4)	2.2 (1.5 to 3.1)	0.6 (0.3 to 1.1)	7.8 (7.8 to 7.8)	53.3 (48.7 to 58.6)	15.0 (11.4 to 19.6)	7.9 (5.5 to 11.4)	2.1 (1.0 to 3.9)	28.2 (28.2 to 28.2)

		2018 US Dollars per capita					2018 PPP per capita				
Country	Year	Health spending	Government health spending	Out-of-pocket health spending	Prepaid private health spending	Development assistance for health	Health spending	Government health spending	Out-of-pocket health spending	Prepaid private health spending	Development assistance for health
Malawi	2002	12.9 (11.7 to 14.2)	3.9 (3.0 to 5.1)	1.8 (1.2 to 2.5)	0.5 (0.2 to 1.0)	6.7 (6.7 to 6.7)	46.7 (42.5 to 51.4)	14.2 (10.8 to 18.4)	6.4 (4.5 to 9.2)	1.9 (0.9 to 3.6)	24.2 (24.2 to 24.2)
Malawi	2003	14.6 (13.4 to 15.9)	3.9 (2.9 to 5.0)	1.7 (1.1 to 2.4)	0.6 (0.3 to 1.3)	8.4 (8.4 to 8.4)	52.8 (48.5 to 57.6)	14.0 (10.5 to 18.3)	6.0 (4.1 to 8.7)	2.3 (1.1 to 4.5)	30.5 (30.5 to 30.5)
Malawi	2004	17.2 (16.0 to 18.5)	3.7 (2.8 to 4.9)	1.6 (1.1 to 2.3)	0.7 (0.3 to 1.4)	11.1 (11.1 to 11.1)	62.2 (58.0 to 67.1)	13.5 (10.1 to 17.7)	5.9 (4.0 to 8.5)	2.5 (1.2 to 4.9)	40.3 (40.3 to 40.3)
Malawi	2005	17.9 (16.6 to 19.3)	3.8 (2.8 to 4.9)	1.7 (1.2 to 2.4)	0.9 (0.4 to 1.7)	11.5 (11.5 to 11.5)	64.8 (60.2 to 70.0)	13.6 (10.2 to 17.7)	6.1 (4.3 to 8.8)	3.2 (1.5 to 6.3)	41.8 (41.8 to 41.8)
Malawi	2006	22.8 (21.4 to 24.5)	3.7 (2.8 to 4.9)	1.9 (1.3 to 2.7)	1.3 (0.6 to 2.6)	15.9 (15.9 to 15.9)	82.7 (77.7 to 88.8)	13.5 (10.2 to 17.7)	6.8 (4.7 to 9.8)	4.7 (2.2 to 9.2)	57.6 (57.6 to 57.6)
Malawi	2007	28.6 (27.0 to 30.7)	4.0 (3.0 to 5.3)	2.3 (1.6 to 3.2)	1.7 (0.8 to 3.5)	20.6 (20.6 to 20.6)	103.7 (97.8 to 111.1)	14.6 (11.0 to 19.1)	8.3 (5.8 to 11.6)	6.1 (2.9 to 12.6)	74.7 (74.7 to 74.7)
Malawi	2008	33.1 (31.1 to 35.5)	4.8 (3.6 to 6.3)	2.6 (1.8 to 3.7)	2.0 (0.9 to 4.1)	23.7 (23.7 to 23.7)	120.0 (112.8 to 128.5)	17.3 (13.2 to 22.8)	9.4 (6.5 to 13.3)	7.4 (3.4 to 14.7)	85.8 (85.8 to 85.8)
Malawi	2009	30.0 (28.1 to 32.3)	4.9 (3.7 to 6.4)	2.7 (1.9 to 3.8)	1.9 (0.9 to 3.6)	20.5 (20.5 to 20.5)	108.5 (101.7 to 117.0)	17.8 (13.5 to 23.2)	9.8 (6.8 to 13.8)	6.7 (3.1 to 13.1)	74.2 (74.2 to 74.2)
Malawi	2010	26.4 (24.5 to 28.7)	5.3 (4.0 to 6.9)	2.7 (1.9 to 3.7)	1.5 (0.7 to 2.9)	16.9 (16.9 to 16.9)	95.5 (88.9 to 103.8)	19.1 (14.5 to 25.1)	9.7 (6.7 to 13.5)	5.4 (2.5 to 10.6)	61.3 (61.3 to 61.3)
Malawi	2011	33.4 (31.6 to 35.7)	5.6 (4.2 to 7.4)	2.7 (1.8 to 3.7)	1.4 (0.6 to 2.7)	23.7 (23.7 to 23.7)	121.0 (114.4 to 129.2)	20.3 (15.4 to 26.9)	9.7 (6.7 to 13.4)	5.0 (2.3 to 9.9)	86.0 (86.0 to 86.0)
Malawi	2012	34.8 (32.9 to 36.9)	5.7 (4.4 to 7.5)	2.8 (1.9 to 3.9)	1.2 (0.6 to 2.4)	25.0 (25.0 to 25.0)	125.8 (119.2 to 133.6)	20.8 (15.9 to 27.3)	10.1 (7.0 to 14.0)	4.4 (2.1 to 8.6)	90.6 (90.6 to 90.6)
Malawi	2013	36.4 (34.4 to 38.9)	6.6 (5.1 to 8.6)	2.9 (2.0 to 4.0)	1.3 (0.6 to 2.5)	25.6 (25.6 to 25.6)	131.9 (124.5 to 140.9)	24.0 (18.4 to 31.0)	10.5 (7.3 to 14.5)	4.8 (2.2 to 9.0)	92.6 (92.6 to 92.6)
Malawi	2014	34.7 (32.3 to 37.5)	7.7 (5.9 to 9.8)	3.1 (2.2 to 4.3)	1.6 (0.8 to 3.0)	22.2 (22.2 to 22.2)	125.5 (117.1 to 135.7)	27.8 (21.3 to 35.6)	11.4 (8.0 to 15.7)	5.8 (2.8 to 10.7)	80.5 (80.5 to 80.5)
Malawi	2015	37.6 (34.9 to 40.8)	8.6 (6.5 to 10.9)	3.5 (2.5 to 4.9)	2.0 (1.0 to 3.9)	23.4 (23.4 to 23.4)	136.1 (126.4 to 147.8)	31.1 (23.5 to 39.4)	12.8 (8.9 to 17.7)	7.3 (3.5 to 14.0)	84.9 (84.9 to 84.9)
Malawi	2016	38.9 (35.8 to 42.2)	9.1 (6.9 to 11.6)	3.8 (2.6 to 5.3)	2.3 (1.1 to 4.4)	23.7 (23.7 to 23.7)	140.7 (129.7 to 152.8)	33.1 (24.9 to 42.1)	13.7 (9.6 to 19.1)	8.3 (4.0 to 16.1)	85.6 (85.6 to 85.6)
Malawi	2017	42.4 (39.3 to 45.7)	9.5 (7.1 to 12.0)	3.9 (2.7 to 5.4)	2.3 (1.1 to 4.5)	26.8 (26.8 to 26.8)	153.5 (142.4 to 165.4)	34.3 (25.9 to 43.6)	14.0 (9.7 to 19.4)	8.3 (4.0 to 16.2)	97.0 (97.0 to 97.0)
Malawi	2018	40.7 (37.6 to 44.3)	9.6 (7.3 to 12.2)	3.9 (2.7 to 5.4)	2.3 (1.1 to 4.6)	24.9 (24.9 to 24.9)	147.5 (136.0 to 160.3)	34.8 (26.3 to 44.2)	14.1 (9.8 to 19.4)	8.4 (4.1 to 16.5)	90.2 (90.1 to 90.3)
Malawi	2019	40.8 (37.1 to 44.7)	9.7 (7.4 to 12.4)	3.9 (2.7 to 5.4)	2.4 (1.2 to 4.6)	24.8 (23.1 to 26.2)	147.7 (134.3 to 162.0)	35.3 (26.7 to 44.7)	14.2 (9.8 to 19.7)	8.6 (4.2 to 16.7)	89.6 (83.6 to 94.8)
Malawi	2020	41.0 (37.3 to 45.5)	9.9 (7.5 to 12.6)	4.0 (2.7 to 5.5)	2.4 (1.2 to 4.7)	24.8 (22.7 to 26.8)	148.6 (135.1 to 164.9)	35.8 (27.0 to 45.5)	14.3 (9.9 to 19.9)	8.7 (4.2 to 17.0)	89.7 (82.3 to 96.9)
Malawi	2021	41.4 (37.3 to 46.0)	10.1 (7.6 to 12.8)	4.0 (2.8 to 5.6)	2.5 (1.2 to 4.8)	24.9 (22.4 to 27.5)	150.0 (135.0 to 166.4)	36.4 (27.5 to 46.3)	14.5 (10.0 to 20.2)	8.9 (4.3 to 17.3)	90.2 (81.2 to 99.6)
Malawi	2022	41.9 (37.4 to 46.8)	10.2 (7.7 to 13.1)	4.1 (2.8 to 5.6)	2.5 (1.2 to 4.9)	25.1 (22.3 to 28.0)	151.8 (135.3 to 169.6)	37.1 (28.0 to 47.3)	14.7 (10.2 to 20.4)	9.1 (4.4 to 17.7)	90.9 (80.7 to 101.5)

Country	Year	2018 US Dollars per capita					2018 PPP per capita				
		Health spending	Government health spending	Out-of-pocket health spending	Prepaid private health spending	Development assistance for health	Health spending	Government health spending	Out-of-pocket health spending	Prepaid private health spending	Development assistance for health
Malawi	2023	42.4 (37.5 to 47.5)	10.4 (7.9 to 13.3)	4.1 (2.9 to 5.7)	2.6 (1.2 to 5.0)	25.3 (22.0 to 28.7)	153.6 (135.9 to 171.9)	37.8 (28.5 to 48.3)	14.9 (10.4 to 20.7)	9.3 (4.5 to 18.1)	91.6 (79.8 to 104.0)
Malawi	2024	42.9 (37.8 to 48.1)	10.7 (8.0 to 13.6)	4.2 (2.9 to 5.8)	2.6 (1.3 to 5.1)	25.5 (22.0 to 29.3)	155.4 (136.9 to 174.1)	38.6 (29.0 to 49.3)	15.1 (10.5 to 21.1)	9.5 (4.6 to 18.5)	92.2 (79.6 to 106.0)
Malawi	2025	43.3 (37.7 to 48.9)	10.9 (8.2 to 13.9)	4.2 (2.9 to 5.9)	2.7 (1.3 to 5.2)	25.5 (21.8 to 29.9)	156.9 (136.6 to 177.2)	39.4 (29.6 to 50.3)	15.3 (10.6 to 21.4)	9.7 (4.7 to 18.9)	92.5 (79.0 to 108.4)
Malawi	2026	43.8 (38.2 to 49.4)	11.1 (8.3 to 14.2)	4.3 (3.0 to 6.0)	2.7 (1.3 to 5.3)	25.6 (21.6 to 30.0)	158.5 (138.5 to 178.9)	40.2 (30.2 to 51.5)	15.6 (10.8 to 21.7)	9.9 (4.8 to 19.3)	92.7 (78.3 to 108.8)
Malawi	2027	44.2 (38.1 to 50.4)	11.3 (8.5 to 14.5)	4.4 (3.0 to 6.1)	2.8 (1.4 to 5.5)	25.7 (21.5 to 30.7)	160.1 (138.1 to 182.4)	41.0 (30.9 to 52.6)	15.8 (10.9 to 22.1)	10.1 (4.9 to 19.8)	93.1 (77.9 to 111.0)
Malawi	2028	44.7 (38.7 to 51.2)	11.6 (8.7 to 14.8)	4.4 (3.1 to 6.2)	2.9 (1.4 to 5.6)	25.8 (21.5 to 31.2)	161.8 (140.1 to 185.4)	41.9 (31.5 to 53.6)	16.0 (11.1 to 22.4)	10.4 (5.0 to 20.2)	93.6 (78.0 to 112.9)
Malawi	2029	45.2 (38.9 to 51.9)	11.8 (8.8 to 15.1)	4.5 (3.1 to 6.3)	2.9 (1.4 to 5.7)	26.0 (21.4 to 31.8)	163.6 (140.7 to 187.8)	42.7 (32.0 to 54.7)	16.3 (11.3 to 22.7)	10.6 (5.1 to 20.6)	94.1 (77.5 to 115.1)
Malawi	2030	45.7 (39.3 to 53.2)	12.0 (9.0 to 15.4)	4.6 (3.2 to 6.4)	3.0 (1.4 to 5.8)	26.1 (21.3 to 32.0)	165.4 (142.3 to 192.8)	43.5 (32.6 to 55.9)	16.5 (11.4 to 23.1)	10.8 (5.3 to 21.0)	94.6 (77.3 to 116.0)
Malawi	2031	46.2 (39.5 to 53.9)	12.2 (9.2 to 15.7)	4.6 (3.2 to 6.5)	3.0 (1.5 to 5.9)	26.3 (21.3 to 33.0)	167.1 (143.0 to 195.3)	44.3 (33.2 to 56.9)	16.7 (11.6 to 23.4)	11.0 (5.4 to 21.5)	95.1 (77.2 to 119.6)
Malawi	2032	46.7 (39.9 to 55.1)	12.5 (9.4 to 16.0)	4.7 (3.2 to 6.6)	3.1 (1.5 to 6.0)	26.4 (21.3 to 33.4)	169.0 (144.3 to 199.6)	45.1 (33.9 to 58.0)	16.9 (11.8 to 23.7)	11.2 (5.5 to 21.9)	95.7 (77.0 to 121.0)
Malawi	2033	47.2 (40.0 to 56.2)	12.7 (9.5 to 16.3)	4.7 (3.3 to 6.7)	3.2 (1.5 to 6.2)	26.6 (21.1 to 34.2)	171.0 (144.9 to 203.7)	45.9 (34.6 to 59.0)	17.1 (11.9 to 24.1)	11.5 (5.6 to 22.3)	96.4 (76.5 to 124.0)
Malawi	2034	47.8 (40.4 to 56.8)	12.9 (9.7 to 16.7)	4.8 (3.3 to 6.8)	3.2 (1.6 to 6.3)	26.9 (21.2 to 34.6)	173.1 (146.3 to 205.6)	46.8 (35.2 to 60.3)	17.4 (12.0 to 24.5)	11.7 (5.7 to 22.7)	97.3 (76.7 to 125.2)
Malawi	2035	48.4 (40.5 to 58.4)	13.1 (9.9 to 17.0)	4.9 (3.4 to 6.9)	3.3 (1.6 to 6.4)	27.1 (21.1 to 35.5)	175.3 (146.8 to 211.5)	47.6 (35.8 to 61.5)	17.6 (12.2 to 24.9)	11.9 (5.8 to 23.2)	98.2 (76.3 to 128.5)
Malawi	2036	49.0 (41.1 to 59.3)	13.4 (10.1 to 17.3)	4.9 (3.4 to 7.0)	3.4 (1.6 to 6.5)	27.4 (21.2 to 36.9)	177.4 (148.9 to 214.9)	48.4 (36.4 to 62.6)	17.8 (12.3 to 25.3)	12.1 (5.9 to 23.6)	99.1 (76.8 to 133.5)
Malawi	2037	49.6 (41.2 to 60.4)	13.6 (10.2 to 17.6)	5.0 (3.4 to 7.1)	3.4 (1.7 to 6.6)	27.6 (21.3 to 37.0)	179.5 (149.2 to 218.7)	49.2 (37.1 to 63.8)	18.0 (12.5 to 25.6)	12.4 (6.1 to 24.1)	99.9 (77.1 to 134.2)
Malawi	2038	50.2 (41.8 to 61.2)	13.8 (10.4 to 18.0)	5.0 (3.5 to 7.2)	3.5 (1.7 to 6.8)	27.8 (21.2 to 38.1)	181.8 (151.5 to 221.8)	50.1 (37.7 to 65.0)	18.2 (12.6 to 25.9)	12.6 (6.2 to 24.5)	100.8 (76.8 to 138.0)
Malawi	2039	50.8 (42.0 to 62.3)	14.1 (10.6 to 18.3)	5.1 (3.5 to 7.2)	3.6 (1.7 to 6.9)	28.1 (21.3 to 38.3)	184.0 (152.1 to 225.6)	51.0 (38.3 to 66.2)	18.5 (12.8 to 26.2)	12.9 (6.3 to 25.0)	101.7 (77.2 to 138.9)
Malawi	2040	51.5 (42.9 to 63.7)	14.3 (10.8 to 18.6)	5.2 (3.6 to 7.3)	3.6 (1.8 to 7.0)	28.4 (21.5 to 38.8)	186.5 (155.3 to 230.6)	51.9 (38.9 to 67.4)	18.7 (12.9 to 26.6)	13.1 (6.4 to 25.4)	102.7 (78.0 to 140.5)
Malawi	2041	52.2 (43.3 to 64.2)	14.6 (10.9 to 18.9)	5.2 (3.6 to 7.4)	3.7 (1.8 to 7.1)	28.7 (21.5 to 40.3)	189.0 (156.9 to 232.4)	52.8 (39.5 to 68.6)	18.9 (13.1 to 26.8)	13.4 (6.6 to 25.9)	103.9 (78.0 to 145.9)
Malawi	2042	52.9 (43.7 to 66.9)	14.8 (11.1 to 19.3)	5.3 (3.6 to 7.5)	3.8 (1.8 to 7.3)	29.1 (21.6 to 42.1)	191.6 (158.2 to 242.2)	53.7 (40.1 to 69.8)	19.1 (13.2 to 27.1)	13.6 (6.7 to 26.3)	105.3 (78.3 to 152.5)
Malawi	2043	53.7 (44.3 to 67.7)	15.1 (11.3 to 19.6)	5.3 (3.7 to 7.6)	3.8 (1.9 to 7.4)	29.4 (21.8 to 43.2)	194.3 (160.4 to 245.1)	54.6 (40.8 to 71.1)	19.3 (13.4 to 27.5)	13.9 (6.8 to 26.8)	106.5 (79.1 to 156.6)

		2018 US Dollars per capita					2018 PPP per capita				
Country	Year	Health spending	Government health spending	Out-of-pocket health spending	Prepaid private health spending	Development assistance for health	Health spending	Government health spending	Out-of-pocket health spending	Prepaid private health spending	Development assistance for health
Malawi	2044	54.4 (44.7 to 69.0)	15.3 (11.4 to 20.0)	5.4 (3.7 to 7.7)	3.9 (1.9 to 7.6)	29.8 (22.0 to 43.6)	197.2 (161.7 to 250.0)	55.5 (41.3 to 72.5)	19.6 (13.5 to 27.8)	14.1 (6.9 to 27.3)	108.0 (79.7 to 158.0)
Malawi	2045	55.3 (45.2 to 71.4)	15.6 (11.6 to 20.4)	5.5 (3.8 to 7.8)	4.0 (1.9 to 7.7)	30.3 (21.9 to 45.6)	200.1 (163.7 to 258.5)	56.4 (41.9 to 73.9)	19.8 (13.7 to 28.1)	14.4 (7.0 to 27.9)	109.6 (79.3 to 165.2)
Malawi	2046	56.1 (45.8 to 72.9)	15.8 (11.7 to 20.8)	5.5 (3.8 to 7.8)	4.0 (2.0 to 7.8)	30.7 (22.1 to 46.5)	203.1 (166.0 to 264.0)	57.3 (42.5 to 75.2)	20.0 (13.9 to 28.4)	14.6 (7.1 to 28.4)	111.2 (80.2 to 168.5)
Malawi	2047	57.0 (46.1 to 74.5)	16.1 (11.9 to 21.1)	5.6 (3.9 to 7.9)	4.1 (2.0 to 8.0)	31.2 (22.4 to 49.2)	206.4 (167.0 to 269.7)	58.3 (43.2 to 76.5)	20.2 (14.0 to 28.7)	14.9 (7.3 to 28.9)	112.9 (81.2 to 178.1)
Malawi	2048	58.0 (46.8 to 78.0)	16.4 (12.1 to 21.5)	5.7 (3.9 to 8.0)	4.2 (2.0 to 8.1)	31.7 (22.7 to 51.2)	209.9 (169.6 to 282.6)	59.4 (44.0 to 77.9)	20.5 (14.2 to 29.1)	15.2 (7.4 to 29.5)	114.9 (82.2 to 185.3)
Malawi	2049	58.9 (47.7 to 79.4)	16.7 (12.3 to 21.9)	5.7 (4.0 to 8.1)	4.3 (2.1 to 8.3)	32.3 (22.9 to 51.9)	213.4 (172.8 to 287.6)	60.4 (44.7 to 79.4)	20.7 (14.3 to 29.5)	15.5 (7.5 to 30.1)	116.8 (83.0 to 187.9)
Malawi	2050	60.0 (48.1 to 82.0)	17.0 (12.5 to 22.3)	5.8 (4.0 to 8.2)	4.3 (2.1 to 8.5)	32.9 (23.3 to 53.1)	217.2 (174.2 to 296.9)	61.4 (45.4 to 80.8)	20.9 (14.5 to 29.8)	15.7 (7.6 to 30.7)	119.1 (84.2 to 192.4)
Malaysia	1995	147.5 (128.5 to 170.4)	70.5 (58.0 to 83.8)	56.3 (43.8 to 69.3)	18.3 (10.2 to 29.9)	2.4 (2.4 to 2.4)	416.5 (362.9 to 481.3)	199.2 (163.9 to 236.5)	158.9 (123.8 to 195.8)	51.7 (28.8 to 84.5)	6.7 (6.7 to 6.7)
Malaysia	1996	155.7 (137.3 to 177.5)	74.7 (63.1 to 87.8)	58.4 (46.5 to 71.5)	19.3 (11.1 to 30.8)	3.2 (3.2 to 3.2)	439.7 (387.6 to 501.1)	211.0 (178.2 to 248.1)	164.9 (131.4 to 202.0)	54.5 (31.5 to 86.9)	9.1 (9.1 to 9.1)
Malaysia	1997	159.6 (141.7 to 180.5)	75.8 (64.6 to 88.3)	59.9 (47.9 to 72.9)	19.9 (11.7 to 30.8)	4.0 (4.0 to 4.0)	450.7 (400.1 to 509.7)	214.2 (182.5 to 249.2)	169.1 (135.4 to 206.0)	56.1 (33.0 to 86.9)	11.3 (11.3 to 11.3)
Malaysia	1998	153.0 (135.9 to 173.3)	71.7 (61.1 to 83.7)	58.4 (47.2 to 69.7)	19.3 (11.7 to 29.8)	3.6 (3.6 to 3.6)	432.0 (383.7 to 489.5)	202.4 (172.6 to 236.3)	165.0 (133.3 to 196.9)	54.6 (33.0 to 84.3)	10.1 (10.1 to 10.1)
Malaysia	1999	159.4 (141.9 to 180.5)	76.6 (65.8 to 88.9)	61.4 (50.7 to 73.6)	20.6 (12.7 to 31.8)	0.9 (0.9 to 0.9)	450.0 (400.8 to 509.8)	216.2 (185.8 to 251.1)	173.3 (143.2 to 207.9)	58.1 (35.9 to 89.9)	2.5 (2.5 to 2.5)
Malaysia	2000	174.3 (155.1 to 196.9)	84.8 (73.2 to 98.6)	66.5 (54.9 to 79.4)	22.4 (14.1 to 35.1)	0.7 (0.7 to 0.7)	492.1 (437.8 to 555.9)	239.3 (206.6 to 278.3)	187.7 (155.1 to 224.2)	63.2 (40.0 to 99.2)	1.9 (1.9 to 1.9)
Malaysia	2001	181.7 (161.9 to 204.1)	90.0 (78.1 to 103.9)	67.4 (55.5 to 80.8)	23.9 (15.1 to 36.6)	0.5 (0.5 to 0.5)	513.2 (457.3 to 576.3)	254.2 (220.7 to 293.5)	190.2 (156.8 to 228.1)	67.4 (42.7 to 103.2)	1.3 (1.3 to 1.3)
Malaysia	2002	193.1 (172.5 to 215.3)	97.5 (85.0 to 111.5)	69.9 (57.9 to 84.0)	25.7 (16.0 to 38.5)	0.1 (0.1 to 0.1)	545.2 (487.1 to 608.1)	275.3 (240.1 to 314.8)	197.3 (163.5 to 237.2)	72.4 (45.2 to 108.8)	0.3 (0.3 to 0.3)
Malaysia	2003	208.1 (187.5 to 231.2)	105.9 (92.3 to 120.0)	74.6 (62.6 to 89.5)	27.4 (17.3 to 41.9)	0.2 (0.2 to 0.2)	587.5 (529.5 to 652.9)	299.0 (260.7 to 338.9)	210.6 (176.7 to 252.6)	77.5 (48.9 to 118.3)	0.4 (0.4 to 0.4)
Malaysia	2004	218.7 (198.0 to 242.1)	111.8 (97.8 to 126.3)	78.9 (66.8 to 93.3)	27.9 (17.8 to 42.5)	0.1 (0.1 to 0.1)	617.5 (559.0 to 683.8)	315.8 (276.1 to 356.6)	222.7 (188.7 to 263.5)	78.8 (50.4 to 119.9)	0.2 (0.2 to 0.2)
Malaysia	2005	226.6 (205.3 to 249.5)	116.4 (102.6 to 130.3)	82.2 (69.2 to 95.6)	28.0 (17.8 to 42.9)	0.1 (0.1 to 0.1)	639.9 (579.8 to 704.7)	328.6 (289.8 to 367.9)	232.0 (195.4 to 270.1)	79.0 (50.2 to 121.1)	0.2 (0.2 to 0.2)
Malaysia	2006	243.4 (220.7 to 267.0)	127.3 (112.8 to 142.7)	86.7 (73.2 to 100.1)	29.3 (18.6 to 43.8)	0.0 (0.0 to 0.0)	687.2 (623.2 to 754.0)	359.4 (318.4 to 403.1)	244.9 (206.7 to 282.5)	82.7 (52.4 to 123.7)	0.1 (0.1 to 0.1)
Malaysia	2007	260.1 (235.6 to 284.4)	137.5 (122.3 to 153.5)	91.5 (76.7 to 106.1)	31.0 (19.9 to 45.7)	0.0 (0.0 to 0.0)	734.5 (665.4 to 803.2)	388.4 (345.3 to 433.6)	258.3 (216.7 to 299.7)	87.7 (56.2 to 129.2)	0.1 (0.1 to 0.1)
Malaysia	2008	272.2 (247.0 to 297.6)	145.6 (129.1 to 162.2)	94.4 (79.2 to 110.0)	32.1 (20.3 to 47.2)	0.0 (0.0 to 0.0)	768.6 (697.6 to 840.4)	411.2 (364.6 to 458.0)	266.7 (223.5 to 310.7)	90.6 (57.3 to 133.3)	0.1 (0.1 to 0.1)

		2018 US Dollars per capita					2018 PPP per capita				
Country	Year	Health spending	Government health spending	Out-of-pocket health spending	Prepaid private health spending	Development assistance for health	Health spending	Government health spending	Out-of-pocket health spending	Prepaid private health spending	Development assistance for health
Malaysia	2009	271.1 (247.2 to 296.6)	145.2 (128.1 to 162.6)	93.5 (78.9 to 108.6)	32.4 (21.0 to 47.3)	0.0 (0.0 to 0.0)	765.7 (698.0 to 837.7)	410.0 (361.7 to 459.2)	264.0 (222.9 to 306.7)	91.6 (59.4 to 133.7)	0.1 (0.1 to 0.1)
Malaysia	2010	283.2 (257.9 to 309.7)	151.6 (134.4 to 168.8)	97.8 (82.7 to 113.8)	33.8 (22.2 to 49.1)	0.0 (0.0 to 0.0)	799.6 (728.4 to 874.5)	428.1 (379.6 to 476.8)	276.1 (233.6 to 321.4)	95.4 (62.6 to 138.7)	0.1 (0.1 to 0.1)
Malaysia	2011	302.0 (275.7 to 330.6)	161.7 (143.7 to 181.5)	104.8 (89.4 to 122.0)	35.4 (23.5 to 51.9)	0.1 (0.1 to 0.1)	852.8 (778.6 to 933.7)	456.6 (405.8 to 512.6)	296.0 (252.5 to 344.6)	99.9 (66.3 to 146.6)	0.2 (0.2 to 0.2)
Malaysia	2012	323.4 (295.7 to 356.3)	173.5 (154.8 to 194.6)	112.6 (96.8 to 130.8)	37.2 (24.1 to 55.4)	0.1 (0.1 to 0.1)	913.3 (834.9 to 1006.0)	489.9 (437.2 to 549.7)	318.0 (273.3 to 369.4)	105.0 (68.1 to 156.3)	0.4 (0.4 to 0.4)
Malaysia	2013	342.7 (313.9 to 377.7)	183.9 (163.9 to 205.2)	120.6 (103.7 to 141.4)	38.2 (24.7 to 57.0)	0.1 (0.1 to 0.1)	967.9 (886.3 to 1066.6)	519.2 (463.0 to 579.5)	340.5 (292.8 to 399.3)	107.8 (69.8 to 161.0)	0.4 (0.4 to 0.4)
Malaysia	2014	370.0 (336.7 to 408.2)	198.2 (177.0 to 222.2)	130.8 (111.3 to 153.7)	40.8 (26.5 to 61.2)	0.1 (0.1 to 0.1)	1045.0 (950.9 to 1152.8)	559.8 (500.0 to 627.5)	369.5 (314.4 to 434.1)	115.3 (74.8 to 172.8)	0.4 (0.4 to 0.4)
Malaysia	2015	393.3 (354.7 to 437.0)	208.2 (183.8 to 234.3)	140.3 (117.8 to 166.0)	44.8 (28.3 to 69.2)	0.1 (0.1 to 0.1)	1110.8 (1001.6 to 1234.0)	587.8 (519.0 to 661.6)	396.2 (332.8 to 468.8)	126.4 (80.0 to 195.4)	0.3 (0.3 to 0.3)
Malaysia	2016	407.4 (365.5 to 454.8)	212.4 (185.4 to 240.6)	147.5 (121.6 to 177.9)	47.3 (29.1 to 74.2)	0.2 (0.2 to 0.2)	1150.5 (1032.2 to 1284.3)	599.8 (523.5 to 679.4)	416.5 (343.3 to 502.3)	133.7 (82.0 to 209.4)	0.5 (0.5 to 0.5)
Malaysia	2017	421.6 (376.3 to 472.2)	218.8 (190.5 to 248.7)	153.1 (126.4 to 184.4)	49.5 (30.4 to 77.6)	0.1 (0.1 to 0.1)	1190.4 (1062.7 to 1333.5)	617.8 (538.0 to 702.3)	432.4 (356.8 to 520.7)	139.8 (86.0 to 219.2)	0.4 (0.4 to 0.4)
Malaysia	2018	433.0 (386.6 to 487.2)	224.5 (195.4 to 254.9)	157.4 (129.7 to 190.0)	51.1 (31.4 to 80.2)	0.1 (0.1 to 0.1)	1222.8 (1091.7 to 1375.9)	633.9 (551.7 to 719.9)	444.3 (366.3 to 536.5)	144.2 (88.7 to 226.4)	0.4 (0.4 to 0.4)
Malaysia	2019	447.0 (397.5 to 500.4)	231.9 (202.1 to 263.3)	162.3 (134.2 to 194.9)	52.7 (32.4 to 82.6)	0.1 (0.1 to 0.1)	1262.3 (1122.6 to 1413.1)	655.0 (570.7 to 743.6)	458.2 (379.0 to 550.4)	148.8 (91.5 to 233.2)	0.4 (0.3 to 0.4)
Malaysia	2020	461.9 (410.4 to 516.9)	239.9 (208.6 to 273.6)	167.5 (138.5 to 202.6)	54.3 (33.4 to 85.1)	0.1 (0.1 to 0.1)	1304.3 (1158.9 to 1459.6)	677.4 (589.0 to 772.6)	473.1 (391.1 to 572.1)	153.4 (94.4 to 240.3)	0.4 (0.3 to 0.4)
Malaysia	2021	476.9 (424.0 to 532.9)	248.2 (216.1 to 282.5)	172.6 (142.1 to 209.1)	56.0 (34.5 to 87.7)	0.1 (0.1 to 0.1)	1346.7 (1197.3 to 1504.9)	700.8 (610.4 to 797.6)	487.4 (401.4 to 590.3)	158.1 (97.3 to 247.7)	0.4 (0.3 to 0.4)
Malaysia	2022	490.8 (436.0 to 548.9)	255.6 (222.4 to 290.9)	177.6 (145.5 to 214.2)	57.5 (35.5 to 90.2)	0.1 (0.1 to 0.1)	1386.0 (1231.3 to 1550.1)	721.7 (628.1 to 821.5)	501.5 (411.0 to 604.9)	162.5 (100.2 to 254.7)	0.4 (0.3 to 0.4)
Malaysia	2023	505.5 (450.2 to 567.5)	263.7 (229.5 to 300.1)	182.5 (150.2 to 220.3)	59.2 (36.6 to 92.8)	0.1 (0.1 to 0.2)	1427.6 (1271.5 to 1602.4)	744.6 (648.0 to 847.5)	515.4 (424.2 to 622.0)	167.1 (103.3 to 262.1)	0.4 (0.3 to 0.4)
Malaysia	2024	520.5 (463.0 to 583.1)	272.0 (236.3 to 310.2)	187.5 (154.2 to 227.7)	60.8 (37.7 to 95.6)	0.1 (0.0 to 0.2)	1469.7 (1307.5 to 1646.6)	768.1 (667.4 to 876.1)	529.4 (435.3 to 643.0)	171.8 (106.3 to 270.1)	0.4 (0.0 to 0.5)
Malaysia	2025	535.7 (476.9 to 600.8)	280.4 (242.5 to 319.9)	192.6 (158.1 to 234.1)	62.5 (38.7 to 98.3)	0.1 (0.0 to 0.2)	1512.8 (1346.8 to 1696.6)	791.9 (684.8 to 903.4)	544.0 (446.4 to 661.2)	176.6 (109.4 to 277.7)	0.3 (0.0 to 0.5)
Malaysia	2026	550.8 (488.8 to 620.2)	288.6 (249.7 to 329.5)	197.8 (161.6 to 240.8)	64.2 (39.8 to 101.0)	0.1 (0.0 to 0.2)	1555.3 (1380.3 to 1751.4)	815.1 (705.0 to 930.5)	558.6 (456.4 to 680.0)	181.3 (112.5 to 285.2)	0.3 (0.0 to 0.5)
Malaysia	2027	566.0 (503.9 to 637.2)	296.9 (255.8 to 339.3)	203.1 (165.1 to 248.3)	65.9 (41.0 to 103.7)	0.1 (0.0 to 0.2)	1598.3 (1423.1 to 1799.3)	838.5 (722.3 to 958.2)	573.4 (466.3 to 701.2)	186.0 (115.7 to 292.8)	0.3 (0.0 to 0.5)
Malaysia	2028	581.6 (515.3 to 656.0)	305.5 (262.7 to 348.8)	208.4 (168.4 to 258.3)	67.6 (42.2 to 106.5)	0.1 (0.0 to 0.2)	1642.5 (1455.2 to 1852.4)	862.8 (741.9 to 984.8)	588.6 (475.5 to 729.5)	190.9 (119.1 to 300.7)	0.2 (0.0 to 0.5)
Malaysia	2029	597.5 (528.6 to 677.9)	314.2 (269.9 to 359.6)	213.8 (171.5 to 266.9)	69.4 (43.3 to 109.3)	0.1 (0.0 to 0.2)	1687.3 (1492.8 to 1914.2)	887.4 (762.1 to 1015.6)	603.8 (484.3 to 753.6)	195.9 (122.3 to 308.7)	0.2 (0.0 to 0.5)

		2018 US Dollars per capita					2018 PPP per capita				
Country	Year	Health spending	Government health spending	Out-of-pocket health spending	Prepaid private health spending	Development assistance for health	Health spending	Government health spending	Out-of-pocket health spending	Prepaid private health spending	Development assistance for health
Malaysia	2030	612.6 (540.9 to 693.2)	322.2 (276.5 to 371.4)	219.2 (174.7 to 274.9)	71.1 (44.5 to 112.1)	0.1 (0.0 to 0.2)	1729.8 (1527.4 to 1957.5)	909.9 (780.9 to 1048.7)	619.0 (493.4 to 776.2)	200.8 (125.5 to 316.5)	0.2 (0.0 to 0.5)
Malaysia	2031	627.1 (551.4 to 712.4)	329.7 (282.0 to 380.8)	224.5 (177.5 to 282.5)	72.9 (45.5 to 114.9)	0.1 (0.0 to 0.2)	1770.9 (1557.2 to 2011.8)	931.0 (796.3 to 1075.4)	634.1 (501.2 to 797.7)	205.7 (128.5 to 324.4)	0.1 (0.0 to 0.5)
Malaysia	2032	641.9 (564.0 to 731.8)	337.2 (288.1 to 389.8)	229.9 (181.0 to 291.0)	74.6 (46.6 to 117.8)	0.0 (0.0 to 0.2)	1812.5 (1592.6 to 2066.5)	952.3 (813.6 to 1100.6)	649.3 (511.1 to 821.7)	210.8 (131.7 to 332.5)	0.1 (0.0 to 0.5)
Malaysia	2033	657.2 (577.2 to 752.2)	345.2 (294.6 to 401.2)	235.4 (183.3 to 298.2)	76.5 (47.8 to 120.9)	0.0 (0.0 to 0.2)	1855.9 (1629.9 to 2124.1)	974.9 (831.9 to 1133.0)	664.8 (517.5 to 842.1)	216.1 (135.1 to 341.3)	0.1 (0.0 to 0.6)
Malaysia	2034	672.7 (585.0 to 775.1)	353.3 (300.8 to 411.3)	241.0 (186.2 to 308.7)	78.4 (49.0 to 124.1)	0.0 (0.0 to 0.2)	1899.6 (1651.9 to 2188.9)	997.6 (849.3 to 1161.6)	680.4 (525.8 to 871.8)	221.4 (138.3 to 350.3)	0.1 (0.0 to 0.6)
Malaysia	2035	688.9 (602.0 to 796.8)	361.8 (308.3 to 423.3)	246.6 (190.0 to 319.5)	80.4 (50.2 to 127.4)	0.0 (0.0 to 0.2)	1945.3 (1700.0 to 2249.9)	1021.8 (870.5 to 1195.2)	696.4 (536.5 to 902.1)	227.0 (141.9 to 359.8)	0.1 (0.0 to 0.6)
Malaysia	2036	705.0 (615.8 to 814.5)	370.3 (315.2 to 433.8)	252.3 (193.8 to 327.9)	82.4 (51.4 to 130.8)	0.0 (0.0 to 0.2)	1990.8 (1739.0 to 2299.9)	1045.7 (890.0 to 1225.0)	712.4 (547.4 to 925.9)	232.7 (145.2 to 369.3)	0.1 (0.0 to 0.6)
Malaysia	2037	721.6 (627.3 to 832.8)	379.1 (321.0 to 446.3)	258.0 (197.8 to 336.3)	84.5 (52.6 to 134.3)	0.0 (0.0 to 0.2)	2037.7 (1771.5 to 2351.6)	1070.5 (906.4 to 1260.4)	728.6 (558.5 to 949.8)	238.5 (148.5 to 379.2)	0.1 (0.0 to 0.6)
Malaysia	2038	738.4 (639.2 to 854.3)	388.1 (327.3 to 456.8)	263.8 (201.0 to 345.9)	86.6 (53.9 to 137.9)	0.0 (0.0 to 0.2)	2085.3 (1805.1 to 2412.3)	1095.8 (924.2 to 1289.8)	744.9 (567.6 to 976.7)	244.5 (152.2 to 389.3)	0.1 (0.0 to 0.6)
Malaysia	2039	755.5 (655.5 to 882.5)	397.2 (334.0 to 468.9)	269.6 (204.6 to 353.6)	88.7 (55.2 to 141.5)	0.0 (0.0 to 0.2)	2133.5 (1851.0 to 2492.1)	1121.5 (943.2 to 1324.1)	761.3 (577.7 to 998.5)	250.6 (155.9 to 399.7)	0.1 (0.0 to 0.6)
Malaysia	2040	773.6 (668.6 to 901.7)	406.9 (340.4 to 483.9)	275.6 (208.5 to 362.5)	91.1 (56.6 to 145.4)	0.0 (0.0 to 0.2)	2184.6 (1888.0 to 2546.4)	1149.2 (961.1 to 1366.4)	778.3 (588.8 to 1023.6)	257.1 (159.9 to 410.7)	0.1 (0.0 to 0.6)
Malaysia	2041	791.4 (680.0 to 922.1)	416.5 (347.1 to 496.0)	281.5 (212.6 to 372.1)	93.4 (58.0 to 149.3)	0.0 (0.0 to 0.2)	2234.8 (1920.2 to 2604.0)	1176.1 (980.1 to 1400.7)	795.0 (600.2 to 1050.7)	263.6 (163.7 to 421.7)	0.1 (0.0 to 0.7)
Malaysia	2042	809.7 (694.2 to 944.7)	426.4 (353.1 to 509.1)	287.5 (216.5 to 381.0)	95.8 (59.3 to 153.4)	0.0 (0.0 to 0.2)	2286.5 (1960.2 to 2667.8)	1204.1 (997.0 to 1437.7)	811.9 (611.3 to 1075.8)	270.4 (167.5 to 433.3)	0.1 (0.0 to 0.7)
Malaysia	2043	828.3 (709.0 to 973.8)	436.5 (361.0 to 520.4)	293.5 (220.2 to 390.1)	98.2 (60.6 to 157.2)	0.0 (0.0 to 0.2)	2338.9 (2002.3 to 2749.9)	1232.7 (1019.6 to 1469.5)	828.8 (621.8 to 1101.5)	277.3 (171.2 to 444.1)	0.1 (0.0 to 0.7)
Malaysia	2044	847.3 (721.7 to 1003.5)	447.0 (368.1 to 536.1)	299.5 (224.0 to 398.7)	100.7 (62.0 to 161.1)	0.0 (0.0 to 0.2)	2392.6 (2037.9 to 2833.7)	1262.3 (1039.5 to 1513.8)	845.9 (632.4 to 1126.0)	284.3 (175.1 to 454.9)	0.1 (0.0 to 0.7)
Malaysia	2045	867.1 (741.8 to 1030.3)	458.2 (376.1 to 549.3)	305.6 (227.7 to 408.2)	103.2 (63.4 to 165.0)	0.0 (0.0 to 0.3)	2448.5 (2094.7 to 2909.4)	1293.9 (1062.0 to 1551.2)	863.1 (643.1 to 1152.6)	291.5 (179.0 to 465.9)	0.1 (0.0 to 0.7)
Malaysia	2046	886.6 (754.9 to 1045.2)	469.3 (385.3 to 563.8)	311.6 (231.5 to 416.9)	105.7 (64.7 to 168.8)	0.0 (0.0 to 0.3)	2503.5 (2131.9 to 2951.6)	1325.2 (1088.1 to 1592.2)	879.8 (653.8 to 1177.1)	298.5 (182.7 to 476.6)	0.1 (0.0 to 0.7)
Malaysia	2047	906.0 (770.7 to 1069.2)	480.5 (390.5 to 578.2)	317.4 (235.9 to 425.2)	108.1 (66.1 to 172.5)	0.0 (0.0 to 0.3)	2558.5 (2176.3 to 3019.2)	1356.8 (1102.8 to 1632.8)	896.3 (666.2 to 1200.8)	305.4 (186.6 to 487.2)	0.1 (0.0 to 0.7)
Malaysia	2048	926.5 (785.9 to 1091.2)	492.5 (399.3 to 595.4)	323.4 (240.1 to 433.9)	110.6 (67.5 to 176.8)	0.0 (0.0 to 0.3)	2616.3 (2219.3 to 3081.4)	1390.7 (1127.5 to 1681.4)	913.1 (677.9 to 1225.2)	312.4 (190.6 to 499.2)	0.1 (0.0 to 0.8)
Malaysia	2049	946.8 (801.3 to 1120.1)	504.4 (406.8 to 611.8)	329.3 (244.9 to 442.3)	113.1 (68.7 to 180.6)	0.0 (0.0 to 0.3)	2673.7 (2262.9 to 3163.2)	1424.4 (1148.7 to 1727.5)	929.8 (691.5 to 1249.0)	319.4 (194.1 to 509.9)	0.1 (0.0 to 0.8)
Malaysia	2050	967.6 (818.3 to 1146.3)	516.8 (415.7 to 629.5)	335.2 (248.9 to 451.1)	115.5 (70.0 to 184.3)	0.0 (0.0 to 0.3)	2732.3 (2310.9 to 3237.1)	1459.3 (1173.8 to 1777.6)	946.6 (702.8 to 1274.0)	326.3 (197.6 to 520.5)	0.1 (0.0 to 0.8)

		2018 US Dollars per capita					2018 PPP per capita				
Country	Year	Health spending	Government health spending	Out-of-pocket health spending	Prepaid private health spending	Development assistance for health	Health spending	Government health spending	Out-of-pocket health spending	Prepaid private health spending	Development assistance for health
Maldives	1995	391.7 (350.1 to 434.3)	98.9 (81.5 to 118.3)	239.8 (207.3 to 272.0)	53.0 (36.1 to 75.4)	0.0 (0.0 to 0.0)	618.6 (553.0 to 685.9)	156.2 (128.8 to 186.9)	378.7 (327.4 to 429.6)	83.7 (57.0 to 119.2)	0.0 (0.0 to 0.0)
Maldives	1996	407.5 (369.6 to 446.6)	106.6 (89.0 to 126.2)	245.6 (217.8 to 273.6)	55.3 (38.6 to 76.5)	0.0 (0.0 to 0.0)	643.6 (583.8 to 705.5)	168.4 (140.5 to 199.4)	387.9 (344.1 to 432.1)	87.3 (61.0 to 120.9)	0.0 (0.0 to 0.0)
Maldives	1997	427.0 (391.1 to 465.8)	117.5 (99.1 to 138.9)	248.5 (223.1 to 276.4)	57.4 (41.1 to 77.8)	3.7 (3.7 to 3.7)	674.5 (617.7 to 735.7)	185.5 (156.5 to 219.5)	392.5 (352.4 to 436.6)	90.7 (64.9 to 122.8)	5.8 (5.8 to 5.8)
Maldives	1998	445.6 (408.8 to 483.6)	134.9 (114.4 to 158.5)	251.7 (226.9 to 279.9)	59.0 (42.0 to 79.7)	0.1 (0.1 to 0.1)	703.8 (645.8 to 763.9)	213.0 (180.7 to 250.4)	397.5 (358.3 to 442.0)	93.1 (66.3 to 125.9)	0.1 (0.1 to 0.1)
Maldives	1999	436.6 (401.4 to 473.1)	139.1 (119.4 to 162.2)	243.7 (218.6 to 270.7)	52.6 (37.2 to 71.6)	1.1 (1.1 to 1.1)	689.6 (634.0 to 747.2)	219.7 (188.5 to 256.3)	385.0 (345.3 to 427.5)	83.1 (58.8 to 113.0)	1.7 (1.7 to 1.7)
Maldives	2000	400.1 (366.9 to 431.7)	132.3 (114.7 to 152.5)	227.8 (205.9 to 250.2)	38.5 (27.0 to 52.8)	1.5 (1.5 to 1.5)	631.9 (579.5 to 681.9)	208.9 (181.2 to 240.8)	359.9 (325.1 to 395.2)	60.8 (42.6 to 83.4)	2.4 (2.4 to 2.4)
Maldives	2001	398.7 (365.3 to 429.8)	130.0 (113.5 to 148.7)	223.2 (201.4 to 244.9)	45.0 (31.5 to 61.0)	0.6 (0.6 to 0.6)	629.8 (577.0 to 678.9)	205.3 (179.3 to 234.8)	352.5 (318.1 to 386.9)	71.1 (49.7 to 96.3)	0.9 (0.9 to 0.9)
Maldives	2002	415.3 (382.3 to 449.0)	133.1 (116.7 to 152.2)	226.3 (203.5 to 251.1)	55.5 (39.2 to 75.8)	0.4 (0.4 to 0.4)	656.0 (603.9 to 709.3)	210.2 (184.3 to 240.4)	357.5 (321.4 to 396.6)	87.6 (61.9 to 119.7)	0.7 (0.7 to 0.7)
Maldives	2003	457.9 (422.7 to 494.9)	153.3 (135.3 to 174.0)	245.4 (221.9 to 272.1)	58.7 (41.8 to 79.6)	0.4 (0.4 to 0.4)	723.2 (667.6 to 781.6)	242.2 (213.7 to 274.8)	387.6 (350.5 to 429.7)	92.7 (66.0 to 125.8)	0.7 (0.7 to 0.7)
Maldives	2004	524.9 (487.1 to 566.9)	183.9 (163.4 to 205.7)	280.8 (255.8 to 309.2)	59.5 (42.6 to 80.1)	0.7 (0.7 to 0.7)	829.1 (769.3 to 895.5)	290.5 (258.1 to 324.9)	443.5 (404.0 to 488.4)	94.0 (67.2 to 126.5)	1.1 (1.1 to 1.1)
Maldives	2005	532.4 (497.8 to 573.2)	195.4 (175.4 to 217.0)	280.1 (255.0 to 309.0)	55.0 (39.4 to 74.3)	1.9 (1.9 to 1.9)	840.8 (786.3 to 905.4)	308.6 (277.0 to 342.8)	442.4 (402.8 to 488.0)	86.8 (62.3 to 117.3)	3.0 (3.0 to 3.0)
Maldives	2006	635.0 (595.5 to 680.9)	266.8 (242.0 to 294.8)	305.0 (278.5 to 335.0)	62.5 (44.6 to 83.4)	0.6 (0.6 to 0.6)	1002.9 (940.6 to 1075.5)	421.4 (382.2 to 465.6)	481.8 (439.8 to 529.1)	98.8 (70.4 to 131.7)	1.0 (1.0 to 1.0)
Maldives	2007	734.3 (691.0 to 783.7)	351.3 (321.5 to 383.7)	318.7 (292.6 to 348.0)	58.3 (41.7 to 79.1)	5.9 (5.9 to 5.9)	1159.8 (1091.5 to 1237.8)	554.9 (507.8 to 606.0)	503.4 (462.2 to 549.7)	92.1 (65.9 to 124.9)	9.4 (9.4 to 9.4)
Maldives	2008	858.7 (809.9 to 913.9)	454.2 (418.5 to 491.8)	332.9 (304.8 to 361.6)	69.3 (49.8 to 93.8)	2.2 (2.2 to 2.2)	1356.3 (1279.3 to 1443.4)	717.4 (661.0 to 776.8)	525.8 (481.4 to 571.2)	109.5 (78.7 to 148.2)	3.6 (3.6 to 3.6)
Maldives	2009	794.5 (748.3 to 843.5)	436.6 (403.5 to 469.4)	301.0 (274.1 to 327.8)	52.3 (38.6 to 69.3)	4.6 (4.6 to 4.6)	1254.9 (1181.9 to 1332.4)	689.6 (637.3 to 741.4)	475.4 (433.0 to 517.8)	82.6 (60.9 to 109.5)	7.3 (7.3 to 7.3)
Maldives	2010	760.9 (716.6 to 807.7)	415.2 (383.1 to 448.1)	287.6 (260.5 to 315.5)	54.2 (40.5 to 71.8)	4.0 (4.0 to 4.0)	1201.9 (1131.9 to 1275.8)	655.8 (605.2 to 707.8)	454.2 (411.4 to 498.4)	85.5 (64.0 to 113.4)	6.3 (6.3 to 6.3)
Maldives	2011	761.0 (717.0 to 804.7)	402.5 (369.7 to 433.7)	274.9 (249.1 to 302.9)	72.9 (55.3 to 96.7)	10.6 (10.6 to 10.6)	1201.9 (1132.4 to 1271.0)	635.8 (584.0 to 684.9)	434.3 (393.5 to 478.4)	115.2 (87.3 to 152.7)	16.7 (16.7 to 16.7)
Maldives	2012	760.8 (719.5 to 804.4)	407.8 (375.8 to 438.5)	253.8 (229.6 to 280.3)	91.9 (70.8 to 118.9)	7.3 (7.3 to 7.3)	1201.6 (1136.4 to 1270.5)	644.1 (593.6 to 692.5)	400.8 (362.6 to 442.8)	145.1 (111.8 to 187.8)	11.6 (11.6 to 11.6)
Maldives	2013	773.1 (732.6 to 818.1)	440.3 (408.4 to 473.5)	235.4 (212.6 to 261.2)	94.6 (72.4 to 121.1)	2.8 (2.8 to 2.8)	1221.0 (1157.1 to 1292.1)	695.4 (645.0 to 747.9)	371.8 (335.8 to 412.6)	149.4 (114.3 to 191.3)	4.5 (4.5 to 4.5)
Maldives	2014	813.2 (766.9 to 862.1)	508.5 (474.7 to 544.8)	215.0 (192.3 to 240.4)	88.1 (66.3 to 113.8)	1.6 (1.6 to 1.6)	1284.4 (1211.3 to 1361.7)	803.1 (749.8 to 860.6)	339.5 (303.7 to 379.7)	139.1 (104.7 to 179.7)	2.6 (2.6 to 2.6)
Maldives	2015	876.2 (822.5 to 931.3)	594.2 (553.9 to 635.2)	194.1 (171.4 to 220.6)	85.4 (62.6 to 114.9)	2.4 (2.4 to 2.4)	1383.9 (1299.0 to 1470.9)	938.5 (874.9 to 1003.4)	306.6 (270.7 to 348.4)	134.9 (98.9 to 181.5)	3.9 (3.9 to 3.9)

Country	Year	2018 US Dollars per capita					2018 PPP per capita				
		Health spending	Government health spending	Out-of-pocket health spending	Prepaid private health spending	Development assistance for health	Health spending	Government health spending	Out-of-pocket health spending	Prepaid private health spending	Development assistance for health
Maldives	2016	974.4 (903.0 to 1046.8)	686.6 (632.9 to 741.5)	195.7 (168.7 to 227.2)	90.1 (62.6 to 125.2)	2.0 (2.0 to 2.0)	1539.1 (1426.2 to 1653.3)	1084.5 (999.6 to 1171.1)	309.2 (266.4 to 358.9)	142.3 (98.9 to 197.7)	3.1 (3.1 to 3.1)
Maldives	2017	960.2 (893.3 to 1032.6)	666.3 (613.7 to 720.7)	199.9 (171.4 to 232.1)	93.6 (65.3 to 129.4)	0.3 (0.3 to 0.3)	1516.6 (1410.9 to 1631.0)	1052.4 (969.3 to 1138.3)	315.8 (270.7 to 366.6)	147.9 (103.1 to 204.4)	0.5 (0.5 to 0.5)
Maldives	2018	982.9 (912.7 to 1059.8)	681.2 (626.6 to 738.1)	204.5 (175.5 to 237.4)	96.9 (67.6 to 134.0)	0.3 (0.3 to 0.3)	1552.4 (1441.6 to 1673.9)	1076.0 (989.7 to 1165.9)	323.0 (277.3 to 375.0)	153.0 (106.9 to 211.7)	0.4 (0.4 to 0.4)
Maldives	2019	1009.6 (937.1 to 1091.5)	701.1 (645.7 to 758.8)	208.4 (178.3 to 242.6)	99.8 (69.7 to 138.3)	0.3 (0.3 to 0.3)	1594.7 (1480.1 to 1724.1)	1107.4 (1019.9 to 1198.6)	329.2 (281.6 to 383.3)	157.7 (110.1 to 218.4)	0.4 (0.4 to 0.5)
Maldives	2020	1034.9 (960.2 to 1116.5)	720.3 (663.0 to 779.8)	211.5 (180.6 to 245.9)	102.7 (71.6 to 142.2)	0.3 (0.3 to 0.3)	1634.5 (1516.6 to 1763.4)	1137.7 (1047.1 to 1231.7)	334.1 (285.3 to 388.4)	162.3 (113.1 to 224.6)	0.4 (0.4 to 0.5)
Maldives	2021	1058.0 (981.1 to 1143.7)	737.8 (678.7 to 799.4)	214.5 (183.3 to 249.2)	105.4 (73.6 to 145.7)	0.3 (0.3 to 0.3)	1671.0 (1549.6 to 1806.4)	1165.4 (1071.9 to 1262.6)	338.7 (289.5 to 393.7)	166.5 (116.2 to 230.2)	0.4 (0.4 to 0.5)
Maldives	2022	1079.7 (1004.4 to 1163.4)	754.6 (694.5 to 817.2)	216.9 (185.6 to 252.8)	107.9 (75.3 to 149.1)	0.3 (0.3 to 0.3)	1705.4 (1586.4 to 1837.5)	1191.9 (1096.9 to 1290.7)	342.6 (293.1 to 399.2)	170.5 (118.9 to 235.5)	0.4 (0.4 to 0.5)
Maldives	2023	1100.1 (1021.7 to 1185.5)	770.4 (708.5 to 835.6)	219.2 (187.2 to 255.6)	110.3 (76.8 to 152.7)	0.3 (0.3 to 0.3)	1737.7 (1613.8 to 1872.5)	1216.7 (1119.0 to 1319.8)	346.2 (295.7 to 403.7)	174.2 (121.2 to 241.2)	0.5 (0.4 to 0.5)
Maldives	2024	1120.7 (1045.1 to 1207.4)	786.1 (722.2 to 853.8)	221.7 (189.7 to 258.6)	112.7 (78.3 to 156.0)	0.3 (0.3 to 0.3)	1770.1 (1650.7 to 1907.1)	1241.6 (1140.7 to 1348.6)	350.1 (299.7 to 408.4)	178.0 (123.6 to 246.5)	0.5 (0.4 to 0.5)
Maldives	2025	1143.2 (1061.5 to 1231.7)	803.2 (738.0 to 873.1)	224.4 (192.2 to 261.8)	115.3 (80.0 to 159.6)	0.3 (0.3 to 0.4)	1805.6 (1676.6 to 1945.5)	1268.6 (1165.6 to 1379.0)	354.4 (303.5 to 413.6)	182.1 (126.3 to 252.0)	0.5 (0.4 to 0.6)
Maldives	2026	1165.2 (1081.7 to 1260.2)	819.9 (753.5 to 891.9)	227.1 (193.8 to 265.6)	117.8 (81.8 to 163.0)	0.3 (0.3 to 0.4)	1840.4 (1708.5 to 1990.4)	1295.1 (1190.1 to 1408.8)	358.8 (306.1 to 419.5)	186.1 (129.2 to 257.5)	0.5 (0.4 to 0.6)
Maldives	2027	1188.5 (1101.6 to 1282.9)	837.7 (769.2 to 912.2)	230.0 (195.1 to 270.5)	120.5 (83.7 to 166.8)	0.3 (0.3 to 0.4)	1877.2 (1739.9 to 2026.3)	1323.0 (1215.0 to 1440.9)	363.3 (308.1 to 427.3)	190.4 (132.2 to 263.4)	0.5 (0.4 to 0.6)
Maldives	2028	1213.0 (1122.6 to 1310.4)	856.2 (785.7 to 934.0)	233.1 (196.6 to 275.3)	123.4 (85.7 to 170.8)	0.3 (0.3 to 0.4)	1915.9 (1773.1 to 2069.8)	1352.3 (1241.0 to 1475.2)	368.2 (310.6 to 434.9)	194.9 (135.4 to 269.7)	0.5 (0.4 to 0.6)
Maldives	2029	1238.8 (1136.6 to 1338.9)	875.7 (803.1 to 957.0)	236.4 (197.5 to 281.5)	126.4 (87.8 to 174.9)	0.3 (0.3 to 0.4)	1956.6 (1795.2 to 2114.7)	1383.1 (1268.5 to 1511.5)	373.4 (312.0 to 444.6)	199.6 (138.8 to 276.3)	0.5 (0.4 to 0.6)
Maldives	2030	1266.0 (1167.2 to 1369.7)	896.3 (821.3 to 981.1)	239.9 (198.3 to 286.4)	129.5 (90.1 to 179.1)	0.3 (0.3 to 0.4)	1999.7 (1843.6 to 2163.4)	1415.7 (1297.2 to 1549.5)	379.0 (313.2 to 452.3)	204.5 (142.2 to 282.9)	0.5 (0.4 to 0.6)
Maldives	2031	1293.6 (1190.8 to 1402.5)	917.0 (839.5 to 1005.5)	243.5 (199.4 to 293.0)	132.7 (92.3 to 182.9)	0.3 (0.3 to 0.4)	2043.2 (1880.9 to 2215.3)	1448.4 (1326.0 to 1588.2)	384.7 (315.0 to 462.8)	209.6 (145.8 to 288.9)	0.5 (0.4 to 0.7)
Maldives	2032	1323.0 (1217.2 to 1429.8)	939.2 (858.6 to 1032.1)	247.4 (200.7 to 299.7)	136.1 (94.7 to 187.4)	0.3 (0.3 to 0.4)	2089.6 (1922.6 to 2258.3)	1483.4 (1356.2 to 1630.2)	390.8 (317.0 to 473.3)	214.9 (149.5 to 296.0)	0.5 (0.4 to 0.7)
Maldives	2033	1354.1 (1238.6 to 1469.0)	962.6 (879.1 to 1058.4)	251.5 (202.1 to 307.8)	139.6 (97.1 to 192.2)	0.4 (0.3 to 0.5)	2138.7 (1956.4 to 2320.3)	1520.3 (1388.4 to 1671.8)	397.3 (319.3 to 486.2)	220.6 (153.4 to 303.6)	0.6 (0.4 to 0.7)
Maldives	2034	1386.3 (1268.4 to 1509.5)	986.7 (899.9 to 1086.1)	255.8 (204.0 to 316.8)	143.3 (99.6 to 197.2)	0.4 (0.3 to 0.5)	2189.6 (2003.4 to 2384.2)	1558.5 (1421.3 to 1715.4)	404.1 (322.2 to 500.4)	226.4 (157.4 to 311.5)	0.6 (0.4 to 0.7)
Maldives	2035	1419.5 (1301.5 to 1553.8)	1011.6 (920.5 to 1113.9)	260.3 (205.8 to 324.5)	147.2 (102.3 to 202.4)	0.4 (0.0 to 0.5)	2242.1 (2055.7 to 2454.2)	1597.9 (1453.9 to 1759.3)	411.2 (325.1 to 512.5)	232.5 (161.6 to 319.7)	0.6 (0.0 to 0.8)
Maldives	2036	1452.5 (1326.2 to 1579.5)	1036.3 (942.5 to 1142.1)	264.8 (207.4 to 331.8)	151.0 (104.9 to 208.1)	0.4 (0.0 to 0.5)	2294.1 (2094.6 to 2494.8)	1636.8 (1488.6 to 1804.0)	418.3 (327.5 to 524.1)	238.5 (165.6 to 328.6)	0.6 (0.0 to 0.8)

Country	Year	2018 US Dollars per capita					2018 PPP per capita				
		Health spending	Government health spending	Out-of-pocket health spending	Prepaid private health spending	Development assistance for health	Health spending	Government health spending	Out-of-pocket health spending	Prepaid private health spending	Development assistance for health
Maldives	2037	1486.7 (1364.6 to 1622.0)	1061.8 (963.0 to 1172.0)	269.6 (209.7 to 339.5)	154.9 (107.5 to 213.7)	0.4 (0.0 to 0.5)	2348.2 (2155.3 to 2561.8)	1677.1 (1521.0 to 1851.1)	425.8 (331.3 to 536.2)	244.7 (169.9 to 337.5)	0.6 (0.0 to 0.8)
Maldives	2038	1521.8 (1392.3 to 1662.4)	1087.9 (985.1 to 1204.2)	274.5 (212.8 to 347.3)	158.9 (110.3 to 219.7)	0.4 (0.0 to 0.5)	2403.6 (2199.1 to 2625.8)	1718.4 (1556.0 to 1901.9)	433.6 (336.1 to 548.5)	251.0 (174.2 to 347.1)	0.6 (0.0 to 0.9)
Maldives	2039	1557.6 (1415.8 to 1702.5)	1114.6 (1007.1 to 1237.0)	279.6 (215.2 to 355.1)	163.1 (113.1 to 225.9)	0.4 (0.0 to 0.6)	2460.2 (2236.2 to 2689.1)	1760.4 (1590.7 to 1953.8)	441.7 (339.9 to 560.9)	257.6 (178.7 to 356.9)	0.6 (0.0 to 0.9)
Maldives	2040	1592.8 (1444.6 to 1751.5)	1140.6 (1029.0 to 1268.9)	284.7 (217.5 to 361.9)	167.1 (115.9 to 231.9)	0.3 (0.0 to 0.6)	2515.8 (2281.7 to 2766.4)	1801.5 (1625.3 to 2004.2)	449.8 (343.5 to 571.6)	263.9 (183.0 to 366.3)	0.6 (0.0 to 0.9)
Maldives	2041	1627.3 (1476.9 to 1793.2)	1166.0 (1049.7 to 1300.4)	289.8 (220.5 to 369.0)	171.1 (118.6 to 237.2)	0.3 (0.0 to 0.6)	2570.2 (2332.7 to 2832.3)	1841.7 (1658.0 to 2053.9)	457.8 (348.3 to 582.8)	270.2 (187.3 to 374.6)	0.5 (0.0 to 0.9)
Maldives	2042	1663.1 (1505.4 to 1831.9)	1192.4 (1071.0 to 1331.5)	295.1 (224.0 to 377.8)	175.2 (121.3 to 242.6)	0.3 (0.0 to 0.6)	2626.8 (2377.8 to 2893.4)	1883.4 (1691.7 to 2103.1)	466.1 (353.8 to 596.8)	276.7 (191.5 to 383.2)	0.5 (0.0 to 1.0)
Maldives	2043	1699.2 (1547.9 to 1875.2)	1218.8 (1091.9 to 1365.1)	300.6 (227.7 to 385.9)	179.3 (124.2 to 249.0)	0.3 (0.0 to 0.6)	2683.8 (2444.8 to 2961.8)	1925.1 (1724.5 to 2156.1)	474.9 (359.7 to 609.5)	283.3 (196.1 to 393.3)	0.5 (0.0 to 1.0)
Maldives	2044	1733.1 (1574.8 to 1928.0)	1243.3 (1112.0 to 1396.1)	306.3 (232.0 to 393.9)	183.2 (126.8 to 255.2)	0.3 (0.0 to 0.7)	2737.3 (2487.4 to 3045.2)	1963.7 (1756.3 to 2205.1)	483.8 (366.4 to 622.2)	289.4 (200.3 to 403.2)	0.5 (0.0 to 1.0)
Maldives	2045	1765.7 (1594.8 to 1964.9)	1266.3 (1130.8 to 1423.3)	312.2 (236.4 to 402.0)	186.9 (129.3 to 260.7)	0.3 (0.0 to 0.7)	2788.9 (2518.9 to 3103.6)	2000.1 (1786.0 to 2248.0)	493.1 (373.4 to 635.0)	295.2 (204.2 to 411.8)	0.5 (0.0 to 1.1)
Maldives	2046	1794.2 (1611.2 to 1992.6)	1285.9 (1147.4 to 1449.0)	317.9 (240.3 to 409.5)	190.1 (131.3 to 265.3)	0.3 (0.0 to 0.7)	2833.9 (2544.9 to 3147.3)	2031.1 (1812.2 to 2288.6)	502.1 (379.5 to 646.8)	300.2 (207.5 to 419.0)	0.5 (0.0 to 1.1)
Maldives	2047	1820.5 (1630.3 to 2031.1)	1303.5 (1159.4 to 1471.2)	323.8 (243.2 to 417.0)	192.9 (133.0 to 269.4)	0.3 (0.0 to 0.7)	2875.4 (2575.0 to 3208.1)	2058.8 (1831.2 to 2323.6)	511.4 (384.2 to 658.6)	304.8 (210.1 to 425.5)	0.5 (0.0 to 1.2)
Maldives	2048	1843.0 (1653.6 to 2048.4)	1317.8 (1169.5 to 1492.0)	329.5 (246.5 to 424.3)	195.4 (134.4 to 272.9)	0.3 (0.0 to 0.8)	2911.0 (2611.7 to 3235.4)	2081.5 (1847.1 to 2356.5)	520.4 (389.3 to 670.2)	308.6 (212.3 to 431.1)	0.5 (0.0 to 1.3)
Maldives	2049	1863.1 (1662.5 to 2087.1)	1330.1 (1176.5 to 1508.3)	335.1 (250.1 to 431.4)	197.5 (135.6 to 277.0)	0.3 (0.0 to 0.8)	2942.6 (2625.9 to 3296.5)	2100.9 (1858.3 to 2382.4)	529.3 (395.0 to 681.4)	312.0 (214.2 to 437.5)	0.5 (0.0 to 1.3)
Maldives	2050	1881.6 (1687.6 to 2116.2)	1341.1 (1183.9 to 1523.8)	340.7 (253.7 to 439.1)	199.5 (136.7 to 281.3)	0.3 (0.0 to 0.8)	2971.9 (2665.6 to 3342.5)	2118.2 (1869.9 to 2406.8)	538.1 (400.7 to 693.5)	315.0 (215.9 to 444.3)	0.5 (0.0 to 1.3)
Mali	1995	20.2 (17.1 to 24.2)	5.8 (4.5 to 7.5)	8.8 (6.0 to 12.4)	0.2 (0.1 to 0.3)	5.5 (5.5 to 5.5)	52.1 (44.0 to 62.3)	15.0 (11.5 to 19.3)	22.6 (15.5 to 31.9)	0.5 (0.2 to 0.9)	14.1 (14.1 to 14.1)
Mali	1996	22.0 (18.0 to 27.3)	5.8 (4.5 to 7.5)	11.9 (8.1 to 16.7)	0.2 (0.1 to 0.3)	4.1 (4.1 to 4.1)	56.6 (46.4 to 70.2)	15.0 (11.6 to 19.2)	30.7 (21.0 to 43.0)	0.5 (0.2 to 0.9)	10.4 (10.4 to 10.4)
Mali	1997	23.1 (18.8 to 28.6)	5.6 (4.3 to 7.2)	13.1 (9.1 to 18.3)	0.2 (0.1 to 0.3)	4.1 (4.1 to 4.1)	59.4 (48.5 to 73.6)	14.5 (11.0 to 18.6)	33.8 (23.3 to 47.1)	0.5 (0.2 to 0.9)	10.6 (10.6 to 10.6)
Mali	1998	20.8 (16.4 to 26.1)	5.4 (4.1 to 7.0)	13.6 (9.4 to 18.8)	0.2 (0.1 to 0.3)	1.6 (1.6 to 1.6)	53.5 (42.2 to 67.1)	13.9 (10.7 to 17.9)	35.0 (24.1 to 48.4)	0.4 (0.2 to 0.8)	4.1 (4.1 to 4.1)
Mali	1999	22.5 (18.1 to 28.0)	5.1 (3.9 to 6.7)	14.0 (9.6 to 19.3)	0.2 (0.1 to 0.3)	3.2 (3.2 to 3.2)	57.8 (46.5 to 72.1)	13.2 (10.1 to 17.2)	35.9 (24.8 to 49.6)	0.4 (0.2 to 0.8)	8.3 (8.3 to 8.3)
Mali	2000	21.3 (16.9 to 26.4)	4.6 (3.5 to 6.0)	13.3 (9.2 to 18.2)	0.1 (0.1 to 0.2)	3.3 (3.3 to 3.3)	54.7 (43.6 to 68.0)	11.8 (9.0 to 15.3)	34.2 (23.7 to 46.9)	0.3 (0.1 to 0.6)	8.5 (8.5 to 8.5)
Mali	2001	23.5 (19.2 to 28.9)	5.0 (3.8 to 6.5)	13.4 (9.3 to 18.6)	0.1 (0.1 to 0.2)	5.1 (5.1 to 5.1)	60.6 (49.3 to 74.3)	12.8 (9.9 to 16.9)	34.5 (24.0 to 47.9)	0.3 (0.1 to 0.6)	13.0 (13.0 to 13.0)

Country	Year	2018 US Dollars per capita					2018 PPP per capita				
		Health spending	Government health spending	Out-of-pocket health spending	Prepaid private health spending	Development assistance for health	Health spending	Government health spending	Out-of-pocket health spending	Prepaid private health spending	Development assistance for health
Mali	2002	21.2 (16.7 to 26.5)	5.3 (4.1 to 6.9)	13.4 (9.3 to 18.4)	0.1 (0.1 to 0.2)	2.3 (2.3 to 2.3)	54.6 (43.1 to 68.3)	13.7 (10.5 to 17.7)	34.6 (24.0 to 47.4)	0.3 (0.1 to 0.6)	6.0 (6.0 to 6.0)
Mali	2003	25.9 (21.4 to 31.1)	6.3 (4.8 to 8.1)	14.0 (9.8 to 19.0)	0.1 (0.1 to 0.3)	5.4 (5.4 to 5.4)	66.6 (55.1 to 80.1)	16.3 (12.5 to 20.9)	36.0 (25.3 to 48.9)	0.4 (0.2 to 0.7)	13.9 (13.9 to 13.9)
Mali	2004	26.6 (22.1 to 31.9)	7.1 (5.5 to 9.4)	13.9 (9.7 to 18.9)	0.1 (0.1 to 0.3)	5.5 (5.5 to 5.5)	68.6 (56.7 to 82.1)	18.3 (14.0 to 24.1)	35.7 (25.1 to 48.8)	0.4 (0.2 to 0.7)	14.1 (14.1 to 14.1)
Mali	2005	29.2 (24.7 to 34.7)	7.5 (5.7 to 9.8)	14.4 (10.1 to 19.6)	0.2 (0.1 to 0.3)	7.2 (7.2 to 7.2)	75.2 (63.5 to 89.4)	19.3 (14.8 to 25.2)	36.9 (26.1 to 50.6)	0.4 (0.2 to 0.8)	18.6 (18.6 to 18.6)
Mali	2006	30.5 (25.6 to 36.6)	7.6 (5.8 to 10.0)	15.5 (10.8 to 21.2)	0.2 (0.1 to 0.3)	7.2 (7.2 to 7.2)	78.5 (65.9 to 94.1)	19.7 (15.0 to 25.7)	39.8 (27.9 to 54.6)	0.5 (0.2 to 0.8)	18.6 (18.6 to 18.6)
Mali	2007	31.9 (26.7 to 38.4)	7.0 (5.3 to 9.1)	16.3 (11.6 to 22.4)	0.2 (0.1 to 0.4)	8.4 (8.4 to 8.4)	82.0 (68.8 to 98.8)	17.9 (13.6 to 23.4)	42.1 (30.0 to 57.7)	0.5 (0.2 to 0.9)	21.5 (21.5 to 21.5)
Mali	2008	33.9 (28.6 to 40.5)	6.1 (4.6 to 7.9)	17.1 (12.2 to 23.2)	0.2 (0.1 to 0.4)	10.5 (10.5 to 10.5)	87.2 (73.7 to 104.1)	15.6 (11.9 to 20.3)	44.1 (31.3 to 59.8)	0.5 (0.2 to 1.0)	27.1 (27.1 to 27.1)
Mali	2009	33.3 (27.9 to 40.2)	5.4 (4.1 to 7.1)	17.8 (12.8 to 24.2)	0.2 (0.1 to 0.4)	9.8 (9.8 to 9.8)	85.6 (71.7 to 103.4)	13.8 (10.5 to 18.2)	45.8 (33.0 to 62.3)	0.6 (0.3 to 1.1)	25.3 (25.3 to 25.3)
Mali	2010	36.9 (31.6 to 44.1)	4.9 (3.7 to 6.4)	18.0 (12.9 to 24.8)	0.3 (0.1 to 0.5)	13.8 (13.8 to 13.8)	95.0 (81.2 to 113.4)	12.5 (9.6 to 16.5)	46.4 (33.2 to 63.7)	0.7 (0.3 to 1.3)	35.4 (35.4 to 35.4)
Mali	2011	36.0 (30.7 to 43.1)	4.7 (3.6 to 6.1)	17.9 (12.5 to 24.8)	0.4 (0.2 to 0.7)	13.0 (13.0 to 13.0)	92.6 (78.9 to 111.0)	12.2 (9.4 to 15.8)	46.0 (32.3 to 63.8)	0.9 (0.4 to 1.7)	33.4 (33.4 to 33.4)
Mali	2012	34.0 (28.8 to 41.0)	4.5 (3.5 to 5.9)	17.3 (12.2 to 24.3)	0.5 (0.2 to 0.9)	11.7 (11.7 to 11.7)	87.5 (74.0 to 105.6)	11.6 (8.9 to 15.2)	44.6 (31.4 to 62.4)	1.3 (0.6 to 2.4)	30.0 (30.0 to 30.0)
Mali	2013	33.1 (27.9 to 39.7)	5.2 (4.0 to 6.8)	16.5 (11.6 to 23.2)	0.7 (0.3 to 1.3)	10.7 (10.7 to 10.7)	85.2 (71.8 to 102.3)	13.3 (10.2 to 17.4)	42.5 (30.0 to 59.7)	1.7 (0.8 to 3.2)	27.6 (27.6 to 27.6)
Mali	2014	32.0 (27.4 to 37.9)	6.2 (4.7 to 8.0)	13.9 (9.9 to 19.5)	0.6 (0.3 to 1.1)	11.3 (11.3 to 11.3)	82.4 (70.6 to 97.5)	15.9 (12.2 to 20.6)	35.9 (25.4 to 50.1)	1.5 (0.7 to 2.8)	29.1 (29.1 to 29.1)
Mali	2015	31.8 (27.4 to 37.3)	7.1 (5.4 to 9.3)	12.7 (9.0 to 17.8)	0.5 (0.2 to 1.0)	11.5 (11.5 to 11.5)	81.9 (70.6 to 95.9)	18.3 (13.9 to 23.8)	32.8 (23.3 to 45.9)	1.3 (0.6 to 2.5)	29.5 (29.5 to 29.5)
Mali	2016	32.8 (28.4 to 37.8)	8.1 (6.1 to 10.5)	12.3 (8.7 to 17.2)	0.4 (0.2 to 0.9)	12.0 (12.0 to 12.0)	84.5 (73.1 to 97.2)	20.8 (15.6 to 27.0)	31.6 (22.3 to 44.2)	1.1 (0.5 to 2.2)	31.0 (31.0 to 31.0)
Mali	2017	32.6 (27.9 to 37.8)	8.5 (6.4 to 11.0)	12.4 (8.8 to 17.4)	0.5 (0.2 to 0.9)	11.2 (11.2 to 11.2)	83.9 (71.9 to 97.3)	21.8 (16.4 to 28.4)	32.0 (22.6 to 44.7)	1.2 (0.6 to 2.3)	28.9 (28.9 to 28.9)
Mali	2018	32.0 (27.5 to 37.5)	8.6 (6.5 to 11.2)	12.5 (8.8 to 17.5)	0.5 (0.2 to 0.9)	10.3 (10.3 to 10.4)	82.3 (70.8 to 96.6)	22.2 (16.7 to 28.8)	32.2 (22.7 to 45.0)	1.2 (0.6 to 2.4)	26.6 (26.6 to 26.6)
Mali	2019	32.1 (27.4 to 37.6)	8.8 (6.6 to 11.3)	12.6 (8.9 to 17.7)	0.5 (0.2 to 0.9)	10.2 (9.5 to 10.8)	82.6 (70.5 to 96.6)	22.6 (17.0 to 29.2)	32.5 (22.9 to 45.5)	1.2 (0.6 to 2.4)	26.3 (24.5 to 27.7)
Mali	2020	32.3 (27.5 to 37.8)	8.9 (6.7 to 11.5)	12.7 (9.0 to 17.8)	0.5 (0.2 to 1.0)	10.1 (9.3 to 11.0)	83.2 (70.8 to 97.3)	23.0 (17.3 to 29.7)	32.8 (23.1 to 45.7)	1.3 (0.6 to 2.5)	26.1 (23.9 to 28.2)
Mali	2021	32.6 (27.7 to 38.6)	9.1 (6.9 to 11.8)	13.0 (9.1 to 18.0)	0.5 (0.2 to 1.0)	10.1 (9.1 to 11.1)	84.0 (71.3 to 99.3)	23.4 (17.7 to 30.3)	33.3 (23.5 to 46.4)	1.3 (0.6 to 2.6)	26.0 (23.4 to 28.6)
Mali	2022	33.0 (28.1 to 38.9)	9.3 (7.0 to 12.0)	13.2 (9.3 to 18.3)	0.5 (0.2 to 1.0)	10.0 (8.9 to 11.2)	85.0 (72.3 to 100.0)	23.8 (18.0 to 30.8)	33.9 (23.9 to 47.0)	1.3 (0.6 to 2.6)	25.9 (23.0 to 28.9)

Country	Year	2018 US Dollars per capita					2018 PPP per capita				
		Health spending	Government health spending	Out-of-pocket health spending	Prepaid private health spending	Development assistance for health	Health spending	Government health spending	Out-of-pocket health spending	Prepaid private health spending	Development assistance for health
Mali	2023	33.3 (28.5 to 39.1)	9.4 (7.2 to 12.2)	13.3 (9.3 to 18.5)	0.5 (0.3 to 1.0)	10.0 (8.7 to 11.4)	85.7 (73.3 to 100.7)	24.3 (18.5 to 31.4)	34.3 (24.0 to 47.7)	1.4 (0.6 to 2.7)	25.7 (22.3 to 29.2)
Mali	2024	33.7 (28.5 to 39.8)	9.6 (7.3 to 12.4)	13.5 (9.5 to 18.8)	0.6 (0.3 to 1.1)	10.0 (8.6 to 11.5)	86.7 (73.4 to 102.5)	24.8 (18.8 to 32.0)	34.9 (24.4 to 48.5)	1.4 (0.7 to 2.8)	25.6 (22.0 to 29.6)
Mali	2025	34.1 (28.8 to 39.7)	9.8 (7.5 to 12.7)	13.8 (9.6 to 19.1)	0.6 (0.3 to 1.1)	9.9 (8.5 to 11.7)	87.7 (74.2 to 102.1)	25.3 (19.3 to 32.8)	35.4 (24.8 to 49.2)	1.5 (0.7 to 2.8)	25.5 (21.8 to 30.0)
Mali	2026	34.5 (29.1 to 41.0)	10.1 (7.7 to 13.1)	14.0 (9.8 to 19.4)	0.6 (0.3 to 1.1)	9.8 (8.3 to 11.6)	88.9 (75.0 to 105.6)	25.9 (19.8 to 33.7)	36.1 (25.2 to 49.8)	1.5 (0.7 to 2.9)	25.3 (21.4 to 29.9)
Mali	2027	35.0 (29.4 to 41.8)	10.3 (7.9 to 13.5)	14.3 (10.0 to 19.8)	0.6 (0.3 to 1.2)	9.8 (8.2 to 11.7)	90.1 (75.8 to 107.6)	26.6 (20.4 to 34.7)	36.8 (25.7 to 51.1)	1.6 (0.7 to 3.0)	25.1 (21.0 to 30.1)
Mali	2028	35.5 (29.7 to 41.8)	10.6 (8.1 to 13.8)	14.6 (10.2 to 20.3)	0.6 (0.3 to 1.2)	9.7 (8.1 to 11.7)	91.4 (76.3 to 107.7)	27.3 (20.8 to 35.6)	37.5 (26.1 to 52.2)	1.6 (0.7 to 3.1)	25.0 (20.9 to 30.1)
Mali	2029	36.0 (29.9 to 43.1)	10.9 (8.3 to 14.2)	14.8 (10.4 to 20.7)	0.6 (0.3 to 1.2)	9.6 (7.9 to 11.9)	92.7 (77.1 to 110.9)	28.0 (21.3 to 36.5)	38.2 (26.6 to 53.3)	1.6 (0.8 to 3.2)	24.8 (20.5 to 30.5)
Mali	2030	36.5 (30.4 to 43.6)	11.2 (8.5 to 14.6)	15.1 (10.5 to 21.2)	0.7 (0.3 to 1.3)	9.6 (7.8 to 11.8)	94.0 (78.2 to 112.2)	28.7 (21.9 to 37.5)	39.0 (27.1 to 54.5)	1.7 (0.8 to 3.3)	24.6 (20.2 to 30.3)
Mali	2031	37.1 (30.8 to 44.2)	11.5 (8.7 to 15.0)	15.4 (10.8 to 21.6)	0.7 (0.3 to 1.3)	9.5 (7.7 to 12.0)	95.5 (79.2 to 113.7)	29.5 (22.4 to 38.6)	39.7 (27.7 to 55.7)	1.7 (0.8 to 3.4)	24.5 (19.8 to 30.9)
Mali	2032	37.7 (31.3 to 45.2)	11.8 (8.9 to 15.4)	15.8 (11.0 to 22.1)	0.7 (0.3 to 1.4)	9.5 (7.7 to 12.0)	97.1 (80.6 to 116.3)	30.4 (23.0 to 39.6)	40.5 (28.2 to 56.9)	1.8 (0.8 to 3.5)	24.4 (19.7 to 30.8)
Mali	2033	38.4 (31.9 to 46.4)	12.1 (9.2 to 15.9)	16.1 (11.1 to 22.5)	0.7 (0.3 to 1.4)	9.4 (7.5 to 12.1)	98.7 (82.1 to 119.5)	31.3 (23.7 to 40.9)	41.4 (28.6 to 57.9)	1.9 (0.9 to 3.6)	24.2 (19.3 to 31.2)
Mali	2034	39.0 (32.5 to 47.1)	12.5 (9.4 to 16.4)	16.4 (11.3 to 22.9)	0.7 (0.3 to 1.4)	9.4 (7.4 to 12.2)	100.5 (83.6 to 121.2)	32.2 (24.3 to 42.2)	42.2 (29.2 to 59.1)	1.9 (0.9 to 3.7)	24.2 (19.0 to 31.5)
Mali	2035	39.8 (32.8 to 48.0)	12.9 (9.7 to 16.9)	16.7 (11.6 to 23.5)	0.8 (0.4 to 1.5)	9.4 (7.3 to 12.4)	102.3 (84.3 to 123.6)	33.1 (25.0 to 43.4)	43.1 (29.8 to 60.4)	2.0 (0.9 to 3.8)	24.1 (18.8 to 31.9)
Mali	2036	40.5 (33.7 to 48.8)	13.3 (10.0 to 17.4)	17.1 (11.8 to 24.0)	0.8 (0.4 to 1.5)	9.4 (7.2 to 12.5)	104.2 (86.6 to 125.6)	34.2 (25.8 to 44.7)	43.9 (30.4 to 61.8)	2.0 (0.9 to 3.9)	24.1 (18.6 to 32.1)
Mali	2037	41.3 (34.0 to 49.8)	13.7 (10.3 to 18.0)	17.4 (12.0 to 24.6)	0.8 (0.4 to 1.6)	9.3 (7.1 to 12.7)	106.2 (87.4 to 128.1)	35.2 (26.6 to 46.2)	44.8 (31.0 to 63.3)	2.1 (1.0 to 4.0)	24.0 (18.3 to 32.6)
Mali	2038	42.1 (34.7 to 50.1)	14.1 (10.7 to 18.6)	17.8 (12.3 to 25.2)	0.8 (0.4 to 1.6)	9.3 (7.1 to 12.8)	108.3 (89.2 to 129.0)	36.4 (27.4 to 47.9)	45.8 (31.6 to 64.9)	2.1 (1.0 to 4.2)	24.0 (18.3 to 33.1)
Mali	2039	42.9 (35.4 to 52.1)	14.6 (11.0 to 19.2)	18.2 (12.5 to 25.8)	0.9 (0.4 to 1.7)	9.3 (7.0 to 12.7)	110.4 (91.2 to 134.1)	37.5 (28.4 to 49.5)	46.7 (32.2 to 66.3)	2.2 (1.0 to 4.3)	24.0 (18.1 to 32.7)
Mali	2040	43.8 (36.3 to 52.7)	15.0 (11.4 to 19.8)	18.5 (12.7 to 26.3)	0.9 (0.4 to 1.7)	9.3 (7.0 to 12.9)	112.7 (93.4 to 135.7)	38.7 (29.4 to 51.1)	47.7 (32.8 to 67.6)	2.3 (1.1 to 4.4)	24.0 (18.0 to 33.2)
Mali	2041	44.7 (36.5 to 54.3)	15.5 (11.8 to 20.5)	18.9 (13.0 to 26.8)	0.9 (0.4 to 1.8)	9.3 (7.0 to 13.4)	115.0 (93.9 to 139.6)	40.0 (30.4 to 52.7)	48.7 (33.4 to 69.1)	2.3 (1.1 to 4.5)	24.0 (17.9 to 34.4)
Mali	2042	45.6 (38.1 to 55.2)	16.0 (12.1 to 21.1)	19.3 (13.3 to 27.4)	0.9 (0.4 to 1.8)	9.4 (6.8 to 13.7)	117.4 (98.0 to 142.2)	41.2 (31.3 to 54.3)	49.7 (34.1 to 70.6)	2.4 (1.1 to 4.7)	24.1 (17.6 to 35.1)
Mali	2043	46.6 (38.6 to 57.5)	16.5 (12.5 to 21.8)	19.7 (13.5 to 28.1)	1.0 (0.5 to 1.9)	9.4 (6.8 to 13.8)	120.0 (99.3 to 147.9)	42.6 (32.2 to 56.0)	50.8 (34.8 to 72.3)	2.5 (1.2 to 4.8)	24.1 (17.5 to 35.5)

Country	Year	2018 US Dollars per capita					2018 PPP per capita				
		Health spending	Government health spending	Out-of-pocket health spending	Prepaid private health spending	Development assistance for health	Health spending	Government health spending	Out-of-pocket health spending	Prepaid private health spending	Development assistance for health
Mali	2044	47.7 (39.0 to 57.9)	17.1 (12.9 to 22.5)	20.2 (13.8 to 28.8)	1.0 (0.5 to 1.9)	9.4 (6.8 to 14.0)	122.6 (100.2 to 149.1)	44.0 (33.3 to 58.0)	51.9 (35.6 to 74.0)	2.5 (1.2 to 4.9)	24.2 (17.4 to 36.1)
Mali	2045	48.7 (39.5 to 60.4)	17.6 (13.3 to 23.3)	20.6 (14.1 to 29.4)	1.0 (0.5 to 2.0)	9.4 (6.7 to 14.4)	125.3 (101.7 to 155.4)	45.4 (34.3 to 60.0)	53.0 (36.3 to 75.7)	2.6 (1.2 to 5.1)	24.3 (17.3 to 37.0)
Mali	2046	49.8 (40.6 to 60.9)	18.2 (13.7 to 24.1)	21.0 (14.4 to 30.1)	1.0 (0.5 to 2.0)	9.5 (6.7 to 14.7)	128.1 (104.5 to 156.8)	46.8 (35.3 to 61.9)	54.2 (37.0 to 77.4)	2.7 (1.3 to 5.2)	24.4 (17.3 to 38.0)
Mali	2047	50.9 (41.4 to 63.5)	18.8 (14.1 to 24.9)	21.5 (14.7 to 30.7)	1.1 (0.5 to 2.1)	9.5 (6.7 to 15.3)	131.0 (106.4 to 163.3)	48.3 (36.3 to 64.2)	55.3 (37.9 to 79.1)	2.8 (1.3 to 5.4)	24.5 (17.3 to 39.4)
Mali	2048	52.1 (42.2 to 64.5)	19.4 (14.5 to 25.7)	22.0 (15.0 to 31.4)	1.1 (0.5 to 2.2)	9.6 (6.7 to 15.7)	134.0 (108.5 to 166.1)	49.8 (37.4 to 66.2)	56.5 (38.7 to 80.8)	2.9 (1.3 to 5.6)	24.8 (17.3 to 40.5)
Mali	2049	53.2 (43.4 to 65.9)	20.0 (14.9 to 26.6)	22.4 (15.3 to 32.1)	1.1 (0.5 to 2.2)	9.7 (6.7 to 16.3)	137.0 (111.7 to 169.5)	51.3 (38.4 to 68.6)	57.8 (39.5 to 82.5)	2.9 (1.4 to 5.7)	24.9 (17.2 to 41.9)
Mali	2050	54.4 (43.9 to 68.3)	20.6 (15.3 to 27.5)	22.9 (15.7 to 32.8)	1.2 (0.6 to 2.3)	9.8 (6.7 to 16.6)	140.1 (113.1 to 175.6)	52.9 (39.4 to 70.8)	59.0 (40.3 to 84.3)	3.0 (1.4 to 5.9)	25.2 (17.2 to 42.7)
Malta	1995	980.9 (926.2 to 1036.8)	720.6 (677.0 to 766.0)	232.5 (204.5 to 263.4)	27.8 (16.7 to 42.9)	0.0 (0.0 to 0.0)	1415.2 (1336.2 to 1495.7)	1039.6 (976.7 to 1105.1)	335.4 (295.0 to 380.0)	40.1 (24.1 to 61.9)	0.0 (0.0 to 0.0)
Malta	1996	1035.4 (989.1 to 1080.3)	759.5 (727.0 to 796.7)	247.9 (224.6 to 273.2)	28.0 (17.4 to 42.1)	0.0 (0.0 to 0.0)	1493.8 (1426.9 to 1558.5)	1095.7 (1048.8 to 1149.3)	357.7 (324.1 to 394.1)	40.4 (25.1 to 60.7)	0.0 (0.0 to 0.0)
Malta	1997	1103.7 (1060.9 to 1148.4)	811.3 (778.6 to 847.7)	265.0 (243.2 to 289.0)	27.5 (17.4 to 41.1)	0.0 (0.0 to 0.0)	1592.3 (1530.5 to 1656.8)	1170.4 (1123.2 to 1223.0)	382.2 (350.8 to 416.9)	39.6 (25.1 to 59.3)	0.0 (0.0 to 0.0)
Malta	1998	1167.3 (1125.4 to 1214.3)	849.9 (816.7 to 888.2)	291.9 (270.1 to 315.0)	25.5 (16.1 to 38.3)	0.0 (0.0 to 0.0)	1684.1 (1623.6 to 1751.9)	1226.2 (1178.3 to 1281.3)	421.1 (389.7 to 454.5)	36.8 (23.2 to 55.3)	0.0 (0.0 to 0.0)
Malta	1999	1222.0 (1177.7 to 1269.8)	880.4 (848.1 to 918.8)	320.1 (297.5 to 344.3)	21.4 (13.7 to 32.5)	0.0 (0.0 to 0.0)	1762.9 (1699.1 to 1831.9)	1270.2 (1223.5 to 1325.6)	461.8 (429.1 to 496.7)	30.9 (19.8 to 46.8)	0.0 (0.0 to 0.0)
Malta	2000	1298.7 (1253.4 to 1343.8)	931.1 (897.8 to 968.4)	350.9 (328.4 to 375.8)	16.7 (10.7 to 24.9)	0.0 (0.0 to 0.0)	1873.6 (1808.2 to 1938.7)	1343.2 (1295.3 to 1397.1)	506.3 (473.7 to 542.1)	24.1 (15.4 to 35.9)	0.0 (0.0 to 0.0)
Malta	2001	1367.4 (1323.1 to 1410.0)	962.2 (928.2 to 997.3)	388.4 (364.6 to 413.2)	16.8 (11.0 to 24.7)	0.0 (0.0 to 0.0)	1972.8 (1908.8 to 2034.2)	1388.2 (1339.2 to 1438.8)	560.4 (525.9 to 596.1)	24.2 (15.9 to 35.6)	0.0 (0.0 to 0.0)
Malta	2002	1497.0 (1450.9 to 1542.1)	1049.7 (1013.9 to 1085.9)	428.0 (404.6 to 453.0)	19.3 (12.7 to 27.8)	0.0 (0.0 to 0.0)	2159.7 (2093.2 to 2224.8)	1514.4 (1462.8 to 1566.6)	617.5 (583.8 to 653.5)	27.8 (18.3 to 40.1)	0.0 (0.0 to 0.0)
Malta	2003	1606.2 (1560.7 to 1650.2)	1120.8 (1085.7 to 1157.9)	459.7 (435.4 to 486.3)	25.7 (17.6 to 36.3)	0.0 (0.0 to 0.0)	2317.3 (2251.7 to 2380.7)	1616.9 (1566.3 to 1670.5)	663.2 (628.2 to 701.5)	37.1 (25.5 to 52.4)	0.0 (0.0 to 0.0)
Malta	2004	1682.9 (1637.1 to 1729.2)	1167.8 (1134.2 to 1206.8)	482.4 (457.2 to 507.8)	32.8 (23.1 to 44.9)	0.0 (0.0 to 0.0)	2427.9 (2361.8 to 2494.7)	1684.7 (1636.2 to 1741.0)	695.9 (659.5 to 732.6)	47.3 (33.3 to 64.8)	0.0 (0.0 to 0.0)
Malta	2005	1792.9 (1747.7 to 1838.7)	1234.4 (1199.4 to 1271.9)	520.1 (494.1 to 544.5)	38.3 (27.2 to 50.4)	0.0 (0.0 to 0.0)	2586.6 (2521.3 to 2652.6)	1780.9 (1730.3 to 1834.9)	750.4 (712.9 to 785.6)	55.3 (39.2 to 72.6)	0.0 (0.0 to 0.0)
Malta	2006	1819.4 (1775.0 to 1865.9)	1234.9 (1199.1 to 1270.8)	544.5 (518.2 to 570.6)	40.0 (28.9 to 51.7)	0.0 (0.0 to 0.0)	2624.8 (2560.8 to 2691.8)	1781.6 (1729.9 to 1833.3)	785.5 (747.7 to 823.2)	57.7 (41.7 to 74.6)	0.0 (0.0 to 0.0)
Malta	2007	1813.2 (1765.1 to 1861.5)	1210.4 (1175.4 to 1247.3)	562.1 (535.6 to 589.6)	40.7 (29.9 to 52.5)	0.0 (0.0 to 0.0)	2615.9 (2546.5 to 2685.6)	1746.2 (1695.7 to 1799.5)	811.0 (772.7 to 850.5)	58.7 (43.2 to 75.8)	0.0 (0.0 to 0.0)
Malta	2008	1799.5 (1752.0 to 1843.9)	1180.5 (1143.5 to 1217.7)	577.4 (550.4 to 604.9)	41.7 (31.2 to 53.8)	0.0 (0.0 to 0.0)	2596.1 (2527.6 to 2660.1)	1703.0 (1649.7 to 1756.8)	832.9 (794.0 to 872.6)	60.2 (45.0 to 77.7)	0.0 (0.0 to 0.0)

Country	Year	2018 US Dollars per capita					2018 PPP per capita				
		Health spending	Government health spending	Out-of-pocket health spending	Prepaid private health spending	Development assistance for health	Health spending	Government health spending	Out-of-pocket health spending	Prepaid private health spending	Development assistance for health
Malta	2009	1767.1 (1720.1 to 1813.4)	1146.1 (1111.3 to 1180.6)	578.4 (552.3 to 605.0)	42.7 (32.4 to 54.9)	0.0 (0.0 to 0.0)	2549.4 (2481.5 to 2616.1)	1653.4 (1603.2 to 1703.2)	834.4 (796.8 to 872.9)	61.5 (46.7 to 79.2)	0.0 (0.0 to 0.0)
Malta	2010	1845.6 (1795.4 to 1891.7)	1196.7 (1161.1 to 1232.9)	604.4 (577.8 to 631.4)	44.5 (33.8 to 57.4)	0.0 (0.0 to 0.0)	2662.6 (2590.3 to 2729.2)	1726.5 (1675.1 to 1778.7)	872.0 (833.6 to 910.9)	64.2 (48.7 to 82.8)	0.0 (0.0 to 0.0)
Malta	2011	1919.0 (1868.5 to 1963.7)	1242.6 (1203.9 to 1279.3)	631.0 (603.5 to 658.4)	45.4 (34.6 to 58.1)	0.0 (0.0 to 0.0)	2768.5 (2695.6 to 2833.0)	1792.6 (1736.8 to 1845.5)	910.3 (870.6 to 949.8)	65.5 (49.9 to 83.9)	0.0 (0.0 to 0.0)
Malta	2012	2036.5 (1985.7 to 2085.2)	1317.9 (1279.0 to 1355.1)	672.1 (644.0 to 701.5)	46.5 (35.7 to 59.7)	0.0 (0.0 to 0.0)	2938.0 (2864.7 to 3008.3)	1901.4 (1845.2 to 1955.0)	969.6 (929.2 to 1012.1)	67.1 (51.5 to 86.1)	0.0 (0.0 to 0.0)
Malta	2013	2218.7 (2167.7 to 2272.8)	1415.2 (1377.4 to 1455.8)	755.1 (725.4 to 787.5)	48.4 (36.9 to 63.1)	0.0 (0.0 to 0.0)	3200.9 (3127.3 to 3279.0)	2041.7 (1987.2 to 2100.3)	1089.4 (1046.6 to 1136.2)	69.8 (53.3 to 91.0)	0.0 (0.0 to 0.0)
Malta	2014	2437.6 (2380.1 to 2493.4)	1517.6 (1477.6 to 1557.5)	867.6 (833.4 to 899.6)	52.4 (39.1 to 69.9)	0.0 (0.0 to 0.0)	3516.6 (3433.7 to 3597.2)	2189.4 (2131.7 to 2246.9)	1251.6 (1202.3 to 1297.8)	75.6 (56.4 to 100.9)	0.0 (0.0 to 0.0)
Malta	2015	2651.5 (2595.9 to 2708.7)	1634.9 (1593.0 to 1679.3)	959.7 (926.5 to 993.3)	56.9 (42.1 to 77.0)	0.0 (0.0 to 0.0)	3825.3 (3745.0 to 3907.8)	2358.7 (2298.2 to 2422.7)	1384.5 (1336.7 to 1433.0)	82.1 (60.7 to 111.0)	0.0 (0.0 to 0.0)
Malta	2016	2798.6 (2725.4 to 2879.0)	1742.9 (1682.8 to 1802.0)	994.9 (947.0 to 1037.3)	60.8 (42.9 to 85.1)	0.0 (0.0 to 0.0)	4037.5 (3931.8 to 4153.5)	2514.5 (2427.8 to 2599.8)	1435.3 (1366.2 to 1496.4)	87.7 (62.0 to 122.7)	0.0 (0.0 to 0.0)
Malta	2017	2944.3 (2865.4 to 3030.4)	1842.7 (1779.1 to 1904.8)	1037.8 (987.0 to 1085.6)	63.7 (45.0 to 89.3)	0.0 (0.0 to 0.0)	4247.7 (4133.9 to 4371.9)	2658.5 (2566.7 to 2748.0)	1497.3 (1423.9 to 1566.1)	91.9 (65.0 to 128.9)	0.0 (0.0 to 0.0)
Malta	2018	2996.6 (2911.6 to 3085.2)	1865.2 (1799.9 to 1927.7)	1066.6 (1011.3 to 1118.2)	64.9 (45.9 to 90.6)	0.0 (0.0 to 0.0)	4323.2 (4200.5 to 4451.0)	2690.8 (2596.6 to 2781.0)	1538.8 (1459.0 to 1613.2)	93.6 (66.2 to 130.7)	0.0 (0.0 to 0.0)
Malta	2019	3056.2 (2966.7 to 3151.2)	1892.5 (1824.7 to 1957.4)	1097.7 (1037.8 to 1156.1)	66.1 (46.6 to 92.2)	0.0 (0.0 to 0.0)	4409.2 (4280.0 to 4546.2)	2730.3 (2632.5 to 2823.9)	1583.6 (1497.1 to 1667.8)	95.3 (67.2 to 133.0)	0.0 (0.0 to 0.0)
Malta	2020	3116.8 (3021.4 to 3213.3)	1920.3 (1847.9 to 1990.0)	1129.2 (1067.8 to 1189.0)	67.3 (47.3 to 93.7)	0.0 (0.0 to 0.0)	4496.5 (4358.9 to 4635.8)	2770.4 (2665.9 to 2871.0)	1629.1 (1540.4 to 1715.3)	97.0 (68.3 to 135.1)	0.0 (0.0 to 0.0)
Malta	2021	3196.2 (3092.5 to 3299.4)	1961.1 (1886.0 to 2035.6)	1166.3 (1097.5 to 1233.6)	68.9 (48.6 to 95.9)	0.0 (0.0 to 0.0)	4611.1 (4461.5 to 4759.9)	2829.2 (2720.9 to 2936.8)	1682.5 (1583.3 to 1779.7)	99.4 (70.1 to 138.4)	0.0 (0.0 to 0.0)
Malta	2022	3277.6 (3162.0 to 3391.0)	2002.2 (1922.6 to 2081.7)	1204.9 (1132.8 to 1279.0)	70.6 (49.8 to 98.4)	0.0 (0.0 to 0.0)	4728.6 (4561.8 to 4892.2)	2888.5 (2773.6 to 3003.2)	1738.2 (1634.2 to 1845.2)	101.8 (71.9 to 141.9)	0.0 (0.0 to 0.0)
Malta	2023	3355.2 (3234.0 to 3471.9)	2039.9 (1956.5 to 2123.5)	1243.3 (1165.2 to 1320.8)	72.0 (50.8 to 100.6)	0.0 (0.0 to 0.0)	4840.5 (4665.6 to 5008.8)	2942.9 (2822.6 to 3063.5)	1793.6 (1681.0 to 1905.4)	103.9 (73.3 to 145.1)	0.0 (0.0 to 0.0)
Malta	2024	3443.7 (3320.2 to 3561.4)	2086.6 (1998.7 to 2171.6)	1283.4 (1197.5 to 1364.9)	73.7 (51.8 to 103.2)	0.0 (0.0 to 0.0)	4968.1 (4789.9 to 5137.9)	3010.3 (2883.5 to 3133.0)	1851.5 (1727.5 to 1969.1)	106.3 (74.8 to 148.8)	0.0 (0.0 to 0.0)
Malta	2025	3524.8 (3394.5 to 3653.5)	2127.4 (2034.0 to 2216.6)	1322.2 (1231.5 to 1407.6)	75.1 (52.8 to 105.1)	0.0 (0.0 to 0.0)	5085.1 (4897.1 to 5270.9)	3069.2 (2934.4 to 3197.9)	1907.6 (1776.6 to 2030.7)	108.3 (76.2 to 151.6)	0.0 (0.0 to 0.0)
Malta	2026	3614.2 (3469.0 to 3744.2)	2173.3 (2075.4 to 2267.4)	1364.1 (1258.4 to 1458.3)	76.7 (54.0 to 107.4)	0.0 (0.0 to 0.0)	5214.1 (5004.6 to 5401.6)	3135.4 (2994.2 to 3271.1)	1968.0 (1815.4 to 2103.8)	110.7 (77.9 to 154.9)	0.0 (0.0 to 0.0)
Malta	2027	3710.0 (3560.2 to 3865.8)	2223.1 (2120.4 to 2324.2)	1408.3 (1292.1 to 1518.8)	78.6 (55.3 to 109.9)	0.0 (0.0 to 0.0)	5352.3 (5136.3 to 5577.1)	3207.2 (3059.1 to 3353.0)	2031.7 (1864.0 to 2191.2)	113.4 (79.7 to 158.6)	0.0 (0.0 to 0.0)
Malta	2028	3810.7 (3649.1 to 3984.1)	2276.7 (2169.7 to 2378.4)	1453.5 (1322.9 to 1578.1)	80.6 (56.5 to 112.7)	0.0 (0.0 to 0.0)	5497.6 (5264.6 to 5747.8)	3284.5 (3130.2 to 3431.3)	2096.9 (1908.5 to 2276.7)	116.2 (81.5 to 162.5)	0.0 (0.0 to 0.0)
Malta	2029	3913.1 (3731.4 to 4103.6)	2331.3 (2222.5 to 2441.4)	1499.2 (1347.9 to 1644.8)	82.6 (57.9 to 115.5)	0.0 (0.0 to 0.0)	5645.4 (5383.3 to 5920.2)	3363.3 (3206.3 to 3522.1)	2162.9 (1944.6 to 2372.9)	119.2 (83.5 to 166.6)	0.0 (0.0 to 0.0)

Country	Year	2018 US Dollars per capita					2018 PPP per capita				
		Health spending	Government health spending	Out-of-pocket health spending	Prepaid private health spending	Development assistance for health	Health spending	Government health spending	Out-of-pocket health spending	Prepaid private health spending	Development assistance for health
Malta	2030	4018.6 (3810.8 to 4232.0)	2387.9 (2271.9 to 2506.8)	1545.8 (1378.9 to 1712.4)	84.8 (59.4 to 118.6)	0.0 (0.0 to 0.0)	5797.6 (5497.8 to 6105.5)	3445.0 (3277.7 to 3616.5)	2230.2 (1989.3 to 2470.5)	122.4 (85.7 to 171.1)	0.0 (0.0 to 0.0)
Malta	2031	4124.7 (3885.7 to 4365.1)	2445.0 (2327.3 to 2568.9)	1592.6 (1407.9 to 1783.9)	87.2 (61.0 to 121.8)	0.0 (0.0 to 0.0)	5950.6 (5605.9 to 6297.5)	3527.3 (3357.5 to 3706.1)	2297.6 (2031.1 to 2573.5)	125.7 (88.0 to 175.7)	0.0 (0.0 to 0.0)
Malta	2032	4232.8 (3978.8 to 4488.0)	2503.5 (2370.9 to 2634.5)	1639.7 (1435.8 to 1857.7)	89.6 (62.6 to 125.1)	0.0 (0.0 to 0.0)	6106.6 (5740.2 to 6474.8)	3611.8 (3420.5 to 3800.7)	2365.5 (2071.4 to 2680.0)	129.3 (90.4 to 180.5)	0.0 (0.0 to 0.0)
Malta	2033	4343.7 (4055.7 to 4635.1)	2564.3 (2427.4 to 2705.1)	1687.2 (1461.0 to 1923.7)	92.2 (64.4 to 128.7)	0.0 (0.0 to 0.0)	6266.6 (5851.1 to 6686.9)	3699.5 (3501.9 to 3902.6)	2434.1 (2107.7 to 2775.3)	133.0 (92.9 to 185.7)	0.0 (0.0 to 0.0)
Malta	2034	4456.4 (4155.0 to 4774.1)	2626.9 (2478.9 to 2773.7)	1734.7 (1491.4 to 1995.4)	94.8 (66.2 to 132.3)	0.0 (0.0 to 0.0)	6429.2 (5994.3 to 6887.5)	3789.8 (3576.2 to 4001.5)	2502.6 (2151.6 to 2878.7)	136.8 (95.5 to 190.9)	0.0 (0.0 to 0.0)
Malta	2035	4570.0 (4244.9 to 4904.4)	2690.3 (2531.6 to 2849.7)	1782.2 (1524.5 to 2073.7)	97.5 (68.0 to 136.0)	0.0 (0.0 to 0.0)	6593.0 (6124.0 to 7075.5)	3881.3 (3652.3 to 4111.2)	2571.1 (2199.4 to 2991.7)	140.6 (98.2 to 196.2)	0.0 (0.0 to 0.0)
Malta	2036	4686.0 (4340.3 to 5048.3)	2756.3 (2586.8 to 2924.5)	1829.5 (1552.3 to 2139.8)	100.2 (70.1 to 140.0)	0.0 (0.0 to 0.0)	6760.5 (6261.6 to 7283.0)	3976.4 (3731.9 to 4219.1)	2639.4 (2239.4 to 3087.0)	144.6 (101.1 to 202.0)	0.0 (0.0 to 0.0)
Malta	2037	4804.8 (4435.1 to 5195.9)	2824.9 (2639.1 to 3006.0)	1876.8 (1580.0 to 2213.6)	103.0 (72.2 to 144.5)	0.0 (0.0 to 0.0)	6931.7 (6398.4 to 7496.0)	4075.4 (3807.4 to 4336.6)	2707.7 (2279.4 to 3193.5)	148.6 (104.1 to 208.5)	0.0 (0.0 to 0.0)
Malta	2038	4923.1 (4536.3 to 5343.2)	2894.0 (2695.1 to 3091.0)	1923.3 (1606.2 to 2285.3)	105.8 (74.2 to 148.3)	0.0 (0.0 to 0.0)	7102.5 (6544.5 to 7708.5)	4175.0 (3888.1 to 4459.4)	2774.8 (2317.2 to 3296.9)	152.7 (107.1 to 213.9)	0.0 (0.0 to 0.0)
Malta	2039	5041.8 (4628.2 to 5483.2)	2964.0 (2753.1 to 3183.5)	1969.2 (1635.6 to 2356.7)	108.6 (76.3 to 152.2)	0.0 (0.0 to 0.0)	7273.7 (6677.0 to 7910.6)	4276.1 (3971.8 to 4592.8)	2841.0 (2359.6 to 3400.0)	156.7 (110.1 to 219.6)	0.0 (0.0 to 0.0)
Malta	2040	5162.6 (4717.3 to 5616.1)	3036.3 (2810.9 to 3271.0)	2014.8 (1663.3 to 2427.3)	111.5 (78.4 to 156.4)	0.0 (0.0 to 0.0)	7448.0 (6805.5 to 8102.3)	4380.4 (4055.2 to 4719.0)	2906.7 (2399.6 to 3501.9)	160.9 (113.2 to 225.7)	0.0 (0.0 to 0.0)
Malta	2041	5284.5 (4823.4 to 5766.1)	3110.2 (2868.1 to 3358.0)	2059.8 (1691.0 to 2496.8)	114.4 (80.3 to 160.7)	0.0 (0.0 to 0.0)	7623.8 (6958.6 to 8318.7)	4487.1 (4137.8 to 4844.5)	2971.6 (2439.6 to 3602.0)	165.1 (115.8 to 231.9)	0.0 (0.0 to 0.0)
Malta	2042	5405.4 (4929.0 to 5898.0)	3184.3 (2929.7 to 3441.8)	2103.7 (1719.6 to 2562.1)	117.4 (82.1 to 164.8)	0.0 (0.0 to 0.0)	7798.3 (7111.0 to 8508.9)	4594.0 (4226.7 to 4965.5)	3035.0 (2480.8 to 3696.3)	169.3 (118.4 to 237.8)	0.0 (0.0 to 0.0)
Malta	2043	5530.5 (5035.3 to 6038.4)	3262.8 (2987.9 to 3540.2)	2147.4 (1753.2 to 2630.4)	120.4 (83.9 to 169.0)	0.0 (0.0 to 0.0)	7978.8 (7264.4 to 8711.4)	4707.1 (4310.6 to 5107.4)	3098.0 (2529.3 to 3794.8)	173.7 (121.1 to 243.9)	0.0 (0.0 to 0.0)
Malta	2044	5656.8 (5149.2 to 6210.7)	3342.9 (3052.5 to 3637.1)	2190.4 (1787.9 to 2691.4)	123.5 (86.2 to 173.3)	0.0 (0.0 to 0.0)	8160.9 (7428.7 to 8960.1)	4822.7 (4403.7 to 5247.1)	3160.0 (2579.3 to 3882.9)	178.2 (124.4 to 250.0)	0.0 (0.0 to 0.0)
Malta	2045	5782.7 (5228.9 to 6402.9)	3424.2 (3124.9 to 3739.5)	2231.9 (1813.9 to 2748.7)	126.7 (88.5 to 177.1)	0.0 (0.0 to 0.0)	8342.6 (7543.6 to 9237.4)	4940.0 (4508.2 to 5394.9)	3219.9 (2616.9 to 3965.4)	182.7 (127.7 to 255.5)	0.0 (0.0 to 0.0)
Malta	2046	5901.1 (5341.1 to 6500.9)	3500.8 (3171.3 to 3835.0)	2270.6 (1837.7 to 2801.0)	129.7 (90.7 to 181.1)	0.0 (0.0 to 0.0)	8513.4 (7705.4 to 9378.7)	5050.6 (4575.2 to 5532.7)	3275.7 (2651.2 to 4040.9)	187.1 (130.9 to 261.3)	0.0 (0.0 to 0.0)
Malta	2047	6021.4 (5473.5 to 6645.2)	3580.3 (3232.8 to 3920.9)	2308.4 (1863.8 to 2856.9)	132.8 (92.9 to 185.6)	0.0 (0.0 to 0.0)	8687.0 (7896.5 to 9586.9)	5165.2 (4663.9 to 5656.6)	3330.3 (2688.9 to 4121.6)	191.5 (134.0 to 267.7)	0.0 (0.0 to 0.0)
Malta	2048	6141.0 (5553.6 to 6777.4)	3659.2 (3308.1 to 4020.9)	2346.0 (1897.0 to 2912.5)	135.8 (94.9 to 190.0)	0.0 (0.0 to 0.0)	8859.5 (8012.0 to 9777.6)	5279.1 (4772.6 to 5800.8)	3384.5 (2736.7 to 4201.8)	195.9 (137.0 to 274.1)	0.0 (0.0 to 0.0)
Malta	2049	6259.9 (5695.5 to 6954.2)	3737.5 (3364.2 to 4116.5)	2383.5 (1927.6 to 2968.1)	138.8 (96.9 to 194.4)	0.0 (0.0 to 0.0)	9031.0 (8216.7 to 10032.7)	5392.0 (4853.5 to 5938.7)	3438.6 (2780.9 to 4282.0)	200.3 (139.8 to 280.5)	0.0 (0.0 to 0.0)
Malta	2050	6377.3 (5766.6 to 7083.0)	3815.0 (3422.0 to 4213.0)	2420.5 (1949.5 to 3022.8)	141.8 (98.8 to 198.6)	0.0 (0.0 to 0.0)	9200.4 (8319.3 to 10218.5)	5503.9 (4936.8 to 6078.1)	3492.0 (2812.4 to 4360.9)	204.6 (142.5 to 286.5)	0.0 (0.0 to 0.0)

		2018 US Dollars per capita					2018 PPP per capita				
Country	Year	Health spending	Government health spending	Out-of-pocket health spending	Prepaid private health spending	Development assistance for health	Health spending	Government health spending	Out-of-pocket health spending	Prepaid private health spending	Development assistance for health
Marshall Islands	1995	498.3 (447.3 to 552.6)	398.0 (351.9 to 448.4)	66.3 (49.7 to 84.8)	24.1 (12.4 to 42.8)	10.0 (10.0 to 10.0)	488.3 (438.3 to 541.5)	390.0 (344.8 to 439.4)	64.9 (48.7 to 83.1)	23.6 (12.2 to 41.9)	9.8 (9.8 to 9.8)
Marshall Islands	1996	435.7 (396.1 to 476.2)	338.4 (303.4 to 375.5)	57.7 (44.1 to 73.7)	22.0 (11.8 to 37.2)	17.5 (17.5 to 17.5)	426.9 (388.1 to 466.6)	331.6 (297.3 to 367.9)	56.6 (43.2 to 72.2)	21.6 (11.5 to 36.5)	17.2 (17.2 to 17.2)
Marshall Islands	1997	411.6 (375.9 to 446.5)	317.2 (286.8 to 347.1)	52.5 (40.9 to 66.9)	21.5 (12.0 to 35.8)	20.4 (20.4 to 20.4)	403.3 (368.4 to 437.5)	310.8 (281.0 to 340.1)	51.4 (40.1 to 65.5)	21.1 (11.7 to 35.1)	20.0 (20.0 to 20.0)
Marshall Islands	1998	415.5 (381.9 to 450.4)	313.3 (286.1 to 341.7)	49.7 (38.9 to 62.8)	21.6 (12.2 to 36.2)	30.9 (30.9 to 30.9)	407.2 (374.2 to 441.4)	307.0 (280.4 to 334.8)	48.8 (38.1 to 61.5)	21.2 (11.9 to 35.4)	30.3 (30.3 to 30.3)
Marshall Islands	1999	508.8 (475.1 to 543.8)	324.2 (296.1 to 351.9)	49.2 (38.6 to 62.3)	22.4 (12.7 to 37.9)	113.0 (113.0 to 113.0)	498.6 (465.5 to 532.9)	317.6 (290.2 to 344.9)	48.2 (37.8 to 61.1)	22.0 (12.4 to 37.1)	110.8 (110.8 to 110.8)
Marshall Islands	2000	459.5 (424.5 to 497.2)	352.5 (322.2 to 382.5)	51.2 (39.9 to 65.0)	24.8 (13.9 to 41.3)	30.9 (30.9 to 30.9)	450.2 (416.0 to 487.2)	345.5 (315.7 to 374.8)	50.2 (39.1 to 63.7)	24.3 (13.6 to 40.4)	30.3 (30.3 to 30.3)
Marshall Islands	2001	443.2 (409.3 to 481.8)	355.3 (324.7 to 387.0)	52.9 (41.2 to 66.5)	25.3 (14.4 to 41.6)	9.7 (9.7 to 9.7)	434.3 (401.0 to 472.1)	348.2 (318.2 to 379.2)	51.9 (40.4 to 65.2)	24.8 (14.1 to 40.7)	9.5 (9.5 to 9.5)
Marshall Islands	2002	436.0 (402.4 to 474.9)	334.9 (306.2 to 364.5)	54.8 (42.8 to 68.3)	24.3 (13.9 to 39.9)	22.0 (22.0 to 22.0)	427.2 (394.3 to 465.4)	328.2 (300.0 to 357.2)	53.7 (42.0 to 66.9)	23.8 (13.7 to 39.1)	21.5 (21.5 to 21.5)
Marshall Islands	2003	461.5 (430.7 to 499.1)	317.9 (290.6 to 347.0)	60.8 (47.9 to 75.6)	23.3 (13.2 to 38.0)	59.5 (59.5 to 59.5)	452.2 (422.1 to 489.1)	311.5 (284.7 to 340.0)	59.6 (46.9 to 74.1)	22.8 (13.0 to 37.2)	58.3 (58.3 to 58.3)
Marshall Islands	2004	634.1 (603.5 to 670.6)	309.8 (283.9 to 338.6)	63.3 (50.4 to 78.6)	22.5 (12.6 to 36.6)	238.5 (238.5 to 238.5)	621.4 (591.4 to 657.1)	303.6 (278.2 to 331.8)	62.0 (49.4 to 77.0)	22.1 (12.3 to 35.8)	233.8 (233.8 to 233.8)
Marshall Islands	2005	729.1 (698.1 to 766.0)	311.3 (285.8 to 339.7)	64.1 (51.1 to 80.1)	22.1 (12.4 to 36.0)	331.6 (331.6 to 331.6)	714.5 (684.0 to 750.6)	305.1 (280.1 to 332.9)	62.8 (50.1 to 78.5)	21.7 (12.1 to 35.3)	324.9 (324.9 to 324.9)
Marshall Islands	2006	559.2 (528.4 to 598.1)	317.3 (289.5 to 345.0)	64.2 (51.5 to 80.7)	21.9 (12.2 to 35.2)	155.8 (155.8 to 155.8)	548.0 (517.8 to 586.1)	310.9 (283.7 to 338.1)	63.0 (50.5 to 79.1)	21.4 (12.0 to 34.5)	152.7 (152.7 to 152.7)
Marshall Islands	2007	575.0 (542.1 to 612.3)	330.4 (301.5 to 361.4)	64.6 (51.8 to 80.8)	22.0 (12.1 to 35.5)	158.0 (158.0 to 158.0)	563.5 (531.2 to 600.0)	323.8 (295.5 to 354.1)	63.3 (50.7 to 79.2)	21.6 (11.8 to 34.8)	154.8 (154.8 to 154.8)
Marshall Islands	2008	581.6 (549.8 to 617.4)	313.3 (285.3 to 342.8)	63.6 (51.1 to 78.9)	20.8 (11.4 to 33.9)	183.8 (183.8 to 183.8)	569.9 (538.7 to 605.0)	307.0 (279.5 to 335.9)	62.3 (50.1 to 77.3)	20.4 (11.2 to 33.2)	180.1 (180.1 to 180.1)
Marshall Islands	2009	581.2 (549.2 to 615.4)	303.0 (275.8 to 332.1)	64.9 (52.1 to 80.1)	20.0 (11.5 to 32.2)	193.2 (193.2 to 193.2)	569.5 (538.2 to 603.0)	297.0 (270.3 to 325.4)	63.6 (51.1 to 78.5)	19.6 (11.2 to 31.6)	189.3 (189.3 to 189.3)
Marshall Islands	2010	416.2 (383.6 to 450.3)	301.1 (272.9 to 329.8)	69.8 (55.9 to 86.0)	19.4 (11.0 to 32.0)	25.8 (25.8 to 25.8)	407.9 (375.9 to 441.3)	295.1 (267.4 to 323.2)	68.4 (54.8 to 84.3)	19.0 (10.8 to 31.4)	25.3 (25.3 to 25.3)
Marshall Islands	2011	435.8 (403.1 to 472.1)	314.9 (285.9 to 345.8)	71.8 (57.7 to 88.0)	19.2 (10.6 to 32.0)	30.0 (30.0 to 30.0)	427.1 (395.0 to 462.6)	308.5 (280.2 to 338.8)	70.3 (56.6 to 86.3)	18.8 (10.4 to 31.4)	29.4 (29.4 to 29.4)
Marshall Islands	2012	585.2 (549.8 to 622.0)	334.2 (305.4 to 366.8)	72.5 (58.4 to 89.0)	19.2 (10.3 to 32.7)	159.2 (159.2 to 159.2)	573.4 (538.8 to 609.5)	327.5 (299.3 to 359.4)	71.1 (57.2 to 87.2)	18.8 (10.1 to 32.1)	156.0 (156.0 to 156.0)
Marshall Islands	2013	459.4 (423.7 to 495.3)	348.8 (317.5 to 381.7)	72.8 (58.0 to 89.9)	19.2 (10.2 to 33.5)	18.6 (18.6 to 18.6)	450.1 (415.2 to 485.4)	341.8 (311.1 to 374.1)	71.3 (56.9 to 88.1)	18.8 (10.0 to 32.9)	18.2 (18.2 to 18.2)
Marshall Islands	2014	540.6 (504.1 to 578.7)	349.8 (318.7 to 381.9)	72.7 (57.3 to 91.3)	18.9 (9.8 to 33.6)	99.1 (99.1 to 99.1)	529.7 (494.0 to 567.1)	342.8 (312.3 to 374.3)	71.2 (56.1 to 89.5)	18.5 (9.6 to 32.9)	97.1 (97.1 to 97.1)
Marshall Islands	2015	540.0 (499.4 to 586.1)	383.4 (346.8 to 422.0)	74.4 (57.4 to 94.6)	19.8 (10.3 to 34.8)	62.4 (62.4 to 62.4)	529.2 (489.4 to 574.3)	375.7 (339.8 to 413.5)	72.9 (56.2 to 92.7)	19.4 (10.1 to 34.1)	61.2 (61.2 to 61.2)

		2018 US Dollars per capita					2018 PPP per capita				
Country	Year	Health spending	Government health spending	Out-of-pocket health spending	Prepaid private health spending	Development assistance for health	Health spending	Government health spending	Out-of-pocket health spending	Prepaid private health spending	Development assistance for health
Marshall Islands	2016	528.9 (479.6 to 585.8)	419.7 (374.3 to 469.9)	75.4 (57.2 to 96.8)	20.6 (10.5 to 37.1)	13.3 (13.3 to 13.3)	518.3 (470.0 to 574.0)	411.3 (366.8 to 460.5)	73.9 (56.1 to 94.8)	20.2 (10.3 to 36.4)	13.0 (13.0 to 13.0)
Marshall Islands	2017	628.0 (568.5 to 691.4)	489.2 (435.8 to 546.8)	76.7 (58.3 to 98.6)	21.5 (10.8 to 39.1)	40.7 (40.7 to 40.7)	615.4 (557.1 to 677.5)	479.3 (427.0 to 535.8)	75.1 (57.1 to 96.6)	21.1 (10.6 to 38.3)	39.9 (39.9 to 39.9)
Marshall Islands	2018	624.8 (564.7 to 687.9)	488.5 (434.9 to 550.7)	76.5 (58.2 to 98.3)	21.4 (10.7 to 38.9)	38.4 (38.4 to 38.4)	612.3 (553.4 to 674.0)	478.7 (426.2 to 539.7)	75.0 (57.0 to 96.3)	21.0 (10.5 to 38.2)	37.6 (37.6 to 37.7)
Marshall Islands	2019	636.9 (572.8 to 704.6)	499.2 (442.4 to 564.8)	77.3 (58.9 to 98.5)	21.7 (10.9 to 39.5)	38.7 (36.1 to 40.9)	624.1 (561.3 to 690.5)	489.2 (433.5 to 553.5)	75.8 (57.7 to 96.6)	21.3 (10.7 to 38.7)	37.9 (35.4 to 40.1)
Marshall Islands	2020	647.7 (579.9 to 719.3)	508.2 (445.8 to 578.0)	78.4 (59.6 to 100.4)	21.9 (11.0 to 40.0)	39.2 (36.0 to 42.4)	634.7 (568.2 to 704.8)	498.0 (436.9 to 566.4)	76.8 (58.5 to 98.4)	21.5 (10.8 to 39.2)	38.5 (35.3 to 41.5)
Marshall Islands	2021	664.3 (596.4 to 740.2)	522.6 (458.3 to 593.3)	79.5 (60.3 to 102.1)	22.2 (11.2 to 40.5)	39.9 (35.9 to 44.1)	650.9 (584.4 to 725.4)	512.1 (449.1 to 581.4)	77.9 (59.1 to 100.1)	21.8 (10.9 to 39.7)	39.1 (35.2 to 43.2)
Marshall Islands	2022	680.2 (609.7 to 760.2)	536.3 (471.1 to 609.1)	80.5 (61.2 to 102.8)	22.6 (11.3 to 41.2)	40.8 (36.2 to 45.5)	666.5 (597.4 to 744.9)	525.6 (461.7 to 596.9)	78.9 (60.0 to 100.7)	22.1 (11.1 to 40.4)	40.0 (35.5 to 44.6)
Marshall Islands	2023	694.7 (621.7 to 774.5)	548.7 (482.0 to 624.7)	81.5 (61.7 to 104.5)	22.8 (11.5 to 41.9)	41.6 (36.2 to 47.2)	680.7 (609.2 to 759.0)	537.7 (472.3 to 612.2)	79.9 (60.5 to 102.4)	22.4 (11.2 to 41.0)	40.7 (35.5 to 46.2)
Marshall Islands	2024	713.1 (637.2 to 796.5)	564.6 (495.0 to 643.0)	82.8 (62.7 to 106.2)	23.2 (11.7 to 42.6)	42.4 (36.6 to 48.8)	698.7 (624.4 to 780.5)	553.3 (485.0 to 630.1)	81.1 (61.5 to 104.1)	22.8 (11.4 to 41.8)	41.5 (35.8 to 47.8)
Marshall Islands	2025	733.4 (654.0 to 819.2)	582.4 (509.5 to 663.3)	84.2 (63.7 to 108.1)	23.7 (11.9 to 43.4)	43.1 (36.8 to 50.5)	718.7 (640.9 to 802.8)	570.7 (499.3 to 650.0)	82.5 (62.4 to 106.0)	23.2 (11.7 to 42.6)	42.2 (36.1 to 49.5)
Marshall Islands	2026	753.2 (676.1 to 840.9)	599.4 (523.0 to 685.3)	85.8 (65.1 to 110.5)	24.3 (12.2 to 44.4)	43.8 (36.9 to 51.3)	738.1 (662.5 to 824.0)	587.4 (512.4 to 671.5)	84.0 (63.8 to 108.3)	23.8 (12.0 to 43.5)	42.9 (36.2 to 50.2)
Marshall Islands	2027	776.2 (692.1 to 870.8)	619.4 (539.2 to 711.6)	87.5 (66.4 to 112.5)	24.9 (12.6 to 45.5)	44.4 (37.3 to 52.9)	760.6 (678.2 to 853.3)	607.0 (528.3 to 697.3)	85.7 (65.0 to 110.3)	24.4 (12.3 to 44.6)	43.5 (36.5 to 51.8)
Marshall Islands	2028	800.1 (714.3 to 902.6)	640.1 (555.4 to 735.4)	89.2 (67.8 to 114.5)	25.6 (13.0 to 46.6)	45.2 (37.8 to 54.5)	784.0 (700.0 to 884.5)	627.2 (544.3 to 720.6)	87.4 (66.4 to 112.2)	25.1 (12.7 to 45.7)	44.3 (37.1 to 53.4)
Marshall Islands	2029	825.2 (735.6 to 932.2)	661.9 (576.3 to 761.2)	91.1 (68.9 to 117.7)	26.4 (13.4 to 47.9)	45.9 (37.9 to 56.2)	808.6 (720.8 to 913.5)	648.6 (564.7 to 745.9)	89.2 (67.5 to 115.3)	25.8 (13.1 to 46.9)	45.0 (37.1 to 55.1)
Marshall Islands	2030	849.7 (752.3 to 956.1)	683.0 (593.1 to 787.5)	93.0 (70.3 to 121.0)	27.1 (13.8 to 49.1)	46.6 (38.2 to 57.1)	832.7 (737.2 to 936.9)	669.3 (581.2 to 771.7)	91.1 (68.9 to 118.5)	26.6 (13.5 to 48.2)	45.7 (37.4 to 56.0)
Marshall Islands	2031	877.8 (777.5 to 993.5)	707.6 (614.1 to 818.5)	94.9 (71.7 to 124.1)	27.9 (14.2 to 50.5)	47.4 (38.4 to 59.4)	860.2 (761.9 to 973.5)	693.4 (601.7 to 802.1)	93.0 (70.2 to 121.6)	27.4 (13.9 to 49.5)	46.4 (37.6 to 58.2)
Marshall Islands	2032	906.8 (802.9 to 1032.7)	732.9 (633.8 to 848.1)	96.9 (72.8 to 127.7)	28.8 (14.7 to 51.9)	48.1 (38.7 to 60.6)	888.5 (786.7 to 1011.9)	718.2 (621.1 to 831.1)	95.0 (71.4 to 125.2)	28.2 (14.4 to 50.8)	47.2 (37.9 to 59.4)
Marshall Islands	2033	933.7 (824.7 to 1061.8)	756.2 (653.4 to 878.1)	98.9 (74.2 to 131.4)	29.6 (15.1 to 53.4)	49.0 (38.9 to 62.8)	914.9 (808.1 to 1040.4)	741.0 (640.3 to 860.5)	97.0 (72.7 to 128.7)	29.0 (14.8 to 52.3)	48.0 (38.1 to 61.6)
Marshall Islands	2034	960.5 (848.8 to 1095.8)	779.3 (670.9 to 906.8)	100.9 (75.6 to 134.7)	30.4 (15.5 to 55.0)	49.9 (39.3 to 64.4)	941.2 (831.8 to 1073.7)	763.6 (657.5 to 888.5)	98.9 (74.0 to 132.0)	29.8 (15.2 to 53.9)	48.9 (38.5 to 63.1)
Marshall Islands	2035	987.0 (870.6 to 1122.6)	801.9 (690.2 to 934.2)	103.0 (76.7 to 137.8)	31.3 (15.9 to 56.7)	50.8 (39.5 to 66.3)	967.1 (853.1 to 1100.0)	785.8 (676.3 to 915.4)	100.9 (75.2 to 135.1)	30.6 (15.6 to 55.5)	49.8 (38.7 to 65.0)
Marshall Islands	2036	1013.5 (889.8 to 1152.7)	824.6 (704.8 to 962.4)	105.0 (78.0 to 141.2)	32.1 (16.3 to 58.1)	51.8 (40.1 to 69.6)	993.1 (871.9 to 1129.6)	808.0 (690.6 to 943.1)	102.9 (76.4 to 138.4)	31.5 (16.0 to 57.0)	50.7 (39.3 to 68.2)

Country	Year	2018 US Dollars per capita					2018 PPP per capita				
		Health spending	Government health spending	Out-of-pocket health spending	Prepaid private health spending	Development assistance for health	Health spending	Government health spending	Out-of-pocket health spending	Prepaid private health spending	Development assistance for health
Marshall Islands	2037	1041.6 (911.7 to 1190.8)	848.8 (724.5 to 993.3)	107.1 (79.2 to 143.5)	33.0 (16.8 to 60.0)	52.7 (40.5 to 70.8)	1020.6 (893.4 to 1166.9)	831.8 (710.0 to 973.3)	104.9 (77.6 to 140.6)	32.3 (16.4 to 58.8)	51.6 (39.7 to 69.3)
Marshall Islands	2038	1069.1 (926.3 to 1228.9)	872.4 (743.7 to 1023.2)	109.1 (80.6 to 147.0)	33.9 (17.2 to 61.8)	53.7 (40.9 to 74.7)	1047.6 (907.7 to 1204.2)	854.9 (728.8 to 1002.7)	106.9 (78.9 to 144.0)	33.2 (16.8 to 60.6)	52.6 (40.1 to 73.2)
Marshall Islands	2039	1093.6 (952.6 to 1255.9)	893.0 (759.7 to 1049.6)	111.2 (82.0 to 150.1)	34.7 (17.6 to 63.6)	54.7 (41.8 to 75.9)	1071.6 (933.5 to 1230.6)	875.1 (744.5 to 1028.5)	109.0 (80.4 to 147.1)	34.0 (17.3 to 62.3)	53.6 (41.0 to 74.4)
Marshall Islands	2040	1118.5 (973.7 to 1282.2)	913.8 (775.2 to 1076.0)	113.3 (83.4 to 152.6)	35.6 (18.0 to 65.2)	55.8 (42.3 to 77.4)	1096.0 (954.1 to 1256.4)	895.4 (759.6 to 1054.4)	111.0 (81.7 to 149.5)	34.9 (17.7 to 63.9)	54.7 (41.5 to 75.9)
Marshall Islands	2041	1144.1 (990.9 to 1314.3)	935.3 (789.0 to 1101.0)	115.3 (84.9 to 155.8)	36.5 (18.4 to 66.7)	57.0 (42.8 to 79.1)	1121.1 (971.0 to 1287.9)	916.5 (773.1 to 1078.9)	113.0 (83.2 to 152.7)	35.7 (18.0 to 65.3)	55.9 (42.0 to 77.5)
Marshall Islands	2042	1171.8 (1004.4 to 1351.5)	958.8 (805.8 to 1129.0)	117.3 (86.3 to 158.9)	37.3 (18.8 to 68.2)	58.3 (43.6 to 83.1)	1148.2 (984.2 to 1324.3)	939.6 (789.6 to 1106.3)	114.9 (84.6 to 155.7)	36.6 (18.4 to 66.8)	57.1 (42.7 to 81.4)
Marshall Islands	2043	1199.3 (1034.4 to 1399.1)	982.4 (821.4 to 1159.0)	119.2 (87.7 to 161.7)	38.1 (19.1 to 69.5)	59.6 (44.1 to 86.0)	1175.2 (1013.6 to 1371.0)	962.7 (804.9 to 1135.7)	116.8 (85.9 to 158.4)	37.3 (18.7 to 68.1)	58.4 (43.2 to 84.3)
Marshall Islands	2044	1220.7 (1049.6 to 1415.5)	999.8 (836.3 to 1182.4)	120.9 (88.9 to 164.3)	38.8 (19.4 to 70.6)	61.1 (45.0 to 88.4)	1196.2 (1028.5 to 1387.1)	979.8 (819.5 to 1158.7)	118.5 (87.2 to 161.0)	38.0 (19.0 to 69.2)	59.9 (44.1 to 86.6)
Marshall Islands	2045	1241.2 (1066.0 to 1432.2)	1016.5 (843.8 to 1203.5)	122.6 (90.0 to 167.0)	39.4 (19.7 to 71.7)	62.7 (45.2 to 94.7)	1216.2 (1044.6 to 1403.5)	996.1 (826.8 to 1179.3)	120.1 (88.2 to 163.6)	38.6 (19.3 to 70.2)	61.5 (44.3 to 92.8)
Marshall Islands	2046	1258.2 (1078.0 to 1458.3)	1029.8 (853.2 to 1226.0)	124.2 (90.9 to 169.4)	39.9 (19.9 to 73.0)	64.4 (46.5 to 99.2)	1232.9 (1056.4 to 1429.0)	1009.1 (836.0 to 1201.3)	121.7 (89.1 to 166.0)	39.1 (19.5 to 71.5)	63.1 (45.6 to 97.2)
Marshall Islands	2047	1265.4 (1076.5 to 1475.9)	1033.3 (849.9 to 1235.6)	125.5 (91.6 to 170.8)	40.3 (20.0 to 74.0)	66.2 (47.5 to 104.3)	1240.0 (1054.9 to 1446.2)	1012.6 (832.8 to 1210.8)	123.0 (89.8 to 167.4)	39.5 (19.6 to 72.5)	64.9 (46.5 to 102.2)
Marshall Islands	2048	1273.1 (1074.9 to 1487.6)	1037.3 (852.2 to 1242.5)	126.8 (92.4 to 172.5)	40.7 (20.1 to 75.0)	68.2 (48.6 to 109.4)	1247.5 (1053.3 to 1457.8)	1016.5 (835.1 to 1217.6)	124.3 (90.5 to 169.0)	39.9 (19.7 to 73.5)	66.9 (47.6 to 107.2)
Marshall Islands	2049	1278.4 (1084.3 to 1498.5)	1039.0 (845.6 to 1248.8)	128.1 (93.1 to 173.8)	41.0 (20.2 to 75.6)	70.2 (50.1 to 113.1)	1252.7 (1062.5 to 1468.4)	1018.1 (828.6 to 1223.7)	125.5 (91.2 to 170.3)	40.2 (19.8 to 74.0)	68.8 (49.1 to 110.9)
Marshall Islands	2050	1286.8 (1083.4 to 1507.3)	1043.7 (843.7 to 1262.0)	129.3 (94.0 to 175.0)	41.3 (20.3 to 76.0)	72.5 (50.9 to 118.2)	1261.0 (1061.6 to 1477.0)	1022.7 (826.8 to 1236.6)	126.7 (92.1 to 171.5)	40.5 (19.9 to 74.4)	71.1 (49.9 to 115.8)
Mauritania	1995	54.5 (45.1 to 66.3)	11.4 (8.7 to 14.8)	34.3 (25.1 to 45.9)	0.6 (0.3 to 1.1)	8.1 (8.1 to 8.1)	187.0 (154.8 to 227.6)	39.2 (29.9 to 50.8)	117.8 (86.2 to 157.6)	2.1 (0.9 to 3.8)	27.9 (27.9 to 27.9)
Mauritania	1996	56.7 (47.0 to 68.5)	12.1 (9.1 to 15.7)	35.4 (25.9 to 47.3)	0.6 (0.3 to 1.1)	8.7 (8.7 to 8.7)	194.7 (161.4 to 235.2)	41.4 (31.3 to 53.8)	121.5 (88.9 to 162.4)	2.1 (0.9 to 3.9)	29.8 (29.8 to 29.8)
Mauritania	1997	50.6 (41.0 to 61.7)	11.2 (8.5 to 14.6)	34.1 (25.0 to 45.5)	0.6 (0.2 to 1.0)	4.6 (4.6 to 4.6)	173.7 (140.8 to 211.7)	38.6 (29.2 to 50.1)	117.2 (85.8 to 156.1)	1.9 (0.9 to 3.5)	16.0 (16.0 to 16.0)
Mauritania	1998	50.0 (40.9 to 61.0)	10.5 (8.0 to 13.6)	34.3 (25.3 to 45.0)	0.5 (0.2 to 0.9)	4.8 (4.8 to 4.8)	171.8 (140.4 to 209.5)	35.9 (27.5 to 46.5)	117.9 (86.7 to 154.6)	1.6 (0.7 to 3.0)	16.4 (16.4 to 16.4)
Mauritania	1999	48.4 (39.1 to 59.9)	9.8 (7.4 to 12.6)	34.6 (25.6 to 45.6)	0.4 (0.2 to 0.7)	3.6 (3.6 to 3.6)	166.0 (134.3 to 205.5)	33.5 (25.4 to 43.3)	118.9 (88.0 to 156.6)	1.2 (0.6 to 2.3)	12.4 (12.4 to 12.4)
Mauritania	2000	47.8 (38.7 to 59.1)	8.4 (6.3 to 10.8)	33.3 (24.5 to 44.6)	0.2 (0.1 to 0.3)	5.9 (5.9 to 5.9)	164.0 (132.9 to 203.0)	28.9 (21.7 to 37.1)	114.2 (84.2 to 153.0)	0.6 (0.3 to 1.2)	20.2 (20.2 to 20.2)
Mauritania	2001	47.1 (38.0 to 58.5)	8.6 (6.5 to 11.1)	33.6 (24.9 to 44.8)	0.2 (0.1 to 0.4)	4.7 (4.7 to 4.7)	161.5 (130.6 to 200.9)	29.4 (22.2 to 38.1)	115.4 (85.6 to 153.7)	0.6 (0.3 to 1.2)	16.1 (16.1 to 16.1)

		2018 US Dollars per capita					2018 PPP per capita				
Country	Year	Health spending	Government health spending	Out-of-pocket health spending	Prepaid private health spending	Development assistance for health	Health spending	Government health spending	Out-of-pocket health spending	Prepaid private health spending	Development assistance for health
Mauritania	2002	50.0 (40.8 to 61.9)	9.6 (7.2 to 12.3)	34.7 (25.9 to 46.7)	0.4 (0.2 to 0.7)	5.3 (5.3 to 5.3)	171.5 (140.2 to 212.5)	32.9 (24.9 to 42.1)	119.2 (89.1 to 160.4)	1.2 (0.6 to 2.3)	18.2 (18.2 to 18.2)
Mauritania	2003	52.5 (43.3 to 65.0)	10.0 (7.6 to 12.8)	34.5 (25.7 to 46.4)	0.5 (0.2 to 0.9)	7.5 (7.5 to 7.5)	180.3 (148.8 to 223.2)	34.4 (26.3 to 43.9)	118.4 (88.4 to 159.2)	1.7 (0.8 to 3.1)	25.8 (25.8 to 25.8)
Mauritania	2004	50.0 (41.3 to 62.0)	10.1 (7.7 to 13.1)	33.0 (24.5 to 45.2)	0.5 (0.3 to 1.0)	6.3 (6.3 to 6.3)	171.6 (141.6 to 212.8)	34.7 (26.4 to 44.8)	113.4 (84.2 to 155.1)	1.9 (0.9 to 3.5)	21.6 (21.6 to 21.6)
Mauritania	2005	45.9 (37.5 to 57.8)	10.8 (8.2 to 13.9)	31.7 (23.5 to 43.8)	0.6 (0.3 to 1.1)	2.7 (2.7 to 2.7)	157.6 (128.8 to 198.3)	37.1 (28.3 to 47.7)	109.0 (80.8 to 150.5)	2.1 (1.0 to 3.9)	9.4 (9.4 to 9.4)
Mauritania	2006	48.4 (39.8 to 60.4)	12.4 (9.5 to 16.0)	31.8 (23.8 to 44.0)	0.7 (0.3 to 1.4)	3.5 (3.5 to 3.5)	166.0 (136.5 to 207.5)	42.4 (32.6 to 55.0)	109.0 (81.7 to 151.1)	2.5 (1.2 to 4.7)	12.2 (12.2 to 12.2)
Mauritania	2007	49.4 (40.9 to 61.5)	12.6 (9.8 to 16.3)	30.5 (22.5 to 42.0)	0.7 (0.4 to 1.4)	5.6 (5.6 to 5.6)	169.7 (140.3 to 211.2)	43.4 (33.5 to 56.1)	104.7 (77.3 to 144.3)	2.5 (1.2 to 4.8)	19.1 (19.1 to 19.1)
Mauritania	2008	48.7 (40.2 to 60.5)	12.8 (10.0 to 16.9)	29.2 (21.1 to 40.3)	0.8 (0.4 to 1.5)	5.8 (5.8 to 5.8)	167.2 (137.9 to 207.8)	44.1 (34.2 to 58.0)	100.4 (72.4 to 138.3)	2.7 (1.3 to 5.1)	20.0 (20.0 to 20.0)
Mauritania	2009	45.1 (36.7 to 56.0)	12.4 (9.6 to 16.1)	27.5 (20.3 to 38.0)	0.7 (0.4 to 1.4)	4.4 (4.4 to 4.4)	154.8 (125.9 to 192.3)	42.7 (33.0 to 55.4)	94.6 (69.7 to 130.6)	2.5 (1.2 to 4.7)	15.0 (15.0 to 15.0)
Mauritania	2010	43.2 (35.1 to 53.0)	12.6 (9.7 to 16.5)	25.9 (18.9 to 35.2)	0.8 (0.4 to 1.4)	3.9 (3.9 to 3.9)	148.4 (120.7 to 182.0)	43.2 (33.4 to 56.6)	88.9 (64.8 to 120.7)	2.7 (1.3 to 5.0)	13.5 (13.5 to 13.5)
Mauritania	2011	42.9 (35.1 to 52.4)	13.3 (10.3 to 17.4)	24.9 (18.0 to 33.7)	1.1 (0.5 to 2.0)	3.6 (3.6 to 3.6)	147.4 (120.6 to 179.9)	45.8 (35.4 to 59.9)	85.5 (61.9 to 115.6)	3.8 (1.8 to 6.9)	12.3 (12.3 to 12.3)
Mauritania	2012	43.1 (35.3 to 53.0)	14.6 (11.2 to 19.2)	24.6 (17.6 to 33.4)	1.3 (0.6 to 2.4)	2.5 (2.5 to 2.5)	147.9 (121.2 to 182.1)	50.2 (38.5 to 65.9)	84.5 (60.3 to 114.5)	4.6 (2.2 to 8.3)	8.7 (8.7 to 8.7)
Mauritania	2013	47.2 (39.2 to 57.6)	16.4 (12.4 to 21.6)	24.8 (17.8 to 33.8)	1.5 (0.7 to 2.8)	4.5 (4.5 to 4.5)	162.0 (134.6 to 197.6)	56.2 (42.5 to 74.1)	85.2 (61.1 to 116.0)	5.3 (2.5 to 9.6)	15.3 (15.3 to 15.3)
Mauritania	2014	52.4 (43.6 to 63.3)	19.0 (14.3 to 24.8)	26.8 (19.3 to 35.8)	2.1 (1.0 to 3.9)	4.6 (4.6 to 4.6)	180.0 (149.8 to 217.3)	65.1 (49.0 to 85.2)	91.8 (66.1 to 122.9)	7.2 (3.4 to 13.3)	15.9 (15.9 to 15.9)
Mauritania	2015	55.5 (45.9 to 67.1)	20.4 (15.4 to 26.6)	28.0 (20.1 to 37.8)	2.4 (1.1 to 4.4)	4.6 (4.6 to 4.6)	190.4 (157.6 to 230.4)	70.1 (52.9 to 91.2)	96.2 (69.0 to 129.6)	8.1 (3.8 to 15.1)	15.9 (15.9 to 15.9)
Mauritania	2016	55.5 (46.4 to 66.6)	20.4 (15.6 to 26.4)	28.1 (20.3 to 38.2)	2.4 (1.1 to 4.6)	4.6 (4.6 to 4.6)	190.6 (159.4 to 228.5)	70.2 (53.4 to 90.7)	96.3 (69.8 to 131.0)	8.3 (3.8 to 15.7)	15.8 (15.8 to 15.8)
Mauritania	2017	57.6 (48.1 to 68.9)	20.3 (15.5 to 26.3)	28.3 (20.5 to 38.4)	2.4 (1.1 to 4.6)	6.6 (6.6 to 6.6)	197.7 (165.2 to 236.4)	69.7 (53.1 to 90.1)	97.1 (70.2 to 131.9)	8.3 (3.8 to 15.8)	22.7 (22.7 to 22.7)
Mauritania	2018	57.8 (47.9 to 69.2)	20.6 (15.7 to 26.7)	28.6 (20.7 to 38.8)	2.5 (1.1 to 4.7)	6.1 (6.1 to 6.1)	198.4 (164.3 to 237.5)	70.8 (54.0 to 91.5)	98.1 (70.9 to 133.3)	8.5 (3.9 to 16.1)	21.0 (21.0 to 21.0)
Mauritania	2019	58.4 (48.6 to 70.5)	20.9 (16.0 to 27.0)	28.9 (20.8 to 39.1)	2.5 (1.2 to 4.8)	6.0 (5.6 to 6.4)	200.3 (166.9 to 241.9)	71.9 (54.8 to 92.8)	99.1 (71.5 to 134.4)	8.6 (4.0 to 16.4)	20.8 (19.4 to 21.9)
Mauritania	2020	58.9 (49.2 to 70.9)	21.2 (16.1 to 27.3)	29.1 (21.0 to 39.6)	2.6 (1.2 to 4.8)	6.0 (5.5 to 6.5)	202.2 (169.1 to 243.4)	72.7 (55.3 to 93.9)	100.0 (72.1 to 135.8)	8.8 (4.0 to 16.6)	20.7 (19.0 to 22.3)
Mauritania	2021	59.4 (49.5 to 71.5)	21.4 (16.3 to 27.7)	29.4 (21.2 to 39.9)	2.6 (1.2 to 4.9)	6.0 (5.4 to 6.7)	204.0 (169.9 to 245.6)	73.5 (55.9 to 95.0)	100.9 (72.8 to 137.1)	8.9 (4.1 to 16.9)	20.7 (18.6 to 22.9)
Mauritania	2022	59.9 (49.9 to 72.5)	21.6 (16.4 to 28.1)	29.6 (21.5 to 40.1)	2.6 (1.2 to 5.0)	6.1 (5.4 to 6.8)	205.8 (171.2 to 248.9)	74.3 (56.4 to 96.3)	101.6 (73.8 to 137.8)	9.0 (4.2 to 17.2)	20.9 (18.5 to 23.3)

Country	Year	2018 US Dollars per capita					2018 PPP per capita				
		Health spending	Government health spending	Out-of-pocket health spending	Prepaid private health spending	Development assistance for health	Health spending	Government health spending	Out-of-pocket health spending	Prepaid private health spending	Development assistance for health
Mauritania	2023	60.5 (50.5 to 73.2)	21.9 (16.6 to 28.4)	29.8 (21.5 to 40.2)	2.7 (1.2 to 5.1)	6.1 (5.3 to 7.0)	207.6 (173.4 to 251.4)	75.1 (57.0 to 97.5)	102.3 (73.8 to 137.8)	9.2 (4.2 to 17.5)	21.0 (18.3 to 23.9)
Mauritania	2024	61.0 (50.4 to 73.8)	22.1 (16.8 to 28.7)	30.0 (21.7 to 40.5)	2.7 (1.2 to 5.2)	6.2 (5.3 to 7.1)	209.4 (172.9 to 253.3)	75.9 (57.6 to 98.4)	103.0 (74.5 to 139.2)	9.3 (4.3 to 17.7)	21.2 (18.3 to 24.5)
Mauritania	2025	61.5 (50.8 to 74.8)	22.4 (16.9 to 29.0)	30.2 (21.8 to 40.7)	2.7 (1.3 to 5.2)	6.2 (5.3 to 7.3)	211.3 (174.5 to 256.7)	76.8 (58.1 to 99.4)	103.7 (74.9 to 139.6)	9.4 (4.3 to 17.9)	21.3 (18.2 to 25.0)
Mauritania	2026	62.1 (50.7 to 75.5)	22.6 (17.1 to 29.3)	30.4 (22.0 to 40.9)	2.8 (1.3 to 5.3)	6.3 (5.3 to 7.3)	213.1 (173.9 to 259.1)	77.7 (58.8 to 100.5)	104.4 (75.4 to 140.5)	9.6 (4.4 to 18.1)	21.5 (18.2 to 25.2)
Mauritania	2027	62.7 (51.7 to 76.0)	22.9 (17.4 to 29.7)	30.6 (22.0 to 41.3)	2.8 (1.3 to 5.3)	6.3 (5.3 to 7.5)	215.2 (177.5 to 260.9)	78.7 (59.6 to 101.9)	105.1 (75.4 to 141.8)	9.7 (4.5 to 18.3)	21.7 (18.1 to 25.8)
Mauritania	2028	63.3 (52.4 to 76.4)	23.2 (17.6 to 30.1)	30.9 (22.1 to 41.7)	2.9 (1.3 to 5.4)	6.4 (5.3 to 7.7)	217.5 (180.0 to 262.4)	79.8 (60.4 to 103.4)	105.9 (75.7 to 143.2)	9.9 (4.5 to 18.6)	21.9 (18.3 to 26.4)
Mauritania	2029	64.1 (53.1 to 77.1)	23.6 (17.9 to 30.6)	31.1 (22.1 to 42.2)	2.9 (1.3 to 5.5)	6.4 (5.3 to 7.9)	220.0 (182.2 to 264.5)	81.0 (61.4 to 105.0)	106.8 (76.0 to 144.9)	10.1 (4.6 to 18.9)	22.1 (18.2 to 27.0)
Mauritania	2030	64.9 (52.9 to 78.5)	24.0 (18.2 to 31.2)	31.4 (22.2 to 42.9)	3.0 (1.4 to 5.6)	6.5 (5.3 to 8.0)	222.7 (181.5 to 269.5)	82.4 (62.5 to 107.1)	107.8 (76.3 to 147.2)	10.2 (4.7 to 19.2)	22.3 (18.2 to 27.4)
Mauritania	2031	65.6 (53.6 to 79.3)	24.4 (18.5 to 31.8)	31.7 (22.4 to 43.4)	3.0 (1.4 to 5.7)	6.6 (5.3 to 8.2)	225.4 (184.1 to 272.4)	83.6 (63.5 to 109.1)	108.8 (77.0 to 149.0)	10.4 (4.8 to 19.5)	22.5 (18.3 to 28.3)
Mauritania	2032	66.5 (54.2 to 81.1)	24.8 (18.8 to 32.3)	32.0 (22.6 to 44.0)	3.1 (1.4 to 5.8)	6.6 (5.3 to 8.4)	228.2 (186.1 to 278.2)	85.0 (64.6 to 110.8)	109.8 (77.4 to 151.1)	10.6 (4.9 to 19.9)	22.8 (18.2 to 28.7)
Mauritania	2033	67.3 (56.0 to 82.3)	25.1 (19.1 to 32.8)	32.3 (22.7 to 44.7)	3.1 (1.4 to 5.9)	6.7 (5.3 to 8.6)	231.1 (192.1 to 282.6)	86.3 (65.6 to 112.5)	110.9 (78.0 to 153.3)	10.8 (5.0 to 20.2)	23.1 (18.3 to 29.6)
Mauritania	2034	68.2 (56.2 to 82.4)	25.6 (19.4 to 33.3)	32.7 (22.9 to 45.3)	3.2 (1.5 to 6.0)	6.8 (5.4 to 8.7)	234.2 (192.8 to 283.0)	87.8 (66.7 to 114.4)	112.1 (78.6 to 155.5)	11.0 (5.0 to 20.5)	23.4 (18.4 to 30.0)
Mauritania	2035	69.2 (55.6 to 83.7)	26.0 (19.7 to 34.0)	33.0 (23.1 to 45.8)	3.3 (1.5 to 6.1)	6.9 (5.4 to 9.0)	237.4 (191.0 to 287.2)	89.2 (67.6 to 116.6)	113.4 (79.2 to 157.1)	11.2 (5.1 to 20.9)	23.7 (18.4 to 31.0)
Mauritania	2036	70.1 (56.8 to 84.8)	26.4 (20.0 to 34.5)	33.4 (23.2 to 46.4)	3.3 (1.5 to 6.2)	7.0 (5.4 to 9.4)	240.7 (194.9 to 291.2)	90.6 (68.8 to 118.5)	114.7 (79.8 to 159.3)	11.4 (5.2 to 21.2)	24.0 (18.6 to 32.3)
Mauritania	2037	71.1 (57.4 to 86.5)	26.8 (20.4 to 35.0)	33.8 (23.4 to 47.0)	3.4 (1.5 to 6.3)	7.1 (5.5 to 9.5)	244.0 (197.1 to 297.1)	92.1 (69.9 to 120.3)	116.0 (80.4 to 161.3)	11.6 (5.3 to 21.6)	24.3 (18.7 to 32.6)
Mauritania	2038	72.1 (59.2 to 87.8)	27.2 (20.6 to 35.5)	34.2 (23.6 to 47.5)	3.4 (1.6 to 6.4)	7.2 (5.5 to 9.9)	247.4 (203.1 to 301.4)	93.5 (70.9 to 122.0)	117.4 (81.1 to 162.9)	11.7 (5.4 to 22.0)	24.7 (18.8 to 34.2)
Mauritania	2039	73.0 (59.5 to 89.9)	27.7 (20.9 to 36.1)	34.6 (23.9 to 48.2)	3.5 (1.6 to 6.5)	7.3 (5.6 to 10.1)	250.8 (204.4 to 308.5)	95.0 (71.8 to 124.0)	118.9 (81.9 to 165.3)	11.9 (5.5 to 22.3)	25.0 (19.1 to 34.6)
Mauritania	2040	74.1 (60.4 to 89.9)	28.1 (21.2 to 36.8)	35.1 (24.1 to 48.9)	3.5 (1.6 to 6.6)	7.4 (5.6 to 10.2)	254.5 (207.5 to 308.5)	96.5 (72.9 to 126.2)	120.4 (82.6 to 167.8)	12.2 (5.6 to 22.7)	25.4 (19.3 to 35.0)
Mauritania	2041	75.2 (60.9 to 92.0)	28.6 (21.5 to 37.4)	35.5 (24.3 to 49.6)	3.6 (1.6 to 6.7)	7.5 (5.7 to 10.4)	258.2 (208.9 to 315.7)	98.0 (74.0 to 128.5)	122.0 (83.3 to 170.1)	12.4 (5.7 to 23.0)	25.8 (19.5 to 35.9)
Mauritania	2042	76.3 (61.8 to 93.6)	29.0 (21.9 to 38.2)	36.0 (24.5 to 50.3)	3.7 (1.7 to 6.8)	7.6 (5.7 to 10.9)	262.0 (212.1 to 321.2)	99.5 (75.1 to 131.0)	123.6 (84.1 to 172.5)	12.6 (5.7 to 23.4)	26.2 (19.6 to 37.5)
Mauritania	2043	77.4 (61.8 to 94.9)	29.4 (22.2 to 38.8)	36.5 (24.8 to 51.0)	3.7 (1.7 to 6.9)	7.8 (5.8 to 11.3)	265.8 (212.2 to 325.9)	101.1 (76.1 to 133.3)	125.3 (85.1 to 175.1)	12.8 (5.8 to 23.8)	26.7 (19.8 to 38.7)

Country	Year	2018 US Dollars per capita					2018 PPP per capita				
		Health spending	Government health spending	Out-of-pocket health spending	Prepaid private health spending	Development assistance for health	Health spending	Government health spending	Out-of-pocket health spending	Prepaid private health spending	Development assistance for health
Mauritania	2044	78.6 (63.2 to 95.4)	29.9 (22.5 to 39.5)	37.0 (25.1 to 51.9)	3.8 (1.7 to 7.0)	7.9 (5.9 to 11.5)	269.9 (217.1 to 327.5)	102.6 (77.2 to 135.6)	127.1 (86.3 to 178.1)	13.0 (5.9 to 24.1)	27.1 (20.1 to 39.3)
Mauritania	2045	79.8 (63.7 to 98.5)	30.4 (22.8 to 40.2)	37.6 (25.5 to 52.8)	3.9 (1.8 to 7.1)	8.0 (5.8 to 12.1)	274.1 (218.6 to 338.3)	104.3 (78.2 to 138.1)	129.0 (87.5 to 181.2)	13.2 (6.1 to 24.5)	27.6 (20.0 to 41.6)
Mauritania	2046	81.1 (65.6 to 99.2)	30.8 (23.1 to 41.0)	38.1 (25.8 to 53.7)	3.9 (1.8 to 7.3)	8.2 (5.9 to 12.5)	278.3 (225.2 to 340.7)	105.9 (79.3 to 140.7)	130.8 (88.7 to 184.4)	13.4 (6.2 to 25.0)	28.1 (20.3 to 43.0)
Mauritania	2047	82.3 (66.9 to 100.8)	31.3 (23.4 to 41.8)	38.7 (26.2 to 54.6)	4.0 (1.8 to 7.4)	8.3 (6.0 to 13.2)	282.7 (229.7 to 346.2)	107.5 (80.4 to 143.4)	132.8 (89.9 to 187.4)	13.7 (6.3 to 25.5)	28.6 (20.6 to 45.2)
Mauritania	2048	83.6 (67.9 to 102.6)	31.8 (23.8 to 42.6)	39.3 (26.5 to 55.5)	4.0 (1.9 to 7.6)	8.5 (6.1 to 13.6)	287.1 (233.1 to 352.4)	109.2 (81.6 to 146.1)	134.8 (91.1 to 190.5)	13.9 (6.4 to 25.9)	29.2 (21.0 to 46.6)
Mauritania	2049	85.0 (68.3 to 104.2)	32.3 (24.2 to 43.2)	39.9 (26.9 to 56.2)	4.1 (1.9 to 7.7)	8.7 (6.2 to 13.9)	291.7 (234.4 to 357.8)	111.0 (83.0 to 148.3)	136.8 (92.3 to 193.0)	14.1 (6.6 to 26.4)	29.8 (21.2 to 47.8)
Mauritania	2050	86.3 (69.2 to 105.6)	32.9 (24.6 to 43.8)	40.5 (27.3 to 57.1)	4.2 (1.9 to 7.8)	8.9 (6.2 to 14.5)	296.4 (237.7 to 362.7)	112.8 (84.4 to 150.4)	138.9 (93.8 to 196.2)	14.4 (6.7 to 26.8)	30.4 (21.4 to 49.9)
Mauritius	1995	122.5 (106.5 to 140.3)	66.3 (54.9 to 78.5)	50.2 (40.3 to 61.8)	6.0 (2.9 to 10.8)	0.1 (0.1 to 0.1)	272.2 (236.7 to 311.6)	147.2 (121.9 to 174.4)	111.5 (89.4 to 137.3)	13.4 (6.5 to 24.1)	0.1 (0.1 to 0.1)
Mauritius	1996	131.4 (115.8 to 148.8)	70.4 (59.4 to 82.4)	52.6 (43.0 to 64.1)	6.1 (3.0 to 11.1)	2.3 (2.3 to 2.3)	291.8 (257.2 to 330.5)	156.4 (131.9 to 183.0)	116.9 (95.5 to 142.3)	13.6 (6.6 to 24.7)	5.0 (5.0 to 5.0)
Mauritius	1997	134.6 (119.5 to 151.4)	73.3 (62.8 to 84.8)	54.9 (45.5 to 65.2)	5.9 (3.0 to 10.5)	0.5 (0.5 to 0.5)	299.0 (265.5 to 336.3)	162.9 (139.6 to 188.4)	121.9 (101.0 to 144.8)	13.1 (6.6 to 23.3)	1.0 (1.0 to 1.0)
Mauritius	1998	143.1 (128.2 to 159.4)	78.6 (67.9 to 90.0)	58.5 (49.1 to 68.6)	5.7 (2.9 to 10.3)	0.3 (0.3 to 0.3)	318.0 (284.7 to 354.1)	174.7 (150.8 to 199.9)	130.0 (109.1 to 152.4)	12.6 (6.5 to 22.8)	0.7 (0.7 to 0.7)
Mauritius	1999	148.3 (133.6 to 164.3)	81.1 (70.8 to 92.7)	61.9 (52.3 to 72.3)	5.1 (2.6 to 9.2)	0.2 (0.2 to 0.2)	329.3 (296.9 to 364.9)	180.1 (157.2 to 205.9)	137.5 (116.2 to 160.7)	11.3 (5.8 to 20.4)	0.4 (0.4 to 0.4)
Mauritius	2000	165.1 (149.5 to 181.6)	89.8 (78.0 to 102.6)	70.6 (60.5 to 82.5)	4.5 (2.3 to 8.2)	0.2 (0.2 to 0.2)	366.8 (332.1 to 403.4)	199.5 (173.3 to 228.0)	156.8 (134.3 to 183.2)	10.0 (5.2 to 18.1)	0.5 (0.5 to 0.5)
Mauritius	2001	175.8 (159.2 to 194.0)	94.6 (82.7 to 107.7)	76.9 (66.4 to 89.6)	4.1 (2.2 to 7.5)	0.2 (0.2 to 0.2)	390.5 (353.6 to 431.0)	210.0 (183.7 to 239.3)	170.9 (147.6 to 198.9)	9.1 (4.8 to 16.6)	0.4 (0.4 to 0.4)
Mauritius	2002	189.7 (173.0 to 207.1)	100.4 (87.8 to 114.5)	85.0 (74.2 to 98.1)	4.2 (2.2 to 7.5)	0.0 (0.0 to 0.0)	421.3 (384.2 to 460.0)	223.0 (194.9 to 254.2)	188.8 (164.9 to 217.8)	9.4 (4.9 to 16.7)	0.1 (0.1 to 0.1)
Mauritius	2003	204.1 (186.4 to 223.3)	106.0 (93.0 to 120.7)	93.4 (82.2 to 107.6)	4.6 (2.3 to 8.2)	0.2 (0.2 to 0.2)	453.4 (414.0 to 496.0)	235.4 (206.7 to 268.2)	207.4 (182.7 to 238.9)	10.1 (5.2 to 18.1)	0.5 (0.5 to 0.5)
Mauritius	2004	220.8 (200.9 to 240.8)	111.9 (98.6 to 125.9)	103.7 (90.5 to 118.6)	5.0 (2.6 to 8.9)	0.2 (0.2 to 0.2)	490.5 (446.2 to 534.9)	248.6 (218.9 to 279.7)	230.3 (201.1 to 263.4)	11.1 (5.9 to 19.8)	0.5 (0.5 to 0.5)
Mauritius	2005	229.3 (209.3 to 248.7)	108.7 (95.8 to 121.6)	115.2 (101.2 to 130.3)	5.1 (2.7 to 8.9)	0.2 (0.2 to 0.2)	509.4 (464.9 to 552.4)	241.5 (212.8 to 270.1)	256.0 (224.9 to 289.5)	11.4 (6.1 to 19.8)	0.5 (0.5 to 0.5)
Mauritius	2006	245.2 (224.0 to 266.4)	109.6 (96.9 to 122.5)	129.7 (114.2 to 146.0)	5.4 (2.8 to 9.1)	0.6 (0.6 to 0.6)	544.7 (497.6 to 591.8)	243.4 (215.2 to 272.0)	288.0 (253.6 to 324.4)	11.9 (6.2 to 20.2)	1.3 (1.3 to 1.3)
Mauritius	2007	263.9 (242.1 to 285.9)	111.4 (98.6 to 125.1)	146.0 (128.4 to 162.6)	5.7 (2.9 to 9.7)	0.8 (0.8 to 0.8)	586.2 (537.7 to 635.1)	247.5 (219.1 to 277.8)	324.3 (285.3 to 361.2)	12.6 (6.5 to 21.5)	1.9 (1.9 to 1.9)
Mauritius	2008	287.6 (265.2 to 311.7)	115.6 (102.6 to 129.7)	166.0 (147.8 to 184.7)	5.9 (3.2 to 10.3)	0.1 (0.1 to 0.1)	638.8 (589.2 to 692.3)	256.7 (227.8 to 288.2)	368.7 (328.3 to 410.3)	13.2 (7.1 to 22.9)	0.2 (0.2 to 0.2)

Country	Year	2018 US Dollars per capita					2018 PPP per capita				
		Health spending	Government health spending	Out-of-pocket health spending	Prepaid private health spending	Development assistance for health	Health spending	Government health spending	Out-of-pocket health spending	Prepaid private health spending	Development assistance for health
Mauritius	2009	313.7 (289.9 to 338.8)	126.5 (112.4 to 141.9)	179.9 (161.3 to 199.4)	6.5 (3.5 to 11.3)	0.8 (0.8 to 0.8)	696.8 (643.9 to 752.5)	281.1 (249.6 to 315.1)	399.5 (358.4 to 443.0)	14.4 (7.8 to 25.2)	1.8 (1.8 to 1.8)
Mauritius	2010	340.8 (314.1 to 368.4)	142.5 (127.0 to 159.6)	189.2 (170.0 to 210.9)	7.1 (3.9 to 12.1)	2.1 (2.1 to 2.1)	757.0 (697.6 to 818.3)	316.4 (282.1 to 354.5)	420.2 (377.7 to 468.6)	15.8 (8.6 to 26.8)	4.6 (4.6 to 4.6)
Mauritius	2011	355.8 (329.7 to 385.1)	152.2 (136.8 to 170.3)	194.0 (175.7 to 216.1)	7.9 (4.3 to 13.2)	1.6 (1.6 to 1.6)	790.2 (732.4 to 855.3)	338.2 (304.0 to 378.2)	430.8 (390.2 to 480.1)	17.7 (9.6 to 29.2)	3.6 (3.6 to 3.6)
Mauritius	2012	378.9 (350.8 to 408.1)	165.4 (149.1 to 184.2)	201.9 (182.3 to 224.4)	9.6 (5.4 to 15.9)	2.0 (2.0 to 2.0)	841.6 (779.3 to 906.6)	367.3 (331.2 to 409.1)	448.5 (404.9 to 498.4)	21.4 (12.1 to 35.3)	4.5 (4.5 to 4.5)
Mauritius	2013	419.4 (388.8 to 450.8)	182.6 (164.4 to 203.5)	220.8 (199.1 to 245.4)	14.2 (8.0 to 23.2)	1.8 (1.8 to 1.8)	931.5 (863.6 to 1001.3)	405.6 (365.2 to 451.9)	490.5 (442.3 to 545.2)	31.6 (17.7 to 51.5)	3.9 (3.9 to 3.9)
Mauritius	2014	476.1 (441.0 to 511.4)	208.8 (186.9 to 231.1)	244.8 (219.8 to 272.7)	20.9 (11.7 to 35.1)	1.7 (1.7 to 1.7)	1057.6 (979.5 to 1136.0)	463.7 (415.1 to 513.4)	543.7 (488.3 to 605.7)	46.4 (26.1 to 78.1)	3.7 (3.7 to 3.7)
Mauritius	2015	517.8 (476.9 to 560.1)	226.5 (201.5 to 252.8)	262.0 (235.1 to 295.5)	28.8 (16.2 to 47.9)	0.5 (0.5 to 0.5)	1150.3 (1059.4 to 1244.0)	503.2 (447.6 to 561.4)	582.1 (522.3 to 656.4)	63.9 (36.0 to 106.3)	1.1 (1.1 to 1.1)
Mauritius	2016	556.9 (509.7 to 609.5)	245.3 (213.9 to 277.3)	274.4 (241.5 to 314.9)	36.2 (20.2 to 60.0)	0.9 (0.9 to 0.9)	1237.1 (1132.1 to 1353.9)	545.0 (475.2 to 615.8)	609.6 (536.4 to 699.4)	80.5 (45.0 to 133.3)	2.0 (2.0 to 2.0)
Mauritius	2017	599.2 (548.7 to 656.0)	271.1 (236.6 to 306.4)	288.0 (252.7 to 330.9)	37.2 (20.8 to 61.8)	2.9 (2.9 to 2.9)	1330.9 (1218.9 to 1457.1)	602.2 (525.4 to 680.6)	639.7 (561.4 to 735.0)	82.6 (46.1 to 137.2)	6.4 (6.4 to 6.4)
Mauritius	2018	618.5 (562.6 to 679.9)	278.1 (242.9 to 314.8)	299.4 (262.8 to 344.1)	38.2 (21.4 to 63.7)	2.7 (2.7 to 2.7)	1373.9 (1249.6 to 1510.2)	617.8 (539.5 to 699.3)	665.1 (583.7 to 764.3)	84.9 (47.5 to 141.6)	6.1 (6.1 to 6.1)
Mauritius	2019	638.2 (581.7 to 702.3)	285.2 (249.3 to 323.2)	310.9 (273.0 to 358.8)	39.3 (22.0 to 65.4)	2.8 (2.6 to 2.9)	1417.5 (1292.1 to 1559.9)	633.5 (553.7 to 718.0)	690.6 (606.5 to 797.1)	87.2 (48.8 to 145.4)	6.2 (5.8 to 6.6)
Mauritius	2020	659.1 (601.6 to 726.9)	293.1 (256.4 to 332.9)	322.8 (282.0 to 371.9)	40.4 (22.6 to 67.5)	2.8 (2.6 to 3.0)	1464.1 (1336.2 to 1614.6)	651.1 (569.6 to 739.3)	717.1 (626.4 to 826.1)	89.6 (50.2 to 149.8)	6.2 (5.7 to 6.8)
Mauritius	2021	684.3 (622.4 to 754.3)	304.3 (265.4 to 344.7)	335.5 (292.0 to 386.8)	41.6 (23.3 to 69.3)	2.9 (2.6 to 3.2)	1519.9 (1382.4 to 1675.5)	675.9 (589.6 to 765.6)	745.2 (648.7 to 859.3)	92.4 (51.8 to 154.0)	6.3 (5.7 to 7.1)
Mauritius	2022	710.6 (645.8 to 780.2)	316.0 (275.4 to 357.6)	348.8 (303.8 to 401.2)	42.9 (24.1 to 71.5)	2.9 (2.5 to 3.3)	1578.5 (1434.5 to 1732.9)	702.0 (611.8 to 794.3)	774.7 (674.8 to 891.1)	95.4 (53.5 to 158.7)	6.4 (5.6 to 7.3)
Mauritius	2023	738.4 (668.3 to 810.5)	328.5 (285.9 to 370.3)	362.6 (315.6 to 419.4)	44.4 (24.9 to 73.8)	2.9 (2.5 to 3.4)	1640.1 (1484.5 to 1800.3)	729.6 (635.1 to 822.6)	805.5 (701.1 to 931.6)	98.5 (55.4 to 163.9)	6.5 (5.6 to 7.5)
Mauritius	2024	767.0 (693.6 to 843.1)	341.3 (295.1 to 386.7)	376.9 (326.6 to 439.2)	45.8 (25.8 to 76.1)	3.0 (2.6 to 3.5)	1703.8 (1540.6 to 1872.8)	758.2 (655.5 to 859.0)	837.2 (725.4 to 975.5)	101.7 (57.3 to 169.1)	6.7 (5.7 to 7.8)
Mauritius	2025	796.0 (718.4 to 876.7)	354.3 (306.2 to 401.2)	391.5 (339.2 to 455.7)	47.2 (26.6 to 78.4)	3.1 (2.6 to 3.6)	1768.2 (1595.8 to 1947.3)	787.0 (680.1 to 891.2)	869.6 (753.5 to 1012.2)	104.8 (59.0 to 174.0)	6.9 (5.8 to 8.1)
Mauritius	2026	825.6 (748.1 to 904.5)	367.5 (317.3 to 419.0)	406.4 (350.6 to 473.2)	48.6 (27.3 to 80.7)	3.1 (2.6 to 3.8)	1833.9 (1661.6 to 2009.2)	816.2 (704.8 to 930.8)	902.7 (778.7 to 1051.2)	108.0 (60.7 to 179.2)	7.0 (5.8 to 8.4)
Mauritius	2027	855.5 (771.0 to 945.6)	380.7 (329.3 to 434.9)	421.6 (362.4 to 494.3)	50.0 (28.1 to 83.0)	3.2 (2.6 to 3.9)	1900.2 (1712.6 to 2100.4)	845.6 (731.4 to 965.9)	936.5 (805.1 to 1098.0)	111.0 (62.4 to 184.4)	7.1 (5.8 to 8.7)
Mauritius	2028	885.2 (797.0 to 982.0)	393.8 (340.2 to 452.4)	436.8 (374.3 to 515.6)	51.4 (28.8 to 85.3)	3.2 (0.0 to 4.1)	1966.2 (1770.4 to 2181.2)	874.8 (755.6 to 1004.8)	970.3 (831.4 to 1145.3)	114.1 (64.0 to 189.4)	7.1 (0.0 to 9.1)
Mauritius	2029	915.1 (819.3 to 1015.0)	407.1 (348.3 to 468.2)	452.1 (385.4 to 539.1)	52.8 (29.7 to 87.6)	3.2 (0.0 to 4.3)	2032.7 (1819.8 to 2254.6)	904.2 (773.6 to 1039.9)	1004.2 (856.1 to 1197.4)	117.2 (65.9 to 194.5)	7.1 (0.0 to 9.5)

Country	Year	2018 US Dollars per capita					2018 PPP per capita				
		Health spending	Government health spending	Out-of-pocket health spending	Prepaid private health spending	Development assistance for health	Health spending	Government health spending	Out-of-pocket health spending	Prepaid private health spending	Development assistance for health
Mauritius	2030	944.6 (842.1 to 1059.1)	420.1 (360.0 to 485.3)	467.3 (396.2 to 561.4)	54.2 (30.5 to 89.9)	3.1 (0.0 to 4.3)	2098.2 (1870.6 to 2352.6)	933.2 (799.7 to 1078.0)	1037.9 (880.0 to 1247.1)	120.3 (67.8 to 199.7)	6.8 (0.0 to 9.6)
Mauritius	2031	974.1 (863.8 to 1094.9)	433.0 (369.2 to 499.2)	482.4 (406.2 to 583.4)	55.6 (31.5 to 92.3)	3.0 (0.0 to 4.5)	2163.6 (1918.6 to 2432.0)	961.9 (820.0 to 1108.8)	1071.6 (902.2 to 1295.8)	123.6 (69.9 to 205.0)	6.6 (0.0 to 9.9)
Mauritius	2032	1003.4 (884.6 to 1141.4)	446.0 (379.7 to 516.4)	497.5 (417.7 to 607.2)	57.1 (32.4 to 94.7)	2.8 (0.0 to 4.6)	2228.8 (1964.8 to 2535.4)	990.6 (843.3 to 1147.0)	1105.2 (927.7 to 1348.7)	126.9 (71.9 to 210.4)	6.2 (0.0 to 10.2)
Mauritius	2033	1032.9 (906.0 to 1171.5)	459.0 (388.6 to 531.2)	512.6 (426.7 to 633.7)	58.6 (33.3 to 97.2)	2.6 (0.0 to 4.8)	2294.4 (2012.4 to 2602.2)	1019.6 (863.1 to 1179.9)	1138.7 (947.8 to 1407.7)	130.3 (74.1 to 216.0)	5.8 (0.0 to 10.6)
Mauritius	2034	1062.4 (927.2 to 1212.9)	472.1 (399.8 to 547.2)	527.6 (436.4 to 654.8)	60.2 (34.3 to 99.8)	2.4 (0.0 to 5.0)	2359.8 (2059.5 to 2694.0)	1048.7 (888.0 to 1215.5)	1172.0 (969.3 to 1454.4)	133.8 (76.3 to 221.7)	5.3 (0.0 to 11.0)
Mauritius	2035	1092.4 (958.8 to 1248.0)	485.6 (408.9 to 563.8)	542.7 (445.6 to 674.9)	61.9 (35.2 to 102.5)	2.2 (0.0 to 5.1)	2426.4 (2129.7 to 2772.1)	1078.7 (908.3 to 1252.3)	1205.5 (989.9 to 1499.2)	137.4 (78.3 to 227.7)	4.9 (0.0 to 11.3)
Mauritius	2036	1122.2 (986.5 to 1287.8)	499.0 (420.3 to 582.7)	557.6 (455.8 to 697.7)	63.5 (36.3 to 105.3)	2.0 (0.0 to 5.3)	2492.6 (2191.2 to 2860.5)	1108.4 (933.6 to 1294.4)	1238.6 (1012.4 to 1549.7)	141.1 (80.6 to 233.9)	4.5 (0.0 to 11.8)
Mauritius	2037	1152.0 (1010.1 to 1318.0)	512.4 (430.4 to 599.9)	572.4 (465.1 to 718.5)	65.3 (37.4 to 108.2)	1.8 (0.0 to 5.3)	2558.8 (2243.6 to 2927.6)	1138.3 (956.1 to 1332.5)	1271.5 (1033.0 to 1595.9)	145.0 (83.0 to 240.3)	4.1 (0.0 to 11.9)
Mauritius	2038	1182.1 (1029.5 to 1357.5)	526.2 (442.2 to 616.6)	587.1 (474.5 to 740.8)	67.1 (38.4 to 111.1)	1.7 (0.0 to 5.5)	2625.7 (2286.8 to 3015.3)	1168.9 (982.2 to 1369.6)	1304.2 (1054.0 to 1645.4)	149.0 (85.4 to 246.8)	3.7 (0.0 to 12.2)
Mauritius	2039	1213.0 (1054.8 to 1397.9)	540.8 (453.1 to 634.9)	601.8 (484.4 to 764.3)	68.9 (39.4 to 114.1)	1.5 (0.0 to 5.7)	2694.3 (2342.9 to 3105.1)	1201.3 (1006.5 to 1410.4)	1336.6 (1075.9 to 1697.6)	153.0 (87.6 to 253.5)	3.3 (0.0 to 12.7)
Mauritius	2040	1243.8 (1076.8 to 1431.3)	555.7 (466.2 to 653.5)	616.0 (493.6 to 787.0)	70.7 (40.4 to 117.1)	1.4 (0.0 to 5.8)	2762.8 (2391.9 to 3179.3)	1234.3 (1035.6 to 1451.6)	1368.4 (1096.3 to 1748.1)	157.1 (89.7 to 260.2)	3.0 (0.0 to 13.0)
Mauritius	2041	1275.3 (1102.6 to 1483.2)	571.3 (476.4 to 674.7)	630.2 (502.5 to 809.1)	72.5 (41.3 to 120.2)	1.3 (0.0 to 6.1)	2832.9 (2449.1 to 3294.6)	1269.0 (1058.3 to 1498.6)	1399.8 (1116.3 to 1797.2)	161.1 (91.7 to 267.1)	2.9 (0.0 to 13.5)
Mauritius	2042	1307.2 (1129.6 to 1523.0)	587.5 (490.5 to 696.1)	644.0 (511.8 to 831.4)	74.4 (42.2 to 123.4)	1.3 (0.0 to 6.5)	2903.6 (2509.2 to 3382.9)	1305.0 (1089.5 to 1546.2)	1430.5 (1136.9 to 1846.8)	165.2 (93.7 to 274.1)	2.9 (0.0 to 14.4)
Mauritius	2043	1339.0 (1153.7 to 1557.4)	604.0 (503.6 to 718.7)	657.5 (519.6 to 849.3)	76.2 (43.1 to 126.6)	1.3 (0.0 to 6.7)	2974.3 (2562.6 to 3459.4)	1341.7 (1118.5 to 1596.5)	1460.4 (1154.2 to 1886.6)	169.4 (95.7 to 281.1)	2.8 (0.0 to 14.8)
Mauritius	2044	1371.0 (1175.9 to 1587.6)	620.9 (515.2 to 739.3)	670.7 (528.8 to 868.9)	78.2 (44.1 to 129.9)	1.2 (0.0 to 7.0)	3045.3 (2612.0 to 3526.4)	1379.1 (1144.4 to 1642.1)	1489.9 (1174.5 to 1930.0)	173.6 (97.9 to 288.5)	2.6 (0.0 to 15.4)
Mauritius	2045	1403.1 (1203.4 to 1622.4)	637.9 (529.2 to 763.2)	683.8 (537.6 to 888.0)	80.2 (45.1 to 133.4)	1.1 (0.0 to 7.0)	3116.6 (2673.0 to 3603.6)	1417.0 (1175.4 to 1695.3)	1518.9 (1194.1 to 1972.4)	178.2 (100.1 to 296.3)	2.5 (0.0 to 15.6)
Mauritius	2046	1434.6 (1228.7 to 1674.9)	654.9 (540.8 to 783.8)	696.4 (546.5 to 904.2)	82.3 (46.1 to 137.0)	1.1 (0.0 to 7.1)	3186.7 (2729.2 to 3720.4)	1454.7 (1201.2 to 1741.0)	1546.8 (1214.0 to 2008.4)	182.8 (102.3 to 304.3)	2.4 (0.0 to 15.8)
Mauritius	2047	1466.5 (1248.3 to 1721.8)	672.2 (557.7 to 808.6)	708.7 (555.7 to 920.7)	84.5 (47.3 to 140.9)	1.0 (0.0 to 7.4)	3257.4 (2772.9 to 3824.4)	1493.2 (1238.7 to 1796.0)	1574.1 (1234.4 to 2045.2)	187.7 (105.0 to 312.9)	2.3 (0.0 to 16.4)
Mauritius	2048	1499.0 (1269.2 to 1753.5)	689.9 (568.8 to 831.7)	721.2 (563.2 to 939.4)	86.8 (48.6 to 144.9)	1.0 (0.0 to 7.5)	3329.6 (2819.2 to 3895.1)	1532.5 (1263.5 to 1847.5)	1602.1 (1251.1 to 2086.7)	192.8 (107.8 to 321.8)	2.2 (0.0 to 16.6)
Mauritius	2049	1531.8 (1305.7 to 1802.9)	707.7 (584.6 to 854.9)	733.9 (571.2 to 958.0)	89.2 (49.9 to 148.7)	1.0 (0.0 to 7.2)	3402.4 (2900.2 to 4004.7)	1572.0 (1298.6 to 1898.9)	1630.2 (1268.7 to 2127.9)	198.1 (110.8 to 330.4)	2.1 (0.0 to 16.1)
Mauritius	2050	1564.8 (1331.8 to 1830.1)	725.6 (595.4 to 880.3)	746.7 (581.6 to 976.2)	91.5 (51.2 to 152.6)	0.9 (0.0 to 7.5)	3475.7 (2958.3 to 4065.2)	1611.8 (1322.5 to 1955.4)	1658.5 (1291.8 to 2168.4)	203.3 (113.7 to 338.9)	2.0 (0.0 to 16.6)

Country	Year	2018 US Dollars per capita					2018 PPP per capita				
		Health spending	Government health spending	Out-of-pocket health spending	Prepaid private health spending	Development assistance for health	Health spending	Government health spending	Out-of-pocket health spending	Prepaid private health spending	Development assistance for health
Mexico	1995	292.0 (261.4 to 326.4)	123.5 (106.2 to 141.9)	162.8 (138.2 to 192.4)	4.1 (1.9 to 7.9)	1.6 (1.6 to 1.6)	637.1 (570.3 to 712.1)	269.5 (231.8 to 309.5)	355.3 (301.6 to 419.8)	8.9 (4.2 to 17.2)	3.4 (3.4 to 3.4)
Mexico	1996	296.8 (270.2 to 325.0)	128.2 (112.7 to 145.7)	162.6 (141.3 to 185.9)	4.6 (2.2 to 8.8)	1.4 (1.4 to 1.4)	647.5 (589.6 to 709.0)	279.6 (245.9 to 317.9)	354.7 (308.2 to 405.5)	10.1 (4.7 to 19.2)	3.1 (3.1 to 3.1)
Mexico	1997	315.4 (290.6 to 343.7)	140.7 (125.0 to 157.9)	167.4 (147.5 to 188.6)	5.6 (2.7 to 10.7)	1.7 (1.7 to 1.7)	688.1 (633.9 to 749.9)	307.0 (272.7 to 344.6)	365.1 (321.9 to 411.5)	12.3 (5.9 to 23.3)	3.7 (3.7 to 3.7)
Mexico	1998	337.1 (312.4 to 363.8)	151.4 (135.3 to 169.1)	173.2 (152.9 to 193.7)	6.5 (3.2 to 11.9)	5.9 (5.9 to 5.9)	735.3 (681.6 to 793.7)	330.3 (295.1 to 368.9)	377.8 (333.5 to 422.5)	14.2 (6.9 to 25.9)	13.0 (13.0 to 13.0)
Mexico	1999	352.2 (326.1 to 378.9)	161.7 (145.0 to 179.4)	181.7 (161.2 to 202.1)	7.7 (3.8 to 13.4)	1.2 (1.2 to 1.2)	768.5 (711.5 to 826.5)	352.9 (316.4 to 391.3)	396.3 (351.7 to 440.9)	16.7 (8.4 to 29.1)	2.6 (2.6 to 2.6)
Mexico	2000	381.5 (353.7 to 409.3)	170.8 (153.8 to 189.4)	197.1 (176.3 to 218.9)	9.5 (5.0 to 16.5)	4.0 (4.0 to 4.0)	832.2 (771.6 to 893.0)	372.6 (335.5 to 413.1)	430.0 (384.7 to 477.5)	20.8 (10.9 to 35.9)	8.8 (8.8 to 8.8)
Mexico	2001	395.5 (366.8 to 423.9)	172.8 (155.4 to 190.3)	210.4 (187.8 to 233.8)	11.0 (5.9 to 18.6)	1.3 (1.3 to 1.3)	862.9 (800.2 to 924.8)	377.1 (339.1 to 415.1)	458.9 (409.8 to 510.1)	24.1 (12.9 to 40.6)	2.8 (2.8 to 2.8)
Mexico	2002	412.8 (383.5 to 442.7)	175.3 (156.6 to 192.6)	223.1 (200.6 to 247.0)	12.3 (6.8 to 20.1)	2.1 (2.1 to 2.1)	900.6 (836.6 to 965.8)	382.4 (341.6 to 420.2)	486.8 (437.6 to 538.9)	26.9 (14.9 to 44.0)	4.6 (4.6 to 4.6)
Mexico	2003	434.0 (404.6 to 466.0)	181.5 (163.2 to 199.7)	238.3 (214.6 to 262.6)	13.8 (7.8 to 22.6)	0.4 (0.4 to 0.4)	946.8 (882.8 to 1016.6)	396.0 (356.0 to 435.7)	519.9 (468.2 to 572.9)	30.0 (17.0 to 49.2)	1.0 (1.0 to 1.0)
Mexico	2004	455.0 (423.8 to 487.5)	191.1 (173.0 to 211.7)	248.2 (223.6 to 273.1)	15.3 (8.8 to 25.3)	0.4 (0.4 to 0.4)	992.7 (924.6 to 1063.6)	417.0 (377.4 to 461.9)	541.6 (487.8 to 595.8)	33.4 (19.2 to 55.2)	0.8 (0.8 to 0.8)
Mexico	2005	461.6 (430.9 to 494.7)	194.6 (175.3 to 216.0)	249.5 (225.1 to 273.5)	16.7 (10.0 to 27.1)	0.9 (0.9 to 0.9)	1007.0 (940.1 to 1079.4)	424.5 (382.4 to 471.1)	544.2 (491.1 to 596.7)	36.3 (21.7 to 59.1)	1.9 (1.9 to 1.9)
Mexico	2006	469.5 (438.6 to 503.3)	202.8 (183.4 to 223.8)	246.9 (222.4 to 272.3)	18.5 (11.3 to 29.3)	1.3 (1.3 to 1.3)	1024.2 (956.8 to 1098.1)	442.4 (400.2 to 488.3)	538.6 (485.1 to 594.0)	40.4 (24.5 to 63.9)	2.8 (2.8 to 2.8)
Mexico	2007	473.2 (440.8 to 507.7)	212.2 (192.7 to 234.6)	238.8 (215.4 to 264.5)	21.3 (13.1 to 33.3)	1.0 (1.0 to 1.0)	1032.5 (961.6 to 1107.7)	462.9 (420.4 to 511.7)	520.9 (470.0 to 577.0)	46.5 (28.5 to 72.7)	2.2 (2.2 to 2.2)
Mexico	2008	469.2 (435.2 to 505.7)	221.1 (200.4 to 242.9)	221.5 (199.1 to 246.2)	25.5 (15.5 to 40.0)	1.0 (1.0 to 1.0)	1023.6 (949.5 to 1103.2)	482.4 (437.2 to 529.8)	483.3 (434.4 to 537.1)	55.6 (33.8 to 87.3)	2.2 (2.2 to 2.2)
Mexico	2009	453.7 (420.9 to 488.3)	220.2 (200.2 to 242.1)	205.7 (184.8 to 229.5)	27.3 (16.3 to 42.1)	0.5 (0.5 to 0.5)	989.8 (918.2 to 1065.3)	480.5 (436.7 to 528.1)	448.7 (403.3 to 500.8)	59.6 (35.6 to 91.8)	1.0 (1.0 to 1.0)
Mexico	2010	472.1 (438.7 to 507.1)	235.4 (215.0 to 258.3)	201.9 (180.8 to 226.7)	29.6 (17.9 to 45.5)	5.2 (5.2 to 5.2)	1030.1 (957.2 to 1106.2)	513.6 (469.1 to 563.4)	440.5 (394.5 to 494.7)	64.5 (39.1 to 99.3)	11.4 (11.4 to 11.4)
Mexico	2011	480.7 (445.2 to 518.1)	247.5 (224.6 to 272.6)	197.3 (177.0 to 222.0)	31.1 (19.1 to 48.0)	4.8 (4.8 to 4.8)	1048.8 (971.3 to 1130.4)	540.0 (490.0 to 594.8)	430.5 (386.1 to 484.2)	67.9 (41.7 to 104.7)	10.4 (10.4 to 10.4)
Mexico	2012	490.8 (456.0 to 528.6)	257.4 (234.6 to 282.3)	198.2 (176.7 to 223.4)	32.5 (20.0 to 50.8)	2.7 (2.7 to 2.7)	1070.8 (994.8 to 1153.1)	561.6 (511.8 to 615.8)	432.5 (385.5 to 487.3)	71.0 (43.6 to 110.9)	5.8 (5.8 to 5.8)
Mexico	2013	492.3 (457.8 to 530.6)	260.1 (238.1 to 283.4)	197.8 (175.9 to 221.5)	33.6 (20.6 to 52.5)	0.8 (0.8 to 0.8)	1074.0 (998.7 to 1157.6)	567.4 (519.5 to 618.2)	431.6 (383.7 to 483.2)	73.2 (44.9 to 114.5)	1.8 (1.8 to 1.8)
Mexico	2014	492.9 (457.1 to 532.4)	259.8 (236.0 to 284.8)	197.8 (176.5 to 221.4)	34.8 (21.3 to 54.1)	0.6 (0.6 to 0.6)	1075.4 (997.1 to 1161.5)	566.9 (514.9 to 621.3)	431.5 (385.0 to 483.0)	75.9 (46.4 to 118.1)	1.2 (1.2 to 1.2)
Mexico	2015	501.0 (460.8 to 546.1)	263.6 (237.1 to 290.4)	200.3 (175.9 to 225.6)	36.2 (21.6 to 58.0)	0.8 (0.8 to 0.8)	1092.9 (1005.3 to 1191.5)	575.1 (517.3 to 633.6)	437.1 (383.7 to 492.2)	79.0 (47.0 to 126.4)	1.8 (1.8 to 1.8)

		2018 US Dollars per capita					2018 PPP per capita				
Country	Year	Health spending	Government health spending	Out-of-pocket health spending	Prepaid private health spending	Development assistance for health	Health spending	Government health spending	Out-of-pocket health spending	Prepaid private health spending	Development assistance for health
Mexico	2016	504.6 (458.5 to 554.0)	264.6 (232.8 to 298.3)	201.6 (174.3 to 230.8)	37.7 (22.3 to 63.8)	0.7 (0.7 to 0.7)	1100.8 (1000.2 to 1208.7)	577.2 (507.9 to 650.9)	439.8 (380.2 to 503.6)	82.4 (48.5 to 139.1)	1.4 (1.4 to 1.4)
Mexico	2017	499.2 (455.5 to 548.5)	257.7 (226.8 to 291.0)	203.1 (175.4 to 232.7)	37.7 (22.2 to 63.7)	0.7 (0.7 to 0.7)	1089.1 (993.6 to 1196.5)	562.2 (494.8 to 634.8)	443.1 (382.7 to 507.7)	82.2 (48.5 to 139.0)	1.6 (1.6 to 1.6)
Mexico	2018	506.8 (460.7 to 556.9)	262.3 (231.1 to 295.6)	205.4 (177.2 to 235.2)	38.5 (22.7 to 65.1)	0.7 (0.7 to 0.7)	1105.7 (1005.2 to 1214.9)	572.1 (504.2 to 644.8)	448.0 (386.7 to 513.1)	84.0 (49.6 to 142.0)	1.5 (1.5 to 1.5)
Mexico	2019	514.4 (469.4 to 565.7)	266.9 (235.6 to 300.5)	207.7 (179.2 to 238.2)	39.2 (23.2 to 66.2)	0.7 (0.6 to 0.7)	1122.3 (1024.0 to 1234.2)	582.2 (513.9 to 655.6)	453.1 (390.9 to 519.7)	85.5 (50.5 to 144.4)	1.5 (1.4 to 1.6)
Mexico	2020	521.9 (473.8 to 573.6)	271.4 (239.5 to 305.2)	209.9 (181.1 to 240.6)	39.9 (23.5 to 67.3)	0.7 (0.6 to 0.8)	1138.5 (1033.7 to 1251.3)	592.1 (522.5 to 665.9)	457.9 (395.1 to 524.9)	87.0 (51.2 to 146.9)	1.5 (1.4 to 1.7)
Mexico	2021	531.2 (483.8 to 584.6)	277.7 (245.2 to 312.5)	212.1 (183.2 to 243.0)	40.7 (23.8 to 68.6)	0.7 (0.6 to 0.8)	1158.9 (1055.6 to 1275.4)	605.9 (534.9 to 681.8)	462.6 (399.6 to 530.1)	88.8 (52.0 to 149.7)	1.6 (1.4 to 1.8)
Mexico	2022	540.4 (491.1 to 593.2)	284.0 (251.1 to 320.2)	214.2 (185.0 to 245.2)	41.5 (24.2 to 69.9)	0.7 (0.6 to 0.8)	1179.0 (1071.4 to 1294.1)	619.6 (547.7 to 698.6)	467.3 (403.6 to 535.0)	90.5 (52.8 to 152.5)	1.6 (1.4 to 1.8)
Mexico	2023	548.7 (500.2 to 604.2)	289.9 (255.8 to 326.4)	215.9 (186.6 to 247.2)	42.2 (24.6 to 71.1)	0.7 (0.6 to 0.9)	1197.1 (1091.3 to 1318.2)	632.4 (558.1 to 712.2)	471.0 (407.1 to 539.3)	92.0 (53.7 to 155.1)	1.6 (1.4 to 1.9)
Mexico	2024	556.6 (505.1 to 611.7)	295.6 (260.6 to 332.6)	217.4 (188.0 to 248.9)	42.9 (25.0 to 72.2)	0.8 (0.7 to 0.9)	1214.3 (1101.9 to 1334.6)	644.8 (568.6 to 725.7)	474.4 (410.1 to 543.0)	93.5 (54.6 to 157.5)	1.7 (1.4 to 1.9)
Mexico	2025	563.3 (513.1 to 620.5)	300.1 (263.8 to 337.6)	218.9 (189.0 to 251.0)	43.5 (25.4 to 73.2)	0.8 (0.7 to 0.9)	1228.9 (1119.3 to 1353.7)	654.7 (575.5 to 736.4)	477.6 (412.4 to 547.5)	94.9 (55.5 to 159.7)	1.7 (1.4 to 2.0)
Mexico	2026	570.2 (516.7 to 627.5)	304.7 (267.8 to 342.9)	220.5 (190.5 to 253.0)	44.2 (25.9 to 74.1)	0.8 (0.7 to 0.9)	1243.9 (1127.3 to 1368.9)	664.9 (584.1 to 748.1)	481.0 (415.6 to 552.0)	96.3 (56.4 to 161.7)	1.7 (1.5 to 2.1)
Mexico	2027	577.1 (524.5 to 635.7)	309.5 (271.7 to 349.1)	222.0 (191.9 to 255.0)	44.8 (26.3 to 74.8)	0.8 (0.7 to 1.0)	1259.0 (1144.3 to 1387.0)	675.1 (592.7 to 761.5)	484.3 (418.7 to 556.3)	97.8 (57.3 to 163.3)	1.8 (1.5 to 2.2)
Mexico	2028	584.1 (528.9 to 644.2)	314.2 (275.4 to 355.0)	223.5 (193.4 to 256.6)	45.5 (26.7 to 75.6)	0.8 (0.7 to 1.0)	1274.3 (1153.9 to 1405.5)	685.5 (600.7 to 774.5)	487.7 (421.9 to 559.8)	99.2 (58.3 to 164.8)	1.8 (1.5 to 2.2)
Mexico	2029	591.1 (536.5 to 649.8)	319.1 (279.1 to 361.8)	225.1 (194.7 to 258.1)	46.2 (27.2 to 76.9)	0.9 (0.7 to 1.1)	1289.7 (1170.4 to 1417.7)	696.1 (609.0 to 789.2)	491.0 (424.7 to 563.2)	100.7 (59.3 to 167.8)	1.9 (1.5 to 2.3)
Mexico	2030	598.3 (542.1 to 659.5)	324.0 (282.8 to 367.9)	226.6 (195.7 to 259.8)	46.8 (27.7 to 78.4)	0.9 (0.7 to 1.1)	1305.2 (1182.7 to 1438.8)	706.9 (616.9 to 802.7)	494.3 (426.9 to 566.7)	102.2 (60.4 to 171.0)	1.9 (1.6 to 2.4)
Mexico	2031	605.6 (548.8 to 668.5)	329.1 (286.9 to 375.5)	228.1 (196.9 to 261.9)	47.5 (28.1 to 79.8)	0.9 (0.7 to 1.1)	1321.3 (1197.2 to 1458.4)	717.9 (626.0 to 819.2)	497.7 (429.6 to 571.4)	103.7 (61.4 to 174.1)	2.0 (1.6 to 2.5)
Mexico	2032	613.2 (554.1 to 677.4)	334.2 (290.6 to 382.1)	229.8 (198.3 to 263.7)	48.3 (28.6 to 81.0)	0.9 (0.7 to 1.2)	1337.7 (1208.9 to 1477.8)	729.2 (634.0 to 833.6)	501.3 (432.7 to 575.3)	105.3 (62.4 to 176.6)	2.0 (1.6 to 2.5)
Mexico	2033	620.5 (561.2 to 688.7)	339.3 (295.3 to 389.2)	231.3 (199.7 to 265.4)	49.0 (28.9 to 82.1)	0.9 (0.7 to 1.2)	1353.7 (1224.2 to 1502.4)	740.3 (644.2 to 849.1)	504.5 (435.6 to 579.0)	106.8 (63.1 to 179.1)	2.1 (1.6 to 2.7)
Mexico	2034	627.5 (565.7 to 696.8)	344.3 (298.4 to 395.5)	232.5 (200.8 to 266.9)	49.6 (29.4 to 83.2)	1.0 (0.8 to 1.3)	1368.9 (1234.1 to 1520.2)	751.2 (651.0 to 862.9)	507.3 (438.1 to 582.3)	108.3 (64.0 to 181.5)	2.1 (1.6 to 2.7)
Mexico	2035	634.7 (572.3 to 708.4)	349.6 (301.9 to 404.6)	233.8 (201.8 to 268.5)	50.3 (29.8 to 84.3)	1.0 (0.8 to 1.3)	1384.8 (1248.6 to 1545.4)	762.7 (658.7 to 882.7)	510.1 (440.3 to 585.7)	109.8 (65.0 to 184.0)	2.2 (1.7 to 2.8)
Mexico	2036	641.6 (575.1 to 715.4)	354.7 (305.0 to 412.2)	235.0 (202.7 to 269.9)	51.0 (30.2 to 85.4)	1.0 (0.8 to 1.4)	1399.8 (1254.6 to 1560.8)	773.7 (665.4 to 899.2)	512.6 (442.2 to 588.8)	111.2 (65.8 to 186.3)	2.2 (1.7 to 3.0)

Country	Year	2018 US Dollars per capita					2018 PPP per capita				
		Health spending	Government health spending	Out-of-pocket health spending	Prepaid private health spending	Development assistance for health	Health spending	Government health spending	Out-of-pocket health spending	Prepaid private health spending	Development assistance for health
Mexico	2037	648.2 (583.0 to 729.5)	359.7 (307.8 to 419.7)	235.9 (203.4 to 270.8)	51.6 (30.5 to 86.4)	1.0 (0.8 to 1.4)	1414.2 (1272.0 to 1591.6)	784.6 (671.5 to 915.7)	514.7 (443.7 to 590.8)	112.6 (66.6 to 188.5)	2.3 (1.7 to 3.1)
Mexico	2038	654.6 (585.1 to 734.6)	364.6 (310.5 to 429.6)	236.7 (204.0 to 271.6)	52.2 (30.8 to 87.7)	1.1 (0.8 to 1.5)	1428.1 (1276.5 to 1602.6)	795.3 (677.5 to 937.1)	516.4 (445.0 to 592.5)	114.0 (67.2 to 191.2)	2.4 (1.8 to 3.3)
Mexico	2039	661.1 (591.2 to 750.5)	369.5 (313.4 to 437.0)	237.6 (204.6 to 272.7)	52.9 (31.1 to 88.7)	1.1 (0.8 to 1.6)	1442.3 (1289.7 to 1637.2)	806.2 (683.7 to 953.4)	518.3 (446.4 to 595.0)	115.4 (67.9 to 193.5)	2.4 (1.8 to 3.4)
Mexico	2040	667.5 (594.1 to 755.2)	374.5 (316.5 to 445.8)	238.4 (205.3 to 273.8)	53.5 (31.3 to 89.4)	1.1 (0.9 to 1.6)	1456.3 (1296.2 to 1647.5)	817.0 (690.4 to 972.6)	520.1 (448.0 to 597.4)	116.7 (68.4 to 195.0)	2.5 (1.9 to 3.5)
Mexico	2041	674.0 (599.3 to 768.6)	379.4 (319.6 to 455.7)	239.3 (206.0 to 275.2)	54.1 (31.7 to 90.4)	1.2 (0.9 to 1.6)	1470.4 (1307.4 to 1676.8)	827.6 (697.3 to 994.3)	522.1 (449.5 to 600.4)	118.1 (69.2 to 197.3)	2.5 (1.9 to 3.6)
Mexico	2042	680.4 (603.2 to 776.9)	384.2 (322.1 to 466.7)	240.2 (206.7 to 276.5)	54.8 (32.1 to 91.6)	1.2 (0.9 to 1.7)	1484.3 (1316.0 to 1694.9)	838.1 (702.8 to 1018.1)	524.1 (450.9 to 603.2)	119.5 (70.0 to 199.8)	2.6 (1.9 to 3.8)
Mexico	2043	686.8 (609.9 to 791.2)	389.0 (324.7 to 473.7)	241.2 (207.4 to 277.7)	55.4 (32.4 to 92.8)	1.2 (0.9 to 1.8)	1498.5 (1330.6 to 1726.2)	848.6 (708.4 to 1033.4)	526.2 (452.5 to 605.8)	121.0 (70.6 to 202.5)	2.7 (2.0 to 3.9)
Mexico	2044	693.6 (612.9 to 797.1)	394.0 (327.5 to 482.7)	242.2 (208.0 to 278.9)	56.1 (32.7 to 94.1)	1.3 (0.9 to 1.9)	1513.1 (1337.2 to 1738.9)	859.5 (714.4 to 1053.1)	528.4 (453.9 to 608.6)	122.5 (71.4 to 205.3)	2.8 (2.0 to 4.1)
Mexico	2045	700.3 (619.1 to 810.3)	399.0 (330.1 to 492.9)	243.2 (208.8 to 280.2)	56.8 (33.1 to 95.5)	1.3 (0.9 to 2.0)	1527.9 (1350.6 to 1767.7)	870.5 (720.2 to 1075.4)	530.6 (455.4 to 611.3)	124.0 (72.2 to 208.3)	2.8 (2.0 to 4.3)
Mexico	2046	706.9 (622.5 to 816.6)	403.8 (332.6 to 499.5)	244.2 (209.6 to 281.4)	57.5 (33.4 to 96.9)	1.3 (0.0 to 2.1)	1542.2 (1358.1 to 1781.5)	881.1 (725.6 to 1089.8)	532.8 (457.3 to 614.0)	125.4 (73.0 to 211.3)	2.9 (0.0 to 4.5)
Mexico	2047	713.6 (627.6 to 829.8)	408.8 (335.9 to 508.4)	245.3 (210.3 to 282.9)	58.2 (33.8 to 98.3)	1.3 (0.0 to 2.2)	1556.8 (1369.2 to 1810.4)	891.8 (732.7 to 1109.2)	535.1 (458.9 to 617.1)	127.0 (73.7 to 214.4)	2.9 (0.0 to 4.7)
Mexico	2048	720.2 (631.5 to 838.6)	413.7 (338.7 to 517.8)	246.3 (211.0 to 284.6)	58.9 (34.1 to 99.7)	1.4 (0.0 to 2.3)	1571.3 (1377.7 to 1829.5)	902.5 (738.9 to 1129.8)	537.3 (460.4 to 620.8)	128.5 (74.5 to 217.5)	3.0 (0.0 to 5.1)
Mexico	2049	726.8 (637.0 to 848.9)	418.6 (341.9 to 525.7)	247.3 (211.7 to 285.8)	59.6 (34.5 to 100.6)	1.4 (0.0 to 2.4)	1585.7 (1389.7 to 1852.1)	913.2 (745.9 to 1146.9)	539.4 (461.9 to 623.6)	130.0 (75.2 to 219.5)	3.1 (0.0 to 5.3)
Mexico	2050	733.3 (640.7 to 858.3)	423.5 (344.9 to 534.1)	248.2 (212.3 to 287.4)	60.3 (34.8 to 101.9)	1.4 (0.0 to 2.5)	1599.9 (1397.8 to 1872.4)	923.9 (752.5 to 1165.2)	541.4 (463.2 to 627.1)	131.5 (76.0 to 222.3)	3.1 (0.0 to 5.5)
Moldova	1995	99.1 (82.9 to 117.3)	68.6 (55.2 to 85.2)	28.7 (20.3 to 38.8)	0.0 (0.0 to 0.1)	1.8 (1.8 to 1.8)	242.1 (202.5 to 286.6)	167.7 (134.8 to 208.1)	70.1 (49.7 to 94.8)	0.1 (0.0 to 0.2)	4.3 (4.3 to 4.3)
Moldova	1996	92.1 (77.4 to 109.9)	63.6 (51.7 to 78.5)	28.4 (20.7 to 37.3)	0.0 (0.0 to 0.1)	0.0 (0.0 to 0.0)	224.9 (189.2 to 268.4)	155.4 (126.4 to 191.7)	69.3 (50.7 to 91.2)	0.1 (0.0 to 0.1)	0.1 (0.1 to 0.1)
Moldova	1997	84.5 (71.9 to 100.7)	56.3 (45.8 to 69.7)	27.9 (20.7 to 36.8)	0.0 (0.0 to 0.0)	0.3 (0.3 to 0.3)	206.4 (175.5 to 245.9)	137.4 (111.8 to 170.2)	68.1 (50.5 to 89.8)	0.1 (0.0 to 0.1)	0.8 (0.8 to 0.8)
Moldova	1998	68.4 (58.0 to 80.9)	41.8 (34.1 to 51.6)	26.0 (19.5 to 33.9)	0.0 (0.0 to 0.0)	0.6 (0.6 to 0.6)	167.1 (141.7 to 197.7)	102.1 (83.2 to 126.0)	63.5 (47.6 to 82.7)	0.0 (0.0 to 0.1)	1.4 (1.4 to 1.4)
Moldova	1999	61.0 (52.4 to 71.8)	32.2 (26.3 to 39.9)	24.6 (18.6 to 32.2)	0.0 (0.0 to 0.0)	4.2 (4.2 to 4.2)	149.0 (128.1 to 175.4)	78.5 (64.1 to 97.6)	60.0 (45.5 to 78.7)	0.0 (0.0 to 0.0)	10.3 (10.3 to 10.3)
Moldova	2000	60.6 (52.2 to 70.8)	31.0 (25.2 to 38.2)	26.2 (20.1 to 34.2)	0.0 (0.0 to 0.0)	3.4 (3.4 to 3.4)	148.0 (127.4 to 172.8)	75.7 (61.6 to 93.4)	64.1 (49.1 to 83.4)	0.0 (0.0 to 0.0)	8.3 (8.3 to 8.3)
Moldova	2001	64.7 (55.7 to 74.9)	32.9 (26.8 to 40.3)	29.2 (22.6 to 37.2)	0.0 (0.0 to 0.0)	2.6 (2.6 to 2.6)	158.0 (136.1 to 183.1)	80.3 (65.4 to 98.4)	71.4 (55.2 to 91.0)	0.0 (0.0 to 0.0)	6.4 (6.4 to 6.4)

		2018 US Dollars per capita					2018 PPP per capita				
Country	Year	Health spending	Government health spending	Out-of-pocket health spending	Prepaid private health spending	Development assistance for health	Health spending	Government health spending	Out-of-pocket health spending	Prepaid private health spending	Development assistance for health
Moldova	2002	76.3 (66.1 to 88.0)	38.3 (31.4 to 47.0)	36.5 (28.6 to 46.2)	0.1 (0.1 to 0.3)	1.4 (1.4 to 1.4)	186.5 (161.5 to 214.9)	93.6 (76.7 to 114.9)	89.2 (69.8 to 113.0)	0.3 (0.2 to 0.7)	3.4 (3.4 to 3.4)
Moldova	2003	88.5 (77.4 to 101.5)	44.5 (36.6 to 54.0)	41.5 (32.7 to 52.7)	0.3 (0.2 to 0.6)	2.2 (2.2 to 2.2)	216.3 (189.1 to 247.9)	108.7 (89.5 to 131.8)	101.3 (80.0 to 128.7)	0.8 (0.4 to 1.5)	5.4 (5.4 to 5.4)
Moldova	2004	109.0 (95.2 to 124.8)	56.6 (46.9 to 67.9)	47.9 (38.2 to 60.2)	0.3 (0.2 to 0.7)	4.2 (4.2 to 4.2)	266.2 (232.5 to 304.9)	138.2 (114.5 to 165.8)	116.9 (93.2 to 147.1)	0.8 (0.4 to 1.6)	10.3 (10.3 to 10.3)
Moldova	2005	128.9 (112.6 to 147.5)	65.8 (54.6 to 78.9)	56.2 (45.2 to 70.5)	0.3 (0.1 to 0.5)	6.6 (6.6 to 6.6)	314.8 (274.9 to 360.4)	160.7 (133.5 to 192.6)	137.3 (110.4 to 172.2)	0.7 (0.3 to 1.3)	16.1 (16.1 to 16.1)
Moldova	2006	143.0 (124.0 to 164.5)	73.8 (61.7 to 87.7)	65.8 (52.9 to 82.1)	0.3 (0.1 to 0.6)	3.1 (3.1 to 3.1)	349.4 (303.0 to 401.7)	180.3 (150.6 to 214.2)	160.8 (129.3 to 200.7)	0.7 (0.3 to 1.4)	7.6 (7.6 to 7.6)
Moldova	2007	159.4 (139.4 to 181.3)	80.5 (67.6 to 95.6)	73.5 (59.9 to 91.6)	0.3 (0.2 to 0.7)	5.0 (5.0 to 5.0)	389.2 (340.4 to 442.8)	196.6 (165.1 to 233.5)	179.5 (146.2 to 223.7)	0.8 (0.4 to 1.6)	12.3 (12.3 to 12.3)
Moldova	2008	175.4 (154.3 to 198.3)	87.4 (73.6 to 103.0)	82.1 (66.6 to 100.7)	0.5 (0.2 to 0.9)	5.4 (5.4 to 5.4)	428.5 (377.0 to 484.3)	213.6 (179.7 to 251.6)	200.5 (162.8 to 246.0)	1.2 (0.5 to 2.2)	13.2 (13.2 to 13.2)
Moldova	2009	185.7 (164.4 to 208.0)	86.9 (73.7 to 102.1)	86.2 (70.2 to 104.8)	1.1 (0.5 to 2.0)	11.5 (11.5 to 11.5)	453.5 (401.7 to 508.2)	212.3 (179.9 to 249.3)	210.6 (171.6 to 256.1)	2.6 (1.2 to 5.0)	28.0 (28.0 to 28.0)
Moldova	2010	193.6 (172.3 to 216.4)	91.6 (78.1 to 107.0)	87.0 (71.2 to 105.8)	1.8 (0.8 to 3.3)	13.2 (13.2 to 13.2)	472.8 (420.8 to 528.5)	223.9 (190.7 to 261.3)	212.5 (174.0 to 258.5)	4.4 (2.0 to 8.1)	32.1 (32.1 to 32.1)
Moldova	2011	194.9 (172.9 to 217.8)	95.4 (80.9 to 111.8)	88.1 (71.8 to 106.6)	2.1 (1.0 to 3.8)	9.3 (9.3 to 9.3)	476.1 (422.3 to 531.9)	233.1 (197.6 to 273.1)	215.3 (175.3 to 260.4)	5.1 (2.4 to 9.3)	22.6 (22.6 to 22.6)
Moldova	2012	191.4 (169.1 to 214.3)	93.9 (79.8 to 109.7)	85.6 (69.5 to 104.7)	2.1 (1.0 to 3.8)	9.7 (9.7 to 9.7)	467.5 (413.0 to 523.4)	229.5 (195.0 to 268.0)	209.2 (169.9 to 255.8)	5.1 (2.4 to 9.2)	23.7 (23.7 to 23.7)
Moldova	2013	195.6 (173.0 to 220.8)	97.9 (82.2 to 115.4)	87.1 (70.7 to 106.8)	2.0 (0.9 to 3.6)	8.6 (8.6 to 8.6)	477.9 (422.7 to 539.2)	239.2 (200.7 to 281.8)	212.8 (172.6 to 261.0)	4.9 (2.3 to 8.9)	21.1 (21.1 to 21.1)
Moldova	2014	203.2 (179.8 to 230.1)	100.8 (84.7 to 120.1)	88.1 (71.2 to 108.7)	2.1 (1.0 to 3.9)	12.3 (12.3 to 12.3)	496.4 (439.1 to 562.1)	246.3 (206.8 to 293.3)	215.2 (173.8 to 265.4)	5.0 (2.3 to 9.5)	29.9 (29.9 to 29.9)
Moldova	2015	205.3 (180.0 to 234.8)	98.9 (81.7 to 118.6)	91.1 (72.9 to 114.2)	2.1 (1.0 to 4.0)	13.1 (13.1 to 13.1)	501.5 (439.8 to 573.5)	241.6 (199.6 to 289.6)	222.6 (178.1 to 279.0)	5.2 (2.5 to 9.7)	32.1 (32.1 to 32.1)
Moldova	2016	203.9 (176.7 to 235.1)	102.3 (84.0 to 124.2)	92.7 (73.0 to 118.7)	2.3 (1.1 to 4.4)	6.6 (6.6 to 6.6)	498.2 (431.7 to 574.2)	249.8 (205.2 to 303.5)	226.6 (178.3 to 289.9)	5.7 (2.8 to 10.7)	16.1 (16.1 to 16.1)
Moldova	2017	211.8 (182.6 to 246.6)	107.2 (88.2 to 130.3)	96.6 (75.8 to 124.1)	2.4 (1.2 to 4.5)	5.6 (5.6 to 5.6)	517.3 (446.0 to 602.2)	261.8 (215.5 to 318.4)	235.9 (185.2 to 303.2)	5.9 (2.9 to 11.0)	13.6 (13.6 to 13.6)
Moldova	2018	215.4 (186.3 to 248.3)	108.7 (89.4 to 132.3)	99.1 (78.1 to 126.4)	2.5 (1.2 to 4.6)	5.1 (5.1 to 5.1)	526.2 (455.0 to 606.4)	265.6 (218.5 to 323.2)	242.1 (190.9 to 308.8)	6.0 (2.9 to 11.2)	12.5 (12.3 to 12.6)
Moldova	2019	219.4 (189.5 to 255.1)	110.1 (90.5 to 134.0)	101.6 (80.1 to 129.9)	2.5 (1.2 to 4.6)	5.3 (4.9 to 5.6)	535.9 (462.8 to 623.0)	268.8 (221.0 to 327.3)	248.1 (195.7 to 317.2)	6.1 (2.9 to 11.3)	12.9 (12.0 to 13.6)
Moldova	2020	223.4 (192.4 to 258.2)	111.4 (91.6 to 135.6)	104.0 (81.8 to 132.6)	2.5 (1.2 to 4.7)	5.5 (5.0 to 5.9)	545.7 (470.1 to 630.6)	272.1 (223.8 to 331.2)	254.1 (199.9 to 323.8)	6.2 (3.0 to 11.5)	13.3 (12.2 to 14.4)
Moldova	2021	227.8 (196.6 to 265.2)	112.8 (92.7 to 137.4)	106.7 (83.9 to 136.3)	2.6 (1.2 to 4.8)	5.7 (5.1 to 6.3)	556.4 (480.1 to 647.9)	275.6 (226.3 to 335.5)	260.8 (204.9 to 332.9)	6.3 (3.0 to 11.7)	13.8 (12.4 to 15.3)
Moldova	2022	229.9 (197.7 to 265.2)	114.1 (93.6 to 139.0)	107.3 (84.6 to 136.5)	2.6 (1.3 to 4.8)	5.9 (5.2 to 6.6)	561.5 (482.8 to 647.8)	278.7 (228.7 to 339.4)	262.0 (206.6 to 333.4)	6.4 (3.1 to 11.8)	14.4 (12.7 to 16.1)

Country	Year	2018 US Dollars per capita					2018 PPP per capita				
		Health spending	Government health spending	Out-of-pocket health spending	Prepaid private health spending	Development assistance for health	Health spending	Government health spending	Out-of-pocket health spending	Prepaid private health spending	Development assistance for health
Moldova	2023	231.9 (200.7 to 269.9)	115.4 (94.6 to 140.9)	107.7 (85.1 to 136.8)	2.6 (1.3 to 4.9)	6.2 (5.4 to 7.1)	566.5 (490.4 to 659.2)	281.9 (231.1 to 344.1)	263.1 (207.8 to 334.1)	6.5 (3.1 to 12.0)	15.1 (13.2 to 17.2)
Moldova	2024	234.0 (201.9 to 271.2)	116.6 (95.6 to 142.6)	108.2 (85.4 to 137.8)	2.7 (1.3 to 5.0)	6.5 (5.6 to 7.6)	571.6 (493.1 to 662.4)	284.9 (233.4 to 348.2)	264.2 (208.5 to 336.7)	6.5 (3.2 to 12.1)	16.0 (13.8 to 18.5)
Moldova	2025	236.4 (204.7 to 275.8)	118.0 (96.6 to 144.3)	108.8 (85.9 to 138.2)	2.7 (1.3 to 5.0)	6.9 (5.8 to 8.1)	577.4 (500.1 to 673.6)	288.3 (236.1 to 352.4)	265.7 (209.8 to 337.6)	6.6 (3.2 to 12.3)	16.8 (14.2 to 19.7)
Moldova	2026	238.5 (205.5 to 276.1)	119.4 (97.5 to 146.2)	109.3 (86.3 to 138.6)	2.8 (1.3 to 5.1)	7.1 (6.0 to 8.3)	582.6 (501.9 to 674.5)	291.5 (238.2 to 357.2)	266.9 (210.8 to 338.5)	6.7 (3.2 to 12.5)	17.4 (14.6 to 20.4)
Moldova	2027	240.8 (208.5 to 280.9)	120.8 (98.5 to 148.2)	109.9 (86.7 to 138.7)	2.8 (1.3 to 5.2)	7.4 (6.2 to 8.8)	588.1 (509.4 to 686.2)	295.0 (240.5 to 362.0)	268.3 (211.8 to 338.7)	6.8 (3.3 to 12.6)	18.0 (15.1 to 21.6)
Moldova	2028	243.3 (210.7 to 282.5)	122.3 (99.7 to 150.3)	110.5 (87.2 to 139.1)	2.8 (1.4 to 5.3)	7.6 (6.4 to 9.3)	594.3 (514.7 to 690.0)	298.7 (243.4 to 367.1)	270.0 (213.1 to 339.8)	6.9 (3.3 to 12.9)	18.7 (15.6 to 22.6)
Moldova	2029	246.5 (213.5 to 286.7)	124.2 (101.1 to 152.5)	111.5 (88.0 to 140.3)	2.9 (1.4 to 5.4)	7.9 (6.5 to 9.8)	602.0 (521.5 to 700.2)	303.3 (247.0 to 372.6)	272.4 (214.9 to 342.7)	7.0 (3.4 to 13.1)	19.4 (15.9 to 23.8)
Moldova	2030	250.1 (216.0 to 290.5)	126.3 (102.8 to 155.3)	112.7 (88.9 to 142.0)	2.9 (1.4 to 5.5)	8.2 (6.7 to 10.1)	610.8 (527.6 to 709.5)	308.4 (251.1 to 379.2)	275.2 (217.1 to 346.9)	7.2 (3.4 to 13.4)	20.0 (16.4 to 24.6)
Moldova	2031	254.1 (219.1 to 295.8)	128.6 (104.6 to 158.3)	114.0 (89.8 to 144.0)	3.0 (1.4 to 5.6)	8.5 (6.9 to 10.6)	620.7 (535.3 to 722.5)	314.2 (255.5 to 386.6)	278.5 (219.4 to 351.8)	7.3 (3.5 to 13.7)	20.7 (16.8 to 25.9)
Moldova	2032	258.7 (222.7 to 301.0)	131.2 (106.5 to 161.6)	115.6 (90.8 to 146.2)	3.1 (1.5 to 5.7)	8.8 (7.1 to 11.1)	631.8 (544.0 to 735.2)	320.5 (260.2 to 394.8)	282.3 (221.9 to 357.2)	7.5 (3.6 to 14.0)	21.5 (17.3 to 27.2)
Moldova	2033	263.6 (227.5 to 307.7)	134.1 (108.7 to 165.0)	117.3 (92.0 to 148.7)	3.1 (1.5 to 5.9)	9.1 (7.2 to 11.6)	644.0 (555.7 to 751.6)	327.5 (265.5 to 403.1)	286.6 (224.7 to 363.3)	7.7 (3.6 to 14.3)	22.2 (17.7 to 28.4)
Moldova	2034	268.8 (231.7 to 312.3)	137.0 (110.9 to 169.0)	119.1 (93.2 to 151.3)	3.2 (1.5 to 6.0)	9.4 (7.4 to 12.0)	656.6 (565.9 to 762.9)	334.8 (270.9 to 412.8)	291.0 (227.7 to 369.6)	7.9 (3.7 to 14.7)	23.0 (18.1 to 29.4)
Moldova	2035	273.9 (235.3 to 319.2)	140.0 (113.1 to 172.8)	120.9 (94.4 to 153.8)	3.3 (1.6 to 6.1)	9.8 (7.6 to 12.7)	669.1 (574.7 to 779.7)	341.9 (276.2 to 422.1)	295.3 (230.6 to 375.6)	8.1 (3.8 to 15.0)	23.8 (18.6 to 31.1)
Moldova	2036	278.7 (240.1 to 326.1)	142.8 (115.1 to 176.6)	122.5 (95.2 to 156.1)	3.4 (1.6 to 6.3)	10.1 (7.8 to 13.6)	680.8 (586.5 to 796.5)	348.7 (281.2 to 431.3)	299.2 (232.5 to 381.2)	8.3 (3.9 to 15.3)	24.7 (19.1 to 33.3)
Moldova	2037	283.4 (242.9 to 331.5)	145.5 (117.1 to 180.7)	124.0 (96.1 to 158.1)	3.5 (1.6 to 6.4)	10.5 (8.1 to 14.1)	692.4 (593.2 to 809.7)	355.4 (286.2 to 441.4)	302.9 (234.8 to 386.3)	8.4 (4.0 to 15.7)	25.6 (19.7 to 34.5)
Moldova	2038	288.2 (248.1 to 337.3)	148.3 (119.2 to 184.0)	125.5 (97.4 to 160.1)	3.5 (1.7 to 6.5)	10.9 (8.3 to 14.9)	704.0 (606.0 to 824.0)	362.2 (291.1 to 449.5)	306.6 (237.8 to 391.1)	8.6 (4.1 to 16.0)	26.5 (20.2 to 36.4)
Moldova	2039	292.8 (250.6 to 342.3)	151.0 (121.1 to 187.5)	127.0 (98.4 to 161.8)	3.6 (1.7 to 6.7)	11.2 (8.5 to 15.3)	715.2 (612.2 to 836.1)	368.8 (295.9 to 457.9)	310.1 (240.3 to 395.2)	8.8 (4.2 to 16.3)	27.5 (20.8 to 37.4)
Moldova	2040	297.4 (255.7 to 347.2)	153.7 (123.1 to 191.4)	128.4 (99.5 to 163.7)	3.7 (1.7 to 6.8)	11.7 (8.9 to 16.0)	726.5 (624.7 to 848.2)	375.4 (300.6 to 467.4)	313.6 (243.1 to 399.8)	9.0 (4.2 to 16.6)	28.5 (21.7 to 39.2)
Moldova	2041	301.6 (258.0 to 353.8)	156.2 (124.9 to 194.9)	129.6 (100.6 to 165.4)	3.7 (1.8 to 6.9)	12.1 (9.1 to 17.2)	736.7 (630.3 to 864.2)	381.4 (305.0 to 476.1)	316.5 (245.7 to 404.1)	9.1 (4.3 to 16.9)	29.6 (22.3 to 41.9)
Moldova	2042	305.7 (262.9 to 357.7)	158.6 (126.6 to 198.3)	130.7 (101.5 to 167.0)	3.8 (1.8 to 7.0)	12.6 (9.4 to 18.3)	746.8 (642.2 to 873.7)	387.4 (309.2 to 484.3)	319.2 (247.9 to 408.0)	9.3 (4.4 to 17.2)	30.8 (22.9 to 44.7)
Moldova	2043	309.8 (264.9 to 364.1)	161.0 (128.3 to 202.0)	131.8 (102.1 to 168.6)	3.9 (1.8 to 7.2)	13.1 (9.7 to 19.4)	756.8 (647.0 to 889.4)	393.3 (313.4 to 493.3)	322.0 (249.3 to 411.7)	9.5 (4.5 to 17.5)	32.1 (23.6 to 47.3)

		2018 US Dollars per capita					2018 PPP per capita				
Country	Year	Health spending	Government health spending	Out-of-pocket health spending	Prepaid private health spending	Development assistance for health	Health spending	Government health spending	Out-of-pocket health spending	Prepaid private health spending	Development assistance for health
Moldova	2044	313.9 (269.6 to 366.8)	163.4 (129.8 to 205.2)	132.8 (102.5 to 170.0)	3.9 (1.9 to 7.3)	13.7 (10.0 to 20.0)	766.7 (658.5 to 896.1)	399.2 (317.2 to 501.3)	324.5 (250.4 to 415.3)	9.6 (4.5 to 17.8)	33.4 (24.5 to 48.9)
Moldova	2045	317.8 (270.5 to 374.3)	165.7 (131.6 to 208.1)	133.8 (102.9 to 171.4)	4.0 (1.9 to 7.4)	14.2 (10.3 to 21.6)	776.3 (660.8 to 914.2)	404.8 (321.5 to 508.3)	326.9 (251.4 to 418.6)	9.8 (4.6 to 18.1)	34.8 (25.2 to 52.7)
Moldova	2046	321.2 (275.6 to 379.4)	167.8 (133.0 to 210.6)	134.5 (103.3 to 172.4)	4.1 (1.9 to 7.5)	14.9 (10.8 to 22.7)	784.6 (673.3 to 926.8)	409.8 (324.8 to 514.5)	328.6 (252.2 to 421.1)	10.0 (4.7 to 18.4)	36.3 (26.3 to 55.5)
Moldova	2047	324.5 (275.4 to 383.3)	169.7 (134.0 to 213.9)	135.1 (103.6 to 173.3)	4.1 (1.9 to 7.6)	15.5 (11.2 to 24.6)	792.7 (672.6 to 936.3)	414.6 (327.4 to 522.4)	330.1 (253.1 to 423.4)	10.1 (4.7 to 18.7)	37.9 (27.4 to 60.1)
Moldova	2048	327.8 (281.1 to 385.0)	171.7 (135.5 to 217.2)	135.8 (104.0 to 174.3)	4.2 (2.0 to 7.8)	16.2 (11.5 to 26.0)	800.8 (686.6 to 940.5)	419.3 (331.0 to 530.4)	331.6 (254.1 to 425.7)	10.2 (4.8 to 19.0)	39.7 (28.2 to 63.4)
Moldova	2049	330.5 (280.2 to 390.6)	173.2 (136.6 to 220.0)	136.1 (104.3 to 174.8)	4.2 (2.0 to 7.8)	17.0 (12.0 to 27.5)	807.3 (684.5 to 954.2)	423.2 (333.6 to 537.3)	332.3 (254.7 to 426.9)	10.4 (4.8 to 19.2)	41.5 (29.4 to 67.1)
Moldova	2050	332.9 (284.6 to 392.4)	174.6 (137.5 to 222.2)	136.2 (104.4 to 174.9)	4.3 (2.0 to 7.9)	17.8 (12.5 to 28.6)	813.1 (695.2 to 958.5)	426.5 (335.8 to 542.7)	332.6 (255.1 to 427.2)	10.5 (4.9 to 19.3)	43.5 (30.5 to 69.9)
Mongolia	1995	57.0 (46.8 to 68.5)	43.3 (33.9 to 54.3)	8.9 (6.2 to 12.6)	2.3 (1.1 to 4.3)	2.4 (2.4 to 2.4)	192.8 (158.2 to 231.5)	146.5 (114.6 to 183.7)	30.2 (21.1 to 42.5)	7.8 (3.6 to 14.5)	8.3 (8.3 to 8.3)
Mongolia	1996	57.8 (47.7 to 69.4)	44.5 (35.0 to 55.3)	9.1 (6.4 to 12.7)	2.0 (0.9 to 3.8)	2.3 (2.3 to 2.3)	195.6 (161.2 to 234.5)	150.5 (118.3 to 186.9)	30.7 (21.7 to 42.9)	6.7 (3.1 to 12.8)	7.7 (7.7 to 7.7)
Mongolia	1997	60.1 (49.5 to 71.9)	47.1 (37.5 to 57.8)	9.5 (6.6 to 13.1)	2.1 (0.9 to 3.9)	1.4 (1.4 to 1.4)	203.0 (167.5 to 243.0)	159.2 (126.7 to 195.3)	32.0 (22.5 to 44.2)	7.0 (3.1 to 13.3)	4.8 (4.8 to 4.8)
Mongolia	1998	64.9 (53.8 to 76.8)	49.6 (39.7 to 60.5)	10.1 (7.1 to 13.9)	1.6 (0.8 to 3.1)	3.6 (3.6 to 3.6)	219.4 (181.8 to 259.7)	167.6 (134.2 to 204.6)	34.0 (23.9 to 47.1)	5.5 (2.5 to 10.6)	12.3 (12.3 to 12.3)
Mongolia	1999	70.7 (59.7 to 83.6)	52.0 (41.6 to 63.9)	11.6 (8.1 to 15.9)	2.5 (1.2 to 4.8)	4.7 (4.7 to 4.7)	239.2 (201.8 to 282.6)	175.7 (140.6 to 216.0)	39.1 (27.5 to 53.6)	8.4 (3.9 to 16.1)	16.0 (16.0 to 16.0)
Mongolia	2000	76.2 (64.3 to 90.3)	55.8 (45.2 to 68.2)	14.7 (10.4 to 20.1)	3.3 (1.5 to 6.1)	2.4 (2.4 to 2.4)	257.7 (217.4 to 305.2)	188.8 (152.8 to 230.7)	49.8 (35.0 to 67.8)	11.0 (5.0 to 20.6)	8.1 (8.1 to 8.1)
Mongolia	2001	81.9 (70.4 to 96.2)	55.1 (45.1 to 67.0)	16.7 (11.9 to 22.5)	3.4 (1.6 to 6.3)	6.8 (6.8 to 6.8)	277.0 (238.0 to 325.4)	186.3 (152.4 to 226.6)	56.3 (40.2 to 76.1)	11.5 (5.3 to 21.3)	22.9 (22.9 to 22.9)
Mongolia	2002	75.6 (64.1 to 89.2)	53.8 (44.2 to 65.2)	17.9 (13.0 to 24.0)	3.3 (1.5 to 6.0)	0.7 (0.7 to 0.7)	255.6 (216.6 to 301.5)	181.8 (149.3 to 220.5)	60.7 (44.0 to 81.2)	11.0 (5.2 to 20.3)	2.2 (2.2 to 2.2)
Mongolia	2003	73.9 (62.5 to 86.4)	50.5 (41.4 to 61.3)	18.6 (13.7 to 24.6)	2.9 (1.3 to 5.4)	1.9 (1.9 to 1.9)	249.7 (211.4 to 292.2)	170.8 (140.0 to 207.2)	62.8 (46.3 to 83.1)	9.7 (4.5 to 18.3)	6.4 (6.4 to 6.4)
Mongolia	2004	74.5 (63.1 to 87.5)	50.0 (40.4 to 60.6)	20.1 (14.8 to 26.7)	2.9 (1.3 to 5.4)	1.5 (1.5 to 1.5)	252.0 (213.4 to 295.7)	169.1 (136.7 to 205.0)	68.0 (50.2 to 90.2)	9.9 (4.5 to 18.3)	5.1 (5.1 to 5.1)
Mongolia	2005	75.5 (64.0 to 87.8)	48.7 (39.7 to 59.2)	21.4 (15.8 to 28.3)	2.7 (1.3 to 5.1)	2.6 (2.6 to 2.6)	255.3 (216.4 to 296.8)	164.8 (134.2 to 200.3)	72.5 (53.6 to 95.6)	9.3 (4.3 to 17.2)	8.8 (8.8 to 8.8)
Mongolia	2006	77.3 (65.2 to 89.9)	50.6 (41.2 to 61.8)	22.0 (16.2 to 28.8)	2.8 (1.3 to 5.3)	2.0 (2.0 to 2.0)	261.3 (220.4 to 303.9)	170.9 (139.5 to 209.1)	74.2 (54.8 to 97.5)	9.6 (4.5 to 18.1)	6.6 (6.6 to 6.6)
Mongolia	2007	87.4 (74.4 to 101.4)	55.4 (45.0 to 67.8)	23.4 (17.1 to 30.6)	2.9 (1.4 to 5.5)	5.8 (5.8 to 5.8)	295.6 (251.5 to 342.8)	187.3 (152.3 to 229.1)	79.0 (57.9 to 103.6)	9.8 (4.6 to 18.5)	19.4 (19.4 to 19.4)
Mongolia	2008	91.8 (78.5 to 106.3)	59.0 (48.3 to 73.2)	25.1 (18.4 to 32.7)	2.8 (1.3 to 5.2)	5.0 (5.0 to 5.0)	310.4 (265.5 to 359.3)	199.5 (163.3 to 247.6)	84.7 (62.3 to 110.6)	9.4 (4.5 to 17.7)	16.8 (16.8 to 16.8)

		2018 US Dollars per capita					2018 PPP per capita				
Country	Year	Health spending	Government health spending	Out-of-pocket health spending	Prepaid private health spending	Development assistance for health	Health spending	Government health spending	Out-of-pocket health spending	Prepaid private health spending	Development assistance for health
Mongolia	2009	89.4 (76.6 to 102.8)	55.2 (45.6 to 68.6)	25.7 (18.9 to 33.6)	2.5 (1.2 to 4.5)	6.1 (6.1 to 6.1)	302.3 (258.8 to 347.6)	186.7 (154.3 to 231.8)	86.7 (64.0 to 113.5)	8.3 (4.0 to 15.3)	20.5 (20.5 to 20.5)
Mongolia	2010	97.9 (85.1 to 111.7)	54.1 (44.6 to 66.5)	28.0 (20.7 to 36.4)	2.4 (1.1 to 4.3)	13.4 (13.4 to 13.4)	330.9 (287.8 to 377.5)	183.0 (150.6 to 224.9)	94.7 (70.1 to 123.1)	8.1 (3.9 to 14.7)	45.1 (45.1 to 45.1)
Mongolia	2011	104.4 (91.0 to 119.2)	57.9 (47.4 to 71.0)	33.1 (24.8 to 42.7)	2.6 (1.3 to 4.6)	10.9 (10.9 to 10.9)	353.1 (307.5 to 402.9)	195.8 (160.4 to 240.2)	111.9 (83.9 to 144.4)	8.7 (4.2 to 15.6)	36.7 (36.7 to 36.7)
Mongolia	2012	115.1 (99.9 to 131.2)	63.4 (52.1 to 76.9)	39.0 (29.3 to 50.5)	2.8 (1.3 to 5.0)	9.8 (9.8 to 9.8)	389.0 (337.8 to 443.6)	214.3 (176.3 to 259.9)	132.0 (99.0 to 170.8)	9.5 (4.5 to 16.7)	33.2 (33.2 to 33.2)
Mongolia	2013	129.7 (113.0 to 148.7)	68.5 (57.0 to 83.8)	46.3 (35.0 to 59.2)	3.2 (1.5 to 5.7)	11.8 (11.8 to 11.8)	438.6 (382.1 to 502.7)	231.6 (192.8 to 283.2)	156.5 (118.5 to 200.1)	10.8 (5.1 to 19.2)	39.8 (39.8 to 39.8)
Mongolia	2014	135.8 (116.9 to 156.7)	72.8 (60.4 to 90.1)	50.9 (38.4 to 64.9)	3.7 (1.8 to 6.7)	8.3 (8.3 to 8.3)	459.3 (395.1 to 529.9)	246.2 (204.1 to 304.6)	172.3 (129.8 to 219.3)	12.6 (6.0 to 22.6)	28.2 (28.2 to 28.2)
Mongolia	2015	142.0 (122.1 to 164.8)	75.4 (61.7 to 93.5)	53.4 (40.2 to 68.6)	4.5 (2.1 to 8.0)	8.7 (8.7 to 8.7)	480.2 (412.9 to 557.1)	255.0 (208.6 to 316.3)	180.5 (136.0 to 231.9)	15.2 (7.1 to 27.1)	29.5 (29.5 to 29.5)
Mongolia	2016	149.7 (129.0 to 174.5)	78.1 (63.2 to 96.9)	53.2 (39.5 to 69.3)	4.9 (2.3 to 8.7)	13.5 (13.5 to 13.5)	506.1 (436.0 to 590.1)	264.0 (213.6 to 327.8)	179.9 (133.4 to 234.1)	16.6 (7.8 to 29.3)	45.7 (45.7 to 45.7)
Mongolia	2017	148.3 (128.6 to 172.2)	67.3 (54.3 to 83.3)	55.6 (41.6 to 73.0)	5.1 (2.4 to 9.0)	20.4 (20.4 to 20.4)	501.4 (434.8 to 582.3)	227.4 (183.6 to 281.6)	188.1 (140.6 to 246.7)	17.1 (8.1 to 30.4)	68.8 (68.8 to 68.8)
Mongolia	2018	151.6 (130.4 to 175.7)	69.0 (55.5 to 85.7)	58.3 (43.5 to 76.5)	5.3 (2.5 to 9.4)	19.1 (19.0 to 19.1)	512.7 (440.9 to 594.1)	233.4 (187.6 to 289.9)	197.0 (146.9 to 258.7)	17.9 (8.4 to 31.6)	64.5 (64.3 to 64.6)
Mongolia	2019	155.9 (134.4 to 182.4)	70.6 (56.6 to 87.8)	61.0 (45.4 to 80.1)	5.4 (2.6 to 9.6)	18.9 (17.6 to 20.0)	527.1 (454.5 to 616.7)	238.7 (191.5 to 296.8)	206.3 (153.6 to 270.7)	18.3 (8.7 to 32.5)	63.8 (59.5 to 67.5)
Mongolia	2020	159.7 (138.1 to 185.3)	72.1 (58.1 to 89.2)	63.0 (46.6 to 82.7)	5.6 (2.6 to 9.9)	19.1 (17.4 to 20.6)	539.8 (467.0 to 626.5)	243.7 (196.5 to 301.7)	212.9 (157.5 to 279.5)	18.8 (8.9 to 33.3)	64.4 (59.0 to 69.7)
Mongolia	2021	163.6 (141.1 to 191.2)	74.0 (59.7 to 91.8)	64.5 (47.4 to 84.4)	5.7 (2.7 to 10.1)	19.3 (17.4 to 21.4)	553.0 (477.0 to 646.3)	250.3 (201.7 to 310.3)	218.1 (160.4 to 285.4)	19.3 (9.1 to 34.2)	65.3 (58.8 to 72.3)
Mongolia	2022	167.2 (144.7 to 194.2)	75.9 (61.0 to 94.4)	65.4 (48.1 to 85.5)	5.8 (2.8 to 10.4)	20.1 (17.8 to 22.6)	565.4 (489.2 to 656.7)	256.7 (206.4 to 319.0)	221.1 (162.6 to 289.2)	19.7 (9.3 to 35.0)	67.8 (60.1 to 76.2)
Mongolia	2023	170.8 (146.8 to 199.0)	77.8 (62.6 to 96.9)	66.0 (48.5 to 86.5)	5.9 (2.8 to 10.6)	21.1 (18.2 to 24.4)	577.6 (496.2 to 672.8)	263.0 (211.6 to 327.5)	223.2 (164.1 to 292.4)	20.1 (9.5 to 35.7)	71.3 (61.4 to 82.5)
Mongolia	2024	174.7 (150.2 to 203.5)	79.6 (64.1 to 99.1)	66.7 (48.9 to 87.1)	6.0 (2.9 to 10.8)	22.2 (18.8 to 26.2)	590.6 (508.0 to 688.0)	269.3 (216.6 to 335.2)	225.6 (165.3 to 294.6)	20.4 (9.7 to 36.4)	75.2 (63.4 to 88.7)
Mongolia	2025	179.6 (153.0 to 209.6)	81.6 (65.6 to 102.3)	68.5 (50.3 to 90.0)	6.2 (2.9 to 11.0)	23.3 (19.5 to 27.9)	607.2 (517.4 to 708.5)	275.8 (221.8 to 345.7)	231.7 (169.9 to 304.3)	20.8 (9.9 to 37.1)	78.9 (65.8 to 94.4)
Mongolia	2026	184.8 (157.8 to 217.8)	83.8 (67.1 to 105.1)	70.5 (51.4 to 93.8)	6.3 (3.0 to 11.2)	24.2 (20.2 to 28.9)	624.9 (533.5 to 736.3)	283.4 (227.0 to 355.4)	238.3 (173.7 to 317.0)	21.3 (10.1 to 37.9)	81.9 (68.1 to 97.7)
Mongolia	2027	190.5 (163.0 to 224.4)	86.3 (69.0 to 108.8)	72.6 (52.6 to 97.9)	6.4 (3.1 to 11.5)	25.1 (20.7 to 30.6)	644.2 (551.1 to 758.6)	291.8 (233.3 to 367.7)	245.6 (177.8 to 331.0)	21.8 (10.3 to 38.8)	85.0 (69.9 to 103.6)
Mongolia	2028	196.7 (166.9 to 231.8)	89.1 (71.1 to 112.5)	75.0 (53.9 to 102.6)	6.6 (3.1 to 11.8)	26.0 (21.4 to 31.7)	665.1 (564.3 to 783.8)	301.4 (240.5 to 380.2)	253.4 (182.1 to 346.9)	22.4 (10.6 to 39.8)	88.0 (72.5 to 107.2)
Mongolia	2029	203.2 (171.4 to 240.8)	92.1 (73.2 to 116.5)	77.4 (54.9 to 107.3)	6.8 (3.2 to 12.1)	26.9 (21.8 to 33.3)	686.8 (579.6 to 814.2)	311.3 (247.5 to 394.0)	261.7 (185.7 to 362.6)	23.0 (10.9 to 41.0)	90.8 (73.8 to 112.7)

		2018 US Dollars per capita					2018 PPP per capita				
Country	Year	Health spending	Government health spending	Out-of-pocket health spending	Prepaid private health spending	Development assistance for health	Health spending	Government health spending	Out-of-pocket health spending	Prepaid private health spending	Development assistance for health
Mongolia	2030	209.7 (177.0 to 247.8)	95.2 (75.6 to 120.3)	80.0 (56.1 to 112.4)	7.0 (3.3 to 12.5)	27.6 (22.3 to 34.3)	709.1 (598.3 to 837.9)	321.7 (255.7 to 406.8)	270.4 (189.6 to 379.9)	23.7 (11.2 to 42.2)	93.3 (75.4 to 115.9)
Mongolia	2031	216.5 (181.5 to 256.8)	98.3 (77.9 to 124.6)	82.7 (57.3 to 118.1)	7.2 (3.4 to 12.9)	28.3 (22.6 to 35.9)	731.8 (613.8 to 868.2)	332.3 (263.2 to 421.3)	279.4 (193.7 to 399.4)	24.4 (11.5 to 43.5)	95.7 (76.3 to 121.5)
Mongolia	2032	223.4 (185.8 to 265.7)	101.6 (80.6 to 128.9)	85.5 (58.6 to 123.6)	7.4 (3.5 to 13.3)	29.0 (22.9 to 37.0)	755.4 (628.1 to 898.3)	343.4 (272.6 to 435.9)	288.9 (198.2 to 417.7)	25.1 (11.9 to 44.9)	97.9 (77.3 to 125.0)
Mongolia	2033	230.7 (191.5 to 279.6)	105.0 (83.1 to 133.2)	88.4 (60.0 to 128.9)	7.7 (3.6 to 13.7)	29.6 (23.1 to 38.5)	779.8 (647.3 to 945.2)	355.0 (281.0 to 450.5)	298.8 (202.9 to 435.9)	26.0 (12.3 to 46.4)	100.1 (78.2 to 130.1)
Mongolia	2034	238.2 (195.4 to 290.8)	108.6 (85.8 to 138.9)	91.3 (61.6 to 134.6)	7.9 (3.8 to 14.1)	30.3 (23.3 to 40.0)	805.4 (660.7 to 983.2)	367.1 (290.2 to 469.7)	308.8 (208.2 to 455.1)	26.8 (12.7 to 47.8)	102.6 (78.8 to 135.1)
Mongolia	2035	245.8 (201.4 to 301.4)	112.2 (88.5 to 144.3)	94.3 (63.1 to 140.1)	8.2 (3.9 to 14.5)	31.1 (23.6 to 41.6)	830.9 (680.8 to 1019.1)	379.3 (299.4 to 487.7)	318.8 (213.5 to 473.7)	27.7 (13.1 to 49.2)	105.1 (79.7 to 140.7)
Mongolia	2036	253.3 (207.9 to 314.4)	115.8 (90.3 to 149.2)	97.2 (64.7 to 145.8)	8.4 (4.0 to 15.0)	31.9 (23.8 to 42.9)	856.3 (703.0 to 1062.9)	391.4 (305.4 to 504.4)	328.6 (218.6 to 492.9)	28.5 (13.5 to 50.6)	107.7 (80.4 to 144.9)
Mongolia	2037	260.8 (212.1 to 319.3)	119.4 (93.3 to 154.8)	100.1 (66.2 to 152.1)	8.7 (4.1 to 15.4)	32.6 (24.0 to 44.7)	881.8 (717.1 to 1079.6)	403.8 (315.5 to 523.3)	338.4 (223.9 to 514.1)	29.4 (14.0 to 52.2)	110.2 (81.2 to 151.1)
Mongolia	2038	268.4 (217.6 to 333.6)	123.0 (95.8 to 159.4)	102.9 (67.7 to 157.6)	9.0 (4.2 to 15.9)	33.5 (24.2 to 47.9)	907.4 (735.6 to 1128.0)	415.9 (323.9 to 538.8)	348.0 (228.9 to 533.0)	30.3 (14.3 to 53.7)	113.2 (81.9 to 161.8)
Mongolia	2039	275.9 (221.4 to 346.1)	126.6 (98.1 to 164.1)	105.7 (69.1 to 163.0)	9.2 (4.4 to 16.3)	34.3 (24.5 to 48.5)	932.6 (748.5 to 1170.3)	428.0 (331.6 to 554.8)	357.5 (233.8 to 550.9)	31.2 (14.7 to 55.3)	116.0 (82.9 to 163.9)
Mongolia	2040	283.4 (227.9 to 358.6)	130.2 (100.6 to 169.5)	108.5 (70.6 to 168.1)	9.5 (4.5 to 16.8)	35.3 (25.3 to 50.7)	958.3 (770.5 to 1212.3)	440.1 (340.1 to 573.2)	366.8 (238.7 to 568.2)	32.0 (15.1 to 56.9)	119.3 (85.5 to 171.3)
Mongolia	2041	290.9 (231.3 to 368.6)	133.6 (102.8 to 174.3)	111.2 (72.0 to 173.0)	9.7 (4.6 to 17.3)	36.3 (25.7 to 52.0)	983.5 (782.1 to 1246.1)	451.8 (347.4 to 589.3)	375.9 (243.5 to 584.8)	32.9 (15.5 to 58.6)	122.9 (87.1 to 175.9)
Mongolia	2042	298.5 (236.3 to 380.8)	137.1 (104.9 to 179.7)	113.8 (73.5 to 177.7)	10.0 (4.7 to 17.8)	37.5 (26.0 to 54.8)	1009.2 (799.0 to 1287.5)	463.7 (354.7 to 607.5)	384.9 (248.4 to 600.7)	33.8 (15.9 to 60.2)	126.7 (88.0 to 185.2)
Mongolia	2043	306.0 (240.9 to 392.4)	140.6 (107.6 to 184.4)	116.4 (74.9 to 182.1)	10.3 (4.8 to 18.3)	38.6 (26.5 to 57.6)	1034.4 (814.4 to 1326.8)	475.5 (363.6 to 623.5)	393.6 (253.1 to 615.7)	34.8 (16.3 to 61.9)	130.6 (89.7 to 194.6)
Mongolia	2044	313.4 (246.5 to 400.8)	144.0 (109.8 to 188.6)	118.9 (76.3 to 186.7)	10.5 (4.9 to 18.8)	39.9 (27.0 to 59.9)	1059.7 (833.5 to 1355.0)	486.9 (371.2 to 637.8)	402.1 (257.8 to 631.1)	35.7 (16.7 to 63.5)	135.0 (91.4 to 202.6)
Mongolia	2045	321.0 (252.7 to 404.5)	147.4 (112.6 to 194.5)	121.4 (77.6 to 191.3)	10.8 (5.1 to 19.3)	41.3 (27.8 to 65.0)	1085.1 (854.3 to 1367.6)	498.5 (380.6 to 657.5)	410.3 (262.5 to 646.7)	36.6 (17.2 to 65.2)	139.8 (94.0 to 219.9)
Mongolia	2046	328.5 (259.7 to 413.9)	150.9 (114.8 to 199.5)	123.7 (79.0 to 195.1)	11.1 (5.2 to 19.8)	42.8 (28.4 to 67.2)	1110.5 (878.1 to 1399.3)	510.1 (388.2 to 674.6)	418.1 (267.1 to 659.5)	37.5 (17.6 to 66.9)	144.8 (96.1 to 227.1)
Mongolia	2047	335.9 (264.7 to 425.9)	154.2 (117.4 to 205.3)	125.9 (80.4 to 198.2)	11.4 (5.3 to 20.4)	44.5 (29.7 to 71.1)	1135.6 (894.9 to 1440.1)	521.3 (397.0 to 694.2)	425.5 (272.0 to 670.1)	38.4 (18.0 to 68.8)	150.3 (100.5 to 240.3)
Mongolia	2048	343.7 (271.6 to 435.8)	157.7 (119.9 to 210.7)	128.1 (81.9 to 202.1)	11.6 (5.5 to 20.9)	46.3 (31.2 to 75.6)	1162.2 (918.2 to 1473.5)	533.2 (405.4 to 712.4)	433.1 (276.9 to 683.4)	39.4 (18.5 to 70.5)	156.6 (105.4 to 255.4)
Mongolia	2049	351.6 (277.9 to 440.9)	161.3 (122.0 to 215.3)	130.3 (83.2 to 206.1)	11.9 (5.6 to 21.3)	48.1 (32.2 to 79.7)	1188.7 (939.5 to 1490.6)	545.2 (412.4 to 728.1)	440.5 (281.3 to 697.0)	40.3 (18.9 to 72.0)	162.8 (108.8 to 269.3)
Mongolia	2050	359.8 (283.2 to 452.8)	165.1 (124.8 to 222.0)	132.4 (84.4 to 210.2)	12.2 (5.7 to 21.8)	50.1 (31.9 to 85.9)	1216.4 (957.4 to 1530.8)	558.1 (421.8 to 750.5)	447.7 (285.4 to 710.8)	41.2 (19.3 to 73.7)	169.4 (107.8 to 290.4)

		2018 US Dollars per capita					2018 PPP per capita				
Country	Year	Health spending	Government health spending	Out-of-pocket health spending	Prepaid private health spending	Development assistance for health	Health spending	Government health spending	Out-of-pocket health spending	Prepaid private health spending	Development assistance for health
Montenegro	1995	560.3 (512.7 to 606.9)	402.7 (367.8 to 441.3)	154.8 (127.5 to 186.4)	1.9 (0.9 to 3.7)	0.8 (0.8 to 0.8)	1231.8 (1127.1 to 1334.3)	885.4 (808.6 to 970.2)	340.4 (280.3 to 409.8)	4.2 (2.0 to 8.2)	1.8 (1.8 to 1.8)
Montenegro	1996	588.3 (547.2 to 629.5)	428.2 (394.6 to 460.9)	158.0 (133.7 to 186.6)	2.0 (0.9 to 4.0)	0.1 (0.1 to 0.1)	1293.4 (1203.1 to 1384.1)	941.4 (867.5 to 1013.4)	347.4 (293.9 to 410.3)	4.4 (2.1 to 8.8)	0.2 (0.2 to 0.2)
Montenegro	1997	578.6 (542.8 to 617.3)	420.4 (390.0 to 450.6)	156.0 (134.5 to 182.3)	2.0 (0.9 to 3.8)	0.2 (0.2 to 0.2)	1272.0 (1193.3 to 1357.1)	924.3 (857.3 to 990.7)	343.0 (295.7 to 400.8)	4.3 (2.0 to 8.4)	0.4 (0.4 to 0.4)
[truncated: 1,359,849 more chars]
